# Clinic-Genomic Association Mining for Colorectal Cancer Using Publicly Available Datasets

**DOI:** 10.1155/2014/170289

**Published:** 2014-06-02

**Authors:** Fang Liu, Yaning Feng, Zhenye Li, Chao Pan, Yuncong Su, Rui Yang, Liying Song, Huilong Duan, Ning Deng

**Affiliations:** ^1^Department of Biomedical Engineering, Key Laboratory for Biomedical Engineering of Ministry of Education, Zhejiang University, Hangzhou 310027, China; ^2^General Hospital of Ningxia Medical University, Yinchuan 750004, China

## Abstract

In recent years, a growing number of researchers began to focus on how to establish associations between clinical and genomic data. However, up to now, there is lack of research mining clinic-genomic associations by comprehensively analysing available gene expression data for a single disease. Colorectal cancer is one of the malignant tumours. A number of genetic syndromes have been proven to be associated with colorectal cancer. This paper presents our research on mining clinic-genomic associations for colorectal cancer under biomedical big data environment. The proposed method is engineered with multiple technologies, including extracting clinical concepts using the unified medical language system (UMLS), extracting genes through the literature mining, and mining clinic-genomic associations through statistical analysis. We applied this method to datasets extracted from both gene expression omnibus (GEO) and genetic association database (GAD). A total of 23517 clinic-genomic associations between 139 clinical concepts and 7914 genes were obtained, of which 3474 associations between 31 clinical concepts and 1689 genes were identified as highly reliable ones. Evaluation and interpretation were performed using UMLS, KEGG, and Gephi, and potential new discoveries were explored. The proposed method is effective in mining valuable knowledge from available biomedical big data and achieves a good performance in bridging clinical data with genomic data for colorectal cancer.

## 1. Introduction

Cancer is one of the major diseases that endanger human life. As American Cancer Society reported, a total of 1,660,290 new cancer cases and 580,350 cancer deaths were projected to occur in the United States in 2013 [1]. In developing countries, such as China, one person is diagnosed with cancer every six minutes, and 8550 people become cancer patients every day [2]. By 2020, the total number of cancer deaths in China is expected to reach 3 million, and the total number of prevalence will reach 6 million [2]. Worldwide, more than 20 million new cancer cases are estimated to be detected by 2030 [3]. Providing much more effective means of early detection and treatment for cancer are still great challenges faced by human beings.

Modern medicine is moving toward the direction of personalized medicine, which refers to the tailoring of medical treatment to the individual characteristics of each patient [4]. Clinical, genetic, protein, and metabolism information of patients are expected to improve the prevention, diagnosis, and treatment of disease together in this medical mode. This will have a great dependence on the successful transformation of basic research results into clinical practice.

With the development of medical informatics and molecular biology, vast amounts of biomedical data have been accumulated. These data cover multiple levels, including both clinical data in macrocosmic aspect and genomic data in microcosmic aspect. However, most clinical data have no corresponding genomic data, while most genomic data have no precise clinical annotation data. Due to the lack of effective linkages, the fruits of basic research have not been translated into clinical practice completely, and problems arising in clinical practice also have not made a big difference to the basic research directions as expected. Exploited value of available biomedical data is far less than the intrinsic value of these data. Therefore, it can deepen our understanding of the origin and progression of disease, by mining association between clinical data and genomic data from massive available biomedical big data, which promote the bidirectional translation between clinical research and basic research, and ultimately achieve the purpose of promoting the development of personalized medicine.

In recent years, a growing number of researchers began to focus on how to establish associations between clinical data and genomic data. The association between clinical data and genomic data is named as clinic-genomic association in this paper, representing that a clinical feature may have an effect on the gene expression value or the gene may dominate the clinical feature. A persuasive research is the Human Disease Network established by Goh et al. [5]. They extracted 1284 disorders, 1777 disease genes, and associations between these disorders and genes from Online Mendelian Inheritance in Man (OMIM) [6] and then built a bipartite graph using these data. Based on the bipartite graph, they generated two biologically relevant networks, the Human Disease Network and the Disease Gene Network, by assuming that two diseases are connected if they share at least one gene and two genes are connected if they are associated with at least one common disease. Several valuable discoveries are then revealed by these two networks. Other related researches include the Phenome-Genome Network [7], Gene Expression Atlas [8], and iCOD [9]. Excellent works have been done, but there are still many aspects that are needed to be improved. Both Human Disease Network and Phenome-Genome Network take many kinds of diseases into consideration, making it difficult for them to focus on the detail of one certain disease. In addition, only conclusive data, instead of experimental data such as gene expression data, have been utilized by Human Disease Network. Gene Expression Atlas curated the original data submitted by various researches within an experiment instead of comprehensive analysis. While the iCOD only analyzed gene expression data obtained by their own experiments, without considering publicly available datasets. So the data source of iCOD is limited amount. In summary, none of these work mined clinic-genomic associations by comprehensively analysing available gene expression data for a single disease.

Colorectal cancer is the second leading cause of cancer death in the United States and the fifth leading cause in China [10]. Compared with most other cancers, the molecular mechanism of colorectal cancer is relatively clear, making it appropriate for the evaluation of research results. Besides, from the clinical perspective, the outcome of colorectal cancer depends greatly on the stage at which it is detected [11]. The 5-year survival rates of colorectal cancer patients diagnosed at distant stage decrease from 90% to 8%, compared with patients diagnosed at localized stage [11]. However, most clinical symptoms of colorectal cancer arise at a later stage, which greatly impedes the early diagnosis and treatment. If the molecular mechanism of clinical signs or symptoms can be revealed, it would be helpful to detect the molecular change before deteriorating of the disease. Therefore, mining clinic-genomic associations for colorectal cancer is a promising solution to this problem.

To this end, this paper takes colorectal cancer as a typical disease to study how to mine clinic-genomic association for a certain disease using public available datasets, aiming at facilitating the diagnosis and treatment of colorectal cancer. As well, the proposed method can provide a general way for promoting preconized medicine for other disease. The proposed method consists of three parts: extracting clinical concepts using the unified medical language system (UMLS) [12]; extracting genes through literature mining; and mining clinic-genomic associations through statistical analysis. A total of 665 colorectal cancer related clinical concepts, 8392 colorectal cancer related genes, and 23517 clinic-genomic associations were obtained using this method. To evaluate this method and interpret the results obtained, we tried different approaches to make these results more intuitive and understandable. UMLS semantic types were used for clinical concepts analysis, the Kyoto Encyclopaedia of Genes and Genomes (KEGG) [13] pathway for gene analysis, and Gephi [14] visualization for clinic-genomic association analysis. Our investigation provides some interesting findings, such as colorectal cancer related disease (osteoporosis) and related symptoms (angina pectoris), demonstrating that the proposed method can achieve a good performance in bridging clinical data with genomic data as well as mining hidden knowledge from available data.

## 2. Materials and Methods

### 2.1. Data Collection and Preprocessing

On one hand, public gene expression data repositories, such as gene expression omnibus (GEO) [15], Stanford microarray database (SMD) [16], and ArrayExpress [8], archive and distribute high-throughput gene expression data submitted by scientific community. Most of these data are accompanied with rich context information including experimental factors and clinical attributes according to the minimum information about a microarray experiment (MIAME) [17] standard, making it ideal for clinic-genomic association mining. As a large database of these, GEO provides the largest gene expression dataset and convenient query and downloading function. Therefore, we took GEO as main data source to perform association mining. On the other hand, with large number of research papers published, several databases integrating research results were established, such as OMIM and genetic association database (GAD) [18]. Data in these databases are generally acknowledged and can be used for evaluation and validation. Compared with OMIM, GAD collects data with less restrict limitations, leading to richer data records. So we take GAD as assistant data source to supplement and evaluate association mining results.

We accessed the GEO site on February 3, 2013. Colorectal cancer related GEO series (GSE) were preliminarily identified as those that passed the custom filter rule [see Supplementary Table S1 (Supplementary Material available online at http://dx.doi.org/10.1155/2014/170289)]. Search statements were constructed using GEO Datasets Advanced Search Builder [19], which aims to perform more refined queries in order to filter down to the most relevant data. A total of 628 GSE were found out and downloaded in simple omnibus format in text (SOFT) format from FTP site of GEO use Aspera Connect tool.

All sample data tables and platform data tables of a GSE are stored in a single SOFT file. Note that it is quite inconvenient and inefficient to read the generally huge line-based, plain text format file each time we parsed 628 GSE files into several sample table files and platform table files, with each sample table file holding data for a certain GEO sample (GSM) and each platform table file holding data for a certain GEO platform (GPL). Most of the clinical information is located in title, source, species, characteristics, and descriptions fields of GSM annotations. We developed in-house Perl program to extract these information into relational database for further analysis. GSM not from human beings and GSM without any keyword of colon, rectum, rectal, hepatic flexure, or sigmoid in the extracted annotations were eliminated during the extracted information. Sample data tables index expression measurements of multiple RNA transcripts with Probe Set IDs, while the external gene identifiers, names, and symbols were stored in platform data tables. In order to allow the same gene measured in different platforms to be unified, Probe Set IDs were mapped to the HUGO (human genome organization) symbols. Since platforms are made by different manufactories and platform data tables are provided correspondingly, UniGene symbols appear in different column of platform data tables irregularly or even disappear, making automatically mapping from Probe Set IDs to gene symbols quite difficult. Thus we implemented this procedure manually in order to make full use of platform data tables. As for platforms not providing HUGO symbols, IDconverter [20] was used. Platform map files were stored in Map Containers format, a data structure of MATLAB, for further use.

GAD was accessed on April 18, 2012. We used keyword-search function of GAD query tool to obtain colorectal cancer related records. Setting search field to “disease” and entering one of colorectal cancer related keywords [see Supplementary Table S2] each time, a total of 4784 records were picked out and downloaded into an excel file for subsequent processing.

### 2.2. Extracting Clinical Concepts Using UMLS

**Figure 1:**
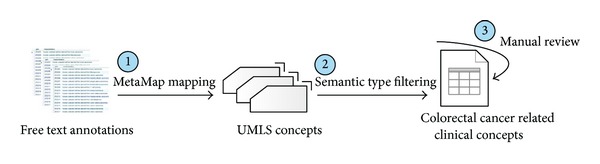
Extracting clinical concepts using UMLS. Three steps were involved. First, free-text annotations were mapped to UMLS concepts using MetaMap. Second, clinical concepts were screened out by semantic types. Finally, a manual review was performed to emitted mapping errors.

GEO annotations are in free-text format and a certain concept is frequently presented in different ways by different scientists, making it difficult to organize and compare data generated from different research institutions. Taking “colon cancer” as an example, it can be descripted as “colon cancer,” “colon carcinoma,” “human carcinoma colon cell,” and so on. In this case, ontology is urgently needed to link these various descriptions together. UMLS is the largest thesaurus in the biomedical domain, collecting biomedical concepts from controlled vocabularies and classifications used in patient records, administrative health data, bibliographic databases, and so on. Each concept is annotated with at least one semantic type from a semantic network that broadly covers the medical domain [21]. In order to extract unified colorectal cancer related clinical concepts effectively, we mapped free-text annotations to UMLS concepts. The process flow is shown in Figure 1.

First, a Java program was developed to map the “characteristics” and “description” field to UMLS concepts by calling MetaMap (a program developed at National Library of Medicine) API [22]. Since “Source,” “Species,” and “Title” contain only some identification information, they were ignored in this process. Concept CUIs, concept names, sematic types, and corresponding original phrases were specified to be outputted and recorded in relational database. Second, we used clinical related semantic types [23] to screen for clinical concepts. Those concepts belonging to any semantic type in Supplementary Table S3 were kept, while the others were excluded. To improve the accuracy of mapping and thus reduce the burden of future work, a manual elimination of incorrectly mapped concepts was performed with the help of the recorded original phrases.

### 2.3. Extracting Genes through Literature Mining

The genetic factors leading to colorectal cancer have been extensively studied, and a large numbers of research papers have been published on the subject. The large body of published biomedical literature is one of the richest data sources for systematically identifying colorectal cancer related genes without microarray expression experiment. In order to obtain nontrivial knowledge quickly and accurately, we took available literature-mining achievements as a data source instead of performing literature mining algorithm directly. GAD was employed in this paper and colorectal cancer related records in GAD has already been picked out and curated in an Excel sheet in Section 2.1. Every record in GAD reflects an association between a gene with a disease through “association” attribute, with “Y” indicating “associated,” “N” indicating “not associated,” and blank indicating “uncertain.” We extracted genes from the “Gene” column and recorded the corresponding association values.

### 2.4. Mining Clinic-Genomic Associations through Statistical Analysis

**Figure 2:**
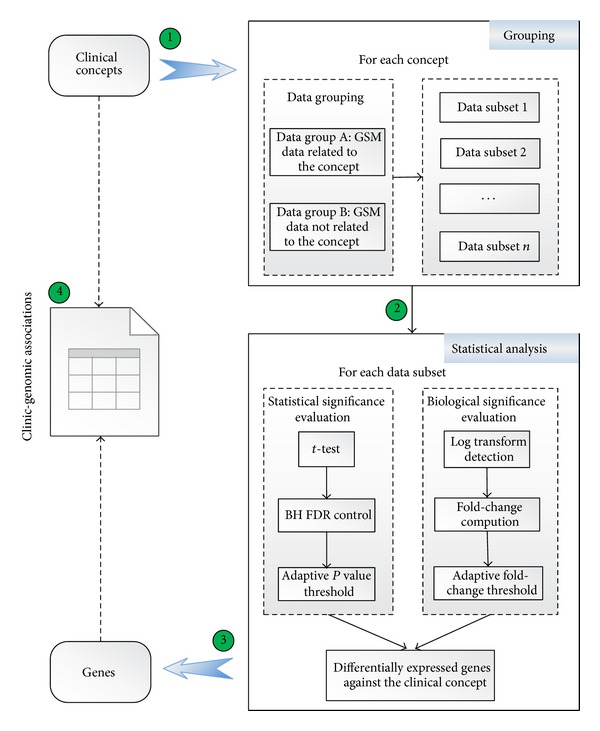
Statistical-analysis-based association mining flow. Four steps were involved. First, for each concept, GSM data were divided into two groups and further organized into different data subsets based on GSE and GPL. Second, for each data subset, differentially expressed genes were screened out according to statistical significance and biological significance. Third, for each concept, differentially expressed genes from every data subset were integrated. Finally, a series of associations were established between each concept with the corresponding differentially expressed genes.

We proposed a statistical-analysis-based clinic-genomic association method for colorectal cancer. The procedure is shown in Figure 2. Clinical information acquired with the help of UMLS was used as data inputs to obtain the corresponding genomic information. For each concept, GSM can be divided into two groups depending on whether the concept was extracted from their annotations. Data group A contains gene expression data of GSM of which annotations contain the concept, while data group B contains data of GSM of which annotations do not contain the concept. Data from different GSEs or different GPLs were heterogeneous regarding of their measuring technologies, measured genes, and preprocessing methods, making it meaningless to analysis them together directly. So we further grouped these data into several subsets according to their GSE and GPL. Each data subset contains data from both group A and group B. Data subset with less than four GSMs involved by data group A or data group B are eliminated in the consideration of significance consideration of statistical analysis.

Hundreds of data subsets need to be analysed and some analysis data subsets contain more than 400 samples. Such a heavy computation burden imposes great challenges on most computation tools. MATLAB is a software with powerful computing capabilities. Most importantly, bioinformatics toolbox of MATLAB is packed with a series of robust and well-tested functions, providing an integrated software environment for genome and proteome analysis. Based on above considerations, we use MATLAB to implement the proposed association mining method.

#### 2.4.1. Statistical Significance Evaluation

To explore genes that are differentially expressed in data group A relative to data group B, we first made a hypothesis that all genes are equally expressed in these two data groups and then we used the “mattest” function, which is provided by the bioinformatics toolbox of MATLAB for classical t-test, to test our hypothesis. A list of *P* values presenting the significance of differential expression between data group A and data group B were figured out. To control the overall probability of type I error, these raw *P* values must be adjusted for multiple testing. Among all adjustment methods for multiple comparisons, the Benjamini and Hochberg procedure [24], abbreviated as “BH FDR,” controlling the proportion of false positives among the genes called as differentially expressed, is probably more appropriately for datasets with very large numbers of genes [25]. Therefore, in this case study, BH FDR control was implemented using the “mafdr” function by setting the “BHFDR” parameter to be “TRUE.” A series of adjusted *P* values was obtained. The default *P* value threshold was 0.05. But if more than 1% of all the measured genes are positive in this case, the threshold will automatically shift to keep only the most significant 1% genes.

#### 2.4.2. Biological Significance Evaluation

Fold-change is defined as the average expression over all samples in a condition divided by the average expression over all samples in another conditions. The average expression should be in constant scale rather than logarithmic or exponential scale. Diverse preprocessing methods were used to obtain the preprocessed data. Some data have been logarithmic transformed, while others not. Most of the processed data did not note the used scale explicitly. So we put forward the following algorithm to detect whether the input data were in log scale. As a general rule, if the scale is around 0 to 16, it is in log scale; if it is around 0 to 40000, it is in original scale. Quantile value was computed to explore data distribution range. The original algorithm refers to GEO2R [26] and the main improvement is using mean value to avoid big noise. MATLAB code was presented in Supplementary Table S4. When input data was identified as in log scale, NeedLogC was set to false. Otherwise, NeedLogC was set to true. Fold-change was computed using the modified “mavolcanoplot” function. NeedLogC was transferred to “mavolcanoplot” function as the parameter value of “LogTrans.” The returned fold-change value has been processed. Positive value means upregulated, while negative means downregulated. The default fold-change threshold was 2. But if none of the measured genes were positive, the threshold would automatically shift to keep the largest 1% absolute fold-change values (but no less than 1.5).

Only genes passing both the *P* value threshold and fold-change threshold were considered as differentially expressed against the clinical concept used to group data. For each concept, genes obtained from all data subsets were combined together to form a set of clinic-genomic associations.

## 3. Results and Discussion

### 3.1. Clinical Concept Datasets

A total of 665 colorectal cancer related clinical concepts, see Supplementary Table S6, were obtained using the UMLS-based method. About 115 concepts (14.5%) resulted from incorrect mapping had been ruled out during the manual review process. The most common type of mapping errors come from abbreviations, including “pain” from “pn,” “Edema” from “ED.” Semantic type mistakes were also found out, such as “Dukes Disease” from “Dukes Stage.”

Clustering clinical concepts based on semantic types and counting the number of concepts in each semantic type can provide us with an intuitive view about what is most concerned in clinical studies of colorectal cancer. Distribution of concepts acquired in this paper among the 20 semantic types was obtained; see Supplementary Figure S1. Neoplastic process, biologically active substance, finding, and disease or syndrome cover more than half of all concepts. The number of GSM related to each concept reflects the importance degree of the concept to some extent. The top 15 concepts relating to the most number of GSM were presented in Table 1, after ignoring general concepts like carcinoma, colon carcinoma, malignant neoplasms, and others similar. According to Table 1, medical history, family history, microsatellite instability, and tobacco use are important clinical information focused in colorectal cancer clinical research.

### 3.2. Genomic Datasets

From GAD, we got 904 genes, of which 247 annotated with “Y,” 159 with “N,” and 823 with “Blank” (overlap exists among these three cases). Association value indicates whether a gene is associated with a disease or not. However, it is not unique for some genes, due to the fact that each value in GAD depends on a single paper, while different papers may have different conclusions. Also due to this, we did not concern the specific association value in the following analysis, but just assume that these genes are related to colorectal cancer somehow. From GEO, we got 7914 genes which are extracted from our mining results of clinic-genomic associations. A total of 8392 genes [see Supplementary Table S7] were obtained from these two data sources after removing duplicates.

#### 3.2.1. Relevance between Genes and Colorectal Cancer

Genes from GAD are extracted from the published literatures, and genes from GEO are picked out according to statistical analysis. The former is more reliable but less abundant, while the latter is just on the contrary. Genes from GEO are extracted from clinic-genomic associations, of which each one was deduced from gene expression data of one or more GSE. If we impose a different restriction on the number of association related GSE, we can get different number of genes. For instance, we got only 1687 genes after requiring more than 1 related GSE. The overlapping rate between genes from GEO and genes from GAD also varies with different restrictions. Assume genes from GAD are reliable, the overlapping rate reflects the relevance between genes from GEO with colorectal cancer to a certain extent. A method to calculate the relevance quantitatively using the overlapping rate was defined as

(1) $$\begin{array}{c} \text{Relevance}\;\text{score}=\;\frac{\text{Overlapping}\;\text{rate}}{\text{Proportion}}. \end{array}$$

Here, “Relevance score” is the quantitative evaluation of association degree between genes with colorectal cancer, “Overlapping rate” is the proportion of overlapping genes in genes from GEO, and “Proportion” is the proportion of genes from GEO in all genes.

**Figure 3:**
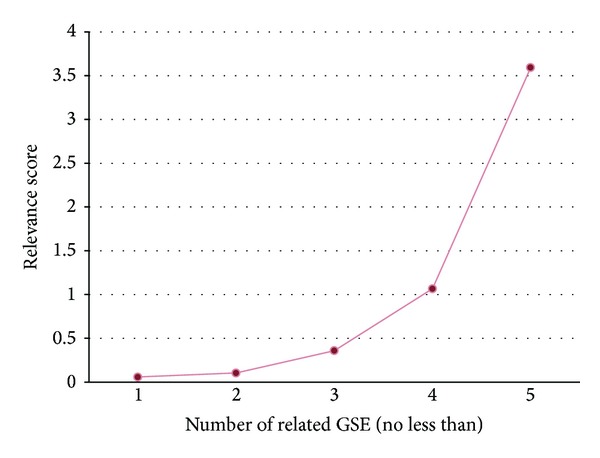
Quantitative evaluation of association degree between genes from GEO with colorectal cancer. The horizontal axis presents the number of association related to GSE, while the vertical axis presents the relevance score computed using formula (1).

Figure 3 demonstrates the relationship between “Relevance score” and the number of relation related GSE. It can be seen that the “Relevance score” increases with the increases of related GSE numbers. This trend can be interpreted from the following perspective. Related GSE are data foundation of clinic-genomic associations. Therefore, more related GSE indicates much more reliable clinic-genomic associations about colorectal cancer and thus the closer association between genes and colorectal cancer.

#### 3.2.2. KEGG Pathway Analysis

To further interpret the obtained results, we can link genomic information with higher order functional information. KEGG is the right knowledge base for systematic analysis of gene functions [13]. Genes are inputted in online analysis tool of KEGG pathway database. Top 10 pathways, see Supplementary Table S5, covering the most amounts of input genes are obtained, including pathways in cancer, proteoglycans in cancer, and focal adhesion pathway. As for focal adhesion pathway, the literature [27] has shown that both primary colorectal cancers and colorectal liver metastases express high levels of FAK (focal adhesion kinase) mRNA and p125 FAK protein. In addition, 51 of the acquired genes involve the colorectal cancer pathway. These genes are listed in Table 2, including important oncogenes (*KRAS *and* CTNNB1*), tumour suppressors (*APC, DCC, TP53, BAX, SMAD2, SMAD4,* and* TGFBR2*), and DNA repair genes (*MLH1, MSH2, MSH3,* and* MSH6*).

### 3.3. Clinic-Genomic Association Datasets

A total of 23517 associations between 7914 genes and 139 concepts were found out. All the associations are listed in Supplementary Table S8. Such a massive amount of associations is difficult to evaluate or interpret directly. Therefore, we used two methods to gain a more profound understanding of these associations. First, we use visualization as a powerful means to leverage the perceptual abilities of humans to find useful information from obtained associations. Shape, colour, distance, and other elements can all be used to corroborate understanding of network. In this paper, Gephi was performed visualization analysis on clinic-genomic associations from different perspectives, including overall view, data-source-feature view, and semantic-type view. Clinical concepts were inputted into Gephi as source nodes, genes as destination nodes, and absolute value of fold-changes as weight of edges. Associations are clustered by the default modularize method, fast unfolding of communities in large networks, and different classes were presented in different colours. Second, the number of association related GSE was taken as a determinant to recognize highly reliable associations.

#### 3.3.1. Overall View

**Figure 4:**
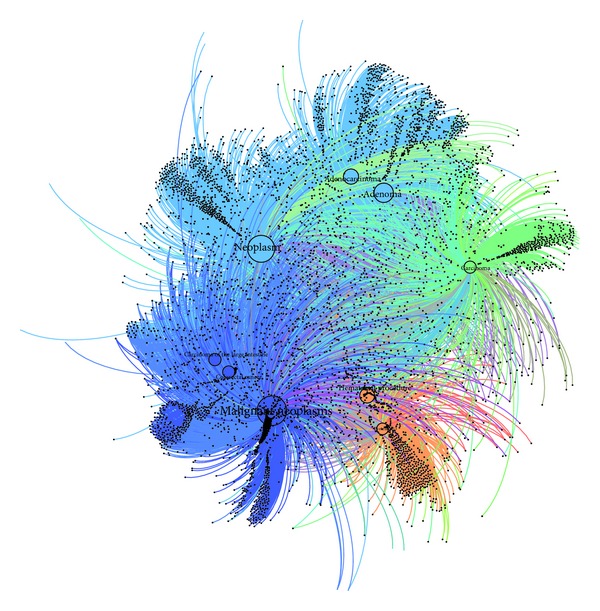
Simplified version of overall view of clinic-genomic associations. Complete version can be found in Supplementary Figure S18. This simplified version was generated by ignoring several unobvious concepts and genes to reflect important associations much clearer.

In this view, all associations were imported to Gephi together. Force atlas was used as layout algorithm. The analysis result was shown in Supplementary Figure S18 and simplified version of the analysis result was shown in Figure 4. The result confirms the complexity of these associations: a gene may relate to multiple clinical concepts and also a clinical concept may associate with multiple genes. But through this visualization method, several important concepts come into sight clearly. These include malignant neoplasms, neoplasm, adenoma, adenocarcinoma, and Protein p53, suggesting that they are connected with large number of genes and they are focused concepts in colorectal cancer research.

Besides, Figure 4 reflects the development process between adenoma and adenocarcinoma. The nearest location in the figure expresses their close relationship, while the bigger size of adenoma node relative to adenocarcinoma node represents that there are more clinic-genomic associations with adenoma. Previous knowledge shows that the development process of colorectal cancer can be divided into several stages, including normal mucosa, adenoma,and adenocarcinoma, noting that adenoma appears earlier than adenoma carcinoma. In clinical practice, patients are always diagnosed based on their clinical symptoms, vital signs, and so forth in an early stage due to the slow arising of colorectal cancer symptoms, resulting in inadequate data from early patient. However, the proposed method in this paper is able to uncover more knowledge about the early stage of colorectal cancer. This lays a good knowledge foundation for the research on the early diagnosis and treatment for colorectal cancer and may reflect the significance of the proposed method to the personalized medicine.

#### 3.3.2. Data-Source-Feature View

**Figure 5:**
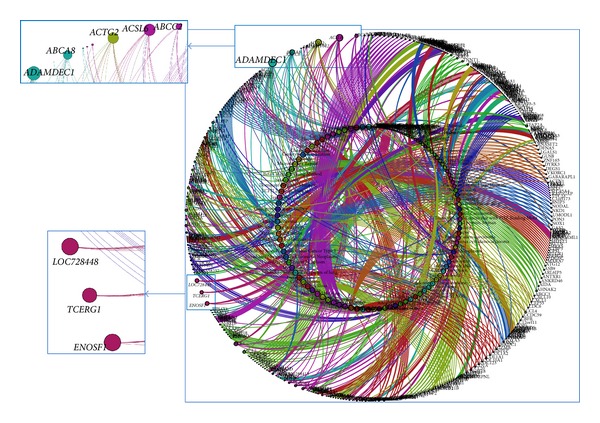
Data-source-feature view of obtained clinic-genomic associations. The number of related genes for each concept was limited to no more than 10. Dual Circle Layout algorithm was employed in Gephi. Several genes stood out from this view.

Due to various features of data source, statistical analysis results of certain data group subsets pass* P* value threshold or fold-change threshold easily, resulting in that some clinical concepts connect with lots of genes. For example, malignant neoplasm links with 1917 genes. From this point, it is inappropriate to simply unite all results together because concepts with little genes will be buried in the visualization results. To highlight the importance of each concept, we reduced the number of related genes to no more than 10. A total of 1075 associations between 139 concepts with 524 genes remained, of which the Gephi outputs was shown in Figure 5. Dual Circle Layout was used in this view for the comparable magnitude of gene and concept number. Nodes of inner circle represent clinical concepts, while nodes of outer circle represent genes. In Figure 5, certain genes, including* ADAMDEC1, ABCC2, ABCA8, ACTG2, ACSL6, LOC728448, TCERG1,* and* ENOSF1* reveal their importance. Among them,* ABCC2* was also included in genes extracted from GAD with blank association value, indicating the potential of complementing GAD with results of statistical analysis method.

#### 3.3.3. Semantic-Type View

Classifying clinic-genomic associations based on semantic type of clinical concepts is helpful to get a deeper understanding of associations involved by each semantic type. We analysed all of the 20 semantic types, respectively. Selected results have been presented in Supplementary Figure S2~S17. Lots of meaningful rediscoveries as well as interesting new findings were obtained. In particular, two typical semantic types, “Disease or Syndrome” and “Sign or Symptom,” are illustrated specifically in this paper in detail.

The “Disease or Syndrome” semantic type covers 676 associations between 10 clinical concept and 445 genes. The Gephi analysis results is shown in Supplementary Figure S6, from which the general acknowledged colorectal cancer related diseases, including inflammatory disease, irritable bowel syndrome, intestinal disease, and inflammatory bowel disease are very conspicuously. Besides, the “Osteoporosis” concept also comes into view. It is not broadly known to the public as colorectal cancer related disease, but some researchers have claimed that osteoporosis is associated with the risk of colorectal adenoma in women recently [28]. Based on statistical-analysis-based association mining method, 22 genes were identified as osteoporosis related. The top 10 genes ordering by* P* value are* C1orf173, TTC23, BCR, TEF, RAP1GAP, SLC45A4, CCDC66, CRISPLD2, IRX5,* and* SAPS1*. Among them, the association between* TTC23* and osteoporosis is also reported by GeneCards [29].

The “Sign or Symptom” semantic type includes 393 associations between 10 clinical concept and 393 genes. The Gephi output is shown in Supplementary Figure S17. In addition to abdominal bloating, the most obvious one, other familiar signs or symptoms, like red stools, diarrheal, constipation, change in bowel habit, and vomiting and nausea, also have a place in Supplementary Figure S2~S17. Furthermore, “Angina Pectoris” appears out of expectation. It is not a common symptom of colorectal cancer, but the eHealthMe website displays a group of data from colon cancer patients who have angina pectoris [30], indicating that angina pectoris probably has potential association with colorectal cancer.

#### 3.3.4. Recognition of Highly Reliable Associations

Different GSE, generally submitted to GEO by different researchers, are basically irrelevant. Therefore, it is of small possibility that an association was obtained by accident if the association can be deduced from more than one GSE. This point was also illustrated in Section 3.2.1 as more related GSE indicates much more reliable clinic-genomic association. Counting the number of association related GSE for every association and then restricting the number to more than one, we got 3474 associations between 31 clinical concepts with 1689 genes. These associations are considered as highly reliable associations and have been marked out in Supplementary Table S8.

Generally, this paper proposed a method to mine associations between clinical data and genomic data using publicly available datasets, which is a great mission in the era of big data. We focused on a typical disease, colorectal cancer, to learn the potential of the vast amounts of existing biomedical data. Colorectal cancer related symptoms, diseases or syndromes, neoplastic processes, and other clinical features have all been covered in this research. This is a novel exploration for little researchers having done such a thorough work for a single disease using this mode. Outcome was appreciated, but there are still lots of space for improvement. First, clinical concepts are regarded as independent during the statistical analysis process to reduce the complexity. Therefore, thoughtful measures should be taken to guarantee accuracy. Second, as an exploration, we only take a representative database, GEO, as data source. Much more datasets could be involved in the future study. Last, to make good use of the association mining results and to share the association mining methods with peer researchers, a publicly available platform would be helpful. For this consideration, such a platform is in process now.

## 4. Conclusions

Aiming at facilitating the diagnosis and treatment of colorectal cancer and also providing a general way for promoting preconized medicine for other disease, this paper proposed a clinic-genomic association mining method for colorectal cancer, which consists of three parts: extracting clinical concepts using UMLS; extracting genes through literature mining; and mining clinic-genomic associations through statistical analysis. Using the proposed method, 23517 clinic-genomic associations between 139 clinical concepts and 7914 genes were obtained. Moreover, 3474 of all these associations, relating 31 clinical concepts with 1689 genes, were identified as highly reliable based on the number of association related GSE. Lots of results have been validated and there are also several new discoveries, including colorectal cancer related disease (osteoporosis) and related symptoms (angina pectoris), demonstrating the correctness and usefulness of the proposed method. These results can be shared with clinical researchers and basic researchers as well as translational researchers to suggest new study directions or to answer some unsettled questions. As bridges between clinical researches and genomic researches, these associations would be helpful to accelerate the bidirectional translation between these two fields. Besides, this method can also be transplanted to analyse other diseases, such as breast cancer and liver cancer. In the future work, we will expand the data sources, blending in ArrayExpress, SMD, to enrich our results. On the other hand, expanding knowledge of clinical concepts by combining UMLS-based concepts with electronic medical records will be an appropriate direction of our research.

## Supplementary Material

The Supplementary Material provides the following results:
Table S1 lists the filter rule used to search GEO for colorectal cancer related GSE. Table S2 lists the colorectal cancer related keywords used to search GAD. Table S3 list clinical related semantic types used to screen out clinical concepts. Table S4 presents the source code of modified logarithmic scale detection algorithm in MATLAB. Table S5 list the top 10 pathways with the most number of genes. Table S6 lists all the colorectal cancer related clinical concepts found by the proposed method. Table S7 lists all the colorectal cancer related genes found by the proposed method. Table S8 lists all the clinic-genomic associations mined out by the proposed method.
Figure S1 shows the concept distribution against semantic types. Figure S2~S17 are Gephi outputs of clinic-genomic associations classified by semantic types.

## Figures and Tables

**Table 1:** Top 15 concepts related to the most number of GSM.

Concept name	GSM count	Rank
Medical history	1004	15
Family history	1002	16
Instability	907	19
Microsatellite repeat	895	20
Recurrence	844	21
Protein p53	824	22
Histology procedure	643	27
Death	570	29
Microsatellite instability	547	32
Primary neoplasm	514	34
Tobacco use	446	36
Encounter due to tobacco use	446	37
Ethnic	446	38
Leukaemia	434	40
Encounter due to therapy	402	41

**Table 2:** The 51 genes of acquired results involving the colorectal cancer pathway.

*AKT1 *	*BIRC5 *	*KRAS *	*MSH6 *	*RHOA *	*TP53 *
*AKT2 *	*BRAF *	*LEF1 *	*MYC *	*SMAD2 *	*BCL2 *
*AKT3 *	*CASP3 *	*MAP2K1 *	*PIK3CA *	*SMAD3 *	*JUN *
*APC *	*CASP9 *	*MAPK1 *	*PIK3CD *	*SMAD4 *	*MSH3 *
*ARAF *	*CCND1 *	*MAPK10 *	*PIK3R1 *	*TCF7 *	*RALGDS *
*AXIN1 *	*CTNNB1 *	*MAPK8 *	*PIK3R2 *	*TCF7L1 *	*TGFBR2 *
*AXIN2 *	*CYCS *	*MAPK9 *	*PIK3R5 *	*TCF7L2 *	*TGFBR1 *
*BAD *	*DCC *	*MLH1 *	*RAC2 *	*TGFB1 *	*RAC3 *
*BAX *	*FOS *	*MSH2 *			

## List of Abbreviations

GAD: Genetic association database
GEO: Gene expression omnibus
GPL: GEO Platform
GSE: GEO series
GSM: GEO sample
KEGG: Kyoto Encyclopaedia of Genes and Genomes
OMIM: Online Mendelian Inheritance in Man
SMD: Stanford microarray database
SOFT: Simple omnibus format in text
UMLS: Unified medical language system
HUGO: Human genome organization.
