# Supplementary material for: Clinic-Genomic Association Mining for Colorectal Cancer Using Publicly Available Datasets
Source: Biomed Res Int. 2014 Jun 2;2014:170289. doi: 10.1155/2014/170289 (PMC4060771; doi:10.1155/2014/170289)
Supplement: Supplementary file 1 — The Supplementary Material provides the following results: Table S1 lists the filter rule used to search GEO for colorectal cancer related GSE. Table S2 lists the colorectal cancer related keywords used to search GAD. Table S3 list clinical related semantic types used to screen out clinical concepts. Table S4 presents the source code of modified logarithmic scale detection algorithm in MATLAB. Table S5 list the top 10 pathways with the most number of genes. Table S6 lists all the colorectal cancer related clinical concepts found by the proposed method. Table S7 lists all the colorectal cancer related genes found by the proposed method. Table S8 lists all the clinic-genomic associations mined out by the proposed method. Figure S1 shows the concept distribution against semantic types. Figure S2~S17 are Gephi outputs of clinic-genomic associations classified by semantic types. [file 170289.f1.pdf]

Clinic-genomic Association Mining for Colorectal Cancer using Publicly Available Datasets

Supplement

Fang Liu[1], Yaning Feng[1], Zhenye Li[2], Chao Pan[1], Yuncong Su[1], Rui Yang[1], Liying Song[1], Huilong Duan[1], Ning Deng[1 §]

1. Department of Biomedical Engineering, Key Laboratory for Biomedical Engineering of Ministry of Education, Zhejiang University, Hangzhou 310027, China

2. General Hospital of Ningxia Medical University, Yinchuan, 750004, China

[§] **Correspondence:**

Ning Deng
Department of Biomedical Engineering
Key Laboratory for Biomedical Engineering of Ministry of Education,
Zhejiang University, Hangzhou, 310027, China.
Tel: +86-571-87951972.
Fax: +86-571-87951960
Email: zju.dengning@gmail.com

Supplementary tables

Table S1

Table S1 is the filter rule used to search GEO for colorectal cancer related GSE.

Table S2

Table S2 is the colorectal cancer related keywords used to search GAD.

Table S3

Table S3 is clinical related semantic types used to screen out clinical concepts.

Table S4

Table S4 is the Matlab code of modified logarithmic scale detection algorithm.

Table S5

Table S5 is the top 10 pathways with the most number of genes.

Table S6

Table S6 lists all the colorectal cancer related clinical concepts found by the proposed method. For Column [Association]: "Y" denotes this concept is associated with one or more genes in this study.

Table S7

Table S7 lists all the colorectal cancer related genes found by the proposed method. For Column [Reliability]: "*" denotes this gene was related to a concept by a highly reliable association, of which the number of related GSE is more than 1.

Table S8

Table S8 lists all the clinic-genomic associations mined out by the proposed method. For Column [Reliability]: "*" denotes this is a highly reliable association, of which the number of related GSE is more than 1.

Supplementary figures

Figure S1

Figure S1 is the concept distribution against semantic types. The size of each area reflects the number of concepts covered by the corresponding semantic type.

Figure S2~S17

Figure S2~S17 are Gephi outputs of clinic-genomic associations classified by semantic types.

Figure S2

Gephi outputs of the remaining clinic-genomic associations after limiting the semantic type of clinical concepts to be “Anatomical Abnormality”.

Figure S3

Gephi outputs of the remaining clinic-genomic associations after limiting the semantic type of clinical concepts to be “Biologically Active Substance”.

Figure S4

Gephi outputs of the remaining clinic-genomic associations after limiting the semantic type of clinical concepts to be “Cell or Molecular Dysfunction”.

Figure S5

Gephi outputs of the remaining clinic-genomic associations after limiting the semantic type of clinical concepts to be “Clinical Attribute”.

Figure S6

Gephi outputs of the remaining clinic-genomic associations after limiting the semantic type of clinical concepts to be “Disease or Syndrome”.

Figure S7

Gephi outputs of the remaining clinic-genomic associations after limiting the semantic type of clinical concepts to be “Finding”.

Figure S8

Gephi outputs of the remaining clinic-genomic associations after limiting the semantic type of clinical concepts to be “Immunologic Factor”.

Figure S9

Gephi outputs of the remaining clinic-genomic associations after limiting the semantic type of clinical concepts to be “Injury or Poisoning”.

Figure S10

Gephi outputs of the remaining clinic-genomic associations after limiting the semantic type of clinical concepts to be "Laboratory or Test Result".

Figure S11

Gephi outputs of the remaining clinic-genomic associations after limiting the semantic type of clinical concepts to be "Laboratory Procedure".

Figure S12

Gephi outputs of the remaining clinic-genomic associations after limiting the semantic type of clinical concepts to be "Mental or Behavioral Dysfunction".

Figure S13

Gephi outputs of the remaining clinic-genomic associations after limiting the semantic type of clinical concepts to be "Natural Phenomenon or Process".

Figure S14

Gephi outputs of the remaining clinic-genomic associations after limiting the semantic type of clinical concepts to be "Neoplastic Process".

Figure S15

Gephi outputs of the remaining clinic-genomic associations after limiting the semantic type of clinical concepts to be "Pathologic Function".

Figure S16

Gephi outputs of the remaining clinic-genomic associations after limiting the semantic type of clinical concepts to be "Phenomenon or Process".

Figure S17

Gephi outputs of the remaining clinic-genomic associations after limiting the semantic type of clinical concepts to be "Sign or Symptom".

Figure S18

Figure S18 is produced by Gephi, displaying the overall view of clinic-genomic associations mined out by the proposed method.

Figure S1

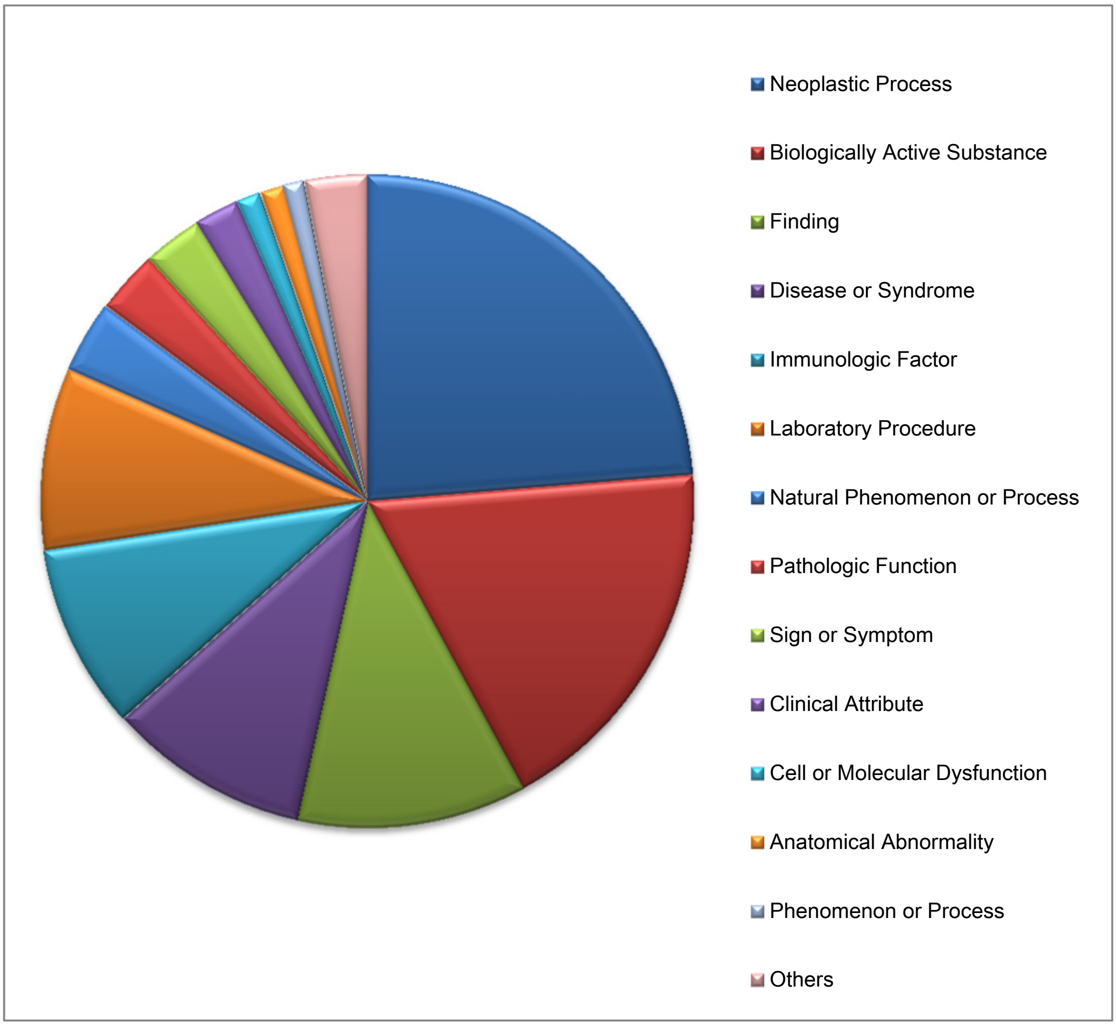
Neoplastic Process
Biologically Active Substance
Finding
Disease or Syndrome
Immunologic Factor
Laboratory Procedure
Natural Phenomenon or Process
Pathologic Function
Sign or Symptom
Clinical Attribute
Cell or Molecular Dysfunction
Anatomical Abnormality
Phenomenon or Process
Others

Figure S2

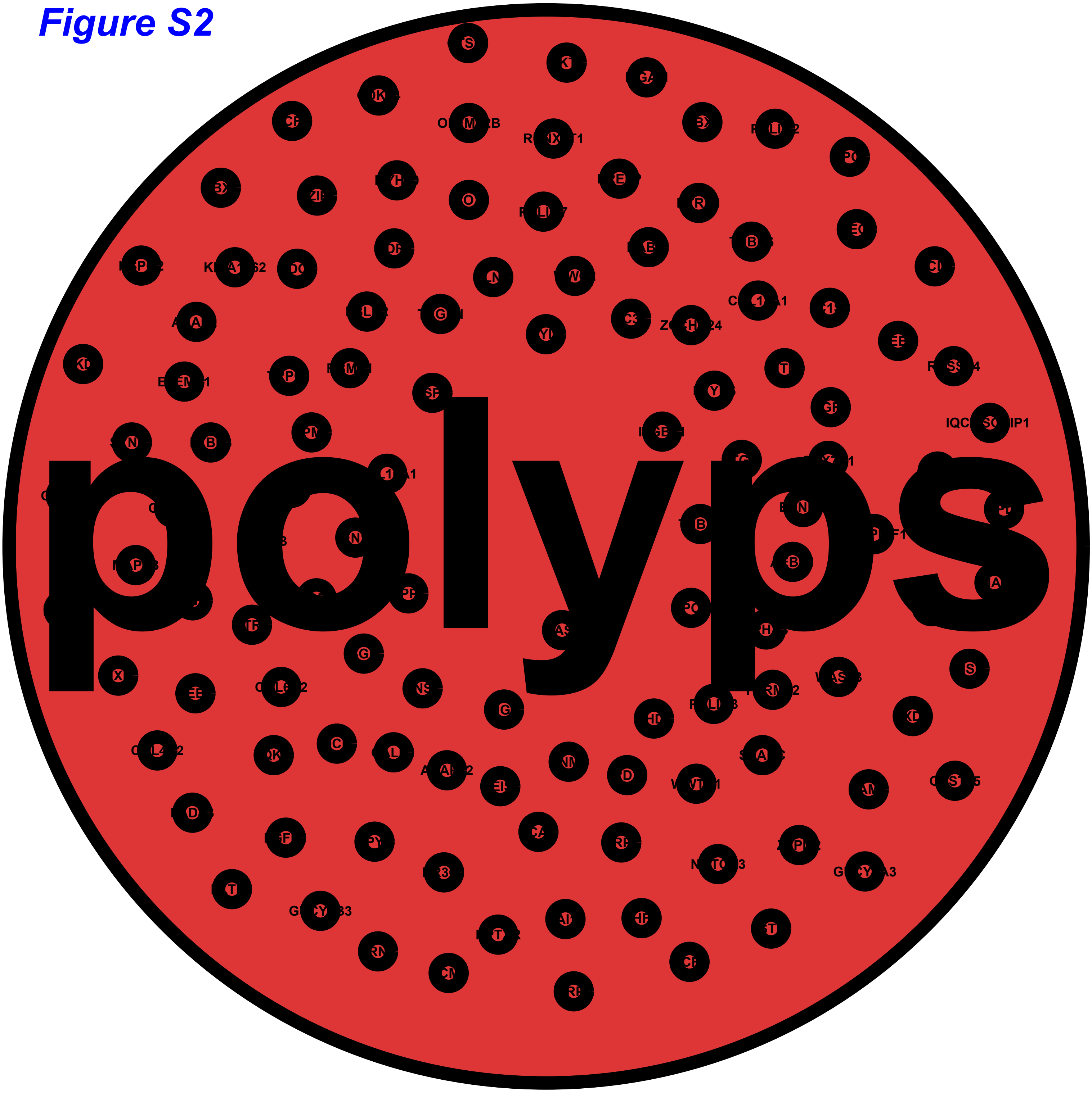

Figure S3

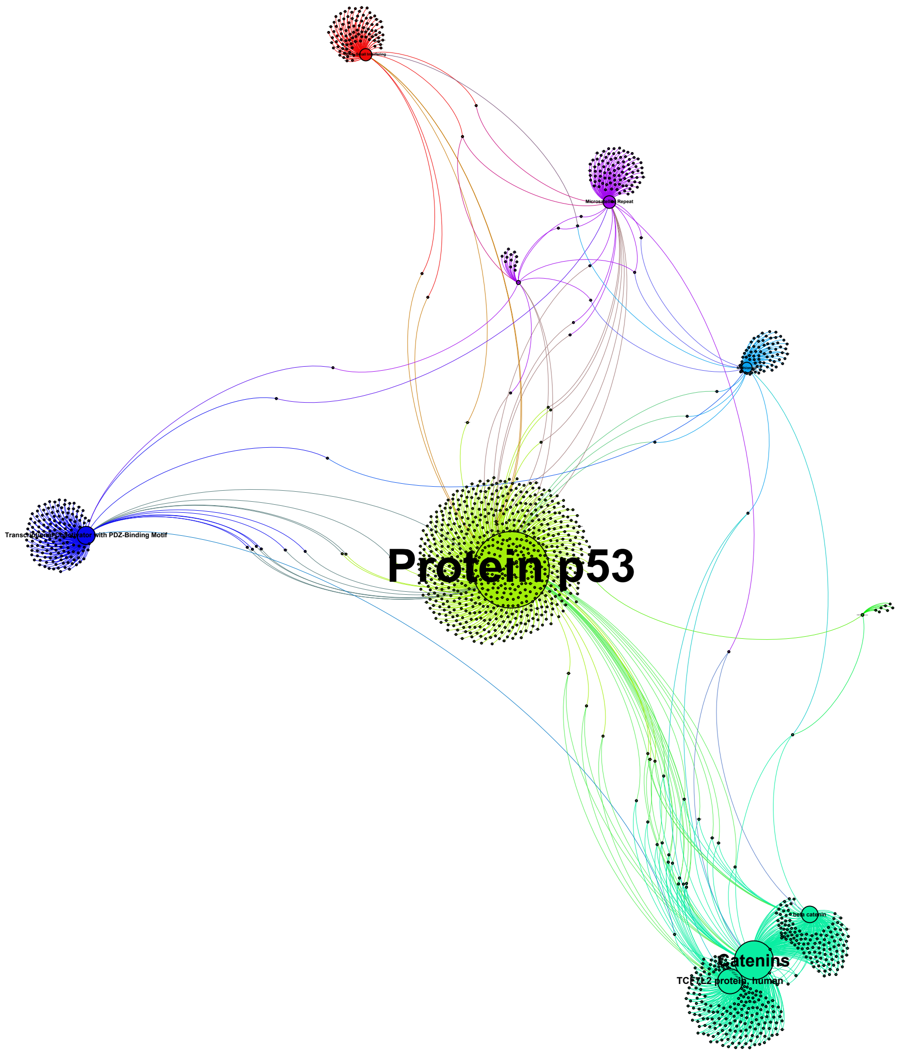

1: Dominant-Negative Mutation

Figure S4

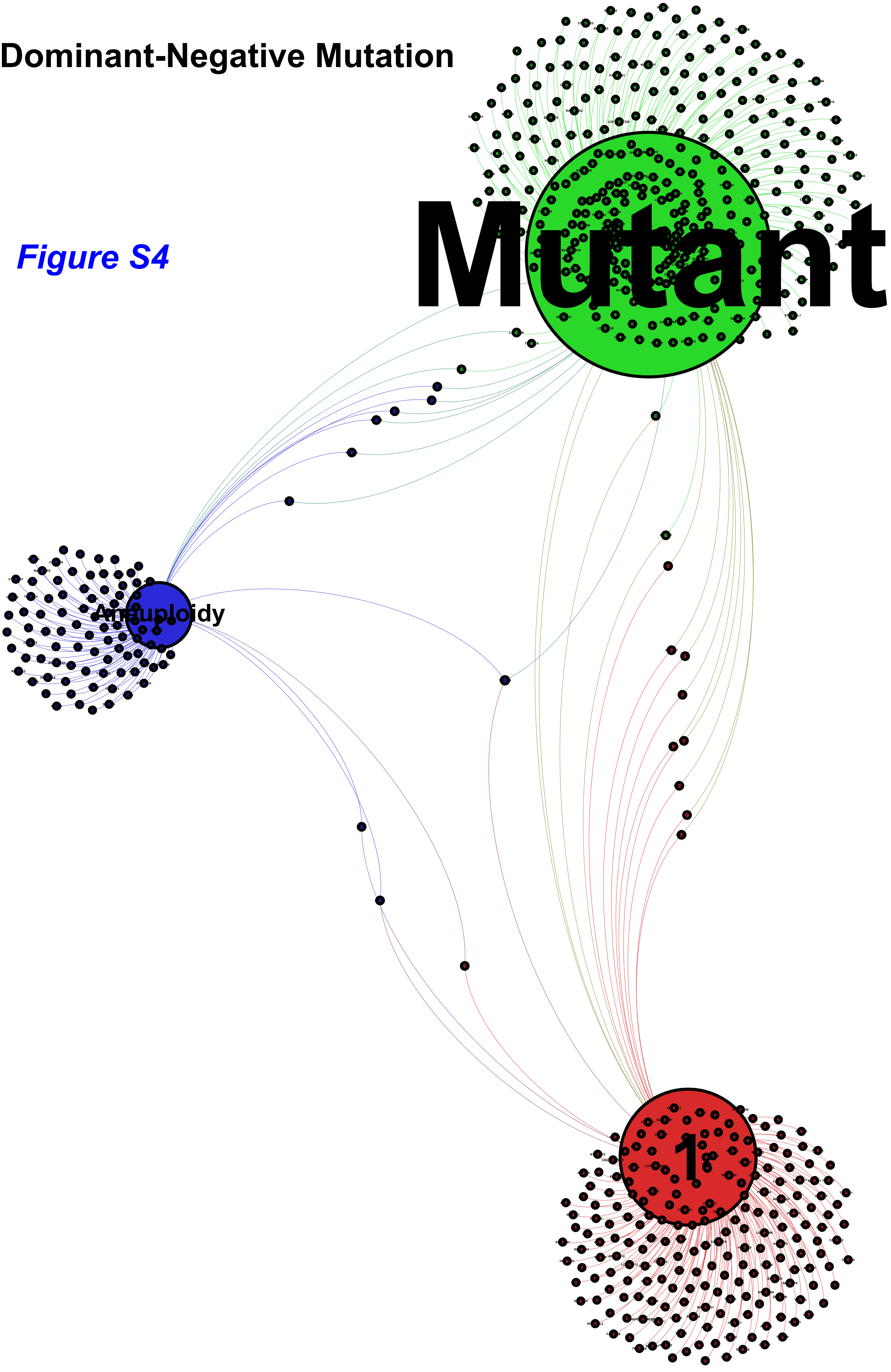

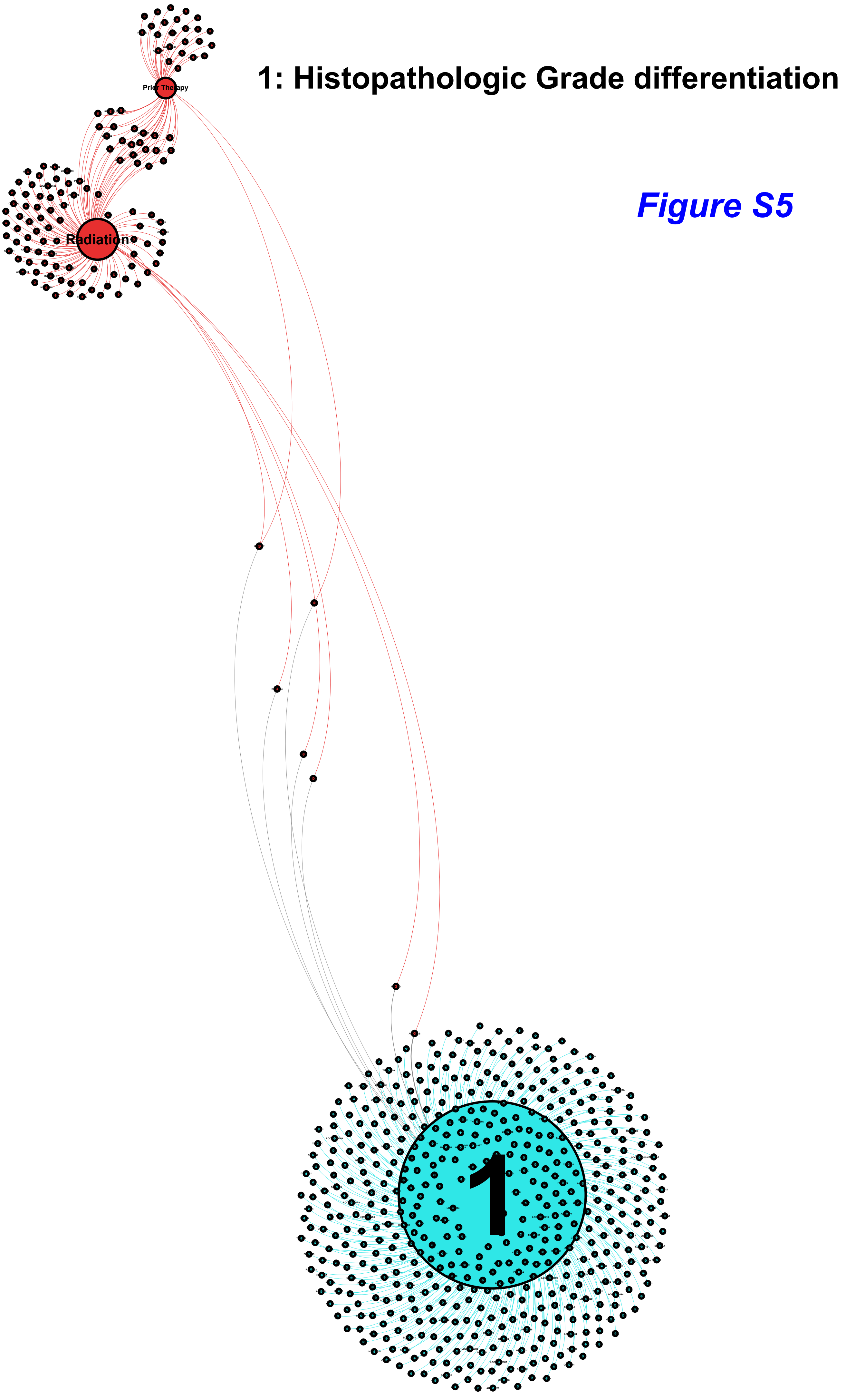
1: Histopathologic Grade differentiation
Prior Therapy
Radiation
1

Figure S5

1: Disease

Figure S6

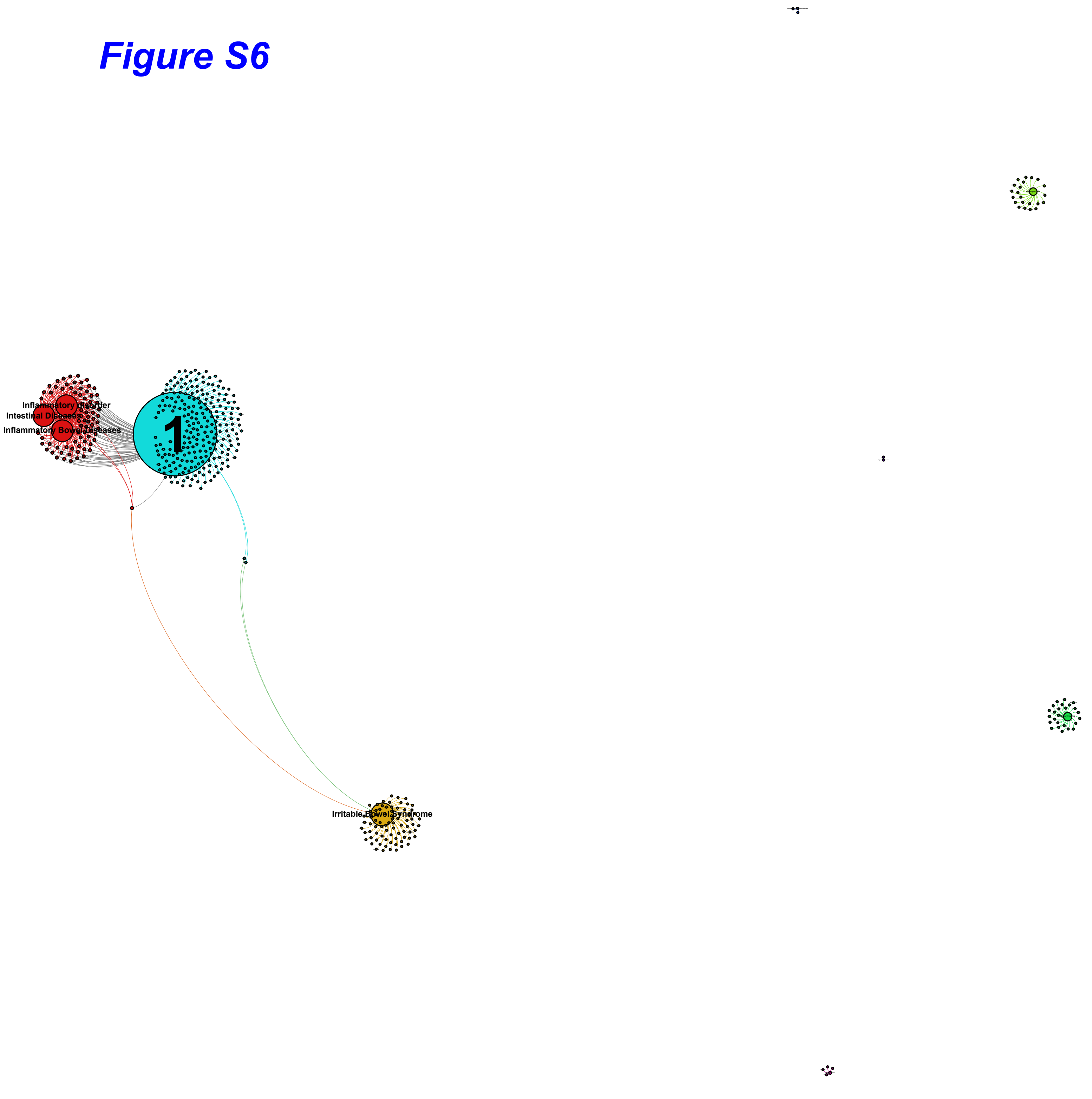

Figure S7

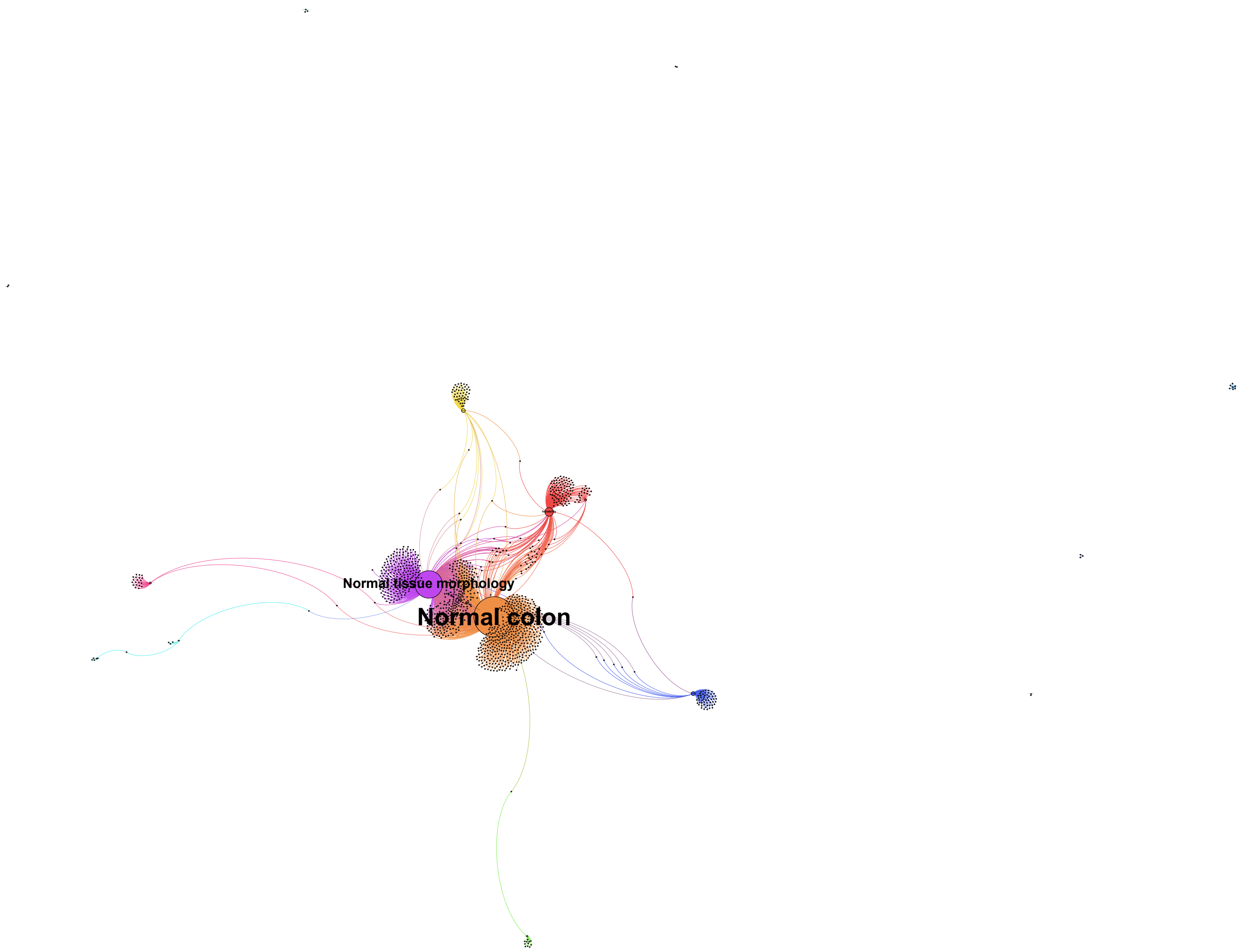

Figure S8

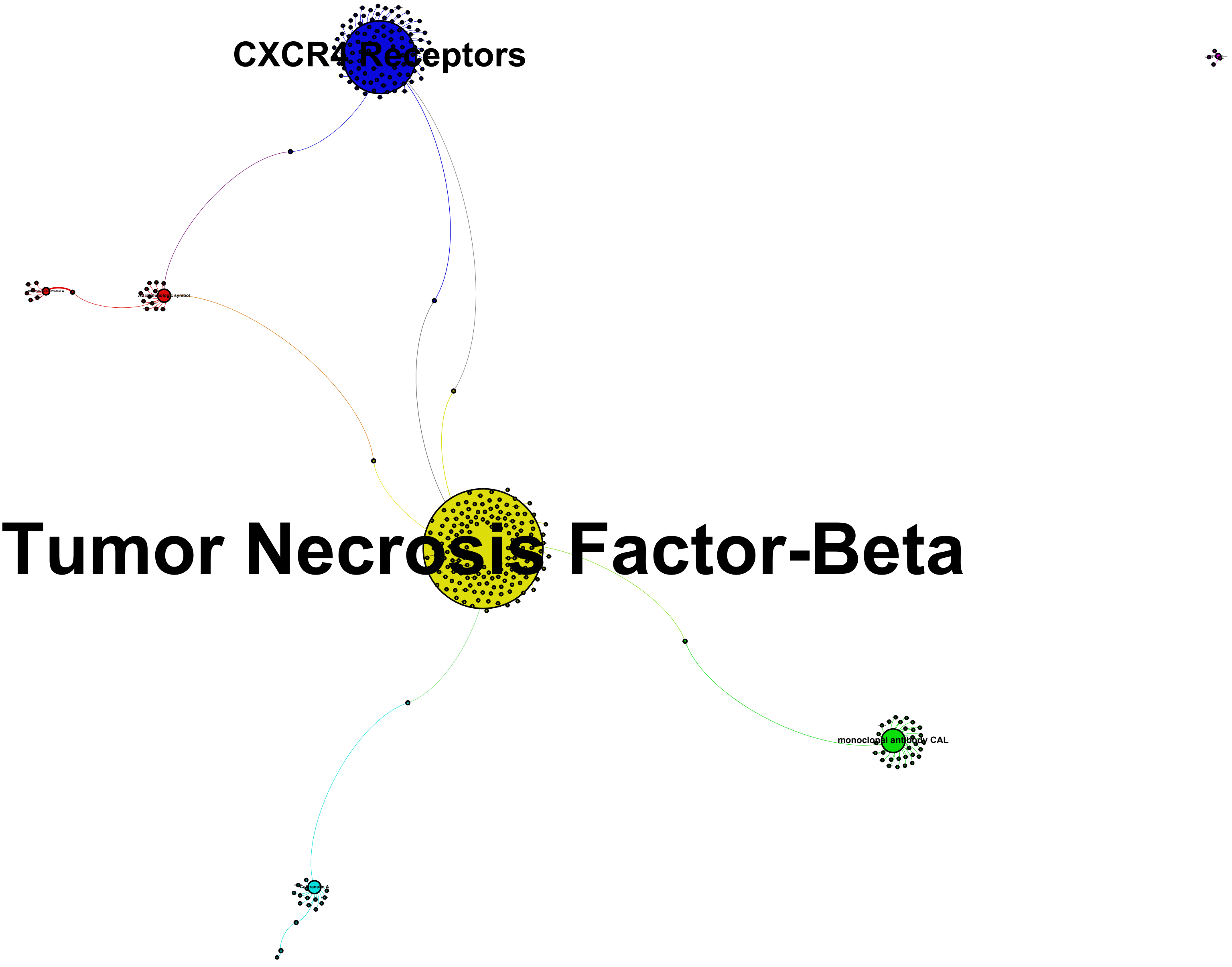
CXCR4 Receptors
Tumor Necrosis Factor-Beta
monoclonal antibody CAL

1: Incised wound

Figure S9

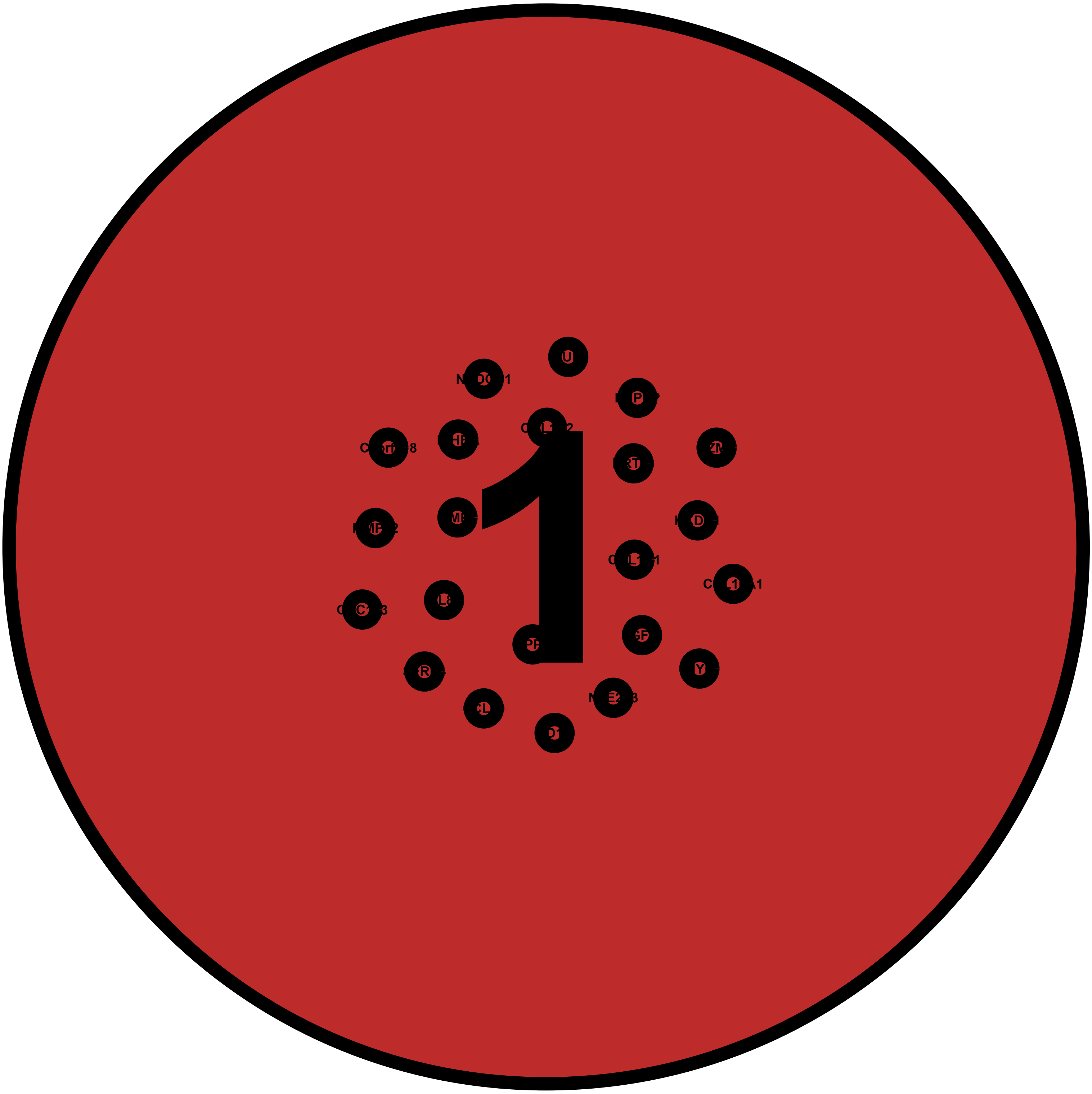

1: Human cells

Figure S10

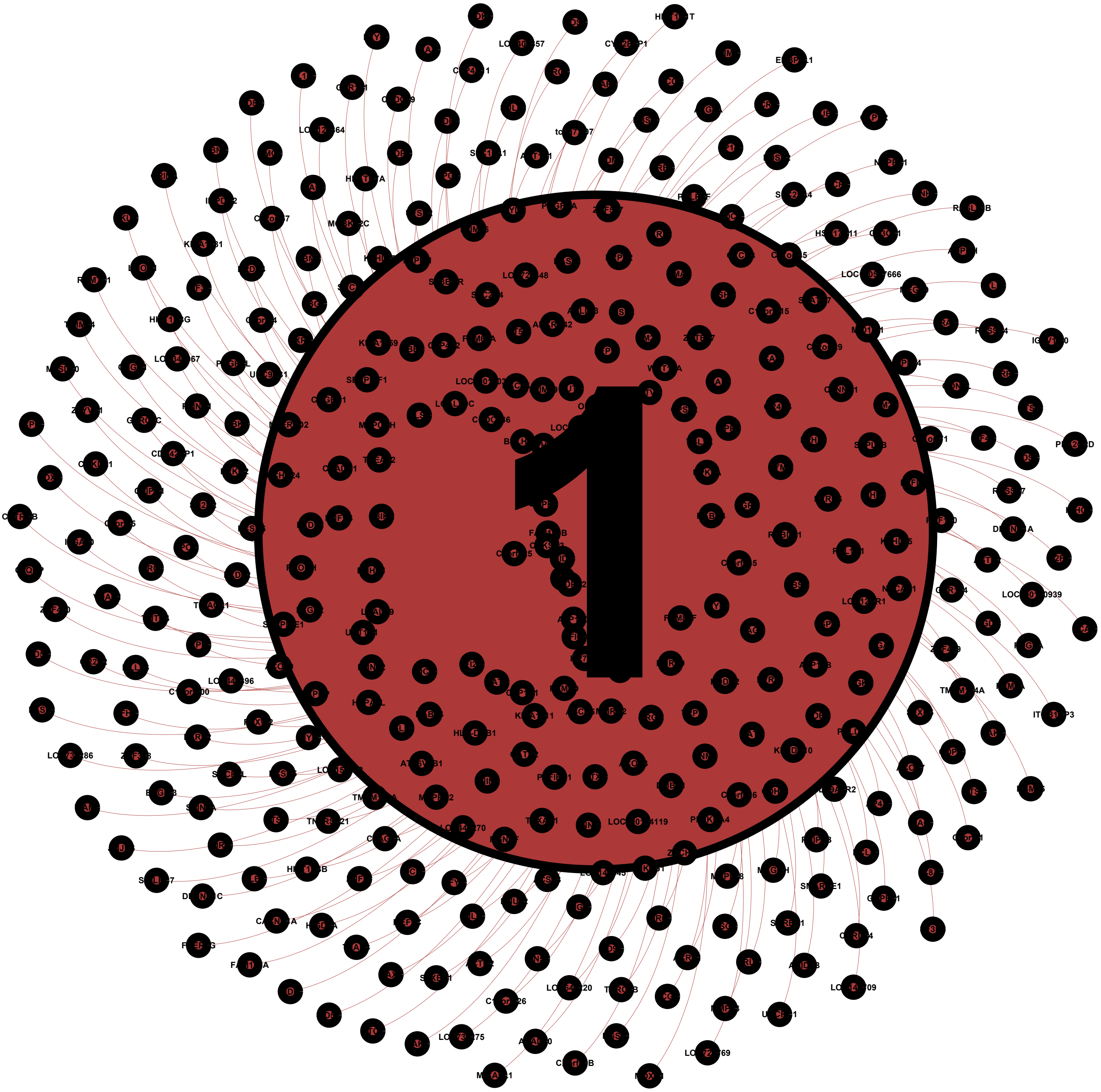

Figure S11

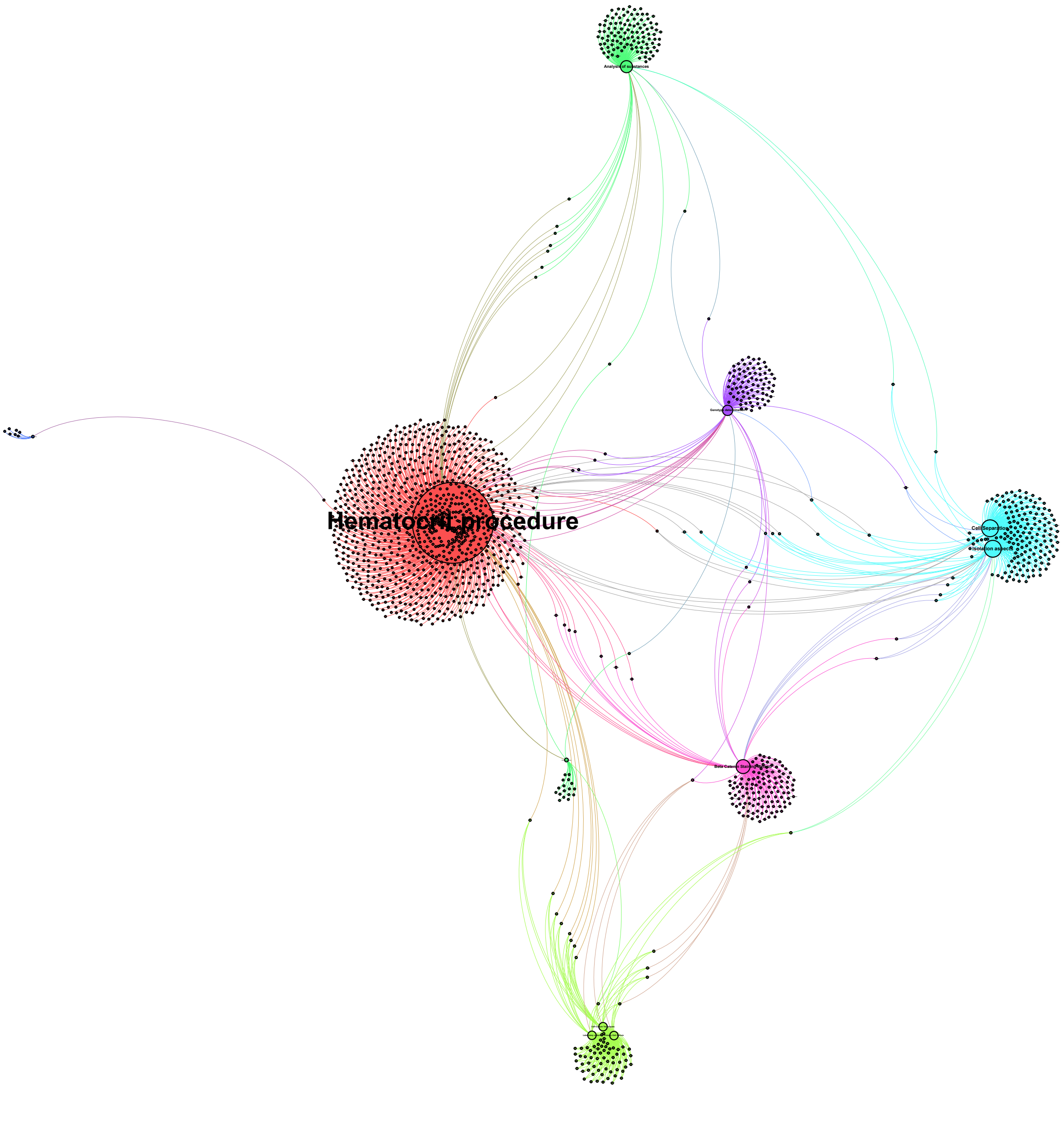
Analysis of substances
Genotype determination
Hematocrit procedure
Cell Separation
Isolation aspects
Beta Catenin Staining

1: Schizophrenia

Figure S12

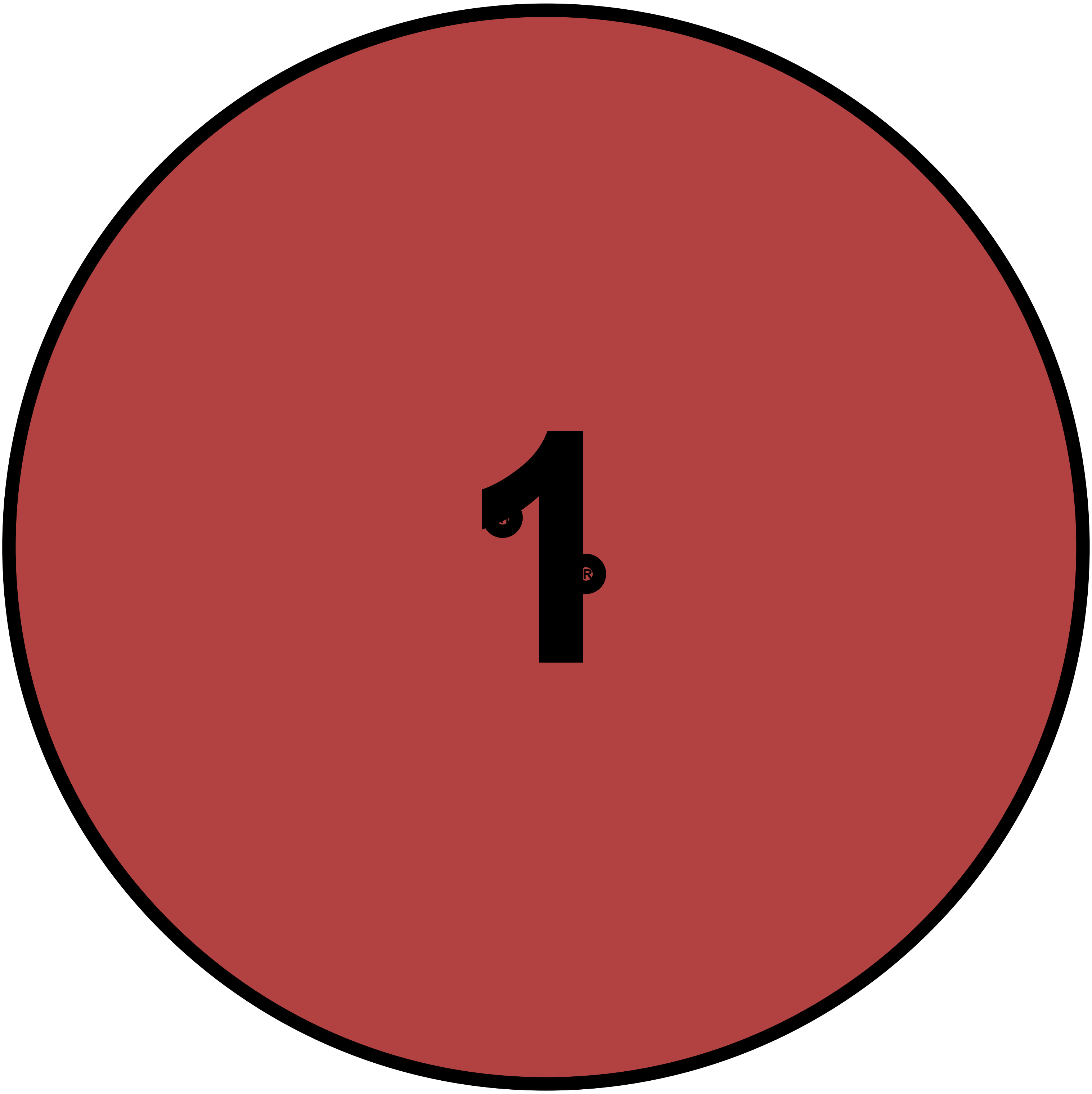

1: Electromagnetic Energy
2: Radiation

Figure S13

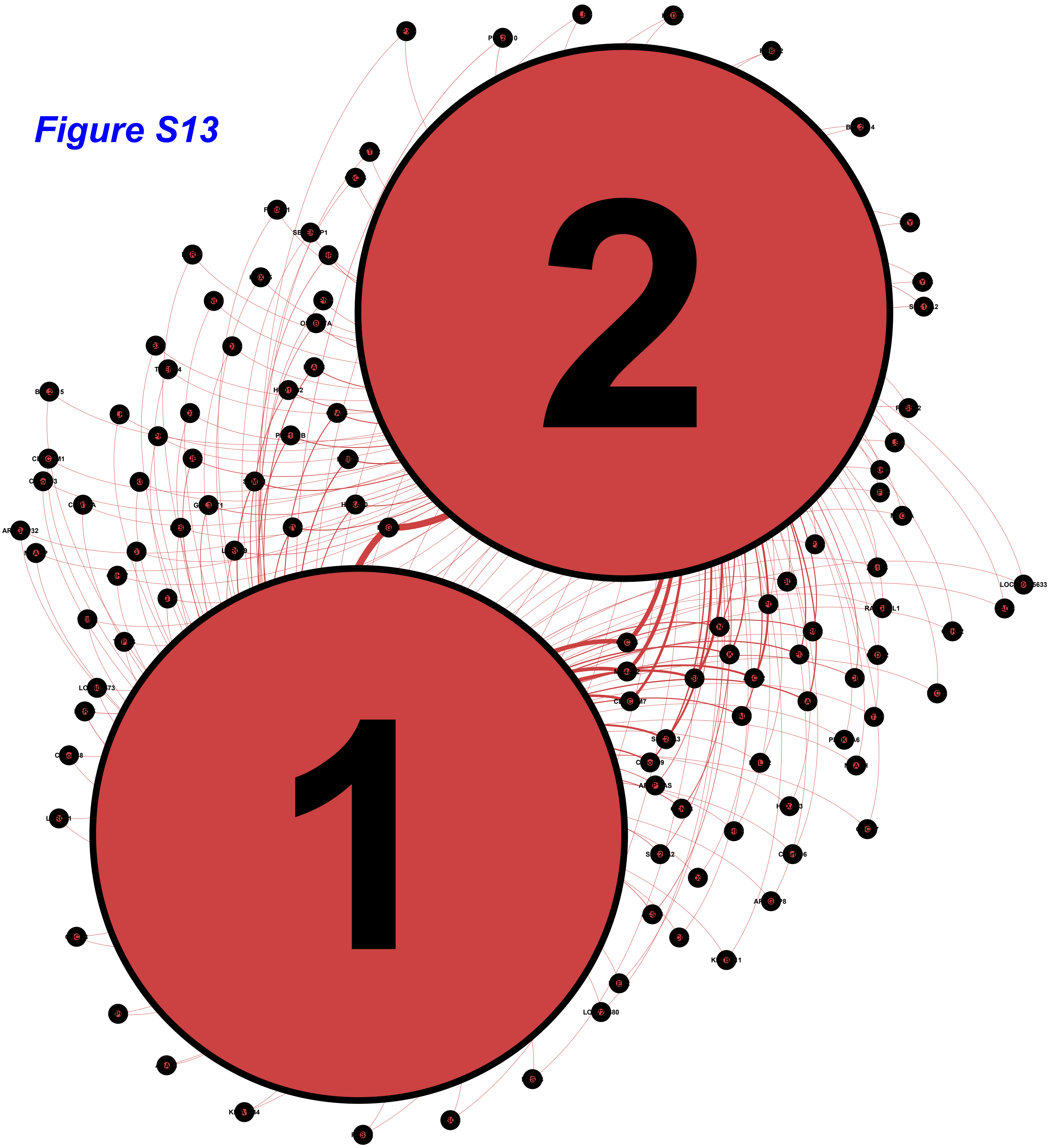

Figure S14

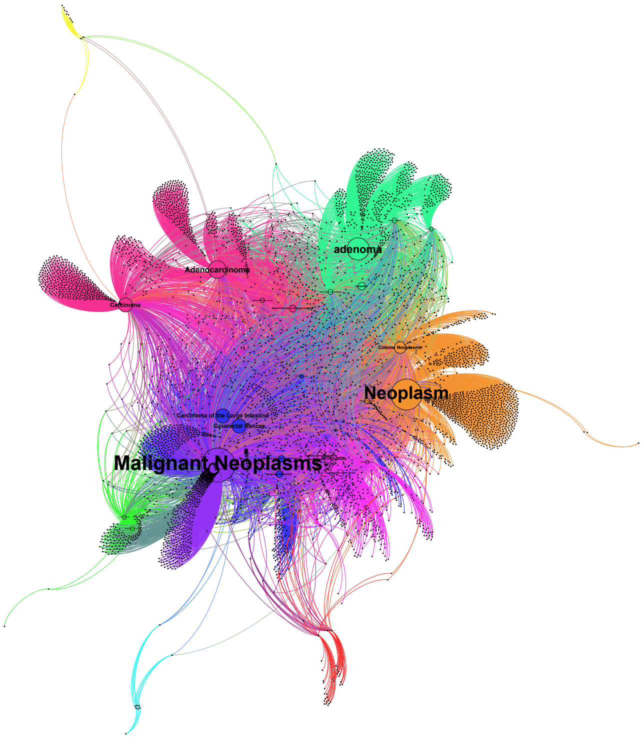
adenoma
Adenocarcinoma
Carcinoma
Colonic Neoplasms
Neoplasm
Carcinoma of the Large Intestine
Colorectal Cancer
Malignant Neoplasms

Figure S15

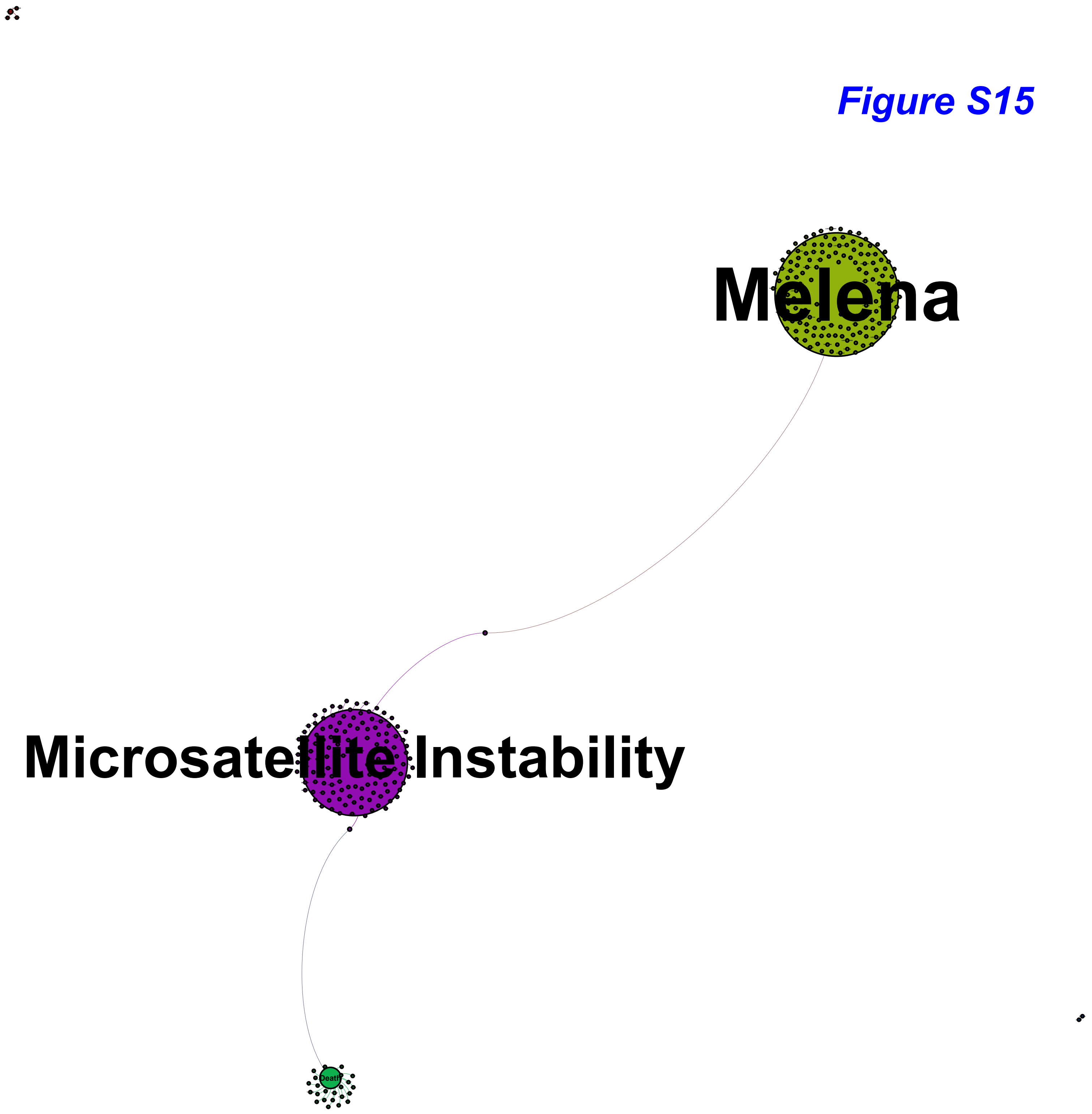

Figure S16

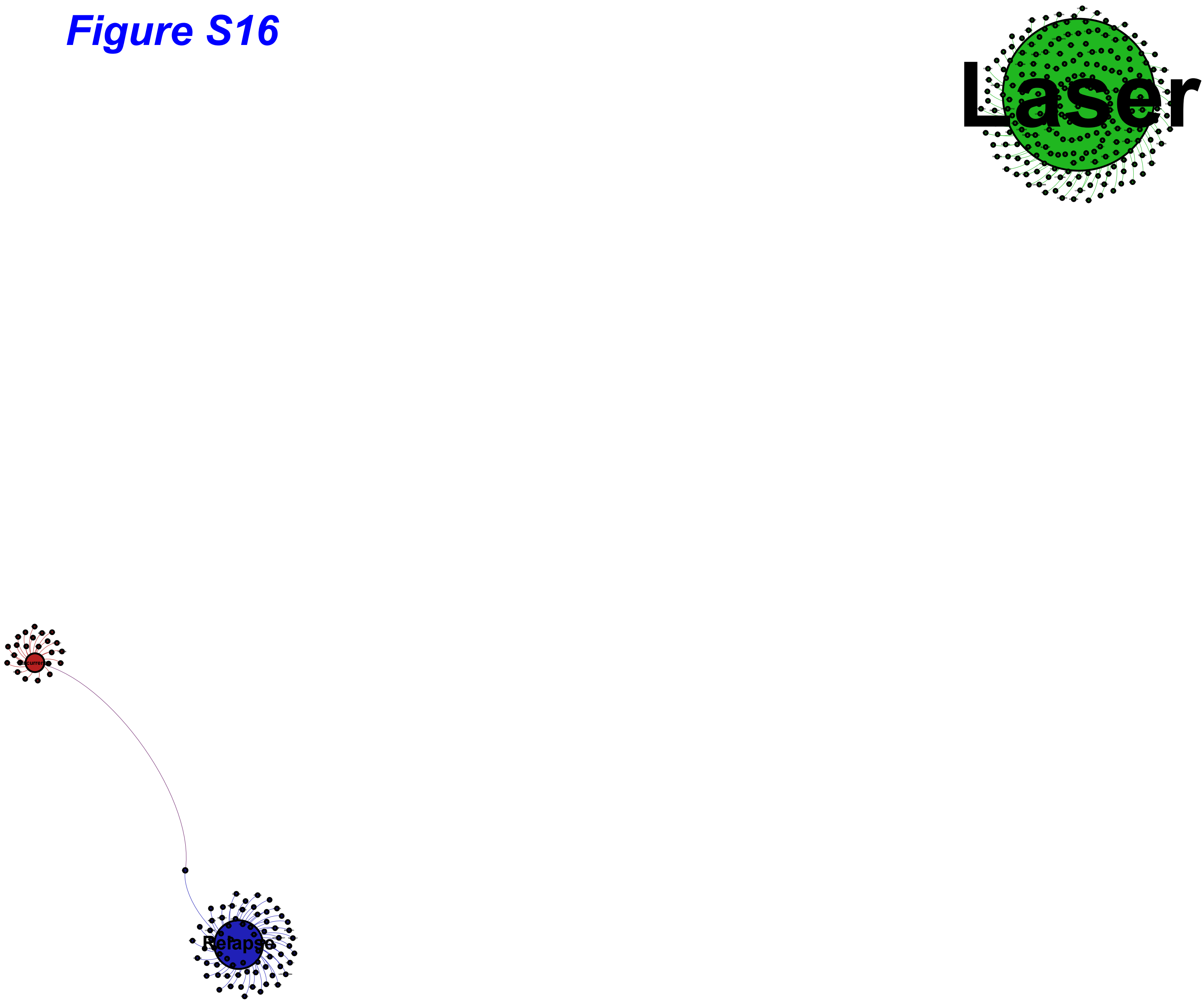

Figure S17

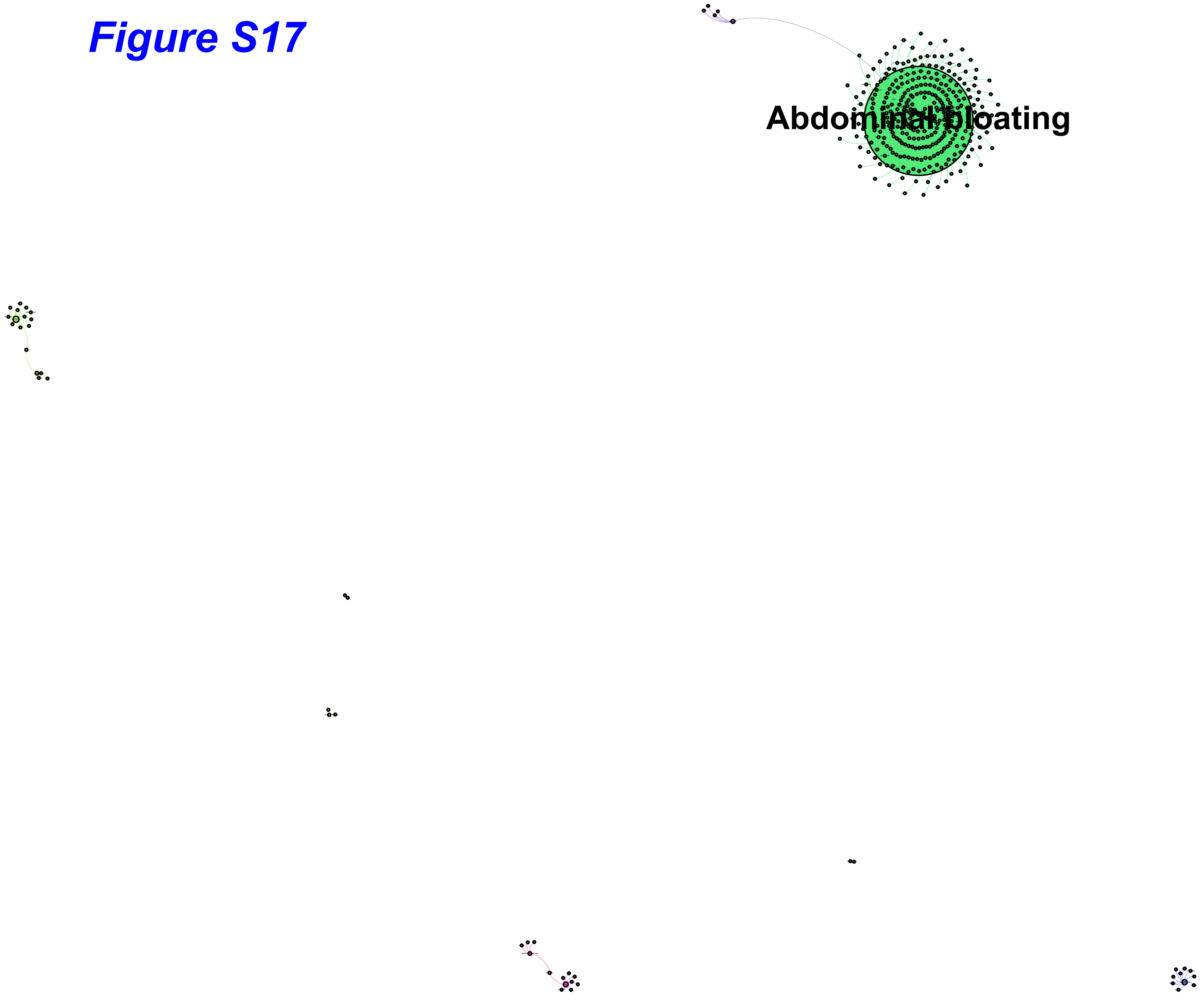

Figure S18

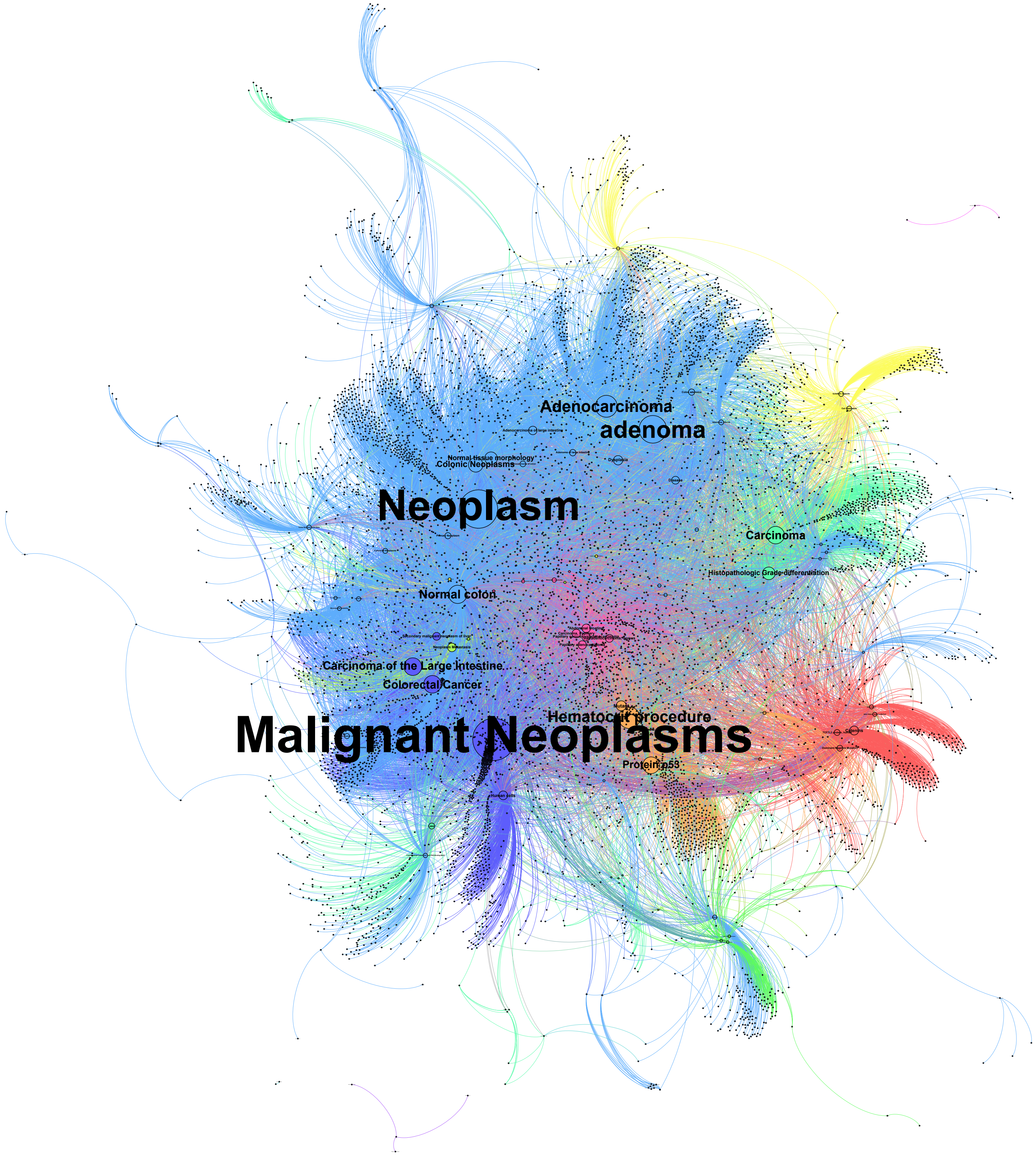
Adenocarcinoma
adenoma
Normal tissue morphology
Colonic Neoplasms
Neoplasm
Carcinoma
Histopathologic Grade differentiation
Normal colon
Carcinoma of the Large Intestine
Colorectal Cancer
Hematocrit procedure
Malignant Neoplasms
Protein p53

Table S1 - Filter rule used to search GEO for colorectal cancer related GSE

Logical operator	Search Field	Key Words
---	All Fields	colon neoplasms
OR	All Fields	rectal neoplasms
OR	All Fields	colorectal neoplasms
OR	All Fields	colon cancer
OR	All Fields	rectal cancer
OR	All Fields	colorectal cancer
OR	All Fields	colon tumor
OR	All Fields	rectal tumor
OR	All Fields	colorectal tumor
OR	All Fields	colon adenoma
OR	All Fields	rectal adenoma
OR	All Fields	colorectal adenoma
OR	All Fields	colon carcinoma
OR	All Fields	rectal carcinoma
OR	All Fields	colorectal carcinoma
OR	All Fields	colon adenocarcinoma
OR	All Fields	rectal adenocarcinoma
OR	All Fields	colorectal adenocarcinoma
AND	Organism	Homo sapiens

Table S2 - Colorectal cancer related keywords used to search GAD

colon neoplasms;	colorectal neoplasms;	rectal neoplasms;
colon cancer;	colorectal cancer;	rectal cancer;
colon adenoma;	colorectal adenoma;	rectal adenoma;
colon carcinoma;	colorectal carcinoma;	rectal carcinoma;
colon adenocarcinoma;	colorectal adenocarcinoma;	rectal adenocarcinoma;
colon polyps;	colorectal polyps;	rectal polyps;
Crohn’s disease;	adenomatous polyposis coli;	inflammatory bowel disease;
Ulcerative colitis and colitis, ulcerative.		

Table S3 - Clinical related semantic types

Neoplastic Process	Finding
Pathologic Function	Phenomenon or Process
Cell or Molecular Dysfunction	Congenital Abnormality
Anatomical Abnormality	Acquired Abnormality
Immunologic Factor	Biologically Active Substance
Disease or Syndrome	Natural Phenomenon or Process
Laboratory or Test Result	Sign or Symptom
Mental or Behavioural Dysfunction	Injury or Poisoning
Experimental Model of Disease	Biologic Function
Laboratory Procedure	Clinical Attribute

Table S4 - Logarithmic scale detection algorithm

```
if(mean(abs(sample_value))<16)
    NeedLogC = false;
else
    quantile_value = [0., 0.25, 0.5, 0.75, 0.99, 1.0];
    Qt = quantile(sample_value,quantile_value);
    NeedLogC = (Qt(5) > 100)|| (Qt(6) -Qt(1) > 50 && Qt(2) > 0)||(Qt(2) > 0
&& Qt(2) < 1 && Qt(4) > 1 && Qt(4) < 2);
end
```

Table S5 - Top 10 pathways with the most number of genes

hsa01100 Metabolic pathways - Homo sapiens (human) (556)
hsa05200 Pathways in cancer - Homo sapiens (human) (216)
hsa04151 PI3K-Akt signaling pathway - Homo sapiens (human) (205)
hsa05166 HTLV-I infection - Homo sapiens (human) (176)
hsa04060 Cytokine-cytokine receptor interaction - Homo sapiens (human) (158)
hsa05205 Proteoglycans in cancer - Homo sapiens (human) (143)
hsa04510 Focal adhesion - Homo sapiens (human) (137)
hsa04010 MAPK signaling pathway - Homo sapiens (human) (133)
hsa05169 Epstein-Barr virus infection - Homo sapiens (human) (130)
hsa04014 Ras signaling pathway - Homo sapiens (human) (126)

Table S6

Index	CUI	Concept Name	Semantic Type	GSM Count	GSE Count	Association
1	C0278008	[D]Change in bowel habit	Sign or Symptom	16	2	Y
2	C0334277	[M]Adenocarcinoma, metastatic, NOS	Neoplastic Process	54	3	Y
3	C1443036	A2 immunologic symbol	Immunologic Factor	6	3	Y
4	C1291077	Abdominal bloating	Sign or Symptom	5	1	Y
5	C0000833	Abscess	Pathologic Function	5	2	Y
6	C0001418	Adenocarcinoma	Neoplastic Process	2403	86	Y
7	C0338106	Adenocarcinoma of colon	Neoplastic Process	706	24	Y
8	C1319315	Adenocarcinoma of large intestine	Neoplastic Process	581	34	Y
9	C0007130	Adenocarcinoma, Mucinous	Neoplastic Process	103	4	Y
10	C0001430	adenoma	Neoplastic Process	516	24	Y
11	C1302401	Adenoma of large intestine	Neoplastic Process	34	3	Y
12	C0206674	Adenoma, Villous	Neoplastic Process	23	2	Y
13	C0002778	Analysis of substances	Laboratory Procedure	1025	27	Y
14	C0002938	Aneuploidy	Cell or Molecular Dysfunction	99	2	Y
15	C0002962	Angina Pectoris	Sign or Symptom	5	2	Y
16	C0549483	Bacterial Abscess	Finding	5	2	Y
17	C0105770	beta catenin	Biologically Active Substance	6	3	Y
18	C1527145	Beta Catenin Staining Method	Laboratory Procedure	6	3	Y
19	C0853879	Breast cancer invasive NOS	Neoplastic Process	6	1	Y
20	C0678222	Breast Carcinoma	Neoplastic Process	36	6	Y
21	C1272329	Bright red color	Finding	147	1	Y
22	C0006675	Calcium	Biologically Active Substance	5	1	Y
23	C0054504	Calgranulin A	Immunologic Factor	7	1	Y
24	C0346627	Cancer of Intestines	Neoplastic Process	8	2	Y
25	C0007113	Cancer of Rectum	Neoplastic Process	570	16	Y
26	C0007097	Carcinoma	Neoplastic Process	1554	94	Y
27	C0009402	Carcinoma of the Large Intestine	Neoplastic Process	2559	131	Y
28	C1134719	Carcinoma, Ductal, Breast	Neoplastic Process	6	1	Y
29	C0007133	Carcinoma, Papillary	Neoplastic Process	7	2	Y
30	C1564904	Catenins	Biologically Active Substance	14	5	Y

31	C0007585	Cell Culture Techniques	Laboratory Procedure	26	6	Y
32	C0007616	Cell Separation	Laboratory Procedure	20	1	Y
33	C0684295	Chemical extraction	Laboratory Procedure	276	20	Y
34	C0850572	Colon adenoma	Neoplastic Process	63	3	Y
35	C0699790	Colon Carcinoma	Neoplastic Process	2497	147	Y
36	C0009375	Colonic Neoplasms	Neoplastic Process	915	27	Y
37	C1527249	Colorectal Cancer	Neoplastic Process	2223	92	Y
38	C0009404	Colorectal Neoplasms	Neoplastic Process	748	23	Y
39	C0009806	Constipation	Sign or Symptom	65	1	Y
40	C0250501	CXCR4 Receptors	Immunologic Factor	12	1	Y
41	C0206701	Cystadenocarcinoma, Serous	Neoplastic Process	18	3	Y
42	C1306577	Death	Finding	570	7	Y
43	C0011860	Diabetes Mellitus, Non-Insulin-Dependent	Disease or Syndrome	73	2	Y
44	C0011991	Diarrhea	Sign or Symptom	97	3	Y
45	C0012634	Disease	Disease or Syndrome	3557	75	Y
46	C1512032	Dominant-Negative Mutation	Cell or Molecular Dysfunction	6	1	Y
47	C1527349	Ductal Breast Carcinoma	Neoplastic Process	19	2	Y
48	C1176475	Ductal Carcinoma	Neoplastic Process	19	2	Y
49	C0334044	Dysplasia	Neoplastic Process	34	2	Y
50	C0034519	Electromagnetic Energy	Natural Phenomenon or Process	76	12	Y
51	C1363945	Encounter due to therapy	Finding	402	5	Y
52	C0577015	Esophagus normal	Finding	8	1	Y
53	C0202010	Fecal analysis procedure	Laboratory Procedure	157	1	Y
54	C0243095	Finding	Sign or Symptom	138	7	Y
55	C0206682	Follicular thyroid carcinoma	Neoplastic Process	6	1	Y
56	C1285573	Genotype determination	Laboratory Procedure	386	33	Y
57	C0018935	Hematocrit procedure	Laboratory Procedure	98	30	Y
58	C0019080	Hemorrhage	Finding	9	2	Y
59	C1333600	Hereditary Malignant Neoplasm	Neoplastic Process	62	4	Y
60	C1333990	Hereditary Non-Polyposis Colon Cancer Type 1	Neoplastic Process	78	3	Y
61	C0009405	Hereditary Nonpolyposis Colorectal Neoplasms	Neoplastic Process	99	4	Y
62	C1511938	Histopathologic Grade differentiation	Clinical Attribute	243	11	Y

63	C0379832	HMGA1b Protein	Biologically Active Substance	6	1	Y
64	C0969693	HMGA1c Protein	Biologically Active Substance	6	1	Y
65	C0427861	Human cells	Laboratory or Test Result	700	46	Y
66	C0000925	Incised wound	Injury or Poisoning	39	1	Y
67	C1299583	Independently able	Finding	96	5	Y
68	C1412014	Infiltrating duct carcinoma	Neoplastic Process	6	1	Y
69	C0332448	Infiltration	Pathologic Function	47	4	Y
70	C0021390	Inflammatory Bowel Diseases	Disease or Syndrome	15	1	Y
71	C1290884	Inflammatory disorder	Disease or Syndrome	15	1	Y
72	C1444783	Instability	Finding	907	9	Y
73	C0021831	Intestinal Diseases	Disease or Syndrome	15	1	Y
74	C0022104	Irritable Bowel Syndrome	Disease or Syndrome	60	3	Y
75	C0220862	isolation aspects	Laboratory Procedure	138	7	Y
76	C0430400	Laboratory culture	Laboratory Procedure	79	18	Y
77	C0458142	Laser	Phenomenon or Process	77	5	Y
78	C0023903	Liver neoplasms	Neoplastic Process	29	1	Y
79	C1140680	Malignant neoplasm of ovary	Neoplastic Process	4	1	Y
80	C0376358	Malignant neoplasm of prostate	Neoplastic Process	59	3	Y
81	C0007115	Malignant neoplasm of thyroid	Neoplastic Process	6	1	Y
82	C0006826	Malignant Neoplasms	Neoplastic Process	6147	251	Y
83	C0007102	Malignant tumor of colon	Neoplastic Process	2401	130	Y
84	C0806909	Maximum	Finding	10	2	Y
85	C0025222	Melena	Pathologic Function	17	1	Y
86	C1384494	Metastatic Carcinoma	Neoplastic Process	14	2	Y
87	C0025646	Methionine	Biologically Active Substance	52	4	Y
88	C0920269	Microsatellite Instability	Pathologic Function	547	6	Y
89	C0282522	Microsatellite Repeat	Biologically Active Substance	895	14	Y
90	C0581124	Mild asthma	Finding	4	1	Y
91	C1135160	monoclonal antibody CAL	Immunologic Factor	58	2	Y
92	C0260037	Multiple tumors	Neoplastic Process	269	1	Y
93	C0596988	Mutant	Cell or Molecular Dysfunction	318	18	Y
94	C0151744	Myocardial Ischemia	Disease or Syndrome	5	2	Y

95	C0027497	Nausea	Sign or Symptom	30	1	Y
96	C1334927	Neck Carcinoma	Neoplastic Process	6	1	Y
97	C0027651	Neoplasm	Neoplastic Process	6094	133	Y
98	C0027627	Neoplasm Metastasis	Neoplastic Process	1231	38	Y
99	C0940500	Normal colon	Finding	913	47	Y
100	C0577035	Normal rectum	Finding	28	1	Y
101	C0332441	Normal tissue morphology	Finding	564	25	Y
102	C0029456	Osteoporosis	Disease or Syndrome	6	1	Y
103	C1563292	Osteoporosis family history	Finding	6	1	Y
104	C0029925	Ovarian Carcinoma	Neoplastic Process	30	3	Y
105	C1441040	P1	Immunologic Factor	66	2	Y
106	C1557397	PAIN	Sign or Symptom	192	6	Y
107	C0001420	Papillary adenocarcinoma	Neoplastic Process	23	5	Y
108	C0334359	Papillary serous cystadenocarcinoma	Neoplastic Process	16	2	Y
109	C0032584	polyps	Anatomical Abnormality	141	5	Y
110	C1449562	Primary Cell Culture	Laboratory Procedure	7	1	Y
111	C0677930	Primary Neoplasm	Neoplastic Process	514	11	Y
112	C1514463	Prior Therapy	Clinical Attribute	61	1	Y
113	C0600139	Prostate carcinoma	Neoplastic Process	398	7	Y
114	C0080055	Protein p53	Biologically Active Substance	824	25	Y
115	C0851346	Radiation	Clinical Attribute<br>Natural Phenomenon or Process	82	13	Y
116	C0034897	Recurrence	Phenomenon or Process	844	9	Y
117	C0278012	Red stools	Sign or Symptom	147	1	Y
118	C0332575	Redness	Finding	171	2	Y
119	C0035020	Relapse	Phenomenon or Process	225	5	Y
120	C0035668	RNA	Biologically Active Substance	2803	70	Y
121	C1099354	RNA, Small Interfering	Biologically Active Substance	229	18	Y
122	C0389073	S100A12 protein, human	Biologically Active Substance	4	1	Y
123	C0036341	Schizophrenia	Mental or Behavioral Dysfunctio	27	1	Y
124	C0947630	Scientific Study	Laboratory Procedure	472	11	Y
125	C0494165	Secondary malignant neoplasm of liver	Neoplastic Process	238	12	Y

126	C0153676	Secondary malignant neoplasm of lung	Neoplastic Process	20	1	Y
127	C0038164	Staphylococcal Protein A	Immunologic Factor	7	2	Y
128	C0038354	Stomach Diseases	Disease or Syndrome	11	2	Y
129	C0577026	Stomach normal	Finding	6	1	Y
130	C0039082	Syndrome	Disease or Syndrome	84	4	Y
131	C0245557	TACSTD2 protein, human	Immunologic Factor	6	1	Y
132	C1336781	TCF7L2 protein, human	Biologically Active Substance	10	3	Y
133	C0456984	Test Result	Finding	202	8	Y
134	C0549473	Thyroid carcinoma	Neoplastic Process	6	1	Y
135	C0558353	Tongue Carcinoma	Neoplastic Process	4	1	Y
136	C1336798	Transcriptional Coactivator with PDZ-Binding Motif	Biologically Active Substance	4	2	Y
137	C0024320	Tumor Necrosis Factor-Beta	Immunologic Factor	11	1	Y
138	C0042963	Vomiting	Sign or Symptom	22	1	Y
139	C1306888	White color finding	Finding	82	6	Y
140	C0049308	5-Methylcytosine	Biologically Active Substance	3	1	
141	C1443037	A3 immunologic symbol	Immunologic Factor	1	2	
142	C0000734	Abdominal mass	Finding	2	1	
143	C0000737	Abdominal Pain	Sign or Symptom	120	3	
144	C0332472	abnormal fragmented structure	Anatomical Abnormality	18	4	
145	C0001175	Acquired Immunodeficiency Syndrome	Disease or Syndrome	1	1	
146	C0427611	ACT	Laboratory Procedure	99	3	
147	C0085669	Acute leukemia	Neoplastic Process	364	6	
148	C0023493	Acute T Cell Leukemia	Neoplastic Process	339	4	
149	C0334276	Adenocarcinoma in situ	Neoplastic Process	2	1	
150	C0279628	Adenocarcinoma of esophagus	Neoplastic Process	2	1	
151	C0007112	Adenocarcinoma of prostate	Neoplastic Process	6	1	
152	C0206681	Adenocarcinoma, Clear Cell	Neoplastic Process	2	1	
153	C0205645	Adenocarcinoma, Tubular	Neoplastic Process	40	2	
154	C1302652	Adenoma of rectum	Neoplastic Process	36	4	
155	C0032580	Adenomatous Polyposis Coli	Neoplastic Process	106	3	
156	C0206677	Adenomatous Polyps	Neoplastic Process	2	1	
157	C0877373	Advanced cancer	Neoplastic Process	80	1	

158	C1510827	Affinity	Natural Phenomenon or Process	10	1	
159	C0429563	After image	Sign or Symptom	16	1	
160	C0085631	Agitation	Sign or Symptom	41	3	
161	C1318045	AKAP13 protein, human	Biologically Active Substance	1	1	
162	C0001924	Albumins	Biologically Active Substance	374	2	
163	C1561581	Allergy Severity - Severe	Finding	1	1	
164	C0439044	Alone	Finding	1	1	
165	C0002520	Amino Acids	Biologically Active Substance	6	1	
166	C1521871	Amplification	Phenomenon or Process	234	8	
167	C0002736	Amyotrophic Lateral Sclerosis	Disease or Syndrome	1	1	
168	C0281778	Anal abscess	Disease or Syndrome	3	1	
169	C0205929	Anal Fistula	Anatomical Abnormality	1	1	
170	C0002871	Anemia	Disease or Syndrome	4	2	
171	C0003090	Ankylosis	Acquired Abnormality<br>Disease or Syndrome	1	1	
172	C0003241	Antibodies	Immunologic Factor	261	36	
173	C0003242	Antibodies, Anti-Idiotypic	Immunologic Factor	18	2	
174	C0368674	ANTIBODY ANTIBODY	Immunologic Factor	10	4	
175	C0598658	antibody conjugate	Immunologic Factor	16	1	
176	C0003320	Antigens	Biologically Active Substance<br>Immunologic Factor	3	1	
177	C0427965	Antimicrobial susceptibility	Finding	42	5	
178	C0003442	Antithymoglobulin	Immunologic Factor	95	2	
179	C0003864	Arthritis	Disease or Syndrome	3	1	
180	C0003962	Ascites	Finding	14	5	
181	C1510438	Assay	Laboratory Procedure	87	9	
182	C0004096	Asthma	Disease or Syndrome	17	2	
183	C0004178	Atmosphere, planetary	Natural Phenomenon or Process	4	1	
184	C1366832	ATPase, Aminophospholipid Transporter-Like, Class I, Type 8A, Mem	Biologically Active Substance	7	2	
185	C1135130	Avastin	Immunologic Factor	118	1	
186	C1332360	AXIN2 protein	Biologically Active Substance	40	1	
187	C0293686	Bad protein	Biologically Active Substance	17	1	

188	C0202333	Barium measurement	Laboratory Procedure	2	1	
189	C0796392	bevacizumab	Immunologic Factor	8	1	
190	C0242210	Binding Protein	Biologically Active Substance	10	3	
191	C0005507	Biological Assay	Laboratory Procedure	87	9	
192	C0005522	Biological Products	Biologically Active Substance	12	1	
193	C0018941	Blood Tests	Laboratory Procedure	2	1	
194	C1259929	BRAF protein, human	Biologically Active Substance	269	7	
195	C0294028	BRCA2 Protein	Biologically Active Substance	1	1	
196	C0858252	Breast adenocarcinoma	Neoplastic Process	339	4	
197	C0006353	Buffers	Biologically Active Substance	60	7	
198	C0006413	Burkitt Lymphoma	Neoplastic Process	339	4	
199	C0872336	C10	Immunologic Factor	9	1	
200	C1443182	CALCULATED	Laboratory Procedure	65	3	
201	C0007012	Carbon Dioxide	Biologically Active Substance	4	1	
202	C0007082	Carcinoembryonic Antigen	Immunologic Factor	158	2	
203	C0596263	Carcinogenesis	Neoplastic Process	4	1	
204	C0007095	Carcinoid Tumor	Neoplastic Process	1	1	
205	C0007099	Carcinoma in Situ	Neoplastic Process	4	2	
206	C0699885	Carcinoma of bladder	Neoplastic Process	339	4	
207	C0684249	Carcinoma of lung	Neoplastic Process	376	7	
208	C0220647	Carcinoma of unknown primary	Neoplastic Process	4	1	
209	C0206659	Carcinoma, Embryonal	Neoplastic Process	20	1	
210	C0206696	Carcinoma, Signet Ring Cell	Neoplastic Process	43	2	
211	C0007138	Carcinoma, Transitional Cell	Neoplastic Process	339	4	
212	C0007140	Carcinosarcoma	Neoplastic Process	13	1	
213	C1110554	Cardiovascular occlusion	Pathologic Function	2	1	
214	C0560175	Carrier of disorder	Finding	4	2	
215	C0699809	Carries	Finding	61	5	
216	C1266100	CASTLE	Neoplastic Process	95	2	
217	C1308290	CCL15 protein, human	Biologically Active Substance	1	1	
218	C0108768	CD14 Antigen	Immunologic Factor	4	1	
219	C0108748	CD19 Antigens	Immunologic Factor	1	2	

220	C0054946	CD20 Antigens	Immunologic Factor	14	3	
221	C0003323	CD4 Antigens	Immunologic Factor	1	1	
222	C0243982	CD44 Antigens	Immunologic Factor	10	2	
223	C0108754	CD56 Antigens	Immunologic Factor	4	1	
224	C1619635	CD71 antigen	Immunologic Factor	1	1	
225	C0104998	CD80 Antigens	Immunologic Factor	1	2	
226	C1505661	CDX2 Protein, human	Biologically Active Substance	20	4	
227	C0007570	Celiac Disease	Disease or Syndrome	1	1	
228	C0007597	Cell hybridization	Laboratory Procedure	4	1	
229	C0154841	Central retinal vein occlusion	Disease or Syndrome	2	1	
230	C0007703	Centrifugation	Laboratory Procedure	4	1	
231	C0007745	Ceramides	Biologically Active Substance	1	1	
232	C0206708	Cervical Intraepithelial Neoplasia	Neoplastic Process	33	1	
233	C0302592	Cervix carcinoma	Neoplastic Process	12	1	
234	C0995188	cetuximab	Immunologic Factor	116	3	
235	C0008013	Chemotactic Factors	Biologically Active Substance<br>Immunologic Factor	4	1	
236	C0008073	Child Development Disorders	Mental or Behavioral Dysfunctio	1	1	
237	C0008550	Chromatography	Laboratory Procedure	10	1	
238	C0008551	Chromatography, Affinity	Laboratory Procedure	10	1	
239	C0008574	Chromium	Biologically Active Substance	43	1	
240	C1257806	Chromosomal Instability	Cell or Molecular Dysfunction	33	1	
241	C0015674	Chronic Fatigue Syndrome	Disease or Syndrome	2	1	
242	C0023434	Chronic Lymphocytic Leukemia	Neoplastic Process	50	1	
243	C0024117	Chronic Obstructive Airway Disease	Disease or Syndrome	2	2	
244	C0542277	Cleans drug injection equipment finding	Finding	21	2	
245	C0683325	clinical aspects	Clinical Attribute	15	2	
246	C1522545	c-Myc Staining Method	Laboratory Procedure	26	5	
247	C0282547	Coculture Techniques	Laboratory Procedure	10	2	
248	C0009221	Codon Nucleotides	Biologically Active Substance	4	3	
249	C0009264	cold temperature	Natural Phenomenon or Process	9	1	
250	C0009319	Colitis	Disease or Syndrome	12	3	

251	C0861461	Colon Cancer Stage IV	Neoplastic Process	32	1	
252	C0009393	Color	Natural Phenomenon or Process	193	3	
253	C0948380	Colorectal cancer metastatic	Neoplastic Process	82	2	
254	C0677947	Colorectal cancer stage I	Neoplastic Process	1	1	
255	C0677948	Colorectal cancer stage II	Neoplastic Process	1	1	
256	C0677949	Colorectal cancer stage III	Neoplastic Process	4	1	
257	C0009450	Communicable Diseases	Disease or Syndrome	2	1	
258	C1441526	COMPUTED	Laboratory Procedure	60	1	
259	C1446561	Concentration measurement	Laboratory Procedure	94	6	
260	C0301869	Conjugate	Immunologic Factor	16	1	
261	C0518609	CONSIDERATION	Finding	16	1	
262	C0678568	cooling	Natural Phenomenon or Process	4	1	
263	C0282523	CpG Islands	Biologically Active Substance	16	1	
264	C1451465	CRK protein, human	Biologically Active Substance	1	1	
265	C0010346	Crohn's disease	Disease or Syndrome	113	3	
266	C0010734	Cytidine Triphosphate	Biologically Active Substance	1	1	
267	C0010803	Cytokeratin	Biologically Active Substance	2	1	
268	C0079189	cytokine	Immunologic Factor	4	1	
269	C0299250	Cytokine Inducible SH2-Containing Protein	Biologically Active Substance	99	1	
270	C0010843	Cytosine	Biologically Active Substance	3	1	
271	C0011061	Deamination	Natural Phenomenon or Process	3	1	
272	C0043096	Decreased body weight	Finding	17	4	
273	C0149871	Deep Vein Thrombosis	Disease or Syndrome	2	1	
274	C0029408	Degenerative polyarthritis	Disease or Syndrome	4	1	
275	C0289848	deleted in colorectal carcinoma protein	Biologically Active Substance	1	2	
276	C0178587	density	Natural Phenomenon or Process	4	1	
277	C0011485	Deoxycytidine	Biologically Active Substance	17	3	
278	C0344315	Depressed mood	Finding	6	2	
279	C0011581	Depressive disorder	Mental or Behavioral Dysfunctio	6	2	
280	C0349217	Depressive episode, unspecified	Mental or Behavioral Dysfunctio	6	2	
281	C1441547	DERIVED	Laboratory Procedure	50	7	
282	C0011682	Desiccation	Natural Phenomenon or Process	16	1	

283	C0442726	Detected	Finding	17	2	
284	C0751115	Digestive Epilepsy	Disease or Syndrome	8	3	
285	C0544452	Disease remission	Finding	137	2	
286	C0012655	Disease susceptibility	Clinical Attribute	1	1	
287	C1510475	Diverticulosis	Anatomical Abnormality<br>Disease or Syndrome	2	1	
288	C0012854	DNA	Biologically Active Substance	1374	73	
289	C0200898	DNA analysis	Laboratory Procedure	4	1	
290	C0753876	DNA Mismatch Repair Protein MLH1	Biologically Active Substance	147	3	
291	C1333234	DNA Mismatch Repair Protein MSH2	Biologically Active Substance	80	2	
292	C1333235	DNA Mismatch Repair Protein PMS2	Biologically Active Substance	97	2	
293	C1442917	DNA probe method	Laboratory Procedure	138	8	
294	C0012929	DNA, Mitochondrial	Biologically Active Substance	9	1	
295	C0012940	DNA-Binding Proteins	Biologically Active Substance	5	2	
296	C0013295	Duodenal Ulcer	Disease or Syndrome	341	5	
297	C0151966	Duodenal ulcer with hemorrhage	Disease or Syndrome	2	1	
298	C0013595	Eczema	Disease or Syndrome	2	1	
299	C0152096	Edward's syndrome NOS	Disease or Syndrome	80	1	
300	C0013687	effusion	Pathologic Function	18	2	
301	C0675313	EHF protein, human	Biologically Active Substance	1	1	
302	C0013855	Electrophoresis	Laboratory Procedure	76	2	
303	C0201699	Electrophoresis, Capillary	Laboratory Procedure	60	1	
304	C0557351	Employed	Finding	20	1	
305	C1527169	Encounter due to donor examination	Finding	32	6	
306	C0040335	Encounter due to tobacco use	Finding	446	1	
307	C0600075	Endurance of activity	Finding	41	4	
308	C1530358	EP300 protein, human	Biologically Active Substance	1	1	
309	C0014544	Epilepsy	Disease or Syndrome	3	1	
310	C0677886	Epithelial ovarian cancer	Neoplastic Process	26	2	
311	C0276242	Equine viral rhinopneumonitis	Disease or Syndrome	4	1	
312	C1173436	Erbitux	Immunologic Factor	118	1	
313	C0152018	Esophageal carcinoma	Neoplastic Process	2	1	

314	C0680174	ethnic	Finding	446	1	
315	C0015295	Exons	Biologically Active Substance	12	1	
316	C0332157	Exposure	Clinical Attribute	109	10	
317	C0728853	Exposure NOS	Phenomenon or Process	45	4	
318	C1172384	EZH2 protein, human	Biologically Active Substance	4	1	
319	C0241889	FAMILY HISTORY	Finding	1002	6	
320	C0015967	Fever	Finding	3	1	
321	C0016057	fibrosarcoma	Neoplastic Process	352	5	
322	C0016107	filtration	Laboratory Procedure	41	3	
323	C1304686	Finding of pH	Laboratory or Test Result	152	6	
324	C1517205	Flare	Sign or Symptom	202	1	
325	C0235665	FLUOR	Pathologic Function	95	2	
326	C0016318	Fluorescent Antibody Technique	Laboratory Procedure	16	1	
327	C1333634	Forkhead Box Protein O4	Biologically Active Substance	20	1	
328	C0596588	FOS Protein	Biologically Active Substance	1	1	
329	C1333647	Functional RNA	Biologically Active Substance	1	1	
330	C0017011	Gamma Rays	Natural Phenomenon or Process	13	1	
331	C0008555	Gas Chromatography	Laboratory Procedure	3	1	
332	C0278701	Gastric Adenocarcinoma	Neoplastic Process	2	1	
333	C1559265	GASTROINTESTINAL	Pathologic Function	8	4	
334	C0151544	Gastrointestinal carcinoma	Neoplastic Process	2	1	
335	C0017185	Gastrointestinal Neoplasms	Neoplastic Process	2	1	
336	C0238198	Gastrointestinal Stromal Tumors	Neoplastic Process	2	1	
337	C0752248	Gene Expression Profiling	Laboratory Procedure	24	3	
338	C0042333	Genetic Diversity	Natural Phenomenon or Process	39	1	
339	C0376343	Genomic Hybridization	Natural Phenomenon or Process	17	2	
340	C0017636	Glioblastoma	Neoplastic Process	371	6	
341	C1333681	GLTSCR2 protein	Biologically Active Substance	1	1	
342	C0017725	Glucose	Biologically Active Substance	3	1	
343	C0017797	Glutamine	Biologically Active Substance	4	1	
344	C0017911	Glycogen	Biologically Active Substance	4	1	
345	C0018021	Goiter	Disease or Syndrome	2	1	

346	C0018099	Gout	Disease or Syndrome	2	1	
347	C0018284	Growth Factor	Biologically Active Substance	1	1	
348	C0299348	G-T mismatch-binding protein	Biologically Active Substance	81	2	
349	C0120446	Guanidine	Biologically Active Substance	4	1	
350	C0677607	Hashimoto Disease	Disease or Syndrome	3	1	
351	C0018621	Hay fever	Disease or Syndrome	2	1	
352	C0018799	Heart Diseases	Disease or Syndrome	2	1	
353	C0018837	Heat	Natural Phenomenon or Process	77	8	
354	C0200627	Hematology procedure	Laboratory Procedure	12	1	
355	C0019046	Hemoglobin	Biologically Active Substance	374	2	
356	C0019134	Heparin	Biologically Active Substance	4	2	
357	C0019158	Hepatitis	Disease or Syndrome	3	1	
358	C0019189	Hepatitis, Chronic	Disease or Syndrome	3	1	
359	C0520463	Hepatitis, Chronic Active	Disease or Syndrome	3	1	
360	C0206624	Hepatoblastoma	Neoplastic Process	33	2	
361	C0948775	High weight	Sign or Symptom	3	1	
362	C0019602	Histidine	Biologically Active Substance	2	2	
363	C0449574	Histologic type	Clinical Attribute	2	2	
364	C0344441	Histology Procedure	Laboratory Procedure	643	5	
365	C1291847	Histone antigen	Immunologic Factor	6	2	
366	C0019647	Histone H3	Biologically Active Substance	6	2	
367	C0019652	Histones	Biologically Active Substance	6	2	
368	C0677043	Histopathology	Pathologic Function	2	2	
369	C0019693	HIV Infections	Disease or Syndrome	1	1	
370	C0086413	HIV Vaccine	Immunologic Factor	1	1	
371	C0019721	HLA Antigens	Immunologic Factor	22	1	
372	C0242617	Homeodomain Proteins	Biologically Active Substance	4	1	
373	C1505203	HP protein, human	Biologically Active Substance	4	3	
374	C0020443	hypercholesterolemia	Disease or Syndrome	2	2	
375	C1522133	Hypercholesterolemia result	Laboratory or Test Result	1	1	
376	C0020507	Hyperplasia	Pathologic Function	11	1	
377	C0242184	Hypoxia	Pathologic Function	6	2	

378	C0215474	IL2-inducible T-cell kinase	Immunologic Factor	1	1	
379	C0021044	Immunohistochemistry	Laboratory Procedure	74	1	
380	C0001551	Immunologic Adjuvants	Immunologic Factor	37	1	
381	C0021067	Immunoperoxidase Techniques	Laboratory Procedure	2	1	
382	C0301871	Immunoprecipitate	Immunologic Factor	38	1	
383	C0021069	Immunoprecipitation	Laboratory Procedure	82	15	
384	C0677874	In complete remission	Finding	9	1	
385	C0544461	Inactivation	Cell or Molecular Dysfunction	3	1	
386	C1439852	Incubated	Laboratory Procedure	10	3	
387	C0021311	Infection	Disease or Syndrome	2	1	
388	C0021359	Infertility	Disease or Syndrome	2	1	
389	C0021368	Inflammation	Pathologic Function	209	2	
390	C0083032	Interleukin 7 Receptor	Immunologic Factor	2	1	
391	C0021760	Interleukin-6	Immunologic Factor	1	1	
392	C0085424	Interleukin-9	Immunologic Factor	1	1	
393	C0221715	Intestinal carcinoma	Neoplastic Process	12	1	
394	C0021843	Intestinal Obstruction	Acquired Abnormality<br>Disease or Syndrome	7	1	
395	C1334274	Invasive Carcinoma	Neoplastic Process	3	1	
396	C0302583	Iron	Biologically Active Substance	4	2	
397	C1282930	Irradiation	Natural Phenomenon or Process	6	1	
398	C0022107	Irritable Mood	Finding	3	1	
399	C0575044	Joint problem	Finding	374	2	
400	C0022526	Karyotype determination procedure	Laboratory Procedure	37	14	
401	C0178725	Karyotype result	Laboratory or Test Result	37	14	
402	C0022564	Keratin	Biologically Active Substance	3	1	
403	C1522225	Knock-out	Experimental Model of Disease	5	2	
404	C0022790	Krukenberg Tumor	Neoplastic Process	1	1	
405	C0079748	Large Cell Lymphoblastic Lymphoma	Neoplastic Process	50	1	
406	C0024302	Large-Cell Lymphomas	Neoplastic Process	20	1	
407	C0023269	leiomyosarcoma	Neoplastic Process	1	1	
408	C0855056	Leiomyosarcoma metastatic	Neoplastic Process	1	1	

409	C0221198	Lesion	Finding	39	1	
410	C0023418	leukemia	Neoplastic Process	434	8	
411	C0023449	Leukemia, Lymphocytic, Acute	Neoplastic Process	49	4	
412	C0023458	Leukemia, Lymphocytic, Chronic	Neoplastic Process	4	1	
413	C0023467	Leukemia, Myelocytic, Acute	Neoplastic Process	2	1	
414	C0023492	Leukemia, T-Cell	Neoplastic Process	339	4	
415	C0950624	Leukocyte L1 Antigen Complex	Immunologic Factor	202	1	
416	C0023749	Linoleic Acid	Biologically Active Substance	3	1	
417	C0023827	liposarcoma	Neoplastic Process	33	2	
418	C1521461	Loss of Chromosome 5q	Cell or Molecular Dysfunction	90	1	
419	C0024121	Lung Neoplasms	Neoplastic Process	1	2	
420	C1524007	Lymphoblastic leukemia	Neoplastic Process	49	4	
421	C0024266	Lymphocytic Choriomeningitis	Disease or Syndrome	8	1	
422	C0024299	Lymphoma	Neoplastic Process	360	6	
423	C0334634	Lymphoma, Small-Cell, Centrocytic	Neoplastic Process	1	1	
424	C0024337	Lysine	Biologically Active Substance	1	1	
425	C0024348	Lysis	Pathologic Function	6	1	
426	C0008015	Macrophage Chemotactic Factors	Immunologic Factor	13	1	
427	C0065491	macrophage inflammatory protein 2	Biologically Active Substance	4	1	
428	C0376579	Macrophage Inflammatory Proteins	Immunologic Factor	4	1	
429	C0079785	Macrophage-1 Antigen	Immunologic Factor	4	1	
430	C0599662	MACS	Laboratory Procedure	26	2	
431	C0761585	MADHIP protein, human	Biologically Active Substance	2	1	
432	C0919482	MAF Protein	Biologically Active Substance	7	3	
433	C1328924	magnetic beads	Natural Phenomenon or Process	1	1	
434	C0563532	Magnetism	Natural Phenomenon or Process	1	1	
435	C0206625	Malignant Mixed Tumor	Neoplastic Process	2	1	
436	C0006142	Malignant neoplasm of breast	Neoplastic Process	17	4	
437	C0007847	Malignant neoplasm of cervix uteri	Neoplastic Process	12	1	
438	C0278484	Malignant neoplasm of colon stage IV	Neoplastic Process	41	3	
439	C0740457	Malignant neoplasm of kidney	Neoplastic Process	12	1	
440	C0345904	Malignant neoplasm of liver	Neoplastic Process	12	1	

441	C0242379	Malignant neoplasm of lung	Neoplastic Process	13	2	
442	C0024623	Malignant neoplasm of stomach	Neoplastic Process	13	2	
443	C0153594	Malignant neoplasm of testis	Neoplastic Process	13	1	
444	C1257930	Mammary Carcinoma, Human	Neoplastic Process	13	1	
445	C0021367	Mammary Ductal Carcinoma	Neoplastic Process	13	1	
446	C0545081	Mantle lymphoma	Neoplastic Process	1	1	
447	C0947322	Manufacturer Name	Finding	30	10	
448	C0577559	Mass of body structure	Finding	6	4	
449	C0262926	Medical History	Finding	1004	8	
450	C0206693	Medullary carcinoma	Neoplastic Process	1	1	
451	C0025202	melanoma	Neoplastic Process	372	6	
452	C0796561	Melanoma vaccine	Immunologic Factor	372	6	
453	C0011570	Mental Depression	Mental or Behavioral Dysfunctio	6	2	
454	C0333983	Metaplastic polyp	Neoplastic Process	11	1	
455	C0220650	Metastatic malignant neoplasm to brain	Neoplastic Process	1	2	
456	C0919989	Metastatic renal carcinoma	Neoplastic Process	1	1	
457	C0278678	Metastatic renal cell carcinoma	Neoplastic Process	1	1	
458	C0025723	Methylation	Natural Phenomenon or Process	99	13	
459	C1449575	Microarray Analysis	Laboratory Procedure	32	5	
460	C1101610	MicroRNAs	Biologically Active Substance	214	10	
461	C0149931	Migraine Disorders	Disease or Syndrome	2	1	
462	C1368354	Mixed Neoplasm	Neoplastic Process	2	1	
463	C0026277	Mixed Salivary Gland Tumor	Neoplastic Process	2	1	
464	C0206627	Mixed Tumor, Mullerian	Neoplastic Process	2	1	
465	C0425245	Mobility as a finding	Finding	1	1	
466	C0562577	Mocking	Finding	3	1	
467	C1513392	Molecular Fingerprint of Tumor	Clinical Attribute	9	1	
468	C0746619	MONOCLONAL	Finding	4	2	
469	C0003250	Monoclonal Antibodies	Immunologic Factor	2	1	
470	C0128897	Monocyte Chemoattractant Protein-1	Immunologic Factor	4	1	
471	C0282566	Monocyte Chemoattractant Proteins	Immunologic Factor	4	1	
472	C1335296	MPP1 protein, human	Biologically Active Substance	1	1	

473	C1513717	Mucinous Differentiation	Finding	1	1	
474	C0334368	Mucin-producing adenocarcinoma	Neoplastic Process	2	1	
475	C0026682	Mucins	Biologically Active Substance	15	2	
476	C0026764	Multiple Myeloma	Neoplastic Process	33	2	
477	C0334108	Multiple polyps	Neoplastic Process	140	4	
478	C0080194	Muscle strain	Injury or Poisoning	6	2	
479	C0670878	MYBBP1A protein, human	Biologically Active Substance	2	1	
480	C0023470	Myeloid Leukemia	Neoplastic Process	2	1	
481	C1172734	natalizumab	Immunologic Factor	1	1	
482	C0027498	Nausea with vomiting	Sign or Symptom	1	1	
483	C0915178	NCOA6 protein, human	Biologically Active Substance	40	10	
484	C0027708	Nephroblastoma	Neoplastic Process	62	1	
485	C0442874	Neuropathy	Disease or Syndrome	1	1	
486	C0027960	Nevus	Neoplastic Process	8	3	
487	C0279781	no positive axillary nodes	Finding	4	1	
488	C0442743	Noninflammatory	Finding	7	1	
489	C0231162	Normal general body function	Finding	3	1	
490	C1527124	Nottingham Score	Clinical Attribute	1	1	
491	C0028602	Nucleic Acid Hybridization	Natural Phenomenon or Process	358	25	
492	C0262584	Oat cell carcinoma	Neoplastic Process	339	4	
493	C1553907	Observation Method - algorithm	Laboratory Procedure	10	2	
494	C0449381	Observation parameter	Finding	60	1	
495	C0069500	oncogene protein DBL	Biologically Active Substance	1	1	
496	C1268822	Optical density function	Clinical Attribute	64	2	
497	C0029463	osteosarcoma	Neoplastic Process	339	4	
498	C1335177	Ovarian Serous Adenocarcinoma	Neoplastic Process	2	1	
499	C1441041	P2	Immunologic Factor	2	1	
500	C0599933	p65	Biologically Active Substance	1	1	
501	C1306837	Papillary Renal Cell Carcinoma	Neoplastic Process	1	1	
502	C0016169	pathologic fistula	Anatomical Abnormality	4	1	
503	C0677042	Pathology processes	Pathologic Function	212	4	
504	C0429403	Pattern ERG P50	Finding	1	1	

505	C0347944	Pelvic mass	Finding	1	1	
506	C0669679	PER2 protein, mammalian	Biologically Active Substance	4	1	
507	C0031019	Perianal abscess	Acquired Abnormality<br>Disease or Syndrome	3	1	
508	C1317608	PERINEURAL INVASION	Pathologic Function	119	1	
509	C0031117	Peripheral Neuropathy	Disease or Syndrome	1	1	
510	C0085096	Peripheral Vascular Diseases	Disease or Syndrome	1	1	
511	C0747548	Pharyngeal Carcinoma	Neoplastic Process	62	1	
512	C1285572	Phenotype determination	Laboratory Procedure	124	4	
513	C0031619	Phosphatidylglycerols	Biologically Active Substance	4	1	
514	C0031789	Phycoerythrin	Biologically Active Substance	7	2	
515	C0311392	Physical findings	Sign or Symptom	7	1	
516	C0700329	Physical shape	Finding	74	6	
517	C0031858	Phytohemagglutinins	Immunologic Factor	3	1	
518	C0032131	Plasmacytoma	Neoplastic Process	33	2	
519	C0032136	Plasmids	Biologically Active Substance	9	1	
520	C0032140	Plasminogen	Biologically Active Substance	1	1	
521	C0032227	Pleural effusion disorder	Pathologic Function	18	2	
522	C0032285	Pneumonia	Disease or Syndrome	1	1	
523	C0518611	Poise	Finding	2	1	
524	C0083956	Polycomb	Biologically Active Substance	1	1	
525	C0032520	Polymerase Chain Reaction	Laboratory Procedure	134	9	
526	C0521516	Polymyalgia	Sign or Symptom	2	1	
527	C0032533	Polymyalgia Rheumatica	Disease or Syndrome	2	1	
528	C1524110	positioning	Biologic Function	208	3	
529	C0032931	Precipitation	Natural Phenomenon or Process	23	3	
530	C1337053	pre-miRNA	Biologically Active Substance	1	1	
531	C0033085	Preservation, Biological	Laboratory Procedure	17	3	
532	C1335475	Primary Carcinoma	Neoplastic Process	13	4	
533	C0019204	Primary carcinoma of the liver cells	Neoplastic Process	1	1	
534	C0553573	Primary infertility	Disease or Syndrome	2	1	
535	C0279000	Primary Liver Carcinoma	Neoplastic Process	12	1	

536	C1306459	Primary malignant neoplasm	Neoplastic Process	4	1	
537	C0475447	Primary tumor site	Clinical Attribute	106	2	
538	C0033213	Problem	Finding	41	3	
539	C1522240	Process	Phenomenon or Process	126	6	
540	C1548180	Production Processing ID	Finding	10	2	
541	C0033382	Proline	Biologically Active Substance	61	2	
542	C0086860	Promotor	Biologically Active Substance	73	6	
543	C0033413	Promotor Regions	Biologically Active Substance	40	1	
544	C1335532	Protein Family	Biologically Active Substance	1	1	
545	C0428479	Protein level - finding	Laboratory or Test Result	4	1	
546	C0067033	Proteinase 3	Immunologic Factor	1	1	
547	C0033684	Proteins	Biologically Active Substance	136	10	
548	C0072470	Proto-Oncogene Protein c-kit	Immunologic Factor	2	1	
549	C0072473	Proto-Oncogene Proteins c-myb	Biologically Active Substance	1	1	
550	C0080065	Proto-Oncogene Proteins c-myc	Biologically Active Substance	26	5	
551	C0700164	proven venom	Biologically Active Substance	1	1	
552	C1257843	pseudomembranous colitis	Disease or Syndrome	2	2	
553	C0033377	Ptosis	Anatomical Abnormality<br>Disease or Syndrome	1	1	
554	C0243114	purification	Laboratory Procedure	98	3	
555	C1446777	Rabbit antigen	Immunologic Factor	11	4	
556	C0034553	Radioactivity	Natural Phenomenon or Process	41	3	
557	C1384668	radiotherapy	Finding	38	1	
558	C0249759	RBL2 protein, human	Biologically Active Substance	2	1	
559	C0596012	Reaches	Finding	64	2	
560	C1524094	Recombinant Cytokines	Immunologic Factor	4	1	
561	C1522558	Recombinant Monocyte Chemoattractant Protein-1	Immunologic Factor	4	1	
562	C1527194	Recombinant Transforming Growth Factor	Biologically Active Substance	1	1	
563	C1522669	Recombinant Tumor Necrosis Factor-Alpha	Immunologic Factor	22	2	
564	C0034885	Rectal Neoplasms	Neoplastic Process	234	4	
565	C1527408	Rectal Tumors	Disease or Syndrome	234	4	
566	C1327622	Regulation of biological process	Biologic Function	20	1	

567	C0035012	Reiter Disease	Disease or Syndrome	3	1	
568	C0919436	REL Protein	Biologically Active Substance	41	1	
569	C0445223	Related personal status	Finding	9	2	
570	C1378703	Renal carcinoma	Neoplastic Process	13	2	
571	C0007134	Renal Cell Carcinoma	Neoplastic Process	1	1	
572	C0151747	Renal tubular disorder	Disease or Syndrome	40	2	
573	C1514850	Replication Error	Cell or Molecular Dysfunction	34	3	
574	C0871261	response	Clinical Attribute	438	16	
575	C0035328	Retinal Vein Occlusion	Disease or Syndrome	2	1	
576	C0003873	Rheumatoid Arthritis	Disease or Syndrome	3	2	
577	C0383269	Rho Guanine Nucleotide Exchange Factor 5	Biologically Active Substance	1	1	
578	C0035696	RNA, Messenger	Biologically Active Substance	376	22	
579	C0043309	Roentgen Rays	Natural Phenomenon or Process	40	10	
580	C0217373	RTN1 protein, human	Biologically Active Substance	2	1	
581	C1522662	sample fixation	Laboratory Procedure	23	1	
582	C1509144	Sample pool	Clinical Attribute	1087	38	
583	C0237849	Scaling	Finding	9	2	
584	C0036285	Scarlet Fever	Disease or Syndrome	3	1	
585	C0751623	Second Primary Cancers	Neoplastic Process	6	1	
586	C0205254	Sedentary	Finding	1	1	
587	C0086972	Separated from cohabitee	Finding	4	1	
588	C0258846	SHH protein, human	Biologically Active Substance	3	1	
589	C0426857	Short arm	Finding	1	1	
590	C0149925	Small cell carcinoma of lung	Neoplastic Process	339	4	
591	C1367790	Small Nuclear Ribonucleoprotein Polypeptide F	Biologically Active Substance	2	1	
592	C0248587	SMARCA4 protein, human	Biologically Active Substance	1	1	
593	C0337664	Smoker	Finding	24	1	
594	C1519386	Smoking Status	Clinical Attribute	374	2	
595	C1519388	Smooth Muscle Actin Staining Method	Laboratory Procedure	2	1	
596	C0037420	Social Interaction	Finding	4	1	
597	C0037473	Sodium	Biologically Active Substance	140	6	
598	C0037494	Sodium Chloride	Biologically Active Substance	102	4	

599	C0746416	Soft mass	Finding	1	1	
600	C0457193	Soft tissue mass	Anatomical Abnormality	1	1	
601	C1409616	Special screening finding	Finding	321	1	
602	C0443640	Specific antibody	Immunologic Factor	8	1	
603	C0037805	Spectrophotometry	Laboratory Procedure	155	3	
604	C0007137	Squamous cell carcinoma	Neoplastic Process	341	5	
605	C0333873	Squamous intraepithelial lesion	Cell or Molecular Dysfunction	2	1	
606	C0278474	Stage I Colon Cancer	Neoplastic Process	16	1	
607	C0278479	Stage II Colon Cancer	Neoplastic Process	25	2	
608	C0280252	stage, colon cancer	Neoplastic Process	25	2	
609	C0487602	Staining method	Laboratory Procedure	51	9	
610	C0699791	Stomach Carcinoma	Neoplastic Process	15	3	
611	C0430414	Stool culture	Laboratory Procedure	6	2	
612	C1510453	strain symptom	Sign or Symptom	6	2	
613	C0075278	Streptavidin	Biologically Active Substance	7	2	
614	C0038435	Stress	Finding	17	2	
615	C0333187	Stricture	Anatomical Abnormality	172	1	
616	C0879615	Stromal Neoplasm	Neoplastic Process	2	1	
617	C0038636	Sucrose	Biologically Active Substance	6	1	
618	C1317973	SUPPORT	Clinical Attribute	17	2	
619	C0549433	Surgical intervention	Finding	9	1	
620	C1457887	Symptoms	Sign or Symptom	649	3	
621	C0869032	Synthesis	Phenomenon or Process	172	8	
622	C0084726	T Cell Transcription Factor 1	Biologically Active Substance	1	1	
623	C0087051	T-Cell Receptor gamma-Chain	Immunologic Factor	91	2	
624	C1566664	TCF Transcription Factors	Biologically Active Substance	1	1	
625	C0039538	Teratoma	Neoplastic Process	13	1	
626	C0080222	TGFB1 protein, human	Biologically Active Substance	1	1	
627	C0040048	Thromboplastin	Immunologic Factor	1	1	
628	C0076582	thymidine 5'-triphosphate	Biologically Active Substance	3	1	
629	C0040284	Tissue culture	Laboratory Procedure	6	2	
630	C0010957	Tissue damage	Injury or Poisoning	40	10	

631	C0920664	tissue preparation	Laboratory Procedure	18	3	
632	C0162621	Titration	Laboratory Procedure	2	1	
633	C1448177	TNF protein, human	Biologically Active Substance	22	2	
634	C0543414	Tobacco use	Finding	446	1	
635	C0040648	TRANSCRIPTION FACTOR	Biologically Active Substance	5	2	
636	C1336780	Transcription Factor 3	Biologically Active Substance	1	1	
637	C0761476	transcriptional coactivator p52	Biologically Active Substance	1	1	
638	C1540850	Transferring	Biologic Function	3	1	
639	C0728827	Transfers	Finding	3	1	
640	C0040690	Transforming Growth Factor beta	Biologically Active Substance	3	2	
641	C0079441	Transforming Growth Factor Beta 2	Biologically Active Substance	1	1	
642	C0040691	Transforming Growth Factors	Biologically Active Substance	1	1	
643	C0279680	Transitional cell carcinoma of bladder	Neoplastic Process	339	4	
644	C0041249	Tryptophan	Biologically Active Substance	2	3	
645	C1269955	Tumor Cell Invasion	Finding	133	3	
646	C0598934	tumor growth	Neoplastic Process	20	1	
647	C1456820	Tumor Necrosis Factor-alpha	Immunologic Factor	22	2	
648	C0041582	Ulcer	Pathologic Function	10	1	
649	C0041609	Ultracentrifugation	Laboratory Procedure	6	1	
650	C0041625	Ultraviolet Rays	Natural Phenomenon or Process	67	3	
651	C0205698	Undifferentiated carcinoma	Neoplastic Process	1	1	
652	C0445356	Unrelated	Finding	19	1	
653	C0042221	Vacuum	Natural Phenomenon or Process	4	1	
654	C0205990	Vaginal Prolapse	Acquired Abnormality	1	1	
655	C1558950	VASCULAR	Pathologic Function	120	2	
656	C0042373	Vascular Diseases	Disease or Syndrome	1	1	
657	C0241669	Venous occlusion	Pathologic Function	2	1	
658	C0042666	Vimentin	Biologically Active Substance	1	1	
659	C0042769	Virus Diseases	Disease or Syndrome	1	1	
660	C0221103	Visual Suppression	Pathologic Function	12	1	
661	C0202498	Warfarin Measurement	Laboratory Procedure	2	1	
662	C0442730	Weakly positive	Finding	1	1	

663	C0949466	Western Blot	Laboratory Procedure	20	1	
664	C0023508	White Blood Cell Count procedure	Laboratory Procedure	3	1	
665	C0043481	Zinc	Biologically Active Substance	4	1	

Table S7

Index	Gene_Symbol	Sources	Reliability
1	AACS	GEO	*
2	AADAT	GEO	*
3	AARSD1	GEO	*
4	ABCA5	GEO	*
5	ABCA6	GEO	*
6	ABCA8	GEO	*
7	ABCB1	Both	*
8	ABCC2	Both	*
9	ABCC3	Both	*
10	ABCG2	Both	*
11	ABHD2	GEO	*
12	ABHD3	GEO	*
13	ABI3BP	GEO	*
14	ABP1	Both	*
15	ACAD10	GEO	*
16	ACADS	GEO	*
17	ACAN	GEO	*
18	ACBD5	GEO	*
19	ACCN2	GEO	*
20	ACP6	GEO	*
21	ACSL5	GEO	*
22	ACSL6	GEO	*
23	ACSS1	GEO	*
24	ACTG2	GEO	*
25	ACTN1	GEO	*
26	ACTR2	GEO	*
27	ACVR1C	GEO	*
28	ACVR2B	GEO	*
29	ADA	Both	*
30	ADAL	GEO	*
31	ADAM12	GEO	*
32	ADAMDEC1	GEO	*
33	ADAMTS1	GEO	*
34	ADAMTS12	GEO	*
35	ADAMTS2	GEO	*
36	ADAMTS6	GEO	*
37	ADAP2	GEO	*
38	ADH1A	Both	*
39	ADH1B	Both	*
40	ADH1C	Both	*
41	ADHFE1	GEO	*
42	ADI1	GEO	*
43	ADPRHL1	GEO	*
44	ADSL	GEO	*
45	ADSSL1	GEO	*
46	AFF4	GEO	*
47	AFMID	GEO	*
48	AGPAT9	GEO	*
49	AGR2	Both	*
50	AGR3	GEO	*
51	AHCY	Both	*
52	AHCYL2	GEO	*
53	AHI1	GEO	*
54	AHNAK	GEO	*
55	AHNAK2	GEO	*
56	AHR	Both	*
57	AIDA	GEO	*
58	AIF1L	GEO	*
59	AIM1	GEO	*
60	AKAP2	GEO	*
61	AKAP5	GEO	*
62	AKR1B10	GEO	*
63	AKR1C1	GEO	*
64	AKR1C3	GEO	*
65	AKT2	GEO	*
66	AKT3	GEO	*
67	ALDH1A1	GEO	*
68	ALDH3B2	GEO	*
69	ALG3	GEO	*
70	ALOX5	Both	*
71	AMACR	Both	*
72	AMIGO2	GEO	*
73	AMOT	GEO	*
74	AMOTL1	GEO	*
75	AMPD2	GEO	*
76	ANGPT2	GEO	*
77	ANGPTL1	GEO	*
78	ANK2	GEO	*
79	ANK3	GEO	*
80	ANKFY1	GEO	*
81	ANKRD22	GEO	*
82	ANKRD29	GEO	*
83	ANKRD46	GEO	*
84	ANKRD6	GEO	*
85	ANKS4B	GEO	*
86	ANO5	GEO	*
87	ANP32E	GEO	*
88	ANPEP	GEO	*
89	ANTXR1	GEO	*
90	ANUBL1	GEO	*
91	ANXA3	GEO	*
92	ANXA4	GEO	*
93	ANXA9	GEO	*
94	AP2M1	GEO	*
95	AP3M1	GEO	*
96	APBB1	GEO	*
97	APBB2	GEO	*
98	APOE	Both	*

99	APOH	GEO	*
100	AQP1	GEO	*
101	AQP8	GEO	*
102	AQR	GEO	*
103	ARAP3	GEO	*
104	ARHGAP15	GEO	*
105	ARHGAP4	GEO	*
106	ARHGAP42	GEO	*
107	ARHGAP44	GEO	*
108	ARHGEF10	GEO	*
109	ARHGEF6	GEO	*
110	ARIH2	GEO	*
111	ARL14	GEO	*
112	ARL4C	GEO	*
113	ARL6IP5	GEO	*
114	ARMC6	GEO	*
115	ARMC8	GEO	*
116	ARMCX1	GEO	*
117	ARNT2	GEO	*
118	ARNTL2	GEO	*
119	ARRB1	GEO	*
120	ARRDC1	GEO	*
121	ARSE	GEO	*
122	ARSJ	GEO	*
123	ASAHL	GEO	*
124	ASAP2	GEO	*
125	ASB2	GEO	*
126	ASB9	GEO	*
127	ASCL2	GEO	*
128	ASNS	GEO	*
129	ASPA	GEO	*
130	ASPH	GEO	*
131	ASPHD1	GEO	*
132	ASPN	GEO	*
133	ASRGL1	GEO	*
134	ATG12	Both	*
135	ATL1	GEO	*
136	ATP10D	GEO	*
137	ATP1B1	GEO	*
138	ATP2C2	GEO	*
139	ATP5S	GEO	*
140	ATP8A1	GEO	*
141	ATP8B2	GEO	*
142	AURKA	Both	*
143	AXIN2	Both	*
144	AXL	GEO	*
145	B3GALNT1	GEO	*
146	B3GALTL	GEO	*
147	B3GNT7	GEO	*
148	BACE2	GEO	*

149	BARX2	GEO	*
150	BAT1	Both	*
151	BCAR3	GEO	*
152	BCAS1	GEO	*
153	BCL2L11	GEO	*
154	BDH1	GEO	*
155	BEST4	GEO	*
156	BEX4	GEO	*
157	BFSP1	GEO	*
158	BGN	GEO	*
159	BHLHB9	GEO	*
160	BMP2	Both	*
161	BMP3	GEO	*
162	BMP7	GEO	*
163	BOP1	GEO	*
164	BRP44	GEO	*
165	BRP44L	GEO	*
166	BSPRY	GEO	*
167	BTC	GEO	*
168	BTG3	GEO	*
169	BTNL3	GEO	*
170	BUB3	GEO	*
171	C10orf10	GEO	*
172	C10orf116	GEO	*
173	C10orf81	GEO	*
174	C10orf99	GEO	*
175	C11orf96	GEO	*
176	C12orf4	GEO	*
177	C12orf75	GEO	*
178	C13orf27	GEO	*
179	C13orf3	GEO	*
180	C14orf139	GEO	*
181	C15orf48	GEO	*
182	C17orf51	GEO	*
183	C17orf68	GEO	*
184	C17orf75	GEO	*
185	C17orf91	GEO	*
186	C1GALT1	GEO	*
187	C1orf115	GEO	*
188	C1orf135	GEO	*
189	C1orf210	GEO	*
190	C1orf59	GEO	*
191	C1orf61	GEO	*
192	C2	Both	*
193	C20orf20	GEO	*
194	C20orf24	GEO	*
195	C20orf46	GEO	*
196	C21orf91	GEO	*
197	C22orf29	GEO	*
198	C2orf88	GEO	*

199	C3orf39	GEO	*
200	C4orf18	GEO	*
201	C4orf19	GEO	*
202	C4orf3	GEO	*
203	C4orf34	GEO	*
204	C4orf46	GEO	*
205	C6orf105	GEO	*
206	C6orf141	GEO	*
207	C6orf150	GEO	*
208	C6orf162	GEO	*
209	C6orf204	GEO	*
210	C6orf35	GEO	*
211	C7	GEO	*
212	C7orf41	GEO	*
213	C7orf44	GEO	*
214	C7orf68	GEO	*
215	C8orf47	GEO	*
216	C9orf125	GEO	*
217	C9orf41	GEO	*
218	C9orf64	GEO	*
219	C9orf68	GEO	*
220	CA1	GEO	*
221	CA12	GEO	*
222	CA2	GEO	*
223	CA4	GEO	*
224	CA7	GEO	*
225	CA9	GEO	*
226	CACHD1	GEO	*
227	CACNA1D	GEO	*
228	CACNA2D1	GEO	*
229	CAD	GEO	*
230	CALD1	GEO	*
231	CAPN5	GEO	*
232	CAPN9	GEO	*
233	CASC3	GEO	*
234	CASP6	Both	*
235	CBFB	GEO	*
236	CBS	Both	*
237	CBX2	GEO	*
238	CBX3	GEO	*
239	CBX6	GEO	*
240	CC2D2A	GEO	*
241	CCDC50	GEO	*
242	CCDC68	GEO	*
243	CCDC69	GEO	*
244	CCDC80	GEO	*
245	CCL14	GEO	*
246	CCL15	GEO	*
247	CCL20	GEO	*
248	CCL23	GEO	*

249	CCL7	Both	*
250	CCNF	GEO	*
251	CCNG2	GEO	*
252	CD109	GEO	*
253	CD14	Both	*
254	CD163	GEO	*
255	CD177	GEO	*
256	CD24	GEO	*
257	CD36	Both	*
258	CD44	GEO	*
259	CD48	GEO	*
260	CD59	GEO	*
261	CD70	GEO	*
262	CD79A	GEO	*
263	CD81	GEO	*
264	CD97	GEO	*
265	CDADC1	GEO	*
266	CDC25B	GEO	*
267	CDC42BPA	GEO	*
268	CDC42EP1	GEO	*
269	CDC42EP5	GEO	*
270	CDC6	GEO	*
271	CDH1	Both	*
272	CDH2	GEO	*
273	CDH3	Both	*
274	CDH6	GEO	*
275	CDK19	GEO	*
276	CDK4	GEO	*
277	CDKL5	GEO	*
278	CDKN1C	GEO	*
279	CDKN2A	Both	*
280	CDKN2B	Both	*
281	CEACAM1	GEO	*
282	CEACAM5	GEO	*
283	CEACAM6	GEO	*
284	CEACAM7	GEO	*
285	CENPC1	GEO	*
286	CERK	GEO	*
287	CES2	Both	*
288	CFB	GEO	*
289	CFD	GEO	*
290	CFH	Both	*
291	CFL2	GEO	*
292	CFTR	Both	*
293	CHGA	GEO	*
294	CHI3L1	GEO	*
295	CHL1	GEO	*
296	CHMP4B	GEO	*
297	CHP2	GEO	*
298	CHRM3	GEO	*

299	CHST11	GEO	*
300	CHST6	GEO	*
301	CIRBP	GEO	*
302	CIRH1A	GEO	*
303	CITED1	GEO	*
304	CKB	GEO	*
305	CLCA1	GEO	*
306	CLCA4	GEO	*
307	CLCN2	GEO	*
308	CLDN1	GEO	*
309	CLDN2	GEO	*
310	CLDN23	GEO	*
311	CLEC1A	GEO	*
312	CLEC2B	GEO	*
313	CLEC3B	GEO	*
314	CLIC3	GEO	*
315	CLIC5	GEO	*
316	CLMN	GEO	*
317	CLN6	GEO	*
318	CLRN3	GEO	*
319	CLU	GEO	*
320	CMAH	GEO	*
321	CMTM4	GEO	*
322	CNIH3	GEO	*
323	CNNM2	GEO	*
324	CNRIP1	GEO	*
325	CNTN1	GEO	*
326	CNTN3	GEO	*
327	COL10A1	GEO	*
328	COL11A1	Both	*
329	COL13A1	GEO	*
330	COL17A1	GEO	*
331	COL1A1	GEO	*
332	COL1A2	GEO	*
333	COL28A1	GEO	*
334	COL3A1	GEO	*
335	COL6A1	GEO	*
336	COL6A2	GEO	*
337	COL9A3	GEO	*
338	COLEC11	GEO	*
339	COLEC12	GEO	*
340	COMT	Both	*
341	COPS2	GEO	*
342	COPS7A	GEO	*
343	COQ10A	GEO	*
344	CORO2A	GEO	*
345	CP	GEO	*
346	CPA3	GEO	*
347	CPM	GEO	*
348	CPNE8	GEO	*

349	CPSF4	GEO	*
350	CPT2	GEO	*
351	CRAT	GEO	*
352	CREB3L1	GEO	*
353	CREG2	GEO	*
354	CRISPLD2	GEO	*
355	CRP	Both	*
356	CSDA	GEO	*
357	CSE1L	GEO	*
358	CSF1R	Both	*
359	CSF2	GEO	*
360	CSGALNACT1	GEO	*
361	CST6	GEO	*
362	CTAGE5	GEO	*
363	CTSC	GEO	*
364	CTSK	GEO	*
365	CTSL1	GEO	*
366	CTSZ	GEO	*
367	CUL9	GEO	*
368	CUZD1	GEO	*
369	CWH43	GEO	*
370	CXADR	GEO	*
371	CXCL1	GEO	*
372	CXCL12	Both	*
373	CXCL16	GEO	*
374	CXCL3	GEO	*
375	CXXC4	GEO	*
376	CYB5R4	GEO	*
377	CYFIP2	GEO	*
378	CYGB	GEO	*
379	CYLD	GEO	*
380	CYP1B1	Both	*
381	CYP39A1	GEO	*
382	CYS1	GEO	*
383	DAPK1	Both	*
384	DAPP1	GEO	*
385	DCLK1	GEO	*
386	DCN	GEO	*
387	DCP2	GEO	*
388	DCPS	GEO	*
389	DCTN4	GEO	*
390	DDAH1	GEO	*
391	DDB1	GEO	*
392	DDI2	GEO	*
393	DDR2	GEO	*
394	DDX50	GEO	*
395	DDX54	GEO	*
396	DDX55	GEO	*
397	DEF6	GEO	*
398	DEGS2	GEO	*

399	DENND1B	Both	*
400	DENND2A	GEO	*
401	DENND5B	GEO	*
402	DES	GEO	*
403	DGKE	GEO	*
404	DGKG	GEO	*
405	DHRS11	GEO	*
406	DHRS8	GEO	*
407	DHRS9	GEO	*
408	DHX30	GEO	*
409	DHX57	GEO	*
410	DIABLO	GEO	*
411	DIO2	GEO	*
412	DKC1	GEO	*
413	DMKN	GEO	*
414	DMXL2	GEO	*
415	DNAH5	GEO	*
416	DNAJC15	GEO	*
417	DNAJC16	GEO	*
418	DNAJC18	GEO	*
419	DNAJC19	GEO	*
420	DNAJC22	GEO	*
421	DNM3	GEO	*
422	DOCK10	GEO	*
423	DOCK11	GEO	*
424	DOCK4	GEO	*
425	DPEP1	GEO	*
426	DPF2	GEO	*
427	DPP7	GEO	*
428	DPY19L1	GEO	*
429	DPY19L4	GEO	*
430	DSC2	GEO	*
431	DSE	GEO	*
432	DST	GEO	*
433	DTD1	GEO	*
434	DUSP14	GEO	*
435	DUSP26	GEO	*
436	DVL2	GEO	*
437	DYNC1LI1	GEO	*
438	DYNC1LI2	GEO	*
439	DZIP3	GEO	*
440	E2F8	GEO	*
441	EARS2	GEO	*
442	EBAG9	GEO	*
443	ECH1	GEO	*
444	ECHDC1	GEO	*
445	ECM2	GEO	*
446	ECT2	GEO	*
447	EDEM2	GEO	*
448	EDIL3	GEO	*

449	EDN3	GEO	*
450	EEF1A2	GEO	*
451	EEPD1	GEO	*
452	EFNA5	GEO	*
453	EFTUD2	GEO	*
454	EGFL6	GEO	*
455	EHF	GEO	*
456	EIF2C2	GEO	*
457	EIF3B	GEO	*
458	EIF5A	GEO	*
459	ELFN2	GEO	*
460	ELL3	GEO	*
461	ELOVL5	GEO	*
462	EMP1	GEO	*
463	EMP2	GEO	*
464	ENC1	GEO	*
465	ENDOD1	GEO	*
466	ENO1	GEO	*
467	ENPP1	GEO	*
468	ENTPD5	Both	*
469	EPB41L1	GEO	*
470	EPB41L3	GEO	*
471	EPDR1	GEO	*
472	EPHA2	GEO	*
473	EPHB2	Both	*
474	EPHB4	GEO	*
475	EPHX2	Both	*
476	EPM2A	GEO	*
477	ERAP2	Both	*
478	ERBB2	Both	*
479	EREG	GEO	*
480	ESM1	GEO	*
481	ESYT1	GEO	*
482	ETFDH	GEO	*
483	ETHE1	GEO	*
484	ETS2	GEO	*
485	ETV1	GEO	*
486	ETV4	GEO	*
487	EVI1	GEO	*
488	EVI2B	GEO	*
489	EXOSC2	GEO	*
490	EXTL2	GEO	*
491	EZH2	GEO	*
492	EZR	GEO	*
493	F12	GEO	*
494	F13A1	Both	*
495	FABP1	GEO	*
496	FABP3	GEO	*
497	FABP4	GEO	*
498	FABP5	GEO	*

499	FABP6	GEO	*
500	FADS1	GEO	*
501	FAM101B	GEO	*
502	FAM105B	GEO	*
503	FAM107A	GEO	*
504	FAM117A	GEO	*
505	FAM117B	GEO	*
506	FAM122B	GEO	*
507	FAM126A	GEO	*
508	FAM129B	GEO	*
509	FAM134C	GEO	*
510	FAM135A	Both	*
511	FAM136A	GEO	*
512	FAM149A	GEO	*
513	FAM150A	GEO	*
514	FAM160B1	GEO	*
515	FAM164A	GEO	*
516	FAM165B	GEO	*
517	FAM171B	GEO	*
518	FAM188B	GEO	*
519	FAM20C	GEO	*
520	FAM36A	GEO	*
521	FAM46C	GEO	*
522	FAM47E	GEO	*
523	FAM55D	GEO	*
524	FAM82A1	GEO	*
525	FAM92A1	GEO	*
526	FAM96A	GEO	*
527	FANCE	GEO	*
528	FAP	GEO	*
529	FASTKD1	GEO	*
530	FAT1	GEO	*
531	FBLN1	GEO	*
532	FBP1	GEO	*
533	FBXO28	GEO	*
534	FBXO7	GEO	*
535	FBXO9	GEO	*
536	FCGBP	GEO	*
537	FCHO1	GEO	*
538	FECH	GEO	*
539	FEN1	GEO	*
540	FERMT1	GEO	*
541	FERMT2	GEO	*
542	FEV	GEO	*
543	FGD1	GEO	*
544	FGD5	GEO	*
545	FGF20	GEO	*
546	FGFR2	GEO	*
547	FGL2	GEO	*
548	FHL1	GEO	*

549	FIBP	GEO	*
550	FKBP10	GEO	*
551	FKBP1B	GEO	*
552	FLJ21511	GEO	*
553	FLJ32063	GEO	*
554	FLJ32255	GEO	*
555	FLJ36848	GEO	*
556	FLJ39051	GEO	*
557	FLJ44606	GEO	*
558	FLJ45248	GEO	*
559	FLVCR2	GEO	*
560	FN1	GEO	*
561	FNBP1	GEO	*
562	FNIP2	GEO	*
563	FOXA1	GEO	*
564	FOXA2	GEO	*
565	FOXF1	GEO	*
566	FOXF2	GEO	*
567	FOXM1	GEO	*
568	FOXO1	GEO	*
569	FOXP1	GEO	*
570	FOXQ1	GEO	*
571	FRMD4B	GEO	*
572	FRMD6	GEO	*
573	FRZB	Both	*
574	FSCN1	GEO	*
575	FTH1	GEO	*
576	FTSJ1	GEO	*
577	FUT1	GEO	*
578	FUT8	GEO	*
579	FXYD1	GEO	*
580	FXYD3	GEO	*
581	FXYD5	GEO	*
582	FXYD6	GEO	*
583	FZD2	GEO	*
584	FZD5	GEO	*
585	FZD9	GEO	*
586	G6PC3	GEO	*
587	G6PD	GEO	*
588	GAB1	GEO	*
589	GABBR1	GEO	*
590	GADD45B	GEO	*
591	GALNAC4S-6ST	GEO	*
592	GALNT12	Both	*
593	GALNT6	GEO	*
594	GALNTL4	GEO	*
595	GAMT	GEO	*
596	GANAB	GEO	*
597	GART	Both	*
598	GAS2	GEO	*

599	GAS6	GEO	*
600	GAS7	Both	*
601	GBP3	GEO	*
602	GCG	GEO	*
603	GCNT2	GEO	*
604	GCNT3	GEO	*
605	GCSH	GEO	*
606	GDF15	GEO	*
607	GDPD2	GEO	*
608	GDPD5	GEO	*
609	GEM	GEO	*
610	GFRA2	GEO	*
611	GGT1	GEO	*
612	GGTA1	GEO	*
613	GIMAP1	GEO	*
614	GIMAP6	GEO	*
615	GINS1	GEO	*
616	GIPC2	GEO	*
617	GJA3	GEO	*
618	GLB1L2	GEO	*
619	GLIS2	GEO	*
620	GLO1	GEO	*
621	GLP2R	Both	*
622	GLS	GEO	*
623	GLS2	GEO	*
624	GLTP	GEO	*
625	GMCL1	GEO	*
626	GMDS	GEO	*
627	GMPS	GEO	*
628	GNA11	GEO	*
629	GNAS	Both	*
630	GNAZ	GEO	*
631	GNB4	Both	*
632	GNG10	GEO	*
633	GNG2	GEO	*
634	GNG4	GEO	*
635	GNG7	GEO	*
636	GNPDA1	GEO	*
637	GNS	GEO	*
638	GOLM1	GEO	*
639	GOLT1A	GEO	*
640	GPA33	GEO	*
641	GPC1	GEO	*
642	GPC6	GEO	*
643	GPCPD1	GEO	*
644	GPD1L	GEO	*
645	GPER	GEO	*
646	GPM6B	GEO	*
647	GPN3	GEO	*
648	GPNMB	GEO	*

649	GPR115	GEO	*
650	GPR137B	GEO	*
651	GPR160	GEO	*
652	GPR176	GEO	*
653	GPR56	GEO	*
654	GPRC5A	GEO	*
655	GPRIN1	GEO	*
656	GPT	GEO	*
657	GPX2	Both	*
658	GPX3	Both	*
659	GRAMD1A	GEO	*
660	GRAMD1B	GEO	*
661	GRAMD3	GEO	*
662	GRB10	GEO	*
663	GREM2	GEO	*
664	GRHL1	GEO	*
665	GRIN2B	GEO	*
666	GRK5	GEO	*
667	GRPEL1	GEO	*
668	GRPEL2	GEO	*
669	GRTP1	GEO	*
670	GSK3A	GEO	*
671	GSN	GEO	*
672	GTF2IRD1	GEO	*
673	GTF3A	GEO	*
674	GTPBP2	GEO	*
675	GUCA2A	GEO	*
676	GUCA2B	GEO	*
677	GZMA	GEO	*
678	GZMB	GEO	*
679	H2AFJ	GEO	*
680	H2AFY2	GEO	*
681	HABP2	GEO	*
682	HAPLN1	GEO	*
683	HAPLN3	GEO	*
684	HBS1L	GEO	*
685	HCN1	GEO	*
686	HDAC9	Both	*
687	HEPACAM2	GEO	*
688	HEXIM1	GEO	*
689	HGD	GEO	*
690	HHLA2	GEO	*
691	HIAT1	GEO	*
692	HIG2	GEO	*
693	HIGD1A	GEO	*
694	HIPK2	GEO	*
695	HIST1H2BG	GEO	*
696	HKDC1	GEO	*
697	HLA-DMA	GEO	*
698	HLA-DRB1	Both	*

699	HLTF	GEO	*
700	HMG20B	GEO	*
701	HMGA1	GEO	*
702	HMGA2	GEO	*
703	HMGN4	GEO	*
704	HNF1B	GEO	*
705	HNF4A	Both	*
706	HNF4G	GEO	*
707	HNMT	Both	*
708	HNRNPA0	GEO	*
709	HNRNPH1	GEO	*
710	HOXA13	GEO	*
711	HOXA5	GEO	*
712	HP	Both	*
713	HPCAL4	GEO	*
714	HPGD	Both	*
715	HPS3	GEO	*
716	HRASLS2	GEO	*
717	HS6ST2	GEO	*
718	HSBP1	GEO	*
719	HSD11B2	GEO	*
720	HSD17B2	Both	*
721	HSDL2	GEO	*
722	HSPA12A	GEO	*
723	HSPA13	GEO	*
724	HSPA2	Both	*
725	HSPB1	GEO	*
726	HSPH1	GEO	*
727	HTR4	GEO	*
728	ICOS	GEO	*
729	ID2	GEO	*
730	IFIT1	GEO	*
731	IFITM1	GEO	*
732	IFITM2	GEO	*
733	IFITM3	Both	*
734	IFRD1	GEO	*
735	IGF1	Both	*
736	IGFBP3	Both	*
737	IGHA1	GEO	*
738	IGHG1	Both	*
739	IGJ	GEO	*
740	IL15	GEO	*
741	IL1R2	GEO	*
742	IL24	GEO	*
743	IL27RA	GEO	*
744	IL6R	GEO	*
745	IL8	Both	*
746	ILDR1	GEO	*
747	INHBA	Both	*
748	INSIG2	GEO	*

749	INTS5	GEO	*
750	IPO5	GEO	*
751	IPO8	GEO	*
752	IRAK2	GEO	*
753	IRF4	GEO	*
754	ISG20	GEO	*
755	ITGA11	GEO	*
756	ITGA5	GEO	*
757	ITGA6	GEO	*
758	ITGAE	GEO	*
759	ITGAV	GEO	*
760	ITGB6	GEO	*
761	ITGB8	GEO	*
762	ITIH2	GEO	*
763	ITIH5	GEO	*
764	ITLN1	Both	*
765	ITM2C	GEO	*
766	ITPR1	GEO	*
767	ITPRIPL2	GEO	*
768	JAG2	GEO	*
769	JAM3	GEO	*
770	JAZF1	GEO	*
771	JMJD6	GEO	*
772	JUB	GEO	*
773	KAL1	GEO	*
774	KANK2	GEO	*
775	KANK4	GEO	*
776	KATNB1	GEO	*
777	KCMF1	GEO	*
778	KCND3	GEO	*
779	KCNE3	GEO	*
780	KCNH2	GEO	*
781	KCTD12	GEO	*
782	KCTD14	GEO	*
783	KDELC1	GEO	*
784	KIAA0040	GEO	*
785	KIAA0828	GEO	*
786	KIAA1199	GEO	*
787	KIAA1217	GEO	*
788	KIAA1244	GEO	*
789	KIAA1257	GEO	*
790	KIAA1324	GEO	*
791	KIAA1324L	GEO	*
792	KIAA1370	GEO	*
793	KIAA1468	GEO	*
794	KIAA1549	GEO	*
795	KIF13B	GEO	*
796	KIF1B	GEO	*
797	KIF20A	GEO	*
798	KIF2C	GEO	*

799	KIF5B	GEO	*
800	KLC2	GEO	*
801	KLC3	GEO	*
802	KLF4	GEO	*
803	KLF6	GEO	*
804	KLF9	GEO	*
805	KLHL11	GEO	*
806	KLHL29	GEO	*
807	KLK10	GEO	*
808	KPNB1	GEO	*
809	KREMEN1	GEO	*
810	KRT20	GEO	*
811	KRT23	GEO	*
812	KRT24	GEO	*
813	KRT6B	GEO	*
814	KRT80	GEO	*
815	KRT86	Both	*
816	L3MBTL3	GEO	*
817	LACTB	GEO	*
818	LAMB1	Both	*
819	LAMB3	GEO	*
820	LAMC2	GEO	*
821	LAPTM5	GEO	*
822	LASS2	GEO	*
823	LDHB	GEO	*
824	LDHD	GEO	*
825	LDLRAD3	GEO	*
826	LEF1	GEO	*
827	LEMD1	GEO	*
828	LEPRE1	GEO	*
829	LEPROTL1	GEO	*
830	LGALS2	GEO	*
831	LGALS4	GEO	*
832	LGR5	GEO	*
833	LIFR	GEO	*
834	LIMCH1	GEO	*
835	LIPE	GEO	*
836	LIPG	GEO	*
837	LITAF	GEO	*
838	LMNB1	GEO	*
839	LMO2	GEO	*
840	LMO6	GEO	*
841	LNX1	GEO	*
842	LOC100127983	GEO	*
843	LOC100132288	GEO	*
844	LOC100133036	GEO	*
845	LOC100287411	GEO	*
846	LOC100288413	GEO	*
847	LOC100289019	GEO	*
848	LOC100289410	GEO	*

849	LOC100506621	GEO	*
850	LOC221710	GEO	*
851	LOC339290	GEO	*
852	LOC344887	GEO	*
853	LOC388796	GEO	*
854	LOC399959	GEO	*
855	LOC400573	GEO	*
856	LOC442198	GEO	*
857	LOC643837	GEO	*
858	LOC654433	GEO	*
859	LOH11CR2A	GEO	*
860	LOX	GEO	*
861	LOXL2	GEO	*
862	LOXL4	GEO	*
863	LPAR1	GEO	*
864	LPAR5	GEO	*
865	LPCAT1	GEO	*
866	LPHN3	GEO	*
867	LPPR4	GEO	*
868	LPXN	GEO	*
869	LRG1	GEO	*
870	LRIG1	GEO	*
871	LRP4	GEO	*
872	LRP8	GEO	*
873	LRRC19	GEO	*
874	LRRC34	GEO	*
875	LRRC8A	GEO	*
876	LRRN2	GEO	*
877	LTBP2	GEO	*
878	LUZP6	GEO	*
879	LXN	GEO	*
880	LY6G6D	GEO	*
881	LY75	GEO	*
882	LYZ	GEO	*
883	M6PR	GEO	*
884	MAB21L2	GEO	*
885	MACC1	GEO	*
886	MAEA	GEO	*
887	MAF	GEO	*
888	MAK16	GEO	*
889	MALL	GEO	*
890	MAMDC2	GEO	*
891	MAML2	GEO	*
892	MAML3	GEO	*
893	MAN1A1	GEO	*
894	MAN2A1	GEO	*
895	MAOA	GEO	*
896	MAOB	GEO	*
897	MAP1B	GEO	*
898	MAP1LC3B	GEO	*

899	MAP2K6	GEO	*
900	MAP3K1	Both	*
901	MAP3K8	GEO	*
902	MAP4K4	GEO	*
903	MAP7D3	GEO	*
904	MAPK1	GEO	*
905	MAPK10	GEO	*
906	MAPK12	GEO	*
907	MAPKAP1	GEO	*
908	MAPKSP1	GEO	*
909	MAPRE1	GEO	*
910	MARVELD2	GEO	*
911	MASTL	GEO	*
912	MAT2B	GEO	*
913	MATN2	GEO	*
914	MCC	GEO	*
915	MCF2L	GEO	*
916	MED13L	GEO	*
917	MED14	GEO	*
918	MED20	GEO	*
919	MEF2C	GEO	*
920	MEIS1	GEO	*
921	MEN1	GEO	*
922	MEP1A	Both	*
923	MEST	GEO	*
924	MET	GEO	*
925	METTL7A	GEO	*
926	MFAP2	GEO	*
927	MFAP3L	GEO	*
928	MGAT4A	GEO	*
929	MGC13057	GEO	*
930	MGC14376	GEO	*
931	MGC39372	GEO	*
932	MGC4172	GEO	*
933	MGLL	GEO	*
934	MICAL2	GEO	*
935	MICB	Both	*
936	MID1	GEO	*
937	MLF2	GEO	*
938	MLLT3	GEO	*
939	MMP1	Both	*
940	MMP11	GEO	*
941	MMP12	Both	*
942	MMP14	GEO	*
943	MMP28	GEO	*
944	MMP3	Both	*
945	MMP7	Both	*
946	MOBKL2B	GEO	*
947	MOCS1	GEO	*
948	MORC4	GEO	*

949	MORN4	GEO	*
950	MPEG1	GEO	*
951	MPPE1	GEO	*
952	MPZL2	GEO	*
953	MRC1	Both	*
954	MRPL37	GEO	*
955	MRPS2	GEO	*
956	MRS2	GEO	*
957	MS4A12	GEO	*
958	MS4A7	GEO	*
959	MSLN	GEO	*
960	MSX2	GEO	*
961	MT1E	GEO	*
962	MT1F	GEO	*
963	MT1H	GEO	*
964	MT1M	GEO	*
965	MT1X	GEO	*
966	MT2A	GEO	*
967	MTHFD1L	GEO	*
968	MTHFD2	GEO	*
969	MTM1	Both	*
970	MUC13	GEO	*
971	MUC16	GEO	*
972	MUM1L1	GEO	*
973	MUSK	GEO	*
974	MXD1	GEO	*
975	MXRA5	GEO	*
976	MXRA7	GEO	*
977	MYC	Both	*
978	MYH11	GEO	*
979	MYH7B	GEO	*
980	MYL9	GEO	*
981	MYLIP	GEO	*
982	MYLK	GEO	*
983	MYO1A	GEO	*
984	MYO5A	GEO	*
985	MYO6	GEO	*
986	MYOM1	GEO	*
987	MYOT	GEO	*
988	NAA30	GEO	*
989	NAAA	GEO	*
990	NAB2	GEO	*
991	NAMPT	GEO	*
992	NANP	GEO	*
993	NAP1L1	GEO	*
994	NAP1L2	GEO	*
995	NAT10	GEO	*
996	NAV1	GEO	*
997	NBEA	GEO	*
998	NCAM1	GEO	*

999	NCRNA00094	GEO	*
1000	NCRNA00118	GEO	*
1001	NCRNA00173	GEO	*
1002	NDN	GEO	*
1003	NDRG2	GEO	*
1004	NEBL	GEO	*
1005	NECAP1	GEO	*
1006	NEDD4L	GEO	*
1007	NEDD9	GEO	*
1008	NEGR1	GEO	*
1009	NEK2	GEO	*
1010	NEK5	GEO	*
1011	NET1	GEO	*
1012	NEU3	GEO	*
1013	NEUROD1	GEO	*
1014	NFAT5	GEO	*
1015	NFE2L3	GEO	*
1016	NFIA	GEO	*
1017	NFIB	GEO	*
1018	NIN	GEO	*
1019	NISCH	GEO	*
1020	NKD1	GEO	*
1021	NKD2	GEO	*
1022	NKX3-1	GEO	*
1023	NLRP2	Both	*
1024	NMD3	GEO	*
1025	NMT1	GEO	*
1026	NNMT	GEO	*
1027	NOC3L	GEO	*
1028	NOC4L	GEO	*
1029	NOD2	Both	*
1030	NONO	GEO	*
1031	NOP14	GEO	*
1032	NOP56	GEO	*
1033	NOSTRIN	GEO	*
1034	NOV	GEO	*
1035	NPM2	GEO	*
1036	NPNT	GEO	*
1037	NR2F2	Both	*
1038	NR3C1	Both	*
1039	NR3C2	GEO	*
1040	NR4A2	GEO	*
1041	NR5A2	GEO	*
1042	NRP1	GEO	*
1043	NSUN5	GEO	*
1044	NT5C3	GEO	*
1045	NT5C3L	GEO	*
1046	NUCB2	GEO	*
1047	NUDT11	GEO	*
1048	NUMBL	GEO	*
1049	NUP88	GEO	*
1050	NUP98	GEO	*
1051	NXT2	GEO	*
1052	ODAM	GEO	*
1053	ODF2	GEO	*
1054	OGFOD1	GEO	*
1055	OGFRL1	GEO	*
1056	OLFML1	GEO	*
1057	OLFML2A	GEO	*
1058	OLFML2B	GEO	*
1059	OSBPL10	GEO	*
1060	OSBPL1A	GEO	*
1061	OSBPL2	GEO	*
1062	OSBPL3	GEO	*
1063	OSBPL6	GEO	*
1064	OSBPL7	GEO	*
1065	OSTbeta	GEO	*
1066	OTUD1	GEO	*
1067	P4HA3	GEO	*
1068	PA2G4	GEO	*
1069	PADI2	GEO	*
1070	PAFAH1B2	GEO	*
1071	PAG1	GEO	*
1072	PAH	GEO	*
1073	PAM	GEO	*
1074	PAPPA	GEO	*
1075	PAPSS2	GEO	*
1076	PAQR5	GEO	*
1077	PAQR8	GEO	*
1078	PAX6	GEO	*
1079	PBK	GEO	*
1080	PBLD	GEO	*
1081	PBX3	GEO	*
1082	PC	GEO	*
1083	PCK1	GEO	*
1084	PCLO	GEO	*
1085	PCOLCE2	GEO	*
1086	PCSK6	GEO	*
1087	PDCD4	GEO	*
1088	PDCD6	GEO	*
1089	PDE10A	GEO	*
1090	PDE2A	GEO	*
1091	PDE4D	GEO	*
1092	PDE9A	GEO	*
1093	PDGFRA	GEO	*
1094	PDGFRL	GEO	*
1095	PDIA6	GEO	*
1096	PDK4	GEO	*
1097	PDLIM3	GEO	*
1098	PDPN	GEO	*

1099	PDS5B	GEO	*
1100	PDXK	GEO	*
1101	PDZD2	GEO	*
1102	PECI	GEO	*
1103	PEG10	GEO	*
1104	PELI1	GEO	*
1105	PELI2	GEO	*
1106	PERLD1	GEO	*
1107	PERP	GEO	*
1108	PFKFB2	GEO	*
1109	PGM5	GEO	*
1110	PHF16	GEO	*
1111	PHF19	GEO	*
1112	PHF20	GEO	*
1113	PHLDA1	GEO	*
1114	PHLDB2	GEO	*
1115	PHLPP2	GEO	*
1116	PIAS2	GEO	*
1117	PIGR	GEO	*
1118	PIGW	GEO	*
1119	PIK3AP1	GEO	*
1120	PIK3CD	GEO	*
1121	PIK3R1	Both	*
1122	PITX1	GEO	*
1123	PJA1	GEO	*
1124	PKIB	GEO	*
1125	PKN3	GEO	*
1126	PLA2G10	GEO	*
1127	PLA2G12A	GEO	*
1128	PLA2G16	GEO	*
1129	PLA2G4A	GEO	*
1130	PLAC8	GEO	*
1131	PLAC9	GEO	*
1132	PLAGL1	GEO	*
1133	PLAU	Both	*
1134	PLAUR	GEO	*
1135	PLCD3	GEO	*
1136	PLCE1	GEO	*
1137	PLCL2	GEO	*
1138	PLD1	GEO	*
1139	PLEKHA3	GEO	*
1140	PLEKHB2	GEO	*
1141	PLEKHF2	GEO	*
1142	PLEKHG6	GEO	*
1143	PLG	GEO	*
1144	PLOD2	GEO	*
1145	PLP1	GEO	*
1146	PLS1	GEO	*
1147	PMAIP1	GEO	*
1148	PMEPA1	GEO	*

1149	PNKD	GEO	*
1150	POF1B	GEO	*
1151	POLD2	GEO	*
1152	POLD4	GEO	*
1153	POLDIP2	GEO	*
1154	POSTN	GEO	*
1155	PPAP2A	GEO	*
1156	PPARGC1B	GEO	*
1157	PPAT	GEO	*
1158	PPEF1	GEO	*
1159	PPFIBP2	GEO	*
1160	PPID	GEO	*
1161	PPL	GEO	*
1162	PPM1H	GEO	*
1163	PPM1K	GEO	*
1164	PPP1CC	GEO	*
1165	PPP1R14C	GEO	*
1166	PPP1R16B	GEO	*
1167	PPP1R2	GEO	*
1168	PPP2CA	GEO	*
1169	PPP2R1B	GEO	*
1170	PPP2R2C	GEO	*
1171	PPP2R3A	GEO	*
1172	PQLC3	GEO	*
1173	PRC1	GEO	*
1174	PRDX6	GEO	*
1175	PREB	GEO	*
1176	PRELID2	GEO	*
1177	PREP	GEO	*
1178	PRICKLE1	GEO	*
1179	PRIM2	GEO	*
1180	PRKACB	GEO	*
1181	PRKAR2B	GEO	*
1182	PRKCH	GEO	*
1183	PRMT5	GEO	*
1184	PROX1	GEO	*
1185	PRPH	GEO	*
1186	PRSS22	GEO	*
1187	PRSS23	GEO	*
1188	PRSS3	GEO	*
1189	PSAT1	GEO	*
1190	PSIP1	GEO	*
1191	PSMF1	GEO	*
1192	PSTPIP2	GEO	*
1193	PTGDR	GEO	*
1194	PTGER2	Both	*
1195	PTGER3	GEO	*
1196	PTGES	Both	*
1197	PTGIS	Both	*
1198	PTP4A1	GEO	*

1199	PTP4A3	GEO	*
1200	PTPLA	GEO	*
1201	PTPRB	GEO	*
1202	PTPRG	GEO	*
1203	PTPRH	GEO	*
1204	PTPRR	GEO	*
1205	PTRF	Both	*
1206	PTRH1	GEO	*
1207	PURB	GEO	*
1208	PUS7	GEO	*
1209	PWP2	GEO	*
1210	PYCARD	GEO	*
1211	PYGL	GEO	*
1212	PYY	GEO	*
1213	PYY2	GEO	*
1214	QPCT	GEO	*
1215	QSOX1	GEO	*
1216	RAB11FIP4	GEO	*
1217	RAB25	GEO	*
1218	RAB27B	GEO	*
1219	RAB34	GEO	*
1220	RAB37	GEO	*
1221	RAB3IP	GEO	*
1222	RAB8B	GEO	*
1223	RABGAP1	GEO	*
1224	RAE1	GEO	*
1225	RAN	GEO	*
1226	RANBP9	GEO	*
1227	RAP2C	GEO	*
1228	RAPH1	GEO	*
1229	RARRES1	GEO	*
1230	RARRES2	Both	*
1231	RASA1	GEO	*
1232	RASAL1	GEO	*
1233	RASEF	GEO	*
1234	RASGRP3	GEO	*
1235	RASSF4	GEO	*
1236	RASSF6	GEO	*
1237	RAVER2	GEO	*
1238	RBL2	GEO	*
1239	RBM18	GEO	*
1240	RBM24	GEO	*
1241	RBMS2	GEO	*
1242	RBMS3	GEO	*
1243	RCAN1	GEO	*
1244	RCAN2	GEO	*
1245	RCAN3	GEO	*
1246	RCC2	GEO	*
1247	RCOR2	GEO	*
1248	RDH5	GEO	*

1249	RDX	GEO	*
1250	RECQL	Both	*
1251	RELB	GEO	*
1252	RELL1	GEO	*
1253	REPS2	GEO	*
1254	RETSAT	GEO	*
1255	REXO2	GEO	*
1256	RFC3	GEO	*
1257	RFFL	GEO	*
1258	RGL1	GEO	*
1259	RGMA	GEO	*
1260	RGNEF	GEO	*
1261	RGS10	GEO	*
1262	RGS12	GEO	*
1263	RGS2	GEO	*
1264	RGS9	GEO	*
1265	RHBDF1	GEO	*
1266	RHEBL1	GEO	*
1267	RHOF	GEO	*
1268	RHOT1	GEO	*
1269	RHOU	GEO	*
1270	RIBC2	GEO	*
1271	RICS	GEO	*
1272	RIN2	GEO	*
1273	RIN3	GEO	*
1274	RIOK3	GEO	*
1275	RIPK2	GEO	*
1276	RMND5A	GEO	*
1277	RNF130	GEO	*
1278	RNF138	GEO	*
1279	RNF144A	GEO	*
1280	RNF152	GEO	*
1281	RNF19B	GEO	*
1282	RNF219	GEO	*
1283	RNF32	GEO	*
1284	RNF43	GEO	*
1285	RNH1	GEO	*
1286	RPN2	GEO	*
1287	RPRD1A	GEO	*
1288	RPS19BP1	GEO	*
1289	RPS6KA2	Both	*
1290	RPUSD4	GEO	*
1291	RRAGD	GEO	*
1292	RSL1D1	GEO	*
1293	RSPRY1	GEO	*
1294	RTEL1	GEO	*
1295	RUNDC3B	GEO	*
1296	RUNX1T1	GEO	*
1297	RUNX2	GEO	*
1298	RUNX3	Both	*

1299	RUVBL1	GEO	*
1300	RUVBL2	GEO	*
1301	S100A11	GEO	*
1302	S100A2	GEO	*
1303	S100P	GEO	*
1304	SAP130	GEO	*
1305	SAP30L	GEO	*
1306	SATB1	GEO	*
1307	SCARA5	GEO	*
1308	SCD	GEO	*
1309	SCG2	GEO	*
1310	SCGN	GEO	*
1311	SCIN	GEO	*
1312	SCN9A	GEO	*
1313	SCNN1B	GEO	*
1314	SCRIB	GEO	*
1315	SDC3	GEO	*
1316	SDCBP2	GEO	*
1317	SDCCAG1	GEO	*
1318	SDPR	GEO	*
1319	SE57-1	GEO	*
1320	SEC14L2	GEO	*
1321	SEH1L	GEO	*
1322	SELENBP1	GEO	*
1323	SEMA4D	GEO	*
1324	SEMA4G	GEO	*
1325	SEMA6A	GEO	*
1326	SEMA6D	GEO	*
1327	SEPP1	Both	*
1328	SEPT8	GEO	*
1329	SERPINB1	GEO	*
1330	SERPINB5	GEO	*
1331	SERPINB9	GEO	*
1332	SERPINH1	GEO	*
1333	SERTAD4	GEO	*
1334	SET	GEO	*
1335	SETBP1	GEO	*
1336	SF3A1	GEO	*
1337	SF3B3	GEO	*
1338	SFRP1	GEO	*
1339	SFRS18	GEO	*
1340	SFXN5	GEO	*
1341	SGCE	GEO	*
1342	SGIP1	GEO	*
1343	SGK1	GEO	*
1344	SGK2	GEO	*
1345	SGK493	GEO	*
1346	SGMS2	GEO	*
1347	SGSM1	GEO	*
1348	SH3D19	GEO	*

1349	SH3TC2	GEO	*
1350	SHANK2	GEO	*
1351	SHMT2	GEO	*
1352	SIGLEC1	GEO	*
1353	SIGLEC6	GEO	*
1354	SIM2	GEO	*
1355	SIPA1L1	GEO	*
1356	SIX4	GEO	*
1357	SLC12A2	GEO	*
1358	SLC12A7	GEO	*
1359	SLC16A9	GEO	*
1360	SLC1A1	GEO	*
1361	SLC1A7	GEO	*
1362	SLC20A1	GEO	*
1363	SLC22A3	GEO	*
1364	SLC22A4	Both	*
1365	SLC25A15	GEO	*
1366	SLC25A23	GEO	*
1367	SLC25A24	GEO	*
1368	SLC25A34	GEO	*
1369	SLC25A37	GEO	*
1370	SLC25A39	GEO	*
1371	SLC26A11	GEO	*
1372	SLC26A2	GEO	*
1373	SLC26A3	Both	*
1374	SLC26A6	GEO	*
1375	SLC27A2	GEO	*
1376	SLC2A1	GEO	*
1377	SLC2A13	GEO	*
1378	SLC2A4	GEO	*
1379	SLC30A10	GEO	*
1380	SLC30A4	GEO	*
1381	SLC35A3	GEO	*
1382	SLC35B4	GEO	*
1383	SLC37A1	GEO	*
1384	SLC38A1	GEO	*
1385	SLC38A5	GEO	*
1386	SLC39A10	GEO	*
1387	SLC39A6	GEO	*
1388	SLC3A2	GEO	*
1389	SLC40A1	GEO	*
1390	SLC41A1	GEO	*
1391	SLC44A1	GEO	*
1392	SLC46A3	GEO	*
1393	SLC4A11	GEO	*
1394	SLC4A4	GEO	*
1395	SLC4A8	GEO	*
1396	SLC6A20	GEO	*
1397	SLC6A6	GEO	*
1398	SLC7A2	GEO	*

1399	SLC7A5	GEO	*
1400	SLC9A2	GEO	*
1401	SLC9A7	GEO	*
1402	SLC9A9	GEO	*
1403	SLCO4A1	GEO	*
1404	SLIT2	GEO	*
1405	SMA4	GEO	*
1406	SMAD2	Both	*
1407	SMAD4	Both	*
1408	SMAD6	GEO	*
1409	SMARCA1	GEO	*
1410	SMPDL3A	GEO	*
1411	SMTN	GEO	*
1412	SMYD5	GEO	*
1413	SNAI1	GEO	*
1414	SNAI2	GEO	*
1415	SNAP25	GEO	*
1416	SNRNP48	GEO	*
1417	SNTB1	GEO	*
1418	SNX12	GEO	*
1419	SORD	GEO	*
1420	SOX4	GEO	*
1421	SOX9	GEO	*
1422	SP3	GEO	*
1423	SP5	GEO	*
1424	SPAG1	GEO	*
1425	SPAG16	GEO	*
1426	SPARC	GEO	*
1427	SPARCL1	GEO	*
1428	SPIB	GEO	*
1429	SPINK4	GEO	*
1430	SPIRE1	GEO	*
1431	SPNS2	GEO	*
1432	SPP1	GEO	*
1433	SPPL2A	GEO	*
1434	SQRDL	GEO	*
1435	SRI	GEO	*
1436	SRPR	GEO	*
1437	SRPX	GEO	*
1438	SRPX2	GEO	*
1439	SRSF6	GEO	*
1440	SSBP2	GEO	*
1441	SSPN	GEO	*
1442	SSR1	GEO	*
1443	SSRP1	GEO	*
1444	SST	GEO	*
1445	ST6GALNAC1	GEO	*
1446	ST6GALNAC2	GEO	*
1447	STAB1	GEO	*
1448	STAMBPL1	GEO	*

1449	STAP2	GEO	*
1450	STAU1	GEO	*
1451	STC1	GEO	*
1452	STC2	GEO	*
1453	STK24	GEO	*
1454	STMN2	GEO	*
1455	STMN3	GEO	*
1456	STON2	GEO	*
1457	STOX2	GEO	*
1458	STRADB	GEO	*
1459	STT3A	GEO	*
1460	STX3	GEO	*
1461	STXBP1	GEO	*
1462	STXBP4	GEO	*
1463	STXBP6	GEO	*
1464	STYK1	GEO	*
1465	STYX	GEO	*
1466	SULF1	GEO	*
1467	SULT1A1	Both	*
1468	SULT1A2	Both	*
1469	SULT2B1	GEO	*
1470	SUSD1	GEO	*
1471	SVIL	GEO	*
1472	SYK	GEO	*
1473	SYNC	GEO	*
1474	SYNCRIP	GEO	*
1475	SYNE2	GEO	*
1476	SYNGAP1	GEO	*
1477	SYNGR3	GEO	*
1478	SYNPO	GEO	*
1479	SYT17	GEO	*
1480	SYTL2	GEO	*
1481	TACSTD2	GEO	*
1482	TAGLN2	GEO	*
1483	TAPBP	GEO	*
1484	TBX20	GEO	*
1485	TC2N	GEO	*
1486	TCEA2	GEO	*
1487	TCEA3	GEO	*
1488	TCF21	GEO	*
1489	TCF4	GEO	*
1490	TCF7	Both	*
1491	TCN1	GEO	*
1492	TCOF1	GEO	*
1493	TEAD3	GEO	*
1494	TEAD4	GEO	*
1495	TERF2	GEO	*
1496	TERT	GEO	*
1497	TESC	GEO	*
1498	TEX10	GEO	*

1499	TFAP4	GEO	*
1500	TFCP2L1	GEO	*
1501	TGFB1	Both	*
1502	TGFBI	GEO	*
1503	TGFBR1	Both	*
1504	TGFBR2	Both	*
1505	TGIF1	GEO	*
1506	TGIF2	GEO	*
1507	THBS2	GEO	*
1508	TIAM1	GEO	*
1509	TIMM17A	GEO	*
1510	TIMP1	Both	*
1511	TIMP2	Both	*
1512	TIMP3	GEO	*
1513	TKT	GEO	*
1514	TLE1	Both	*
1515	TM4SF1	GEO	*
1516	TM7SF3	GEO	*
1517	TMC7	GEO	*
1518	TMCC3	GEO	*
1519	TMEFF1	GEO	*
1520	TMEM125	GEO	*
1521	TMEM127	GEO	*
1522	TMEM133	GEO	*
1523	TMEM139	GEO	*
1524	TMEM158	GEO	*
1525	TMEM167A	GEO	*
1526	TMEM171	GEO	*
1527	TMEM182	GEO	*
1528	TMEM185B	GEO	*
1529	TMEM222	GEO	*
1530	TMEM30A	GEO	*
1531	TMEM39B	GEO	*
1532	TMEM41B	GEO	*
1533	TMEM43	GEO	*
1534	TMEM54	GEO	*
1535	TMEM63A	GEO	*
1536	TMEM98	GEO	*
1537	TMEM99	GEO	*
1538	TMPRSS13	GEO	*
1539	TMPRSS2	GEO	*
1540	TMPRSS3	GEO	*
1541	TMTC2	GEO	*
1542	TNFRSF10B	Both	*
1543	TNFRSF11B	GEO	*
1544	TNFRSF12A	GEO	*
1545	TNFRSF21	GEO	*
1546	TNFSF10	GEO	*
1547	TNFSF11	GEO	*
1548	TNFSF9	GEO	*

1549	TNRC6C	GEO	*
1550	TNS1	GEO	*
1551	TNXA	GEO	*
1552	TOMM22	GEO	*
1553	TOMM34	GEO	*
1554	TOP1MT	GEO	*
1555	TOX3	GEO	*
1556	TP53I3	GEO	*
1557	TP53INP2	GEO	*
1558	TPD52L2	GEO	*
1559	TPH1	GEO	*
1560	TPSG1	GEO	*
1561	TPX2	GEO	*
1562	TRAF3IP3	GEO	*
1563	TRAPPC4	GEO	*
1564	TRIB3	GEO	*
1565	TRIM22	GEO	*
1566	TRIM23	GEO	*
1567	TRIM28	GEO	*
1568	TRIM8	GEO	*
1569	TRIP13	GEO	*
1570	TRMT11	GEO	*
1571	TRPC1	GEO	*
1572	TRPM6	GEO	*
1573	TRPS1	GEO	*
1574	TSC22D1	GEO	*
1575	TSHZ1	GEO	*
1576	TSPAN1	GEO	*
1577	TSPAN5	GEO	*
1578	TSPAN6	GEO	*
1579	TSPAN7	GEO	*
1580	TSPYL2	GEO	*
1581	TST	GEO	*
1582	TTC22	GEO	*
1583	TTC26	GEO	*
1584	TTL	GEO	*
1585	TTLL6	GEO	*
1586	TTLL7	GEO	*
1587	TUBAL3	GEO	*
1588	TUBB3	GEO	*
1589	TUBB6	GEO	*
1590	TUSC1	GEO	*
1591	TWSG1	GEO	*
1592	TXNDC5	GEO	*
1593	TXNDC9	GEO	*
1594	TXNIP	GEO	*
1595	TYMS	Both	*
1596	U2AF1	GEO	*
1597	UBASH3B	GEO	*
1598	UBE2E2	GEO	*

1599	UBE2H	GEO	*
1600	UBFD1	GEO	*
1601	UBQLN1	GEO	*
1602	UBR7	GEO	*
1603	UBXN8	GEO	*
1604	UFSP2	GEO	*
1605	UGDH	GEO	*
1606	UGGT1	GEO	*
1607	UGP2	GEO	*
1608	UGT1A1	Both	*
1609	UGT1A8	Both	*
1610	UGT2B15	Both	*
1611	UGT2B17	GEO	*
1612	UNC119B	GEO	*
1613	UNC5C	GEO	*
1614	UPP1	GEO	*
1615	USP2	GEO	*
1616	USP38	GEO	*
1617	USP5	GEO	*
1618	VARS	GEO	*
1619	VAT1L	GEO	*
1620	VCAM1	GEO	*
1621	VDAC3	GEO	*
1622	VILL	GEO	*
1623	VIP	GEO	*
1624	VIPR1	GEO	*
1625	VPS41	GEO	*
1626	VPS4A	GEO	*
1627	VSIG2	GEO	*
1628	VSNL1	GEO	*
1629	VWA2	GEO	*
1630	VWA5A	GEO	*
1631	VWF	GEO	*
1632	WASF1	GEO	*
1633	WASF2	GEO	*
1634	WBP5	GEO	*
1635	WDFY1	GEO	*
1636	WDR66	GEO	*
1637	WDR74	GEO	*
1638	WNT2	GEO	*
1639	WSB2	GEO	*
1640	WWTR1	GEO	*
1641	XBP1	GEO	*
1642	XKR4	GEO	*
1643	XPO5	GEO	*
1644	XPOT	GEO	*
1645	XRCC4	Both	*
1646	YARS	GEO	*
1647	YRDC	GEO	*
1648	ZC3H12C	GEO	*

1649	ZCCHC24	GEO	*
1650	ZDHHC2	GEO	*
1651	ZEB1	GEO	*
1652	ZEB2	GEO	*
1653	ZFP36L2	GEO	*
1654	ZFPM2	GEO	*
1655	ZFYVE26	GEO	*
1656	ZFYVE28	GEO	*
1657	ZG16	GEO	*
1658	ZMAT3	GEO	*
1659	ZMIZ1	Both	*
1660	ZNF134	GEO	*
1661	ZNF148	GEO	*
1662	ZNF217	GEO	*
1663	ZNF280C	GEO	*
1664	ZNF282	GEO	*
1665	ZNF302	GEO	*
1666	ZNF304	GEO	*
1667	ZNF311	GEO	*
1668	ZNF350	GEO	*
1669	ZNF385A	GEO	*
1670	ZNF426	GEO	*
1671	ZNF467	GEO	*
1672	ZNF473	GEO	*
1673	ZNF502	GEO	*
1674	ZNF512B	GEO	*
1675	ZNF526	GEO	*
1676	ZNF542	GEO	*
1677	ZNF575	GEO	*
1678	ZNF586	GEO	*
1679	ZNF592	GEO	*
1680	ZNF630	GEO	*
1681	ZNF702P	GEO	*
1682	ZNF74	GEO	*
1683	ZNF75A	GEO	*
1684	ZNF846	GEO	*
1685	ZNRF3	GEO	*
1686	ZRANB1	GEO	*
1687	ZW10	GEO	*
1688	ZYG11B	GEO	*
1689	ZZEF1	GEO	*
1690	A1CF	GEO	
1691	A2LD1	GEO	
1692	A2M	GEO	
1693	A4GALT	GEO	
1694	A4GNT	GEO	
1695	AAA1	GEO	
1696	AAAS	GEO	
1697	AADACL1	GEO	
1698	AAK1	GEO	

1699	AASS	GEO	
1700	ABAT	GEO	
1701	ABCA1	GEO	
1702	ABCA13	GEO	
1703	ABCB11	GEO	
1704	ABCB4	GEO	
1705	ABCB6	GEO	
1706	ABCC1	Both	
1707	ABCC13	GEO	
1708	ABCC4	Both	
1709	ABCC5	GEO	
1710	ABCC6	GEO	
1711	ABCC8	GEO	
1712	ABCD1	GEO	
1713	ABCE1	GEO	
1714	ABCF1	GEO	
1715	ABHD10	GEO	
1716	ABHD11	GEO	
1717	ABHD12	GEO	
1718	ABHD12B	GEO	
1719	ABHD15	GEO	
1720	ABHD6	GEO	
1721	ABHD7	GEO	
1722	ABHD8	GEO	
1723	ABI2	GEO	
1724	ABL1	GEO	
1725	ABLIM1	Both	
1726	ABLIM2	GEO	
1727	ABLIM3	GEO	
1728	ABO	GAD	
1729	ABTB2	GEO	
1730	ACAA1	GEO	
1731	ACAA2	GEO	
1732	ACACB	GEO	
1733	ACAD9	GEO	
1734	ACADM	GEO	
1735	ACADVL	GEO	
1736	ACAT1	GEO	
1737	ACBD3	GEO	
1738	ACBD7	GEO	
1739	ACE	GAD	
1740	ACE2	GEO	
1741	ACER2	GEO	
1742	ACHE	GEO	
1743	ACLY	GEO	
1744	ACO2	GEO	
1745	ACOT1	GEO	
1746	ACOT11	GEO	
1747	ACOT4	GEO	
1748	ACOT7	GEO	
1749	ACOT9	GEO	
1750	ACOX1	GEO	
1751	ACOX3	GEO	
1752	ACP1	GAD	
1753	ACP5	GEO	
1754	ACRBP	GEO	
1755	ACRV1	GEO	
1756	ACSF2	GEO	
1757	ACSL1	GEO	
1758	ACSL3	GEO	
1759	ACSM3	GEO	
1760	ACSS2	GEO	
1761	ACTA2	GEO	
1762	ACTB	GEO	
1763	ACTR3B	GEO	
1764	ACVR1B	GEO	
1765	ACVR2A	GAD	
1766	ACVRL1	GEO	
1767	ACY1	GEO	
1768	ADAD1	GAD	
1769	ADAM10	GEO	
1770	ADAM17	GAD	
1771	ADAM22	GEO	
1772	ADAM23	GEO	
1773	ADAM28	GEO	
1774	ADAM32	GEO	
1775	ADAMTS10	GEO	
1776	ADAMTS17	GEO	
1777	ADAMTS3	GEO	
1778	ADAMTS4	GEO	
1779	ADAMTS5	GEO	
1780	ADAMTS7	GEO	
1781	ADAMTS8	GEO	
1782	ADAMTS9	GEO	
1783	ADAMTSL1	GEO	
1784	ADAMTSL2	GEO	
1785	ADAMTSL3	GEO	
1786	ADAMTSL5	GEO	
1787	ADAP1	GEO	
1788	ADAR	GAD	
1789	ADARB1	GEO	
1790	ADCK2	GEO	
1791	ADCK4	GEO	
1792	ADCY2	GEO	
1793	ADCY5	GEO	
1794	ADCY6	GEO	
1795	ADCY9	GEO	
1796	ADCYAP1	GEO	
1797	ADD1	GEO	
1798	ADFP	GEO	

1799	ADH4	GEO	
1800	ADH5	GEO	
1801	ADH6	GEO	
1802	ADIPOQ	Both	
1803	ADIPOR1	GAD	
1804	ADM	GEO	
1805	ADORA1	GEO	
1806	ADORA2A	GEO	
1807	ADPGK	GEO	
1808	ADPRH	GEO	
1809	ADRA2C	GEO	
1810	ADRB1	GEO	
1811	ADRB2	Both	
1812	ADRB3	GAD	
1813	ADRBK1	GEO	
1814	ADRBK2	GEO	
1815	ADSV	GEO	
1816	AEBP1	GEO	
1817	AES	GEO	
1818	AFAP1-AS	GEO	
1819	AFAP1L1	GEO	
1820	AFF1	GEO	
1821	AFF2	GEO	
1822	AFF3	GEO	
1823	AFG3L1P	GEO	
1824	AGA	GEO	
1825	AGAP11	GEO	
1826	AGFG1	GEO	
1827	AGFG2	GEO	
1828	AGGF1	GEO	
1829	AGK	GEO	
1830	AGMAT	GEO	
1831	AGPAT3	GEO	
1832	AGPAT4	GEO	
1833	AGPAT5	GEO	
1834	AGPAT7	GEO	
1835	AGPHD1	GEO	
1836	AGRN	GEO	
1837	AGT	Both	
1838	AGTR1	Both	
1839	AGTR2	GEO	
1840	AGXT	Both	
1841	AGXT2	GEO	
1842	AGXT2L2	GEO	
1843	AHRR	GEO	
1844	AHSA1	GEO	
1845	AHSG	GEO	
1846	AIF1	GEO	
1847	AIFM3	GEO	
1848	AIG1	GEO	

1849	AIM1L	GEO	
1850	AIM2	GEO	
1851	AIP	GAD	
1852	AIPL1	GEO	
1853	AK3	GEO	
1854	AK4	GEO	
1855	AKAP1	GEO	
1856	AKAP10	Both	
1857	AKAP11	GEO	
1858	AKAP12	GEO	
1859	AKAP13	GEO	
1860	AKAP7	GEO	
1861	AKAP8L	GEO	
1862	AKAP9	Both	
1863	AKR1B1	GEO	
1864	AKR1C2	GEO	
1865	AKR1C4	GEO	
1866	AKR1CL2	GEO	
1867	AKR7A3	GEO	
1868	AKT1	GAD	
1869	AKT1S1	GEO	
1870	ALAS1	GEO	
1871	ALB	GEO	
1872	ALCAM	GEO	
1873	ALDH1A2	GEO	
1874	ALDH1A3	GEO	
1875	ALDH1B1	GEO	
1876	ALDH1L1	GAD	
1877	ALDH2	GAD	
1878	ALDH3A1	GEO	
1879	ALDH4A1	GEO	
1880	ALDH5A1	GEO	
1881	ALDH6A1	GEO	
1882	ALDH7A1	GEO	
1883	ALDOB	GEO	
1884	ALDOC	GEO	
1885	ALG11	GEO	
1886	ALG2	GEO	
1887	ALG5	GEO	
1888	ALOX12	GAD	
1889	ALOX5AP	GEO	
1890	ALPI	GEO	
1891	ALPK3	GEO	
1892	ALPPL2	GEO	
1893	ALS2CR4	GEO	
1894	AMBP	GEO	
1895	AMBRA1	GEO	
1896	AMD1	GAD	
1897	AMIGO3	GAD	
1898	AMMECR1	GEO	

1899	AMOTL2	GEO	
1900	AMPD1	Both	
1901	AMT	Both	
1902	AMY2B	GEO	
1903	AMZ2	GEO	
1904	ANAPC13	GAD	
1905	ANAPC4	GEO	
1906	ANG	GEO	
1907	ANGPT1	GEO	
1908	ANGPTL4	GEO	
1909	ANK1	GEO	
1910	ANKFN1	GEO	
1911	ANKHD1	GEO	
1912	ANKHD1-EIF4EI	GEO	
1913	ANKLE2	GEO	
1914	ANKMY2	GEO	
1915	ANKRD10	GEO	
1916	ANKRD11	GEO	
1917	ANKRD13A	GEO	
1918	ANKRD16	GEO	
1919	ANKRD18A	GEO	
1920	ANKRD20A2	GEO	
1921	ANKRD20B	GEO	
1922	ANKRD26	GEO	
1923	ANKRD28	GEO	
1924	ANKRD36	GEO	
1925	ANKRD36B	GEO	
1926	ANKRD37	GEO	
1927	ANKRD42	GEO	
1928	ANKRD43	GEO	
1929	ANKRD5	GEO	
1930	ANKRD53	GEO	
1931	ANKRD56	GEO	
1932	ANKRD57	GEO	
1933	ANLN	GEO	
1934	ANO10	GEO	
1935	ANO3	GEO	
1936	ANO4	GEO	
1937	ANO8	GEO	
1938	ANP32A	GEO	
1939	ANTXR2	GEO	
1940	ANXA1	GEO	
1941	ANXA10	GEO	
1942	ANXA11	GEO	
1943	ANXA13	GEO	
1944	ANXA2P1	GEO	
1945	ANXA2P2	GEO	
1946	ANXA6	GEO	
1947	AOC2	GEO	
1948	AOC3	GEO	
1949	AOX1	GAD	
1950	AP1G1	GEO	
1951	AP1S2	GEO	
1952	AP1S3	GEO	
1953	AP2B1	GEO	
1954	AP3B2	GEO	
1955	AP3D1	GEO	
1956	AP3S1	GEO	
1957	AP4B1	GEO	
1958	AP4S1	GEO	
1959	APBA3	GEO	
1960	APBB1IP	GEO	
1961	APC	Both	
1962	APCDD1	GEO	
1963	APCS	GEO	
1964	APEG1	GEO	
1965	APEH	GAD	
1966	APEX1	GAD	
1967	APG4B	GEO	
1968	APH1B	GEO	
1969	APIP	GEO	
1970	APLF	GEO	
1971	APLN	GEO	
1972	APLP2	GEO	
1973	APOA1	Both	
1974	APOA2	GEO	
1975	APOB	Both	
1976	APOB48R	GEO	
1977	APOBEC1	GEO	
1978	APOBEC3C	GEO	
1979	APOBEC3F	GEO	
1980	APOBEC3G	GEO	
1981	APOC1	GEO	
1982	APOC2	GEO	
1983	APOC3	Both	
1984	APOL1	GEO	
1985	APOL2	GEO	
1986	APOL3	GEO	
1987	APOL6	GEO	
1988	APOLD1	GEO	
1989	APOM	GEO	
1990	APOO	GEO	
1991	APPL2	GEO	
1992	APRT	GEO	
1993	AQP3	GEO	
1994	AQP9	GEO	
1995	AR	GAD	
1996	ARAF	GEO	
1997	ARAP2	GEO	
1998	ARC	GEO	

1999	AREG	GEO	
2000	ARF1	GEO	
2001	ARF3	GEO	
2002	ARF5	GEO	
2003	ARFGAP2	GEO	
2004	ARFGEF2	GEO	
2005	ARFRP1	GEO	
2006	ARGBP2	GEO	
2007	ARGLU1	GEO	
2008	ARHGAP1	GEO	
2009	ARHGAP12	GEO	
2010	ARHGAP18	GEO	
2011	ARHGAP20	GEO	
2012	ARHGAP22	GEO	
2013	ARHGAP23	GEO	
2014	ARHGAP25	GEO	
2015	ARHGAP26	GEO	
2016	ARHGAP27	GEO	
2017	ARHGAP28	GEO	
2018	ARHGAP29	GEO	
2019	ARHGAP32	GEO	
2020	ARHGAP8	GEO	
2021	ARHGEF10L	GEO	
2022	ARHGEF17	GEO	
2023	ARHGEF2	GEO	
2024	ARHGEF26	GEO	
2025	ARHGEF35	GEO	
2026	ARHGEF37	GEO	
2027	ARHGEF4	GEO	
2028	ARHGEF40	GEO	
2029	ARID1A	GEO	
2030	ARID1B	GEO	
2031	ARID3A	GEO	
2032	ARID5B	GEO	
2033	ARIH1	GEO	
2034	ARL11	GAD	
2035	ARL13B	GEO	
2036	ARL2	GEO	
2037	ARL4	GEO	
2038	ARL4A	GEO	
2039	ARL4D	GEO	
2040	ARL5B	GEO	
2041	ARL6	GEO	
2042	ARL8A	GEO	
2043	ARL8B	GEO	
2044	ARMC10	GEO	
2045	ARMC4	GEO	
2046	ARMC7	GEO	
2047	ARMCX2	GEO	
2048	ARMCX3	GEO	

2049	ARMCX4	GEO	
2050	ARMCX5	GEO	
2051	ARMS2	GAD	
2052	ARNT	GAD	
2053	ARNTL	GEO	
2054	ARPC1A	GAD	
2055	ARPC2	GAD	
2056	ARPC3	GEO	
2057	ARPC4	GEO	
2058	ARPC5L	GEO	
2059	ARPP19	GEO	
2060	ARRDC4	GEO	
2061	ARSB	GEO	
2062	ARSF	GEO	
2063	ARSG	GEO	
2064	ARSK	GEO	
2065	ART3	GEO	
2066	ARV1	GEO	
2067	ARVCF	GEO	
2068	ASAH1	GEO	
2069	ASAM	GEO	
2070	ASAP3	GEO	
2071	ASB4	GEO	
2072	ASCC3	Both	
2073	ASCL5	GEO	
2074	ASF1A	GEO	
2075	ASGR1	GEO	
2076	ASGR2	GEO	
2077	ASH1L	GEO	
2078	ASL	GEO	
2079	ASMT	GEO	
2080	ASMTL	GEO	
2081	ASPHD2	GEO	
2082	ASPM	GEO	
2083	ASTN2	GEO	
2084	ASXL1	GEO	
2085	ASXL3	GEO	
2086	ATAD2	GEO	
2087	ATAD3A	GEO	
2088	ATAD5	GEO	
2089	ATF5	GEO	
2090	ATG10	GEO	
2091	ATG16L1	GAD	
2092	ATG2B	GAD	
2093	ATG4A	GEO	
2094	ATG4B	GEO	
2095	ATG5	GAD	
2096	ATG7	GEO	
2097	ATG9B	GAD	
2098	ATIC	Both	

2099	ATM	Both	
2100	ATMIN	GEO	
2101	ATN1	GEO	
2102	ATOH1	GEO	
2103	ATOH8	GEO	
2104	ATP10A	GEO	
2105	ATP10B	GEO	
2106	ATP11A	GEO	
2107	ATP11B	GEO	
2108	ATP13A2	GEO	
2109	ATP13A3	GEO	
2110	ATP13A4	GEO	
2111	ATP1A1	GEO	
2112	ATP1A2	GEO	
2113	ATP1B2	GEO	
2114	ATP2A1	GEO	
2115	ATP2A2	Both	
2116	ATP2A3	Both	
2117	ATP2C1	GEO	
2118	ATP5C1	GEO	
2119	ATP5F1	GEO	
2120	ATP5G2	GEO	
2121	ATP5J	GEO	
2122	ATP5O	GEO	
2123	ATP6	GAD	
2124	ATP6AP1L	GEO	
2125	ATP6V0A2	GEO	
2126	ATP6V0A4	GEO	
2127	ATP6V0B	GEO	
2128	ATP6V0D1	GEO	
2129	ATP6V0E1	GEO	
2130	ATP6V1B1	GEO	
2131	ATP6V1C1	GEO	
2132	ATP6V1C2	GEO	
2133	ATP6V1E1	GEO	
2134	ATP6V1E2	GEO	
2135	ATP6V1F	GEO	
2136	ATP6V1G2	GEO	
2137	ATP7B	GEO	
2138	ATP8B1	GEO	
2139	ATPBD4	GEO	
2140	ATPIF1	GEO	
2141	ATR	Both	
2142	ATRIP	GEO	
2143	ATRX	GEO	
2144	ATXN1	GEO	
2145	ATXN2L	GAD	
2146	AUH	GEO	
2147	AUTS2	GEO	
2148	AVEN	GEO	

2149	AVIL	GEO	
2150	AVL9	GEO	
2151	AXIN1	GAD	
2152	AZGP1	GEO	
2153	AZIN1	GEO	
2154	B2M	GEO	
2155	B3GALT5	GEO	
2156	B3GNT3	GEO	
2157	B3GNT4	GEO	
2158	B3GNT6	GEO	
2159	B3GNTL1	GEO	
2160	B4GALNT1	GEO	
2161	B4GALNT2	GEO	
2162	B4GALNT4	GEO	
2163	B4GALT1	GEO	
2164	B4GALT2	GEO	
2165	B4GALT5	GEO	
2166	B4GALT6	GEO	
2167	BACH1	GEO	
2168	BACH2	GAD	
2169	BAD	GEO	
2170	BAG3	GEO	
2171	BAG5	GEO	
2172	BAHCC1	GEO	
2173	BAIAP2	GEO	
2174	BAIAP2L2	GEO	
2175	BAMBI	GAD	
2176	BAP1	GEO	
2177	BAPX1	GEO	
2178	BARD1	GAD	
2179	BARX1	GEO	
2180	BASP1	GEO	
2181	BAT2	GAD	
2182	BAT2L2	GEO	
2183	BAT3	GEO	
2184	BAX	GAD	
2185	BAZ2B	GEO	
2186	BBC3	GEO	
2187	BBS10	GEO	
2188	BBS4	GEO	
2189	BBS5	GEO	
2190	BBS9	GEO	
2191	BCAN	GEO	
2192	BCAP29	GEO	
2193	BCAP31	GEO	
2194	BCAS3	GEO	
2195	BCAS4	GEO	
2196	BCAT1	GEO	
2197	BCHE	GEO	
2198	BCKDHB	GEO	

2199	BCL10	GEO	
2200	BCL11A	GEO	
2201	BCL2	GEO	
2202	BCL2A1	GEO	
2203	BCL2L1	Both	
2204	BCL2L12	GEO	
2205	BCL2L14	GEO	
2206	BCL2L15	GEO	
2207	BCL3	GAD	
2208	BCL6	GEO	
2209	BCL6B	GEO	
2210	BCL8	GEO	
2211	BCL9	GEO	
2212	BCLAF1	GEO	
2213	BCLP	GEO	
2214	BCORL1	GEO	
2215	BCR	GEO	
2216	BCYRN1	GEO	
2217	BDKRB2	GEO	
2218	BDNF	GEO	
2219	BEND6	GEO	
2220	BEND7	GEO	
2221	BEST2	GEO	
2222	BEX1	GEO	
2223	BEX2	GEO	
2224	BF	GEO	
2225	BFAR	GEO	
2226	BHLHB3	GEO	
2227	BHLHE22	GEO	
2228	BHLHE40	GEO	
2229	BHMT	GAD	
2230	BID	Both	
2231	BIK	GEO	
2232	BIRC2	GEO	
2233	BIRC3	GEO	
2234	BIRC5	GAD	
2235	BIRC6	GEO	
2236	BLCAP	GEO	
2237	BLK	GEO	
2238	BLM	GAD	
2239	BLMH	GEO	
2240	BLNK	GEO	
2241	BLOC1S2	GEO	
2242	BLVRA	GEO	
2243	BM039	GEO	
2244	BMP1	GAD	
2245	BMP2K	GEO	
2246	BMP4	Both	
2247	BMP5	GEO	
2248	BMP8A	GEO	
2249	BMP8B	GEO	
2250	BMPR1B	GEO	
2251	BMPR2	GAD	
2252	BMS1	GEO	
2253	BMS1P1	GEO	
2254	BMX	GEO	
2255	BNIP2	GEO	
2256	BNIP3	GEO	
2257	BNIP3L	GEO	
2258	BPGM	GEO	
2259	BPI	Both	
2260	BRAF	GAD	
2261	BRCA1	Both	
2262	BRCA2	Both	
2263	BRD1	GEO	
2264	BRD2	GEO	
2265	BRD3	GEO	
2266	BRD4	GEO	
2267	BRD8	GEO	
2268	BRF2	GEO	
2269	BRI3BP	GEO	
2270	BRSK2	GEO	
2271	BRUNOL4	GAD	
2272	BRWD1	GAD	
2273	BRWD3	GEO	
2274	BSG	GEO	
2275	BSN	GAD	
2276	BTBD1	GEO	
2277	BTBD3	GEO	
2278	BTBD7	GEO	
2279	BTG1	GEO	
2280	BTK	GEO	
2281	BTN2A2	GEO	
2282	BTN2A3	GEO	
2283	BTN3A3	GEO	
2284	BTNL2	Both	
2285	BTNL8	GEO	
2286	BTRC	Both	
2287	BUB1	GEO	
2288	BUD31	GEO	
2289	BVES	GEO	
2290	BYSL	GEO	
2291	BZW1	GEO	
2292	C10orf11	GEO	
2293	C10orf112	GEO	
2294	C10orf118	GEO	
2295	C10orf137	GEO	
2296	C10orf140	GEO	
2297	C10ORF26	GEO	
2298	C10orf32	GEO	

2299	C10orf4	GEO	
2300	C10orf47	GEO	
2301	C10orf54	GEO	
2302	C10orf58	GEO	
2303	C10orf67	GAD	
2304	C10orf71	GEO	
2305	C10orf72	GEO	
2306	C11orf20	GEO	
2307	C11orf30	Both	
2308	C11orf33	GEO	
2309	C11orf41	GEO	
2310	C11orf45	GEO	
2311	C11orf53	Both	
2312	C11orf54	GEO	
2313	C11orf58	GEO	
2314	C11orf63	GEO	
2315	C11orf65	GEO	
2316	C11orf71	GEO	
2317	C11orf80	GEO	
2318	C11orf82	GEO	
2319	C11orf86	GEO	
2320	C11orf9	GEO	
2321	C11orf93	GEO	
2322	C12orf11	GEO	
2323	C12orf27	GEO	
2324	C12orf29	GEO	
2325	C12ORF30	GAD	
2326	C12orf32	GEO	
2327	C12orf35	GEO	
2328	C12orf44	GEO	
2329	C12orf48	GEO	
2330	C12orf57	GEO	
2331	C12orf59	GEO	
2332	C13orf15	GEO	
2333	C13orf18	GEO	
2334	C13orf23	GEO	
2335	C13orf30	GEO	
2336	C13ORF31	GAD	
2337	C14orf100	GEO	
2338	C14orf106	GEO	
2339	C14orf115	GEO	
2340	C14orf118	GEO	
2341	C14orf126	GEO	
2342	C14orf132	GEO	
2343	C14orf138	GEO	
2344	C14orf145	GEO	
2345	C14ORF147	GEO	
2346	C14orf149	GEO	
2347	C14orf159	GEO	
2348	C14orf176	GEO	

2349	C14orf28	GEO	
2350	C14orf4	GEO	
2351	C14orf49	GEO	
2352	C14orf50	GEO	
2353	C14orf58	GEO	
2354	C15orf24	GEO	
2355	C15orf29	GEO	
2356	C15orf42	GEO	
2357	C15orf57	GEO	
2358	C15orf58	GEO	
2359	C15orf61	GEO	
2360	C16orf13	GEO	
2361	C16orf46	GEO	
2362	C16orf53	GEO	
2363	C16orf75	Both	
2364	C16orf81	GEO	
2365	C16orf89	GEO	
2366	C17orf27	GEO	
2367	C17orf39	GEO	
2368	C17orf45	GEO	
2369	C17orf67	GEO	
2370	C17orf69	GEO	
2371	C17orf70	GEO	
2372	C17orf73	GEO	
2373	C17orf76	GEO	
2374	C17orf79	GEO	
2375	C17orf81	GEO	
2376	C17orf85	GEO	
2377	C18orf17	GEO	
2378	C18orf24	GEO	
2379	C18orf54	GEO	
2380	C19orf10	GAD	
2381	C19orf2	GEO	
2382	C19orf23	GEO	
2383	C19orf33	GEO	
2384	C19orf40	GEO	
2385	C19orf42	GEO	
2386	C19orf43	GEO	
2387	C19orf45	GEO	
2388	C19orf48	GEO	
2389	C19orf51	GEO	
2390	C19orf59	GEO	
2391	C19orf6	GEO	
2392	C19orf66	GEO	
2393	C19orf70	GEO	
2394	C19orf77	GEO	
2395	C1orf106	GEO	
2396	C1orf107	GEO	
2397	C1ORF108	GEO	
2398	C1orf116	GEO	

2399	C1orf125	GEO	
2400	C1orf133	GEO	
2401	C1orf161	GEO	
2402	C1orf173	GEO	
2403	C1orf174	GEO	
2404	C1orf175	GEO	
2405	C1orf178	GEO	
2406	C1orf186	GEO	
2407	C1orf21	GEO	
2408	C1orf226	GEO	
2409	C1orf52	GEO	
2410	C1orf56	GEO	
2411	C1orf57	GEO	
2412	C1orf66	GEO	
2413	C1orf67	GEO	
2414	C1orf75	GEO	
2415	C1orf77	GEO	
2416	C1orf81	GAD	
2417	C1orf97	GEO	
2418	C1QA	GEO	
2419	C1QB	GEO	
2420	C1QBP	GEO	
2421	C1QC	GEO	
2422	C1QTNF1	GEO	
2423	C1R	GEO	
2424	C1RL	GEO	
2425	C1S	GEO	
2426	C20orf103	GEO	
2427	C20orf11	GEO	
2428	C20orf117	GEO	
2429	C20orf118	GEO	
2430	C20orf132	GEO	
2431	C20orf151	GEO	
2432	C20orf152	GEO	
2433	C20orf194	GEO	
2434	C20ORF198	GEO	
2435	C20orf39	GEO	
2436	C20orf4	GEO	
2437	C20orf42	GEO	
2438	C20orf43	GEO	
2439	C20orf74	GEO	
2440	C20orf94	GEO	
2441	C20orf96	GEO	
2442	C20orf98	GEO	
2443	C21orf129	GEO	
2444	C21orf2	GEO	
2445	C21orf49	GEO	
2446	C21orf56	GEO	
2447	C21orf57	GEO	
2448	C21orf58	GEO	

2449	C21orf63	GEO	
2450	C21orf66	GEO	
2451	C21orf7	GEO	
2452	C21orf70	GEO	
2453	C21orf71	GEO	
2454	C21orf88	GEO	
2455	C22orf16	GEO	
2456	C22orf34	GEO	
2457	C22orf45	GEO	
2458	C2CD4A	GEO	
2459	C2orf14	GEO	
2460	C2orf27	GEO	
2461	C2orf28	GEO	
2462	C2orf3	GEO	
2463	C2orf40	GEO	
2464	C2orf55	GEO	
2465	C2orf58	GEO	
2466	C2orf61	GEO	
2467	C2orf65	GAD	
2468	C2orf67	GEO	
2469	C2orf68	GEO	
2470	C2orf69	GEO	
2471	C2orf7	GEO	
2472	C2orf70	GEO	
2473	C2orf72	GEO	
2474	C2orf76	GEO	
2475	C2orf89	GEO	
2476	C3	Both	
2477	C3orf10	GEO	
2478	C3orf16	GEO	
2479	C3orf23	GEO	
2480	C3orf31	GEO	
2481	C3orf32	GEO	
2482	C3orf34	GEO	
2483	C3orf35	GEO	
2484	C3orf38	GEO	
2485	C3orf65	GEO	
2486	C3orf67	GEO	
2487	C3orf70	GEO	
2488	C4A	GEO	
2489	C4BPA	GEO	
2490	C4BPB	GEO	
2491	C4orf14	GEO	
2492	C4ORF16	GEO	
2493	C4orf27	GEO	
2494	C4orf30	GEO	
2495	C4orf39	GEO	
2496	C4orf48	GEO	
2497	C4orf49	GEO	
2498	C4orf52	GEO	

2499	C5	GEO	
2500	C5AR1	GEO	
2501	C5orf13	GEO	
2502	C5orf17	GEO	
2503	C5orf20	GEO	
2504	C5orf22	GEO	
2505	C5orf27	GEO	
2506	C5orf30	GEO	
2507	C5orf35	GEO	
2508	C5orf4	GEO	
2509	C5orf46	GEO	
2510	C5ORF5	GEO	
2511	C5orf56	GAD	
2512	C6	GEO	
2513	C6ORF10	GAD	
2514	C6ORF106	GAD	
2515	C6orf108	GEO	
2516	C6orf111	GEO	
2517	C6orf118	GEO	
2518	C6orf132	GEO	
2519	C6orf136	GEO	
2520	C6orf145	GEO	
2521	C6orf15	GEO	
2522	C6orf168	GEO	
2523	C6orf170	GEO	
2524	C6ORF173	GAD	
2525	C6orf174	GEO	
2526	C6orf192	GEO	
2527	C6orf26	GEO	
2528	C6orf29	GEO	
2529	C6orf57	GEO	
2530	C6orf64	GEO	
2531	C7orf23	GEO	
2532	C7orf30	GEO	
2533	C7orf31	GEO	
2534	C7orf36	GEO	
2535	C7orf38	GEO	
2536	C7orf46	GEO	
2537	C7ORF47	GEO	
2538	C7orf54	GEO	
2539	C8A	GEO	
2540	C8ORF30A	GEO	
2541	C8orf33	GEO	
2542	C8orf4	GEO	
2543	C8orf46	GEO	
2544	C8orf60	GEO	
2545	C8orf74	GEO	
2546	C8orf83	GEO	
2547	C8orf85	GAD	
2548	C9orf127	GEO	

2549	C9orf140	GEO	
2550	C9orf152	GEO	
2551	C9ORF19	GEO	
2552	C9orf23	GEO	
2553	C9orf24	GEO	
2554	C9orf3	GEO	
2555	C9orf30	GEO	
2556	C9orf40	GEO	
2557	C9orf45	GEO	
2558	C9orf61	GEO	
2559	C9orf72	GEO	
2560	C9orf86	GEO	
2561	C9orf91	GEO	
2562	CA10	GEO	
2563	CA11	GEO	
2564	CA13	GEO	
2565	CA6	Both	
2566	CAB39L	GEO	
2567	CABLES1	GEO	
2568	CABP1	GEO	
2569	CABP7	GEO	
2570	CACNA1A	GEO	
2571	CACNA1H	GEO	
2572	CACNA2D2	GEO	
2573	CACNA2D4	GEO	
2574	CACNB2	GEO	
2575	CACNB4	GEO	
2576	CACNG1	GEO	
2577	CADM1	Both	
2578	CADM2	GAD	
2579	CADM3	GEO	
2580	CADM4	GEO	
2581	CADPS2	GEO	
2582	CALB1	GEO	
2583	CALB2	GAD	
2584	CALCOCO2	GEO	
2585	CALCRL	GEO	
2586	CALHM2	GEO	
2587	CALM1	GEO	
2588	CALML4	GEO	
2589	CALU	GEO	
2590	CAMK1	GEO	
2591	CAMK2D	GEO	
2592	CAMK2N1	GEO	
2593	CAMKK2	GEO	
2594	CAMSAP1L1	GEO	
2595	CAP2	GEO	
2596	CAPG	GEO	
2597	CAPN10	Both	
2598	CAPN13	GEO	

2599	CAPN2	GEO	
2600	CAPN3	GEO	
2601	CAPN6	GEO	
2602	CAPN8	GEO	
2603	CAPRIN2	GEO	
2604	CAPZA2	GEO	
2605	CAPZB	GEO	
2606	CARD11	GEO	
2607	CARD14	GEO	
2608	CARD15	GAD	
2609	CARD16	GEO	
2610	CARD4	GAD	
2611	CARD8	GAD	
2612	CARD9	GAD	
2613	CARKD	GEO	
2614	CARM1	GEO	
2615	CARS	GEO	
2616	CARS2	GEO	
2617	CAS1	GEO	
2618	CASC4	GEO	
2619	CASC5	GEO	
2620	CASD1	GEO	
2621	CASK	GEO	
2622	CASP1	GEO	
2623	CASP10	GAD	
2624	CASP14	GAD	
2625	CASP3	GAD	
2626	CASP5	GEO	
2627	CASP7	GAD	
2628	CASP8	GAD	
2629	CASP8AP2	Both	
2630	CASP9	GAD	
2631	CASQ2	GEO	
2632	CASR	GAD	
2633	CASS4	GEO	
2634	CASZ1	GEO	
2635	CAT	GAD	
2636	CATSPER2	GEO	
2637	CAV1	Both	
2638	CAV2	GEO	
2639	CBFA2T2	GEO	
2640	CBFA2T3	GEO	
2641	CBLB	GEO	
2642	CBLC	GEO	
2643	CBLN1	GEO	
2644	CBLN2	GEO	
2645	CBX4	GEO	
2646	CBX5	GEO	
2647	CBX7	GEO	
2648	CBX8	GEO	

2649	CBY1	GEO	
2650	CC50B	GEO	
2651	CCBL1	GEO	
2652	CCBP2	GEO	
2653	CCDC101	GAD	
2654	CCDC102B	GEO	
2655	CCDC104	GEO	
2656	CCDC108	GEO	
2657	CCDC109A	GEO	
2658	CCDC112	GEO	
2659	CCDC113	GEO	
2660	CCDC12	GEO	
2661	CCDC123	GEO	
2662	CCDC127	GEO	
2663	CCDC130	GEO	
2664	CCDC132	GEO	
2665	CCDC136	GEO	
2666	CCDC139	GAD	
2667	CCDC14	GEO	
2668	CCDC144A	GEO	
2669	CCDC144B	GEO	
2670	CCDC146	GEO	
2671	CCDC149	GEO	
2672	CCDC153	GEO	
2673	CCDC16	GEO	
2674	CCDC18	GEO	
2675	CCDC19	GEO	
2676	CCDC24	GEO	
2677	CCDC28A	GEO	
2678	CCDC28B	GEO	
2679	CCDC29	GEO	
2680	CCDC45	GEO	
2681	CCDC46	GEO	
2682	CCDC51	GEO	
2683	CCDC52	GEO	
2684	CCDC59	GEO	
2685	CCDC64	GEO	
2686	CCDC66	GEO	
2687	CCDC71	GEO	
2688	CCDC74B	GEO	
2689	CCDC76	GEO	
2690	CCDC8	GEO	
2691	CCDC82	GEO	
2692	CCDC84	GEO	
2693	CCDC85B	GEO	
2694	CCDC86	GEO	
2695	CCDC88A	GEO	
2696	CCDC88B	GEO	
2697	CCDC92	GEO	
2698	CCDC93	GEO	

2699	CCL1	GAD	
2700	CCL11	Both	
2701	CCL13	GEO	
2702	CCL14-CCL15	GEO	
2703	CCL18	GEO	
2704	CCL19	GEO	
2705	CCL2	Both	
2706	CCL22	GEO	
2707	CCL24	Both	
2708	CCL26	GAD	
2709	CCL28	GEO	
2710	CCL3	GEO	
2711	CCL4	GEO	
2712	CCL4L1	GEO	
2713	CCNA2	GEO	
2714	CCNB1IP1	GEO	
2715	CCNB2	GEO	
2716	CCNB3	GEO	
2717	CCND1	Both	
2718	CCND2	GEO	
2719	CCND3	GEO	
2720	CCNDBP1	GEO	
2721	CCNE2	GEO	
2722	CCNH	GAD	
2723	CCNI	GEO	
2724	CCNJL	GEO	
2725	CCNL1	GEO	
2726	CCNL2	GEO	
2727	CCNT1	GEO	
2728	CCNU	GEO	
2729	CCNY	Both	
2730	CCNYL1	GEO	
2731	CCPG1	GEO	
2732	CCR1	GEO	
2733	CCR2	Both	
2734	CCR5	GAD	
2735	CCR6	Both	
2736	CCR7	GEO	
2737	CCRL2	GEO	
2738	CCT2	GEO	
2739	CCT6A	GEO	
2740	CD163L1	GEO	
2741	CD164L2	GEO	
2742	CD19	GAD	
2743	CD1A	GEO	
2744	CD1B	GEO	
2745	CD1D	GEO	
2746	CD1E	GEO	
2747	CD209	Both	
2748	CD22	GEO	
2749	CD226	GEO	
2750	CD27	GEO	
2751	CD28	GAD	
2752	CD300A	GEO	
2753	CD302	GEO	
2754	CD33	GEO	
2755	CD34	GEO	
2756	CD3D	GEO	
2757	CD3E	GEO	
2758	CD3EAP	Both	
2759	CD40	GEO	
2760	CD46	GEO	
2761	CD47	GEO	
2762	CD53	GEO	
2763	CD55	GEO	
2764	CD58	GEO	
2765	CD5L	GEO	
2766	CD6	GEO	
2767	CD68	GEO	
2768	CD74	GEO	
2769	CD84	GEO	
2770	CD86	Both	
2771	CD8A	GEO	
2772	CD9	GEO	
2773	CD93	GEO	
2774	CD99	GEO	
2775	CD99L2	GEO	
2776	CDA	Both	
2777	CDC123	GEO	
2778	CDC14A	Both	
2779	CDC14B	GEO	
2780	CDC16	GEO	
2781	CDC2	GEO	
2782	CDC2L1	GEO	
2783	CDC34	GEO	
2784	CDC37L1	GEO	
2785	CDC40	GAD	
2786	CDC42	GEO	
2787	CDC42SE1	GEO	
2788	CDC42SE2	GEO	
2789	CDC45	GEO	
2790	CDC45L	GAD	
2791	CDCA2	GEO	
2792	CDCA3	GEO	
2793	CDCA5	GEO	
2794	CDCA7	GEO	
2795	CDCA8	GEO	
2796	CDGAP	GEO	
2797	CDH11	GEO	
2798	CDH13	GEO	

2799	CDH17	GEO	
2800	CDH19	GEO	
2801	CDH23	GEO	
2802	CDH24	GEO	
2803	CDH26	GEO	
2804	CDH29	GAD	
2805	CDH4	GEO	
2806	CDH5	GEO	
2807	CDH8	GEO	
2808	CDHR1	GEO	
2809	CDHR5	GEO	
2810	CDK1	GEO	
2811	CDK11A	GEO	
2812	CDK13	GEO	
2813	CDK14	GEO	
2814	CDK15	GEO	
2815	CDK3	GEO	
2816	CDK5RAP2	GEO	
2817	CDK6	Both	
2818	CDK7	Both	
2819	CDK8	GEO	
2820	CDK9	GEO	
2821	CDKAL1	GAD	
2822	CDKL1	GEO	
2823	CDKL2	GEO	
2824	CDKL3	GEO	
2825	CDKN1A	GAD	
2826	CDKN1B	GAD	
2827	CDKN2AIPNL	GEO	
2828	CDKN2B-AS	GEO	
2829	CDKN2C	GEO	
2830	CDRT4	GEO	
2831	CDS1	GEO	
2832	CDS2	GEO	
2833	CDT1	GEO	
2834	CDV3	GEO	
2835	CDX1	Both	
2836	CDX2	Both	
2837	CEACAM21	GEO	
2838	CEACAM3	GEO	
2839	CEACAM4	GEO	
2840	CEBPD	GEO	
2841	CECR2	GEO	
2842	CECR5	GEO	
2843	CEL	GEO	
2844	CELA3A	GEO	
2845	CELA3B	GEO	
2846	CELF2	GEO	
2847	CELF4	GEO	
2848	CELP	GEO	

2849	CELSR1	GEO	
2850	CELSR2	GEO	
2851	CELSR3	GEO	
2852	CENPA	GEO	
2853	CENPN	GEO	
2854	CENPP	GEO	
2855	CENPV	GEO	
2856	CEP110	GEO	
2857	CEP170	GEO	
2858	CEP250	GEO	
2859	CEP55	GEO	
2860	CEP57	GEO	
2861	CEP63	GAD	
2862	CEP68	GEO	
2863	CEP70	GEO	
2864	CEP72	Both	
2865	CEPT1	GEO	
2866	CERKL	GEO	
2867	CES3	GEO	
2868	CES4A	GEO	
2869	CETN2	GEO	
2870	CETP	Both	
2871	CFC1	GEO	
2872	CFDP1	GEO	
2873	CFHL1	GEO	
2874	CFHR1	GEO	
2875	CFI	GEO	
2876	CFLAR	Both	
2877	CFP	GEO	
2878	CGB1	GEO	
2879	CGGBP1	GEO	
2880	CGI-38	GEO	
2881	CGN	GEO	
2882	CGNL1	GEO	
2883	CGREF1	GEO	
2884	CH25H	GEO	
2885	CHAC1	GEO	
2886	CHAF1A	GEO	
2887	CHCHD5	GEO	
2888	CHCHD6	GEO	
2889	CHCHD7	GEO	
2890	CHD2	GEO	
2891	CHD3	GEO	
2892	CHD7	GEO	
2893	CHD9	GEO	
2894	CHEK1	Both	
2895	CHEK2	GAD	
2896	CHERP	GEO	
2897	CHFR	GEO	
2898	CHGB	GEO	

2899	CHKA	GEO	
2900	CHMP1B	GEO	
2901	CHMP2B	GEO	
2902	CHMP4A	GEO	
2903	CHN1	GEO	
2904	CHN2	GEO	
2905	CHODL	GEO	
2906	CHORDC1	GEO	
2907	CHP	GEO	
2908	CHPF	GEO	
2909	CHPT1	GEO	
2910	CHRDL1	GEO	
2911	CHRDL2	GEO	
2912	CHRFAM7A	GEO	
2913	CHRM2	GEO	
2914	CHRNA1	GEO	
2915	CHRNA5	GEO	
2916	CHRND	GEO	
2917	CHRNE	GEO	
2918	CHST15	GEO	
2919	CHST3	GEO	
2920	CHST5	GEO	
2921	CHST7	GEO	
2922	CHUK	GEO	
2923	CHURC1	GEO	
2924	CI152	GEO	
2925	CIAPIN1	GEO	
2926	CIB1	GEO	
2927	CIB2	GEO	
2928	CIDEC	GEO	
2929	CIITA	Both	
2930	CILP	GEO	
2931	CILP2	GEO	
2932	CISD1	GEO	
2933	CIT	GEO	
2934	CITED2	GEO	
2935	CKAP5	GEO	
2936	CKIP-1	GEO	
2937	CKLF	GEO	
2938	CKM	GEO	
2939	CKMT1	GEO	
2940	CKMT1A	GEO	
2941	CKMT1B	GEO	
2942	CKMT2	GEO	
2943	CKS2	GEO	
2944	CLASP2	GEO	
2945	CLASRP	GEO	
2946	CLC	GEO	
2947	CLCC1	GEO	
2948	CLCN4	GEO	

2949	CLCN5	GEO	
2950	CLCN7	GEO	
2951	CLDN10	GEO	
2952	CLDN15	GEO	
2953	CLDN18	GEO	
2954	CLDN4	GEO	
2955	CLDN7	GEO	
2956	CLDN8	GEO	
2957	CLDND1	GEO	
2958	CLEC10A	GEO	
2959	CLEC11A	GEO	
2960	CLEC16A	GAD	
2961	CLEC1B	GAD	
2962	CLEC2D	GEO	
2963	CLEC4D	GEO	
2964	CLEC4G	GEO	
2965	CLEC5A	GEO	
2966	CLEC7A	Both	
2967	CLECL1	GEO	
2968	CLGN	GEO	
2969	CLIC2	GEO	
2970	CLIP3	GEO	
2971	CLIP4	GEO	
2972	CLK1	GEO	
2973	CLN3	Both	
2974	CLN5	GEO	
2975	CLNK	GEO	
2976	CLPTM1L	GAD	
2977	CLSTN2	GAD	
2978	CLTA	GEO	
2979	CLTB	GEO	
2980	CLTCL1	GEO	
2981	CLUAP1	GEO	
2982	CLYBL	GEO	
2983	CMAS	GEO	
2984	CMBL	GEO	
2985	CMC1	GEO	
2986	CMKLR1	GEO	
2987	CMTM3	GEO	
2988	CMTM7	GEO	
2989	CMTM8	GEO	
2990	CNDP2	GEO	
2991	CNFN	GEO	
2992	CNGA3	GEO	
2993	CNGB1	GEO	
2994	CNIH4	GEO	
2995	CNKSR1	GEO	
2996	CNKSR3	GEO	
2997	CNN1	GEO	
2998	CNNM3	GEO	

2999	CNNM4	GEO	
3000	CNOT2	GEO	
3001	CNOT7	GEO	
3002	CNOT8	GEO	
3003	CNPY2	GEO	
3004	CNPY4	GEO	
3005	CNR1	Both	
3006	CNTFR	GEO	
3007	CNTN4	GEO	
3008	CNTNAP1	GEO	
3009	CNTNAP2	GEO	
3010	CNTROB	GEO	
3011	COBL	GEO	
3012	COBLL1	GEO	
3013	COCH	GEO	
3014	COL11A2	GEO	
3015	COL12A1	GEO	
3016	COL14A1	GEO	
3017	COL15A1	GEO	
3018	COL16A1	GEO	
3019	COL18A1	GEO	
3020	COL24A1	GEO	
3021	COL2A1	GEO	
3022	COL4A1	GEO	
3023	COL4A2	GEO	
3024	COL4A4	GEO	
3025	COL4A5	GEO	
3026	COL4A6	GEO	
3027	COL5A1	GEO	
3028	COL5A2	GEO	
3029	COL5A3	GEO	
3030	COL6A3	GEO	
3031	COL7A1	GEO	
3032	COL8A1	GEO	
3033	COL9A2	GEO	
3034	COLQ	GEO	
3035	COMMD1	GEO	
3036	COMMD3	GEO	
3037	COMMD6	GEO	
3038	COMP	GEO	
3039	COPA	GEO	
3040	COPG	GEO	
3041	COPS3	GEO	
3042	COPS4	GEO	
3043	COPS6	GEO	
3044	COPZ1	GEO	
3045	COPZ2	GEO	
3046	CORO1A	GEO	
3047	CORO1C	GEO	
3048	CORO2B	GEO	

3049	COX1	GAD	
3050	COX11	GAD	
3051	COX15	GEO	
3052	COX2	GAD	
3053	COX3	GAD	
3054	COX4I1	GEO	
3055	COX6C	GEO	
3056	COX7A1	GEO	
3057	COX8A	GEO	
3058	CP110	GEO	
3059	CPA4	Both	
3060	CPA6	GEO	
3061	CPB1	GEO	
3062	CPB2	GEO	
3063	CPD	GEO	
3064	CPE	GEO	
3065	CPEB1	GEO	
3066	CPEB2	GEO	
3067	CPEB3	GEO	
3068	CPEB4	Both	
3069	CPNE3	GEO	
3070	CPNE7	GEO	
3071	CPPED1	GEO	
3072	CPS1	GEO	
3073	CPS1-IT	GEO	
3074	CPSF2	GEO	
3075	CPT1A	GEO	
3076	CPT1B	GEO	
3077	CR1	GEO	
3078	CR2	GEO	
3079	CRABP2	GEO	
3080	CRAC1	GAD	
3081	CRB2	GEO	
3082	CREBZF	GEO	
3083	CRELD2	GEO	
3084	CRHBP	GEO	
3085	CRIM1	GEO	
3086	CRIP2	GEO	
3087	CRIPAK	GEO	
3088	CRISPLD1	GEO	
3089	CRK	GEO	
3090	CRLF1	GEO	
3091	CRNKL1	GEO	
3092	CROP	GEO	
3093	CROT	GEO	
3094	CRTAM	GEO	
3095	CRTAP	GEO	
3096	CRTC3	GEO	
3097	CRY2	GEO	
3098	CRYAB	GEO	

3099	CRYBA2	GEO	
3100	CRYBG3	GEO	
3101	CRYGS	GEO	
3102	CRYZ	GEO	
3103	CRYZL1	GEO	
3104	CSAG2	GEO	
3105	CSDE1	GEO	
3106	CSF3	GEO	
3107	CSF3R	GEO	
3108	CSHL1	GEO	
3109	CSK	GEO	
3110	CSMD1	GEO	
3111	CSNK1A1	GEO	
3112	CSNK1G1	GEO	
3113	CSNK2A1	Both	
3114	CSNK2B	GEO	
3115	CSPG5	GEO	
3116	CSPP1	GEO	
3117	CSRP1	GEO	
3118	CSRP2	GEO	
3119	CST1	GEO	
3120	CST2	GEO	
3121	CST4	GEO	
3122	CST7	GEO	
3123	CSTA	GEO	
3124	CSTF1	GEO	
3125	CTAG1A	GEO	
3126	CTBP1	GEO	
3127	CTDSPL2	GEO	
3128	CTGF	GEO	
3129	CTH	GEO	
3130	CTHRC1	GEO	
3131	CTLA4	Both	
3132	CTNNA2	GEO	
3133	CTNNAL1	GEO	
3134	CTNNB1	Both	
3135	CTNNBL1	GEO	
3136	CTNND1	GEO	
3137	CTNND2	GEO	
3138	CTPS	GEO	
3139	CTPS2	GEO	
3140	CTSA	GEO	
3141	CTSB	Both	
3142	CTSD	GEO	
3143	CTSE	GEO	
3144	CTSG	GEO	
3145	CTSH	GEO	
3146	CTSO	GEO	
3147	CTSS	GEO	
3148	CTSW	GEO	

3149	CTTN	GEO	
3150	CTTNBP2	GEO	
3151	CTTNBP2NL	GEO	
3152	CUBN	GAD	
3153	CUEDC2	GEO	
3154	CUL2	GAD	
3155	CUL4A	GEO	
3156	CUTC	GEO	
3157	CUX1	GEO	
3158	CWF19L2	GEO	
3159	CX3CL1	Both	
3160	CX3CR1	GAD	
3161	CXCL10	GEO	
3162	CXCL11	Both	
3163	CXCL13	GEO	
3164	CXCL14	GEO	
3165	CXCL2	GEO	
3166	CXCL5	Both	
3167	CXCL6	GEO	
3168	CXCL9	Both	
3169	CXCR3	GEO	
3170	CXCR4	Both	
3171	CXorf18	GEO	
3172	CXorf21	GEO	
3173	CXorf30	GEO	
3174	CXorf40A	GEO	
3175	CXorf40B	GEO	
3176	CXorf57	GEO	
3177	CXorf9	GEO	
3178	CXXC5	GEO	
3179	CXYorf3	GEO	
3180	CYB5A	GEO	
3181	CYB5B	GEO	
3182	CYB5D1	GEO	
3183	CYB5R1	GEO	
3184	CYB5R2	GEO	
3185	CYBA	Both	
3186	CYBRD1	GEO	
3187	CYCS	GEO	
3188	CYFIP1	GEO	
3189	CYHR1	GEO	
3190	CYorf15A	GEO	
3191	CYorf15B	GEO	
3192	CYP11A1	GAD	
3193	CYP17A1	GAD	
3194	CYP19A1	GAD	
3195	CYP1A1	Both	
3196	CYP1A2	GAD	
3197	CYP20A1	GEO	
3198	CYP24A1	Both	

3199	CYP27B1	GAD	
3200	CYP2A13	GEO	
3201	CYP2A6	GAD	
3202	CYP2B6	GEO	
3203	CYP2B7P1	GEO	
3204	CYP2C18	GEO	
3205	CYP2C19	GAD	
3206	CYP2C8	Both	
3207	CYP2C9	Both	
3208	CYP2D6	GAD	
3209	CYP2E1	Both	
3210	CYP2J2	GEO	
3211	CYP2R1	GEO	
3212	CYP2S1	GEO	
3213	CYP2U1	GEO	
3214	CYP2W1	Both	
3215	CYP3A4	Both	
3216	CYP3A5	Both	
3217	CYP4A11	GEO	
3218	CYP4F12	GEO	
3219	CYP4F2	GEO	
3220	CYP4F22	GEO	
3221	CYP4F3	GEO	
3222	CYP4V2	GEO	
3223	CYP4X1	GEO	
3224	CYP7A1	GAD	
3225	CYR61	GEO	
3226	CYSLTR1	GEO	
3227	CYTB	GAD	
3228	CYTH2	GEO	
3229	D2HGDH	GEO	
3230	DAB2	GEO	
3231	DACH	GEO	
3232	DACH1	GEO	
3233	DACT1	Both	
3234	DACT2	GEO	
3235	DACT3	GEO	
3236	DAG1	Both	
3237	DAO	Both	
3238	DAP	GEO	
3239	DAPK2	GEO	
3240	DARC	GEO	
3241	DARS	GEO	
3242	DARS2	GEO	
3243	DAZAP1	GEO	
3244	DBN1	GEO	
3245	DBP	GEO	
3246	DCAF13	GEO	
3247	DCAF16	GEO	
3248	DCAF6	GEO	

3249	DCAF8	GEO	
3250	DCAKD	GEO	
3251	DCC	Both	
3252	DCDC2	GEO	
3253	DCLK2	GEO	
3254	DCP1A	GEO	
3255	DCP1B	GEO	
3256	DCTD	GEO	
3257	DCTN3	GEO	
3258	DCTN5	Both	
3259	DCTN6	GEO	
3260	DCUN1D1	GEO	
3261	DCUN1D4	GEO	
3262	DCUN1D5	GEO	
3263	DDA1	GEO	
3264	DDA3	GEO	
3265	DDB2	GEO	
3266	DDC	GEO	
3267	DDHD1	GEO	
3268	DDIT4	GEO	
3269	DDN	GEO	
3270	DDOST	GEO	
3271	DDX10	GEO	
3272	DDX17	GEO	
3273	DDX18	GEO	
3274	DDX21	GEO	
3275	DDX25	GAD	
3276	DDX26B	GEO	
3277	DDX27	GEO	
3278	DDX31	GEO	
3279	DDX3X	GEO	
3280	DDX3Y	GEO	
3281	DDX47	GEO	
3282	DDX53	GEO	
3283	DDX60	GEO	
3284	DEDD2	GEO	
3285	DEFA3	GEO	
3286	DEFA5	Both	
3287	DEFA6	GEO	
3288	DEFB1	Both	
3289	DEFB4	GAD	
3290	DEGS1	GEO	
3291	DENND1A	GEO	
3292	DENND1C	GEO	
3293	DENND2C	GEO	
3294	DENND3	GEO	
3295	DENND5A	GEO	
3296	DENR	GEO	
3297	DEPDC6	GEO	
3298	DEXI	GEO	

3299	DFNA5	Both	
3300	DGAT2	GEO	
3301	DGCR10	GEO	
3302	DGCR8	GEO	
3303	DGKA	GEO	
3304	DGKD	GEO	
3305	DGKH	GEO	
3306	DHCR7	GEO	
3307	DHDPSL	GEO	
3308	DHFR	Both	
3309	DHR11	GEO	
3310	DHRS12	GEO	
3311	DHRS2	GEO	
3312	DHRS7	GEO	
3313	DHRS7C	GEO	
3314	DHX15	GEO	
3315	DHX36	GAD	
3316	DIAPH1	GEO	
3317	DIAPH2	GEO	
3318	DIAPH3	GEO	
3319	DIMT1L	GEO	
3320	DIO1	GEO	
3321	DIO3-OS	GEO	
3322	DIP2C	GEO	
3323	DIRAS1	GEO	
3324	DIRC1	GEO	
3325	DIS3	GEO	
3326	DISP2	GEO	
3327	DIXDC1	GEO	
3328	DKFZP434F142	GEO	
3329	DKFZP434L187	GEO	
3330	DKFZP564O0823	GEO	
3331	DKK1	GEO	
3332	DKK3	Both	
3333	DKK4	GEO	
3334	DLC1	GEO	
3335	DLD	GAD	
3336	DLEU1	GEO	
3337	DLEU2	GEO	
3338	DLG1	GEO	
3339	DLG3	GEO	
3340	DLG4	GEO	
3341	DLG5	GAD	
3342	DLGAP4	GEO	
3343	DLL1	GEO	
3344	DLX1	GEO	
3345	DLX2	GEO	
3346	DLX4	GEO	
3347	DMBT1	Both	
3348	DMBX1	GEO	

3349	DMD	GEO	
3350	DMGDH	GEO	
3351	DMN	GEO	
3352	DMRT2	GEO	
3353	DMXL1	GEO	
3354	DNAH12	GAD	
3355	DNAH14	GEO	
3356	DNAI2	GEO	
3357	DNAJA1	GEO	
3358	DNAJA2	GEO	
3359	DNAJB1	GEO	
3360	DNAJB4	GEO	
3361	DNAJB6	GEO	
3362	DNAJB9	GEO	
3363	DNAJC12	GEO	
3364	DNAJC2	GEO	
3365	DNAJC24	GEO	
3366	DNAJC3	GEO	
3367	DNAJC4	GEO	
3368	DNAJC7	GEO	
3369	DNAL1	GEO	
3370	DNALI1	GEO	
3371	DNASE1	GEO	
3372	DNASE1L3	GEO	
3373	DNHD1	GEO	
3374	DNM1	GEO	
3375	DNM1L	GEO	
3376	DNMT1	GAD	
3377	DNMT3A	GAD	
3378	DNMT3B	GAD	
3379	DOC2BL	GEO	
3380	DOCK1	GEO	
3381	DOCK2	GEO	
3382	DOCK3	GAD	
3383	DOCK7	GEO	
3384	DOCK8	GEO	
3385	DOK1	GEO	
3386	DOK4	GEO	
3387	DOLPP1	GEO	
3388	DOPEY2	GEO	
3389	DPAGT1	GEO	
3390	DPEP2	GEO	
3391	DPF1	GEO	
3392	DPF3	GEO	
3393	DPH2	GEO	
3394	DPH3	GEO	
3395	DPH5	GEO	
3396	DPM1	GEO	
3397	DPP10	GEO	
3398	DPP4	GEO	

3399	DPP6	GEO	
3400	DPP8	GEO	
3401	DPT	GEO	
3402	DPY19L2P2	GEO	
3403	DPY30	GEO	
3404	DPYD	Both	
3405	DPYS	GAD	
3406	DPYSL3	GEO	
3407	DPYSL4	GEO	
3408	DQ515897	GAD	
3409	DQX1	GEO	
3410	DRAM	GEO	
3411	DRAM1	GEO	
3412	DRD1IP	GEO	
3413	DRD2	GAD	
3414	DRD3	GAD	
3415	DRD4	GAD	
3416	DSC3	GEO	
3417	DSCAML1	GEO	
3418	DSCR1L1	GEO	
3419	DSEL	GEO	
3420	DSG2	GEO	
3421	DSG3	GEO	
3422	DTL	GEO	
3423	DTNB	GEO	
3424	DTNBP1	GEO	
3425	DTWD1	GEO	
3426	DTX2	GEO	
3427	DTX3L	GEO	
3428	DTX4	GEO	
3429	DUOX2	GEO	
3430	DUOXA2	GEO	
3431	DUS3L	GEO	
3432	DUS4L	GEO	
3433	DUSP1	GEO	
3434	DUSP10	GEO	
3435	DUSP13	GEO	
3436	DUSP19	GEO	
3437	DUSP21	GEO	
3438	DUSP27	GEO	
3439	DUSP4	GEO	
3440	DUSP5	GEO	
3441	DUSP6	GEO	
3442	DUSP8	GEO	
3443	DUSP9	GEO	
3444	DUT	GEO	
3445	DUXAP10	GEO	
3446	DYNC1I1	GEO	
3447	DYNC2H1	GEO	
3448	DYNLL1	GEO	
3449	DYNLRB1	GEO	
3450	DYNLT1	GEO	
3451	DYRK2	GEO	
3452	DYRK3	GEO	
3453	DYRK4	GEO	
3454	DYX1C1	GEO	
3455	DZIP1	GEO	
3456	DZIP1L	GEO	
3457	E2F1	GEO	
3458	E2F2	Both	
3459	E2F3	GEO	
3460	E2F5	GEO	
3461	EBF1	GEO	
3462	ECHDC2	GEO	
3463	ECM1	Both	
3464	ECOP	GEO	
3465	ECRP	GEO	
3466	ECSCR	GEO	
3467	EDAR	GEO	
3468	EDARADD	GEO	
3469	EDEM1	GEO	
3470	EDG2	GEO	
3471	EDN1	GEO	
3472	EDNRA	GEO	
3473	EEF1A1	GEO	
3474	EEF1D	GEO	
3475	EEF1E1	GEO	
3476	EEF2K	GEO	
3477	EFCAB4B	GEO	
3478	EFCAB7	GEO	
3479	EFEMP1	Both	
3480	EFHA1	GEO	
3481	EFHA2	GEO	
3482	EFHC2	GEO	
3483	EFNA1	GEO	
3484	EFNA3	GEO	
3485	EFNB1	GEO	
3486	EFNB3	GEO	
3487	EFR3A	GEO	
3488	EFS	GEO	
3489	EFTUD1	GEO	
3490	EGF	GAD	
3491	EGFL7	GEO	
3492	EGFLAM	GEO	
3493	EGFR	Both	
3494	EGLN3	GEO	
3495	EGR1	GEO	
3496	EGR2	GEO	
3497	EGR4	GEO	
3498	EHBP1L1	GEO	

3499	EHD1	GEO	
3500	EHD2	GEO	
3501	EHD4	GEO	
3502	EHHADH	GEO	
3503	EHMT1	GAD	
3504	EHMT2	GAD	
3505	EID1	GEO	
3506	EIF1	GEO	
3507	EIF1AX	GEO	
3508	EIF1AY	GEO	
3509	EIF1B	GEO	
3510	EIF2AK2	GEO	
3511	EIF2AK4	GEO	
3512	EIF2B1	GEO	
3513	EIF2S2	GEO	
3514	EIF3C	GAD	
3515	EIF3E	GEO	
3516	EIF3F	GEO	
3517	EIF3H	GAD	
3518	EIF3L	GEO	
3519	EIF4A1	GEO	
3520	EIF4B	GEO	
3521	EIF4E	GEO	
3522	EIF4E3	GEO	
3523	EIF4EBP1	GEO	
3524	EIF4EBP2	GEO	
3525	EIF4G1	GEO	
3526	EIF4G2	GEO	
3527	EIF4H	GAD	
3528	EIF5	GEO	
3529	EIF5B	GEO	
3530	ELAC1	GEO	
3531	ELANE	GEO	
3532	ELAVL1	GEO	
3533	ELF1	GEO	
3534	ELF2	GEO	
3535	ELF3	GEO	
3536	ELMOD2	GEO	
3537	ELOVL6	GEO	
3538	ELTD1	GEO	
3539	EMCN	GEO	
3540	EME2	GEO	
3541	EMID1	GEO	
3542	EMID2	GEO	
3543	EMILIN1	GEO	
3544	EML1	GEO	
3545	EML3	GEO	
3546	EML4	GEO	
3547	EML6	GEO	
3548	EMP3	GEO	

3549	EMR2	GEO	
3550	ENAH	GEO	
3551	ENAM	GEO	
3552	ENHO	GEO	
3553	ENO3	GEO	
3554	ENOSF1	GEO	
3555	ENOX1	GEO	
3556	ENPEP	GEO	
3557	ENPP2	GEO	
3558	ENPP3	GEO	
3559	ENPP5	GEO	
3560	ENPP6	GEO	
3561	ENTPD3	GEO	
3562	ENTPD4	GEO	
3563	ENTPD6	GEO	
3564	ENY2	GEO	
3565	EPB41L2	GEO	
3566	EPB41L4B	GEO	
3567	EPB41L5	GEO	
3568	EPB49	GEO	
3569	EPCAM	GEO	
3570	EPHA1	GEO	
3571	EPHA10	GEO	
3572	EPHA3	GEO	
3573	EPHA4	GEO	
3574	EPHA7	GEO	
3575	EPHB3	GEO	
3576	EPHX1	GAD	
3577	EPN3	GEO	
3578	EPOR	GEO	
3579	EPPK1	GEO	
3580	EPS15	GEO	
3581	EPS8	GEO	
3582	EPS8L2	GEO	
3583	EPS8L3	GEO	
3584	EPSTI1	GEO	
3585	ERBB2IP	GEO	
3586	ERBB3	GAD	
3587	ERBB4	Both	
3588	ERCC1	Both	
3589	ERCC2	GAD	
3590	ERCC3	GAD	
3591	ERCC4	GAD	
3592	ERCC5	GAD	
3593	ERCC6	Both	
3594	ERG	GEO	
3595	ERGIC1	GEO	
3596	ERGIC3	GEO	
3597	ERI1	GEO	
3598	ERICH1	GEO	

3599	ERN2	GEO	
3600	ERO1L	GEO	
3601	ERRFI1	GEO	
3602	ESAM	GEO	
3603	ESCO1	GEO	
3604	ESF1	GEO	
3605	ESPL1	GEO	
3606	ESPN	GEO	
3607	ESPNP	GEO	
3608	ESR1	Both	
3609	ESR2	GAD	
3610	ESRP1	GEO	
3611	ESRRA	GEO	
3612	ESRRG	GAD	
3613	ESYT2	GEO	
3614	ETF1	GEO	
3615	ETFA	GEO	
3616	ETNK2	GEO	
3617	ETS1	GEO	
3618	ETV6	GEO	
3619	ETV7	GEO	
3620	EVL	GEO	
3621	EVPL	GEO	
3622	EVX1	GEO	
3623	EXD3	GEO	
3624	EXDL2	GEO	
3625	EXO1	GAD	
3626	EXOC1	GEO	
3627	EXOC2	GAD	
3628	EXOC3	GEO	
3629	EXOC5	GEO	
3630	EXOC6B	GEO	
3631	EXOG	GEO	
3632	EXOSC1	GEO	
3633	EXOSC4	GEO	
3634	EXOSC8	GEO	
3635	EXPH5	GEO	
3636	EXT1	GEO	
3637	EYA1	GEO	
3638	EYA2	GEO	
3639	EYA3	GEO	
3640	EYA4	GEO	
3641	F11	GEO	
3642	F11R	GEO	
3643	F13B	GAD	
3644	F2	Both	
3645	F2R	GEO	
3646	F2RL1	GEO	
3647	F2RL2	GEO	
3648	F5	Both	

3649	F7	GEO	
3650	F8	GEO	
3651	FA2H	GEO	
3652	FAAH	GAD	
3653	FABP2	GAD	
3654	FADS2	GEO	
3655	FADS3	GEO	
3656	FAF1	GAD	
3657	FAIM	GEO	
3658	FAIM2	GEO	
3659	FAIM3	GEO	
3660	FAM102B	GEO	
3661	FAM105A	GEO	
3662	FAM107B	GEO	
3663	FAM108C1	GEO	
3664	FAM110A	GEO	
3665	FAM110B	GEO	
3666	FAM111A	GEO	
3667	FAM113B	GEO	
3668	FAM114A1	GEO	
3669	FAM116A	GEO	
3670	FAM119A	GEO	
3671	FAM120A	GEO	
3672	FAM120B	GEO	
3673	FAM122C	GEO	
3674	FAM123A	GEO	
3675	FAM124A	GEO	
3676	FAM125A	GEO	
3677	FAM126B	GEO	
3678	FAM127A	GEO	
3679	FAM129A	GEO	
3680	FAM131A	GEO	
3681	FAM131C	GEO	
3682	FAM132A	GEO	
3683	FAM134B	GEO	
3684	FAM13A	GEO	
3685	FAM13C	GEO	
3686	FAM14A	GEO	
3687	FAM150B	GEO	
3688	FAM151A	GEO	
3689	FAM156A	GEO	
3690	FAM160A1	GEO	
3691	FAM161B	GEO	
3692	FAM162A	GEO	
3693	FAM167A	GEO	
3694	FAM169A	GEO	
3695	FAM173B	GEO	
3696	FAM176A	GEO	
3697	FAM179B	GEO	
3698	FAM184B	GEO	

3699	FAM189A1	GEO	
3700	FAM189A2	GEO	
3701	FAM195A	GEO	
3702	FAM195B	GEO	
3703	FAM198B	GEO	
3704	FAM19A1	GEO	
3705	FAM19A2	GEO	
3706	FAM200A	GEO	
3707	FAM20A	GEO	
3708	FAM26D	GEO	
3709	FAM29A	GEO	
3710	FAM38A	GEO	
3711	FAM38B	GEO	
3712	FAM3A	GEO	
3713	FAM3B	GEO	
3714	FAM3D	GEO	
3715	FAM40B	GEO	
3716	FAM41C	GEO	
3717	FAM43A	GEO	
3718	FAM44B	GEO	
3719	FAM46A	GAD	
3720	FAM48A	GEO	
3721	FAM49B	GEO	
3722	FAM50A	GEO	
3723	FAM50B	GEO	
3724	FAM55A	GEO	
3725	FAM55C	GEO	
3726	FAM57A	GEO	
3727	FAM5C	GEO	
3728	FAM60A	GEO	
3729	FAM62B	GEO	
3730	FAM65B	GEO	
3731	FAM69A	GEO	
3732	FAM69B	GEO	
3733	FAM71F2	GEO	
3734	FAM73B	GEO	
3735	FAM78A	GEO	
3736	FAM7A1	GEO	
3737	FAM7A3	GEO	
3738	FAM82A	GEO	
3739	FAM83D	GEO	
3740	FAM83E	GEO	
3741	FAM83F	GEO	
3742	FAM83H	GEO	
3743	FAM84A	GEO	
3744	FAM84B	GEO	
3745	FAM89A	GEO	
3746	FAM89B	GEO	
3747	FAM91A2	GEO	
3748	FAM92B	GAD	

3749	FAM95B1	GEO	
3750	FAM98B	GEO	
3751	FANCA	GEO	
3752	FANCB	GEO	
3753	FANCD2	GAD	
3754	FANCG	Both	
3755	FANCI	GEO	
3756	FANCL	GEO	
3757	FAR2	GEO	
3758	FARP1	GEO	
3759	FARP2	GEO	
3760	FARSB	GEO	
3761	FAS	Both	
3762	FASLG	GAD	
3763	FASN	GEO	
3764	FASTKD3	GEO	
3765	FBLIM1	GEO	
3766	FBLN2	GEO	
3767	FBLN5	GEO	
3768	FBN1	GEO	
3769	FBRS	GEO	
3770	FBXL11	GEO	
3771	FBXL13	GEO	
3772	FBXL14	GEO	
3773	FBXL2	GEO	
3774	FBXO11	GEO	
3775	FBXO16	GEO	
3776	FBXO17	GEO	
3777	FBXO2	GEO	
3778	FBXO27	GEO	
3779	FBXO3	GEO	
3780	FBXO32	GEO	
3781	FBXO38	GAD	
3782	FBXO44	GEO	
3783	FBXO46	GEO	
3784	FBXO8	GEO	
3785	FBXW2	GEO	
3786	FCAR	GEO	
3787	FCER1A	Both	
3788	FCER1G	GEO	
3789	FCF1	GEO	
3790	FCGR1A	GAD	
3791	FCGR2A	Both	
3792	FCGR2C	GAD	
3793	FCGR3A	GAD	
3794	FCGR3B	GEO	
3795	FCGRT	GEO	
3796	FCHSD1	GEO	
3797	FCN3	GEO	
3798	FCRL2	GEO	

3799	FCRL3	GAD	
3800	FDFT1	GEO	
3801	FEM1B	GEO	
3802	FER	GEO	
3803	FER1L4	GEO	
3804	FGA	GEO	
3805	FGB	Both	
3806	FGD2	GEO	
3807	FGD4	GEO	
3808	FGD6	GEO	
3809	FGF10	GEO	
3810	FGF3	GEO	
3811	FGF4	GAD	
3812	FGF7	GEO	
3813	FGF9	GEO	
3814	FGFBP1	GEO	
3815	FGFR1	GEO	
3816	FGFR1OP	GAD	
3817	FGFR3	GEO	
3818	FGFR4	Both	
3819	FGFRL1	GEO	
3820	FGG	GEO	
3821	FGGY	GEO	
3822	FGL1	GEO	
3823	FH	GEO	
3824	FHIT	GAD	
3825	FHOD3	GEO	
3826	FIBCD1	GEO	
3827	FIG4	GEO	
3828	FIGN	GEO	
3829	FJX1	GEO	
3830	FKBP11	GEO	
3831	FKBP1A	GEO	
3832	FKBP3	GEO	
3833	FKBP4	GEO	
3834	FKBP5	GEO	
3835	FKSG30	GEO	
3836	FKSG49	GEO	
3837	FLG	GAD	
3838	FLI1	GEO	
3839	FLJ10357	GEO	
3840	FLJ10404	GEO	
3841	FLJ10826	GEO	
3842	FLJ10945	GEO	
3843	FLJ11017	GEO	
3844	FLJ11151	GEO	
3845	FLJ12610	GEO	
3846	FLJ13391	GEO	
3847	FLJ13744	GEO	
3848	FLJ14186	GEO	

3849	FLJ14213	GEO	
3850	FLJ14981	GEO	
3851	FLJ20152	GEO	
3852	FLJ20184	GEO	
3853	FLJ20273	GEO	
3854	FLJ20489	GEO	
3855	FLJ20647	GEO	
3856	FLJ22167	GEO	
3857	FLJ22763	GEO	
3858	FLJ23867	GEO	
3859	FLJ27352	GEO	
3860	FLJ30901	GEO	
3861	FLJ31306	GEO	
3862	FLJ32679	GEO	
3863	FLJ33996	GEO	
3864	FLJ34515	GEO	
3865	FLJ34969	GEO	
3866	FLJ35700	GEO	
3867	FLJ35776	GEO	
3868	FLJ36031	GEO	
3869	FLJ36840	GEO	
3870	FLJ37464	GEO	
3871	FLJ37644	GEO	
3872	FLJ38379	GEO	
3873	FLJ40113	GEO	
3874	FLJ40292	GEO	
3875	FLJ41603	GEO	
3876	FLJ41757	GEO	
3877	FLJ43276	GEO	
3878	FLJ43855	GEO	
3879	FLJ45139	GAD	
3880	FLJ45340	GEO	
3881	FLJ45445	GEO	
3882	FLJ45803	Both	
3883	FLJ45949	GEO	
3884	FLNA	GEO	
3885	FLNB	GEO	
3886	FLNC	GEO	
3887	FLOT1	GEO	
3888	FLRT2	GEO	
3889	FLRT3	GEO	
3890	FLT1	GAD	
3891	FLT3LG	GEO	
3892	FMN2	GEO	
3893	FMNL2	GEO	
3894	FMO1	GEO	
3895	FMO3	Both	
3896	FMO4	GEO	
3897	FMO5	GEO	
3898	FN3K	GEO	

3899	FNBP4	GEO	
3900	FNDC3A	GEO	
3901	FNDC3B	GEO	
3902	FNDC4	GEO	
3903	FOLH1	GAD	
3904	FOLR1	Both	
3905	FOLR2	GEO	
3906	FOS	GEO	
3907	FOSB	GEO	
3908	FOSL1	GEO	
3909	FOSL2	GEO	
3910	FOXA3	GEO	
3911	FOXC1	GEO	
3912	FOXC2	GEO	
3913	FOXD1	GEO	
3914	FOXD2	GEO	
3915	FOXD3	GEO	
3916	FOXJ3	GEO	
3917	FOXL2	GEO	
3918	FOXN2	GEO	
3919	FOXN3	GEO	
3920	FOXO1A	GEO	
3921	FOXP2	GEO	
3922	FOXRED2	GEO	
3923	FPGS	GAD	
3924	FPR1	GEO	
3925	FPRL1	GEO	
3926	FRAP1	GAD	
3927	FRAS1	GEO	
3928	FRAT2	GEO	
3929	FREM1	GEO	
3930	FRG1	GEO	
3931	FRG1B	GEO	
3932	FRK	GEO	
3933	FRMD1	GEO	
3934	FRMD3	GEO	
3935	FRMD4A	GEO	
3936	FRYL	GEO	
3937	FSIP2	GEO	
3938	FST	GEO	
3939	FSTL1	GEO	
3940	FSTL3	GEO	
3941	FSTL5	GEO	
3942	FTCD	GEO	
3943	FTL	GEO	
3944	FUBP1	GEO	
3945	FUCA1	GEO	
3946	FUNDC1	GEO	
3947	FURIN	Both	
3948	FUT10	GEO	

3949	FUT2	Both	
3950	FUT3	GEO	
3951	FUT4	GEO	
3952	FUT6	GEO	
3953	FXR1	GEO	
3954	FXYD2	GEO	
3955	FY	GAD	
3956	FYB	GEO	
3957	FYCO1	GEO	
3958	FZD3	GEO	
3959	FZD8	GEO	
3960	G0S2	GEO	
3961	GABARAPL1	GEO	
3962	GABPB1	GEO	
3963	GABRA2	GEO	
3964	GABRB2	GEO	
3965	GAD1	GEO	
3966	GADD45A	Both	
3967	GAGE1	GEO	
3968	GAGE12F	GEO	
3969	GAL	GEO	
3970	GAL3ST1	GEO	
3971	GALIG	GEO	
3972	GALK2	GEO	
3973	GALM	GEO	
3974	GALNT1	GEO	
3975	GALNT14	GEO	
3976	GALNT4	GEO	
3977	GALR2	GEO	
3978	GAN	GEO	
3979	GAP43	GEO	
3980	GAPDH	GEO	
3981	GAPVD1	GEO	
3982	GAR1	GEO	
3983	GAS1	GEO	
3984	GAS5	GEO	
3985	GAST	GEO	
3986	GATA2	GEO	
3987	GATA3	GEO	
3988	GATA6	GAD	
3989	GATAD2A	GEO	
3990	GATM	GEO	
3991	GBA	GEO	
3992	GBA2	GEO	
3993	GBA3	GEO	
3994	GBAS	GEO	
3995	GBE1	GEO	
3996	GBGT1	GEO	
3997	GBP1	GEO	
3998	GBP2	GEO	

3999	GBP4	GEO	
4000	GC	GEO	
4001	GCA	GEO	
4002	GCAT	GEO	
4003	GCC2	GEO	
4004	GCFC1	GEO	
4005	GCKR	GAD	
4006	GCLM	GEO	
4007	GCN1L1	GEO	
4008	GCOM1	GEO	
4009	GDA	GEO	
4010	GDAP1	GEO	
4011	GDE1	GEO	
4012	GDF11	GEO	
4013	GDI1	GEO	
4014	GEFT	GEO	
4015	GEMIN5	GEO	
4016	GET4	GEO	
4017	GFI1	GEO	
4018	GFOD1	GEO	
4019	GFPT1	GEO	
4020	GFPT2	GEO	
4021	GFRA1	GEO	
4022	GGA1	GEO	
4023	GGA2	GEO	
4024	GGA3	GEO	
4025	GGCT	GEO	
4026	GGCX	GEO	
4027	GGH	Both	
4028	GGT2	GEO	
4029	GGT3P	GEO	
4030	GGT5	GEO	
4031	GGT6	GEO	
4032	GGT7	GEO	
4033	GGT8P	GEO	
4034	GGTLA1	GEO	
4035	GH1	Both	
4036	GHR	GEO	
4037	GHRL	GAD	
4038	GHSR	Both	
4039	GIF	GAD	
4040	GIMAP2	GEO	
4041	GIMAP4	GEO	
4042	GIMAP5	GEO	
4043	GIMAP7	GEO	
4044	GIMAP8	GEO	
4045	GINS2	GEO	
4046	GIPC1	GEO	
4047	GIPR	GEO	
4048	GIT2	GEO	

4049	GIYD2	GEO	
4050	GJA1	GEO	
4051	GJB2	GEO	
4052	GJB3	GEO	
4053	GJB4	GEO	
4054	GJB5	GEO	
4055	GJB6	GEO	
4056	GJC1	GEO	
4057	GK	GEO	
4058	GLA	GEO	
4059	GLCCI1	GEO	
4060	GLCE	GEO	
4061	GLDN	GEO	
4062	GLG1	GEO	
4063	GLI1	Both	
4064	GLI2	GEO	
4065	GLI3	Both	
4066	GLIPR1	GEO	
4067	GLIPR2	GEO	
4068	GLIS3	GEO	
4069	GLMN	GEO	
4070	GLOD5	GEO	
4071	GLP1R	GEO	
4072	GLRA2	GEO	
4073	GLRX	GEO	
4074	GLRX3	GEO	
4075	GLT8D1	GEO	
4076	GLUD1	GEO	
4077	GLUL	GEO	
4078	GLYATL1	GEO	
4079	GMPPB	GAD	
4080	GMPR	GEO	
4081	GNAI1	GEO	
4082	GNAL	GEO	
4083	GNAO1	GEO	
4084	GNAQ	GEO	
4085	GNB1	GEO	
4086	GNB1L	GEO	
4087	GNB3	GEO	
4088	GNE	GEO	
4089	GNG11	GEO	
4090	GNG13	GEO	
4091	GNL3	GEO	
4092	GNLY	GEO	
4093	GNPAT	GEO	
4094	GNPDA2	GEO	
4095	GOLGA2B	GEO	
4096	GOLGA6	GEO	
4097	GOLGA8E	GEO	
4098	GOLGA9P	GEO	

4099	GOLPH2	GEO	
4100	GOLT1B	GEO	
4101	GON4L	GEO	
4102	GORASP1	GEO	
4103	GP9	GEO	
4104	GPAM	GEO	
4105	GPAT2	GEO	
4106	GPATCH2	GEO	
4107	GPATCH4	GEO	
4108	GPBAR1	GAD	
4109	GPC3	GEO	
4110	GPC5	GAD	
4111	GPD2	GEO	
4112	GPHA2	GEO	
4113	GPLD1	GEO	
4114	GPM6A	GEO	
4115	GPR107	GEO	
4116	GPR109B	GEO	
4117	GPR110	GEO	
4118	GPR112	GEO	
4119	GPR120	GEO	
4120	GPR124	GEO	
4121	GPR125	GEO	
4122	GPR126	GEO	
4123	GPR128	GEO	
4124	GPR132	GEO	
4125	GPR135	GEO	
4126	GPR143	GEO	
4127	GPR153	GEO	
4128	GPR155	GEO	
4129	GPR156	GEO	
4130	GPR161	GEO	
4131	GPR17	GEO	
4132	GPR172A	GEO	
4133	GPR172B	GEO	
4134	GPR177	GEO	
4135	GPR27	GEO	
4136	GPR30	GEO	
4137	GPR34	GEO	
4138	GPR37	GEO	
4139	GPR39	GEO	
4140	GPR44	GEO	
4141	GPR82	GEO	
4142	GPR89A	GEO	
4143	GPR98	GEO	
4144	GPRASP2	GEO	
4145	GPRC5B	GEO	
4146	GPRC5C	GEO	
4147	GPRC5D	GEO	
4148	GPS2	GEO	

4149	GPSM1	GEO	
4150	GPSM2	GEO	
4151	GPT2	GEO	
4152	GPX1	Both	
4153	GPX4	GAD	
4154	GPX8	GEO	
4155	GRAMD1C	GEO	
4156	GRASP	GEO	
4157	GREB1	GEO	
4158	GREM1	Both	
4159	GRHL2	GEO	
4160	GRHPR	GEO	
4161	GRIA3	GEO	
4162	GRIA4	GEO	
4163	GRIK2	GEO	
4164	GRIK4	GEO	
4165	GRIN1	GEO	
4166	GRIN2A	GEO	
4167	GRIN2D	GEO	
4168	GRINA	GEO	
4169	GRLF1	GEO	
4170	GRM8	GEO	
4171	GSDMB	Both	
4172	GSDML	GAD	
4173	GSG1L	GEO	
4174	GSPT1	GEO	
4175	GSR	GEO	
4176	GSTA1	GAD	
4177	GSTA2	GAD	
4178	GSTA4	GAD	
4179	GSTK1	GEO	
4180	GSTM1	GAD	
4181	GSTM2	Both	
4182	GSTM3	Both	
4183	GSTO1	Both	
4184	GSTO2	GAD	
4185	GSTP1	GAD	
4186	GSTT1	Both	
4187	GSTT2	GAD	
4188	GTF2B	GEO	
4189	GTF2E2	GAD	
4190	GTF2F2	GEO	
4191	GTF2H1	GEO	
4192	GTF2H2	GEO	
4193	GTF2H2D	GEO	
4194	GTF2I	GEO	
4195	GTF2IRD2	GEO	
4196	GTF2IRD2B	GEO	
4197	GTPBP1	GEO	
4198	GTPBP3	GEO	

4199	GTPBP4	GEO	
4200	GTPBP5	GEO	
4201	GTPBP6	GEO	
4202	GTSF1	GEO	
4203	GTSF1L	GEO	
4204	GUCA1B	GEO	
4205	GUCY1A3	GEO	
4206	GUCY1B2	GEO	
4207	GUCY1B3	GEO	
4208	GUCY2C	GEO	
4209	GUK1	GEO	
4210	GUSB	GEO	
4211	GUSBP3	GEO	
4212	GXYLT2	GEO	
4213	GYG1	GEO	
4214	GYG2	GEO	
4215	GYLTL1B	GEO	
4216	GYPA	GAD	
4217	GYPC	GEO	
4218	GZMK	GEO	
4219	H19	GEO	
4220	H1FX	GEO	
4221	H2AFX	GEO	
4222	H2AFY	GEO	
4223	H2BFS	GEO	
4224	H3F3B	GEO	
4225	HABP4	GAD	
4226	HACE1	GEO	
4227	HADH	GEO	
4228	HAMP	GEO	
4229	HAND1	GEO	
4230	HAPLN2	GEO	
4231	HAS1	GEO	
4232	HAUS4	GEO	
4233	HAUS7	GEO	
4234	HAVCR1	GEO	
4235	HAVCR2	GEO	
4236	HBB	GEO	
4237	HBE1	GEO	
4238	HBEGF	GEO	
4239	HBG1	GEO	
4240	HBG2	GEO	
4241	HCCS	GEO	
4242	hCG_1776259	GEO	
4243	hCG_1783494	GEO	
4244	hCG_1981531	GEO	
4245	HCG4	GEO	
4246	HCG8	GEO	
4247	HCLS1	GEO	
4248	HCP5	GEO	

4249	HDAC2	GEO	
4250	HDAC4	GEO	
4251	HDDC2	GEO	
4252	HDGF	GEO	
4253	HDGF2	GEO	
4254	HDGFRP3	GEO	
4255	HDX	GEO	
4256	HEATR2	GEO	
4257	HEATR4	GEO	
4258	HEATR6	GEO	
4259	HEATR7A	GEO	
4260	HEATR7B1	GEO	
4261	HECTD2	GEO	
4262	HEG1	GEO	
4263	HELLS	GEO	
4264	HEMK1	GEO	
4265	HEPACAM	GEO	
4266	HEPH	GEO	
4267	HERC1	GEO	
4268	HERC2	GAD	
4269	HERPUD1	GEO	
4270	HES5	GEO	
4271	HES6	GEO	
4272	HFE	GAD	
4273	HFE2	GAD	
4274	HGFAC	GEO	
4275	HHEX	GEO	
4276	HHIP	Both	
4277	HIATL1	GEO	
4278	HIBADH	GEO	
4279	HIBCH	GEO	
4280	HIC1	Both	
4281	HIF1A	GAD	
4282	HIG1	GEO	
4283	HIGD2A	GEO	
4284	HIP1	GEO	
4285	HIPK3	GEO	
4286	HISPPD2A	GEO	
4287	HIST1H1B	GEO	
4288	HIST1H1C	GEO	
4289	HIST1H1D	GAD	
4290	HIST1H1T	GEO	
4291	HIST1H2AC	GEO	
4292	HIST1H2AD	GEO	
4293	HIST1H2AG	GEO	
4294	HIST1H2BB	GEO	
4295	HIST1H2BC	GEO	
4296	HIST1H2BD	GEO	
4297	HIST1H2BE	GEO	
4298	HIST1H2BF	GEO	

4299	HIST1H2BH	GEO	
4300	HIST1H2BI	GEO	
4301	HIST1H2BJ	GEO	
4302	HIST1H2BK	GEO	
4303	HIST1H2BL	GEO	
4304	HIST1H2BM	GEO	
4305	HIST1H2BN	GEO	
4306	HIST1H2BO	GEO	
4307	HIST1H3C	GEO	
4308	HIST1H3D	GEO	
4309	HIST1H3F	GEO	
4310	HIST1H3G	GEO	
4311	HIST1H3H	GEO	
4312	HIST1H3J	GEO	
4313	HIST1H4B	GEO	
4314	HIST1H4C	GEO	
4315	HIST1H4D	GEO	
4316	HIST2H2AA3	GEO	
4317	HIST2H2AA4	GEO	
4318	HIST2H2AC	GEO	
4319	HIST2H2BE	GEO	
4320	HIST3H2A	GEO	
4321	HIST3H2BB	GEO	
4322	HIST4H4	GEO	
4323	HIVEP2	GEO	
4324	HK1	GEO	
4325	HK2	GEO	
4326	HLA	GAD	
4327	HLA-A	Both	
4328	HLA-B	GAD	
4329	HLA-C	Both	
4330	HLA-DMB	GEO	
4331	HLA-DOA	GEO	
4332	HLA-DPA1	Both	
4333	HLA-DPB1	GEO	
4334	HLA-DQA1	Both	
4335	HLA-DQB1	Both	
4336	HLA-DRA	Both	
4337	HLA-DRB4	GEO	
4338	HLA-DRB5	GAD	
4339	HLA-E	GEO	
4340	HLA-F	GEO	
4341	HLA-G	Both	
4342	HLF	GEO	
4343	HM13	GEO	
4344	HMCN1	GEO	
4345	HMCN2	GEO	
4346	HMFN0839	GEO	
4347	HMGB1	GEO	
4348	HMGB3	GEO	

4349	HMGCLL1	GEO	
4350	HMGCR	Both	
4351	HMGCS2	Both	
4352	HMGN5	GEO	
4353	HMMR	GEO	
4354	HMOX1	Both	
4355	HMP19	GEO	
4356	HMSD	GEO	
4357	HN1	GEO	
4358	HN1L	GEO	
4359	HNF1A	Both	
4360	HNRNPA1	GEO	
4361	HNRNPA3	GEO	
4362	HNRNPAB	GEO	
4363	HNRNPC	GEO	
4364	HNRNPD	GEO	
4365	HNRNPH2	GEO	
4366	HNRNPH3	GEO	
4367	HNRNPL	GEO	
4368	HNRNPM	GEO	
4369	HNRNPUL1	GEO	
4370	HNRPH1	GEO	
4371	HOMER1	GEO	
4372	HOMER3	GEO	
4373	HOM-TES-103	GEO	
4374	HOPX	GEO	
4375	HORMAD1	GEO	
4376	HORMAD2	GAD	
4377	HOXA10	GEO	
4378	HOXA3	GEO	
4379	HOXB13	GEO	
4380	HOXB4	GEO	
4381	HOXB6	GEO	
4382	HOXB7	GEO	
4383	HOXB9	GEO	
4384	HOXC10	GEO	
4385	HOXC13	GEO	
4386	HOXC5	GEO	
4387	HOXC6	GEO	
4388	HOXD10	GEO	
4389	HOXD11	GEO	
4390	HOXD13	GEO	
4391	HOXD4	GEO	
4392	HP1BP3	GEO	
4393	HPCAL1	GEO	
4394	HPD	GEO	
4395	HPDL	GEO	
4396	HPGDS	GEO	
4397	HPRP8BP	GEO	
4398	HPRT1	GAD	

4399	HPSE	GEO	
4400	HPX	GEO	
4401	HR	GEO	
4402	HRAS	Both	
4403	HRASLS3	GEO	
4404	HRASLS5	GEO	
4405	HRBL	GEO	
4406	HRCT1	GEO	
4407	HRG	GEO	
4408	HRH1	GEO	
4409	HRK	GEO	
4410	HRNBP3	GEO	
4411	HS2ST1	GEO	
4412	HS6ST1	GEO	
4413	HSA272196	GEO	
4414	HSBP1L1	GEO	
4415	HSD11B1	GEO	
4416	HSD17B1	GEO	
4417	HSD17B11	GEO	
4418	HSD17B12	GEO	
4419	HSD17B3	Both	
4420	HSD17B4	GEO	
4421	HSD17B6	GEO	
4422	HSD17B7	GEO	
4423	HSF1	GEO	
4424	HSH2D	GEO	
4425	HSP90AA1	GEO	
4426	HSP90AB1	GEO	
4427	HSPA1A	GEO	
4428	HSPA1B	GAD	
4429	HSPA4	GEO	
4430	HSPA4L	GEO	
4431	HSPA6	GEO	
4432	HSPB2	GEO	
4433	HSPB3	GEO	
4434	HSPB8	GEO	
4435	HSPC159	GEO	
4436	HSPG2	GEO	
4437	HTR2B	GEO	
4438	HTRA1	GEO	
4439	HTRA3	GEO	
4440	HUS1	GEO	
4441	HYAL1	GEO	
4442	HYDIN	GEO	
4443	HYI	GEO	
4444	HYLS1	GEO	
4445	HYMAI	GEO	
4446	IARS	GEO	
4447	IBD2	GAD	
4448	IBD5	GAD	

4449	IBSP	GEO	
4450	ICA1L	GEO	
4451	ICAM1	Both	
4452	ICOSLG	Both	
4453	ID1	GEO	
4454	ID2B	GEO	
4455	ID4	GEO	
4456	IDH1	GEO	
4457	IDH2	GEO	
4458	IDH3A	GEO	
4459	IDI2	GEO	
4460	IDS	GEO	
4461	IDUA	GEO	
4462	IER3	GEO	
4463	IER5	GEO	
4464	IFFO2	GEO	
4465	IFI27	GEO	
4466	IFI27L1	GEO	
4467	IFI27L2	GEO	
4468	IFI30	GEO	
4469	IFI44	GEO	
4470	IFI6	GEO	
4471	IFIH1	GEO	
4472	IFIT2	GEO	
4473	IFIT3	GEO	
4474	IFIT5	GEO	
4475	IFITM4P	GEO	
4476	IFNA4	GEO	
4477	IFNAR2	GEO	
4478	IFNG	GAD	
4479	IFNGR1	Both	
4480	IFRD2	GEO	
4481	IFT57	GEO	
4482	IFT74	GEO	
4483	IFT80	GEO	
4484	IFT81	GEO	
4485	IFT88	GEO	
4486	IGF1R	Both	
4487	IGF2	GEO	
4488	IGF2BP1	GEO	
4489	IGF2R	GEO	
4490	IGFBP1	GAD	
4491	IGFBP2	GEO	
4492	IGFBP5	GEO	
4493	IGFBP6	GEO	
4494	IGFBP7	GEO	
4495	IGFBPL1	GEO	
4496	IGFL2	GEO	
4497	IGFL4	GEO	
4498	IGHD	GEO	

4499	IGHG3	GEO	
4500	IGHM	GEO	
4501	IGHMBP2	GEO	
4502	IGHV3-48	GEO	
4503	IGK	GEO	
4504	IGKC	GEO	
4505	IGKV1-5	GEO	
4506	IGKV1D-8	GEO	
4507	IGKV3-20	GEO	
4508	IGKV4-1	GEO	
4509	IGL	GEO	
4510	IGLC7	GEO	
4511	IGLJ3	GEO	
4512	IGLL1	GEO	
4513	IGLL3	GEO	
4514	IGLL3P	GEO	
4515	IGLL5	GEO	
4516	IGLV1-36	GEO	
4517	IGLV1-40	GEO	
4518	IGLV1-44	GEO	
4519	IGLV2-23	GEO	
4520	IGLV4-60	GEO	
4521	IGLV6-57	GEO	
4522	IGSF3	GEO	
4523	IGSF6	GAD	
4524	IGSF9	GEO	
4525	IHH	Both	
4526	IKBKB	Both	
4527	IKBKE	GEO	
4528	IKBKG	GEO	
4529	IKBL	GAD	
4530	IKZF1	Both	
4531	IL10	GAD	
4532	IL10RA	GEO	
4533	IL11	Both	
4534	IL11RA	GEO	
4535	IL12A	GAD	
4536	IL12B	GAD	
4537	IL12RB1	GAD	
4538	IL12RB2	GEO	
4539	IL13	GAD	
4540	IL15RA	GEO	
4541	IL16	Both	
4542	IL17F	GAD	
4543	IL17RB	GEO	
4544	IL17RD	GEO	
4545	IL17REL	GAD	
4546	IL18	GAD	
4547	IL18BP	GEO	
4548	IL18R1	GAD	

4549	IL18RAP	GAD	
4550	IL19	GAD	
4551	IL1A	Both	
4552	IL1B	Both	
4553	IL1F7	GEO	
4554	IL1R1	Both	
4555	IL1RAP	GEO	
4556	IL1RAPL1	GEO	
4557	IL1RN	Both	
4558	IL2	GAD	
4559	IL20RA	GEO	
4560	IL21	GAD	
4561	IL21R	GEO	
4562	IL22	GAD	
4563	IL22RA1	GEO	
4564	IL23A	Both	
4565	IL23R	GAD	
4566	IL26	GAD	
4567	IL27	GAD	
4568	IL2RA	GAD	
4569	IL2RB	Both	
4570	IL2RG	GEO	
4571	IL32	GEO	
4572	IL33	GEO	
4573	IL3RA	GEO	
4574	IL4	GAD	
4575	IL4R	GAD	
4576	IL5	GAD	
4577	IL6	Both	
4578	IL6ST	GEO	
4579	IL6STP	GAD	
4580	IL9R	GEO	
4581	ILDR2	GEO	
4582	ILF3	GEO	
4583	ILVBL	GEO	
4584	IMPA2	GEO	
4585	IMPACT	GEO	
4586	IMPDH1	GEO	
4587	IMPDH2	GEO	
4588	INA	GEO	
4589	ING1	GAD	
4590	ING2	GEO	
4591	INHBE	GEO	
4592	INO80C	GEO	
4593	INPP1	GEO	
4594	INPP5A	GEO	
4595	INPP5D	GEO	
4596	INPP5E	GEO	
4597	INS	GAD	
4598	INSIG1	GEO	

4599	INSL4	GAD	
4600	INSL5	GEO	
4601	INSL6	GAD	
4602	INSM1	GEO	
4603	INSR	Both	
4604	INTS10	GEO	
4605	INTS12	GEO	
4606	INTS2	GEO	
4607	INTS6	GEO	
4608	INVS	GEO	
4609	IP6K1	GAD	
4610	IPO4	GEO	
4611	IPO7	GEO	
4612	IPO9	GEO	
4613	IQCA1	GEO	
4614	IQCE	GEO	
4615	IQCG	GEO	
4616	IQCH	GEO	
4617	IQCJ-SCHIP1	GEO	
4618	IQCK	GEO	
4619	IQGAP1	GEO	
4620	IQGAP2	GEO	
4621	IQGAP3	GEO	
4622	IQSEC1	GEO	
4623	IRAK1	GEO	
4624	IRAK3	GAD	
4625	IRF1	GAD	
4626	IRF2BP2	GEO	
4627	IRF3	GEO	
4628	IRF5	GAD	
4629	IRF6	GEO	
4630	IRF7	GEO	
4631	IRF8	GEO	
4632	IRF9	GEO	
4633	IRGM	GAD	
4634	IRS1	GAD	
4635	IRS2	GAD	
4636	IRX3	GEO	
4637	IRX4	GEO	
4638	IRX5	GEO	
4639	ISCU	GEO	
4640	ISG20L2	GEO	
4641	ISL1	GEO	
4642	ISM1	GEO	
4643	ISX	GEO	
4644	ISYNA1	GEO	
4645	ITFG1	GEO	
4646	ITGA1	GEO	
4647	ITGA10	GEO	
4648	ITGA2	Both	

4649	ITGA3	GEO	
4650	ITGA4	GEO	
4651	ITGA7	GEO	
4652	ITGA8	GEO	
4653	ITGAL	Both	
4654	ITGAM	GEO	
4655	ITGB1	GEO	
4656	ITGB1BP3	GEO	
4657	ITGB3	GAD	
4658	ITGB4	GEO	
4659	ITGB7	GAD	
4660	ITGBL1	GEO	
4661	ITIH1	GEO	
4662	ITK	GEO	
4663	ITM2A	GEO	
4664	ITPA	Both	
4665	ITPKA	GEO	
4666	ITPKB	GEO	
4667	ITPKC	GEO	
4668	ITPR2	GEO	
4669	ITPR3	GEO	
4670	ITPRIPL1	GEO	
4671	IYD	GEO	
4672	JAG1	GEO	
4673	JAK1	GEO	
4674	JAK2	GAD	
4675	JAK3	GEO	
4676	JAKMIP2	GEO	
4677	JAKMIP3	GEO	
4678	JAM2	GEO	
4679	JARID1A	GEO	
4680	JARID1D	GEO	
4681	JCLN	GEO	
4682	JDP2	GEO	
4683	JMJD1A	GEO	
4684	JMJD1C	GEO	
4685	JMJD2C	GEO	
4686	JMJD7	GEO	
4687	JMY	GEO	
4688	JPH1	GEO	
4689	JPH2	GEO	
4690	JUN	Both	
4691	JUNB	GEO	
4692	JUP	GEO	
4693	K1F21B	GAD	
4694	KALRN	GEO	
4695	KANK1	GEO	
4696	KAT2A	GEO	
4697	KAT2B	GEO	
4698	KATNA1	GEO	

4699	KATNAL1	GEO	
4700	KAZ	GEO	
4701	KAZALD1	GEO	
4702	KBTBD11	GEO	
4703	KBTBD2	GEO	
4704	KCNAB2	GEO	
4705	KCNC1	GEO	
4706	KCNC3	GEO	
4707	KCNE1	GEO	
4708	KCNE2	GEO	
4709	KCNE4	GEO	
4710	KCNG3	GEO	
4711	KCNH8	GEO	
4712	KCNIP2	GEO	
4713	KCNIP4	GEO	
4714	KCNJ13	GEO	
4715	KCNJ15	GEO	
4716	KCNK10	GEO	
4717	KCNK15	GEO	
4718	KCNMA1	GEO	
4719	KCNMB1	GEO	
4720	KCNN3	GEO	
4721	KCNN4	Both	
4722	KCNRG	GEO	
4723	KCNV1	GEO	
4724	KCP	GEO	
4725	KCTD1	GEO	
4726	KCTD20	GEO	
4727	KCTD5	GEO	
4728	KCTD6	GEO	
4729	KDELR2	GEO	
4730	KDELR3	GEO	
4731	KDM2B	GEO	
4732	KDM4C	GEO	
4733	KDM5D	GEO	
4734	KDM6B	GEO	
4735	KDR	GAD	
4736	KEAP1	GEO	
4737	KEL	GAD	
4738	KHDC1L	GEO	
4739	KHSRP	GEO	
4740	KIAA0020	GEO	
4741	KIAA0090	GEO	
4742	KIAA0101	GEO	
4743	KIAA0141	GEO	
4744	KIAA0146	GEO	
4745	KIAA0182	GEO	
4746	KIAA0226	GEO	
4747	KIAA0232	GEO	
4748	KIAA0247	GEO	
4749	KIAA0319	GEO	
4750	KIAA0355	GEO	
4751	KIAA0368	GEO	
4752	KIAA0406	GEO	
4753	KIAA0415	GEO	
4754	KIAA0430	GEO	
4755	KIAA0467	GEO	
4756	KIAA0485	GEO	
4757	KIAA0495	GEO	
4758	KIAA0513	GEO	
4759	KIAA0562	GEO	
4760	KIAA0564	GEO	
4761	KIAA0574	GEO	
4762	KIAA0802	GEO	
4763	KIAA0895	GEO	
4764	KIAA0907	GEO	
4765	KIAA1109	Both	
4766	KIAA1161	GEO	
4767	KIAA1211	GEO	
4768	KIAA1274	GEO	
4769	KIAA1333	GEO	
4770	KIAA1377	GEO	
4771	KIAA1430	GEO	
4772	KIAA1432	GEO	
4773	KIAA1462	GEO	
4774	KIAA1467	GEO	
4775	KIAA1522	GEO	
4776	KIAA1586	GEO	
4777	KIAA1598	GEO	
4778	KIAA1609	GEO	
4779	KIAA1632	GEO	
4780	KIAA1659	GEO	
4781	KIAA1683	GEO	
4782	KIAA1712	GEO	
4783	KIAA1731	GEO	
4784	KIAA1737	GEO	
4785	KIAA1754	GEO	
4786	KIAA1755	GEO	
4787	KIAA1797	GEO	
4788	KIAA1826	GEO	
4789	KIAA1881	GEO	
4790	KIAA1912	GEO	
4791	KIAA1920	GEO	
4792	KIAA1984	GEO	
4793	KIAA2022	GEO	
4794	KIF11	GEO	
4795	KIF13A	GEO	
4796	KIF15	GEO	
4797	KIF16B	GEO	
4798	KIF18B	GEO	

4799	KIF19	GEO	
4800	KIF1A	GEO	
4801	KIF21A	GEO	
4802	KIF21B	Both	
4803	KIF26B	GEO	
4804	KIF4A	GEO	
4805	KIF5A	GEO	
4806	KIF5C	GEO	
4807	KIF9	GEO	
4808	KIR2DL2	GAD	
4809	KIR2DL3	GAD	
4810	KIR2DS2	GAD	
4811	KIR2DS4	GAD	
4812	KIR3DL1	GAD	
4813	KISS1R	GEO	
4814	KIT	GEO	
4815	KITLG	GEO	
4816	KLB	GEO	
4817	KLF10	GEO	
4818	KLF11	GEO	
4819	KLF13	GEO	
4820	KLF15	GEO	
4821	KLF2	GEO	
4822	KLF5	GEO	
4823	KLF7	GEO	
4824	KLF8	GEO	
4825	KLHDC1	GAD	
4826	KLHDC10	GEO	
4827	KLHDC8B	GEO	
4828	KLHL13	GEO	
4829	KLHL14	GEO	
4830	KLHL15	GEO	
4831	KLHL24	GEO	
4832	KLHL34	GEO	
4833	KLHL35	GEO	
4834	KLHL4	GEO	
4835	KLHL5	GEO	
4836	KLHL7	GEO	
4837	KLK1	GEO	
4838	KLK11	GEO	
4839	KLK12	GEO	
4840	KLK15	GEO	
4841	KLK6	GEO	
4842	KLK7	GEO	
4843	KLKB1	GEO	
4844	KLRB1	GEO	
4845	KLRC4	GAD	
4846	KLRF1	GEO	
4847	KLRG2	GEO	
4848	KLRK1	Both	
4849	KMO	GEO	
4850	KNG1	GEO	
4851	KPNA1	GEO	
4852	KPNA2	GEO	
4853	KPNA4	GEO	
4854	KPNA5	GEO	
4855	KPNA7	GAD	
4856	KRAS	Both	
4857	KRT10	GEO	
4858	KRT14	GEO	
4859	KRT17P3	GEO	
4860	KRT18	GEO	
4861	KRT18P28	GEO	
4862	KRT18P40	GEO	
4863	KRT19	GEO	
4864	KRT19P2	GEO	
4865	KRT3	GEO	
4866	KRT34	GEO	
4867	KRT37	GEO	
4868	KRT38	GAD	
4869	KRT39	GEO	
4870	KRT5	GEO	
4871	KRT7	GEO	
4872	KRT74	GEO	
4873	KRT8	Both	
4874	KRT8P15	GEO	
4875	KRT8P41	GEO	
4876	KRTAP1-3	GEO	
4877	KRTAP4-1	GEO	
4878	KRTAP5-5	GEO	
4879	KRTCAP3	GEO	
4880	KRTDAP	GEO	
4881	KRTHA5	GEO	
4882	KSR2	GEO	
4883	KTN1	GEO	
4884	KYNU	GEO	
4885	L1CAM	GEO	
4886	L1TD1	GEO	
4887	L2HGDH	GEO	
4888	L3MBTL1	GEO	
4889	L3MBTL2	GEO	
4890	L3MBTL4	GEO	
4891	LACTB2	GEO	
4892	LAD1	GEO	
4893	LAMA1	GEO	
4894	LAMA2	GEO	
4895	LAMA3	GEO	
4896	LAMA4	GEO	
4897	LAMB2	GEO	
4898	LAMP1	GEO	

4899	LAMP3	GEO	
4900	LANCL3	GEO	
4901	LAPTM4B	Both	
4902	LARGE	GEO	
4903	LARP1	GEO	
4904	LARP4	GEO	
4905	LARP6	GEO	
4906	LARP7	GEO	
4907	LASP1	GEO	
4908	LASS4	GEO	
4909	LASS5	GEO	
4910	LASS6	GEO	
4911	LATS2	GEO	
4912	LAYN	GEO	
4913	LBA1	GEO	
4914	LBP	GEO	
4915	LBR	GEO	
4916	LCA5	GEO	
4917	LCE1C	GEO	
4918	LCK	GEO	
4919	LCMT2	GEO	
4920	LCN15	GEO	
4921	LCN2	GEO	
4922	LCOR	GEO	
4923	LCORL	GEO	
4924	LCP1	GEO	
4925	LCP2	GEO	
4926	LCT	GAD	
4927	LDB2	GEO	
4928	LDB3	GEO	
4929	LDHA	GEO	
4930	LEAP2	GEO	
4931	LEFTY1	GEO	
4932	LEP	GAD	
4933	LEPR	Both	
4934	LEPREL1	GEO	
4935	LEPROT	GEO	
4936	LETM1	GEO	
4937	LETM2	GEO	
4938	LFNG	GEO	
4939	LGALS1	GEO	
4940	LGALS3	GEO	
4941	LGALS7	GEO	
4942	LGALS7B	GEO	
4943	LGALS8	GEO	
4944	LGALS9	GEO	
4945	LGALS9C	GEO	
4946	LGALSL	GEO	
4947	LGI1	GEO	
4948	LGI4	GEO	

4949	LGMN	GEO	
4950	LGR4	GEO	
4951	LHFP	GEO	
4952	LHFPL2	GEO	
4953	LHPP	GEO	
4954	LHX2	GEO	
4955	LHX3	GEO	
4956	LHX6	GEO	
4957	LIAS	GEO	
4958	LIF	GAD	
4959	LIG1	GAD	
4960	LIG3	Both	
4961	LIG4	GAD	
4962	LILRA3	GEO	
4963	LILRB3	GEO	
4964	LILRB4	GEO	
4965	LILRB5	GEO	
4966	LIMA1	GEO	
4967	LIMD2	GEO	
4968	LIMK1	GEO	
4969	LIMS1	GEO	
4970	LIMS3	GEO	
4971	LIN28B	GAD	
4972	LIN52	GEO	
4973	LIN7A	GEO	
4974	LIN9	GEO	
4975	LINC00152	GEO	
4976	LINC00265	GEO	
4977	LIPC	GAD	
4978	LLGL2	GEO	
4979	LMAN1	GAD	
4980	LMAN2	GEO	
4981	LMBRD1	GEO	
4982	LMF1	GEO	
4983	LMLN	GEO	
4984	LMNA	GEO	
4985	LMNB2	GEO	
4986	LMO3	GEO	
4987	LMO4	GEO	
4988	LMO7	GEO	
4989	LMOD1	GEO	
4990	LNP1	GEO	
4991	LNX2	GEO	
4992	LOC100124692	GEO	
4993	LOC100128252	GEO	
4994	LOC100128288	GEO	
4995	LOC100128328	GEO	
4996	LOC100128343	GEO	
4997	LOC100128511	GEO	
4998	LOC100128701	GEO	

4999	LOC100128893	GEO	
5000	LOC100128988	GEO	
5001	LOC100129069	GEO	
5002	LOC100129113	GEO	
5003	LOC100129503	GEO	
5004	LOC100129637	GEO	
5005	LOC100130097	GEO	
5006	LOC100130357	GEO	
5007	LOC100130468	GEO	
5008	LOC100130776	GEO	
5009	LOC100131257	GEO	
5010	LOC100131564	GEO	
5011	LOC100131582	GEO	
5012	LOC100131656	GEO	
5013	LOC100131763	GEO	
5014	LOC100132153	GEO	
5015	LOC100132273	GEO	
5016	LOC100132891	GEO	
5017	LOC100132999	GEO	
5018	LOC100133005	GEO	
5019	LOC100133056	GEO	
5020	LOC100133572	GEO	
5021	LOC100133660	GEO	
5022	LOC100133920	GEO	
5023	LOC100134119	GEO	
5024	LOC100190939	GEO	
5025	LOC100216546	GEO	
5026	LOC100240728	GEO	
5027	LOC100287547	GEO	
5028	LOC100287689	GEO	
5029	LOC100287896	GEO	
5030	LOC100288092	GEO	
5031	LOC100288781	GEO	
5032	LOC100289026	GEO	
5033	LOC100289632	GEO	
5034	LOC100290278	GEO	
5035	LOC100292909	GEO	
5036	LOC100292959	GEO	
5037	LOC100499467	GEO	
5038	LOC100505483	GEO	
5039	LOC100505503	GEO	
5040	LOC100505522	GEO	
5041	LOC100505584	GEO	
5042	LOC100505633	GEO	
5043	LOC100505712	GEO	
5044	LOC100505730	GEO	
5045	LOC100505875	GEO	
5046	LOC100505876	GEO	
5047	LOC100506076	GEO	
5048	LOC100506168	GEO	

5049	LOC100506493	GEO	
5050	LOC100506517	GEO	
5051	LOC100506542	GEO	
5052	LOC100506589	GEO	
5053	LOC100506591	GEO	
5054	LOC100506653	GEO	
5055	LOC100506659	GEO	
5056	LOC100506727	GEO	
5057	LOC100506748	GEO	
5058	LOC100506781	GEO	
5059	LOC100506860	GEO	
5060	LOC100506922	GEO	
5061	LOC100506941	GEO	
5062	LOC100506979	GEO	
5063	LOC100507039	GEO	
5064	LOC100507153	GEO	
5065	LOC100507165	GEO	
5066	LOC100507192	GEO	
5067	LOC100507193	GEO	
5068	LOC100507198	GEO	
5069	LOC100507273	GEO	
5070	LOC100507328	GEO	
5071	LOC100507376	GEO	
5072	LOC100507448	GEO	
5073	LOC100507531	GEO	
5074	LOC100507619	GEO	
5075	LOC100507666	GEO	
5076	LOC100507804	GEO	
5077	LOC100507851	GEO	
5078	LOC100508797	GEO	
5079	LOC100508909	GEO	
5080	LOC100509223	GEO	
5081	LOC100509231	GEO	
5082	LOC100509635	GEO	
5083	LOC100509683	GEO	
5084	LOC100509749	GEO	
5085	LOC100509968	GEO	
5086	LOC100510224	GEO	
5087	LOC100510507	GEO	
5088	LOC100510525	GEO	
5089	LOC120364	GEO	
5090	LOC120376	Both	
5091	LOC126235	GEO	
5092	LOC144501	GEO	
5093	LOC145837	GEO	
5094	LOC146336	GEO	
5095	LOC147645	GEO	
5096	LOC148709	GEO	
5097	LOC149134	GEO	
5098	LOC149351	GEO	

5099	LOC150759	GEO	
5100	LOC153546	GEO	
5101	LOC153684	GEO	
5102	LOC157562	GEO	
5103	LOC157860	GEO	
5104	LOC158257	GEO	
5105	LOC158960	GEO	
5106	LOC199800	GEO	
5107	LOC201229	GEO	
5108	LOC201895	GEO	
5109	LOC202451	GEO	
5110	LOC219347	GEO	
5111	LOC220906	GEO	
5112	LOC253012	GEO	
5113	LOC253264	GEO	
5114	LOC254057	GEO	
5115	LOC255512	GEO	
5116	LOC25845	GEO	
5117	LOC26010	GEO	
5118	LOC26102	GEO	
5119	LOC283104	GEO	
5120	LOC283177	GEO	
5121	LOC283352	GEO	
5122	LOC283454	GEO	
5123	LOC283508	GEO	
5124	LOC283516	GEO	
5125	LOC283551	GEO	
5126	LOC283701	GEO	
5127	LOC283859	GEO	
5128	LOC284058	GEO	
5129	LOC284120	GEO	
5130	LOC284417	GEO	
5131	LOC284454	GEO	
5132	LOC284542	GEO	
5133	LOC284801	GEO	
5134	LOC284804	GEO	
5135	LOC285016	GEO	
5136	LOC285074	GEO	
5137	LOC285535	GEO	
5138	LOC285628	GEO	
5139	LOC285636	GEO	
5140	LOC285708	GEO	
5141	LOC285733	GEO	
5142	LOC285735	GEO	
5143	LOC285878	GEO	
5144	LOC285943	GEO	
5145	LOC285989	GEO	
5146	LOC286272	GEO	
5147	LOC286440	GEO	
5148	LOC286467	GEO	
5149	LOC338667	GEO	
5150	LOC339988	GEO	
5151	LOC340843	GEO	
5152	LOC342918	GEO	
5153	LOC344967	GEO	
5154	LOC374443	GEO	
5155	LOC375190	GEO	
5156	LOC387763	GEO	
5157	LOC387921	GEO	
5158	LOC388335	GEO	
5159	LOC388564	GEO	
5160	LOC388886	GEO	
5161	LOC389023	GEO	
5162	LOC389160	GEO	
5163	LOC389249	GEO	
5164	LOC389634	GEO	
5165	LOC389834	GEO	
5166	LOC399744	GEO	
5167	LOC399753	GEO	
5168	LOC400128	GEO	
5169	LOC401022	GEO	
5170	LOC401093	GEO	
5171	LOC401233	GEO	
5172	LOC401357	GEO	
5173	LOC401522	GEO	
5174	LOC404266	GEO	
5175	LOC439990	GEO	
5176	LOC440173	GEO	
5177	LOC440233	GEO	
5178	LOC440302	GEO	
5179	LOC440338	GEO	
5180	LOC440354	GEO	
5181	LOC440396	GEO	
5182	LOC440459	GEO	
5183	LOC440589	GEO	
5184	LOC440607	GEO	
5185	LOC440838	GEO	
5186	LOC440896	GEO	
5187	LOC441124	GEO	
5188	LOC441282	GEO	
5189	LOC441426	GEO	
5190	LOC441644	GEO	
5191	LOC441775	GEO	
5192	LOC442270	GEO	
5193	LOC51233	GEO	
5194	LOC541469	GEO	
5195	LOC541471	GEO	
5196	LOC54492	GEO	
5197	LOC553137	GEO	
5198	LOC56920	GEO	

5199	LOC572558	GEO	
5200	LOC595101	GEO	
5201	LOC63928	GEO	
5202	LOC642103	GEO	
5203	LOC642826	GEO	
5204	LOC642869	GEO	
5205	LOC643014	GEO	
5206	LOC643072	GEO	
5207	LOC643100	GEO	
5208	LOC643201	GEO	
5209	LOC643220	GEO	
5210	LOC643792	GEO	
5211	LOC643940	GEO	
5212	LOC644090	GEO	
5213	LOC644192	GEO	
5214	LOC644242	GEO	
5215	LOC644246	GEO	
5216	LOC644538	GEO	
5217	LOC644662	GEO	
5218	LOC644768	GEO	
5219	LOC644774	GEO	
5220	LOC645195	GEO	
5221	LOC645249	GEO	
5222	LOC645323	GEO	
5223	LOC645381	GEO	
5224	LOC646014	GEO	
5225	LOC646168	GEO	
5226	LOC646626	GEO	
5227	LOC646627	GEO	
5228	LOC646652	GEO	
5229	LOC646701	GEO	
5230	LOC646851	GEO	
5231	LOC647000	GEO	
5232	LOC647500	GEO	
5233	LOC647954	GEO	
5234	LOC647979	GEO	
5235	LOC648541	GEO	
5236	LOC648795	GEO	
5237	LOC650331	GEO	
5238	LOC650794	GEO	
5239	LOC651255	GEO	
5240	LOC652005	GEO	
5241	LOC653198	GEO	
5242	LOC653391	GEO	
5243	LOC653483	GEO	
5244	LOC653602	GEO	
5245	LOC727770	GEO	
5246	LOC727820	GEO	
5247	LOC727869	GEO	
5248	LOC727916	GEO	

5249	LOC727924	GEO	
5250	LOC727944	GEO	
5251	LOC727995	GEO	
5252	LOC728047	GEO	
5253	LOC728153	GEO	
5254	LOC728264	GEO	
5255	LOC728377	GEO	
5256	LOC728392	GEO	
5257	LOC728448	GEO	
5258	LOC728449	GEO	
5259	LOC728725	GEO	
5260	LOC728769	GEO	
5261	LOC728897	GEO	
5262	LOC728903	GEO	
5263	LOC729088	GEO	
5264	LOC729680	GEO	
5265	LOC729852	GEO	
5266	LOC729983	GEO	
5267	LOC730102	GEO	
5268	LOC730286	GEO	
5269	LOC730755	GEO	
5270	LOC731049	GEO	
5271	LOC731275	GEO	
5272	LOC731404	GEO	
5273	LOC731966	GEO	
5274	LOC81691	GEO	
5275	LOC90835	GEO	
5276	LOC91316	GEO	
5277	LOC92482	GEO	
5278	LOC96610	GEO	
5279	LOH12CR1	GEO	
5280	LONP1	GEO	
5281	LONP2	GEO	
5282	LONRF2	GEO	
5283	LONRF3	GEO	
5284	LPCAT2	GEO	
5285	LPGAT1	GEO	
5286	LPHN1	GEO	
5287	LPHN2	GEO	
5288	LPIN1	GEO	
5289	LPIN2	GEO	
5290	LPL	Both	
5291	LPP	GEO	
5292	LQK1	GEO	
5293	LRCH2	GEO	
5294	LRCH4	GEO	
5295	LRFN4	GEO	
5296	LRMP	GEO	
5297	LRP10	GEO	
5298	LRP2	GEO	

5299	LRP3	GEO	
5300	LRP5	GEO	
5301	LRPAP1	GEO	
5302	LRRC1	GEO	
5303	LRRC16A	GEO	
5304	LRRC2	GEO	
5305	LRRC27	GEO	
5306	LRRC31	GEO	
5307	LRRC32	GEO	
5308	LRRC3B	GEO	
5309	LRRC56	GEO	
5310	LRRC57	GEO	
5311	LRRC58	GEO	
5312	LRRC6	GEO	
5313	LRRC66	GEO	
5314	LRRC8C	GEO	
5315	LRRFIP1	GEO	
5316	LRRFIP2	GEO	
5317	LRRK2	Both	
5318	LRRN4	GEO	
5319	LRRN5	GEO	
5320	LRTM1	GEO	
5321	LSM11	GEO	
5322	LSM6	GEO	
5323	LSP1	GEO	
5324	LST1	GEO	
5325	LTA	Both	
5326	LTA4H	GEO	
5327	LTB	GEO	
5328	LTB4DH	GEO	
5329	LTB4R	GEO	
5330	LTBP1	GAD	
5331	LTBP3	GEO	
5332	LTBP4	Both	
5333	LTK	GEO	
5334	LTN1	GEO	
5335	LUC7L2	GEO	
5336	LUM	GEO	
5337	LUZP1	GEO	
5338	LY6E	GEO	
5339	LY6K	GEO	
5340	LY9	GEO	
5341	LYG1	GEO	
5342	LYN	GEO	
5343	LYNX1	GEO	
5344	LYPD5	GEO	
5345	LYPLAL1	GEO	
5346	LYRM1	GEO	
5347	LYRM2	GEO	
5348	LYRM4	GAD	

5349	LYRM5	GEO	
5350	LYSMD2	GEO	
5351	LYST	GEO	
5352	LYVE1	GEO	
5353	LZTS1	GEO	
5354	M6PRBP1	GEO	
5355	MACF1	GEO	
5356	MACROD1	GEO	
5357	MAD1L1	GEO	
5358	MAD2L1	GEO	
5359	MADCAM1	GEO	
5360	MADH3	GEO	
5361	MAEL	GEO	
5362	MAFB	GEO	
5363	MAFF	GEO	
5364	MAFG	GEO	
5365	MAFK	GEO	
5366	MAG	GEO	
5367	MAGEA1	GEO	
5368	MAGEA12	GEO	
5369	MAGEA2	GEO	
5370	MAGEA3	GEO	
5371	MAGEA4	GEO	
5372	MAGEA6	GEO	
5373	MAGEB2	GEO	
5374	MAGED1	GEO	
5375	MAGED2	GEO	
5376	MAGED4	GEO	
5377	MAGEL2	GEO	
5378	MAGI2	Both	
5379	MAGOH	GEO	
5380	MAGT1	GEO	
5381	MAK	GEO	
5382	MAL	GEO	
5383	MAL2	GEO	
5384	MALT1	GEO	
5385	MAMLD1	GEO	
5386	MAN1C1	GEO	
5387	MAN2B2	GEO	
5388	MAN2C1	GEO	
5389	MANBA	Both	
5390	MANSC1	GEO	
5391	MAP1A	GEO	
5392	MAP2	GEO	
5393	MAP2K1	GEO	
5394	MAP2K3	GEO	
5395	MAP2K4	GAD	
5396	MAP2K5	GEO	
5397	MAP3K3	GEO	
5398	MAP3K4	GEO	

5399	MAP3K7	Both	
5400	MAP3K7IP1	GAD	
5401	MAP4	GEO	
5402	MAP7	GEO	
5403	MAP7D2	GEO	
5404	MAPK11	GEO	
5405	MAPK13	GEO	
5406	MAPK8	GEO	
5407	MAPK8IP1	GEO	
5408	MAPK8IP3	GEO	
5409	MAPK9	GEO	
5410	MAPKAPK2	GEO	
5411	MAPRE2	Both	
5412	MAPT	GEO	
5413	MARCH3	GEO	
5414	MARCH5	GEO	
5415	MARCKS	GEO	
5416	MARCKSL1	GEO	
5417	MARK1	GEO	
5418	MARK3	GEO	
5419	MARVELD1	GEO	
5420	MARVELD3	GEO	
5421	MASP2	GAD	
5422	MAST4	GEO	
5423	MAT2A	Both	
5424	MATN1	GEO	
5425	MAU2	GEO	
5426	MB	GEO	
5427	MBD1	GEO	
5428	MBD2	GEO	
5429	MBD4	GEO	
5430	MBD5	GEO	
5431	MBIP	GEO	
5432	MBL2	GAD	
5433	MBNL2	GEO	
5434	MBP	GEO	
5435	MBTD1	GEO	
5436	MC4R	GAD	
5437	MC5R	GEO	
5438	MCART1	GEO	
5439	MCCC1	GEO	
5440	MCFD2	GEO	
5441	MCL1	GEO	
5442	MCM2	GEO	
5443	MCM3	GEO	
5444	MCM3AP	GEO	
5445	MCM4	GEO	
5446	MCM7	GEO	
5447	MCM8	GEO	
5448	MCOLN2	GEO	

5449	MCOLN3	GEO	
5450	MCPH1	GEO	
5451	MCTP1	GEO	
5452	MCTP2	GEO	
5453	MDFI	GEO	
5454	MDFIC	GEO	
5455	MDGA1	GEO	
5456	MDH1	GEO	
5457	MDK	Both	
5458	MDM2	Both	
5459	MDM4	GEO	
5460	MDS025	GEO	
5461	MDS1	GEO	
5462	ME1	GEO	
5463	ME3	GEO	
5464	MEAF6	GEO	
5465	MECOM	GEO	
5466	MED1	GEO	
5467	MED10	GEO	
5468	MED12L	GEO	
5469	MED21	GEO	
5470	MED25	GEO	
5471	MED30	GEO	
5472	MEF2A	GEO	
5473	MEF2D	GEO	
5474	MEFV	GAD	
5475	MEG3	GEO	
5476	MEGF6	GEO	
5477	MEGF9	GEO	
5478	MEIS3P1	GEO	
5479	MEOX2	GEO	
5480	MESDC1	GEO	
5481	MESP2	GEO	
5482	METTL10	GEO	
5483	METTL11A	GEO	
5484	METTL13	GEO	
5485	METTL2A	GEO	
5486	METTL9	GEO	
5487	MEX3A	GEO	
5488	MEX3D	GEO	
5489	MFAP4	GEO	
5490	MFAP5	GEO	
5491	MFGE8	GEO	
5492	MFI2	GEO	
5493	MFSD10	GEO	
5494	MFSD4	GEO	
5495	MGA	GEO	
5496	MGAM	GEO	
5497	MGAT1	GEO	
5498	MGC10233	GEO	

5499	MGC10981	GEO	
5500	MGC11134	GEO	
5501	MGC11242	GEO	
5502	MGC12966	GEO	
5503	MGC13053	GEO	
5504	MGC16384	GEO	
5505	MGC21881	GEO	
5506	MGC22793	GEO	
5507	MGC23985	GEO	
5508	MGC24975	GEO	
5509	MGC29506	GEO	
5510	MGC3207	GEO	
5511	MGC3234	GEO	
5512	MGC33486	GEO	
5513	MGC4171	GEO	
5514	MGC4294	GEO	
5515	MGC4308	GEO	
5516	MGC45438	GEO	
5517	MGC45800	GEO	
5518	MGC48628	GEO	
5519	MGC57346	GEO	
5520	MGEA5	GEO	
5521	MGMT	GAD	
5522	MGP	GEO	
5523	MGST1	Both	
5524	MGST3	GEO	
5525	MHC	GAD	
5526	MIA	GEO	
5527	MIA2	GEO	
5528	MIB1	GEO	
5529	MIB2	GEO	
5530	MICA	GAD	
5531	MICALL1	GEO	
5532	MID1IP1	GEO	
5533	MID2	GEO	
5534	MIER3	GEO	
5535	MIF	Both	
5536	MINK1	GEO	
5537	MIPEP	GEO	
5538	MIR17HG	GEO	
5539	MIR214	GEO	
5540	MIRHG1	GEO	
5541	MKI67	GEO	
5542	MKKS	GEO	
5543	MKX	GEO	
5544	MLEC	GEO	
5545	MLF1	GEO	
5546	MLH1	GAD	
5547	MLH3	GAD	
5548	MLKL	GEO	

5549	MLL	GEO	
5550	MLL2	GEO	
5551	MLL5	GEO	
5552	MLLT4	GEO	
5553	MLLT6	GEO	
5554	MLXIP	GEO	
5555	MLXIPL	GEO	
5556	MMAB	GEO	
5557	MMD2	GAD	
5558	MMP10	GEO	
5559	MMP13	GEO	
5560	MMP17	GEO	
5561	MMP19	GEO	
5562	MMP2	Both	
5563	MMP23A	GEO	
5564	MMP9	Both	
5565	MMRN1	Both	
5566	MMRN2	GEO	
5567	MMS19	GEO	
5568	MN1	GEO	
5569	MNX1	GEO	
5570	MOBKL2C	GEO	
5571	MOBP	GEO	
5572	MOCOS	GAD	
5573	MOGAT2	GEO	
5574	MOGAT3	GEO	
5575	MON2	GEO	
5576	MORF4L2	GEO	
5577	MOSC2	GEO	
5578	MOSPD1	GEO	
5579	MOXD1	GEO	
5580	MPDZ	GEO	
5581	MPHOSPH8	GEO	
5582	MPHOSPH9	GEO	
5583	MPO	GAD	
5584	MPP2	GEO	
5585	MPP4	GEO	
5586	MPP6	GEO	
5587	MPRIP	GEO	
5588	MPV17	GEO	
5589	MPZL1	GEO	
5590	MPZL3	GEO	
5591	MR1	GEO	
5592	MRAP2	GEO	
5593	MRAS	GEO	
5594	MRC2	GEO	
5595	MRE11A	GEO	
5596	MRFAP1	GEO	
5597	MRGPRF	GEO	
5598	MRGX3	GEO	

5599	M-RIP	GEO	
5600	MRLC2	GEO	
5601	MRP63	GEO	
5602	MRPL11	GEO	
5603	MRPL12	GEO	
5604	MRPL13	GEO	
5605	MRPL18	GEO	
5606	MRPL20	GEO	
5607	MRPL21	GEO	
5608	MRPL34	GEO	
5609	MRPL35	GEO	
5610	MRPL39	GEO	
5611	MRPS10	GEO	
5612	MRPS17	GEO	
5613	MRPS18C	GEO	
5614	MRPS22	GEO	
5615	MRPS23	GEO	
5616	MRPS31	GEO	
5617	MRPS35	GEO	
5618	MRPS5	GEO	
5619	MRPS6	GEO	
5620	MRVI1	GEO	
5621	MS	GAD	
5622	MS4A4A	GEO	
5623	MS4A5	GEO	
5624	MS4A6A	GEO	
5625	MS4A8B	GEO	
5626	MSH2	GAD	
5627	MSH3	Both	
5628	MSH6	GAD	
5629	MSI1	GEO	
5630	MSI2	GEO	
5631	MSL1	GEO	
5632	MSMB	GEO	
5633	MSN	GEO	
5634	MSRB2	GEO	
5635	MSRB3	GEO	
5636	MST1	GAD	
5637	MST150	GEO	
5638	MST1R	GAD	
5639	MST4	GEO	
5640	MSTO2P	GEO	
5641	MSX1	GEO	
5642	MT1G	GEO	
5643	MT1JP	GEO	
5644	MT1L	GEO	
5645	MT1P2	GEO	
5646	MT4	GEO	
5647	MTA2	GEO	
5648	MTCH1	GEO	

5649	MTCH2	GEO	
5650	MT-CO1	GAD	
5651	MT-CO2	GAD	
5652	MTDH	GEO	
5653	MTERFD1	GEO	
5654	MTERFD2	GEO	
5655	MTHFD1	Both	
5656	MTHFD2L	GEO	
5657	MTHFR	GAD	
5658	MTHFS	GAD	
5659	MTHFSD	GEO	
5660	MTL5	GEO	
5661	MTMR1	GEO	
5662	MTMR10	GEO	
5663	MTMR11	GEO	
5664	MTMR2	GEO	
5665	MTMR3	GAD	
5666	MTMR6	GEO	
5667	MTMR7	GEO	
5668	MTR	Both	
5669	MTRF1L	GEO	
5670	MTRR	Both	
5671	MTSS1	GEO	
5672	MTTP	GEO	
5673	MTUS1	GEO	
5674	MTUS2	GEO	
5675	MTX3	GEO	
5676	MUC1	GEO	
5677	MUC12	GEO	
5678	MUC17	GEO	
5679	MUC19	GAD	
5680	MUC2	GEO	
5681	MUC20	GEO	
5682	MUC3A	Both	
5683	MUC3B	GEO	
5684	MUC4	GEO	
5685	MUC5AC	GEO	
5686	MUC5B	GEO	
5687	MUCDHL	GEO	
5688	MUPCDH	GEO	
5689	MUSTN1	GEO	
5690	MUTYH	GAD	
5691	MVP	GEO	
5692	MXD3	GEO	
5693	MXI1	GEO	
5694	MYADM	GEO	
5695	MYB	GEO	
5696	MYBBP1A	GEO	
5697	MYBL1	GEO	
5698	MYBL2	GEO	

5699	MYBPC1	GEO	
5700	MYCBP2	GEO	
5701	MYCL1	GAD	
5702	MYEOV	GEO	
5703	MYEOV2	GEO	
5704	MYH1	GAD	
5705	MYH10	GEO	
5706	MYH14	GEO	
5707	MYL10	GEO	
5708	MYL4	GEO	
5709	MYL6	GEO	
5710	MYL6B	GEO	
5711	MYLK2	GAD	
5712	MYO10	GEO	
5713	MYO15B	GEO	
5714	MYO19	GEO	
5715	MYO1C	GEO	
5716	MYO1D	GEO	
5717	MYO1H	GEO	
5718	MYO3A	GAD	
5719	MYO7B	GEO	
5720	MYO9B	GAD	
5721	MYOCD	GEO	
5722	MYOF	GEO	
5723	MYOZ2	GEO	
5724	MYST3	GEO	
5725	MYT1	GEO	
5726	MZT2A	GEO	
5727	MZT2B	GEO	
5728	N4BP2	GEO	
5729	N4BP2L2	GEO	
5730	N4BP3	GEO	
5731	NA	GAD	
5732	NAA15	GEO	
5733	NAALADL1	GEO	
5734	NAALADL2	GEO	
5735	NAB1	GEO	
5736	NACAD	GEO	
5737	NACAP1	GEO	
5738	NACC1	GEO	
5739	NADK	GEO	
5740	NAGK	GEO	
5741	NAGS	GEO	
5742	NAIP	GEO	
5743	NALCN	GEO	
5744	NANS	GEO	
5745	NAP1L3	GEO	
5746	NAP1L4	GEO	
5747	NAPEPLD	GEO	
5748	NAPRT1	GEO	

5749	NAPSB	GEO	
5750	NARG2	GEO	
5751	NAT1	GAD	
5752	NAT12	GEO	
5753	NAT2	Both	
5754	NAT8	GEO	
5755	NAV2	GEO	
5756	NAV3	GEO	
5757	NBEAL2	GEO	
5758	NBLA00301	GEO	
5759	NBN	Both	
5760	NBPF3	GEO	
5761	NCALD	GEO	
5762	NCAM2	GEO	
5763	NCAPG2	GEO	
5764	NCAPH	GEO	
5765	NCBP1	GEO	
5766	NCEH1	GEO	
5767	NCF1	GEO	
5768	NCF2	GEO	
5769	NCF4	Both	
5770	NCL	GEO	
5771	NCOA2	GEO	
5772	NCOA3	GEO	
5773	NCOA4	GEO	
5774	NCOA7	GEO	
5775	NCOR1	GEO	
5776	NCOR2	GEO	
5777	NCRNA00081	GEO	
5778	NCRNA00152	GEO	
5779	NCRNA00161	GEO	
5780	NCRNA00182	GEO	
5781	NCRNA00185	GEO	
5782	NCRNA00238	GEO	
5783	NCRNA00253	GEO	
5784	NCRNA00260	GEO	
5785	NCRNA00261	GEO	
5786	NCRNA00286A	GEO	
5787	NCRNA00294	GEO	
5788	NCS1	GEO	
5789	ND1	GAD	
5790	ND2	GAD	
5791	ND3	Both	
5792	ND4	GAD	
5793	ND4L	GAD	
5794	ND5	GAD	
5795	ND6	GAD	
5796	NDE1	GEO	
5797	NDFIP2	GEO	
5798	NDRG1	GAD	

5799	NDRG4	GEO	
5800	NDST3	GAD	
5801	NDUFA10	GEO	
5802	NDUFA11	GEO	
5803	NDUFAB1	GAD	
5804	NDUFAF4	GEO	
5805	NDUFB8	GEO	
5806	NDUFS3	GEO	
5807	NDUFS4	GEO	
5808	NDUFV1	GEO	
5809	NEAT1	GEO	
5810	NEB	GEO	
5811	NEDD8	GEO	
5812	NEFH	GEO	
5813	NEIL1	Both	
5814	NEIL2	GAD	
5815	NEIL3	Both	
5816	NEK1	GEO	
5817	NEK11	GEO	
5818	NEK3	GEO	
5819	NEK9	GEO	
5820	NELF	GEO	
5821	NELL1	GAD	
5822	NELL2	GEO	
5823	NENF	GEO	
5824	NEO1	GEO	
5825	NES	GEO	
5826	NETO2	GEO	
5827	NEU4	GEO	
5828	NEURL1B	GEO	
5829	NEURL2	GEO	
5830	NEURL3	GEO	
5831	NEUROG3	GEO	
5832	NF1	GEO	
5833	NFATC1	GEO	
5834	NFATC2IP	GEO	
5835	NFE2	GEO	
5836	NFE2L2	GAD	
5837	NFIC	GEO	
5838	NFKB1	GAD	
5839	NFKBIA	Both	
5840	NFKBIB	GEO	
5841	NFKBIE	GEO	
5842	NFKBIL1	GAD	
5843	NFKBIZ	GEO	
5844	NFS1	GEO	
5845	NFX1	GEO	
5846	NFXL1	GEO	
5847	NFYB	GEO	
5848	NGDN	GEO	

5849	NGEF	GEO	
5850	NGFR	GEO	
5851	NGFRAP1	GEO	
5852	NGLY1	GEO	
5853	NGRN	GEO	
5854	NHEDC2	GEO	
5855	NHLRC2	GEO	
5856	NHP2	GEO	
5857	NHP2L1	GEO	
5858	NHSL1	GEO	
5859	NICN1	GAD	
5860	NID1	GEO	
5861	NINJ1	GEO	
5862	NINJ2	GEO	
5863	NINL	GEO	
5864	NIPAL2	GEO	
5865	NIPAL3	GEO	
5866	NIPSNAP1	GEO	
5867	NIT2	GEO	
5868	NKAP	GAD	
5869	NKIRAS1	GEO	
5870	NKTR	GEO	
5871	NKX2	GAD	
5872	NKX2-3	Both	
5873	NKX3	GAD	
5874	NLGN3	GEO	
5875	NLGN4X	GEO	
5876	NLGN4Y	GEO	
5877	NLK	GAD	
5878	NLRC3	GEO	
5879	NLRC5	GEO	
5880	NLRP1	Both	
5881	NLRP10	GAD	
5882	NLRP11	Both	
5883	NLRP12	GAD	
5884	NLRP13	GAD	
5885	NLRP14	GAD	
5886	NLRP3	GAD	
5887	NLRP4	GAD	
5888	NLRP5	GAD	
5889	NLRP6	GAD	
5890	NLRP7	GAD	
5891	NLRP8	GAD	
5892	NLRP9	GAD	
5893	NME1	GAD	
5894	NME1-NME2	GEO	
5895	NME3	GEO	
5896	NME4	GEO	
5897	NME7	GEO	
5898	NMES1	GEO	

5899	NMNAT3	GEO	
5900	NMT2	GEO	
5901	NNAT	GEO	
5902	NNT	GEO	
5903	NOB1	GEO	
5904	NOC2L	GEO	
5905	NOD1	GAD	
5906	NODAL	GEO	
5907	NOL3	GEO	
5908	NOL5A	GEO	
5909	NOL6	GEO	
5910	NOL8	GEO	
5911	NOL9	GEO	
5912	NOLC1	GEO	
5913	NOP16	GEO	
5914	NOP2	GEO	
5915	NOP5/NOP58	GEO	
5916	NOS1	Both	
5917	NOS2	GEO	
5918	NOS2A	Both	
5919	NOS3	Both	
5920	NOTCH1	GEO	
5921	NOTCH2NL	GEO	
5922	NOTCH3	GEO	
5923	NOTUM	GEO	
5924	NOVA1	GEO	
5925	NOX1	GEO	
5926	NOX4	GEO	
5927	NOXO1	GEO	
5928	NPAS2	GEO	
5929	NPAS3	GEO	
5930	NPC1	GEO	
5931	NPC1L1	GEO	
5932	NPC2	GEO	
5933	NPDC1	GEO	
5934	NPHP1	GEO	
5935	NPIP	GEO	
5936	NPIPL3	GEO	
5937	NPL	GEO	
5938	NPLOC4	GEO	
5939	NPM1	GEO	
5940	NPR1	GEO	
5941	NPR2	GEO	
5942	NPSR1	GAD	
5943	NPTXR	GEO	
5944	NPVF	GEO	
5945	NPW	GEO	
5946	NPY	GEO	
5947	NPY1R	GEO	
5948	NPY6R	GEO	

5949	NQO1	Both	
5950	NQO2	GEO	
5951	NR0B1	GEO	
5952	NR1D1	GEO	
5953	NR1D2	GEO	
5954	NR1H3	GAD	
5955	NR1H4	GEO	
5956	NR1I2	Both	
5957	NR4A1	GEO	
5958	NR6A1	GEO	
5959	NRAMP	GAD	
5960	NRAP	GEO	
5961	NRARP	GEO	
5962	NRAS	Both	
5963	NRBP2	GEO	
5964	NRG1	GEO	
5965	NRG2	GEO	
5966	NRG4	GEO	
5967	NRIP1	GEO	
5968	NRIP3	GEO	
5969	NRK	GEO	
5970	NRN1	GEO	
5971	NRP2	GEO	
5972	NRXN1	GEO	
5973	NSA2	GEO	
5974	NSD1	GEO	
5975	NSFL1C	GEO	
5976	NSG1	GEO	
5977	NSMAF	GEO	
5978	NSUN5P1	GEO	
5979	NSUN7	GEO	
5980	NT5DC1	GEO	
5981	NT5DC2	GEO	
5982	NT5E	Both	
5983	NT5M	GEO	
5984	NTAN1	GEO	
5985	NTHL1	GAD	
5986	NTM	GEO	
5987	NTN1	GEO	
5988	NTN2L	GEO	
5989	NTN4	GEO	
5990	NTN5	GEO	
5991	NTNG2	GEO	
5992	NTRK2	GEO	
5993	NTS	GEO	
5994	NTSR1	GEO	
5995	NUAK1	GEO	
5996	NUB1	GEO	
5997	NUDC	GEO	
5998	NUDCD1	GEO	

5999	NUDCD2	GEO	
6000	NUDT1	Both	
6001	NUDT12	GEO	
6002	NUDT5	GEO	
6003	NUDT6	GEO	
6004	NUDT7	GEO	
6005	NUDT9	GEO	
6006	NUFIP1	GEO	
6007	NUP107	GEO	
6008	NUP205	GEO	
6009	NUP37	GEO	
6010	NUP85	GEO	
6011	NUPL1	GEO	
6012	NUPR1	Both	
6013	NUTF2	GEO	
6014	NXF1	GEO	
6015	NXF3	GEO	
6016	NXN	GEO	
6017	NXPH4	GEO	
6018	OAS1	GEO	
6019	OAS3	GEO	
6020	OAT	GEO	
6021	OAZ2	GEO	
6022	OAZ3	GEO	
6023	OBFC2A	GEO	
6024	OBSL1	GEO	
6025	OCIAD2	GEO	
6026	OCLN	GEO	
6027	OCSP	GEO	
6028	OCTN3	GAD	
6029	ODC1	Both	
6030	ODF3B	GEO	
6031	ODZ2	GEO	
6032	ODZ3	GEO	
6033	OFCC1	GEO	
6034	OGDH	GEO	
6035	OGG1	GAD	
6036	OGN	GEO	
6037	OGT	GEO	
6038	OIP5	GEO	
6039	OIT3	GEO	
6040	OLA1	GEO	
6041	OLFM1	GEO	
6042	OLFM4	GEO	
6043	OLFML3	GEO	
6044	OLIG3	GAD	
6045	OLR1	GEO	
6046	OPCML	GEO	
6047	OPRM1	GAD	
6048	OPTN	GEO	

6049	OR10D1P	GEO	
6050	OR2B3	GEO	
6051	OR2H2	GEO	
6052	OR2S2	GEO	
6053	OR4N4	GEO	
6054	OR51E2	GEO	
6055	OR51I1	GEO	
6056	ORAI2	GEO	
6057	ORF1-FL49	GEO	
6058	ORM1	GEO	
6059	ORM2	GEO	
6060	ORMDL2	GEO	
6061	ORMDL3	GAD	
6062	OS9	GAD	
6063	OSBP2	GEO	
6064	OSGIN1	GEO	
6065	OSMR	GEO	
6066	OSTalpha	GEO	
6067	OSTF1	GEO	
6068	OSTM1	GEO	
6069	OTC	GEO	
6070	OTOP2	GEO	
6071	OTOR	GEO	
6072	OTUB1	GEO	
6073	OTUD3	GAD	
6074	OVOL1	GEO	
6075	OVOL2	GEO	
6076	OXCT1	GEO	
6077	OXGR1	GEO	
6078	OXR1	GEO	
6079	OXTR	GEO	
6080	P2RX1	GEO	
6081	P2RX4	GEO	
6082	P2RX5	GEO	
6083	P2RX7	Both	
6084	P2RY1	GEO	
6085	P2RY12	GEO	
6086	P2RY13	GEO	
6087	P2RY14	GEO	
6088	P4HA2	Both	
6089	PABPN1	GEO	
6090	PACRG	GEO	
6091	PACSIN1	GEO	
6092	PADI1	GEO	
6093	PADI4	GAD	
6094	PAFAH1B1	GEO	
6095	PAFAH1B3	GEO	
6096	PAFAH2	GEO	
6097	PAIP2B	GEO	
6098	PAK1	GEO	

6099	PAK3	GEO	
6100	PAK4	GEO	
6101	PAK6	GEO	
6102	PALB2	GAD	
6103	PALLD	GEO	
6104	PALM	GEO	
6105	PALM3	GEO	
6106	PALMD	GEO	
6107	PAMR1	GEO	
6108	PAN3	GEO	
6109	PANX2	GEO	
6110	PAOX	GEO	
6111	PAQR3	GEO	
6112	PAQR4	GEO	
6113	PAQR6	GEO	
6114	PAQR7	GEO	
6115	PARD3	GEO	
6116	PARD6B	GEO	
6117	PARD6G	GEO	
6118	PARL	GEO	
6119	PARM1	GEO	
6120	PARN	GEO	
6121	PARP1	GAD	
6122	PARP10	GEO	
6123	PARP4	GAD	
6124	PARP9	GEO	
6125	PART1	GEO	
6126	PARVA	GEO	
6127	PARVB	GEO	
6128	PARVG	GEO	
6129	PASD1	GEO	
6130	PASK	GEO	
6131	PATE1	GEO	
6132	PATZ1	GEO	
6133	PAX1	GEO	
6134	PAX2	GEO	
6135	PBRM1	GEO	
6136	PBX1	GEO	
6137	PBX2	GEO	
6138	PCBP3	GEO	
6139	PCCA	GEO	
6140	PCDH1	GEO	
6141	PCDH12	GEO	
6142	PCDH18	GEO	
6143	PCDH19	GEO	
6144	PCDH20	GEO	
6145	PCDH7	GEO	
6146	PCDH9	GEO	
6147	PCDHA1	GEO	
6148	PCDHA4	GEO	

6149	PCDHA6	GEO	
6150	PCDHGA1	GEO	
6151	PCDHGA3	GEO	
6152	PCF11	GEO	
6153	PCGF5	GEO	
6154	PCID2	GEO	
6155	PCM1	GEO	
6156	PCNA	GAD	
6157	PCNX	GEO	
6158	PCNXL3	GEO	
6159	PCP4	GEO	
6160	PCSK1	GEO	
6161	PCSK1N	GEO	
6162	PCSK2	GEO	
6163	PCSK5	GEO	
6164	PCSK7	GEO	
6165	PCSK9	GEO	
6166	PCTP	GEO	
6167	PDAP1	GEO	
6168	PDCD1	Both	
6169	PDCD1LG2	GAD	
6170	PDCD2	GEO	
6171	PDCD2L	GEO	
6172	PDE12	GEO	
6173	PDE1A	GEO	
6174	PDE4A	GEO	
6175	PDE4B	GEO	
6176	PDE4DIP	GEO	
6177	PDE5A	GEO	
6178	PDE6A	GEO	
6179	PDE6B	GEO	
6180	PDE7B	GEO	
6181	PDGFA	GEO	
6182	PDGFC	GEO	
6183	PDGFD	GEO	
6184	PDGFRB	GEO	
6185	PDHA1	GEO	
6186	PDHB	GEO	
6187	PDIA3	GEO	
6188	PDIA4	GEO	
6189	PDK1	Both	
6190	PDK3	GEO	
6191	PDLIM1	Both	
6192	PDLIM2	GEO	
6193	PDLIM4	GEO	
6194	PDLIM5	GEO	
6195	PDLIM7	GEO	
6196	PDP1	GEO	
6197	PDPK1	GEO	
6198	PDPR	GEO	

6199	PDS5A	GEO	
6200	PDSS1	GEO	
6201	PDSS2	GEO	
6202	PDXP	GEO	
6203	PDZD3	GEO	
6204	PDZD7	GEO	
6205	PDZK1	GEO	
6206	PDZK1IP1	GEO	
6207	PDZK1P1	GEO	
6208	PDZRN3	GEO	
6209	PDZRN4	GEO	
6210	PEA15	GEO	
6211	PEAR1	GEO	
6212	PEBP1	GEO	
6213	PEBP4	GEO	
6214	PECAM1	Both	
6215	PECR	GEO	
6216	PEF1	GEO	
6217	PEG3	GEO	
6218	PELO	GEO	
6219	PEO1	GEO	
6220	PER1	GEO	
6221	PER2	GEO	
6222	PER3	GEO	
6223	PET112L	GEO	
6224	PEX11A	GEO	
6225	PEX12	GEO	
6226	PEX13	GEO	
6227	PEX2	GEO	
6228	PEX26	GEO	
6229	PFAS	GEO	
6230	PFDN2	GEO	
6231	PFDN4	GEO	
6232	PFDN5	GEO	
6233	PFKFB1	GEO	
6234	PFKFB3	GEO	
6235	PFKFB4	GEO	
6236	PFKP	GEO	
6237	PGAM1	GEO	
6238	PGAM2	GEO	
6239	PGAP1	GEO	
6240	PGBD1	GEO	
6241	PGBD5	GEO	
6242	PGCP	GEO	
6243	PGDS	GEO	
6244	PGK2	GEO	
6245	PGM1	GEO	
6246	PGM2	GEO	
6247	PGM5P2	GEO	
6248	PGR	GAD	

6249	PHACTR1	GEO	
6250	PHC2	GEO	
6251	PHC3	GEO	
6252	PHEX	GEO	
6253	PHF11	GEO	
6254	PHF13	GEO	
6255	PHF14	GEO	
6256	PHF15	GEO	
6257	PHF2	GEO	
6258	PHF20L1	GEO	
6259	PHF6	GEO	
6260	PHF7	GEO	
6261	PHGR1	GEO	
6262	PHIP	GEO	
6263	PHKA1	GEO	
6264	PHKA2	GEO	
6265	PHKB	GEO	
6266	PHLDA2	GEO	
6267	PHLDA3	GEO	
6268	PHLPPL	GEO	
6269	PHOX2B	GAD	
6270	PHPT1	GEO	
6271	PHTF1	GEO	
6272	PHTF2	GEO	
6273	PI15	GEO	
6274	PI16	GEO	
6275	PI3	GEO	
6276	PI4K2B	GEO	
6277	PIAS1	GEO	
6278	PIAS3	GEO	
6279	PIBF1	GEO	
6280	PICALM	GEO	
6281	PICK1	GEO	
6282	PIGG	GEO	
6283	PIGH	GEO	
6284	PIGL	GEO	
6285	PIGN	GEO	
6286	PIGQ	GEO	
6287	PIGS	GEO	
6288	PIGU	GEO	
6289	PIGZ	GEO	
6290	PIK3C2A	GEO	
6291	PIK3C2B	GEO	
6292	PIK3C3	GEO	
6293	PIK3CA	GAD	
6294	PIK3R2	GEO	
6295	PIK3R4	GEO	
6296	PIK3R5	GEO	
6297	PIM1	GEO	
6298	PIM2	GEO	

6299	PINK1	GEO	
6300	PION	GEO	
6301	PIP5K1A	GEO	
6302	PIP5K1B	GEO	
6303	PIP5K1C	GEO	
6304	PIPOX	GEO	
6305	PIR	GEO	
6306	PITPNB	GEO	
6307	PITPNC1	GEO	
6308	PITPNM2	GEO	
6309	PITPNM3	GEO	
6310	PITX2	GEO	
6311	PIWIL1	GEO	
6312	PIWIL4	GEO	
6313	PKD1	GEO	
6314	PKD1L3	GEO	
6315	PKD1P1	GEO	
6316	PKD2	GEO	
6317	PKDCC	GEO	
6318	PKHD1L1	GEO	
6319	PKIA	GEO	
6320	PKIG	GEO	
6321	PKLR	GEO	
6322	PKM2	GEO	
6323	PKN1	GEO	
6324	PKN2	GEO	
6325	PKNOX1	GEO	
6326	PKNOX2	GEO	
6327	PKP1	GEO	
6328	PKP2	GEO	
6329	PKP4	GEO	
6330	PLA1A	GEO	
6331	PLA2G12B	GEO	
6332	PLA2G2A	Both	
6333	PLA2G2D	GEO	
6334	PLA2G2E	GAD	
6335	PLA2G3	GEO	
6336	PLA2G6	GEO	
6337	PLA2G7	Both	
6338	PLA2R1	GEO	
6339	PLAB	GAD	
6340	PLAC1	GEO	
6341	PLAC2	GEO	
6342	PLAC4	GEO	
6343	PLAGL2	GEO	
6344	PLAT	Both	
6345	PLBD1	GEO	
6346	PLCB4	GEO	
6347	PLCD1	GEO	
6348	PLCG2	GEO	

6349	PLCH1	GEO	
6350	PLCL1	GEO	
6351	PLCL3	GEO	
6352	PLCXD2	GEO	
6353	PLCXD3	GEO	
6354	PLD2	GAD	
6355	PLD6	GEO	
6356	PLEC	GEO	
6357	PLEK	GEO	
6358	PLEK2	GEO	
6359	PLEKHA4	GEO	
6360	PLEKHA6	GEO	
6361	PLEKHA7	GEO	
6362	PLEKHG1	GEO	
6363	PLEKHG2	GEO	
6364	PLEKHG4	GEO	
6365	PLEKHG5	GEO	
6366	PLEKHH1	GEO	
6367	PLEKHJ1	GEO	
6368	PLEKHO1	GEO	
6369	PLIN	GEO	
6370	PLIN1	GEO	
6371	PLIN2	GEO	
6372	PLIN4	GEO	
6373	PLIN5	GEO	
6374	PLK1	GEO	
6375	PLK2	GEO	
6376	PLN	GEO	
6377	PLOD3	GEO	
6378	PLS3	GEO	
6379	PLSCR1	GEO	
6380	PLSCR4	GEO	
6381	PLTP	GEO	
6382	PLVAP	GEO	
6383	PLXDC1	GEO	
6384	PLXNA1	GEO	
6385	PLXNA2	GEO	
6386	PLXNB1	GEO	
6387	PLXND1	GEO	
6388	PM20D2	GEO	
6389	PMFBP1	GEO	
6390	PMP2	GEO	
6391	PMP22	GEO	
6392	PMS1	GAD	
6393	PMS2	GAD	
6394	PMS2CL	GEO	
6395	PNCK	GEO	
6396	PNKP	GAD	
6397	PNLIPRP2	GEO	
6398	PNMA1	GEO	

6399	PNMA2	GEO	
6400	PNMAL1	GEO	
6401	PNN	GEO	
6402	PNP	GEO	
6403	PNPLA3	GEO	
6404	PNPLA6	GEO	
6405	PNPLA8	GEO	
6406	PNPT1	GEO	
6407	POFUT1	GEO	
6408	POFUT2	GAD	
6409	POLA1	GEO	
6410	POLB	GAD	
6411	POLI	GEO	
6412	POLN	GEO	
6413	POLQ	GEO	
6414	POLR1C	GEO	
6415	POLR1D	GEO	
6416	POLR2B	GEO	
6417	POLR2E	GEO	
6418	POLR2I	GEO	
6419	POLR2J2	GEO	
6420	POLR2L	GEO	
6421	POLR3F	GEO	
6422	POLR3G	GEO	
6423	POM121	GEO	
6424	POMC	GAD	
6425	POMP	GEO	
6426	POMT2	GEO	
6427	PON1	GAD	
6428	PON2	GAD	
6429	PON3	GEO	
6430	POPDC2	GEO	
6431	POPDC3	GEO	
6432	POR	GEO	
6433	PORCN	GEO	
6434	POU2AF1	Both	
6435	POU2F1	GAD	
6436	POU5FIP1	GAD	
6437	POU6F1	GEO	
6438	pp9099	GEO	
6439	PPA1	GEO	
6440	PPAP2B	GEO	
6441	PPARA	GEO	
6442	PPARD	GAD	
6443	PPARG	Both	
6444	PPARGC1A	Both	
6445	PPFIA1	GEO	
6446	PPFIA3	GEO	
6447	PPFIBP1	GEO	
6448	PPHLN1	GEO	

6449	PPIA	GEO	
6450	PPIB	GEO	
6451	PPIEL	GEO	
6452	PPIG	GEO	
6453	PPIH	GEO	
6454	PPIL3	GEO	
6455	PPM1F	GEO	
6456	PPM1M	GEO	
6457	PPP1CB	GEO	
6458	PPP1R12B	GEO	
6459	PPP1R12C	GEO	
6460	PPP1R13B	GEO	
6461	PPP1R13L	GAD	
6462	PPP1R14A	GEO	
6463	PPP1R14D	GEO	
6464	PPP1R15A	GEO	
6465	PPP1R1B	GEO	
6466	PPP1R3A	GAD	
6467	PPP1R3B	GEO	
6468	PPP1R3C	GEO	
6469	PPP1R3D	GEO	
6470	PPP1R9A	GEO	
6471	PPP2CB	GEO	
6472	PPP3CA	GEO	
6473	PPP4C	GEO	
6474	PPP4R1L	GEO	
6475	PPP4R4	GEO	
6476	PPP5C	GEO	
6477	PPP6R3	GEO	
6478	PPRC1	GEO	
6479	PPTC7	GEO	
6480	PPY	GEO	
6481	PQLC1	GEO	
6482	PRAC	GEO	
6483	PRAP1	GEO	
6484	PRB4	GEO	
6485	PRDM1	Both	
6486	PRDM11	GEO	
6487	PRDM16	GEO	
6488	PRDM2	GAD	
6489	PRDM8	GEO	
6490	PRDX4	GEO	
6491	PRELP	GEO	
6492	PREPL	GEO	
6493	PREX1	GEO	
6494	PREX2	GEO	
6495	PRF1	GEO	
6496	PRH2	GEO	
6497	PRIC285	GEO	
6498	PRICKLE4	GEO	

6499	PRIMA1	GEO	
6500	PRKAA1	GAD	
6501	PRKAA2	GEO	
6502	PRKAB1	GEO	
6503	PRKAG2	GAD	
6504	PRKAR1A	GEO	
6505	PRKAR2A	GEO	
6506	PRKCA	GEO	
6507	PRKCDBP	GEO	
6508	PRKCI	GEO	
6509	PRKD2	GEO	
6510	PRKD3	GEO	
6511	PRKDC	GEO	
6512	PRKG2	GEO	
6513	PRKX	GEO	
6514	PRLR	GEO	
6515	PRMT3	GEO	
6516	PRNP	Both	
6517	PROC	GEO	
6518	PROCR	GEO	
6519	PRODH	GEO	
6520	PROK2	GEO	
6521	PROM1	GEO	
6522	PROM2	GEO	
6523	PRORSD1P	GEO	
6524	PROS1	GEO	
6525	ProSAPiP1	GEO	
6526	PROSC	GEO	
6527	PRPF6	GEO	
6528	PRPS2	GEO	
6529	PRR11	GEO	
6530	PRR15	GEO	
6531	PRR16	GEO	
6532	PRR5	GEO	
6533	PRR7	GEO	
6534	PRRC1	GEO	
6535	PRRG3	GEO	
6536	PRRG4	GEO	
6537	PRRT1	GEO	
6538	PRRT2	GEO	
6539	PRRT3	GEO	
6540	PRRX1	GEO	
6541	PRSS1	GEO	
6542	PRSS12	GEO	
6543	PRSS2	GEO	
6544	PRSS36	GEO	
6545	PRSS37	GEO	
6546	PRSS54	GEO	
6547	PRSS8	GEO	
6548	PRUNE2	GEO	

6549	PSAP	GEO	
6550	PSD2	GEO	
6551	PSD3	GEO	
6552	PSD4	GEO	
6553	PSG2	GEO	
6554	PSG4	GEO	
6555	PSG9	GEO	
6556	PSKH2	GEO	
6557	PSMA1	GEO	
6558	PSMA7	GEO	
6559	PSMB1	GEO	
6560	PSMB3	GEO	
6561	PSMB4	GEO	
6562	PSMB6	GEO	
6563	PSMB7	GEO	
6564	PSMB8	GEO	
6565	PSMB9	Both	
6566	PSMC4	GEO	
6567	PSMD12	GEO	
6568	PSMD13	GEO	
6569	PSMD3	GEO	
6570	PSMD4	GEO	
6571	PSMD5	GEO	
6572	PSMD8	GEO	
6573	PSME4	GEO	
6574	PSMG1	Both	
6575	PSMG4	GEO	
6576	PSORS1C1	GAD	
6577	PSPH	GEO	
6578	PSRC1	GAD	
6579	PTAR1	GEO	
6580	PTBP1	GEO	
6581	PTCD1	GEO	
6582	PTCH	GAD	
6583	PTCHD1	GEO	
6584	PTD012	GEO	
6585	PTEN	Both	
6586	PTGER4	Both	
6587	PTGFR	GEO	
6588	PTGR1	GEO	
6589	PTGS1	Both	
6590	PTGS2	Both	
6591	PTK2	GEO	
6592	PTK2B	GEO	
6593	PTK6	GEO	
6594	PTK7	GEO	
6595	PTMA	GEO	
6596	PTMS	GEO	
6597	PTN	GEO	
6598	PTOV1	GEO	

6599	PTPLAD1	GEO	
6600	PTPLAD2	GEO	
6601	PTPN11	Both	
6602	PTPN13	Both	
6603	PTPN18	GEO	
6604	PTPN2	GAD	
6605	PTPN21	Both	
6606	PTPN22	Both	
6607	PTPN5	GAD	
6608	PTPRC	Both	
6609	PTPRE	GEO	
6610	PTPRJ	GAD	
6611	PTPRK	GEO	
6612	PTPRM	GEO	
6613	PTPRN2	GEO	
6614	PTPRO	GEO	
6615	PTPRS	Both	
6616	PTPRT	GAD	
6617	PTPRU	GEO	
6618	PTPRZ1	GEO	
6619	PTS	GEO	
6620	PTTG1	GEO	
6621	PTTG1IP	GEO	
6622	PTX3	GEO	
6623	PURA	GEO	
6624	PUS10	GAD	
6625	PVRL1	GEO	
6626	PVRL2	GEO	
6627	PVRL3	GEO	
6628	PVRL4	GEO	
6629	PWP1	GEO	
6630	PWWP2B	GEO	
6631	PXDN	GEO	
6632	PXMP2	GEO	
6633	PXMP4	GEO	
6634	PYCR1	GEO	
6635	PYCR2	GEO	
6636	PYGB	GEO	
6637	PYGM	GEO	
6638	PYGO1	GAD	
6639	Q8NBX4	GEO	
6640	QKI	GEO	
6641	QPCTL	GEO	
6642	QRICH2	GEO	
6643	QSER1	GEO	
6644	QTRT1	GEO	
6645	RAB11FIP2	GEO	
6646	RAB11FIP3	GEO	
6647	RAB12	GEO	
6648	RAB14	GEO	

6649	RAB15	GEO	
6650	RAB18	GEO	
6651	RAB22A	GEO	
6652	RAB24	GEO	
6653	RAB26	GEO	
6654	RAB27A	GEO	
6655	RAB28	GEO	
6656	RAB31	GEO	
6657	RAB36	GEO	
6658	RAB38	GEO	
6659	RAB39B	GEO	
6660	RAB3B	GEO	
6661	RAB3D	GEO	
6662	RAB3GAP1	GEO	
6663	RAB3IL1	GEO	
6664	RAB40B	GEO	
6665	RAB6B	GEO	
6666	RAB6IP1	GEO	
6667	RAB7B	GEO	
6668	RAB8A	GEO	
6669	RAB9A	GEO	
6670	RAB9B	GEO	
6671	RABEP1	GEO	
6672	RABGAP1L	GEO	
6673	RABGGTB	GEO	
6674	RABL4	GEO	
6675	RABL5	GEO	
6676	RAC2	GEO	
6677	RAC3	GEO	
6678	RACGAP1	GEO	
6679	RAD17	GEO	
6680	RAD18	GAD	
6681	RAD21	GEO	
6682	RAD23A	GEO	
6683	RAD23B	Both	
6684	RAD51	GAD	
6685	RAD51C	GEO	
6686	RAD52	GAD	
6687	RAD54B	Both	
6688	RAD9A	GAD	
6689	RAET1L	GEO	
6690	RAI1	GEO	
6691	RAI14	GEO	
6692	RAI4	GAD	
6693	RALA	GEO	
6694	RALGAPA1	GEO	
6695	RALGAPA2	GEO	
6696	RALGDS	GEO	
6697	RALGPS2	GEO	
6698	RANBP17	GEO	

6699	RANBP5	GEO	
6700	RAP1GAP	GEO	
6701	RAP2A	GEO	
6702	RAP2B	GEO	
6703	RAPGEF2	GEO	
6704	RAPGEF4	GEO	
6705	RAPGEF5	GEO	
6706	RAPGEFL1	GEO	
6707	RARA	GEO	
6708	RARG	GEO	
6709	RARRES3	GEO	
6710	RAS	GAD	
6711	RASA4	GEO	
6712	RASD1	GEO	
6713	RASGEF1A	GEO	
6714	RASGRF2	GEO	
6715	RASGRP1	GEO	
6716	RASL10A	GEO	
6717	RASL11B	GEO	
6718	RASL12	GEO	
6719	RASSF1	GAD	
6720	RASSF10	GEO	
6721	RASSF2	GEO	
6722	RASSF5	GEO	
6723	RASSF7	GEO	
6724	RASSF8	GEO	
6725	RAX2	GEO	
6726	RB1	Both	
6727	RBBP4	GEO	
6728	RBBP5	GEO	
6729	RBBP6	GEO	
6730	RBCK1	GEO	
6731	RBFOX2	GEO	
6732	RBKS	GEO	
6733	RBM15	GEO	
6734	RBM20	GEO	
6735	RBM22	GEO	
6736	RBM25	GEO	
6737	RBM26	GEO	
6738	RBM28	GEO	
6739	RBM33	GEO	
6740	RBM39	GEO	
6741	RBM4	GEO	
6742	RBM47	GEO	
6743	RBM5	GEO	
6744	RBMS1	GEO	
6745	RBMY1A1	GEO	
6746	RBP1	GEO	
6747	RBP2	GEO	
6748	RBP4	GEO	
6749	RBP5	GEO	
6750	RBP7	GEO	
6751	RBPJ	GEO	
6752	RBPMS	GEO	
6753	RBPMS2	GEO	
6754	RC3H1	GEO	
6755	RCBTB1	GEO	
6756	RCBTB2	GEO	
6757	RCHY1	GEO	
6758	RDBP	GEO	
6759	RDH10	GEO	
6760	RDH13	GEO	
6761	RDM1	GEO	
6762	RECK	GEO	
6763	RECQL4	GEO	
6764	REEP1	GEO	
6765	REEP5	GEO	
6766	REEP6	Both	
6767	REG1A	GEO	
6768	REG1B	GEO	
6769	REG3A	GEO	
6770	REG4	GEO	
6771	REL	GAD	
6772	RELL2	GEO	
6773	RELN	GEO	
6774	REP15	GEO	
6775	REPIN1	GEO	
6776	REPS1	GEO	
6777	RER1	GEO	
6778	RERG	GEO	
6779	RERGL	GEO	
6780	RETN	GAD	
6781	RETNLB	GEO	
6782	REV3L	GEO	
6783	REXO1	GEO	
6784	RFC1	Both	
6785	RFC5	GEO	
6786	RFESD	GEO	
6787	RFK	GEO	
6788	RFNG	GEO	
6789	RFT1	GEO	
6790	RFTN1	GEO	
6791	RFX4	GEO	
6792	RFX5	GEO	
6793	RFX6	GEO	
6794	RGAG4	GEO	
6795	RGPD1	GEO	
6796	RGPD4	GEO	
6797	RGPD5	GEO	
6798	RGS1	GEO	

6799	RGS13	GEO	
6800	RGS14	GEO	
6801	RGS16	GEO	
6802	RGS19IP1	GEO	
6803	RGS3	GEO	
6804	RGS4	GEO	
6805	RGS5	GEO	
6806	RGSL1	GEO	
6807	RHAG	GEO	
6808	RHBDF2	GEO	
6809	RHBDL1	GEO	
6810	RHBDL2	GEO	
6811	RHBG	GEO	
6812	RHD	GAD	
6813	RHEB	GEO	
6814	RHOA	GAD	
6815	RHOB	GEO	
6816	RHOBTB3	GEO	
6817	RHOH	GEO	
6818	RHOJ	GEO	
6819	RHOQ	GEO	
6820	RHPN2	Both	
6821	RIBC1	GEO	
6822	RIC8A	GEO	
6823	RICH2	GEO	
6824	RICTOR	GEO	
6825	RILP	GEO	
6826	RIMBP3	GEO	
6827	RIMKLB	GEO	
6828	RIMS1	GEO	
6829	RIMS2	GEO	
6830	RIMS3	GEO	
6831	RIOK1	GEO	
6832	RIOK2	GEO	
6833	RIPK1	Both	
6834	RIPK3	GEO	
6835	RIPK4	GEO	
6836	RIT1	GEO	
6837	RMI1	GAD	
6838	RMND1	GEO	
6839	RNASE1	Both	
6840	RNASE4	GEO	
6841	RNASEH1	GEO	
6842	RNASEH2A	GEO	
6843	RNASEH2B	GEO	
6844	RNASEK	GEO	
6845	RNASEL	Both	
6846	RNASET2	Both	
6847	RND1	GEO	
6848	RND3	GEO	

6849	RNF10	GEO	
6850	RNF112	GEO	
6851	RNF123	GAD	
6852	RNF125	GEO	
6853	RNF128	GEO	
6854	RNF13	GEO	
6855	RNF135	GEO	
6856	RNF141	GEO	
6857	RNF144B	GEO	
6858	RNF148	GEO	
6859	RNF157	GEO	
6860	RNF160	GEO	
6861	RNF170	GEO	
6862	RNF180	GEO	
6863	RNF183	GEO	
6864	RNF186	Both	
6865	RNF212	GEO	
6866	RNF213	GEO	
6867	RNF217	GEO	
6868	RNF220	GEO	
6869	RNF5	GEO	
6870	RNF6	GEO	
6871	RNF7	GEO	
6872	RNF8	GEO	
6873	RNFT1	GEO	
6874	RNFT2	GEO	
6875	RNGTT	GEO	
6876	RNMTL1	GEO	
6877	RNPEPL1	GAD	
6878	RNPS1	GEO	
6879	RNR1	GAD	
6880	RNR2	GAD	
6881	RNU12	GEO	
6882	ROBO1	GEO	
6883	ROBO2	GEO	
6884	ROCK1	GEO	
6885	ROD1	GEO	
6886	ROM1	GEO	
6887	ROMO1	GEO	
6888	ROPN1	GEO	
6889	ROR1	GEO	
6890	ROR2	GEO	
6891	RORA	GEO	
6892	RP11-298P3.3	GEO	
6893	RP4-691N24.1	GEO	
6894	RP5-1022P6.2	GEO	
6895	RP9P	GEO	
6896	RPA4	GEO	
6897	RPAIN	GEO	
6898	RPF2	GEO	

6899	RPGR	GEO	
6900	RPGRIP1L	GEO	
6901	RPH3AL	GAD	
6902	RPIB9	GEO	
6903	RPL10A	GEO	
6904	RPL13A	GEO	
6905	RPL17	GEO	
6906	RPL22L1	GEO	
6907	RPL23A	GEO	
6908	RPL28	GEO	
6909	RPL29	GEO	
6910	RPL35A	GEO	
6911	RPL37	GEO	
6912	RPL37A	GEO	
6913	RPL38	GEO	
6914	RPL39	GEO	
6915	RPL3L	GEO	
6916	RPL4	GEO	
6917	RPL5	GEO	
6918	RPL8	GEO	
6919	RPP14	GEO	
6920	RPP25	GEO	
6921	RPRD1B	GEO	
6922	RPRM	Both	
6923	RPS10	GEO	
6924	RPS11	GEO	
6925	RPS15A	GEO	
6926	RPS17	GEO	
6927	RPS24	GEO	
6928	RPS27	GEO	
6929	RPS27A	GEO	
6930	RPS28	GEO	
6931	RPS4Y1	GEO	
6932	RPS4Y2	GEO	
6933	RPS6KA1	GAD	
6934	RPS6KA3	GEO	
6935	RPS6KA5	GEO	
6936	RPS6KA6	GEO	
6937	RPS6KB1	GAD	
6938	RPS6KB2	Both	
6939	RRAD	GEO	
6940	RREB1	GEO	
6941	RRM2	GEO	
6942	RRM2B	GAD	
6943	RRN3	GEO	
6944	RSAD2	GEO	
6945	RSF1	GEO	
6946	RSL24D1	GEO	
6947	RSPH1	GEO	
6948	RSPH3	GEO	

6949	RSPO2	GEO	
6950	RSPO3	GEO	
6951	RSRC1	GEO	
6952	RSRC2	GEO	
6953	RTDR1	GEO	
6954	RTKN2	GEO	
6955	RTN1	GEO	
6956	RTP3	GEO	
6957	RTTN	GEO	
6958	RUFY2	GEO	
6959	RUNX1	Both	
6960	RUSC2	GEO	
6961	RWDD4	GEO	
6962	RXRA	Both	
6963	RYBP	GEO	
6964	RYR1	GEO	
6965	S100A10	GEO	
6966	S100A14	GEO	
6967	S100A4	GEO	
6968	S100A5	GEO	
6969	S100A6	GEO	
6970	S100A8	GEO	
6971	S100A9	GEO	
6972	S100B	GEO	
6973	S100PBP	GEO	
6974	S100Z	GAD	
6975	S1PR1	GEO	
6976	SAA1	GEO	
6977	SAA2	GEO	
6978	SAA4	GEO	
6979	SAFB2	GEO	
6980	SAHH3	GEO	
6981	SALL1	GEO	
6982	SALL2	GEO	
6983	SALL4	GEO	
6984	SAMD12	GEO	
6985	SAMD13	GEO	
6986	SAMD15	GEO	
6987	SAMD4A	GEO	
6988	SAMD5	GEO	
6989	SAMD8	GEO	
6990	SAMD9	GEO	
6991	SAMD9L	GEO	
6992	SAMM50	GEO	
6993	SAPS1	GEO	
6994	SAR1A	GEO	
6995	SARS2	GEO	
6996	SART3	GEO	
6997	SAT1	GEO	
6998	SAT2	GEO	

6999	SATB2	Both	
7000	SBDS	GEO	
7001	SBF1	GEO	
7002	SBF2	GEO	
7003	SBNO2	Both	
7004	SCAF1	GEO	
7005	SCAMP1	GEO	
7006	SCAMP2	GEO	
7007	SCAMP5	GEO	
7008	SCAND1	GEO	
7009	SCAPER	GEO	
7010	SCARA3	GEO	
7011	SCARB2	GEO	
7012	SCCPDH	GEO	
7013	SCD5	GEO	
7014	SCG3	GEO	
7015	SCG5	GEO	
7016	SCGB2A1	GEO	
7017	SCHIP1	GEO	
7018	SCLY	GEO	
7019	SCML1	GEO	
7020	SCML4	GEO	
7021	SCN3A	GEO	
7022	SCN3B	GEO	
7023	SCN7A	GEO	
7024	SCN8A	GEO	
7025	SCNN1A	GEO	
7026	SCRN2	GEO	
7027	SCRN3	GEO	
7028	SCUBE2	GEO	
7029	SDC1	GEO	
7030	SDC2	GEO	
7031	SDC4	GEO	
7032	SDF4	GEO	
7033	SDK1	GEO	
7034	SDR16C5	GEO	
7035	SEC11A	GEO	
7036	SEC13	GEO	
7037	SEC14L1	GEO	
7038	SEC14L4	GEO	
7039	SEC16A	GEO	
7040	SEC16B	GEO	
7041	SEC22B	GEO	
7042	SEC22C	GEO	
7043	SEC23B	GEO	
7044	SEC24D	GEO	
7045	SEC31A	GEO	
7046	SEC31B	GEO	
7047	SEC61A2	GEO	
7048	SEC61B	GEO	

7049	SEC62	GEO	
7050	SECISBP2	GAD	
7051	SECTM1	GEO	
7052	SEL1L	GEO	
7053	SEL1L2	GEO	
7054	SEL1L3	GEO	
7055	SELE	GAD	
7056	SELK	GEO	
7057	SELL	Both	
7058	SELM	GEO	
7059	SELP	GAD	
7060	SELPLG	GEO	
7061	SELS	GAD	
7062	SEMA3C	GEO	
7063	SEMA3D	GEO	
7064	SEMA3E	GEO	
7065	SEMA4A	GEO	
7066	SEMA4B	GEO	
7067	SEMA4F	GEO	
7068	SEMA5A	GEO	
7069	SEMA7A	GEO	
7070	SEMG1	GEO	
7071	SENP6	GEO	
7072	SEP15	GAD	
7073	SEPN1	GEO	
7074	SEPSECS	GEO	
7075	SEPT10	GEO	
7076	SEPT11	GEO	
7077	SEPT2	GEO	
7078	SEPT3	GEO	
7079	SEPT4	GEO	
7080	SEPT6	GEO	
7081	SEPT7	GEO	
7082	SEPT9	GEO	
7083	SEPW1	GEO	
7084	SEPX1	GEO	
7085	SERF1A	GEO	
7086	SERF2	GEO	
7087	SERINC2	GEO	
7088	SERINC3	GEO	
7089	SERINC5	GEO	
7090	SERPINA1	Both	
7091	SERPINA3	GEO	
7092	SERPINA4	GEO	
7093	SERPINA5	GEO	
7094	SERPINA6	GEO	
7095	SERPINB2	GEO	
7096	SERPINC1	GEO	
7097	SERPINE1	Both	
7098	SERPINE2	GEO	

7099	SERPINF1	Both	
7100	SERPINF2	GEO	
7101	SERPING1	GEO	
7102	SERPINI1	GEO	
7103	SERTAD1	GEO	
7104	SERTAD2	GEO	
7105	SESN3	GEO	
7106	SESTD1	GEO	
7107	SETD2	GEO	
7108	SETD4	GEO	
7109	SETD5	GEO	
7110	SEZ6L2	GEO	
7111	SF1	GEO	
7112	SF3A2	GEO	
7113	SF3B1	GEO	
7114	SF3B4	GEO	
7115	SF3B5	GEO	
7116	SFI1	GEO	
7117	SFMBT2	GEO	
7118	SFN	GEO	
7119	SFPQ	GEO	
7120	SFRP2	Both	
7121	SFRP4	Both	
7122	SFRS1	GEO	
7123	SFRS15	GEO	
7124	SFRS2B	GEO	
7125	SFRS3	GEO	
7126	SFRS4	GEO	
7127	SFSWAP	GEO	
7128	SFTA2	GEO	
7129	SFTPB	GEO	
7130	SFTPC	GEO	
7131	SGCB	GEO	
7132	SGCD	GEO	
7133	SGK269	GEO	
7134	SGK494	GEO	
7135	SGPP2	GEO	
7136	SGSH	GEO	
7137	SGSM3	GEO	
7138	SH2B2	GEO	
7139	SH2B3	GEO	
7140	SH2D2A	GEO	
7141	SH2D3A	GEO	
7142	SH2D6	GEO	
7143	SH3BGR	GEO	
7144	SH3BGRL2	GEO	
7145	SH3BGRL3	GEO	
7146	SH3BP4	GEO	
7147	SH3D20	GEO	
7148	SH3GL2	GEO	

7149	SH3GLB1	GAD	
7150	SH3PXD2A	GEO	
7151	SH3RF2	GEO	
7152	SH3TC1	GEO	
7153	SH3YL1	GEO	
7154	SHANK3	GEO	
7155	SHC3	GEO	
7156	SHC4	GEO	
7157	SHD	GEO	
7158	SHE	GEO	
7159	SHH	GEO	
7160	SHISA5	GEO	
7161	SHMT1	GAD	
7162	SHOX2	GEO	
7163	SHPK	GEO	
7164	SHQ1	GEO	
7165	ShrmL	GEO	
7166	SHROOM2	GEO	
7167	SHROOM3	GEO	
7168	SHROOM4	GEO	
7169	SI	GEO	
7170	SIAE	GEO	
7171	SIDT1	GEO	
7172	SIGLEC15	GEO	
7173	SIGLEC7	GEO	
7174	SIK1	GEO	
7175	SIK3	GEO	
7176	SIP1	GEO	
7177	SIPA1L2	GEO	
7178	SIPA1L3	GEO	
7179	SIRPA	GEO	
7180	SIRT5	GEO	
7181	SIX1	GEO	
7182	SKA3	GEO	
7183	SKIL	GEO	
7184	SKP1	GEO	
7185	SKP2	GEO	
7186	SLA2	GEO	
7187	SLAMF1	GEO	
7188	SLAMF7	GEO	
7189	SLAMF8	GEO	
7190	SLC10A1	GEO	
7191	SLC10A2	GAD	
7192	SLC11A1	GAD	
7193	SLC12A3	GEO	
7194	SLC13A2	GEO	
7195	SLC13A3	GEO	
7196	SLC13A5	GEO	
7197	SLC14A1	Both	
7198	SLC15A1	Both	

7199	SLC15A2	GEO	
7200	SLC16A1	GEO	
7201	SLC16A14	GEO	
7202	SLC16A2	GEO	
7203	SLC16A4	GEO	
7204	SLC16A5	GEO	
7205	SLC16A6	GEO	
7206	SLC16A7	GEO	
7207	SLC17A4	GEO	
7208	SLC17A9	GEO	
7209	SLC18A1	GEO	
7210	SLC19A1	Both	
7211	SLC1A3	GEO	
7212	SLC1A5	GEO	
7213	SLC22A1	GEO	
7214	SLC22A11	GEO	
7215	SLC22A15	GEO	
7216	SLC22A18AS	GEO	
7217	SLC22A2	GEO	
7218	SLC22A23	Both	
7219	SLC22A5	Both	
7220	SLC22A6	GAD	
7221	SLC23A1	GAD	
7222	SLC23A2	GAD	
7223	SLC25A1	GEO	
7224	SLC25A13	GEO	
7225	SLC25A14	GEO	
7226	SLC25A20	GEO	
7227	SLC25A21	GEO	
7228	SLC25A22	GEO	
7229	SLC25A25	GEO	
7230	SLC25A30	GEO	
7231	SLC25A32	GEO	
7232	SLC25A4	GEO	
7233	SLC25A5	GEO	
7234	SLC25A6	GEO	
7235	SLC26A4	GEO	
7236	SLC28A3	GEO	
7237	SLC29A1	GEO	
7238	SLC2A14	GEO	
7239	SLC2A3	GEO	
7240	SLC2A4RG	GEO	
7241	SLC30A1	GEO	
7242	SLC30A3	GEO	
7243	SLC31A2	GEO	
7244	SLC34A2	GEO	
7245	SLC35A1	GEO	
7246	SLC35A2	GEO	
7247	SLC35D1	GEO	
7248	SLC35D3	GEO	
7249	SLC35E1	GEO	
7250	SLC36A1	GEO	
7251	SLC36A4	GEO	
7252	SLC37A2	GEO	
7253	SLC37A4	GEO	
7254	SLC38A10	GEO	
7255	SLC38A2	GEO	
7256	SLC38A3	GEO	
7257	SLC38A4	GEO	
7258	SLC39A13	GEO	
7259	SLC39A3	GEO	
7260	SLC39A4	GEO	
7261	SLC39A5	GEO	
7262	SLC39A8	GEO	
7263	SLC3A1	GEO	
7264	SLC41A2	GEO	
7265	SLC44A2	GEO	
7266	SLC44A3	GEO	
7267	SLC44A4	GEO	
7268	SLC45A4	GEO	
7269	SLC46A1	GEO	
7270	SLC47A1	GEO	
7271	SLC48A1	GEO	
7272	SLC4A3	GEO	
7273	SLC5A1	GEO	
7274	SLC5A6	GEO	
7275	SLC5A9	GEO	
7276	SLC6A10P	GEO	
7277	SLC6A13	GEO	
7278	SLC6A14	GEO	
7279	SLC6A3	GEO	
7280	SLC6A4	GAD	
7281	SLC6A8	GEO	
7282	SLC7A1	GEO	
7283	SLC7A11	GEO	
7284	SLC7A6	GEO	
7285	SLC7A7	GEO	
7286	SLC9A3R1	GEO	
7287	SLC9A3R2	GEO	
7288	SLC9A8	GEO	
7289	SLCO1B1	GAD	
7290	SLCO1B3	GEO	
7291	SLCO2A1	GEO	
7292	SLCO2B1	GEO	
7293	SLCO5A1	GEO	
7294	SLFN5	GEO	
7295	SLIT3	GEO	
7296	SLK	GEO	
7297	SLMO2	GEO	
7298	SLPI	GEO	

7299	SMAD1	GEO	
7300	SMAD3	Both	
7301	SMAD5	GEO	
7302	SMAD7	Both	
7303	SMAGP	GEO	
7304	SMAP1	GEO	
7305	SMARCA2	GEO	
7306	SMARCA4	GEO	
7307	SMARCA5	GEO	
7308	SMARCD3	GEO	
7309	SMARCE1	GEO	
7310	SMC5	GEO	
7311	SMCHD1	GEO	
7312	SMCR7L	GEO	
7313	SMCY	GEO	
7314	SMEK2	GEO	
7315	SMG7	GEO	
7316	SMN1	GEO	
7317	SMNDC1	GEO	
7318	SMO	GEO	
7319	SMOX	GEO	
7320	SMPD1	GEO	
7321	SMPD3	GEO	
7322	SMU1	GEO	
7323	SMURF1	GAD	
7324	SMURF2	GEO	
7325	SMYD1	GEO	
7326	SMYD3	GAD	
7327	SNAP23	GEO	
7328	SNAPC3	GEO	
7329	SND1	GEO	
7330	SNHG1	GEO	
7331	SNHG7	GEO	
7332	SNIP	GEO	
7333	SNN	GEO	
7334	SNORA71A	GEO	
7335	SNORD104	GEO	
7336	SNRPA1	GEO	
7337	SNRPB2	GEO	
7338	SNRPE	GEO	
7339	SNRPF	GEO	
7340	SNRPN	GEO	
7341	SNTB2	GEO	
7342	SNX10	GEO	
7343	SNX19	GEO	
7344	SNX25	GEO	
7345	SNX26	GEO	
7346	SNX4	GEO	
7347	SNX5	GEO	
7348	SNX8	GEO	

7349	SOAT1	GEO	
7350	SOAT2	GEO	
7351	SOCS2	GEO	
7352	SOCS3	GEO	
7353	SOCS5	GEO	
7354	SOD2	Both	
7355	SOD3	GEO	
7356	SOHLH1	GEO	
7357	SOHLH2	GEO	
7358	SORBS1	GEO	
7359	SORBS2	GEO	
7360	SORBS3	GEO	
7361	SORCS1	GEO	
7362	SORCS2	GEO	
7363	SOS2	GEO	
7364	SOSTDC1	GEO	
7365	SOX1	GEO	
7366	SOX10	GEO	
7367	SOX15	GEO	
7368	SOX18	GEO	
7369	SOX30	GEO	
7370	SOX8	GEO	
7371	SP140	GEO	
7372	SP140L	GEO	
7373	SP8	GEO	
7374	SPACA1	GEO	
7375	SPAG17	GAD	
7376	SPAG4	GEO	
7377	SPAG9	GEO	
7378	SPATA13	GEO	
7379	SPATA17	GEO	
7380	SPATA18	GEO	
7381	SPATA2	GEO	
7382	SPATA5	GEO	
7383	SPATA6	GEO	
7384	SPATA7	GEO	
7385	SPCS1	GEO	
7386	SPCS3	GEO	
7387	SPDEF	GEO	
7388	SPECC1	GEO	
7389	SPEG	GEO	
7390	SPESP1	GEO	
7391	SPG11	GEO	
7392	SPG20	GEO	
7393	SPI1	GEO	
7394	SPIN2A	GEO	
7395	SPIN3	GEO	
7396	SPINK1	GEO	
7397	SPINK2	GEO	
7398	SPINK5	GEO	

7399	SPINT1	GEO	
7400	SPIRE2	GEO	
7401	SPOCK1	GEO	
7402	SPOCK2	GEO	
7403	SPON1	GEO	
7404	SPON2	GEO	
7405	SPOPL	GEO	
7406	SPPL2B	GEO	
7407	SPRR1B	GEO	
7408	SPRR3	GEO	
7409	SPSB1	GEO	
7410	SPSB4	GEO	
7411	SPTA1	GEO	
7412	SPTB	GEO	
7413	SPTBN1	GEO	
7414	SPTLC1	GEO	
7415	SPTLC3	GEO	
7416	SPTY2D1	GEO	
7417	SQLE	GEO	
7418	SQSTM1	GEO	
7419	SR140	GEO	
7420	SRC	GEO	
7421	SRCAP	GEO	
7422	SRD5A1	GEO	
7423	SREBF1	GEO	
7424	SREK1IP1	GEO	
7425	SRGAP1	GEO	
7426	SRGAP2P1	GEO	
7427	SRGN	GEO	
7428	SRMS	GEO	
7429	SRP72	GEO	
7430	SRPK2	GEO	
7431	SRPK3	GEO	
7432	SRPRB	GEO	
7433	SRRM1	GEO	
7434	SRRM2	GEO	
7435	SRRM3	GEO	
7436	SRSF1	GEO	
7437	SRSF11	GEO	
7438	SRSF3	GEO	
7439	SRSF4	GEO	
7440	SRSF5	GEO	
7441	SSBP3	GEO	
7442	SSFA2	GEO	
7443	SSH1	Both	
7444	SSH2	GAD	
7445	SSH3	GEO	
7446	SSR3	GEO	
7447	SSTR1	GEO	
7448	SSTR2	GEO	

7449	SSX1	GEO	
7450	SSX2IP	GEO	
7451	ST13	GEO	
7452	ST14	GEO	
7453	ST3GAL2	GEO	
7454	ST3GAL4	GEO	
7455	ST3GAL6	GEO	
7456	ST6GAL1	GEO	
7457	ST6GALNAC6	GEO	
7458	ST7	GEO	
7459	ST7L	GEO	
7460	ST8SIA4	GEO	
7461	STAG3	GEO	
7462	STAM2	GEO	
7463	STARD10	GEO	
7464	STARD4	GEO	
7465	STARD7	GEO	
7466	STARD9	GEO	
7467	STAT1	GEO	
7468	STAT3	GAD	
7469	STAT4	Both	
7470	STAT5A	GEO	
7471	STAT6	Both	
7472	STEAP1	GEO	
7473	STIM2	GEO	
7474	STIP1	GEO	
7475	STK17A	GEO	
7476	STK17B	GEO	
7477	STK3	GEO	
7478	STK31	GEO	
7479	STK33	GEO	
7480	STK39	GEO	
7481	STOM	GEO	
7482	STOML2	GEO	
7483	STON1	GEO	
7484	STRADA	GEO	
7485	STRN3	GEO	
7486	STUB1	GEO	
7487	STX6	GEO	
7488	STX7	GEO	
7489	STX8	Both	
7490	STXBP3	GEO	
7491	STXBP5L	GEO	
7492	SUB1	GEO	
7493	SUGT1	GEO	
7494	SULT1A3	GEO	
7495	SULT1A4	GEO	
7496	SULT1B1	GEO	
7497	SULT1C2	GEO	
7498	SULT2A1	GEO	

7499	SUMO3	GEO	
7500	SUPT16H	GEO	
7501	SUPT4H1	GEO	
7502	SURF1	GAD	
7503	SURF4	GEO	
7504	SUSD4	GEO	
7505	SUV420H1	GEO	
7506	SUZ12	GEO	
7507	SV2B	GEO	
7508	SVEP1	GEO	
7509	SVOP	GEO	
7510	SVOPL	GEO	
7511	SYCE1L	GEO	
7512	SYCP3	GEO	
7513	SYDE1	GEO	
7514	SYDE2	GEO	
7515	SYMPK	GAD	
7516	SYNC1	GEO	
7517	SYNE1	GEO	
7518	SYNJ2	GEO	
7519	SYNJ2BP	GEO	
7520	SYNM	GEO	
7521	SYNPO2	GEO	
7522	SYPL1	GEO	
7523	SYT1	GEO	
7524	SYT7	GEO	
7525	SYTL1	GEO	
7526	SYTL3	GEO	
7527	SYTL4	GEO	
7528	SYTL5	GEO	
7529	T1A-2	GEO	
7530	TAB2	GEO	
7531	TACC1	GEO	
7532	TACC2	GEO	
7533	TACR2	GEO	
7534	TAF13	GEO	
7535	TAF15	GEO	
7536	TAF1D	GEO	
7537	TAF8	GEO	
7538	TAF9B	GEO	
7539	TAGAP	Both	
7540	TAGLN	GEO	
7541	TANC2	GEO	
7542	TANK	GEO	
7543	TAOK3	GEO	
7544	TAP1	GEO	
7545	TAP2	GEO	
7546	TARBP1	GEO	
7547	TARDBP	GEO	
7548	TARP	GEO	

7549	TARSL2	GEO	
7550	TAS2R14	GAD	
7551	TAS2R16	GEO	
7552	TATDN1	GEO	
7553	TAZ	GEO	
7554	TBC1D1	GEO	
7555	TBC1D16	GEO	
7556	TBC1D19	GEO	
7557	TBC1D22A	GEO	
7558	TBC1D8	GEO	
7559	TBC1D9	GEO	
7560	TBCB	GEO	
7561	TBCD	GEO	
7562	TBCEL	GEO	
7563	TBL1X	GEO	
7564	TBPL1	GEO	
7565	TBR1	GAD	
7566	TBRG1	GEO	
7567	TBRG4	GEO	
7568	TBX2	GEO	
7569	TBX3	GEO	
7570	TBX6	GEO	
7571	TBXAS1	GEO	
7572	tcag7.907	GEO	
7573	TCEAL2	GEO	
7574	TCEAL3	GEO	
7575	TCEAL4	GEO	
7576	TCEB1	GEO	
7577	TCEB2	GEO	
7578	TCERG1	GEO	
7579	TCF19	GEO	
7580	TCF25	GEO	
7581	TCF3	GEO	
7582	TCF7L1	GEO	
7583	TCF7L2	GAD	
7584	TCFL5	GEO	
7585	TCN2	GAD	
7586	TCP11L1	GEO	
7587	TCP11L2	GEO	
7588	TCTE3	GEO	
7589	TDE2L	GEO	
7590	TDG	GEO	
7591	TDGF1	GEO	
7592	TDP1	Both	
7593	TDP2	GEO	
7594	TDRD10	GEO	
7595	TDRD7	GEO	
7596	TDRD9	GAD	
7597	TDRKH	GEO	
7598	TEAD2	GEO	

7599	TECTA	GEO	
7600	TEF	GEO	
7601	TEK	GEO	
7602	TEKT4	GEO	
7603	TEP1	GEO	
7604	TET1	GEO	
7605	TET2	GEO	
7606	TEX11	GEO	
7607	TEX19	GEO	
7608	TEX261	GEO	
7609	TEX9	GEO	
7610	TF	Both	
7611	TFAM	GEO	
7612	TFAP2A	GEO	
7613	TFDP1	GEO	
7614	TFDP2	GEO	
7615	TFE3	GEO	
7616	TFEB	GEO	
7617	TFF1	GEO	
7618	TFF2	GEO	
7619	TFF3	GEO	
7620	TFG	GEO	
7621	TFPI	GEO	
7622	TFPI2	GEO	
7623	TFRC	GAD	
7624	TG	GEO	
7625	TGFA	GEO	
7626	TGFB1I1	GEO	
7627	TGFBR3	GEO	
7628	TGFBRI	GAD	
7629	TGIF	GEO	
7630	TGM2	GEO	
7631	TGM4	GEO	
7632	TGS1	GEO	
7633	TH1L	GEO	
7634	THAP2	GEO	
7635	THBD	GEO	
7636	THBS1	Both	
7637	THBS4	GEO	
7638	THEM5	GEO	
7639	THG1L	GEO	
7640	THNSL1	GEO	
7641	THNSL2	GEO	
7642	THOC4	GEO	
7643	THRA	GEO	
7644	THRAP3	GEO	
7645	THRB	GEO	
7646	THSD7A	GEO	
7647	THUMPD1	GEO	
7648	THY1	GEO	

7649	TIAL1	GEO	
7650	TICAM1	GEO	
7651	TIE1	GEO	
7652	TIFA	GEO	
7653	TIGD2	GEO	
7654	TIGD7	GEO	
7655	TIMELESS	GEO	
7656	TIMM13	GEO	
7657	TIMM17B	GEO	
7658	TIMM23	GEO	
7659	TIMM44	GEO	
7660	TIMM8B	GEO	
7661	TIMP4	GEO	
7662	TINAG	GEO	
7663	TINAGL1	GEO	
7664	TIPARP	GEO	
7665	TIPRL	GEO	
7666	TJP2	GEO	
7667	TJP3	GEO	
7668	TKTL1	GEO	
7669	TLCD2	GEO	
7670	TLE2	GEO	
7671	TLE3	GEO	
7672	TLE4	GEO	
7673	TLN2	GEO	
7674	TLR1	Both	
7675	TLR2	GAD	
7676	TLR3	GEO	
7677	TLR4	Both	
7678	TLR5	GAD	
7679	TLR6	GAD	
7680	TLR7	GEO	
7681	TLR9	GAD	
7682	TLX2	GEO	
7683	TM16F	GEO	
7684	TM2D3	GEO	
7685	TM4SF18	GEO	
7686	TM4SF19	GEO	
7687	TM4SF2	GEO	
7688	TM4SF20	GEO	
7689	TM4SF4	GEO	
7690	TM4SF9	GEO	
7691	TM7SF2	GEO	
7692	TM9SF2	GEO	
7693	TM9SF3	GEO	
7694	TMBIM1	GEO	
7695	TMBIM4	GEO	
7696	TMC4	GEO	
7697	TMC5	GEO	
7698	TMC6	GEO	

7699	TMCC2	GEO	
7700	TMCO3	GEO	
7701	TMCO7	GEO	
7702	TMED3	GEO	
7703	TMED6	GEO	
7704	TMED8	GEO	
7705	TMED9	GEO	
7706	TMEFF2	GEO	
7707	TMEM1	GEO	
7708	TMEM100	GEO	
7709	TMEM106A	GEO	
7710	TMEM109	GEO	
7711	TMEM111	GEO	
7712	TMEM132A	GEO	
7713	TMEM132B	GEO	
7714	TMEM136	GEO	
7715	TMEM14A	GEO	
7716	TMEM151B	GEO	
7717	TMEM159	GEO	
7718	TMEM160	GEO	
7719	TMEM161A	GEO	
7720	TMEM164	GEO	
7721	TMEM166	GEO	
7722	TMEM16E	GEO	
7723	TMEM16G	GEO	
7724	TMEM170A	GEO	
7725	TMEM170B	GEO	
7726	TMEM173	GEO	
7727	TMEM178	GEO	
7728	TMEM18	GEO	
7729	TMEM181	GEO	
7730	TMEM184A	GEO	
7731	TMEM184C	GEO	
7732	TMEM189-UBE2	GEO	
7733	TMEM191A	GEO	
7734	TMEM195	GEO	
7735	TMEM200A	GEO	
7736	TMEM201	GEO	
7737	TMEM206	GEO	
7738	TMEM217	GEO	
7739	TMEM220	GEO	
7740	TMEM223	GEO	
7741	TMEM25	GEO	
7742	TMEM27	GEO	
7743	TMEM30B	GEO	
7744	TMEM33	GEO	
7745	TMEM35	GEO	
7746	TMEM37	GEO	
7747	TMEM38B	GEO	
7748	TMEM45A	GEO	

7749	TMEM45B	GEO	
7750	TMEM47	GEO	
7751	TMEM49	GEO	
7752	TMEM50A	GEO	
7753	TMEM56	GEO	
7754	TMEM60	GEO	
7755	TMEM61	GEO	
7756	TMEM62	GEO	
7757	TMEM63C	GEO	
7758	TMEM65	GEO	
7759	TMEM66	GEO	
7760	TMEM67	GEO	
7761	TMEM72	GEO	
7762	TMEM86B	GEO	
7763	TMEM87A	GEO	
7764	TMEM91	GEO	
7765	TMEM92	GEO	
7766	TMEM97	GEO	
7767	TMEPAI	GEO	
7768	TMIGD	GEO	
7769	TMIGD1	GEO	
7770	TMLHE	GEO	
7771	TMOD1	GEO	
7772	TMOD2	GEO	
7773	TMOD3	GEO	
7774	TMPRSS15	GEO	
7775	TMPRSS4	GEO	
7776	TMPRSS6	GEO	
7777	TMSB10	GEO	
7778	TMSB15B	GEO	
7779	TMSB4X	GEO	
7780	TMTC4	GEO	
7781	TMX3	GEO	
7782	TNC	GEO	
7783	TNF	GAD	
7784	TNFAIP1	GEO	
7785	TNFAIP3	Both	
7786	TNFAIP6	GEO	
7787	TNFAIP8	GEO	
7788	TNFAIP8L1	GEO	
7789	TNFRSF10A	Both	
7790	TNFRSF10D	GEO	
7791	TNFRSF11A	GEO	
7792	TNFRSF13B	GEO	
7793	TNFRSF17	GEO	
7794	TNFRSF18	GEO	
7795	TNFRSF19	GEO	
7796	TNFRSF1A	GAD	
7797	TNFRSF1B	Both	
7798	TNFRSF25	GEO	

7799	TNFRSF6B	GAD	
7800	TNFSF12	GEO	
7801	TNFSF15	Both	
7802	TNFSF18	GAD	
7803	TNIP1	GEO	
7804	TNKS2	GEO	
7805	TNMD	GEO	
7806	TNNC1	GEO	
7807	TNNC2	GEO	
7808	TNNI2	GEO	
7809	TNNI3	GEO	
7810	TNNT1	GEO	
7811	TNPO1	GEO	
7812	TNPO2	GEO	
7813	TNRC18	GEO	
7814	TNRC6B	GEO	
7815	TNS3	GEO	
7816	TNS4	GEO	
7817	TNXB	GEO	
7818	TOB1	GEO	
7819	TOLLIP	GEO	
7820	TOM1L2	GEO	
7821	TOMM40	GEO	
7822	TOP1	GEO	
7823	TOP3A	GEO	
7824	TOP3B	GEO	
7825	TOR1AIP2	GEO	
7826	TOX	GEO	
7827	TP53	Both	
7828	TP53BP1	GAD	
7829	TP53BP2	GAD	
7830	TP53I11	GEO	
7831	TP53I5	GEO	
7832	TP53TG5	GEO	
7833	TP73	Both	
7834	TPBG	GEO	
7835	TPD52L1	GEO	
7836	TPK1	GEO	
7837	TPM1	GEO	
7838	TPM2	GEO	
7839	TPM3	GEO	
7840	TPM4	GEO	
7841	TPMT	Both	
7842	TPP2	GEO	
7843	TPPP	Both	
7844	TPR	GEO	
7845	TPRG1	GEO	
7846	TPRXL	GEO	
7847	TPSAB1	GEO	
7848	TPSB2	GEO	

7849	TPST1	GEO	
7850	TPST2	GEO	
7851	TPT1	GEO	
7852	TPTE	GEO	
7853	TRA	GAD	
7854	TRABD	GEO	
7855	TRAC	GEO	
7856	TRAF3	GEO	
7857	TRAF6	GAD	
7858	TRAIP	Both	
7859	TRAK1	GEO	
7860	TRAM2	GEO	
7861	TRANK1	GEO	
7862	TRAPPC10	GEO	
7863	TRAPPC6A	GEO	
7864	TRBC1	GEO	
7865	TRDMT1	GEO	
7866	TRHDE	GEO	
7867	TRIB1	Both	
7868	TRIF	GEO	
7869	TRIM14	GEO	
7870	TRIM15	GEO	
7871	TRIM2	GEO	
7872	TRIM21	GEO	
7873	TRIM25	GEO	
7874	TRIM29	GEO	
7875	TRIM31	GEO	
7876	TRIM33	GEO	
7877	TRIM37	GEO	
7878	TRIM38	GEO	
7879	TRIM44	GEO	
7880	TRIM55	GEO	
7881	TRIM56	GEO	
7882	TRIM7	GEO	
7883	TRIM9	GEO	
7884	TRIO	GEO	
7885	TRIP11	GEO	
7886	TRMT1	GEO	
7887	TRMT112	GEO	
7888	TRMT12	GEO	
7889	TRMT2B	GEO	
7890	TRND	GAD	
7891	TRNG	GAD	
7892	TRNL2	GAD	
7893	TRNN	GAD	
7894	TRNQ	GAD	
7895	TRNR	GAD	
7896	TRNT	GAD	
7897	TROVE2	GEO	
7898	TRPA1	GEO	

7899	TRPC4	GEO	
7900	TRPC4AP	GEO	
7901	TRPM4	GEO	
7902	TRPM7	GAD	
7903	TRPV1	GEO	
7904	TRPV2	GEO	
7905	TRPV6	GEO	
7906	TRUB1	GEO	
7907	TS13	GAD	
7908	TSC1	GAD	
7909	TSC2	GAD	
7910	TSC22D3	GEO	
7911	TSEN15	GEO	
7912	TSGA14	GEO	
7913	TSHZ3	GEO	
7914	TSLP	GEO	
7915	TSNAXIP1	GEO	
7916	TSPAN11	GEO	
7917	TSPAN12	GEO	
7918	TSPAN13	GEO	
7919	TSPAN14	GEO	
7920	TSPAN2	GEO	
7921	TSPAN3	GEO	
7922	TSPAN31	GEO	
7923	TSPAN33	GEO	
7924	TSPAN4	GEO	
7925	TSPAN8	Both	
7926	TSPY1	GEO	
7927	TSPYL1	GEO	
7928	TSSC4	GEO	
7929	TTBK2	GEO	
7930	TTC13	GEO	
7931	TTC17	GEO	
7932	TTC18	GEO	
7933	TTC19	GEO	
7934	TTC23	GEO	
7935	TTC25	GEO	
7936	TTC30B	GEO	
7937	TTC37	GEO	
7938	TTC39B	GEO	
7939	TTC39C	GEO	
7940	TTC5	GEO	
7941	TTC6	GEO	
7942	TTC7B	GEO	
7943	TTK	GAD	
7944	TTLL3	GEO	
7945	TTLL5	GEO	
7946	TTR	GEO	
7947	TTRAP	GEO	
7948	TTYH2	GEO	
7949	TUB	GEO	
7950	TUBA1A	GEO	
7951	TUBA4A	GEO	
7952	TUBB2A	GEO	
7953	TUBB2B	GEO	
7954	TUBGCP2	GEO	
7955	TUFM	GEO	
7956	TUG1	GEO	
7957	TUSC3	GEO	
7958	TUSC5	GEO	
7959	TUT1	GEO	
7960	TWIST1	GEO	
7961	TXLNG	GEO	
7962	TXN	GEO	
7963	TXNDC12	GEO	
7964	TXNL4B	GEO	
7965	TXNRD1	Both	
7966	TXNRD2	GAD	
7967	TYK2	GAD	
7968	TYMP	GEO	
7969	TYROBP	GEO	
7970	U2AF2	GEO	
7971	UAP1L1	GEO	
7972	UBA7	GAD	
7973	UBAC2	GEO	
7974	UBAP2	GEO	
7975	UBC	GEO	
7976	UBD	GEO	
7977	UBE2C	GEO	
7978	UBE2D2	GEO	
7979	UBE2D3	GEO	
7980	UBE2F	GEO	
7981	UBE2G1	GEO	
7982	UBE2G2	GEO	
7983	UBE2K	GEO	
7984	UBE2L3	GAD	
7985	UBE2L6	GEO	
7986	UBE2M	GEO	
7987	UBE2N	GEO	
7988	UBE2R2	GEO	
7989	UBE2S	GEO	
7990	UBE2V1	GEO	
7991	UBE2W	GEO	
7992	UBE3A	GEO	
7993	UBE3C	GEO	
7994	UBP1	GEO	
7995	UBQLN2	GEO	
7996	UBQLN4	GEO	
7997	UBR4	GEO	
7998	UBR5	GEO	

7999	UBXN6	GEO	
8000	UCA1	GEO	
8001	UCHL1	GEO	
8002	UCHL3	GEO	
8003	UCN3	GEO	
8004	UCP2	Both	
8005	UGCG	GEO	
8006	UGT1A	GAD	
8007	UGT1A10	Both	
8008	UGT1A3	Both	
8009	UGT1A4	Both	
8010	UGT1A6	Both	
8011	UGT1A7	GAD	
8012	UGT1A9	GAD	
8013	UGT2A3	GEO	
8014	UGT2B4	Both	
8015	UGT2B7	Both	
8016	UGT8	GEO	
8017	UHMK1	GEO	
8018	UHRF2	GEO	
8019	ULA4	GEO	
8020	ULBP2	GEO	
8021	ULK4	GEO	
8022	UMODL1	GEO	
8023	UMPS	GAD	
8024	UNC119	GEO	
8025	UNC13A	GEO	
8026	UNC5A	GEO	
8027	UNC5B	GEO	
8028	UNC5CL	GEO	
8029	UNC93B1	GEO	
8030	UNKL	GEO	
8031	UNQ9368	GEO	
8032	UPB1	Both	
8033	UPF1	GEO	
8034	UPF3A	GEO	
8035	UPK3A	GEO	
8036	UPK3B	GEO	
8037	UQCC	GEO	
8038	UQCRC1	GEO	
8039	URB1	GEO	
8040	URG4	GEO	
8041	USE1	GEO	
8042	USH1C	GEO	
8043	USH2A	GEO	
8044	USO1	GEO	
8045	USP1	GEO	
8046	USP12	GAD	
8047	USP13	GEO	
8048	USP14	GEO	

8049	USP18	GEO	
8050	USP19	GEO	
8051	USP22	GEO	
8052	USP24	GEO	
8053	USP25	GAD	
8054	USP33	GEO	
8055	USP34	GEO	
8056	USP36	GEO	
8057	USP4	GAD	
8058	USP42	GEO	
8059	USP45	GEO	
8060	USP48	GEO	
8061	USP49	GEO	
8062	USP53	GEO	
8063	USP7	GEO	
8064	USP9Y	GEO	
8065	UST	GEO	
8066	UTP11L	GEO	
8067	UTP14A	GEO	
8068	UTRN	GEO	
8069	UXS1	GEO	
8070	VAMP1	GEO	
8071	VAMP8	GEO	
8072	VAPA	GEO	
8073	VAPB	GEO	
8074	VASN	GEO	
8075	VAV3	GEO	
8076	VCAN	GEO	
8077	VCL	GEO	
8078	VDAC1	GEO	
8079	VDR	GAD	
8080	VEGF	GAD	
8081	VEGFA	Both	
8082	VGF	GEO	
8083	VGLL1	GEO	
8084	VGLL4	GEO	
8085	VHL	GAD	
8086	VIL1	GEO	
8087	VIM	GEO	
8088	VKORC1	GEO	
8089	VMA21	GEO	
8090	VMD2L1	GEO	
8091	VMD2L2	GEO	
8092	VNN1	GEO	
8093	VNN3	GEO	
8094	VPS13B	GEO	
8095	VPS13C	GEO	
8096	VPS13D	GEO	
8097	VPS18	GEO	
8098	VPS26A	GEO	

8099	VPS37C	GEO	
8100	VPS4B	GEO	
8101	VPS53	GEO	
8102	VPS8	GEO	
8103	VRK2	GEO	
8104	VSIG4	GEO	
8105	VSTM2A	GEO	
8106	VSTM2L	GEO	
8107	VTN	GEO	
8108	VWA5B2	GEO	
8109	VWCE	GEO	
8110	VWDE	GEO	
8111	WAC	GEO	
8112	WAS	GEO	
8113	WASF3	GEO	
8114	WASH3P	GEO	
8115	WBP4	GEO	
8116	WBSCR22	GEO	
8117	WBSCR27	GEO	
8118	WDFY3	GEO	
8119	WDR1	GEO	
8120	WDR12	GEO	
8121	WDR31	GEO	
8122	WDR33	GEO	
8123	WDR4	GEO	
8124	WDR43	GEO	
8125	WDR46	GEO	
8126	WDR5	GEO	
8127	WDR51B	GEO	
8128	WDR52	GEO	
8129	WDR59	GEO	
8130	WDR61	GEO	
8131	WDR67	GEO	
8132	WDR7	GEO	
8133	WDR71	GEO	
8134	WDR72	GEO	
8135	WDR77	GEO	
8136	WDR78	GEO	
8137	WDR79	GEO	
8138	WDR86	GEO	
8139	WDR90	GEO	
8140	WDSOF1	GEO	
8141	WDYHV1	GEO	
8142	WFDC1	GEO	
8143	WFDC2	GEO	
8144	WHAMM	GEO	
8145	WHAMML1	GEO	
8146	WHDC1L1	GEO	
8147	WHSC1	GEO	
8148	WIF1	GAD	

8149	WIPF1	GEO	
8150	WISP1	Both	
8151	WISP2	GEO	
8152	WISP3	GAD	
8153	WLS	GEO	
8154	WNK1	GEO	
8155	WNK2	GEO	
8156	WNK4	GEO	
8157	WNT10A	GEO	
8158	WNT11	GEO	
8159	WNT16	GEO	
8160	WNT2B	GAD	
8161	WNT4	GEO	
8162	WNT5A	GEO	
8163	WNT6	GEO	
8164	WNT7A	GEO	
8165	WNT7B	GEO	
8166	WRN	GAD	
8167	WSB1	GEO	
8168	WSCD1	GEO	
8169	WT1	GEO	
8170	WTAP	GEO	
8171	WWC2	GEO	
8172	WWP1	GEO	
8173	XCR1	GEO	
8174	XDH	Both	
8175	XIAP	GEO	
8176	XIST	GEO	
8177	XK	GEO	
8178	XKR9	GEO	
8179	XKRX	GEO	
8180	XLKD1	GEO	
8181	XPA	GAD	
8182	XPC	GAD	
8183	XPNPEP2	GEO	
8184	XPO1	GEO	
8185	XPO7	GEO	
8186	XRCC1	GAD	
8187	XRCC2	GAD	
8188	XRCC3	GAD	
8189	XRCC5	GAD	
8190	XRRA1	GEO	
8191	YAP1	GEO	
8192	YBX2	GEO	
8193	YIF1A	GEO	
8194	YIPF3	GEO	
8195	YIPF5	GEO	
8196	YJEFN3	GEO	
8197	YKT6	GEO	
8198	YLPM1	GEO	

8199	YPEL1	GEO	
8200	YPEL5	GEO	
8201	YTHDC1	GEO	
8202	YTHDC2	GEO	
8203	YTHDF1	GEO	
8204	YWHAQ	GEO	
8205	ZADH2	GEO	
8206	ZAK	GEO	
8207	ZAP70	GEO	
8208	ZBED2	GEO	
8209	ZBED5	GEO	
8210	ZBTB1	GEO	
8211	ZBTB10	GEO	
8212	ZBTB16	GEO	
8213	ZBTB20	GEO	
8214	ZBTB26	GEO	
8215	ZBTB33	GEO	
8216	ZBTB38	GAD	
8217	ZBTB4	GEO	
8218	ZBTB40	GEO	
8219	ZBTB44	GEO	
8220	ZBTB46	GEO	
8221	ZBTB47	GEO	
8222	ZBTB7A	GEO	
8223	ZBTB7C	GEO	
8224	ZBTB8	GEO	
8225	ZC3H11A	GEO	
8226	ZC3H12A	GEO	
8227	ZC3H13	GEO	
8228	ZC3H14	GEO	
8229	ZC3H4	GEO	
8230	ZC3H7A	GEO	
8231	ZC3H7B	GEO	
8232	ZCCHC10	GEO	
8233	ZCCHC11	GEO	
8234	ZCCHC2	GEO	
8235	ZCCHC4	GEO	
8236	ZCCHC7	GEO	
8237	ZCRB1	GEO	
8238	ZDBF2	GEO	
8239	ZDHHC1	GAD	
8240	ZDHHC12	GEO	
8241	ZDHHC14	GEO	
8242	ZDHHC17	GEO	
8243	ZDHHC21	GEO	
8244	ZDHHC22	GEO	
8245	ZDHHC6	GEO	
8246	ZDHHC8P	GEO	
8247	ZDHHC9	GEO	
8248	ZFAND2A	GEO	

8249	ZFAND2B	GEO	
8250	ZFAND5	GEO	
8251	ZFAND6	Both	
8252	ZFAT	GAD	
8253	ZFP14	GEO	
8254	ZFP3	GEO	
8255	ZFP36	GEO	
8256	ZFP36L1	GEO	
8257	ZFP37	GEO	
8258	ZFP41	GEO	
8259	ZFP62	GEO	
8260	ZFR2	GEO	
8261	ZFYVE1	GEO	
8262	ZFYVE19	GEO	
8263	ZG16B	GEO	
8264	ZGPAT	GAD	
8265	ZHX1	GEO	
8266	ZIC2	GEO	
8267	ZKSCAN1	GEO	
8268	ZMAT2	GEO	
8269	ZMIZ2	GEO	
8270	ZMPSTE24	GEO	
8271	ZMYM1	GEO	
8272	ZMYM5	GEO	
8273	ZMYND11	GEO	
8274	ZMYND19	GEO	
8275	ZMYND8	GEO	
8276	ZNF101	GEO	
8277	ZNF114	GEO	
8278	ZNF124	GEO	
8279	ZNF133	GEO	
8280	ZNF160	GEO	
8281	ZNF165	GEO	
8282	ZNF18	GEO	
8283	ZNF185	GEO	
8284	ZNF197	GEO	
8285	ZNF204	GEO	
8286	ZNF204P	GEO	
8287	ZNF207	GEO	
8288	ZNF219	GEO	
8289	ZNF223	GEO	
8290	ZNF23	GEO	
8291	ZNF230	GEO	
8292	ZNF235	GEO	
8293	ZNF236	GEO	
8294	ZNF238	GEO	
8295	ZNF248	GEO	
8296	ZNF25	GEO	
8297	ZNF252	GEO	
8298	ZNF253	GEO	

8299	ZNF259	GEO	
8300	ZNF260	GEO	
8301	ZNF264	GEO	
8302	ZNF266	GAD	
8303	ZNF271	GEO	
8304	ZNF273	GEO	
8305	ZNF280B	GEO	
8306	ZNF300	GAD	
8307	ZNF318	GEO	
8308	ZNF321	GEO	
8309	ZNF323	GEO	
8310	ZNF331	GEO	
8311	ZNF33A	GEO	
8312	ZNF33B	GEO	
8313	ZNF35	GEO	
8314	ZNF358	GEO	
8315	ZNF362	GEO	
8316	ZNF364	GEO	
8317	ZNF365	GAD	
8318	ZNF37A	GEO	
8319	ZNF37B	GEO	
8320	ZNF385B	GEO	
8321	ZNF396	GEO	
8322	ZNF409	GEO	
8323	ZNF410	GEO	
8324	ZNF420	GEO	
8325	ZNF432	GEO	
8326	ZNF433	GEO	
8327	ZNF438	GEO	
8328	ZNF440	GEO	
8329	ZNF451	GEO	
8330	ZNF460	GEO	
8331	ZNF468	GEO	
8332	ZNF474	GEO	
8333	ZNF483	GEO	
8334	ZNF488	GEO	
8335	ZNF503	GEO	
8336	ZNF506	GEO	
8337	ZNF511	GEO	
8338	ZNF512	GEO	
8339	ZNF521	GEO	
8340	ZNF525	GEO	
8341	ZNF529	GEO	
8342	ZNF543	GEO	
8343	ZNF557	GEO	
8344	ZNF558	GEO	
8345	ZNF559	GEO	
8346	ZNF568	GEO	
8347	ZNF569	GEO	
8348	ZNF57	GEO	
8349	ZNF587	GEO	
8350	ZNF593	GEO	
8351	ZNF606	GEO	
8352	ZNF608	GEO	
8353	ZNF614	GEO	
8354	ZNF618	GEO	
8355	ZNF638	GEO	
8356	ZNF639	GEO	
8357	ZNF641	GEO	
8358	ZNF649	GEO	
8359	ZNF655	GEO	
8360	ZNF662	GEO	
8361	ZNF677	GEO	
8362	ZNF678	GAD	
8363	ZNF680	GEO	
8364	ZNF7	GEO	
8365	ZNF703	GEO	
8366	ZNF704	GEO	
8367	ZNF707	GEO	
8368	ZNF710	GEO	
8369	ZNF711	GEO	
8370	ZNF721	GEO	
8371	ZNF750	GEO	
8372	ZNF763	GEO	
8373	ZNF771	GEO	
8374	ZNF776	GEO	
8375	ZNF777	GEO	
8376	ZNF781	GEO	
8377	ZNF785	GEO	
8378	ZNF791	GEO	
8379	ZNF800	GEO	
8380	ZNF814	GEO	
8381	ZNF816A	GEO	
8382	ZNF83	GEO	
8383	ZNF830	GEO	
8384	ZNF91	GEO	
8385	ZNHIT4	GEO	
8386	ZNRD1	GEO	
8387	ZP3	GEO	
8388	ZPBP2M	GAD	
8389	ZSCAN20	GEO	
8390	ZSCAN29	GEO	
8391	ZSWIM6	GEO	
8392	ZWILCH	GEO	

Table S8

Index	Concept Name	Gene Symbol	P Value	Fold-change	Reliability
1	A2 immunologic symbol	RECQL	0.034624	2.050608	*
2	A2 immunologic symbol	CASP6	0.032689	2.052381	*
3	A2 immunologic symbol	SDCCAG1	0.032204	2.077786	*
4	A2 immunologic symbol	HPGD	0.019631	2.129045	*
5	A2 immunologic symbol	DHRS8	0.024697	2.177333	*
6	A2 immunologic symbol	LMO6	0.00842	2.274688	*
7	A2 immunologic symbol	PIK3CD	0.024979	2.287016	*
8	A2 immunologic symbol	ARMC8	0.016887	2.395928	*
9	A2 immunologic symbol	LOC442198	0.002815	2.42495	*
10	A2 immunologic symbol	COPS2	0.002843	2.445779	*
11	A2 immunologic symbol	DHRS9	0.017019	2.445886	*
12	A2 immunologic symbol	XRCC4	0.000009	2.4507	*
13	A2 immunologic symbol	SE57-1	0.001417	2.659529	*
14	A2 immunologic symbol	HMGA1	0.015533	3.141493	*
15	A2 immunologic symbol	LXN	0.000021	3.400538	*
16	Adenocarcinoma	KIAA1324L	0	-18.457231	*
17	Adenocarcinoma	C10orf116	0.000079	-17.403378	*
18	Adenocarcinoma	CXXC4	0.011913	-13.989064	*
19	Adenocarcinoma	ETV1	0.000269	-10.047961	*
20	Adenocarcinoma	PEG10	0.000017	-10.000323	*
21	Adenocarcinoma	MYOT	0	-8.832191	*
22	Adenocarcinoma	ABCG2	0.000006	-8.486486	*
23	Adenocarcinoma	ELOVL5	0.002373	-8.192065	*
24	Adenocarcinoma	CHP2	0	-7.890496	*
25	Adenocarcinoma	ADAMDEC1	0	-7.583396	*
26	Adenocarcinoma	STC2	0.001389	-7.084797	*
27	Adenocarcinoma	DMKN	0.000002	-6.451331	*
28	Adenocarcinoma	GCNT2	0.000012	-6.439363	*
29	Adenocarcinoma	CWH43	0.000007	-6.340542	*
30	Adenocarcinoma	PKIB	0	-6.214375	*
31	Adenocarcinoma	SYT17	0.012334	-5.998397	*
32	Adenocarcinoma	PYGL	0.000515	-5.887689	*
33	Adenocarcinoma	CXCL12	0	-5.810221	*
34	Adenocarcinoma	SGCE	0.000049	-5.555721	*
35	Adenocarcinoma	FKBP1B	0.006104	-5.456181	*
36	Adenocarcinoma	ARHGEF10	0.00136	-5.2396	*
37	Adenocarcinoma	GUCA2A	0	-5.139376	*
38	Adenocarcinoma	SYNE2	0.000007	-5.052631	*
39	Adenocarcinoma	FNBP1	0.000342	-4.850535	*
40	Adenocarcinoma	F13A1	0	-4.646816	*
41	Adenocarcinoma	DHRS11	0.000021	-4.552529	*
42	Adenocarcinoma	TERT	0.000343	-4.323769	*
43	Adenocarcinoma	HSD17B2	0.000052	-4.175568	*
44	Adenocarcinoma	UGT2B15	0.000038	-4.136587	*
45	Adenocarcinoma	ABCA8	0	-4.100368	*
46	Adenocarcinoma	AGPAT9	0.000063	-4.032915	*
47	Adenocarcinoma	PADI2	0	-4.009806	*
48	Adenocarcinoma	LRRC19	0.000069	-4.006808	*

49	Adenocarcinoma	ENPP1	0.014929	-3.88051	*
50	Adenocarcinoma	TSPAN7	0	-3.857845	*
51	Adenocarcinoma	HPGD	0.000201	-3.819098	*
52	Adenocarcinoma	FADS1	0.001623	-3.794635	*
53	Adenocarcinoma	AKR1B10	0.000036	-3.779094	*
54	Adenocarcinoma	ARL14	0.000007	-3.700351	*
55	Adenocarcinoma	C17orf51	0.000722	-3.679578	*
56	Adenocarcinoma	CC2D2A	0.001301	-3.660587	*
57	Adenocarcinoma	RUNDC3B	0.000001	-3.626594	*
58	Adenocarcinoma	C10orf99	0.000028	-3.620081	*
59	Adenocarcinoma	HIGD1A	0.000009	-3.524229	*
60	Adenocarcinoma	PAX6	0.011213	-3.414723	*
61	Adenocarcinoma	ZNF575	0.000035	-3.378636	*
62	Adenocarcinoma	FLJ32063	0.000003	-3.350273	*
63	Adenocarcinoma	FABP4	0	-3.34833	*
64	Adenocarcinoma	AHCYL2	0.000007	-3.330288	*
65	Adenocarcinoma	C2orf88	0.000052	-3.296037	*
66	Adenocarcinoma	GALNTL4	0.000002	-3.263114	*
67	Adenocarcinoma	CA12	0.00004	-3.220574	*
68	Adenocarcinoma	PLCE1	0.000012	-3.149416	*
69	Adenocarcinoma	SCGN	0.000292	-3.132719	*
70	Adenocarcinoma	ARHGAP44	0.000001	-3.126782	*
71	Adenocarcinoma	SLC4A4	0.00007	-3.104855	*
72	Adenocarcinoma	FCHO1	0.000743	-3.027181	*
73	Adenocarcinoma	SULT1A2	0.000079	-2.995262	*
74	Adenocarcinoma	DENND2A	0.000061	-2.97707	*
75	Adenocarcinoma	TSPAN1	0.000226	-2.971613	*
76	Adenocarcinoma	ADSSL1	0.000081	-2.839162	*
77	Adenocarcinoma	CLDN23	0.0002	-2.834475	*
78	Adenocarcinoma	LOC400573	0.000123	-2.834344	*
79	Adenocarcinoma	SLC9A2	0	-2.813065	*
80	Adenocarcinoma	MMP28	0.000034	-2.79525	*
81	Adenocarcinoma	RASAL1	0.00043	-2.788003	*
82	Adenocarcinoma	PREP	0.000223	-2.778618	*
83	Adenocarcinoma	TRMT11	0.003115	-2.773075	*
84	Adenocarcinoma	PAQR5	0.000042	-2.689435	*
85	Adenocarcinoma	LOC100287411	0.000004	-2.686546	*
86	Adenocarcinoma	SMTN	0.000006	-2.682277	*
87	Adenocarcinoma	PPP2R3A	0.000044	-2.652001	*
88	Adenocarcinoma	SELENBP1	0.000389	-2.638804	*
89	Adenocarcinoma	NR5A2	0.0003	-2.636983	*
90	Adenocarcinoma	CPM	0.000051	-2.621781	*
91	Adenocarcinoma	FZD9	0.000734	-2.619644	*
92	Adenocarcinoma	HLA-DMA	0.013339	-2.592323	*
93	Adenocarcinoma	SLC4A11	0.002357	-2.551325	*
94	Adenocarcinoma	PAM	0.001986	-2.533144	*
95	Adenocarcinoma	LOC643837	0.002357	-2.510325	*
96	Adenocarcinoma	ACVR1C	0.000001	-2.484118	*
97	Adenocarcinoma	PPID	0.000025	-2.462194	*
98	Adenocarcinoma	GOLM1	0.000252	-2.421836	*

99	Adenocarcinoma	SPPL2A	0.000009	-2.398902	*
100	Adenocarcinoma	NR3C2	0	-2.393348	*
101	Adenocarcinoma	STMN2	0	-2.356428	*
102	Adenocarcinoma	CITED1	0.018098	-2.318043	*
103	Adenocarcinoma	CUZD1	0.018448	-2.286498	*
104	Adenocarcinoma	TUBB3	0.01862	-2.25415	*
105	Adenocarcinoma	FLJ36848	0.000256	-2.20681	*
106	Adenocarcinoma	WASF1	0.003376	-2.193545	*
107	Adenocarcinoma	SULT1A1	0.000014	-2.150609	*
108	Adenocarcinoma	GPRIN1	0.017406	-2.116363	*
109	Adenocarcinoma	ATP5S	0.000026	-2.103186	*
110	Adenocarcinoma	TERF2	0.000034	-2.084908	*
111	Adenocarcinoma	FAM47E	0.00003	-2.074512	*
112	Adenocarcinoma	C6orf35	0.003732	-2.059435	*
113	Adenocarcinoma	FXYD1	0.000047	-2.055213	*
114	Adenocarcinoma	UGDH	0.000001	-2.048215	*
115	Adenocarcinoma	NR4A2	0.000059	-2.023673	*
116	Adenocarcinoma	DZIP3	0.000445	-2.009873	*
117	Adenocarcinoma	SGK2	0.000043	-2.008301	*
118	Adenocarcinoma	LOC100289019	0.000053	-2.00295	*
119	Adenocarcinoma	AQR	0.000296	2.013872	*
120	Adenocarcinoma	TMEM63A	0.008735	2.023791	*
121	Adenocarcinoma	PDXK	0.003521	2.060404	*
122	Adenocarcinoma	TMEM125	0.001162	2.062651	*
123	Adenocarcinoma	ZNF592	0.000427	2.076689	*
124	Adenocarcinoma	PDS5B	0.006445	2.122209	*
125	Adenocarcinoma	FLJ44606	0.000048	2.122325	*
126	Adenocarcinoma	SUSD1	0.000374	2.137045	*
127	Adenocarcinoma	PC	0.006981	2.162074	*
128	Adenocarcinoma	GLB1L2	0.002379	2.201614	*
129	Adenocarcinoma	CDC42BPA	0.006472	2.23118	*
130	Adenocarcinoma	PERLD1	0.000202	2.239739	*
131	Adenocarcinoma	FLJ32255	0.007139	2.25239	*
132	Adenocarcinoma	MYO6	0.005232	2.256995	*
133	Adenocarcinoma	TSHZ1	0.002619	2.258493	*
134	Adenocarcinoma	RAB11FIP4	0.000193	2.259819	*
135	Adenocarcinoma	ERBB2	0.005397	2.261878	*
136	Adenocarcinoma	LITAF	0.001964	2.30379	*
137	Adenocarcinoma	MAP3K1	0.000315	2.351966	*
138	Adenocarcinoma	SFXN5	0.001824	2.360293	*
139	Adenocarcinoma	C7orf41	0.015161	2.370543	*
140	Adenocarcinoma	RAPH1	0.000951	2.451737	*
141	Adenocarcinoma	DEGS2	0.001696	2.452281	*
142	Adenocarcinoma	SEMA4D	0.000714	2.508583	*
143	Adenocarcinoma	MYLIP	0.007311	2.533921	*
144	Adenocarcinoma	ADAL	0.006595	2.538842	*
145	Adenocarcinoma	GNG4	0	2.58884	*
146	Adenocarcinoma	MPZL2	0.008012	2.610388	*
147	Adenocarcinoma	EPB41L1	0.015535	2.634985	*
148	Adenocarcinoma	ZNF846	0.000075	2.640985	*

149	Adenocarcinoma	LRG1	0.000004	2.679377	*
150	Adenocarcinoma	ITGB8	0.002911	2.8341	*
151	Adenocarcinoma	ANXA9	0.002645	2.859143	*
152	Adenocarcinoma	ZNF311	0.000006	2.916672	*
153	Adenocarcinoma	EMP2	0	2.937258	*
154	Adenocarcinoma	C1orf210	0.005073	3.074608	*
155	Adenocarcinoma	CLN6	0.000026	3.107396	*
156	Adenocarcinoma	ILDR1	0.001696	3.135222	*
157	Adenocarcinoma	FLVCR2	0.001619	3.136576	*
158	Adenocarcinoma	ARRB1	0.001064	3.503076	*
159	Adenocarcinoma	SULT2B1	0.002194	3.553501	*
160	Adenocarcinoma	PRSS23	0.000746	3.686045	*
161	Adenocarcinoma	HNF1B	0.000005	3.743232	*
162	Adenocarcinoma	ZNF426	0.004086	3.758404	*
163	Adenocarcinoma	TMPRSS13	0.000011	4.056654	*
164	Adenocarcinoma	RAB27B	0.001103	4.173163	*
165	Adenocarcinoma	CA9	0.007306	4.241781	*
166	Adenocarcinoma	PCSK6	0.000006	4.313309	*
167	Adenocarcinoma	VIPR1	0.002716	4.590326	*
168	Adenocarcinoma	C9orf68	0.000667	4.683553	*
169	Adenocarcinoma	MAP3K8	0.001798	4.862673	*
170	Adenocarcinoma	GPR115	0.000714	5.636709	*
171	Adenocarcinoma	TACSTD2	0.000025	5.877645	*
172	Adenocarcinoma	RASEF	0.015961	7.831564	*
173	Adenocarcinoma	LIMCH1	0.00008	7.831938	*
174	Adenocarcinoma	FRMD4B	0.000042	8.351405	*
175	Adenocarcinoma	ERAP2	0.000144	9.820729	*
176	Adenocarcinoma	C1orf59	0.000653	11.139442	*
177	Adenocarcinoma	GPX3	0.000001	12.302366	*
178	Adenocarcinoma	QPCT	0.000144	43.221112	*
179	Adenocarcinoma of large intestine	MYOT	0	-8.832191	*
180	Adenocarcinoma of large intestine	PLP1	0	-8.565039	*
181	Adenocarcinoma of large intestine	RAVER2	0	2.08924	*
182	Adenocarcinoma of large intestine	STOX2	0	2.16469	*
183	Adenocarcinoma of large intestine	GCNT2	0	2.239828	*
184	Adenocarcinoma of large intestine	SPIB	0	2.38534	*
185	Adenocarcinoma of large intestine	SRI	0	2.528823	*
186	Adenocarcinoma of large intestine	PAQR5	0	2.542337	*
187	Adenocarcinoma of large intestine	PDE9A	0	2.559518	*
188	Adenocarcinoma of large intestine	PLCE1	0	2.627492	*
189	Adenocarcinoma of large intestine	HIGD1A	0	2.629734	*
190	Adenocarcinoma of large intestine	NR5A2	0	2.707146	*
191	Adenocarcinoma of large intestine	STMN2	0	2.790942	*
192	Adenocarcinoma of large intestine	HTR4	0	2.796495	*
193	Adenocarcinoma of large intestine	ACADS	0	2.83351	*
194	Adenocarcinoma of large intestine	SLC25A34	0	2.894841	*
195	Adenocarcinoma of large intestine	ABI3BP	0	3.004368	*
196	Adenocarcinoma of large intestine	UGDH	0	3.083284	*
197	Adenocarcinoma of large intestine	XKR4	0	3.097864	*
198	Adenocarcinoma of large intestine	SCGN	0	3.561313	*

199	Adenocarcinoma of large intestine	GPX3	0	3.748229	*
200	Adenocarcinoma of large intestine	TSPAN7	0	4.352056	*
201	Adenocarcinoma of large intestine	SLC16A9	0	4.611893	*
202	Adenocarcinoma of large intestine	CLDN23	0	4.827579	*
203	Adenocarcinoma of large intestine	ADAMDEC1	0	5.194066	*
204	Adenocarcinoma of large intestine	CXCL12	0	5.943106	*
205	Adenocarcinoma of large intestine	SCARA5	0	6.298371	*
206	Adenocarcinoma of large intestine	OSTbeta	0	6.304743	*
207	Adenocarcinoma of large intestine	SFRP1	0	6.684432	*
208	Adenocarcinoma of large intestine	PKIB	0	6.760776	*
209	Adenocarcinoma of large intestine	MAMDC2	0	7.045446	*
210	Adenocarcinoma of large intestine	HSD17B2	0	7.781235	*
211	Adenocarcinoma of large intestine	ADH1A	0	11.751594	*
212	Adenocarcinoma of large intestine	GUCA2B	0	24.924778	*
213	Adenocarcinoma of large intestine	GUCA2A	0	26.964687	*
214	Adenocarcinoma of large intestine	AQP8	0	44.069335	*
215	adenoma	SLC26A3	0	-38.08636	*
216	adenoma	GUCA2A	0	-28.722896	*
217	adenoma	ABCA8	0	-23.213255	*
218	adenoma	CLCA4	0	-21.193232	*
219	adenoma	CA1	0	-18.850535	*
220	adenoma	CA4	0	-17.421036	*
221	adenoma	CD177	0	-16.277312	*
222	adenoma	PCK1	0	-16.18203	*
223	adenoma	CDKN2B	0	-14.536097	*
224	adenoma	ADAMDEC1	0	-12.069765	*
225	adenoma	GCG	0.000008	-11.58098	*
226	adenoma	CXCL12	0	-11.210708	*
227	adenoma	EDN3	0	-10.750175	*
228	adenoma	AQP8	0	-10.15701	*
229	adenoma	TRPM6	0	-9.613446	*
230	adenoma	ANK2	0	-9.366054	*
231	adenoma	TCF21	0	-9.193144	*
232	adenoma	SLC4A4	0	-9.098508	*
233	adenoma	NEUROD1	0.000008	-8.968304	*
234	adenoma	FBLN1	0	-8.162717	*
235	adenoma	GDPD2	0	-7.595737	*
236	adenoma	CLDN23	0	-7.499048	*
237	adenoma	USP2	0.000001	-7.413002	*
238	adenoma	FXYD1	0	-7.370164	*
239	adenoma	CHGA	0	-7.303062	*
240	adenoma	GUCA2B	0	-7.05847	*
241	adenoma	CA2	0	-6.781211	*
242	adenoma	SEMA6D	0	-6.121416	*
243	adenoma	C2orf88	0	-6.019888	*
244	adenoma	PHLPP2	0	-5.956011	*
245	adenoma	SCARA5	0	-5.907949	*
246	adenoma	ANO5	0	-5.903868	*
247	adenoma	MS4A12	0	-5.885629	*
248	adenoma	NR5A2	0	-5.628608	*

249	adenoma	MEIS1	0	-5.618535	*
250	adenoma	TSPAN7	0	-5.59846	*
251	adenoma	HAPLN1	0	-5.595542	*
252	adenoma	TRPC1	0	-5.588125	*
253	adenoma	CPNE8	0	-5.539105	*
254	adenoma	CP	0	-5.496611	*
255	adenoma	ZG16	0	-5.473881	*
256	adenoma	EDIL3	0	-5.471718	*
257	adenoma	PAPPA	0.000031	-5.221384	*
258	adenoma	NR3C1	0	-5.199333	*
259	adenoma	C14orf139	0	-5.19712	*
260	adenoma	PKIB	0	-5.194257	*
261	adenoma	MYLK	0	-5.191709	*
262	adenoma	IGF1	0	-5.019269	*
263	adenoma	HPGD	0	-4.952038	*
264	adenoma	PAG1	0	-4.888206	*
265	adenoma	TMEM171	0	-4.881186	*
266	adenoma	EPB41L3	0	-4.876504	*
267	adenoma	DHRS11	0	-4.865647	*
268	adenoma	ITIH5	0	-4.713737	*
269	adenoma	LPAR1	0	-4.679545	*
270	adenoma	CEACAM7	0	-4.676861	*
271	adenoma	SCG2	0	-4.649106	*
272	adenoma	NDN	0	-4.581847	*
273	adenoma	RUNDC3B	0	-4.530686	*
274	adenoma	CNNM2	0	-4.530382	*
275	adenoma	SETBP1	0	-4.422574	*
276	adenoma	C11orf96	0.000082	-4.344237	*
277	adenoma	COLEC12	0	-4.307452	*
278	adenoma	TRAF3IP3	0.000018	-4.30323	*
279	adenoma	CES2	0	-4.196009	*
280	adenoma	KCTD12	0	-4.172007	*
281	adenoma	PDE2A	0	-4.168294	*
282	adenoma	TMCC3	0	-4.090314	*
283	adenoma	GPX3	0	-4.07824	*
284	adenoma	RARRES2	0.000004	-3.962436	*
285	adenoma	MPEG1	0.000003	-3.942707	*
286	adenoma	SPARCL1	0	-3.9365	*
287	adenoma	RGMA	0	-3.847742	*
288	adenoma	SNAI2	0	-3.844742	*
289	adenoma	MALL	0	-3.830649	*
290	adenoma	CDH2	0	-3.829926	*
291	adenoma	UNC5C	0	-3.802816	*
292	adenoma	PPP1R16B	0.000004	-3.797815	*
293	adenoma	MGAT4A	0	-3.779783	*
294	adenoma	XKR4	0.000001	-3.770258	*
295	adenoma	CYP1B1	0.000036	-3.743165	*
296	adenoma	AQP1	0	-3.717151	*
297	adenoma	ADAMTS1	0.000015	-3.716882	*
298	adenoma	IL6R	0	-3.695784	*

299	adenoma	LOC344887	0.000002	-3.69458	*
300	adenoma	GPC6	0	-3.69371	*
301	adenoma	DOCK10	0	-3.6903	*
302	adenoma	RASGRP3	0	-3.606281	*
303	adenoma	ZEB2	0	-3.603844	*
304	adenoma	CCL14	0	-3.57784	*
305	adenoma	CLIC5	0	-3.536466	*
306	adenoma	NAAA	0	-3.52473	*
307	adenoma	HIGD1A	0	-3.491594	*
308	adenoma	FGL2	0	-3.475052	*
309	adenoma	RUNX1T1	0	-3.43287	*
310	adenoma	DCN	0	-3.426964	*
311	adenoma	AHCYL2	0	-3.42458	*
312	adenoma	SMPDL3A	0	-3.413709	*
313	adenoma	VCAM1	0.000004	-3.409172	*
314	adenoma	FRMD6	0	-3.373895	*
315	adenoma	FAM107A	0.000004	-3.324142	*
316	adenoma	MAF	0	-3.308581	*
317	adenoma	TIMP2	0	-3.292712	*
318	adenoma	POSTN	0	-3.273193	*
319	adenoma	SEMA6A	0	-3.255544	*
320	adenoma	TP53INP2	0	-3.238175	*
321	adenoma	RAB37	0	-3.23089	*
322	adenoma	CCDC80	0	-3.229232	*
323	adenoma	PRKAR2B	0	-3.223903	*
324	adenoma	PLAC9	0.000002	-3.223752	*
325	adenoma	CMAH	0	-3.208679	*
326	adenoma	MS4A7	0	-3.165402	*
327	adenoma	RELL1	0	-3.162769	*
328	adenoma	RBMS3	0	-3.119037	*
329	adenoma	ZCCHC24	0	-3.100495	*
330	adenoma	SLC46A3	0	-3.09383	*
331	adenoma	PTPRR	0.000004	-3.091864	*
332	adenoma	CNRIP1	0	-3.067872	*
333	adenoma	PDGFRA	0	-3.06471	*
334	adenoma	PLOD2	0	-3.053873	*
335	adenoma	GNB4	0	-3.011228	*
336	adenoma	GIMAP6	0	-3.01085	*
337	adenoma	HDAC9	0	-3.003966	*
338	adenoma	CFH	0	-2.998647	*
339	adenoma	MCC	0	-2.970271	*
340	adenoma	PRDX6	0	-2.956727	*
341	adenoma	DDR2	0	-2.955575	*
342	adenoma	SLC9A9	0	-2.950103	*
343	adenoma	GGTA1	0	-2.9497	*
344	adenoma	TTC22	0	-2.946713	*
345	adenoma	CACNA2D1	0.000001	-2.935371	*
346	adenoma	LOC100506621	0.000001	-2.922869	*
347	adenoma	BGN	0.000001	-2.91178	*
348	adenoma	ADAP2	0.000006	-2.907607	*

349	adenoma	SLC20A1	0.000001	-2.894122	*
350	adenoma	PTGIS	0.000001	-2.890805	*
351	adenoma	LIFR	0.000027	-2.862675	*
352	adenoma	SGK1	0	-2.849016	*
353	adenoma	APOE	0.000001	-2.846108	*
354	adenoma	MRC1	0	-2.839779	*
355	adenoma	STAB1	0	-2.839099	*
356	adenoma	F13A1	0.000005	-2.837147	*
357	adenoma	RDX	0	-2.833988	*
358	adenoma	TNXA	0	-2.832863	*
359	adenoma	CTSZ	0.000004	-2.829326	*
360	adenoma	SNAP25	0.000026	-2.820829	*
361	adenoma	TCF4	0.000003	-2.812337	*
362	adenoma	GNG2	0	-2.792069	*
363	adenoma	CD48	0.00002	-2.756235	*
364	adenoma	CLCN2	0	-2.755245	*
365	adenoma	SYNC	0	-2.707972	*
366	adenoma	AKT3	0	-2.686562	*
367	adenoma	COL13A1	0	-2.675191	*
368	adenoma	ASPN	0.00001	-2.658627	*
369	adenoma	GPNMB	0.000002	-2.653335	*
370	adenoma	SLC30A4	0	-2.646617	*
371	adenoma	RGL1	0	-2.64603	*
372	adenoma	MEF2C	0.000009	-2.629412	*
373	adenoma	C6orf204	0	-2.60794	*
374	adenoma	COL28A1	0.000001	-2.595261	*
375	adenoma	CYGB	0	-2.574414	*
376	adenoma	SSBP2	0	-2.561707	*
377	adenoma	MXD1	0	-2.559257	*
378	adenoma	FNBP1	0	-2.557199	*
379	adenoma	ABCA6	0.000001	-2.55698	*
380	adenoma	TIMP3	0.000001	-2.53521	*
381	adenoma	GIMAP1	0.00001	-2.527932	*
382	adenoma	RAB34	0.000001	-2.511206	*
383	adenoma	SDPR	0	-2.501807	*
384	adenoma	ANTXR1	0	-2.49782	*
385	adenoma	PDLIM3	0.000001	-2.484118	*
386	adenoma	LOX	0.000005	-2.460449	*
387	adenoma	ITM2C	0	-2.45619	*
388	adenoma	SIGLEC1	0.000003	-2.44309	*
389	adenoma	CD163	0.000005	-2.433077	*
390	adenoma	CYS1	0.000004	-2.42959	*
391	adenoma	CSF1R	0.000004	-2.42014	*
392	adenoma	GNA11	0	-2.408995	*
393	adenoma	ZFPM2	0	-2.389381	*
394	adenoma	SLIT2	0	-2.388864	*
395	adenoma	BCL2L11	0	-2.372495	*
396	adenoma	PTGER3	0	-2.372401	*
397	adenoma	CPM	0.000022	-2.370853	*
398	adenoma	EVI2B	0.000026	-2.370211	*

399	adenoma	CD36	0	-2.36807	*
400	adenoma	SLC2A13	0	-2.35371	*
401	adenoma	OLFML1	0	-2.352103	*
402	adenoma	GAS7	0	-2.331612	*
403	adenoma	NRP1	0.000002	-2.319185	*
404	adenoma	TMEM133	0	-2.318497	*
405	adenoma	MYO5A	0.000001	-2.291889	*
406	adenoma	AXL	0.000021	-2.290225	*
407	adenoma	RCAN1	0.000002	-2.289081	*
408	adenoma	GLTP	0	-2.27136	*
409	adenoma	ARHGAP15	0.000001	-2.248758	*
410	adenoma	ZNF542	0.000001	-2.229413	*
411	adenoma	ANKRD6	0.000007	-2.224363	*
412	adenoma	JAZF1	0.000001	-2.210426	*
413	adenoma	FERMT2	0	-2.20204	*
414	adenoma	B3GNT7	0.000007	-2.180997	*
415	adenoma	CYLD	0	-2.180294	*
416	adenoma	UGP2	0	-2.178624	*
417	adenoma	AMOTL1	0.000013	-2.173702	*
418	adenoma	ARMCX1	0.000001	-2.17268	*
419	adenoma	ARHGAP42	0	-2.119791	*
420	adenoma	IGFBP3	0	-2.099543	*
421	adenoma	LOC100127983	0.000008	-2.096915	*
422	adenoma	CLEC2B	0.000009	-2.096686	*
423	adenoma	HLA-DRB1	0.000007	-2.095995	*
424	adenoma	8-Sep	0	-2.09136	*
425	adenoma	ARHGEF6	0.000013	-2.079279	*
426	adenoma	FAM126A	0.000001	-2.078326	*
427	adenoma	TNS1	0.000001	-2.071872	*
428	adenoma	C17orf91	0.000002	-2.0713	*
429	adenoma	AKAP2	0	-2.069514	*
430	adenoma	JAM3	0.000002	-2.051995	*
431	adenoma	FXYD6	0	-2.037228	*
432	adenoma	COL6A2	0	-2.03661	*
433	adenoma	COL3A1	0	-2.027751	*
434	adenoma	ZNF304	0	-2.023245	*
435	adenoma	FN1	0	-2.022209	*
436	adenoma	ECM2	0	-2.01929	*
437	adenoma	DKC1	0	2.10166	*
438	adenoma	GPX2	0	2.225244	*
439	adenoma	ITGA6	0	2.419872	*
440	adenoma	BACE2	0	2.571998	*
441	adenoma	FERMT1	0	2.605975	*
442	adenoma	MET	0	3.407449	*
443	adenoma	NEBL	0	3.427539	*
444	adenoma	ANXA3	0	3.510112	*
445	adenoma	ETV4	0	3.658835	*
446	adenoma	RNF43	0	3.789269	*
447	adenoma	ZNRF3	0	3.957787	*
448	adenoma	SORD	0	3.981631	*

449	adenoma	CD44	0	4.366169	*
450	adenoma	S100P	0	4.459644	*
451	adenoma	GALNT6	0	5.744695	*
452	adenoma	AXIN2	0	6.490564	*
453	beta catenin	KIAA1199	0	-9.475267	*
454	beta catenin	PROX1	0	-8.678827	*
455	beta catenin	FGF20	0	-7.012846	*
456	beta catenin	TNFRSF11B	0	-5.709377	*
457	beta catenin	DCLK1	0	-5.445256	*
458	beta catenin	C4orf46	0	-4.443408	*
459	beta catenin	MYH7B	0	-4.287094	*
460	beta catenin	MAP1LC3B	0	-4.269795	*
461	beta catenin	HNRNPA0	0	-4.257481	*
462	beta catenin	SP5	0	-4.186757	*
463	beta catenin	SYK	0	-4.131503	*
464	beta catenin	NAA30	0	-4.091132	*
465	beta catenin	MAK16	0	-3.859284	*
466	beta catenin	PIGW	0	-3.823781	*
467	beta catenin	PFKFB2	0.000001	-3.665898	*
468	beta catenin	RASA1	0	-3.615429	*
469	beta catenin	LEPROTL1	0	-3.588382	*
470	beta catenin	PNKD	0	-3.522663	*
471	beta catenin	SYNGR3	0.000001	-3.480191	*
472	beta catenin	NXT2	0	-3.361643	*
473	beta catenin	LOC100288413	0	-3.361643	*
474	beta catenin	KIF1B	0	-3.096918	*
475	beta catenin	SCRIB	0	-3.08086	*
476	beta catenin	MAPK1	0	-2.998346	*
477	beta catenin	ASCL2	0	-2.911306	*
478	beta catenin	DOCK11	0	-2.904588	*
479	beta catenin	YRDC	0	-2.901234	*
480	beta catenin	PLEKHA3	0	-2.85304	*
481	beta catenin	AIDA	0	-2.83497	*
482	beta catenin	PPP1R2	0.000001	-2.828427	*
483	beta catenin	NCRNA00118	0	-2.74632	*
484	beta catenin	CDK19	0	-2.724201	*
485	beta catenin	MARVELD2	0	-2.713209	*
486	beta catenin	PLEKHB2	0	-2.655837	*
487	beta catenin	TMEM41B	0	-2.620787	*
488	beta catenin	SLC38A1	0	-2.596677	*
489	beta catenin	NAP1L1	0.000001	-2.549121	*
490	beta catenin	TSPAN5	0	-2.543238	*
491	beta catenin	DYNC1LI2	0	-2.518387	*
492	beta catenin	TOMM22	0.000001	-2.467984	*
493	beta catenin	ETS2	0.000001	-2.452353	*
494	beta catenin	GINS1	0.000001	-2.42839	*
495	beta catenin	RABGAP1	0	-2.413009	*
496	beta catenin	PTRH1	0	-2.408831	*
497	beta catenin	ATG12	0	-2.399111	*
498	beta catenin	FAM105B	0	-2.366082	*

499	beta catenin	TM7SF3	0	-2.308038	*
500	beta catenin	TTL	0	-2.282846	*
501	beta catenin	PLA2G12A	0.000001	-2.269697	*
502	beta catenin	CXCL16	0	-2.265768	*
503	beta catenin	ANTXR1	0	-2.23845	*
504	beta catenin	TNRC6C	0	-2.23845	*
505	beta catenin	SAP130	0	-2.186061	*
506	beta catenin	VPS41	0	-2.184798	*
507	beta catenin	LOC221710	0	-2.149747	*
508	beta catenin	SNX12	0	-2.147265	*
509	beta catenin	HMGN4	0	-2.136131	*
510	beta catenin	UBR7	0.000001	-2.133664	*
511	beta catenin	SAP30L	0	-2.132432	*
512	beta catenin	KIF5B	0	-2.125055	*
513	beta catenin	SNTB1	0	-2.123828	*
514	beta catenin	DNAJC19	0.000001	-2.099433	*
515	beta catenin	CCNF	0.000001	-2.084932	*
516	beta catenin	TSC22D1	0	-2.057416	*
517	beta catenin	PRSS3	0.000001	-2.056228	*
518	beta catenin	PPP1R14C	0	-2.05504	*
519	beta catenin	TMEM127	0.000001	-2.050298	*
520	beta catenin	EXOSC2	0	-2.049114	*
521	beta catenin	PPP1CC	0.000001	-2.008103	*
522	beta catenin	LOC100289410	0.000001	-2.002312	*
523	beta catenin	RBMS2	0.000001	2.001156	*
524	beta catenin	GEM	0	2.010424	*
525	beta catenin	GRK5	0.000001	2.011586	*
526	beta catenin	APBB2	0.000001	2.020903	*
527	beta catenin	OLFML2A	0	2.03261	*
528	beta catenin	SH3TC2	0.000001	2.038489	*
529	beta catenin	ARL6IP5	0	2.044385	*
530	beta catenin	PPL	0	2.045566	*
531	beta catenin	CHST6	0.000001	2.049114	*
532	beta catenin	PYCARD	0	2.051482	*
533	beta catenin	UBE2H	0	2.074121	*
534	beta catenin	PQLC3	0	2.105506	*
535	beta catenin	SLC22A4	0	2.110376	*
536	beta catenin	KRT80	0	2.133664	*
537	beta catenin	GLIS2	0	2.141072	*
538	beta catenin	PLA2G16	0	2.148505	*
539	beta catenin	PLD1	0	2.159704	*
540	beta catenin	PRSS23	0	2.160951	*
541	beta catenin	MICAL2	0	2.168454	*
542	beta catenin	C17orf91	0	2.175982	*
543	beta catenin	LOXL4	0.000001	2.175982	*
544	beta catenin	ZNF467	0.000001	2.201266	*
545	beta catenin	NAV1	0	2.215297	*
546	beta catenin	ANKRD46	0	2.241037	*
547	beta catenin	SYTL2	0	2.247519	*
548	beta catenin	GPRC5A	0	2.257929	*

549	beta catenin	FNIP2	0	2.292095	*
550	beta catenin	CTAGE5	0	2.337554	*
551	beta catenin	MAML3	0.000001	2.348381	*
552	beta catenin	ASB9	0	2.390811	*
553	beta catenin	ZEB1	0	2.400497	*
554	beta catenin	CSF2	0	2.403272	*
555	beta catenin	FLJ39051	0.000001	2.414403	*
556	beta catenin	CD14	0	2.418591	*
557	beta catenin	PLAUR	0	2.436821	*
558	beta catenin	MGC39372	0	2.438228	*
559	beta catenin	ABCC3	0	2.456606	*
560	beta catenin	STRADB	0	2.459446	*
561	beta catenin	AHNAK2	0	2.462289	*
562	beta catenin	SYNPO	0.000001	2.486587	*
563	beta catenin	TXNIP	0	2.522755	*
564	beta catenin	LIPG	0.000001	2.587693	*
565	beta catenin	C10orf116	0.000001	2.592181	*
566	beta catenin	LACTB	0.000001	2.623816	*
567	beta catenin	FHL1	0	2.8481	*
568	beta catenin	ELFN2	0.000001	2.921414	*
569	beta catenin	PRICKLE1	0	2.946836	*
570	beta catenin	PTGER2	0	3.068429	*
571	beta catenin	ZMIZ1	0	3.123868	*
572	beta catenin	CLIC3	0	3.138336	*
573	beta catenin	ARHGAP42	0	3.154694	*
574	beta catenin	CD70	0	3.330718	*
575	beta catenin	DENND5B	0	3.400704	*
576	beta catenin	FAM20C	0	3.418429	*
577	beta catenin	ZCCHC24	0	3.452162	*
578	beta catenin	TMEM158	0	3.54307	*
579	beta catenin	RASSF4	0	3.682877	*
580	beta catenin	TNFSF9	0	3.695663	*
581	beta catenin	RTEL1	0	3.808352	*
582	beta catenin	IFIT1	0	3.828201	*
583	beta catenin	RARRES2	0	3.899619	*
584	beta catenin	CFL2	0.000001	3.899619	*
585	beta catenin	CREG2	0.000001	3.917681	*
586	beta catenin	LOC399959	0	3.924476	*
587	beta catenin	KRT86	0	3.940376	*
588	beta catenin	TRIM22	0	4.351964	*
589	beta catenin	TMEM133	0	4.356995	*
590	beta catenin	ATP8A1	0	4.484664	*
591	beta catenin	LOC654433	0	4.642816	*
592	beta catenin	CNIH3	0	5.429553	*
593	beta catenin	LAPTM5	0	6.607785	*
594	beta catenin	CCL7	0	7.559723	*
595	beta catenin	TIMP3	0	8.544523	*
596	beta catenin	SPARC	0	12.809717	*
597	Beta Catenin Staining Method	KIAA1199	0	-9.475267	*
598	Beta Catenin Staining Method	PROX1	0	-8.678827	*

599	Beta Catenin Staining Method	FGF20	0	-7.012846	*
600	Beta Catenin Staining Method	TNFRSF11B	0	-5.709377	*
601	Beta Catenin Staining Method	DCLK1	0	-5.445256	*
602	Beta Catenin Staining Method	C4orf46	0	-4.443408	*
603	Beta Catenin Staining Method	MYH7B	0	-4.287094	*
604	Beta Catenin Staining Method	MAP1LC3B	0	-4.269795	*
605	Beta Catenin Staining Method	HNRNPA0	0	-4.257481	*
606	Beta Catenin Staining Method	SP5	0	-4.186757	*
607	Beta Catenin Staining Method	SYK	0	-4.131503	*
608	Beta Catenin Staining Method	NAA30	0	-4.091132	*
609	Beta Catenin Staining Method	MAK16	0	-3.859284	*
610	Beta Catenin Staining Method	PIGW	0	-3.823781	*
611	Beta Catenin Staining Method	PFKFB2	0.000001	-3.665898	*
612	Beta Catenin Staining Method	RASA1	0	-3.615429	*
613	Beta Catenin Staining Method	LEPROTL1	0	-3.588382	*
614	Beta Catenin Staining Method	PNKD	0	-3.522663	*
615	Beta Catenin Staining Method	SYNGR3	0.000001	-3.480191	*
616	Beta Catenin Staining Method	NXT2	0	-3.361643	*
617	Beta Catenin Staining Method	LOC100288413	0	-3.361643	*
618	Beta Catenin Staining Method	KIF1B	0	-3.096918	*
619	Beta Catenin Staining Method	SCRIB	0	-3.08086	*
620	Beta Catenin Staining Method	MAPK1	0	-2.998346	*
621	Beta Catenin Staining Method	ASCL2	0	-2.911306	*
622	Beta Catenin Staining Method	DOCK11	0	-2.904588	*
623	Beta Catenin Staining Method	YRDC	0	-2.901234	*
624	Beta Catenin Staining Method	PLEKHA3	0	-2.85304	*
625	Beta Catenin Staining Method	AIDA	0	-2.83497	*
626	Beta Catenin Staining Method	PPP1R2	0.000001	-2.828427	*
627	Beta Catenin Staining Method	NCRNA00118	0	-2.74632	*
628	Beta Catenin Staining Method	CDK19	0	-2.724201	*
629	Beta Catenin Staining Method	MARVELD2	0	-2.713209	*
630	Beta Catenin Staining Method	PLEKHB2	0	-2.655837	*
631	Beta Catenin Staining Method	TMEM41B	0	-2.620787	*
632	Beta Catenin Staining Method	SLC38A1	0	-2.596677	*
633	Beta Catenin Staining Method	NAP1L1	0.000001	-2.549121	*
634	Beta Catenin Staining Method	TSPAN5	0	-2.543238	*
635	Beta Catenin Staining Method	DYNC1LI2	0	-2.518387	*
636	Beta Catenin Staining Method	TOMM22	0.000001	-2.467984	*
637	Beta Catenin Staining Method	ETS2	0.000001	-2.452353	*
638	Beta Catenin Staining Method	GINS1	0.000001	-2.42839	*
639	Beta Catenin Staining Method	RABGAP1	0	-2.413009	*
640	Beta Catenin Staining Method	PTRH1	0	-2.408831	*
641	Beta Catenin Staining Method	ATG12	0	-2.399111	*
642	Beta Catenin Staining Method	FAM105B	0	-2.366082	*
643	Beta Catenin Staining Method	TM7SF3	0	-2.308038	*
644	Beta Catenin Staining Method	TTL	0	-2.282846	*
645	Beta Catenin Staining Method	PLA2G12A	0.000001	-2.269697	*
646	Beta Catenin Staining Method	CXCL16	0	-2.265768	*
647	Beta Catenin Staining Method	ANTXR1	0	-2.23845	*
648	Beta Catenin Staining Method	TNRC6C	0	-2.23845	*

649	Beta Catenin Staining Method	SAP130	0	-2.186061	*
650	Beta Catenin Staining Method	VPS41	0	-2.184798	*
651	Beta Catenin Staining Method	LOC221710	0	-2.149747	*
652	Beta Catenin Staining Method	SNX12	0	-2.147265	*
653	Beta Catenin Staining Method	HMGN4	0	-2.136131	*
654	Beta Catenin Staining Method	UBR7	0.000001	-2.133664	*
655	Beta Catenin Staining Method	SAP30L	0	-2.132432	*
656	Beta Catenin Staining Method	KIF5B	0	-2.125055	*
657	Beta Catenin Staining Method	SNTB1	0	-2.123828	*
658	Beta Catenin Staining Method	DNAJC19	0.000001	-2.099433	*
659	Beta Catenin Staining Method	CCNF	0.000001	-2.084932	*
660	Beta Catenin Staining Method	TSC22D1	0	-2.057416	*
661	Beta Catenin Staining Method	PRSS3	0.000001	-2.056228	*
662	Beta Catenin Staining Method	PPP1R14C	0	-2.05504	*
663	Beta Catenin Staining Method	TMEM127	0.000001	-2.050298	*
664	Beta Catenin Staining Method	EXOSC2	0	-2.049114	*
665	Beta Catenin Staining Method	PPP1CC	0.000001	-2.008103	*
666	Beta Catenin Staining Method	LOC100289410	0.000001	-2.002312	*
667	Beta Catenin Staining Method	RBMS2	0.000001	2.001156	*
668	Beta Catenin Staining Method	GEM	0	2.010424	*
669	Beta Catenin Staining Method	GRK5	0.000001	2.011586	*
670	Beta Catenin Staining Method	APBB2	0.000001	2.020903	*
671	Beta Catenin Staining Method	OLFML2A	0	2.03261	*
672	Beta Catenin Staining Method	SH3TC2	0.000001	2.038489	*
673	Beta Catenin Staining Method	ARL6IP5	0	2.044385	*
674	Beta Catenin Staining Method	PPL	0	2.045566	*
675	Beta Catenin Staining Method	CHST6	0.000001	2.049114	*
676	Beta Catenin Staining Method	PYCARD	0	2.051482	*
677	Beta Catenin Staining Method	UBE2H	0	2.074121	*
678	Beta Catenin Staining Method	PQLC3	0	2.105506	*
679	Beta Catenin Staining Method	SLC22A4	0	2.110376	*
680	Beta Catenin Staining Method	KRT80	0	2.133664	*
681	Beta Catenin Staining Method	GLIS2	0	2.141072	*
682	Beta Catenin Staining Method	PLA2G16	0	2.148505	*
683	Beta Catenin Staining Method	PLD1	0	2.159704	*
684	Beta Catenin Staining Method	PRSS23	0	2.160951	*
685	Beta Catenin Staining Method	MICAL2	0	2.168454	*
686	Beta Catenin Staining Method	C17orf91	0	2.175982	*
687	Beta Catenin Staining Method	LOXL4	0.000001	2.175982	*
688	Beta Catenin Staining Method	ZNF467	0.000001	2.201266	*
689	Beta Catenin Staining Method	NAV1	0	2.215297	*
690	Beta Catenin Staining Method	ANKRD46	0	2.241037	*
691	Beta Catenin Staining Method	SYTL2	0	2.247519	*
692	Beta Catenin Staining Method	GPRC5A	0	2.257929	*
693	Beta Catenin Staining Method	FNIP2	0	2.292095	*
694	Beta Catenin Staining Method	CTAGE5	0	2.337554	*
695	Beta Catenin Staining Method	MAML3	0.000001	2.348381	*
696	Beta Catenin Staining Method	ASB9	0	2.390811	*
697	Beta Catenin Staining Method	ZEB1	0	2.400497	*
698	Beta Catenin Staining Method	CSF2	0	2.403272	*

699	Beta Catenin Staining Method	FLJ39051	0.000001	2.414403	*
700	Beta Catenin Staining Method	CD14	0	2.418591	*
701	Beta Catenin Staining Method	PLAUR	0	2.436821	*
702	Beta Catenin Staining Method	MGC39372	0	2.438228	*
703	Beta Catenin Staining Method	ABCC3	0	2.456606	*
704	Beta Catenin Staining Method	STRADB	0	2.459446	*
705	Beta Catenin Staining Method	AHNAK2	0	2.462289	*
706	Beta Catenin Staining Method	SYNPO	0.000001	2.486587	*
707	Beta Catenin Staining Method	TXNIP	0	2.522755	*
708	Beta Catenin Staining Method	LIPG	0.000001	2.587693	*
709	Beta Catenin Staining Method	C10orf116	0.000001	2.592181	*
710	Beta Catenin Staining Method	LACTB	0.000001	2.623816	*
711	Beta Catenin Staining Method	FHL1	0	2.8481	*
712	Beta Catenin Staining Method	ELFN2	0.000001	2.921414	*
713	Beta Catenin Staining Method	PRICKLE1	0	2.946836	*
714	Beta Catenin Staining Method	PTGER2	0	3.068429	*
715	Beta Catenin Staining Method	ZMIZ1	0	3.123868	*
716	Beta Catenin Staining Method	CLIC3	0	3.138336	*
717	Beta Catenin Staining Method	ARHGAP42	0	3.154694	*
718	Beta Catenin Staining Method	CD70	0	3.330718	*
719	Beta Catenin Staining Method	DENND5B	0	3.400704	*
720	Beta Catenin Staining Method	FAM20C	0	3.418429	*
721	Beta Catenin Staining Method	ZCCHC24	0	3.452162	*
722	Beta Catenin Staining Method	TMEM158	0	3.54307	*
723	Beta Catenin Staining Method	RASSF4	0	3.682877	*
724	Beta Catenin Staining Method	TNFSF9	0	3.695663	*
725	Beta Catenin Staining Method	RTEL1	0	3.808352	*
726	Beta Catenin Staining Method	IFIT1	0	3.828201	*
727	Beta Catenin Staining Method	RARRES2	0	3.899619	*
728	Beta Catenin Staining Method	CFL2	0.000001	3.899619	*
729	Beta Catenin Staining Method	CREG2	0.000001	3.917681	*
730	Beta Catenin Staining Method	LOC399959	0	3.924476	*
731	Beta Catenin Staining Method	KRT86	0	3.940376	*
732	Beta Catenin Staining Method	TRIM22	0	4.351964	*
733	Beta Catenin Staining Method	TMEM133	0	4.356995	*
734	Beta Catenin Staining Method	ATP8A1	0	4.484664	*
735	Beta Catenin Staining Method	LOC654433	0	4.642816	*
736	Beta Catenin Staining Method	CNIH3	0	5.429553	*
737	Beta Catenin Staining Method	LAPTM5	0	6.607785	*
738	Beta Catenin Staining Method	CCL7	0	7.559723	*
739	Beta Catenin Staining Method	TIMP3	0	8.544523	*
740	Beta Catenin Staining Method	SPARC	0	12.809717	*
741	Cancer of Intestines	IL1R2	0.02893	-1.828304	*
742	Cancer of Intestines	ACSL5	0.031822	-1.638764	*
743	Cancer of Intestines	ARRDC1	0.040085	-1.527261	*
744	Cancer of Intestines	MMP14	0.031822	-1.500352	*
745	Carcinoma	SERTAD4	0.002393	-26.609207	*
746	Carcinoma	FBP1	0.000005	-22.826068	*
747	Carcinoma	ARSE	0.002025	-12.861074	*
748	Carcinoma	ERAP2	0	-9.886786	*

749	Carcinoma	CACNA1D	0	-9.810036	*
750	Carcinoma	DAPK1	0.000614	-9.535901	*
751	Carcinoma	RARRES1	0.010559	-9.419662	*
752	Carcinoma	ZNF702P	0.000307	-8.758415	*
753	Carcinoma	HOXA13	0.00048	-8.215634	*
754	Carcinoma	ANKRD22	0.017211	-7.7229	*
755	Carcinoma	AKR1C1	0.000001	-7.260831	*
756	Carcinoma	DHRS9	0.000035	-7.206478	*
757	Carcinoma	DEGS2	0.000299	-5.971163	*
758	Carcinoma	FOXA1	0.006118	-5.809477	*
759	Carcinoma	REPS2	0.003467	-5.700731	*
760	Carcinoma	AIM1	0.000025	-5.693764	*
761	Carcinoma	NEDD9	0.001359	-5.574342	*
762	Carcinoma	CYP1B1	0.00005	-4.808267	*
763	Carcinoma	RAB27B	0.006599	-4.788443	*
764	Carcinoma	ARSJ	0.0003	-4.71256	*
765	Carcinoma	RASEF	0.00048	-4.566345	*
766	Carcinoma	EPB41L1	0.002224	-4.503736	*
767	Carcinoma	LNX1	0.0029	-4.119568	*
768	Carcinoma	HIPK2	0.00007	-4.108589	*
769	Carcinoma	LIMCH1	0.015341	-4.042084	*
770	Carcinoma	PRSS23	0.006367	-4.011224	*
771	Carcinoma	PLCE1	0.000049	-3.826154	*
772	Carcinoma	CA12	0.011739	-3.807353	*
773	Carcinoma	BARX2	0.004206	-3.6405	*
774	Carcinoma	PIK3CD	0.000059	-3.561643	*
775	Carcinoma	ATP2C2	0.020803	-3.550565	*
776	Carcinoma	NR2F2	0.000006	-3.433272	*
777	Carcinoma	GPR160	0.00235	-3.364098	*
778	Carcinoma	LAMB1	0.002	-3.170907	*
779	Carcinoma	PIK3AP1	0.014261	-3.129655	*
780	Carcinoma	ABCC2	0.000588	-3.091683	*
781	Carcinoma	MPZL2	0.000008	-3.032397	*
782	Carcinoma	GNG4	0	-2.988227	*
783	Carcinoma	PBX3	0.011198	-2.953107	*
784	Carcinoma	NPNT	0.011749	-2.875041	*
785	Carcinoma	SYNE2	0.008076	-2.850088	*
786	Carcinoma	BSPRY	0.003115	-2.764997	*
787	Carcinoma	MAN2A1	0.000057	-2.752675	*
788	Carcinoma	ACSS1	0.003523	-2.672372	*
789	Carcinoma	ZNF586	0.000003	-2.663323	*
790	Carcinoma	SLC27A2	0.00009	-2.651214	*
791	Carcinoma	EEF1A2	0.00007	-2.613331	*
792	Carcinoma	GRHL1	0.000182	-2.601681	*
793	Carcinoma	STAMBPL1	0.006244	-2.502025	*
794	Carcinoma	ARL4C	0.000043	-2.461046	*
795	Carcinoma	SYTL2	0.003519	-2.440433	*
796	Carcinoma	KIAA1217	0.00013	-2.403687	*
797	Carcinoma	EREG	0	-2.395042	*
798	Carcinoma	PDS5B	0.018761	-2.371514	*

799	Carcinoma	XBP1	0.011529	-2.366994	*
800	Carcinoma	EMP2	0.000633	-2.317463	*
801	Carcinoma	SERPINB1	0.001463	-2.266946	*
802	Carcinoma	RAPH1	0.00001	-2.239885	*
803	Carcinoma	RAB11FIP4	0.002325	-2.172103	*
804	Carcinoma	QSOX1	0.002393	-2.168546	*
805	Carcinoma	ELL3	0.00193	-2.108727	*
806	Carcinoma	SLC44A1	0.000174	-2.106095	*
807	Carcinoma	INSIG2	0.002839	-2.09161	*
808	Carcinoma	DMXL2	0.000795	-2.063702	*
809	Carcinoma	PIK3R1	0.00118	-2.043013	*
810	Carcinoma	KIAA1370	0.012881	-2.039845	*
811	Carcinoma	C7orf44	0.009816	-2.039357	*
812	Carcinoma	SIGLEC6	0.000067	-2.024289	*
813	Carcinoma	TMEM63A	0.013614	-2.022844	*
814	Carcinoma	AACS	0.002463	-2.021075	*
815	Carcinoma	FAM129B	0.018431	-2.008576	*
816	Carcinoma	OSBPL2	0.001874	-2.000963	*
817	Carcinoma	FSCN1	0.000056	2.003697	*
818	Carcinoma	SMTN	0.000796	2.023584	*
819	Carcinoma	TRMT11	0.001825	2.024834	*
820	Carcinoma	SLC4A11	0.000001	2.054373	*
821	Carcinoma	ADPRHL1	0.004315	2.109918	*
822	Carcinoma	DPY19L1	0.019895	2.154546	*
823	Carcinoma	ANUBL1	0.012704	2.154727	*
824	Carcinoma	SLC26A6	0.000209	2.169796	*
825	Carcinoma	COL1A1	0.010699	2.170064	*
826	Carcinoma	KCNH2	0.000327	2.170806	*
827	Carcinoma	TERF2	0.000108	2.317082	*
828	Carcinoma	PCOLCE2	0.002315	2.320568	*
829	Carcinoma	EPM2A	0.00542	2.36856	*
830	Carcinoma	MAPK12	0.002018	2.415846	*
831	Carcinoma	HLA-DMA	0.003255	2.453573	*
832	Carcinoma	GPRIN1	0.000188	2.520089	*
833	Carcinoma	DST	0.002839	2.577493	*
834	Carcinoma	ZNF280C	0.000169	2.639105	*
835	Carcinoma	LMO2	0.003088	2.727032	*
836	Carcinoma	GALNTL4	0.002898	2.729237	*
837	Carcinoma	WASF1	0.004814	2.804322	*
838	Carcinoma	TMEFF1	0.010507	2.806665	*
839	Carcinoma	TUBB3	0.000112	2.840619	*
840	Carcinoma	SDC3	0.000336	3.082092	*
841	Carcinoma	RASAL1	0.000162	3.090275	*
842	Carcinoma	ARHGEF10	0.001683	3.10512	*
843	Carcinoma	RCOR2	0.000653	3.169315	*
844	Carcinoma	SMARCA1	0.000189	3.232357	*
845	Carcinoma	AADAT	0.000842	3.273583	*
846	Carcinoma	ADSSL1	0.000648	3.275167	*
847	Carcinoma	CDKN1C	0.000089	3.298703	*
848	Carcinoma	L3MBTL3	0.002877	3.366847	*

849	Carcinoma	CC2D2A	0.010947	3.518482	*
850	Carcinoma	C17orf51	0.001754	3.522841	*
851	Carcinoma	COL9A3	0.001359	3.615701	*
852	Carcinoma	STMN3	0.002926	3.825362	*
853	Carcinoma	FGD1	0.000414	3.826284	*
854	Carcinoma	CUZD1	0.003313	3.900527	*
855	Carcinoma	SNAI1	0.001507	3.985514	*
856	Carcinoma	MID1	0.003952	4.048901	*
857	Carcinoma	PCLO	0.000001	4.074492	*
858	Carcinoma	ZNF350	0.000692	4.085018	*
859	Carcinoma	SLC38A5	0.000005	4.327239	*
860	Carcinoma	MAP7D3	0.000039	4.543284	*
861	Carcinoma	KIAA1324L	0.002761	4.677679	*
862	Carcinoma	ZNF512B	0.000083	5.376174	*
863	Carcinoma	MSLN	0.001306	5.394888	*
864	Carcinoma	FADS1	0.000174	5.544922	*
865	Carcinoma	ZNF134	0.000012	5.94454	*
866	Carcinoma	FAM101B	0.006891	6.088541	*
867	Carcinoma	MAP1B	0.001076	6.681865	*
868	Carcinoma	NBEA	0.000129	6.980152	*
869	Carcinoma	RRAGD	0.002877	8.080971	*
870	Carcinoma	ZNF75A	0.000001	10.526195	*
871	Carcinoma	TCEA2	0.000204	11.266432	*
872	Carcinoma	C1orf61	0.001569	18.455713	*
873	Carcinoma	COL6A1	0.000299	36.33223	*
874	Carcinoma	STC2	0	64.421292	*
875	Carcinoma of the Large Intestine	CA1	0	-70.177195	*
876	Carcinoma of the Large Intestine	AGR3	0.000472	-58.557365	*
877	Carcinoma of the Large Intestine	CLCA1	0	-33.172078	*
878	Carcinoma of the Large Intestine	SLC26A3	0	-32.059519	*
879	Carcinoma of the Large Intestine	ADH1C	0	-17.399607	*
880	Carcinoma of the Large Intestine	GUCA2A	0	-16.294008	*
881	Carcinoma of the Large Intestine	CA2	0	-16.253863	*
882	Carcinoma of the Large Intestine	FAM55D	0	-15.875662	*
883	Carcinoma of the Large Intestine	HEPACAM2	0	-13.446451	*
884	Carcinoma of the Large Intestine	MYH11	0.000001	-12.464317	*
885	Carcinoma of the Large Intestine	FCGBP	0	-11.435381	*
886	Carcinoma of the Large Intestine	IGJ	0	-9.782349	*
887	Carcinoma of the Large Intestine	PIGR	0.00014	-8.779625	*
888	Carcinoma of the Large Intestine	CA12	0.000136	-5.903099	*
889	Carcinoma of the Large Intestine	ST6GALNAC1	0	-4.4897	*
890	Carcinoma of the Large Intestine	VSIG2	0.000043	-4.221688	*
891	Carcinoma of the Large Intestine	PLA2G10	0	-3.722207	*
892	Carcinoma of the Large Intestine	TSPAN1	0.000001	-3.277753	*
893	Carcinoma of the Large Intestine	ITM2C	0.000001	-2.872164	*
894	Carcinoma of the Large Intestine	C6orf105	0.000115	-2.77523	*
895	Carcinoma of the Large Intestine	CAPN9	0.000016	-2.643441	*
896	Carcinoma of the Large Intestine	AGR2	0.000531	-2.405779	*
897	Carcinoma of the Large Intestine	ABP1	0.000179	-2.385219	*
898	Carcinoma of the Large Intestine	MYO1A	0.000642	-2.309044	*

899	Carcinoma of the Large Intestine	BCAS1	0.000006	-2.292699	*
900	Carcinoma of the Large Intestine	SCNN1B	0.000177	-2.287546	*
901	Carcinoma of the Large Intestine	FAM46C	0.000021	-2.273251	*
902	Carcinoma of the Large Intestine	FRZB	0.000071	-2.263728	*
903	Carcinoma of the Large Intestine	BTNL3	0.000071	-2.24713	*
904	Carcinoma of the Large Intestine	LPAR1	0	-2.101231	*
905	Carcinoma of the Large Intestine	LGALS4	0.000004	-2.055385	*
906	Carcinoma of the Large Intestine	IL1R2	0.02893	-1.828304	*
907	Carcinoma of the Large Intestine	ACSL5	0.031822	-1.638764	*
908	Carcinoma of the Large Intestine	ARRDC1	0.040085	-1.527261	*
909	Carcinoma of the Large Intestine	ZNF282	0	2.036182	*
910	Carcinoma of the Large Intestine	SET	0	2.042096	*
911	Carcinoma of the Large Intestine	ITIH2	0	2.398352	*
912	Carcinoma of the Large Intestine	LTBP2	0	2.53017	*
913	Carcinoma of the Large Intestine	EEPD1	0	2.633427	*
914	Carcinoma of the Large Intestine	FAM188B	0	2.650885	*
915	Carcinoma of the Large Intestine	C7orf68	0.000001	2.713761	*
916	Carcinoma of the Large Intestine	TOP1MT	0	2.723722	*
917	Carcinoma of the Large Intestine	TCOF1	0	2.727922	*
918	Carcinoma of the Large Intestine	GPR176	0	2.759087	*
919	Carcinoma of the Large Intestine	HABP2	0	2.896576	*
920	Carcinoma of the Large Intestine	GRB10	0	2.927995	*
921	Carcinoma of the Large Intestine	SLC41A1	0	2.968926	*
922	Carcinoma of the Large Intestine	PPEF1	0	2.974042	*
923	Carcinoma of the Large Intestine	SGIP1	0	3.035792	*
924	Carcinoma of the Large Intestine	SOX4	0	3.075368	*
925	Carcinoma of the Large Intestine	GDPD5	0	3.12273	*
926	Carcinoma of the Large Intestine	KIAA1257	0	3.140853	*
927	Carcinoma of the Large Intestine	SEC14L2	0	3.188415	*
928	Carcinoma of the Large Intestine	DNAH5	0	3.204015	*
929	Carcinoma of the Large Intestine	PDE10A	0	3.215748	*
930	Carcinoma of the Large Intestine	MMP14	0	3.249611	*
931	Carcinoma of the Large Intestine	CDKN2A	0	3.262812	*
932	Carcinoma of the Large Intestine	RNF32	0	3.26672	*
933	Carcinoma of the Large Intestine	SLC9A7	0	3.2869	*
934	Carcinoma of the Large Intestine	ITGA11	0	3.303535	*
935	Carcinoma of the Large Intestine	ADAMTS2	0	3.332996	*
936	Carcinoma of the Large Intestine	P4HA3	0	3.358196	*
937	Carcinoma of the Large Intestine	BFSP1	0	3.394537	*
938	Carcinoma of the Large Intestine	LPCAT1	0	3.398193	*
939	Carcinoma of the Large Intestine	TCF7	0	3.484109	*
940	Carcinoma of the Large Intestine	IRAK2	0	3.526085	*
941	Carcinoma of the Large Intestine	ADAMTS6	0	3.581777	*
942	Carcinoma of the Large Intestine	GLS2	0	3.614199	*
943	Carcinoma of the Large Intestine	ALDH3B2	0	3.771741	*
944	Carcinoma of the Large Intestine	HS6ST2	0	3.806945	*
945	Carcinoma of the Large Intestine	TBX20	0	3.863409	*
946	Carcinoma of the Large Intestine	HAPLN3	0	3.864986	*
947	Carcinoma of the Large Intestine	LEPRE1	0	3.929111	*
948	Carcinoma of the Large Intestine	FAM150A	0	3.955729	*

949	Carcinoma of the Large Intestine	CSGALNACT1	0	3.983069	*
950	Carcinoma of the Large Intestine	VWA2	0	3.985745	*
951	Carcinoma of the Large Intestine	LRRC34	0	4.101056	*
952	Carcinoma of the Large Intestine	PJA1	0	4.103211	*
953	Carcinoma of the Large Intestine	GAS2	0	4.130475	*
954	Carcinoma of the Large Intestine	PTP4A3	0	4.198752	*
955	Carcinoma of the Large Intestine	ACSL6	0	4.21929	*
956	Carcinoma of the Large Intestine	C1orf135	0	4.242459	*
957	Carcinoma of the Large Intestine	SNTB1	0	4.273359	*
958	Carcinoma of the Large Intestine	TNFSF11	0	4.302181	*
959	Carcinoma of the Large Intestine	MTHFD1L	0	4.380449	*
960	Carcinoma of the Large Intestine	STC1	0	4.395318	*
961	Carcinoma of the Large Intestine	ODAM	0	4.447008	*
962	Carcinoma of the Large Intestine	WDR66	0	4.447168	*
963	Carcinoma of the Large Intestine	CSF2	0	4.465932	*
964	Carcinoma of the Large Intestine	PAH	0	4.501848	*
965	Carcinoma of the Large Intestine	LOXL2	0	4.528196	*
966	Carcinoma of the Large Intestine	NEK5	0	4.545746	*
967	Carcinoma of the Large Intestine	GRHL1	0	4.760864	*
968	Carcinoma of the Large Intestine	PLAU	0	4.841344	*
969	Carcinoma of the Large Intestine	DIO2	0	4.877771	*
970	Carcinoma of the Large Intestine	HCN1	0	4.993712	*
971	Carcinoma of the Large Intestine	ICOS	0	5.13095	*
972	Carcinoma of the Large Intestine	CYP39A1	0	5.382667	*
973	Carcinoma of the Large Intestine	CDH6	0	5.400039	*
974	Carcinoma of the Large Intestine	GRIN2B	0	5.605313	*
975	Carcinoma of the Large Intestine	PDPN	0	5.623195	*
976	Carcinoma of the Large Intestine	EGFL6	0	5.80168	*
977	Carcinoma of the Large Intestine	ANGPT2	0	5.811083	*
978	Carcinoma of the Large Intestine	ADAMTS12	0	6.346981	*
979	Carcinoma of the Large Intestine	KRT80	0	6.582092	*
980	Carcinoma of the Large Intestine	JUB	0	6.6753	*
981	Carcinoma of the Large Intestine	MFAP2	0	6.758639	*
982	Carcinoma of the Large Intestine	COL11A1	0	7.090537	*
983	Carcinoma of the Large Intestine	SPP1	0	7.465531	*
984	Carcinoma of the Large Intestine	ESM1	0	7.595544	*
985	Carcinoma of the Large Intestine	COL10A1	0	7.669827	*
986	Carcinoma of the Large Intestine	LEMD1	0	7.698407	*
987	Carcinoma of the Large Intestine	INHBA	0	7.715281	*
988	Carcinoma of the Large Intestine	KAL1	0	8.67666	*
989	Carcinoma of the Large Intestine	ADAM12	0	9.08807	*
990	Carcinoma of the Large Intestine	KIAA1199	0	10.5275	*
991	Carcinoma of the Large Intestine	CHI3L1	0	10.717129	*
992	Carcinoma of the Large Intestine	MSX2	0	11.332886	*
993	Carcinoma of the Large Intestine	CDH3	0	16.214804	*
994	Carcinoma of the Large Intestine	FAP	0	18.387175	*
995	Carcinoma of the Large Intestine	MMP7	0	32.841944	*
996	Catenins	KIAA1199	0	-9.475267	*
997	Catenins	PROX1	0	-8.678827	*
998	Catenins	FGF20	0	-7.012846	*

999	Catenins	TNFRSF11B	0	-5.709377	*
1000	Catenins	DCLK1	0	-5.445256	*
1001	Catenins	C4orf46	0	-4.443408	*
1002	Catenins	MYH7B	0	-4.287094	*
1003	Catenins	MAP1LC3B	0	-4.269795	*
1004	Catenins	HNRNPA0	0	-4.257481	*
1005	Catenins	SP5	0	-4.186757	*
1006	Catenins	SYK	0	-4.131503	*
1007	Catenins	NAA30	0	-4.091132	*
1008	Catenins	MAK16	0	-3.859284	*
1009	Catenins	PIGW	0	-3.823781	*
1010	Catenins	PFKFB2	0.000001	-3.665898	*
1011	Catenins	RASA1	0	-3.615429	*
1012	Catenins	LEPROTL1	0	-3.588382	*
1013	Catenins	PNKD	0	-3.522663	*
1014	Catenins	SYNGR3	0.000001	-3.480191	*
1015	Catenins	NXT2	0	-3.361643	*
1016	Catenins	LOC100288413	0	-3.361643	*
1017	Catenins	KIF1B	0	-3.096918	*
1018	Catenins	SCRIB	0	-3.08086	*
1019	Catenins	MAPK1	0	-2.998346	*
1020	Catenins	ASCL2	0	-2.911306	*
1021	Catenins	DOCK11	0	-2.904588	*
1022	Catenins	YRDC	0	-2.901234	*
1023	Catenins	PLEKHA3	0	-2.85304	*
1024	Catenins	AIDA	0	-2.83497	*
1025	Catenins	PPP1R2	0.000001	-2.828427	*
1026	Catenins	NCRNA00118	0	-2.74632	*
1027	Catenins	CDK19	0	-2.724201	*
1028	Catenins	MARVELD2	0	-2.713209	*
1029	Catenins	PLEKHB2	0	-2.655837	*
1030	Catenins	TMEM41B	0	-2.620787	*
1031	Catenins	SLC38A1	0	-2.596677	*
1032	Catenins	NAP1L1	0.000001	-2.549121	*
1033	Catenins	TSPAN5	0	-2.543238	*
1034	Catenins	DYNC1LI2	0	-2.518387	*
1035	Catenins	TOMM22	0.000001	-2.467984	*
1036	Catenins	ETS2	0.000001	-2.452353	*
1037	Catenins	GINS1	0.000001	-2.42839	*
1038	Catenins	RABGAP1	0	-2.413009	*
1039	Catenins	PTRH1	0	-2.408831	*
1040	Catenins	ATG12	0	-2.399111	*
1041	Catenins	FAM105B	0	-2.366082	*
1042	Catenins	TM7SF3	0	-2.308038	*
1043	Catenins	TTL	0	-2.282846	*
1044	Catenins	PLA2G12A	0.000001	-2.269697	*
1045	Catenins	CXCL16	0	-2.265768	*
1046	Catenins	ANTXR1	0	-2.23845	*
1047	Catenins	TNRC6C	0	-2.23845	*
1048	Catenins	SAP130	0	-2.186061	*

1049	Catenins	VPS41	0	-2.184798	*
1050	Catenins	LOC221710	0	-2.149747	*
1051	Catenins	SNX12	0	-2.147265	*
1052	Catenins	HMGN4	0	-2.136131	*
1053	Catenins	UBR7	0.000001	-2.133664	*
1054	Catenins	SAP30L	0	-2.132432	*
1055	Catenins	KIF5B	0	-2.125055	*
1056	Catenins	SNTB1	0	-2.123828	*
1057	Catenins	DNAJC19	0.000001	-2.099433	*
1058	Catenins	CCNF	0.000001	-2.084932	*
1059	Catenins	TSC22D1	0	-2.057416	*
1060	Catenins	PRSS3	0.000001	-2.056228	*
1061	Catenins	PPP1R14C	0	-2.05504	*
1062	Catenins	TMEM127	0.000001	-2.050298	*
1063	Catenins	EXOSC2	0	-2.049114	*
1064	Catenins	PPP1CC	0.000001	-2.008103	*
1065	Catenins	LOC100289410	0.000001	-2.002312	*
1066	Catenins	RBMS2	0.000001	2.001156	*
1067	Catenins	GEM	0	2.010424	*
1068	Catenins	GRK5	0.000001	2.011586	*
1069	Catenins	APBB2	0.000001	2.020903	*
1070	Catenins	OLFML2A	0	2.03261	*
1071	Catenins	SH3TC2	0.000001	2.038489	*
1072	Catenins	ARL6IP5	0	2.044385	*
1073	Catenins	PPL	0	2.045566	*
1074	Catenins	CHST6	0.000001	2.049114	*
1075	Catenins	PYCARD	0	2.051482	*
1076	Catenins	UBE2H	0	2.074121	*
1077	Catenins	PQLC3	0	2.105506	*
1078	Catenins	SLC22A4	0	2.110376	*
1079	Catenins	KRT80	0	2.133664	*
1080	Catenins	GLIS2	0	2.141072	*
1081	Catenins	PLA2G16	0	2.148505	*
1082	Catenins	PLD1	0	2.159704	*
1083	Catenins	PRSS23	0	2.160951	*
1084	Catenins	MICAL2	0	2.168454	*
1085	Catenins	C17orf91	0	2.175982	*
1086	Catenins	LOXL4	0.000001	2.175982	*
1087	Catenins	ZNF467	0.000001	2.201266	*
1088	Catenins	NAV1	0	2.215297	*
1089	Catenins	ANKRD46	0	2.241037	*
1090	Catenins	SYTL2	0	2.247519	*
1091	Catenins	GPRC5A	0	2.257929	*
1092	Catenins	FNIP2	0	2.292095	*
1093	Catenins	CTAGE5	0	2.337554	*
1094	Catenins	MAML3	0.000001	2.348381	*
1095	Catenins	ASB9	0	2.390811	*
1096	Catenins	ZEB1	0	2.400497	*
1097	Catenins	CSF2	0	2.403272	*
1098	Catenins	FLJ39051	0.000001	2.414403	*

1099	Catenins	CD14	0	2.418591	*
1100	Catenins	PLAUR	0	2.436821	*
1101	Catenins	MGC39372	0	2.438228	*
1102	Catenins	ABCC3	0	2.456606	*
1103	Catenins	STRADB	0	2.459446	*
1104	Catenins	AHNAK2	0	2.462289	*
1105	Catenins	SYNPO	0.000001	2.486587	*
1106	Catenins	TXNIP	0	2.522755	*
1107	Catenins	LIPG	0.000001	2.587693	*
1108	Catenins	C10orf116	0.000001	2.592181	*
1109	Catenins	LACTB	0.000001	2.623816	*
1110	Catenins	FHL1	0	2.8481	*
1111	Catenins	ELFN2	0.000001	2.921414	*
1112	Catenins	PRICKLE1	0	2.946836	*
1113	Catenins	PTGER2	0	3.068429	*
1114	Catenins	ZMIZ1	0	3.123868	*
1115	Catenins	CLIC3	0	3.138336	*
1116	Catenins	ARHGAP42	0	3.154694	*
1117	Catenins	CD70	0	3.330718	*
1118	Catenins	DENND5B	0	3.400704	*
1119	Catenins	FAM20C	0	3.418429	*
1120	Catenins	ZCCHC24	0	3.452162	*
1121	Catenins	TMEM158	0	3.54307	*
1122	Catenins	RASSF4	0	3.682877	*
1123	Catenins	TNFSF9	0	3.695663	*
1124	Catenins	RTEL1	0	3.808352	*
1125	Catenins	IFIT1	0	3.828201	*
1126	Catenins	RARRES2	0	3.899619	*
1127	Catenins	CFL2	0.000001	3.899619	*
1128	Catenins	CREG2	0.000001	3.917681	*
1129	Catenins	LOC399959	0	3.924476	*
1130	Catenins	KRT86	0	3.940376	*
1131	Catenins	TRIM22	0	4.351964	*
1132	Catenins	TMEM133	0	4.356995	*
1133	Catenins	ATP8A1	0	4.484664	*
1134	Catenins	LOC654433	0	4.642816	*
1135	Catenins	CNIH3	0	5.429553	*
1136	Catenins	LAPTM5	0	6.607785	*
1137	Catenins	CCL7	0	7.559723	*
1138	Catenins	TIMP3	0	8.544523	*
1139	Catenins	SPARC	0	12.809717	*
1140	Colon Carcinoma	BCAS1	0.004661	-17.453571	*
1141	Colon Carcinoma	AKAP5	0.000543	-7.672727	*
1142	Colon Carcinoma	SLC16A9	0.00274	-4.44136	*
1143	Colon Carcinoma	TP53I3	0.003331	-2.596453	*
1144	Colon Carcinoma	FANCE	0.005681	2.118416	*
1145	Colon Carcinoma	REXO2	0.005681	2.160419	*
1146	Colon Carcinoma	ZNF526	0.005925	2.162342	*
1147	Colon Carcinoma	RHEBL1	0.005681	2.261905	*
1148	Colon Carcinoma	RPS19BP1	0.003331	2.26466	*

1149	Colon Carcinoma	AARSD1	0.00613	2.270759	*
1150	Colon Carcinoma	ODF2	0.005925	2.309888	*
1151	Colon Carcinoma	DVL2	0.004792	2.341063	*
1152	Colon Carcinoma	MED20	0.005681	2.355722	*
1153	Colon Carcinoma	SEH1L	0.004518	2.360705	*
1154	Colon Carcinoma	VDAC3	0.004518	2.387827	*
1155	Colon Carcinoma	CASC3	0.005681	2.403027	*
1156	Colon Carcinoma	TMEM99	0.004518	2.45	*
1157	Colon Carcinoma	DHX57	0.004792	2.473856	*
1158	Colon Carcinoma	LIPE	0.006116	2.478632	*
1159	Colon Carcinoma	DDX55	0.004661	2.49934	*
1160	Colon Carcinoma	UBQLN1	0.004661	2.508627	*
1161	Colon Carcinoma	AP2M1	0.004792	2.519507	*
1162	Colon Carcinoma	INTS5	0.004661	2.532124	*
1163	Colon Carcinoma	DHX30	0.003998	2.555366	*
1164	Colon Carcinoma	SNRNP48	0.004094	2.563389	*
1165	Colon Carcinoma	GSK3A	0.00274	2.598728	*
1166	Colon Carcinoma	BAT1	0.004146	2.612366	*
1167	Colon Carcinoma	DIABLO	0.004661	2.621987	*
1168	Colon Carcinoma	WSB2	0.004392	2.622514	*
1169	Colon Carcinoma	SLC25A15	0.005681	2.654579	*
1170	Colon Carcinoma	POLDIP2	0.003716	2.671356	*
1171	Colon Carcinoma	ADSL	0.005568	2.705135	*
1172	Colon Carcinoma	ZNF74	0.004146	2.735369	*
1173	Colon Carcinoma	TFAP4	0.005681	2.735507	*
1174	Colon Carcinoma	FAM136A	0.005777	2.752235	*
1175	Colon Carcinoma	MLF2	0.000543	2.764901	*
1176	Colon Carcinoma	DDX54	0.003115	2.767251	*
1177	Colon Carcinoma	MEN1	0.005391	2.787758	*
1178	Colon Carcinoma	ZW10	0.004351	2.787995	*
1179	Colon Carcinoma	RNH1	0.004792	2.804233	*
1180	Colon Carcinoma	TMEM222	0.004146	2.815126	*
1181	Colon Carcinoma	TMEM43	0.005925	2.829317	*
1182	Colon Carcinoma	AP3M1	0.004518	2.900102	*
1183	Colon Carcinoma	DCPS	0.005681	2.902001	*
1184	Colon Carcinoma	PDIA6	0.004518	2.90568	*
1185	Colon Carcinoma	MRPL37	0.003331	2.913286	*
1186	Colon Carcinoma	TRAPPC4	0.004101	2.913919	*
1187	Colon Carcinoma	EDEM2	0.00581	2.927928	*
1188	Colon Carcinoma	VPS4A	0.005681	2.938373	*
1189	Colon Carcinoma	ALG3	0.005978	2.939251	*
1190	Colon Carcinoma	FAM134C	0.005681	2.947311	*
1191	Colon Carcinoma	NUMBL	0.004222	2.960859	*
1192	Colon Carcinoma	TMEM39B	0.004518	2.961722	*
1193	Colon Carcinoma	SHMT2	0.001709	2.980376	*
1194	Colon Carcinoma	PAFAH1B2	0.004661	2.988591	*
1195	Colon Carcinoma	STT3A	0.00531	2.993854	*
1196	Colon Carcinoma	SLC25A39	0.002827	3.001813	*
1197	Colon Carcinoma	GNS	0.004518	3.015521	*
1198	Colon Carcinoma	EXTL2	0.004146	3.033154	*

1199	Colon Carcinoma	M6PR	0.005681	3.035629	*
1200	Colon Carcinoma	DDB1	0.004146	3.057781	*
1201	Colon Carcinoma	PSMF1	0.005681	3.094299	*
1202	Colon Carcinoma	RHOT1	0.005681	3.134272	*
1203	Colon Carcinoma	BUB3	0.004183	3.142091	*
1204	Colon Carcinoma	DDX50	0.002827	3.14262	*
1205	Colon Carcinoma	ZRANB1	0.005681	3.146275	*
1206	Colon Carcinoma	NOP56	0.00613	3.148926	*
1207	Colon Carcinoma	PWP2	0.004792	3.149072	*
1208	Colon Carcinoma	PHF20	0.006116	3.164103	*
1209	Colon Carcinoma	CPSF4	0.004078	3.175705	*
1210	Colon Carcinoma	NANP	0.004255	3.185112	*
1211	Colon Carcinoma	RPUSD4	0.00274	3.207434	*
1212	Colon Carcinoma	AACS	0.005142	3.239776	*
1213	Colon Carcinoma	COPS7A	0.00274	3.259742	*
1214	Colon Carcinoma	GANAB	0.00274	3.283045	*
1215	Colon Carcinoma	KATNB1	0.005681	3.285481	*
1216	Colon Carcinoma	OSBPL10	0.004146	3.287311	*
1217	Colon Carcinoma	RUVBL2	0.006112	3.290995	*
1218	Colon Carcinoma	SSRP1	0.004661	3.296498	*
1219	Colon Carcinoma	SRPR	0.005681	3.296962	*
1220	Colon Carcinoma	GMPS	0.004518	3.311997	*
1221	Colon Carcinoma	FIBP	0.002702	3.321244	*
1222	Colon Carcinoma	NOC4L	0.003716	3.340548	*
1223	Colon Carcinoma	RCC2	0.005681	3.350207	*
1224	Colon Carcinoma	RPN2	0.00274	3.377154	*
1225	Colon Carcinoma	PA2G4	0.00274	3.385057	*
1226	Colon Carcinoma	NOP14	0.004661	3.39778	*
1227	Colon Carcinoma	KPNB1	0.004792	3.418362	*
1228	Colon Carcinoma	PPP2CA	0.005558	3.478573	*
1229	Colon Carcinoma	EZH2	0.005681	3.483052	*
1230	Colon Carcinoma	LASS2	0.00613	3.511735	*
1231	Colon Carcinoma	NAT10	0.004518	3.51483	*
1232	Colon Carcinoma	NEU3	0.005925	3.553583	*
1233	Colon Carcinoma	SYNGAP1	0.0052	3.554779	*
1234	Colon Carcinoma	ENO1	0.00274	3.587509	*
1235	Colon Carcinoma	SF3B3	0.005501	3.613169	*
1236	Colon Carcinoma	DPF2	0.005681	3.620165	*
1237	Colon Carcinoma	HIAT1	0.005479	3.620712	*
1238	Colon Carcinoma	TRIM28	0.004518	3.638133	*
1239	Colon Carcinoma	PRMT5	0.003998	3.657105	*
1240	Colon Carcinoma	G6PD	0.00274	3.692506	*
1241	Colon Carcinoma	USP5	0.003716	3.700412	*
1242	Colon Carcinoma	CSDA	0.005077	3.70852	*
1243	Colon Carcinoma	ARMC6	0.004518	3.744797	*
1244	Colon Carcinoma	RAN	0.006116	3.745088	*
1245	Colon Carcinoma	LUZP6	0.004518	3.782718	*
1246	Colon Carcinoma	VARS	0.00184	3.789916	*
1247	Colon Carcinoma	PREB	0.004956	3.832184	*
1248	Colon Carcinoma	RUVBL1	0.005681	3.875942	*

1249	Colon Carcinoma	TCF7	0.004146	3.880342	*
1250	Colon Carcinoma	PHF16	0.00274	3.915459	*
1251	Colon Carcinoma	NMT1	0.005681	3.920484	*
1252	Colon Carcinoma	POLD2	0.005681	3.983102	*
1253	Colon Carcinoma	KLHL11	0.004146	4.02168	*
1254	Colon Carcinoma	ESYT1	0.004518	4.039931	*
1255	Colon Carcinoma	TEAD4	0.005681	4.044044	*
1256	Colon Carcinoma	TXNDC5	0.003331	4.108174	*
1257	Colon Carcinoma	GART	0.004661	4.111965	*
1258	Colon Carcinoma	UNC119B	0.005142	4.129353	*
1259	Colon Carcinoma	IPO5	0.000543	4.148212	*
1260	Colon Carcinoma	SMYD5	0.005205	4.192783	*
1261	Colon Carcinoma	EFTUD2	0.004518	4.19284	*
1262	Colon Carcinoma	NT5C3L	0.004078	4.209745	*
1263	Colon Carcinoma	NSUN5	0.00274	4.223239	*
1264	Colon Carcinoma	GPN3	0.005925	4.245142	*
1265	Colon Carcinoma	OGFOD1	0.002827	4.274142	*
1266	Colon Carcinoma	ZNF473	0.000543	4.320616	*
1267	Colon Carcinoma	KLC2	0.005799	4.347443	*
1268	Colon Carcinoma	MORN4	0.004078	4.44856	*
1269	Colon Carcinoma	COQ10A	0.00274	4.468316	*
1270	Colon Carcinoma	NONO	0.002827	4.4933	*
1271	Colon Carcinoma	TOMM34	0.004392	4.503749	*
1272	Colon Carcinoma	NOC3L	0.005681	4.69697	*
1273	Colon Carcinoma	FTSJ1	0.004078	4.767414	*
1274	Colon Carcinoma	TPD52L2	0.004078	4.788684	*
1275	Colon Carcinoma	LDHB	0.002827	4.81117	*
1276	Colon Carcinoma	DTD1	0.005681	4.857716	*
1277	Colon Carcinoma	XPO5	0.00184	4.886191	*
1278	Colon Carcinoma	G6PC3	0.00274	4.886922	*
1279	Colon Carcinoma	WDR74	0.004146	5.075321	*
1280	Colon Carcinoma	AHCY	0.004518	5.223647	*
1281	Colon Carcinoma	TEX10	0.00274	5.426288	*
1282	Colon Carcinoma	CIRH1A	0.00274	5.542027	*
1283	Colon Carcinoma	TSPYL2	0.00274	5.747126	*
1284	Colon Carcinoma	TMEM185B	0.003716	5.837496	*
1285	Colon Carcinoma	CDK4	0.004518	6.24989	*
1286	Colon Carcinoma	HSPA13	0.005681	6.341991	*
1287	Colon Carcinoma	YARS	0.00274	6.348424	*
1288	Colon Carcinoma	DPP7	0.006116	6.682502	*
1289	Colon Carcinoma	SIM2	0.0052	7.317708	*
1290	Colon Carcinoma	GRPEL2	0.004661	7.668546	*
1291	Colon Carcinoma	MTHFD2	0.004146	8.186467	*
1292	Colon Carcinoma	CD81	0.004661	8.248565	*
1293	Colon Carcinoma	ASNS	0.005077	9.20214	*
1294	Colon Carcinoma	ACCN2	0.004094	10.236928	*
1295	Colon Carcinoma	GRB10	0.003716	10.421296	*
1296	Colon Carcinoma	LDLRAD3	0.00613	11.487923	*
1297	Colon Carcinoma	FOXQ1	0.00581	11.687135	*
1298	Colon Carcinoma	SLC39A6	0.006116	11.756536	*

1299	Colon Carcinoma	SLC3A2	0.004792	14.29764	*
1300	Colon Carcinoma	PITX1	0.004952	22.45	*
1301	Colon Carcinoma	HS6ST2	0.004518	24.301075	*
1302	Colon Carcinoma	FXYD5	0.004698	64.324786	*
1303	Colonic Neoplasms	COL1A1	0	-58.559107	*
1304	Colonic Neoplasms	IGHG1	0	-15.238681	*
1305	Colonic Neoplasms	CA2	0.000006	-9.288727	*
1306	Colonic Neoplasms	CXCL12	0	-8.439793	*
1307	Colonic Neoplasms	SLC4A4	0	-7.982638	*
1308	Colonic Neoplasms	AKR1B10	0	-6.975418	*
1309	Colonic Neoplasms	ADH1C	0	-6.643184	*
1310	Colonic Neoplasms	CEACAM7	0.000003	-6.587967	*
1311	Colonic Neoplasms	PLA2G10	0	-6.113778	*
1312	Colonic Neoplasms	MT1M	0	-5.911706	*
1313	Colonic Neoplasms	ABCA8	0	-5.605008	*
1314	Colonic Neoplasms	IGHA1	0	-5.251841	*
1315	Colonic Neoplasms	CKB	0	-4.979591	*
1316	Colonic Neoplasms	DES	0	-4.751915	*
1317	Colonic Neoplasms	KRT24	0	-4.737791	*
1318	Colonic Neoplasms	MGC13057	0	-4.559318	*
1319	Colonic Neoplasms	MAOB	0	-4.38741	*
1320	Colonic Neoplasms	TNS1	0	-4.345642	*
1321	Colonic Neoplasms	LDHD	0	-3.865801	*
1322	Colonic Neoplasms	SMPDL3A	0.000003	-3.486516	*
1323	Colonic Neoplasms	C7	0	-3.336013	*
1324	Colonic Neoplasms	PDCD4	0	-3.025533	*
1325	Colonic Neoplasms	ITM2C	0	-3.013275	*
1326	Colonic Neoplasms	SELENBP1	0	-2.780562	*
1327	Colonic Neoplasms	KLF9	0	-2.712093	*
1328	Colonic Neoplasms	MGLL	0	-2.547536	*
1329	Colonic Neoplasms	CCL15	0	-2.514962	*
1330	Colonic Neoplasms	MYL9	0	-2.274491	*
1331	Colonic Neoplasms	GPX3	0	-2.082077	*
1332	Colonic Neoplasms	EPHB2	0	2.414518	*
1333	Colonic Neoplasms	ANXA3	0	2.482915	*
1334	Colonic Neoplasms	CXCL3	0	5.969079	*
1335	Colonic Neoplasms	CXCL1	0	6.760238	*
1336	Colorectal Cancer	CA1	0	-70.177195	*
1337	Colorectal Cancer	AGR3	0.000472	-58.557365	*
1338	Colorectal Cancer	CLCA1	0	-33.172078	*
1339	Colorectal Cancer	SLC26A3	0	-32.059519	*
1340	Colorectal Cancer	ADH1C	0	-17.399607	*
1341	Colorectal Cancer	GUCA2A	0	-16.294008	*
1342	Colorectal Cancer	CA2	0	-16.253863	*
1343	Colorectal Cancer	FAM55D	0	-15.875662	*
1344	Colorectal Cancer	HEPACAM2	0	-13.446451	*
1345	Colorectal Cancer	MYH11	0.000001	-12.464317	*
1346	Colorectal Cancer	FCGBP	0	-11.435381	*
1347	Colorectal Cancer	IGJ	0	-9.782349	*
1348	Colorectal Cancer	PIGR	0.00014	-8.779625	*

1349	Colorectal Cancer	CA12	0.000136	-5.903099	*
1350	Colorectal Cancer	ST6GALNAC1	0	-4.4897	*
1351	Colorectal Cancer	VSIG2	0.000043	-4.221688	*
1352	Colorectal Cancer	PLA2G10	0	-3.722207	*
1353	Colorectal Cancer	TSPAN1	0.000001	-3.277753	*
1354	Colorectal Cancer	ITM2C	0.000001	-2.872164	*
1355	Colorectal Cancer	C6orf105	0.000115	-2.77523	*
1356	Colorectal Cancer	CAPN9	0.000016	-2.643441	*
1357	Colorectal Cancer	AGR2	0.000531	-2.405779	*
1358	Colorectal Cancer	ABP1	0.000179	-2.385219	*
1359	Colorectal Cancer	MYO1A	0.000642	-2.309044	*
1360	Colorectal Cancer	BCAS1	0.000006	-2.292699	*
1361	Colorectal Cancer	SCNN1B	0.000177	-2.287546	*
1362	Colorectal Cancer	FAM46C	0.000021	-2.273251	*
1363	Colorectal Cancer	FRZB	0.000071	-2.263728	*
1364	Colorectal Cancer	BTNL3	0.000071	-2.24713	*
1365	Colorectal Cancer	LPAR1	0	-2.101231	*
1366	Colorectal Cancer	LGALS4	0.000004	-2.055385	*
1367	Colorectal Cancer	ZNF282	0	2.036182	*
1368	Colorectal Cancer	SET	0	2.042096	*
1369	Colorectal Cancer	ITIH2	0	2.398352	*
1370	Colorectal Cancer	LTBP2	0	2.53017	*
1371	Colorectal Cancer	EEPD1	0	2.633427	*
1372	Colorectal Cancer	FAM188B	0	2.650885	*
1373	Colorectal Cancer	C7orf68	0.000001	2.713761	*
1374	Colorectal Cancer	TOP1MT	0	2.723722	*
1375	Colorectal Cancer	TCOF1	0	2.727922	*
1376	Colorectal Cancer	GPR176	0	2.759087	*
1377	Colorectal Cancer	HABP2	0	2.896576	*
1378	Colorectal Cancer	GRB10	0	2.927995	*
1379	Colorectal Cancer	SLC41A1	0	2.968926	*
1380	Colorectal Cancer	PPEF1	0	2.974042	*
1381	Colorectal Cancer	SGIP1	0	3.035792	*
1382	Colorectal Cancer	SOX4	0	3.075368	*
1383	Colorectal Cancer	GDPD5	0	3.12273	*
1384	Colorectal Cancer	KIAA1257	0	3.140853	*
1385	Colorectal Cancer	SEC14L2	0	3.188415	*
1386	Colorectal Cancer	DNAH5	0	3.204015	*
1387	Colorectal Cancer	PDE10A	0	3.215748	*
1388	Colorectal Cancer	CDKN2A	0	3.262812	*
1389	Colorectal Cancer	RNF32	0	3.26672	*
1390	Colorectal Cancer	SLC9A7	0	3.2869	*
1391	Colorectal Cancer	ITGA11	0	3.303535	*
1392	Colorectal Cancer	ADAMTS2	0	3.332996	*
1393	Colorectal Cancer	P4HA3	0	3.358196	*
1394	Colorectal Cancer	BFSP1	0	3.394537	*
1395	Colorectal Cancer	LPCAT1	0	3.398193	*
1396	Colorectal Cancer	TCF7	0	3.484109	*
1397	Colorectal Cancer	IRAK2	0	3.526085	*
1398	Colorectal Cancer	ADAMTS6	0	3.581777	*

1399	Colorectal Cancer	GLS2	0	3.614199	*
1400	Colorectal Cancer	ALDH3B2	0	3.771741	*
1401	Colorectal Cancer	HS6ST2	0	3.806945	*
1402	Colorectal Cancer	TBX20	0	3.863409	*
1403	Colorectal Cancer	HAPLN3	0	3.864986	*
1404	Colorectal Cancer	LEPRE1	0	3.929111	*
1405	Colorectal Cancer	FAM150A	0	3.955729	*
1406	Colorectal Cancer	CSGALNACT1	0	3.983069	*
1407	Colorectal Cancer	VWA2	0	3.985745	*
1408	Colorectal Cancer	LRRC34	0	4.101056	*
1409	Colorectal Cancer	PJA1	0	4.103211	*
1410	Colorectal Cancer	GAS2	0	4.130475	*
1411	Colorectal Cancer	PTP4A3	0	4.198752	*
1412	Colorectal Cancer	ACSL6	0	4.21929	*
1413	Colorectal Cancer	C1orf135	0	4.242459	*
1414	Colorectal Cancer	SNTB1	0	4.273359	*
1415	Colorectal Cancer	TNFSF11	0	4.302181	*
1416	Colorectal Cancer	MTHFD1L	0	4.380449	*
1417	Colorectal Cancer	STC1	0	4.395318	*
1418	Colorectal Cancer	ODAM	0	4.447008	*
1419	Colorectal Cancer	WDR66	0	4.447168	*
1420	Colorectal Cancer	CSF2	0	4.465932	*
1421	Colorectal Cancer	PAH	0	4.501848	*
1422	Colorectal Cancer	LOXL2	0	4.528196	*
1423	Colorectal Cancer	NEK5	0	4.545746	*
1424	Colorectal Cancer	GRHL1	0	4.760864	*
1425	Colorectal Cancer	PLAU	0	4.841344	*
1426	Colorectal Cancer	DIO2	0	4.877771	*
1427	Colorectal Cancer	HCN1	0	4.993712	*
1428	Colorectal Cancer	ICOS	0	5.13095	*
1429	Colorectal Cancer	CYP39A1	0	5.382667	*
1430	Colorectal Cancer	CDH6	0	5.400039	*
1431	Colorectal Cancer	GRIN2B	0	5.605313	*
1432	Colorectal Cancer	PDPN	0	5.623195	*
1433	Colorectal Cancer	EGFL6	0	5.80168	*
1434	Colorectal Cancer	ANGPT2	0	5.811083	*
1435	Colorectal Cancer	ADAMTS12	0	6.346981	*
1436	Colorectal Cancer	KRT80	0	6.582092	*
1437	Colorectal Cancer	JUB	0	6.6753	*
1438	Colorectal Cancer	MFAP2	0	6.758639	*
1439	Colorectal Cancer	COL11A1	0	7.090537	*
1440	Colorectal Cancer	SPP1	0	7.465531	*
1441	Colorectal Cancer	ESM1	0	7.595544	*
1442	Colorectal Cancer	COL10A1	0	7.669827	*
1443	Colorectal Cancer	LEMD1	0	7.698407	*
1444	Colorectal Cancer	INHBA	0	7.715281	*
1445	Colorectal Cancer	KAL1	0	8.67666	*
1446	Colorectal Cancer	ADAM12	0	9.08807	*
1447	Colorectal Cancer	KIAA1199	0	10.5275	*
1448	Colorectal Cancer	CHI3L1	0	10.717129	*

1449	Colorectal Cancer	MSX2	0	11.332886	*
1450	Colorectal Cancer	CDH3	0	16.214804	*
1451	Colorectal Cancer	FAP	0	18.387175	*
1452	Colorectal Cancer	MMP7	0	32.841944	*
1453	Colorectal Neoplasms	GUCA2A	0	-16.294008	*
1454	Colorectal Neoplasms	SLC26A3	0	-13.167276	*
1455	Colorectal Neoplasms	IFITM1	0	4.28338	*
1456	Colorectal Neoplasms	CXCL1	0	5.226033	*
1457	Disease	KCND3	0.000126	3.374661	*
1458	Dysplasia	SLC26A3	0	-38.08636	*
1459	Dysplasia	GCG	0.000008	-11.58098	*
1460	Dysplasia	NEUROD1	0.000008	-8.968304	*
1461	Dysplasia	USP2	0.000001	-7.413002	*
1462	Dysplasia	CHGA	0	-7.303062	*
1463	Dysplasia	CA1	0	-6.471103	*
1464	Dysplasia	MS4A12	0.000002	-5.212096	*
1465	Dysplasia	NR3C1	0	-5.199333	*
1466	Dysplasia	CA4	0.000006	-4.974505	*
1467	Dysplasia	PAG1	0	-4.492958	*
1468	Dysplasia	CDKN2B	0.000002	-4.005555	*
1469	Dysplasia	CLDN23	0.000001	-3.972223	*
1470	Dysplasia	SLC4A4	0.000001	-3.939493	*
1471	Dysplasia	LOC344887	0.000002	-3.69458	*
1472	Dysplasia	EDN3	0.000003	-3.516323	*
1473	Dysplasia	PKIB	0.000005	-3.330976	*
1474	Dysplasia	TIMP2	0	-3.292712	*
1475	Dysplasia	CP	0.000001	-3.280806	*
1476	Dysplasia	ADAMDEC1	0.000001	-3.259981	*
1477	Dysplasia	TP53INP2	0	-3.238175	*
1478	Dysplasia	PLAC9	0.000002	-3.223752	*
1479	Dysplasia	PTPRR	0.000004	-3.091864	*
1480	Dysplasia	C2orf88	0.000004	-2.991349	*
1481	Dysplasia	ANK2	0	-2.956341	*
1482	Dysplasia	POSTN	0	-2.944184	*
1483	Dysplasia	CPNE8	0	-2.902986	*
1484	Dysplasia	F13A1	0.000005	-2.837147	*
1485	Dysplasia	TMEM171	0.000006	-2.783301	*
1486	Dysplasia	COL13A1	0	-2.675191	*
1487	Dysplasia	SNAI2	0	-2.66951	*
1488	Dysplasia	DHRS11	0.000006	-2.66922	*
1489	Dysplasia	GPNMB	0.000002	-2.653335	*
1490	Dysplasia	MXD1	0	-2.559257	*
1491	Dysplasia	ZEB2	0.000001	-2.528816	*
1492	Dysplasia	COLEC12	0	-2.485879	*
1493	Dysplasia	DCN	0	-2.458779	*
1494	Dysplasia	SIGLEC1	0.000003	-2.44309	*
1495	Dysplasia	SEMA6D	0.000002	-2.40759	*
1496	Dysplasia	IL6R	0	-2.389857	*
1497	Dysplasia	C14orf139	0	-2.318159	*
1498	Dysplasia	LPAR1	0	-2.271204	*

1499	Dysplasia	NRP1	0.000003	-2.253391	*
1500	Dysplasia	ARHGAP15	0.000001	-2.248758	*
1501	Dysplasia	ITIH5	0	-2.235978	*
1502	Dysplasia	EDIL3	0	-2.227613	*
1503	Dysplasia	GNA11	0.000002	-2.227225	*
1504	Dysplasia	FRMD6	0.000006	-2.221702	*
1505	Dysplasia	NDN	0.000001	-2.218137	*
1506	Dysplasia	TMCC3	0.000007	-2.19297	*
1507	Dysplasia	TRPC1	0	-2.185608	*
1508	Dysplasia	B3GNT7	0.000007	-2.180997	*
1509	Dysplasia	MGAT4A	0	-2.169524	*
1510	Dysplasia	ARHGAP42	0	-2.119791	*
1511	Dysplasia	IGFBP3	0	-2.099543	*
1512	Dysplasia	LOC100127983	0.000008	-2.096915	*
1513	Dysplasia	C17orf91	0.000002	-2.0713	*
1514	Dysplasia	COL6A2	0	-2.03661	*
1515	Dysplasia	COL3A1	0	-2.027751	*
1516	Dysplasia	DOCK10	0.000008	-2.010241	*
1517	Hematocrit procedure	LYZ	0	-553.965593	*
1518	Hematocrit procedure	UGT1A1	0	-289.754891	*
1519	Hematocrit procedure	POF1B	0	-256.130843	*
1520	Hematocrit procedure	LGALS4	0.000001	-249.000167	*
1521	Hematocrit procedure	TOX3	0	-188.756066	*
1522	Hematocrit procedure	GPX2	0	-174.729443	*
1523	Hematocrit procedure	S100P	0	-164.471276	*
1524	Hematocrit procedure	TM4SF1	0.000002	-117.507367	*
1525	Hematocrit procedure	HKDC1	0.000001	-65.399986	*
1526	Hematocrit procedure	CLRN3	0	-63.568249	*
1527	Hematocrit procedure	LY75	0	-63.005992	*
1528	Hematocrit procedure	AMIGO2	0.000001	-44.444089	*
1529	Hematocrit procedure	TGFBI	0	-44.172228	*
1530	Hematocrit procedure	AKR1C3	0.000007	-41.255223	*
1531	Hematocrit procedure	GIPC2	0.000001	-38.52687	*
1532	Hematocrit procedure	LGR5	0.000002	-36.312211	*
1533	Hematocrit procedure	SPAG16	0.000005	-27.406277	*
1534	Hematocrit procedure	GCNT3	0.000006	-27.110823	*
1535	Hematocrit procedure	CD24	0.000001	-25.242614	*
1536	Hematocrit procedure	HNMT	0.000005	-21.776943	*
1537	Hematocrit procedure	TNFSF10	0.000002	-18.515111	*
1538	Hematocrit procedure	KIAA1199	0	-17.561393	*
1539	Hematocrit procedure	ITGB6	0.000001	-16.820715	*
1540	Hematocrit procedure	SLC22A3	0.00001	-16.487081	*
1541	Hematocrit procedure	RASSF6	0.000001	-16.482922	*
1542	Hematocrit procedure	LOC339290	0.000008	-12.446039	*
1543	Hematocrit procedure	PMEPA1	0.000003	-12.174786	*
1544	Hematocrit procedure	MUC13	0.000058	-11.700251	*
1545	Hematocrit procedure	LRIG1	0	-10.409232	*
1546	Hematocrit procedure	HIST1H2BG	0.000042	-10.401582	*
1547	Hematocrit procedure	ANKS4B	0.000008	-9.996164	*
1548	Hematocrit procedure	NCRNA00094	0.000004	-9.731985	*

1549	Hematocrit procedure	TMPRSS2	0	-9.451476	*
1550	Hematocrit procedure	HGD	0.000002	-8.781096	*
1551	Hematocrit procedure	MAP2K6	0	-8.252522	*
1552	Hematocrit procedure	AMACR	0.000008	-7.819271	*
1553	Hematocrit procedure	MAML2	0.000002	-7.453422	*
1554	Hematocrit procedure	GMDS	0	-7.063824	*
1555	Hematocrit procedure	TST	0.000009	-6.449722	*
1556	Hematocrit procedure	TMEM139	0.000003	-6.3594	*
1557	Hematocrit procedure	CEACAM1	0.000008	-5.954795	*
1558	Hematocrit procedure	H2AFJ	0	-5.259377	*
1559	Hematocrit procedure	SPNS2	0.000025	-4.766598	*
1560	Hematocrit procedure	SMAD6	0.000001	-4.666987	*
1561	Hematocrit procedure	SERPINB1	0.000006	-4.559866	*
1562	Hematocrit procedure	CTSC	0	-4.484061	*
1563	Hematocrit procedure	STON2	0.000011	-4.358066	*
1564	Hematocrit procedure	TAGLN2	0	-4.272309	*
1565	Hematocrit procedure	C20orf24	0	-4.02621	*
1566	Hematocrit procedure	ABHD2	0.000006	-3.99146	*
1567	Hematocrit procedure	KCNE3	0.000001	-3.944067	*
1568	Hematocrit procedure	ISG20	0.000055	-3.692702	*
1569	Hematocrit procedure	TXNDC9	0.000004	-3.677724	*
1570	Hematocrit procedure	MCF2L	0.000008	-3.374845	*
1571	Hematocrit procedure	NET1	0	-3.263517	*
1572	Hematocrit procedure	TGFBR2	0.000016	-3.161223	*
1573	Hematocrit procedure	ANXA4	0.000014	-3.10173	*
1574	Hematocrit procedure	ECH1	0.000006	-3.002113	*
1575	Hematocrit procedure	FZD5	0.000021	-2.968789	*
1576	Hematocrit procedure	NT5C3	0.000002	-2.927017	*
1577	Hematocrit procedure	SLC12A7	0	-2.913571	*
1578	Hematocrit procedure	HSDL2	0.000005	-2.883179	*
1579	Hematocrit procedure	EEPD1	0.000002	-2.850552	*
1580	Hematocrit procedure	ZFP36L2	0.000004	-2.797318	*
1581	Hematocrit procedure	GOLT1A	0.000024	-2.695651	*
1582	Hematocrit procedure	POLD4	0.000018	-2.59396	*
1583	Hematocrit procedure	SLC2A1	0.000044	-2.562317	*
1584	Hematocrit procedure	ACP6	0	-2.543673	*
1585	Hematocrit procedure	NUP98	0.000004	-2.419936	*
1586	Hematocrit procedure	PLS1	0.00001	-2.369724	*
1587	Hematocrit procedure	CHMP4B	0.000095	-2.343461	*
1588	Hematocrit procedure	ZNF630	0.000001	-2.331547	*
1589	Hematocrit procedure	HNRNPH1	0.000008	-2.244705	*
1590	Hematocrit procedure	KIF13B	0.000002	-2.140129	*
1591	Hematocrit procedure	LRRC8A	0.00026	-2.128568	*
1592	Hematocrit procedure	S100A11	0.000071	-2.068479	*
1593	Hematocrit procedure	FTH1	0.000001	-2.066176	*
1594	Hematocrit procedure	HNF4A	0.000009	-2.034895	*
1595	Hematocrit procedure	GPR56	0.000287	-2.03382	*
1596	Hematocrit procedure	FABP5	0.000142	2.003741	*
1597	Hematocrit procedure	TRIM23	0	2.043023	*
1598	Hematocrit procedure	CCDC69	0	2.051861	*

1599	Hematocrit procedure	CUL9	0.000024	2.055442	*
1600	Hematocrit procedure	HMG20B	0.000004	2.058405	*
1601	Hematocrit procedure	CBX6	0.000044	2.060868	*
1602	Hematocrit procedure	SFRS18	0	2.071387	*
1603	Hematocrit procedure	C3orf39	0.000125	2.082127	*
1604	Hematocrit procedure	SMAD2	0.000023	2.082566	*
1605	Hematocrit procedure	PTPRG	0.000062	2.084443	*
1606	Hematocrit procedure	C17orf68	0.000008	2.107659	*
1607	Hematocrit procedure	RGS10	0.000017	2.113307	*
1608	Hematocrit procedure	NKX3-1	0.000018	2.119512	*
1609	Hematocrit procedure	ZYG11B	0.000024	2.149045	*
1610	Hematocrit procedure	FECH	0.000165	2.201653	*
1611	Hematocrit procedure	PBK	0.00004	2.213962	*
1612	Hematocrit procedure	GNG10	0.000082	2.228616	*
1613	Hematocrit procedure	PIAS2	0.000005	2.235642	*
1614	Hematocrit procedure	RNF138	0.000051	2.239473	*
1615	Hematocrit procedure	TYMS	0.000001	2.30578	*
1616	Hematocrit procedure	MED13L	0.000012	2.322913	*
1617	Hematocrit procedure	HSPB1	0.000012	2.343433	*
1618	Hematocrit procedure	HEXIM1	0.000003	2.358521	*
1619	Hematocrit procedure	KANK2	0.000005	2.367209	*
1620	Hematocrit procedure	MYO5A	0	2.367748	*
1621	Hematocrit procedure	RPRD1A	0.000009	2.390988	*
1622	Hematocrit procedure	NISCH	0.000011	2.402661	*
1623	Hematocrit procedure	SYNCRIP	0	2.42249	*
1624	Hematocrit procedure	NFAT5	0.000137	2.425518	*
1625	Hematocrit procedure	STYX	0.000003	2.447138	*
1626	Hematocrit procedure	UBXN8	0	2.509628	*
1627	Hematocrit procedure	PSIP1	0.000032	2.535774	*
1628	Hematocrit procedure	GABBR1	0.000003	2.587627	*
1629	Hematocrit procedure	C21orf91	0	2.609282	*
1630	Hematocrit procedure	ITPRIPL2	0.000075	2.686906	*
1631	Hematocrit procedure	DHX30	0.000003	2.694996	*
1632	Hematocrit procedure	CIRBP	0.000011	2.747841	*
1633	Hematocrit procedure	SGSM1	0	2.890026	*
1634	Hematocrit procedure	NAB2	0.000002	2.897102	*
1635	Hematocrit procedure	FAM160B1	0.000002	2.933826	*
1636	Hematocrit procedure	CTSL1	0.000013	3.010972	*
1637	Hematocrit procedure	PTRF	0.000032	3.038161	*
1638	Hematocrit procedure	SLC39A6	0	3.146657	*
1639	Hematocrit procedure	FAT1	0.000013	3.147665	*
1640	Hematocrit procedure	CLU	0.000081	3.269041	*
1641	Hematocrit procedure	ACVR2B	0.000005	3.390992	*
1642	Hematocrit procedure	BTG3	0.000026	3.443329	*
1643	Hematocrit procedure	ST6GALNAC2	0	3.444906	*
1644	Hematocrit procedure	PTGES	0	3.474348	*
1645	Hematocrit procedure	SLC25A37	0.000003	3.556737	*
1646	Hematocrit procedure	MXRA7	0.000007	3.70477	*
1647	Hematocrit procedure	APBB2	0.000002	3.772794	*
1648	Hematocrit procedure	MT1E	0.000058	3.774363	*

1649	Hematocrit procedure	JAG2	0.000111	3.78717	*
1650	Hematocrit procedure	SLC26A11	0.000007	3.832825	*
1651	Hematocrit procedure	TMEM98	0	3.881347	*
1652	Hematocrit procedure	TWSG1	0.000008	3.912104	*
1653	Hematocrit procedure	ZDHHC2	0.000001	3.993547	*
1654	Hematocrit procedure	FZD2	0	4.030252	*
1655	Hematocrit procedure	PSTPIP2	0.000008	4.10078	*
1656	Hematocrit procedure	GPC1	0.000011	4.112104	*
1657	Hematocrit procedure	AIF1L	0.000064	4.187511	*
1658	Hematocrit procedure	GPR137B	0.000096	4.529965	*
1659	Hematocrit procedure	RNF144A	0.000002	4.736778	*
1660	Hematocrit procedure	RHOF	0.000006	4.754852	*
1661	Hematocrit procedure	PDGFRL	0.000039	4.789713	*
1662	Hematocrit procedure	FRMD6	0.000002	5.304811	*
1663	Hematocrit procedure	C1orf115	0.00001	5.876075	*
1664	Hematocrit procedure	FAM164A	0.000005	5.904321	*
1665	Hematocrit procedure	CFL2	0	6.04272	*
1666	Hematocrit procedure	KLC3	0.000003	6.402367	*
1667	Hematocrit procedure	MFAP3L	0.000033	6.479532	*
1668	Hematocrit procedure	TIMP2	0.000006	8.705022	*
1669	Hematocrit procedure	ZC3H12C	0.000033	8.930023	*
1670	Hematocrit procedure	MOCS1	0	9.33562	*
1671	Hematocrit procedure	OSBPL6	0	9.871076	*
1672	Hematocrit procedure	PLAGL1	0.000041	10.015007	*
1673	Hematocrit procedure	ID2	0.000001	10.221989	*
1674	Hematocrit procedure	GJA3	0.000018	10.47161	*
1675	Hematocrit procedure	GAMT	0	10.974298	*
1676	Hematocrit procedure	C8orf47	0.000005	12.045282	*
1677	Hematocrit procedure	TUBB6	0.000001	12.188465	*
1678	Hematocrit procedure	SIX4	0.000011	12.215531	*
1679	Hematocrit procedure	SEPP1	0	12.288043	*
1680	Hematocrit procedure	OGFRL1	0.000003	12.864371	*
1681	Hematocrit procedure	MT1X	0.000004	13.99134	*
1682	Hematocrit procedure	SERPINB9	0.000001	14.452624	*
1683	Hematocrit procedure	B3GALNT1	0.000003	15.014644	*
1684	Hematocrit procedure	NCRNA00173	0.000006	16.974223	*
1685	Hematocrit procedure	RBM24	0.000003	17.565277	*
1686	Hematocrit procedure	SMAD4	0.000004	18.303144	*
1687	Hematocrit procedure	SPIRE1	0.000001	18.354058	*
1688	Hematocrit procedure	RUNDC3B	0.000012	22.999973	*
1689	Hematocrit procedure	FAM92A1	0.000002	34.853387	*
1690	Hematocrit procedure	FAM171B	0.000005	38.639504	*
1691	Hematocrit procedure	DSE	0.000008	44.952396	*
1692	Hematocrit procedure	PHLDB2	0	79.357183	*
1693	Hematocrit procedure	NLRP2	0	110.051078	*
1694	Hematocrit procedure	WBP5	0.000004	121.981655	*
1695	Hematocrit procedure	HLTF	0.000003	143.882085	*
1696	Hematocrit procedure	RDX	0.000002	174.326299	*
1697	Histopathologic Grade differentiation	KRT23	0	-31.554983	*
1698	Histopathologic Grade differentiation	ABCC2	0.000003	-27.577196	*

1699	Histopathologic Grade differentiation	TCEA2	0.000183	-11.483034	*
1700	Histopathologic Grade differentiation	TIAM1	0.003852	-9.403248	*
1701	Histopathologic Grade differentiation	PPP2R2C	0.00274	-9.26497	*
1702	Histopathologic Grade differentiation	MSLN	0.000014	-7.882214	*
1703	Histopathologic Grade differentiation	GALNAC4S-6S	0.000036	-7.880769	*
1704	Histopathologic Grade differentiation	GGT1	0.000343	-7.50888	*
1705	Histopathologic Grade differentiation	C20orf46	0.00026	-7.033766	*
1706	Histopathologic Grade differentiation	ZFYVE28	0.012802	-6.738983	*
1707	Histopathologic Grade differentiation	ATL1	0.001975	-6.449874	*
1708	Histopathologic Grade differentiation	RUNX3	0.0004	-6.375437	*
1709	Histopathologic Grade differentiation	NPM2	0.009835	-5.9987	*
1710	Histopathologic Grade differentiation	LEMD1	0.001041	-5.571908	*
1711	Histopathologic Grade differentiation	NAV1	0.000001	-5.450984	*
1712	Histopathologic Grade differentiation	FSCN1	0.000618	-5.406554	*
1713	Histopathologic Grade differentiation	MAN1A1	0.000002	-4.80628	*
1714	Histopathologic Grade differentiation	DGKG	0.000138	-4.561431	*
1715	Histopathologic Grade differentiation	LPAR5	0.000338	-4.020064	*
1716	Histopathologic Grade differentiation	FLJ45248	0.003884	-3.840689	*
1717	Histopathologic Grade differentiation	ZNF302	0.004005	-3.387918	*
1718	Histopathologic Grade differentiation	LRP4	0.000123	-2.918713	*
1719	Histopathologic Grade differentiation	HSPA2	0.003738	-2.778928	*
1720	Histopathologic Grade differentiation	GRAMD1A	0.006135	-2.759221	*
1721	Histopathologic Grade differentiation	RIBC2	0.007882	-2.74858	*
1722	Histopathologic Grade differentiation	ZNF502	0.001534	-2.742984	*
1723	Histopathologic Grade differentiation	STC2	0.012034	-2.733117	*
1724	Histopathologic Grade differentiation	DNAJC22	0.000113	-2.570394	*
1725	Histopathologic Grade differentiation	SLC4A11	0.004788	-2.437059	*
1726	Histopathologic Grade differentiation	LOC100132288	0.008019	-2.425738	*
1727	Histopathologic Grade differentiation	ZNF385A	0.006949	-2.40828	*
1728	Histopathologic Grade differentiation	DEF6	0.002951	-2.398968	*
1729	Histopathologic Grade differentiation	FGD1	0.002489	-2.258271	*
1730	Histopathologic Grade differentiation	OSBPL3	0.00043	-2.251679	*
1731	Histopathologic Grade differentiation	KCNH2	0.000051	-2.224479	*
1732	Histopathologic Grade differentiation	RNF219	0.008574	-2.149713	*
1733	Histopathologic Grade differentiation	CYB5R4	0.000476	-2.133322	*
1734	Histopathologic Grade differentiation	SLC25A24	0.006832	2.002397	*
1735	Histopathologic Grade differentiation	FAM135A	0.00889	2.024645	*
1736	Histopathologic Grade differentiation	DGKE	0.000418	2.036216	*
1737	Histopathologic Grade differentiation	ZNF217	0.003393	2.093951	*
1738	Histopathologic Grade differentiation	DENND1B	0.000814	2.109635	*
1739	Histopathologic Grade differentiation	PLEKHF2	0.005589	2.12744	*
1740	Histopathologic Grade differentiation	SIPA1L1	0.00484	2.129215	*
1741	Histopathologic Grade differentiation	GTPBP2	0.002516	2.129316	*
1742	Histopathologic Grade differentiation	DMXL2	0.000002	2.133636	*
1743	Histopathologic Grade differentiation	CDKL5	0.005535	2.175485	*
1744	Histopathologic Grade differentiation	GALNT12	0.000014	2.180533	*
1745	Histopathologic Grade differentiation	SVIL	0.000427	2.21181	*
1746	Histopathologic Grade differentiation	SLC1A1	0.000085	2.304518	*
1747	Histopathologic Grade differentiation	CRAT	0.000121	2.348142	*
1748	Histopathologic Grade differentiation	ZNF586	0.000828	2.416349	*

1749	Histopathologic Grade differentiation	PRKCH	0.01198	2.425591	*
1750	Histopathologic Grade differentiation	NFIB	0.000008	2.477069	*
1751	Histopathologic Grade differentiation	ASAP2	0.006933	2.509466	*
1752	Histopathologic Grade differentiation	TGFB1	0.005622	2.541969	*
1753	Histopathologic Grade differentiation	ITGAV	0.003534	2.552711	*
1754	Histopathologic Grade differentiation	ANK3	0.001074	2.569722	*
1755	Histopathologic Grade differentiation	MASTL	0.007512	2.686504	*
1756	Histopathologic Grade differentiation	PTPLA	0.009947	2.737588	*
1757	Histopathologic Grade differentiation	ETS2	0.003852	2.809661	*
1758	Histopathologic Grade differentiation	CD59	0.00056	2.817171	*
1759	Histopathologic Grade differentiation	CCNG2	0.00773	2.837451	*
1760	Histopathologic Grade differentiation	STXBP6	0.000146	2.88386	*
1761	Histopathologic Grade differentiation	INSIG2	0.001715	2.962214	*
1762	Histopathologic Grade differentiation	C4orf3	0.000074	3.039577	*
1763	Histopathologic Grade differentiation	HOXA5	0.001394	3.145148	*
1764	Histopathologic Grade differentiation	OSBPL1A	0.004372	3.223307	*
1765	Histopathologic Grade differentiation	CACNA1D	0.00063	3.233841	*
1766	Histopathologic Grade differentiation	MATN2	0.016257	3.309188	*
1767	Histopathologic Grade differentiation	SPAG1	0.010306	3.349719	*
1768	Histopathologic Grade differentiation	CHRM3	0.005942	3.357614	*
1769	Histopathologic Grade differentiation	FAM149A	0.001074	3.45578	*
1770	Histopathologic Grade differentiation	ZNF702P	0.000015	3.689532	*
1771	Histopathologic Grade differentiation	PELI1	0.001394	3.751149	*
1772	Histopathologic Grade differentiation	NRP1	0.001394	3.85201	*
1773	Histopathologic Grade differentiation	NFIA	0.003384	3.900428	*
1774	Histopathologic Grade differentiation	MLLT3	0.000055	4.176684	*
1775	Histopathologic Grade differentiation	OTUD1	0.000435	4.246859	*
1776	Histopathologic Grade differentiation	ARSJ	0.000914	4.580518	*
1777	Histopathologic Grade differentiation	TLE1	0.000074	4.643999	*
1778	Histopathologic Grade differentiation	PLCE1	0.000134	5.168786	*
1779	Histopathologic Grade differentiation	GAB1	0.004005	5.4968	*
1780	Histopathologic Grade differentiation	LAMB1	0.000086	5.890751	*
1781	Histopathologic Grade differentiation	SLC7A2	0.000371	6.327241	*
1782	Histopathologic Grade differentiation	PPM1K	0.001653	6.563098	*
1783	Histopathologic Grade differentiation	ERAP2	0.011713	8.452647	*
1784	Histopathologic Grade differentiation	ACSS1	0.002112	8.548423	*
1785	Histopathologic Grade differentiation	FOXA1	0.000001	9.243613	*
1786	Histopathologic Grade differentiation	HOXA13	0.000008	9.675248	*
1787	Histopathologic Grade differentiation	CYP1B1	0.000014	10.335299	*
1788	Histopathologic Grade differentiation	PDE10A	0.000551	12.412028	*
1789	Histopathologic Grade differentiation	DAPK1	0.000001	12.970432	*
1790	Histopathologic Grade differentiation	RUNX2	0.000628	14.460861	*
1791	Human cells	TAPBP	0	4.01667	*
1792	Instability	MYH11	0	2.897326	*
1793	Malignant Neoplasms	C9orf125	0.000072	-1298.37944	*
1794	Malignant Neoplasms	AHNAK	0	-494.159908	*
1795	Malignant Neoplasms	REXO2	0	-404.064243	*
1796	Malignant Neoplasms	CIRH1A	0	-269.504631	*
1797	Malignant Neoplasms	PPP2CA	0	-205.443988	*
1798	Malignant Neoplasms	DDB1	0	-153.182139	*

1799	Malignant Neoplasms	SRSF6	0	-116.639748	*
1800	Malignant Neoplasms	PHF20	0	-112.379561	*
1801	Malignant Neoplasms	TRIM8	0	-101.821502	*
1802	Malignant Neoplasms	STAU1	0	-86.688335	*
1803	Malignant Neoplasms	EIF3B	0	-83.887792	*
1804	Malignant Neoplasms	RNF130	0	-77.051157	*
1805	Malignant Neoplasms	CA1	0	-70.177195	*
1806	Malignant Neoplasms	TLE1	0	-63.322744	*
1807	Malignant Neoplasms	TNFRSF10B	0	-51.235551	*
1808	Malignant Neoplasms	GRPEL1	0	-48.248593	*
1809	Malignant Neoplasms	PELI1	0	-46.465923	*
1810	Malignant Neoplasms	WASF2	0	-43.582996	*
1811	Malignant Neoplasms	CDADC1	0	-42.793207	*
1812	Malignant Neoplasms	TMEM43	0	-39.55442	*
1813	Malignant Neoplasms	UFSP2	0	-39.012736	*
1814	Malignant Neoplasms	TPD52L2	0	-38.64877	*
1815	Malignant Neoplasms	RHOT1	0	-37.758579	*
1816	Malignant Neoplasms	WDR74	0	-35.403596	*
1817	Malignant Neoplasms	CLCA1	0	-33.172078	*
1818	Malignant Neoplasms	SLC26A3	0	-32.059519	*
1819	Malignant Neoplasms	CD97	0	-31.776763	*
1820	Malignant Neoplasms	FOXP1	0	-30.01888	*
1821	Malignant Neoplasms	ATP10D	0.000282	-27.855315	*
1822	Malignant Neoplasms	FAM134C	0	-26.248413	*
1823	Malignant Neoplasms	PAFAH1B2	0	-24.078481	*
1824	Malignant Neoplasms	EIF2C2	0	-23.573257	*
1825	Malignant Neoplasms	U2AF1	0	-21.809927	*
1826	Malignant Neoplasms	ADH1C	0	-17.399607	*
1827	Malignant Neoplasms	GUCA2A	0	-16.294008	*
1828	Malignant Neoplasms	CA2	0	-16.253863	*
1829	Malignant Neoplasms	RSL1D1	0	-16.1776	*
1830	Malignant Neoplasms	FAM55D	0	-15.875662	*
1831	Malignant Neoplasms	AMPD2	0	-15.760984	*
1832	Malignant Neoplasms	AKT2	0	-15.115608	*
1833	Malignant Neoplasms	HEPACAM2	0	-13.446451	*
1834	Malignant Neoplasms	AKAP5	0.000488	-13.182194	*
1835	Malignant Neoplasms	MYH11	0.000001	-12.464317	*
1836	Malignant Neoplasms	FCGBP	0	-11.435381	*
1837	Malignant Neoplasms	CBS	0.000649	-10.364884	*
1838	Malignant Neoplasms	JMJD6	0	-10.33527	*
1839	Malignant Neoplasms	IGJ	0	-9.782349	*
1840	Malignant Neoplasms	PIGR	0.00014	-8.779625	*
1841	Malignant Neoplasms	ABCA5	0.00005	-8.236745	*
1842	Malignant Neoplasms	PTGDR	0	-8.086832	*
1843	Malignant Neoplasms	PYY	0	-7.608405	*
1844	Malignant Neoplasms	LDLRAD3	0.000538	-7.493235	*
1845	Malignant Neoplasms	CA12	0.000136	-5.903099	*
1846	Malignant Neoplasms	ADAMDEC1	0	-5.125187	*
1847	Malignant Neoplasms	CHGA	0	-4.56489	*
1848	Malignant Neoplasms	ST6GALNAC1	0	-4.4897	*

1849	Malignant Neoplasms	SLC16A9	0.00274	-4.44136	*
1850	Malignant Neoplasms	VSIG2	0.000043	-4.221688	*
1851	Malignant Neoplasms	ITLN1	0	-3.790869	*
1852	Malignant Neoplasms	PLA2G10	0	-3.722207	*
1853	Malignant Neoplasms	TSPAN1	0.000001	-3.277753	*
1854	Malignant Neoplasms	PLAC8	0	-3.158613	*
1855	Malignant Neoplasms	PADI2	0	-3.074963	*
1856	Malignant Neoplasms	ETFDH	0.000246	-2.961829	*
1857	Malignant Neoplasms	PDCD4	0	-2.945796	*
1858	Malignant Neoplasms	HIGD1A	0	-2.895566	*
1859	Malignant Neoplasms	ITM2C	0.000001	-2.872164	*
1860	Malignant Neoplasms	C6orf105	0.000115	-2.77523	*
1861	Malignant Neoplasms	ENTPD5	0	-2.665119	*
1862	Malignant Neoplasms	CAPN9	0.000016	-2.643441	*
1863	Malignant Neoplasms	TP53I3	0.003331	-2.596453	*
1864	Malignant Neoplasms	PDE4D	0	-2.490553	*
1865	Malignant Neoplasms	FOXF2	0	-2.48074	*
1866	Malignant Neoplasms	PLCE1	0	-2.457619	*
1867	Malignant Neoplasms	ABP1	0.000179	-2.385219	*
1868	Malignant Neoplasms	PRKACB	0	-2.333442	*
1869	Malignant Neoplasms	MYO1A	0.000642	-2.309044	*
1870	Malignant Neoplasms	BCAS1	0.000006	-2.292699	*
1871	Malignant Neoplasms	SCNN1B	0.000177	-2.287546	*
1872	Malignant Neoplasms	METTL7A	0	-2.279117	*
1873	Malignant Neoplasms	FAM46C	0.000021	-2.273251	*
1874	Malignant Neoplasms	FRZB	0.000071	-2.263728	*
1875	Malignant Neoplasms	BTNL3	0.000071	-2.24713	*
1876	Malignant Neoplasms	ACAD10	0.000112	-2.180166	*
1877	Malignant Neoplasms	EDIL3	0	-2.146757	*
1878	Malignant Neoplasms	LPAR1	0	-2.101231	*
1879	Malignant Neoplasms	SEMA6A	0	-2.093876	*
1880	Malignant Neoplasms	LGALS4	0.000004	-2.055385	*
1881	Malignant Neoplasms	ZNF282	0	2.036182	*
1882	Malignant Neoplasms	SET	0	2.042096	*
1883	Malignant Neoplasms	CDK4	0	2.062929	*
1884	Malignant Neoplasms	STT3A	0	2.077468	*
1885	Malignant Neoplasms	ENO1	0.000002	2.078688	*
1886	Malignant Neoplasms	RUVBL2	0	2.080554	*
1887	Malignant Neoplasms	FANCE	0.005681	2.118416	*
1888	Malignant Neoplasms	RNH1	0.000316	2.124698	*
1889	Malignant Neoplasms	C22orf29	0	2.132896	*
1890	Malignant Neoplasms	ZNF526	0.005925	2.162342	*
1891	Malignant Neoplasms	PDZD2	0	2.185247	*
1892	Malignant Neoplasms	CPSF4	0.000711	2.187018	*
1893	Malignant Neoplasms	RAN	0	2.207012	*
1894	Malignant Neoplasms	RHEBL1	0.005681	2.261905	*
1895	Malignant Neoplasms	RPS19BP1	0.003331	2.26466	*
1896	Malignant Neoplasms	FUT8	0	2.267452	*
1897	Malignant Neoplasms	PITX1	0.000001	2.26755	*
1898	Malignant Neoplasms	AARSD1	0.00613	2.270759	*

1899	Malignant Neoplasms	KIF20A	0	2.295043	*
1900	Malignant Neoplasms	ODF2	0.005925	2.309888	*
1901	Malignant Neoplasms	CBFB	0	2.334836	*
1902	Malignant Neoplasms	CDC42EP1	0.000001	2.339437	*
1903	Malignant Neoplasms	DVL2	0.004792	2.341063	*
1904	Malignant Neoplasms	MED20	0.005681	2.355722	*
1905	Malignant Neoplasms	SEH1L	0.004518	2.360705	*
1906	Malignant Neoplasms	VDAC3	0.004518	2.387827	*
1907	Malignant Neoplasms	ITIH2	0	2.398352	*
1908	Malignant Neoplasms	LY6G6D	0	2.400382	*
1909	Malignant Neoplasms	NOP56	0.000001	2.40229	*
1910	Malignant Neoplasms	CASC3	0.005681	2.403027	*
1911	Malignant Neoplasms	TEAD3	0.000061	2.404121	*
1912	Malignant Neoplasms	TMEM99	0.004518	2.45	*
1913	Malignant Neoplasms	DHX57	0.004792	2.473856	*
1914	Malignant Neoplasms	LIPE	0.006116	2.478632	*
1915	Malignant Neoplasms	VARS	0	2.490948	*
1916	Malignant Neoplasms	DDX55	0.004661	2.49934	*
1917	Malignant Neoplasms	UBQLN1	0.004661	2.508627	*
1918	Malignant Neoplasms	AP2M1	0.004792	2.519507	*
1919	Malignant Neoplasms	LTBP2	0	2.53017	*
1920	Malignant Neoplasms	INTS5	0.004661	2.532124	*
1921	Malignant Neoplasms	DHX30	0.003998	2.555366	*
1922	Malignant Neoplasms	KIAA1468	0.000649	2.560686	*
1923	Malignant Neoplasms	SNRNP48	0.004094	2.563389	*
1924	Malignant Neoplasms	CDC6	0	2.585624	*
1925	Malignant Neoplasms	AHCY	0.000002	2.587594	*
1926	Malignant Neoplasms	GSK3A	0.00274	2.598728	*
1927	Malignant Neoplasms	RUVBL1	0	2.600979	*
1928	Malignant Neoplasms	BAT1	0.004146	2.612366	*
1929	Malignant Neoplasms	DIABLO	0.004661	2.621987	*
1930	Malignant Neoplasms	WSB2	0.004392	2.622514	*
1931	Malignant Neoplasms	ENC1	0	2.6297	*
1932	Malignant Neoplasms	EEPD1	0	2.633427	*
1933	Malignant Neoplasms	FGD5	0	2.646435	*
1934	Malignant Neoplasms	FAM188B	0	2.650885	*
1935	Malignant Neoplasms	SLC25A15	0.005681	2.654579	*
1936	Malignant Neoplasms	RHBDF1	0.000215	2.662688	*
1937	Malignant Neoplasms	POLDIP2	0.003716	2.671356	*
1938	Malignant Neoplasms	ADSL	0.005568	2.705135	*
1939	Malignant Neoplasms	C7orf68	0.000001	2.713761	*
1940	Malignant Neoplasms	RFC3	0	2.720527	*
1941	Malignant Neoplasms	TOP1MT	0	2.723722	*
1942	Malignant Neoplasms	TCOF1	0	2.727922	*
1943	Malignant Neoplasms	ZNF74	0.004146	2.735369	*
1944	Malignant Neoplasms	TFAP4	0.005681	2.735507	*
1945	Malignant Neoplasms	FAM136A	0.005777	2.752235	*
1946	Malignant Neoplasms	EIF5A	0	2.754041	*
1947	Malignant Neoplasms	GPR176	0	2.759087	*
1948	Malignant Neoplasms	MLF2	0.000543	2.764901	*

1949	Malignant Neoplasms	DDX54	0.003115	2.767251	*
1950	Malignant Neoplasms	MEN1	0.005391	2.787758	*
1951	Malignant Neoplasms	ZW10	0.004351	2.787995	*
1952	Malignant Neoplasms	TMEM222	0.004146	2.815126	*
1953	Malignant Neoplasms	SORD	0	2.867057	*
1954	Malignant Neoplasms	HABP2	0	2.896576	*
1955	Malignant Neoplasms	AP3M1	0.004518	2.900102	*
1956	Malignant Neoplasms	DCPS	0.005681	2.902001	*
1957	Malignant Neoplasms	PDIA6	0.004518	2.90568	*
1958	Malignant Neoplasms	MRPL37	0.003331	2.913286	*
1959	Malignant Neoplasms	TRAPPC4	0.004101	2.913919	*
1960	Malignant Neoplasms	EDEM2	0.00581	2.927928	*
1961	Malignant Neoplasms	GRB10	0	2.927995	*
1962	Malignant Neoplasms	VPS4A	0.005681	2.938373	*
1963	Malignant Neoplasms	ALG3	0.005978	2.939251	*
1964	Malignant Neoplasms	NUMBL	0.004222	2.960859	*
1965	Malignant Neoplasms	TMEM39B	0.004518	2.961722	*
1966	Malignant Neoplasms	SLC41A1	0	2.968926	*
1967	Malignant Neoplasms	PPEF1	0	2.974042	*
1968	Malignant Neoplasms	SHMT2	0	2.982462	*
1969	Malignant Neoplasms	SLC25A39	0.002827	3.001813	*
1970	Malignant Neoplasms	GNS	0.004518	3.015521	*
1971	Malignant Neoplasms	EXTL2	0.004146	3.033154	*
1972	Malignant Neoplasms	M6PR	0.005681	3.035629	*
1973	Malignant Neoplasms	SGIP1	0	3.035792	*
1974	Malignant Neoplasms	SOX4	0	3.075368	*
1975	Malignant Neoplasms	PSMF1	0.005681	3.094299	*
1976	Malignant Neoplasms	TEAD4	0	3.121837	*
1977	Malignant Neoplasms	GDPD5	0	3.12273	*
1978	Malignant Neoplasms	KIAA1257	0	3.140853	*
1979	Malignant Neoplasms	BUB3	0.004183	3.142091	*
1980	Malignant Neoplasms	DDX50	0.002827	3.14262	*
1981	Malignant Neoplasms	ZRANB1	0.005681	3.146275	*
1982	Malignant Neoplasms	PWP2	0.004792	3.149072	*
1983	Malignant Neoplasms	NANP	0.004255	3.185112	*
1984	Malignant Neoplasms	SEC14L2	0	3.188415	*
1985	Malignant Neoplasms	DNAH5	0	3.204015	*
1986	Malignant Neoplasms	RPUSD4	0.00274	3.207434	*
1987	Malignant Neoplasms	PDE10A	0	3.215748	*
1988	Malignant Neoplasms	TRPS1	0.000313	3.223952	*
1989	Malignant Neoplasms	AACS	0.005142	3.239776	*
1990	Malignant Neoplasms	CA9	0	3.241118	*
1991	Malignant Neoplasms	COPS7A	0.00274	3.259742	*
1992	Malignant Neoplasms	GRAMD1B	0	3.261789	*
1993	Malignant Neoplasms	CDKN2A	0	3.262812	*
1994	Malignant Neoplasms	RNF32	0	3.26672	*
1995	Malignant Neoplasms	GANAB	0.00274	3.283045	*
1996	Malignant Neoplasms	KATNB1	0.005681	3.285481	*
1997	Malignant Neoplasms	SLC9A7	0	3.2869	*
1998	Malignant Neoplasms	OSBPL10	0.004146	3.287311	*

1999	Malignant Neoplasms	SSRP1	0.004661	3.296498	*
2000	Malignant Neoplasms	SRPR	0.005681	3.296962	*
2001	Malignant Neoplasms	ITGA11	0	3.303535	*
2002	Malignant Neoplasms	GMPS	0.004518	3.311997	*
2003	Malignant Neoplasms	FIBP	0.002702	3.321244	*
2004	Malignant Neoplasms	ADAMTS2	0	3.332996	*
2005	Malignant Neoplasms	NOC4L	0.003716	3.340548	*
2006	Malignant Neoplasms	RCC2	0.005681	3.350207	*
2007	Malignant Neoplasms	P4HA3	0	3.358196	*
2008	Malignant Neoplasms	RPN2	0.00274	3.377154	*
2009	Malignant Neoplasms	PA2G4	0.00274	3.385057	*
2010	Malignant Neoplasms	BMP7	0	3.38987	*
2011	Malignant Neoplasms	BFSP1	0	3.394537	*
2012	Malignant Neoplasms	NOP14	0.004661	3.39778	*
2013	Malignant Neoplasms	LPCAT1	0	3.398193	*
2014	Malignant Neoplasms	KPNB1	0.004792	3.418362	*
2015	Malignant Neoplasms	PTPRH	0.000291	3.466	*
2016	Malignant Neoplasms	EZH2	0.005681	3.483052	*
2017	Malignant Neoplasms	TCF7	0	3.484109	*
2018	Malignant Neoplasms	LASS2	0.00613	3.511735	*
2019	Malignant Neoplasms	NAT10	0.004518	3.51483	*
2020	Malignant Neoplasms	IRAK2	0	3.526085	*
2021	Malignant Neoplasms	NEU3	0.005925	3.553583	*
2022	Malignant Neoplasms	SYNGAP1	0.0052	3.554779	*
2023	Malignant Neoplasms	C13orf3	0	3.560351	*
2024	Malignant Neoplasms	ADAMTS6	0	3.581777	*
2025	Malignant Neoplasms	SF3B3	0.005501	3.613169	*
2026	Malignant Neoplasms	GLS2	0	3.614199	*
2027	Malignant Neoplasms	DPF2	0.005681	3.620165	*
2028	Malignant Neoplasms	HIAT1	0.005479	3.620712	*
2029	Malignant Neoplasms	TRIM28	0.004518	3.638133	*
2030	Malignant Neoplasms	PRMT5	0.003998	3.657105	*
2031	Malignant Neoplasms	G6PD	0.00274	3.692506	*
2032	Malignant Neoplasms	USP5	0.003716	3.700412	*
2033	Malignant Neoplasms	CSDA	0.005077	3.70852	*
2034	Malignant Neoplasms	RGNEF	0.000156	3.713951	*
2035	Malignant Neoplasms	SIM2	0	3.727166	*
2036	Malignant Neoplasms	NKD1	0	3.731564	*
2037	Malignant Neoplasms	ARMC6	0.004518	3.744797	*
2038	Malignant Neoplasms	ALDH3B2	0	3.771741	*
2039	Malignant Neoplasms	LUZP6	0.004518	3.782718	*
2040	Malignant Neoplasms	ECT2	0	3.796085	*
2041	Malignant Neoplasms	HS6ST2	0	3.806945	*
2042	Malignant Neoplasms	PREB	0.004956	3.832184	*
2043	Malignant Neoplasms	TBX20	0	3.863409	*
2044	Malignant Neoplasms	HAPLN3	0	3.864986	*
2045	Malignant Neoplasms	PHF16	0.00274	3.915459	*
2046	Malignant Neoplasms	NMT1	0.005681	3.920484	*
2047	Malignant Neoplasms	LEPRE1	0	3.929111	*
2048	Malignant Neoplasms	FAM150A	0	3.955729	*

2049	Malignant Neoplasms	CSGALNACT1	0	3.983069	*
2050	Malignant Neoplasms	POLD2	0.005681	3.983102	*
2051	Malignant Neoplasms	VWA2	0	3.985745	*
2052	Malignant Neoplasms	KLHL11	0.004146	4.02168	*
2053	Malignant Neoplasms	ESYT1	0.004518	4.039931	*
2054	Malignant Neoplasms	ARNTL2	0	4.06639	*
2055	Malignant Neoplasms	LRRC34	0	4.101056	*
2056	Malignant Neoplasms	PJA1	0	4.103211	*
2057	Malignant Neoplasms	TXNDC5	0.003331	4.108174	*
2058	Malignant Neoplasms	GART	0.004661	4.111965	*
2059	Malignant Neoplasms	IL24	0	4.120371	*
2060	Malignant Neoplasms	UNC119B	0.005142	4.129353	*
2061	Malignant Neoplasms	GAS2	0	4.130475	*
2062	Malignant Neoplasms	IPO5	0.000543	4.148212	*
2063	Malignant Neoplasms	SMYD5	0.005205	4.192783	*
2064	Malignant Neoplasms	EFTUD2	0.004518	4.19284	*
2065	Malignant Neoplasms	PTP4A3	0	4.198752	*
2066	Malignant Neoplasms	NT5C3L	0.004078	4.209745	*
2067	Malignant Neoplasms	ACSL6	0	4.21929	*
2068	Malignant Neoplasms	NSUN5	0.00274	4.223239	*
2069	Malignant Neoplasms	C1orf135	0	4.242459	*
2070	Malignant Neoplasms	GPN3	0.005925	4.245142	*
2071	Malignant Neoplasms	SNTB1	0	4.273359	*
2072	Malignant Neoplasms	OGFOD1	0.002827	4.274142	*
2073	Malignant Neoplasms	TNFSF11	0	4.302181	*
2074	Malignant Neoplasms	ZNF473	0.000543	4.320616	*
2075	Malignant Neoplasms	KLC2	0.005799	4.347443	*
2076	Malignant Neoplasms	MTHFD1L	0	4.380449	*
2077	Malignant Neoplasms	STC1	0	4.395318	*
2078	Malignant Neoplasms	ODAM	0	4.447008	*
2079	Malignant Neoplasms	WDR66	0	4.447168	*
2080	Malignant Neoplasms	MORN4	0.004078	4.44856	*
2081	Malignant Neoplasms	CSF2	0	4.465932	*
2082	Malignant Neoplasms	COQ10A	0.00274	4.468316	*
2083	Malignant Neoplasms	NONO	0.002827	4.4933	*
2084	Malignant Neoplasms	PAH	0	4.501848	*
2085	Malignant Neoplasms	TOMM34	0.004392	4.503749	*
2086	Malignant Neoplasms	LOXL2	0	4.528196	*
2087	Malignant Neoplasms	NEK5	0	4.545746	*
2088	Malignant Neoplasms	CD81	0.00447	4.590045	*
2089	Malignant Neoplasms	OLFML2B	0	4.638492	*
2090	Malignant Neoplasms	MMP11	0	4.677703	*
2091	Malignant Neoplasms	NOC3L	0.005681	4.69697	*
2092	Malignant Neoplasms	GRHL1	0	4.760864	*
2093	Malignant Neoplasms	FTSJ1	0.004078	4.767414	*
2094	Malignant Neoplasms	LDHB	0.002827	4.81117	*
2095	Malignant Neoplasms	PLAU	0	4.841344	*
2096	Malignant Neoplasms	DTD1	0.005681	4.857716	*
2097	Malignant Neoplasms	DIO2	0	4.877771	*
2098	Malignant Neoplasms	XPO5	0.00184	4.886191	*

2099	Malignant Neoplasms	G6PC3	0.00274	4.886922	*
2100	Malignant Neoplasms	HCN1	0	4.993712	*
2101	Malignant Neoplasms	ICOS	0	5.13095	*
2102	Malignant Neoplasms	COLEC11	0	5.278582	*
2103	Malignant Neoplasms	CYP39A1	0	5.382667	*
2104	Malignant Neoplasms	CDH6	0	5.400039	*
2105	Malignant Neoplasms	TEX10	0.00274	5.426288	*
2106	Malignant Neoplasms	GRIN2B	0	5.605313	*
2107	Malignant Neoplasms	PDPN	0	5.623195	*
2108	Malignant Neoplasms	TSPYL2	0.00274	5.747126	*
2109	Malignant Neoplasms	EGFL6	0	5.80168	*
2110	Malignant Neoplasms	ANGPT2	0	5.811083	*
2111	Malignant Neoplasms	TMEM185B	0.003716	5.837496	*
2112	Malignant Neoplasms	HNF1B	0.000037	5.861257	*
2113	Malignant Neoplasms	HSPA13	0.005681	6.341991	*
2114	Malignant Neoplasms	ADAMTS12	0	6.346981	*
2115	Malignant Neoplasms	YARS	0.00274	6.348424	*
2116	Malignant Neoplasms	SLC7A5	0	6.486225	*
2117	Malignant Neoplasms	KRT80	0	6.582092	*
2118	Malignant Neoplasms	MMP12	0	6.617377	*
2119	Malignant Neoplasms	JUB	0	6.6753	*
2120	Malignant Neoplasms	DPP7	0.006116	6.682502	*
2121	Malignant Neoplasms	MFAP2	0	6.758639	*
2122	Malignant Neoplasms	COL11A1	0	7.090537	*
2123	Malignant Neoplasms	SPP1	0	7.465531	*
2124	Malignant Neoplasms	GRPEL2	0.004661	7.668546	*
2125	Malignant Neoplasms	COL10A1	0	7.669827	*
2126	Malignant Neoplasms	LEMD1	0	7.698407	*
2127	Malignant Neoplasms	DAPP1	0.000647	7.985603	*
2128	Malignant Neoplasms	MTHFD2	0.004146	8.186467	*
2129	Malignant Neoplasms	INHBA	0	8.535336	*
2130	Malignant Neoplasms	ESM1	0	8.550091	*
2131	Malignant Neoplasms	KAL1	0	8.67666	*
2132	Malignant Neoplasms	ADAM12	0	9.08807	*
2133	Malignant Neoplasms	ASNS	0.005077	9.20214	*
2134	Malignant Neoplasms	ACCN2	0.004094	10.236928	*
2135	Malignant Neoplasms	KIAA1199	0	10.5275	*
2136	Malignant Neoplasms	CHI3L1	0	10.717129	*
2137	Malignant Neoplasms	BMP2	0.000377	10.915956	*
2138	Malignant Neoplasms	MSX2	0	11.332886	*
2139	Malignant Neoplasms	FOXQ1	0.00581	11.687135	*
2140	Malignant Neoplasms	SLC39A6	0.006116	11.756536	*
2141	Malignant Neoplasms	SLC3A2	0.004792	14.29764	*
2142	Malignant Neoplasms	EFNA5	0.000114	15.739517	*
2143	Malignant Neoplasms	CDH3	0	16.214804	*
2144	Malignant Neoplasms	FAP	0	18.387175	*
2145	Malignant Neoplasms	MMP3	0	19.590228	*
2146	Malignant Neoplasms	MMP7	0	32.841944	*
2147	Malignant Neoplasms	APOH	0.000095	45.459999	*
2148	Malignant Neoplasms	FXYD5	0.004698	64.324786	*

2149	Malignant Neoplasms	AGR3	0.000067	99.473247	*
2150	Malignant Neoplasms	AGR2	0.000047	239.538376	*
2151	Malignant tumor of colon	BCAS1	0.004661	-17.453571	*
2152	Malignant tumor of colon	AKAP5	0.000543	-7.672727	*
2153	Malignant tumor of colon	SLC16A9	0.00274	-4.44136	*
2154	Malignant tumor of colon	TP53I3	0.003331	-2.596453	*
2155	Malignant tumor of colon	FANCE	0.005681	2.118416	*
2156	Malignant tumor of colon	REXO2	0.005681	2.160419	*
2157	Malignant tumor of colon	ZNF526	0.005925	2.162342	*
2158	Malignant tumor of colon	RHEBL1	0.005681	2.261905	*
2159	Malignant tumor of colon	RPS19BP1	0.003331	2.26466	*
2160	Malignant tumor of colon	AARSD1	0.00613	2.270759	*
2161	Malignant tumor of colon	ODF2	0.005925	2.309888	*
2162	Malignant tumor of colon	DVL2	0.004792	2.341063	*
2163	Malignant tumor of colon	MED20	0.005681	2.355722	*
2164	Malignant tumor of colon	SEH1L	0.004518	2.360705	*
2165	Malignant tumor of colon	VDAC3	0.004518	2.387827	*
2166	Malignant tumor of colon	CASC3	0.005681	2.403027	*
2167	Malignant tumor of colon	TMEM99	0.004518	2.45	*
2168	Malignant tumor of colon	DHX57	0.004792	2.473856	*
2169	Malignant tumor of colon	LIPE	0.006116	2.478632	*
2170	Malignant tumor of colon	DDX55	0.004661	2.49934	*
2171	Malignant tumor of colon	UBQLN1	0.004661	2.508627	*
2172	Malignant tumor of colon	AP2M1	0.004792	2.519507	*
2173	Malignant tumor of colon	INTS5	0.004661	2.532124	*
2174	Malignant tumor of colon	DHX30	0.003998	2.555366	*
2175	Malignant tumor of colon	SNRNP48	0.004094	2.563389	*
2176	Malignant tumor of colon	GSK3A	0.00274	2.598728	*
2177	Malignant tumor of colon	BAT1	0.004146	2.612366	*
2178	Malignant tumor of colon	DIABLO	0.004661	2.621987	*
2179	Malignant tumor of colon	WSB2	0.004392	2.622514	*
2180	Malignant tumor of colon	SLC25A15	0.005681	2.654579	*
2181	Malignant tumor of colon	POLDIP2	0.003716	2.671356	*
2182	Malignant tumor of colon	ADSL	0.005568	2.705135	*
2183	Malignant tumor of colon	ZNF74	0.004146	2.735369	*
2184	Malignant tumor of colon	TFAP4	0.005681	2.735507	*
2185	Malignant tumor of colon	FAM136A	0.005777	2.752235	*
2186	Malignant tumor of colon	MLF2	0.000543	2.764901	*
2187	Malignant tumor of colon	DDX54	0.003115	2.767251	*
2188	Malignant tumor of colon	MEN1	0.005391	2.787758	*
2189	Malignant tumor of colon	ZW10	0.004351	2.787995	*
2190	Malignant tumor of colon	RNH1	0.004792	2.804233	*
2191	Malignant tumor of colon	TMEM222	0.004146	2.815126	*
2192	Malignant tumor of colon	TMEM43	0.005925	2.829317	*
2193	Malignant tumor of colon	AP3M1	0.004518	2.900102	*
2194	Malignant tumor of colon	DCPS	0.005681	2.902001	*
2195	Malignant tumor of colon	PDIA6	0.004518	2.90568	*
2196	Malignant tumor of colon	MRPL37	0.003331	2.913286	*
2197	Malignant tumor of colon	TRAPPC4	0.004101	2.913919	*
2198	Malignant tumor of colon	EDEM2	0.00581	2.927928	*

2199	Malignant tumor of colon	VPS4A	0.005681	2.938373	*
2200	Malignant tumor of colon	ALG3	0.005978	2.939251	*
2201	Malignant tumor of colon	FAM134C	0.005681	2.947311	*
2202	Malignant tumor of colon	NUMBL	0.004222	2.960859	*
2203	Malignant tumor of colon	TMEM39B	0.004518	2.961722	*
2204	Malignant tumor of colon	SHMT2	0.001709	2.980376	*
2205	Malignant tumor of colon	PAFAH1B2	0.004661	2.988591	*
2206	Malignant tumor of colon	STT3A	0.00531	2.993854	*
2207	Malignant tumor of colon	SLC25A39	0.002827	3.001813	*
2208	Malignant tumor of colon	GNS	0.004518	3.015521	*
2209	Malignant tumor of colon	EXTL2	0.004146	3.033154	*
2210	Malignant tumor of colon	M6PR	0.005681	3.035629	*
2211	Malignant tumor of colon	DDB1	0.004146	3.057781	*
2212	Malignant tumor of colon	PSMF1	0.005681	3.094299	*
2213	Malignant tumor of colon	RHOT1	0.005681	3.134272	*
2214	Malignant tumor of colon	BUB3	0.004183	3.142091	*
2215	Malignant tumor of colon	DDX50	0.002827	3.14262	*
2216	Malignant tumor of colon	ZRANB1	0.005681	3.146275	*
2217	Malignant tumor of colon	NOP56	0.00613	3.148926	*
2218	Malignant tumor of colon	PWP2	0.004792	3.149072	*
2219	Malignant tumor of colon	PHF20	0.006116	3.164103	*
2220	Malignant tumor of colon	CPSF4	0.004078	3.175705	*
2221	Malignant tumor of colon	NANP	0.004255	3.185112	*
2222	Malignant tumor of colon	RPUSD4	0.00274	3.207434	*
2223	Malignant tumor of colon	AACS	0.005142	3.239776	*
2224	Malignant tumor of colon	COPS7A	0.00274	3.259742	*
2225	Malignant tumor of colon	GANAB	0.00274	3.283045	*
2226	Malignant tumor of colon	KATNB1	0.005681	3.285481	*
2227	Malignant tumor of colon	OSBPL10	0.004146	3.287311	*
2228	Malignant tumor of colon	RUVBL2	0.006112	3.290995	*
2229	Malignant tumor of colon	SSRP1	0.004661	3.296498	*
2230	Malignant tumor of colon	SRPR	0.005681	3.296962	*
2231	Malignant tumor of colon	GMPS	0.004518	3.311997	*
2232	Malignant tumor of colon	FIBP	0.002702	3.321244	*
2233	Malignant tumor of colon	NOC4L	0.003716	3.340548	*
2234	Malignant tumor of colon	RCC2	0.005681	3.350207	*
2235	Malignant tumor of colon	RPN2	0.00274	3.377154	*
2236	Malignant tumor of colon	PA2G4	0.00274	3.385057	*
2237	Malignant tumor of colon	NOP14	0.004661	3.39778	*
2238	Malignant tumor of colon	KPNB1	0.004792	3.418362	*
2239	Malignant tumor of colon	PPP2CA	0.005558	3.478573	*
2240	Malignant tumor of colon	EZH2	0.005681	3.483052	*
2241	Malignant tumor of colon	LASS2	0.00613	3.511735	*
2242	Malignant tumor of colon	NAT10	0.004518	3.51483	*
2243	Malignant tumor of colon	NEU3	0.005925	3.553583	*
2244	Malignant tumor of colon	SYNGAP1	0.0052	3.554779	*
2245	Malignant tumor of colon	ENO1	0.00274	3.587509	*
2246	Malignant tumor of colon	SF3B3	0.005501	3.613169	*
2247	Malignant tumor of colon	DPF2	0.005681	3.620165	*
2248	Malignant tumor of colon	HIAT1	0.005479	3.620712	*

2249	Malignant tumor of colon	TRIM28	0.004518	3.638133	*
2250	Malignant tumor of colon	PRMT5	0.003998	3.657105	*
2251	Malignant tumor of colon	G6PD	0.00274	3.692506	*
2252	Malignant tumor of colon	USP5	0.003716	3.700412	*
2253	Malignant tumor of colon	CSDA	0.005077	3.70852	*
2254	Malignant tumor of colon	ARMC6	0.004518	3.744797	*
2255	Malignant tumor of colon	RAN	0.006116	3.745088	*
2256	Malignant tumor of colon	LUZP6	0.004518	3.782718	*
2257	Malignant tumor of colon	VARS	0.00184	3.789916	*
2258	Malignant tumor of colon	PREB	0.004956	3.832184	*
2259	Malignant tumor of colon	RUVBL1	0.005681	3.875942	*
2260	Malignant tumor of colon	TCF7	0.004146	3.880342	*
2261	Malignant tumor of colon	PHF16	0.00274	3.915459	*
2262	Malignant tumor of colon	NMT1	0.005681	3.920484	*
2263	Malignant tumor of colon	POLD2	0.005681	3.983102	*
2264	Malignant tumor of colon	KLHL11	0.004146	4.02168	*
2265	Malignant tumor of colon	ESYT1	0.004518	4.039931	*
2266	Malignant tumor of colon	TEAD4	0.005681	4.044044	*
2267	Malignant tumor of colon	TXNDC5	0.003331	4.108174	*
2268	Malignant tumor of colon	GART	0.004661	4.111965	*
2269	Malignant tumor of colon	UNC119B	0.005142	4.129353	*
2270	Malignant tumor of colon	IPO5	0.000543	4.148212	*
2271	Malignant tumor of colon	SMYD5	0.005205	4.192783	*
2272	Malignant tumor of colon	EFTUD2	0.004518	4.19284	*
2273	Malignant tumor of colon	NT5C3L	0.004078	4.209745	*
2274	Malignant tumor of colon	NSUN5	0.00274	4.223239	*
2275	Malignant tumor of colon	GPN3	0.005925	4.245142	*
2276	Malignant tumor of colon	OGFOD1	0.002827	4.274142	*
2277	Malignant tumor of colon	ZNF473	0.000543	4.320616	*
2278	Malignant tumor of colon	KLC2	0.005799	4.347443	*
2279	Malignant tumor of colon	MORN4	0.004078	4.44856	*
2280	Malignant tumor of colon	COQ10A	0.00274	4.468316	*
2281	Malignant tumor of colon	NONO	0.002827	4.4933	*
2282	Malignant tumor of colon	TOMM34	0.004392	4.503749	*
2283	Malignant tumor of colon	NOC3L	0.005681	4.69697	*
2284	Malignant tumor of colon	FTSJ1	0.004078	4.767414	*
2285	Malignant tumor of colon	TPD52L2	0.004078	4.788684	*
2286	Malignant tumor of colon	LDHB	0.002827	4.81117	*
2287	Malignant tumor of colon	DTD1	0.005681	4.857716	*
2288	Malignant tumor of colon	XPO5	0.00184	4.886191	*
2289	Malignant tumor of colon	G6PC3	0.00274	4.886922	*
2290	Malignant tumor of colon	WDR74	0.004146	5.075321	*
2291	Malignant tumor of colon	AHCY	0.004518	5.223647	*
2292	Malignant tumor of colon	TEX10	0.00274	5.426288	*
2293	Malignant tumor of colon	CIRH1A	0.00274	5.542027	*
2294	Malignant tumor of colon	TSPYL2	0.00274	5.747126	*
2295	Malignant tumor of colon	TMEM185B	0.003716	5.837496	*
2296	Malignant tumor of colon	CDK4	0.004518	6.24989	*
2297	Malignant tumor of colon	HSPA13	0.005681	6.341991	*
2298	Malignant tumor of colon	YARS	0.00274	6.348424	*

2299	Malignant tumor of colon	DPP7	0.006116	6.682502	*
2300	Malignant tumor of colon	SIM2	0.0052	7.317708	*
2301	Malignant tumor of colon	GRPEL2	0.004661	7.668546	*
2302	Malignant tumor of colon	MTHFD2	0.004146	8.186467	*
2303	Malignant tumor of colon	CD81	0.004661	8.248565	*
2304	Malignant tumor of colon	ASNS	0.005077	9.20214	*
2305	Malignant tumor of colon	ACCN2	0.004094	10.236928	*
2306	Malignant tumor of colon	GRB10	0.003716	10.421296	*
2307	Malignant tumor of colon	LDLRAD3	0.00613	11.487923	*
2308	Malignant tumor of colon	FOXQ1	0.00581	11.687135	*
2309	Malignant tumor of colon	SLC39A6	0.006116	11.756536	*
2310	Malignant tumor of colon	SLC3A2	0.004792	14.29764	*
2311	Malignant tumor of colon	PITX1	0.004952	22.45	*
2312	Malignant tumor of colon	HS6ST2	0.004518	24.301075	*
2313	Malignant tumor of colon	FXYD5	0.004698	64.324786	*
2314	Mutant	TGFBI	0	-3.000013	*
2315	Mutant	FOXA2	0.000003	18.266987	*
2316	Mutant	TM4SF1	0.000002	117.507367	*
2317	Neoplasm	PYY	0	-32.95913	*
2318	Neoplasm	FAM92A1	0.004874	-22.777354	*
2319	Neoplasm	SCGN	0	-21.371687	*
2320	Neoplasm	ITGA5	0.000005	-20.20304	*
2321	Neoplasm	BEST4	0	-18.781625	*
2322	Neoplasm	CA7	0	-17.501116	*
2323	Neoplasm	TPH1	0	-16.890323	*
2324	Neoplasm	GUCA2A	0	-16.294008	*
2325	Neoplasm	CLCA1	0	-13.979504	*
2326	Neoplasm	FLJ21511	0	-13.766835	*
2327	Neoplasm	SLC26A3	0	-13.167276	*
2328	Neoplasm	PKIB	0	-12.911139	*
2329	Neoplasm	AQP8	0	-12.860219	*
2330	Neoplasm	EPDR1	0.00804	-12.748749	*
2331	Neoplasm	MYH11	0.000001	-12.464317	*
2332	Neoplasm	SLC26A2	0	-11.558375	*
2333	Neoplasm	HSPA12A	0.001873	-11.29625	*
2334	Neoplasm	SST	0	-10.965373	*
2335	Neoplasm	AKAP2	0.000037	-10.381697	*
2336	Neoplasm	ADAMDEC1	0	-10.152414	*
2337	Neoplasm	ARHGAP4	0.009376	-10.004707	*
2338	Neoplasm	MXRA7	0.000057	-9.581207	*
2339	Neoplasm	APBB1	0.002109	-9.463769	*
2340	Neoplasm	ARNT2	0.000153	-9.389616	*
2341	Neoplasm	CA1	0	-9.053477	*
2342	Neoplasm	SLC4A4	0	-8.667366	*
2343	Neoplasm	BMP3	0	-8.145102	*
2344	Neoplasm	GREM2	0	-7.997592	*
2345	Neoplasm	PADI2	0	-7.936984	*
2346	Neoplasm	LRRC19	0	-7.805174	*
2347	Neoplasm	SLC30A10	0	-7.69742	*
2348	Neoplasm	DNAJC15	0.005998	-7.693302	*

2349	Neoplasm	EZR	0.000093	-7.689489	*
2350	Neoplasm	ABCG2	0	-7.647563	*
2351	Neoplasm	C7	0	-7.633682	*
2352	Neoplasm	CEACAM7	0	-7.545962	*
2353	Neoplasm	PLP1	0	-7.473971	*
2354	Neoplasm	ANO5	0	-7.472716	*
2355	Neoplasm	SPIB	0	-7.409097	*
2356	Neoplasm	IL1R2	0	-7.380576	*
2357	Neoplasm	CCL23	0	-7.357297	*
2358	Neoplasm	AHCYL2	0	-7.340119	*
2359	Neoplasm	CFD	0	-7.220538	*
2360	Neoplasm	AKR1B10	0	-6.975418	*
2361	Neoplasm	ADA	0.000918	-6.871434	*
2362	Neoplasm	MAMDC2	0	-6.862575	*
2363	Neoplasm	KLHL29	0.000035	-6.781406	*
2364	Neoplasm	PECI	0.000314	-6.594706	*
2365	Neoplasm	SCARA5	0	-6.568566	*
2366	Neoplasm	MT1X	0.000104	-6.547131	*
2367	Neoplasm	PGM5	0	-6.534718	*
2368	Neoplasm	ANGPTL1	0	-6.533121	*
2369	Neoplasm	ABCA8	0	-6.270179	*
2370	Neoplasm	PTGDR	0	-6.211751	*
2371	Neoplasm	PDE9A	0	-6.156069	*
2372	Neoplasm	GUCA2B	0	-6.089189	*
2373	Neoplasm	NEDD4L	0	-6.022941	*
2374	Neoplasm	ZCCHC24	0.000066	-5.924274	*
2375	Neoplasm	MT1M	0	-5.911706	*
2376	Neoplasm	BHLHB9	0.000023	-5.832213	*
2377	Neoplasm	FABP1	0	-5.810727	*
2378	Neoplasm	SMPDL3A	0	-5.769912	*
2379	Neoplasm	GLP2R	0	-5.737624	*
2380	Neoplasm	TSPAN7	0	-5.645584	*
2381	Neoplasm	ADH1B	0	-5.554404	*
2382	Neoplasm	KLF4	0	-5.50973	*
2383	Neoplasm	COL1A1	0	-5.27529	*
2384	Neoplasm	RNF152	0	-5.212137	*
2385	Neoplasm	ANPEP	0	-5.185258	*
2386	Neoplasm	PDK4	0	-5.092727	*
2387	Neoplasm	KCTD14	0.00797	-5.072233	*
2388	Neoplasm	PLAUR	0.000681	-4.982758	*
2389	Neoplasm	UGT1A8	0	-4.980708	*
2390	Neoplasm	VSIG2	0	-4.980351	*
2391	Neoplasm	CKB	0	-4.979591	*
2392	Neoplasm	DUSP26	0	-4.84096	*
2393	Neoplasm	SULT1A2	0	-4.83369	*
2394	Neoplasm	STYK1	0	-4.813761	*
2395	Neoplasm	PLAC8	0	-4.784491	*
2396	Neoplasm	DES	0	-4.751915	*
2397	Neoplasm	SHANK2	0.002489	-4.738355	*
2398	Neoplasm	KRT24	0	-4.737791	*

2399	Neoplasm	STMN2	0	-4.728854	*
2400	Neoplasm	CYFIP2	0.009193	-4.705447	*
2401	Neoplasm	ABI3BP	0	-4.621097	*
2402	Neoplasm	MGC13057	0	-4.559318	*
2403	Neoplasm	CNTN3	0	-4.497293	*
2404	Neoplasm	SLC2A4	0	-4.477195	*
2405	Neoplasm	CA2	0	-4.439932	*
2406	Neoplasm	TNFRSF21	0.001076	-4.374131	*
2407	Neoplasm	LPHN3	0	-4.236286	*
2408	Neoplasm	ITM2C	0	-4.229678	*
2409	Neoplasm	CHGA	0	-4.229168	*
2410	Neoplasm	XKR4	0	-4.223866	*
2411	Neoplasm	RIN3	0.001752	-4.184932	*
2412	Neoplasm	MYOM1	0	-4.090315	*
2413	Neoplasm	LIFR	0	-4.027879	*
2414	Neoplasm	ABCA5	0	-3.975181	*
2415	Neoplasm	TSPAN5	0.007824	-3.9319	*
2416	Neoplasm	ASB2	0	-3.873691	*
2417	Neoplasm	LDHD	0	-3.865801	*
2418	Neoplasm	PTPRH	0.000006	-3.847104	*
2419	Neoplasm	DST	0	-3.84509	*
2420	Neoplasm	EDIL3	0	-3.838001	*
2421	Neoplasm	NOD2	0.001996	-3.814115	*
2422	Neoplasm	NCAM1	0	-3.803955	*
2423	Neoplasm	PDCD4	0	-3.780027	*
2424	Neoplasm	KREMEN1	0.001422	-3.771902	*
2425	Neoplasm	MYOT	0	-3.765735	*
2426	Neoplasm	LOC100133036	0.000455	-3.761576	*
2427	Neoplasm	ASPA	0	-3.759596	*
2428	Neoplasm	ENDOD1	0	-3.756257	*
2429	Neoplasm	HPGD	0	-3.728274	*
2430	Neoplasm	ABHD3	0	-3.704323	*
2431	Neoplasm	FAM107A	0	-3.676289	*
2432	Neoplasm	CLMN	0	-3.65346	*
2433	Neoplasm	IL27RA	0.010921	-3.625801	*
2434	Neoplasm	FGFR2	0	-3.619085	*
2435	Neoplasm	KRT20	0	-3.617039	*
2436	Neoplasm	FGL2	0	-3.611344	*
2437	Neoplasm	SYTL2	0	-3.600182	*
2438	Neoplasm	TTLL6	0	-3.584756	*
2439	Neoplasm	UGDH	0	-3.557058	*
2440	Neoplasm	MT1F	0.000013	-3.521489	*
2441	Neoplasm	NR3C2	0	-3.51958	*
2442	Neoplasm	MT1H	0.000111	-3.498151	*
2443	Neoplasm	UGP2	0	-3.476231	*
2444	Neoplasm	IL6R	0	-3.472127	*
2445	Neoplasm	C1orf115	0	-3.453543	*
2446	Neoplasm	CHL1	0	-3.44257	*
2447	Neoplasm	SEPP1	0.000081	-3.413388	*
2448	Neoplasm	NAP1L2	0	-3.408223	*

2449	Neoplasm	LGALS4	0	-3.375029	*
2450	Neoplasm	GNG7	0	-3.345331	*
2451	Neoplasm	FTH1	0	-3.343445	*
2452	Neoplasm	GSN	0	-3.342984	*
2453	Neoplasm	MGLL	0	-3.330854	*
2454	Neoplasm	SCN9A	0	-3.33028	*
2455	Neoplasm	VWA5A	0.000217	-3.294359	*
2456	Neoplasm	SLC9A2	0	-3.285396	*
2457	Neoplasm	CMAH	0	-3.269691	*
2458	Neoplasm	ENTPD5	0	-3.256894	*
2459	Neoplasm	ASAHL	0	-3.234965	*
2460	Neoplasm	LRRN2	0	-3.229834	*
2461	Neoplasm	PPP2R3A	0	-3.227965	*
2462	Neoplasm	TP53INP2	0	-3.225235	*
2463	Neoplasm	ADHFE1	0	-3.214011	*
2464	Neoplasm	IL8	0	-3.187774	*
2465	Neoplasm	GCNT3	0	-3.173418	*
2466	Neoplasm	SLC9A9	0	-3.13727	*
2467	Neoplasm	PRPH	0	-3.134704	*
2468	Neoplasm	COL1A2	0	-3.116379	*
2469	Neoplasm	ZZEF1	0	-3.11274	*
2470	Neoplasm	RCAN2	0	-3.079993	*
2471	Neoplasm	H2AFY2	0.001143	-3.062461	*
2472	Neoplasm	RPS6KA2	0.000906	-3.03861	*
2473	Neoplasm	RGS9	0	-3.036885	*
2474	Neoplasm	SSPN	0	-2.996206	*
2475	Neoplasm	ETFDH	0	-2.976442	*
2476	Neoplasm	SCNN1B	0	-2.974333	*
2477	Neoplasm	MATN2	0	-2.966749	*
2478	Neoplasm	NIN	0.002411	-2.950444	*
2479	Neoplasm	PLCL2	0	-2.919786	*
2480	Neoplasm	SMA4	0.002489	-2.881049	*
2481	Neoplasm	SEMA4G	0.000002	-2.809155	*
2482	Neoplasm	BCAR3	0	-2.801465	*
2483	Neoplasm	ETHE1	0	-2.789342	*
2484	Neoplasm	PLCE1	0	-2.783588	*
2485	Neoplasm	SELENBP1	0	-2.780562	*
2486	Neoplasm	LGALS2	0	-2.769808	*
2487	Neoplasm	FAM82A1	0	-2.720413	*
2488	Neoplasm	SRI	0	-2.719712	*
2489	Neoplasm	C21orf91	0.002861	-2.717137	*
2490	Neoplasm	KLF9	0	-2.712093	*
2491	Neoplasm	NEGR1	0	-2.694065	*
2492	Neoplasm	CERK	0.000002	-2.692917	*
2493	Neoplasm	LPAR1	0	-2.687341	*
2494	Neoplasm	METTL7A	0	-2.682637	*
2495	Neoplasm	MUSK	0	-2.675855	*
2496	Neoplasm	MAOA	0	-2.629701	*
2497	Neoplasm	GPER	0	-2.589188	*
2498	Neoplasm	ARAP3	0.001833	-2.566309	*

2499	Neoplasm	PKN3	0.004844	-2.558328	*
2500	Neoplasm	STXBP1	0.000493	-2.546501	*
2501	Neoplasm	GCNT2	0	-2.528225	*
2502	Neoplasm	MALL	0	-2.526251	*
2503	Neoplasm	CDADC1	0.010743	-2.517064	*
2504	Neoplasm	CCL15	0	-2.514962	*
2505	Neoplasm	GNAZ	0.006346	-2.490276	*
2506	Neoplasm	GFRA2	0	-2.441722	*
2507	Neoplasm	SLC25A23	0	-2.433656	*
2508	Neoplasm	FAM122B	0.001461	-2.429466	*
2509	Neoplasm	RDH5	0	-2.421524	*
2510	Neoplasm	PAPSS2	0.000012	-2.421199	*
2511	Neoplasm	LDLRAD3	0.004268	-2.392044	*
2512	Neoplasm	PBLD	0.000056	-2.361208	*
2513	Neoplasm	FEV	0	-2.345562	*
2514	Neoplasm	AMOT	0.000438	-2.342343	*
2515	Neoplasm	NUCB2	0.000906	-2.339025	*
2516	Neoplasm	C12orf75	0.001734	-2.317002	*
2517	Neoplasm	EPHX2	0	-2.313872	*
2518	Neoplasm	CCL20	0	-2.310437	*
2519	Neoplasm	GADD45B	0.000069	-2.281425	*
2520	Neoplasm	RETSAT	0	-2.247195	*
2521	Neoplasm	MT2A	0.000103	-2.240731	*
2522	Neoplasm	FAM165B	0.002833	-2.214773	*
2523	Neoplasm	MAP4K4	0.00762	-2.208468	*
2524	Neoplasm	KDELC1	0.000398	-2.193848	*
2525	Neoplasm	SLC35A3	0.000006	-2.193479	*
2526	Neoplasm	FAM117A	0.001005	-2.147714	*
2527	Neoplasm	AFMID	0.007178	-2.142616	*
2528	Neoplasm	ASRGL1	0.000606	-2.141308	*
2529	Neoplasm	GCSH	0.000331	-2.129068	*
2530	Neoplasm	C13orf27	0.005311	-2.12821	*
2531	Neoplasm	ACTN1	0.002111	-2.095127	*
2532	Neoplasm	NDRG2	0	-2.076818	*
2533	Neoplasm	TFCP2L1	0.000006	-2.064292	*
2534	Neoplasm	ITGAE	0.001469	-2.060081	*
2535	Neoplasm	GPM6B	0.00016	-2.054248	*
2536	Neoplasm	PPARGC1B	0	-2.053557	*
2537	Neoplasm	PYY2	0	-2.037223	*
2538	Neoplasm	RGS12	0.000145	-2.034738	*
2539	Neoplasm	HBS1L	0.007833	-2.022446	*
2540	Neoplasm	STXBP4	0.000915	-2.020477	*
2541	Neoplasm	MTM1	0	-2.004337	*
2542	Neoplasm	NUP88	0.000044	-2.000945	*
2543	Neoplasm	CBX3	0.000002	2.001406	*
2544	Neoplasm	RICS	0.002281	2.00378	*
2545	Neoplasm	PPAT	0	2.003797	*
2546	Neoplasm	NAV1	0.003047	2.006438	*
2547	Neoplasm	AHI1	0.001956	2.00725	*
2548	Neoplasm	LEF1	0.000003	2.020075	*

2549	Neoplasm	KLF6	0.000317	2.026256	*
2550	Neoplasm	SLC37A1	0.003869	2.05366	*
2551	Neoplasm	BTC	0.002633	2.061006	*
2552	Neoplasm	RAB25	0	2.064122	*
2553	Neoplasm	RUVBL2	0	2.080554	*
2554	Neoplasm	PLAU	0	2.089529	*
2555	Neoplasm	RAB3IP	0.006591	2.091907	*
2556	Neoplasm	EVI1	0	2.095975	*
2557	Neoplasm	CDC42EP5	0.004285	2.097259	*
2558	Neoplasm	S100A11	0	2.125055	*
2559	Neoplasm	RELB	0.000032	2.12627	*
2560	Neoplasm	ITPR1	0.002765	2.138918	*
2561	Neoplasm	PRELID2	0.002978	2.143646	*
2562	Neoplasm	GZMB	0	2.14732	*
2563	Neoplasm	C7orf68	0	2.153691	*
2564	Neoplasm	RFFL	0.011567	2.168506	*
2565	Neoplasm	NAMPT	0.003272	2.17292	*
2566	Neoplasm	PIK3AP1	0.009172	2.191098	*
2567	Neoplasm	RUVBL1	0	2.19596	*
2568	Neoplasm	TMPRSS3	0.000022	2.198838	*
2569	Neoplasm	LIPG	0	2.199623	*
2570	Neoplasm	FOXO1	0.002141	2.202191	*
2571	Neoplasm	EPHA2	0.000809	2.238186	*
2572	Neoplasm	PELI1	0.008021	2.291038	*
2573	Neoplasm	MICAL2	0.007571	2.297255	*
2574	Neoplasm	MICB	0	2.307009	*
2575	Neoplasm	DIO2	0	2.327507	*
2576	Neoplasm	LRIG1	0.000003	2.3356	*
2577	Neoplasm	CAD	0	2.341609	*
2578	Neoplasm	SORD	0.000001	2.341712	*
2579	Neoplasm	SOX4	0	2.357896	*
2580	Neoplasm	RNF19B	0.000829	2.360887	*
2581	Neoplasm	AHCY	0	2.368393	*
2582	Neoplasm	CBFB	0	2.373268	*
2583	Neoplasm	CALD1	0.002728	2.375107	*
2584	Neoplasm	GART	0.000004	2.378751	*
2585	Neoplasm	PHLDA1	0.000078	2.380134	*
2586	Neoplasm	EPHB2	0	2.414518	*
2587	Neoplasm	CFTR	0	2.433296	*
2588	Neoplasm	ASPH	0.002489	2.433736	*
2589	Neoplasm	TKT	0	2.451599	*
2590	Neoplasm	TMTC2	0.005608	2.454877	*
2591	Neoplasm	MYC	0	2.456466	*
2592	Neoplasm	GNPDA1	0	2.457018	*
2593	Neoplasm	PSAT1	0.000001	2.45864	*
2594	Neoplasm	LAMB3	0.000398	2.471631	*
2595	Neoplasm	ANXA3	0	2.482915	*
2596	Neoplasm	PLEKHG6	0.000018	2.486377	*
2597	Neoplasm	PRSS22	0	2.510166	*
2598	Neoplasm	SLC2A1	0.011553	2.526756	*

2599	Neoplasm	CDK4	0	2.532799	*
2600	Neoplasm	LYZ	0.000146	2.538796	*
2601	Neoplasm	PUS7	0	2.587607	*
2602	Neoplasm	GBP3	0.011258	2.604555	*
2603	Neoplasm	SATB1	0.000622	2.633717	*
2604	Neoplasm	SLC6A20	0	2.639076	*
2605	Neoplasm	SERPINB5	0.000106	2.639584	*
2606	Neoplasm	SRPX2	0	2.686563	*
2607	Neoplasm	RAE1	0	2.721182	*
2608	Neoplasm	SGK493	0.000098	2.724975	*
2609	Neoplasm	XPOT	0	2.753208	*
2610	Neoplasm	FEN1	0.000001	2.754245	*
2611	Neoplasm	LAMC2	0.002514	2.755622	*
2612	Neoplasm	IL15	0.013563	2.811039	*
2613	Neoplasm	GAS6	0.001571	2.831992	*
2614	Neoplasm	PTPRB	0.011339	2.840252	*
2615	Neoplasm	SULT2B1	0	2.841578	*
2616	Neoplasm	FAP	0	2.874475	*
2617	Neoplasm	TC2N	0.000451	2.897627	*
2618	Neoplasm	ARNTL2	0	2.916175	*
2619	Neoplasm	EMP1	0.001917	2.939115	*
2620	Neoplasm	TGIF2	0	2.950146	*
2621	Neoplasm	ENC1	0	2.981497	*
2622	Neoplasm	SHMT2	0	2.982462	*
2623	Neoplasm	WNT2	0	3.026185	*
2624	Neoplasm	TCN1	0.000005	3.04367	*
2625	Neoplasm	RIPK2	0	3.046851	*
2626	Neoplasm	C6orf141	0.000221	3.055869	*
2627	Neoplasm	F12	0	3.082553	*
2628	Neoplasm	CSE1L	0	3.113288	*
2629	Neoplasm	AHR	0.001909	3.127997	*
2630	Neoplasm	TRIP13	0	3.129853	*
2631	Neoplasm	TMC7	0.003133	3.179847	*
2632	Neoplasm	EHF	0.001859	3.193743	*
2633	Neoplasm	GALNT6	0	3.209837	*
2634	Neoplasm	FOXM1	0	3.25519	*
2635	Neoplasm	B3GALTL	0.021845	3.260611	*
2636	Neoplasm	RFC3	0	3.266386	*
2637	Neoplasm	KIAA1244	0.004306	3.268442	*
2638	Neoplasm	HSPH1	0	3.304369	*
2639	Neoplasm	MMP11	0	3.315333	*
2640	Neoplasm	NEK2	0	3.324418	*
2641	Neoplasm	PHF19	0	3.363376	*
2642	Neoplasm	GTF2IRD1	0	3.438892	*
2643	Neoplasm	ISG20	0.001535	3.472504	*
2644	Neoplasm	GRTP1	0.007655	3.51089	*
2645	Neoplasm	SOX9	0	3.525201	*
2646	Neoplasm	AURKA	0	3.60953	*
2647	Neoplasm	ASPHD1	0.000059	3.685163	*
2648	Neoplasm	NOSTRIN	0.000008	3.735735	*

2649	Neoplasm	RNF43	0	3.773159	*
2650	Neoplasm	AXIN2	0	3.780513	*
2651	Neoplasm	ECT2	0	3.796085	*
2652	Neoplasm	SGK1	0.001646	3.807677	*
2653	Neoplasm	C6orf150	0.000637	3.823718	*
2654	Neoplasm	TEAD4	0	3.834439	*
2655	Neoplasm	CEACAM6	0	3.836542	*
2656	Neoplasm	MTHFD1L	0	3.850213	*
2657	Neoplasm	KIF2C	0	3.85728	*
2658	Neoplasm	PERP	0.00016	3.924924	*
2659	Neoplasm	TNFRSF12A	0	3.960342	*
2660	Neoplasm	COL3A1	0	3.961379	*
2661	Neoplasm	KIAA1549	0	3.982443	*
2662	Neoplasm	DUSP14	0	4.00981	*
2663	Neoplasm	PMAIP1	0	4.0525	*
2664	Neoplasm	CRISPLD2	0.003101	4.130039	*
2665	Neoplasm	HRASLS2	0.005377	4.150647	*
2666	Neoplasm	MAML3	0.000367	4.210823	*
2667	Neoplasm	ATP1B1	0.011528	4.236133	*
2668	Neoplasm	LRP8	0	4.270411	*
2669	Neoplasm	MACC1	0	4.314678	*
2670	Neoplasm	CFB	0	4.493717	*
2671	Neoplasm	KRT6B	0	4.60545	*
2672	Neoplasm	ITGB6	0.010505	4.691657	*
2673	Neoplasm	COL11A1	0	4.702894	*
2674	Neoplasm	TIMP1	0	4.70745	*
2675	Neoplasm	COL10A1	0	4.817021	*
2676	Neoplasm	HIG2	0	4.883932	*
2677	Neoplasm	MXRA5	0	4.893707	*
2678	Neoplasm	SCD	0	5.187389	*
2679	Neoplasm	C10orf81	0	5.202957	*
2680	Neoplasm	JUB	0	5.213452	*
2681	Neoplasm	KLK10	0	5.296705	*
2682	Neoplasm	C2	0	5.353924	*
2683	Neoplasm	HNMT	0.000612	5.36264	*
2684	Neoplasm	SLC12A2	0	5.407283	*
2685	Neoplasm	CDC25B	0	5.477842	*
2686	Neoplasm	IFITM2	0	5.573947	*
2687	Neoplasm	ANGPT2	0	5.576995	*
2688	Neoplasm	FUT1	0	5.600347	*
2689	Neoplasm	C4orf18	0.000603	5.634592	*
2690	Neoplasm	IFITM3	0	5.692057	*
2691	Neoplasm	CORO2A	0.00024	5.714594	*
2692	Neoplasm	SLC6A6	0	5.867304	*
2693	Neoplasm	RIN2	0.003922	6.118772	*
2694	Neoplasm	SLCO4A1	0	6.200143	*
2695	Neoplasm	TPX2	0	6.228968	*
2696	Neoplasm	HKDC1	0	6.281857	*
2697	Neoplasm	NFE2L3	0	6.347771	*
2698	Neoplasm	PDPN	0	6.509429	*

2699	Neoplasm	PLA2G16	0.000041	6.576225	*
2700	Neoplasm	SLC22A3	0	6.579632	*
2701	Neoplasm	MMP12	0	6.617377	*
2702	Neoplasm	MOBKL2B	0.000002	6.742703	*
2703	Neoplasm	CXCL1	0	6.760238	*
2704	Neoplasm	TGFBI	0	6.776373	*
2705	Neoplasm	HNF4G	0.000197	6.892561	*
2706	Neoplasm	IFITM1	0	7.052299	*
2707	Neoplasm	PPFIBP2	0.002111	7.097961	*
2708	Neoplasm	TUSC1	0.00533	7.119789	*
2709	Neoplasm	CXCL3	0	7.233392	*
2710	Neoplasm	SPP1	0	7.465531	*
2711	Neoplasm	RHOU	0.005679	7.751974	*
2712	Neoplasm	PLA2G4A	0.000714	7.759894	*
2713	Neoplasm	VSNL1	0	7.895906	*
2714	Neoplasm	SLC7A5	0	8.08427	*
2715	Neoplasm	ACAN	0	8.376905	*
2716	Neoplasm	ASCL2	0	9.699198	*
2717	Neoplasm	RGS2	0.004578	9.788947	*
2718	Neoplasm	S100A2	0	9.973432	*
2719	Neoplasm	C4orf19	0.009848	10.587365	*
2720	Neoplasm	TRIB3	0	11.35587	*
2721	Neoplasm	NKD2	0	11.439044	*
2722	Neoplasm	ETV4	0	11.501499	*
2723	Neoplasm	TESC	0	13.19731	*
2724	Neoplasm	KRT80	0	13.520844	*
2725	Neoplasm	CHI3L1	0	13.656201	*
2726	Neoplasm	GIPC2	0.004653	14.36361	*
2727	Neoplasm	CEACAM5	0.000107	14.723837	*
2728	Neoplasm	CDH3	0	15.2885	*
2729	Neoplasm	THBS2	0	17.081759	*
2730	Neoplasm	MMP1	0	17.200457	*
2731	Neoplasm	COL17A1	0.000002	18.405641	*
2732	Neoplasm	ALOX5	0.001012	19.6864	*
2733	Neoplasm	CLDN1	0	24.94407	*
2734	Neoplasm	KIAA1199	0	25.62671	*
2735	Neoplasm	FABP6	0	28.317192	*
2736	Neoplasm	ALDH1A1	0.011623	28.481741	*
2737	Neoplasm	INHBA	0	29.760912	*
2738	Neoplasm	CREB3L1	0.000055	32.962055	*
2739	Neoplasm	DPEP1	0	35.291528	*
2740	Neoplasm	FOXQ1	0	40.632401	*
2741	Neoplasm	MMP3	0	41.143075	*
2742	Neoplasm	MMP7	0	53.848067	*
2743	Neoplasm	KRT23	0	77.872506	*
2744	Neoplasm Metastasis	ITLN1	0	-292.109149	*
2745	Neoplasm Metastasis	ZG16	0	-33.964274	*
2746	Neoplasm Metastasis	CLCA4	0	-26.62882	*
2747	Neoplasm Metastasis	CA4	0	-15.845909	*
2748	Neoplasm Metastasis	SPINK4	0	-13.759937	*

2749	Neoplasm Metastasis	CLCA1	0	-13.405836	*
2750	Neoplasm Metastasis	CEACAM7	0	-13.361052	*
2751	Neoplasm Metastasis	FCGBP	0	-11.391719	*
2752	Neoplasm Metastasis	CA1	0	-10.844196	*
2753	Neoplasm Metastasis	ACTG2	0	-9.991796	*
2754	Neoplasm Metastasis	VSIG2	0	-9.837015	*
2755	Neoplasm Metastasis	MAB21L2	0	-9.207376	*
2756	Neoplasm Metastasis	VIP	0	-8.944965	*
2757	Neoplasm Metastasis	GUCA2A	0	-8.661259	*
2758	Neoplasm Metastasis	IGJ	0	-8.072572	*
2759	Neoplasm Metastasis	FAM55D	0	-7.716639	*
2760	Neoplasm Metastasis	AQP8	0	-7.15219	*
2761	Neoplasm Metastasis	MS4A12	0	-6.806925	*
2762	Neoplasm Metastasis	CA2	0	-6.540386	*
2763	Neoplasm Metastasis	MMP3	0	-5.405218	*
2764	Neoplasm Metastasis	ADAMDEC1	0	-5.086967	*
2765	Neoplasm Metastasis	C6ORF105	0	-4.303634	*
2766	Neoplasm Metastasis	TPSG1	0	-3.941812	*
2767	Neoplasm Metastasis	IL1R2	0	-3.923934	*
2768	Neoplasm Metastasis	MYH11	0	-3.527813	*
2769	Neoplasm Metastasis	TCF21	0	-3.244161	*
2770	Neoplasm Metastasis	SPARCL1	0.000004	-3.096174	*
2771	Neoplasm Metastasis	PYY	0	-3.042799	*
2772	Neoplasm Metastasis	ITM2C	0.000001	-2.872164	*
2773	Neoplasm Metastasis	PIGR	0	-2.867408	*
2774	Neoplasm Metastasis	BCAS1	0	-2.845971	*
2775	Neoplasm Metastasis	PLAC8	0	-2.715367	*
2776	Neoplasm Metastasis	SDCBP2	0.000331	-2.495947	*
2777	Neoplasm Metastasis	FOXF1	0	-2.466208	*
2778	Neoplasm Metastasis	TSPAN1	0	-2.411097	*
2779	Neoplasm Metastasis	FAM46C	0.000005	-2.22057	*
2780	Neoplasm Metastasis	LGALS4	0.000004	-2.055385	*
2781	Neoplasm Metastasis	NNMT	0.000001	2.298865	*
2782	Neoplasm Metastasis	BGN	0	2.314266	*
2783	Neoplasm Metastasis	COLEC11	0	5.278582	*
2784	Neoplasm Metastasis	SPP1	0	5.857485	*
2785	Neoplasm Metastasis	C10orf10	0.000003	7.305578	*
2786	Neoplasm Metastasis	HP	0.000009	21.852969	*
2787	Neoplasm Metastasis	CRP	0.000002	25.311911	*
2788	Normal colon	FXYD5	0.004697	-64.324786	*
2789	Normal colon	CDH3	0	-33.651977	*
2790	Normal colon	HS6ST2	0.004518	-24.301075	*
2791	Normal colon	PITX1	0.004952	-22.45	*
2792	Normal colon	SLC3A2	0.004792	-14.29764	*
2793	Normal colon	SLC39A6	0.006116	-11.756536	*
2794	Normal colon	LDLRAD3	0.00613	-11.487923	*
2795	Normal colon	GRB10	0.003715	-10.421296	*
2796	Normal colon	ACCN2	0.004094	-10.236928	*
2797	Normal colon	ASNS	0.005077	-9.20214	*
2798	Normal colon	FOXQ1	0	-8.865371	*

2799	Normal colon	KIAA1199	0	-8.794741	*
2800	Normal colon	CD81	0.00466	-8.248565	*
2801	Normal colon	TESC	0	-7.681462	*
2802	Normal colon	GRPEL2	0.00466	-7.668546	*
2803	Normal colon	CLDN1	0	-7.38952	*
2804	Normal colon	DPP7	0.006116	-6.682502	*
2805	Normal colon	C20orf20	0	-6.620339	*
2806	Normal colon	ETV4	0	-6.46072	*
2807	Normal colon	YARS	0.00274	-6.348424	*
2808	Normal colon	HSPA13	0.005681	-6.341991	*
2809	Normal colon	CDK4	0.004518	-6.24989	*
2810	Normal colon	ASCL2	0	-6.206657	*
2811	Normal colon	TMEM185B	0.003715	-5.837496	*
2812	Normal colon	TSPYL2	0.00274	-5.747126	*
2813	Normal colon	CIRH1A	0.00274	-5.542027	*
2814	Normal colon	FKBP10	0	-5.486196	*
2815	Normal colon	TEX10	0	-5.289375	*
2816	Normal colon	AHCY	0.004518	-5.223647	*
2817	Normal colon	WDR74	0.004146	-5.075321	*
2818	Normal colon	COL1A1	0	-4.999549	*
2819	Normal colon	TRIB3	0	-4.969046	*
2820	Normal colon	G6PC3	0.00274	-4.886922	*
2821	Normal colon	XPO5	0.00184	-4.886191	*
2822	Normal colon	DTD1	0.005681	-4.857716	*
2823	Normal colon	LDHB	0.002827	-4.81117	*
2824	Normal colon	FTSJ1	0.004078	-4.767414	*
2825	Normal colon	NOC3L	0.005681	-4.69697	*
2826	Normal colon	CLDN2	0	-4.68898	*
2827	Normal colon	NKD2	0	-4.664191	*
2828	Normal colon	TOMM34	0.004391	-4.503749	*
2829	Normal colon	NONO	0.002827	-4.4933	*
2830	Normal colon	COQ10A	0.00274	-4.468316	*
2831	Normal colon	MORN4	0.004078	-4.44856	*
2832	Normal colon	RNF43	0	-4.365724	*
2833	Normal colon	SOX4	0	-4.357218	*
2834	Normal colon	KLC2	0.005798	-4.347443	*
2835	Normal colon	ZNF473	0.000543	-4.320616	*
2836	Normal colon	SERPINB5	0	-4.320446	*
2837	Normal colon	OGFOD1	0.002827	-4.274142	*
2838	Normal colon	GPN3	0.005925	-4.245142	*
2839	Normal colon	NSUN5	0.00274	-4.223239	*
2840	Normal colon	NT5C3L	0.004078	-4.209745	*
2841	Normal colon	EFTUD2	0.004518	-4.19284	*
2842	Normal colon	SMYD5	0.005204	-4.192783	*
2843	Normal colon	IPO5	0.000543	-4.148212	*
2844	Normal colon	UNC119B	0.005142	-4.129353	*
2845	Normal colon	GART	0.00466	-4.111965	*
2846	Normal colon	TXNDC5	0.003331	-4.108174	*
2847	Normal colon	PHLDA1	0	-4.083523	*
2848	Normal colon	ESYT1	0.004518	-4.039931	*

2849	Normal colon	KLHL11	0.004146	-4.02168	*
2850	Normal colon	KIF20A	0	-4.011787	*
2851	Normal colon	ENC1	0	-4.009264	*
2852	Normal colon	POLD2	0.005681	-3.983102	*
2853	Normal colon	NMT1	0.005681	-3.920484	*
2854	Normal colon	PHF16	0.00274	-3.915459	*
2855	Normal colon	TCF7	0.004146	-3.880342	*
2856	Normal colon	RUVBL1	0.005681	-3.875942	*
2857	Normal colon	PREB	0.004956	-3.832184	*
2858	Normal colon	VARS	0.00184	-3.789916	*
2859	Normal colon	LUZP6	0.004518	-3.782718	*
2860	Normal colon	MMP11	0	-3.765944	*
2861	Normal colon	PUS7	0	-3.759485	*
2862	Normal colon	RAN	0.006116	-3.745088	*
2863	Normal colon	ARMC6	0.004518	-3.744797	*
2864	Normal colon	CSDA	0.005077	-3.70852	*
2865	Normal colon	USP5	0.003715	-3.700412	*
2866	Normal colon	G6PD	0.00274	-3.692506	*
2867	Normal colon	SLC7A5	0	-3.690876	*
2868	Normal colon	PRMT5	0.003998	-3.657105	*
2869	Normal colon	TRIM28	0.004518	-3.638133	*
2870	Normal colon	SOX9	0	-3.633093	*
2871	Normal colon	HIAT1	0.005479	-3.620712	*
2872	Normal colon	DPF2	0.005681	-3.620165	*
2873	Normal colon	SF3B3	0.005501	-3.613169	*
2874	Normal colon	ENO1	0.00274	-3.587509	*
2875	Normal colon	SYNGAP1	0.0052	-3.554779	*
2876	Normal colon	NEU3	0.005925	-3.553583	*
2877	Normal colon	TGFBI	0	-3.529137	*
2878	Normal colon	NAT10	0.004518	-3.51483	*
2879	Normal colon	SLCO4A1	0	-3.51321	*
2880	Normal colon	LASS2	0.00613	-3.511735	*
2881	Normal colon	TIMP1	0	-3.500653	*
2882	Normal colon	EZH2	0.005681	-3.483052	*
2883	Normal colon	PPP2CA	0.005558	-3.478573	*
2884	Normal colon	STC2	0	-3.424821	*
2885	Normal colon	KPNB1	0.004792	-3.418362	*
2886	Normal colon	NOP14	0.00466	-3.39778	*
2887	Normal colon	PA2G4	0.00274	-3.385057	*
2888	Normal colon	RPN2	0.00274	-3.377154	*
2889	Normal colon	HSPH1	0	-3.366472	*
2890	Normal colon	RCC2	0.005681	-3.350207	*
2891	Normal colon	NOC4L	0.003715	-3.340548	*
2892	Normal colon	FIBP	0.002702	-3.321244	*
2893	Normal colon	GMPS	0.004518	-3.311997	*
2894	Normal colon	SRPR	0.005681	-3.296962	*
2895	Normal colon	SSRP1	0.00466	-3.296498	*
2896	Normal colon	RUVBL2	0.006111	-3.290995	*
2897	Normal colon	OSBPL10	0.004146	-3.287311	*
2898	Normal colon	KATNB1	0.005681	-3.285481	*

2899	Normal colon	GANAB	0.00274	-3.283045	*
2900	Normal colon	COPS7A	0.00274	-3.259742	*
2901	Normal colon	AACS	0.005142	-3.239776	*
2902	Normal colon	RPUSD4	0.00274	-3.207434	*
2903	Normal colon	NANP	0.004254	-3.185112	*
2904	Normal colon	CPSF4	0.004078	-3.175705	*
2905	Normal colon	PHF20	0.006116	-3.164103	*
2906	Normal colon	PWP2	0.004792	-3.149072	*
2907	Normal colon	NOP56	0.00613	-3.148926	*
2908	Normal colon	ZRANB1	0.005681	-3.146275	*
2909	Normal colon	PPM1H	0	-3.143884	*
2910	Normal colon	DDX50	0.002827	-3.14262	*
2911	Normal colon	BUB3	0.004182	-3.142091	*
2912	Normal colon	RHOT1	0.005681	-3.134272	*
2913	Normal colon	TNFRSF12A	0	-3.118433	*
2914	Normal colon	PSMF1	0.005681	-3.094299	*
2915	Normal colon	DDB1	0.004146	-3.057781	*
2916	Normal colon	M6PR	0.005681	-3.035629	*
2917	Normal colon	EXTL2	0.004146	-3.033154	*
2918	Normal colon	GNS	0.004518	-3.015521	*
2919	Normal colon	SLC25A39	0.002827	-3.001813	*
2920	Normal colon	STT3A	0.00531	-2.993854	*
2921	Normal colon	PAFAH1B2	0.00466	-2.988591	*
2922	Normal colon	TMEM39B	0.004518	-2.961722	*
2923	Normal colon	NUMBL	0.004221	-2.960859	*
2924	Normal colon	FAM134C	0.005681	-2.947311	*
2925	Normal colon	ALG3	0.005977	-2.939251	*
2926	Normal colon	VPS4A	0.005681	-2.938373	*
2927	Normal colon	EDEM2	0.00581	-2.927928	*
2928	Normal colon	TRAPPC4	0.004101	-2.913919	*
2929	Normal colon	MRPL37	0.003331	-2.913286	*
2930	Normal colon	PDIA6	0.004518	-2.90568	*
2931	Normal colon	DCPS	0.005681	-2.902001	*
2932	Normal colon	AP3M1	0.004518	-2.900102	*
2933	Normal colon	TMEM43	0.005925	-2.829317	*
2934	Normal colon	TMEM222	0.004146	-2.815126	*
2935	Normal colon	RNH1	0.004792	-2.804233	*
2936	Normal colon	ZW10	0.004351	-2.787995	*
2937	Normal colon	MEN1	0.00539	-2.787758	*
2938	Normal colon	DDX54	0.003115	-2.767251	*
2939	Normal colon	MLF2	0.000543	-2.764901	*
2940	Normal colon	FAM136A	0.005777	-2.752235	*
2941	Normal colon	TFAP4	0.005681	-2.735507	*
2942	Normal colon	ZNF74	0.004146	-2.735369	*
2943	Normal colon	ADSL	0.005568	-2.705135	*
2944	Normal colon	POLDIP2	0.003715	-2.671356	*
2945	Normal colon	SLC25A15	0.005681	-2.654579	*
2946	Normal colon	WSB2	0.004391	-2.622514	*
2947	Normal colon	DIABLO	0.00466	-2.621987	*
2948	Normal colon	BAT1	0.004146	-2.612366	*

2949	Normal colon	GSK3A	0.00274	-2.598728	*
2950	Normal colon	CSE1L	0	-2.585994	*
2951	Normal colon	SHMT2	0	-2.581007	*
2952	Normal colon	SNRNP48	0.004094	-2.563389	*
2953	Normal colon	DHX30	0.003998	-2.555366	*
2954	Normal colon	MYC	0	-2.554701	*
2955	Normal colon	GTF3A	0	-2.548275	*
2956	Normal colon	INTS5	0.00466	-2.532124	*
2957	Normal colon	AP2M1	0.004792	-2.519507	*
2958	Normal colon	UBQLN1	0.00466	-2.508627	*
2959	Normal colon	DDX55	0.00466	-2.49934	*
2960	Normal colon	LIPE	0.006116	-2.478632	*
2961	Normal colon	DHX57	0.004792	-2.473856	*
2962	Normal colon	TMEM99	0.004518	-2.45	*
2963	Normal colon	BOP1	0	-2.427855	*
2964	Normal colon	GTF2IRD1	0	-2.424836	*
2965	Normal colon	CASC3	0.005681	-2.403027	*
2966	Normal colon	HIG2	0	-2.401973	*
2967	Normal colon	VDAC3	0.004518	-2.387827	*
2968	Normal colon	SEH1L	0.004518	-2.360705	*
2969	Normal colon	MED20	0.005681	-2.355722	*
2970	Normal colon	DVL2	0.004792	-2.341063	*
2971	Normal colon	ODF2	0.005925	-2.309888	*
2972	Normal colon	TEAD4	0	-2.27404	*
2973	Normal colon	AARSD1	0.00613	-2.270759	*
2974	Normal colon	RPS19BP1	0.003331	-2.26466	*
2975	Normal colon	RHEBL1	0.005681	-2.261905	*
2976	Normal colon	TOP1MT	0	-2.231585	*
2977	Normal colon	JAG2	0	-2.227974	*
2978	Normal colon	SLC12A2	0	-2.217707	*
2979	Normal colon	TGIF2	0	-2.200708	*
2980	Normal colon	SIM2	0	-2.181533	*
2981	Normal colon	SNTB1	0	-2.173823	*
2982	Normal colon	DKC1	0	-2.17118	*
2983	Normal colon	ZNF526	0.005925	-2.162342	*
2984	Normal colon	REXO2	0.005681	-2.160419	*
2985	Normal colon	TPD52L2	0.000076	-2.120266	*
2986	Normal colon	FANCE	0.005681	-2.118416	*
2987	Normal colon	MTHFD2	0	-2.072072	*
2988	Normal colon	TGIF1	0	-2.062134	*
2989	Normal colon	LOC388796	0	-2.0231	*
2990	Normal colon	CBX2	0	-2.016764	*
2991	Normal colon	BDH1	0.00027	2.003198	*
2992	Normal colon	TP53I3	0.000116	2.067676	*
2993	Normal colon	MGC4172	0	2.094416	*
2994	Normal colon	CPT2	0	2.12269	*
2995	Normal colon	PAQR8	0.000098	2.124901	*
2996	Normal colon	MXD1	0.000104	2.154847	*
2997	Normal colon	OSBPL7	0	2.218376	*
2998	Normal colon	IRF4	0	2.266363	*

2999	Normal colon	CLEC3B	0	2.369313	*
3000	Normal colon	TST	0	2.41631	*
3001	Normal colon	PLCE1	0	2.444334	*
3002	Normal colon	GPD1L	0	2.445453	*
3003	Normal colon	TMPRSS2	0	2.447403	*
3004	Normal colon	CES2	0	2.491005	*
3005	Normal colon	SQRDL	0	2.493801	*
3006	Normal colon	C10orf99	0	2.501012	*
3007	Normal colon	CLIC5	0	2.517516	*
3008	Normal colon	C1orf115	0	2.557922	*
3009	Normal colon	CPM	0.000051	2.621781	*
3010	Normal colon	LOH11CR2A	0	2.644305	*
3011	Normal colon	BMP2	0	2.656796	*
3012	Normal colon	C4orf34	0.000284	2.70772	*
3013	Normal colon	MAOA	0	2.729773	*
3014	Normal colon	PAPSS2	0	2.847457	*
3015	Normal colon	C15orf48	0	2.86025	*
3016	Normal colon	LDHD	0	2.885824	*
3017	Normal colon	SULT1A1	0.000206	2.925544	*
3018	Normal colon	CAPN5	0	2.957012	*
3019	Normal colon	TCEA3	0	3.023788	*
3020	Normal colon	CCDC68	0.000028	3.028445	*
3021	Normal colon	CEACAM1	0.000043	3.080438	*
3022	Normal colon	TMEM54	0	3.098006	*
3023	Normal colon	KIAA0828	0	3.099127	*
3024	Normal colon	SCGN	0.000292	3.132719	*
3025	Normal colon	PADI2	0	3.380171	*
3026	Normal colon	TSPAN1	0	3.412649	*
3027	Normal colon	SLC26A2	0	3.424724	*
3028	Normal colon	MALL	0	3.451365	*
3029	Normal colon	GPT	0.000045	3.535019	*
3030	Normal colon	SLC9A2	0	3.718974	*
3031	Normal colon	TUBAL3	0	3.723097	*
3032	Normal colon	SCARA5	0	3.7548	*
3033	Normal colon	GCNT3	0	3.833073	*
3034	Normal colon	MYO1A	0	3.964463	*
3035	Normal colon	EPB41L3	0	4.068592	*
3036	Normal colon	SDCBP2	0	4.330043	*
3037	Normal colon	GPA33	0	4.331913	*
3038	Normal colon	MEP1A	0	4.336707	*
3039	Normal colon	FXYD3	0	4.380328	*
3040	Normal colon	HHLA2	0	4.409889	*
3041	Normal colon	SLC16A9	0.00274	4.44136	*
3042	Normal colon	TSPAN7	0	4.469347	*
3043	Normal colon	SLC4A4	0.000152	4.563736	*
3044	Normal colon	MAPK10	0.010117	4.758095	*
3045	Normal colon	PLAC8	0	5.024936	*
3046	Normal colon	CA12	0	5.0407	*
3047	Normal colon	METTL7A	0	5.071267	*
3048	Normal colon	ADH1B	0	5.287184	*

3049	Normal colon	HSD11B2	0	5.500039	*
3050	Normal colon	UGT2B17	0	5.587305	*
3051	Normal colon	CHP2	0	5.687153	*
3052	Normal colon	VSIG2	0	5.798946	*
3053	Normal colon	IGJ	0	6.442851	*
3054	Normal colon	AKAP5	0.000543	7.672727	*
3055	Normal colon	HSD17B2	0	8.104694	*
3056	Normal colon	SLC26A3	0	9.094399	*
3057	Normal colon	CEACAM7	0	9.432207	*
3058	Normal colon	ZG16	0	12.084817	*
3059	Normal colon	FCGBP	0	12.734993	*
3060	Normal colon	CA2	0	14.04996	*
3061	Normal colon	GUCA2A	0	16.142228	*
3062	Normal colon	CA4	0	16.245571	*
3063	Normal colon	BCAS1	0.00466	17.453571	*
3064	Normal colon	CA1	0	19.690103	*
3065	Normal colon	FAM55D	0	23.508689	*
3066	Normal colon	HPGD	0	23.718251	*
3067	Normal tissue morphology	HSD17B2	0	-7.781235	*
3068	Normal tissue morphology	PYY	0	-7.20626	*
3069	Normal tissue morphology	PKIB	0	-6.760776	*
3070	Normal tissue morphology	MGC13057	0	-6.316289	*
3071	Normal tissue morphology	CLEC3B	0	-5.526861	*
3072	Normal tissue morphology	SCNN1B	0	-5.373861	*
3073	Normal tissue morphology	HPGD	0	-5.351308	*
3074	Normal tissue morphology	ADAMDEC1	0	-5.194066	*
3075	Normal tissue morphology	FLJ21511	0	-4.982366	*
3076	Normal tissue morphology	KLF4	0	-4.841207	*
3077	Normal tissue morphology	CLDN23	0	-4.827579	*
3078	Normal tissue morphology	SRPX	0	-4.822471	*
3079	Normal tissue morphology	MGC4172	0	-4.678043	*
3080	Normal tissue morphology	GPT	0	-4.443061	*
3081	Normal tissue morphology	SCIN	0	-4.434475	*
3082	Normal tissue morphology	CA7	0	-4.360658	*
3083	Normal tissue morphology	TSPAN7	0	-4.352056	*
3084	Normal tissue morphology	ADH1B	0	-4.346492	*
3085	Normal tissue morphology	MYOT	0	-4.164428	*
3086	Normal tissue morphology	NR3C2	0	-4.11659	*
3087	Normal tissue morphology	EDN3	0	-3.87703	*
3088	Normal tissue morphology	FAM107A	0	-3.744748	*
3089	Normal tissue morphology	SCGN	0	-3.561313	*
3090	Normal tissue morphology	LDHD	0	-3.492451	*
3091	Normal tissue morphology	UGDH	0	-3.083284	*
3092	Normal tissue morphology	KRT24	0	-2.892109	*
3093	Normal tissue morphology	TEAD4	0	-2.851295	*
3094	Normal tissue morphology	ACADS	0	-2.83351	*
3095	Normal tissue morphology	ENC1	0	-2.780937	*
3096	Normal tissue morphology	ENTPD5	0	-2.764317	*
3097	Normal tissue morphology	NR5A2	0	-2.707146	*
3098	Normal tissue morphology	S100A11	0	-2.650915	*

3099	Normal tissue morphology	PLCE1	0	-2.627492	*
3100	Normal tissue morphology	RetSat	0	-2.572066	*
3101	Normal tissue morphology	RDH5	0	-2.567677	*
3102	Normal tissue morphology	SRI	0	-2.528823	*
3103	Normal tissue morphology	HHLA2	0	-2.489899	*
3104	Normal tissue morphology	NEDD4L	0	-2.466577	*
3105	Normal tissue morphology	MTHFD2	0	-2.406167	*
3106	Normal tissue morphology	SPIB	0	-2.38534	*
3107	Normal tissue morphology	GNA11	0	-2.359015	*
3108	Normal tissue morphology	UGP2	0	-2.354849	*
3109	Normal tissue morphology	GCNT2	0	-2.239828	*
3110	Normal tissue morphology	ETFDH	0	-2.226034	*
3111	Normal tissue morphology	SHMT2	0	-2.205136	*
3112	Normal tissue morphology	BRP44L	0	-2.200729	*
3113	Normal tissue morphology	DHRS9	0	-2.198375	*
3114	Normal tissue morphology	CPT2	0	-2.196555	*
3115	Normal tissue morphology	PLCD3	0	-2.104867	*
3116	Normal tissue morphology	GLTP	0	-2.101629	*
3117	Normal tissue morphology	ZZEF1	0	-2.045019	*
3118	Normal tissue morphology	MORC4	0	-2.012692	*
3119	Normal tissue morphology	BCAR3	0.000012	2.114651	*
3120	Normal tissue morphology	PRDX6	0	2.131065	*
3121	Normal tissue morphology	SLC1A7	0.000001	2.224365	*
3122	Normal tissue morphology	C20orf20	0	2.373628	*
3123	Normal tissue morphology	STAP2	0	2.586791	*
3124	Normal tissue morphology	CLMN	0.000006	2.591893	*
3125	Normal tissue morphology	SQRDL	0.000007	2.686297	*
3126	Normal tissue morphology	GPD1L	0.000012	2.724242	*
3127	Normal tissue morphology	RIOK3	0	2.792375	*
3128	Normal tissue morphology	MTM1	0.000012	2.817681	*
3129	Normal tissue morphology	VILL	0.000003	2.882666	*
3130	Normal tissue morphology	MYO1A	0.000007	2.962363	*
3131	Normal tissue morphology	FABP1	0.000006	3.032035	*
3132	Normal tissue morphology	IL1R2	0.000004	3.085178	*
3133	Normal tissue morphology	TST	0.000001	3.086522	*
3134	Normal tissue morphology	PAPSS2	0.000007	3.090265	*
3135	Normal tissue morphology	MGC14376	0	3.22169	*
3136	Normal tissue morphology	PPAP2A	0	3.324126	*
3137	Normal tissue morphology	GPA33	0.000001	3.429682	*
3138	Normal tissue morphology	ETV4	0	3.474337	*
3139	Normal tissue morphology	ABHD3	0.000008	3.47626	*
3140	Normal tissue morphology	DSC2	0.000004	3.593929	*
3141	Normal tissue morphology	ETHE1	0.000001	3.835435	*
3142	Normal tissue morphology	STMN2	0	3.987021	*
3143	Normal tissue morphology	FXYD3	0	4.060949	*
3144	Normal tissue morphology	PLAC8	0.000012	4.1363	*
3145	Normal tissue morphology	CES2	0.000001	4.217799	*
3146	Normal tissue morphology	FLJ32063	0.00001	4.349534	*
3147	Normal tissue morphology	TSPAN1	0.000001	4.421269	*
3148	Normal tissue morphology	HIGD1A	0	4.470799	*

3149	Normal tissue morphology	AKR1B10	0.000004	4.757943	*
3150	Normal tissue morphology	SDCBP2	0.000001	5.00517	*
3151	Normal tissue morphology	LRRC19	0.000007	5.393298	*
3152	Normal tissue morphology	CDH3	0	5.7184	*
3153	Normal tissue morphology	KRT20	0.00001	7.551308	*
3154	Normal tissue morphology	SLC4A4	0.000001	9.124767	*
3155	Normal tissue morphology	CA2	0.000006	9.288727	*
3156	Normal tissue morphology	CLDN1	0	10.740069	*
3157	Primary Neoplasm	CA2	0	-74.544734	*
3158	Primary Neoplasm	ABCA8	0	-4.810661	*
3159	Primary Neoplasm	PLG	0.000007	-3.798711	*
3160	Primary Neoplasm	CTSK	0	2.261573	*
3161	Primary Neoplasm	IGHG1	0	2.321187	*
3162	Primary Neoplasm	PDPN	0	2.501963	*
3163	Primary Neoplasm	COL1A2	0	2.549568	*
3164	Primary Neoplasm	SULF1	0	2.936796	*
3165	Primary Neoplasm	COL11A1	0	4.702894	*
3166	Protein p53	NLRP2	0	-110.051078	*
3167	Protein p53	OGFRL1	0.000003	-12.864371	*
3168	Protein p53	SEPP1	0	-12.288043	*
3169	Protein p53	TUBB6	0.000001	-12.188465	*
3170	Protein p53	FAM92A1	0.000008	-11.867889	*
3171	Protein p53	GAMT	0	-10.974298	*
3172	Protein p53	GJA3	0.000018	-10.47161	*
3173	Protein p53	ID2	0.000001	-10.221989	*
3174	Protein p53	PLAGL1	0.000041	-10.015007	*
3175	Protein p53	OSBPL6	0	-9.871076	*
3176	Protein p53	MOCS1	0	-9.33562	*
3177	Protein p53	ZC3H12C	0.000033	-8.930023	*
3178	Protein p53	MFAP3L	0.000033	-6.479532	*
3179	Protein p53	C1orf115	0.00001	-5.876075	*
3180	Protein p53	FRMD6	0.000002	-5.304811	*
3181	Protein p53	PDGFRL	0.000039	-4.789713	*
3182	Protein p53	RNF144A	0.000002	-4.736778	*
3183	Protein p53	GPR137B	0.000096	-4.529965	*
3184	Protein p53	AIF1L	0.000064	-4.187511	*
3185	Protein p53	PSTPIP2	0.000008	-4.10078	*
3186	Protein p53	FZD2	0	-4.030252	*
3187	Protein p53	ZDHHC2	0.000001	-3.993547	*
3188	Protein p53	SMAD4	0.000049	-3.987658	*
3189	Protein p53	TMEM98	0	-3.881347	*
3190	Protein p53	SLC26A11	0.000007	-3.832825	*
3191	Protein p53	JAG2	0.000111	-3.78717	*
3192	Protein p53	APBB2	0.000002	-3.772794	*
3193	Protein p53	SLC25A37	0.000003	-3.556737	*
3194	Protein p53	PTGES	0	-3.474348	*
3195	Protein p53	ST6GALNAC2	0	-3.444906	*
3196	Protein p53	BTG3	0.000026	-3.443329	*
3197	Protein p53	CLU	0.000081	-3.269041	*
3198	Protein p53	CST6	0.000119	-3.246943	*

3199	Protein p53	SLC39A6	0	-3.146657	*
3200	Protein p53	PTRF	0.000032	-3.038161	*
3201	Protein p53	CTSL1	0.000013	-3.010972	*
3202	Protein p53	CIRBP	0.000044	-2.97714	*
3203	Protein p53	NAB2	0.000002	-2.897102	*
3204	Protein p53	SGSM1	0	-2.890026	*
3205	Protein p53	C21orf91	0	-2.609282	*
3206	Protein p53	GABBR1	0.000003	-2.587627	*
3207	Protein p53	UBXN8	0	-2.509628	*
3208	Protein p53	STYX	0.000003	-2.447138	*
3209	Protein p53	RPRD1A	0.000009	-2.390988	*
3210	Protein p53	MYO5A	0	-2.367748	*
3211	Protein p53	KANK2	0.000005	-2.367209	*
3212	Protein p53	HEXIM1	0.000003	-2.358521	*
3213	Protein p53	TWSG1	0.000017	-2.305563	*
3214	Protein p53	CENPC1	0.000015	-2.302085	*
3215	Protein p53	RNF138	0.000051	-2.239473	*
3216	Protein p53	PIAS2	0.000005	-2.235642	*
3217	Protein p53	NKX3-1	0.000018	-2.119512	*
3218	Protein p53	C17orf68	0.000008	-2.107659	*
3219	Protein p53	PTPRG	0.000062	-2.084443	*
3220	Protein p53	SMAD2	0.000023	-2.082566	*
3221	Protein p53	SFRS18	0	-2.071387	*
3222	Protein p53	HMG20B	0.000004	-2.058405	*
3223	Protein p53	CCDC69	0	-2.051861	*
3224	Protein p53	TRIM23	0	-2.043023	*
3225	Protein p53	HNF4A	0.000009	2.034895	*
3226	Protein p53	ZNF630	0.000001	2.331547	*
3227	Protein p53	PLS1	0.00001	2.369724	*
3228	Protein p53	ACP6	0	2.543673	*
3229	Protein p53	SLC2A1	0.000044	2.562317	*
3230	Protein p53	GOLT1A	0.000024	2.695651	*
3231	Protein p53	ZFP36L2	0.000004	2.797318	*
3232	Protein p53	EEPD1	0.000002	2.850552	*
3233	Protein p53	NT5C3	0.000002	2.927017	*
3234	Protein p53	FZD5	0.000021	2.968789	*
3235	Protein p53	ANXA4	0.000014	3.10173	*
3236	Protein p53	NET1	0	3.263517	*
3237	Protein p53	KCNE3	0.000001	3.944067	*
3238	Protein p53	ABHD2	0.000006	3.99146	*
3239	Protein p53	TAGLN2	0	4.272309	*
3240	Protein p53	STON2	0.000011	4.358066	*
3241	Protein p53	CTSC	0	4.484061	*
3242	Protein p53	SMAD6	0.000001	4.666987	*
3243	Protein p53	SPNS2	0.000025	4.766598	*
3244	Protein p53	H2AFJ	0	5.259377	*
3245	Protein p53	CEACAM1	0.000008	5.954795	*
3246	Protein p53	GNAS	0.000106	6.351093	*
3247	Protein p53	TMEM139	0.000003	6.3594	*
3248	Protein p53	MAML2	0.000002	7.453422	*

3249	Protein p53	MAP2K6	0	8.252522	*
3250	Protein p53	HGD	0.000002	8.781096	*
3251	Protein p53	TMPRSS2	0	9.451476	*
3252	Protein p53	CD24	0.000002	10.314369	*
3253	Protein p53	HIST1H2BG	0.000042	10.401582	*
3254	Protein p53	LRIG1	0	10.409232	*
3255	Protein p53	MUC13	0.000058	11.700251	*
3256	Protein p53	PMEPA1	0.000003	12.174786	*
3257	Protein p53	HKDC1	0.000002	14.892166	*
3258	Protein p53	RASSF6	0.000001	16.482922	*
3259	Protein p53	SLC22A3	0.00001	16.487081	*
3260	Protein p53	ITGB6	0.000001	16.820715	*
3261	Protein p53	KIAA1199	0	17.561393	*
3262	Protein p53	HNMT	0.000005	21.776943	*
3263	Protein p53	LGR5	0.000002	36.312211	*
3264	Protein p53	AKR1C3	0.000007	41.255223	*
3265	Protein p53	TGFBI	0	44.172228	*
3266	Protein p53	AMIGO2	0.000001	44.444089	*
3267	Protein p53	TOX3	0.000054	56.49921	*
3268	Protein p53	LY75	0	63.005992	*
3269	Protein p53	CLRN3	0	63.568249	*
3270	Protein p53	S100P	0	164.471276	*
3271	Protein p53	GPX2	0	174.729443	*
3272	Protein p53	POF1B	0	256.130843	*
3273	Secondary malignant neoplasm of liver	ZG16	0	-31.956839	*
3274	Secondary malignant neoplasm of liver	CLCA4	0	-26.707483	*
3275	Secondary malignant neoplasm of liver	CA4	0	-15.845909	*
3276	Secondary malignant neoplasm of liver	ACTG2	0	-13.707272	*
3277	Secondary malignant neoplasm of liver	CLCA1	0	-13.405836	*
3278	Secondary malignant neoplasm of liver	CEACAM7	0	-13.361052	*
3279	Secondary malignant neoplasm of liver	SPINK4	0	-12.488802	*
3280	Secondary malignant neoplasm of liver	FCGBP	0	-11.391719	*
3281	Secondary malignant neoplasm of liver	CA1	0	-10.844196	*
3282	Secondary malignant neoplasm of liver	VSIG2	0	-9.837015	*
3283	Secondary malignant neoplasm of liver	VIP	0	-8.944965	*
3284	Secondary malignant neoplasm of liver	IGJ	0	-8.072572	*
3285	Secondary malignant neoplasm of liver	MAB21L2	0	-7.859847	*
3286	Secondary malignant neoplasm of liver	FAM55D	0	-7.716639	*
3287	Secondary malignant neoplasm of liver	MMP3	0	-7.549345	*
3288	Secondary malignant neoplasm of liver	AQP8	0	-7.15219	*
3289	Secondary malignant neoplasm of liver	MS4A12	0	-6.806925	*
3290	Secondary malignant neoplasm of liver	CA2	0	-6.679325	*
3291	Secondary malignant neoplasm of liver	FBLN1	0	-5.683315	*
3292	Secondary malignant neoplasm of liver	ADAMDEC1	0	-5.236325	*
3293	Secondary malignant neoplasm of liver	VWF	0	-4.529824	*
3294	Secondary malignant neoplasm of liver	C6ORF105	0	-4.303634	*
3295	Secondary malignant neoplasm of liver	SPARCL1	0	-4.05342	*
3296	Secondary malignant neoplasm of liver	TPSG1	0	-3.941812	*
3297	Secondary malignant neoplasm of liver	MYH11	0	-3.895139	*
3298	Secondary malignant neoplasm of liver	IL1R2	0	-3.846801	*

3299	Secondary malignant neoplasm of liver	TCF21	0	-3.700641	*
3300	Secondary malignant neoplasm of liver	PIGR	0	-3.622438	*
3301	Secondary malignant neoplasm of liver	TSPAN1	0.000001	-3.277753	*
3302	Secondary malignant neoplasm of liver	PYY	0	-3.042799	*
3303	Secondary malignant neoplasm of liver	CD79A	0	-2.942539	*
3304	Secondary malignant neoplasm of liver	ITM2C	0.000001	-2.872164	*
3305	Secondary malignant neoplasm of liver	BCAS1	0	-2.845971	*
3306	Secondary malignant neoplasm of liver	PLAC8	0	-2.810572	*
3307	Secondary malignant neoplasm of liver	FOXF1	0	-2.736251	*
3308	Secondary malignant neoplasm of liver	CPA3	0	-2.60624	*
3309	Secondary malignant neoplasm of liver	SDCBP2	0.000331	-2.495947	*
3310	Secondary malignant neoplasm of liver	DIO2	0	-2.431205	*
3311	Secondary malignant neoplasm of liver	DDR2	0	-2.222107	*
3312	Secondary malignant neoplasm of liver	LGALS4	0.000004	-2.055385	*
3313	Secondary malignant neoplasm of liver	COLEC11	0	5.278582	*
3314	Secondary malignant neoplasm of liver	HP	0.000009	21.852969	*
3315	Secondary malignant neoplasm of liver	CRP	0.000002	25.311911	*
3316	Transcriptional Coactivator with PDZ-Binding Mo	WWTR1	0	-14.386729	*
3317	Transcriptional Coactivator with PDZ-Binding Mo	TSPAN6	0	-11.611641	*
3318	Transcriptional Coactivator with PDZ-Binding Mo	HMGA2	0	-10.672552	*
3319	Transcriptional Coactivator with PDZ-Binding Mo	DDAH1	0	-7.025009	*
3320	Transcriptional Coactivator with PDZ-Binding Mo	BRP44	0.000004	-6.554565	*
3321	Transcriptional Coactivator with PDZ-Binding Mo	SH3D19	0	-5.241574	*
3322	Transcriptional Coactivator with PDZ-Binding Mo	CD109	0	-5.112988	*
3323	Transcriptional Coactivator with PDZ-Binding Mo	MAT2B	0	-4.798222	*
3324	Transcriptional Coactivator with PDZ-Binding Mo	RAB8B	0	-4.642816	*
3325	Transcriptional Coactivator with PDZ-Binding Mo	SLC7A5	0	-4.640135	*
3326	Transcriptional Coactivator with PDZ-Binding Mo	MED14	0	-4.589488	*
3327	Transcriptional Coactivator with PDZ-Binding Mo	ITGAV	0	-4.570969	*
3328	Transcriptional Coactivator with PDZ-Binding Mo	FAM96A	0	-4.257481	*
3329	Transcriptional Coactivator with PDZ-Binding Mo	E2F8	0.000001	-4.16024	*
3330	Transcriptional Coactivator with PDZ-Binding Mo	C9orf41	0.000001	-3.904127	*
3331	Transcriptional Coactivator with PDZ-Binding Mo	GLO1	0	-3.823781	*
3332	Transcriptional Coactivator with PDZ-Binding Mo	RSPRY1	0	-3.736446	*
3333	Transcriptional Coactivator with PDZ-Binding Mo	STMN3	0.000002	-3.611254	*
3334	Transcriptional Coactivator with PDZ-Binding Mo	PRC1	0	-3.506423	*
3335	Transcriptional Coactivator with PDZ-Binding Mo	USP38	0	-3.456152	*
3336	Transcriptional Coactivator with PDZ-Binding Mo	GPCPD1	0	-3.424358	*
3337	Transcriptional Coactivator with PDZ-Binding Mo	DCTN4	0	-3.41054	*
3338	Transcriptional Coactivator with PDZ-Binding Mo	UBE2E2	0	-3.258407	*
3339	Transcriptional Coactivator with PDZ-Binding Mo	IPO8	0.000003	-3.256525	*
3340	Transcriptional Coactivator with PDZ-Binding Mo	C9orf64	0.000001	-3.24901	*
3341	Transcriptional Coactivator with PDZ-Binding Mo	TMEM167A	0.000002	-3.247133	*
3342	Transcriptional Coactivator with PDZ-Binding Mo	SSR1	0	-3.169305	*
3343	Transcriptional Coactivator with PDZ-Binding Mo	TGFBR1	0	-3.165646	*
3344	Transcriptional Coactivator with PDZ-Binding Mo	RANBP9	0	-3.134713	*
3345	Transcriptional Coactivator with PDZ-Binding Mo	HPS3	0	-3.091556	*
3346	Transcriptional Coactivator with PDZ-Binding Mo	RBM18	0	-3.07375	*
3347	Transcriptional Coactivator with PDZ-Binding Mo	PDCD6	0	-3.007018	*
3348	Transcriptional Coactivator with PDZ-Binding Mo	PTP4A1	0	-2.977634	*

3349	Transcriptional Coactivator with PDZ-Binding Mo	LMNB1	0.000001	-2.974197	*
3350	Transcriptional Coactivator with PDZ-Binding Mo	TWSG1	0	-2.907945	*
3351	Transcriptional Coactivator with PDZ-Binding Mo	NECAP1	0	-2.904588	*
3352	Transcriptional Coactivator with PDZ-Binding Mo	TMEM182	0	-2.899559	*
3353	Transcriptional Coactivator with PDZ-Binding Mo	SERPINH1	0	-2.869568	*
3354	Transcriptional Coactivator with PDZ-Binding Mo	ADI1	0.000003	-2.861292	*
3355	Transcriptional Coactivator with PDZ-Binding Mo	SGMS2	0.000001	-2.857988	*
3356	Transcriptional Coactivator with PDZ-Binding Mo	ACBD5	0	-2.844812	*
3357	Transcriptional Coactivator with PDZ-Binding Mo	UBFD1	0	-2.813761	*
3358	Transcriptional Coactivator with PDZ-Binding Mo	FAM36A	0	-2.80889	*
3359	Transcriptional Coactivator with PDZ-Binding Mo	FBXO28	0.000002	-2.794325	*
3360	Transcriptional Coactivator with PDZ-Binding Mo	C17orf75	0	-2.770219	*
3361	Transcriptional Coactivator with PDZ-Binding Mo	UBASH3B	0	-2.768619	*
3362	Transcriptional Coactivator with PDZ-Binding Mo	MEST	0.000002	-2.694467	*
3363	Transcriptional Coactivator with PDZ-Binding Mo	C20orf24	0	-2.646649	*
3364	Transcriptional Coactivator with PDZ-Binding Mo	DDI2	0	-2.642066	*
3365	Transcriptional Coactivator with PDZ-Binding Mo	FASTKD1	0	-2.642066	*
3366	Transcriptional Coactivator with PDZ-Binding Mo	FBXO9	0	-2.614738	*
3367	Transcriptional Coactivator with PDZ-Binding Mo	DPY19L4	0	-2.592181	*
3368	Transcriptional Coactivator with PDZ-Binding Mo	SRI	0.000005	-2.575763	*
3369	Transcriptional Coactivator with PDZ-Binding Mo	GMCL1	0	-2.574276	*
3370	Transcriptional Coactivator with PDZ-Binding Mo	C12orf4	0.000002	-2.552068	*
3371	Transcriptional Coactivator with PDZ-Binding Mo	RBL2	0	-2.549121	*
3372	Transcriptional Coactivator with PDZ-Binding Mo	COMT	0.000002	-2.546178	*
3373	Transcriptional Coactivator with PDZ-Binding Mo	FAM117B	0	-2.544708	*
3374	Transcriptional Coactivator with PDZ-Binding Mo	EBAG9	0	-2.525671	*
3375	Transcriptional Coactivator with PDZ-Binding Mo	ACTR2	0.000001	-2.506776	*
3376	Transcriptional Coactivator with PDZ-Binding Mo	C1GALT1	0	-2.502436	*
3377	Transcriptional Coactivator with PDZ-Binding Mo	PURB	0	-2.486587	*
3378	Transcriptional Coactivator with PDZ-Binding Mo	MRPS2	0.000001	-2.485151	*
3379	Transcriptional Coactivator with PDZ-Binding Mo	ANP32E	0.000001	-2.483716	*
3380	Transcriptional Coactivator with PDZ-Binding Mo	CDH1	0	-2.479415	*
3381	Transcriptional Coactivator with PDZ-Binding Mo	GLS	0.000002	-2.429793	*
3382	Transcriptional Coactivator with PDZ-Binding Mo	RAP2C	0	-2.425586	*
3383	Transcriptional Coactivator with PDZ-Binding Mo	MAPKSP1	0	-2.419988	*
3384	Transcriptional Coactivator with PDZ-Binding Mo	MAPKAP1	0	-2.390811	*
3385	Transcriptional Coactivator with PDZ-Binding Mo	ANKFY1	0	-2.390811	*
3386	Transcriptional Coactivator with PDZ-Binding Mo	PRIM2	0.000003	-2.34567	*
3387	Transcriptional Coactivator with PDZ-Binding Mo	PRDX6	0	-2.324091	*
3388	Transcriptional Coactivator with PDZ-Binding Mo	ARIH2	0.000005	-2.320067	*
3389	Transcriptional Coactivator with PDZ-Binding Mo	AP3M1	0	-2.284165	*
3390	Transcriptional Coactivator with PDZ-Binding Mo	UPP1	0.000003	-2.284165	*
3391	Transcriptional Coactivator with PDZ-Binding Mo	DNAJC16	0.000003	-2.28021	*
3392	Transcriptional Coactivator with PDZ-Binding Mo	MPPE1	0	-2.277577	*
3393	Transcriptional Coactivator with PDZ-Binding Mo	WDFY1	0	-2.274948	*
3394	Transcriptional Coactivator with PDZ-Binding Mo	DYNC1LI1	0	-2.272321	*
3395	Transcriptional Coactivator with PDZ-Binding Mo	HIGD1A	0.000001	-2.265768	*
3396	Transcriptional Coactivator with PDZ-Binding Mo	KCMF1	0.000002	-2.263152	*
3397	Transcriptional Coactivator with PDZ-Binding Mo	SLC39A10	0	-2.246221	*
3398	Transcriptional Coactivator with PDZ-Binding Mo	TTC26	0	-2.222988	*

3399	Transcriptional Coactivator with PDZ-Binding Mo	ZNF148	0.000001	-2.220421	*
3400	Transcriptional Coactivator with PDZ-Binding Mo	ECHDC1	0	-2.207632	*
3401	Transcriptional Coactivator with PDZ-Binding Mo	AFF4	0	-2.19365	*
3402	Transcriptional Coactivator with PDZ-Binding Mo	C6orf162	0.000002	-2.188587	*
3403	Transcriptional Coactivator with PDZ-Binding Mo	UGGT1	0	-2.163449	*
3404	Transcriptional Coactivator with PDZ-Binding Mo	CMTM4	0	-2.155964	*
3405	Transcriptional Coactivator with PDZ-Binding Mo	TMEM30A	0.000002	-2.152232	*
3406	Transcriptional Coactivator with PDZ-Binding Mo	SYNCRIP	0	-2.126283	*
3407	Transcriptional Coactivator with PDZ-Binding Mo	SP3	0	-2.125055	*
3408	Transcriptional Coactivator with PDZ-Binding Mo	MAPRE1	0	-2.125055	*
3409	Transcriptional Coactivator with PDZ-Binding Mo	ZMAT3	0	-2.122601	*
3410	Transcriptional Coactivator with PDZ-Binding Mo	TIMM17A	0	-2.117703	*
3411	Transcriptional Coactivator with PDZ-Binding Mo	TAGLN2	0	-2.11648	*
3412	Transcriptional Coactivator with PDZ-Binding Mo	RCAN3	0.000004	-2.098221	*
3413	Transcriptional Coactivator with PDZ-Binding Mo	EPHB4	0	-2.084932	*
3414	Transcriptional Coactivator with PDZ-Binding Mo	SF3A1	0.000001	-2.083728	*
3415	Transcriptional Coactivator with PDZ-Binding Mo	MRS2	0.000001	-2.072923	*
3416	Transcriptional Coactivator with PDZ-Binding Mo	ANKRD29	0.000002	-2.072923	*
3417	Transcriptional Coactivator with PDZ-Binding Mo	CERK	0	-2.05504	*
3418	Transcriptional Coactivator with PDZ-Binding Mo	HSBP1	0.000005	-2.049114	*
3419	Transcriptional Coactivator with PDZ-Binding Mo	EARS2	0.000001	-2.04793	*
3420	Transcriptional Coactivator with PDZ-Binding Mo	MAEA	0.000001	-2.040845	*
3421	Transcriptional Coactivator with PDZ-Binding Mo	STK24	0	-2.037312	*
3422	Transcriptional Coactivator with PDZ-Binding Mo	NMD3	0.000002	-2.034959	*
3423	Transcriptional Coactivator with PDZ-Binding Mo	CXADR	0	-2.024408	*
3424	Transcriptional Coactivator with PDZ-Binding Mo	FBXO7	0.000002	-2.012748	*
3425	Transcriptional Coactivator with PDZ-Binding Mo	CACHD1	0	2.039667	*
3426	Transcriptional Coactivator with PDZ-Binding Mo	GRAMD3	0	2.057416	*
3427	Transcriptional Coactivator with PDZ-Binding Mo	IL8	0.000001	2.066945	*
3428	Transcriptional Coactivator with PDZ-Binding Mo	NRP1	0.000004	2.081322	*
3429	Transcriptional Coactivator with PDZ-Binding Mo	GPNMB	0.000004	2.083728	*
3430	Transcriptional Coactivator with PDZ-Binding Mo	CHST11	0	2.105506	*
3431	Transcriptional Coactivator with PDZ-Binding Mo	DNAJC18	0	2.141072	*
3432	Transcriptional Coactivator with PDZ-Binding Mo	KIAA0040	0	2.183537	*
3433	Transcriptional Coactivator with PDZ-Binding Mo	PPP2R1B	0.000003	2.194917	*
3434	Transcriptional Coactivator with PDZ-Binding Mo	DCP2	0	2.221704	*
3435	Transcriptional Coactivator with PDZ-Binding Mo	DOCK4	0	2.242332	*
3436	Transcriptional Coactivator with PDZ-Binding Mo	CLEC1A	0	2.285484	*
3437	Transcriptional Coactivator with PDZ-Binding Mo	C7orf41	0.000002	2.300052	*
3438	Transcriptional Coactivator with PDZ-Binding Mo	KIAA1324	0.000005	2.349738	*
3439	Transcriptional Coactivator with PDZ-Binding Mo	TTLL7	0.000001	2.353813	*
3440	Transcriptional Coactivator with PDZ-Binding Mo	STX3	0.000002	2.379788	*
3441	Transcriptional Coactivator with PDZ-Binding Mo	KDELC1	0	2.394957	*
3442	Transcriptional Coactivator with PDZ-Binding Mo	SLC35B4	0	2.415798	*
3443	Transcriptional Coactivator with PDZ-Binding Mo	LPXN	0.000004	2.519842	*
3444	Transcriptional Coactivator with PDZ-Binding Mo	CCDC50	0	2.532976	*
3445	Transcriptional Coactivator with PDZ-Binding Mo	GDF15	0.000001	2.550594	*
3446	Transcriptional Coactivator with PDZ-Binding Mo	ATP8B2	0.000001	2.559449	*
3447	Transcriptional Coactivator with PDZ-Binding Mo	PAG1	0	2.608704	*
3448	Transcriptional Coactivator with PDZ-Binding Mo	RMND5A	0	2.719485	*

3449	Transcriptional Coactivator with PDZ-Binding Mo	IFRD1	0	2.733659	*
3450	Transcriptional Coactivator with PDZ-Binding Mo	ISG20	0.000005	2.830061	*
3451	Transcriptional Coactivator with PDZ-Binding Mo	ZFYVE26	0	2.843169	*
3452	Transcriptional Coactivator with PDZ-Binding Mo	SRPX	0	2.864599	*
3453	Transcriptional Coactivator with PDZ-Binding Mo	PELI2	0	2.975915	*
3454	Transcriptional Coactivator with PDZ-Binding Mo	BEX4	0.000003	2.975915	*
3455	Transcriptional Coactivator with PDZ-Binding Mo	SLC4A8	0.000001	3.303892	*
3456	Transcriptional Coactivator with PDZ-Binding Mo	CYP39A1	0	3.334568	*
3457	Transcriptional Coactivator with PDZ-Binding Mo	FAM46C	0	3.338423	*
3458	Transcriptional Coactivator with PDZ-Binding Mo	CYFIP2	0.000001	3.430297	*
3459	Transcriptional Coactivator with PDZ-Binding Mo	ABCB1	0	3.655326	*
3460	Transcriptional Coactivator with PDZ-Binding Mo	NOV	0.000001	3.717072	*
3461	Transcriptional Coactivator with PDZ-Binding Mo	FABP3	0.000003	3.931282	*
3462	Transcriptional Coactivator with PDZ-Binding Mo	NUDT11	0	3.990769	*
3463	Transcriptional Coactivator with PDZ-Binding Mo	DNM3	0.000002	4.095861	*
3464	Transcriptional Coactivator with PDZ-Binding Mo	HPCAL4	0	4.555155	*
3465	Transcriptional Coactivator with PDZ-Binding Mo	CNTN1	0.000001	4.921734	*
3466	Transcriptional Coactivator with PDZ-Binding Mo	GZMA	0.000002	5.101188	*
3467	Transcriptional Coactivator with PDZ-Binding Mo	SLC40A1	0.000001	6.165281	*
3468	Transcriptional Coactivator with PDZ-Binding Mo	MUC16	0	6.356944	*
3469	Transcriptional Coactivator with PDZ-Binding Mo	GNG2	0	6.848715	*
3470	Transcriptional Coactivator with PDZ-Binding Mo	MUM1L1	0	7.000704	*
3471	Transcriptional Coactivator with PDZ-Binding Mo	IL24	0	7.700756	*
3472	Transcriptional Coactivator with PDZ-Binding Mo	KANK4	0	8.121052	*
3473	Transcriptional Coactivator with PDZ-Binding Mo	LPPR4	0	11.145067	*
3474	Transcriptional Coactivator with PDZ-Binding Mo	VAT1L	0	14.604422	*
3475	[D]Change in bowel habit	CCL19	0.037172	-1.740825	
3476	[D]Change in bowel habit	LTB	0.005796	-1.62032	
3477	[D]Change in bowel habit	CXCL13	0.044292	-1.556556	
3478	[D]Change in bowel habit	HLA-DQB1	0.029771	-1.533981	
3479	[M]Adenocarcinoma, metastatic, NOS	MMP3	0	-12.325909	
3480	[M]Adenocarcinoma, metastatic, NOS	ZG16	0	-10.328443	
3481	[M]Adenocarcinoma, metastatic, NOS	CA1	0	-8.678027	
3482	[M]Adenocarcinoma, metastatic, NOS	CLCA4	0	-8.27553	
3483	[M]Adenocarcinoma, metastatic, NOS	ITLN1	0	-7.726315	
3484	[M]Adenocarcinoma, metastatic, NOS	MS4A12	0	-6.291874	
3485	[M]Adenocarcinoma, metastatic, NOS	CLCA1	0	-6.180429	
3486	[M]Adenocarcinoma, metastatic, NOS	NKX2-3	0	-5.14191	
3487	[M]Adenocarcinoma, metastatic, NOS	SPINK4	0	-5.116714	
3488	[M]Adenocarcinoma, metastatic, NOS	CA4	0	-5.097768	
3489	[M]Adenocarcinoma, metastatic, NOS	DES	0	-4.777964	
3490	[M]Adenocarcinoma, metastatic, NOS	CXCL5	0	-4.741591	
3491	[M]Adenocarcinoma, metastatic, NOS	FCGBP	0	-4.299719	
3492	[M]Adenocarcinoma, metastatic, NOS	GPR109B	0	-4.22727	
3493	[M]Adenocarcinoma, metastatic, NOS	MAB21L2	0	-3.972382	
3494	[M]Adenocarcinoma, metastatic, NOS	DUOX2	0	-3.895751	
3495	[M]Adenocarcinoma, metastatic, NOS	ACTG2	0	-3.768196	
3496	[M]Adenocarcinoma, metastatic, NOS	ST6GALNAC1	0	-3.708631	
3497	[M]Adenocarcinoma, metastatic, NOS	MUC2	0.000153	-3.660982	
3498	[M]Adenocarcinoma, metastatic, NOS	MYH11	0	-3.650643	

3499	[M]Adenocarcinoma, metastatic, NOS	VIP	0	-3.608082	
3500	[M]Adenocarcinoma, metastatic, NOS	AQP8	0.000283	-3.606689	
3501	[M]Adenocarcinoma, metastatic, NOS	PIGR	0	-3.54694	
3502	[M]Adenocarcinoma, metastatic, NOS	CEACAM7	0	-3.457317	
3503	[M]Adenocarcinoma, metastatic, NOS	SYNM	0.000003	-3.429671	
3504	[M]Adenocarcinoma, metastatic, NOS	PI3	0.000001	-3.26821	
3505	[M]Adenocarcinoma, metastatic, NOS	CNN1	0.000002	-3.189323	
3506	[M]Adenocarcinoma, metastatic, NOS	IGJ	0	-3.153647	
3507	[M]Adenocarcinoma, metastatic, NOS	GPR120	0	-3.090857	
3508	[M]Adenocarcinoma, metastatic, NOS	SLC26A3	0.000002	-3.052374	
3509	[M]Adenocarcinoma, metastatic, NOS	SYNPO2	0	-3.051997	
3510	[M]Adenocarcinoma, metastatic, NOS	MUC4	0.00002	-3.016173	
3511	[M]Adenocarcinoma, metastatic, NOS	GUCA2A	0.000014	-3.013317	
3512	[M]Adenocarcinoma, metastatic, NOS	TRPA1	0	-3.007264	
3513	[M]Adenocarcinoma, metastatic, NOS	PLA2G2A	0	-2.977722	
3514	[M]Adenocarcinoma, metastatic, NOS	CHRDL1	0.00006	-2.90997	
3515	[M]Adenocarcinoma, metastatic, NOS	SCARA5	0	-2.875792	
3516	[M]Adenocarcinoma, metastatic, NOS	ASB2	0	-2.866584	
3517	[M]Adenocarcinoma, metastatic, NOS	PLN	0.000004	-2.78013	
3518	[M]Adenocarcinoma, metastatic, NOS	CASQ2	0.000029	-2.774595	
3519	[M]Adenocarcinoma, metastatic, NOS	PLAC8	0	-2.765247	
3520	[M]Adenocarcinoma, metastatic, NOS	IGHM	0.000027	-2.749567	
3521	[M]Adenocarcinoma, metastatic, NOS	LOC646627	0.00001	-2.725	
3522	[M]Adenocarcinoma, metastatic, NOS	C6orf105	0	-2.709693	
3523	[M]Adenocarcinoma, metastatic, NOS	TNFRSF17	0.000002	-2.702203	
3524	[M]Adenocarcinoma, metastatic, NOS	GREM1	0	-2.701485	
3525	[M]Adenocarcinoma, metastatic, NOS	FOXF1	0	-2.682872	
3526	[M]Adenocarcinoma, metastatic, NOS	PCDH18	0	-2.649088	
3527	[M]Adenocarcinoma, metastatic, NOS	CCL11	0	-2.618263	
3528	[M]Adenocarcinoma, metastatic, NOS	LMOD1	0	-2.614908	
3529	[M]Adenocarcinoma, metastatic, NOS	DHRS9	0	-2.591567	
3530	[M]Adenocarcinoma, metastatic, NOS	IL1B	0	-2.580174	
3531	[M]Adenocarcinoma, metastatic, NOS	DUOXA2	0.000001	-2.535967	
3532	[M]Adenocarcinoma, metastatic, NOS	CXCL1	0	-2.512596	
3533	[M]Adenocarcinoma, metastatic, NOS	ADAMDEC1	0	-2.512056	
3534	[M]Adenocarcinoma, metastatic, NOS	TSPAN11	0	-2.459401	
3535	[M]Adenocarcinoma, metastatic, NOS	IGHA1	0.000001	-2.447489	
3536	[M]Adenocarcinoma, metastatic, NOS	FAM55A	0	-2.436927	
3537	[M]Adenocarcinoma, metastatic, NOS	LCN2	0.000232	-2.434002	
3538	[M]Adenocarcinoma, metastatic, NOS	ARL14	0	-2.410036	
3539	[M]Adenocarcinoma, metastatic, NOS	IGL@	0.000011	-2.408791	
3540	[M]Adenocarcinoma, metastatic, NOS	IGLC7	0.000005	-2.407871	
3541	[M]Adenocarcinoma, metastatic, NOS	MYOCD	0.000027	-2.407244	
3542	[M]Adenocarcinoma, metastatic, NOS	CXCL11	0.000293	-2.399521	
3543	[M]Adenocarcinoma, metastatic, NOS	IGHD	0.000086	-2.398826	
3544	[M]Adenocarcinoma, metastatic, NOS	IL8	0.000031	-2.381619	
3545	[M]Adenocarcinoma, metastatic, NOS	HOXD13	0.000237	-2.319667	
3546	[M]Adenocarcinoma, metastatic, NOS	NR3C2	0	-2.310118	
3547	[M]Adenocarcinoma, metastatic, NOS	CES3	0	-2.308536	
3548	[M]Adenocarcinoma, metastatic, NOS	GCNT3	0.000002	-2.296123	

3549	[M]Adenocarcinoma, metastatic, NOS	TPSG1	0	-2.286308	
3550	[M]Adenocarcinoma, metastatic, NOS	IGKC	0.000003	-2.260557	
3551	[M]Adenocarcinoma, metastatic, NOS	TTLL6	0.000037	-2.253982	
3552	[M]Adenocarcinoma, metastatic, NOS	CXCL3	0	-2.244682	
3553	[M]Adenocarcinoma, metastatic, NOS	PTGER4	0.000059	-2.243168	
3554	[M]Adenocarcinoma, metastatic, NOS	CFD	0.000028	-2.220972	
3555	[M]Adenocarcinoma, metastatic, NOS	CCL20	0.000002	-2.202755	
3556	[M]Adenocarcinoma, metastatic, NOS	CLC	0.000002	-2.199556	
3557	[M]Adenocarcinoma, metastatic, NOS	UGT8	0	-2.160099	
3558	[M]Adenocarcinoma, metastatic, NOS	PGM5	0	-2.140712	
3559	[M]Adenocarcinoma, metastatic, NOS	ITM2C	0	-2.137483	
3560	[M]Adenocarcinoma, metastatic, NOS	NEGR1	0.00022	-2.136557	
3561	[M]Adenocarcinoma, metastatic, NOS	ATP1A2	0.000042	-2.129877	
3562	[M]Adenocarcinoma, metastatic, NOS	COL12A1	0.000068	-2.127289	
3563	[M]Adenocarcinoma, metastatic, NOS	BTNL8	0.000156	-2.112837	
3564	[M]Adenocarcinoma, metastatic, NOS	TSPAN1	0.000005	-2.103625	
3565	[M]Adenocarcinoma, metastatic, NOS	CCL28	0	-2.100519	
3566	[M]Adenocarcinoma, metastatic, NOS	BEST2	0.000001	-2.082137	
3567	[M]Adenocarcinoma, metastatic, NOS	MXD1	0.000061	-2.07945	
3568	[M]Adenocarcinoma, metastatic, NOS	LOC401093	0.000015	-2.07855	
3569	[M]Adenocarcinoma, metastatic, NOS	IGK@	0.000105	-2.073315	
3570	[M]Adenocarcinoma, metastatic, NOS	TACR2	0.000003	-2.066205	
3571	[M]Adenocarcinoma, metastatic, NOS	FHL1	0.000016	-2.065244	
3572	[M]Adenocarcinoma, metastatic, NOS	SPINK5	0.000008	-2.063378	
3573	[M]Adenocarcinoma, metastatic, NOS	NOS2	0	-2.030442	
3574	[M]Adenocarcinoma, metastatic, NOS	EDN3	0.000008	-2.029476	
3575	[M]Adenocarcinoma, metastatic, NOS	RHBDL2	0.000018	-2.008757	
3576	[M]Adenocarcinoma, metastatic, NOS	IL1R2	0.000005	-2.004665	
3577	[M]Adenocarcinoma, metastatic, NOS	SPP1	0.000061	2.517021	
3578	Abdominal bloating	FABP1	0	-2308.77868	
3579	Abdominal bloating	MUC12	0	-209.373649	
3580	Abdominal bloating	CLCA1	0	-166.329655	
3581	Abdominal bloating	SLC26A3	0	-146.423622	
3582	Abdominal bloating	KIAA1324	0	-91.654443	
3583	Abdominal bloating	KRT20	0	-86.507882	
3584	Abdominal bloating	HMGCS2	0	-83.639346	
3585	Abdominal bloating	MS4A12	0	-82.448661	
3586	Abdominal bloating	LOC646627	0	-72.555607	
3587	Abdominal bloating	CEACAM7	0	-68.625248	
3588	Abdominal bloating	REG4	0	-63.627426	
3589	Abdominal bloating	RPS4Y1	0	-62.909971	
3590	Abdominal bloating	ZG16	0	-59.824355	
3591	Abdominal bloating	UGT2B17	0	-59.327625	
3592	Abdominal bloating	REG3A	0	-58.73218	
3593	Abdominal bloating	SPINK4	0	-57.286765	
3594	Abdominal bloating	CLRN3	0	-53.143041	
3595	Abdominal bloating	NOX1	0	-52.860669	
3596	Abdominal bloating	CDX2	0	-50.815172	
3597	Abdominal bloating	ITLN1	0	-48.922536	
3598	Abdominal bloating	GPX2	0	-44.495062	

3599	Abdominal bloating	MUC2	0	-44.020129	
3600	Abdominal bloating	MEP1A	0	-43.346431	
3601	Abdominal bloating	CDH17	0	-41.09036	
3602	Abdominal bloating	ASCL2	0	-39.896756	
3603	Abdominal bloating	PLA2G2A	0	-38.307471	
3604	Abdominal bloating	RETNLB	0	-35.592131	
3605	Abdominal bloating	CDX1	0	-34.634482	
3606	Abdominal bloating	MUC13	0	-34.226901	
3607	Abdominal bloating	CYorf15B	0	-31.324209	
3608	Abdominal bloating	DEFA6	0.000002	-31.267773	
3609	Abdominal bloating	REG1A	0	-31.051786	
3610	Abdominal bloating	CDHR5	0	-30.500108	
3611	Abdominal bloating	PCK1	0	-29.962275	
3612	Abdominal bloating	POF1B	0	-29.119571	
3613	Abdominal bloating	LEFTY1	0	-28.018078	
3614	Abdominal bloating	PRAC	0	-26.629458	
3615	Abdominal bloating	GPA33	0	-25.961832	
3616	Abdominal bloating	CELP	0	-25.83888	
3617	Abdominal bloating	DPEP1	0	-24.530945	
3618	Abdominal bloating	HOXA10	0	-23.590861	
3619	Abdominal bloating	EPS8L3	0	-23.070948	
3620	Abdominal bloating	HOXB13	0	-22.04816	
3621	Abdominal bloating	C10orf99	0	-21.576673	
3622	Abdominal bloating	FUT6	0	-21.479253	
3623	Abdominal bloating	MYH11	0	-20.433715	
3624	Abdominal bloating	LCN15	0.000001	-20.371923	
3625	Abdominal bloating	DEFA5	0	-20.030758	
3626	Abdominal bloating	SI	0	-19.435363	
3627	Abdominal bloating	GUCY2C	0	-19.410843	
3628	Abdominal bloating	PRAP1	0	-18.986976	
3629	Abdominal bloating	ISX	0	-18.757539	
3630	Abdominal bloating	TRIM31	0	-18.493633	
3631	Abdominal bloating	AZGP1	0	-18.268344	
3632	Abdominal bloating	ATP10B	0	-18.175808	
3633	Abdominal bloating	NCRNA00261	0	-18.025907	
3634	Abdominal bloating	CEACAM1	0	-17.985376	
3635	Abdominal bloating	DDX3Y	0	-17.800707	
3636	Abdominal bloating	C9orf152	0	-17.626246	
3637	Abdominal bloating	ADH1C	0	-17.540833	
3638	Abdominal bloating	TNMD	0	-17.275699	
3639	Abdominal bloating	CCL14-CCL15	0	-16.911808	
3640	Abdominal bloating	C17orf76	0	-16.851722	
3641	Abdominal bloating	DMBT1	0	-16.468198	
3642	Abdominal bloating	EIF1AY	0	-15.942025	
3643	Abdominal bloating	UGT8	0	-15.892361	
3644	Abdominal bloating	GRM8	0	-15.886257	
3645	Abdominal bloating	CYP2B6	0	-15.761221	
3646	Abdominal bloating	PLCB4	0	-15.332196	
3647	Abdominal bloating	LGR5	0	-15.182773	
3648	Abdominal bloating	HNF4A	0	-14.899509	

3649	Abdominal bloating	SLC39A5	0	-14.674513	
3650	Abdominal bloating	FOXA2	0	-14.585559	
3651	Abdominal bloating	FAM55D	0	-14.452564	
3652	Abdominal bloating	NAT2	0	-14.367226	
3653	Abdominal bloating	DDC	0.000004	-14.048608	
3654	Abdominal bloating	KDM5D	0	-14.018731	
3655	Abdominal bloating	PPP1R1B	0	-13.860104	
3656	Abdominal bloating	REG1B	0.000004	-13.700289	
3657	Abdominal bloating	CDHR1	0.000002	-13.435	
3658	Abdominal bloating	HSD11B2	0	-13.191935	
3659	Abdominal bloating	CYorf15A	0	-13.120595	
3660	Abdominal bloating	VIL1	0	-12.874192	
3661	Abdominal bloating	IHH	0	-12.64539	
3662	Abdominal bloating	PRSS3	0	-12.612004	
3663	Abdominal bloating	CA4	0	-12.226428	
3664	Abdominal bloating	CFTR	0	-12.120136	
3665	Abdominal bloating	LRRC19	0	-12.10652	
3666	Abdominal bloating	HEPACAM2	0	-11.977799	
3667	Abdominal bloating	CA1	0	-11.911745	
3668	Abdominal bloating	PYY	0	-11.650642	
3669	Abdominal bloating	MUC17	0	-11.632019	
3670	Abdominal bloating	SLC27A2	0	-11.578725	
3671	Abdominal bloating	BMP4	0	-11.187964	
3672	Abdominal bloating	C1orf125	0	-11.185226	
3673	Abdominal bloating	VAV3	0	-11.114608	
3674	Abdominal bloating	TDGF1	0	-10.987569	
3675	Abdominal bloating	AQP8	0	-10.740918	
3676	Abdominal bloating	UGT1A1	0.000009	-10.703886	
3677	Abdominal bloating	RNF43	0	-10.673008	
3678	Abdominal bloating	NR1I2	0	-10.406598	
3679	Abdominal bloating	ACE2	0	-10.391024	
3680	Abdominal bloating	CST1	0	-10.002693	
3681	Abdominal bloating	ATOH1	0	-9.724825	
3682	Abdominal bloating	XK	0	-9.618045	
3683	Abdominal bloating	FGFR4	0	-9.613006	
3684	Abdominal bloating	FCGBP	0	-9.477318	
3685	Abdominal bloating	SATB2	0	-9.470035	
3686	Abdominal bloating	ANXA13	0	-9.348086	
3687	Abdominal bloating	HES6	0	-9.321556	
3688	Abdominal bloating	LOC100288092	0	-9.320831	
3689	Abdominal bloating	ACSL6	0	-9.315487	
3690	Abdominal bloating	XPNPEP2	0	-9.301162	
3691	Abdominal bloating	HOXA13	0	-9.274783	
3692	Abdominal bloating	CEL	0	-9.263451	
3693	Abdominal bloating	LY6G6D	0	-9.216926	
3694	Abdominal bloating	HORMAD1	0	-9.182895	
3695	Abdominal bloating	GUCA2A	0	-8.899813	
3696	Abdominal bloating	UGT1A8	0	-8.768448	
3697	Abdominal bloating	PNLIPRP2	0.000007	-8.746048	
3698	Abdominal bloating	ATP2A3	0	-8.635258	

3699	Abdominal bloating	RNF186	0	-8.458191	
3700	Abdominal bloating	NKX2-3	0	-8.456895	
3701	Abdominal bloating	IGJ	0.00001	-8.451432	
3702	Abdominal bloating	AKAP9	0	-8.393213	
3703	Abdominal bloating	PIP5K1B	0.000002	-8.330803	
3704	Abdominal bloating	KCNIP2	0	-8.266577	
3705	Abdominal bloating	TMED6	0	-8.254297	
3706	Abdominal bloating	PITX1	0	-8.246631	
3707	Abdominal bloating	TMPRSS2	0.000005	-8.057812	
3708	Abdominal bloating	GIPC2	0.000014	-7.999395	
3709	Abdominal bloating	C2orf89	0	-7.927386	
3710	Abdominal bloating	TSPAN1	0.000008	-7.88324	
3711	Abdominal bloating	C13orf18	0	-7.86143	
3712	Abdominal bloating	FLJ32063	0	-7.836731	
3713	Abdominal bloating	SLC12A2	0	-7.773392	
3714	Abdominal bloating	GMDS	0	-7.715403	
3715	Abdominal bloating	MOGAT3	0	-7.714644	
3716	Abdominal bloating	ABCB11	0	-7.702104	
3717	Abdominal bloating	CNTNAP2	0	-7.623161	
3718	Abdominal bloating	DLX1	0	-7.544396	
3719	Abdominal bloating	CETP	0.000005	-7.541725	
3720	Abdominal bloating	PIGR	0	-7.518368	
3721	Abdominal bloating	IL17RB	0	-7.505405	
3722	Abdominal bloating	PROX1	0	-7.50077	
3723	Abdominal bloating	ABCC2	0	-7.455205	
3724	Abdominal bloating	SLC5A1	0.000007	-7.441822	
3725	Abdominal bloating	LOC100507192	0.000008	-7.403896	
3726	Abdominal bloating	ITGA6	0	-7.252551	
3727	Abdominal bloating	NCRNA00253	0	-7.179077	
3728	Abdominal bloating	KLF4	0	-7.172949	
3729	Abdominal bloating	NCRNA00185	0	-7.166042	
3730	Abdominal bloating	SLC18A1	0	-7.164393	
3731	Abdominal bloating	CYP2J2	0	-7.112121	
3732	Abdominal bloating	MYO7B	0	-7.099161	
3733	Abdominal bloating	F11	0.000001	-7.022134	
3734	Abdominal bloating	LOC400573	0	-6.986016	
3735	Abdominal bloating	MYO1A	0	-6.968781	
3736	Abdominal bloating	LOC157860	0	-6.967505	
3737	Abdominal bloating	SOX8	0	-6.793978	
3738	Abdominal bloating	FAM55A	0	-6.765863	
3739	Abdominal bloating	EPB41L4B	0.000001	-6.736532	
3740	Abdominal bloating	PCCA	0	-6.671335	
3741	Abdominal bloating	CKMT1A	0.000003	-6.650253	
3742	Abdominal bloating	GPR160	0	-6.556168	
3743	Abdominal bloating	HOXD13	0	-6.528536	
3744	Abdominal bloating	GPR120	0.000006	-6.51586	
3745	Abdominal bloating	ARSE	0	-6.509081	
3746	Abdominal bloating	GAL	0	-6.468017	
3747	Abdominal bloating	SYNPO2	0	-6.442706	
3748	Abdominal bloating	CAPN6	0.000001	-6.433942	

3749	Abdominal bloating	CYP4F2	0.000012	-6.410884	
3750	Abdominal bloating	L1TD1	0	-6.396891	
3751	Abdominal bloating	SLC3A1	0	-6.260852	
3752	Abdominal bloating	PARM1	0.000007	-6.236635	
3753	Abdominal bloating	NOS2	0	-6.1485	
3754	Abdominal bloating	LOC145837	0.000001	-6.129473	
3755	Abdominal bloating	DUSP27	0.000006	-6.126684	
3756	Abdominal bloating	BCAN	0	-6.00412	
3757	Abdominal bloating	FAIM2	0.000015	-5.959748	
3758	Abdominal bloating	CHP2	0	-5.936097	
3759	Abdominal bloating	GUCA2B	0	-5.932057	
3760	Abdominal bloating	ARHGAP27	0.000007	-5.885347	
3761	Abdominal bloating	PRLR	0	-5.866977	
3762	Abdominal bloating	USP9Y	0.000002	-5.855325	
3763	Abdominal bloating	BTNL8	0.000015	-5.847233	
3764	Abdominal bloating	ABCB1	0	-5.822776	
3765	Abdominal bloating	KIAA1199	0	-5.763513	
3766	Abdominal bloating	A1CF	0.000002	-5.701754	
3767	Abdominal bloating	SLC1A7	0	-5.693207	
3768	Abdominal bloating	TMEM54	0	-5.642958	
3769	Abdominal bloating	FAM84A	0	-5.633227	
3770	Abdominal bloating	CA12	0	-5.567145	
3771	Abdominal bloating	PTGER4	0.000001	-5.542314	
3772	Abdominal bloating	FAM134B	0	-5.512663	
3773	Abdominal bloating	FAR2	0	-5.46811	
3774	Abdominal bloating	GPR128	0	-5.434922	
3775	Abdominal bloating	MYLK	0	-5.414259	
3776	Abdominal bloating	SYT7	0	-5.411638	
3777	Abdominal bloating	RNASE4	0.000002	-5.404236	
3778	Abdominal bloating	RBP4	0	-5.395766	
3779	Abdominal bloating	FREM1	0	-5.380023	
3780	Abdominal bloating	TPSG1	0	-5.30033	
3781	Abdominal bloating	KBTBD11	0.000009	-5.292846	
3782	Abdominal bloating	GPRC5D	0.000005	-5.247907	
3783	Abdominal bloating	GIPR	0	-5.202	
3784	Abdominal bloating	STARD10	0.000001	-5.180497	
3785	Abdominal bloating	CHGA	0	-5.147575	
3786	Abdominal bloating	KALRN	0.000009	-5.107389	
3787	Abdominal bloating	BEST2	0	-5.095133	
3788	Abdominal bloating	CHD7	0	-5.075944	
3789	Abdominal bloating	SLC13A2	0.000014	-5.068962	
3790	Abdominal bloating	DDAH1	0.000009	-5.061878	
3791	Abdominal bloating	TESC	0.00001	-5.014794	
3792	Abdominal bloating	ANG	0	-5.012935	
3793	Abdominal bloating	EDN3	0	-4.996522	
3794	Abdominal bloating	SEL1L2	0	-4.985393	
3795	Abdominal bloating	KIAA1984	0	-4.963625	
3796	Abdominal bloating	ADCYAP1	0	-4.925003	
3797	Abdominal bloating	DNASE1L3	0.000005	-4.904384	
3798	Abdominal bloating	MRAP2	0.000014	-4.871309	

3799	Abdominal bloating	APOB48R	0.000006	-4.869789	
3800	Abdominal bloating	HOXD10	0	-4.836483	
3801	Abdominal bloating	CLDN15	0	-4.82792	
3802	Abdominal bloating	ASPA	0.000018	-4.823707	
3803	Abdominal bloating	LOC26102	0.000017	-4.740998	
3804	Abdominal bloating	GRIA4	0.000006	-4.718374	
3805	Abdominal bloating	IGLV6-57	0	-4.703152	
3806	Abdominal bloating	FABP6	0.000006	-4.681751	
3807	Abdominal bloating	DSEL	0	-4.644315	
3808	Abdominal bloating	CKB	0	-4.64174	
3809	Abdominal bloating	TF	0	-4.635545	
3810	Abdominal bloating	PTPRN2	0	-4.573172	
3811	Abdominal bloating	CAB39L	0.000007	-4.564665	
3812	Abdominal bloating	RTP3	0.000017	-4.539891	
3813	Abdominal bloating	AMACR	0	-4.51568	
3814	Abdominal bloating	WNK2	0.000001	-4.491796	
3815	Abdominal bloating	PER2	0	-4.468161	
3816	Abdominal bloating	LOC100240728	0	-4.468025	
3817	Abdominal bloating	PKHD1L1	0	-4.466044	
3818	Abdominal bloating	CHN2	0	-4.448822	
3819	Abdominal bloating	CES2	0	-4.440582	
3820	Abdominal bloating	SLC26A2	0.000019	-4.437685	
3821	Abdominal bloating	PTPRO	0	-4.432594	
3822	Abdominal bloating	DKFZP434L18[illegible]	0	-4.427344	
3823	Abdominal bloating	HSD17B3	0	-4.399416	
3824	Abdominal bloating	GABRA2	0	-4.390212	
3825	Abdominal bloating	SRRM3	0	-4.388701	
3826	Abdominal bloating	NEK3	0.000017	-4.388514	
3827	Abdominal bloating	C3orf65	0	-4.364095	
3828	Abdominal bloating	FGGY	0	-4.355683	
3829	Abdominal bloating	EML6	0	-4.322767	
3830	Abdominal bloating	RPL3L	0.00002	-4.302074	
3831	Abdominal bloating	NOTUM	0.000012	-4.301176	
3832	Abdominal bloating	RFFL	0.000003	-4.285123	
3833	Abdominal bloating	TMEM151B	0.000016	-4.251667	
3834	Abdominal bloating	C2orf61	0	-4.232169	
3835	Abdominal bloating	GALR2	0.000001	-4.209086	
3836	Abdominal bloating	CPS1-IT	0.000001	-4.189593	
3837	Abdominal bloating	CD6	0	-4.177068	
3838	Abdominal bloating	FOXC2	0.000001	-4.152818	
3839	Abdominal bloating	LOC100288781	0	-4.124622	
3840	Abdominal bloating	HOXB9	0	-4.110946	
3841	Abdominal bloating	FAM26D	0	-4.101276	
3842	Abdominal bloating	JPH1	0	-4.086549	
3843	Abdominal bloating	MYOCD	0	-4.05474	
3844	Abdominal bloating	LOC100505633	0.000002	-4.040562	
3845	Abdominal bloating	RHAG	0	-4.034003	
3846	Abdominal bloating	AIF1	0	-4.017584	
3847	Abdominal bloating	ZBTB7A	0	-3.983099	
3848	Abdominal bloating	TUSC5	0	-3.964784	

3849	Abdominal bloating	LOC572558	0	-3.936	
3850	Abdominal bloating	ME1	0.000016	-3.897653	
3851	Abdominal bloating	LOC100509223	0	-3.896447	
3852	Abdominal bloating	ARHGAP20	0.000019	-3.85838	
3853	Abdominal bloating	FMO5	0	-3.843681	
3854	Abdominal bloating	FLJ22763	0.000006	-3.782035	
3855	Abdominal bloating	NFATC1	0	-3.763127	
3856	Abdominal bloating	HNMT	0	-3.762275	
3857	Abdominal bloating	LOC25845	0	-3.746591	
3858	Abdominal bloating	ENPP3	0	-3.737595	
3859	Abdominal bloating	FGD2	0	-3.73574	
3860	Abdominal bloating	SIRT5	0.000003	-3.70643	
3861	Abdominal bloating	TMCC2	0	-3.696502	
3862	Abdominal bloating	GRTP1	0.000005	-3.692202	
3863	Abdominal bloating	HR	0.000002	-3.66396	
3864	Abdominal bloating	MS4A5	0.000012	-3.646129	
3865	Abdominal bloating	CA10	0.000019	-3.602751	
3866	Abdominal bloating	PIGZ	0	-3.60197	
3867	Abdominal bloating	WDR59	0	-3.597508	
3868	Abdominal bloating	CSHL1	0.000012	-3.592693	
3869	Abdominal bloating	TCF21	0	-3.561143	
3870	Abdominal bloating	PAH	0	-3.559849	
3871	Abdominal bloating	AFF2	0.000002	-3.53782	
3872	Abdominal bloating	LOC283177	0	-3.460578	
3873	Abdominal bloating	CDH8	0.000001	-3.457635	
3874	Abdominal bloating	LOC100506591	0.000001	-3.4189	
3875	Abdominal bloating	LRRC66	0	-3.41537	
3876	Abdominal bloating	LAMA4	0	-3.362465	
3877	Abdominal bloating	C14orf176	0.000001	-3.352164	
3878	Abdominal bloating	ALG2	0	-3.33906	
3879	Abdominal bloating	BCL11A	0	-3.319183	
3880	Abdominal bloating	C8orf46	0.00001	-3.306362	
3881	Abdominal bloating	AKAP1	0.000001	-3.244937	
3882	Abdominal bloating	RBM26	0.000002	-3.226504	
3883	Abdominal bloating	KLK1	0.000006	-3.223835	
3884	Abdominal bloating	DIAPH2	0.000015	-3.207873	
3885	Abdominal bloating	MTUS1	0	-3.201365	
3886	Abdominal bloating	RNF6	0	-3.179625	
3887	Abdominal bloating	SEMA4G	0.000012	-3.168755	
3888	Abdominal bloating	MINK1	0	-3.16079	
3889	Abdominal bloating	PRPH	0.00002	-3.113268	
3890	Abdominal bloating	C19orf6	0.000012	-3.091872	
3891	Abdominal bloating	PHACTR1	0	-3.086872	
3892	Abdominal bloating	C6orf15	0.000009	-3.084821	
3893	Abdominal bloating	IDI2	0	-3.079875	
3894	Abdominal bloating	IL1B	0	-3.077073	
3895	Abdominal bloating	PBLD	0.000002	-3.040638	
3896	Abdominal bloating	GPR172B	0.000009	-3.007794	
3897	Abdominal bloating	TRIB3	0.000011	-2.978401	
3898	Abdominal bloating	MAG	0.000001	-2.9736	

3899	Abdominal bloating	IRX4	0.000018	-2.9407	
3900	Abdominal bloating	KCNE3	0.000009	-2.895039	
3901	Abdominal bloating	C8orf74	0.000001	-2.893661	
3902	Abdominal bloating	PRDM8	0.000016	-2.837176	
3903	Abdominal bloating	NDFIP2	0	-2.805343	
3904	Abdominal bloating	CACNB4	0	-2.736415	
3905	Abdominal bloating	GLRX	0.000003	-2.73054	
3906	Abdominal bloating	TMEM56	0.000007	-2.693111	
3907	Abdominal bloating	ASB9	0.00002	-2.603547	
3908	Abdominal bloating	AKR7A3	0	-2.579497	
3909	Abdominal bloating	NEDD9	0.000003	-2.536093	
3910	Abdominal bloating	PPP2R2C	0	-2.529724	
3911	Abdominal bloating	CDC42SE2	0	-2.503239	
3912	Abdominal bloating	CDC42	0.000001	-2.497118	
3913	Abdominal bloating	TCF3	0.000001	-2.48319	
3914	Abdominal bloating	PREP	0	-2.47868	
3915	Abdominal bloating	C13orf23	0	-2.469742	
3916	Abdominal bloating	UGT1A6	0.000003	-2.380072	
3917	Abdominal bloating	FRYL	0	-2.354414	
3918	Abdominal bloating	ALDH1B1	0.000014	-2.332173	
3919	Abdominal bloating	ART3	0	-2.314089	
3920	Abdominal bloating	REPS2	0	-2.309815	
3921	Abdominal bloating	L3MBTL1	0	-2.265791	
3922	Abdominal bloating	ZNF511	0	-2.15189	
3923	Abdominal bloating	EREG	0.000008	-2.058147	
3924	Abdominal bloating	PPIEL	0	-2.004902	
3925	Abscess	UGT2A3	0.040416	1.545498	
3926	Adenocarcinoma	OSTalpha	0.000026	-47.033402	
3927	Adenocarcinoma	COL6A1	0.000411	-28.023475	
3928	Adenocarcinoma	GCG	0	-16.899049	
3929	Adenocarcinoma	SLC30A10	0.000006	-16.146988	
3930	Adenocarcinoma	AQP8	0.000001	-15.832717	
3931	Adenocarcinoma	DNAJC12	0.002663	-14.269987	
3932	Adenocarcinoma	C12orf29	0.00027	-13.128954	
3933	Adenocarcinoma	STK33	0.01202	-11.276326	
3934	Adenocarcinoma	C1orf61	0.018839	-9.579861	
3935	Adenocarcinoma	VSTM2A	0	-9.14717	
3936	Adenocarcinoma	CXorf57	0.006263	-8.627943	
3937	Adenocarcinoma	PLP1	0	-8.565039	
3938	Adenocarcinoma	EMR2	0.000098	-8.515725	
3939	Adenocarcinoma	NR1H4	0.000846	-8.412746	
3940	Adenocarcinoma	GNAI1	0.00045	-8.167461	
3941	Adenocarcinoma	NPY6R	0	-8.003144	
3942	Adenocarcinoma	ROBO1	0.010314	-7.391541	
3943	Adenocarcinoma	PRDM16	0.030623	-7.14597	
3944	Adenocarcinoma	CCL23	0.000022	-7.094239	
3945	Adenocarcinoma	MFAP2	0.004677	-6.950453	
3946	Adenocarcinoma	NRXN1	0	-6.901082	
3947	Adenocarcinoma	KANK4	0.000582	-6.736475	
3948	Adenocarcinoma	ANGPTL1	0.000002	-6.700761	

3949	Adenocarcinoma	SLC25A34	0.000022	-6.674234	
3950	Adenocarcinoma	PCDHGA1	0.00004	-6.51504	
3951	Adenocarcinoma	GNG13	0	-6.173533	
3952	Adenocarcinoma	DHRS9	0.000007	-6.14952	
3953	Adenocarcinoma	PTGDR	0	-6.016754	
3954	Adenocarcinoma	KIAA2022	0.000001	-5.99665	
3955	Adenocarcinoma	RCOR2	0.000034	-5.979644	
3956	Adenocarcinoma	ADH1C	0.00002	-5.903407	
3957	Adenocarcinoma	GUCA2B	0.000279	-5.893998	
3958	Adenocarcinoma	PIK3CD	0.011996	-5.838027	
3959	Adenocarcinoma	MAMDC2	0	-5.826741	
3960	Adenocarcinoma	DNM3	0.003505	-5.795441	
3961	Adenocarcinoma	C2orf58	0	-5.665292	
3962	Adenocarcinoma	LGI4	0	-5.496047	
3963	Adenocarcinoma	IL1R2	0.000052	-5.493194	
3964	Adenocarcinoma	CDH19	0	-5.453266	
3965	Adenocarcinoma	RNF220	0.000002	-5.421607	
3966	Adenocarcinoma	NTRK2	0.002325	-5.420538	
3967	Adenocarcinoma	RGS13	0	-5.365318	
3968	Adenocarcinoma	SERPINI1	0.000034	-5.307325	
3969	Adenocarcinoma	SVOPL	0	-5.28447	
3970	Adenocarcinoma	PCSK2	0.000018	-5.268769	
3971	Adenocarcinoma	SPARCL1	0	-5.244626	
3972	Adenocarcinoma	FADS2	0.009782	-5.227213	
3973	Adenocarcinoma	SCN7A	0	-5.18474	
3974	Adenocarcinoma	AHNAK2	0.006163	-5.163912	
3975	Adenocarcinoma	C20orf194	0.011216	-5.127653	
3976	Adenocarcinoma	ZNF525	0.001611	-5.032233	
3977	Adenocarcinoma	BMP3	0.00004	-4.988584	
3978	Adenocarcinoma	SPIB	0.000008	-4.962092	
3979	Adenocarcinoma	SORCS1	0	-4.888339	
3980	Adenocarcinoma	FGL2	0	-4.775091	
3981	Adenocarcinoma	KCNV1	0	-4.774511	
3982	Adenocarcinoma	PDZD3	0.000026	-4.758979	
3983	Adenocarcinoma	FBXO27	0.019162	-4.73933	
3984	Adenocarcinoma	CKB	0.009854	-4.716011	
3985	Adenocarcinoma	VPS53	0.031742	-4.702636	
3986	Adenocarcinoma	DPP10	0.000008	-4.664912	
3987	Adenocarcinoma	TEX11	0.000007	-4.646122	
3988	Adenocarcinoma	LOC389023	0.000323	-4.643267	
3989	Adenocarcinoma	ADH1B	0.000129	-4.601914	
3990	Adenocarcinoma	HIPK2	0.019907	-4.586053	
3991	Adenocarcinoma	SNAI1	0.036191	-4.580315	
3992	Adenocarcinoma	PCDHGA3	0.02964	-4.558381	
3993	Adenocarcinoma	C7	0.000004	-4.513299	
3994	Adenocarcinoma	WSCD1	0.000004	-4.401202	
3995	Adenocarcinoma	BMX	0	-4.36962	
3996	Adenocarcinoma	KRTDAP	0.039655	-4.287323	
3997	Adenocarcinoma	TMEM195	0.009197	-4.27626	
3998	Adenocarcinoma	LRRN2	0.000038	-4.27253	

3999	Adenocarcinoma	OSTBETA	0.000019	-4.25459	
4000	Adenocarcinoma	LOC441644	0.000673	-4.164103	
4001	Adenocarcinoma	ATP1A2	0.000005	-4.144251	
4002	Adenocarcinoma	ZNF75A	0.000756	-4.135019	
4003	Adenocarcinoma	UGT1A8	0.000039	-4.117387	
4004	Adenocarcinoma	MID1	0.031742	-4.106245	
4005	Adenocarcinoma	PLAC8	0.000164	-4.104069	
4006	Adenocarcinoma	LOC645195	0.018949	-4.100791	
4007	Adenocarcinoma	OTOR	0.042846	-4.052284	
4008	Adenocarcinoma	RNF148	0.0398	-4.051476	
4009	Adenocarcinoma	SLC7A11	0.000153	-4.030736	
4010	Adenocarcinoma	CDH23	0.042984	-4.01686	
4011	Adenocarcinoma	FAM101B	0.011797	-4.016207	
4012	Adenocarcinoma	PTPRS	0.017946	-4.014396	
4013	Adenocarcinoma	CA2	0.000238	-3.967964	
4014	Adenocarcinoma	MOGAT2	0.000064	-3.948991	
4015	Adenocarcinoma	HHEX	0.001742	-3.935474	
4016	Adenocarcinoma	UGT2B17	0.000072	-3.933187	
4017	Adenocarcinoma	SLC26A2	0.000445	-3.928833	
4018	Adenocarcinoma	ARHGEF4	0.03862	-3.905656	
4019	Adenocarcinoma	ZNF134	0.001018	-3.903334	
4020	Adenocarcinoma	MAPK8IP1	0.005277	-3.885466	
4021	Adenocarcinoma	SCIN	0.000238	-3.855473	
4022	Adenocarcinoma	XKR4	0.000003	-3.845193	
4023	Adenocarcinoma	FAM55D	0.000219	-3.823774	
4024	Adenocarcinoma	SOX10	0	-3.805372	
4025	Adenocarcinoma	BMP5	0.000002	-3.798608	
4026	Adenocarcinoma	MGC48628	0.014958	-3.78765	
4027	Adenocarcinoma	XLKD1	0	-3.776766	
4028	Adenocarcinoma	PCLO	0.003695	-3.771198	
4029	Adenocarcinoma	FXYD3	0.000061	-3.760548	
4030	Adenocarcinoma	CSF1R	0.0008	-3.757652	
4031	Adenocarcinoma	APLF	0.007661	-3.743234	
4032	Adenocarcinoma	JARID1D	0	-3.727566	
4033	Adenocarcinoma	RIMKLB	0.009664	-3.714023	
4034	Adenocarcinoma	PCDH9	0	-3.700223	
4035	Adenocarcinoma	CAPN13	0	-3.658466	
4036	Adenocarcinoma	CES2	0.000229	-3.655126	
4037	Adenocarcinoma	TUBAL3	0.00023	-3.650291	
4038	Adenocarcinoma	PCK1	0.000292	-3.635801	
4039	Adenocarcinoma	HEPACAM2	0.00047	-3.61294	
4040	Adenocarcinoma	FLJ38379	0	-3.608873	
4041	Adenocarcinoma	PALLD	0.011243	-3.58331	
4042	Adenocarcinoma	CYB5B	0.000508	-3.579075	
4043	Adenocarcinoma	MAOA	0.00002	-3.573062	
4044	Adenocarcinoma	ZNF483	0.00139	-3.550036	
4045	Adenocarcinoma	CES3	0.000071	-3.541904	
4046	Adenocarcinoma	GPT	0.000045	-3.535019	
4047	Adenocarcinoma	ITM2C	0	-3.52899	
4048	Adenocarcinoma	SCUBE2	0	-3.527884	

4049	Adenocarcinoma	CNTN3	0.000217	-3.524586	
4050	Adenocarcinoma	SULT1B1	0.000027	-3.521881	
4051	Adenocarcinoma	RFX6	0.000515	-3.498165	
4052	Adenocarcinoma	SPATA17	0.024221	-3.493367	
4053	Adenocarcinoma	SLC17A4	0.000614	-3.493234	
4054	Adenocarcinoma	CRHBP	0.000022	-3.460216	
4055	Adenocarcinoma	LOC100508909	0.000124	-3.458099	
4056	Adenocarcinoma	VILL	0.000078	-3.451077	
4057	Adenocarcinoma	RDH5	0.000083	-3.450638	
4058	Adenocarcinoma	NAP1L2	0.000003	-3.425142	
4059	Adenocarcinoma	SLC38A4	0.000772	-3.421945	
4060	Adenocarcinoma	ANGPT2	0.036078	-3.420177	
4061	Adenocarcinoma	DSCR1L1	0	-3.414215	
4062	Adenocarcinoma	MFGE8	0.018949	-3.402006	
4063	Adenocarcinoma	HAPLN3	0.042984	-3.398351	
4064	Adenocarcinoma	SCARA5	0.000018	-3.382623	
4065	Adenocarcinoma	MIB2	0.029988	-3.380345	
4066	Adenocarcinoma	UGT1A1	0.000008	-3.369615	
4067	Adenocarcinoma	EYA2	0.000292	-3.360873	
4068	Adenocarcinoma	SF3B3	0.018215	-3.356775	
4069	Adenocarcinoma	IGLV4-60	0.000904	-3.356766	
4070	Adenocarcinoma	CKMT1A	0.000012	-3.35529	
4071	Adenocarcinoma	FRMD1	0.000195	-3.352684	
4072	Adenocarcinoma	PCDH20	0.000651	-3.343165	
4073	Adenocarcinoma	FRZB	0	-3.333763	
4074	Adenocarcinoma	GRIA3	0.000081	-3.324625	
4075	Adenocarcinoma	GCNT3	0.000089	-3.318624	
4076	Adenocarcinoma	PCDH19	0.000016	-3.31126	
4077	Adenocarcinoma	PDE6A	0.000883	-3.297526	
4078	Adenocarcinoma	HHLA2	0.000288	-3.269107	
4079	Adenocarcinoma	ZBTB7C	0.000141	-3.266716	
4080	Adenocarcinoma	FABP1	0.000007	-3.261659	
4081	Adenocarcinoma	DEFB1	0.000319	-3.249128	
4082	Adenocarcinoma	ABI3BP	0.00013	-3.247527	
4083	Adenocarcinoma	KCNE2	0.000013	-3.246601	
4084	Adenocarcinoma	LOC100506591	0.000001	-3.241716	
4085	Adenocarcinoma	ZFP37	0.001264	-3.238204	
4086	Adenocarcinoma	GRIN2A	0.000005	-3.236666	
4087	Adenocarcinoma	LOC644768	0.016103	-3.233501	
4088	Adenocarcinoma	CACNA2D2	0.000088	-3.22725	
4089	Adenocarcinoma	WFDC2	0.00064	-3.224515	
4090	Adenocarcinoma	AMPD1	0.000073	-3.220971	
4091	Adenocarcinoma	DBN1	0.016198	-3.210761	
4092	Adenocarcinoma	UPP1	0.025227	-3.191424	
4093	Adenocarcinoma	B4GALNT4	0.002911	-3.173298	
4094	Adenocarcinoma	ZZEF1	0.000261	-3.16765	
4095	Adenocarcinoma	ADAMTSL1	0.000071	-3.164431	
4096	Adenocarcinoma	GFRA1	0.000011	-3.150197	
4097	Adenocarcinoma	HAPLN1	0.000036	-3.142222	
4098	Adenocarcinoma	SCN9A	0.027295	-3.140818	

4099	Adenocarcinoma	B4GALNT2	0.000254	-3.139801	
4100	Adenocarcinoma	TMEM178	0.014645	-3.135835	
4101	Adenocarcinoma	LDHD	0.000027	-3.129231	
4102	Adenocarcinoma	PCBP3	0.012595	-3.124921	
4103	Adenocarcinoma	PHLPP2	0.000004	-3.124354	
4104	Adenocarcinoma	HES5	0.000044	-3.121633	
4105	Adenocarcinoma	EXD3	0.000012	-3.121086	
4106	Adenocarcinoma	SH2D6	0.000009	-3.104952	
4107	Adenocarcinoma	ANK2	0.003982	-3.104127	
4108	Adenocarcinoma	CACNA1H	0.016916	-3.097672	
4109	Adenocarcinoma	PAPSS2	0.000007	-3.090265	
4110	Adenocarcinoma	TMEM72	0.000079	-3.087539	
4111	Adenocarcinoma	FBLN1	0	-3.084209	
4112	Adenocarcinoma	PRSS36	0.016942	-3.080705	
4113	Adenocarcinoma	CEACAM1	0.000043	-3.080438	
4114	Adenocarcinoma	LOC285878	0.000002	-3.080395	
4115	Adenocarcinoma	SLC16A9	0.000347	-3.076333	
4116	Adenocarcinoma	SMO	0.002554	-3.073195	
4117	Adenocarcinoma	NFATC1	0.002194	-3.070761	
4118	Adenocarcinoma	FAM150B	0.000003	-3.058634	
4119	Adenocarcinoma	CDKL1	0.000668	-3.054882	
4120	Adenocarcinoma	METTL7A	0.000069	-3.051391	
4121	Adenocarcinoma	KLF4	0.000031	-3.042323	
4122	Adenocarcinoma	DLG4	0.002446	-3.042022	
4123	Adenocarcinoma	KIF16B	0.000012	-3.030102	
4124	Adenocarcinoma	CCDC68	0.000028	-3.028445	
4125	Adenocarcinoma	PTMS	0.001302	-3.02596	
4126	Adenocarcinoma	SPINK2	0.000162	-3.0247	
4127	Adenocarcinoma	ZNF662	0.000025	-3.018874	
4128	Adenocarcinoma	GFRA2	0.000011	-3.012423	
4129	Adenocarcinoma	PCSK1N	0.008874	-2.986282	
4130	Adenocarcinoma	TTC23	0.000018	-2.979335	
4131	Adenocarcinoma	GRIK2	0.000184	-2.978048	
4132	Adenocarcinoma	FAM46C	0.000015	-2.977186	
4133	Adenocarcinoma	DOCK4	0.001516	-2.975124	
4134	Adenocarcinoma	C9orf24	0.000511	-2.974354	
4135	Adenocarcinoma	IGFBP2	0.000009	-2.971447	
4136	Adenocarcinoma	CLCN4	0.035854	-2.971417	
4137	Adenocarcinoma	RASGEF1A	0.015566	-2.957298	
4138	Adenocarcinoma	USP7	0.000112	-2.951311	
4139	Adenocarcinoma	TFDP1	0.004751	-2.946349	
4140	Adenocarcinoma	C2orf14	0.009811	-2.939705	
4141	Adenocarcinoma	CHL1	0	-2.93542	
4142	Adenocarcinoma	THRB	0.000477	-2.92102	
4143	Adenocarcinoma	ETHE1	0.000003	-2.915623	
4144	Adenocarcinoma	TNFRSF17	0	-2.910197	
4145	Adenocarcinoma	FAM189A1	0.000032	-2.904059	
4146	Adenocarcinoma	PMFBP1	0.015446	-2.902076	
4147	Adenocarcinoma	CELA3B	0.000095	-2.901027	
4148	Adenocarcinoma	B3GALT5	0	-2.892246	

4149	Adenocarcinoma	TMEM171	0.000043	-2.885524	
4150	Adenocarcinoma	KLRB1	0	-2.8841	
4151	Adenocarcinoma	BCHE	0.000148	-2.873688	
4152	Adenocarcinoma	PRKAR2B	0.000238	-2.861024	
4153	Adenocarcinoma	MRE11A	0	-2.859549	
4154	Adenocarcinoma	PPP1R12B	0	-2.859112	
4155	Adenocarcinoma	CDKN1C	0.002964	-2.858738	
4156	Adenocarcinoma	DPT	0	-2.856891	
4157	Adenocarcinoma	PDGFRA	0.000026	-2.841994	
4158	Adenocarcinoma	ZDHHC8P	0.032276	-2.837954	
4159	Adenocarcinoma	KRT20	0.000255	-2.836996	
4160	Adenocarcinoma	NDUFAF4	0.000015	-2.827497	
4161	Adenocarcinoma	DHRS7C	0.000528	-2.814171	
4162	Adenocarcinoma	SLC1A7	0.000015	-2.805395	
4163	Adenocarcinoma	ZBTB1	0.014641	-2.799238	
4164	Adenocarcinoma	ENHO	0.000267	-2.795258	
4165	Adenocarcinoma	DSCAML1	0.00004	-2.791862	
4166	Adenocarcinoma	SGK1	0.000007	-2.786882	
4167	Adenocarcinoma	SIGLEC1	0.000004	-2.786226	
4168	Adenocarcinoma	HTR4	0.000108	-2.785586	
4169	Adenocarcinoma	HMGCS2	0.000347	-2.784072	
4170	Adenocarcinoma	KIAA1683	0.000381	-2.781161	
4171	Adenocarcinoma	DSC2	0.000053	-2.779295	
4172	Adenocarcinoma	ETFDH	0.000023	-2.757237	
4173	Adenocarcinoma	GSN	0	-2.753586	
4174	Adenocarcinoma	KIAA1430	0.035483	-2.748274	
4175	Adenocarcinoma	KIAA0319	0.000411	-2.747012	
4176	Adenocarcinoma	TMEM56	0.000041	-2.746816	
4177	Adenocarcinoma	CCDC18	0	-2.745822	
4178	Adenocarcinoma	SYDE1	0.01648	-2.738516	
4179	Adenocarcinoma	DCN	0	-2.734984	
4180	Adenocarcinoma	PRKG2	0.000925	-2.733283	
4181	Adenocarcinoma	TCEA2	0.015902	-2.730884	
4182	Adenocarcinoma	GPD1L	0.000012	-2.724242	
4183	Adenocarcinoma	MMP17	0.000352	-2.72376	
4184	Adenocarcinoma	CCNB3	0.011381	-2.721795	
4185	Adenocarcinoma	PHGR1	0.000007	-2.72038	
4186	Adenocarcinoma	C4orf34	0.000284	-2.70772	
4187	Adenocarcinoma	FTH1	0	-2.706549	
4188	Adenocarcinoma	FGF9	0.000671	-2.701905	
4189	Adenocarcinoma	C1orf115	0.000028	-2.700641	
4190	Adenocarcinoma	LIFR	0.000886	-2.695526	
4191	Adenocarcinoma	PRIC285	0	-2.689612	
4192	Adenocarcinoma	SQRDL	0.000007	-2.686297	
4193	Adenocarcinoma	RBM47	0.00004	-2.686042	
4194	Adenocarcinoma	CCDC88A	0.008983	-2.677126	
4195	Adenocarcinoma	MYO1A	0.000243	-2.673352	
4196	Adenocarcinoma	SRPX	0.00029	-2.663967	
4197	Adenocarcinoma	FMO5	0.000299	-2.660514	
4198	Adenocarcinoma	PTGER4	0	-2.652074	

4199	Adenocarcinoma	FAM55A	0.000402	-2.645677	
4200	Adenocarcinoma	USP2	0.000085	-2.636411	
4201	Adenocarcinoma	PIR	0.036703	-2.634643	
4202	Adenocarcinoma	PTPRZ1	0.000041	-2.633858	
4203	Adenocarcinoma	MALL	0.00003	-2.631392	
4204	Adenocarcinoma	KRT24	0.000012	-2.625213	
4205	Adenocarcinoma	SOS2	0.0023	-2.623539	
4206	Adenocarcinoma	TMEM25	0.007064	-2.614747	
4207	Adenocarcinoma	C4orf19	0.000122	-2.613077	
4208	Adenocarcinoma	CLU	0.000096	-2.607711	
4209	Adenocarcinoma	CORO2B	0.000078	-2.602121	
4210	Adenocarcinoma	SSPN	0	-2.600631	
4211	Adenocarcinoma	BCAS3	0.006133	-2.599382	
4212	Adenocarcinoma	FOXA1	0.000292	-2.593877	
4213	Adenocarcinoma	STOX2	0.000242	-2.591556	
4214	Adenocarcinoma	SCNN1B	0	-2.586467	
4215	Adenocarcinoma	GYLTL1B	0.009026	-2.582526	
4216	Adenocarcinoma	BMP2	0.000162	-2.579571	
4217	Adenocarcinoma	FGD1	0.009411	-2.575283	
4218	Adenocarcinoma	GPA33	0.000186	-2.573758	
4219	Adenocarcinoma	PECI	0.000276	-2.573106	
4220	Adenocarcinoma	CD36	0.000013	-2.571099	
4221	Adenocarcinoma	SMEK2	0.000543	-2.568301	
4222	Adenocarcinoma	WDR78	0.00079	-2.565834	
4223	Adenocarcinoma	C2orf72	0.000037	-2.559506	
4224	Adenocarcinoma	L3MBTL3	0.010271	-2.554239	
4225	Adenocarcinoma	SFRP1	0.000885	-2.554028	
4226	Adenocarcinoma	JAG1	0.031742	-2.55137	
4227	Adenocarcinoma	MUM1L1	0.000125	-2.545621	
4228	Adenocarcinoma	TCEA3	0.000297	-2.544402	
4229	Adenocarcinoma	LOC286467	0.013368	-2.533699	
4230	Adenocarcinoma	CDC14A	0.000013	-2.52973	
4231	Adenocarcinoma	DST	0.002601	-2.519345	
4232	Adenocarcinoma	PDE9A	0.000063	-2.516274	
4233	Adenocarcinoma	SULT1A3	0.000217	-2.511955	
4234	Adenocarcinoma	SLC35A1	0.017529	-2.507512	
4235	Adenocarcinoma	MAF	0	-2.491759	
4236	Adenocarcinoma	DCAF8	0.000282	-2.488817	
4237	Adenocarcinoma	PDAP1	0	-2.486359	
4238	Adenocarcinoma	P2RY1	0.000279	-2.484963	
4239	Adenocarcinoma	POLR3G	0.003399	-2.482919	
4240	Adenocarcinoma	RETSAT	0.000202	-2.47862	
4241	Adenocarcinoma	PGDS	0	-2.473337	
4242	Adenocarcinoma	LPAR1	0.000066	-2.468301	
4243	Adenocarcinoma	BMP2K	0.000903	-2.464008	
4244	Adenocarcinoma	RHOF	0.000258	-2.461934	
4245	Adenocarcinoma	AVIL	0.000001	-2.461099	
4246	Adenocarcinoma	HADH	0.000053	-2.460606	
4247	Adenocarcinoma	A1CF	0.000028	-2.459517	
4248	Adenocarcinoma	COMMD1	0.029247	-2.454604	

4249	Adenocarcinoma	TNNT1	0.009531	-2.453879	
4250	Adenocarcinoma	PDE4DIP	0.000528	-2.449105	
4251	Adenocarcinoma	C1orf161	0.000928	-2.442114	
4252	Adenocarcinoma	LOC219347	0.000163	-2.439474	
4253	Adenocarcinoma	PRIMA1	0.000018	-2.435454	
4254	Adenocarcinoma	ASAP3	0.000135	-2.432569	
4255	Adenocarcinoma	KIAA0415	0.000053	-2.431677	
4256	Adenocarcinoma	ZNF781	0.019188	-2.431412	
4257	Adenocarcinoma	ELOVL6	0.000034	-2.429614	
4258	Adenocarcinoma	LANCL3	0.000299	-2.429492	
4259	Adenocarcinoma	DPY19L1	0.022989	-2.429196	
4260	Adenocarcinoma	CDK3	0.000077	-2.425672	
4261	Adenocarcinoma	PTP4A1	0.000012	-2.42263	
4262	Adenocarcinoma	SLC36A1	0.000061	-2.421944	
4263	Adenocarcinoma	SAMD13	0.000173	-2.421725	
4264	Adenocarcinoma	MACROD1	0.001816	-2.420346	
4265	Adenocarcinoma	FA2H	0.000221	-2.420133	
4266	Adenocarcinoma	TDP2	0.000009	-2.417162	
4267	Adenocarcinoma	LTBP4	0.00135	-2.414385	
4268	Adenocarcinoma	CNNM2	0.000885	-2.413334	
4269	Adenocarcinoma	C17orf76	0.000252	-2.408479	
4270	Adenocarcinoma	GPR44	0.000131	-2.40323	
4271	Adenocarcinoma	GNA11	0.000116	-2.400654	
4272	Adenocarcinoma	PGM1	0.000017	-2.39987	
4273	Adenocarcinoma	ENDOD1	0	-2.391819	
4274	Adenocarcinoma	GATM	0.000842	-2.391762	
4275	Adenocarcinoma	CNNM4	0.000074	-2.390598	
4276	Adenocarcinoma	EIF4E3	0.000052	-2.389208	
4277	Adenocarcinoma	SDCBP2	0.000135	-2.388524	
4278	Adenocarcinoma	PLIN1	0.000566	-2.387082	
4279	Adenocarcinoma	SLC22A5	0.000006	-2.383758	
4280	Adenocarcinoma	NAAA	0.000629	-2.377968	
4281	Adenocarcinoma	LOC100289632	0.000206	-2.377517	
4282	Adenocarcinoma	LOC100505483	0.000238	-2.372839	
4283	Adenocarcinoma	ZFP3	0.000004	-2.365486	
4284	Adenocarcinoma	LOC100292909	0.000445	-2.365269	
4285	Adenocarcinoma	MTM1	0.000299	-2.356838	
4286	Adenocarcinoma	C17orf45	0.002196	-2.355972	
4287	Adenocarcinoma	ZNF280C	0.005439	-2.353348	
4288	Adenocarcinoma	USP22	0.000098	-2.348289	
4289	Adenocarcinoma	ACADS	0.000036	-2.347116	
4290	Adenocarcinoma	PLCD3	0.000143	-2.342746	
4291	Adenocarcinoma	SATB2	0.000228	-2.342286	
4292	Adenocarcinoma	GLTP	0.000012	-2.341238	
4293	Adenocarcinoma	CLMN	0.000297	-2.341175	
4294	Adenocarcinoma	SHROOM3	0.000168	-2.337508	
4295	Adenocarcinoma	SMN1	0.000037	-2.337197	
4296	Adenocarcinoma	NQO1	0.045197	-2.337024	
4297	Adenocarcinoma	NT5DC1	0.002429	-2.331847	
4298	Adenocarcinoma	L1CAM	0.009183	-2.330945	

4299	Adenocarcinoma	CPT2	0.000047	-2.326475	
4300	Adenocarcinoma	SEZ6L2	0.008437	-2.324393	
4301	Adenocarcinoma	STMN3	0.017987	-2.322412	
4302	Adenocarcinoma	LGALS2	0	-2.3223	
4303	Adenocarcinoma	PAQR3	0.000101	-2.321024	
4304	Adenocarcinoma	APPL2	0.000084	-2.313575	
4305	Adenocarcinoma	ALDH6A1	0.000229	-2.310912	
4306	Adenocarcinoma	LETM1	0.000028	-2.307655	
4307	Adenocarcinoma	SLC44A1	0.000107	-2.300155	
4308	Adenocarcinoma	KLF8	0.000744	-2.298547	
4309	Adenocarcinoma	IDH3A	0.000023	-2.296197	
4310	Adenocarcinoma	PSMG4	0.000001	-2.294456	
4311	Adenocarcinoma	KBTBD11	0.000046	-2.294318	
4312	Adenocarcinoma	CYP1A1	0.000543	-2.287438	
4313	Adenocarcinoma	LRMP	0.00005	-2.283572	
4314	Adenocarcinoma	SIP1	0.001898	-2.282944	
4315	Adenocarcinoma	GLIPR2	0.000116	-2.281672	
4316	Adenocarcinoma	SLC35D1	0.000296	-2.279274	
4317	Adenocarcinoma	RAVER2	0.000005	-2.277227	
4318	Adenocarcinoma	PTGS1	0	-2.277219	
4319	Adenocarcinoma	CMAH	0	-2.27504	
4320	Adenocarcinoma	LOC285016	0	-2.270859	
4321	Adenocarcinoma	PRRT3	0.031742	-2.269879	
4322	Adenocarcinoma	BCL2L15	0.000028	-2.268541	
4323	Adenocarcinoma	MAPT	0.000094	-2.264895	
4324	Adenocarcinoma	MOCS1	0.000648	-2.261519	
4325	Adenocarcinoma	KRT19P2	0.000135	-2.260004	
4326	Adenocarcinoma	PRDX6	0.000048	-2.2515	
4327	Adenocarcinoma	CCNB1IP1	0.00089	-2.251411	
4328	Adenocarcinoma	C12orf35	0.012926	-2.251069	
4329	Adenocarcinoma	SHD	0.000098	-2.248522	
4330	Adenocarcinoma	VGF	0.002771	-2.246975	
4331	Adenocarcinoma	FAM107A	0.000228	-2.246606	
4332	Adenocarcinoma	FLNB	0.000297	-2.244974	
4333	Adenocarcinoma	PEX26	0.000241	-2.233614	
4334	Adenocarcinoma	ATP10A	0.000191	-2.232913	
4335	Adenocarcinoma	ADH1A	0.000744	-2.228283	
4336	Adenocarcinoma	C6orf192	0.000017	-2.2255	
4337	Adenocarcinoma	NGEF	0.012939	-2.22528	
4338	Adenocarcinoma	FAM149A	0.000141	-2.224178	
4339	Adenocarcinoma	HES6	0.005871	-2.223512	
4340	Adenocarcinoma	EPM2A	0.00732	-2.22277	
4341	Adenocarcinoma	ASCC3	0.00228	-2.221894	
4342	Adenocarcinoma	DLEU2	0.005991	-2.219421	
4343	Adenocarcinoma	C6orf136	0.000023	-2.216746	
4344	Adenocarcinoma	ANO10	0.000053	-2.207056	
4345	Adenocarcinoma	FZD5	0.000266	-2.202912	
4346	Adenocarcinoma	NPC1	0.005498	-2.202911	
4347	Adenocarcinoma	GALM	0.000162	-2.201462	
4348	Adenocarcinoma	SRGAP2P1	0.040502	-2.198353	

4349	Adenocarcinoma	SYNE1	0.004655	-2.198253	
4350	Adenocarcinoma	LIMA1	0.000009	-2.19754	
4351	Adenocarcinoma	DAPK2	0.000157	-2.196591	
4352	Adenocarcinoma	KATNA1	0.000199	-2.194489	
4353	Adenocarcinoma	GPR34	0	-2.186546	
4354	Adenocarcinoma	OSBPL7	0.000066	-2.183997	
4355	Adenocarcinoma	FKBP5	0.000164	-2.181115	
4356	Adenocarcinoma	AKAP9	0	-2.17955	
4357	Adenocarcinoma	ZNF235	0.010992	-2.173037	
4358	Adenocarcinoma	MIER3	0.000032	-2.171328	
4359	Adenocarcinoma	LRRC56	0.016442	-2.171161	
4360	Adenocarcinoma	CUL4A	0.003765	-2.169395	
4361	Adenocarcinoma	GBA2	0.000285	-2.169336	
4362	Adenocarcinoma	ZDHHC14	0.001595	-2.159739	
4363	Adenocarcinoma	CDS1	0.000028	-2.155889	
4364	Adenocarcinoma	MXD1	0.000104	-2.154847	
4365	Adenocarcinoma	MYO1D	0.000211	-2.153935	
4366	Adenocarcinoma	SLAMF7	0	-2.153695	
4367	Adenocarcinoma	LTA4H	0.002101	-2.153022	
4368	Adenocarcinoma	C9orf125	0.000112	-2.151976	
4369	Adenocarcinoma	OSBPL1A	0.000137	-2.149565	
4370	Adenocarcinoma	PPIL3	0.001382	-2.146776	
4371	Adenocarcinoma	ATOH8	0.000883	-2.140852	
4372	Adenocarcinoma	MGC13053	0.030623	-2.132961	
4373	Adenocarcinoma	UQCRC1	0.000063	-2.132919	
4374	Adenocarcinoma	STAP2	0.000189	-2.132629	
4375	Adenocarcinoma	NPY	0.000112	-2.125373	
4376	Adenocarcinoma	GOLGA2B	0.000001	-2.124954	
4377	Adenocarcinoma	PAQR8	0.000098	-2.124901	
4378	Adenocarcinoma	LQK1	0.016981	-2.123736	
4379	Adenocarcinoma	C14orf159	0.000015	-2.122629	
4380	Adenocarcinoma	IQCH	0.036289	-2.122224	
4381	Adenocarcinoma	TST	0.000007	-2.12104	
4382	Adenocarcinoma	ABCA5	0	-2.119836	
4383	Adenocarcinoma	PLD1	0.000135	-2.115702	
4384	Adenocarcinoma	GLOD5	0.000271	-2.113893	
4385	Adenocarcinoma	MRPL35	0.000297	-2.11346	
4386	Adenocarcinoma	LOC440173	0.005212	-2.113276	
4387	Adenocarcinoma	SRI	0.000263	-2.112696	
4388	Adenocarcinoma	NHSL1	0.012222	-2.106623	
4389	Adenocarcinoma	RBKS	0.02468	-2.104914	
4390	Adenocarcinoma	ICOSLG	0.010816	-2.101714	
4391	Adenocarcinoma	GLCCI1	0.000282	-2.101006	
4392	Adenocarcinoma	PCDHA6	0.000109	-2.10056	
4393	Adenocarcinoma	ZNF512B	0.000971	-2.099994	
4394	Adenocarcinoma	SULT1A4	0.041846	-2.099947	
4395	Adenocarcinoma	PDSS2	0.000052	-2.099445	
4396	Adenocarcinoma	PPARGC1A	0.000582	-2.097672	
4397	Adenocarcinoma	BCL2	0.000006	-2.095351	
4398	Adenocarcinoma	CBX7	0	-2.092974	

4399	Adenocarcinoma	CASD1	0.00029	-2.091419	
4400	Adenocarcinoma	FLNC	0.004847	-2.088416	
4401	Adenocarcinoma	CHODL	0	-2.088244	
4402	Adenocarcinoma	CYP2E1	0.014868	-2.085049	
4403	Adenocarcinoma	CDC14B	0.012127	-2.084686	
4404	Adenocarcinoma	GREB1	0.01461	-2.083184	
4405	Adenocarcinoma	FAM57A	0.000002	-2.081282	
4406	Adenocarcinoma	SLCO2A1	0	-2.079539	
4407	Adenocarcinoma	IFRD1	0.008114	-2.074017	
4408	Adenocarcinoma	PKN1	0.011259	-2.070597	
4409	Adenocarcinoma	CALM1	0.00025	-2.069654	
4410	Adenocarcinoma	GDPD5	0.016985	-2.067767	
4411	Adenocarcinoma	TP53I3	0.000116	-2.067676	
4412	Adenocarcinoma	ATG4A	0.000012	-2.067547	
4413	Adenocarcinoma	ACAT1	0.000077	-2.066848	
4414	Adenocarcinoma	FRYL	0	-2.063168	
4415	Adenocarcinoma	SHPK	0.000083	-2.058742	
4416	Adenocarcinoma	TSC22D3	0	-2.056851	
4417	Adenocarcinoma	UGP2	0.00004	-2.056388	
4418	Adenocarcinoma	ASNS	0.014034	-2.055824	
4419	Adenocarcinoma	SCGB2A1	0	-2.055265	
4420	Adenocarcinoma	PARVB	0.005278	-2.054067	
4421	Adenocarcinoma	BCAR3	0.00004	-2.053906	
4422	Adenocarcinoma	LRRC3B	0.000693	-2.049939	
4423	Adenocarcinoma	A2LD1	0.008161	-2.049601	
4424	Adenocarcinoma	ACSF2	0.000282	-2.048502	
4425	Adenocarcinoma	BRP44L	0.000027	-2.046297	
4426	Adenocarcinoma	TMED8	0.042885	-2.045735	
4427	Adenocarcinoma	ENY2	0.004086	-2.045432	
4428	Adenocarcinoma	DLL1	0	-2.044593	
4429	Adenocarcinoma	CMAS	0.000052	-2.042658	
4430	Adenocarcinoma	ABHD3	0.000238	-2.042615	
4431	Adenocarcinoma	S100A14	0.000183	-2.042127	
4432	Adenocarcinoma	MTRF1L	0.002296	-2.041783	
4433	Adenocarcinoma	SLC26A6	0.003129	-2.041744	
4434	Adenocarcinoma	HIBCH	0.000127	-2.041443	
4435	Adenocarcinoma	EIF1B	0.000508	-2.038089	
4436	Adenocarcinoma	VWA5B2	0.018618	-2.036685	
4437	Adenocarcinoma	ST7L	0	-2.034764	
4438	Adenocarcinoma	C10orf11	0.011491	-2.032364	
4439	Adenocarcinoma	CAPN5	0.000138	-2.031624	
4440	Adenocarcinoma	TRPM4	0.000312	-2.029588	
4441	Adenocarcinoma	STRADB	0.005989	-2.02463	
4442	Adenocarcinoma	DACT2	0.000588	-2.023093	
4443	Adenocarcinoma	AIFM3	0.000415	-2.023087	
4444	Adenocarcinoma	SMU1	0	-2.016487	
4445	Adenocarcinoma	SSBP2	0.000699	-2.016057	
4446	Adenocarcinoma	MTUS1	0.000071	-2.012135	
4447	Adenocarcinoma	LRRFIP2	0.000067	-2.011938	
4448	Adenocarcinoma	VSIG2	0	-2.008233	

4449	Adenocarcinoma	MFAP4	0	-2.007624	
4450	Adenocarcinoma	IMPDH2	0.004059	-2.005733	
4451	Adenocarcinoma	ITGA7	0.012048	-2.005136	
4452	Adenocarcinoma	STAG3	0.000114	-2.003641	
4453	Adenocarcinoma	KLHL34	0.039552	-2.003225	
4454	Adenocarcinoma	BDH1	0.00027	-2.003198	
4455	Adenocarcinoma	ROPN1	0.000748	-2.002696	
4456	Adenocarcinoma	MLLT4	0.000011	2.000196	
4457	Adenocarcinoma	TARBP1	0.018222	2.002354	
4458	Adenocarcinoma	AGFG2	0.001534	2.002586	
4459	Adenocarcinoma	CCNE2	0.012386	2.00458	
4460	Adenocarcinoma	PLEKHF2	0.01482	2.005755	
4461	Adenocarcinoma	TMC6	0.007496	2.008402	
4462	Adenocarcinoma	KIAA0146	0.01651	2.008495	
4463	Adenocarcinoma	APLP2	0.000747	2.009413	
4464	Adenocarcinoma	MAP2K5	0.005543	2.010427	
4465	Adenocarcinoma	CABLES1	0.042929	2.010532	
4466	Adenocarcinoma	RNF10	0.011082	2.010613	
4467	Adenocarcinoma	TTC13	0.001583	2.013917	
4468	Adenocarcinoma	MPZL3	0.018949	2.016272	
4469	Adenocarcinoma	FLJ14186	0.020715	2.017305	
4470	Adenocarcinoma	LOC440233	0.011545	2.022371	
4471	Adenocarcinoma	CTSD	0.003264	2.022791	
4472	Adenocarcinoma	PEX12	0.009672	2.023821	
4473	Adenocarcinoma	PIGS	0.012617	2.023824	
4474	Adenocarcinoma	ZADH2	0.003732	2.025558	
4475	Adenocarcinoma	LIG3	0.005179	2.025952	
4476	Adenocarcinoma	CTDSPL2	0.003521	2.030729	
4477	Adenocarcinoma	SRSF4	0.001886	2.0322	
4478	Adenocarcinoma	HISPPD2A	0.002718	2.033578	
4479	Adenocarcinoma	ACAD9	0.002388	2.034113	
4480	Adenocarcinoma	CHCHD7	0.036078	2.034328	
4481	Adenocarcinoma	FGGY	0.000004	2.034343	
4482	Adenocarcinoma	SERPINB1	0.003339	2.035626	
4483	Adenocarcinoma	C20orf151	0.006762	2.04069	
4484	Adenocarcinoma	ZNF710	0.028376	2.042024	
4485	Adenocarcinoma	TDGF1	0.000062	2.047152	
4486	Adenocarcinoma	FBXL11	0.012526	2.048065	
4487	Adenocarcinoma	ABHD15	0.004987	2.050014	
4488	Adenocarcinoma	FAM49B	0.028217	2.05083	
4489	Adenocarcinoma	RXRA	0.002161	2.053207	
4490	Adenocarcinoma	TMLHE	0.006576	2.053379	
4491	Adenocarcinoma	PLA2G12B	0.00002	2.059122	
4492	Adenocarcinoma	CHRNA5	0.017729	2.059834	
4493	Adenocarcinoma	SLC7A1	0.000292	2.065309	
4494	Adenocarcinoma	RNF160	0.004704	2.065911	
4495	Adenocarcinoma	FURIN	0.000396	2.067994	
4496	Adenocarcinoma	FAM50A	0.035293	2.068622	
4497	Adenocarcinoma	FASTKD3	0.004758	2.069163	
4498	Adenocarcinoma	SLC44A2	0.00644	2.07083	

4499	Adenocarcinoma	ST14	0.007306	2.07137	
4500	Adenocarcinoma	ARV1	0.038233	2.072915	
4501	Adenocarcinoma	DAP	0.013659	2.082527	
4502	Adenocarcinoma	ABLIM1	0.01873	2.083449	
4503	Adenocarcinoma	SYPL1	0.026715	2.083636	
4504	Adenocarcinoma	CETN2	0.008965	2.085826	
4505	Adenocarcinoma	DKFZP434F142	0.020248	2.087216	
4506	Adenocarcinoma	LOC441124	0.025198	2.087968	
4507	Adenocarcinoma	C15orf57	0.028017	2.089913	
4508	Adenocarcinoma	SLC9A8	0.039552	2.0946	
4509	Adenocarcinoma	LOC404266	0.042984	2.097443	
4510	Adenocarcinoma	RAB3D	0.028797	2.104136	
4511	Adenocarcinoma	ZNF830	0.018226	2.105094	
4512	Adenocarcinoma	PHF15	0.002689	2.106723	
4513	Adenocarcinoma	IGHMBP2	0	2.107544	
4514	Adenocarcinoma	MRPL13	0.045197	2.109255	
4515	Adenocarcinoma	ATPBD4	0.039632	2.110409	
4516	Adenocarcinoma	MBD5	0.000286	2.110792	
4517	Adenocarcinoma	ATAD5	0.01731	2.112755	
4518	Adenocarcinoma	XDH	0.011897	2.113082	
4519	Adenocarcinoma	TPD52L2	0.000076	2.120266	
4520	Adenocarcinoma	WDR52	0.012397	2.122044	
4521	Adenocarcinoma	KIAA1370	0.00136	2.123193	
4522	Adenocarcinoma	C11orf80	0.000048	2.123685	
4523	Adenocarcinoma	ATAD2	0.03939	2.124891	
4524	Adenocarcinoma	GINS1	0.000928	2.126108	
4525	Adenocarcinoma	SNX12	0.025932	2.126917	
4526	Adenocarcinoma	BMP4	0	2.128916	
4527	Adenocarcinoma	FGFR4	0.000252	2.129458	
4528	Adenocarcinoma	PEX11A	0.007955	2.13103	
4529	Adenocarcinoma	CPEB3	0.014097	2.132011	
4530	Adenocarcinoma	CYP4F3	0.000099	2.13287	
4531	Adenocarcinoma	DPP8	0.018275	2.135528	
4532	Adenocarcinoma	SLC12A2	0.000235	2.139849	
4533	Adenocarcinoma	BRCA2	0.010457	2.141749	
4534	Adenocarcinoma	FAM111A	0.007069	2.156683	
4535	Adenocarcinoma	OPCML	0.010676	2.157732	
4536	Adenocarcinoma	ZBTB26	0.017602	2.15914	
4537	Adenocarcinoma	DMXL2	0.017847	2.159518	
4538	Adenocarcinoma	BCAP31	0.026776	2.161095	
4539	Adenocarcinoma	UCA1	0.000017	2.16163	
4540	Adenocarcinoma	IFI27L1	0.041846	2.171184	
4541	Adenocarcinoma	TRANK1	0.00427	2.178654	
4542	Adenocarcinoma	TDRD7	0.017108	2.182988	
4543	Adenocarcinoma	LOC399744	0.020248	2.193034	
4544	Adenocarcinoma	ACE2	0.000003	2.193072	
4545	Adenocarcinoma	RBL2	0.013185	2.193334	
4546	Adenocarcinoma	CD59	0.040114	2.197907	
4547	Adenocarcinoma	LOC401357	0.020715	2.199755	
4548	Adenocarcinoma	MAP7D2	0.000082	2.203802	

4549	Adenocarcinoma	KIAA1217	0.017918	2.205406	
4550	Adenocarcinoma	LGALS8	0.000526	2.205876	
4551	Adenocarcinoma	SPG11	0.006445	2.206429	
4552	Adenocarcinoma	HSD17B4	0.012331	2.209442	
4553	Adenocarcinoma	N4BP2L2	0.000223	2.213151	
4554	Adenocarcinoma	ZNF586	0.006751	2.217234	
4555	Adenocarcinoma	POMP	0.018322	2.219702	
4556	Adenocarcinoma	MORC4	0.017623	2.220515	
4557	Adenocarcinoma	OVOL1	0.014986	2.224552	
4558	Adenocarcinoma	RAI14	0.000238	2.235023	
4559	Adenocarcinoma	KCNAB2	0.014467	2.235303	
4560	Adenocarcinoma	IL9R	0.004811	2.23845	
4561	Adenocarcinoma	ZNF321	0.00555	2.240933	
4562	Adenocarcinoma	PIGU	0.007058	2.241053	
4563	Adenocarcinoma	ZNF37B	0.014132	2.245885	
4564	Adenocarcinoma	PDIA6	0.030623	2.24798	
4565	Adenocarcinoma	WBP4	0.002242	2.250237	
4566	Adenocarcinoma	TMEM206	0.000151	2.251382	
4567	Adenocarcinoma	EREG	0.000001	2.274809	
4568	Adenocarcinoma	CCDC144A	0.002615	2.282153	
4569	Adenocarcinoma	CYHR1	0.042984	2.282635	
4570	Adenocarcinoma	PPP1R3D	0.007247	2.284234	
4571	Adenocarcinoma	CYP2B6	0.000389	2.285989	
4572	Adenocarcinoma	FAM65B	0.005425	2.287983	
4573	Adenocarcinoma	WNT7B	0.010676	2.288709	
4574	Adenocarcinoma	PITX2	0.000652	2.304323	
4575	Adenocarcinoma	FAM110A	0.002196	2.30707	
4576	Adenocarcinoma	PIK3C2B	0.0016	2.314515	
4577	Adenocarcinoma	CPD	0.00152	2.315221	
4578	Adenocarcinoma	MYC	0.000043	2.32116	
4579	Adenocarcinoma	ROD1	0.011852	2.323082	
4580	Adenocarcinoma	CHMP4B	0.007819	2.329571	
4581	Adenocarcinoma	NCOA3	0.010373	2.330103	
4582	Adenocarcinoma	NAPRT1	0.00448	2.334459	
4583	Adenocarcinoma	KIAA0101	0.000736	2.338754	
4584	Adenocarcinoma	SNX19	0.015688	2.338817	
4585	Adenocarcinoma	CBFA2T2	0.003492	2.340377	
4586	Adenocarcinoma	C13orf27	0.000057	2.341832	
4587	Adenocarcinoma	DYNLL1	0.000048	2.343521	
4588	Adenocarcinoma	ZNF816A	0.014441	2.344824	
4589	Adenocarcinoma	DGAT2	0.002791	2.344958	
4590	Adenocarcinoma	TCF19	0.000751	2.347528	
4591	Adenocarcinoma	JAG2	0.000098	2.357991	
4592	Adenocarcinoma	C22orf29	0.011787	2.366756	
4593	Adenocarcinoma	SLC15A2	0.000136	2.368835	
4594	Adenocarcinoma	FLJ45445	0.020715	2.37061	
4595	Adenocarcinoma	ITPKB	0.005735	2.373689	
4596	Adenocarcinoma	FLJ45340	0.038464	2.377321	
4597	Adenocarcinoma	CMTM7	0.04009	2.379482	
4598	Adenocarcinoma	GCC2	0.013908	2.380315	

4599	Adenocarcinoma	RAPGEFL1	0.009411	2.382267	
4600	Adenocarcinoma	FXYD5	0.018523	2.391601	
4601	Adenocarcinoma	TNFRSF11B	0.000263	2.412846	
4602	Adenocarcinoma	IFITM3	0	2.427902	
4603	Adenocarcinoma	TDRKH	0.000822	2.431352	
4604	Adenocarcinoma	LINC00265	0.03724	2.437513	
4605	Adenocarcinoma	LOC254057	0.007004	2.439625	
4606	Adenocarcinoma	TOP1MT	0.000095	2.441191	
4607	Adenocarcinoma	TSPAN31	0.000926	2.442075	
4608	Adenocarcinoma	C9orf127	0.000268	2.449939	
4609	Adenocarcinoma	LOC153684	0.012193	2.456051	
4610	Adenocarcinoma	CBX2	0.000072	2.45772	
4611	Adenocarcinoma	ZNF148	0.036078	2.459233	
4612	Adenocarcinoma	TRAPPC6A	0.01862	2.471927	
4613	Adenocarcinoma	ELL3	0.002258	2.478822	
4614	Adenocarcinoma	ENTPD6	0.018279	2.494725	
4615	Adenocarcinoma	RAD21	0.026732	2.504876	
4616	Adenocarcinoma	C19orf40	0.014467	2.506698	
4617	Adenocarcinoma	AMIGO2	0.000027	2.510275	
4618	Adenocarcinoma	ATP2C2	0.01685	2.511174	
4619	Adenocarcinoma	IRAK1	0.03394	2.518028	
4620	Adenocarcinoma	ITGA2	0.001489	2.52092	
4621	Adenocarcinoma	DOPEY2	0.002288	2.524589	
4622	Adenocarcinoma	LOC388796	0.000056	2.525723	
4623	Adenocarcinoma	ASCL2	0.000003	2.526144	
4624	Adenocarcinoma	IFFO2	0.016092	2.532587	
4625	Adenocarcinoma	GTF2H1	0.004072	2.534975	
4626	Adenocarcinoma	UBE2D3	0.003021	2.554795	
4627	Adenocarcinoma	CCDC144B	0.018374	2.555585	
4628	Adenocarcinoma	PAN3	0.001597	2.557215	
4629	Adenocarcinoma	IFITM1	0	2.566659	
4630	Adenocarcinoma	SPTY2D1	0.003619	2.569926	
4631	Adenocarcinoma	SNX5	0.010029	2.577579	
4632	Adenocarcinoma	LOC283859	0.000002	2.593191	
4633	Adenocarcinoma	RND3	0.001702	2.605872	
4634	Adenocarcinoma	PPP1R3B	0.016459	2.606331	
4635	Adenocarcinoma	C7orf38	0.006848	2.612116	
4636	Adenocarcinoma	RGNEF	0.006426	2.615305	
4637	Adenocarcinoma	CYFIP1	0.000034	2.616223	
4638	Adenocarcinoma	AZGP1	0	2.626652	
4639	Adenocarcinoma	AKAP11	0.014979	2.628001	
4640	Adenocarcinoma	AFF1	0.005425	2.628107	
4641	Adenocarcinoma	PURA	0.003342	2.664896	
4642	Adenocarcinoma	COBLL1	0.016908	2.668886	
4643	Adenocarcinoma	PBX3	0.025527	2.669613	
4644	Adenocarcinoma	APOL2	0.01821	2.698816	
4645	Adenocarcinoma	CLIC3	0.000063	2.704234	
4646	Adenocarcinoma	TTC30B	0.006274	2.752626	
4647	Adenocarcinoma	TIMP1	0	2.756339	
4648	Adenocarcinoma	GPR143	0	2.760934	

4649	Adenocarcinoma	MUC4	0.012425	2.762498	
4650	Adenocarcinoma	UHMK1	0.002791	2.783461	
4651	Adenocarcinoma	FAM122C	0.003202	2.78738	
4652	Adenocarcinoma	GRM8	0	2.795039	
4653	Adenocarcinoma	ZC3H12A	0.00122	2.795748	
4654	Adenocarcinoma	TMED3	0.006285	2.798591	
4655	Adenocarcinoma	C11orf54	0.008977	2.808257	
4656	Adenocarcinoma	LCMT2	0.010346	2.822198	
4657	Adenocarcinoma	CCDC132	0.000536	2.831538	
4658	Adenocarcinoma	CATSPER2	0.018483	2.833397	
4659	Adenocarcinoma	SCCPDH	0.006988	2.849435	
4660	Adenocarcinoma	LOC100131257	0.012388	2.851215	
4661	Adenocarcinoma	TMEM62	0.027993	2.856251	
4662	Adenocarcinoma	FARP1	0.026674	2.881692	
4663	Adenocarcinoma	FAM71F2	0.011727	2.884242	
4664	Adenocarcinoma	COL1A1	0.000263	2.887807	
4665	Adenocarcinoma	TANK	0.023542	2.892938	
4666	Adenocarcinoma	ABCC2	0.000964	2.897253	
4667	Adenocarcinoma	ABCC6	0	2.910024	
4668	Adenocarcinoma	GRHL1	0.013773	2.937916	
4669	Adenocarcinoma	BBC3	0.027568	2.941099	
4670	Adenocarcinoma	SERINC5	0.021028	2.946837	
4671	Adenocarcinoma	RNF43	0.003329	2.958404	
4672	Adenocarcinoma	ENC1	0.009294	2.966357	
4673	Adenocarcinoma	PNPT1	0.014572	3.010962	
4674	Adenocarcinoma	ACSL6	0	3.021882	
4675	Adenocarcinoma	PDK4	0.01686	3.077281	
4676	Adenocarcinoma	TATDN1	0.026409	3.102705	
4677	Adenocarcinoma	SMAD7	0.017651	3.151831	
4678	Adenocarcinoma	CXCL3	0	3.22057	
4679	Adenocarcinoma	CELP	0.000551	3.237431	
4680	Adenocarcinoma	FLJ37464	0.010554	3.330586	
4681	Adenocarcinoma	SPATA2	0.037223	3.39365	
4682	Adenocarcinoma	PTP4A3	0.000168	3.409801	
4683	Adenocarcinoma	PPM1H	0.000249	3.479492	
4684	Adenocarcinoma	SDR16C5	0.002619	3.493107	
4685	Adenocarcinoma	DTX3L	0.013671	3.496114	
4686	Adenocarcinoma	SLC27A2	0.008191	3.496874	
4687	Adenocarcinoma	THNSL2	0.006697	3.507126	
4688	Adenocarcinoma	CXCL1	0	3.562836	
4689	Adenocarcinoma	LBA1	0.005487	3.581658	
4690	Adenocarcinoma	PADI1	0.013528	3.590778	
4691	Adenocarcinoma	CAPG	0.024028	3.592218	
4692	Adenocarcinoma	SEMA4A	0.013238	3.598787	
4693	Adenocarcinoma	SNTB1	0.000297	3.60564	
4694	Adenocarcinoma	LY6G6D	0	3.652899	
4695	Adenocarcinoma	PVRL4	0.027295	3.701049	
4696	Adenocarcinoma	RELL1	0.002229	3.822744	
4697	Adenocarcinoma	ATPIF1	0.002194	3.844013	
4698	Adenocarcinoma	TGFBR3	0.00996	3.84745	

4699	Adenocarcinoma	STK39	0.020248	3.866313	
4700	Adenocarcinoma	ACOT4	0.02204	3.910514	
4701	Adenocarcinoma	AGT	0	3.928558	
4702	Adenocarcinoma	DGKH	0.001127	3.936771	
4703	Adenocarcinoma	LAMP3	0.015046	3.989819	
4704	Adenocarcinoma	CFB	0	4.05669	
4705	Adenocarcinoma	AIM1	0.010382	4.087678	
4706	Adenocarcinoma	ANKRD56	0.005986	4.091798	
4707	Adenocarcinoma	TRIB1	0.03724	4.19406	
4708	Adenocarcinoma	TNFSF15	0.004444	4.207166	
4709	Adenocarcinoma	NFKBIZ	0.000107	4.258509	
4710	Adenocarcinoma	NF1	0.002911	4.274836	
4711	Adenocarcinoma	CD14	0.004876	4.275405	
4712	Adenocarcinoma	SYTL2	0.000034	4.459447	
4713	Adenocarcinoma	AQP3	0.011612	4.731538	
4714	Adenocarcinoma	IGFL4	0.005425	4.858818	
4715	Adenocarcinoma	PHF11	0.007757	4.871831	
4716	Adenocarcinoma	PLA2R1	0.015492	4.90688	
4717	Adenocarcinoma	GNE	0.007974	5.119714	
4718	Adenocarcinoma	CRIM1	0.000358	5.229024	
4719	Adenocarcinoma	ZSCAN29	0.008183	5.250697	
4720	Adenocarcinoma	SERTAD4	0.020576	5.290643	
4721	Adenocarcinoma	C10orf4	0.003603	5.589526	
4722	Adenocarcinoma	TTC39B	0.001303	5.907317	
4723	Adenocarcinoma	CACNA1D	0.009493	6.168673	
4724	Adenocarcinoma	CTSH	0.001249	6.183643	
4725	Adenocarcinoma	ZNF776	0.015162	6.449528	
4726	Adenocarcinoma	TLE4	0.000979	6.654461	
4727	Adenocarcinoma	MGC10981	0.006523	7.241934	
4728	Adenocarcinoma	ARL4C	0.007496	7.491655	
4729	Adenocarcinoma	C9orf64	0.010313	7.497865	
4730	Adenocarcinoma	ARHGEF40	0.000006	8.995607	
4731	Adenocarcinoma	PROM2	0.001809	11.004361	
4732	Adenocarcinoma	CDH3	0.000012	12.544509	
4733	Adenocarcinoma	CLDN1	0.000089	12.600585	
4734	Adenocarcinoma	FBP1	0.001648	16.172329	
4735	Adenocarcinoma of colon	AKR1B10	0.00018	-7.408942	
4736	Adenocarcinoma of colon	ABCG2	0.000246	-6.568358	
4737	Adenocarcinoma of colon	DHRS9	0.000007	-6.14952	
4738	Adenocarcinoma of colon	PKIB	0.000009	-6.113732	
4739	Adenocarcinoma of colon	CHP2	0.000168	-5.791364	
4740	Adenocarcinoma of colon	IL1R2	0.000052	-5.493194	
4741	Adenocarcinoma of colon	GCNT2	0.000098	-4.728089	
4742	Adenocarcinoma of colon	ADH1B	0.000129	-4.601914	
4743	Adenocarcinoma of colon	SLC4A4	0.000152	-4.563736	
4744	Adenocarcinoma of colon	DHRS11	0.000021	-4.552529	
4745	Adenocarcinoma of colon	CWH43	0.000237	-4.42692	
4746	Adenocarcinoma of colon	HSD17B2	0.000052	-4.175568	
4747	Adenocarcinoma of colon	UGT2B15	0.000038	-4.136587	
4748	Adenocarcinoma of colon	PLAC8	0.000164	-4.104069	

4749	Adenocarcinoma of colon	AGPAT9	0.000063	-4.032915	
4750	Adenocarcinoma of colon	ARHGAP44	0.000252	-4.0241	
4751	Adenocarcinoma of colon	LRRC19	0.000069	-4.006808	
4752	Adenocarcinoma of colon	CA2	0.000238	-3.967964	
4753	Adenocarcinoma of colon	MOGAT2	0.000064	-3.948991	
4754	Adenocarcinoma of colon	UGT2B17	0.000072	-3.933187	
4755	Adenocarcinoma of colon	SCIN	0.000238	-3.855473	
4756	Adenocarcinoma of colon	TSPAN7	0.00024	-3.837825	
4757	Adenocarcinoma of colon	FAM55D	0.000219	-3.823774	
4758	Adenocarcinoma of colon	HPGD	0.000201	-3.819098	
4759	Adenocarcinoma of colon	PADI2	0.00016	-3.803735	
4760	Adenocarcinoma of colon	FXYD3	0.000061	-3.760548	
4761	Adenocarcinoma of colon	ABCA8	0.000244	-3.701992	
4762	Adenocarcinoma of colon	ARL14	0.000007	-3.700351	
4763	Adenocarcinoma of colon	CES2	0.000229	-3.655126	
4764	Adenocarcinoma of colon	TUBAL3	0.00023	-3.650291	
4765	Adenocarcinoma of colon	PCK1	0.000292	-3.635801	
4766	Adenocarcinoma of colon	C10orf99	0.000028	-3.620081	
4767	Adenocarcinoma of colon	MAOA	0.00002	-3.573062	
4768	Adenocarcinoma of colon	CES3	0.000071	-3.541904	
4769	Adenocarcinoma of colon	GPT	0.000045	-3.535019	
4770	Adenocarcinoma of colon	HIGD1A	0.000009	-3.524229	
4771	Adenocarcinoma of colon	SULT1B1	0.000027	-3.521881	
4772	Adenocarcinoma of colon	VILL	0.000078	-3.451077	
4773	Adenocarcinoma of colon	RDH5	0.000083	-3.450638	
4774	Adenocarcinoma of colon	SLC9A2	0.000098	-3.438543	
4775	Adenocarcinoma of colon	ZNF575	0.000035	-3.378636	
4776	Adenocarcinoma of colon	EYA2	0.000292	-3.360873	
4777	Adenocarcinoma of colon	CKMT1A	0.000012	-3.35529	
4778	Adenocarcinoma of colon	NR3C2	0.000074	-3.350237	
4779	Adenocarcinoma of colon	AHCYL2	0.000007	-3.330288	
4780	Adenocarcinoma of colon	GCNT3	0.000089	-3.318624	
4781	Adenocarcinoma of colon	C2orf88	0.000052	-3.296037	
4782	Adenocarcinoma of colon	HHLA2	0.000288	-3.269107	
4783	Adenocarcinoma of colon	ZBTB7C	0.000141	-3.266716	
4784	Adenocarcinoma of colon	FABP1	0.000007	-3.261659	
4785	Adenocarcinoma of colon	CA12	0.00004	-3.220574	
4786	Adenocarcinoma of colon	STMN2	0.00025	-3.185564	
4787	Adenocarcinoma of colon	ZZEF1	0.000261	-3.16765	
4788	Adenocarcinoma of colon	PLCE1	0.000012	-3.149416	
4789	Adenocarcinoma of colon	SCGN	0.000292	-3.132719	
4790	Adenocarcinoma of colon	LDHD	0.000027	-3.129231	
4791	Adenocarcinoma of colon	PAPSS2	0.000007	-3.090265	
4792	Adenocarcinoma of colon	CEACAM1	0.000043	-3.080438	
4793	Adenocarcinoma of colon	METTL7A	0.000069	-3.051391	
4794	Adenocarcinoma of colon	KLF4	0.000031	-3.042323	
4795	Adenocarcinoma of colon	KIF16B	0.000012	-3.030102	
4796	Adenocarcinoma of colon	CCDC68	0.000028	-3.028445	
4797	Adenocarcinoma of colon	SULT1A2	0.000079	-2.995262	
4798	Adenocarcinoma of colon	TTC23	0.000018	-2.979335	

4799	Adenocarcinoma of colon	FAM46C	0.000015	-2.977186	
4800	Adenocarcinoma of colon	DENND2A	0.000061	-2.97707	
4801	Adenocarcinoma of colon	TSPAN1	0.000226	-2.971613	
4802	Adenocarcinoma of colon	SULT1A1	0.000206	-2.925544	
4803	Adenocarcinoma of colon	ETHE1	0.000003	-2.915623	
4804	Adenocarcinoma of colon	TMEM171	0.000043	-2.885524	
4805	Adenocarcinoma of colon	PRKAR2B	0.000238	-2.861024	
4806	Adenocarcinoma of colon	KRT20	0.000255	-2.836996	
4807	Adenocarcinoma of colon	CLDN23	0.0002	-2.834475	
4808	Adenocarcinoma of colon	LOC400573	0.000123	-2.834344	
4809	Adenocarcinoma of colon	SLC1A7	0.000015	-2.805395	
4810	Adenocarcinoma of colon	MMP28	0.000034	-2.79525	
4811	Adenocarcinoma of colon	SGK1	0.000007	-2.786882	
4812	Adenocarcinoma of colon	DSC2	0.000053	-2.779295	
4813	Adenocarcinoma of colon	PPID	0.000083	-2.778977	
4814	Adenocarcinoma of colon	PPP2R3A	0.000074	-2.772831	
4815	Adenocarcinoma of colon	ETFDH	0.000023	-2.757237	
4816	Adenocarcinoma of colon	TMEM56	0.000041	-2.746816	
4817	Adenocarcinoma of colon	GPD1L	0.000012	-2.724242	
4818	Adenocarcinoma of colon	C4orf34	0.000284	-2.70772	
4819	Adenocarcinoma of colon	SQRDL	0.000007	-2.686297	
4820	Adenocarcinoma of colon	RBM47	0.00004	-2.686042	
4821	Adenocarcinoma of colon	MYO1A	0.000243	-2.673352	
4822	Adenocarcinoma of colon	SRPX	0.00029	-2.663967	
4823	Adenocarcinoma of colon	FMO5	0.000299	-2.660514	
4824	Adenocarcinoma of colon	NR5A2	0.0003	-2.636983	
4825	Adenocarcinoma of colon	USP2	0.000085	-2.636411	
4826	Adenocarcinoma of colon	MALL	0.00003	-2.631392	
4827	Adenocarcinoma of colon	KRT24	0.000012	-2.625213	
4828	Adenocarcinoma of colon	CPM	0.000051	-2.621781	
4829	Adenocarcinoma of colon	C4orf19	0.000122	-2.613077	
4830	Adenocarcinoma of colon	FOXA1	0.000292	-2.593877	
4831	Adenocarcinoma of colon	BMP2	0.000162	-2.579571	
4832	Adenocarcinoma of colon	UGDH	0.000029	-2.5748	
4833	Adenocarcinoma of colon	GPA33	0.000186	-2.573758	
4834	Adenocarcinoma of colon	PECI	0.000276	-2.573106	
4835	Adenocarcinoma of colon	C2orf72	0.000037	-2.559506	
4836	Adenocarcinoma of colon	TCEA3	0.000297	-2.544402	
4837	Adenocarcinoma of colon	SULT1A3	0.000217	-2.511955	
4838	Adenocarcinoma of colon	RETSAT	0.000202	-2.47862	
4839	Adenocarcinoma of colon	MYOT	0.00017	-2.475259	
4840	Adenocarcinoma of colon	LPAR1	0.000066	-2.468301	
4841	Adenocarcinoma of colon	RHOF	0.000258	-2.461934	
4842	Adenocarcinoma of colon	HADH	0.000053	-2.460606	
4843	Adenocarcinoma of colon	A1CF	0.000028	-2.459517	
4844	Adenocarcinoma of colon	ASAP3	0.000135	-2.432569	
4845	Adenocarcinoma of colon	KIAA0415	0.000053	-2.431677	
4846	Adenocarcinoma of colon	ELOVL6	0.000034	-2.429614	
4847	Adenocarcinoma of colon	FLJ32063	0.000136	-2.428407	
4848	Adenocarcinoma of colon	PTP4A1	0.000012	-2.42263	

4849	Adenocarcinoma of colon	GOLM1	0.000252	-2.421836	
4850	Adenocarcinoma of colon	SAMD13	0.000173	-2.421725	
4851	Adenocarcinoma of colon	FA2H	0.000221	-2.420133	
4852	Adenocarcinoma of colon	TDP2	0.000009	-2.417162	
4853	Adenocarcinoma of colon	C17orf76	0.000252	-2.408479	
4854	Adenocarcinoma of colon	GNA11	0.000116	-2.400654	
4855	Adenocarcinoma of colon	PGM1	0.000017	-2.39987	
4856	Adenocarcinoma of colon	SPPL2A	0.000009	-2.398902	
4857	Adenocarcinoma of colon	CNNM4	0.000074	-2.390598	
4858	Adenocarcinoma of colon	EIF4E3	0.000052	-2.389208	
4859	Adenocarcinoma of colon	SDCBP2	0.000135	-2.388524	
4860	Adenocarcinoma of colon	FAM47E	0.000256	-2.385583	
4861	Adenocarcinoma of colon	LOC100289632	0.000206	-2.377517	
4862	Adenocarcinoma of colon	LOC100287411	0.00014	-2.377259	
4863	Adenocarcinoma of colon	LOC100505483	0.000238	-2.372839	
4864	Adenocarcinoma of colon	MTM1	0.000299	-2.356838	
4865	Adenocarcinoma of colon	USP22	0.000098	-2.348289	
4866	Adenocarcinoma of colon	RUNDC3B	0.000168	-2.344697	
4867	Adenocarcinoma of colon	PLCD3	0.000143	-2.342746	
4868	Adenocarcinoma of colon	GLTP	0.000012	-2.341238	
4869	Adenocarcinoma of colon	CLMN	0.000297	-2.341175	
4870	Adenocarcinoma of colon	SHROOM3	0.000168	-2.337508	
4871	Adenocarcinoma of colon	CPT2	0.000047	-2.326475	
4872	Adenocarcinoma of colon	APPL2	0.000084	-2.313575	
4873	Adenocarcinoma of colon	ALDH6A1	0.000229	-2.310912	
4874	Adenocarcinoma of colon	LETM1	0.000028	-2.307655	
4875	Adenocarcinoma of colon	SLC44A1	0.000107	-2.300155	
4876	Adenocarcinoma of colon	IDH3A	0.000023	-2.296197	
4877	Adenocarcinoma of colon	KBTBD11	0.000046	-2.294318	
4878	Adenocarcinoma of colon	GLIPR2	0.000116	-2.281672	
4879	Adenocarcinoma of colon	SLC35D1	0.000296	-2.279274	
4880	Adenocarcinoma of colon	KRT19P2	0.000135	-2.260004	
4881	Adenocarcinoma of colon	PRDX6	0.000048	-2.2515	
4882	Adenocarcinoma of colon	SHD	0.000098	-2.248522	
4883	Adenocarcinoma of colon	FAM107A	0.000228	-2.246606	
4884	Adenocarcinoma of colon	FLNB	0.000297	-2.244974	
4885	Adenocarcinoma of colon	PEX26	0.000241	-2.233614	
4886	Adenocarcinoma of colon	FAM149A	0.000141	-2.224178	
4887	Adenocarcinoma of colon	C6orf136	0.000023	-2.216746	
4888	Adenocarcinoma of colon	ANO10	0.000053	-2.207056	
4889	Adenocarcinoma of colon	FLJ36848	0.000256	-2.20681	
4890	Adenocarcinoma of colon	FZD5	0.000266	-2.202912	
4891	Adenocarcinoma of colon	GALM	0.000162	-2.201462	
4892	Adenocarcinoma of colon	LIMA1	0.000009	-2.19754	
4893	Adenocarcinoma of colon	OSBPL7	0.000066	-2.183997	
4894	Adenocarcinoma of colon	FKBP5	0.000164	-2.181115	
4895	Adenocarcinoma of colon	GBA2	0.000285	-2.169336	
4896	Adenocarcinoma of colon	CDS1	0.000028	-2.155889	
4897	Adenocarcinoma of colon	MXD1	0.000104	-2.154847	
4898	Adenocarcinoma of colon	MYO1D	0.000211	-2.153935	

4899	Adenocarcinoma of colon	C9orf125	0.000112	-2.151976	
4900	Adenocarcinoma of colon	UQCRC1	0.000063	-2.132919	
4901	Adenocarcinoma of colon	STAP2	0.000189	-2.132629	
4902	Adenocarcinoma of colon	PAQR8	0.000098	-2.124901	
4903	Adenocarcinoma of colon	C14orf159	0.000015	-2.122629	
4904	Adenocarcinoma of colon	TST	0.000007	-2.12104	
4905	Adenocarcinoma of colon	PLD1	0.000135	-2.115702	
4906	Adenocarcinoma of colon	GLOD5	0.000271	-2.113893	
4907	Adenocarcinoma of colon	MRPL35	0.000297	-2.11346	
4908	Adenocarcinoma of colon	ATP5S	0.000026	-2.103186	
4909	Adenocarcinoma of colon	GLCCI1	0.000282	-2.101006	
4910	Adenocarcinoma of colon	PCDHA6	0.000109	-2.10056	
4911	Adenocarcinoma of colon	PDSS2	0.000052	-2.099445	
4912	Adenocarcinoma of colon	CASD1	0.00029	-2.091419	
4913	Adenocarcinoma of colon	CALM1	0.00025	-2.069654	
4914	Adenocarcinoma of colon	TP53I3	0.000116	-2.067676	
4915	Adenocarcinoma of colon	ATG4A	0.000012	-2.067547	
4916	Adenocarcinoma of colon	ACAT1	0.000077	-2.066848	
4917	Adenocarcinoma of colon	SHPK	0.000083	-2.058742	
4918	Adenocarcinoma of colon	UGP2	0.00004	-2.056388	
4919	Adenocarcinoma of colon	FXYD1	0.000047	-2.055213	
4920	Adenocarcinoma of colon	BCAR3	0.00004	-2.053906	
4921	Adenocarcinoma of colon	BRP44L	0.000027	-2.046297	
4922	Adenocarcinoma of colon	CMAS	0.000052	-2.042658	
4923	Adenocarcinoma of colon	ABHD3	0.000238	-2.042615	
4924	Adenocarcinoma of colon	S100A14	0.000183	-2.042127	
4925	Adenocarcinoma of colon	HIBCH	0.000127	-2.041443	
4926	Adenocarcinoma of colon	CAPN5	0.000138	-2.031624	
4927	Adenocarcinoma of colon	MTUS1	0.000071	-2.012135	
4928	Adenocarcinoma of colon	LRRFIP2	0.000067	-2.011938	
4929	Adenocarcinoma of colon	SGK2	0.000043	-2.008301	
4930	Adenocarcinoma of colon	BDH1	0.00027	-2.003198	
4931	Adenocarcinoma of colon	LOC100289019	0.000053	-2.00295	
4932	Adenocarcinoma of colon	GRHL2	0	2.061635	
4933	Adenocarcinoma of colon	TPD52L2	0.000076	2.120266	
4934	Adenocarcinoma of colon	SLC12A2	0.000235	2.139849	
4935	Adenocarcinoma of colon	RAI14	0.000238	2.235023	
4936	Adenocarcinoma of colon	FOXO1	0	2.245879	
4937	Adenocarcinoma of colon	TMEM206	0.000151	2.251382	
4938	Adenocarcinoma of colon	MYC	0.000043	2.32116	
4939	Adenocarcinoma of colon	C13orf27	0.000057	2.341832	
4940	Adenocarcinoma of colon	JAG2	0.000098	2.357991	
4941	Adenocarcinoma of colon	TNFRSF11B	0.000263	2.412846	
4942	Adenocarcinoma of colon	TOP1MT	0.000095	2.441191	
4943	Adenocarcinoma of colon	CBX2	0.000072	2.45772	
4944	Adenocarcinoma of colon	AMIGO2	0.000027	2.510275	
4945	Adenocarcinoma of colon	GPR126	0.002669	2.512988	
4946	Adenocarcinoma of colon	LOC388796	0.000056	2.525723	
4947	Adenocarcinoma of colon	CLIC3	0.000063	2.704234	
4948	Adenocarcinoma of colon	COL18A1	0.000076	2.847955	

4949	Adenocarcinoma of colon	COL1A1	0.000263	2.887807	
4950	Adenocarcinoma of colon	PTP4A3	0.000168	3.409801	
4951	Adenocarcinoma of colon	PPM1H	0.000249	3.479492	
4952	Adenocarcinoma of colon	SNTB1	0.000297	3.60564	
4953	Adenocarcinoma of colon	CDH3	0.000012	12.544509	
4954	Adenocarcinoma of colon	CLDN1	0.000089	12.600585	
4955	Adenocarcinoma of large intestine	OSTalpha	0.000026	-47.033402	
4956	Adenocarcinoma of large intestine	MMP7	0	-18.824692	
4957	Adenocarcinoma of large intestine	SLC30A10	0.000006	-16.146988	
4958	Adenocarcinoma of large intestine	FOXQ1	0	-15.928664	
4959	Adenocarcinoma of large intestine	KIAA1199	0	-13.538706	
4960	Adenocarcinoma of large intestine	IL8	0	-12.714234	
4961	Adenocarcinoma of large intestine	CLDN1	0	-10.740069	
4962	Adenocarcinoma of large intestine	VSTM2A	0	-9.14717	
4963	Adenocarcinoma of large intestine	ABCG2	0.000006	-8.486486	
4964	Adenocarcinoma of large intestine	NR1H4	0.000846	-8.412746	
4965	Adenocarcinoma of large intestine	NPY6R	0	-8.003144	
4966	Adenocarcinoma of large intestine	CHP2	0	-7.890496	
4967	Adenocarcinoma of large intestine	CCL23	0.000022	-7.094239	
4968	Adenocarcinoma of large intestine	COL11A1	0	-7.083734	
4969	Adenocarcinoma of large intestine	NRXN1	0	-6.901082	
4970	Adenocarcinoma of large intestine	KANK4	0.000582	-6.736475	
4971	Adenocarcinoma of large intestine	ABCA8	0.000001	-6.705704	
4972	Adenocarcinoma of large intestine	ANGPTL1	0.000002	-6.700761	
4973	Adenocarcinoma of large intestine	CWH43	0.000007	-6.340542	
4974	Adenocarcinoma of large intestine	GNG13	0	-6.173533	
4975	Adenocarcinoma of large intestine	PTGDR	0	-6.016754	
4976	Adenocarcinoma of large intestine	KIAA2022	0.000001	-5.99665	
4977	Adenocarcinoma of large intestine	ADH1C	0.00002	-5.903407	
4978	Adenocarcinoma of large intestine	CDH3	0	-5.7184	
4979	Adenocarcinoma of large intestine	C2orf58	0	-5.665292	
4980	Adenocarcinoma of large intestine	LGI4	0	-5.496047	
4981	Adenocarcinoma of large intestine	PHLDA1	0	-5.471027	
4982	Adenocarcinoma of large intestine	CDH19	0	-5.453266	
4983	Adenocarcinoma of large intestine	RGS13	0	-5.365318	
4984	Adenocarcinoma of large intestine	SVOPL	0	-5.28447	
4985	Adenocarcinoma of large intestine	PCSK2	0.000018	-5.268769	
4986	Adenocarcinoma of large intestine	UGT2B15	0.00016	-5.249733	
4987	Adenocarcinoma of large intestine	SCN7A	0	-5.18474	
4988	Adenocarcinoma of large intestine	SYNE2	0.000007	-5.052631	
4989	Adenocarcinoma of large intestine	BMP3	0.00004	-4.988584	
4990	Adenocarcinoma of large intestine	LOC144501	0	-4.899047	
4991	Adenocarcinoma of large intestine	SORCS1	0	-4.888339	
4992	Adenocarcinoma of large intestine	KCNV1	0	-4.774511	
4993	Adenocarcinoma of large intestine	PDZD3	0.000026	-4.758979	
4994	Adenocarcinoma of large intestine	DPP10	0.000008	-4.664912	
4995	Adenocarcinoma of large intestine	TEX11	0.000007	-4.646122	
4996	Adenocarcinoma of large intestine	LOC389023	0.000323	-4.643267	
4997	Adenocarcinoma of large intestine	COL1A2	0	-4.622895	
4998	Adenocarcinoma of large intestine	BGN	0	-4.605698	

4999	Adenocarcinoma of large intestine	C7	0.000004	-4.513299	
5000	Adenocarcinoma of large intestine	WSCD1	0.000004	-4.401202	
5001	Adenocarcinoma of large intestine	BMX	0	-4.36962	
5002	Adenocarcinoma of large intestine	LRRN2	0.000038	-4.27253	
5003	Adenocarcinoma of large intestine	SLC7A5	0	-4.182042	
5004	Adenocarcinoma of large intestine	ATP1A2	0.000005	-4.144251	
5005	Adenocarcinoma of large intestine	UGT1A8	0.000039	-4.117387	
5006	Adenocarcinoma of large intestine	TRIB3	0	-4.096233	
5007	Adenocarcinoma of large intestine	NFE2L3	0	-4.056847	
5008	Adenocarcinoma of large intestine	ESM1	0	-4.042148	
5009	Adenocarcinoma of large intestine	SLC26A2	0.000445	-3.928833	
5010	Adenocarcinoma of large intestine	SOX10	0	-3.805372	
5011	Adenocarcinoma of large intestine	BMP5	0.000002	-3.798608	
5012	Adenocarcinoma of large intestine	TGFBI	0	-3.780405	
5013	Adenocarcinoma of large intestine	AKR1B10	0.000036	-3.779094	
5014	Adenocarcinoma of large intestine	PCDH9	0	-3.700223	
5015	Adenocarcinoma of large intestine	CAPN13	0	-3.658466	
5016	Adenocarcinoma of large intestine	RUNDC3B	0.000001	-3.626594	
5017	Adenocarcinoma of large intestine	HEPACAM2	0.00047	-3.61294	
5018	Adenocarcinoma of large intestine	FLJ38379	0	-3.608873	
5019	Adenocarcinoma of large intestine	FLJ13391	0	-3.591249	
5020	Adenocarcinoma of large intestine	SCUBE2	0	-3.527884	
5021	Adenocarcinoma of large intestine	CNTN3	0.000217	-3.524586	
5022	Adenocarcinoma of large intestine	TIMP1	0	-3.518513	
5023	Adenocarcinoma of large intestine	RFX6	0.000515	-3.498165	
5024	Adenocarcinoma of large intestine	SLC17A4	0.000614	-3.493234	
5025	Adenocarcinoma of large intestine	ETV4	0	-3.474337	
5026	Adenocarcinoma of large intestine	CRHBP	0.000022	-3.460216	
5027	Adenocarcinoma of large intestine	LOC100508909	0.000124	-3.458099	
5028	Adenocarcinoma of large intestine	C2orf88	0.000402	-3.444938	
5029	Adenocarcinoma of large intestine	NAP1L2	0.000003	-3.425142	
5030	Adenocarcinoma of large intestine	SLC38A4	0.000772	-3.421945	
5031	Adenocarcinoma of large intestine	SALL4	0	-3.38018	
5032	Adenocarcinoma of large intestine	UGT1A1	0.000008	-3.369615	
5033	Adenocarcinoma of large intestine	IGLV4-60	0.000904	-3.356766	
5034	Adenocarcinoma of large intestine	DHRS11	0.000073	-3.355101	
5035	Adenocarcinoma of large intestine	FRMD1	0.000195	-3.352684	
5036	Adenocarcinoma of large intestine	FLJ32063	0.000003	-3.350273	
5037	Adenocarcinoma of large intestine	PCDH20	0.000651	-3.343165	
5038	Adenocarcinoma of large intestine	GRIA3	0.000081	-3.324625	
5039	Adenocarcinoma of large intestine	PCDH19	0.000016	-3.31126	
5040	Adenocarcinoma of large intestine	PDE6A	0.000883	-3.297526	
5041	Adenocarcinoma of large intestine	PDPN	0	-3.271364	
5042	Adenocarcinoma of large intestine	DEFB1	0.000319	-3.249128	
5043	Adenocarcinoma of large intestine	KCNE2	0.000013	-3.246601	
5044	Adenocarcinoma of large intestine	LOC100506591	0.000001	-3.241716	
5045	Adenocarcinoma of large intestine	GRIN2A	0.000005	-3.236666	
5046	Adenocarcinoma of large intestine	CA12	0.000051	-3.229288	
5047	Adenocarcinoma of large intestine	CACNA2D2	0.000088	-3.22725	
5048	Adenocarcinoma of large intestine	WFDC2	0.00064	-3.224515	

5049	Adenocarcinoma of large intestine	AMPD1	0.000073	-3.220971	
5050	Adenocarcinoma of large intestine	ADAMTSL1	0.000071	-3.164431	
5051	Adenocarcinoma of large intestine	GFRA1	0.000011	-3.150197	
5052	Adenocarcinoma of large intestine	HAPLN1	0.000036	-3.142222	
5053	Adenocarcinoma of large intestine	B4GALNT2	0.000254	-3.139801	
5054	Adenocarcinoma of large intestine	ARHGAP44	0.000001	-3.126782	
5055	Adenocarcinoma of large intestine	PHLPP2	0.000004	-3.124354	
5056	Adenocarcinoma of large intestine	HES5	0.000044	-3.121633	
5057	Adenocarcinoma of large intestine	EXD3	0.000012	-3.121086	
5058	Adenocarcinoma of large intestine	C10orf99	0.000092	-3.112238	
5059	Adenocarcinoma of large intestine	SH2D6	0.000009	-3.104952	
5060	Adenocarcinoma of large intestine	SLC4A4	0.00007	-3.104855	
5061	Adenocarcinoma of large intestine	TMEM72	0.000079	-3.087539	
5062	Adenocarcinoma of large intestine	LOC285878	0.000002	-3.080395	
5063	Adenocarcinoma of large intestine	CDC25B	0	-3.070827	
5064	Adenocarcinoma of large intestine	FAM150B	0.000003	-3.058634	
5065	Adenocarcinoma of large intestine	CDKL1	0.000668	-3.054882	
5066	Adenocarcinoma of large intestine	SPINK2	0.000162	-3.0247	
5067	Adenocarcinoma of large intestine	ZNF662	0.000025	-3.018874	
5068	Adenocarcinoma of large intestine	GFRA2	0.000011	-3.012423	
5069	Adenocarcinoma of large intestine	GRIK2	0.000184	-2.978048	
5070	Adenocarcinoma of large intestine	C9orf24	0.000511	-2.974354	
5071	Adenocarcinoma of large intestine	IGFBP2	0.000009	-2.971447	
5072	Adenocarcinoma of large intestine	ARL14	0.000094	-2.965791	
5073	Adenocarcinoma of large intestine	LRRC19	0.000619	-2.954561	
5074	Adenocarcinoma of large intestine	USP7	0.000112	-2.951311	
5075	Adenocarcinoma of large intestine	THRB	0.000477	-2.92102	
5076	Adenocarcinoma of large intestine	LRP8	0	-2.905623	
5077	Adenocarcinoma of large intestine	FAM189A1	0.000032	-2.904059	
5078	Adenocarcinoma of large intestine	CELA3B	0.000095	-2.901027	
5079	Adenocarcinoma of large intestine	BCHE	0.000148	-2.873688	
5080	Adenocarcinoma of large intestine	PDGFRA	0.000026	-2.841994	
5081	Adenocarcinoma of large intestine	DHRS7C	0.000528	-2.814171	
5082	Adenocarcinoma of large intestine	ENHO	0.000267	-2.795258	
5083	Adenocarcinoma of large intestine	DSCAML1	0.00004	-2.791862	
5084	Adenocarcinoma of large intestine	SIGLEC1	0.000004	-2.786226	
5085	Adenocarcinoma of large intestine	HMGCS2	0.000347	-2.784072	
5086	Adenocarcinoma of large intestine	KIAA1683	0.000381	-2.781161	
5087	Adenocarcinoma of large intestine	FXYD1	0.000052	-2.759432	
5088	Adenocarcinoma of large intestine	KIAA0319	0.000411	-2.747012	
5089	Adenocarcinoma of large intestine	PRKG2	0.000925	-2.733283	
5090	Adenocarcinoma of large intestine	CPM	0.000638	-2.731148	
5091	Adenocarcinoma of large intestine	PHGR1	0.000007	-2.72038	
5092	Adenocarcinoma of large intestine	PADI2	0.00005	-2.707104	
5093	Adenocarcinoma of large intestine	FGF9	0.000671	-2.701905	
5094	Adenocarcinoma of large intestine	C1orf115	0.000028	-2.700641	
5095	Adenocarcinoma of large intestine	LIFR	0.000886	-2.695526	
5096	Adenocarcinoma of large intestine	LOC100287411	0.000004	-2.686546	
5097	Adenocarcinoma of large intestine	PPP2R3A	0.000044	-2.652001	
5098	Adenocarcinoma of large intestine	FLJ36848	0.00035	-2.648614	

5099	Adenocarcinoma of large intestine	FAM55A	0.000402	-2.645677	
5100	Adenocarcinoma of large intestine	SELENBP1	0.000389	-2.638804	
5101	Adenocarcinoma of large intestine	PTPRZ1	0.000041	-2.633858	
5102	Adenocarcinoma of large intestine	SMOX	0	-2.620293	
5103	Adenocarcinoma of large intestine	FZD9	0.000734	-2.619644	
5104	Adenocarcinoma of large intestine	SGK2	0.00017	-2.610315	
5105	Adenocarcinoma of large intestine	CLU	0.000096	-2.607711	
5106	Adenocarcinoma of large intestine	F13A1	0.000026	-2.603464	
5107	Adenocarcinoma of large intestine	CORO2B	0.000078	-2.602121	
5108	Adenocarcinoma of large intestine	CD36	0.000013	-2.571099	
5109	Adenocarcinoma of large intestine	WDR78	0.00079	-2.565834	
5110	Adenocarcinoma of large intestine	S100A11	0	-2.556185	
5111	Adenocarcinoma of large intestine	FXYD5	0	-2.548632	
5112	Adenocarcinoma of large intestine	MUM1L1	0.000125	-2.545621	
5113	Adenocarcinoma of large intestine	CDC14A	0.000013	-2.52973	
5114	Adenocarcinoma of large intestine	DCAF8	0.000282	-2.488817	
5115	Adenocarcinoma of large intestine	P2RY1	0.000279	-2.484963	
5116	Adenocarcinoma of large intestine	ACVR1C	0.000001	-2.484118	
5117	Adenocarcinoma of large intestine	FABP4	0.000429	-2.467217	
5118	Adenocarcinoma of large intestine	BMP2K	0.000903	-2.464008	
5119	Adenocarcinoma of large intestine	PPID	0.000025	-2.462194	
5120	Adenocarcinoma of large intestine	AVIL	0.000001	-2.461099	
5121	Adenocarcinoma of large intestine	PDE4DIP	0.000528	-2.449105	
5122	Adenocarcinoma of large intestine	C1orf161	0.000928	-2.442114	
5123	Adenocarcinoma of large intestine	LOC219347	0.000163	-2.439474	
5124	Adenocarcinoma of large intestine	PRIMA1	0.000018	-2.435454	
5125	Adenocarcinoma of large intestine	LANCL3	0.000299	-2.429492	
5126	Adenocarcinoma of large intestine	CDK3	0.000077	-2.425672	
5127	Adenocarcinoma of large intestine	SLC36A1	0.000061	-2.421944	
5128	Adenocarcinoma of large intestine	CNNM2	0.000885	-2.413334	
5129	Adenocarcinoma of large intestine	GPR44	0.000131	-2.40323	
5130	Adenocarcinoma of large intestine	GATM	0.000842	-2.391762	
5131	Adenocarcinoma of large intestine	LOC400573	0.000263	-2.3904	
5132	Adenocarcinoma of large intestine	PLIN1	0.000566	-2.387082	
5133	Adenocarcinoma of large intestine	SLC22A5	0.000006	-2.383758	
5134	Adenocarcinoma of large intestine	AHCYL2	0.000628	-2.379079	
5135	Adenocarcinoma of large intestine	NAAA	0.000629	-2.377968	
5136	Adenocarcinoma of large intestine	TEAD4	0	-2.373796	
5137	Adenocarcinoma of large intestine	C20orf20	0	-2.373628	
5138	Adenocarcinoma of large intestine	MTHFD1L	0	-2.368066	
5139	Adenocarcinoma of large intestine	ZFP3	0.000004	-2.365486	
5140	Adenocarcinoma of large intestine	LOC100292909	0.000445	-2.365269	
5141	Adenocarcinoma of large intestine	SATB2	0.000228	-2.342286	
5142	Adenocarcinoma of large intestine	KLF8	0.000744	-2.298547	
5143	Adenocarcinoma of large intestine	PSMG4	0.000001	-2.294456	
5144	Adenocarcinoma of large intestine	AGPAT9	0.000539	-2.293253	
5145	Adenocarcinoma of large intestine	LRMP	0.00005	-2.283572	
5146	Adenocarcinoma of large intestine	BCL2L15	0.000028	-2.268541	
5147	Adenocarcinoma of large intestine	MAPT	0.000094	-2.264895	
5148	Adenocarcinoma of large intestine	MOCS1	0.000648	-2.261519	

5149	Adenocarcinoma of large intestine	RHBDF2	0	-2.25911	
5150	Adenocarcinoma of large intestine	SLC9A2	0.00037	-2.258692	
5151	Adenocarcinoma of large intestine	ATP10A	0.000191	-2.232913	
5152	Adenocarcinoma of large intestine	MMP28	0.000395	-2.225691	
5153	Adenocarcinoma of large intestine	WISP1	0	-2.212239	
5154	Adenocarcinoma of large intestine	DAPK2	0.000157	-2.196591	
5155	Adenocarcinoma of large intestine	MIER3	0.000032	-2.171328	
5156	Adenocarcinoma of large intestine	SULT1A1	0.000014	-2.150609	
5157	Adenocarcinoma of large intestine	OSBPL1A	0.000137	-2.149565	
5158	Adenocarcinoma of large intestine	ATP5S	0.000093	-2.147486	
5159	Adenocarcinoma of large intestine	ATOH8	0.000883	-2.140852	
5160	Adenocarcinoma of large intestine	RIPK2	0	-2.137108	
5161	Adenocarcinoma of large intestine	NPY	0.000112	-2.125373	
5162	Adenocarcinoma of large intestine	GOLGA2B	0.000001	-2.124954	
5163	Adenocarcinoma of large intestine	PPARGC1A	0.000582	-2.097672	
5164	Adenocarcinoma of large intestine	BCL2	0.000006	-2.095351	
5165	Adenocarcinoma of large intestine	FLJ44606	0.000687	-2.08379	
5166	Adenocarcinoma of large intestine	FAM47E	0.00003	-2.074512	
5167	Adenocarcinoma of large intestine	DENND2A	0.000268	-2.067171	
5168	Adenocarcinoma of large intestine	SULT1A2	0.000112	-2.054603	
5169	Adenocarcinoma of large intestine	LRRC3B	0.000693	-2.049939	
5170	Adenocarcinoma of large intestine	ACSF2	0.000282	-2.048502	
5171	Adenocarcinoma of large intestine	TRPM4	0.000312	-2.029588	
5172	Adenocarcinoma of large intestine	FJX1	0	-2.028496	
5173	Adenocarcinoma of large intestine	ZNF575	0.000162	-2.028027	
5174	Adenocarcinoma of large intestine	DACT2	0.000588	-2.023093	
5175	Adenocarcinoma of large intestine	AIFM3	0.000415	-2.023087	
5176	Adenocarcinoma of large intestine	SSBP2	0.000699	-2.016057	
5177	Adenocarcinoma of large intestine	MTHFD2	0	-2.009216	
5178	Adenocarcinoma of large intestine	STAG3	0.000114	-2.003641	
5179	Adenocarcinoma of large intestine	ROPN1	0.000748	-2.002696	
5180	Adenocarcinoma of large intestine	CCBP2	0	2.027799	
5181	Adenocarcinoma of large intestine	ZZEF1	0	2.045019	
5182	Adenocarcinoma of large intestine	CITED2	0	2.074953	
5183	Adenocarcinoma of large intestine	WISP2	0	2.08814	
5184	Adenocarcinoma of large intestine	GLTP	0	2.101629	
5185	Adenocarcinoma of large intestine	PLCD3	0	2.104867	
5186	Adenocarcinoma of large intestine	PPARGC1B	0	2.109637	
5187	Adenocarcinoma of large intestine	TP53INP2	0	2.11798	
5188	Adenocarcinoma of large intestine	AFF3	0	2.168501	
5189	Adenocarcinoma of large intestine	TNFRSF13B	0	2.195702	
5190	Adenocarcinoma of large intestine	CPT2	0	2.196555	
5191	Adenocarcinoma of large intestine	DHRS9	0	2.198375	
5192	Adenocarcinoma of large intestine	BRP44L	0	2.200729	
5193	Adenocarcinoma of large intestine	ETFDH	0	2.226034	
5194	Adenocarcinoma of large intestine	RPIB9	0	2.251726	
5195	Adenocarcinoma of large intestine	TMEFF2	0	2.267269	
5196	Adenocarcinoma of large intestine	KIF5C	0	2.283957	
5197	Adenocarcinoma of large intestine	AKAP7	0	2.2988	
5198	Adenocarcinoma of large intestine	NKX2-3	0	2.302686	

5199	Adenocarcinoma of large intestine	LGI1	0	2.314818	
5200	Adenocarcinoma of large intestine	PMP2	0	2.341572	
5201	Adenocarcinoma of large intestine	UGP2	0	2.354849	
5202	Adenocarcinoma of large intestine	GNA11	0	2.359015	
5203	Adenocarcinoma of large intestine	TCF21	0	2.391464	
5204	Adenocarcinoma of large intestine	NEDD4L	0	2.466577	
5205	Adenocarcinoma of large intestine	PPAP2A	0	2.488416	
5206	Adenocarcinoma of large intestine	HHLA2	0	2.489899	
5207	Adenocarcinoma of large intestine	TMEM100	0	2.527418	
5208	Adenocarcinoma of large intestine	LOC643940	0	2.564427	
5209	Adenocarcinoma of large intestine	FUCA1	0	2.564942	
5210	Adenocarcinoma of large intestine	RDH5	0	2.567677	
5211	Adenocarcinoma of large intestine	RetSat	0	2.572066	
5212	Adenocarcinoma of large intestine	MGC14376	0	2.624985	
5213	Adenocarcinoma of large intestine	ASAHL	0	2.687594	
5214	Adenocarcinoma of large intestine	PHLPPL	0	2.688005	
5215	Adenocarcinoma of large intestine	PLCD1	0	2.749102	
5216	Adenocarcinoma of large intestine	EPB41L3	0	2.762123	
5217	Adenocarcinoma of large intestine	ENTPD5	0	2.764317	
5218	Adenocarcinoma of large intestine	LOC285016	0	2.803106	
5219	Adenocarcinoma of large intestine	EDG2	0	2.8128	
5220	Adenocarcinoma of large intestine	KRT24	0	2.892109	
5221	Adenocarcinoma of large intestine	GREM2	0	3.005481	
5222	Adenocarcinoma of large intestine	OGN	0	3.035103	
5223	Adenocarcinoma of large intestine	HMFN0839	0	3.055462	
5224	Adenocarcinoma of large intestine	KCNIP4	0	3.090417	
5225	Adenocarcinoma of large intestine	CNTFR	0	3.157038	
5226	Adenocarcinoma of large intestine	GPM6B	0	3.220923	
5227	Adenocarcinoma of large intestine	MAL	0	3.354032	
5228	Adenocarcinoma of large intestine	OTOP2	0	3.429898	
5229	Adenocarcinoma of large intestine	LDHD	0	3.492451	
5230	Adenocarcinoma of large intestine	FAM107A	0	3.744748	
5231	Adenocarcinoma of large intestine	EDN3	0	3.87703	
5232	Adenocarcinoma of large intestine	MATN2	0	3.91557	
5233	Adenocarcinoma of large intestine	LOC441282	0	4.058839	
5234	Adenocarcinoma of large intestine	NR3C2	0	4.11659	
5235	Adenocarcinoma of large intestine	KIAA0828	0	4.161943	
5236	Adenocarcinoma of large intestine	PRPH	0	4.228059	
5237	Adenocarcinoma of large intestine	PDK4	0	4.232559	
5238	Adenocarcinoma of large intestine	ADH1B	0	4.346492	
5239	Adenocarcinoma of large intestine	CA7	0	4.360658	
5240	Adenocarcinoma of large intestine	SCIN	0	4.434475	
5241	Adenocarcinoma of large intestine	GPT	0	4.443061	
5242	Adenocarcinoma of large intestine	PLAC9	0	4.463959	
5243	Adenocarcinoma of large intestine	LOC340843	0	4.503637	
5244	Adenocarcinoma of large intestine	VIP	0	4.583618	
5245	Adenocarcinoma of large intestine	MGC4172	0	4.678043	
5246	Adenocarcinoma of large intestine	SRPX	0	4.822471	
5247	Adenocarcinoma of large intestine	KLF4	0	4.841207	
5248	Adenocarcinoma of large intestine	UGT1A10	0	4.847321	

5249	Adenocarcinoma of large intestine	FLJ21511	0	4.982366	
5250	Adenocarcinoma of large intestine	PCOLCE2	0	5.114989	
5251	Adenocarcinoma of large intestine	VMD2L2	0	5.32281	
5252	Adenocarcinoma of large intestine	HPGD	0	5.351308	
5253	Adenocarcinoma of large intestine	SCNN1B	0	5.373861	
5254	Adenocarcinoma of large intestine	CLEC3B	0	5.526861	
5255	Adenocarcinoma of large intestine	PI16	0	5.715154	
5256	Adenocarcinoma of large intestine	VMD2L1	0	5.755075	
5257	Adenocarcinoma of large intestine	HSD11B2	0	6.08248	
5258	Adenocarcinoma of large intestine	MGC13057	0	6.316289	
5259	Adenocarcinoma of large intestine	PYY	0	7.20626	
5260	Adenocarcinoma of large intestine	CFD	0	7.915842	
5261	Adenocarcinoma of large intestine	MT1M	0	8.12885	
5262	Adenocarcinoma of large intestine	LOC63928	0	8.915585	
5263	Adenocarcinoma of large intestine	TMIGD	0	10.791315	
5264	Adenocarcinoma of large intestine	MS4A12	0	17.955812	
5265	Adenocarcinoma of large intestine	CA4	0	18.548553	
5266	Adenocarcinoma of large intestine	CLCA4	0	31.629654	
5267	Adenocarcinoma of large intestine	CA1	0	36.110772	
5268	Adenocarcinoma, Mucinous	LY6G6D	0	-4.234524	
5269	Adenocarcinoma, Mucinous	CELP	0.000244	-3.495462	
5270	Adenocarcinoma, Mucinous	DNASE1	0.001751	-3.430109	
5271	Adenocarcinoma, Mucinous	ASCL2	0.000001	-3.062706	
5272	Adenocarcinoma, Mucinous	ACSL6	0.000001	-2.83988	
5273	Adenocarcinoma, Mucinous	CAB39L	0.000001	-2.735909	
5274	Adenocarcinoma, Mucinous	MAP7D2	0.000006	-2.688518	
5275	Adenocarcinoma, Mucinous	GRM8	0.000002	-2.68718	
5276	Adenocarcinoma, Mucinous	KRT23	0.000539	-2.685039	
5277	Adenocarcinoma, Mucinous	LOC283859	0.000012	-2.618039	
5278	Adenocarcinoma, Mucinous	SLC13A3	0.00001	-2.557739	
5279	Adenocarcinoma, Mucinous	EREG	0	-2.553703	
5280	Adenocarcinoma, Mucinous	GPR143	0.000005	-2.545016	
5281	Adenocarcinoma, Mucinous	PIPOX	0.001914	-2.285877	
5282	Adenocarcinoma, Mucinous	ABAT	0.000517	-2.258946	
5283	Adenocarcinoma, Mucinous	GNG4	0.00147	-2.257787	
5284	Adenocarcinoma, Mucinous	ACE2	0.000001	-2.234792	
5285	Adenocarcinoma, Mucinous	SLC1A7	0.000132	-2.20973	
5286	Adenocarcinoma, Mucinous	C13orf18	0	-2.197989	
5287	Adenocarcinoma, Mucinous	PLA2G12B	0.000003	-2.136562	
5288	Adenocarcinoma, Mucinous	REEP1	0	-2.120391	
5289	Adenocarcinoma, Mucinous	RNF43	0	-2.095993	
5290	Adenocarcinoma, Mucinous	XPNPEP2	0.001566	-2.073633	
5291	Adenocarcinoma, Mucinous	FGGY	0.000002	-2.066334	
5292	Adenocarcinoma, Mucinous	EIF2S2	0.000003	-2.022957	
5293	Adenocarcinoma, Mucinous	TFF1	0.000664	2.595315	
5294	Adenocarcinoma, Mucinous	REG4	0.000281	3.555368	
5295	adenoma	SPINK5	0.000031	-32.239686	
5296	adenoma	ACHE	0.000024	-23.943237	
5297	adenoma	SLC25A34	0.000046	-21.203959	
5298	adenoma	DHRS9	0.000068	-17.872567	

5299	adenoma	OSTBETA	0.000025	-17.297428	
5300	adenoma	THOC4	0.002192	-15.850692	
5301	adenoma	DPEP2	0.000041	-15.654621	
5302	adenoma	OLFM1	0	-15.232331	
5303	adenoma	STMN2	0.000003	-14.933347	
5304	adenoma	BTNL8	0.000017	-14.197677	
5305	adenoma	SLC15A1	0.000019	-13.109778	
5306	adenoma	SFSWAP	0.000385	-12.019069	
5307	adenoma	CYBRD1	0.000011	-11.498188	
5308	adenoma	PYY	0	-11.401911	
5309	adenoma	CRYBA2	0.000001	-11.209007	
5310	adenoma	C11orf86	0.000005	-11.074642	
5311	adenoma	ABCG2	0.000001	-10.987225	
5312	adenoma	LGALS2	0.000022	-10.968017	
5313	adenoma	FBXO16	0.002042	-10.302455	
5314	adenoma	APBB1	0.000007	-9.770749	
5315	adenoma	SAA1	0.00016	-9.645666	
5316	adenoma	IQGAP3	0.001662	-9.509695	
5317	adenoma	DNAJC3	0.001686	-9.227685	
5318	adenoma	FOXM1	0.001886	-8.896249	
5319	adenoma	TAF13	0.000076	-8.867731	
5320	adenoma	KIAA0090	0.000315	-8.86213	
5321	adenoma	CDKN2B-AS	0.000002	-8.855571	
5322	adenoma	NME3	0.000161	-8.793324	
5323	adenoma	MAGEA3	0.000022	-8.032836	
5324	adenoma	CDA	0.000052	-7.974413	
5325	adenoma	MAGEA6	0.00001	-7.748368	
5326	adenoma	CCDC102B	0.000001	-7.700813	
5327	adenoma	YKT6	0.000744	-7.467236	
5328	adenoma	SDCBP2	0.000007	-7.428168	
5329	adenoma	ZNF568	0.000024	-7.327563	
5330	adenoma	PLEKHG2	0.000054	-7.265051	
5331	adenoma	HSD17B2	0.000023	-7.156495	
5332	adenoma	FEV	0.000002	-7.139037	
5333	adenoma	FLJ35700	0.000058	-7.106864	
5334	adenoma	LMNB2	0.001886	-7.090754	
5335	adenoma	TPH1	0.000003	-7.005897	
5336	adenoma	PKNOX2	0.000046	-6.995376	
5337	adenoma	RAB9B	0.000039	-6.901206	
5338	adenoma	ENPP6	0.000082	-6.893884	
5339	adenoma	CDCA8	0.001018	-6.831727	
5340	adenoma	CHP2	0	-6.7631	
5341	adenoma	LOC646627	0	-6.74501	
5342	adenoma	DGCR8	0.000704	-6.718962	
5343	adenoma	BEST4	0.000005	-6.576351	
5344	adenoma	SYNE1	0.000014	-6.563564	
5345	adenoma	GPR109B	0	-6.520218	
5346	adenoma	CELA3A	0.000054	-6.497474	
5347	adenoma	CLN6	0.001478	-6.49325	
5348	adenoma	HRCT1	0.000044	-6.487225	

5349	adenoma	TRIO	0.000934	-6.447365	
5350	adenoma	PKP4	0.000111	-6.417608	
5351	adenoma	SLC26A2	0	-6.351358	
5352	adenoma	CHGB	0.000032	-6.347379	
5353	adenoma	SLCO2B1	0.000011	-6.318094	
5354	adenoma	ACVR1B	0.00116	-6.25544	
5355	adenoma	PRKAA2	0.000019	-6.127948	
5356	adenoma	GPAT2	0.000035	-6.11638	
5357	adenoma	CA7	0	-6.05505	
5358	adenoma	DSEL	0.000007	-5.961644	
5359	adenoma	CELA3B	0.000055	-5.881904	
5360	adenoma	CTSW	0.00006	-5.831313	
5361	adenoma	SLC35E1	0.00013	-5.757664	
5362	adenoma	GNB1	0.000526	-5.737384	
5363	adenoma	SPTLC3	0	-5.719148	
5364	adenoma	SOSTDC1	0.000007	-5.715365	
5365	adenoma	CLECL1	0.000024	-5.673399	
5366	adenoma	POR	0.000845	-5.635963	
5367	adenoma	FAM126B	0.001247	-5.576524	
5368	adenoma	CBX5	0.001842	-5.542697	
5369	adenoma	C17orf70	0.00013	-5.532599	
5370	adenoma	HTR4	0.000024	-5.522219	
5371	adenoma	E2F1	0.001821	-5.423845	
5372	adenoma	IL16	0.000032	-5.408401	
5373	adenoma	B2M	0.001522	-5.406274	
5374	adenoma	SLC6A13	0.000054	-5.392697	
5375	adenoma	MESP2	0.000034	-5.381215	
5376	adenoma	FAM150B	0.000003	-5.356415	
5377	adenoma	CHAF1A	0.000672	-5.355374	
5378	adenoma	FIGN	0.000032	-5.328371	
5379	adenoma	DPP10	0	-5.255341	
5380	adenoma	LRRK2	0	-5.211608	
5381	adenoma	ABI3BP	0	-5.15203	
5382	adenoma	GPR153	0.000831	-5.134941	
5383	adenoma	LRRN2	0.000004	-5.114688	
5384	adenoma	GPRC5B	0.000054	-5.056463	
5385	adenoma	QSER1	0.001662	-5.042886	
5386	adenoma	CDH19	0	-5.022701	
5387	adenoma	CAMKK2	0.000251	-5.000891	
5388	adenoma	POLDIP2	0.000656	-4.984634	
5389	adenoma	PPTC7	0.002137	-4.909444	
5390	adenoma	KIAA2022	0.000001	-4.891469	
5391	adenoma	TMEM72	0.000001	-4.884725	
5392	adenoma	INSM1	0	-4.88269	
5393	adenoma	PRRC1	0.000109	-4.870486	
5394	adenoma	CXCL5	0	-4.838213	
5395	adenoma	GZMK	0.000021	-4.771295	
5396	adenoma	GUCA1B	0	-4.730904	
5397	adenoma	SECTM1	0.000043	-4.703083	
5398	adenoma	CLIP3	0.000019	-4.692329	

5399	adenoma	HHLA2	0.000005	-4.674398	
5400	adenoma	WSCD1	0	-4.610428	
5401	adenoma	SERPINE1	0	-4.593549	
5402	adenoma	RPS6KB2	0.000132	-4.579522	
5403	adenoma	ILF3	0.000279	-4.568197	
5404	adenoma	DPP4	0.000066	-4.500848	
5405	adenoma	PCDH9	0	-4.498007	
5406	adenoma	MYOT	0.000002	-4.483537	
5407	adenoma	FLJ36848	0.000032	-4.481406	
5408	adenoma	SNX8	0.000854	-4.462603	
5409	adenoma	TUSC3	0.000001	-4.451097	
5410	adenoma	COX15	0.000588	-4.445678	
5411	adenoma	ARF5	0.00013	-4.444786	
5412	adenoma	CD1D	0.000006	-4.435071	
5413	adenoma	FAM132A	0.000022	-4.373596	
5414	adenoma	ATRIP	0.000315	-4.366136	
5415	adenoma	LOC647979	0.001018	-4.328323	
5416	adenoma	PAK6	0.000745	-4.323249	
5417	adenoma	HMOX1	0.000006	-4.31922	
5418	adenoma	SPIB	0	-4.317744	
5419	adenoma	HSPA4	0.001417	-4.309139	
5420	adenoma	LOC399959	0.000022	-4.287609	
5421	adenoma	RTN1	0	-4.266108	
5422	adenoma	PASK	0.00077	-4.240231	
5423	adenoma	LOC100507192	0.000082	-4.220012	
5424	adenoma	PPP4C	0.00187	-4.187227	
5425	adenoma	AQP9	0.000002	-4.184974	
5426	adenoma	HIST1H1C	0.000005	-4.183722	
5427	adenoma	H19	0.000013	-4.174771	
5428	adenoma	GPM6B	0	-4.161942	
5429	adenoma	ILVBL	0.00013	-4.144674	
5430	adenoma	CCL2	0.000015	-4.121636	
5431	adenoma	PLP1	0.000004	-4.115264	
5432	adenoma	RGS2	0.000028	-4.115168	
5433	adenoma	WDR46	0.001018	-4.111929	
5434	adenoma	SDC3	0.000082	-4.088098	
5435	adenoma	LAMA2	0	-4.08393	
5436	adenoma	LBR	0.000241	-4.078122	
5437	adenoma	PTPLAD1	0.001886	-4.066034	
5438	adenoma	MGP	0	-4.058043	
5439	adenoma	NLGN4X	0	-4.057908	
5440	adenoma	SLC25A1	0.001005	-4.035714	
5441	adenoma	ATP6V0B	0.000159	-3.983904	
5442	adenoma	ANP32A	0.00038	-3.971833	
5443	adenoma	DPT	0	-3.964112	
5444	adenoma	PCDH19	0.00002	-3.957706	
5445	adenoma	DPYD	0	-3.957465	
5446	adenoma	MAGEA2	0.000008	-3.948847	
5447	adenoma	LOC25845	0.000002	-3.940293	
5448	adenoma	SCN7A	0.000001	-3.937932	

5449	adenoma	MEP1A	0	-3.928076	
5450	adenoma	RPS27A	0.000251	-3.905075	
5451	adenoma	VIL1	0.001542	-3.894469	
5452	adenoma	RBP5	0.00001	-3.891229	
5453	adenoma	H2AFX	0.002042	-3.877215	
5454	adenoma	FGF9	0	-3.874986	
5455	adenoma	TMEM222	0.000995	-3.874331	
5456	adenoma	RAB14	0.001286	-3.872952	
5457	adenoma	GNG11	0.000054	-3.848307	
5458	adenoma	KCTD5	0.001408	-3.841062	
5459	adenoma	IQGAP1	0.001467	-3.831449	
5460	adenoma	UHMK1	0.000082	-3.826934	
5461	adenoma	CGN	0.001018	-3.815548	
5462	adenoma	KIF18B	0.00115	-3.804853	
5463	adenoma	PRR5	0.000536	-3.798945	
5464	adenoma	NHP2L1	0.00019	-3.791524	
5465	adenoma	HDGFRP3	0.00009	-3.781041	
5466	adenoma	SCGN	0.000009	-3.779157	
5467	adenoma	MAGEA12	0.000002	-3.774247	
5468	adenoma	NRXN1	0.000002	-3.768761	
5469	adenoma	FAM38A	0.000799	-3.762828	
5470	adenoma	ACLY	0.001475	-3.75369	
5471	adenoma	SEMA3E	0.000001	-3.749221	
5472	adenoma	ARHGAP20	0	-3.749217	
5473	adenoma	SET	0.000251	-3.748505	
5474	adenoma	GATAD2A	0.000082	-3.724059	
5475	adenoma	LY9	0.000072	-3.719374	
5476	adenoma	TIPRL	0.00148	-3.704312	
5477	adenoma	SOD2	0	-3.701291	
5478	adenoma	BCHE	0.000066	-3.695978	
5479	adenoma	ZNF75A	0	-3.693634	
5480	adenoma	RHOF	0.000004	-3.660494	
5481	adenoma	ZNF124	0.00115	-3.65106	
5482	adenoma	NEU4	0.000076	-3.65104	
5483	adenoma	GUCY1A3	0.000002	-3.647659	
5484	adenoma	HMCN1	0.000082	-3.643733	
5485	adenoma	ATAD3A	0.000419	-3.641075	
5486	adenoma	COMP	0.000063	-3.625885	
5487	adenoma	LOC646701	0.000082	-3.621953	
5488	adenoma	TPK1	0.000016	-3.612082	
5489	adenoma	BAG5	0.002107	-3.61189	
5490	adenoma	PHPT1	0.000745	-3.596465	
5491	adenoma	INTS6	0.000536	-3.583784	
5492	adenoma	C2orf40	0.000056	-3.580672	
5493	adenoma	EYA2	0	-3.577915	
5494	adenoma	KIF16B	0.000001	-3.576707	
5495	adenoma	IL1R2	0	-3.568313	
5496	adenoma	NEURL3	0.000006	-3.521461	
5497	adenoma	LPHN2	0	-3.519243	
5498	adenoma	VIPR1	0	-3.513648	

5499	adenoma	DDOST	0.000387	-3.511996	
5500	adenoma	APLP2	0.000744	-3.51052	
5501	adenoma	EIF1AX	0.001622	-3.499155	
5502	adenoma	SDC1	0.000536	-3.462107	
5503	adenoma	IL1R1	0.000025	-3.461987	
5504	adenoma	CLEC10A	0.000005	-3.459407	
5505	adenoma	CPNE3	0.000419	-3.458522	
5506	adenoma	GAS1	0.000002	-3.452579	
5507	adenoma	TFAM	0.000283	-3.448504	
5508	adenoma	MRPS5	0.001616	-3.438726	
5509	adenoma	ESR1	0.000054	-3.434047	
5510	adenoma	AHRR	0.000075	-3.424512	
5511	adenoma	CD14	0.000013	-3.41472	
5512	adenoma	NDUFV1	0.001768	-3.405372	
5513	adenoma	PPIB	0.00001	-3.403223	
5514	adenoma	MFAP4	0	-3.393558	
5515	adenoma	AIF1	0.000082	-3.388451	
5516	adenoma	SLC17A4	0.000002	-3.387147	
5517	adenoma	MLXIP	0.001034	-3.386402	
5518	adenoma	CD300A	0.000001	-3.383942	
5519	adenoma	TIMM13	0.000871	-3.379752	
5520	adenoma	PCDH7	0.000022	-3.361762	
5521	adenoma	SRPX	0.000007	-3.357829	
5522	adenoma	NOL9	0.00149	-3.357671	
5523	adenoma	S100A8	0.000001	-3.352349	
5524	adenoma	NOVA1	0.000072	-3.351954	
5525	adenoma	RNF213	0.002078	-3.329022	
5526	adenoma	IDH3A	0.001709	-3.325995	
5527	adenoma	USP1	0.000745	-3.324365	
5528	adenoma	PAMR1	0.000002	-3.31603	
5529	adenoma	ZNF791	0.000279	-3.314098	
5530	adenoma	SAMD9	0.000058	-3.312558	
5531	adenoma	PDE7B	0	-3.311536	
5532	adenoma	CHL1	0.000017	-3.308172	
5533	adenoma	WTAP	0.000189	-3.308103	
5534	adenoma	TBC1D22A	0.002108	-3.297469	
5535	adenoma	RAB8A	0.002192	-3.294049	
5536	adenoma	UPP1	0.000072	-3.293296	
5537	adenoma	FAM110B	0.000001	-3.283492	
5538	adenoma	NCF4	0.000022	-3.282698	
5539	adenoma	C20orf194	0	-3.28122	
5540	adenoma	CDCA7	0.000744	-3.27528	
5541	adenoma	HNRNPC	0.000704	-3.273079	
5542	adenoma	ATG7	0.001182	-3.263631	
5543	adenoma	LOC401022	0	-3.262621	
5544	adenoma	PRR11	0.001855	-3.244226	
5545	adenoma	SFMBT2	0.000001	-3.24373	
5546	adenoma	EMCN	0.000009	-3.242664	
5547	adenoma	TRIM14	0.002192	-3.230347	
5548	adenoma	PGAM1	0.001197	-3.223489	

5549	adenoma	PDPK1	0.000588	-3.202151	
5550	adenoma	NAP1L4	0.00038	-3.201689	
5551	adenoma	U2AF1	0.000123	-3.201448	
5552	adenoma	WIPF1	0.000006	-3.18889	
5553	adenoma	MIR214	0.000015	-3.185454	
5554	adenoma	CCNYL1	0.000005	-3.18485	
5555	adenoma	NCL	0.000082	-3.177353	
5556	adenoma	PTPRC	0.000015	-3.171861	
5557	adenoma	EBF1	0.000026	-3.164785	
5558	adenoma	ZC3H7A	0.001616	-3.163694	
5559	adenoma	SLC38A1	0.000658	-3.163284	
5560	adenoma	ST14	0.001286	-3.153344	
5561	adenoma	LOC100508909	0.000004	-3.147079	
5562	adenoma	PDE6A	0	-3.146689	
5563	adenoma	EPPK1	0.000193	-3.14574	
5564	adenoma	HIPK2	0.001981	-3.139042	
5565	adenoma	MIB1	0.002122	-3.136432	
5566	adenoma	HLF	0.000001	-3.135844	
5567	adenoma	KEAP1	0.001597	-3.129816	
5568	adenoma	CNTN1	0.000002	-3.122925	
5569	adenoma	PADI2	0	-3.121707	
5570	adenoma	KCNIP2	0.000042	-3.119855	
5571	adenoma	DNASE1L3	0.000027	-3.119775	
5572	adenoma	RCAN2	0	-3.119317	
5573	adenoma	IFRD2	0.000931	-3.11365	
5574	adenoma	C1S	0	-3.112694	
5575	adenoma	HTR2B	0.000205	-3.10838	
5576	adenoma	TRIM25	0.000315	-3.102752	
5577	adenoma	CCL18	0.000032	-3.10227	
5578	adenoma	NKX2-3	0.000074	-3.096098	
5579	adenoma	SRSF3	0.001034	-3.09025	
5580	adenoma	MMP2	0.000043	-3.089364	
5581	adenoma	PLA2G10	0	-3.086968	
5582	adenoma	GGA3	0.000526	-3.066348	
5583	adenoma	NFIA	0.000419	-3.064992	
5584	adenoma	DES	0.000264	-3.063024	
5585	adenoma	HSD11B1	0.000217	-3.061173	
5586	adenoma	CCL28	0.000005	-3.053933	
5587	adenoma	C5orf46	0.000007	-3.04792	
5588	adenoma	TNFAIP6	0	-3.046939	
5589	adenoma	APPL2	0	-3.042915	
5590	adenoma	OTUB1	0.000279	-3.037385	
5591	adenoma	LRRC19	0	-3.037023	
5592	adenoma	BCAT1	0.000001	-3.015072	
5593	adenoma	PMP22	0.000002	-3.011268	
5594	adenoma	SLC39A3	0.000776	-3.004355	
5595	adenoma	HIP1	0.000051	-3.001035	
5596	adenoma	RNASEK	0.001468	-3.000378	
5597	adenoma	FAM20A	0.000019	-2.998583	
5598	adenoma	PROK2	0.0001	-2.985051	

5599	adenoma	RERG	0	-2.9816	
5600	adenoma	PAQR5	0	-2.981438	
5601	adenoma	MAFB	0.000031	-2.981246	
5602	adenoma	MATN2	0	-2.980745	
5603	adenoma	NDUFA11	0.00165	-2.972663	
5604	adenoma	ADAM28	0.00001	-2.969096	
5605	adenoma	CCDC68	0	-2.964773	
5606	adenoma	ITPR1	0.000003	-2.958448	
5607	adenoma	CTBP1	0.000745	-2.953216	
5608	adenoma	PDE5A	0	-2.953031	
5609	adenoma	HPSE	0.000018	-2.95219	
5610	adenoma	NNMT	0.00001	-2.951044	
5611	adenoma	GJB2	0.000007	-2.946941	
5612	adenoma	SSR1	0.000905	-2.942659	
5613	adenoma	GREM1	0.010844	-2.942333	
5614	adenoma	SFRP2	0.000001	-2.937539	
5615	adenoma	CLIP4	0.000009	-2.937125	
5616	adenoma	CAMK2N1	0	-2.936385	
5617	adenoma	MXI1	0	-2.933559	
5618	adenoma	BRI3BP	0.001018	-2.933366	
5619	adenoma	ISM1	0	-2.933199	
5620	adenoma	ARPC5L	0.000536	-2.924573	
5621	adenoma	SOX10	0.000003	-2.909432	
5622	adenoma	C13orf15	0	-2.90779	
5623	adenoma	MAGT1	0.001062	-2.907141	
5624	adenoma	BCL2A1	0.000003	-2.90518	
5625	adenoma	GIMAP7	0	-2.904896	
5626	adenoma	SPEG	0.000001	-2.903121	
5627	adenoma	BAHCC1	0.000001	-2.897976	
5628	adenoma	ELOVL6	0.000025	-2.895058	
5629	adenoma	AK4	0.00127	-2.893044	
5630	adenoma	EIF3B	0.001726	-2.892745	
5631	adenoma	GALNT12	0	-2.885483	
5632	adenoma	CNN1	0.000235	-2.885419	
5633	adenoma	MTDH	0.002092	-2.883258	
5634	adenoma	PWP1	0.000849	-2.881134	
5635	adenoma	IQCA1	0.000001	-2.880315	
5636	adenoma	COL14A1	0.000027	-2.880001	
5637	adenoma	SSPN	0	-2.878351	
5638	adenoma	FBXO9	0.000279	-2.877366	
5639	adenoma	SART3	0.002181	-2.867926	
5640	adenoma	MCM7	0.001197	-2.864753	
5641	adenoma	YIF1A	0.001408	-2.862812	
5642	adenoma	LOC100132891	0.000007	-2.857789	
5643	adenoma	LRFN4	0.001286	-2.854454	
5644	adenoma	WHSC1	0.001286	-2.851894	
5645	adenoma	ITGA8	0.000005	-2.847531	
5646	adenoma	ZDHHC12	0.002087	-2.845202	
5647	adenoma	HNRNPA3	0.000536	-2.845125	
5648	adenoma	SCD5	0.000011	-2.844648	

5649	adenoma	ATP8B2	0.000011	-2.842167	
5650	adenoma	ARF1	0.000744	-2.842141	
5651	adenoma	GLUD1	0.002122	-2.840846	
5652	adenoma	LOC645323	0.000025	-2.839439	
5653	adenoma	FOXF1	0.000035	-2.838865	
5654	adenoma	ZNF677	0	-2.838379	
5655	adenoma	SLC36A1	0.000001	-2.838295	
5656	adenoma	STOML2	0.002108	-2.83758	
5657	adenoma	RFTN1	0.000018	-2.831749	
5658	adenoma	IL6	0.000005	-2.827798	
5659	adenoma	HHIP	0	-2.822572	
5660	adenoma	HAS1	0.000008	-2.816339	
5661	adenoma	ITM2A	0.000004	-2.813288	
5662	adenoma	MRPL11	0.000588	-2.811379	
5663	adenoma	ATP6V0D1	0.001669	-2.798414	
5664	adenoma	CECR5	0.00013	-2.790449	
5665	adenoma	TBC1D9	0	-2.789546	
5666	adenoma	CCDC8	0.000013	-2.786944	
5667	adenoma	TPM3	0.001005	-2.786129	
5668	adenoma	LRRC16A	0.001885	-2.781349	
5669	adenoma	GLIPR2	0	-2.779996	
5670	adenoma	CASK	0.000677	-2.777413	
5671	adenoma	CPSF4	0.001018	-2.776651	
5672	adenoma	C20orf103	0.000006	-2.775791	
5673	adenoma	PPP2R3A	0	-2.771681	
5674	adenoma	SFPQ	0.000462	-2.769325	
5675	adenoma	HOXD4	0	-2.764491	
5676	adenoma	PAK4	0.001018	-2.764101	
5677	adenoma	SGCE	0	-2.761216	
5678	adenoma	ARPC4	0.000385	-2.760967	
5679	adenoma	KPNB1	0.000526	-2.760426	
5680	adenoma	TNXB	0	-2.759854	
5681	adenoma	FAM120A	0.00162	-2.758222	
5682	adenoma	LCP2	0.000001	-2.756829	
5683	adenoma	PDGFRL	0.000134	-2.755988	
5684	adenoma	CLIC2	0.000004	-2.754874	
5685	adenoma	KLRK1	0.000001	-2.753093	
5686	adenoma	DAZAP1	0.000817	-2.746504	
5687	adenoma	JAM2	0	-2.74367	
5688	adenoma	SRPRB	0.000404	-2.742546	
5689	adenoma	ATG4B	0.000385	-2.741098	
5690	adenoma	A2M	0	-2.737539	
5691	adenoma	SMARCA4	0.000385	-2.735501	
5692	adenoma	PDGFD	0	-2.728425	
5693	adenoma	VPS37C	0.00013	-2.725503	
5694	adenoma	GPR172A	0.001468	-2.717345	
5695	adenoma	RBPMS2	0.000012	-2.716139	
5696	adenoma	FCGR3B	0.000001	-2.715186	
5697	adenoma	ZFP36L2	0.002061	-2.713243	
5698	adenoma	HIC1	0	-2.712245	

5699	adenoma	ST8SIA4	0.000004	-2.708861	
5700	adenoma	FAM101B	0.000016	-2.705595	
5701	adenoma	PDCD4	0	-2.702497	
5702	adenoma	KIAA1211	0	-2.700693	
5703	adenoma	ACP5	0.000011	-2.698381	
5704	adenoma	ANKRD43	0	-2.694987	
5705	adenoma	MMP23A	0	-2.689529	
5706	adenoma	STARD7	0.00001	-2.687573	
5707	adenoma	HLA-DPA1	0.000001	-2.685678	
5708	adenoma	ADCY2	0	-2.684973	
5709	adenoma	MAOA	0	-2.684169	
5710	adenoma	LONP2	0.001468	-2.682588	
5711	adenoma	CDKL1	0	-2.680807	
5712	adenoma	SGK2	0	-2.680358	
5713	adenoma	DOCK2	0.000011	-2.677831	
5714	adenoma	RNPS1	0.000447	-2.670389	
5715	adenoma	QKI	0.000001	-2.669064	
5716	adenoma	DZIP1	0.000004	-2.664127	
5717	adenoma	MAB21L2	0	-2.662187	
5718	adenoma	PRELP	0.000259	-2.66053	
5719	adenoma	TMEM33	0.000576	-2.657315	
5720	adenoma	LOC728392	0.000002	-2.655484	
5721	adenoma	FAM38B	0	-2.654328	
5722	adenoma	SPG20	0.000013	-2.650279	
5723	adenoma	SMPD1	0.000052	-2.647116	
5724	adenoma	HNRNPH1	0.001009	-2.646206	
5725	adenoma	NPAS3	0.000004	-2.641488	
5726	adenoma	MED1	0.000942	-2.641451	
5727	adenoma	KHSRP	0.001874	-2.641428	
5728	adenoma	CEACAM21	0	-2.641099	
5729	adenoma	PPAP2A	0	-2.635656	
5730	adenoma	DCUN1D1	0.002141	-2.63365	
5731	adenoma	CHERP	0.00044	-2.629727	
5732	adenoma	PTBP1	0.000849	-2.62492	
5733	adenoma	MLL5	0.000817	-2.618467	
5734	adenoma	PPIH	0.001617	-2.618016	
5735	adenoma	PLEKHJ1	0.001303	-2.606714	
5736	adenoma	UBE3C	0.000809	-2.604562	
5737	adenoma	FLJ13744	0.000006	-2.603456	
5738	adenoma	ACTR2	0.00013	-2.597419	
5739	adenoma	PEG3	0.000003	-2.596887	
5740	adenoma	WDR5	0.001709	-2.596871	
5741	adenoma	CCNY	0.000251	-2.595556	
5742	adenoma	C15orf61	0.000745	-2.595194	
5743	adenoma	DPYSL3	0.000011	-2.593683	
5744	adenoma	SRI	0	-2.591347	
5745	adenoma	LSP1	0.000003	-2.589832	
5746	adenoma	GIMAP4	0	-2.582242	
5747	adenoma	NLRC3	0.000001	-2.580819	
5748	adenoma	ATP5S	0	-2.576273	

5749	adenoma	TNPO2	0.000536	-2.573375	
5750	adenoma	ITGA4	0	-2.560951	
5751	adenoma	EFEMP1	0.000005	-2.560292	
5752	adenoma	NACAD	0	-2.55511	
5753	adenoma	ITGAL	0.000014	-2.552855	
5754	adenoma	PRICKLE1	0.000006	-2.552477	
5755	adenoma	COL1A2	0.000056	-2.549058	
5756	adenoma	FYB	0.000003	-2.54851	
5757	adenoma	PTPRH	0	-2.546098	
5758	adenoma	IL1RN	0.000005	-2.545738	
5759	adenoma	CCR2	0.000009	-2.545477	
5760	adenoma	WWTR1	0	-2.544909	
5761	adenoma	COL10A1	0.000076	-2.543026	
5762	adenoma	TWIST1	0	-2.542915	
5763	adenoma	LOC100128252	0.000019	-2.538955	
5764	adenoma	REXO1	0.000011	-2.536593	
5765	adenoma	METTL7A	0	-2.536472	
5766	adenoma	FAM55C	0.000009	-2.534637	
5767	adenoma	KLF4	0	-2.531525	
5768	adenoma	CHRNA1	0.000001	-2.530816	
5769	adenoma	FOLR2	0.000043	-2.529423	
5770	adenoma	FOXF2	0.000023	-2.529215	
5771	adenoma	PLCL2	0	-2.528678	
5772	adenoma	PTN	0	-2.525943	
5773	adenoma	PCDH18	0.000016	-2.525923	
5774	adenoma	FPR1	0.000001	-2.518758	
5775	adenoma	PEBP1	0.001299	-2.513655	
5776	adenoma	MRPL12	0.00142	-2.513479	
5777	adenoma	NR3C2	0	-2.508277	
5778	adenoma	CD84	0.000019	-2.50676	
5779	adenoma	DOCK8	0.000023	-2.505324	
5780	adenoma	LOC388796	0.000087	-2.504446	
5781	adenoma	KRT20	0.000067	-2.501505	
5782	adenoma	TRBC1	0.000025	-2.501359	
5783	adenoma	ADCY9	0	-2.496971	
5784	adenoma	RELN	0.000002	-2.493594	
5785	adenoma	SERPINF1	0.000012	-2.489622	
5786	adenoma	SHQ1	0.000588	-2.48934	
5787	adenoma	HNRNPA1	0.000677	-2.488945	
5788	adenoma	PTPLAD2	0	-2.488703	
5789	adenoma	EMILIN1	0.000018	-2.488391	
5790	adenoma	LAPTM5	0.000074	-2.486932	
5791	adenoma	CACNG1	0	-2.483709	
5792	adenoma	TLCD2	0.000008	-2.481418	
5793	adenoma	PSMD13	0.000159	-2.480783	
5794	adenoma	GTPBP1	0.000283	-2.479649	
5795	adenoma	RHOJ	0.000001	-2.478906	
5796	adenoma	SALL1	0.000009	-2.477997	
5797	adenoma	MSN	0.000019	-2.474954	
5798	adenoma	GIMAP8	0.000018	-2.474757	

5799	adenoma	SUMO3	0.000536	-2.472359	
5800	adenoma	RUNX2	0.000176	-2.470737	
5801	adenoma	H2AFY	0.000368	-2.468491	
5802	adenoma	FZD8	0.000042	-2.467017	
5803	adenoma	LOC100506941	0.000211	-2.466491	
5804	adenoma	PGCP	0	-2.464507	
5805	adenoma	TUFM	0.000214	-2.46378	
5806	adenoma	WAS	0.000016	-2.463565	
5807	adenoma	EFS	0.000001	-2.461236	
5808	adenoma	TSPAN3	0.000007	-2.46009	
5809	adenoma	MRPL20	0.000283	-2.456302	
5810	adenoma	CCT2	0.00092	-2.451786	
5811	adenoma	TXNRD1	0.001408	-2.450252	
5812	adenoma	IGSF3	0	-2.449265	
5813	adenoma	C1orf174	0.001726	-2.448482	
5814	adenoma	HNF4A	0	-2.448363	
5815	adenoma	TMEM47	0.000001	-2.445538	
5816	adenoma	WDR43	0.002218	-2.44204	
5817	adenoma	APRT	0.000817	-2.441156	
5818	adenoma	PTGS2	0.000015	-2.437461	
5819	adenoma	ANGPTL4	0.000032	-2.435153	
5820	adenoma	CERK	0	-2.432117	
5821	adenoma	GJC1	0.00003	-2.430532	
5822	adenoma	HLA-DPB1	0.000004	-2.42914	
5823	adenoma	NAMPT	0.000001	-2.429104	
5824	adenoma	IL18BP	0.00002	-2.423986	
5825	adenoma	GLI1	0.000009	-2.42391	
5826	adenoma	FBN1	0.000002	-2.4229	
5827	adenoma	NRK	0.000002	-2.420558	
5828	adenoma	AGTR1	0.000014	-2.417771	
5829	adenoma	LOC389249	0	-2.417206	
5830	adenoma	SUZ12	0.000576	-2.41633	
5831	adenoma	SCAMP1	0.001921	-2.415113	
5832	adenoma	SHE	0.000029	-2.413364	
5833	adenoma	ARHGEF17	0.000001	-2.412614	
5834	adenoma	RAD23B	0.002247	-2.411764	
5835	adenoma	FTH1	0	-2.404593	
5836	adenoma	OLFML2A	0.000002	-2.402962	
5837	adenoma	FAM116A	0.000536	-2.401477	
5838	adenoma	CYSLTR1	0	-2.399178	
5839	adenoma	SRP72	0.000745	-2.397696	
5840	adenoma	CA12	0	-2.396817	
5841	adenoma	MPDZ	0.000005	-2.394859	
5842	adenoma	CHRDL1	0.000014	-2.391992	
5843	adenoma	DUT	0.001823	-2.391851	
5844	adenoma	ACAT1	0	-2.391491	
5845	adenoma	THSD7A	0.000006	-2.39099	
5846	adenoma	LOC100133660	0	-2.389821	
5847	adenoma	SALL2	0.000034	-2.388978	
5848	adenoma	AEBP1	0.000161	-2.388711	

5849	adenoma	NHSL1	0	-2.387015	
5850	adenoma	SYNPO2	0.000116	-2.386825	
5851	adenoma	RPP14	0.000726	-2.382162	
5852	adenoma	CHI3L1	0.00012	-2.381957	
5853	adenoma	SSTR2	0	-2.3807	
5854	adenoma	LDB2	0.000015	-2.378466	
5855	adenoma	GZMA	0.000012	-2.377783	
5856	adenoma	AGTR2	0.000004	-2.37389	
5857	adenoma	ITGBL1	0.000011	-2.373081	
5858	adenoma	UBE2N	0.001886	-2.372987	
5859	adenoma	NADK	0.000881	-2.368055	
5860	adenoma	FAM13C	0.000001	-2.367818	
5861	adenoma	EVL	0.000017	-2.365015	
5862	adenoma	FOXC1	0.00015	-2.36447	
5863	adenoma	NOP14	0.000536	-2.362674	
5864	adenoma	TUBA4A	0.001163	-2.351077	
5865	adenoma	RMND5A	0	-2.349248	
5866	adenoma	RECK	0	-2.348067	
5867	adenoma	FBXO11	0.001033	-2.347149	
5868	adenoma	AAK1	0	-2.345226	
5869	adenoma	EGR4	0.000011	-2.344928	
5870	adenoma	TMCO3	0.001874	-2.344777	
5871	adenoma	CALHM2	0.000001	-2.344725	
5872	adenoma	FMO5	0	-2.34472	
5873	adenoma	PRKAR2A	0.002076	-2.343637	
5874	adenoma	SPARC	0.000001	-2.342933	
5875	adenoma	FAM78A	0.000021	-2.339397	
5876	adenoma	VIM	0	-2.336821	
5877	adenoma	EREG	0.025572	-2.336315	
5878	adenoma	CD3D	0.000015	-2.333476	
5879	adenoma	FAM156A	0.000419	-2.333365	
5880	adenoma	LUM	0.000004	-2.332403	
5881	adenoma	SOCS3	0.000008	-2.329779	
5882	adenoma	NCRNA00294	0	-2.329114	
5883	adenoma	ARF3	0.000656	-2.327501	
5884	adenoma	CLMN	0	-2.326686	
5885	adenoma	COPZ2	0.000012	-2.324949	
5886	adenoma	HSP90AA1	0.002227	-2.323099	
5887	adenoma	SNRPA1	0.001286	-2.320686	
5888	adenoma	COL11A1	0.000058	-2.320574	
5889	adenoma	FBXO17	0.000111	-2.317715	
5890	adenoma	HSPC159	0.000034	-2.315064	
5891	adenoma	CYR61	0.000231	-2.314814	
5892	adenoma	PDIA3	0.002126	-2.314593	
5893	adenoma	MLEC	0.001417	-2.311297	
5894	adenoma	VSTM2L	0.000002	-2.307386	
5895	adenoma	ACOT7	0.00013	-2.307244	
5896	adenoma	ITK	0.00002	-2.305504	
5897	adenoma	GNG7	0	-2.304948	
5898	adenoma	PLAC4	0.000031	-2.303985	

5899	adenoma	CCL3	0.000005	-2.302453	
5900	adenoma	CLEC3B	0.000002	-2.301045	
5901	adenoma	NDUFS3	0.001353	-2.298996	
5902	adenoma	PPP1R3C	0.000007	-2.298722	
5903	adenoma	APOLD1	0.000012	-2.298628	
5904	adenoma	COX8A	0.00142	-2.297831	
5905	adenoma	MORF4L2	0.000279	-2.297727	
5906	adenoma	PAFAH1B2	0.001148	-2.297254	
5907	adenoma	C14orf58	0.000001	-2.296371	
5908	adenoma	RGS4	0.000044	-2.294983	
5909	adenoma	INHBA	0.000049	-2.294081	
5910	adenoma	CMKLR1	0.000252	-2.293257	
5911	adenoma	SPCS3	0.001018	-2.292755	
5912	adenoma	MIER3	0.000002	-2.292537	
5913	adenoma	ITPKA	0.000008	-2.28995	
5914	adenoma	SPTLC1	0.00083	-2.289582	
5915	adenoma	SPPL2A	0.000001	-2.288713	
5916	adenoma	IGSF9	0.000003	-2.288617	
5917	adenoma	EGFR	0.000217	-2.287943	
5918	adenoma	ECSCR	0.000017	-2.284871	
5919	adenoma	IPO9	0.002341	-2.282007	
5920	adenoma	KCP	0.000043	-2.281315	
5921	adenoma	SEC61B	0.000679	-2.280103	
5922	adenoma	SH2B3	0	-2.278978	
5923	adenoma	IRF3	0.000849	-2.278059	
5924	adenoma	STOX2	0.000013	-2.27665	
5925	adenoma	TMEM223	0.001286	-2.274972	
5926	adenoma	UPF1	0.002073	-2.273385	
5927	adenoma	FAM129A	0	-2.273048	
5928	adenoma	EIF4G1	0.00148	-2.272896	
5929	adenoma	NAP1L3	0.000007	-2.269711	
5930	adenoma	LOC100509683	0.000054	-2.268836	
5931	adenoma	SLC31A2	0	-2.267722	
5932	adenoma	PTX3	0.000036	-2.267174	
5933	adenoma	PDLIM4	0	-2.260782	
5934	adenoma	LOC284417	0.046729	-2.257994	
5935	adenoma	VPS4A	0.00206	-2.257618	
5936	adenoma	COL1A1	0.004352	-2.256916	
5937	adenoma	MSRB3	0.000006	-2.25566	
5938	adenoma	CR1	0	-2.252357	
5939	adenoma	DPY30	0.001558	-2.251484	
5940	adenoma	THBS1	0.000001	-2.250588	
5941	adenoma	SURF4	0.000385	-2.249222	
5942	adenoma	RPL8	0.001616	-2.249196	
5943	adenoma	CCT6A	0.002122	-2.248994	
5944	adenoma	RETSAT	0	-2.248071	
5945	adenoma	U2AF2	0.001714	-2.24803	
5946	adenoma	SLC25A23	0	-2.247985	
5947	adenoma	STRADB	0	-2.243845	
5948	adenoma	RBMS1	0	-2.242471	

5949	adenoma	DKK3	0	-2.238933	
5950	adenoma	ARHGAP25	0.000013	-2.238708	
5951	adenoma	SERPING1	0.00012	-2.235442	
5952	adenoma	PNMAL1	0.000007	-2.234743	
5953	adenoma	C2orf28	0.002108	-2.233873	
5954	adenoma	TNFRSF25	0.000015	-2.23356	
5955	adenoma	FNDC4	0.000031	-2.233419	
5956	adenoma	KDELR2	0.00202	-2.233364	
5957	adenoma	IL27RA	0.000041	-2.23143	
5958	adenoma	KRT10	0.000726	-2.228459	
5959	adenoma	TAGLN	0.000196	-2.227987	
5960	adenoma	COL12A1	0.000002	-2.227158	
5961	adenoma	HNRNPM	0.00019	-2.225713	
5962	adenoma	GFPT2	0.000196	-2.225548	
5963	adenoma	FAM195A	0.000905	-2.225473	
5964	adenoma	PPP1CC	0.001286	-2.225143	
5965	adenoma	ST3GAL6	0.000007	-2.217513	
5966	adenoma	CD86	0.000017	-2.21729	
5967	adenoma	C15orf48	0.000019	-2.215116	
5968	adenoma	LOC100507165	0.000001	-2.214475	
5969	adenoma	CRLF1	0.000157	-2.214226	
5970	adenoma	SLC1A3	0.000003	-2.213189	
5971	adenoma	GLOD5	0	-2.212898	
5972	adenoma	CTTN	0.002192	-2.209567	
5973	adenoma	MAGED4	0	-2.208615	
5974	adenoma	DENR	0.000995	-2.207376	
5975	adenoma	CSF3	0.000005	-2.207	
5976	adenoma	EXOSC2	0.001558	-2.205137	
5977	adenoma	AP3D1	0.000335	-2.20501	
5978	adenoma	CCL4	0	-2.204871	
5979	adenoma	RUNX3	0.000003	-2.204119	
5980	adenoma	MS4A6A	0.000006	-2.202884	
5981	adenoma	MEG3	0.000017	-2.201433	
5982	adenoma	GADD45B	0.000001	-2.199071	
5983	adenoma	PPHLN1	0.002183	-2.193358	
5984	adenoma	CILP2	0.000112	-2.193145	
5985	adenoma	C14orf132	0.000001	-2.192972	
5986	adenoma	NPR1	0.000101	-2.192109	
5987	adenoma	SPCS1	0.000905	-2.190451	
5988	adenoma	MRP63	0.000315	-2.189177	
5989	adenoma	TARP	0.000004	-2.185572	
5990	adenoma	SLC16A7	0.000049	-2.185442	
5991	adenoma	NDUFA10	0.001565	-2.184389	
5992	adenoma	FST	0.000004	-2.184259	
5993	adenoma	AKAP12	0	-2.18236	
5994	adenoma	TSPAN4	0.000036	-2.182007	
5995	adenoma	TBCD	0.000002	-2.181333	
5996	adenoma	TROVE2	0.001768	-2.179221	
5997	adenoma	ZMAT2	0.001565	-2.177922	
5998	adenoma	ETS1	0.000009	-2.177302	

5999	adenoma	SLCO2A1	0.000048	-2.17587	
6000	adenoma	NEURL1B	0	-2.170687	
6001	adenoma	ACSS2	0	-2.168338	
6002	adenoma	PLCE1	0	-2.168136	
6003	adenoma	XIAP	0.00003	-2.166723	
6004	adenoma	LOC100510525	0.001987	-2.16276	
6005	adenoma	PLCXD3	0.000162	-2.161158	
6006	adenoma	MTCH1	0.000132	-2.160284	
6007	adenoma	HLA-DOA	0.000026	-2.159713	
6008	adenoma	CD3E	0.000009	-2.159225	
6009	adenoma	CARS	0.001558	-2.154752	
6010	adenoma	OLFML3	0.000002	-2.154377	
6011	adenoma	SPESP1	0.000006	-2.154017	
6012	adenoma	AGXT2L2	0	-2.153903	
6013	adenoma	OSTM1	0.000001	-2.15371	
6014	adenoma	ITGA5	0.000006	-2.152342	
6015	adenoma	HEG1	0.000007	-2.150429	
6016	adenoma	CRYAB	0	-2.149325	
6017	adenoma	HSPA6	0.000025	-2.143799	
6018	adenoma	FMO1	0	-2.143737	
6019	adenoma	RAB3GAP1	0.001286	-2.142946	
6020	adenoma	CCL14-CCL15	0	-2.142142	
6021	adenoma	C2orf7	0.000003	-2.142112	
6022	adenoma	ZC3H4	0.000251	-2.141748	
6023	adenoma	KLF9	0	-2.141047	
6024	adenoma	UBE2E2	0	-2.140143	
6025	adenoma	HNRNPAB	0.001286	-2.139983	
6026	adenoma	MMP19	0	-2.137628	
6027	adenoma	MAGEA4	0.000103	-2.136	
6028	adenoma	AKAP9	0	-2.135119	
6029	adenoma	TMBIM4	0.002319	-2.134269	
6030	adenoma	PLIN5	0.000025	-2.132731	
6031	adenoma	METTL2A	0.002192	-2.131646	
6032	adenoma	C19orf59	0.000056	-2.130184	
6033	adenoma	COL5A2	0.000219	-2.129854	
6034	adenoma	C10orf72	0.000002	-2.12672	
6035	adenoma	ARFGAP2	0.000947	-2.125914	
6036	adenoma	TMEM45B	0	-2.125697	
6037	adenoma	ESRRA	0.00038	-2.122543	
6038	adenoma	C5orf30	0	-2.122045	
6039	adenoma	MYO1A	0	-2.121306	
6040	adenoma	MTCH2	0.000823	-2.118692	
6041	adenoma	CTSC	0.000005	-2.116743	
6042	adenoma	BCAP29	0.000079	-2.114582	
6043	adenoma	KAT2B	0	-2.114163	
6044	adenoma	INPP5A	0.000745	-2.113808	
6045	adenoma	HNRNPD	0.000688	-2.113807	
6046	adenoma	PIP5K1B	0.000059	-2.11295	
6047	adenoma	GSPT1	0.002315	-2.112942	
6048	adenoma	RPN2	0.002197	-2.112401	

6049	adenoma	PER3	0.000003	-2.111868	
6050	adenoma	LHFP	0	-2.111542	
6051	adenoma	9-Sep	0.001886	-2.111405	
6052	adenoma	HTRA1	0.000003	-2.110659	
6053	adenoma	FLI1	0.000014	-2.109904	
6054	adenoma	PREX2	0.000014	-2.108912	
6055	adenoma	TMEM45A	0.000253	-2.108061	
6056	adenoma	UCHL1	0.000017	-2.107934	
6057	adenoma	USP7	0.002313	-2.107195	
6058	adenoma	PTPRM	0.000026	-2.103882	
6059	adenoma	NUDT5	0.001047	-2.100914	
6060	adenoma	PLEK	0.000007	-2.10009	
6061	adenoma	CALD1	0	-2.099286	
6062	adenoma	CMTM3	0.000019	-2.095868	
6063	adenoma	PDE1A	0.000005	-2.095001	
6064	adenoma	DUSP1	0.000027	-2.094565	
6065	adenoma	BASP1	0.000003	-2.092224	
6066	adenoma	SRGN	0.000058	-2.092168	
6067	adenoma	SORCS2	0.000131	-2.088112	
6068	adenoma	TNC	0.000004	-2.086421	
6069	adenoma	EEF2K	0	-2.085721	
6070	adenoma	ANGPT1	0.000001	-2.084996	
6071	adenoma	SEC13	0.00127	-2.082674	
6072	adenoma	ERBB4	0.000028	-2.082636	
6073	adenoma	GEM	0.000202	-2.082297	
6074	adenoma	ZZEF1	0	-2.079989	
6075	adenoma	ST7L	0.000001	-2.079927	
6076	adenoma	SLK	0.002108	-2.079769	
6077	adenoma	PTPN22	0	-2.078373	
6078	adenoma	AP1S2	0	-2.078222	
6079	adenoma	GLI3	0	-2.077675	
6080	adenoma	ZNF331	0.000008	-2.076409	
6081	adenoma	LAYN	0.000006	-2.076001	
6082	adenoma	PRDM1	0.000004	-2.074792	
6083	adenoma	KIAA0495	0.000042	-2.074599	
6084	adenoma	MARCKS	0	-2.070555	
6085	adenoma	SLC41A2	0	-2.06901	
6086	adenoma	CD33	0.000014	-2.066985	
6087	adenoma	ARID1A	0.001362	-2.065637	
6088	adenoma	FUCA1	0.000083	-2.063597	
6089	adenoma	PRRG3	0.000241	-2.063196	
6090	adenoma	PDLIM2	0	-2.062956	
6091	adenoma	NPLOC4	0.00102	-2.061305	
6092	adenoma	KMO	0.000103	-2.06096	
6093	adenoma	S1PR1	0.000005	-2.059119	
6094	adenoma	PEX26	0.000003	-2.0586	
6095	adenoma	GIMAP5	0.000009	-2.058582	
6096	adenoma	TEX261	0.002108	-2.058162	
6097	adenoma	ADAMTS5	0.000002	-2.057966	
6098	adenoma	MOBKL2B	0.000001	-2.057892	

6099	adenoma	NXN	0.000157	-2.057025	
6100	adenoma	FAM69B	0.000183	-2.056589	
6101	adenoma	GPD2	0.000027	-2.05634	
6102	adenoma	SCAMP2	0.000699	-2.054668	
6103	adenoma	ATP2A2	0.000849	-2.054087	
6104	adenoma	KIAA1161	0	-2.053903	
6105	adenoma	CARKD	0.001399	-2.053087	
6106	adenoma	TMEM170A	0	-2.050047	
6107	adenoma	NT5DC1	0.001617	-2.048611	
6108	adenoma	UXS1	0.002092	-2.048549	
6109	adenoma	EIF4E	0.001597	-2.047933	
6110	adenoma	CTSA	0	-2.047801	
6111	adenoma	CNNM4	0.000002	-2.047069	
6112	adenoma	ZEB1	0.000002	-2.044585	
6113	adenoma	ARNTL	0.000073	-2.04229	
6114	adenoma	MYO15B	0.000082	-2.0422	
6115	adenoma	COPZ1	0.001622	-2.040516	
6116	adenoma	MAGEL2	0.000183	-2.039831	
6117	adenoma	FAS	0.000076	-2.038597	
6118	adenoma	HBE1	0.00004	-2.036462	
6119	adenoma	CSF3R	0.000164	-2.03287	
6120	adenoma	ANKRD13A	0.000007	-2.030677	
6121	adenoma	CLEC7A	0.000065	-2.030124	
6122	adenoma	LAMA4	0.000027	-2.027234	
6123	adenoma	POLR2I	0.001754	-2.026762	
6124	adenoma	LAMP1	0.000831	-2.026175	
6125	adenoma	OGN	0.000001	-2.025852	
6126	adenoma	C1orf106	0	-2.024221	
6127	adenoma	UBAP2	0.000536	-2.022488	
6128	adenoma	BCL6	0.000057	-2.021258	
6129	adenoma	PDK4	0.000152	-2.020213	
6130	adenoma	GAGE1	0.000233	-2.019314	
6131	adenoma	TSC22D3	0.000004	-2.018925	
6132	adenoma	MUC3A	0.048315	-2.013011	
6133	adenoma	SAMD4A	0.000086	-2.010683	
6134	adenoma	CIAPIN1	0.00166	-2.010351	
6135	adenoma	C1R	0.000035	-2.002437	
6136	adenoma	CPEB3	0.000056	-2.001948	
6137	adenoma	HSD17B11	0	-2.000595	
6138	adenoma	C6orf105	0.023984	2.001804	
6139	adenoma	PDE4A	0.000001	2.004338	
6140	adenoma	SH3BP4	0.000001	2.007444	
6141	adenoma	STXBP6	0	2.00751	
6142	adenoma	ODC1	0	2.008864	
6143	adenoma	IPO7	0	2.011644	
6144	adenoma	RAN	0	2.014146	
6145	adenoma	PRMT5	0	2.015463	
6146	adenoma	NUDCD1	0	2.036035	
6147	adenoma	BID	0	2.042064	
6148	adenoma	ME3	0.000002	2.043797	

6149	adenoma	FAM84A	0.000001	2.045372	
6150	adenoma	CCDC113	0.000018	2.049514	
6151	adenoma	ATIC	0	2.054203	
6152	adenoma	APIP	0.000003	2.055785	
6153	adenoma	ADH1C	0.039518	2.076173	
6154	adenoma	SOX4	0	2.082992	
6155	adenoma	DCAF13	0	2.083548	
6156	adenoma	HSD11B2	0.016034	2.098063	
6157	adenoma	NIT2	0	2.101524	
6158	adenoma	DARS	0	2.104854	
6159	adenoma	ANKMY2	0.001507	2.10559	
6160	adenoma	NHP2	0	2.111937	
6161	adenoma	GNPAT	0.001005	2.114705	
6162	adenoma	VRK2	0.000279	2.115817	
6163	adenoma	MIF	0	2.132363	
6164	adenoma	FAM114A1	0.001468	2.13396	
6165	adenoma	RPL39	0.000279	2.151141	
6166	adenoma	NUP37	0	2.158289	
6167	adenoma	HIVEP2	0.000897	2.164829	
6168	adenoma	ZFP36L1	0.000002	2.168508	
6169	adenoma	KIAA1468	0.002209	2.169194	
6170	adenoma	ABCE1	0	2.173804	
6171	adenoma	WDR51B	0.000001	2.181121	
6172	adenoma	ZNF703	0	2.1845	
6173	adenoma	MRPS23	0	2.184607	
6174	adenoma	BRWD3	0.000279	2.190952	
6175	adenoma	NBEAL2	0.000001	2.208919	
6176	adenoma	STAMBPL1	0	2.216805	
6177	adenoma	TGIF	0	2.220626	
6178	adenoma	C19orf66	0.000953	2.229038	
6179	adenoma	ATP5C1	0.002218	2.234407	
6180	adenoma	C19orf45	0.000004	2.234818	
6181	adenoma	TMX3	0.000335	2.240365	
6182	adenoma	CCNG2	0.000397	2.244222	
6183	adenoma	TPT1	0.00013	2.24515	
6184	adenoma	C12orf11	0	2.245976	
6185	adenoma	NQO1	0	2.248091	
6186	adenoma	CDK4	0	2.260905	
6187	adenoma	HNMT	0.001714	2.267072	
6188	adenoma	CAPN9	0.002423	2.272972	
6189	adenoma	ID1	0	2.273737	
6190	adenoma	EEF1A1	0.001617	2.283183	
6191	adenoma	NOSTRIN	0.001005	2.292271	
6192	adenoma	CCDC123	0.00005	2.300184	
6193	adenoma	RPL37	0.000934	2.310832	
6194	adenoma	METTL11A	0	2.311416	
6195	adenoma	TBXAS1	0	2.320069	
6196	adenoma	ZC3H14	0.000744	2.331669	
6197	adenoma	PPA1	0	2.331879	
6198	adenoma	SAMD5	0.000007	2.332025	

6199	adenoma	CSE1L	0	2.333721	
6200	adenoma	QTRT1	0.000082	2.345266	
6201	adenoma	ZNF223	0.000608	2.348284	
6202	adenoma	SLC29A1	0.000001	2.350985	
6203	adenoma	RPL23A	0.001018	2.351467	
6204	adenoma	NPM1	0	2.352414	
6205	adenoma	MYC	0.000001	2.361317	
6206	adenoma	C16orf13	0	2.374332	
6207	adenoma	C20orf42	0.000001	2.37868	
6208	adenoma	CIRH1A	0	2.380956	
6209	adenoma	IPO4	0	2.409518	
6210	adenoma	PAFAH2	0.001886	2.419046	
6211	adenoma	NPDC1	0	2.434599	
6212	adenoma	MAN2B2	0.000685	2.456711	
6213	adenoma	MAPK10	0	2.45868	
6214	adenoma	B3GNT6	0.000551	2.474493	
6215	adenoma	TP53	0	2.480542	
6216	adenoma	RNF170	0.002228	2.487114	
6217	adenoma	CHEK1	0	2.489834	
6218	adenoma	CCPG1	0.001018	2.503718	
6219	adenoma	LOC728903	0.000688	2.508559	
6220	adenoma	CARD16	0.001537	2.509046	
6221	adenoma	TCTE3	0.001308	2.520806	
6222	adenoma	CKS2	0	2.538513	
6223	adenoma	GART	0	2.543509	
6224	adenoma	RNF183	0.000008	2.570806	
6225	adenoma	PHKA1	0	2.595054	
6226	adenoma	ND3	0.000825	2.602602	
6227	adenoma	ZNF641	0.000315	2.611286	
6228	adenoma	ABCC1	0	2.612634	
6229	adenoma	KCNN4	0.000001	2.61492	
6230	adenoma	PDZK1IP1	0.000001	2.634638	
6231	adenoma	S100A11	0	2.657114	
6232	adenoma	TMEM161A	0	2.677676	
6233	adenoma	WDR77	0	2.680779	
6234	adenoma	DCAF6	0.000189	2.68267	
6235	adenoma	TRIP11	0.001005	2.69839	
6236	adenoma	TLR4	0.000001	2.710705	
6237	adenoma	LXN	0.000744	2.719226	
6238	adenoma	TSPAN8	0.001874	2.723702	
6239	adenoma	MYOF	0.000937	2.741709	
6240	adenoma	SOX9	0	2.764121	
6241	adenoma	BYSL	0	2.771924	
6242	adenoma	RGPD5	0.001018	2.77218	
6243	adenoma	CRB2	0	2.774161	
6244	adenoma	STAM2	0.002073	2.777863	
6245	adenoma	GUSBP3	0.000995	2.782899	
6246	adenoma	RFK	0	2.784735	
6247	adenoma	MAST4	0.001885	2.785578	
6248	adenoma	PECAM1	0.000662	2.829075	

6249	adenoma	ARID3A	0.000002	2.853683	
6250	adenoma	GLYATL1	0.004167	2.900595	
6251	adenoma	RGNEF	0	2.908313	
6252	adenoma	HOXA10	0.000013	2.919364	
6253	adenoma	C14orf50	0.000315	2.943752	
6254	adenoma	SELL	0.001054	2.986705	
6255	adenoma	SCIN	0.001803	3.022945	
6256	adenoma	ENC1	0	3.029091	
6257	adenoma	FLJ37644	0	3.036109	
6258	adenoma	ST6GALNAC1	0.000551	3.039416	
6259	adenoma	PNN	0.000004	3.054676	
6260	adenoma	TNNC2	0.005876	3.095065	
6261	adenoma	LOC731404	0	3.098819	
6262	adenoma	IGFBP2	0.000006	3.15496	
6263	adenoma	PGAP1	0.001286	3.165192	
6264	adenoma	LOC648541	0.000001	3.171951	
6265	adenoma	GRAMD1A	0	3.245093	
6266	adenoma	GTF2IRD1	0	3.248998	
6267	adenoma	CLIC3	0.000551	3.262252	
6268	adenoma	LOC100510507	0.002263	3.316777	
6269	adenoma	ASB4	0.000032	3.317859	
6270	adenoma	ACSM3	0.001699	3.397599	
6271	adenoma	TEAD4	0	3.449699	
6272	adenoma	PPP5C	0.000526	3.470088	
6273	adenoma	SLC12A2	0	3.496985	
6274	adenoma	GDF15	0	3.513165	
6275	adenoma	ZNF33B	0.000179	3.526628	
6276	adenoma	FOXA1	0.001617	3.654072	
6277	adenoma	ALDH1A1	0.000279	3.695608	
6278	adenoma	LOC253012	0.004352	3.719364	
6279	adenoma	CDH3	0	3.87931	
6280	adenoma	RAD54B	0	3.96964	
6281	adenoma	LYZ	0.000002	4.135976	
6282	adenoma	NFE2L3	0	4.295445	
6283	adenoma	TBX3	0	4.394285	
6284	adenoma	RETNLB	0.004352	4.671211	
6285	adenoma	SLAMF1	0.001573	4.846754	
6286	adenoma	ANKRD37	0.001874	4.910783	
6287	adenoma	DUSP4	0.000002	5.133971	
6288	adenoma	EDN1	0.001647	5.610711	
6289	adenoma	TGFBI	0.000001	5.758242	
6290	adenoma	MB	0.000995	5.842515	
6291	adenoma	OLFM4	0.000001	6.020404	
6292	adenoma	GABRB2	0.000551	6.767267	
6293	adenoma	LGR5	0	7.99232	
6294	adenoma	MMP7	0	23.740355	
6295	Adenoma of large intestine	GUCA2A	0	-28.722896	
6296	Adenoma of large intestine	ABCA8	0	-23.213255	
6297	Adenoma of large intestine	CA1	0	-18.850535	
6298	Adenoma of large intestine	CA4	0	-17.421036	

6299	Adenoma of large intestine	PCK1	0	-16.18203	
6300	Adenoma of large intestine	CDKN2B	0	-14.536097	
6301	Adenoma of large intestine	ADAMDEC1	0	-12.069765	
6302	Adenoma of large intestine	CXCL12	0	-11.210708	
6303	Adenoma of large intestine	EDN3	0	-10.750175	
6304	Adenoma of large intestine	ANK2	0	-9.366054	
6305	Adenoma of large intestine	SLC4A4	0	-9.098508	
6306	Adenoma of large intestine	FBLN1	0	-8.162717	
6307	Adenoma of large intestine	GDPD2	0	-7.595737	
6308	Adenoma of large intestine	CLDN23	0	-7.499048	
6309	Adenoma of large intestine	FXYD1	0	-7.370164	
6310	Adenoma of large intestine	CA2	0	-6.781211	
6311	Adenoma of large intestine	CHP2	0	-6.7631	
6312	Adenoma of large intestine	LOC646627	0	-6.74501	
6313	Adenoma of large intestine	SLC26A2	0	-6.351358	
6314	Adenoma of large intestine	SEMA6D	0	-6.121416	
6315	Adenoma of large intestine	C2orf88	0	-6.019888	
6316	Adenoma of large intestine	PHLPP2	0	-5.956011	
6317	Adenoma of large intestine	SCARA5	0	-5.907949	
6318	Adenoma of large intestine	ANO5	0	-5.903868	
6319	Adenoma of large intestine	SLC26A3	0	-5.850903	
6320	Adenoma of large intestine	NR5A2	0	-5.628608	
6321	Adenoma of large intestine	TSPAN7	0	-5.59846	
6322	Adenoma of large intestine	HAPLN1	0	-5.595542	
6323	Adenoma of large intestine	CPNE8	0	-5.539105	
6324	Adenoma of large intestine	MEIS1	0	-5.519158	
6325	Adenoma of large intestine	CP	0	-5.496611	
6326	Adenoma of large intestine	ZG16	0	-5.473881	
6327	Adenoma of large intestine	EDIL3	0	-5.471718	
6328	Adenoma of large intestine	DPP10	0	-5.255341	
6329	Adenoma of large intestine	LRRK2	0	-5.211608	
6330	Adenoma of large intestine	C14orf139	0	-5.19712	
6331	Adenoma of large intestine	PKIB	0	-5.194257	
6332	Adenoma of large intestine	MYLK	0	-5.191709	
6333	Adenoma of large intestine	ABI3BP	0	-5.15203	
6334	Adenoma of large intestine	HPGD	0	-4.952038	
6335	Adenoma of large intestine	PAG1	0	-4.888206	
6336	Adenoma of large intestine	TMEM171	0	-4.881186	
6337	Adenoma of large intestine	EPB41L3	0	-4.876504	
6338	Adenoma of large intestine	DHRS11	0	-4.865647	
6339	Adenoma of large intestine	NR3C1	0	-4.840088	
6340	Adenoma of large intestine	ITIH5	0	-4.713737	
6341	Adenoma of large intestine	LPAR1	0	-4.679545	
6342	Adenoma of large intestine	CEACAM7	0	-4.676861	
6343	Adenoma of large intestine	WSCD1	0	-4.610428	
6344	Adenoma of large intestine	NDN	0	-4.581847	
6345	Adenoma of large intestine	RUNDC3B	0	-4.530686	
6346	Adenoma of large intestine	CNNM2	0	-4.530382	
6347	Adenoma of large intestine	SETBP1	0	-4.422574	
6348	Adenoma of large intestine	COLEC12	0	-4.307452	

6349	Adenoma of large intestine	RTN1	0	-4.266108	
6350	Adenoma of large intestine	CES2	0	-4.196009	
6351	Adenoma of large intestine	KCTD12	0	-4.172007	
6352	Adenoma of large intestine	PDE2A	0	-4.168294	
6353	Adenoma of large intestine	TMCC3	0	-4.090314	
6354	Adenoma of large intestine	GPX3	0	-4.07824	
6355	Adenoma of large intestine	SPARCL1	0	-3.9365	
6356	Adenoma of large intestine	MEP1A	0	-3.928076	
6357	Adenoma of large intestine	IGF1	0	-3.926801	
6358	Adenoma of large intestine	FGF9	0	-3.874986	
6359	Adenoma of large intestine	RGMA	0	-3.847742	
6360	Adenoma of large intestine	SNAI2	0	-3.844742	
6361	Adenoma of large intestine	MALL	0	-3.830649	
6362	Adenoma of large intestine	UNC5C	0	-3.802816	
6363	Adenoma of large intestine	MGAT4A	0	-3.779783	
6364	Adenoma of large intestine	ARHGAP20	0	-3.749217	
6365	Adenoma of large intestine	AQP1	0	-3.717151	
6366	Adenoma of large intestine	IL6R	0	-3.695784	
6367	Adenoma of large intestine	ZEB2	0	-3.603844	
6368	Adenoma of large intestine	CCL14	0	-3.57784	
6369	Adenoma of large intestine	IL1R2	0	-3.568313	
6370	Adenoma of large intestine	CLIC5	0	-3.536466	
6371	Adenoma of large intestine	NAAA	0	-3.52473	
6372	Adenoma of large intestine	VIPR1	0	-3.513648	
6373	Adenoma of large intestine	HIGD1A	0	-3.491594	
6374	Adenoma of large intestine	FGL2	0	-3.475052	
6375	Adenoma of large intestine	RUNX1T1	0	-3.43287	
6376	Adenoma of large intestine	DCN	0	-3.426964	
6377	Adenoma of large intestine	AHCYL2	0	-3.42458	
6378	Adenoma of large intestine	SMPDL3A	0	-3.413709	
6379	Adenoma of large intestine	PDE7B	0	-3.311536	
6380	Adenoma of large intestine	MAF	0	-3.308581	
6381	Adenoma of large intestine	POSTN	0	-3.273193	
6382	Adenoma of large intestine	LOC401022	0	-3.262621	
6383	Adenoma of large intestine	SEMA6A	0	-3.255544	
6384	Adenoma of large intestine	RAB37	0	-3.23089	
6385	Adenoma of large intestine	PRKAR2B	0	-3.223903	
6386	Adenoma of large intestine	MS4A7	0	-3.165402	
6387	Adenoma of large intestine	RELL1	0	-3.162769	
6388	Adenoma of large intestine	PADI2	0	-3.121707	
6389	Adenoma of large intestine	RCAN2	0	-3.119317	
6390	Adenoma of large intestine	RBMS3	0	-3.119037	
6391	Adenoma of large intestine	PLA2G10	0	-3.086968	
6392	Adenoma of large intestine	CNRIP1	0	-3.067872	
6393	Adenoma of large intestine	PLOD2	0	-3.053873	
6394	Adenoma of large intestine	APPL2	0	-3.042915	
6395	Adenoma of large intestine	LRRC19	0	-3.037023	
6396	Adenoma of large intestine	GNB4	0	-3.011228	
6397	Adenoma of large intestine	HDAC9	0	-3.003966	
6398	Adenoma of large intestine	PAQR5	0	-2.981438	

6399	Adenoma of large intestine	MATN2	0	-2.980745	
6400	Adenoma of large intestine	CCDC68	0	-2.964773	
6401	Adenoma of large intestine	PRDX6	0	-2.956727	
6402	Adenoma of large intestine	DDR2	0	-2.955575	
6403	Adenoma of large intestine	PDE5A	0	-2.953031	
6404	Adenoma of large intestine	SLC9A9	0	-2.950103	
6405	Adenoma of large intestine	GGTA1	0	-2.9497	
6406	Adenoma of large intestine	TTC22	0	-2.946713	
6407	Adenoma of large intestine	CAMK2N1	0	-2.936385	
6408	Adenoma of large intestine	MXI1	0	-2.933559	
6409	Adenoma of large intestine	GALNT12	0	-2.885483	
6410	Adenoma of large intestine	SSPN	0	-2.878351	
6411	Adenoma of large intestine	MRC1	0	-2.839779	
6412	Adenoma of large intestine	HHIP	0	-2.822572	
6413	Adenoma of large intestine	GLIPR2	0	-2.779996	
6414	Adenoma of large intestine	PPP2R3A	0	-2.771681	
6415	Adenoma of large intestine	SGCE	0	-2.761216	
6416	Adenoma of large intestine	A2M	0	-2.737539	
6417	Adenoma of large intestine	PDGFD	0	-2.728425	
6418	Adenoma of large intestine	TIMP2	0	-2.720069	
6419	Adenoma of large intestine	SYNC	0	-2.707972	
6420	Adenoma of large intestine	PDCD4	0	-2.702497	
6421	Adenoma of large intestine	KIAA1211	0	-2.700693	
6422	Adenoma of large intestine	ANKRD43	0	-2.694987	
6423	Adenoma of large intestine	AKT3	0	-2.686562	
6424	Adenoma of large intestine	SLC46A3	0	-2.685321	
6425	Adenoma of large intestine	MAOA	0	-2.684169	
6426	Adenoma of large intestine	CDKL1	0	-2.680807	
6427	Adenoma of large intestine	SGK2	0	-2.680358	
6428	Adenoma of large intestine	ZCCHC24	0	-2.660645	
6429	Adenoma of large intestine	SLC30A4	0	-2.646617	
6430	Adenoma of large intestine	RGL1	0	-2.64603	
6431	Adenoma of large intestine	PPAP2A	0	-2.635656	
6432	Adenoma of large intestine	C6orf204	0	-2.60794	
6433	Adenoma of large intestine	SRI	0	-2.591347	
6434	Adenoma of large intestine	ATP5S	0	-2.576273	
6435	Adenoma of large intestine	SSBP2	0	-2.561707	
6436	Adenoma of large intestine	FNBP1	0	-2.557199	
6437	Adenoma of large intestine	PTPRH	0	-2.546098	
6438	Adenoma of large intestine	METTL7A	0	-2.536472	
6439	Adenoma of large intestine	KLF4	0	-2.531525	
6440	Adenoma of large intestine	PLCL2	0	-2.528678	
6441	Adenoma of large intestine	NR3C2	0	-2.508277	
6442	Adenoma of large intestine	ANTXR1	0	-2.49782	
6443	Adenoma of large intestine	ADCY9	0	-2.496971	
6444	Adenoma of large intestine	PTPLAD2	0	-2.488703	
6445	Adenoma of large intestine	PGCP	0	-2.464507	
6446	Adenoma of large intestine	ITM2C	0	-2.45619	
6447	Adenoma of large intestine	IGSF3	0	-2.449265	
6448	Adenoma of large intestine	HNF4A	0	-2.448363	

6449	Adenoma of large intestine	CERK	0	-2.432117	
6450	Adenoma of large intestine	GNA11	0	-2.408995	
6451	Adenoma of large intestine	FTH1	0	-2.404593	
6452	Adenoma of large intestine	CA12	0	-2.396817	
6453	Adenoma of large intestine	ACAT1	0	-2.391491	
6454	Adenoma of large intestine	LOC100133660	0	-2.389821	
6455	Adenoma of large intestine	NHSL1	0	-2.387015	
6456	Adenoma of large intestine	BCL2L11	0	-2.372495	
6457	Adenoma of large intestine	SLC2A13	0	-2.35371	
6458	Adenoma of large intestine	RMND5A	0	-2.349248	
6459	Adenoma of large intestine	AAK1	0	-2.345226	
6460	Adenoma of large intestine	FMO5	0	-2.34472	
6461	Adenoma of large intestine	VIM	0	-2.336821	
6462	Adenoma of large intestine	NCRNA00294	0	-2.329114	
6463	Adenoma of large intestine	CLMN	0	-2.326686	
6464	Adenoma of large intestine	TMEM133	0	-2.318497	
6465	Adenoma of large intestine	GNG7	0	-2.304948	
6466	Adenoma of large intestine	SH2B3	0	-2.278978	
6467	Adenoma of large intestine	GLTP	0	-2.27136	
6468	Adenoma of large intestine	SLC31A2	0	-2.267722	
6469	Adenoma of large intestine	RETSAT	0	-2.248071	
6470	Adenoma of large intestine	SLC25A23	0	-2.247985	
6471	Adenoma of large intestine	STRADB	0	-2.243845	
6472	Adenoma of large intestine	GLOD5	0	-2.212898	
6473	Adenoma of large intestine	CYLD	0	-2.180294	
6474	Adenoma of large intestine	UGP2	0	-2.178624	
6475	Adenoma of large intestine	NEURL1B	0	-2.170687	
6476	Adenoma of large intestine	ACSS2	0	-2.168338	
6477	Adenoma of large intestine	PLCE1	0	-2.168136	
6478	Adenoma of large intestine	AGXT2L2	0	-2.153903	
6479	Adenoma of large intestine	CCL14-CCL15	0	-2.142142	
6480	Adenoma of large intestine	KLF9	0	-2.141047	
6481	Adenoma of large intestine	AKAP9	0	-2.135119	
6482	Adenoma of large intestine	TMEM45B	0	-2.125697	
6483	Adenoma of large intestine	C5orf30	0	-2.122045	
6484	Adenoma of large intestine	MYO1A	0	-2.121306	
6485	Adenoma of large intestine	KAT2B	0	-2.114163	
6486	Adenoma of large intestine	EEF2K	0	-2.085721	
6487	Adenoma of large intestine	ZZEF1	0	-2.079989	
6488	Adenoma of large intestine	MARCKS	0	-2.070555	
6489	Adenoma of large intestine	SLC41A2	0	-2.06901	
6490	Adenoma of large intestine	KIAA1161	0	-2.053903	
6491	Adenoma of large intestine	TMEM170A	0	-2.050047	
6492	Adenoma of large intestine	CTSA	0	-2.047801	
6493	Adenoma of large intestine	C1orf106	0	-2.024221	
6494	Adenoma of large intestine	HSD17B11	0	-2.000595	
6495	Adenoma of large intestine	IPO7	0	2.011644	
6496	Adenoma of large intestine	RAN	0	2.014146	
6497	Adenoma of large intestine	PRMT5	0	2.015463	
6498	Adenoma of large intestine	NUDCD1	0	2.036035	

6499	Adenoma of large intestine	BID	0	2.042064	
6500	Adenoma of large intestine	ATIC	0	2.054203	
6501	Adenoma of large intestine	DCAF13	0	2.083548	
6502	Adenoma of large intestine	NIT2	0	2.101524	
6503	Adenoma of large intestine	DKC1	0	2.10166	
6504	Adenoma of large intestine	DARS	0	2.104854	
6505	Adenoma of large intestine	GPX2	0	2.105398	
6506	Adenoma of large intestine	NHP2	0	2.111937	
6507	Adenoma of large intestine	MIF	0	2.132363	
6508	Adenoma of large intestine	NUP37	0	2.158289	
6509	Adenoma of large intestine	ABCE1	0	2.173804	
6510	Adenoma of large intestine	ZNF703	0	2.1845	
6511	Adenoma of large intestine	MRPS23	0	2.184607	
6512	Adenoma of large intestine	STAMBPL1	0	2.216805	
6513	Adenoma of large intestine	C12orf11	0	2.245976	
6514	Adenoma of large intestine	CDK4	0	2.260905	
6515	Adenoma of large intestine	ID1	0	2.273737	
6516	Adenoma of large intestine	METTL11A	0	2.311416	
6517	Adenoma of large intestine	PPA1	0	2.331879	
6518	Adenoma of large intestine	CSE1L	0	2.333721	
6519	Adenoma of large intestine	NPM1	0	2.352414	
6520	Adenoma of large intestine	C16orf13	0	2.374332	
6521	Adenoma of large intestine	CIRH1A	0	2.380956	
6522	Adenoma of large intestine	IPO4	0	2.409518	
6523	Adenoma of large intestine	ITGA6	0	2.419872	
6524	Adenoma of large intestine	CHEK1	0	2.489834	
6525	Adenoma of large intestine	CKS2	0	2.538513	
6526	Adenoma of large intestine	GART	0	2.543509	
6527	Adenoma of large intestine	PHKA1	0	2.595054	
6528	Adenoma of large intestine	FERMT1	0	2.605975	
6529	Adenoma of large intestine	ABCC1	0	2.612634	
6530	Adenoma of large intestine	TMEM161A	0	2.677676	
6531	Adenoma of large intestine	WDR77	0	2.680779	
6532	Adenoma of large intestine	BYSL	0	2.771924	
6533	Adenoma of large intestine	ENC1	0	3.029091	
6534	Adenoma of large intestine	GTF2IRD1	0	3.248998	
6535	Adenoma of large intestine	MET	0	3.407449	
6536	Adenoma of large intestine	NEBL	0	3.427539	
6537	Adenoma of large intestine	TEAD4	0	3.449699	
6538	Adenoma of large intestine	ANXA3	0	3.510112	
6539	Adenoma of large intestine	RNF43	0	3.789269	
6540	Adenoma of large intestine	RAD54B	0	3.96964	
6541	Adenoma of large intestine	SORD	0	3.981631	
6542	Adenoma of large intestine	NFE2L3	0	4.295445	
6543	Adenoma of large intestine	CD44	0	4.366169	
6544	Adenoma of large intestine	S100P	0	4.459644	
6545	Adenoma of large intestine	GALNT6	0	5.744695	
6546	Adenoma of large intestine	AXIN2	0	6.490564	
6547	Adenoma, Villous	SAA1	0.00016	-9.645666	
6548	Adenoma, Villous	MAGEA3	0.000022	-8.032836	

6549	Adenoma, Villous	MAGEA6	0.00001	-7.748368	
6550	Adenoma, Villous	GPR109B	0	-6.520218	
6551	Adenoma, Villous	CXCL5	0	-4.838213	
6552	Adenoma, Villous	SERPINE1	0	-4.593549	
6553	Adenoma, Villous	AQP9	0.000002	-4.184974	
6554	Adenoma, Villous	H19	0.000013	-4.174771	
6555	Adenoma, Villous	MAGEA2	0.000008	-3.948847	
6556	Adenoma, Villous	CDH2	0	-3.829926	
6557	Adenoma, Villous	MAGEA12	0.000002	-3.774247	
6558	Adenoma, Villous	CYP1B1	0.000036	-3.743165	
6559	Adenoma, Villous	SOD2	0	-3.701291	
6560	Adenoma, Villous	BCHE	0.000066	-3.695978	
6561	Adenoma, Villous	COMP	0.000063	-3.625885	
6562	Adenoma, Villous	C2orf40	0.000056	-3.580672	
6563	Adenoma, Villous	GAS1	0.000002	-3.452579	
6564	Adenoma, Villous	CD300A	0.000001	-3.383942	
6565	Adenoma, Villous	S100A8	0.000001	-3.352349	
6566	Adenoma, Villous	LOC100506621	0.000005	-3.233709	
6567	Adenoma, Villous	KCNIP2	0.000042	-3.119855	
6568	Adenoma, Villous	HTR2B	0.000205	-3.10838	
6569	Adenoma, Villous	CCL18	0.000032	-3.10227	
6570	Adenoma, Villous	DES	0.000264	-3.063024	
6571	Adenoma, Villous	HSD11B1	0.000217	-3.061173	
6572	Adenoma, Villous	C5orf46	0.000007	-3.04792	
6573	Adenoma, Villous	TNFAIP6	0	-3.046939	
6574	Adenoma, Villous	BCAT1	0.000001	-3.015072	
6575	Adenoma, Villous	FAM20A	0.000019	-2.998583	
6576	Adenoma, Villous	PROK2	0.0001	-2.985051	
6577	Adenoma, Villous	NNMT	0.00001	-2.951044	
6578	Adenoma, Villous	ISM1	0	-2.933199	
6579	Adenoma, Villous	C13orf15	0	-2.90779	
6580	Adenoma, Villous	BCL2A1	0.000003	-2.90518	
6581	Adenoma, Villous	BAHCC1	0.000001	-2.897976	
6582	Adenoma, Villous	CNN1	0.000235	-2.885419	
6583	Adenoma, Villous	IQCA1	0.000001	-2.880315	
6584	Adenoma, Villous	CFH	0.000006	-2.86537	
6585	Adenoma, Villous	APOE	0.000001	-2.846108	
6586	Adenoma, Villous	LOC645323	0.000025	-2.839439	
6587	Adenoma, Villous	IL6	0.000005	-2.827798	
6588	Adenoma, Villous	HAS1	0.000008	-2.816339	
6589	Adenoma, Villous	C20orf103	0.000006	-2.775791	
6590	Adenoma, Villous	LCP2	0.000001	-2.756829	
6591	Adenoma, Villous	PDGFRL	0.000134	-2.755988	
6592	Adenoma, Villous	RBPMS2	0.000012	-2.716139	
6593	Adenoma, Villous	FCGR3B	0.000001	-2.715186	
6594	Adenoma, Villous	MMP23A	0	-2.689529	
6595	Adenoma, Villous	PRELP	0.000259	-2.66053	
6596	Adenoma, Villous	FLJ13744	0.000006	-2.603456	
6597	Adenoma, Villous	POSTN	0.000006	-2.598065	
6598	Adenoma, Villous	DPYSL3	0.000011	-2.593683	

6599	Adenoma, Villous	MYLK	0.000003	-2.57498	
6600	Adenoma, Villous	NACAD	0	-2.55511	
6601	Adenoma, Villous	COL1A2	0.000056	-2.549058	
6602	Adenoma, Villous	IL1RN	0.000005	-2.545738	
6603	Adenoma, Villous	COL10A1	0.000076	-2.543026	
6604	Adenoma, Villous	TWIST1	0	-2.542915	
6605	Adenoma, Villous	REXO1	0.000011	-2.536593	
6606	Adenoma, Villous	FPR1	0.000001	-2.518758	
6607	Adenoma, Villous	LOC388796	0.000087	-2.504446	
6608	Adenoma, Villous	PTGIS	0.000007	-2.490377	
6609	Adenoma, Villous	PDLIM3	0.000001	-2.484118	
6610	Adenoma, Villous	CACNG1	0	-2.483709	
6611	Adenoma, Villous	RUNX2	0.000176	-2.470737	
6612	Adenoma, Villous	LOC100506941	0.000211	-2.466491	
6613	Adenoma, Villous	PTGS2	0.000015	-2.437461	
6614	Adenoma, Villous	BGN	0.000009	-2.436523	
6615	Adenoma, Villous	ANGPTL4	0.000032	-2.435153	
6616	Adenoma, Villous	CD163	0.000005	-2.433077	
6617	Adenoma, Villous	GJC1	0.00003	-2.430532	
6618	Adenoma, Villous	NAMPT	0.000001	-2.429104	
6619	Adenoma, Villous	NRK	0.000002	-2.420558	
6620	Adenoma, Villous	AGTR1	0.000014	-2.417771	
6621	Adenoma, Villous	SLIT2	0	-2.388864	
6622	Adenoma, Villous	AEBP1	0.000161	-2.388711	
6623	Adenoma, Villous	SYNPO2	0.000116	-2.386825	
6624	Adenoma, Villous	CHI3L1	0.00012	-2.381957	
6625	Adenoma, Villous	AGTR2	0.000004	-2.37389	
6626	Adenoma, Villous	ITGBL1	0.000011	-2.373081	
6627	Adenoma, Villous	FOXC1	0.00015	-2.36447	
6628	Adenoma, Villous	EGR4	0.000011	-2.344928	
6629	Adenoma, Villous	SOCS3	0.000008	-2.329779	
6630	Adenoma, Villous	COPZ2	0.000012	-2.324949	
6631	Adenoma, Villous	COL11A1	0.000058	-2.320574	
6632	Adenoma, Villous	FBXO17	0.000111	-2.317715	
6633	Adenoma, Villous	CYR61	0.000231	-2.314814	
6634	Adenoma, Villous	ANTXR1	0.000156	-2.309902	
6635	Adenoma, Villous	PAPPA	0.000133	-2.308965	
6636	Adenoma, Villous	VSTM2L	0.000002	-2.307386	
6637	Adenoma, Villous	LOX	0.000121	-2.30601	
6638	Adenoma, Villous	PLAC4	0.000031	-2.303985	
6639	Adenoma, Villous	CCL3	0.000005	-2.302453	
6640	Adenoma, Villous	APOLD1	0.000012	-2.298628	
6641	Adenoma, Villous	RGS4	0.000044	-2.294983	
6642	Adenoma, Villous	INHBA	0.000049	-2.294081	
6643	Adenoma, Villous	CMKLR1	0.000252	-2.293257	
6644	Adenoma, Villous	EGFR	0.000217	-2.287943	
6645	Adenoma, Villous	KCP	0.000043	-2.281315	
6646	Adenoma, Villous	FAM129A	0	-2.273048	
6647	Adenoma, Villous	PTX3	0.000036	-2.267174	
6648	Adenoma, Villous	ZFPM2	0.000152	-2.264309	

6649	Adenoma, Villous	MEIS1	0.000027	-2.261566	
6650	Adenoma, Villous	PDLIM4	0	-2.260782	
6651	Adenoma, Villous	CR1	0	-2.252357	
6652	Adenoma, Villous	SERPING1	0.00012	-2.235442	
6653	Adenoma, Villous	PNMAL1	0.000007	-2.234743	
6654	Adenoma, Villous	FNDC4	0.000031	-2.233419	
6655	Adenoma, Villous	TNS1	0.000193	-2.232016	
6656	Adenoma, Villous	IL27RA	0.000041	-2.23143	
6657	Adenoma, Villous	TAGLN	0.000196	-2.227987	
6658	Adenoma, Villous	COL12A1	0.000002	-2.227158	
6659	Adenoma, Villous	GFPT2	0.000196	-2.225548	
6660	Adenoma, Villous	ABCA6	0.000186	-2.222895	
6661	Adenoma, Villous	LOC100507165	0.000001	-2.214475	
6662	Adenoma, Villous	CRLF1	0.000157	-2.214226	
6663	Adenoma, Villous	SLC1A3	0.000003	-2.213189	
6664	Adenoma, Villous	FAM107A	0.000189	-2.210349	
6665	Adenoma, Villous	MAGED4	0	-2.208615	
6666	Adenoma, Villous	CSF3	0.000005	-2.207	
6667	Adenoma, Villous	CCL4	0	-2.204871	
6668	Adenoma, Villous	RUNX3	0.000003	-2.204119	
6669	Adenoma, Villous	MEG3	0.000017	-2.201433	
6670	Adenoma, Villous	ADAMTS1	0.000128	-2.201077	
6671	Adenoma, Villous	GADD45B	0.000001	-2.199071	
6672	Adenoma, Villous	SNAP25	0.00003	-2.19496	
6673	Adenoma, Villous	CILP2	0.000112	-2.193145	
6674	Adenoma, Villous	NPR1	0.000101	-2.192109	
6675	Adenoma, Villous	C11orf96	0.000186	-2.192061	
6676	Adenoma, Villous	SLC16A7	0.000049	-2.185442	
6677	Adenoma, Villous	FST	0.000004	-2.184259	
6678	Adenoma, Villous	TBCD	0.000002	-2.181333	
6679	Adenoma, Villous	AMOTL1	0.000013	-2.173702	
6680	Adenoma, Villous	CYS1	0.000098	-2.164214	
6681	Adenoma, Villous	PLCXD3	0.000162	-2.161158	
6682	Adenoma, Villous	RARRES2	0.000071	-2.157	
6683	Adenoma, Villous	SPESP1	0.000006	-2.154017	
6684	Adenoma, Villous	ITGA5	0.000006	-2.152342	
6685	Adenoma, Villous	HSPA6	0.000025	-2.143799	
6686	Adenoma, Villous	FMO1	0	-2.143737	
6687	Adenoma, Villous	PDGFRA	0.000061	-2.140299	
6688	Adenoma, Villous	MMP19	0	-2.137628	
6689	Adenoma, Villous	MAGEA4	0.000103	-2.136	
6690	Adenoma, Villous	PLIN5	0.000025	-2.132731	
6691	Adenoma, Villous	C19orf59	0.000056	-2.130184	
6692	Adenoma, Villous	COL5A2	0.000219	-2.129854	
6693	Adenoma, Villous	CCDC80	0.000019	-2.125219	
6694	Adenoma, Villous	HTRA1	0.000003	-2.110659	
6695	Adenoma, Villous	TMEM45A	0.000253	-2.108061	
6696	Adenoma, Villous	UCHL1	0.000017	-2.107934	
6697	Adenoma, Villous	PLEK	0.000007	-2.10009	
6698	Adenoma, Villous	CLEC2B	0.000009	-2.096686	

6699	Adenoma, Villous	DUSP1	0.000027	-2.094565	
6700	Adenoma, Villous	BASP1	0.000003	-2.092224	
6701	Adenoma, Villous	SRGN	0.000058	-2.092168	
6702	Adenoma, Villous	SORCS2	0.000131	-2.088112	
6703	Adenoma, Villous	TNC	0.000004	-2.086421	
6704	Adenoma, Villous	ANKRD6	0.000012	-2.085973	
6705	Adenoma, Villous	ERBB4	0.000028	-2.082636	
6706	Adenoma, Villous	GEM	0.000202	-2.082297	
6707	Adenoma, Villous	ZNF331	0.000008	-2.076409	
6708	Adenoma, Villous	SDPR	0.000164	-2.07585	
6709	Adenoma, Villous	KIAA0495	0.000042	-2.074599	
6710	Adenoma, Villous	CD33	0.000014	-2.066985	
6711	Adenoma, Villous	PRRG3	0.000241	-2.063196	
6712	Adenoma, Villous	KMO	0.000103	-2.06096	
6713	Adenoma, Villous	ADAMTS5	0.000002	-2.057966	
6714	Adenoma, Villous	SGK1	0.00007	-2.057487	
6715	Adenoma, Villous	NXN	0.000157	-2.057025	
6716	Adenoma, Villous	FAM69B	0.000183	-2.056589	
6717	Adenoma, Villous	DDR2	0.000006	-2.040428	
6718	Adenoma, Villous	MAGEL2	0.000183	-2.039831	
6719	Adenoma, Villous	HBE1	0.00004	-2.036462	
6720	Adenoma, Villous	FERMT2	0.000147	-2.035527	
6721	Adenoma, Villous	CSF3R	0.000164	-2.03287	
6722	Adenoma, Villous	CLEC7A	0.000065	-2.030124	
6723	Adenoma, Villous	FN1	0	-2.022209	
6724	Adenoma, Villous	BCL6	0.000057	-2.021258	
6725	Adenoma, Villous	PDK4	0.000152	-2.020213	
6726	Adenoma, Villous	GAGE1	0.000233	-2.019314	
6727	Adenoma, Villous	TIMP3	0.000002	-2.018156	
6728	Adenoma, Villous	SAMD4A	0.000086	-2.010683	
6729	Adenoma, Villous	C1R	0.000035	-2.002437	
6730	Analysis of substances	ARHGEF35	0	-7.488002	
6731	Analysis of substances	EXTL2	0	-5.523496	
6732	Analysis of substances	LAYN	0	-5.380104	
6733	Analysis of substances	COCH	0	-4.365475	
6734	Analysis of substances	F2RL2	0	-4.358104	
6735	Analysis of substances	FOLR2	0	-4.354878	
6736	Analysis of substances	OLFML1	0	-4.210707	
6737	Analysis of substances	ADAM22	0	-4.146953	
6738	Analysis of substances	APOBEC3G	0	-3.994392	
6739	Analysis of substances	CD209	0	-3.810027	
6740	Analysis of substances	ARL6	0	-3.805882	
6741	Analysis of substances	ARSK	0	-3.787382	
6742	Analysis of substances	FAM98B	0	-3.784455	
6743	Analysis of substances	DUSP19	0	-3.783262	
6744	Analysis of substances	CACNA2D1	0	-3.776128	
6745	Analysis of substances	GLMN	0	-3.7706	
6746	Analysis of substances	FAM38B	0	-3.68497	
6747	Analysis of substances	PART1	0	-3.476514	
6748	Analysis of substances	NUDCD2	0	-3.462796	

6749	Analysis of substances	DMRT2	0	-3.445976	
6750	Analysis of substances	ERI1	0	-3.412467	
6751	Analysis of substances	NCAM2	0	-3.409691	
6752	Analysis of substances	ADAMTS5	0	-3.320862	
6753	Analysis of substances	SSX2IP	0	-3.293521	
6754	Analysis of substances	ZMYM1	0	-3.175626	
6755	Analysis of substances	AFAP1-AS	0	-3.158008	
6756	Analysis of substances	ARSB	0	-3.157866	
6757	Analysis of substances	STK33	0	-3.15434	
6758	Analysis of substances	LRCH2	0	-3.13753	
6759	Analysis of substances	ELAC1	0	-3.133587	
6760	Analysis of substances	C2orf3	0	-3.119606	
6761	Analysis of substances	KLHL4	0	-3.115538	
6762	Analysis of substances	NEGR1	0	-3.109224	
6763	Analysis of substances	TMEM217	0	-3.058297	
6764	Analysis of substances	JAZF1	0	-3.055828	
6765	Analysis of substances	GABRB2	0	-3.038167	
6766	Analysis of substances	CDH13	0	-3.037865	
6767	Analysis of substances	FLRT2	0	-2.998259	
6768	Analysis of substances	SCRN3	0	-2.975479	
6769	Analysis of substances	IFT81	0	-2.948432	
6770	Analysis of substances	SNTB2	0	-2.933168	
6771	Analysis of substances	NIPAL3	0	-2.928013	
6772	Analysis of substances	EFCAB7	0	-2.910264	
6773	Analysis of substances	TIAM1	0	-2.890659	
6774	Analysis of substances	ALS2CR4	0	-2.845566	
6775	Analysis of substances	NEK11	0	-2.807794	
6776	Analysis of substances	POPDC2	0	-2.773582	
6777	Analysis of substances	ARMCX2	0	-2.749416	
6778	Analysis of substances	L2HGDH	0	-2.740655	
6779	Analysis of substances	ENPEP	0	-2.720477	
6780	Analysis of substances	ANKRD29	0	-2.705061	
6781	Analysis of substances	ZNF568	0	-2.70436	
6782	Analysis of substances	CSTA	0	-2.695119	
6783	Analysis of substances	NLGN4X	0	-2.688744	
6784	Analysis of substances	ADIPOQ	0	-2.688394	
6785	Analysis of substances	CLCN4	0	-2.680616	
6786	Analysis of substances	PRR16	0	-2.671408	
6787	Analysis of substances	AMOTL1	0	-2.637285	
6788	Analysis of substances	SV2B	0	-2.627161	
6789	Analysis of substances	BFSP1	0	-2.624916	
6790	Analysis of substances	PEG10	0	-2.613561	
6791	Analysis of substances	AVEN	0	-2.608917	
6792	Analysis of substances	FAM13C	0	-2.593055	
6793	Analysis of substances	LETM2	0	-2.571902	
6794	Analysis of substances	SLC16A2	0	-2.564288	
6795	Analysis of substances	DDX53	0	-2.541946	
6796	Analysis of substances	USE1	0	-2.501124	
6797	Analysis of substances	SEMA7A	0	-2.49877	
6798	Analysis of substances	CD226	0	-2.490584	

6799	Analysis of substances	IL12RB2	0	-2.478854	
6800	Analysis of substances	CPB1	0	-2.476549	
6801	Analysis of substances	ZNF323	0	-2.438389	
6802	Analysis of substances	SLC7A2	0	-2.437702	
6803	Analysis of substances	LUZP1	0	-2.416945	
6804	Analysis of substances	DIO1	0	-2.414962	
6805	Analysis of substances	TRHDE	0	-2.406423	
6806	Analysis of substances	NALCN	0	-2.405892	
6807	Analysis of substances	BMX	0	-2.404528	
6808	Analysis of substances	C6orf168	0	-2.401093	
6809	Analysis of substances	ANK2	0	-2.400795	
6810	Analysis of substances	RGL1	0	-2.39065	
6811	Analysis of substances	PDZRN4	0	-2.383322	
6812	Analysis of substances	DMGDH	0	-2.363053	
6813	Analysis of substances	ADAMTSL3	0	-2.351637	
6814	Analysis of substances	TRPC4	0	-2.350511	
6815	Analysis of substances	EGFLAM	0	-2.343421	
6816	Analysis of substances	ADAM23	0	-2.327761	
6817	Analysis of substances	PLCL1	0	-2.313588	
6818	Analysis of substances	F8	0	-2.290006	
6819	Analysis of substances	DYX1C1	0	-2.2856	
6820	Analysis of substances	KIAA0562	0	-2.276218	
6821	Analysis of substances	CBLB	0	-2.268181	
6822	Analysis of substances	WDR66	0	-2.260226	
6823	Analysis of substances	ANXA10	0	-2.250434	
6824	Analysis of substances	RIBC1	0	-2.234544	
6825	Analysis of substances	SARS2	0	-2.232155	
6826	Analysis of substances	RBPMS2	0	-2.220385	
6827	Analysis of substances	PCDH12	0	-2.2155	
6828	Analysis of substances	ARHGAP28	0	-2.212309	
6829	Analysis of substances	SHC4	0	-2.211017	
6830	Analysis of substances	ANKRD26	0	-2.202664	
6831	Analysis of substances	THAP2	0	-2.19701	
6832	Analysis of substances	C6orf174	0	-2.186645	
6833	Analysis of substances	PDZD2	0	-2.171307	
6834	Analysis of substances	CTNNA2	0	-2.161941	
6835	Analysis of substances	HPSE	0	-2.138557	
6836	Analysis of substances	ICA1L	0	-2.137896	
6837	Analysis of substances	ZNF23	0	-2.137212	
6838	Analysis of substances	RELN	0	-2.135254	
6839	Analysis of substances	MAGEA1	0	-2.123421	
6840	Analysis of substances	HDX	0	-2.113324	
6841	Analysis of substances	PDE6B	0	-2.11024	
6842	Analysis of substances	ZNF711	0	-2.105322	
6843	Analysis of substances	PLXDC1	0	-2.079149	
6844	Analysis of substances	TIGD7	0	-2.077586	
6845	Analysis of substances	NLRP11	0	-2.046876	
6846	Analysis of substances	NAV3	0	-2.040246	
6847	Analysis of substances	SLC26A4	0	-2.027787	
6848	Analysis of substances	UCN3	0	-2.023249	

6849	Analysis of substances	RNF212	0	-2.021613	
6850	Analysis of substances	KIAA1609	0	-2.010578	
6851	Analysis of substances	CNPY4	0	-2.010254	
6852	Aneuploidy	SRSF6	0.000049	-9.816535	
6853	Aneuploidy	C1orf115	0.000005	-9.309666	
6854	Aneuploidy	PDGFRL	0.000022	-9.227954	
6855	Aneuploidy	LPAR1	0.000016	-7.172098	
6856	Aneuploidy	KLK7	0.000005	-6.899787	
6857	Aneuploidy	SRPX	0.000029	-6.292872	
6858	Aneuploidy	HBG1	0.000055	-5.571564	
6859	Aneuploidy	OSMR	0	-5.5707	
6860	Aneuploidy	PBX1	0.000039	-5.1807	
6861	Aneuploidy	FER	0.000047	-5.166103	
6862	Aneuploidy	ANKRD6	0.000002	-4.762719	
6863	Aneuploidy	PPP3CA	0	-4.576634	
6864	Aneuploidy	CUTC	0.000001	-4.212615	
6865	Aneuploidy	AKT3	0.000048	-4.209524	
6866	Aneuploidy	PTP4A1	0.000018	-3.936562	
6867	Aneuploidy	PKIA	0.000015	-3.848526	
6868	Aneuploidy	GRHL2	0.000046	-3.758155	
6869	Aneuploidy	NUTF2	0.000053	-3.548485	
6870	Aneuploidy	KCTD12	0.000019	-3.407851	
6871	Aneuploidy	SSR3	0.000048	-3.337015	
6872	Aneuploidy	DST	0.00003	-3.323802	
6873	Aneuploidy	BFSP1	0.00004	-3.282172	
6874	Aneuploidy	CPNE3	0.000005	-3.030344	
6875	Aneuploidy	LAPTM4B	0.000001	-2.918159	
6876	Aneuploidy	ZNF7	0.000022	-2.894222	
6877	Aneuploidy	EBAG9	0.000033	-2.890125	
6878	Aneuploidy	ESRP1	0.000009	-2.720009	
6879	Aneuploidy	FECH	0.000005	-2.677403	
6880	Aneuploidy	ETFDH	0.000039	-2.655925	
6881	Aneuploidy	UBE2W	0.000001	-2.628488	
6882	Aneuploidy	PQLC1	0.000033	-2.596523	
6883	Aneuploidy	LYN	0	-2.5736	
6884	Aneuploidy	RAD54B	0.000047	-2.514686	
6885	Aneuploidy	SNN	0.000055	-2.417009	
6886	Aneuploidy	AP3S1	0.000033	-2.351749	
6887	Aneuploidy	ARHGAP12	0.000017	-2.34276	
6888	Aneuploidy	CEP70	0.000031	-2.31722	
6889	Aneuploidy	AZIN1	0.000047	-2.281083	
6890	Aneuploidy	COX15	0.000005	-2.277368	
6891	Aneuploidy	CEPT1	0.000005	-2.269288	
6892	Aneuploidy	GPR125	0.000016	-2.247221	
6893	Aneuploidy	RAB11FIP2	0.000016	-2.23979	
6894	Aneuploidy	UBR5	0.00001	-2.205252	
6895	Aneuploidy	TSPAN14	0.000048	-2.188828	
6896	Aneuploidy	SMARCA5	0.000048	-2.18391	
6897	Aneuploidy	SEC23B	0.000039	-2.131515	
6898	Aneuploidy	SIM2	0.000032	-2.104766	

6899	Aneuploidy	ABHD10	0.000013	-2.075524	
6900	Aneuploidy	C10orf137	0.000039	-2.042979	
6901	Aneuploidy	IQCK	0.000039	-2.023703	
6902	Aneuploidy	NQO1	0.000005	-2.001271	
6903	Aneuploidy	RPS6KA3	0.000032	2.009533	
6904	Aneuploidy	ATR	0.000029	2.0478	
6905	Aneuploidy	OSBPL2	0.00002	2.064886	
6906	Aneuploidy	MTMR1	0.000004	2.073537	
6907	Aneuploidy	HAUS7	0.000054	2.098542	
6908	Aneuploidy	NUP98	0.00003	2.141092	
6909	Aneuploidy	OSBPL10	0.000008	2.156816	
6910	Aneuploidy	KRT8	0.000041	2.184921	
6911	Aneuploidy	BLCAP	0.000016	2.187234	
6912	Aneuploidy	DDX27	0.000031	2.27204	
6913	Aneuploidy	MYST3	0.000054	2.310365	
6914	Aneuploidy	AHNAK	0.00004	2.34324	
6915	Aneuploidy	TUG1	0.000052	2.375275	
6916	Aneuploidy	CXorf40A	0.00004	2.387005	
6917	Aneuploidy	LMNA	0.000003	2.395559	
6918	Aneuploidy	RIT1	0.00003	2.417808	
6919	Aneuploidy	TCF7	0.000031	2.492176	
6920	Aneuploidy	KLHL7	0.000028	2.496174	
6921	Aneuploidy	OVOL2	0.000023	2.497405	
6922	Aneuploidy	UCHL3	0.000036	2.716893	
6923	Aneuploidy	TMEM111	0.000021	2.845	
6924	Aneuploidy	FAM179B	0.000052	3.069813	
6925	Aneuploidy	TXNDC9	0.00003	3.143618	
6926	Aneuploidy	MSH3	0.000023	3.212932	
6927	Aneuploidy	C7orf23	0.000001	3.31648	
6928	Aneuploidy	LTN1	0.000051	3.395212	
6929	Aneuploidy	DYNC1LI2	0.000012	3.526746	
6930	Aneuploidy	FLNA	0.000032	3.527161	
6931	Aneuploidy	S100A10	0.000005	3.599249	
6932	Aneuploidy	PURA	0.000002	3.77063	
6933	Aneuploidy	PIP5K1B	0.000021	3.783596	
6934	Aneuploidy	TRAK1	0.000004	4.255449	
6935	Aneuploidy	MUC13	0.000022	4.460228	
6936	Aneuploidy	NET1	0.00002	4.535664	
6937	Aneuploidy	PRKCI	0.000004	4.556852	
6938	Aneuploidy	PCSK6	0.00001	4.589261	
6939	Aneuploidy	ZNF432	0.000039	4.663858	
6940	Aneuploidy	NCOR1	0	4.743417	
6941	Aneuploidy	PLTP	0.000049	4.971416	
6942	Aneuploidy	S100A11	0.000039	5.224193	
6943	Aneuploidy	TRIM44	0.000029	5.262296	
6944	Aneuploidy	AGRN	0.000039	5.378967	
6945	Aneuploidy	ITGA6	0.000058	5.49551	
6946	Aneuploidy	ME3	0.000041	6.606196	
6947	Aneuploidy	FAM3A	0.000015	6.724106	
6948	Aneuploidy	SMAP1	0.000028	6.901903	

6949	Aneuploidy	CD302	0.000023	9.177254	
6950	Aneuploidy	FKBP11	0.000048	10.745881	
6951	Aneuploidy	CGREF1	0.000005	15.282677	
6952	Aneuploidy	NPL	0.000022	20.611888	
6953	Aneuploidy	GSTT1	0.000001	30.451178	
6954	Aneuploidy	NNT	0.00002	43.733952	
6955	Aneuploidy	PYCARD	0.000013	50.307447	
6956	Angina Pectoris	TEF	0.02675	2.017657	
6957	Angina Pectoris	GRAMD1C	0.00001	2.035444	
6958	Angina Pectoris	LOC286272	0.007181	2.097509	
6959	Angina Pectoris	BCR	0.007228	2.115363	
6960	Angina Pectoris	CRISPLD2	0	2.115448	
6961	Angina Pectoris	FLJ10404	0.019742	2.135739	
6962	Angina Pectoris	PIK3R5	0	2.136157	
6963	Angina Pectoris	WDR33	0.00082	2.155976	
6964	Angina Pectoris	CCDC66	0.007251	2.215777	
6965	Bacterial Abscess	UGT2A3	0.040416	1.545498	
6966	Breast cancer invasive NOS	ENOSF1	0.04975	-2.163659	
6967	Breast cancer invasive NOS	LOC728448	0.004106	2.432727	
6968	Breast cancer invasive NOS	TCERG1	0.047633	2.472226	
6969	Breast Carcinoma	ENOSF1	0.04975	-2.163659	
6970	Breast Carcinoma	LOC728448	0.004106	2.432727	
6971	Breast Carcinoma	TCERG1	0.047633	2.472226	
6972	Bright red color	KRT7	0.014466	-3.354952	
6973	Bright red color	TNNT1	0.015899	-3.260496	
6974	Bright red color	KLHL14	0.026774	-2.208134	
6975	Bright red color	TFAP2A	0.013004	-2.126966	
6976	Bright red color	PRAC	0.005446	2.523307	
6977	Calcium	DEFA6	0	-1.514054	
6978	Calgranulin A	TNNC2	0.009995	-2.582217	
6979	Calgranulin A	DDC	0.003443	-2.572034	
6980	Calgranulin A	MAPK13	0.000019	-2.28648	
6981	Calgranulin A	LYNX1	0.011944	2.022824	
6982	Calgranulin A	FOS	0.000013	2.073813	
6983	Calgranulin A	TEAD2	0.000157	2.084096	
6984	Calgranulin A	HLA-DPB1	0.038891	2.311579	
6985	Calgranulin A	LUM	0.004926	2.331764	
6986	Calgranulin A	FOSB	0.000023	2.405711	
6987	Calgranulin A	HLA-DRA	0.001366	2.558353	
6988	Calgranulin A	ARGBP2	0.031991	2.744366	
6989	Calgranulin A	CKIP-1	0.028965	2.807099	
6990	Calgranulin A	C5orf4	0	3.160021	
6991	Calgranulin A	RHOB	0.041968	3.179405	
6992	Calgranulin A	IL2RB	0.04056	5.00008	
6993	Cancer of Rectum	DLGAP4	0.009754	2.082438	
6994	Carcinoma	AFAP1-AS	0.000001	-39.118393	
6995	Carcinoma	RGS12	0.000106	-28.905205	
6996	Carcinoma	TNPO2	0.000055	-25.002175	
6997	Carcinoma	MGC13053	0.000057	-22.477033	
6998	Carcinoma	MGC10981	0.000001	-21.885806	

6999	Carcinoma	CHERP	0.000111	-21.388739	
7000	Carcinoma	SLC40A1	0.019761	-18.769421	
7001	Carcinoma	KCNC1	0.000038	-15.744929	
7002	Carcinoma	LOC120376	0.00199	-14.159446	
7003	Carcinoma	AKR1C2	0	-13.205982	
7004	Carcinoma	AUTS2	0.000907	-11.509164	
7005	Carcinoma	C11orf93	0.003741	-11.199361	
7006	Carcinoma	ZMYND8	0.000009	-10.590426	
7007	Carcinoma	LILRB4	0.00013	-10.501573	
7008	Carcinoma	PLAC8	0.017	-10.303294	
7009	Carcinoma	TRPS1	0.002804	-9.784698	
7010	Carcinoma	ADAM23	0.000027	-9.674894	
7011	Carcinoma	ZNF776	0.010347	-9.121891	
7012	Carcinoma	PCDH1	0.000089	-8.652112	
7013	Carcinoma	TP53TG5	0.000045	-8.044095	
7014	Carcinoma	SLC9A2	0.001748	-8.042279	
7015	Carcinoma	TNFRSF13B	0.000001	-7.912258	
7016	Carcinoma	C1orf21	0.000634	-7.66282	
7017	Carcinoma	SLC1A1	0.018372	-7.648422	
7018	Carcinoma	NR0B1	0.000001	-7.433996	
7019	Carcinoma	HIST1H3J	0.000022	-7.061221	
7020	Carcinoma	FXYD2	0.000023	-6.952451	
7021	Carcinoma	MYL10	0.000064	-6.80726	
7022	Carcinoma	MEX3D	0.000012	-6.751953	
7023	Carcinoma	MAGEA2	0.000022	-6.692525	
7024	Carcinoma	RUNX1	0.000057	-6.446082	
7025	Carcinoma	FER1L4	0.013956	-6.206674	
7026	Carcinoma	CXorf21	0.00001	-6.107469	
7027	Carcinoma	C4BPB	0.008991	-6.069479	
7028	Carcinoma	GABRB2	0.00184	-6.023043	
7029	Carcinoma	TMPRSS15	0	-5.885783	
7030	Carcinoma	ALPPL2	0.000033	-5.871586	
7031	Carcinoma	ATP6V1C2	0.01626	-5.677063	
7032	Carcinoma	AQP3	0.001129	-5.364991	
7033	Carcinoma	ZBTB7A	0.00012	-5.346095	
7034	Carcinoma	FOXA2	0.000005	-5.318482	
7035	Carcinoma	BMP2	0.019169	-5.26016	
7036	Carcinoma	CDH19	0.000007	-5.241121	
7037	Carcinoma	OSGIN1	0.000009	-5.16969	
7038	Carcinoma	WHAMM	0.000064	-5.165357	
7039	Carcinoma	STAP2	0.010853	-5.162804	
7040	Carcinoma	SIDT1	0.010526	-5.151468	
7041	Carcinoma	BLK	0.00009	-5.091386	
7042	Carcinoma	COL16A1	0.000065	-4.965684	
7043	Carcinoma	DGKE	0.001155	-4.927506	
7044	Carcinoma	TGM4	0.000074	-4.923004	
7045	Carcinoma	FRMD4B	0.00013	-4.912172	
7046	Carcinoma	PROM2	0.018737	-4.883214	
7047	Carcinoma	STXBP5L	0.00007	-4.860969	
7048	Carcinoma	CSPG5	0.000021	-4.835955	

7049	Carcinoma	SLC7A11	0.000076	-4.824935	
7050	Carcinoma	RETNLB	0.004526	-4.7277	
7051	Carcinoma	CAV2	0.000071	-4.694315	
7052	Carcinoma	EHD1	0.000117	-4.6369	
7053	Carcinoma	SP140L	0.007072	-4.634434	
7054	Carcinoma	SLC12A3	0.000001	-4.594117	
7055	Carcinoma	DTX4	0.011343	-4.56354	
7056	Carcinoma	DNAJC24	0.000058	-4.555036	
7057	Carcinoma	SIPA1L2	0.017535	-4.538822	
7058	Carcinoma	ALDH3A1	0.00002	-4.533677	
7059	Carcinoma	CAPN2	0.000135	-4.52737	
7060	Carcinoma	NF1	0.021889	-4.510667	
7061	Carcinoma	FAM84B	0.003637	-4.477328	
7062	Carcinoma	PBX2	0.000057	-4.417423	
7063	Carcinoma	DSC3	0.000065	-4.416867	
7064	Carcinoma	ANKRD56	0.003557	-4.409937	
7065	Carcinoma	AKR1B10	0.000008	-4.399986	
7066	Carcinoma	POLQ	0.000003	-4.393509	
7067	Carcinoma	ITPR3	0.00001	-4.386942	
7068	Carcinoma	MAU2	0.000035	-4.369071	
7069	Carcinoma	OR2H2	0.000076	-4.363852	
7070	Carcinoma	ZNF433	0.006244	-4.299435	
7071	Carcinoma	TTC39B	0.012589	-4.259494	
7072	Carcinoma	RBBP6	0.000111	-4.251571	
7073	Carcinoma	DLC1	0.019597	-4.226791	
7074	Carcinoma	CRIM1	0.012668	-4.211752	
7075	Carcinoma	MYBPC1	0.000026	-4.207932	
7076	Carcinoma	TACSTD2	0.0105	-4.206254	
7077	Carcinoma	LRTM1	0.000034	-4.194771	
7078	Carcinoma	B4GALT2	0.000077	-4.17918	
7079	Carcinoma	FHL1	0.000076	-4.160872	
7080	Carcinoma	IGFBP5	0.000058	-4.150725	
7081	Carcinoma	DEPDC6	0.002924	-4.148052	
7082	Carcinoma	FAM107B	0.007931	-4.135441	
7083	Carcinoma	AKR1C3	0.000049	-4.104425	
7084	Carcinoma	LRCH4	0.000018	-4.057453	
7085	Carcinoma	SF1	0.000019	-4.036428	
7086	Carcinoma	GRHL2	0.000689	-4.002688	
7087	Carcinoma	NRAP	0.000065	-3.994532	
7088	Carcinoma	ARVCF	0.000047	-3.994171	
7089	Carcinoma	KYNU	0.000064	-3.985251	
7090	Carcinoma	GPATCH2	0.000057	-3.964785	
7091	Carcinoma	DAG1	0.000008	-3.961159	
7092	Carcinoma	SEC61B	0.013417	-3.939735	
7093	Carcinoma	EFNA5	0.000027	-3.918006	
7094	Carcinoma	ATP6V0A2	0.000092	-3.904458	
7095	Carcinoma	NTSR1	0.000039	-3.86174	
7096	Carcinoma	PCSK5	0.000065	-3.823234	
7097	Carcinoma	TCF3	0.000023	-3.804097	
7098	Carcinoma	A4GNT	0.000076	-3.802451	

7099	Carcinoma	DUSP8	0.000057	-3.781893	
7100	Carcinoma	ARFRP1	0.000068	-3.777238	
7101	Carcinoma	LHX3	0.000106	-3.751055	
7102	Carcinoma	NFKBIZ	0.002582	-3.749321	
7103	Carcinoma	TLE4	0.000023	-3.727494	
7104	Carcinoma	SDPR	0.000127	-3.715142	
7105	Carcinoma	SLC3A1	0.000083	-3.699211	
7106	Carcinoma	CAV1	0.000089	-3.674352	
7107	Carcinoma	NAALADL2	0.001281	-3.642886	
7108	Carcinoma	ZNF35	0.000009	-3.628832	
7109	Carcinoma	UNKL	0.00007	-3.617383	
7110	Carcinoma	NTN4	0.01322	-3.616901	
7111	Carcinoma	PTGES	0.000109	-3.568756	
7112	Carcinoma	LY6G6D	0	-3.55764	
7113	Carcinoma	C5orf4	0.000015	-3.553788	
7114	Carcinoma	LOC100506653	0.000084	-3.549835	
7115	Carcinoma	S100A11	0.028446	-3.539459	
7116	Carcinoma	EGLN3	0.000057	-3.524001	
7117	Carcinoma	LOC253012	0.00918	-3.52357	
7118	Carcinoma	HNRNPD	0.000091	-3.523349	
7119	Carcinoma	FKSG49	0.00004	-3.492342	
7120	Carcinoma	C8orf60	0.000036	-3.47396	
7121	Carcinoma	SEMA4A	0.002877	-3.47258	
7122	Carcinoma	LOC100506076	0.001415	-3.437059	
7123	Carcinoma	PTPRO	0.000127	-3.436619	
7124	Carcinoma	C1orf66	0.000072	-3.43163	
7125	Carcinoma	AIPL1	0.000122	-3.419601	
7126	Carcinoma	ACSM3	0.020028	-3.40773	
7127	Carcinoma	ETNK2	0.000077	-3.39408	
7128	Carcinoma	IFNA4	0.00013	-3.38553	
7129	Carcinoma	PIM1	0.000045	-3.359225	
7130	Carcinoma	CD22	0.000068	-3.347774	
7131	Carcinoma	IPO9	0.000027	-3.334726	
7132	Carcinoma	PIGL	0.000051	-3.309136	
7133	Carcinoma	PICK1	0.000035	-3.292785	
7134	Carcinoma	IDH2	0.007791	-3.287394	
7135	Carcinoma	ACSL1	0.003741	-3.286705	
7136	Carcinoma	SULT2B1	0.000015	-3.282291	
7137	Carcinoma	N4BP3	0.000034	-3.277198	
7138	Carcinoma	TNFRSF25	0.000032	-3.245756	
7139	Carcinoma	TJP2	0.027142	-3.241607	
7140	Carcinoma	GJB3	0.020803	-3.240817	
7141	Carcinoma	CCDC71	0.000036	-3.239238	
7142	Carcinoma	SPRR1B	0.000029	-3.234156	
7143	Carcinoma	NDE1	0.00006	-3.218425	
7144	Carcinoma	GCLM	0.000043	-3.217085	
7145	Carcinoma	LARP7	0.000036	-3.209232	
7146	Carcinoma	EFNA1	0.000131	-3.20448	
7147	Carcinoma	ATG10	0.00001	-3.145049	
7148	Carcinoma	PRRG4	0.008991	-3.144815	

7149	Carcinoma	PROS1	0.008658	-3.137914	
7150	Carcinoma	CLIC3	0.001264	-3.134384	
7151	Carcinoma	SYTL5	0.013972	-3.12653	
7152	Carcinoma	AKR1B1	0.000064	-3.098509	
7153	Carcinoma	XKR9	0.00356	-3.095881	
7154	Carcinoma	GPATCH4	0.000123	-3.09419	
7155	Carcinoma	CELP	0.001409	-3.093503	
7156	Carcinoma	CIDEC	0.000044	-3.086425	
7157	Carcinoma	CEACAM5	0.000082	-3.080702	
7158	Carcinoma	NOV	0.000079	-3.067204	
7159	Carcinoma	ARL4D	0.000061	-3.058711	
7160	Carcinoma	MLLT3	0.021883	-3.051412	
7161	Carcinoma	ZNF814	0.005521	-3.047155	
7162	Carcinoma	MTSS1	0.00199	-3.036982	
7163	Carcinoma	MUC4	0.002306	-3.020212	
7164	Carcinoma	DDB2	0.000135	-3.019466	
7165	Carcinoma	PLEC	0.00004	-3.016577	
7166	Carcinoma	MAFG	0.000056	-3.016256	
7167	Carcinoma	LOC100509749	0.00011	-3.00381	
7168	Carcinoma	GAD1	0.000098	-2.996413	
7169	Carcinoma	LRRFIP1	0.000135	-2.995153	
7170	Carcinoma	NDUFB8	0.00009	-2.994833	
7171	Carcinoma	ITGB8	0.000414	-2.981847	
7172	Carcinoma	SET	0.000135	-2.975907	
7173	Carcinoma	SMARCA2	0.019039	-2.975702	
7174	Carcinoma	RAB11FIP3	0.01942	-2.96213	
7175	Carcinoma	SLC39A8	0.000023	-2.95567	
7176	Carcinoma	ACSL6	0	-2.953245	
7177	Carcinoma	MCTP2	0.000296	-2.947277	
7178	Carcinoma	PCSK6	0.002966	-2.944609	
7179	Carcinoma	HNF1B	0.006418	-2.936906	
7180	Carcinoma	CEACAM6	0.000107	-2.933895	
7181	Carcinoma	CPT1A	0.000098	-2.928516	
7182	Carcinoma	LAMA2	0.00013	-2.928162	
7183	Carcinoma	OSTF1	0.002982	-2.928067	
7184	Carcinoma	CEACAM4	0.00013	-2.922632	
7185	Carcinoma	CBLN1	0.000057	-2.920639	
7186	Carcinoma	MTDH	0.000025	-2.917288	
7187	Carcinoma	TGFBR3	0.001804	-2.91611	
7188	Carcinoma	TMPRSS13	0.001981	-2.913978	
7189	Carcinoma	PECAM1	0.000629	-2.905212	
7190	Carcinoma	ST6GALNAC1	0.001264	-2.902098	
7191	Carcinoma	TNNC2	0.013299	-2.899656	
7192	Carcinoma	CLDN10	0.000042	-2.876005	
7193	Carcinoma	NINJ2	0.000038	-2.875023	
7194	Carcinoma	GLYATL1	0.005448	-2.873433	
7195	Carcinoma	KPNA1	0.000106	-2.865401	
7196	Carcinoma	DNAJB9	0.000077	-2.85796	
7197	Carcinoma	USP19	0.000032	-2.851396	
7198	Carcinoma	TPM1	0.017314	-2.850394	

7199	Carcinoma	SH2D3A	0.000038	-2.8453	
7200	Carcinoma	STEAP1	0.000035	-2.831924	
7201	Carcinoma	GPR143	0	-2.826944	
7202	Carcinoma	FLI1	0.000033	-2.8209	
7203	Carcinoma	PHF20L1	0.009641	-2.820861	
7204	Carcinoma	MAPK8IP3	0.000026	-2.820238	
7205	Carcinoma	NSUN5P1	0.000077	-2.816299	
7206	Carcinoma	C11orf41	0.010566	-2.801903	
7207	Carcinoma	FTH1	0.000068	-2.789514	
7208	Carcinoma	LARP1	0.000035	-2.783966	
7209	Carcinoma	TNFAIP8	0.003766	-2.770951	
7210	Carcinoma	CA4	0.000031	-2.754742	
7211	Carcinoma	GRM8	0	-2.743257	
7212	Carcinoma	SOX15	0.000061	-2.741587	
7213	Carcinoma	DLG3	0.012931	-2.720056	
7214	Carcinoma	LOC100507619	0.00009	-2.7176	
7215	Carcinoma	S100A10	0.011201	-2.714502	
7216	Carcinoma	WLS	0.013364	-2.712913	
7217	Carcinoma	PPFIA3	0.000042	-2.697868	
7218	Carcinoma	ARAP2	0.000112	-2.693515	
7219	Carcinoma	MAP2K3	0.000076	-2.689806	
7220	Carcinoma	MORC4	0.003306	-2.687133	
7221	Carcinoma	SLC6A13	0.000018	-2.683649	
7222	Carcinoma	ZNF311	0.005635	-2.676074	
7223	Carcinoma	TAOK3	0.000019	-2.671247	
7224	Carcinoma	HLA-DMB	0.000008	-2.666941	
7225	Carcinoma	RREB1	0.00013	-2.654906	
7226	Carcinoma	FBRS	0.000074	-2.647857	
7227	Carcinoma	DUSP13	0.000076	-2.645843	
7228	Carcinoma	LIMS1	0.000018	-2.635937	
7229	Carcinoma	SFSWAP	0.000046	-2.635046	
7230	Carcinoma	CABLES1	0.004404	-2.633219	
7231	Carcinoma	CARM1	0.000106	-2.630905	
7232	Carcinoma	FNIP2	0.006509	-2.623718	
7233	Carcinoma	ZNF248	0.007847	-2.615369	
7234	Carcinoma	C2orf3	0.000116	-2.598566	
7235	Carcinoma	C11orf20	0.027142	-2.590815	
7236	Carcinoma	RB1	0.009877	-2.585316	
7237	Carcinoma	NEDD4L	0.021441	-2.568173	
7238	Carcinoma	C16orf75	0.007107	-2.565298	
7239	Carcinoma	B3GNT3	0.020608	-2.56527	
7240	Carcinoma	LOC283859	0.000008	-2.563235	
7241	Carcinoma	SF3A2	0.00012	-2.549798	
7242	Carcinoma	KHSRP	0.000012	-2.547093	
7243	Carcinoma	TMEM65	0.003599	-2.53359	
7244	Carcinoma	RBPMS	0.001179	-2.532386	
7245	Carcinoma	CD53	0.000005	-2.529536	
7246	Carcinoma	LOC202451	0.00726	-2.527351	
7247	Carcinoma	IKBKG	0.000046	-2.520416	
7248	Carcinoma	IL2RG	0.000027	-2.51786	

7249	Carcinoma	RHOBTB3	0.00004	-2.517528	
7250	Carcinoma	FOXP1	0.001437	-2.501914	
7251	Carcinoma	THRA	0.000072	-2.50089	
7252	Carcinoma	RGS3	0.000103	-2.480314	
7253	Carcinoma	PRKD3	0.020608	-2.463485	
7254	Carcinoma	ABCC6	0.002893	-2.458853	
7255	Carcinoma	PIK3R2	0.00006	-2.454348	
7256	Carcinoma	USP7	0.000103	-2.446032	
7257	Carcinoma	CYP2C9	0.000064	-2.443591	
7258	Carcinoma	FLJ45803	0.049225	-2.438337	
7259	Carcinoma	OTUB1	0.000065	-2.435734	
7260	Carcinoma	HDGF	0.000061	-2.43274	
7261	Carcinoma	PDE10A	0.013599	-2.432703	
7262	Carcinoma	PPP6R3	0.000072	-2.430979	
7263	Carcinoma	PDIA6	0.011343	-2.417617	
7264	Carcinoma	ABHD12	0.010834	-2.414374	
7265	Carcinoma	CD99	0.000135	-2.408596	
7266	Carcinoma	CPD	0.000059	-2.40344	
7267	Carcinoma	TBX6	0.000027	-2.401927	
7268	Carcinoma	ZNF321	0.000388	-2.39441	
7269	Carcinoma	ASCL2	0.000021	-2.389458	
7270	Carcinoma	ZNF543	0.016527	-2.376952	
7271	Carcinoma	TFF3	0.000073	-2.370632	
7272	Carcinoma	C20orf20	0.000111	-2.360427	
7273	Carcinoma	DGAT2	0.001415	-2.355231	
7274	Carcinoma	B3GNT6	0.001471	-2.351516	
7275	Carcinoma	EML3	0.000077	-2.342924	
7276	Carcinoma	PPP1R3B	0.012843	-2.341575	
7277	Carcinoma	SDC4	0.005018	-2.33946	
7278	Carcinoma	GFOD1	0.00012	-2.339134	
7279	Carcinoma	GPX3	0.003811	-2.337361	
7280	Carcinoma	BRF2	0.000106	-2.336616	
7281	Carcinoma	C2orf68	0.012644	-2.331396	
7282	Carcinoma	ARL13B	0.019408	-2.33092	
7283	Carcinoma	RASA1	0.025446	-2.328683	
7284	Carcinoma	PERLD1	0.003088	-2.328353	
7285	Carcinoma	PTEN	0.000054	-2.325082	
7286	Carcinoma	ANK3	0.017535	-2.3094	
7287	Carcinoma	KRT18P28	0.020608	-2.30616	
7288	Carcinoma	CSK	0.000082	-2.30579	
7289	Carcinoma	XDH	0.006461	-2.305729	
7290	Carcinoma	CPEB3	0.010839	-2.297587	
7291	Carcinoma	GBP3	0.01964	-2.292779	
7292	Carcinoma	ITPKB	0.014041	-2.289924	
7293	Carcinoma	ZNF846	0.004833	-2.285798	
7294	Carcinoma	GDE1	0.003952	-2.285521	
7295	Carcinoma	PLA2G12B	0	-2.281188	
7296	Carcinoma	ZNF440	0.002132	-2.280803	
7297	Carcinoma	ATP11B	0.015688	-2.275942	
7298	Carcinoma	RNASEH2B	0.000106	-2.270795	

7299	Carcinoma	MAP7D2	0.00008	-2.270565	
7300	Carcinoma	GALNT12	0.003604	-2.256357	
7301	Carcinoma	GPR172A	0.000105	-2.253436	
7302	Carcinoma	RGNEF	0.002134	-2.252946	
7303	Carcinoma	TTC23	0.000633	-2.246011	
7304	Carcinoma	TLE1	0.014187	-2.240267	
7305	Carcinoma	ZNF763	0.009078	-2.239459	
7306	Carcinoma	SH3BP4	0.017548	-2.238482	
7307	Carcinoma	PITX2	0.003088	-2.238318	
7308	Carcinoma	GAB1	0.009535	-2.235567	
7309	Carcinoma	ZNF606	0.000114	-2.231185	
7310	Carcinoma	IFT57	0.000122	-2.219589	
7311	Carcinoma	CHMP4B	0.026658	-2.207659	
7312	Carcinoma	HSDL2	0.021362	-2.199854	
7313	Carcinoma	PRELID2	0.012883	-2.199767	
7314	Carcinoma	FAM3A	0.000907	-2.189602	
7315	Carcinoma	ESYT2	0.047652	-2.186254	
7316	Carcinoma	KIAA0564	0.019704	-2.182719	
7317	Carcinoma	CAPN9	0.005284	-2.180483	
7318	Carcinoma	PFKFB2	0.013461	-2.180231	
7319	Carcinoma	CYP2B6	0.001339	-2.179072	
7320	Carcinoma	RPRD1B	0.029311	-2.177681	
7321	Carcinoma	CRAT	0.000336	-2.175564	
7322	Carcinoma	TSHZ1	0.020543	-2.167068	
7323	Carcinoma	UCA1	0.00003	-2.163084	
7324	Carcinoma	FDFT1	0.003341	-2.162342	
7325	Carcinoma	SPG11	0.001987	-2.158412	
7326	Carcinoma	LOC158960	0.010615	-2.158122	
7327	Carcinoma	SPTA1	0.003867	-2.155778	
7328	Carcinoma	PABPN1	0.000111	-2.154486	
7329	Carcinoma	KATNB1	0.00011	-2.151478	
7330	Carcinoma	CCDC52	0.007328	-2.134391	
7331	Carcinoma	SP8	0.003663	-2.133573	
7332	Carcinoma	C6orf105	0.013019	-2.126648	
7333	Carcinoma	CYP4F3	0.000143	-2.124238	
7334	Carcinoma	SENP6	0.000121	-2.120086	
7335	Carcinoma	ACE2	0.00002	-2.115815	
7336	Carcinoma	SPTBN1	0.006153	-2.114518	
7337	Carcinoma	PLEKHF2	0.008057	-2.111063	
7338	Carcinoma	FLJ32063	0.000026	-2.110672	
7339	Carcinoma	KRT18P40	0.026329	-2.109748	
7340	Carcinoma	DEDD2	0.012192	-2.10962	
7341	Carcinoma	PHTF1	0.000076	-2.104901	
7342	Carcinoma	LITAF	0.016345	-2.100854	
7343	Carcinoma	MMAB	0.006901	-2.100348	
7344	Carcinoma	B4GALT1	0.026518	-2.094797	
7345	Carcinoma	PNCK	0.010007	-2.09411	
7346	Carcinoma	DHRS11	0.01925	-2.090078	
7347	Carcinoma	ZNF468	0.004192	-2.088397	
7348	Carcinoma	ITPKC	0.000072	-2.087498	

7349	Carcinoma	BCAP31	0.029102	-2.085391	
7350	Carcinoma	LOC100130097	0.004961	-2.082549	
7351	Carcinoma	CDKL5	0.000542	-2.081966	
7352	Carcinoma	FLJ21511	0.012163	-2.080542	
7353	Carcinoma	FAM189A2	0.000084	-2.073444	
7354	Carcinoma	SCAPER	0.000231	-2.070198	
7355	Carcinoma	SPINT1	0.001129	-2.067415	
7356	Carcinoma	C13orf18	0	-2.063961	
7357	Carcinoma	RNF180	0.017077	-2.063449	
7358	Carcinoma	ZNF217	0.005104	-2.063444	
7359	Carcinoma	LOC100131656	0.015819	-2.058042	
7360	Carcinoma	G6PD	0.000125	-2.057143	
7361	Carcinoma	FGGY	0.000003	-2.05571	
7362	Carcinoma	HSD11B2	0.025277	-2.055615	
7363	Carcinoma	PIK3C2B	0.017314	-2.05405	
7364	Carcinoma	DCPS	0.009252	-2.052663	
7365	Carcinoma	SEMA4D	0.004604	-2.049904	
7366	Carcinoma	COL17A1	0.007833	-2.046672	
7367	Carcinoma	FGFR4	0.001061	-2.045508	
7368	Carcinoma	ITGA2	0.016406	-2.042484	
7369	Carcinoma	EXPH5	0.002828	-2.040899	
7370	Carcinoma	C1orf57	0.013028	-2.035422	
7371	Carcinoma	XKRX	0.012647	-2.033572	
7372	Carcinoma	LOC647954	0.012644	-2.032923	
7373	Carcinoma	AP2B1	0.006656	-2.029773	
7374	Carcinoma	WDR52	0.004621	-2.027418	
7375	Carcinoma	MAP2K1	0.00009	-2.026253	
7376	Carcinoma	KRT8P15	0.011623	-2.01938	
7377	Carcinoma	USP53	0.018686	-2.012765	
7378	Carcinoma	C15orf58	0.020557	-2.011262	
7379	Carcinoma	PDIA3	0.0074	-2.010856	
7380	Carcinoma	MAP3K1	0.01661	-2.008542	
7381	Carcinoma	BMP4	0.003044	-2.00842	
7382	Carcinoma	DENND1B	0.006742	-2.008021	
7383	Carcinoma	IFFO2	0.01325	-2.007613	
7384	Carcinoma	DZIP3	0.012271	2.000518	
7385	Carcinoma	TSGA14	0.003612	2.001251	
7386	Carcinoma	ARHGAP22	0.020434	2.00748	
7387	Carcinoma	WDR4	0.005951	2.011851	
7388	Carcinoma	ALDH1A2	0.005643	2.015339	
7389	Carcinoma	RSPH3	0.006247	2.021823	
7390	Carcinoma	CLTA	0.005428	2.021952	
7391	Carcinoma	STRADB	0.012465	2.031674	
7392	Carcinoma	RHOB	0.013164	2.036848	
7393	Carcinoma	GPHA2	0.025676	2.037398	
7394	Carcinoma	SULT1A4	0.048053	2.043884	
7395	Carcinoma	C10orf11	0.014983	2.044568	
7396	Carcinoma	SF3B1	0.001362	2.046207	
7397	Carcinoma	CES3	0.013431	2.049517	
7398	Carcinoma	SPACA1	0.0005	2.051074	

7399	Carcinoma	RP4-691N24.1	0.002206	2.058134	
7400	Carcinoma	OSBPL3	0.011867	2.060366	
7401	Carcinoma	C14orf149	0.001989	2.060639	
7402	Carcinoma	PLXND1	0.004196	2.061164	
7403	Carcinoma	SYCE1L	0.008967	2.064878	
7404	Carcinoma	PLEKHG5	0.018026	2.065478	
7405	Carcinoma	CHST7	0.002742	2.069626	
7406	Carcinoma	TMCO7	0.019128	2.078171	
7407	Carcinoma	AGK	0.021273	2.090902	
7408	Carcinoma	SMN1	0.004016	2.093915	
7409	Carcinoma	ZDHHC14	0.010779	2.093977	
7410	Carcinoma	BRSK2	0.009768	2.10978	
7411	Carcinoma	MPP6	0.012893	2.112255	
7412	Carcinoma	GPR135	0.004404	2.116307	
7413	Carcinoma	NQO2	0.045141	2.11926	
7414	Carcinoma	ZP3	0.04518	2.121726	
7415	Carcinoma	RPAIN	0.008464	2.123614	
7416	Carcinoma	EIF4A1	0.019891	2.125377	
7417	Carcinoma	PMS2CL	0.014843	2.137439	
7418	Carcinoma	LOC728264	0.045305	2.137676	
7419	Carcinoma	MED12L	0.019408	2.159997	
7420	Carcinoma	ARL4A	0.013857	2.163612	
7421	Carcinoma	HMGA1	0.007385	2.165079	
7422	Carcinoma	KIAA1826	0.005547	2.176751	
7423	Carcinoma	EGFL7	0.008716	2.181817	
7424	Carcinoma	ANKRD10	0.004034	2.183903	
7425	Carcinoma	PM20D2	0.015341	2.186986	
7426	Carcinoma	VGF	0.009595	2.187983	
7427	Carcinoma	RASA4	0.002876	2.204158	
7428	Carcinoma	CYB5R4	0.013965	2.206459	
7429	Carcinoma	ASF1A	0.000454	2.209496	
7430	Carcinoma	ANTXR1	0.005943	2.210334	
7431	Carcinoma	SAT1	0.017781	2.21315	
7432	Carcinoma	GSR	0.047652	2.223884	
7433	Carcinoma	PDPR	0.00954	2.22585	
7434	Carcinoma	RP9P	0.006461	2.23255	
7435	Carcinoma	MIRHG1	0.016461	2.247712	
7436	Carcinoma	ENO3	0.003437	2.252535	
7437	Carcinoma	SNTB2	0.003741	2.254721	
7438	Carcinoma	BBS10	0.018909	2.261239	
7439	Carcinoma	PREX1	0.018223	2.266672	
7440	Carcinoma	KIAA0232	0.018762	2.272248	
7441	Carcinoma	LOC647500	0.015184	2.281428	
7442	Carcinoma	CPNE7	0.008849	2.291022	
7443	Carcinoma	LTBP4	0.011681	2.291729	
7444	Carcinoma	DYNC1LI1	0.008863	2.304749	
7445	Carcinoma	ZNF385A	0.008706	2.321134	
7446	Carcinoma	ITGA7	0.002233	2.331223	
7447	Carcinoma	MMP17	0.001663	2.338375	
7448	Carcinoma	ASPN	0.044548	2.341592	

7449	Carcinoma	RAB3IL1	0.019379	2.349284	
7450	Carcinoma	LEPREL1	0.02253	2.360884	
7451	Carcinoma	PIP5K1C	0.01578	2.365342	
7452	Carcinoma	PCSK1N	0.027142	2.377122	
7453	Carcinoma	TRIM9	0.020712	2.379126	
7454	Carcinoma	MAP1A	0.019503	2.382149	
7455	Carcinoma	ERICH1	0.017968	2.389311	
7456	Carcinoma	SEPN1	0.020423	2.391331	
7457	Carcinoma	HN1	0.004632	2.418086	
7458	Carcinoma	RBMS1	0.019433	2.430764	
7459	Carcinoma	LGALSL	0.029316	2.432193	
7460	Carcinoma	BLOC1S2	0.003456	2.447418	
7461	Carcinoma	FKBP1A	0.000076	2.45199	
7462	Carcinoma	ZCCHC4	0.018582	2.454218	
7463	Carcinoma	CUTC	0.012466	2.468057	
7464	Carcinoma	ZEB1	0.009143	2.526585	
7465	Carcinoma	ZBTB40	0.014354	2.54084	
7466	Carcinoma	ATMIN	0.025676	2.544496	
7467	Carcinoma	ZBTB1	0.021035	2.547114	
7468	Carcinoma	SLC30A3	0.020557	2.548024	
7469	Carcinoma	LOC646652	0.011458	2.550961	
7470	Carcinoma	COMMD1	0.028719	2.552143	
7471	Carcinoma	NQO1	0.047652	2.558355	
7472	Carcinoma	AP3B2	0.018762	2.560671	
7473	Carcinoma	HNRNPH2	0.000135	2.561835	
7474	Carcinoma	FBXL2	0.00872	2.562055	
7475	Carcinoma	POPDC3	0.014155	2.564107	
7476	Carcinoma	SEC31A	0.000607	2.569136	
7477	Carcinoma	DDN	0.004634	2.571268	
7478	Carcinoma	ADAMTSL5	0.026518	2.572127	
7479	Carcinoma	SIX1	0.001158	2.576043	
7480	Carcinoma	CHPF	0.010208	2.58291	
7481	Carcinoma	ZNF302	0.027281	2.604239	
7482	Carcinoma	SFRS2B	0.016461	2.61632	
7483	Carcinoma	CRTAP	0.012843	2.643348	
7484	Carcinoma	RTDR1	0.010584	2.649156	
7485	Carcinoma	PALM	0.016478	2.656587	
7486	Carcinoma	TNNT1	0.014912	2.669609	
7487	Carcinoma	ASMTL	0.00947	2.67043	
7488	Carcinoma	PAM	0.016492	2.671382	
7489	Carcinoma	CLGN	0.020764	2.686142	
7490	Carcinoma	GOLGA6	0.018173	2.68629	
7491	Carcinoma	PLXNB1	0.001182	2.694092	
7492	Carcinoma	FLJ32679	0.017874	2.701101	
7493	Carcinoma	NELL2	0.010266	2.717178	
7494	Carcinoma	ADRA2C	0.01626	2.728654	
7495	Carcinoma	ZFP41	0.027142	2.75588	
7496	Carcinoma	NELF	0.014908	2.774092	
7497	Carcinoma	GART	0.000003	2.782632	
7498	Carcinoma	PGM5P2	0.01626	2.824512	

7499	Carcinoma	DUSP9	0.006294	2.869153	
7500	Carcinoma	NACAD	0.020608	2.882066	
7501	Carcinoma	ABCC5	0.007342	2.882723	
7502	Carcinoma	GREM1	0.016862	2.896806	
7503	Carcinoma	NFATC1	0.021083	2.915825	
7504	Carcinoma	GALNAC4S-6S	0.012981	2.922235	
7505	Carcinoma	LRP3	0.01558	2.923191	
7506	Carcinoma	NME4	0.020338	2.93605	
7507	Carcinoma	GEFT	0.006398	2.939987	
7508	Carcinoma	COMP	0.029502	2.972799	
7509	Carcinoma	PTMS	0.001307	2.976809	
7510	Carcinoma	GDAP1	0.012813	2.990239	
7511	Carcinoma	PDE4A	0.011824	2.9928	
7512	Carcinoma	NDRG4	0.004771	3.020168	
7513	Carcinoma	UAP1L1	0.006871	3.024504	
7514	Carcinoma	IGFBP6	0.019008	3.032017	
7515	Carcinoma	LIMD2	0.025676	3.10859	
7516	Carcinoma	LOC441426	0.000088	3.110782	
7517	Carcinoma	PDZD7	0.016037	3.124893	
7518	Carcinoma	NEFH	0.025676	3.133159	
7519	Carcinoma	C21orf66	0.018737	3.197915	
7520	Carcinoma	LOC441644	0.008169	3.201406	
7521	Carcinoma	TIMP3	0.021111	3.211283	
7522	Carcinoma	LOC100132288	0.025676	3.212723	
7523	Carcinoma	LOC283701	0.004163	3.214429	
7524	Carcinoma	GYLTL1B	0.010325	3.226717	
7525	Carcinoma	EFHA2	0.014967	3.230156	
7526	Carcinoma	FLJ35776	0.012002	3.250152	
7527	Carcinoma	B4GALNT1	0.006499	3.267059	
7528	Carcinoma	DPF1	0.027057	3.288169	
7529	Carcinoma	SMO	0.00053	3.291159	
7530	Carcinoma	TEKT4	0.025676	3.35916	
7531	Carcinoma	CACNA1H	0.000771	3.401727	
7532	Carcinoma	DPYSL4	0.001602	3.401975	
7533	Carcinoma	MAN1A1	0.010712	3.408983	
7534	Carcinoma	ARSG	0.00797	3.447322	
7535	Carcinoma	TSPAN33	0.00681	3.449669	
7536	Carcinoma	CYB5B	0.003809	3.454204	
7537	Carcinoma	SYDE1	0.000414	3.541521	
7538	Carcinoma	LOC645195	0.012012	3.547287	
7539	Carcinoma	LOC389834	0.002	3.584732	
7540	Carcinoma	IFRD1	0.005119	3.616756	
7541	Carcinoma	SLC6A8	0.010493	3.629509	
7542	Carcinoma	ANGPT2	0.008951	3.632042	
7543	Carcinoma	PAOX	0.021273	3.639009	
7544	Carcinoma	NINL	0.000217	3.642402	
7545	Carcinoma	LASS4	0.004814	3.649492	
7546	Carcinoma	PCBP3	0.009055	3.652086	
7547	Carcinoma	PET112L	0.000084	3.703134	
7548	Carcinoma	ZNF502	0.020608	3.730266	

7549	Carcinoma	AHNAK2	0.013741	3.760479	
7550	Carcinoma	NAV1	0.025228	3.786932	
7551	Carcinoma	OIT3	0.000239	3.814152	
7552	Carcinoma	LMF1	0.002134	3.82222	
7553	Carcinoma	LOC286467	0.000182	3.857226	
7554	Carcinoma	CALD1	0.019039	3.876866	
7555	Carcinoma	ADORA2A	0.019117	3.956696	
7556	Carcinoma	MAPK11	0.00413	3.97709	
7557	Carcinoma	MN1	0.010693	4.00112	
7558	Carcinoma	VAMP1	0.025647	4.05013	
7559	Carcinoma	TMEM63C	0.018308	4.069646	
7560	Carcinoma	NOTUM	0.018442	4.079847	
7561	Carcinoma	LOC643837	0.012843	4.100147	
7562	Carcinoma	GPSM1	0.000834	4.116343	
7563	Carcinoma	TRMT12	0.007172	4.133027	
7564	Carcinoma	LEMD1	0.016029	4.145322	
7565	Carcinoma	ZDHHC22	0.02253	4.160992	
7566	Carcinoma	SMYD5	0.000078	4.21254	
7567	Carcinoma	DLG4	0.000012	4.232465	
7568	Carcinoma	EMP1	0.010214	4.32057	
7569	Carcinoma	TMEM45A	0.002825	4.344767	
7570	Carcinoma	TNNI3	0.020023	4.417284	
7571	Carcinoma	HES6	0.013728	4.449856	
7572	Carcinoma	EMP3	0.00921	4.641864	
7573	Carcinoma	DEF6	0.027142	4.670714	
7574	Carcinoma	TTYH2	0.011594	4.672787	
7575	Carcinoma	MAPK8IP1	0.000188	4.78588	
7576	Carcinoma	BCL8	0.003038	4.8871	
7577	Carcinoma	FTCD	0.008226	4.892626	
7578	Carcinoma	GOLGA8E	0.019223	4.907303	
7579	Carcinoma	DKK3	0.027281	4.920574	
7580	Carcinoma	SIRPA	0.017968	4.936256	
7581	Carcinoma	DMKN	0.006213	4.960156	
7582	Carcinoma	NPM2	0.005643	5.015901	
7583	Carcinoma	SLC22A15	0.010403	5.27288	
7584	Carcinoma	COL6A2	0.017968	5.316226	
7585	Carcinoma	AHRR	0.021847	5.341179	
7586	Carcinoma	FNBP1	0.010856	5.377556	
7587	Carcinoma	CD40	0.017968	5.44852	
7588	Carcinoma	C1orf67	0.017168	5.505956	
7589	Carcinoma	CLIP4	0.008863	5.511869	
7590	Carcinoma	LOC100128328	0.008706	5.518642	
7591	Carcinoma	ZNF114	0.007566	5.529534	
7592	Carcinoma	MRGPRF	0.015184	5.602956	
7593	Carcinoma	MSRB3	0.018037	5.614288	
7594	Carcinoma	DNAH14	0.007847	5.94509	
7595	Carcinoma	AGAP11	0.000223	5.970225	
7596	Carcinoma	VWDE	0.013538	6.020041	
7597	Carcinoma	AKR1CL2	0.000595	6.170824	
7598	Carcinoma	TSNAXIP1	0.000648	6.21202	

7599	Carcinoma	C20orf194	0.010102	6.301346	
7600	Carcinoma	DOC2BL	0.014088	6.582898	
7601	Carcinoma	DFNA5	0.002582	6.744855	
7602	Carcinoma	SERPINE2	0.006501	6.769717	
7603	Carcinoma	PRDM16	0.005406	6.800626	
7604	Carcinoma	LOC253264	0.027281	6.926622	
7605	Carcinoma	FBXO27	0.002336	7.163194	
7606	Carcinoma	ZDBF2	0.004311	7.852688	
7607	Carcinoma	LOC440459	0.002816	8.066444	
7608	Carcinoma	DNM3	0.000001	8.136646	
7609	Carcinoma	MFAP2	0.006418	8.223447	
7610	Carcinoma	SOX8	0.004544	8.312914	
7611	Carcinoma	LOC149351	0.000174	8.619757	
7612	Carcinoma	SESN3	0.001534	9.090617	
7613	Carcinoma	SERPINI1	0.000008	9.45675	
7614	Carcinoma	C10orf116	0.017419	9.864284	
7615	Carcinoma	HLTF	0.017132	10.799318	
7616	Carcinoma	WASF3	0.000232	11.667004	
7617	Carcinoma	EMR2	0.000149	11.752461	
7618	Carcinoma	LOC100288413	0	12.241052	
7619	Carcinoma	NES	0.000577	12.481898	
7620	Carcinoma	CKB	0.001631	13.080778	
7621	Carcinoma	SLC16A6	0.000593	13.471531	
7622	Carcinoma	KRT23	0.011023	13.780799	
7623	Carcinoma	B4GALNT4	0.000008	14.138318	
7624	Carcinoma	FCF1	0.000076	15.378772	
7625	Carcinoma	PRKACB	0.01626	16.234631	
7626	Carcinoma	PTPRS	0.000282	18.370832	
7627	Carcinoma	FERMT2	0.002326	24.879964	
7628	Carcinoma	BEX2	0.003088	33.278956	
7629	Carcinoma	ID2B	0.000027	38.430629	
7630	Carcinoma	STAT5A	0.000106	51.764099	
7631	Carcinoma of the Large Intestine	MUC4	0.001993	-60.311531	
7632	Carcinoma of the Large Intestine	MUC2	0.001405	-60.058351	
7633	Carcinoma of the Large Intestine	FOXP2	0.002498	-27.659136	
7634	Carcinoma of the Large Intestine	B3GNT6	0.000062	-27.448227	
7635	Carcinoma of the Large Intestine	SCGB2A1	0.00296	-25.494518	
7636	Carcinoma of the Large Intestine	MMP28	0.00029	-24.383882	
7637	Carcinoma of the Large Intestine	KCNMA1	0.000529	-21.649194	
7638	Carcinoma of the Large Intestine	LRMP	0.0036	-20.996778	
7639	Carcinoma of the Large Intestine	CWH43	0.00122	-20.467763	
7640	Carcinoma of the Large Intestine	SERTAD4	0.000207	-20.029386	
7641	Carcinoma of the Large Intestine	SPDEF	0.003466	-18.98549	
7642	Carcinoma of the Large Intestine	LOC100509635	0.000651	-16.450784	
7643	Carcinoma of the Large Intestine	CHST5	0.003988	-16.204897	
7644	Carcinoma of the Large Intestine	ANO5	0.000982	-15.875208	
7645	Carcinoma of the Large Intestine	AKAP5	0.000488	-13.182194	
7646	Carcinoma of the Large Intestine	PTGDR	0.001995	-11.897712	
7647	Carcinoma of the Large Intestine	CD1D	0.000358	-11.888861	
7648	Carcinoma of the Large Intestine	KLF9	0.003783	-11.537512	

7649	Carcinoma of the Large Intestine	SPINK4	0	-10.604966	
7650	Carcinoma of the Large Intestine	SCIN	0.000675	-10.535578	
7651	Carcinoma of the Large Intestine	ZG16	0.000001	-10.523496	
7652	Carcinoma of the Large Intestine	ACOT11	0.004247	-10.396793	
7653	Carcinoma of the Large Intestine	METTL7A	0.000403	-10.26555	
7654	Carcinoma of the Large Intestine	PPP1R9A	0.000004	-10.144455	
7655	Carcinoma of the Large Intestine	CA4	0.000001	-10.09118	
7656	Carcinoma of the Large Intestine	DQX1	0.004044	-10.025782	
7657	Carcinoma of the Large Intestine	CREB3L1	0.003101	-9.594399	
7658	Carcinoma of the Large Intestine	RAB27B	0.000026	-9.499798	
7659	Carcinoma of the Large Intestine	CACNB2	0.000114	-9.483538	
7660	Carcinoma of the Large Intestine	ASAP3	0.002559	-9.13715	
7661	Carcinoma of the Large Intestine	ZBTB7C	0.00056	-9.045275	
7662	Carcinoma of the Large Intestine	FOXA1	0.003339	-8.939768	
7663	Carcinoma of the Large Intestine	ALDH1A1	0.003273	-8.905504	
7664	Carcinoma of the Large Intestine	PADI2	0.00277	-8.762223	
7665	Carcinoma of the Large Intestine	FABP1	0	-8.719388	
7666	Carcinoma of the Large Intestine	SYT17	0.000365	-8.713691	
7667	Carcinoma of the Large Intestine	KCNK10	0.000558	-8.692129	
7668	Carcinoma of the Large Intestine	LOC100129069	0.001754	-8.641304	
7669	Carcinoma of the Large Intestine	HMGCS2	0.000405	-8.54771	
7670	Carcinoma of the Large Intestine	HGD	0.001124	-8.34414	
7671	Carcinoma of the Large Intestine	FLJ32063	0.00281	-8.296896	
7672	Carcinoma of the Large Intestine	CLCA4	0.000002	-8.038073	
7673	Carcinoma of the Large Intestine	CEACAM7	0.000001	-7.835955	
7674	Carcinoma of the Large Intestine	NR3C2	0.000114	-7.832787	
7675	Carcinoma of the Large Intestine	ITM2A	0.004857	-7.7829	
7676	Carcinoma of the Large Intestine	TPBG	0.005167	-7.771458	
7677	Carcinoma of the Large Intestine	KLF4	0.003466	-7.611532	
7678	Carcinoma of the Large Intestine	GLIS3	0.000782	-7.497044	
7679	Carcinoma of the Large Intestine	ERN2	0.000972	-7.486607	
7680	Carcinoma of the Large Intestine	MOGAT2	0.000358	-7.460037	
7681	Carcinoma of the Large Intestine	B3GALT5	0.000358	-7.341713	
7682	Carcinoma of the Large Intestine	OR51I1	0.004706	-7.291565	
7683	Carcinoma of the Large Intestine	EFNA5	0.000405	-7.221174	
7684	Carcinoma of the Large Intestine	DOCK8	0.004993	-7.212796	
7685	Carcinoma of the Large Intestine	LAMA1	0.002619	-7.153433	
7686	Carcinoma of the Large Intestine	C14orf50	0.000358	-7.032244	
7687	Carcinoma of the Large Intestine	GRAMD3	0.000488	-6.964465	
7688	Carcinoma of the Large Intestine	CCNJL	0.000163	-6.772087	
7689	Carcinoma of the Large Intestine	C11orf93	0.001565	-6.711384	
7690	Carcinoma of the Large Intestine	SPATA18	0.003433	-6.664482	
7691	Carcinoma of the Large Intestine	FARSB	0.00277	-6.56314	
7692	Carcinoma of the Large Intestine	DPF3	0.000202	-6.507991	
7693	Carcinoma of the Large Intestine	PLCE1	0.00071	-6.424479	
7694	Carcinoma of the Large Intestine	MDFIC	0.000923	-6.391231	
7695	Carcinoma of the Large Intestine	PLCL2	0.003101	-6.299826	
7696	Carcinoma of the Large Intestine	CHGA	0.000004	-6.084005	
7697	Carcinoma of the Large Intestine	CBFA2T3	0.000405	-6.06337	
7698	Carcinoma of the Large Intestine	HPGDS	0.00138	-5.994216	

7699	Carcinoma of the Large Intestine	PSMG4	0.00332	-5.931413	
7700	Carcinoma of the Large Intestine	EDIL3	0.001296	-5.905179	
7701	Carcinoma of the Large Intestine	SLC9A2	0.004579	-5.796233	
7702	Carcinoma of the Large Intestine	FRMD3	0.004638	-5.691552	
7703	Carcinoma of the Large Intestine	SLC16A7	0.003386	-5.613971	
7704	Carcinoma of the Large Intestine	ACTG2	0	-5.576115	
7705	Carcinoma of the Large Intestine	AQP8	0.000157	-5.570023	
7706	Carcinoma of the Large Intestine	CHP2	0.004857	-5.506835	
7707	Carcinoma of the Large Intestine	MS4A12	0.000032	-5.360331	
7708	Carcinoma of the Large Intestine	PDE4D	0.000331	-5.300381	
7709	Carcinoma of the Large Intestine	LOC643072	0.003988	-5.284507	
7710	Carcinoma of the Large Intestine	PRKACB	0.000052	-5.284016	
7711	Carcinoma of the Large Intestine	RNF125	0.003737	-5.236229	
7712	Carcinoma of the Large Intestine	SIDT1	0.000358	-5.216442	
7713	Carcinoma of the Large Intestine	MATN2	0.000471	-5.191578	
7714	Carcinoma of the Large Intestine	MCTP2	0.000114	-5.145834	
7715	Carcinoma of the Large Intestine	VSTM2A	0.002883	-5.04434	
7716	Carcinoma of the Large Intestine	NR5A2	0.00211	-5.041885	
7717	Carcinoma of the Large Intestine	TNFRSF11A	0.000529	-4.980546	
7718	Carcinoma of the Large Intestine	EFHC2	0.000215	-4.968653	
7719	Carcinoma of the Large Intestine	VILL	0.000651	-4.956432	
7720	Carcinoma of the Large Intestine	GOLM1	0.000215	-4.926988	
7721	Carcinoma of the Large Intestine	DENND1B	0.002619	-4.908923	
7722	Carcinoma of the Large Intestine	C2orf88	0.001516	-4.861751	
7723	Carcinoma of the Large Intestine	KIAA1731	0.004141	-4.835946	
7724	Carcinoma of the Large Intestine	DEGS2	0.004217	-4.794887	
7725	Carcinoma of the Large Intestine	KSR2	0.004857	-4.793552	
7726	Carcinoma of the Large Intestine	TRPM6	0.000711	-4.765605	
7727	Carcinoma of the Large Intestine	TEX9	0.003737	-4.753316	
7728	Carcinoma of the Large Intestine	TEP1	0.00237	-4.711473	
7729	Carcinoma of the Large Intestine	KLK1	0.00332	-4.696096	
7730	Carcinoma of the Large Intestine	LOC100506589	0.001854	-4.673174	
7731	Carcinoma of the Large Intestine	CCL28	0.000605	-4.654903	
7732	Carcinoma of the Large Intestine	C16orf53	0.003149	-4.649649	
7733	Carcinoma of the Large Intestine	BMP2	0.004341	-4.567065	
7734	Carcinoma of the Large Intestine	GNE	0.004141	-4.563829	
7735	Carcinoma of the Large Intestine	TOX	0.001493	-4.475666	
7736	Carcinoma of the Large Intestine	PARVA	0.002922	-4.438447	
7737	Carcinoma of the Large Intestine	SSTR1	0.003988	-4.42735	
7738	Carcinoma of the Large Intestine	SIAE	0.001854	-4.411799	
7739	Carcinoma of the Large Intestine	RAB27A	0.00002	-4.39503	
7740	Carcinoma of the Large Intestine	ARMCX4	0.003737	-4.391212	
7741	Carcinoma of the Large Intestine	LOC727916	0.005164	-4.380807	
7742	Carcinoma of the Large Intestine	FAS	0.002883	-4.344731	
7743	Carcinoma of the Large Intestine	LXN	0.001245	-4.295182	
7744	Carcinoma of the Large Intestine	LOC100128288	0.004857	-4.240265	
7745	Carcinoma of the Large Intestine	FYB	0.003433	-4.225236	
7746	Carcinoma of the Large Intestine	DDX26B	0.004644	-4.223636	
7747	Carcinoma of the Large Intestine	ANG	0.000488	-4.192727	
7748	Carcinoma of the Large Intestine	LOC100216546	0.000776	-4.176673	

7749	Carcinoma of the Large Intestine	EBF1	0.004544	-4.164009	
7750	Carcinoma of the Large Intestine	TEX11	0.001604	-4.140414	
7751	Carcinoma of the Large Intestine	PLAC8	0.000009	-4.134202	
7752	Carcinoma of the Large Intestine	STYK1	0.000446	-4.069078	
7753	Carcinoma of the Large Intestine	TCEA3	0.003014	-4.058089	
7754	Carcinoma of the Large Intestine	CXCL14	0.000133	-4.05581	
7755	Carcinoma of the Large Intestine	LOC728903	0.001325	-4.04142	
7756	Carcinoma of the Large Intestine	PAFAH2	0.001196	-4.022292	
7757	Carcinoma of the Large Intestine	KCTD1	0.00296	-4.01414	
7758	Carcinoma of the Large Intestine	GPR27	0.004668	-4.013727	
7759	Carcinoma of the Large Intestine	QSOX1	0.003047	-3.986606	
7760	Carcinoma of the Large Intestine	UBQLN4	0.004931	-3.93261	
7761	Carcinoma of the Large Intestine	EHF	0.001404	-3.901229	
7762	Carcinoma of the Large Intestine	CARD16	0.003031	-3.878799	
7763	Carcinoma of the Large Intestine	CAPN13	0.002883	-3.8648	
7764	Carcinoma of the Large Intestine	TTLL5	0.004629	-3.848962	
7765	Carcinoma of the Large Intestine	DST	0	-3.84509	
7766	Carcinoma of the Large Intestine	C4orf34	0.000493	-3.841658	
7767	Carcinoma of the Large Intestine	BCAR3	0.001754	-3.82109	
7768	Carcinoma of the Large Intestine	C1orf21	0.000694	-3.812153	
7769	Carcinoma of the Large Intestine	SYTL2	0.001871	-3.807608	
7770	Carcinoma of the Large Intestine	ITLN1	0	-3.790869	
7771	Carcinoma of the Large Intestine	ACVRL1	0.002015	-3.786947	
7772	Carcinoma of the Large Intestine	PHF7	0.004347	-3.782336	
7773	Carcinoma of the Large Intestine	CCDC68	0.002058	-3.775933	
7774	Carcinoma of the Large Intestine	ADH6	0.002922	-3.740311	
7775	Carcinoma of the Large Intestine	HNRNPD	0.004611	-3.731405	
7776	Carcinoma of the Large Intestine	RAB26	0.00113	-3.719949	
7777	Carcinoma of the Large Intestine	SPECC1	0.004848	-3.698745	
7778	Carcinoma of the Large Intestine	SFRS18	0.001805	-3.6981	
7779	Carcinoma of the Large Intestine	ABCA5	0.004638	-3.659734	
7780	Carcinoma of the Large Intestine	CLMN	0.000358	-3.643807	
7781	Carcinoma of the Large Intestine	FAM149A	0.004517	-3.641557	
7782	Carcinoma of the Large Intestine	MAOA	0.00219	-3.624132	
7783	Carcinoma of the Large Intestine	C14orf106	0.004644	-3.614466	
7784	Carcinoma of the Large Intestine	FBLN1	0.000002	-3.606652	
7785	Carcinoma of the Large Intestine	VPS26A	0.002559	-3.599722	
7786	Carcinoma of the Large Intestine	TRAPPC10	0.00293	-3.583657	
7787	Carcinoma of the Large Intestine	TPSG1	0	-3.549494	
7788	Carcinoma of the Large Intestine	DMXL2	0.002619	-3.539085	
7789	Carcinoma of the Large Intestine	ARMC8	0.00345	-3.52509	
7790	Carcinoma of the Large Intestine	ADAMDEC1	0.000004	-3.51873	
7791	Carcinoma of the Large Intestine	FLJ27352	0.001463	-3.511211	
7792	Carcinoma of the Large Intestine	REG4	0.000059	-3.503367	
7793	Carcinoma of the Large Intestine	PTPN21	0.001057	-3.479038	
7794	Carcinoma of the Large Intestine	SAMD13	0.000333	-3.468445	
7795	Carcinoma of the Large Intestine	FRMD4B	0.0037	-3.467352	
7796	Carcinoma of the Large Intestine	NCRNA00081	0.000927	-3.424376	
7797	Carcinoma of the Large Intestine	PYY	0.000183	-3.420898	
7798	Carcinoma of the Large Intestine	AKAP10	0.003047	-3.41148	

7799	Carcinoma of the Large Intestine	LIMA1	0.003047	-3.393403	
7800	Carcinoma of the Large Intestine	EHHADH	0.004243	-3.390529	
7801	Carcinoma of the Large Intestine	ENTPD5	0.004217	-3.376918	
7802	Carcinoma of the Large Intestine	FLJ23867	0.000215	-3.375153	
7803	Carcinoma of the Large Intestine	PGAP1	0.001395	-3.368329	
7804	Carcinoma of the Large Intestine	RAP1GAP	0.004544	-3.366309	
7805	Carcinoma of the Large Intestine	SEMA6A	0.003988	-3.359076	
7806	Carcinoma of the Large Intestine	KIAA1324	0.00219	-3.346923	
7807	Carcinoma of the Large Intestine	MGC21881	0.004483	-3.345753	
7808	Carcinoma of the Large Intestine	MMP3	0.000106	-3.344679	
7809	Carcinoma of the Large Intestine	MGLL	0	-3.330854	
7810	Carcinoma of the Large Intestine	SBF2	0.000987	-3.325026	
7811	Carcinoma of the Large Intestine	ENOSF1	0.000324	-3.307115	
7812	Carcinoma of the Large Intestine	ATP8B1	0.001005	-3.299743	
7813	Carcinoma of the Large Intestine	NAB1	0.001404	-3.298022	
7814	Carcinoma of the Large Intestine	CAPN5	0.00049	-3.289094	
7815	Carcinoma of the Large Intestine	SLC44A1	0.000488	-3.283266	
7816	Carcinoma of the Large Intestine	PRORSD1P	0.000953	-3.274898	
7817	Carcinoma of the Large Intestine	SLC22A23	0.000488	-3.272734	
7818	Carcinoma of the Large Intestine	LOC100289632	0.002906	-3.25408	
7819	Carcinoma of the Large Intestine	CHD9	0.000795	-3.253819	
7820	Carcinoma of the Large Intestine	DAPP1	0.004176	-3.24881	
7821	Carcinoma of the Large Intestine	SLC35A1	0.000136	-3.233704	
7822	Carcinoma of the Large Intestine	SIPA1L2	0.004857	-3.210274	
7823	Carcinoma of the Large Intestine	KIAA1370	0.002922	-3.207262	
7824	Carcinoma of the Large Intestine	GNAQ	0.001906	-3.202496	
7825	Carcinoma of the Large Intestine	PTPRH	0.004811	-3.182022	
7826	Carcinoma of the Large Intestine	PLEKHH1	0.001405	-3.180597	
7827	Carcinoma of the Large Intestine	KIF13A	0.000802	-3.177314	
7828	Carcinoma of the Large Intestine	ALDH6A1	0.002957	-3.169574	
7829	Carcinoma of the Large Intestine	PPP1R12B	0.003424	-3.145353	
7830	Carcinoma of the Large Intestine	LEAP2	0.000488	-3.125762	
7831	Carcinoma of the Large Intestine	AHNAK	0.002883	-3.114242	
7832	Carcinoma of the Large Intestine	MEIS3P1	0.002861	-3.102961	
7833	Carcinoma of the Large Intestine	TET2	0.00293	-3.096987	
7834	Carcinoma of the Large Intestine	SPARCL1	0.000004	-3.096174	
7835	Carcinoma of the Large Intestine	CMAS	0.000711	-3.085735	
7836	Carcinoma of the Large Intestine	TFF3	0.004638	-3.069182	
7837	Carcinoma of the Large Intestine	SELENBP1	0.000071	-3.066158	
7838	Carcinoma of the Large Intestine	C2orf72	0.002451	-3.066078	
7839	Carcinoma of the Large Intestine	C4orf19	0.001296	-3.063415	
7840	Carcinoma of the Large Intestine	DARC	0.000001	-3.028116	
7841	Carcinoma of the Large Intestine	VIP	0.000001	-3.019982	
7842	Carcinoma of the Large Intestine	SR140	0.003805	-3.019369	
7843	Carcinoma of the Large Intestine	SCAPER	0.004247	-3.017734	
7844	Carcinoma of the Large Intestine	AKR1B10	0.000698	-3.002267	
7845	Carcinoma of the Large Intestine	LOC400573	0.001278	-2.984874	
7846	Carcinoma of the Large Intestine	ACACB	0.004517	-2.983934	
7847	Carcinoma of the Large Intestine	KIAA1468	0.000776	-2.972723	
7848	Carcinoma of the Large Intestine	DTWD1	0.004638	-2.969544	

7849	Carcinoma of the Large Intestine	FOXP1	0.000365	-2.942729	
7850	Carcinoma of the Large Intestine	MON2	0.000711	-2.938999	
7851	Carcinoma of the Large Intestine	FAM48A	0.002854	-2.936586	
7852	Carcinoma of the Large Intestine	VMD2L1	0.000134	-2.885943	
7853	Carcinoma of the Large Intestine	CEP70	0.001068	-2.88124	
7854	Carcinoma of the Large Intestine	CWF19L2	0.003589	-2.85482	
7855	Carcinoma of the Large Intestine	FGD4	0.000358	-2.846781	
7856	Carcinoma of the Large Intestine	C9orf125	0.000623	-2.833038	
7857	Carcinoma of the Large Intestine	RBM25	0.000802	-2.822374	
7858	Carcinoma of the Large Intestine	HSBP1L1	0.000488	-2.82191	
7859	Carcinoma of the Large Intestine	ABCC3	0.002833	-2.821428	
7860	Carcinoma of the Large Intestine	PPP2R3A	0.003288	-2.821136	
7861	Carcinoma of the Large Intestine	GPA33	0.000665	-2.816748	
7862	Carcinoma of the Large Intestine	RNASE4	0.000488	-2.815054	
7863	Carcinoma of the Large Intestine	PLCD1	0.004517	-2.809502	
7864	Carcinoma of the Large Intestine	SULT1A3	0.003634	-2.807614	
7865	Carcinoma of the Large Intestine	VWF	0.000708	-2.797215	
7866	Carcinoma of the Large Intestine	SAHH3	0.000006	-2.796622	
7867	Carcinoma of the Large Intestine	LYST	0.000207	-2.788893	
7868	Carcinoma of the Large Intestine	LOC100128893	0.004141	-2.787412	
7869	Carcinoma of the Large Intestine	TLE1	0.004773	-2.779947	
7870	Carcinoma of the Large Intestine	CPEB3	0.005131	-2.761783	
7871	Carcinoma of the Large Intestine	EXPH5	0.000446	-2.756191	
7872	Carcinoma of the Large Intestine	LOC100506168	0.003101	-2.737855	
7873	Carcinoma of the Large Intestine	TNFRSF10D	0.00281	-2.734711	
7874	Carcinoma of the Large Intestine	HSD11B2	0.000411	-2.707869	
7875	Carcinoma of the Large Intestine	SGSM3	0.002619	-2.702811	
7876	Carcinoma of the Large Intestine	LOC100506493	0.005131	-2.692526	
7877	Carcinoma of the Large Intestine	NHSL1	0.002223	-2.69232	
7878	Carcinoma of the Large Intestine	PCLO	0.003424	-2.682969	
7879	Carcinoma of the Large Intestine	LOC342918	0.031445	-2.676083	
7880	Carcinoma of the Large Intestine	NEDD4L	0.00332	-2.675522	
7881	Carcinoma of the Large Intestine	TRUB1	0.016286	-2.67491	
7882	Carcinoma of the Large Intestine	TPT1	0.004857	-2.663384	
7883	Carcinoma of the Large Intestine	GPATCH2	0.003649	-2.652061	
7884	Carcinoma of the Large Intestine	SPON1	0.000432	-2.646048	
7885	Carcinoma of the Large Intestine	CCL13	0.000261	-2.615183	
7886	Carcinoma of the Large Intestine	C10orf54	0.003149	-2.612426	
7887	Carcinoma of the Large Intestine	CPA3	0	-2.60624	
7888	Carcinoma of the Large Intestine	NAPEPLD	0.001781	-2.604727	
7889	Carcinoma of the Large Intestine	SMCHD1	0.003343	-2.585213	
7890	Carcinoma of the Large Intestine	EPB41L4B	0.000062	-2.549991	
7891	Carcinoma of the Large Intestine	ETFDH	0.003339	-2.525804	
7892	Carcinoma of the Large Intestine	PIGN	0.001057	-2.524512	
7893	Carcinoma of the Large Intestine	PALLD	0.000893	-2.519625	
7894	Carcinoma of the Large Intestine	CCDC112	0.001371	-2.515317	
7895	Carcinoma of the Large Intestine	ZSWIM6	0.003636	-2.505999	
7896	Carcinoma of the Large Intestine	CASD1	0.004245	-2.501637	
7897	Carcinoma of the Large Intestine	SDCBP2	0.000331	-2.495947	
7898	Carcinoma of the Large Intestine	FUBP1	0.000607	-2.495646	

7899	Carcinoma of the Large Intestine	RNASEL	0.004644	-2.494672	
7900	Carcinoma of the Large Intestine	MYOF	0.003778	-2.488286	
7901	Carcinoma of the Large Intestine	PLD1	0.0037	-2.482875	
7902	Carcinoma of the Large Intestine	AMY2B	0.004544	-2.481498	
7903	Carcinoma of the Large Intestine	FOXF2	0	-2.48074	
7904	Carcinoma of the Large Intestine	TMEM30B	0.000555	-2.470234	
7905	Carcinoma of the Large Intestine	SLCO2A1	0.000002	-2.469144	
7906	Carcinoma of the Large Intestine	CAMK2D	0.000541	-2.468768	
7907	Carcinoma of the Large Intestine	ACAD10	0.002237	-2.454179	
7908	Carcinoma of the Large Intestine	AP4B1	0.000582	-2.447063	
7909	Carcinoma of the Large Intestine	FOXF1	0	-2.435512	
7910	Carcinoma of the Large Intestine	CD74	0.037564	-2.433393	
7911	Carcinoma of the Large Intestine	MYO1C	0.003288	-2.416458	
7912	Carcinoma of the Large Intestine	RBBP4	0.004235	-2.412335	
7913	Carcinoma of the Large Intestine	GLTP	0.00293	-2.401717	
7914	Carcinoma of the Large Intestine	CRYZL1	0.004152	-2.394317	
7915	Carcinoma of the Large Intestine	SYTL4	0.001604	-2.391109	
7916	Carcinoma of the Large Intestine	KIAA1109	0.003038	-2.378572	
7917	Carcinoma of the Large Intestine	PGM5	0.000314	-2.364494	
7918	Carcinoma of the Large Intestine	WDR7	0.005167	-2.346086	
7919	Carcinoma of the Large Intestine	SCARA5	0.000025	-2.332101	
7920	Carcinoma of the Large Intestine	PAPSS2	0.004629	-2.332038	
7921	Carcinoma of the Large Intestine	FAM162A	0.00025	-2.33122	
7922	Carcinoma of the Large Intestine	CCDC14	0.00051	-2.328058	
7923	Carcinoma of the Large Intestine	HNF1B	0.003493	-2.321626	
7924	Carcinoma of the Large Intestine	RABGAP1L	0.003737	-2.314043	
7925	Carcinoma of the Large Intestine	C15orf48	0.000105	-2.264361	
7926	Carcinoma of the Large Intestine	EFCAB4B	0.004437	-2.264241	
7927	Carcinoma of the Large Intestine	TSPAN13	0.002922	-2.261416	
7928	Carcinoma of the Large Intestine	AGXT2L2	0.00293	-2.258722	
7929	Carcinoma of the Large Intestine	MAB21L2	0.000009	-2.254028	
7930	Carcinoma of the Large Intestine	NKTR	0.002223	-2.246367	
7931	Carcinoma of the Large Intestine	MS4A8B	0.000135	-2.245197	
7932	Carcinoma of the Large Intestine	EYA3	0.046824	-2.232375	
7933	Carcinoma of the Large Intestine	TMEM43	0.004638	-2.226105	
7934	Carcinoma of the Large Intestine	RGMA	0.000001	-2.216106	
7935	Carcinoma of the Large Intestine	FAM161B	0.00488	-2.207343	
7936	Carcinoma of the Large Intestine	TMIGD	0.000335	-2.196593	
7937	Carcinoma of the Large Intestine	C8orf83	0.001854	-2.182153	
7938	Carcinoma of the Large Intestine	TCF21	0	-2.173875	
7939	Carcinoma of the Large Intestine	DNAJC4	0.002861	-2.171379	
7940	Carcinoma of the Large Intestine	ANKRD36	0.005131	-2.167662	
7941	Carcinoma of the Large Intestine	CTSG	0.000001	-2.15913	
7942	Carcinoma of the Large Intestine	TSPAN7	0.000705	-2.148268	
7943	Carcinoma of the Large Intestine	TMEM54	0.000211	-2.141421	
7944	Carcinoma of the Large Intestine	Q8NBX4	0.000102	-2.127648	
7945	Carcinoma of the Large Intestine	SDCCAG1	0.00091	-2.126706	
7946	Carcinoma of the Large Intestine	ZNF252	0.002619	-2.12491	
7947	Carcinoma of the Large Intestine	FAM200A	0.003763	-2.122202	
7948	Carcinoma of the Large Intestine	NSG1	0	-2.121418	

7949	Carcinoma of the Large Intestine	RHAG	0.046824	-2.120604	
7950	Carcinoma of the Large Intestine	PLAT	0.000028	-2.107739	
7951	Carcinoma of the Large Intestine	UNC5B	0.003512	-2.103171	
7952	Carcinoma of the Large Intestine	ACAA1	0.000802	-2.099543	
7953	Carcinoma of the Large Intestine	EPCAM	0.005086	-2.095787	
7954	Carcinoma of the Large Intestine	NEUROG3	0.000017	-2.095584	
7955	Carcinoma of the Large Intestine	LOC284454	0.004125	-2.081457	
7956	Carcinoma of the Large Intestine	ECHDC2	0.004558	-2.078043	
7957	Carcinoma of the Large Intestine	ADSV	0.000576	-2.070961	
7958	Carcinoma of the Large Intestine	CD79A	0.000427	-2.069078	
7959	Carcinoma of the Large Intestine	SYNJ2BP	0.000369	-2.059174	
7960	Carcinoma of the Large Intestine	TMSB10	0.000217	-2.0569	
7961	Carcinoma of the Large Intestine	VPS13D	0.003805	-2.055066	
7962	Carcinoma of the Large Intestine	PPP1R13B	0.004557	-2.039713	
7963	Carcinoma of the Large Intestine	GRIN1	0.001189	-2.033231	
7964	Carcinoma of the Large Intestine	MTHFD2L	0.004857	-2.02129	
7965	Carcinoma of the Large Intestine	MALL	0.00058	-2.001755	
7966	Carcinoma of the Large Intestine	CYP2R1	0.001404	-2.000093	
7967	Carcinoma of the Large Intestine	CBX3	0.000002	2.001406	
7968	Carcinoma of the Large Intestine	ZMYND19	0	2.00155	
7969	Carcinoma of the Large Intestine	LONP1	0	2.008715	
7970	Carcinoma of the Large Intestine	RGS12	0	2.009921	
7971	Carcinoma of the Large Intestine	MTA2	0.000001	2.010076	
7972	Carcinoma of the Large Intestine	AAA1	0	2.01351	
7973	Carcinoma of the Large Intestine	KIF15	0	2.015372	
7974	Carcinoma of the Large Intestine	GOLT1B	0	2.015554	
7975	Carcinoma of the Large Intestine	RIPK2	0	2.016821	
7976	Carcinoma of the Large Intestine	MTERFD1	0.000001	2.018794	
7977	Carcinoma of the Large Intestine	KIF11	0.000001	2.025286	
7978	Carcinoma of the Large Intestine	NUP107	0	2.028406	
7979	Carcinoma of the Large Intestine	CCDC59	0	2.030633	
7980	Carcinoma of the Large Intestine	PSD2	0	2.036907	
7981	Carcinoma of the Large Intestine	TIMELESS	0	2.051896	
7982	Carcinoma of the Large Intestine	PHF11	0.001695	2.062975	
7983	Carcinoma of the Large Intestine	TMEM91	0	2.063926	
7984	Carcinoma of the Large Intestine	OIP5	0.000001	2.065998	
7985	Carcinoma of the Large Intestine	KDM2B	0.004141	2.06911	
7986	Carcinoma of the Large Intestine	RUVBL1	0	2.069813	
7987	Carcinoma of the Large Intestine	NUP205	0.000001	2.074642	
7988	Carcinoma of the Large Intestine	STT3A	0	2.077468	
7989	Carcinoma of the Large Intestine	ENO1	0.000002	2.078688	
7990	Carcinoma of the Large Intestine	RUVBL2	0	2.080554	
7991	Carcinoma of the Large Intestine	ADCK4	0	2.081367	
7992	Carcinoma of the Large Intestine	QPCTL	0	2.085368	
7993	Carcinoma of the Large Intestine	BPI	0	2.085919	
7994	Carcinoma of the Large Intestine	RFC5	0.000001	2.096438	
7995	Carcinoma of the Large Intestine	EPPK1	0.000001	2.101393	
7996	Carcinoma of the Large Intestine	L3MBTL2	0	2.106203	
7997	Carcinoma of the Large Intestine	C20orf132	0	2.106547	
7998	Carcinoma of the Large Intestine	PSMA1	0.004439	2.107412	

7999	Carcinoma of the Large Intestine	SLC25A32	0.000001	2.113102	
8000	Carcinoma of the Large Intestine	GINS2	0.000001	2.11675	
8001	Carcinoma of the Large Intestine	NUP85	0.000001	2.11777	
8002	Carcinoma of the Large Intestine	C1orf107	0.002301	2.118563	
8003	Carcinoma of the Large Intestine	RBM28	0	2.120363	
8004	Carcinoma of the Large Intestine	COL18A1	0	2.120507	
8005	Carcinoma of the Large Intestine	NCAPG2	0	2.125408	
8006	Carcinoma of the Large Intestine	FCHSD1	0	2.126384	
8007	Carcinoma of the Large Intestine	CAD	0.000001	2.127343	
8008	Carcinoma of the Large Intestine	C22orf29	0	2.132896	
8009	Carcinoma of the Large Intestine	GTF2IRD1	0	2.133547	
8010	Carcinoma of the Large Intestine	FANCL	0	2.139324	
8011	Carcinoma of the Large Intestine	GZMB	0	2.14732	
8012	Carcinoma of the Large Intestine	KIF2C	0.000001	2.154208	
8013	Carcinoma of the Large Intestine	OAS3	0.000001	2.155018	
8014	Carcinoma of the Large Intestine	TIE1	0	2.16211	
8015	Carcinoma of the Large Intestine	PORCN	0	2.162538	
8016	Carcinoma of the Large Intestine	ANO3	0	2.166345	
8017	Carcinoma of the Large Intestine	FANCG	0	2.166676	
8018	Carcinoma of the Large Intestine	CCL4	0	2.174968	
8019	Carcinoma of the Large Intestine	PDZD2	0	2.185247	
8020	Carcinoma of the Large Intestine	PCBP3	0	2.186495	
8021	Carcinoma of the Large Intestine	CPSF4	0.000711	2.187018	
8022	Carcinoma of the Large Intestine	SLC38A3	0	2.187399	
8023	Carcinoma of the Large Intestine	RAB15	0	2.188293	
8024	Carcinoma of the Large Intestine	JAG2	0.000001	2.191192	
8025	Carcinoma of the Large Intestine	WDR43	0.000001	2.194866	
8026	Carcinoma of the Large Intestine	C10orf71	0	2.194946	
8027	Carcinoma of the Large Intestine	CSNK2B	0.000285	2.197823	
8028	Carcinoma of the Large Intestine	OLR1	0.000557	2.207088	
8029	Carcinoma of the Large Intestine	GTPBP5	0	2.213754	
8030	Carcinoma of the Large Intestine	ARIH1	0.000605	2.21698	
8031	Carcinoma of the Large Intestine	HOXB6	0.022575	2.220702	
8032	Carcinoma of the Large Intestine	CLCN7	0	2.234566	
8033	Carcinoma of the Large Intestine	KIF19	0	2.251696	
8034	Carcinoma of the Large Intestine	POLR1D	0.000001	2.252838	
8035	Carcinoma of the Large Intestine	PTCD1	0	2.255095	
8036	Carcinoma of the Large Intestine	KIAA1430	0.004953	2.262002	
8037	Carcinoma of the Large Intestine	NUP37	0.000001	2.262069	
8038	Carcinoma of the Large Intestine	FUT8	0	2.267452	
8039	Carcinoma of the Large Intestine	PITX1	0.000001	2.26755	
8040	Carcinoma of the Large Intestine	CHD7	0.000445	2.281194	
8041	Carcinoma of the Large Intestine	CBX4	0	2.293435	
8042	Carcinoma of the Large Intestine	KIF20A	0	2.295043	
8043	Carcinoma of the Large Intestine	TMEM109	0	2.298206	
8044	Carcinoma of the Large Intestine	SLC26A4	0	2.301524	
8045	Carcinoma of the Large Intestine	MYO1H	0	2.304578	
8046	Carcinoma of the Large Intestine	MICB	0	2.307009	
8047	Carcinoma of the Large Intestine	BHLHE22	0	2.322857	
8048	Carcinoma of the Large Intestine	HS2ST1	0.000002	2.328743	

8049	Carcinoma of the Large Intestine	CECR2	0	2.332804	
8050	Carcinoma of the Large Intestine	CBFB	0	2.334836	
8051	Carcinoma of the Large Intestine	CDC42EP1	0.000001	2.339437	
8052	Carcinoma of the Large Intestine	SORD	0.000001	2.341712	
8053	Carcinoma of the Large Intestine	POLN	0	2.353199	
8054	Carcinoma of the Large Intestine	USH2A	0	2.355116	
8055	Carcinoma of the Large Intestine	NRK	0	2.357796	
8056	Carcinoma of the Large Intestine	DOK1	0	2.368591	
8057	Carcinoma of the Large Intestine	AMPD2	0	2.370905	
8058	Carcinoma of the Large Intestine	COL24A1	0	2.375741	
8059	Carcinoma of the Large Intestine	HEATR4	0	2.383003	
8060	Carcinoma of the Large Intestine	MRPS17	0.000001	2.391333	
8061	Carcinoma of the Large Intestine	KIF9	0	2.398162	
8062	Carcinoma of the Large Intestine	LY6G6D	0	2.400382	
8063	Carcinoma of the Large Intestine	NOP56	0.000001	2.40229	
8064	Carcinoma of the Large Intestine	ZWILCH	0	2.403617	
8065	Carcinoma of the Large Intestine	CDH26	0	2.404148	
8066	Carcinoma of the Large Intestine	JAKMIP2	0	2.406255	
8067	Carcinoma of the Large Intestine	CBS	0.000703	2.411396	
8068	Carcinoma of the Large Intestine	AOC2	0	2.41181	
8069	Carcinoma of the Large Intestine	ITGAM	0	2.411873	
8070	Carcinoma of the Large Intestine	ILF3	0.00296	2.417933	
8071	Carcinoma of the Large Intestine	RFX4	0	2.420886	
8072	Carcinoma of the Large Intestine	ARNTL2	0	2.425129	
8073	Carcinoma of the Large Intestine	ADFP	0.00024	2.429958	
8074	Carcinoma of the Large Intestine	ANGPTL4	0.000173	2.440066	
8075	Carcinoma of the Large Intestine	ADAMTSL2	0.000012	2.453786	
8076	Carcinoma of the Large Intestine	PSAT1	0.000001	2.45864	
8077	Carcinoma of the Large Intestine	NFE2L3	0	2.463088	
8078	Carcinoma of the Large Intestine	CSE1L	0.000001	2.467005	
8079	Carcinoma of the Large Intestine	MAK	0	2.472108	
8080	Carcinoma of the Large Intestine	EYA1	0	2.472297	
8081	Carcinoma of the Large Intestine	ANKFN1	0	2.472601	
8082	Carcinoma of the Large Intestine	C12orf11	0	2.477935	
8083	Carcinoma of the Large Intestine	KRT39	0	2.480666	
8084	Carcinoma of the Large Intestine	CARD11	0	2.480952	
8085	Carcinoma of the Large Intestine	PRDM8	0	2.481532	
8086	Carcinoma of the Large Intestine	PKD1L3	0	2.481772	
8087	Carcinoma of the Large Intestine	CA6	0	2.484544	
8088	Carcinoma of the Large Intestine	VARS	0	2.490948	
8089	Carcinoma of the Large Intestine	ICAM1	0	2.498583	
8090	Carcinoma of the Large Intestine	IQCH	0	2.516142	
8091	Carcinoma of the Large Intestine	CYP1B1	0.000314	2.525663	
8092	Carcinoma of the Large Intestine	CCDC92	0.00358	2.52573	
8093	Carcinoma of the Large Intestine	PLD6	0.000062	2.530931	
8094	Carcinoma of the Large Intestine	NPHP1	0	2.545304	
8095	Carcinoma of the Large Intestine	CEP250	0	2.550342	
8096	Carcinoma of the Large Intestine	RAPGEF4	0	2.551132	
8097	Carcinoma of the Large Intestine	BCL6B	0	2.554916	
8098	Carcinoma of the Large Intestine	DCC	0	2.558436	

8099	Carcinoma of the Large Intestine	NALCN	0	2.560201	
8100	Carcinoma of the Large Intestine	MCM7	0	2.562626	
8101	Carcinoma of the Large Intestine	PSMB8	0.004857	2.5672	
8102	Carcinoma of the Large Intestine	UBE2L6	0.000001	2.570384	
8103	Carcinoma of the Large Intestine	TTC25	0	2.571125	
8104	Carcinoma of the Large Intestine	PSMG1	0.000001	2.572526	
8105	Carcinoma of the Large Intestine	TMEM14A	0.00296	2.575291	
8106	Carcinoma of the Large Intestine	CDC6	0	2.585624	
8107	Carcinoma of the Large Intestine	AHCY	0.000002	2.587594	
8108	Carcinoma of the Large Intestine	ABCA13	0	2.590263	
8109	Carcinoma of the Large Intestine	CCBL1	0	2.59711	
8110	Carcinoma of the Large Intestine	CASS4	0	2.601324	
8111	Carcinoma of the Large Intestine	PSAP	0.002393	2.610683	
8112	Carcinoma of the Large Intestine	CTNNBL1	0	2.612824	
8113	Carcinoma of the Large Intestine	NPVF	0	2.613849	
8114	Carcinoma of the Large Intestine	LIN7A	0	2.628195	
8115	Carcinoma of the Large Intestine	FAM176A	0	2.634253	
8116	Carcinoma of the Large Intestine	MMP13	0	2.64178	
8117	Carcinoma of the Large Intestine	FBXL13	0	2.642704	
8118	Carcinoma of the Large Intestine	FGD5	0	2.646435	
8119	Carcinoma of the Large Intestine	WDR31	0	2.648876	
8120	Carcinoma of the Large Intestine	TMEM161A	0	2.649004	
8121	Carcinoma of the Large Intestine	CCNA2	0.000001	2.6516	
8122	Carcinoma of the Large Intestine	PPM1F	0.001854	2.653937	
8123	Carcinoma of the Large Intestine	EIF3B	0.001493	2.65534	
8124	Carcinoma of the Large Intestine	RHBDF1	0.000215	2.662688	
8125	Carcinoma of the Large Intestine	ALDH4A1	0.000232	2.672146	
8126	Carcinoma of the Large Intestine	NEB	0	2.673266	
8127	Carcinoma of the Large Intestine	ZNF474	0	2.681025	
8128	Carcinoma of the Large Intestine	MSLN	0	2.690638	
8129	Carcinoma of the Large Intestine	BOP1	0	2.693284	
8130	Carcinoma of the Large Intestine	AP1S2	0.003236	2.693499	
8131	Carcinoma of the Large Intestine	CGNL1	0.000004	2.694607	
8132	Carcinoma of the Large Intestine	GBP1	0	2.698104	
8133	Carcinoma of the Large Intestine	CDC25B	0.000001	2.70041	
8134	Carcinoma of the Large Intestine	EIF2AK2	0.004176	2.716897	
8135	Carcinoma of the Large Intestine	STAU1	0.004644	2.717209	
8136	Carcinoma of the Large Intestine	RFC3	0	2.720527	
8137	Carcinoma of the Large Intestine	XKRX	0	2.73967	
8138	Carcinoma of the Large Intestine	EIF5A	0	2.754041	
8139	Carcinoma of the Large Intestine	NPC1L1	0	2.754101	
8140	Carcinoma of the Large Intestine	FEN1	0.000001	2.754245	
8141	Carcinoma of the Large Intestine	LOC647979	0.004217	2.755327	
8142	Carcinoma of the Large Intestine	PTPRU	0	2.768652	
8143	Carcinoma of the Large Intestine	ABCC2	0	2.78484	
8144	Carcinoma of the Large Intestine	NCOA3	0.003805	2.809919	
8145	Carcinoma of the Large Intestine	CAMSAP1L1	0.002817	2.822373	
8146	Carcinoma of the Large Intestine	CLNK	0	2.822808	
8147	Carcinoma of the Large Intestine	ADAMTS3	0	2.824047	
8148	Carcinoma of the Large Intestine	TEAD4	0.000001	2.833619	

8149	Carcinoma of the Large Intestine	LGR5	0.000001	2.855969	
8150	Carcinoma of the Large Intestine	LMNB2	0	2.858719	
8151	Carcinoma of the Large Intestine	NT5DC2	0	2.876001	
8152	Carcinoma of the Large Intestine	EFNA3	0	2.90539	
8153	Carcinoma of the Large Intestine	HNRNPL	0	2.905758	
8154	Carcinoma of the Large Intestine	DYX1C1	0	2.918594	
8155	Carcinoma of the Large Intestine	NNMT	0.000273	2.93126	
8156	Carcinoma of the Large Intestine	C11orf45	0	2.931764	
8157	Carcinoma of the Large Intestine	SLC7A5	0.000001	2.932664	
8158	Carcinoma of the Large Intestine	CFI	0.000174	2.93439	
8159	Carcinoma of the Large Intestine	MED25	0.000405	2.953415	
8160	Carcinoma of the Large Intestine	C4orf39	0	2.956402	
8161	Carcinoma of the Large Intestine	GPX1	0.000488	2.957755	
8162	Carcinoma of the Large Intestine	INHBE	0.000642	2.971835	
8163	Carcinoma of the Large Intestine	SHMT2	0	2.982462	
8164	Carcinoma of the Large Intestine	C10orf10	0.000005	2.992878	
8165	Carcinoma of the Large Intestine	MAEL	0	2.996037	
8166	Carcinoma of the Large Intestine	PPP4R4	0	2.999379	
8167	Carcinoma of the Large Intestine	MACC1	0.003549	3.024789	
8168	Carcinoma of the Large Intestine	KIF4A	0.000001	3.052197	
8169	Carcinoma of the Large Intestine	GINS1	0	3.07162	
8170	Carcinoma of the Large Intestine	F12	0	3.082553	
8171	Carcinoma of the Large Intestine	EDAR	0	3.098888	
8172	Carcinoma of the Large Intestine	STAT1	0	3.107809	
8173	Carcinoma of the Large Intestine	LCA5	0	3.115799	
8174	Carcinoma of the Large Intestine	TRIP13	0	3.129853	
8175	Carcinoma of the Large Intestine	RNF219	0.000471	3.147289	
8176	Carcinoma of the Large Intestine	STK31	0	3.147914	
8177	Carcinoma of the Large Intestine	F5	0.00014	3.231423	
8178	Carcinoma of the Large Intestine	CA9	0	3.241118	
8179	Carcinoma of the Large Intestine	NUTF2	0	3.241433	
8180	Carcinoma of the Large Intestine	CEP55	0	3.248565	
8181	Carcinoma of the Large Intestine	SOD2	0	3.254564	
8182	Carcinoma of the Large Intestine	FOXM1	0	3.25519	
8183	Carcinoma of the Large Intestine	GRAMD1B	0	3.261789	
8184	Carcinoma of the Large Intestine	PIWIL1	0	3.34161	
8185	Carcinoma of the Large Intestine	CERKL	0	3.342308	
8186	Carcinoma of the Large Intestine	CDH2	0	3.362404	
8187	Carcinoma of the Large Intestine	BMP7	0	3.38987	
8188	Carcinoma of the Large Intestine	MCM4	0	3.415645	
8189	Carcinoma of the Large Intestine	TACSTD2	0	3.446898	
8190	Carcinoma of the Large Intestine	MIF	0.000001	3.504093	
8191	Carcinoma of the Large Intestine	GPSM2	0.000018	3.562648	
8192	Carcinoma of the Large Intestine	IFIT5	0.002833	3.586507	
8193	Carcinoma of the Large Intestine	VNN1	0.000533	3.670827	
8194	Carcinoma of the Large Intestine	PBK	0.000001	3.714962	
8195	Carcinoma of the Large Intestine	SIM2	0	3.727166	
8196	Carcinoma of the Large Intestine	NKD1	0	3.731564	
8197	Carcinoma of the Large Intestine	KLK6	0	3.733988	
8198	Carcinoma of the Large Intestine	ECT2	0	3.796085	

8199	Carcinoma of the Large Intestine	APOE	0	3.797515	
8200	Carcinoma of the Large Intestine	TMPRSS3	0	3.798986	
8201	Carcinoma of the Large Intestine	MMP11	0	3.817899	
8202	Carcinoma of the Large Intestine	TMEM97	0	3.826889	
8203	Carcinoma of the Large Intestine	KCNJ15	0	3.837106	
8204	Carcinoma of the Large Intestine	B3GALTL	0.049356	3.840546	
8205	Carcinoma of the Large Intestine	AQP9	0.000053	3.849158	
8206	Carcinoma of the Large Intestine	HSD11B1	0.000121	3.932743	
8207	Carcinoma of the Large Intestine	PMAIP1	0	4.0525	
8208	Carcinoma of the Large Intestine	CLDN2	0	4.07628	
8209	Carcinoma of the Large Intestine	CXCL9	0	4.116745	
8210	Carcinoma of the Large Intestine	IL24	0	4.120371	
8211	Carcinoma of the Large Intestine	FTL	0.003014	4.124829	
8212	Carcinoma of the Large Intestine	IFITM1	0	4.28338	
8213	Carcinoma of the Large Intestine	ASGR2	0.000553	4.302714	
8214	Carcinoma of the Large Intestine	SERPINA5	0.000352	4.345646	
8215	Carcinoma of the Large Intestine	SRPX2	0	4.507537	
8216	Carcinoma of the Large Intestine	CD81	0.00447	4.590045	
8217	Carcinoma of the Large Intestine	OLFML2B	0	4.638492	
8218	Carcinoma of the Large Intestine	SULT2A1	0.000605	4.720173	
8219	Carcinoma of the Large Intestine	FMO3	0.000071	4.720673	
8220	Carcinoma of the Large Intestine	SERPINF2	0.00067	4.841546	
8221	Carcinoma of the Large Intestine	CXCL10	0	4.8737	
8222	Carcinoma of the Large Intestine	SLCO1B3	0	5.007902	
8223	Carcinoma of the Large Intestine	DACH1	0.001964	5.017765	
8224	Carcinoma of the Large Intestine	CXCL1	0	5.226033	
8225	Carcinoma of the Large Intestine	COLEC11	0	5.278582	
8226	Carcinoma of the Large Intestine	COL1A2	0	5.292898	
8227	Carcinoma of the Large Intestine	CP	0.00038	5.509002	
8228	Carcinoma of the Large Intestine	CPB2	0.000432	5.613978	
8229	Carcinoma of the Large Intestine	C3	0.000006	5.668511	
8230	Carcinoma of the Large Intestine	SLC13A5	0.000331	6.076943	
8231	Carcinoma of the Large Intestine	C8A	0.000531	6.120991	
8232	Carcinoma of the Large Intestine	SRSF6	0.000001	6.156758	
8233	Carcinoma of the Large Intestine	DSC3	0	6.40146	
8234	Carcinoma of the Large Intestine	MMP12	0	6.617377	
8235	Carcinoma of the Large Intestine	RBP4	0.000557	6.658636	
8236	Carcinoma of the Large Intestine	ASGR1	0.000048	6.679332	
8237	Carcinoma of the Large Intestine	C5	0.00011	6.687382	
8238	Carcinoma of the Large Intestine	APOH	0.000429	6.75181	
8239	Carcinoma of the Large Intestine	FCN3	0.000009	6.844049	
8240	Carcinoma of the Large Intestine	APOC2	0.000051	6.904064	
8241	Carcinoma of the Large Intestine	AGXT	0.000773	6.935567	
8242	Carcinoma of the Large Intestine	HPD	0.000531	7.014538	
8243	Carcinoma of the Large Intestine	C6	0.00046	7.054535	
8244	Carcinoma of the Large Intestine	SAA4	0.000529	7.065	
8245	Carcinoma of the Large Intestine	CXCL3	0	7.233392	
8246	Carcinoma of the Large Intestine	PLG	0.000498	7.361402	
8247	Carcinoma of the Large Intestine	ITIH1	0.000337	7.554118	
8248	Carcinoma of the Large Intestine	ZAK	0.000658	7.747274	

8249	Carcinoma of the Large Intestine	C4BPA	0.000535	7.948833	
8250	Carcinoma of the Large Intestine	APOC1	0.000001	8.000367	
8251	Carcinoma of the Large Intestine	TTR	0.000676	8.109081	
8252	Carcinoma of the Large Intestine	CYP2C8	0.000673	8.207707	
8253	Carcinoma of the Large Intestine	DSG3	0	8.93798	
8254	Carcinoma of the Large Intestine	LBP	0.000127	8.984359	
8255	Carcinoma of the Large Intestine	HAMP	0.000059	9.033264	
8256	Carcinoma of the Large Intestine	IL1A	0	9.050041	
8257	Carcinoma of the Large Intestine	TCN1	0	9.094781	
8258	Carcinoma of the Large Intestine	KRT23	0	9.22809	
8259	Carcinoma of the Large Intestine	APCS	0.000082	9.596353	
8260	Carcinoma of the Large Intestine	KNG1	0.000331	10.388199	
8261	Carcinoma of the Large Intestine	HRG	0.000312	11.044669	
8262	Carcinoma of the Large Intestine	SERPINC1	0.000285	11.791284	
8263	Carcinoma of the Large Intestine	SERPINA3	0.000001	11.942192	
8264	Carcinoma of the Large Intestine	APOB	0.000088	13.0809	
8265	Carcinoma of the Large Intestine	AHSG	0.000112	13.371622	
8266	Carcinoma of the Large Intestine	APOC3	0.000181	13.701454	
8267	Carcinoma of the Large Intestine	HPX	0.000192	14.287143	
8268	Carcinoma of the Large Intestine	FGG	0.000014	14.651355	
8269	Carcinoma of the Large Intestine	F2	0.000047	15.443368	
8270	Carcinoma of the Large Intestine	VTN	0.000044	15.844447	
8271	Carcinoma of the Large Intestine	ORM2	0.00009	16.094898	
8272	Carcinoma of the Large Intestine	CYP2E1	0.000072	16.227626	
8273	Carcinoma of the Large Intestine	FGL1	0.000039	18.32827	
8274	Carcinoma of the Large Intestine	TF	0.000068	18.608249	
8275	Carcinoma of the Large Intestine	GC	0.000013	20.947027	
8276	Carcinoma of the Large Intestine	AMBP	0.000021	21.099729	
8277	Carcinoma of the Large Intestine	APOA2	0.000023	21.365529	
8278	Carcinoma of the Large Intestine	HP	0.000009	21.852969	
8279	Carcinoma of the Large Intestine	ALB	0.000007	23.019959	
8280	Carcinoma of the Large Intestine	APOA1	0.000018	23.169381	
8281	Carcinoma of the Large Intestine	FGB	0.000008	23.984148	
8282	Carcinoma of the Large Intestine	FGA	0.000008	25.158992	
8283	Carcinoma of the Large Intestine	CRP	0.000002	25.311911	
8284	Carcinoma of the Large Intestine	ORM1	0.000008	25.514711	
8285	Carcinoma, Ductal, Breast	ENOSF1	0.04975	-2.163659	
8286	Carcinoma, Ductal, Breast	LOC728448	0.004106	2.432727	
8287	Carcinoma, Ductal, Breast	TCERG1	0.047633	2.472226	
8288	Carcinoma, Papillary	FABP1	0	-1907.03953	
8289	Carcinoma, Papillary	CEACAM5	0	-234.186195	
8290	Carcinoma, Papillary	CEACAM6	0	-196.986603	
8291	Carcinoma, Papillary	MUC12	0	-147.031096	
8292	Carcinoma, Papillary	LGALS4	0	-133.938134	
8293	Carcinoma, Papillary	OLFM4	0	-132.620855	
8294	Carcinoma, Papillary	GPX2	0	-127.021925	
8295	Carcinoma, Papillary	SLC26A3	0	-122.746328	
8296	Carcinoma, Papillary	CEACAM7	0	-109.698036	
8297	Carcinoma, Papillary	CLCA1	0	-94.02019	
8298	Carcinoma, Papillary	REG4	0	-90.92623	

8299	Carcinoma, Papillary	CDH17	0	-90.475329	
8300	Carcinoma, Papillary	S100P	0	-87.011091	
8301	Carcinoma, Papillary	CLRN3	0	-85.084843	
8302	Carcinoma, Papillary	MUC13	0	-80.78052	
8303	Carcinoma, Papillary	DEFA6	0.000001	-69.423082	
8304	Carcinoma, Papillary	REG1B	0	-68.002356	
8305	Carcinoma, Papillary	ZG16	0	-67.278447	
8306	Carcinoma, Papillary	NOX1	0	-63.09699	
8307	Carcinoma, Papillary	TFF3	0	-60.824063	
8308	Carcinoma, Papillary	ALDOB	0.000065	-60.651111	
8309	Carcinoma, Papillary	KRT20	0	-60.542768	
8310	Carcinoma, Papillary	REG1A	0	-55.481058	
8311	Carcinoma, Papillary	MUC2	0	-47.110146	
8312	Carcinoma, Papillary	LOC646627	0	-46.907823	
8313	Carcinoma, Papillary	FERMT1	0	-46.804189	
8314	Carcinoma, Papillary	SPINK1	0	-45.571975	
8315	Carcinoma, Papillary	MMP3	0	-43.145098	
8316	Carcinoma, Papillary	SPINK4	0	-41.336371	
8317	Carcinoma, Papillary	TRIM31	0	-40.91445	
8318	Carcinoma, Papillary	C10orf99	0	-39.48309	
8319	Carcinoma, Papillary	MEP1A	0	-39.010654	
8320	Carcinoma, Papillary	ADH1C	0	-38.897862	
8321	Carcinoma, Papillary	REG3A	0	-37.386354	
8322	Carcinoma, Papillary	CCL20	0	-37.225024	
8323	Carcinoma, Papillary	UGT2B17	0	-37.166028	
8324	Carcinoma, Papillary	MMP12	0	-35.405148	
8325	Carcinoma, Papillary	VIL1	0	-34.559985	
8326	Carcinoma, Papillary	MUC4	0	-31.860587	
8327	Carcinoma, Papillary	ITLN1	0	-31.223227	
8328	Carcinoma, Papillary	RETNLB	0	-30.930316	
8329	Carcinoma, Papillary	DUOX2	0	-29.770406	
8330	Carcinoma, Papillary	CCL14-CCL15	0	-29.181088	
8331	Carcinoma, Papillary	CFTR	0	-28.559981	
8332	Carcinoma, Papillary	CDX2	0	-27.888768	
8333	Carcinoma, Papillary	HNF4A	0	-27.391584	
8334	Carcinoma, Papillary	TFF1	0	-27.36202	
8335	Carcinoma, Papillary	CDX1	0	-25.96671	
8336	Carcinoma, Papillary	MS4A12	0	-25.165937	
8337	Carcinoma, Papillary	EPS8L3	0	-24.243884	
8338	Carcinoma, Papillary	FUT6	0	-24.21147	
8339	Carcinoma, Papillary	TDGF1	0	-23.01447	
8340	Carcinoma, Papillary	AREG	0	-20.623005	
8341	Carcinoma, Papillary	PLAC8	0	-20.435763	
8342	Carcinoma, Papillary	CXCL5	0	-20.354652	
8343	Carcinoma, Papillary	GPA33	0	-20.076328	
8344	Carcinoma, Papillary	IL8	0	-19.986084	
8345	Carcinoma, Papillary	DEFA5	0	-19.416091	
8346	Carcinoma, Papillary	ATP10B	0	-19.304022	
8347	Carcinoma, Papillary	TFF2	0	-18.889391	
8348	Carcinoma, Papillary	ANXA13	0	-18.712436	

8349	Carcinoma, Papillary	NAT2	0	-18.580529	
8350	Carcinoma, Papillary	DPEP1	0	-18.280346	
8351	Carcinoma, Papillary	COL17A1	0	-18.089649	
8352	Carcinoma, Papillary	TOX3	0	-18.043021	
8353	Carcinoma, Papillary	NR1I2	0	-17.97656	
8354	Carcinoma, Papillary	CLCA4	0	-17.954325	
8355	Carcinoma, Papillary	CEACAM1	0	-17.498997	
8356	Carcinoma, Papillary	NCRNA00261	0	-16.558295	
8357	Carcinoma, Papillary	RAPGEFL1	0	-16.546089	
8358	Carcinoma, Papillary	CYP2B6	0	-16.479378	
8359	Carcinoma, Papillary	PCSK1	0	-16.077903	
8360	Carcinoma, Papillary	HSD17B2	0	-15.879286	
8361	Carcinoma, Papillary	PIGR	0	-15.791576	
8362	Carcinoma, Papillary	HYAL1	0	-15.769106	
8363	Carcinoma, Papillary	CEL	0	-15.73894	
8364	Carcinoma, Papillary	CDHR5	0	-15.360779	
8365	Carcinoma, Papillary	ABP1	0	-15.284525	
8366	Carcinoma, Papillary	DUOXA2	0	-14.076323	
8367	Carcinoma, Papillary	DMBT1	0	-14.059784	
8368	Carcinoma, Papillary	C2orf89	0	-13.913439	
8369	Carcinoma, Papillary	CCL24	0	-13.868413	
8370	Carcinoma, Papillary	POF1B	0	-13.783513	
8371	Carcinoma, Papillary	FAM55D	0	-13.713159	
8372	Carcinoma, Papillary	TCN1	0	-13.703881	
8373	Carcinoma, Papillary	LY6G6D	0	-13.265429	
8374	Carcinoma, Papillary	CXCL3	0	-12.963999	
8375	Carcinoma, Papillary	GUCY2C	0	-12.854353	
8376	Carcinoma, Papillary	ABCC2	0	-12.749951	
8377	Carcinoma, Papillary	ASCL2	0	-12.637184	
8378	Carcinoma, Papillary	PRSS3	0	-12.609422	
8379	Carcinoma, Papillary	RNF186	0	-12.384311	
8380	Carcinoma, Papillary	LEFTY1	0	-12.355129	
8381	Carcinoma, Papillary	ITGA2	0	-12.007321	
8382	Carcinoma, Papillary	UGT2A3	0	-11.883993	
8383	Carcinoma, Papillary	ETS2	0	-11.746334	
8384	Carcinoma, Papillary	CDCA7	0	-11.650209	
8385	Carcinoma, Papillary	LRRC19	0	-11.632686	
8386	Carcinoma, Papillary	IYD	0.000002	-11.449527	
8387	Carcinoma, Papillary	CAPN8	0	-11.387377	
8388	Carcinoma, Papillary	TMPRSS4	0	-11.347615	
8389	Carcinoma, Papillary	LGR5	0	-11.340232	
8390	Carcinoma, Papillary	ARL14	0	-11.298451	
8391	Carcinoma, Papillary	CYP4F12	0	-11.258556	
8392	Carcinoma, Papillary	PPARG	0	-11.068195	
8393	Carcinoma, Papillary	FLJ32063	0	-10.914528	
8394	Carcinoma, Papillary	C15orf48	0	-10.898246	
8395	Carcinoma, Papillary	C13orf18	0	-10.846061	
8396	Carcinoma, Papillary	CPA6	0	-10.780454	
8397	Carcinoma, Papillary	PLA2G2A	0	-10.777462	
8398	Carcinoma, Papillary	CYP3A5	0	-10.613874	

8399	Carcinoma, Papillary	SLC39A5	0	-10.592831	
8400	Carcinoma, Papillary	WNK4	0	-10.53082	
8401	Carcinoma, Papillary	MYO7B	0	-10.519156	
8402	Carcinoma, Papillary	IHH	0	-10.483387	
8403	Carcinoma, Papillary	USH1C	0.000001	-10.298427	
8404	Carcinoma, Papillary	KIAA1755	0	-10.290099	
8405	Carcinoma, Papillary	CHP2	0	-10.288031	
8406	Carcinoma, Papillary	AQP8	0	-10.17501	
8407	Carcinoma, Papillary	C1orf125	0	-10.171498	
8408	Carcinoma, Papillary	SDR16C5	0	-10.152288	
8409	Carcinoma, Papillary	CDHR1	0	-10.120138	
8410	Carcinoma, Papillary	EREG	0	-10.109754	
8411	Carcinoma, Papillary	DDC	0.000007	-9.987592	
8412	Carcinoma, Papillary	LOC283859	0	-9.895492	
8413	Carcinoma, Papillary	C4BPB	0	-9.851183	
8414	Carcinoma, Papillary	CELP	0	-9.82629	
8415	Carcinoma, Papillary	LOC100506781	0	-9.674363	
8416	Carcinoma, Papillary	TRIM15	0	-9.533264	
8417	Carcinoma, Papillary	LCN2	0	-9.502444	
8418	Carcinoma, Papillary	ACE2	0	-9.380392	
8419	Carcinoma, Papillary	FCGBP	0	-9.244394	
8420	Carcinoma, Papillary	FUT3	0	-9.228442	
8421	Carcinoma, Papillary	ACSL5	0	-9.186695	
8422	Carcinoma, Papillary	FAM3D	0.000028	-9.18585	
8423	Carcinoma, Papillary	MUC17	0	-9.023207	
8424	Carcinoma, Papillary	GPR120	0	-8.956525	
8425	Carcinoma, Papillary	HSD11B2	0	-8.89267	
8426	Carcinoma, Papillary	XPNPEP2	0	-8.60786	
8427	Carcinoma, Papillary	OVOL1	0	-8.597389	
8428	Carcinoma, Papillary	GUCA2A	0	-8.587404	
8429	Carcinoma, Papillary	VIP	0	-8.555389	
8430	Carcinoma, Papillary	HEPACAM2	0	-8.44935	
8431	Carcinoma, Papillary	TNMD	0.000003	-8.338848	
8432	Carcinoma, Papillary	ISX	0	-8.188952	
8433	Carcinoma, Papillary	GPSM2	0	-8.184673	
8434	Carcinoma, Papillary	HES6	0	-8.085848	
8435	Carcinoma, Papillary	PRAP1	0.000001	-8.080921	
8436	Carcinoma, Papillary	GIPC2	0.000048	-8.040863	
8437	Carcinoma, Papillary	FCRL2	0	-8.032766	
8438	Carcinoma, Papillary	TNS4	0	-7.977346	
8439	Carcinoma, Papillary	CALML4	0	-7.974588	
8440	Carcinoma, Papillary	BTNL8	0	-7.914229	
8441	Carcinoma, Papillary	HEPH	0	-7.911607	
8442	Carcinoma, Papillary	FREM1	0	-7.902116	
8443	Carcinoma, Papillary	CTSE	0	-7.893692	
8444	Carcinoma, Papillary	DHRS9	0	-7.647905	
8445	Carcinoma, Papillary	ACSL6	0	-7.63187	
8446	Carcinoma, Papillary	HKDC1	0	-7.545197	
8447	Carcinoma, Papillary	GPRC5A	0.000001	-7.533718	
8448	Carcinoma, Papillary	LRP4	0	-7.396141	

8449	Carcinoma, Papillary	GUCA2B	0	-7.370613	
8450	Carcinoma, Papillary	GREM1	0	-7.366178	
8451	Carcinoma, Papillary	BCL2L15	0	-7.282307	
8452	Carcinoma, Papillary	AGT	0	-7.263824	
8453	Carcinoma, Papillary	GNLY	0	-7.242917	
8454	Carcinoma, Papillary	CDA	0.000027	-7.127235	
8455	Carcinoma, Papillary	PRR15	0.000003	-7.030772	
8456	Carcinoma, Papillary	CA4	0	-7.001725	
8457	Carcinoma, Papillary	SLC12A2	0.000057	-6.97687	
8458	Carcinoma, Papillary	L1TD1	0	-6.788547	
8459	Carcinoma, Papillary	FUT4	0	-6.776435	
8460	Carcinoma, Papillary	MAB21L2	0.000005	-6.769377	
8461	Carcinoma, Papillary	BCL2L14	0.000052	-6.735917	
8462	Carcinoma, Papillary	ITPKA	0	-6.714468	
8463	Carcinoma, Papillary	FAR2	0	-6.656479	
8464	Carcinoma, Papillary	CA1	0	-6.653338	
8465	Carcinoma, Papillary	ADAMDEC1	0.000001	-6.623965	
8466	Carcinoma, Papillary	A1CF	0	-6.592665	
8467	Carcinoma, Papillary	SERPINB1	0	-6.495901	
8468	Carcinoma, Papillary	PIP5K1B	0.000023	-6.487347	
8469	Carcinoma, Papillary	GMDS	0.000011	-6.419386	
8470	Carcinoma, Papillary	BAIAP2L2	0	-6.385218	
8471	Carcinoma, Papillary	FGFR4	0	-6.3624	
8472	Carcinoma, Papillary	ADCYAP1	0	-6.313629	
8473	Carcinoma, Papillary	GPR109B	0	-6.245199	
8474	Carcinoma, Papillary	SLCO1B3	0	-6.222918	
8475	Carcinoma, Papillary	CHRDL2	0.000088	-6.219112	
8476	Carcinoma, Papillary	C2CD4A	0.000007	-6.16549	
8477	Carcinoma, Papillary	CCL18	0	-6.16452	
8478	Carcinoma, Papillary	KLF4	0	-6.113691	
8479	Carcinoma, Papillary	IGJ	0.000002	-6.019768	
8480	Carcinoma, Papillary	HNF4G	0.000022	-6.002455	
8481	Carcinoma, Papillary	ANG	0	-5.987403	
8482	Carcinoma, Papillary	ADH6	0.000059	-5.964809	
8483	Carcinoma, Papillary	GSDMB	0	-5.963061	
8484	Carcinoma, Papillary	PLEK2	0	-5.873165	
8485	Carcinoma, Papillary	SLC25A15	0	-5.806055	
8486	Carcinoma, Papillary	TNFRSF11A	0	-5.780951	
8487	Carcinoma, Papillary	HBEGF	0.000001	-5.731401	
8488	Carcinoma, Papillary	NOTUM	0	-5.67124	
8489	Carcinoma, Papillary	DUSP27	0.000001	-5.65128	
8490	Carcinoma, Papillary	MLLT4	0	-5.609246	
8491	Carcinoma, Papillary	KLF5	0.000089	-5.584101	
8492	Carcinoma, Papillary	IL1A	0.000031	-5.502987	
8493	Carcinoma, Papillary	GPR128	0	-5.484955	
8494	Carcinoma, Papillary	MYO1A	0.000007	-5.46119	
8495	Carcinoma, Papillary	PROX1	0	-5.446828	
8496	Carcinoma, Papillary	ATOH1	0	-5.444901	
8497	Carcinoma, Papillary	ATP2A3	0	-5.40515	
8498	Carcinoma, Papillary	IL1RN	0	-5.399412	

8499	Carcinoma, Papillary	MYB	0.000031	-5.339766	
8500	Carcinoma, Papillary	RNASE4	0.000054	-5.303132	
8501	Carcinoma, Papillary	AIM1L	0.000001	-5.271137	
8502	Carcinoma, Papillary	CYP4F2	0.000017	-5.248093	
8503	Carcinoma, Papillary	EDN3	0.000015	-5.20948	
8504	Carcinoma, Papillary	PAPPA	0	-5.186495	
8505	Carcinoma, Papillary	FAM55A	0	-5.170335	
8506	Carcinoma, Papillary	ADAMTS8	0	-5.087833	
8507	Carcinoma, Papillary	SYTL5	0.000001	-5.074791	
8508	Carcinoma, Papillary	SLC1A7	0	-5.056292	
8509	Carcinoma, Papillary	JPH1	0.000003	-5.036581	
8510	Carcinoma, Papillary	FAM84A	0.000001	-4.927383	
8511	Carcinoma, Papillary	C11orf96	0.000004	-4.926027	
8512	Carcinoma, Papillary	BCAN	0	-4.91567	
8513	Carcinoma, Papillary	RNF43	0.000004	-4.902531	
8514	Carcinoma, Papillary	GGH	0	-4.831501	
8515	Carcinoma, Papillary	MTMR11	0	-4.816396	
8516	Carcinoma, Papillary	CYP2C18	0	-4.781955	
8517	Carcinoma, Papillary	SKP2	0.000001	-4.764183	
8518	Carcinoma, Papillary	PLN	0.000003	-4.763244	
8519	Carcinoma, Papillary	SLC6A14	0.000001	-4.753219	
8520	Carcinoma, Papillary	PCCA	0	-4.727795	
8521	Carcinoma, Papillary	PPP1R14D	0	-4.715926	
8522	Carcinoma, Papillary	SPAG1	0	-4.707864	
8523	Carcinoma, Papillary	ASB2	0.000001	-4.66307	
8524	Carcinoma, Papillary	ISG20	0.000001	-4.65649	
8525	Carcinoma, Papillary	PTGER4	0	-4.62419	
8526	Carcinoma, Papillary	PTPRO	0.000003	-4.514872	
8527	Carcinoma, Papillary	LOC400573	0	-4.508623	
8528	Carcinoma, Papillary	FLJ22763	0.000047	-4.477473	
8529	Carcinoma, Papillary	THBS1	0.000034	-4.455782	
8530	Carcinoma, Papillary	CXorf57	0.000034	-4.444357	
8531	Carcinoma, Papillary	SMAGP	0.000002	-4.443238	
8532	Carcinoma, Papillary	CLC	0.000042	-4.303155	
8533	Carcinoma, Papillary	IL22RA1	0	-4.298135	
8534	Carcinoma, Papillary	GGT5	0	-4.261632	
8535	Carcinoma, Papillary	IL3RA	0	-4.248029	
8536	Carcinoma, Papillary	NOSTRIN	0.00002	-4.238162	
8537	Carcinoma, Papillary	MXD1	0.000001	-4.230895	
8538	Carcinoma, Papillary	CCDC68	0	-4.210842	
8539	Carcinoma, Papillary	KRT6B	0	-4.201677	
8540	Carcinoma, Papillary	LOC100507192	0.000018	-4.201003	
8541	Carcinoma, Papillary	C21orf58	0.000007	-4.20044	
8542	Carcinoma, Papillary	CTLA4	0	-4.16658	
8543	Carcinoma, Papillary	LOC727924	0.000004	-4.093745	
8544	Carcinoma, Papillary	IL1B	0	-3.996748	
8545	Carcinoma, Papillary	TRABD	0	-3.995177	
8546	Carcinoma, Papillary	KRTAP1-3	0	-3.981206	
8547	Carcinoma, Papillary	CCL22	0	-3.94827	
8548	Carcinoma, Papillary	NEK3	0	-3.934227	

8549	Carcinoma, Papillary	DNASE1L3	0	-3.928455	
8550	Carcinoma, Papillary	C19orf6	0	-3.927157	
8551	Carcinoma, Papillary	FGF10	0.000002	-3.831623	
8552	Carcinoma, Papillary	MYH14	0	-3.763604	
8553	Carcinoma, Papillary	IL21R	0.000002	-3.754832	
8554	Carcinoma, Papillary	DIO3-OS	0	-3.739599	
8555	Carcinoma, Papillary	PITPNM2	0.000033	-3.726033	
8556	Carcinoma, Papillary	DPEP2	0.000001	-3.658076	
8557	Carcinoma, Papillary	GEM	0	-3.655963	
8558	Carcinoma, Papillary	CXCL6	0.000003	-3.568917	
8559	Carcinoma, Papillary	CYP4F3	0.000001	-3.521572	
8560	Carcinoma, Papillary	ADAP1	0.000014	-3.518351	
8561	Carcinoma, Papillary	PREP	0.000056	-3.476493	
8562	Carcinoma, Papillary	SLC13A3	0.000001	-3.459158	
8563	Carcinoma, Papillary	UCA1	0.000022	-3.439624	
8564	Carcinoma, Papillary	LOC100288781	0.000016	-3.392744	
8565	Carcinoma, Papillary	GNG4	0.000013	-3.38225	
8566	Carcinoma, Papillary	AGPAT4	0.000011	-3.370208	
8567	Carcinoma, Papillary	STAMBPL1	0.000029	-3.276841	
8568	Carcinoma, Papillary	ABCC3	0.000092	-3.276345	
8569	Carcinoma, Papillary	LOC100130776	0	-3.246003	
8570	Carcinoma, Papillary	FGGY	0	-3.244354	
8571	Carcinoma, Papillary	NAT8	0.00002	-3.243067	
8572	Carcinoma, Papillary	NR4A1	0.000013	-3.238111	
8573	Carcinoma, Papillary	APOH	0.000062	-3.237986	
8574	Carcinoma, Papillary	VNN3	0	-3.232821	
8575	Carcinoma, Papillary	FUT2	0.000052	-3.227239	
8576	Carcinoma, Papillary	SLC44A3	0.000001	-3.221509	
8577	Carcinoma, Papillary	HMMR	0.000007	-3.186672	
8578	Carcinoma, Papillary	EZR	0.000052	-3.156803	
8579	Carcinoma, Papillary	RBP2	0.000024	-3.085531	
8580	Carcinoma, Papillary	CPS1-IT	0.000003	-3.06693	
8581	Carcinoma, Papillary	LOC25845	0	-3.055152	
8582	Carcinoma, Papillary	SLC26A2	0	-3.017912	
8583	Carcinoma, Papillary	FCGR3B	0.000039	-3.007925	
8584	Carcinoma, Papillary	PIGZ	0	-2.983579	
8585	Carcinoma, Papillary	FLJ40292	0.000011	-2.979467	
8586	Carcinoma, Papillary	LOC146336	0.000004	-2.970969	
8587	Carcinoma, Papillary	KLK15	0.000001	-2.960357	
8588	Carcinoma, Papillary	MYO1D	0	-2.95017	
8589	Carcinoma, Papillary	AOC3	0.000028	-2.93578	
8590	Carcinoma, Papillary	TRPM6	0.000002	-2.917801	
8591	Carcinoma, Papillary	APOBEC1	0.000029	-2.89506	
8592	Carcinoma, Papillary	ZCCHC4	0.000001	-2.88691	
8593	Carcinoma, Papillary	FNDC3A	0.000034	-2.874024	
8594	Carcinoma, Papillary	CACNA1H	0.000035	-2.872445	
8595	Carcinoma, Papillary	CACNA2D1	0.000002	-2.866887	
8596	Carcinoma, Papillary	LGALS3	0.000015	-2.855915	
8597	Carcinoma, Papillary	SEMA4G	0.000032	-2.846607	
8598	Carcinoma, Papillary	LOC730102	0	-2.826886	

8599	Carcinoma, Papillary	SLC7A5	0.000071	-2.807862	
8600	Carcinoma, Papillary	LDB3	0.000059	-2.782039	
8601	Carcinoma, Papillary	GRIN2B	0.000032	-2.761053	
8602	Carcinoma, Papillary	AKAP1	0.000042	-2.754207	
8603	Carcinoma, Papillary	ALG2	0	-2.720427	
8604	Carcinoma, Papillary	TUBA4A	0.000002	-2.672017	
8605	Carcinoma, Papillary	ENPP3	0.000006	-2.663276	
8606	Carcinoma, Papillary	ZFR2	0	-2.597847	
8607	Carcinoma, Papillary	ELF1	0	-2.591772	
8608	Carcinoma, Papillary	MFI2	0	-2.579749	
8609	Carcinoma, Papillary	CDT1	0.000017	-2.575066	
8610	Carcinoma, Papillary	GNE	0.000001	-2.530543	
8611	Carcinoma, Papillary	PLEKHG6	0	-2.51952	
8612	Carcinoma, Papillary	PIWIL1	0.00002	-2.515579	
8613	Carcinoma, Papillary	NR6A1	0	-2.484779	
8614	Carcinoma, Papillary	C20orf152	0	-2.456379	
8615	Carcinoma, Papillary	FRYL	0	-2.448948	
8616	Carcinoma, Papillary	CCNB2	0.000009	-2.401553	
8617	Carcinoma, Papillary	MDGA1	0.000001	-2.39211	
8618	Carcinoma, Papillary	SH3D20	0.000002	-2.390295	
8619	Carcinoma, Papillary	ASPH	0	-2.356866	
8620	Carcinoma, Papillary	EDAR	0.000007	-2.345715	
8621	Carcinoma, Papillary	EXOSC8	0.000081	-2.328203	
8622	Carcinoma, Papillary	3-Mar	0.000001	-2.292671	
8623	Carcinoma, Papillary	LASS6	0	-2.280427	
8624	Carcinoma, Papillary	ZAK	0	-2.26803	
8625	Carcinoma, Papillary	TXLNG	0.000001	-2.247802	
8626	Carcinoma, Papillary	CELF4	0.000024	-2.205617	
8627	Carcinoma, Papillary	LLGL2	0.000058	-2.200024	
8628	Carcinoma, Papillary	RFC3	0.000059	-2.175271	
8629	Carcinoma, Papillary	GTF3A	0	-2.132585	
8630	Carcinoma, Papillary	LEPR	0.000049	-2.094402	
8631	Carcinoma, Papillary	AKR7A3	0.000007	-2.091531	
8632	Carcinoma, Papillary	PRRG4	0.000001	-2.079988	
8633	Carcinoma, Papillary	C12orf75	0.000015	-2.062463	
8634	Carcinoma, Papillary	FBRS	0	-2.042991	
8635	Carcinoma, Papillary	ZFP36L2	0.000014	-2.027757	
8636	Carcinoma, Papillary	CADM4	0	2.577503	
8637	Catenins	REG1A	0.000149	-36.576833	
8638	Catenins	AKAP12	0.000382	-19.882979	
8639	Catenins	SERPINB5	0.00007	-14.794114	
8640	Catenins	FAM129A	0.000243	-14.358025	
8641	Catenins	MT1M	0.000016	-11.080972	
8642	Catenins	PRUNE2	0.000015	-9.361624	
8643	Catenins	BAT3	0.000087	-9.181054	
8644	Catenins	ZNF204P	0.000614	-8.492386	
8645	Catenins	SPIN3	0.000147	-7.489164	
8646	Catenins	ZNF426	0.000112	-5.90991	
8647	Catenins	ZNF558	0.000114	-5.356884	
8648	Catenins	LIMCH1	0.000303	-4.710667	

8649	Catenins	SYNJ2	0.000382	-4.635881	
8650	Catenins	UGT1A6	0.000406	-4.496336	
8651	Catenins	PTGR1	0.000001	-4.486203	
8652	Catenins	FAM50B	0.000018	-4.335119	
8653	Catenins	LOC100505875	0.000492	-4.152001	
8654	Catenins	TPRXL	0.00018	-4.054795	
8655	Catenins	RTTN	0.000002	-3.816143	
8656	Catenins	TUG1	0.000015	-3.752775	
8657	Catenins	INO80C	0.000135	-3.731187	
8658	Catenins	ESYT2	0.000458	-3.665878	
8659	Catenins	RHOU	0.000078	-3.614124	
8660	Catenins	TNRC18	0.000082	-3.412363	
8661	Catenins	ATXN1	0.000166	-3.387009	
8662	Catenins	PIK3C3	0.000003	-3.343665	
8663	Catenins	TXNDC9	0.000143	-3.328385	
8664	Catenins	DSC2	0.000018	-3.231242	
8665	Catenins	MECOM	0.000112	-3.198311	
8666	Catenins	ING2	0.000177	-3.185976	
8667	Catenins	TTC39C	0.00052	-3.154439	
8668	Catenins	ARGLU1	0.000279	-3.146774	
8669	Catenins	DNAJB6	0.00007	-3.07301	
8670	Catenins	PIGN	0.000049	-3.062276	
8671	Catenins	MSN	0.000121	-3.047942	
8672	Catenins	ALDH3A1	0.00048	-3.025803	
8673	Catenins	ASH1L	0.000058	-2.991166	
8674	Catenins	SEPW1	0.000014	-2.899693	
8675	Catenins	PRRG4	0.000001	-2.878893	
8676	Catenins	HOXB7	0.000109	-2.850091	
8677	Catenins	NBN	0.000065	-2.803623	
8678	Catenins	SLC16A1	0.000175	-2.715163	
8679	Catenins	MRPL35	0.000564	-2.712121	
8680	Catenins	SGCB	0.000015	-2.689522	
8681	Catenins	TM9SF3	0.000143	-2.59314	
8682	Catenins	ZCCHC2	0.000382	-2.590748	
8683	Catenins	ZNF271	0.000626	-2.562873	
8684	Catenins	HOXA13	0.000143	-2.550304	
8685	Catenins	UBE3C	0.000058	-2.522976	
8686	Catenins	DSG2	0.000062	-2.460754	
8687	Catenins	ZC3H13	0.00008	-2.449023	
8688	Catenins	VAPB	0.000259	-2.433886	
8689	Catenins	SUGT1	0.000413	-2.430777	
8690	Catenins	DOLPP1	0.000064	-2.403751	
8691	Catenins	GPS2	0.000015	-2.357439	
8692	Catenins	GAPVD1	0.000259	-2.351562	
8693	Catenins	HIST1H1C	0.000058	-2.309695	
8694	Catenins	FAM91A2	0.000614	-2.287029	
8695	Catenins	ROD1	0.000114	-2.270856	
8696	Catenins	VAPA	0.000437	-2.236095	
8697	Catenins	FRG1	0.000112	-2.210131	
8698	Catenins	YTHDC2	0.000096	-2.176141	

8699	Catenins	PSMD5	0.000018	-2.135035	
8700	Catenins	ARPC5L	0.000064	-2.134362	
8701	Catenins	UPF3A	0.000487	-2.116966	
8702	Catenins	MAPRE2	0.000392	-2.088331	
8703	Catenins	GGCX	0.000191	-2.075676	
8704	Catenins	FAM122B	0.000129	-2.05692	
8705	Catenins	RB1	0.000078	-2.05352	
8706	Catenins	GPR107	0.000266	-2.051135	
8707	Catenins	TMEM50A	0.000259	-2.047113	
8708	Catenins	SH3BGRL2	0.000259	-2.031019	
8709	Catenins	C17orf85	0.000287	-2.030274	
8710	Catenins	MBD4	0.000087	-2.02786	
8711	Catenins	MYST3	0.000605	-2.012837	
8712	Catenins	HN1	0.000033	-2.003492	
8713	Catenins	TMEM18	0.000257	2.026822	
8714	Catenins	ZFP62	0.000058	2.032576	
8715	Catenins	C2orf28	0.00052	2.041845	
8716	Catenins	C1orf57	0.000615	2.042778	
8717	Catenins	MAGED1	0.000376	2.073153	
8718	Catenins	EXOSC1	0.000259	2.075314	
8719	Catenins	SLC17A9	0.000143	2.079349	
8720	Catenins	TAF1D	0.00007	2.095709	
8721	Catenins	PREPL	0.000058	2.095728	
8722	Catenins	MIPEP	0.00007	2.116788	
8723	Catenins	SLC39A4	0.000378	2.128275	
8724	Catenins	CHST3	0.000407	2.132689	
8725	Catenins	C6orf108	0.000196	2.158936	
8726	Catenins	GBAS	0.000367	2.202874	
8727	Catenins	BIRC6	0.000564	2.20328	
8728	Catenins	TIMM8B	0.000254	2.230223	
8729	Catenins	SCCPDH	0.000259	2.231617	
8730	Catenins	ARL2	0.000607	2.246019	
8731	Catenins	FRAT2	0.000418	2.247018	
8732	Catenins	TPD52L1	0.000315	2.270668	
8733	Catenins	ARPC3	0.000612	2.271593	
8734	Catenins	HSBP1L1	0.000067	2.281044	
8735	Catenins	ATP5G2	0.000259	2.290497	
8736	Catenins	ANKHD1-EIF4	0.000437	2.305972	
8737	Catenins	RBCK1	0.000486	2.340571	
8738	Catenins	KIAA0141	0.000179	2.349644	
8739	Catenins	AGPHD1	0.000091	2.36445	
8740	Catenins	ZFAND2B	0.000489	2.366606	
8741	Catenins	EIF4B	0.000312	2.381829	
8742	Catenins	CXXC5	0.000079	2.389303	
8743	Catenins	SLC38A10	0.000553	2.412111	
8744	Catenins	GART	0.000434	2.418457	
8745	Catenins	WDR4	0.000532	2.42595	
8746	Catenins	PYGL	0.000602	2.427988	
8747	Catenins	SLC25A6	0.00034	2.434351	
8748	Catenins	SNORD104	0.000624	2.440978	

8749	Catenins	TBC1D1	0.000308	2.448241	
8750	Catenins	C4orf14	0.000437	2.47461	
8751	Catenins	GDF11	0.000432	2.480144	
8752	Catenins	DPY30	0.000143	2.4865	
8753	Catenins	MARVELD1	0.000568	2.514161	
8754	Catenins	NOB1	0.000553	2.523267	
8755	Catenins	SMARCD3	0.000128	2.530565	
8756	Catenins	C9orf86	0.000367	2.540431	
8757	Catenins	SLC25A21	0.000271	2.542119	
8758	Catenins	DYRK4	0.00007	2.615305	
8759	Catenins	HAUS4	0.000177	2.620348	
8760	Catenins	RPL13A	0.000331	2.645398	
8761	Catenins	WDYHV1	0.000235	2.653118	
8762	Catenins	GAS5	0.000142	2.653625	
8763	Catenins	GAMT	0.000143	2.666422	
8764	Catenins	SH3YL1	0.000002	2.683318	
8765	Catenins	ALDOC	0.000382	2.711191	
8766	Catenins	KLK12	0.000259	2.740593	
8767	Catenins	PKIG	0.000445	2.741006	
8768	Catenins	CNDP2	0.00012	2.744491	
8769	Catenins	RPL35A	0.000129	2.785454	
8770	Catenins	DNAJC4	0.000125	2.793849	
8771	Catenins	DPH5	0.000437	2.807949	
8772	Catenins	SH2D2A	0.000499	2.836379	
8773	Catenins	CLN3	0.000412	2.84375	
8774	Catenins	MMS19	0.000018	2.84468	
8775	Catenins	C9orf40	0.000602	2.854545	
8776	Catenins	KRTCAP3	0.000414	2.902283	
8777	Catenins	ARHGEF10	0.000279	2.912243	
8778	Catenins	LOC255512	0.000361	2.94404	
8779	Catenins	CPS1	0.000002	2.967478	
8780	Catenins	ATP13A2	0.0002	3.004528	
8781	Catenins	ASPHD2	0.000341	3.03538	
8782	Catenins	MDK	0.000446	3.092344	
8783	Catenins	ATPBD4	0.000112	3.109684	
8784	Catenins	THNSL1	0.000149	3.124434	
8785	Catenins	HYI	0.000441	3.155569	
8786	Catenins	KAZ	0.000187	3.209017	
8787	Catenins	NEO1	0.000564	3.231681	
8788	Catenins	KLF11	0.000219	3.248634	
8789	Catenins	HSPB1	0.00007	3.269693	
8790	Catenins	AIFM3	0.000228	3.295077	
8791	Catenins	AGMAT	0.000114	3.339683	
8792	Catenins	ATP6V1E2	0.000492	3.370556	
8793	Catenins	NAGS	0.00012	3.375294	
8794	Catenins	NOX1	0.000279	3.434399	
8795	Catenins	C19orf45	0.000612	3.436083	
8796	Catenins	PCCA	0.000125	3.463857	
8797	Catenins	LOC729680	0.000244	3.501818	
8798	Catenins	ARHGAP32	0.00003	3.550023	

8799	Catenins	CNFN	0.000266	3.585442	
8800	Catenins	FLJ41757	0.00048	3.605058	
8801	Catenins	LTBP3	0.000002	3.619344	
8802	Catenins	FAM119A	0.000005	3.765769	
8803	Catenins	PROCR	0.000259	3.835196	
8804	Catenins	MCCC1	0.000187	3.848696	
8805	Catenins	FADS2	0.000551	3.903226	
8806	Catenins	LOC283104	0.000279	4.155987	
8807	Catenins	GXYLT2	0.000569	4.264637	
8808	Catenins	FSCN1	0.000014	4.370552	
8809	Catenins	FLJ36031	0.000142	4.480278	
8810	Catenins	ZNF358	0.000279	4.585557	
8811	Catenins	PHLDA3	0.00002	4.586603	
8812	Catenins	RAB26	0.000042	4.644335	
8813	Catenins	SHH	0.000143	4.677488	
8814	Catenins	FLJ37644	0.000413	4.712144	
8815	Catenins	CDC42EP5	0.000176	4.798045	
8816	Catenins	PDGFC	0.000001	4.989535	
8817	Catenins	MXRA7	0.000001	4.994552	
8818	Catenins	SORBS3	0.000259	5.101449	
8819	Catenins	GALNT12	0.000082	5.173536	
8820	Catenins	C1QTNF1	0.000228	5.327957	
8821	Catenins	C4orf19	0.000367	5.339512	
8822	Catenins	LMF1	0.000513	5.430435	
8823	Catenins	APH1B	0.000114	5.553459	
8824	Catenins	ABCC4	0.000219	5.561098	
8825	Catenins	TCF4	0.000341	5.712788	
8826	Catenins	PRODH	0.000408	5.875641	
8827	Catenins	ZNF704	0.000142	5.88162	
8828	Catenins	C11orf30	0.000021	5.900891	
8829	Catenins	CDH3	0.000564	5.904728	
8830	Catenins	MRAS	0.000002	5.970402	
8831	Catenins	IL1F7	0.000275	5.996226	
8832	Catenins	GPR124	0.000137	6.185279	
8833	Catenins	SLC6A10P	0.000156	6.252374	
8834	Catenins	WNT4	0.000393	6.517647	
8835	Catenins	DLX2	0.000271	6.697201	
8836	Catenins	FUT1	0.000219	6.783496	
8837	Catenins	C11orf53	0.000115	7.249453	
8838	Catenins	C11orf93	0.000061	7.250724	
8839	Catenins	KLC3	0.000382	7.331104	
8840	Catenins	CIB2	0.000564	8.107981	
8841	Catenins	RAC3	0.000196	8.302583	
8842	Catenins	RHBDL1	0.000075	8.661224	
8843	Catenins	TNFRSF18	0.000187	9.901515	
8844	Catenins	GNAL	0.00003	11.805769	
8845	Catenins	NPL	0.000551	12.480296	
8846	Catenins	LOC100289026	0.000015	13.35725	
8847	Catenins	PARVG	0.00005	15.210227	
8848	Catenins	TLE2	0.000486	15.588353	

8849	Catenins	ABCB1	0.000482	18.547662	
8850	Catenins	UNC5A	0.000607	21.517442	
8851	Catenins	EPB41L3	0.000351	22.172662	
8852	Catenins	TCEA3	0.000058	23.553522	
8853	Catenins	LITAF	0.000053	26.621554	
8854	Catenins	ZIC2	0.000437	31.843284	
8855	Catenins	PXDN	0.00003	34.689751	
8856	Catenins	CYB5A	0.000114	497.719424	
8857	Cell Culture Techniques	MAP7	0.000652	-7.749575	
8858	Cell Culture Techniques	MAPK13	0.000093	-5.139744	
8859	Cell Culture Techniques	ROD1	0.000155	-4.802888	
8860	Cell Culture Techniques	RAB3IP	0.000218	-4.603243	
8861	Cell Culture Techniques	WWP1	0.000529	-3.9455	
8862	Cell Culture Techniques	RNASET2	0.000088	-3.751019	
8863	Cell Culture Techniques	PPFIBP2	0.000541	-3.362162	
8864	Cell Culture Techniques	BAZ2B	0.000375	-3.040968	
8865	Cell Culture Techniques	CYP2R1	0.000348	-2.97435	
8866	Cell Culture Techniques	PHIP	0.00018	-2.916824	
8867	Cell Culture Techniques	OCIAD2	0.000516	-2.862596	
8868	Cell Culture Techniques	SYK	0.000144	-2.815386	
8869	Cell Culture Techniques	KIF21A	0.000493	-2.764031	
8870	Cell Culture Techniques	LOC387921	0.000603	-2.703054	
8871	Cell Culture Techniques	SLC25A5	0.000533	-2.551182	
8872	Cell Culture Techniques	MRPS35	0.000166	-2.52774	
8873	Cell Culture Techniques	PPIG	0.000425	-2.505525	
8874	Cell Culture Techniques	GUSB	0.00007	-2.451689	
8875	Cell Culture Techniques	ANXA4	0.000425	-2.440149	
8876	Cell Culture Techniques	ZNF165	0.000062	-2.402117	
8877	Cell Culture Techniques	TMEM41B	0.000615	-2.399405	
8878	Cell Culture Techniques	ZNF721	0.000664	-2.377136	
8879	Cell Culture Techniques	ZNF800	0.000597	-2.347068	
8880	Cell Culture Techniques	C4ORF16	0.000093	-2.332935	
8881	Cell Culture Techniques	FLJ34969	0.000597	-2.282324	
8882	Cell Culture Techniques	CCDC76	0.000599	-2.225771	
8883	Cell Culture Techniques	TARDBP	0.000574	-2.221869	
8884	Cell Culture Techniques	RNF141	0.000516	-2.206677	
8885	Cell Culture Techniques	C11ORF71	0.000545	-2.102567	
8886	Cell Culture Techniques	RPS24	0.000286	-2.072647	
8887	Cell Culture Techniques	DGKD	0.000219	-2.026221	
8888	Cell Culture Techniques	RPL5	0.000282	-2.004885	
8889	Cell Culture Techniques	RGS10	0.000375	2.006736	
8890	Cell Culture Techniques	CCDC127	0.000514	2.023144	
8891	Cell Culture Techniques	RBMS2	0.000284	2.071696	
8892	Cell Culture Techniques	TNFAIP1	0.000398	2.089194	
8893	Cell Culture Techniques	GAPDH	0.000425	2.158625	
8894	Cell Culture Techniques	ADAMTSL5	0.000486	2.27493	
8895	Cell Culture Techniques	NPR2	0.000399	2.282587	
8896	Cell Culture Techniques	SDF4	0.000665	2.289924	
8897	Cell Culture Techniques	MST150	0.000125	2.311495	
8898	Cell Culture Techniques	SEC22C	0.000369	2.345013	

8899	Cell Culture Techniques	NTAN1	0.00061	2.400488	
8900	Cell Culture Techniques	RIC8A	0.000436	2.464435	
8901	Cell Culture Techniques	ZBTB4	0.000201	2.481596	
8902	Cell Culture Techniques	SLC35E1	0.000436	2.585884	
8903	Cell Culture Techniques	PNMA1	0.000603	2.646874	
8904	Cell Culture Techniques	ABI2	0.000191	2.657783	
8905	Cell Culture Techniques	PVRL2	0.000187	2.739545	
8906	Cell Culture Techniques	M-RIP	0.000292	2.875153	
8907	Cell Culture Techniques	MPV17	0.000334	2.87788	
8908	Cell Culture Techniques	SPARC	0.000665	2.977244	
8909	Cell Culture Techniques	EXT1	0.000425	3.142807	
8910	Cell Culture Techniques	DYRK3	0.000062	3.199536	
8911	Cell Culture Techniques	TCEAL3	0.000602	3.283354	
8912	Cell Culture Techniques	VKORC1	0.000031	3.288607	
8913	Cell Culture Techniques	TPM1	0.000436	3.396558	
8914	Cell Culture Techniques	KIAA1754	0.000235	3.642638	
8915	Cell Culture Techniques	HSPB2	0.000603	3.783672	
8916	Cell Culture Techniques	FKSG30	0.000436	3.868274	
8917	Cell Culture Techniques	MYADM	0.000189	3.982227	
8918	Cell Culture Techniques	DFNA5	0.000086	4.008277	
8919	Cell Culture Techniques	PELO	0.000599	4.297751	
8920	Cell Culture Techniques	RAB6IP1	0.000219	4.356722	
8921	Cell Culture Techniques	NINJ1	0.00058	4.385068	
8922	Cell Culture Techniques	GABARAPL1	0.000031	4.474687	
8923	Cell Culture Techniques	ACTN1	0.000001	4.790828	
8924	Cell Culture Techniques	MSN	0.000155	4.827876	
8925	Cell Culture Techniques	VIM	0.000441	4.942041	
8926	Cell Culture Techniques	ACTA2	0.000155	5.024653	
8927	Cell Culture Techniques	CYR61	0.000093	5.299898	
8928	Cell Culture Techniques	DEGS1	0.000035	5.682363	
8929	Cell Culture Techniques	LGALS1	0.000071	5.933415	
8930	Cell Culture Techniques	NGFRAP1	0.000501	6.226585	
8931	Cell Culture Techniques	COL1A2	0.000375	10.191927	
8932	Cell Culture Techniques	FAM20C	0.000468	10.313947	
8933	Cell Culture Techniques	DKK3	0.000597	10.416492	
8934	Cell Culture Techniques	VASN	0.000441	12.264142	
8935	Cell Separation	CFH	0.001969	-11.140652	
8936	Cell Separation	RBM15	0.004791	-3.808127	
8937	Cell Separation	UQCC	0.005408	-3.337098	
8938	Cell Separation	PDCD6	0.000732	-3.30841	
8939	Cell Separation	HELLS	0.004596	-3.245423	
8940	Cell Separation	ZNF160	0.004021	-3.209786	
8941	Cell Separation	POLR2J2	0.004215	-3.038889	
8942	Cell Separation	ZNF207	0.004792	-2.972298	
8943	Cell Separation	WHSC1	0.001268	-2.880382	
8944	Cell Separation	LOC100129637	0.002981	-2.861468	
8945	Cell Separation	PLXND1	0.003286	-2.855506	
8946	Cell Separation	AFG3L1P	0.002109	-2.846511	
8947	Cell Separation	LOC595101	0.000327	-2.818768	
8948	Cell Separation	BMS1P1	0.000316	-2.804696	

8949	Cell Separation	PRKDC	0.00241	-2.803191	
8950	Cell Separation	ANGPT1	0.00071	-2.796247	
8951	Cell Separation	FAM173B	0.003152	-2.753963	
8952	Cell Separation	CDCA7	0.004748	-2.753797	
8953	Cell Separation	RGNEF	0.005098	-2.707656	
8954	Cell Separation	SRSF4	0.002427	-2.698683	
8955	Cell Separation	MMRN2	0.001969	-2.660392	
8956	Cell Separation	KIAA1377	0.001162	-2.631271	
8957	Cell Separation	ATP2C2	0.005467	-2.576461	
8958	Cell Separation	CDKN2C	0.001025	-2.504487	
8959	Cell Separation	LOC399753	0.006485	-2.480526	
8960	Cell Separation	CRIPAK	0.005174	-2.417205	
8961	Cell Separation	PTK2	0.001884	-2.380796	
8962	Cell Separation	IFT80	0.003512	-2.361122	
8963	Cell Separation	C2orf67	0.003336	-2.341392	
8964	Cell Separation	ZADH2	0.006011	-2.325923	
8965	Cell Separation	CREBZF	0.001794	-2.300725	
8966	Cell Separation	MYO19	0.006064	-2.275494	
8967	Cell Separation	CHD3	0.004639	-2.229819	
8968	Cell Separation	NOC2L	0.003181	-2.226973	
8969	Cell Separation	BRCA1	0.005839	-2.224053	
8970	Cell Separation	NF1	0.004108	-2.212946	
8971	Cell Separation	CHD9	0.004107	-2.20314	
8972	Cell Separation	XPO1	0.000517	-2.193761	
8973	Cell Separation	TTC23	0.005733	-2.18306	
8974	Cell Separation	EML4	0.002343	-2.177747	
8975	Cell Separation	SGK269	0.005301	-2.172195	
8976	Cell Separation	PHKA2	0.002728	-2.171707	
8977	Cell Separation	RBBP4	0.005467	-2.163389	
8978	Cell Separation	LOC100507153	0.002967	-2.133094	
8979	Cell Separation	ZBTB44	0.000125	-2.120909	
8980	Cell Separation	WDR90	0.003152	-2.115398	
8981	Cell Separation	PATZ1	0.002785	-2.11183	
8982	Cell Separation	ZNF248	0.004792	-2.098686	
8983	Cell Separation	SFRS4	0.003692	-2.091499	
8984	Cell Separation	SGK494	0.001756	-2.052257	
8985	Cell Separation	USP13	0.001498	-2.050189	
8986	Cell Separation	ALDH7A1	0.004792	-2.049889	
8987	Cell Separation	MPHOSPH9	0.004116	-2.020827	
8988	Cell Separation	KIAA0467	0.002485	-2.017488	
8989	Cell Separation	OSBPL3	0.000732	2.00378	
8990	Cell Separation	SERTAD1	0.003463	2.024163	
8991	Cell Separation	JMJD6	0.001662	2.039276	
8992	Cell Separation	RAP2B	0.000671	2.040388	
8993	Cell Separation	NDFIP2	0.002765	2.051303	
8994	Cell Separation	TRIM15	0.006381	2.052602	
8995	Cell Separation	IFNAR2	0.002588	2.053709	
8996	Cell Separation	HN1	0.006181	2.061183	
8997	Cell Separation	SLC25A25	0.00301	2.074173	
8998	Cell Separation	KLF10	0.001147	2.087965	

8999	Cell Separation	SBDS	0.001552	2.112631	
9000	Cell Separation	ATP6V0E1	0.005141	2.127361	
9001	Cell Separation	RNF7	0.004791	2.144269	
9002	Cell Separation	LHFPL2	0.006011	2.147585	
9003	Cell Separation	HIST1H2BF	0.000517	2.162432	
9004	Cell Separation	RHPN2	0.003692	2.162838	
9005	Cell Separation	MAP1LC3B	0.004108	2.164625	
9006	Cell Separation	ALAS1	0.004861	2.183211	
9007	Cell Separation	HIST1H2BE	0.003152	2.190406	
9008	Cell Separation	SELK	0.000728	2.191273	
9009	Cell Separation	CDV3	0.005041	2.199458	
9010	Cell Separation	PERP	0.00022	2.202057	
9011	Cell Separation	HLA-F	0.004108	2.207022	
9012	Cell Separation	AMOTL2	0.001552	2.213932	
9013	Cell Separation	PPP2CB	0.006104	2.239827	
9014	Cell Separation	ARL8B	0.005085	2.245124	
9015	Cell Separation	CFLAR	0.003477	2.250863	
9016	Cell Separation	DNAJB1	0.00241	2.280388	
9017	Cell Separation	PDE12	0.001268	2.282691	
9018	Cell Separation	S100A11	0.0036	2.290681	
9019	Cell Separation	IER5	0.004748	2.297363	
9020	Cell Separation	ARL5B	0.000244	2.30292	
9021	Cell Separation	FOXN2	0.001756	2.30656	
9022	Cell Separation	ERRFI1	0.004065	2.30757	
9023	Cell Separation	NFKBIE	0.005973	2.353556	
9024	Cell Separation	MTHFSD	0.005298	2.359503	
9025	Cell Separation	STK17A	0.000125	2.396145	
9026	Cell Separation	HIGD1A	0.001756	2.400591	
9027	Cell Separation	PLCXD2	0.004147	2.435619	
9028	Cell Separation	HIST1H2BI	0.002829	2.438257	
9029	Cell Separation	GLS	0.001794	2.44452	
9030	Cell Separation	TPMT	0.004143	2.452997	
9031	Cell Separation	PIM1	0.001147	2.458919	
9032	Cell Separation	LGALS8	0.001679	2.471341	
9033	Cell Separation	CCDC51	0.006104	2.480221	
9034	Cell Separation	HIST1H2BK	0.001662	2.514361	
9035	Cell Separation	SERTAD2	0.0044	2.534205	
9036	Cell Separation	AREG	0.000389	2.539078	
9037	Cell Separation	OCLN	0.001052	2.551247	
9038	Cell Separation	CPEB2	0.005988	2.565899	
9039	Cell Separation	MDFI	0.004314	2.570407	
9040	Cell Separation	HCCS	0.004748	2.584927	
9041	Cell Separation	C6orf132	0.001087	2.612396	
9042	Cell Separation	RAB22A	0.001756	2.613734	
9043	Cell Separation	ANXA3	0.00508	2.635969	
9044	Cell Separation	MXD1	0.000125	2.649865	
9045	Cell Separation	RALA	0.002459	2.658884	
9046	Cell Separation	TPM4	0.003336	2.660044	
9047	Cell Separation	TNFSF9	0.000316	2.669576	
9048	Cell Separation	RND3	0.000125	2.709598	

9049	Cell Separation	TIPARP	0.004639	2.712231	
9050	Cell Separation	DUSP10	0.000754	2.723635	
9051	Cell Separation	SFN	0.004323	2.72591	
9052	Cell Separation	EPB41L5	0.00032	2.728434	
9053	Cell Separation	KLF4	0.001474	2.76071	
9054	Cell Separation	OBFC2A	0.003595	2.787294	
9055	Cell Separation	TAP1	0.003545	2.824943	
9056	Cell Separation	FGD6	0.004373	2.855279	
9057	Cell Separation	NAMPT	0.005085	2.873318	
9058	Cell Separation	ZNF165	0.002504	2.875868	
9059	Cell Separation	CLDN4	0.00071	2.911135	
9060	Cell Separation	SEMA7A	0.001969	2.932253	
9061	Cell Separation	GJB3	0.004107	2.938013	
9062	Cell Separation	NFKBIB	0.003234	2.941764	
9063	Cell Separation	TAGAP	0.001969	2.980919	
9064	Cell Separation	CD47	0.005041	2.995743	
9065	Cell Separation	EZR	0.00178	3.023584	
9066	Cell Separation	CLIC5	0.004147	3.081047	
9067	Cell Separation	DPH3	0.001748	3.091732	
9068	Cell Separation	LAMB3	0.005141	3.103992	
9069	Cell Separation	LMO7	0.005034	3.110319	
9070	Cell Separation	LOC100506979	0.004639	3.130702	
9071	Cell Separation	HIST1H2BH	0.000517	3.147934	
9072	Cell Separation	TICAM1	0.000191	3.159202	
9073	Cell Separation	GAN	0.002595	3.170409	
9074	Cell Separation	IL23A	0.001147	3.182919	
9075	Cell Separation	LOC440896	0.001764	3.190618	
9076	Cell Separation	CLDN23	0.005467	3.243061	
9077	Cell Separation	H2BFS	0.00071	3.246703	
9078	Cell Separation	CHMP2B	0.000732	3.259293	
9079	Cell Separation	MAFF	0.00071	3.270931	
9080	Cell Separation	HIST1H2BD	0.001211	3.297822	
9081	Cell Separation	GADD45A	0.005141	3.349209	
9082	Cell Separation	IFRD1	0.000229	3.364611	
9083	Cell Separation	DDA1	0.006252	3.442754	
9084	Cell Separation	USP53	0.003981	3.463071	
9085	Cell Separation	RHOF	0.00178	3.465639	
9086	Cell Separation	RAB9A	0.003181	3.471487	
9087	Cell Separation	PPP1R15A	0.006064	3.541813	
9088	Cell Separation	LOC439990	0.004107	3.5739	
9089	Cell Separation	ID2	0.003434	3.739528	
9090	Cell Separation	HIST1H2BG	0.004577	3.788688	
9091	Cell Separation	PPP4R1L	0.003181	3.813426	
9092	Cell Separation	GPRC5A	0.001717	3.866214	
9093	Cell Separation	EDN1	0.001662	3.996734	
9094	Cell Separation	KLF6	0.000125	4.047716	
9095	Cell Separation	ZFAND2A	0.001662	4.179808	
9096	Cell Separation	TNFAIP3	0.001662	4.181002	
9097	Cell Separation	FOSL1	0.002785	4.261609	
9098	Cell Separation	HIST2H2AA3	0.002775	4.307458	

9099	Cell Separation	CXCL16	0.001552	4.316383	
9100	Cell Separation	NCEH1	0.004107	4.415097	
9101	Cell Separation	PMAIP1	0.002949	4.535741	
9102	Cell Separation	TUBB2A	0.001648	4.71727	
9103	Cell Separation	TM4SF1	0.004059	4.932563	
9104	Cell Separation	NFKBIA	0.004108	5.051207	
9105	Cell Separation	KIAA1609	0.003254	5.184877	
9106	Cell Separation	DUSP5	0.001662	5.413098	
9107	Cell Separation	EMP1	0.001764	5.681152	
9108	Cell Separation	PLAUR	0.000229	5.92149	
9109	Cell Separation	IL32	0.002728	6.867646	
9110	Cell Separation	C10orf116	0.003181	7.514991	
9111	Cell Separation	BIRC3	0.00642	7.908702	
9112	Cell Separation	LAMC2	0.003986	9.26201	
9113	Cell Separation	CDA	0.005862	9.815259	
9114	Chemical extraction	COL1A1	0	-5.27529	
9115	Chemical extraction	MMP7	0	-4.132738	
9116	Chemical extraction	SPP1	0	-4.130239	
9117	Chemical extraction	IL8	0	-3.187774	
9118	Chemical extraction	KRT23	0	-3.15084	
9119	Chemical extraction	COL1A2	0	-3.116379	
9120	Chemical extraction	TGFBI	0	-2.936139	
9121	Chemical extraction	INHBA	0	-2.666466	
9122	Chemical extraction	HKDC1	0	-2.660582	
9123	Chemical extraction	NFE2L3	0	-2.463859	
9124	Chemical extraction	CCL20	0	-2.310437	
9125	Chemical extraction	SFRP4	0	-2.294594	
9126	Chemical extraction	MMP12	0	-2.283803	
9127	Chemical extraction	COL10A1	0	-2.176511	
9128	Chemical extraction	MYC	0	-2.131113	
9129	Chemical extraction	JUB	0	-2.121437	
9130	Chemical extraction	GZMB	0	-2.088884	
9131	Chemical extraction	CDC123	0	2.016474	
9132	Chemical extraction	C6orf118	0	2.143404	
9133	Chemical extraction	CD1A	0	2.195462	
9134	Chemical extraction	NUDCD1	0	2.265265	
9135	Chemical extraction	ENPEP	0	2.363024	
9136	Colon adenoma	SYNE1	0.000014	-6.563564	
9137	Colon adenoma	MEIS1	0	-5.618535	
9138	Colon adenoma	TRPC1	0	-5.588125	
9139	Colon adenoma	FBLN1	0	-5.117333	
9140	Colon adenoma	CDH19	0	-5.022701	
9141	Colon adenoma	GZMK	0.000021	-4.771295	
9142	Colon adenoma	ADAMDEC1	0	-4.340817	
9143	Colon adenoma	TRAF3IP3	0.000018	-4.30323	
9144	Colon adenoma	LOC399959	0.000022	-4.287609	
9145	Colon adenoma	CXCL12	0.000005	-4.244649	
9146	Colon adenoma	CCL2	0.000015	-4.121636	
9147	Colon adenoma	LAMA2	0	-4.08393	
9148	Colon adenoma	NLGN4X	0	-4.057908	

9149	Colon adenoma	PCDH19	0.00002	-3.957706	
9150	Colon adenoma	DPYD	0	-3.957465	
9151	Colon adenoma	RBP5	0.00001	-3.891229	
9152	Colon adenoma	CPNE8	0	-3.806743	
9153	Colon adenoma	PPP1R16B	0.000004	-3.797815	
9154	Colon adenoma	SCGN	0.000009	-3.779157	
9155	Colon adenoma	ADAMTS1	0.000015	-3.716882	
9156	Colon adenoma	GPC6	0	-3.69371	
9157	Colon adenoma	DOCK10	0	-3.6903	
9158	Colon adenoma	RASGRP3	0	-3.606281	
9159	Colon adenoma	TNXA	0.000016	-3.570317	
9160	Colon adenoma	LPHN2	0	-3.519243	
9161	Colon adenoma	CP	0.000005	-3.490476	
9162	Colon adenoma	ZEB2	0	-3.428728	
9163	Colon adenoma	VCAM1	0.000004	-3.409172	
9164	Colon adenoma	FRMD6	0	-3.373895	
9165	Colon adenoma	PCDH7	0.000022	-3.361762	
9166	Colon adenoma	CDH2	0.000003	-3.347133	
9167	Colon adenoma	FAM107A	0.000004	-3.324142	
9168	Colon adenoma	PDE2A	0	-3.322784	
9169	Colon adenoma	SEMA6D	0.000009	-3.321159	
9170	Colon adenoma	CHL1	0.000017	-3.308172	
9171	Colon adenoma	FAM110B	0.000001	-3.283492	
9172	Colon adenoma	C20orf194	0	-3.28122	
9173	Colon adenoma	SFMBT2	0.000001	-3.24373	
9174	Colon adenoma	EMCN	0.000009	-3.242664	
9175	Colon adenoma	CCDC80	0	-3.229232	
9176	Colon adenoma	WIPF1	0.000006	-3.18889	
9177	Colon adenoma	MIR214	0.000015	-3.185454	
9178	Colon adenoma	PTPRC	0.000015	-3.171861	
9179	Colon adenoma	EBF1	0.000026	-3.164785	
9180	Colon adenoma	SPARCL1	0	-3.124702	
9181	Colon adenoma	C1S	0	-3.112694	
9182	Colon adenoma	ZCCHC24	0	-3.100495	
9183	Colon adenoma	SCARA5	0.000006	-3.070863	
9184	Colon adenoma	DCN	0.000005	-3.053733	
9185	Colon adenoma	SLIT2	0.000006	-3.014042	
9186	Colon adenoma	PMP22	0.000002	-3.011268	
9187	Colon adenoma	GIMAP6	0	-3.01085	
9188	Colon adenoma	MAF	0	-3.003109	
9189	Colon adenoma	CFH	0	-2.998647	
9190	Colon adenoma	GNB4	0.000011	-2.991988	
9191	Colon adenoma	RERG	0	-2.9816	
9192	Colon adenoma	MCC	0	-2.970271	
9193	Colon adenoma	ADAM28	0.00001	-2.969096	
9194	Colon adenoma	ITPR1	0.000003	-2.958448	
9195	Colon adenoma	NR3C1	0	-2.950447	
9196	Colon adenoma	CLIP4	0.000009	-2.937125	
9197	Colon adenoma	CACNA2D1	0.000001	-2.935371	
9198	Colon adenoma	FGL2	0	-2.932828	

9199	Colon adenoma	FAM126A	0.000002	-2.92626	
9200	Colon adenoma	ITIH5	0	-2.924913	
9201	Colon adenoma	ADAP2	0.000006	-2.907607	
9202	Colon adenoma	GIMAP7	0	-2.904896	
9203	Colon adenoma	XKR4	0.000027	-2.89146	
9204	Colon adenoma	COL14A1	0.000027	-2.880001	
9205	Colon adenoma	LIFR	0.000027	-2.862675	
9206	Colon adenoma	LOC100132891	0.000007	-2.857789	
9207	Colon adenoma	ATP8B2	0.000011	-2.842167	
9208	Colon adenoma	RDX	0	-2.833988	
9209	Colon adenoma	RFTN1	0.000018	-2.831749	
9210	Colon adenoma	SNAI2	0.000004	-2.825814	
9211	Colon adenoma	SNAP25	0.000026	-2.820829	
9212	Colon adenoma	ITM2A	0.000004	-2.813288	
9213	Colon adenoma	LOC100506621	0.000027	-2.813074	
9214	Colon adenoma	GNG2	0	-2.792069	
9215	Colon adenoma	TBC1D9	0	-2.789546	
9216	Colon adenoma	CCDC8	0.000013	-2.786944	
9217	Colon adenoma	CMAH	0.000015	-2.777597	
9218	Colon adenoma	SSBP2	0.000003	-2.772938	
9219	Colon adenoma	TCF21	0.000002	-2.771469	
9220	Colon adenoma	CD48	0.00002	-2.756235	
9221	Colon adenoma	CLIC2	0.000004	-2.754874	
9222	Colon adenoma	KLRK1	0.000001	-2.753093	
9223	Colon adenoma	JAM2	0	-2.74367	
9224	Colon adenoma	HIC1	0	-2.712245	
9225	Colon adenoma	ST8SIA4	0.000004	-2.708861	
9226	Colon adenoma	COLEC12	0.000001	-2.704382	
9227	Colon adenoma	ACP5	0.000011	-2.698381	
9228	Colon adenoma	HLA-DPA1	0.000001	-2.685678	
9229	Colon adenoma	DOCK2	0.000011	-2.677831	
9230	Colon adenoma	QKI	0.000001	-2.669064	
9231	Colon adenoma	DZIP1	0.000004	-2.664127	
9232	Colon adenoma	MAB21L2	0	-2.662187	
9233	Colon adenoma	ASPN	0.00001	-2.658627	
9234	Colon adenoma	LOC728392	0.000002	-2.655484	
9235	Colon adenoma	SPG20	0.000013	-2.650279	
9236	Colon adenoma	NPAS3	0.000004	-2.641488	
9237	Colon adenoma	MEF2C	0.000009	-2.629412	
9238	Colon adenoma	IGFBP3	0.000009	-2.597797	
9239	Colon adenoma	MPEG1	0.000005	-2.595565	
9240	Colon adenoma	LSP1	0.000003	-2.589832	
9241	Colon adenoma	GIMAP4	0	-2.582242	
9242	Colon adenoma	NLRC3	0.000001	-2.580819	
9243	Colon adenoma	CYGB	0	-2.574414	
9244	Colon adenoma	AQP1	0.000021	-2.561552	
9245	Colon adenoma	ITGA4	0	-2.560951	
9246	Colon adenoma	EFEMP1	0.000005	-2.560292	
9247	Colon adenoma	ITGAL	0.000014	-2.552855	
9248	Colon adenoma	PRICKLE1	0.000006	-2.552477	

9249	Colon adenoma	FYB	0.000003	-2.54851	
9250	Colon adenoma	CCR2	0.000009	-2.545477	
9251	Colon adenoma	WWTR1	0	-2.544909	
9252	Colon adenoma	IGF1	0.000006	-2.543039	
9253	Colon adenoma	LPAR1	0	-2.541552	
9254	Colon adenoma	LOC100128252	0.000019	-2.538955	
9255	Colon adenoma	FAM55C	0.000009	-2.534637	
9256	Colon adenoma	C14orf139	0.000001	-2.533271	
9257	Colon adenoma	GIMAP1	0.00001	-2.527932	
9258	Colon adenoma	RAB34	0.000001	-2.511206	
9259	Colon adenoma	DOCK8	0.000023	-2.505324	
9260	Colon adenoma	SDPR	0	-2.501807	
9261	Colon adenoma	TRBC1	0.000025	-2.501359	
9262	Colon adenoma	MYLK	0.000001	-2.491934	
9263	Colon adenoma	SERPINF1	0.000012	-2.489622	
9264	Colon adenoma	EMILIN1	0.000018	-2.488391	
9265	Colon adenoma	RHOJ	0.000001	-2.478906	
9266	Colon adenoma	SETBP1	0.000004	-2.475948	
9267	Colon adenoma	PRKAR2B	0	-2.475068	
9268	Colon adenoma	MSN	0.000019	-2.474954	
9269	Colon adenoma	GIMAP8	0.000018	-2.474757	
9270	Colon adenoma	TCF4	0.000003	-2.46412	
9271	Colon adenoma	WAS	0.000016	-2.463565	
9272	Colon adenoma	EFS	0.000001	-2.461236	
9273	Colon adenoma	LOX	0.000005	-2.460449	
9274	Colon adenoma	EDIL3	0.000027	-2.455198	
9275	Colon adenoma	RUNX1T1	0	-2.446776	
9276	Colon adenoma	TMEM47	0.000001	-2.445538	
9277	Colon adenoma	COL28A1	0.000013	-2.435502	
9278	Colon adenoma	GAS7	0.000016	-2.4351	
9279	Colon adenoma	ZNF542	0.000014	-2.432356	
9280	Colon adenoma	HLA-DPB1	0.000004	-2.42914	
9281	Colon adenoma	CLEC2B	0.000012	-2.426989	
9282	Colon adenoma	IL18BP	0.00002	-2.423986	
9283	Colon adenoma	GLI1	0.000009	-2.42391	
9284	Colon adenoma	CSF1R	0.000004	-2.42014	
9285	Colon adenoma	ARHGEF17	0.000001	-2.412614	
9286	Colon adenoma	MXD1	0.000011	-2.401134	
9287	Colon adenoma	CYSLTR1	0	-2.399178	
9288	Colon adenoma	FXYD6	0.000002	-2.395755	
9289	Colon adenoma	MPDZ	0.000005	-2.394859	
9290	Colon adenoma	TIMP3	0.000001	-2.394643	
9291	Colon adenoma	CHRDL1	0.000014	-2.391992	
9292	Colon adenoma	THSD7A	0.000006	-2.39099	
9293	Colon adenoma	ZFPM2	0	-2.389381	
9294	Colon adenoma	RGMA	0.000018	-2.388756	
9295	Colon adenoma	LDB2	0.000015	-2.378466	
9296	Colon adenoma	GZMA	0.000012	-2.377783	
9297	Colon adenoma	CPM	0.000022	-2.370853	
9298	Colon adenoma	EVI2B	0.000026	-2.370211	

9299	Colon adenoma	CD36	0	-2.36807	
9300	Colon adenoma	FAM13C	0.000001	-2.367818	
9301	Colon adenoma	ZNF304	0.000001	-2.36777	
9302	Colon adenoma	EVL	0.000017	-2.365015	
9303	Colon adenoma	OLFML1	0	-2.352103	
9304	Colon adenoma	RECK	0	-2.348067	
9305	Colon adenoma	CALHM2	0.000001	-2.344725	
9306	Colon adenoma	SPARC	0.000001	-2.342933	
9307	Colon adenoma	FAM78A	0.000021	-2.339397	
9308	Colon adenoma	EPB41L3	0.000002	-2.336952	
9309	Colon adenoma	CD3D	0.000015	-2.333476	
9310	Colon adenoma	LUM	0.000004	-2.332403	
9311	Colon adenoma	NRP1	0.000002	-2.319185	
9312	Colon adenoma	NDN	0	-2.313748	
9313	Colon adenoma	FNBP1	0.000007	-2.312437	
9314	Colon adenoma	IL6R	0	-2.308615	
9315	Colon adenoma	ITK	0.00002	-2.305504	
9316	Colon adenoma	SCG2	0.000016	-2.300982	
9317	Colon adenoma	ANTXR1	0.000015	-2.293843	
9318	Colon adenoma	MYO5A	0.000001	-2.291889	
9319	Colon adenoma	AXL	0.000021	-2.290225	
9320	Colon adenoma	RCAN1	0.000002	-2.289081	
9321	Colon adenoma	ECSCR	0.000017	-2.284871	
9322	Colon adenoma	STOX2	0.000013	-2.27665	
9323	Colon adenoma	C6orf204	0.000014	-2.262931	
9324	Colon adenoma	MSRB3	0.000006	-2.25566	
9325	Colon adenoma	RBMS1	0	-2.242471	
9326	Colon adenoma	DKK3	0	-2.238933	
9327	Colon adenoma	ARHGAP25	0.000013	-2.238708	
9328	Colon adenoma	TNFRSF25	0.000015	-2.23356	
9329	Colon adenoma	CD86	0.000017	-2.21729	
9330	Colon adenoma	JAZF1	0.000001	-2.210426	
9331	Colon adenoma	MS4A6A	0.000006	-2.202884	
9332	Colon adenoma	FERMT2	0	-2.20204	
9333	Colon adenoma	C14orf132	0.000001	-2.192972	
9334	Colon adenoma	STAB1	0.000004	-2.188313	
9335	Colon adenoma	TARP	0.000004	-2.185572	
9336	Colon adenoma	AKAP12	0	-2.18236	
9337	Colon adenoma	ETS1	0.000009	-2.177302	
9338	Colon adenoma	ARMCX1	0.000001	-2.17268	
9339	Colon adenoma	HLA-DOA	0.000026	-2.159713	
9340	Colon adenoma	CD3E	0.000009	-2.159225	
9341	Colon adenoma	PDGFRA	0.000012	-2.158381	
9342	Colon adenoma	OLFML3	0.000002	-2.154377	
9343	Colon adenoma	HEG1	0.000007	-2.150429	
9344	Colon adenoma	CRYAB	0	-2.149325	
9345	Colon adenoma	CYLD	0.000003	-2.139364	
9346	Colon adenoma	C10orf72	0.000002	-2.12672	
9347	Colon adenoma	CTSC	0.000005	-2.116743	
9348	Colon adenoma	LHFP	0	-2.111542	

9349	Colon adenoma	FLI1	0.000014	-2.109904	
9350	Colon adenoma	PREX2	0.000014	-2.108912	
9351	Colon adenoma	PDLIM3	0.000001	-2.107972	
9352	Colon adenoma	PTPRM	0.000026	-2.103882	
9353	Colon adenoma	CALD1	0	-2.099286	
9354	Colon adenoma	DDR2	0	-2.098418	
9355	Colon adenoma	HLA-DRB1	0.000007	-2.095995	
9356	Colon adenoma	CMTM3	0.000019	-2.095868	
9357	Colon adenoma	ANGPT1	0.000001	-2.084996	
9358	Colon adenoma	ST7L	0.000001	-2.079927	
9359	Colon adenoma	ARHGEF6	0.000013	-2.079279	
9360	Colon adenoma	PTPN22	0	-2.078373	
9361	Colon adenoma	AP1S2	0	-2.078222	
9362	Colon adenoma	LAYN	0.000006	-2.076001	
9363	Colon adenoma	PRDM1	0.000004	-2.074792	
9364	Colon adenoma	TNS1	0.000001	-2.071872	
9365	Colon adenoma	AKAP2	0	-2.069514	
9366	Colon adenoma	S1PR1	0.000005	-2.059119	
9367	Colon adenoma	GIMAP5	0.000009	-2.058582	
9368	Colon adenoma	AMOTL1	0.000017	-2.055109	
9369	Colon adenoma	JAM3	0.000002	-2.051995	
9370	Colon adenoma	PLOD2	0.000001	-2.048507	
9371	Colon adenoma	ZEB1	0.000002	-2.044585	
9372	Colon adenoma	CTSZ	0.000023	-2.03718	
9373	Colon adenoma	LAMA4	0.000027	-2.027234	
9374	Colon adenoma	ECM2	0	-2.01929	
9375	Colon adenoma	TSC22D3	0.000004	-2.018925	
9376	Colon adenoma	RAB37	0.000019	-2.009905	
9377	Colon adenoma	RBMS3	0.000001	-2.002071	
9378	Colon adenoma	CCDC113	0.000018	2.049514	
9379	Colon adenoma	ITGA6	0.000001	2.117016	
9380	Colon adenoma	ZNRF3	0.00001	2.73187	
9381	Colon Carcinoma	MAPK10	0.010117	-4.758095	
9382	Colon Carcinoma	DLGAP4	0.009754	-2.082438	
9383	Colon Carcinoma	NOC2L	0.026594	2.104099	
9384	Colon Carcinoma	MORC4	0.004279	2.112565	
9385	Colon Carcinoma	LOC90835	0.001125	2.166442	
9386	Colon Carcinoma	SFRS3	0.023214	2.178891	
9387	Colon Carcinoma	EXOSC8	0.023557	2.246594	
9388	Colon Carcinoma	COMT	0.0087	2.27312	
9389	Colon Carcinoma	DARS2	0.003721	2.328628	
9390	Colon Carcinoma	ENC1	0.005205	2.353768	
9391	Colon Carcinoma	CSPP1	0.004279	2.417897	
9392	Colon Carcinoma	RPL35A	0.023557	2.488761	
9393	Colon Carcinoma	SLC7A6	0.005899	2.538138	
9394	Colon Carcinoma	UBE2V1	0.004279	2.625998	
9395	Colon Carcinoma	MCM8	0.004563	2.888232	
9396	Colon Carcinoma	C13orf3	0.010117	3.221261	
9397	Colon Carcinoma	BUB1	0.003759	3.279526	
9398	Colon Carcinoma	FANCI	0.0087	3.309157	

9399	Colon Carcinoma	CDC6	0.011855	3.551337	
9400	Colon Carcinoma	WDSOF1	0.001352	3.709982	
9401	Colon Carcinoma	RELL2	0.003759	3.855649	
9402	Colon Carcinoma	CCDC86	0.002201	3.867938	
9403	Colon Carcinoma	E2F5	0.005272	3.890783	
9404	Colon Carcinoma	XPOT	0.014459	3.992622	
9405	Colon Carcinoma	KIF20A	0.000391	5.468525	
9406	Colon Carcinoma	HMMR	0.000025	5.804377	
9407	Colonic Neoplasms	RFTN1	0	-143.024155	
9408	Colonic Neoplasms	CHI3L1	0	-36.962963	
9409	Colonic Neoplasms	HLA-DRA	0	-35.365312	
9410	Colonic Neoplasms	CD53	0	-32.87	
9411	Colonic Neoplasms	PLSCR4	0	-26.631579	
9412	Colonic Neoplasms	ETS1	0	-23.917786	
9413	Colonic Neoplasms	APBB1IP	0	-23.133745	
9414	Colonic Neoplasms	NNMT	0	-20.655502	
9415	Colonic Neoplasms	SYNPO2	0	-20.006999	
9416	Colonic Neoplasms	HLA-C	0	-19.127079	
9417	Colonic Neoplasms	CXCR4	0	-18.861111	
9418	Colonic Neoplasms	FRMD6	0	-17.813401	
9419	Colonic Neoplasms	CD93	0	-17.171569	
9420	Colonic Neoplasms	COL6A3	0	-16.084115	
9421	Colonic Neoplasms	COL5A1	0	-15.698732	
9422	Colonic Neoplasms	ADH1B	0.000163	-14.659768	
9423	Colonic Neoplasms	RAB31	0	-14.615832	
9424	Colonic Neoplasms	DDR2	0	-14.540682	
9425	Colonic Neoplasms	HLA-DQA1	0	-14.451419	
9426	Colonic Neoplasms	MPEG1	0	-13.380615	
9427	Colonic Neoplasms	LOC387763	0	-12.319389	
9428	Colonic Neoplasms	CYR61	0	-11.912235	
9429	Colonic Neoplasms	TYROBP	0	-11.03887	
9430	Colonic Neoplasms	NTN2L	0	-10.973491	
9431	Colonic Neoplasms	PLN	0.000977	-10.884812	
9432	Colonic Neoplasms	SLC26A3	0.000007	-10.831482	
9433	Colonic Neoplasms	CA7	0.000012	-10.114109	
9434	Colonic Neoplasms	PMP22	0	-9.964404	
9435	Colonic Neoplasms	LAPTM5	0	-9.543955	
9436	Colonic Neoplasms	CA1	0	-9.053477	
9437	Colonic Neoplasms	RGS2	0	-8.964338	
9438	Colonic Neoplasms	DUSP1	0	-8.9331	
9439	Colonic Neoplasms	CLU	0.000387	-8.79937	
9440	Colonic Neoplasms	SGCD	0	-8.547325	
9441	Colonic Neoplasms	AQP3	0	-8.176899	
9442	Colonic Neoplasms	GREM2	0	-7.997592	
9443	Colonic Neoplasms	HEG1	0	-7.925011	
9444	Colonic Neoplasms	MS4A6A	0	-7.855981	
9445	Colonic Neoplasms	INSL5	0.000002	-7.817222	
9446	Colonic Neoplasms	MYH11	0.000259	-7.704745	
9447	Colonic Neoplasms	CHGA	0.000013	-7.627212	
9448	Colonic Neoplasms	JCLN	0	-7.580735	

9449	Colonic Neoplasms	CTGF	0	-7.551444	
9450	Colonic Neoplasms	KRT20	0.00001	-7.551308	
9451	Colonic Neoplasms	SCNN1B	0.000009	-7.536482	
9452	Colonic Neoplasms	CFD	0	-7.220538	
9453	Colonic Neoplasms	HLA-DPB1	0	-7.119269	
9454	Colonic Neoplasms	FN1	0	-7.10163	
9455	Colonic Neoplasms	PKIB	0.000007	-6.929861	
9456	Colonic Neoplasms	CFI	0	-6.66807	
9457	Colonic Neoplasms	CA4	0	-6.659526	
9458	Colonic Neoplasms	PIGZ	0.00064	-6.641919	
9459	Colonic Neoplasms	LCP1	0	-6.638313	
9460	Colonic Neoplasms	OLFML2A	0	-6.633112	
9461	Colonic Neoplasms	SCARA5	0	-6.568566	
9462	Colonic Neoplasms	MYLK	0	-6.548969	
9463	Colonic Neoplasms	ANGPTL1	0	-6.533121	
9464	Colonic Neoplasms	ADAMTS9	0	-6.507463	
9465	Colonic Neoplasms	ALOX5AP	0	-6.301728	
9466	Colonic Neoplasms	CDH5	0	-6.20998	
9467	Colonic Neoplasms	PDE9A	0	-6.156069	
9468	Colonic Neoplasms	CLCA1	0	-6.035607	
9469	Colonic Neoplasms	SCGN	0	-6.014832	
9470	Colonic Neoplasms	CYBRD1	0	-5.945017	
9471	Colonic Neoplasms	PYY	0.000007	-5.934439	
9472	Colonic Neoplasms	AQP8	0	-5.791971	
9473	Colonic Neoplasms	LOH11CR2A	0	-5.768411	
9474	Colonic Neoplasms	C1RL	0	-5.755454	
9475	Colonic Neoplasms	IGJ	0.000115	-5.661065	
9476	Colonic Neoplasms	C1QA	0	-5.647079	
9477	Colonic Neoplasms	CLEC3B	0	-5.646384	
9478	Colonic Neoplasms	DNASE1L3	0	-5.645055	
9479	Colonic Neoplasms	SOCS3	0	-5.633423	
9480	Colonic Neoplasms	ADH4	0	-5.622253	
9481	Colonic Neoplasms	TNFAIP3	0	-5.612813	
9482	Colonic Neoplasms	HCLS1	0	-5.575544	
9483	Colonic Neoplasms	MUC4	0.000036	-5.548673	
9484	Colonic Neoplasms	LRRC19	0.000007	-5.393298	
9485	Colonic Neoplasms	CD177	0	-5.309023	
9486	Colonic Neoplasms	EDN3	0.000002	-5.280036	
9487	Colonic Neoplasms	APOL3	0	-5.279365	
9488	Colonic Neoplasms	ANPEP	0	-5.185258	
9489	Colonic Neoplasms	GUCA2B	0	-5.131965	
9490	Colonic Neoplasms	HIC1	0	-5.11955	
9491	Colonic Neoplasms	MUCDHL	0.000006	-5.11399	
9492	Colonic Neoplasms	PYY2	0.000003	-5.050842	
9493	Colonic Neoplasms	SDCBP2	0.000001	-5.00517	
9494	Colonic Neoplasms	VSIG2	0	-4.980351	
9495	Colonic Neoplasms	PFDN5	0	-4.975111	
9496	Colonic Neoplasms	SPIB	0.000001	-4.851853	
9497	Colonic Neoplasms	HTRA3	0	-4.850199	
9498	Colonic Neoplasms	RTN1	0.000009	-4.816378	

9499	Colonic Neoplasms	ENTPD5	0.00001	-4.795418	
9500	Colonic Neoplasms	FSTL1	0	-4.781491	
9501	Colonic Neoplasms	CTSO	0	-4.751652	
9502	Colonic Neoplasms	FLJ21511	0.000001	-4.750982	
9503	Colonic Neoplasms	CNR1	0	-4.666203	
9504	Colonic Neoplasms	TTR	0.000004	-4.645625	
9505	Colonic Neoplasms	WFDC1	0	-4.638388	
9506	Colonic Neoplasms	ABI3BP	0	-4.621097	
9507	Colonic Neoplasms	GCNT2	0.000158	-4.60045	
9508	Colonic Neoplasms	GUCA2A	0	-4.594706	
9509	Colonic Neoplasms	CILP	0	-4.580109	
9510	Colonic Neoplasms	ABCC13	0.00001	-4.56359	
9511	Colonic Neoplasms	LGALS4	0.000001	-4.552136	
9512	Colonic Neoplasms	CFHR1	0	-4.520135	
9513	Colonic Neoplasms	SLC2A4	0	-4.477195	
9514	Colonic Neoplasms	MGC4172	0	-4.475538	
9515	Colonic Neoplasms	PI16	0	-4.454597	
9516	Colonic Neoplasms	MS4A7	0	-4.432326	
9517	Colonic Neoplasms	TSPAN1	0.000001	-4.421269	
9518	Colonic Neoplasms	EVL	0	-4.374878	
9519	Colonic Neoplasms	NTN1	0	-4.369826	
9520	Colonic Neoplasms	FLJ32063	0.00001	-4.349534	
9521	Colonic Neoplasms	LGALS2	0.000009	-4.324702	
9522	Colonic Neoplasms	ADAMDEC1	0.000008	-4.322431	
9523	Colonic Neoplasms	SPINK5	0.000007	-4.320209	
9524	Colonic Neoplasms	MT1JP	0	-4.303425	
9525	Colonic Neoplasms	INHBA	0	-4.300894	
9526	Colonic Neoplasms	SERPINF1	0	-4.291443	
9527	Colonic Neoplasms	TNXA	0.000599	-4.287645	
9528	Colonic Neoplasms	TF	0.000015	-4.271676	
9529	Colonic Neoplasms	PLVAP	0	-4.256339	
9530	Colonic Neoplasms	LPHN3	0	-4.236286	
9531	Colonic Neoplasms	XKR4	0	-4.223866	
9532	Colonic Neoplasms	CES2	0.000001	-4.217799	
9533	Colonic Neoplasms	HLA-DOA	0	-4.194262	
9534	Colonic Neoplasms	P2RY13	0	-4.177498	
9535	Colonic Neoplasms	S100A9	0	-4.138247	
9536	Colonic Neoplasms	PLAC8	0.000012	-4.1363	
9537	Colonic Neoplasms	UGT2B4	0	-4.116567	
9538	Colonic Neoplasms	MYOM1	0	-4.090315	
9539	Colonic Neoplasms	EFS	0	-4.075452	
9540	Colonic Neoplasms	FXYD3	0	-4.060949	
9541	Colonic Neoplasms	C11orf33	0.000012	-4.060275	
9542	Colonic Neoplasms	CD84	0	-4.047519	
9543	Colonic Neoplasms	UGT2B7	0	-3.964991	
9544	Colonic Neoplasms	KCNE4	0	-3.956416	
9545	Colonic Neoplasms	ABCA1	0	-3.952417	
9546	Colonic Neoplasms	PPP1R14A	0	-3.931288	
9547	Colonic Neoplasms	NEU4	0.000001	-3.914172	
9548	Colonic Neoplasms	PCK1	0	-3.913386	

9549	Colonic Neoplasms	11-Sep	0	-3.876221	
9550	Colonic Neoplasms	ASB2	0	-3.873691	
9551	Colonic Neoplasms	PTPRH	0.000006	-3.847104	
9552	Colonic Neoplasms	ETHE1	0.000001	-3.835435	
9553	Colonic Neoplasms	PLG	0.000007	-3.798711	
9554	Colonic Neoplasms	KLF4	0	-3.786586	
9555	Colonic Neoplasms	CFH	0	-3.78256	
9556	Colonic Neoplasms	ASPA	0	-3.759596	
9557	Colonic Neoplasms	NMES1	0	-3.734037	
9558	Colonic Neoplasms	AOC3	0	-3.687249	
9559	Colonic Neoplasms	MYOT	0	-3.685809	
9560	Colonic Neoplasms	COX7A1	0	-3.641942	
9561	Colonic Neoplasms	P2RY14	0.000446	-3.628568	
9562	Colonic Neoplasms	SULT1A2	0	-3.60841	
9563	Colonic Neoplasms	DSC2	0.000004	-3.593929	
9564	Colonic Neoplasms	LOXL2	0	-3.584082	
9565	Colonic Neoplasms	UGT1A8	0.000013	-3.581855	
9566	Colonic Neoplasms	SOSTDC1	0.000002	-3.574382	
9567	Colonic Neoplasms	AKAP12	0.000109	-3.559876	
9568	Colonic Neoplasms	SELPLG	0	-3.558714	
9569	Colonic Neoplasms	C2orf40	0	-3.530774	
9570	Colonic Neoplasms	FEV	0.000012	-3.526807	
9571	Colonic Neoplasms	HSD17B2	0.00001	-3.522316	
9572	Colonic Neoplasms	MT1F	0.000013	-3.521489	
9573	Colonic Neoplasms	NR3C2	0	-3.51958	
9574	Colonic Neoplasms	RHBDL2	0	-3.502465	
9575	Colonic Neoplasms	MT1H	0.000111	-3.498151	
9576	Colonic Neoplasms	C17orf91	0	-3.497267	
9577	Colonic Neoplasms	GC	0.000002	-3.48362	
9578	Colonic Neoplasms	ABHD3	0.000008	-3.47626	
9579	Colonic Neoplasms	IL6R	0	-3.472127	
9580	Colonic Neoplasms	MTSS1	0.000145	-3.463537	
9581	Colonic Neoplasms	C1orf115	0	-3.453543	
9582	Colonic Neoplasms	SLAMF8	0	-3.437739	
9583	Colonic Neoplasms	EPHA10	0.000008	-3.435053	
9584	Colonic Neoplasms	BASP1	0	-3.435026	
9585	Colonic Neoplasms	GPA33	0.000001	-3.429682	
9586	Colonic Neoplasms	KRT19	0.000002	-3.421713	
9587	Colonic Neoplasms	APOC1	0	-3.418014	
9588	Colonic Neoplasms	MATN1	0	-3.416291	
9589	Colonic Neoplasms	SEPP1	0.000081	-3.413388	
9590	Colonic Neoplasms	APOA2	0.000002	-3.396929	
9591	Colonic Neoplasms	CRISPLD2	0	-3.394717	
9592	Colonic Neoplasms	NR5A2	0.000094	-3.394343	
9593	Colonic Neoplasms	EYA2	0	-3.391627	
9594	Colonic Neoplasms	TGM2	0	-3.386721	
9595	Colonic Neoplasms	COL6A2	0	-3.38534	
9596	Colonic Neoplasms	ZBTB16	0	-3.381649	
9597	Colonic Neoplasms	ADAMTS1	0	-3.379906	
9598	Colonic Neoplasms	STYK1	0	-3.378539	

9599	Colonic Neoplasms	CD14	0	-3.370307	
9600	Colonic Neoplasms	CBX7	0	-3.346332	
9601	Colonic Neoplasms	DCN	0.000641	-3.343137	
9602	Colonic Neoplasms	GSN	0	-3.342984	
9603	Colonic Neoplasms	SRI	0.000009	-3.33486	
9604	Colonic Neoplasms	SPON2	0	-3.332667	
9605	Colonic Neoplasms	FXYD1	0	-3.331368	
9606	Colonic Neoplasms	PLEKHO1	0	-3.328679	
9607	Colonic Neoplasms	CHL1	0.000158	-3.319252	
9608	Colonic Neoplasms	DKFZP564O08	0.000001	-3.288601	
9609	Colonic Neoplasms	IL11RA	0	-3.283501	
9610	Colonic Neoplasms	XLKD1	0	-3.279393	
9611	Colonic Neoplasms	CTSG	0	-3.250081	
9612	Colonic Neoplasms	FAM107A	0	-3.249412	
9613	Colonic Neoplasms	PDGFRB	0	-3.236921	
9614	Colonic Neoplasms	LRRN2	0	-3.229834	
9615	Colonic Neoplasms	TP53INP2	0	-3.225235	
9616	Colonic Neoplasms	HBB	0	-3.217454	
9617	Colonic Neoplasms	TXNIP	0	-3.20474	
9618	Colonic Neoplasms	FOXF1	0.000296	-3.198543	
9619	Colonic Neoplasms	FPRL1	0.000011	-3.169335	
9620	Colonic Neoplasms	CYP3A4	0.000003	-3.150967	
9621	Colonic Neoplasms	TMEM220	0	-3.143404	
9622	Colonic Neoplasms	SIDT1	0	-3.139651	
9623	Colonic Neoplasms	SLC9A9	0	-3.13727	
9624	Colonic Neoplasms	SCNN1A	0.000005	-3.135971	
9625	Colonic Neoplasms	PRPH	0	-3.134704	
9626	Colonic Neoplasms	MRVI1	0	-3.128852	
9627	Colonic Neoplasms	NBLA00301	0	-3.122942	
9628	Colonic Neoplasms	TP53I5	0.000002	-3.120461	
9629	Colonic Neoplasms	PXDN	0	-3.110963	
9630	Colonic Neoplasms	PALLD	0	-3.110605	
9631	Colonic Neoplasms	FLJ20152	0.000005	-3.093667	
9632	Colonic Neoplasms	TST	0.000001	-3.086522	
9633	Colonic Neoplasms	IL1R2	0.000004	-3.085178	
9634	Colonic Neoplasms	VSIG4	0	-3.085124	
9635	Colonic Neoplasms	C5orf4	0	-3.081489	
9636	Colonic Neoplasms	HMGCS2	0.000001	-3.080903	
9637	Colonic Neoplasms	ITGAL	0	-3.080831	
9638	Colonic Neoplasms	EPS15	0.000149	-3.080798	
9639	Colonic Neoplasms	RCAN2	0	-3.079993	
9640	Colonic Neoplasms	C6orf29	0.000003	-3.059207	
9641	Colonic Neoplasms	PIGR	0.000268	-3.039826	
9642	Colonic Neoplasms	RGS9	0	-3.036885	
9643	Colonic Neoplasms	FABP1	0.000006	-3.032035	
9644	Colonic Neoplasms	HIG1	0.000002	-3.027138	
9645	Colonic Neoplasms	MT1E	0	-3.02559	
9646	Colonic Neoplasms	BCLP	0.000001	-3.020425	
9647	Colonic Neoplasms	C1QB	0	-3.019289	
9648	Colonic Neoplasms	ZNF575	0	-3.018086	

9649	Colonic Neoplasms	ACACB	0.000014	-3.000493	
9650	Colonic Neoplasms	CYP2C8	0.000023	-2.984006	
9651	Colonic Neoplasms	DUSP26	0	-2.971737	
9652	Colonic Neoplasms	PAH	0.000012	-2.971188	
9653	Colonic Neoplasms	MATN2	0	-2.966749	
9654	Colonic Neoplasms	MYO1A	0.000007	-2.962363	
9655	Colonic Neoplasms	FAM3D	0.000002	-2.95883	
9656	Colonic Neoplasms	TM4SF2	0.000002	-2.95869	
9657	Colonic Neoplasms	TUBB2B	0.000163	-2.956319	
9658	Colonic Neoplasms	SMPD3	0.000001	-2.930047	
9659	Colonic Neoplasms	PECAM1	0	-2.921229	
9660	Colonic Neoplasms	CALCRL	0	-2.917643	
9661	Colonic Neoplasms	MGP	0	-2.910034	
9662	Colonic Neoplasms	DHRS11	0.000418	-2.904213	
9663	Colonic Neoplasms	KRT14	0.000004	-2.898214	
9664	Colonic Neoplasms	AHCYL2	0.000358	-2.889336	
9665	Colonic Neoplasms	PCDH7	0	-2.885336	
9666	Colonic Neoplasms	PPAP2A	0	-2.884461	
9667	Colonic Neoplasms	VILL	0.000003	-2.882666	
9668	Colonic Neoplasms	FMO3	0.000103	-2.866698	
9669	Colonic Neoplasms	MT1G	0.000021	-2.861547	
9670	Colonic Neoplasms	MUSTN1	0	-2.844608	
9671	Colonic Neoplasms	ST6GALNAC6	0	-2.840149	
9672	Colonic Neoplasms	CYP4F12	0.000003	-2.837124	
9673	Colonic Neoplasms	SSPN	0	-2.832805	
9674	Colonic Neoplasms	CLDN7	0.000004	-2.827828	
9675	Colonic Neoplasms	TMEM45B	0.000002	-2.820958	
9676	Colonic Neoplasms	NOTCH3	0	-2.820302	
9677	Colonic Neoplasms	NEDD4L	0.00001	-2.819899	
9678	Colonic Neoplasms	MTM1	0.000012	-2.817681	
9679	Colonic Neoplasms	SEMA4G	0.000002	-2.809155	
9680	Colonic Neoplasms	CHST5	0.000004	-2.795886	
9681	Colonic Neoplasms	PPARGC1A	0.000012	-2.794212	
9682	Colonic Neoplasms	RGS19IP1	0.00001	-2.784519	
9683	Colonic Neoplasms	OPTN	0	-2.78375	
9684	Colonic Neoplasms	FLJ11017	0.000002	-2.782371	
9685	Colonic Neoplasms	LGALS1	0	-2.775313	
9686	Colonic Neoplasms	CTSL1	0	-2.769246	
9687	Colonic Neoplasms	LRRC1	0.000013	-2.764394	
9688	Colonic Neoplasms	LOC440838	0.000153	-2.754453	
9689	Colonic Neoplasms	TUB	0.000002	-2.748186	
9690	Colonic Neoplasms	KIAA0574	0	-2.743239	
9691	Colonic Neoplasms	PDE5A	0.000791	-2.738766	
9692	Colonic Neoplasms	GPC6	0	-2.734977	
9693	Colonic Neoplasms	TIMP2	0	-2.734756	
9694	Colonic Neoplasms	CCL14	0.00017	-2.725208	
9695	Colonic Neoplasms	MGC4171	0.00001	-2.724735	
9696	Colonic Neoplasms	HIGD2A	0.000015	-2.723354	
9697	Colonic Neoplasms	GOLPH2	0.000005	-2.702898	
9698	Colonic Neoplasms	MYL6	0	-2.69657	

9699	Colonic Neoplasms	SATB2	0.000007	-2.679973	
9700	Colonic Neoplasms	CGN	0.000009	-2.669418	
9701	Colonic Neoplasms	APOE	0	-2.667596	
9702	Colonic Neoplasms	ACADS	0	-2.654603	
9703	Colonic Neoplasms	CD163	0	-2.639177	
9704	Colonic Neoplasms	MT1X	0.000586	-2.638727	
9705	Colonic Neoplasms	ZZEF1	0	-2.63172	
9706	Colonic Neoplasms	HSD17B6	0.000957	-2.620009	
9707	Colonic Neoplasms	KIAA0513	0	-2.615759	
9708	Colonic Neoplasms	PXMP2	0	-2.613262	
9709	Colonic Neoplasms	PIP5K1B	0.000006	-2.603886	
9710	Colonic Neoplasms	FLNC	0.000273	-2.597163	
9711	Colonic Neoplasms	CLMN	0.000006	-2.591893	
9712	Colonic Neoplasms	STAP2	0	-2.586791	
9713	Colonic Neoplasms	FMO4	0.000009	-2.582345	
9714	Colonic Neoplasms	TCF21	0	-2.579042	
9715	Colonic Neoplasms	MSN	0	-2.567381	
9716	Colonic Neoplasms	CKMT1	0.000013	-2.56467	
9717	Colonic Neoplasms	FLJ30901	0	-2.56354	
9718	Colonic Neoplasms	UGP2	0	-2.548487	
9719	Colonic Neoplasms	STOM	0	-2.543023	
9720	Colonic Neoplasms	NANS	0.000001	-2.539289	
9721	Colonic Neoplasms	C9orf19	0	-2.538741	
9722	Colonic Neoplasms	MXRA5	0	-2.538398	
9723	Colonic Neoplasms	SERPINA3	0	-2.531452	
9724	Colonic Neoplasms	CNGA3	0	-2.530801	
9725	Colonic Neoplasms	TDE2L	0.000006	-2.522827	
9726	Colonic Neoplasms	TEF	0	-2.522685	
9727	Colonic Neoplasms	CD74	0	-2.520342	
9728	Colonic Neoplasms	LOC201895	0.000001	-2.520197	
9729	Colonic Neoplasms	RetSat	0.000009	-2.51694	
9730	Colonic Neoplasms	THBD	0	-2.516051	
9731	Colonic Neoplasms	CD3D	0	-2.513778	
9732	Colonic Neoplasms	HLA-DMB	0	-2.513279	
9733	Colonic Neoplasms	GNAI1	0.000428	-2.500595	
9734	Colonic Neoplasms	AP1S2	0	-2.500455	
9735	Colonic Neoplasms	ADH6	0	-2.494864	
9736	Colonic Neoplasms	FLJ20273	0.000008	-2.483385	
9737	Colonic Neoplasms	SERPINA6	0.000004	-2.474711	
9738	Colonic Neoplasms	PLIN	0	-2.469331	
9739	Colonic Neoplasms	FCER1G	0	-2.459957	
9740	Colonic Neoplasms	GNA11	0	-2.459933	
9741	Colonic Neoplasms	FBLN5	0.000608	-2.450308	
9742	Colonic Neoplasms	ANK3	0	-2.447477	
9743	Colonic Neoplasms	GFRA2	0	-2.441722	
9744	Colonic Neoplasms	FLJ34515	0	-2.434938	
9745	Colonic Neoplasms	KLKB1	0.000008	-2.428999	
9746	Colonic Neoplasms	ANXA11	0.000009	-2.425022	
9747	Colonic Neoplasms	FARP1	0	-2.423072	
9748	Colonic Neoplasms	RDH5	0	-2.421524	

9749	Colonic Neoplasms	PAPSS2	0.000012	-2.421199	
9750	Colonic Neoplasms	LDB2	0	-2.411734	
9751	Colonic Neoplasms	LTBP4	0	-2.401606	
9752	Colonic Neoplasms	FOS	0	-2.399607	
9753	Colonic Neoplasms	TGM4	0.000001	-2.393012	
9754	Colonic Neoplasms	RIOK3	0.000007	-2.387403	
9755	Colonic Neoplasms	PLAT	0	-2.385488	
9756	Colonic Neoplasms	KLF6	0	-2.383907	
9757	Colonic Neoplasms	HOM-TES-103	0	-2.379865	
9758	Colonic Neoplasms	PCSK6	0.000006	-2.366172	
9759	Colonic Neoplasms	PBLD	0.000056	-2.361208	
9760	Colonic Neoplasms	PLCE1	0	-2.360601	
9761	Colonic Neoplasms	TNFSF10	0	-2.359087	
9762	Colonic Neoplasms	NCF1	0	-2.355677	
9763	Colonic Neoplasms	DST	0	-2.355175	
9764	Colonic Neoplasms	FOXN3	0	-2.338288	
9765	Colonic Neoplasms	PPP1R12C	0	-2.334646	
9766	Colonic Neoplasms	TRPM4	0.000006	-2.333557	
9767	Colonic Neoplasms	ARRDC4	0	-2.325857	
9768	Colonic Neoplasms	FGFR2	0	-2.321512	
9769	Colonic Neoplasms	MMP9	0	-2.315868	
9770	Colonic Neoplasms	EPHX2	0	-2.313872	
9771	Colonic Neoplasms	SYTL2	0.000012	-2.312394	
9772	Colonic Neoplasms	RILP	0	-2.310785	
9773	Colonic Neoplasms	ABCA6	0	-2.307646	
9774	Colonic Neoplasms	SPI1	0	-2.303508	
9775	Colonic Neoplasms	SH3BGRL2	0.000004	-2.297834	
9776	Colonic Neoplasms	IL6ST	0.00012	-2.293095	
9777	Colonic Neoplasms	C20orf39	0	-2.290544	
9778	Colonic Neoplasms	PLCD1	0	-2.282423	
9779	Colonic Neoplasms	SLC25A23	0	-2.28045	
9780	Colonic Neoplasms	FLJ14981	0.000001	-2.278628	
9781	Colonic Neoplasms	RBMS1	0	-2.274364	
9782	Colonic Neoplasms	AES	0	-2.268848	
9783	Colonic Neoplasms	CXorf9	0	-2.259197	
9784	Colonic Neoplasms	MAOA	0	-2.259184	
9785	Colonic Neoplasms	EIF4E3	0	-2.254867	
9786	Colonic Neoplasms	LRRC32	0	-2.252156	
9787	Colonic Neoplasms	RHOB	0.000018	-2.246938	
9788	Colonic Neoplasms	MT2A	0.000103	-2.240731	
9789	Colonic Neoplasms	SELM	0	-2.237822	
9790	Colonic Neoplasms	CAS1	0	-2.229294	
9791	Colonic Neoplasms	PDE2A	0	-2.228758	
9792	Colonic Neoplasms	SLC1A7	0.000001	-2.224365	
9793	Colonic Neoplasms	IL1B	0	-2.19842	
9794	Colonic Neoplasms	SLC35A3	0.000006	-2.193479	
9795	Colonic Neoplasms	GALIG	0	-2.192145	
9796	Colonic Neoplasms	GNAQ	0.000004	-2.183832	
9797	Colonic Neoplasms	GSTM2	0.000115	-2.175818	
9798	Colonic Neoplasms	ORF1-FL49	0.000001	-2.166765	

9799	Colonic Neoplasms	GPD1L	0.000012	-2.159267	
9800	Colonic Neoplasms	PTK6	0.000009	-2.155801	
9801	Colonic Neoplasms	CASQ2	0.000094	-2.147198	
9802	Colonic Neoplasms	CAMK2N1	0.000957	-2.143111	
9803	Colonic Neoplasms	CDH19	0.000056	-2.136546	
9804	Colonic Neoplasms	ACP5	0	-2.136245	
9805	Colonic Neoplasms	OLFML2B	0	-2.133967	
9806	Colonic Neoplasms	HLA-DQB1	0	-2.132024	
9807	Colonic Neoplasms	ZNF219	0	-2.12931	
9808	Colonic Neoplasms	CALM1	0	-2.124523	
9809	Colonic Neoplasms	ADD1	0.000146	-2.122039	
9810	Colonic Neoplasms	GPR176	0	-2.118895	
9811	Colonic Neoplasms	PINK1	0	-2.117277	
9812	Colonic Neoplasms	BCAR3	0.000012	-2.114651	
9813	Colonic Neoplasms	LOC285535	0	-2.111181	
9814	Colonic Neoplasms	CHP	0.000003	-2.107398	
9815	Colonic Neoplasms	GBP2	0.000284	-2.102945	
9816	Colonic Neoplasms	KIAA1737	0	-2.102382	
9817	Colonic Neoplasms	ShrmL	0.00001	-2.101674	
9818	Colonic Neoplasms	FLJ12610	0.000009	-2.095074	
9819	Colonic Neoplasms	IGL@	0	-2.092871	
9820	Colonic Neoplasms	CRY2	0	-2.091582	
9821	Colonic Neoplasms	TOM1L2	0	-2.085025	
9822	Colonic Neoplasms	VAMP8	0.000007	-2.083103	
9823	Colonic Neoplasms	TMEM49	0	-2.07887	
9824	Colonic Neoplasms	UGDH	0.000167	-2.075216	
9825	Colonic Neoplasms	MYO1C	0	-2.074156	
9826	Colonic Neoplasms	NDRG2	0	-2.071264	
9827	Colonic Neoplasms	PLXNA2	0.000002	-2.068311	
9828	Colonic Neoplasms	TFCP2L1	0.000006	-2.064292	
9829	Colonic Neoplasms	TDRD10	0	-2.058953	
9830	Colonic Neoplasms	SQRDL	0.000007	-2.056851	
9831	Colonic Neoplasms	INPP5A	0	-2.056516	
9832	Colonic Neoplasms	GPM6B	0.00016	-2.054248	
9833	Colonic Neoplasms	BTK	0	-2.048221	
9834	Colonic Neoplasms	LARGE	0	-2.046539	
9835	Colonic Neoplasms	PPARGC1B	0	-2.043279	
9836	Colonic Neoplasms	FLJ41603	0	-2.04177	
9837	Colonic Neoplasms	S100A10	0.000004	-2.036907	
9838	Colonic Neoplasms	SYTL4	0.000002	-2.034218	
9839	Colonic Neoplasms	CLTB	0.000008	-2.034168	
9840	Colonic Neoplasms	MAGI2	0	-2.029715	
9841	Colonic Neoplasms	CPT2	0	-2.027885	
9842	Colonic Neoplasms	METTL7A	0.000214	-2.025191	
9843	Colonic Neoplasms	MXI1	0	-2.021021	
9844	Colonic Neoplasms	POLD4	0.000002	-2.014145	
9845	Colonic Neoplasms	LBA1	0	-2.012908	
9846	Colonic Neoplasms	S100B	0	-2.012075	
9847	Colonic Neoplasms	PCSK1N	0.000002	-2.008034	
9848	Colonic Neoplasms	LOC653483	0.00001	-2.006975	

9849	Colonic Neoplasms	COL6A1	0	-2.006013	
9850	Colonic Neoplasms	CTNND1	0.000001	-2.001972	
9851	Colonic Neoplasms	PRELP	0	-2.000704	
9852	Colonic Neoplasms	IL2RB	0	-2.00014	
9853	Colonic Neoplasms	IBSP	0	2.003274	
9854	Colonic Neoplasms	TG	0	2.006777	
9855	Colonic Neoplasms	WDR51B	0	2.007203	
9856	Colonic Neoplasms	CCT2	0.000355	2.00934	
9857	Colonic Neoplasms	C5orf46	0	2.015334	
9858	Colonic Neoplasms	LOC642869	0.000007	2.017381	
9859	Colonic Neoplasms	HDAC2	0.000892	2.017489	
9860	Colonic Neoplasms	CBX3	0.000003	2.02162	
9861	Colonic Neoplasms	G0S2	0.000527	2.038066	
9862	Colonic Neoplasms	NME1-NME2	0.000103	2.03843	
9863	Colonic Neoplasms	DKK4	0	2.040699	
9864	Colonic Neoplasms	C7orf36	0	2.064753	
9865	Colonic Neoplasms	BMS1	0.000648	2.07506	
9866	Colonic Neoplasms	GAPDH	0.000008	2.076062	
9867	Colonic Neoplasms	EIF2C2	0.000983	2.081042	
9868	Colonic Neoplasms	PPRC1	0.000565	2.084755	
9869	Colonic Neoplasms	COL1A2	0.000004	2.086781	
9870	Colonic Neoplasms	KIAA0907	0.000141	2.092142	
9871	Colonic Neoplasms	TUBA4A	0.000277	2.100791	
9872	Colonic Neoplasms	CLEC5A	0	2.101128	
9873	Colonic Neoplasms	FLJ10826	0.000009	2.102346	
9874	Colonic Neoplasms	GSTO1	0.000868	2.103585	
9875	Colonic Neoplasms	SFTA2	0	2.108096	
9876	Colonic Neoplasms	SNRPE	0.000957	2.108572	
9877	Colonic Neoplasms	ACAN	0	2.11174	
9878	Colonic Neoplasms	CTSK	0	2.137342	
9879	Colonic Neoplasms	EGFL6	0.000026	2.144819	
9880	Colonic Neoplasms	RPL28	0.000284	2.146094	
9881	Colonic Neoplasms	SOX4	0.00051	2.147721	
9882	Colonic Neoplasms	IFITM1	0.000001	2.159871	
9883	Colonic Neoplasms	IL24	0	2.168089	
9884	Colonic Neoplasms	TKT	0.000214	2.173743	
9885	Colonic Neoplasms	ZNF259	0.000263	2.187872	
9886	Colonic Neoplasms	LPGAT1	0.000214	2.192642	
9887	Colonic Neoplasms	CCND2	0.000586	2.19821	
9888	Colonic Neoplasms	LIPG	0	2.199623	
9889	Colonic Neoplasms	ALG5	0	2.216436	
9890	Colonic Neoplasms	SHMT2	0.000499	2.231696	
9891	Colonic Neoplasms	SULF1	0.000019	2.235576	
9892	Colonic Neoplasms	FEN1	0.000282	2.247685	
9893	Colonic Neoplasms	TRIM21	0.000005	2.260939	
9894	Colonic Neoplasms	CEACAM5	0.000104	2.273474	
9895	Colonic Neoplasms	IGHMBP2	0	2.281069	
9896	Colonic Neoplasms	DIAPH1	0.000277	2.289976	
9897	Colonic Neoplasms	NPAS2	0.000307	2.31264	
9898	Colonic Neoplasms	CMTM8	0	2.325083	

9899	Colonic Neoplasms	LOC100133056	0	2.328852	
9900	Colonic Neoplasms	SNRPF	0.000586	2.337388	
9901	Colonic Neoplasms	CCND1	0.000167	2.37227	
9902	Colonic Neoplasms	KIAA1257	0	2.392024	
9903	Colonic Neoplasms	GALNT6	0	2.39372	
9904	Colonic Neoplasms	PTTG1	0.000355	2.399344	
9905	Colonic Neoplasms	BARX1	0.000009	2.406034	
9906	Colonic Neoplasms	COL11A1	0	2.408898	
9907	Colonic Neoplasms	hCG_1981531	0	2.40923	
9908	Colonic Neoplasms	E2F3	0.000284	2.418337	
9909	Colonic Neoplasms	CCL11	0.000018	2.447059	
9910	Colonic Neoplasms	NOL8	0.000116	2.460398	
9911	Colonic Neoplasms	PDXK	0.000174	2.460748	
9912	Colonic Neoplasms	MARCKSL1	0.000038	2.462448	
9913	Colonic Neoplasms	PDPN	0	2.501963	
9914	Colonic Neoplasms	C12orf48	0.000192	2.508405	
9915	Colonic Neoplasms	PRSS22	0	2.510166	
9916	Colonic Neoplasms	PKM2	0.000527	2.525776	
9917	Colonic Neoplasms	GNL3	0.000862	2.527467	
9918	Colonic Neoplasms	SERPINE2	0.00017	2.527904	
9919	Colonic Neoplasms	TAF1D	0.00002	2.528423	
9920	Colonic Neoplasms	IARS	0.000066	2.534859	
9921	Colonic Neoplasms	GPR56	0	2.556583	
9922	Colonic Neoplasms	OLA1	0.000115	2.567925	
9923	Colonic Neoplasms	KLK7	0	2.5896	
9924	Colonic Neoplasms	SLC7A5	0.000115	2.609348	
9925	Colonic Neoplasms	C2orf70	0	2.620584	
9926	Colonic Neoplasms	MGC4308	0.000012	2.624252	
9927	Colonic Neoplasms	SLC6A20	0	2.639076	
9928	Colonic Neoplasms	SRPX2	0	2.686563	
9929	Colonic Neoplasms	ZNF593	0.000689	2.699951	
9930	Colonic Neoplasms	METTL13	0.000434	2.712642	
9931	Colonic Neoplasms	RAD54B	0.000645	2.728082	
9932	Colonic Neoplasms	RUVBL2	0.000983	2.736516	
9933	Colonic Neoplasms	SULT1C2	0.000809	2.7538	
9934	Colonic Neoplasms	GTF2IRD1	0.000086	2.765354	
9935	Colonic Neoplasms	FUT1	0	2.815054	
9936	Colonic Neoplasms	RAD23B	0.00064	2.859517	
9937	Colonic Neoplasms	WISP1	0	2.875563	
9938	Colonic Neoplasms	CDK1	0.000371	2.916923	
9939	Colonic Neoplasms	TPD52L1	0.000259	2.917089	
9940	Colonic Neoplasms	CXCL6	0	2.921339	
9941	Colonic Neoplasms	CCT6A	0.000387	2.927714	
9942	Colonic Neoplasms	DTL	0.000149	2.931445	
9943	Colonic Neoplasms	NFE2L3	0.000163	2.939272	
9944	Colonic Neoplasms	ENC1	0	2.981497	
9945	Colonic Neoplasms	NOP16	0.000752	3.023389	
9946	Colonic Neoplasms	RNF183	0	3.027376	
9947	Colonic Neoplasms	MMP11	0	3.072586	
9948	Colonic Neoplasms	UTP14A	0.000259	3.072644	

9949	Colonic Neoplasms	PPAT	0.000009	3.082995	
9950	Colonic Neoplasms	CSE1L	0.000237	3.144743	
9951	Colonic Neoplasms	ANKLE2	0.000987	3.151218	
9952	Colonic Neoplasms	CCNT1	0	3.171019	
9953	Colonic Neoplasms	DKC1	0.000152	3.185826	
9954	Colonic Neoplasms	KIF26B	0	3.195147	
9955	Colonic Neoplasms	CCL20	0.000014	3.200013	
9956	Colonic Neoplasms	EEF1E1	0.000375	3.219963	
9957	Colonic Neoplasms	FAM150A	0	3.226216	
9958	Colonic Neoplasms	MKI67	0.000284	3.320027	
9959	Colonic Neoplasms	DSG3	0	3.327281	
9960	Colonic Neoplasms	GART	0.000355	3.327338	
9961	Colonic Neoplasms	KCNH8	0	3.352898	
9962	Colonic Neoplasms	CKS2	0.000011	3.385444	
9963	Colonic Neoplasms	IGFL2	0	3.42052	
9964	Colonic Neoplasms	KPNA2	0.000286	3.504182	
9965	Colonic Neoplasms	SOX9	0	3.525201	
9966	Colonic Neoplasms	PUS7	0.000326	3.547257	
9967	Colonic Neoplasms	DACH1	0	3.730219	
9968	Colonic Neoplasms	SORD	0.000036	3.795572	
9969	Colonic Neoplasms	HSPH1	0.000271	3.842682	
9970	Colonic Neoplasms	TRIP13	0.000158	3.871768	
9971	Colonic Neoplasms	PERP	0.00016	3.924924	
9972	Colonic Neoplasms	RRM2	0.000829	3.969704	
9973	Colonic Neoplasms	BUB1	0.000689	3.980634	
9974	Colonic Neoplasms	MACC1	0	4.066287	
9975	Colonic Neoplasms	DPEP1	0	4.128487	
9976	Colonic Neoplasms	ECT2	0.000004	4.15265	
9977	Colonic Neoplasms	NEK2	0.000166	4.180412	
9978	Colonic Neoplasms	HOMER1	0.000221	4.184058	
9979	Colonic Neoplasms	SLC12A2	0	4.198403	
9980	Colonic Neoplasms	KLK12	0	4.257902	
9981	Colonic Neoplasms	CENPA	0.000102	4.326599	
9982	Colonic Neoplasms	MAD2L1	0.000242	4.362649	
9983	Colonic Neoplasms	MET	0.000367	4.404876	
9984	Colonic Neoplasms	KRT6B	0	4.60545	
9985	Colonic Neoplasms	MMP10	0	4.667151	
9986	Colonic Neoplasms	PI3	0	4.702185	
9987	Colonic Neoplasms	SCD	0.00023	4.81501	
9988	Colonic Neoplasms	COL10A1	0	4.817021	
9989	Colonic Neoplasms	C10orf81	0	5.202957	
9990	Colonic Neoplasms	ASCL2	0	5.255321	
9991	Colonic Neoplasms	KLK10	0	5.296705	
9992	Colonic Neoplasms	CXCL5	0	5.376774	
9993	Colonic Neoplasms	CDH3	0.000006	5.459597	
9994	Colonic Neoplasms	VSNL1	0.000983	5.515369	
9995	Colonic Neoplasms	RNF43	0.000174	6.508152	
9996	Colonic Neoplasms	S100A2	0.000066	7.870126	
9997	Colonic Neoplasms	MMP7	0	8.884969	
9998	Colonic Neoplasms	TGFBI	0.000296	9.104031	

9999	Colonic Neoplasms	SERPINB5	0.000641	38.043312	
10000	Colorectal Cancer	MUC4	0.001993	-60.311531	
10001	Colorectal Cancer	MUC2	0.001405	-60.058351	
10002	Colorectal Cancer	FOXP2	0.002498	-27.659136	
10003	Colorectal Cancer	B3GNT6	0.000062	-27.448227	
10004	Colorectal Cancer	SCGB2A1	0.00296	-25.494518	
10005	Colorectal Cancer	MMP28	0.00029	-24.383882	
10006	Colorectal Cancer	KCNMA1	0.000529	-21.649194	
10007	Colorectal Cancer	LRMP	0.0036	-20.996778	
10008	Colorectal Cancer	CWH43	0.00122	-20.467763	
10009	Colorectal Cancer	SERTAD4	0.000207	-20.029386	
10010	Colorectal Cancer	SPDEF	0.003466	-18.98549	
10011	Colorectal Cancer	LOC100509635	0.000651	-16.450784	
10012	Colorectal Cancer	CHST5	0.003988	-16.204897	
10013	Colorectal Cancer	ANO5	0.000982	-15.875208	
10014	Colorectal Cancer	AKAP5	0.000488	-13.182194	
10015	Colorectal Cancer	PTGDR	0.001995	-11.897712	
10016	Colorectal Cancer	CD1D	0.000358	-11.888861	
10017	Colorectal Cancer	KLF9	0.003783	-11.537512	
10018	Colorectal Cancer	SPINK4	0	-10.604966	
10019	Colorectal Cancer	SCIN	0.000675	-10.535578	
10020	Colorectal Cancer	ZG16	0.000001	-10.523496	
10021	Colorectal Cancer	ACOT11	0.004247	-10.396793	
10022	Colorectal Cancer	METTL7A	0.000403	-10.26555	
10023	Colorectal Cancer	PPP1R9A	0.000004	-10.144455	
10024	Colorectal Cancer	CA4	0.000001	-10.09118	
10025	Colorectal Cancer	DQX1	0.004044	-10.025782	
10026	Colorectal Cancer	CREB3L1	0.003101	-9.594399	
10027	Colorectal Cancer	RAB27B	0.000026	-9.499798	
10028	Colorectal Cancer	CACNB2	0.000114	-9.483538	
10029	Colorectal Cancer	ASAP3	0.002559	-9.13715	
10030	Colorectal Cancer	ZBTB7C	0.00056	-9.045275	
10031	Colorectal Cancer	FOXA1	0.003339	-8.939768	
10032	Colorectal Cancer	ALDH1A1	0.003273	-8.905504	
10033	Colorectal Cancer	PADI2	0.00277	-8.762223	
10034	Colorectal Cancer	FABP1	0	-8.719388	
10035	Colorectal Cancer	SYT17	0.000365	-8.713691	
10036	Colorectal Cancer	KCNK10	0.000558	-8.692129	
10037	Colorectal Cancer	LOC100129069	0.001754	-8.641304	
10038	Colorectal Cancer	HMGCS2	0.000405	-8.54771	
10039	Colorectal Cancer	HGD	0.001124	-8.34414	
10040	Colorectal Cancer	FLJ32063	0.00281	-8.296896	
10041	Colorectal Cancer	CLCA4	0.000002	-8.038073	
10042	Colorectal Cancer	CEACAM7	0.000001	-7.835955	
10043	Colorectal Cancer	NR3C2	0.000114	-7.832787	
10044	Colorectal Cancer	ITM2A	0.004857	-7.7829	
10045	Colorectal Cancer	TPBG	0.005167	-7.771458	
10046	Colorectal Cancer	KLF4	0.003466	-7.611532	
10047	Colorectal Cancer	GLIS3	0.000782	-7.497044	
10048	Colorectal Cancer	ERN2	0.000972	-7.486607	

10049	Colorectal Cancer	MOGAT2	0.000358	-7.460037	
10050	Colorectal Cancer	B3GALT5	0.000358	-7.341713	
10051	Colorectal Cancer	OR51I1	0.004706	-7.291565	
10052	Colorectal Cancer	EFNA5	0.000405	-7.221174	
10053	Colorectal Cancer	DOCK8	0.004993	-7.212796	
10054	Colorectal Cancer	LAMA1	0.002619	-7.153433	
10055	Colorectal Cancer	C14orf50	0.000358	-7.032244	
10056	Colorectal Cancer	GRAMD3	0.000488	-6.964465	
10057	Colorectal Cancer	CCNJL	0.000163	-6.772087	
10058	Colorectal Cancer	C11orf93	0.001565	-6.711384	
10059	Colorectal Cancer	SPATA18	0.003433	-6.664482	
10060	Colorectal Cancer	FARSB	0.00277	-6.56314	
10061	Colorectal Cancer	DPF3	0.000202	-6.507991	
10062	Colorectal Cancer	PLCE1	0.00071	-6.424479	
10063	Colorectal Cancer	MDFIC	0.000923	-6.391231	
10064	Colorectal Cancer	PLCL2	0.003101	-6.299826	
10065	Colorectal Cancer	CHGA	0.000004	-6.084005	
10066	Colorectal Cancer	CBFA2T3	0.000405	-6.06337	
10067	Colorectal Cancer	HPGDS	0.00138	-5.994216	
10068	Colorectal Cancer	PSMG4	0.00332	-5.931413	
10069	Colorectal Cancer	EDIL3	0.001296	-5.905179	
10070	Colorectal Cancer	SLC9A2	0.004579	-5.796233	
10071	Colorectal Cancer	FRMD3	0.004638	-5.691552	
10072	Colorectal Cancer	SLC16A7	0.003386	-5.613971	
10073	Colorectal Cancer	ACTG2	0	-5.576115	
10074	Colorectal Cancer	AQP8	0.000157	-5.570023	
10075	Colorectal Cancer	CHP2	0.004857	-5.506835	
10076	Colorectal Cancer	MS4A12	0.000032	-5.360331	
10077	Colorectal Cancer	PDE4D	0.000331	-5.300381	
10078	Colorectal Cancer	LOC643072	0.003988	-5.284507	
10079	Colorectal Cancer	PRKACB	0.000052	-5.284016	
10080	Colorectal Cancer	RNF125	0.003737	-5.236229	
10081	Colorectal Cancer	SIDT1	0.000358	-5.216442	
10082	Colorectal Cancer	MATN2	0.000471	-5.191578	
10083	Colorectal Cancer	MCTP2	0.000114	-5.145834	
10084	Colorectal Cancer	VSTM2A	0.002883	-5.04434	
10085	Colorectal Cancer	NR5A2	0.00211	-5.041885	
10086	Colorectal Cancer	TNFRSF11A	0.000529	-4.980546	
10087	Colorectal Cancer	EFHC2	0.000215	-4.968653	
10088	Colorectal Cancer	VILL	0.000651	-4.956432	
10089	Colorectal Cancer	GOLM1	0.000215	-4.926988	
10090	Colorectal Cancer	DENND1B	0.002619	-4.908923	
10091	Colorectal Cancer	C2orf88	0.001516	-4.861751	
10092	Colorectal Cancer	KIAA1731	0.004141	-4.835946	
10093	Colorectal Cancer	DEGS2	0.004217	-4.794887	
10094	Colorectal Cancer	KSR2	0.004857	-4.793552	
10095	Colorectal Cancer	TRPM6	0.000711	-4.765605	
10096	Colorectal Cancer	TEX9	0.003737	-4.753316	
10097	Colorectal Cancer	TEP1	0.00237	-4.711473	
10098	Colorectal Cancer	KLK1	0.00332	-4.696096	

10099	Colorectal Cancer	LOC100506589	0.001854	-4.673174	
10100	Colorectal Cancer	CCL28	0.000605	-4.654903	
10101	Colorectal Cancer	C16orf53	0.003149	-4.649649	
10102	Colorectal Cancer	BMP2	0.004341	-4.567065	
10103	Colorectal Cancer	GNE	0.004141	-4.563829	
10104	Colorectal Cancer	TOX	0.001493	-4.475666	
10105	Colorectal Cancer	PARVA	0.002922	-4.438447	
10106	Colorectal Cancer	SSTR1	0.003988	-4.42735	
10107	Colorectal Cancer	SIAE	0.001854	-4.411799	
10108	Colorectal Cancer	RAB27A	0.00002	-4.39503	
10109	Colorectal Cancer	ARMCX4	0.003737	-4.391212	
10110	Colorectal Cancer	LOC727916	0.005164	-4.380807	
10111	Colorectal Cancer	FAS	0.002883	-4.344731	
10112	Colorectal Cancer	LXN	0.001245	-4.295182	
10113	Colorectal Cancer	LOC100128288	0.004857	-4.240265	
10114	Colorectal Cancer	FYB	0.003433	-4.225236	
10115	Colorectal Cancer	DDX26B	0.004644	-4.223636	
10116	Colorectal Cancer	ANG	0.000488	-4.192727	
10117	Colorectal Cancer	LOC100216546	0.000776	-4.176673	
10118	Colorectal Cancer	EBF1	0.004544	-4.164009	
10119	Colorectal Cancer	TEX11	0.001604	-4.140414	
10120	Colorectal Cancer	PLAC8	0.000009	-4.134202	
10121	Colorectal Cancer	STYK1	0.000446	-4.069078	
10122	Colorectal Cancer	TCEA3	0.003014	-4.058089	
10123	Colorectal Cancer	CXCL14	0.000133	-4.05581	
10124	Colorectal Cancer	LOC728903	0.001325	-4.04142	
10125	Colorectal Cancer	PAFAH2	0.001196	-4.022292	
10126	Colorectal Cancer	KCTD1	0.00296	-4.01414	
10127	Colorectal Cancer	GPR27	0.004668	-4.013727	
10128	Colorectal Cancer	QSOX1	0.003047	-3.986606	
10129	Colorectal Cancer	UBQLN4	0.004931	-3.93261	
10130	Colorectal Cancer	EHF	0.001404	-3.901229	
10131	Colorectal Cancer	CARD16	0.003031	-3.878799	
10132	Colorectal Cancer	CAPN13	0.002883	-3.8648	
10133	Colorectal Cancer	TTLL5	0.004629	-3.848962	
10134	Colorectal Cancer	DST	0	-3.84509	
10135	Colorectal Cancer	C4orf34	0.000493	-3.841658	
10136	Colorectal Cancer	BCAR3	0.001754	-3.82109	
10137	Colorectal Cancer	C1orf21	0.000694	-3.812153	
10138	Colorectal Cancer	SYTL2	0.001871	-3.807608	
10139	Colorectal Cancer	ITLN1	0	-3.790869	
10140	Colorectal Cancer	ACVRL1	0.002015	-3.786947	
10141	Colorectal Cancer	PHF7	0.004347	-3.782336	
10142	Colorectal Cancer	CCDC68	0.002058	-3.775933	
10143	Colorectal Cancer	ADH6	0.002922	-3.740311	
10144	Colorectal Cancer	HNRNPD	0.004611	-3.731405	
10145	Colorectal Cancer	RAB26	0.00113	-3.719949	
10146	Colorectal Cancer	SPECC1	0.004848	-3.698745	
10147	Colorectal Cancer	SFRS18	0.001805	-3.6981	
10148	Colorectal Cancer	ABCA5	0.004638	-3.659734	

10149	Colorectal Cancer	CLMN	0.000358	-3.643807	
10150	Colorectal Cancer	FAM149A	0.004517	-3.641557	
10151	Colorectal Cancer	MAOA	0.00219	-3.624132	
10152	Colorectal Cancer	C14orf106	0.004644	-3.614466	
10153	Colorectal Cancer	FBLN1	0.000002	-3.606652	
10154	Colorectal Cancer	VPS26A	0.002559	-3.599722	
10155	Colorectal Cancer	TRAPPC10	0.00293	-3.583657	
10156	Colorectal Cancer	TPSG1	0	-3.549494	
10157	Colorectal Cancer	DMXL2	0.002619	-3.539085	
10158	Colorectal Cancer	ARMC8	0.00345	-3.52509	
10159	Colorectal Cancer	ADAMDEC1	0.000004	-3.51873	
10160	Colorectal Cancer	FLJ27352	0.001463	-3.511211	
10161	Colorectal Cancer	REG4	0.000059	-3.503367	
10162	Colorectal Cancer	PTPN21	0.001057	-3.479038	
10163	Colorectal Cancer	SAMD13	0.000333	-3.468445	
10164	Colorectal Cancer	FRMD4B	0.0037	-3.467352	
10165	Colorectal Cancer	NCRNA00081	0.000927	-3.424376	
10166	Colorectal Cancer	PYY	0.000183	-3.420898	
10167	Colorectal Cancer	AKAP10	0.003047	-3.41148	
10168	Colorectal Cancer	LIMA1	0.003047	-3.393403	
10169	Colorectal Cancer	EHHADH	0.004243	-3.390529	
10170	Colorectal Cancer	ENTPD5	0.004217	-3.376918	
10171	Colorectal Cancer	FLJ23867	0.000215	-3.375153	
10172	Colorectal Cancer	PGAP1	0.001395	-3.368329	
10173	Colorectal Cancer	RAP1GAP	0.004544	-3.366309	
10174	Colorectal Cancer	SEMA6A	0.003988	-3.359076	
10175	Colorectal Cancer	KIAA1324	0.00219	-3.346923	
10176	Colorectal Cancer	MGC21881	0.004483	-3.345753	
10177	Colorectal Cancer	MMP3	0.000106	-3.344679	
10178	Colorectal Cancer	MGLL	0	-3.330854	
10179	Colorectal Cancer	SBF2	0.000987	-3.325026	
10180	Colorectal Cancer	ENOSF1	0.000324	-3.307115	
10181	Colorectal Cancer	ATP8B1	0.001005	-3.299743	
10182	Colorectal Cancer	NAB1	0.001404	-3.298022	
10183	Colorectal Cancer	CAPN5	0.00049	-3.289094	
10184	Colorectal Cancer	SLC44A1	0.000488	-3.283266	
10185	Colorectal Cancer	PRORSD1P	0.000953	-3.274898	
10186	Colorectal Cancer	SLC22A23	0.000488	-3.272734	
10187	Colorectal Cancer	LOC100289632	0.002906	-3.25408	
10188	Colorectal Cancer	CHD9	0.000795	-3.253819	
10189	Colorectal Cancer	DAPP1	0.004176	-3.24881	
10190	Colorectal Cancer	SLC35A1	0.000136	-3.233704	
10191	Colorectal Cancer	SIPA1L2	0.004857	-3.210274	
10192	Colorectal Cancer	KIAA1370	0.002922	-3.207262	
10193	Colorectal Cancer	GNAQ	0.001906	-3.202496	
10194	Colorectal Cancer	PTPRH	0.004811	-3.182022	
10195	Colorectal Cancer	PLEKHH1	0.001405	-3.180597	
10196	Colorectal Cancer	KIF13A	0.000802	-3.177314	
10197	Colorectal Cancer	ALDH6A1	0.002957	-3.169574	
10198	Colorectal Cancer	PPP1R12B	0.003424	-3.145353	

10199	Colorectal Cancer	LEAP2	0.000488	-3.125762	
10200	Colorectal Cancer	AHNAK	0.002883	-3.114242	
10201	Colorectal Cancer	MEIS3P1	0.002861	-3.102961	
10202	Colorectal Cancer	TET2	0.00293	-3.096987	
10203	Colorectal Cancer	SPARCL1	0.000004	-3.096174	
10204	Colorectal Cancer	CMAS	0.000711	-3.085735	
10205	Colorectal Cancer	TFF3	0.004638	-3.069182	
10206	Colorectal Cancer	SELENBP1	0.000071	-3.066158	
10207	Colorectal Cancer	C2orf72	0.002451	-3.066078	
10208	Colorectal Cancer	C4orf19	0.001296	-3.063415	
10209	Colorectal Cancer	DARC	0.000001	-3.028116	
10210	Colorectal Cancer	VIP	0.000001	-3.019982	
10211	Colorectal Cancer	SR140	0.003805	-3.019369	
10212	Colorectal Cancer	SCAPER	0.004247	-3.017734	
10213	Colorectal Cancer	AKR1B10	0.000698	-3.002267	
10214	Colorectal Cancer	LOC400573	0.001278	-2.984874	
10215	Colorectal Cancer	ACACB	0.004517	-2.983934	
10216	Colorectal Cancer	KIAA1468	0.000776	-2.972723	
10217	Colorectal Cancer	DTWD1	0.004638	-2.969544	
10218	Colorectal Cancer	FOXP1	0.000365	-2.942729	
10219	Colorectal Cancer	MON2	0.000711	-2.938999	
10220	Colorectal Cancer	FAM48A	0.002854	-2.936586	
10221	Colorectal Cancer	VMD2L1	0.000134	-2.885943	
10222	Colorectal Cancer	CEP70	0.001068	-2.88124	
10223	Colorectal Cancer	CWF19L2	0.003589	-2.85482	
10224	Colorectal Cancer	FGD4	0.000358	-2.846781	
10225	Colorectal Cancer	C9orf125	0.000623	-2.833038	
10226	Colorectal Cancer	RBM25	0.000802	-2.822374	
10227	Colorectal Cancer	HSBP1L1	0.000488	-2.82191	
10228	Colorectal Cancer	ABCC3	0.002833	-2.821428	
10229	Colorectal Cancer	PPP2R3A	0.003288	-2.821136	
10230	Colorectal Cancer	GPA33	0.000665	-2.816748	
10231	Colorectal Cancer	RNASE4	0.000488	-2.815054	
10232	Colorectal Cancer	PLCD1	0.004517	-2.809502	
10233	Colorectal Cancer	SULT1A3	0.003634	-2.807614	
10234	Colorectal Cancer	VWF	0.000708	-2.797215	
10235	Colorectal Cancer	SAHH3	0.000006	-2.796622	
10236	Colorectal Cancer	LYST	0.000207	-2.788893	
10237	Colorectal Cancer	LOC100128893	0.004141	-2.787412	
10238	Colorectal Cancer	TLE1	0.004773	-2.779947	
10239	Colorectal Cancer	CPEB3	0.005131	-2.761783	
10240	Colorectal Cancer	EXPH5	0.000446	-2.756191	
10241	Colorectal Cancer	LOC100506168	0.003101	-2.737855	
10242	Colorectal Cancer	TNFRSF10D	0.00281	-2.734711	
10243	Colorectal Cancer	HSD11B2	0.000411	-2.707869	
10244	Colorectal Cancer	SGSM3	0.002619	-2.702811	
10245	Colorectal Cancer	LOC100506493	0.005131	-2.692526	
10246	Colorectal Cancer	NHSL1	0.002223	-2.69232	
10247	Colorectal Cancer	PCLO	0.003424	-2.682969	
10248	Colorectal Cancer	LOC342918	0.031445	-2.676083	

10249	Colorectal Cancer	NEDD4L	0.00332	-2.675522	
10250	Colorectal Cancer	TRUB1	0.016286	-2.67491	
10251	Colorectal Cancer	TPT1	0.004857	-2.663384	
10252	Colorectal Cancer	GPATCH2	0.003649	-2.652061	
10253	Colorectal Cancer	SPON1	0.000432	-2.646048	
10254	Colorectal Cancer	CCL13	0.000261	-2.615183	
10255	Colorectal Cancer	C10orf54	0.003149	-2.612426	
10256	Colorectal Cancer	CPA3	0	-2.60624	
10257	Colorectal Cancer	NAPEPLD	0.001781	-2.604727	
10258	Colorectal Cancer	SMCHD1	0.003343	-2.585213	
10259	Colorectal Cancer	EPB41L4B	0.000062	-2.549991	
10260	Colorectal Cancer	ETFDH	0.003339	-2.525804	
10261	Colorectal Cancer	PIGN	0.001057	-2.524512	
10262	Colorectal Cancer	PALLD	0.000893	-2.519625	
10263	Colorectal Cancer	CCDC112	0.001371	-2.515317	
10264	Colorectal Cancer	ZSWIM6	0.003636	-2.505999	
10265	Colorectal Cancer	CASD1	0.004245	-2.501637	
10266	Colorectal Cancer	SDCBP2	0.000331	-2.495947	
10267	Colorectal Cancer	FUBP1	0.000607	-2.495646	
10268	Colorectal Cancer	RNASEL	0.004644	-2.494672	
10269	Colorectal Cancer	MYOF	0.003778	-2.488286	
10270	Colorectal Cancer	PLD1	0.0037	-2.482875	
10271	Colorectal Cancer	AMY2B	0.004544	-2.481498	
10272	Colorectal Cancer	FOXF2	0	-2.48074	
10273	Colorectal Cancer	TMEM30B	0.000555	-2.470234	
10274	Colorectal Cancer	SLCO2A1	0.000002	-2.469144	
10275	Colorectal Cancer	CAMK2D	0.000541	-2.468768	
10276	Colorectal Cancer	ACAD10	0.002237	-2.454179	
10277	Colorectal Cancer	AP4B1	0.000582	-2.447063	
10278	Colorectal Cancer	FOXF1	0	-2.435512	
10279	Colorectal Cancer	CD74	0.037564	-2.433393	
10280	Colorectal Cancer	MYO1C	0.003288	-2.416458	
10281	Colorectal Cancer	RBBP4	0.004235	-2.412335	
10282	Colorectal Cancer	GLTP	0.00293	-2.401717	
10283	Colorectal Cancer	CRYZL1	0.004152	-2.394317	
10284	Colorectal Cancer	SYTL4	0.001604	-2.391109	
10285	Colorectal Cancer	KIAA1109	0.003038	-2.378572	
10286	Colorectal Cancer	PGM5	0.000314	-2.364494	
10287	Colorectal Cancer	WDR7	0.005167	-2.346086	
10288	Colorectal Cancer	SCARA5	0.000025	-2.332101	
10289	Colorectal Cancer	PAPSS2	0.004629	-2.332038	
10290	Colorectal Cancer	FAM162A	0.00025	-2.33122	
10291	Colorectal Cancer	CCDC14	0.00051	-2.328058	
10292	Colorectal Cancer	HNF1B	0.003493	-2.321626	
10293	Colorectal Cancer	RABGAP1L	0.003737	-2.314043	
10294	Colorectal Cancer	C15orf48	0.000105	-2.264361	
10295	Colorectal Cancer	EFCAB4B	0.004437	-2.264241	
10296	Colorectal Cancer	TSPAN13	0.002922	-2.261416	
10297	Colorectal Cancer	AGXT2L2	0.00293	-2.258722	
10298	Colorectal Cancer	MAB21L2	0.000009	-2.254028	

10299	Colorectal Cancer	NKTR	0.002223	-2.246367	
10300	Colorectal Cancer	MS4A8B	0.000135	-2.245197	
10301	Colorectal Cancer	EYA3	0.046824	-2.232375	
10302	Colorectal Cancer	TMEM43	0.004638	-2.226105	
10303	Colorectal Cancer	RGMA	0.000001	-2.216106	
10304	Colorectal Cancer	FAM161B	0.00488	-2.207343	
10305	Colorectal Cancer	TMIGD	0.000335	-2.196593	
10306	Colorectal Cancer	C8orf83	0.001854	-2.182153	
10307	Colorectal Cancer	TCF21	0	-2.173875	
10308	Colorectal Cancer	DNAJC4	0.002861	-2.171379	
10309	Colorectal Cancer	ANKRD36	0.005131	-2.167662	
10310	Colorectal Cancer	CTSG	0.000001	-2.15913	
10311	Colorectal Cancer	TSPAN7	0.000705	-2.148268	
10312	Colorectal Cancer	TMEM54	0.000211	-2.141421	
10313	Colorectal Cancer	Q8NBX4	0.000102	-2.127648	
10314	Colorectal Cancer	SDCCAG1	0.00091	-2.126706	
10315	Colorectal Cancer	ZNF252	0.002619	-2.12491	
10316	Colorectal Cancer	FAM200A	0.003763	-2.122202	
10317	Colorectal Cancer	NSG1	0	-2.121418	
10318	Colorectal Cancer	RHAG	0.046824	-2.120604	
10319	Colorectal Cancer	PLAT	0.000028	-2.107739	
10320	Colorectal Cancer	UNC5B	0.003512	-2.103171	
10321	Colorectal Cancer	ACAA1	0.000802	-2.099543	
10322	Colorectal Cancer	EPCAM	0.005086	-2.095787	
10323	Colorectal Cancer	NEUROG3	0.000017	-2.095584	
10324	Colorectal Cancer	LOC284454	0.004125	-2.081457	
10325	Colorectal Cancer	ECHDC2	0.004558	-2.078043	
10326	Colorectal Cancer	ADSV	0.000576	-2.070961	
10327	Colorectal Cancer	CD79A	0.000427	-2.069078	
10328	Colorectal Cancer	SYNJ2BP	0.000369	-2.059174	
10329	Colorectal Cancer	TMSB10	0.000217	-2.0569	
10330	Colorectal Cancer	VPS13D	0.003805	-2.055066	
10331	Colorectal Cancer	PPP1R13B	0.004557	-2.039713	
10332	Colorectal Cancer	GRIN1	0.001189	-2.033231	
10333	Colorectal Cancer	MTHFD2L	0.004857	-2.02129	
10334	Colorectal Cancer	MALL	0.00058	-2.001755	
10335	Colorectal Cancer	CYP2R1	0.001404	-2.000093	
10336	Colorectal Cancer	CBX3	0.000002	2.001406	
10337	Colorectal Cancer	ZMYND19	0	2.00155	
10338	Colorectal Cancer	LONP1	0	2.008715	
10339	Colorectal Cancer	RGS12	0	2.009921	
10340	Colorectal Cancer	MTA2	0.000001	2.010076	
10341	Colorectal Cancer	AAA1	0	2.01351	
10342	Colorectal Cancer	KIF15	0	2.015372	
10343	Colorectal Cancer	GOLT1B	0	2.015554	
10344	Colorectal Cancer	RIPK2	0	2.016821	
10345	Colorectal Cancer	MTERFD1	0.000001	2.018794	
10346	Colorectal Cancer	KIF11	0.000001	2.025286	
10347	Colorectal Cancer	NUP107	0	2.028406	
10348	Colorectal Cancer	CCDC59	0	2.030633	

10349	Colorectal Cancer	PSD2	0	2.036907	
10350	Colorectal Cancer	TIMELESS	0	2.051896	
10351	Colorectal Cancer	PHF11	0.001695	2.062975	
10352	Colorectal Cancer	TMEM91	0	2.063926	
10353	Colorectal Cancer	OIP5	0.000001	2.065998	
10354	Colorectal Cancer	KDM2B	0.004141	2.06911	
10355	Colorectal Cancer	RUVBL1	0	2.069813	
10356	Colorectal Cancer	NUP205	0.000001	2.074642	
10357	Colorectal Cancer	STT3A	0	2.077468	
10358	Colorectal Cancer	ENO1	0.000002	2.078688	
10359	Colorectal Cancer	RUVBL2	0	2.080554	
10360	Colorectal Cancer	ADCK4	0	2.081367	
10361	Colorectal Cancer	QPCTL	0	2.085368	
10362	Colorectal Cancer	BPI	0	2.085919	
10363	Colorectal Cancer	RFC5	0.000001	2.096438	
10364	Colorectal Cancer	EPPK1	0.000001	2.101393	
10365	Colorectal Cancer	L3MBTL2	0	2.106203	
10366	Colorectal Cancer	C20orf132	0	2.106547	
10367	Colorectal Cancer	PSMA1	0.004439	2.107412	
10368	Colorectal Cancer	SLC25A32	0.000001	2.113102	
10369	Colorectal Cancer	GINS2	0.000001	2.11675	
10370	Colorectal Cancer	NUP85	0.000001	2.11777	
10371	Colorectal Cancer	C1orf107	0.002301	2.118563	
10372	Colorectal Cancer	RBM28	0	2.120363	
10373	Colorectal Cancer	COL18A1	0	2.120507	
10374	Colorectal Cancer	NCAPG2	0	2.125408	
10375	Colorectal Cancer	FCHSD1	0	2.126384	
10376	Colorectal Cancer	CAD	0.000001	2.127343	
10377	Colorectal Cancer	C22orf29	0	2.132896	
10378	Colorectal Cancer	GTF2IRD1	0	2.133547	
10379	Colorectal Cancer	FANCL	0	2.139324	
10380	Colorectal Cancer	GZMB	0	2.14732	
10381	Colorectal Cancer	KIF2C	0.000001	2.154208	
10382	Colorectal Cancer	OAS3	0.000001	2.155018	
10383	Colorectal Cancer	TIE1	0	2.16211	
10384	Colorectal Cancer	PORCN	0	2.162538	
10385	Colorectal Cancer	ANO3	0	2.166345	
10386	Colorectal Cancer	FANCG	0	2.166676	
10387	Colorectal Cancer	CCL4	0	2.174968	
10388	Colorectal Cancer	PDZD2	0	2.185247	
10389	Colorectal Cancer	PCBP3	0	2.186495	
10390	Colorectal Cancer	CPSF4	0.000711	2.187018	
10391	Colorectal Cancer	SLC38A3	0	2.187399	
10392	Colorectal Cancer	RAB15	0	2.188293	
10393	Colorectal Cancer	JAG2	0.000001	2.191192	
10394	Colorectal Cancer	WDR43	0.000001	2.194866	
10395	Colorectal Cancer	C10orf71	0	2.194946	
10396	Colorectal Cancer	CSNK2B	0.000285	2.197823	
10397	Colorectal Cancer	OLR1	0.000557	2.207088	
10398	Colorectal Cancer	GTPBP5	0	2.213754	

10399	Colorectal Cancer	ARIH1	0.000605	2.21698	
10400	Colorectal Cancer	HOXB6	0.022575	2.220702	
10401	Colorectal Cancer	CLCN7	0	2.234566	
10402	Colorectal Cancer	KIF19	0	2.251696	
10403	Colorectal Cancer	POLR1D	0.000001	2.252838	
10404	Colorectal Cancer	PTCD1	0	2.255095	
10405	Colorectal Cancer	KIAA1430	0.004953	2.262002	
10406	Colorectal Cancer	NUP37	0.000001	2.262069	
10407	Colorectal Cancer	FUT8	0	2.267452	
10408	Colorectal Cancer	PITX1	0.000001	2.26755	
10409	Colorectal Cancer	CHD7	0.000445	2.281194	
10410	Colorectal Cancer	CBX4	0	2.293435	
10411	Colorectal Cancer	KIF20A	0	2.295043	
10412	Colorectal Cancer	TMEM109	0	2.298206	
10413	Colorectal Cancer	SLC26A4	0	2.301524	
10414	Colorectal Cancer	MYO1H	0	2.304578	
10415	Colorectal Cancer	MICB	0	2.307009	
10416	Colorectal Cancer	BHLHE22	0	2.322857	
10417	Colorectal Cancer	HS2ST1	0.000002	2.328743	
10418	Colorectal Cancer	CECR2	0	2.332804	
10419	Colorectal Cancer	CBFB	0	2.334836	
10420	Colorectal Cancer	CDC42EP1	0.000001	2.339437	
10421	Colorectal Cancer	SORD	0.000001	2.341712	
10422	Colorectal Cancer	POLN	0	2.353199	
10423	Colorectal Cancer	USH2A	0	2.355116	
10424	Colorectal Cancer	NRK	0	2.357796	
10425	Colorectal Cancer	DOK1	0	2.368591	
10426	Colorectal Cancer	AMPD2	0	2.370905	
10427	Colorectal Cancer	COL24A1	0	2.375741	
10428	Colorectal Cancer	HEATR4	0	2.383003	
10429	Colorectal Cancer	MRPS17	0.000001	2.391333	
10430	Colorectal Cancer	KIF9	0	2.398162	
10431	Colorectal Cancer	LY6G6D	0	2.400382	
10432	Colorectal Cancer	NOP56	0.000001	2.40229	
10433	Colorectal Cancer	ZWILCH	0	2.403617	
10434	Colorectal Cancer	CDH26	0	2.404148	
10435	Colorectal Cancer	JAKMIP2	0	2.406255	
10436	Colorectal Cancer	CBS	0.000703	2.411396	
10437	Colorectal Cancer	AOC2	0	2.41181	
10438	Colorectal Cancer	ITGAM	0	2.411873	
10439	Colorectal Cancer	ILF3	0.00296	2.417933	
10440	Colorectal Cancer	RFX4	0	2.420886	
10441	Colorectal Cancer	ARNTL2	0	2.425129	
10442	Colorectal Cancer	ADFP	0.00024	2.429958	
10443	Colorectal Cancer	ANGPTL4	0.000173	2.440066	
10444	Colorectal Cancer	ADAMTSL2	0.000012	2.453786	
10445	Colorectal Cancer	PSAT1	0.000001	2.45864	
10446	Colorectal Cancer	NFE2L3	0	2.463088	
10447	Colorectal Cancer	CSE1L	0.000001	2.467005	
10448	Colorectal Cancer	MAK	0	2.472108	

10449	Colorectal Cancer	EYA1	0	2.472297	
10450	Colorectal Cancer	ANKFN1	0	2.472601	
10451	Colorectal Cancer	C12orf11	0	2.477935	
10452	Colorectal Cancer	KRT39	0	2.480666	
10453	Colorectal Cancer	CARD11	0	2.480952	
10454	Colorectal Cancer	PRDM8	0	2.481532	
10455	Colorectal Cancer	PKD1L3	0	2.481772	
10456	Colorectal Cancer	CA6	0	2.484544	
10457	Colorectal Cancer	VARS	0	2.490948	
10458	Colorectal Cancer	ICAM1	0	2.498583	
10459	Colorectal Cancer	IQCH	0	2.516142	
10460	Colorectal Cancer	CYP1B1	0.000314	2.525663	
10461	Colorectal Cancer	CCDC92	0.00358	2.52573	
10462	Colorectal Cancer	PLD6	0.000062	2.530931	
10463	Colorectal Cancer	NPHP1	0	2.545304	
10464	Colorectal Cancer	CEP250	0	2.550342	
10465	Colorectal Cancer	RAPGEF4	0	2.551132	
10466	Colorectal Cancer	BCL6B	0	2.554916	
10467	Colorectal Cancer	DCC	0	2.558436	
10468	Colorectal Cancer	NALCN	0	2.560201	
10469	Colorectal Cancer	MCM7	0	2.562626	
10470	Colorectal Cancer	PSMB8	0.004857	2.5672	
10471	Colorectal Cancer	UBE2L6	0.000001	2.570384	
10472	Colorectal Cancer	TTC25	0	2.571125	
10473	Colorectal Cancer	PSMG1	0.000001	2.572526	
10474	Colorectal Cancer	TMEM14A	0.00296	2.575291	
10475	Colorectal Cancer	CDC6	0	2.585624	
10476	Colorectal Cancer	AHCY	0.000002	2.587594	
10477	Colorectal Cancer	ABCA13	0	2.590263	
10478	Colorectal Cancer	CCBL1	0	2.59711	
10479	Colorectal Cancer	CASS4	0	2.601324	
10480	Colorectal Cancer	PSAP	0.002393	2.610683	
10481	Colorectal Cancer	CTNNBL1	0	2.612824	
10482	Colorectal Cancer	NPVF	0	2.613849	
10483	Colorectal Cancer	LIN7A	0	2.628195	
10484	Colorectal Cancer	FAM176A	0	2.634253	
10485	Colorectal Cancer	MMP13	0	2.64178	
10486	Colorectal Cancer	FBXL13	0	2.642704	
10487	Colorectal Cancer	FGD5	0	2.646435	
10488	Colorectal Cancer	WDR31	0	2.648876	
10489	Colorectal Cancer	TMEM161A	0	2.649004	
10490	Colorectal Cancer	CCNA2	0.000001	2.6516	
10491	Colorectal Cancer	PPM1F	0.001854	2.653937	
10492	Colorectal Cancer	EIF3B	0.001493	2.65534	
10493	Colorectal Cancer	RHBDF1	0.000215	2.662688	
10494	Colorectal Cancer	ALDH4A1	0.000232	2.672146	
10495	Colorectal Cancer	NEB	0	2.673266	
10496	Colorectal Cancer	ZNF474	0	2.681025	
10497	Colorectal Cancer	MSLN	0	2.690638	
10498	Colorectal Cancer	BOP1	0	2.693284	

10499	Colorectal Cancer	AP1S2	0.003236	2.693499	
10500	Colorectal Cancer	CGNL1	0.000004	2.694607	
10501	Colorectal Cancer	GBP1	0	2.698104	
10502	Colorectal Cancer	CDC25B	0.000001	2.70041	
10503	Colorectal Cancer	EIF2AK2	0.004176	2.716897	
10504	Colorectal Cancer	STAU1	0.004644	2.717209	
10505	Colorectal Cancer	RFC3	0	2.720527	
10506	Colorectal Cancer	XKRX	0	2.73967	
10507	Colorectal Cancer	EIF5A	0	2.754041	
10508	Colorectal Cancer	NPC1L1	0	2.754101	
10509	Colorectal Cancer	FEN1	0.000001	2.754245	
10510	Colorectal Cancer	LOC647979	0.004217	2.755327	
10511	Colorectal Cancer	PTPRU	0	2.768652	
10512	Colorectal Cancer	ABCC2	0	2.78484	
10513	Colorectal Cancer	NCOA3	0.003805	2.809919	
10514	Colorectal Cancer	CAMSAP1L1	0.002817	2.822373	
10515	Colorectal Cancer	CLNK	0	2.822808	
10516	Colorectal Cancer	ADAMTS3	0	2.824047	
10517	Colorectal Cancer	TEAD4	0.000001	2.833619	
10518	Colorectal Cancer	LGR5	0.000001	2.855969	
10519	Colorectal Cancer	LMNB2	0	2.858719	
10520	Colorectal Cancer	NT5DC2	0	2.876001	
10521	Colorectal Cancer	EFNA3	0	2.90539	
10522	Colorectal Cancer	HNRNPL	0	2.905758	
10523	Colorectal Cancer	DYX1C1	0	2.918594	
10524	Colorectal Cancer	NNMT	0.000273	2.93126	
10525	Colorectal Cancer	C11orf45	0	2.931764	
10526	Colorectal Cancer	SLC7A5	0.000001	2.932664	
10527	Colorectal Cancer	CFI	0.000174	2.93439	
10528	Colorectal Cancer	MED25	0.000405	2.953415	
10529	Colorectal Cancer	C4orf39	0	2.956402	
10530	Colorectal Cancer	GPX1	0.000488	2.957755	
10531	Colorectal Cancer	INHBE	0.000642	2.971835	
10532	Colorectal Cancer	SHMT2	0	2.982462	
10533	Colorectal Cancer	C10orf10	0.000005	2.992878	
10534	Colorectal Cancer	MAEL	0	2.996037	
10535	Colorectal Cancer	PPP4R4	0	2.999379	
10536	Colorectal Cancer	MACC1	0.003549	3.024789	
10537	Colorectal Cancer	KIF4A	0.000001	3.052197	
10538	Colorectal Cancer	GINS1	0	3.07162	
10539	Colorectal Cancer	F12	0	3.082553	
10540	Colorectal Cancer	EDAR	0	3.098888	
10541	Colorectal Cancer	STAT1	0	3.107809	
10542	Colorectal Cancer	LCA5	0	3.115799	
10543	Colorectal Cancer	TRIP13	0	3.129853	
10544	Colorectal Cancer	RNF219	0.000471	3.147289	
10545	Colorectal Cancer	STK31	0	3.147914	
10546	Colorectal Cancer	F5	0.00014	3.231423	
10547	Colorectal Cancer	CA9	0	3.241118	
10548	Colorectal Cancer	NUTF2	0	3.241433	

10549	Colorectal Cancer	CEP55	0	3.248565	
10550	Colorectal Cancer	MMP14	0	3.249611	
10551	Colorectal Cancer	SOD2	0	3.254564	
10552	Colorectal Cancer	FOXM1	0	3.25519	
10553	Colorectal Cancer	GRAMD1B	0	3.261789	
10554	Colorectal Cancer	PIWIL1	0	3.34161	
10555	Colorectal Cancer	CERKL	0	3.342308	
10556	Colorectal Cancer	CDH2	0	3.362404	
10557	Colorectal Cancer	BMP7	0	3.38987	
10558	Colorectal Cancer	MCM4	0	3.415645	
10559	Colorectal Cancer	TACSTD2	0	3.446898	
10560	Colorectal Cancer	MIF	0.000001	3.504093	
10561	Colorectal Cancer	GPSM2	0.000018	3.562648	
10562	Colorectal Cancer	IFIT5	0.002833	3.586507	
10563	Colorectal Cancer	VNN1	0.000533	3.670827	
10564	Colorectal Cancer	PBK	0.000001	3.714962	
10565	Colorectal Cancer	SIM2	0	3.727166	
10566	Colorectal Cancer	NKD1	0	3.731564	
10567	Colorectal Cancer	KLK6	0	3.733988	
10568	Colorectal Cancer	ECT2	0	3.796085	
10569	Colorectal Cancer	APOE	0	3.797515	
10570	Colorectal Cancer	TMPRSS3	0	3.798986	
10571	Colorectal Cancer	MMP11	0	3.817899	
10572	Colorectal Cancer	TMEM97	0	3.826889	
10573	Colorectal Cancer	KCNJ15	0	3.837106	
10574	Colorectal Cancer	B3GALTL	0.049356	3.840546	
10575	Colorectal Cancer	AQP9	0.000053	3.849158	
10576	Colorectal Cancer	HSD11B1	0.000121	3.932743	
10577	Colorectal Cancer	PMAIP1	0	4.0525	
10578	Colorectal Cancer	CLDN2	0	4.07628	
10579	Colorectal Cancer	CXCL9	0	4.116745	
10580	Colorectal Cancer	IL24	0	4.120371	
10581	Colorectal Cancer	FTL	0.003014	4.124829	
10582	Colorectal Cancer	IFITM1	0	4.28338	
10583	Colorectal Cancer	ASGR2	0.000553	4.302714	
10584	Colorectal Cancer	SERPINA5	0.000352	4.345646	
10585	Colorectal Cancer	SRPX2	0	4.507537	
10586	Colorectal Cancer	CD81	0.00447	4.590045	
10587	Colorectal Cancer	OLFML2B	0	4.638492	
10588	Colorectal Cancer	SULT2A1	0.000605	4.720173	
10589	Colorectal Cancer	FMO3	0.000071	4.720673	
10590	Colorectal Cancer	SERPINF2	0.00067	4.841546	
10591	Colorectal Cancer	CXCL10	0	4.8737	
10592	Colorectal Cancer	SLCO1B3	0	5.007902	
10593	Colorectal Cancer	DACH1	0.001964	5.017765	
10594	Colorectal Cancer	CXCL1	0	5.226033	
10595	Colorectal Cancer	COLEC11	0	5.278582	
10596	Colorectal Cancer	COL1A2	0	5.292898	
10597	Colorectal Cancer	CP	0.00038	5.509002	
10598	Colorectal Cancer	CPB2	0.000432	5.613978	

10599	Colorectal Cancer	C3	0.000006	5.668511	
10600	Colorectal Cancer	SLC13A5	0.000331	6.076943	
10601	Colorectal Cancer	C8A	0.000531	6.120991	
10602	Colorectal Cancer	SRSF6	0.000001	6.156758	
10603	Colorectal Cancer	DSC3	0	6.40146	
10604	Colorectal Cancer	MMP12	0	6.617377	
10605	Colorectal Cancer	RBP4	0.000557	6.658636	
10606	Colorectal Cancer	ASGR1	0.000048	6.679332	
10607	Colorectal Cancer	C5	0.00011	6.687382	
10608	Colorectal Cancer	APOH	0.000429	6.75181	
10609	Colorectal Cancer	FCN3	0.000009	6.844049	
10610	Colorectal Cancer	APOC2	0.000051	6.904064	
10611	Colorectal Cancer	AGXT	0.000773	6.935567	
10612	Colorectal Cancer	HPD	0.000531	7.014538	
10613	Colorectal Cancer	C6	0.00046	7.054535	
10614	Colorectal Cancer	SAA4	0.000529	7.065	
10615	Colorectal Cancer	CXCL3	0	7.233392	
10616	Colorectal Cancer	PLG	0.000498	7.361402	
10617	Colorectal Cancer	ITIH1	0.000337	7.554118	
10618	Colorectal Cancer	ZAK	0.000658	7.747274	
10619	Colorectal Cancer	C4BPA	0.000535	7.948833	
10620	Colorectal Cancer	APOC1	0.000001	8.000367	
10621	Colorectal Cancer	TTR	0.000676	8.109081	
10622	Colorectal Cancer	CYP2C8	0.000673	8.207707	
10623	Colorectal Cancer	DSG3	0	8.93798	
10624	Colorectal Cancer	LBP	0.000127	8.984359	
10625	Colorectal Cancer	HAMP	0.000059	9.033264	
10626	Colorectal Cancer	IL1A	0	9.050041	
10627	Colorectal Cancer	TCN1	0	9.094781	
10628	Colorectal Cancer	KRT23	0	9.22809	
10629	Colorectal Cancer	APCS	0.000082	9.596353	
10630	Colorectal Cancer	KNG1	0.000331	10.388199	
10631	Colorectal Cancer	HRG	0.000312	11.044669	
10632	Colorectal Cancer	SERPINC1	0.000285	11.791284	
10633	Colorectal Cancer	SERPINA3	0.000001	11.942192	
10634	Colorectal Cancer	APOB	0.000088	13.0809	
10635	Colorectal Cancer	AHSG	0.000112	13.371622	
10636	Colorectal Cancer	APOC3	0.000181	13.701454	
10637	Colorectal Cancer	HPX	0.000192	14.287143	
10638	Colorectal Cancer	FGG	0.000014	14.651355	
10639	Colorectal Cancer	F2	0.000047	15.443368	
10640	Colorectal Cancer	VTN	0.000044	15.844447	
10641	Colorectal Cancer	ORM2	0.00009	16.094898	
10642	Colorectal Cancer	CYP2E1	0.000072	16.227626	
10643	Colorectal Cancer	FGL1	0.000039	18.32827	
10644	Colorectal Cancer	TF	0.000068	18.608249	
10645	Colorectal Cancer	GC	0.000013	20.947027	
10646	Colorectal Cancer	AMBP	0.000021	21.099729	
10647	Colorectal Cancer	APOA2	0.000023	21.365529	
10648	Colorectal Cancer	HP	0.000009	21.852969	

10649	Colorectal Cancer	ALB	0.000007	23.019959	
10650	Colorectal Cancer	APOA1	0.000018	23.169381	
10651	Colorectal Cancer	FGB	0.000008	23.984148	
10652	Colorectal Cancer	FGA	0.000008	25.158992	
10653	Colorectal Cancer	CRP	0.000002	25.311911	
10654	Colorectal Cancer	ORM1	0.000008	25.514711	
10655	Colorectal Neoplasms	GCG	0	-26.920825	
10656	Colorectal Neoplasms	CLCA1	0	-13.979504	
10657	Colorectal Neoplasms	MYH11	0.000001	-12.464317	
10658	Colorectal Neoplasms	SLC26A2	0	-11.558375	
10659	Colorectal Neoplasms	ADAMDEC1	0	-10.152414	
10660	Colorectal Neoplasms	KLF4	0	-5.50973	
10661	Colorectal Neoplasms	ABCA8	0	-5.466247	
10662	Colorectal Neoplasms	LRRC19	0	-5.381388	
10663	Colorectal Neoplasms	PADI2	0	-5.278838	
10664	Colorectal Neoplasms	FLJ21511	0	-4.96684	
10665	Colorectal Neoplasms	F13A1	0	-4.717275	
10666	Colorectal Neoplasms	HSD11B2	0	-4.642659	
10667	Colorectal Neoplasms	REP15	0	-4.575296	
10668	Colorectal Neoplasms	SPARCL1	0	-4.431656	
10669	Colorectal Neoplasms	ITM2C	0	-4.229678	
10670	Colorectal Neoplasms	FRZB	0	-4.185366	
10671	Colorectal Neoplasms	CKB	0	-4.185161	
10672	Colorectal Neoplasms	DSCR1L1	0	-4.065147	
10673	Colorectal Neoplasms	JARID1D	0	-3.998035	
10674	Colorectal Neoplasms	DST	0	-3.84509	
10675	Colorectal Neoplasms	MEP1A	0	-3.653428	
10676	Colorectal Neoplasms	KRT20	0	-3.617039	
10677	Colorectal Neoplasms	NSMAF	0	-3.490679	
10678	Colorectal Neoplasms	TNFRSF17	0	-3.482547	
10679	Colorectal Neoplasms	CHL1	0	-3.44257	
10680	Colorectal Neoplasms	CD47	0	-3.425163	
10681	Colorectal Neoplasms	B3GALT5	0	-3.421405	
10682	Colorectal Neoplasms	LGALS4	0	-3.375029	
10683	Colorectal Neoplasms	FTH1	0	-3.343445	
10684	Colorectal Neoplasms	MGLL	0	-3.330854	
10685	Colorectal Neoplasms	SLC9A2	0	-3.285396	
10686	Colorectal Neoplasms	KLRB1	0	-3.231369	
10687	Colorectal Neoplasms	ATP8B1	0	-3.228551	
10688	Colorectal Neoplasms	CCDC18	0	-3.194796	
10689	Colorectal Neoplasms	PTGER4	0	-3.176752	
10690	Colorectal Neoplasms	GCNT3	0	-3.173418	
10691	Colorectal Neoplasms	FBLN1	0	-3.123876	
10692	Colorectal Neoplasms	DPT	0	-3.027548	
10693	Colorectal Neoplasms	CPA3	0	-2.996718	
10694	Colorectal Neoplasms	SSPN	0	-2.996206	
10695	Colorectal Neoplasms	SCNN1B	0	-2.974333	
10696	Colorectal Neoplasms	PRIC285	0	-2.887749	
10697	Colorectal Neoplasms	GSN	0	-2.854124	
10698	Colorectal Neoplasms	FGL2	0	-2.817655	

10699	Colorectal Neoplasms	ETHE1	0	-2.789342	
10700	Colorectal Neoplasms	LGALS2	0	-2.769808	
10701	Colorectal Neoplasms	STMN2	0	-2.750087	
10702	Colorectal Neoplasms	ENDOD1	0	-2.748799	
10703	Colorectal Neoplasms	KIT	0	-2.722354	
10704	Colorectal Neoplasms	SRI	0	-2.719712	
10705	Colorectal Neoplasms	PDGFRA	0	-2.653227	
10706	Colorectal Neoplasms	TTRAP	0	-2.65296	
10707	Colorectal Neoplasms	ASAHL	0	-2.645352	
10708	Colorectal Neoplasms	SPIB	0	-2.629843	
10709	Colorectal Neoplasms	MAOA	0	-2.629701	
10710	Colorectal Neoplasms	AOC3	0	-2.617512	
10711	Colorectal Neoplasms	NR3C2	0	-2.616894	
10712	Colorectal Neoplasms	ENTPD5	0	-2.582646	
10713	Colorectal Neoplasms	MALL	0	-2.526251	
10714	Colorectal Neoplasms	CMAH	0	-2.481824	
10715	Colorectal Neoplasms	LMNA	0	-2.46552	
10716	Colorectal Neoplasms	AKAP9	0	-2.41686	
10717	Colorectal Neoplasms	CAPN5	0	-2.323863	
10718	Colorectal Neoplasms	CCL23	0	-2.282087	
10719	Colorectal Neoplasms	FRYL	0	-2.255563	
10720	Colorectal Neoplasms	ENTPD3	0	-2.224412	
10721	Colorectal Neoplasms	ST7L	0	-2.223282	
10722	Colorectal Neoplasms	VSIG2	0	-2.221568	
10723	Colorectal Neoplasms	BHLHB3	0	-2.202249	
10724	Colorectal Neoplasms	LOC285016	0	-2.201143	
10725	Colorectal Neoplasms	LPAR1	0	-2.101231	
10726	Colorectal Neoplasms	FAS	0	-2.09611	
10727	Colorectal Neoplasms	SLC22A18AS	0	-2.068933	
10728	Colorectal Neoplasms	PYY2	0	-2.037223	
10729	Colorectal Neoplasms	PAG1	0	-2.033606	
10730	Colorectal Neoplasms	TSPAN3	0	-2.021741	
10731	Colorectal Neoplasms	HERPUD1	0	-2.008187	
10732	Colorectal Neoplasms	CBX3	0.000002	2.001406	
10733	Colorectal Neoplasms	LONP1	0	2.008715	
10734	Colorectal Neoplasms	MTA2	0.000001	2.010076	
10735	Colorectal Neoplasms	KIF15	0	2.015372	
10736	Colorectal Neoplasms	GOLT1B	0	2.015554	
10737	Colorectal Neoplasms	RIPK2	0	2.016821	
10738	Colorectal Neoplasms	MTERFD1	0.000001	2.018794	
10739	Colorectal Neoplasms	KIF11	0.000001	2.025286	
10740	Colorectal Neoplasms	NUP107	0	2.028406	
10741	Colorectal Neoplasms	CCDC59	0	2.030633	
10742	Colorectal Neoplasms	SET	0	2.042096	
10743	Colorectal Neoplasms	TIMELESS	0	2.051896	
10744	Colorectal Neoplasms	OIP5	0.000001	2.065998	
10745	Colorectal Neoplasms	RUVBL1	0	2.069813	
10746	Colorectal Neoplasms	NUP205	0.000001	2.074642	
10747	Colorectal Neoplasms	STT3A	0	2.077468	
10748	Colorectal Neoplasms	ENO1	0.000002	2.078688	

10749	Colorectal Neoplasms	RUVBL2	0	2.080554	
10750	Colorectal Neoplasms	RFC5	0.000001	2.096438	
10751	Colorectal Neoplasms	EPPK1	0.000001	2.101393	
10752	Colorectal Neoplasms	SLC25A32	0.000001	2.113102	
10753	Colorectal Neoplasms	GINS2	0.000001	2.11675	
10754	Colorectal Neoplasms	NUP85	0.000001	2.11777	
10755	Colorectal Neoplasms	RBM28	0	2.120363	
10756	Colorectal Neoplasms	NCAPG2	0	2.125408	
10757	Colorectal Neoplasms	CAD	0.000001	2.127343	
10758	Colorectal Neoplasms	GTF2IRD1	0	2.133547	
10759	Colorectal Neoplasms	FANCL	0	2.139324	
10760	Colorectal Neoplasms	GZMB	0	2.14732	
10761	Colorectal Neoplasms	KIF2C	0.000001	2.154208	
10762	Colorectal Neoplasms	OAS3	0.000001	2.155018	
10763	Colorectal Neoplasms	FANCG	0	2.166676	
10764	Colorectal Neoplasms	CCL4	0	2.174968	
10765	Colorectal Neoplasms	RAB15	0	2.188293	
10766	Colorectal Neoplasms	JAG2	0.000001	2.191192	
10767	Colorectal Neoplasms	WDR43	0.000001	2.194866	
10768	Colorectal Neoplasms	POLR1D	0.000001	2.252838	
10769	Colorectal Neoplasms	NUP37	0.000001	2.262069	
10770	Colorectal Neoplasms	FUT8	0	2.267452	
10771	Colorectal Neoplasms	PITX1	0.000001	2.26755	
10772	Colorectal Neoplasms	KIF20A	0	2.295043	
10773	Colorectal Neoplasms	MICB	0	2.307009	
10774	Colorectal Neoplasms	COL11A1	0	2.322616	
10775	Colorectal Neoplasms	HS2ST1	0.000002	2.328743	
10776	Colorectal Neoplasms	CBFB	0	2.334836	
10777	Colorectal Neoplasms	CDC42EP1	0.000001	2.339437	
10778	Colorectal Neoplasms	SORD	0.000001	2.341712	
10779	Colorectal Neoplasms	MRPS17	0.000001	2.391333	
10780	Colorectal Neoplasms	NOP56	0.000001	2.40229	
10781	Colorectal Neoplasms	ZWILCH	0	2.403617	
10782	Colorectal Neoplasms	ARNTL2	0	2.425129	
10783	Colorectal Neoplasms	COL1A2	0	2.433157	
10784	Colorectal Neoplasms	PSAT1	0.000001	2.45864	
10785	Colorectal Neoplasms	NFE2L3	0	2.463088	
10786	Colorectal Neoplasms	CSE1L	0.000001	2.467005	
10787	Colorectal Neoplasms	C12orf11	0	2.477935	
10788	Colorectal Neoplasms	VARS	0	2.490948	
10789	Colorectal Neoplasms	ICAM1	0	2.498583	
10790	Colorectal Neoplasms	FAP	0	2.53712	
10791	Colorectal Neoplasms	MCM7	0	2.562626	
10792	Colorectal Neoplasms	UBE2L6	0.000001	2.570384	
10793	Colorectal Neoplasms	PSMG1	0.000001	2.572526	
10794	Colorectal Neoplasms	CDC6	0	2.585624	
10795	Colorectal Neoplasms	AHCY	0.000002	2.587594	
10796	Colorectal Neoplasms	COL10A1	0	2.650829	
10797	Colorectal Neoplasms	CCNA2	0.000001	2.6516	
10798	Colorectal Neoplasms	BOP1	0	2.693284	

10799	Colorectal Neoplasms	GBP1	0	2.698104	
10800	Colorectal Neoplasms	CDC25B	0.000001	2.70041	
10801	Colorectal Neoplasms	C7orf68	0.000001	2.713761	
10802	Colorectal Neoplasms	RFC3	0	2.720527	
10803	Colorectal Neoplasms	EIF5A	0	2.754041	
10804	Colorectal Neoplasms	FEN1	0.000001	2.754245	
10805	Colorectal Neoplasms	TEAD4	0.000001	2.833619	
10806	Colorectal Neoplasms	LGR5	0.000001	2.855969	
10807	Colorectal Neoplasms	LMNB2	0	2.858719	
10808	Colorectal Neoplasms	NT5DC2	0	2.876001	
10809	Colorectal Neoplasms	HNRNPL	0	2.905758	
10810	Colorectal Neoplasms	SLC7A5	0.000001	2.932664	
10811	Colorectal Neoplasms	SHMT2	0	2.982462	
10812	Colorectal Neoplasms	THBS2	0	2.987513	
10813	Colorectal Neoplasms	KIF4A	0.000001	3.052197	
10814	Colorectal Neoplasms	GINS1	0	3.07162	
10815	Colorectal Neoplasms	F12	0	3.082553	
10816	Colorectal Neoplasms	STAT1	0	3.107809	
10817	Colorectal Neoplasms	TRIP13	0	3.129853	
10818	Colorectal Neoplasms	NUTF2	0	3.241433	
10819	Colorectal Neoplasms	CEP55	0	3.248565	
10820	Colorectal Neoplasms	SOD2	0	3.254564	
10821	Colorectal Neoplasms	FOXM1	0	3.25519	
10822	Colorectal Neoplasms	MCM4	0	3.415645	
10823	Colorectal Neoplasms	TACSTD2	0	3.446898	
10824	Colorectal Neoplasms	MIF	0.000001	3.504093	
10825	Colorectal Neoplasms	PBK	0.000001	3.714962	
10826	Colorectal Neoplasms	ECT2	0	3.796085	
10827	Colorectal Neoplasms	TMEM97	0	3.826889	
10828	Colorectal Neoplasms	B3GALTL	0.049356	3.840546	
10829	Colorectal Neoplasms	PMAIP1	0	4.0525	
10830	Colorectal Neoplasms	CXCL9	0	4.116745	
10831	Colorectal Neoplasms	AGT	0	4.16549	
10832	Colorectal Neoplasms	CFB	0	4.493717	
10833	Colorectal Neoplasms	CXCL10	0	4.8737	
10834	Colorectal Neoplasms	SRSF6	0.000001	6.156758	
10835	Colorectal Neoplasms	MMP12	0	6.617377	
10836	Colorectal Neoplasms	CXCL3	0	7.233392	
10837	Colorectal Neoplasms	SPP1	0	7.465531	
10838	Constipation	KLK10	0.032298	-2.616816	
10839	Constipation	ALDH3B2	0.036643	-2.046938	
10840	CXCR4 Receptors	ATP6V0A4	0	-5.585106	
10841	CXCR4 Receptors	BTG1	0	-4.77867	
10842	CXCR4 Receptors	DPYSL3	0	-4.290677	
10843	CXCR4 Receptors	ARL4	0	-3.689439	
10844	CXCR4 Receptors	HBG2	0	-3.305153	
10845	CXCR4 Receptors	MGST1	0	-3.166764	
10846	CXCR4 Receptors	MIB2	0	-2.94675	
10847	CXCR4 Receptors	MYT1	0	-2.83628	
10848	CXCR4 Receptors	FSTL3	0	-2.83047	

10849	CXCR4 Receptors	FGF3	0	-2.760704	
10850	CXCR4 Receptors	MFAP2	0	-2.703116	
10851	CXCR4 Receptors	CORO1A	0	-2.530584	
10852	CXCR4 Receptors	TPRXL	0	-2.441202	
10853	CXCR4 Receptors	PHLDA1	0	-2.412442	
10854	CXCR4 Receptors	WDR72	0	-2.407779	
10855	CXCR4 Receptors	ITM2C	0	-2.390478	
10856	CXCR4 Receptors	PALLD	0	-2.361907	
10857	CXCR4 Receptors	ABLIM2	0	-2.341479	
10858	CXCR4 Receptors	CLEC11A	0	-2.301792	
10859	CXCR4 Receptors	TXNDC12	0	-2.241355	
10860	CXCR4 Receptors	ABCD1	0	-2.204834	
10861	CXCR4 Receptors	PPARG	0	-2.200591	
10862	CXCR4 Receptors	KCNH2	0	-2.200463	
10863	CXCR4 Receptors	ATP1A1	0	-2.152291	
10864	CXCR4 Receptors	KRT8	0	-2.147206	
10865	CXCR4 Receptors	NAT12	0	-2.121314	
10866	CXCR4 Receptors	FGFRL1	0	-2.001723	
10867	CXCR4 Receptors	WDR71	0	2.007033	
10868	CXCR4 Receptors	LOC285989	0	2.036076	
10869	CXCR4 Receptors	GAMT	0	2.041534	
10870	CXCR4 Receptors	CD99L2	0	2.073986	
10871	CXCR4 Receptors	ARNT2	0	2.090516	
10872	CXCR4 Receptors	CDS1	0	2.110718	
10873	CXCR4 Receptors	C18orf17	0	2.110862	
10874	CXCR4 Receptors	UGT1A3	0	2.117134	
10875	CXCR4 Receptors	AP1S2	0	2.141072	
10876	CXCR4 Receptors	FLJ20647	0	2.149431	
10877	CXCR4 Receptors	ASB4	0	2.159825	
10878	CXCR4 Receptors	PDHA1	0	2.171695	
10879	CXCR4 Receptors	MAPK9	0	2.176151	
10880	CXCR4 Receptors	MIA	0	2.188193	
10881	CXCR4 Receptors	LRIG1	0	2.194723	
10882	CXCR4 Receptors	EPHB4	0	2.197242	
10883	CXCR4 Receptors	DDAH1	0	2.220748	
10884	CXCR4 Receptors	FUCA1	0	2.238861	
10885	CXCR4 Receptors	RBP7	0	2.244218	
10886	CXCR4 Receptors	IGFBP2	0	2.246886	
10887	CXCR4 Receptors	CXYorf3	0	2.316379	
10888	CXCR4 Receptors	CRIM1	0	2.32511	
10889	CXCR4 Receptors	ARMC10	0	2.340054	
10890	CXCR4 Receptors	TMEM60	0	2.347597	
10891	CXCR4 Receptors	RAB9A	0	2.353768	
10892	CXCR4 Receptors	MSI2	0	2.357038	
10893	CXCR4 Receptors	SLC27A2	0	2.368735	
10894	CXCR4 Receptors	MAOA	0	2.374199	
10895	CXCR4 Receptors	CTPS2	0	2.408193	
10896	CXCR4 Receptors	CYBRD1	0	2.419522	
10897	CXCR4 Receptors	BCAS4	0	2.485147	
10898	CXCR4 Receptors	TUBB2B	0	2.490356	

10899	CXCR4 Receptors	MID2	0	2.521398	
10900	CXCR4 Receptors	EDARADD	0	2.537187	
10901	CXCR4 Receptors	COPS6	0	2.667141	
10902	CXCR4 Receptors	GYG2	0	2.668019	
10903	CXCR4 Receptors	SCD5	0	2.672064	
10904	CXCR4 Receptors	IFITM2	0	2.713718	
10905	CXCR4 Receptors	MRPS6	0	2.713819	
10906	CXCR4 Receptors	NUAK1	0	2.719478	
10907	CXCR4 Receptors	FLJ20489	0	2.72879	
10908	CXCR4 Receptors	DUS4L	0	2.753515	
10909	CXCR4 Receptors	ROR2	0	2.869131	
10910	CXCR4 Receptors	BCAP29	0	2.933524	
10911	CXCR4 Receptors	MGC22793	0	2.957372	
10912	CXCR4 Receptors	CNKSR3	0	3.035668	
10913	CXCR4 Receptors	FAIM3	0	3.054141	
10914	CXCR4 Receptors	BMP7	0	3.078688	
10915	CXCR4 Receptors	4-Sep	0	3.135722	
10916	CXCR4 Receptors	PCSK1N	0	3.227366	
10917	CXCR4 Receptors	IDS	0	3.259512	
10918	CXCR4 Receptors	FLJ14213	0	3.370477	
10919	CXCR4 Receptors	MARCKS	0	3.388638	
10920	CXCR4 Receptors	HPGD	0	3.415895	
10921	CXCR4 Receptors	MSX1	0	3.456937	
10922	CXCR4 Receptors	MCOLN2	0	3.546994	
10923	CXCR4 Receptors	GTPBP6	0	3.926675	
10924	CXCR4 Receptors	CD99	0	4.141912	
10925	CXCR4 Receptors	ENC1	0	4.237816	
10926	CXCR4 Receptors	SYK	0	4.271278	
10927	CXCR4 Receptors	C1orf178	0	4.311505	
10928	CXCR4 Receptors	SLC1A3	0	4.363429	
10929	CXCR4 Receptors	KIF1A	0	4.453252	
10930	CXCR4 Receptors	AXL	0	4.584111	
10931	CXCR4 Receptors	NPC2	0	4.716078	
10932	CXCR4 Receptors	CXCL16	0	4.768333	
10933	CXCR4 Receptors	NCALD	0	5.058307	
10934	CXCR4 Receptors	LTB4DH	0	6.60835	
10935	CXCR4 Receptors	TGFBI	0	7.416049	
10936	CXCR4 Receptors	IRX3	0	7.50161	
10937	CXCR4 Receptors	ID2	0	8.349183	
10938	CXCR4 Receptors	TMEM98	0	9.656715	
10939	Cystadenocarcinoma, Serous	S100P	0	-82.154941	
10940	Cystadenocarcinoma, Serous	REG1B	0	-70.857102	
10941	Cystadenocarcinoma, Serous	GPX2	0	-69.45286	
10942	Cystadenocarcinoma, Serous	CEACAM6	0	-56.828195	
10943	Cystadenocarcinoma, Serous	CCL14-CCL15	0	-54.92139	
10944	Cystadenocarcinoma, Serous	REG1A	0	-52.321797	
10945	Cystadenocarcinoma, Serous	NOX1	0	-44.954576	
10946	Cystadenocarcinoma, Serous	REG4	0	-37.815357	
10947	Cystadenocarcinoma, Serous	REG3A	0	-34.754038	
10948	Cystadenocarcinoma, Serous	SPINK1	0	-33.962536	

10949	Cystadenocarcinoma, Serous	TFF1	0	-33.69438	
10950	Cystadenocarcinoma, Serous	OLFM4	0	-33.365508	
10951	Cystadenocarcinoma, Serous	MMP3	0	-31.898754	
10952	Cystadenocarcinoma, Serous	TFF3	0	-27.989672	
10953	Cystadenocarcinoma, Serous	CDX2	0	-27.541242	
10954	Cystadenocarcinoma, Serous	CEACAM5	0	-27.4734	
10955	Cystadenocarcinoma, Serous	RNF128	0	-24.735046	
10956	Cystadenocarcinoma, Serous	CLRN3	0	-24.087738	
10957	Cystadenocarcinoma, Serous	DUOX2	0	-23.706224	
10958	Cystadenocarcinoma, Serous	DPEP1	0	-22.948238	
10959	Cystadenocarcinoma, Serous	VIL1	0	-22.752675	
10960	Cystadenocarcinoma, Serous	TDGF1	0	-22.484115	
10961	Cystadenocarcinoma, Serous	CDX1	0	-22.432702	
10962	Cystadenocarcinoma, Serous	MUC12	0	-22.333287	
10963	Cystadenocarcinoma, Serous	CDH17	0	-20.334856	
10964	Cystadenocarcinoma, Serous	SPINK4	0	-20.057011	
10965	Cystadenocarcinoma, Serous	CCL20	0	-19.659908	
10966	Cystadenocarcinoma, Serous	ZG16	0	-19.622259	
10967	Cystadenocarcinoma, Serous	ASCL2	0	-18.789439	
10968	Cystadenocarcinoma, Serous	NCRNA00261	0	-18.728798	
10969	Cystadenocarcinoma, Serous	MUC3B	0	-18.430544	
10970	Cystadenocarcinoma, Serous	CYP2B6	0	-18.418425	
10971	Cystadenocarcinoma, Serous	NR1I2	0	-18.259394	
10972	Cystadenocarcinoma, Serous	C10orf99	0	-17.438821	
10973	Cystadenocarcinoma, Serous	DEFA5	0	-17.406271	
10974	Cystadenocarcinoma, Serous	LGALS4	0	-17.382549	
10975	Cystadenocarcinoma, Serous	FERMT1	0	-16.645031	
10976	Cystadenocarcinoma, Serous	EPS8L3	0	-16.501052	
10977	Cystadenocarcinoma, Serous	AREG	0	-16.424613	
10978	Cystadenocarcinoma, Serous	HNF4A	0	-16.093492	
10979	Cystadenocarcinoma, Serous	MUC13	0	-16.045853	
10980	Cystadenocarcinoma, Serous	TRIM15	0	-15.932123	
10981	Cystadenocarcinoma, Serous	COL17A1	0	-15.589913	
10982	Cystadenocarcinoma, Serous	MUC2	0	-15.363161	
10983	Cystadenocarcinoma, Serous	GUCY2C	0	-14.853224	
10984	Cystadenocarcinoma, Serous	TOX3	0	-14.777092	
10985	Cystadenocarcinoma, Serous	POF1B	0	-14.571769	
10986	Cystadenocarcinoma, Serous	DSG3	0	-14.530676	
10987	Cystadenocarcinoma, Serous	MS4A12	0	-14.085907	
10988	Cystadenocarcinoma, Serous	FABP1	0	-13.894196	
10989	Cystadenocarcinoma, Serous	RETNLB	0	-13.782077	
10990	Cystadenocarcinoma, Serous	DMBT1	0	-13.352612	
10991	Cystadenocarcinoma, Serous	DDC	0	-13.169677	
10992	Cystadenocarcinoma, Serous	TRIM31	0	-13.133129	
10993	Cystadenocarcinoma, Serous	ATP10B	0	-13.072314	
10994	Cystadenocarcinoma, Serous	TFF2	0	-12.99526	
10995	Cystadenocarcinoma, Serous	USH1C	0	-12.525619	
10996	Cystadenocarcinoma, Serous	C4BPB	0	-12.509657	
10997	Cystadenocarcinoma, Serous	CEACAM1	0	-12.46148	
10998	Cystadenocarcinoma, Serous	LEFTY1	0	-12.422975	

10999	Cystadenocarcinoma, Serous	FUT6	0	-12.192326	
11000	Cystadenocarcinoma, Serous	PLA2G2A	0	-12.160032	
11001	Cystadenocarcinoma, Serous	SLC39A5	0	-11.982667	
11002	Cystadenocarcinoma, Serous	PPARG	0	-11.629432	
11003	Cystadenocarcinoma, Serous	TSPAN8	0.000001	-11.589339	
11004	Cystadenocarcinoma, Serous	ARL14	0	-11.524536	
11005	Cystadenocarcinoma, Serous	LY6G6D	0	-11.369396	
11006	Cystadenocarcinoma, Serous	GPA33	0	-11.225798	
11007	Cystadenocarcinoma, Serous	EREG	0	-10.567779	
11008	Cystadenocarcinoma, Serous	WNK4	0	-10.504423	
11009	Cystadenocarcinoma, Serous	PRSS3	0	-10.250363	
11010	Cystadenocarcinoma, Serous	CELP	0	-10.197448	
11011	Cystadenocarcinoma, Serous	C2CD4A	0	-10.087429	
11012	Cystadenocarcinoma, Serous	AKR1C3	0	-10.035394	
11013	Cystadenocarcinoma, Serous	AGR2	0	-10.021181	
11014	Cystadenocarcinoma, Serous	SERPINB5	0	-9.884888	
11015	Cystadenocarcinoma, Serous	MUC17	0	-9.614083	
11016	Cystadenocarcinoma, Serous	IL8	0	-9.604294	
11017	Cystadenocarcinoma, Serous	SDR16C5	0	-9.597397	
11018	Cystadenocarcinoma, Serous	ACSL5	0	-9.545692	
11019	Cystadenocarcinoma, Serous	IYD	0	-9.50085	
11020	Cystadenocarcinoma, Serous	CDA	0	-9.435744	
11021	Cystadenocarcinoma, Serous	C2orf89	0	-9.125418	
11022	Cystadenocarcinoma, Serous	MMP12	0	-8.861331	
11023	Cystadenocarcinoma, Serous	LOC283859	0	-8.620237	
11024	Cystadenocarcinoma, Serous	C17orf73	0	-8.61921	
11025	Cystadenocarcinoma, Serous	CFTR	0	-8.61752	
11026	Cystadenocarcinoma, Serous	UGT1A1	0	-8.597117	
11027	Cystadenocarcinoma, Serous	ACE2	0	-8.548223	
11028	Cystadenocarcinoma, Serous	CEL	0	-8.459869	
11029	Cystadenocarcinoma, Serous	FOXA2	0	-8.315197	
11030	Cystadenocarcinoma, Serous	TCN1	0	-8.297113	
11031	Cystadenocarcinoma, Serous	C13orf18	0	-8.222109	
11032	Cystadenocarcinoma, Serous	ETS2	0	-8.020716	
11033	Cystadenocarcinoma, Serous	FGFR4	0	-8.014647	
11034	Cystadenocarcinoma, Serous	PRAP1	0	-7.870295	
11035	Cystadenocarcinoma, Serous	LRRC19	0	-7.83463	
11036	Cystadenocarcinoma, Serous	RAPGEFL1	0	-7.820147	
11037	Cystadenocarcinoma, Serous	ARSE	0	-7.7546	
11038	Cystadenocarcinoma, Serous	CDCA7	0	-7.739772	
11039	Cystadenocarcinoma, Serous	TMED6	0	-7.707651	
11040	Cystadenocarcinoma, Serous	MMP1	0	-7.694799	
11041	Cystadenocarcinoma, Serous	IHH	0	-7.687852	
11042	Cystadenocarcinoma, Serous	ITGA2	0	-7.686786	
11043	Cystadenocarcinoma, Serous	SLCO1B3	0	-7.669545	
11044	Cystadenocarcinoma, Serous	FUT4	0	-7.668576	
11045	Cystadenocarcinoma, Serous	ABCC2	0	-7.641195	
11046	Cystadenocarcinoma, Serous	LOC100288092	0	-7.52454	
11047	Cystadenocarcinoma, Serous	CTSE	0	-7.505301	
11048	Cystadenocarcinoma, Serous	CA1	0	-7.331237	

11049	Cystadenocarcinoma, Serous	PLCB4	0	-7.325962	
11050	Cystadenocarcinoma, Serous	TFPI	0	-7.29702	
11051	Cystadenocarcinoma, Serous	BCL2L15	0	-7.283747	
11052	Cystadenocarcinoma, Serous	SLC25A15	0	-7.20281	
11053	Cystadenocarcinoma, Serous	GIPC2	0	-7.001278	
11054	Cystadenocarcinoma, Serous	PLS1	0	-6.972854	
11055	Cystadenocarcinoma, Serous	CDHR5	0	-6.933833	
11056	Cystadenocarcinoma, Serous	TMEM37	0	-6.905502	
11057	Cystadenocarcinoma, Serous	GPR109B	0	-6.894752	
11058	Cystadenocarcinoma, Serous	CCL18	0	-6.84854	
11059	Cystadenocarcinoma, Serous	GPR120	0	-6.801171	
11060	Cystadenocarcinoma, Serous	UGT2A3	0	-6.76391	
11061	Cystadenocarcinoma, Serous	BAIAP2L2	0	-6.716377	
11062	Cystadenocarcinoma, Serous	FAM3D	0	-6.713634	
11063	Cystadenocarcinoma, Serous	HEPACAM2	0	-6.674208	
11064	Cystadenocarcinoma, Serous	PARM1	0	-6.580271	
11065	Cystadenocarcinoma, Serous	HNF4G	0	-6.505807	
11066	Cystadenocarcinoma, Serous	PIP5K1B	0	-6.480194	
11067	Cystadenocarcinoma, Serous	SLC12A2	0	-6.471073	
11068	Cystadenocarcinoma, Serous	CALML4	0	-6.443105	
11069	Cystadenocarcinoma, Serous	BCL2L14	0	-6.401529	
11070	Cystadenocarcinoma, Serous	MYO7B	0	-6.377428	
11071	Cystadenocarcinoma, Serous	C19orf77	0	-6.357863	
11072	Cystadenocarcinoma, Serous	XPNPEP2	0	-6.276759	
11073	Cystadenocarcinoma, Serous	SATB2	0	-6.275615	
11074	Cystadenocarcinoma, Serous	C1orf125	0	-6.244888	
11075	Cystadenocarcinoma, Serous	PROX1	0	-6.189935	
11076	Cystadenocarcinoma, Serous	UGT8	0	-6.189233	
11077	Cystadenocarcinoma, Serous	NQO1	0	-6.143242	
11078	Cystadenocarcinoma, Serous	FXYD3	0	-6.076999	
11079	Cystadenocarcinoma, Serous	GMDS	0	-6.065593	
11080	Cystadenocarcinoma, Serous	FOXA3	0	-6.009293	
11081	Cystadenocarcinoma, Serous	FABP6	0	-5.886784	
11082	Cystadenocarcinoma, Serous	LRP4	0	-5.803077	
11083	Cystadenocarcinoma, Serous	SLC5A9	0	-5.801662	
11084	Cystadenocarcinoma, Serous	XK	0	-5.784991	
11085	Cystadenocarcinoma, Serous	SLC6A14	0	-5.745974	
11086	Cystadenocarcinoma, Serous	CENPV	0	-5.718385	
11087	Cystadenocarcinoma, Serous	ADCYAP1	0	-5.713573	
11088	Cystadenocarcinoma, Serous	FAM84A	0	-5.670863	
11089	Cystadenocarcinoma, Serous	ITPKA	0	-5.662952	
11090	Cystadenocarcinoma, Serous	IL1A	0	-5.605946	
11091	Cystadenocarcinoma, Serous	PTPRO	0	-5.590431	
11092	Cystadenocarcinoma, Serous	SLCO4A1	0	-5.56316	
11093	Cystadenocarcinoma, Serous	MYB	0	-5.529193	
11094	Cystadenocarcinoma, Serous	MXD1	0	-5.527451	
11095	Cystadenocarcinoma, Serous	LOC100507192	0	-5.501085	
11096	Cystadenocarcinoma, Serous	CYP3A5	0	-5.495996	
11097	Cystadenocarcinoma, Serous	L1TD1	0	-5.423907	
11098	Cystadenocarcinoma, Serous	CLDN2	0	-5.419162	

11099	Cystadenocarcinoma, Serous	ACSL6	0	-5.417387	
11100	Cystadenocarcinoma, Serous	PLEK2	0	-5.40684	
11101	Cystadenocarcinoma, Serous	HEPH	0	-5.404014	
11102	Cystadenocarcinoma, Serous	NOTUM	0	-5.367001	
11103	Cystadenocarcinoma, Serous	BCAN	0	-5.337806	
11104	Cystadenocarcinoma, Serous	CXCL6	0	-5.3255	
11105	Cystadenocarcinoma, Serous	TM4SF20	0	-5.325471	
11106	Cystadenocarcinoma, Serous	GPSM2	0	-5.205413	
11107	Cystadenocarcinoma, Serous	HSD11B2	0	-5.204042	
11108	Cystadenocarcinoma, Serous	RNASE4	0	-5.148506	
11109	Cystadenocarcinoma, Serous	SLC1A7	0	-5.143245	
11110	Cystadenocarcinoma, Serous	RNF43	0	-5.136936	
11111	Cystadenocarcinoma, Serous	SKP2	0	-5.111432	
11112	Cystadenocarcinoma, Serous	CHRDL2	0	-5.080581	
11113	Cystadenocarcinoma, Serous	ANG	0	-5.070518	
11114	Cystadenocarcinoma, Serous	FAR2	0	-5.035241	
11115	Cystadenocarcinoma, Serous	ABCB1	0	-5.031336	
11116	Cystadenocarcinoma, Serous	TRPA1	0	-4.983687	
11117	Cystadenocarcinoma, Serous	DOK4	0	-4.95273	
11118	Cystadenocarcinoma, Serous	HKDC1	0	-4.94349	
11119	Cystadenocarcinoma, Serous	PCCA	0	-4.848573	
11120	Cystadenocarcinoma, Serous	IL22RA1	0	-4.829037	
11121	Cystadenocarcinoma, Serous	CYP2J2	0	-4.80905	
11122	Cystadenocarcinoma, Serous	ITGA6	0	-4.808474	
11123	Cystadenocarcinoma, Serous	MYO1A	0	-4.80138	
11124	Cystadenocarcinoma, Serous	GGH	0	-4.797569	
11125	Cystadenocarcinoma, Serous	SMAGP	0	-4.784792	
11126	Cystadenocarcinoma, Serous	LOC285943	0	-4.715135	
11127	Cystadenocarcinoma, Serous	KIF21B	0	-4.664001	
11128	Cystadenocarcinoma, Serous	PPP1R1B	0	-4.641362	
11129	Cystadenocarcinoma, Serous	FA2H	0	-4.582455	
11130	Cystadenocarcinoma, Serous	PTPRH	0	-4.55447	
11131	Cystadenocarcinoma, Serous	AKR1C2	0	-4.545569	
11132	Cystadenocarcinoma, Serous	PPP1R14D	0	-4.527544	
11133	Cystadenocarcinoma, Serous	TESC	0	-4.483325	
11134	Cystadenocarcinoma, Serous	JPH1	0	-4.465564	
11135	Cystadenocarcinoma, Serous	FIBCD1	0	-4.4655	
11136	Cystadenocarcinoma, Serous	CLC	0	-4.414	
11137	Cystadenocarcinoma, Serous	TMEM54	0	-4.413032	
11138	Cystadenocarcinoma, Serous	LOC400573	0	-4.411655	
11139	Cystadenocarcinoma, Serous	PTGS2	0	-4.392851	
11140	Cystadenocarcinoma, Serous	GNG4	0	-4.344921	
11141	Cystadenocarcinoma, Serous	EDN3	0	-4.304979	
11142	Cystadenocarcinoma, Serous	CKMT1A	0	-4.304626	
11143	Cystadenocarcinoma, Serous	A1CF	0	-4.287529	
11144	Cystadenocarcinoma, Serous	DNASE1L3	0	-4.286649	
11145	Cystadenocarcinoma, Serous	SDCBP2	0	-4.284871	
11146	Cystadenocarcinoma, Serous	NEK3	0	-4.279619	
11147	Cystadenocarcinoma, Serous	IL33	0	-4.215678	
11148	Cystadenocarcinoma, Serous	GSDMB	0	-4.205399	

11149	Cystadenocarcinoma, Serous	CYP2S1	0	-4.204536	
11150	Cystadenocarcinoma, Serous	ANKRD22	0	-4.199105	
11151	Cystadenocarcinoma, Serous	VAV3	0	-4.169535	
11152	Cystadenocarcinoma, Serous	PLA2G10	0	-4.164058	
11153	Cystadenocarcinoma, Serous	SERPINB1	0	-4.16136	
11154	Cystadenocarcinoma, Serous	ALDH1A1	0	-4.14217	
11155	Cystadenocarcinoma, Serous	SLC44A3	0	-4.113565	
11156	Cystadenocarcinoma, Serous	TST	0	-4.11211	
11157	Cystadenocarcinoma, Serous	TRPM6	0	-4.110825	
11158	Cystadenocarcinoma, Serous	ID1	0	-4.108161	
11159	Cystadenocarcinoma, Serous	F12	0	-4.095443	
11160	Cystadenocarcinoma, Serous	CCND2	0	-4.052119	
11161	Cystadenocarcinoma, Serous	GRM8	0.000001	-4.001204	
11162	Cystadenocarcinoma, Serous	PLBD1	0	-3.987028	
11163	Cystadenocarcinoma, Serous	AKR1C1	0	-3.968148	
11164	Cystadenocarcinoma, Serous	IL1B	0	-3.840513	
11165	Cystadenocarcinoma, Serous	HOXD13	0	-3.816547	
11166	Cystadenocarcinoma, Serous	NOSTRIN	0.000001	-3.807538	
11167	Cystadenocarcinoma, Serous	LOC100288781	0	-3.783391	
11168	Cystadenocarcinoma, Serous	CES2	0	-3.782455	
11169	Cystadenocarcinoma, Serous	F2RL2	0	-3.772986	
11170	Cystadenocarcinoma, Serous	STAMBPL1	0	-3.734189	
11171	Cystadenocarcinoma, Serous	FLJ22763	0	-3.684372	
11172	Cystadenocarcinoma, Serous	EPS8	0	-3.659213	
11173	Cystadenocarcinoma, Serous	MET	0.000001	-3.658213	
11174	Cystadenocarcinoma, Serous	GPR128	0	-3.644486	
11175	Cystadenocarcinoma, Serous	TTLL6	0	-3.560995	
11176	Cystadenocarcinoma, Serous	SLC13A3	0	-3.549366	
11177	Cystadenocarcinoma, Serous	ENPP3	0	-3.533505	
11178	Cystadenocarcinoma, Serous	HOXD10	0	-3.531011	
11179	Cystadenocarcinoma, Serous	MTMR11	0	-3.519941	
11180	Cystadenocarcinoma, Serous	RBP2	0	-3.516945	
11181	Cystadenocarcinoma, Serous	PAQR8	0	-3.516316	
11182	Cystadenocarcinoma, Serous	GRTP1	0	-3.481063	
11183	Cystadenocarcinoma, Serous	TPSG1	0	-3.468164	
11184	Cystadenocarcinoma, Serous	LOC146336	0.000001	-3.4242	
11185	Cystadenocarcinoma, Serous	BMP2	0	-3.407192	
11186	Cystadenocarcinoma, Serous	IGHG1	0	-3.405295	
11187	Cystadenocarcinoma, Serous	LOC653602	0	-3.368795	
11188	Cystadenocarcinoma, Serous	NHSL1	0	-3.332856	
11189	Cystadenocarcinoma, Serous	VSNL1	0	-3.317272	
11190	Cystadenocarcinoma, Serous	NFE2L3	0	-3.316706	
11191	Cystadenocarcinoma, Serous	BIRC3	0	-3.312392	
11192	Cystadenocarcinoma, Serous	ME1	0	-3.310242	
11193	Cystadenocarcinoma, Serous	GLRX	0	-3.287368	
11194	Cystadenocarcinoma, Serous	NRARP	0	-3.268686	
11195	Cystadenocarcinoma, Serous	CTSW	0	-3.266565	
11196	Cystadenocarcinoma, Serous	C19orf6	0	-3.261974	
11197	Cystadenocarcinoma, Serous	SCML1	0	-3.250374	
11198	Cystadenocarcinoma, Serous	PREP	0	-3.250109	

11199	Cystadenocarcinoma, Serous	ANXA4	0	-3.248235	
11200	Cystadenocarcinoma, Serous	FGGY	0	-3.247028	
11201	Cystadenocarcinoma, Serous	PHLDA1	0	-3.23686	
11202	Cystadenocarcinoma, Serous	IL3RA	0	-3.221795	
11203	Cystadenocarcinoma, Serous	PIWIL1	0	-3.211322	
11204	Cystadenocarcinoma, Serous	CASP1	0	-3.191909	
11205	Cystadenocarcinoma, Serous	MST4	0	-3.191669	
11206	Cystadenocarcinoma, Serous	CD44	0	-3.190672	
11207	Cystadenocarcinoma, Serous	CASP5	0	-3.138952	
11208	Cystadenocarcinoma, Serous	FLJ40292	0	-3.125208	
11209	Cystadenocarcinoma, Serous	SEMA4G	0	-3.104605	
11210	Cystadenocarcinoma, Serous	APOBEC1	0	-3.102199	
11211	Cystadenocarcinoma, Serous	NAMPT	0	-3.086387	
11212	Cystadenocarcinoma, Serous	ADAP1	0.000001	-3.079807	
11213	Cystadenocarcinoma, Serous	FAM105A	0	-3.05774	
11214	Cystadenocarcinoma, Serous	HR	0	-3.048948	
11215	Cystadenocarcinoma, Serous	AXIN2	0	-3.038785	
11216	Cystadenocarcinoma, Serous	FAM108C1	0.000001	-3.033542	
11217	Cystadenocarcinoma, Serous	FOXP2	0	-3.021112	
11218	Cystadenocarcinoma, Serous	PIGZ	0	-2.955051	
11219	Cystadenocarcinoma, Serous	PRSS1	0	-2.934141	
11220	Cystadenocarcinoma, Serous	ST14	0.000001	-2.868657	
11221	Cystadenocarcinoma, Serous	ETHE1	0	-2.837609	
11222	Cystadenocarcinoma, Serous	UNC5CL	0	-2.83193	
11223	Cystadenocarcinoma, Serous	ANKRD57	0	-2.729228	
11224	Cystadenocarcinoma, Serous	MYH14	0	-2.719676	
11225	Cystadenocarcinoma, Serous	MLKL	0	-2.699129	
11226	Cystadenocarcinoma, Serous	AKR7A3	0	-2.688567	
11227	Cystadenocarcinoma, Serous	SH3BGRL2	0	-2.683424	
11228	Cystadenocarcinoma, Serous	LOC25845	0	-2.682844	
11229	Cystadenocarcinoma, Serous	LOC283508	0	-2.635752	
11230	Cystadenocarcinoma, Serous	PLD1	0	-2.630923	
11231	Cystadenocarcinoma, Serous	GNE	0	-2.624701	
11232	Cystadenocarcinoma, Serous	FMO5	0	-2.599217	
11233	Cystadenocarcinoma, Serous	ASPH	0.000001	-2.584645	
11234	Cystadenocarcinoma, Serous	ZNF703	0	-2.563232	
11235	Cystadenocarcinoma, Serous	CD46	0	-2.504414	
11236	Cystadenocarcinoma, Serous	LIMA1	0	-2.47099	
11237	Cystadenocarcinoma, Serous	KITLG	0	-2.464546	
11238	Cystadenocarcinoma, Serous	3-Mar	0	-2.45257	
11239	Cystadenocarcinoma, Serous	RAB15	0	-2.450992	
11240	Cystadenocarcinoma, Serous	LASS6	0	-2.439166	
11241	Cystadenocarcinoma, Serous	C13orf23	0	-2.428961	
11242	Cystadenocarcinoma, Serous	ZAK	0	-2.411691	
11243	Cystadenocarcinoma, Serous	FRYL	0	-2.391115	
11244	Cystadenocarcinoma, Serous	SOD2	0.000001	-2.363852	
11245	Cystadenocarcinoma, Serous	MUC3A	0	-2.363092	
11246	Cystadenocarcinoma, Serous	CDK8	0	-2.326525	
11247	Cystadenocarcinoma, Serous	LNX2	0	-2.292161	
11248	Cystadenocarcinoma, Serous	PDSS1	0	-2.276462	

11249	Cystadenocarcinoma, Serous	FAM102B	0	-2.264493	
11250	Cystadenocarcinoma, Serous	MYO15B	0	-2.233657	
11251	Cystadenocarcinoma, Serous	RAPGEF5	0	-2.212988	
11252	Cystadenocarcinoma, Serous	PHF19	0	-2.145259	
11253	Cystadenocarcinoma, Serous	HCCS	0	-2.084728	
11254	Cystadenocarcinoma, Serous	CMAS	0	-2.040655	
11255	Cystadenocarcinoma, Serous	FAM195B	0	2.085873	
11256	Death	MFAP2	0	2.032549	
11257	Death	CALD1	0	2.066687	
11258	Death	IGHG1	0	2.086508	
11259	Death	TPM2	0	2.094737	
11260	Death	MMP11	0	2.155168	
11261	Death	TMEM158	0	2.168515	
11262	Death	CTSK	0	2.189639	
11263	Death	SPOCK1	0	2.207104	
11264	Death	WNT5A	0	2.230763	
11265	Death	TAGLN	0	2.230853	
11266	Death	COL15A1	0	2.260351	
11267	Death	SFRP4	0	2.276769	
11268	Death	SULF1	0	2.319154	
11269	Death	MYL9	0.000001	2.334374	
11270	Death	COL6A1	0	2.48556	
11271	Death	MMP3	0	2.756449	
11272	Death	COL11A1	0	2.790647	
11273	Death	TNC	0	2.807085	
11274	Death	MMP1	0.000001	2.832884	
11275	Death	MYH11	0	2.897326	
11276	Death	GAS1	0	3.007882	
11277	Death	MFAP5	0	3.321188	
11278	Death	GREM1	0	3.545274	
11279	Death	ACTG2	0	4.912122	
11280	Diabetes Mellitus, Non-Insulin-Dependent	TLX2	0.027605	-2.413984	
11281	Diabetes Mellitus, Non-Insulin-Dependent	RAX2	0.000615	-2.076901	
11282	Diarrhea	SYNGR3	0.000295	-2.022727	
11283	Disease	CDHR1	0.000406	-4.901889	
11284	Disease	AXIN2	0.000046	-4.587413	
11285	Disease	ZNRF3	0.000007	-3.318925	
11286	Disease	ZNF321	0.000074	-3.298804	
11287	Disease	ZNF777	0.000089	-3.27148	
11288	Disease	RNF43	0.000046	-3.214493	
11289	Disease	B4GALT6	0.000355	-3.16038	
11290	Disease	LOC100505730	0.000125	-3.101631	
11291	Disease	FOXA2	0.000059	-2.956134	
11292	Disease	DDX31	0.000436	-2.917829	
11293	Disease	NPIPL3	0.000109	-2.876296	
11294	Disease	RHEB	0.00009	-2.82394	
11295	Disease	C19orf2	0.00002	-2.78316	
11296	Disease	EFNA3	0.000334	-2.774743	
11297	Disease	AMACR	0.000009	-2.733981	
11298	Disease	CCDC113	0.000034	-2.696156	

11299	Disease	ARHGAP8	0.000084	-2.637195	
11300	Disease	CYP2S1	0.000037	-2.636245	
11301	Disease	TOP1MT	0.000007	-2.597406	
11302	Disease	CFTR	0.000155	-2.533039	
11303	Disease	TMEM201	0.000349	-2.453447	
11304	Disease	ZNF703	0.000227	-2.404688	
11305	Disease	NMNAT3	0.000004	-2.403053	
11306	Disease	ABHD11	0.000124	-2.312944	
11307	Disease	PPM1H	0.000417	-2.307378	
11308	Disease	L2HGDH	0.000098	-2.267435	
11309	Disease	MACROD1	0.000251	-2.25448	
11310	Disease	IYD	0.000401	-2.237171	
11311	Disease	EXPH5	0.00009	-2.225301	
11312	Disease	AADAT	0.000126	-2.18463	
11313	Disease	POF1B	0.000296	-2.176879	
11314	Disease	C8orf33	0.00002	-2.154067	
11315	Disease	NETO2	0.000371	-2.153651	
11316	Disease	PHF14	0.000107	-2.152611	
11317	Disease	GGH	0.000049	-2.142053	
11318	Disease	ALDH1B1	0.000296	-2.07956	
11319	Disease	MEST	0.000006	-2.067237	
11320	Disease	WNK4	0.000292	-2.063297	
11321	Disease	CIRH1A	0.00011	-2.061629	
11322	Disease	PRMT3	0.000227	-2.055489	
11323	Disease	RAB40B	0.00004	-2.046246	
11324	Disease	PUS7	0.000124	-2.045736	
11325	Disease	ZKSCAN1	0.000046	-2.042176	
11326	Disease	TBRG4	0.000124	-2.040378	
11327	Disease	CXADR	0.000271	-2.036179	
11328	Disease	ZNF704	0.000172	-2.025003	
11329	Disease	SERF1A	0.000025	-2.011575	
11330	Disease	LOC644246	0.00014	2.00332	
11331	Disease	CROT	0.000005	2.005005	
11332	Disease	FAM82A1	0.000022	2.006338	
11333	Disease	GPR82	0.000184	2.015709	
11334	Disease	AKR7A3	0	2.030897	
11335	Disease	MFAP2	0	2.032549	
11336	Disease	APOL6	0.000126	2.03811	
11337	Disease	PIGL	0.000012	2.04441	
11338	Disease	HLA-DRA	0.000068	2.047747	
11339	Disease	CADM3	0.000175	2.049044	
11340	Disease	DAO	0.000086	2.052386	
11341	Disease	ALDH6A1	0	2.052514	
11342	Disease	CRELD2	0.000328	2.054979	
11343	Disease	CALD1	0	2.066687	
11344	Disease	XCR1	0.000226	2.073231	
11345	Disease	PARP9	0.000398	2.084306	
11346	Disease	IGHG1	0	2.086508	
11347	Disease	P2RX1	0.000032	2.088197	
11348	Disease	ARRDC4	0.000074	2.090461	

11349	Disease	GPR34	0.000002	2.091083	
11350	Disease	NLRC5	0.00029	2.092173	
11351	Disease	HLA-DRB1	0.000069	2.093284	
11352	Disease	TPM2	0	2.094737	
11353	Disease	BLNK	0.000085	2.111861	
11354	Disease	CPT2	0	2.112139	
11355	Disease	CHRND	0.000019	2.137341	
11356	Disease	PECI	0.000002	2.141699	
11357	Disease	STAT1	0.000143	2.144517	
11358	Disease	CYSLTR1	0.000331	2.152448	
11359	Disease	ASB2	0.000139	2.154799	
11360	Disease	MMP11	0	2.155168	
11361	Disease	IGLV2-23	0.000046	2.167195	
11362	Disease	KIAA1632	0.000005	2.167211	
11363	Disease	CFD	0.000026	2.168027	
11364	Disease	PLCE1	0.00002	2.168151	
11365	Disease	TMEM158	0	2.168515	
11366	Disease	C7orf31	0.000191	2.171703	
11367	Disease	GBP2	0.00039	2.184644	
11368	Disease	CTSK	0	2.189639	
11369	Disease	TNXB	0.000028	2.189652	
11370	Disease	SPOCK1	0	2.207104	
11371	Disease	MPEG1	0.000029	2.209908	
11372	Disease	CD1E	0.000019	2.221066	
11373	Disease	WNT5A	0	2.230763	
11374	Disease	TAGLN	0	2.230853	
11375	Disease	PSKH2	0.000086	2.235311	
11376	Disease	RASA4	0.000187	2.240732	
11377	Disease	FOLR2	0	2.249153	
11378	Disease	EDIL3	0.000024	2.249874	
11379	Disease	ETFDH	0	2.250393	
11380	Disease	LILRB5	0.000019	2.255364	
11381	Disease	COL15A1	0	2.260351	
11382	Disease	LOC100128343	0.000129	2.260726	
11383	Disease	SFRP4	0	2.276769	
11384	Disease	LOC728392	0.000239	2.27988	
11385	Disease	MATN2	0.000139	2.281556	
11386	Disease	TRDMT1	0.000091	2.284247	
11387	Disease	CYP4F22	0.000208	2.288948	
11388	Disease	UBE2L6	0.00012	2.29084	
11389	Disease	ACADM	0	2.297784	
11390	Disease	IGKV4-1	0.000009	2.298194	
11391	Disease	HLA-DPA1	0.00009	2.303741	
11392	Disease	LST1	0.000179	2.307241	
11393	Disease	SAMD15	0.000128	2.310095	
11394	Disease	MAL	0	2.310783	
11395	Disease	KLRF1	0.000028	2.313962	
11396	Disease	CTSC	0.000008	2.318629	
11397	Disease	SULF1	0	2.319154	
11398	Disease	GSG1L	0.000192	2.325745	

11399	Disease	MYL9	0.000001	2.334374	
11400	Disease	ASXL3	0.000235	2.338404	
11401	Disease	ANK2	0.000058	2.343017	
11402	Disease	ZDHHC2	0.000056	2.348905	
11403	Disease	WNT7A	0.000007	2.367889	
11404	Disease	C14orf50	0.000205	2.374697	
11405	Disease	ATP2A3	0.000234	2.375061	
11406	Disease	TSLP	0.000104	2.391528	
11407	Disease	CARD16	0.000164	2.397914	
11408	Disease	ITGA8	0	2.410465	
11409	Disease	TNFRSF11A	0.000034	2.413834	
11410	Disease	MPP4	0.000023	2.430435	
11411	Disease	IGF1	0.000005	2.435048	
11412	Disease	PEBP4	0.000165	2.448878	
11413	Disease	KCNIP4	0.000014	2.458813	
11414	Disease	CD27	0.000145	2.47417	
11415	Disease	CCR2	0.000251	2.474624	
11416	Disease	ASAP3	0	2.477554	
11417	Disease	COL6A1	0	2.48556	
11418	Disease	VWA5A	0.000004	2.491612	
11419	Disease	MYO1A	0.000193	2.501102	
11420	Disease	BTN3A3	0.000042	2.50311	
11421	Disease	TEK	0.000102	2.512206	
11422	Disease	LOC727944	0.000194	2.512875	
11423	Disease	LOC100129503	0.000009	2.525499	
11424	Disease	KLF15	0.000004	2.528165	
11425	Disease	HS2ST1	0	2.537825	
11426	Disease	POU6F1	0.000005	2.545149	
11427	Disease	C1orf186	0.000018	2.545849	
11428	Disease	FSIP2	0.000161	2.571723	
11429	Disease	IGLL5	0.000342	2.579993	
11430	Disease	NDRG2	0	2.584615	
11431	Disease	CD40	0.000376	2.586738	
11432	Disease	TPSAB1	0.000139	2.589849	
11433	Disease	GPR156	0.000042	2.609076	
11434	Disease	CNTN4	0.000202	2.617743	
11435	Disease	PSMB9	0.000013	2.620499	
11436	Disease	CIITA	0.000374	2.620771	
11437	Disease	FGFR2	0.000189	2.622704	
11438	Disease	SFMBT2	0.00009	2.625821	
11439	Disease	HNF1B	0.000018	2.630577	
11440	Disease	GATM	0.000122	2.671051	
11441	Disease	CLEC10A	0	2.717354	
11442	Disease	GRIN2A	0.000165	2.719133	
11443	Disease	TNXA	0.000014	2.726412	
11444	Disease	EPHA7	0.000018	2.726774	
11445	Disease	SCN9A	0.000046	2.731662	
11446	Disease	HLA-DMA	0.000045	2.735534	
11447	Disease	KLRB1	0.000009	2.735812	
11448	Disease	TPSB2	0.000206	2.738168	

11449	Disease	ACADS	0	2.749732	
11450	Disease	BCL2	0	2.750296	
11451	Disease	MMP3	0	2.756449	
11452	Disease	DQX1	0.000018	2.767648	
11453	Disease	LOC727916	0.000037	2.775279	
11454	Disease	CA12	0.000001	2.782355	
11455	Disease	COL11A1	0	2.790647	
11456	Disease	PDGFD	0	2.80325	
11457	Disease	HPGD	0.00018	2.803399	
11458	Disease	TNC	0	2.807085	
11459	Disease	CES3	0.000086	2.812385	
11460	Disease	PRIMA1	0	2.825465	
11461	Disease	MMP1	0.000001	2.832884	
11462	Disease	F13A1	0.000121	2.843092	
11463	Disease	GPR17	0.000017	2.851898	
11464	Disease	COL4A5	0.000147	2.855223	
11465	Disease	LOC100287411	0.000204	2.858673	
11466	Disease	LOC100290278	0.000002	2.878548	
11467	Disease	FXYD1	0	2.882093	
11468	Disease	MYH11	0	2.897326	
11469	Disease	PKNOX2	0.000043	2.90358	
11470	Disease	NRG4	0.00019	2.908078	
11471	Disease	PTPRZ1	0.000001	2.912852	
11472	Disease	CLEC4G	0.000005	2.917786	
11473	Disease	TLR7	0	2.922728	
11474	Disease	C5orf20	0.000092	2.932144	
11475	Disease	SPINK2	0.000064	2.9342	
11476	Disease	SCN3B	0.000111	2.943865	
11477	Disease	ADH1A	0	2.943926	
11478	Disease	MOBP	0.000005	2.950457	
11479	Disease	EPHX2	0.000006	2.950808	
11480	Disease	CNR1	0.000019	2.95424	
11481	Disease	HMGCLL1	0	2.998557	
11482	Disease	BCHE	0.000029	3.000749	
11483	Disease	CHL1	0.000002	3.004944	
11484	Disease	CDK3	0.000002	3.006202	
11485	Disease	GAS1	0	3.007882	
11486	Disease	NAPSB	0.000169	3.015791	
11487	Disease	ALDH1A1	0.000027	3.016823	
11488	Disease	GPR112	0.000142	3.021562	
11489	Disease	ADAM28	0	3.066647	
11490	Disease	HPGDS	0.000011	3.070972	
11491	Disease	FCER1A	0	3.089502	
11492	Disease	CYP2C9	0.000071	3.108913	
11493	Disease	ATP13A4	0.000011	3.125522	
11494	Disease	PBLD	0.000116	3.132459	
11495	Disease	SCN3A	0.000024	3.137641	
11496	Disease	LOC100509968	0.000037	3.156914	
11497	Disease	GGTA1	0	3.160593	
11498	Disease	ELANE	0.000029	3.181908	

11499	Disease	C1orf175	0.000024	3.193148	
11500	Disease	GPR44	0	3.246309	
11501	Disease	SLC39A5	0.000169	3.308423	
11502	Disease	P2RY12	0.000002	3.308991	
11503	Disease	MFAP5	0	3.321188	
11504	Disease	ACSM3	0	3.334575	
11505	Disease	SPTLC3	0.000002	3.354069	
11506	Disease	FAM19A2	0.000002	3.37614	
11507	Disease	STOX2	0.000001	3.37804	
11508	Disease	PADI2	0.000045	3.386891	
11509	Disease	FAM55A	0.000029	3.394189	
11510	Disease	SLC3A1	0.000006	3.449694	
11511	Disease	FLJ32063	0.000043	3.452081	
11512	Disease	ADH6	0	3.466088	
11513	Disease	AFF3	0	3.468184	
11514	Disease	NAP1L2	0.000001	3.470326	
11515	Disease	HTR4	0	3.487336	
11516	Disease	XKR4	0.000002	3.514954	
11517	Disease	GREM1	0	3.545274	
11518	Disease	LOC285878	0.000005	3.556821	
11519	Disease	UNC5C	0.000002	3.564054	
11520	Disease	CEACAM21	0.000002	3.591098	
11521	Disease	SOX10	0	3.6005	
11522	Disease	MT1F	0.000012	3.615807	
11523	Disease	SAMD9L	0.000353	3.700721	
11524	Disease	ATP1A2	0.000005	3.753078	
11525	Disease	ENPP6	0	3.772164	
11526	Disease	FAM150B	0	3.794586	
11527	Disease	ANPEP	0.000236	3.883953	
11528	Disease	EGFR	0	3.938269	
11529	Disease	SEMA3E	0.000051	3.986633	
11530	Disease	C16orf89	0.000001	4.018783	
11531	Disease	LPHN3	0	4.153555	
11532	Disease	ABI3BP	0.000001	4.204579	
11533	Disease	RDH5	0	4.333768	
11534	Disease	PCSK2	0.000029	4.360948	
11535	Disease	ZBTB7C	0.000081	4.378162	
11536	Disease	FOXD3	0.000122	4.420381	
11537	Disease	TRHDE	0	4.450674	
11538	Disease	STMN2	0	4.479676	
11539	Disease	LIFR	0	4.487269	
11540	Disease	CRHBP	0	4.512409	
11541	Disease	GPM6A	0.000016	4.526743	
11542	Disease	AMPD1	0	4.595113	
11543	Disease	SCUBE2	0	4.629786	
11544	Disease	TNFRSF17	0.000092	4.731065	
11545	Disease	UGT1A1	0	4.819847	
11546	Disease	HAPLN1	0	4.884333	
11547	Disease	COL4A6	0	4.890482	
11548	Disease	ACTG2	0	4.912122	

11549	Disease	GFRA2	0	4.919921	
11550	Disease	CXCL12	0	4.977537	
11551	Disease	PTGDR	0	5.047868	
11552	Disease	SCN7A	0	5.082512	
11553	Disease	HHIP	0	5.086195	
11554	Disease	CNTN3	0	5.094855	
11555	Disease	BMP5	0	5.099133	
11556	Disease	CP	0	5.105175	
11557	Disease	CDH19	0	5.240154	
11558	Disease	BTNL8	0.000004	5.259471	
11559	Disease	SCARA5	0	5.512619	
11560	Disease	KIAA2022	0.000001	5.531687	
11561	Disease	UGT1A10	0	5.560979	
11562	Disease	DNASE1L3	0	5.794383	
11563	Disease	ANGPTL1	0.000009	5.862399	
11564	Disease	SH3GL2	0	5.893378	
11565	Disease	NPY1R	0.000001	5.973849	
11566	Disease	SPIB	0	6.04239	
11567	Disease	MT1M	0.000192	6.085901	
11568	Disease	SOSTDC1	0	6.103031	
11569	Disease	CWH43	0	6.105076	
11570	Disease	CA7	0.000041	6.213849	
11571	Disease	ADAMDEC1	0	6.26407	
11572	Disease	ADH1B	0.000002	6.364333	
11573	Disease	GLP2R	0	6.40128	
11574	Disease	UGT1A8	0	6.439246	
11575	Disease	HMGCS2	0	6.614129	
11576	Disease	MAMDC2	0	6.863978	
11577	Disease	SLC38A4	0	7.038634	
11578	Disease	MYOT	0	7.149669	
11579	Disease	USP2	0.000012	7.323947	
11580	Disease	HLA-DQA1	0.000006	7.356785	
11581	Disease	NPY6R	0	7.53012	
11582	Disease	NRXN1	0	7.559167	
11583	Disease	IGHA1	0	7.658346	
11584	Disease	ABCA8	0	7.762877	
11585	Disease	BEST4	0.00001	7.918943	
11586	Disease	FAM5C	0	8.075867	
11587	Disease	ADH1C	0	8.224757	
11588	Disease	UGT2B15	0.000005	8.436109	
11589	Disease	SLC25A34	0.000001	8.565647	
11590	Disease	HLA-DQB1	0.000025	8.791445	
11591	Disease	GCNT2	0	10.107209	
11592	Disease	OSTalpha	0.000147	10.193135	
11593	Disease	PLP1	0	11.208816	
11594	Disease	VSTM2A	0	16.865782	
11595	Dominant-Negative Mutation	CYB5A	0.000114	-497.719424	
11596	Dominant-Negative Mutation	PXDN	0.00003	-34.689751	
11597	Dominant-Negative Mutation	ZIC2	0.000437	-31.843284	
11598	Dominant-Negative Mutation	LITAF	0.000053	-26.621554	

11599	Dominant-Negative Mutation	TCEA3	0.000058	-23.553522	
11600	Dominant-Negative Mutation	EPB41L3	0.000351	-22.172662	
11601	Dominant-Negative Mutation	UNC5A	0.000607	-21.517442	
11602	Dominant-Negative Mutation	ABCB1	0.000482	-18.547662	
11603	Dominant-Negative Mutation	TLE2	0.000486	-15.588353	
11604	Dominant-Negative Mutation	PARVG	0.00005	-15.210227	
11605	Dominant-Negative Mutation	LOC100289026	0.000015	-13.35725	
11606	Dominant-Negative Mutation	NPL	0.000551	-12.480296	
11607	Dominant-Negative Mutation	GNAL	0.00003	-11.805769	
11608	Dominant-Negative Mutation	TNFRSF18	0.000187	-9.901515	
11609	Dominant-Negative Mutation	RHBDL1	0.000075	-8.661224	
11610	Dominant-Negative Mutation	TIMP3	0.000064	-8.627737	
11611	Dominant-Negative Mutation	RAC3	0.000196	-8.302583	
11612	Dominant-Negative Mutation	CIB2	0.000564	-8.107981	
11613	Dominant-Negative Mutation	KLC3	0.000382	-7.331104	
11614	Dominant-Negative Mutation	C11orf93	0.000061	-7.250724	
11615	Dominant-Negative Mutation	C11orf53	0.000115	-7.249453	
11616	Dominant-Negative Mutation	TTL	0.000202	-7.123457	
11617	Dominant-Negative Mutation	FUT1	0.000219	-6.783496	
11618	Dominant-Negative Mutation	DLX2	0.000271	-6.697201	
11619	Dominant-Negative Mutation	WNT4	0.000393	-6.517647	
11620	Dominant-Negative Mutation	SLC6A10P	0.000156	-6.252374	
11621	Dominant-Negative Mutation	GPR124	0.000137	-6.185279	
11622	Dominant-Negative Mutation	IL1F7	0.000275	-5.996226	
11623	Dominant-Negative Mutation	MRAS	0.000002	-5.970402	
11624	Dominant-Negative Mutation	CDH3	0.000564	-5.904728	
11625	Dominant-Negative Mutation	C11orf30	0.000021	-5.900891	
11626	Dominant-Negative Mutation	ZNF704	0.000142	-5.88162	
11627	Dominant-Negative Mutation	PRODH	0.000408	-5.875641	
11628	Dominant-Negative Mutation	TCF4	0.000341	-5.712788	
11629	Dominant-Negative Mutation	ABCC4	0.000219	-5.561098	
11630	Dominant-Negative Mutation	APH1B	0.000114	-5.553459	
11631	Dominant-Negative Mutation	LMF1	0.000513	-5.430435	
11632	Dominant-Negative Mutation	C4orf19	0.000367	-5.339512	
11633	Dominant-Negative Mutation	C1QTNF1	0.000228	-5.327957	
11634	Dominant-Negative Mutation	GALNT12	0.000082	-5.173536	
11635	Dominant-Negative Mutation	SORBS3	0.000259	-5.101449	
11636	Dominant-Negative Mutation	MXRA7	0.000001	-4.994552	
11637	Dominant-Negative Mutation	PDGFC	0.000001	-4.989535	
11638	Dominant-Negative Mutation	CD70	0.000555	-4.937659	
11639	Dominant-Negative Mutation	CDC42EP5	0.000176	-4.798045	
11640	Dominant-Negative Mutation	FLJ37644	0.000413	-4.712144	
11641	Dominant-Negative Mutation	SHH	0.000143	-4.677488	
11642	Dominant-Negative Mutation	RAB26	0.000042	-4.644335	
11643	Dominant-Negative Mutation	PHLDA3	0.00002	-4.586603	
11644	Dominant-Negative Mutation	ZNF358	0.000279	-4.585557	
11645	Dominant-Negative Mutation	FLJ36031	0.000142	-4.480278	
11646	Dominant-Negative Mutation	FSCN1	0.000014	-4.370552	
11647	Dominant-Negative Mutation	GXYLT2	0.000569	-4.264637	
11648	Dominant-Negative Mutation	LOC283104	0.000279	-4.155987	

11649	Dominant-Negative Mutation	FADS2	0.000551	-3.903226	
11650	Dominant-Negative Mutation	MCCC1	0.000187	-3.848696	
11651	Dominant-Negative Mutation	PROCR	0.000259	-3.835196	
11652	Dominant-Negative Mutation	FAM119A	0.000005	-3.765769	
11653	Dominant-Negative Mutation	LTBP3	0.000002	-3.619344	
11654	Dominant-Negative Mutation	FLJ41757	0.00048	-3.605058	
11655	Dominant-Negative Mutation	CNFN	0.000266	-3.585442	
11656	Dominant-Negative Mutation	ARHGAP32	0.00003	-3.550023	
11657	Dominant-Negative Mutation	LOC729680	0.000244	-3.501818	
11658	Dominant-Negative Mutation	PCCA	0.000125	-3.463857	
11659	Dominant-Negative Mutation	C19orf45	0.000612	-3.436083	
11660	Dominant-Negative Mutation	NOX1	0.000279	-3.434399	
11661	Dominant-Negative Mutation	NAGS	0.00012	-3.375294	
11662	Dominant-Negative Mutation	ATP6V1E2	0.000492	-3.370556	
11663	Dominant-Negative Mutation	AGMAT	0.000114	-3.339683	
11664	Dominant-Negative Mutation	AIFM3	0.000228	-3.295077	
11665	Dominant-Negative Mutation	HSPB1	0.00007	-3.269693	
11666	Dominant-Negative Mutation	KLF11	0.000219	-3.248634	
11667	Dominant-Negative Mutation	NEO1	0.000564	-3.231681	
11668	Dominant-Negative Mutation	KAZ	0.000187	-3.209017	
11669	Dominant-Negative Mutation	HYI	0.000441	-3.155569	
11670	Dominant-Negative Mutation	THNSL1	0.000149	-3.124434	
11671	Dominant-Negative Mutation	ATPBD4	0.000112	-3.109684	
11672	Dominant-Negative Mutation	MDK	0.000446	-3.092344	
11673	Dominant-Negative Mutation	ASPHD2	0.000341	-3.03538	
11674	Dominant-Negative Mutation	ATP13A2	0.0002	-3.004528	
11675	Dominant-Negative Mutation	CPS1	0.000002	-2.967478	
11676	Dominant-Negative Mutation	LOC255512	0.000361	-2.94404	
11677	Dominant-Negative Mutation	ARHGEF10	0.000279	-2.912243	
11678	Dominant-Negative Mutation	KRTCAP3	0.000414	-2.902283	
11679	Dominant-Negative Mutation	C9orf40	0.000602	-2.854545	
11680	Dominant-Negative Mutation	MMS19	0.000018	-2.84468	
11681	Dominant-Negative Mutation	CLN3	0.000412	-2.84375	
11682	Dominant-Negative Mutation	SH2D2A	0.000499	-2.836379	
11683	Dominant-Negative Mutation	DPH5	0.000437	-2.807949	
11684	Dominant-Negative Mutation	DNAJC4	0.000125	-2.793849	
11685	Dominant-Negative Mutation	RPL35A	0.000129	-2.785454	
11686	Dominant-Negative Mutation	CNDP2	0.00012	-2.744491	
11687	Dominant-Negative Mutation	PKIG	0.000445	-2.741006	
11688	Dominant-Negative Mutation	KLK12	0.000259	-2.740593	
11689	Dominant-Negative Mutation	ALDOC	0.000382	-2.711191	
11690	Dominant-Negative Mutation	SH3YL1	0.000002	-2.683318	
11691	Dominant-Negative Mutation	GAMT	0.000143	-2.666422	
11692	Dominant-Negative Mutation	GAS5	0.000142	-2.653625	
11693	Dominant-Negative Mutation	WDYHV1	0.000235	-2.653118	
11694	Dominant-Negative Mutation	RPL13A	0.000331	-2.645398	
11695	Dominant-Negative Mutation	HAUS4	0.000177	-2.620348	
11696	Dominant-Negative Mutation	DYRK4	0.00007	-2.615305	
11697	Dominant-Negative Mutation	SLC25A21	0.000271	-2.542119	
11698	Dominant-Negative Mutation	C9orf86	0.000367	-2.540431	

11699	Dominant-Negative Mutation	STRADB	0.000143	-2.532233	
11700	Dominant-Negative Mutation	SMARCD3	0.000128	-2.530565	
11701	Dominant-Negative Mutation	NOB1	0.000553	-2.523267	
11702	Dominant-Negative Mutation	MARVELD1	0.000568	-2.514161	
11703	Dominant-Negative Mutation	DPY30	0.000143	-2.4865	
11704	Dominant-Negative Mutation	GDF11	0.000432	-2.480144	
11705	Dominant-Negative Mutation	C4orf14	0.000437	-2.47461	
11706	Dominant-Negative Mutation	TBC1D1	0.000308	-2.448241	
11707	Dominant-Negative Mutation	SNORD104	0.000624	-2.440978	
11708	Dominant-Negative Mutation	SLC25A6	0.00034	-2.434351	
11709	Dominant-Negative Mutation	PYGL	0.000602	-2.427988	
11710	Dominant-Negative Mutation	WDR4	0.000532	-2.42595	
11711	Dominant-Negative Mutation	GART	0.000434	-2.418457	
11712	Dominant-Negative Mutation	SLC38A10	0.000553	-2.412111	
11713	Dominant-Negative Mutation	CXXC5	0.000079	-2.389303	
11714	Dominant-Negative Mutation	EIF4B	0.000312	-2.381829	
11715	Dominant-Negative Mutation	ZFAND2B	0.000489	-2.366606	
11716	Dominant-Negative Mutation	AGPHD1	0.000091	-2.36445	
11717	Dominant-Negative Mutation	KIAA0141	0.000179	-2.349644	
11718	Dominant-Negative Mutation	RBCK1	0.000486	-2.340571	
11719	Dominant-Negative Mutation	ANKHD1-EIF4	0.000437	-2.305972	
11720	Dominant-Negative Mutation	ATP5G2	0.000259	-2.290497	
11721	Dominant-Negative Mutation	HSBP1L1	0.000067	-2.281044	
11722	Dominant-Negative Mutation	ARPC3	0.000612	-2.271593	
11723	Dominant-Negative Mutation	TPD52L1	0.000315	-2.270668	
11724	Dominant-Negative Mutation	FRAT2	0.000418	-2.247018	
11725	Dominant-Negative Mutation	ARL2	0.000607	-2.246019	
11726	Dominant-Negative Mutation	SCCPDH	0.000259	-2.231617	
11727	Dominant-Negative Mutation	TIMM8B	0.000254	-2.230223	
11728	Dominant-Negative Mutation	BIRC6	0.000564	-2.20328	
11729	Dominant-Negative Mutation	GBAS	0.000367	-2.202874	
11730	Dominant-Negative Mutation	C6orf108	0.000196	-2.158936	
11731	Dominant-Negative Mutation	CHST3	0.000407	-2.132689	
11732	Dominant-Negative Mutation	SLC39A4	0.000378	-2.128275	
11733	Dominant-Negative Mutation	MIPEP	0.00007	-2.116788	
11734	Dominant-Negative Mutation	PREPL	0.000058	-2.095728	
11735	Dominant-Negative Mutation	TAF1D	0.00007	-2.095709	
11736	Dominant-Negative Mutation	SLC17A9	0.000143	-2.079349	
11737	Dominant-Negative Mutation	EXOSC1	0.000259	-2.075314	
11738	Dominant-Negative Mutation	MAGED1	0.000376	-2.073153	
11739	Dominant-Negative Mutation	C1orf57	0.000615	-2.042778	
11740	Dominant-Negative Mutation	C2orf28	0.00052	-2.041845	
11741	Dominant-Negative Mutation	ZFP62	0.000058	-2.032576	
11742	Dominant-Negative Mutation	TMEM18	0.000257	-2.026822	
11743	Dominant-Negative Mutation	HN1	0.000033	2.003492	
11744	Dominant-Negative Mutation	MYST3	0.000605	2.012837	
11745	Dominant-Negative Mutation	MBD4	0.000087	2.02786	
11746	Dominant-Negative Mutation	C17orf85	0.000287	2.030274	
11747	Dominant-Negative Mutation	SH3BGRL2	0.000259	2.031019	
11748	Dominant-Negative Mutation	TMEM50A	0.000259	2.047113	

11749	Dominant-Negative Mutation	GPR107	0.000266	2.051135	
11750	Dominant-Negative Mutation	RB1	0.000078	2.05352	
11751	Dominant-Negative Mutation	FAM122B	0.000129	2.05692	
11752	Dominant-Negative Mutation	GGCX	0.000191	2.075676	
11753	Dominant-Negative Mutation	MAPRE2	0.000392	2.088331	
11754	Dominant-Negative Mutation	UPF3A	0.000487	2.116966	
11755	Dominant-Negative Mutation	ARPC5L	0.000064	2.134362	
11756	Dominant-Negative Mutation	PSMD5	0.000018	2.135035	
11757	Dominant-Negative Mutation	YTHDC2	0.000096	2.176141	
11758	Dominant-Negative Mutation	FRG1	0.000112	2.210131	
11759	Dominant-Negative Mutation	VAPA	0.000437	2.236095	
11760	Dominant-Negative Mutation	ROD1	0.000114	2.270856	
11761	Dominant-Negative Mutation	FAM91A2	0.000614	2.287029	
11762	Dominant-Negative Mutation	HIST1H1C	0.000058	2.309695	
11763	Dominant-Negative Mutation	GAPVD1	0.000259	2.351562	
11764	Dominant-Negative Mutation	GPS2	0.000015	2.357439	
11765	Dominant-Negative Mutation	DOLPP1	0.000064	2.403751	
11766	Dominant-Negative Mutation	SUGT1	0.000413	2.430777	
11767	Dominant-Negative Mutation	VAPB	0.000259	2.433886	
11768	Dominant-Negative Mutation	ZC3H13	0.00008	2.449023	
11769	Dominant-Negative Mutation	DSG2	0.000062	2.460754	
11770	Dominant-Negative Mutation	UBE3C	0.000058	2.522976	
11771	Dominant-Negative Mutation	HOXA13	0.000143	2.550304	
11772	Dominant-Negative Mutation	ZNF271	0.000626	2.562873	
11773	Dominant-Negative Mutation	ZCCHC2	0.000382	2.590748	
11774	Dominant-Negative Mutation	TM9SF3	0.000143	2.59314	
11775	Dominant-Negative Mutation	SGCB	0.000015	2.689522	
11776	Dominant-Negative Mutation	MRPL35	0.000564	2.712121	
11777	Dominant-Negative Mutation	SLC16A1	0.000175	2.715163	
11778	Dominant-Negative Mutation	NBN	0.000065	2.803623	
11779	Dominant-Negative Mutation	HOXB7	0.000109	2.850091	
11780	Dominant-Negative Mutation	PRRG4	0.000001	2.878893	
11781	Dominant-Negative Mutation	SEPW1	0.000014	2.899693	
11782	Dominant-Negative Mutation	ASH1L	0.000058	2.991166	
11783	Dominant-Negative Mutation	ALDH3A1	0.00048	3.025803	
11784	Dominant-Negative Mutation	MSN	0.000121	3.047942	
11785	Dominant-Negative Mutation	PIGN	0.000049	3.062276	
11786	Dominant-Negative Mutation	DNAJB6	0.00007	3.07301	
11787	Dominant-Negative Mutation	ARGLU1	0.000279	3.146774	
11788	Dominant-Negative Mutation	TTC39C	0.00052	3.154439	
11789	Dominant-Negative Mutation	ING2	0.000177	3.185976	
11790	Dominant-Negative Mutation	MECOM	0.000112	3.198311	
11791	Dominant-Negative Mutation	DSC2	0.000018	3.231242	
11792	Dominant-Negative Mutation	TXNDC9	0.000143	3.328385	
11793	Dominant-Negative Mutation	PIK3C3	0.000003	3.343665	
11794	Dominant-Negative Mutation	ATXN1	0.000166	3.387009	
11795	Dominant-Negative Mutation	TNRC18	0.000082	3.412363	
11796	Dominant-Negative Mutation	RHOU	0.000078	3.614124	
11797	Dominant-Negative Mutation	ESYT2	0.000458	3.665878	
11798	Dominant-Negative Mutation	INO80C	0.000135	3.731187	

11799	Dominant-Negative Mutation	TUG1	0.000015	3.752775	
11800	Dominant-Negative Mutation	RTTN	0.000002	3.816143	
11801	Dominant-Negative Mutation	GEM	0.000143	3.9926	
11802	Dominant-Negative Mutation	TPRXL	0.00018	4.054795	
11803	Dominant-Negative Mutation	LOC100505875	0.000492	4.152001	
11804	Dominant-Negative Mutation	FAM50B	0.000018	4.335119	
11805	Dominant-Negative Mutation	PTGR1	0.000001	4.486203	
11806	Dominant-Negative Mutation	UGT1A6	0.000406	4.496336	
11807	Dominant-Negative Mutation	SYNJ2	0.000382	4.635881	
11808	Dominant-Negative Mutation	LIMCH1	0.000303	4.710667	
11809	Dominant-Negative Mutation	ZNF558	0.000114	5.356884	
11810	Dominant-Negative Mutation	ZNF426	0.000112	5.90991	
11811	Dominant-Negative Mutation	SH3TC2	0.000208	6.079137	
11812	Dominant-Negative Mutation	SPIN3	0.000147	7.489164	
11813	Dominant-Negative Mutation	ZNF204P	0.000614	8.492386	
11814	Dominant-Negative Mutation	BAT3	0.000087	9.181054	
11815	Dominant-Negative Mutation	PRUNE2	0.000015	9.361624	
11816	Dominant-Negative Mutation	RASA1	0.00018	9.621369	
11817	Dominant-Negative Mutation	MT1M	0.000016	11.080972	
11818	Dominant-Negative Mutation	FAM129A	0.000243	14.358025	
11819	Dominant-Negative Mutation	SERPINB5	0.00007	14.794114	
11820	Dominant-Negative Mutation	AKAP12	0.000382	19.882979	
11821	Dominant-Negative Mutation	REG1A	0.000149	36.576833	
11822	Ductal Breast Carcinoma	ENOSF1	0.04975	-2.163659	
11823	Ductal Breast Carcinoma	LOC728448	0.004106	2.432727	
11824	Ductal Breast Carcinoma	TCERG1	0.047633	2.472226	
11825	Ductal Carcinoma	ENOSF1	0.04975	-2.163659	
11826	Ductal Carcinoma	LOC728448	0.004106	2.432727	
11827	Ductal Carcinoma	TCERG1	0.047633	2.472226	
11828	Dysplasia	CLCA4	0.000074	-84.873382	
11829	Dysplasia	SPINK5	0.000031	-32.239686	
11830	Dysplasia	ACHE	0.000024	-23.943237	
11831	Dysplasia	SLC25A34	0.000046	-21.203959	
11832	Dysplasia	ZG16	0.000004	-20.112805	
11833	Dysplasia	ANO5	0.000073	-19.197245	
11834	Dysplasia	DHRS9	0.000068	-17.872567	
11835	Dysplasia	OSTBETA	0.000025	-17.297428	
11836	Dysplasia	DPEP2	0.000041	-15.654621	
11837	Dysplasia	OLFM1	0	-15.232331	
11838	Dysplasia	STMN2	0.000003	-14.933347	
11839	Dysplasia	BTNL8	0.000017	-14.197677	
11840	Dysplasia	SLC15A1	0.000019	-13.109778	
11841	Dysplasia	CYBRD1	0.000011	-11.498188	
11842	Dysplasia	PYY	0	-11.401911	
11843	Dysplasia	CRYBA2	0.000001	-11.209007	
11844	Dysplasia	C11orf86	0.000005	-11.074642	
11845	Dysplasia	CEACAM7	0.000002	-11.049524	
11846	Dysplasia	LGALS2	0.000022	-10.968017	
11847	Dysplasia	CD177	0.000001	-10.95502	
11848	Dysplasia	MALL	0.000001	-10.58266	

11849	Dysplasia	CA2	0.000003	-10.158465	
11850	Dysplasia	GUCA2B	0.000001	-9.894457	
11851	Dysplasia	APBB1	0.000007	-9.770749	
11852	Dysplasia	TRPM6	0	-9.613446	
11853	Dysplasia	EPB41L3	0.000002	-9.566043	
11854	Dysplasia	TCF21	0	-9.193144	
11855	Dysplasia	HPGD	0.00001	-9.155656	
11856	Dysplasia	TAF13	0.000076	-8.867731	
11857	Dysplasia	CDKN2B-AS	0.000002	-8.855571	
11858	Dysplasia	CDA	0.000052	-7.974413	
11859	Dysplasia	CCDC102B	0.000001	-7.700813	
11860	Dysplasia	SDCBP2	0.000007	-7.428168	
11861	Dysplasia	ZNF568	0.000024	-7.327563	
11862	Dysplasia	GUCA2A	0	-7.294218	
11863	Dysplasia	AQP8	0.000004	-7.284831	
11864	Dysplasia	PLEKHG2	0.000054	-7.265051	
11865	Dysplasia	HSD17B2	0.000023	-7.156495	
11866	Dysplasia	FEV	0.000002	-7.139037	
11867	Dysplasia	FLJ35700	0.000058	-7.106864	
11868	Dysplasia	GDPD2	0.000029	-7.091095	
11869	Dysplasia	TPH1	0.000003	-7.005897	
11870	Dysplasia	PKNOX2	0.000046	-6.995376	
11871	Dysplasia	RAB9B	0.000039	-6.901206	
11872	Dysplasia	ENPP6	0.000082	-6.893884	
11873	Dysplasia	BEST4	0.000005	-6.576351	
11874	Dysplasia	CELA3A	0.000054	-6.497474	
11875	Dysplasia	HRCT1	0.000044	-6.487225	
11876	Dysplasia	CHGB	0.000032	-6.347379	
11877	Dysplasia	SLCO2B1	0.000011	-6.318094	
11878	Dysplasia	PRKAA2	0.000019	-6.127948	
11879	Dysplasia	GPAT2	0.000035	-6.11638	
11880	Dysplasia	CA7	0	-6.05505	
11881	Dysplasia	DSEL	0.000007	-5.961644	
11882	Dysplasia	CELA3B	0.000055	-5.881904	
11883	Dysplasia	CTSW	0.00006	-5.831313	
11884	Dysplasia	SOSTDC1	0.000007	-5.715365	
11885	Dysplasia	CLECL1	0.000024	-5.673399	
11886	Dysplasia	HTR4	0.000024	-5.522219	
11887	Dysplasia	IL16	0.000032	-5.408401	
11888	Dysplasia	SLC6A13	0.000054	-5.392697	
11889	Dysplasia	MESP2	0.000034	-5.381215	
11890	Dysplasia	NR5A2	0.000001	-5.359415	
11891	Dysplasia	FAM150B	0.000003	-5.356415	
11892	Dysplasia	FIGN	0.000032	-5.328371	
11893	Dysplasia	PAPPA	0.000031	-5.221384	
11894	Dysplasia	FN1	0.000045	-5.179577	
11895	Dysplasia	LRRN2	0.000004	-5.114688	
11896	Dysplasia	GPRC5B	0.000054	-5.056463	
11897	Dysplasia	IGF1	0	-5.019269	
11898	Dysplasia	KIAA2022	0.000001	-4.891469	

11899	Dysplasia	TMEM72	0.000001	-4.884725	
11900	Dysplasia	INSM1	0	-4.88269	
11901	Dysplasia	ABCA8	0.000001	-4.762877	
11902	Dysplasia	SECTM1	0.000043	-4.703083	
11903	Dysplasia	CLIP3	0.000019	-4.692329	
11904	Dysplasia	HHLA2	0.000005	-4.674398	
11905	Dysplasia	CD36	0.000005	-4.6646	
11906	Dysplasia	SCARA5	0.000025	-4.659075	
11907	Dysplasia	SCG2	0	-4.649106	
11908	Dysplasia	RDX	0.000005	-4.580242	
11909	Dysplasia	HAPLN1	0.000008	-4.558387	
11910	Dysplasia	DPP4	0.000066	-4.500848	
11911	Dysplasia	PCDH9	0	-4.498007	
11912	Dysplasia	MYOT	0.000002	-4.483537	
11913	Dysplasia	FLJ36848	0.000032	-4.481406	
11914	Dysplasia	TUSC3	0.000001	-4.451097	
11915	Dysplasia	CD1D	0.000006	-4.435071	
11916	Dysplasia	FAM132A	0.000022	-4.373596	
11917	Dysplasia	C11orf96	0.000082	-4.344237	
11918	Dysplasia	HMOX1	0.000006	-4.31922	
11919	Dysplasia	SPIB	0	-4.317744	
11920	Dysplasia	PRKAR2B	0.000005	-4.287648	
11921	Dysplasia	GNG2	0.000015	-4.266163	
11922	Dysplasia	PCK1	0	-4.260282	
11923	Dysplasia	HIST1H1C	0.000005	-4.183722	
11924	Dysplasia	GPM6B	0	-4.161942	
11925	Dysplasia	PLP1	0.000004	-4.115264	
11926	Dysplasia	RGS2	0.000028	-4.115168	
11927	Dysplasia	AXL	0.000036	-4.105729	
11928	Dysplasia	SDC3	0.000082	-4.088098	
11929	Dysplasia	MEIS1	0	-4.074388	
11930	Dysplasia	CD48	0.000082	-4.071704	
11931	Dysplasia	SEMA6A	0.000004	-4.015729	
11932	Dysplasia	SLC9A9	0.000001	-3.986887	
11933	Dysplasia	GPX3	0.000015	-3.976098	
11934	Dysplasia	DPT	0	-3.964112	
11935	Dysplasia	RARRES2	0.000004	-3.962436	
11936	Dysplasia	MPEG1	0.000003	-3.942707	
11937	Dysplasia	SCN7A	0.000001	-3.937932	
11938	Dysplasia	LOX	0.000043	-3.915415	
11939	Dysplasia	SPARCL1	0.000024	-3.888472	
11940	Dysplasia	LIFR	0.000039	-3.882601	
11941	Dysplasia	FGL2	0.000025	-3.87092	
11942	Dysplasia	GNG11	0.000054	-3.848307	
11943	Dysplasia	ECM2	0.00009	-3.801265	
11944	Dysplasia	HDGFRP3	0.00009	-3.781041	
11945	Dysplasia	XKR4	0.000001	-3.770258	
11946	Dysplasia	NRXN1	0.000002	-3.768761	
11947	Dysplasia	SEMA3E	0.000001	-3.749221	
11948	Dysplasia	LY9	0.000072	-3.719374	

11949	Dysplasia	EVI2B	0.000071	-3.698574	
11950	Dysplasia	ZNF75A	0	-3.693634	
11951	Dysplasia	HIGD1A	0.000009	-3.688449	
11952	Dysplasia	CPM	0.000065	-3.663781	
11953	Dysplasia	RHOF	0.000004	-3.660494	
11954	Dysplasia	NEU4	0.000076	-3.65104	
11955	Dysplasia	MCC	0.000021	-3.64725	
11956	Dysplasia	HMCN1	0.000082	-3.643733	
11957	Dysplasia	MRC1	0.000037	-3.622318	
11958	Dysplasia	LOC646701	0.000082	-3.621953	
11959	Dysplasia	TPK1	0.000016	-3.612082	
11960	Dysplasia	KIF16B	0.000001	-3.576707	
11961	Dysplasia	CLIC5	0.000033	-3.544831	
11962	Dysplasia	CD163	0.000054	-3.537225	
11963	Dysplasia	NEURL3	0.000006	-3.521461	
11964	Dysplasia	VCAM1	0.000091	-3.475122	
11965	Dysplasia	IL1R1	0.000025	-3.461987	
11966	Dysplasia	CLEC10A	0.000005	-3.459407	
11967	Dysplasia	HLA-DRB1	0.00008	-3.452479	
11968	Dysplasia	ESR1	0.000054	-3.434047	
11969	Dysplasia	AHRR	0.000075	-3.424512	
11970	Dysplasia	CD14	0.000013	-3.41472	
11971	Dysplasia	MFAP4	0	-3.393558	
11972	Dysplasia	AIF1	0.000082	-3.388451	
11973	Dysplasia	SLC17A4	0.000002	-3.387147	
11974	Dysplasia	GIMAP6	0.000036	-3.387008	
11975	Dysplasia	KCTD12	0.00001	-3.361388	
11976	Dysplasia	SRPX	0.000007	-3.357829	
11977	Dysplasia	FBLN1	0	-3.352787	
11978	Dysplasia	SMPDL3A	0.000025	-3.352141	
11979	Dysplasia	NOVA1	0.000072	-3.351954	
11980	Dysplasia	PAMR1	0.000002	-3.31603	
11981	Dysplasia	SAMD9	0.000058	-3.312558	
11982	Dysplasia	RCAN1	0.000031	-3.300913	
11983	Dysplasia	UPP1	0.000072	-3.293296	
11984	Dysplasia	SLC20A1	0.000031	-3.292437	
11985	Dysplasia	NCF4	0.000022	-3.282698	
11986	Dysplasia	AKAP2	0.000068	-3.231506	
11987	Dysplasia	CMAH	0	-3.208679	
11988	Dysplasia	MEF2C	0.000019	-3.189628	
11989	Dysplasia	CCNYL1	0.000005	-3.18485	
11990	Dysplasia	LOC100508909	0.000004	-3.147079	
11991	Dysplasia	HLF	0.000001	-3.135844	
11992	Dysplasia	CNTN1	0.000002	-3.122925	
11993	Dysplasia	DNASE1L3	0.000027	-3.119775	
11994	Dysplasia	PHLPP2	0.000001	-3.096838	
11995	Dysplasia	NKX2-3	0.000074	-3.096098	
11996	Dysplasia	SLC46A3	0	-3.09383	
11997	Dysplasia	CXCL12	0.000005	-3.092806	
11998	Dysplasia	MMP2	0.000043	-3.089364	

11999	Dysplasia	GPC6	0.000002	-3.072173	
12000	Dysplasia	CCL14	0.000017	-3.067341	
12001	Dysplasia	MAF	0.000048	-3.067136	
12002	Dysplasia	PDGFRA	0	-3.06471	
12003	Dysplasia	TRAF3IP3	0.000031	-3.059537	
12004	Dysplasia	CCL28	0.000005	-3.053933	
12005	Dysplasia	HIP1	0.000051	-3.001035	
12006	Dysplasia	MAFB	0.000031	-2.981246	
12007	Dysplasia	AKT3	0.00001	-2.971808	
12008	Dysplasia	HPSE	0.000018	-2.95219	
12009	Dysplasia	GJB2	0.000007	-2.946941	
12010	Dysplasia	LOC100506621	0.000001	-2.922869	
12011	Dysplasia	SOX10	0.000003	-2.909432	
12012	Dysplasia	ELOVL6	0.000025	-2.895058	
12013	Dysplasia	RUNDC3B	0.000008	-2.875203	
12014	Dysplasia	CLCN2	0.000002	-2.858268	
12015	Dysplasia	RAB34	0.000006	-2.849977	
12016	Dysplasia	SGK1	0	-2.849016	
12017	Dysplasia	ITGA8	0.000005	-2.847531	
12018	Dysplasia	SCD5	0.000011	-2.844648	
12019	Dysplasia	STAB1	0	-2.839099	
12020	Dysplasia	FOXF1	0.000035	-2.838865	
12021	Dysplasia	ZNF677	0	-2.838379	
12022	Dysplasia	SLC36A1	0.000001	-2.838295	
12023	Dysplasia	TNXA	0	-2.832863	
12024	Dysplasia	CSF1R	0.000019	-2.829504	
12025	Dysplasia	CTSZ	0.000004	-2.829326	
12026	Dysplasia	AHCYL2	0.000019	-2.820278	
12027	Dysplasia	TCF4	0.000003	-2.812337	
12028	Dysplasia	UNC5C	0	-2.801776	
12029	Dysplasia	MS4A7	0.000046	-2.800661	
12030	Dysplasia	PPP1R16B	0.000034	-2.800474	
12031	Dysplasia	GGTA1	0.000004	-2.796653	
12032	Dysplasia	JAZF1	0.000002	-2.794106	
12033	Dysplasia	RGL1	0.000069	-2.783913	
12034	Dysplasia	CNRIP1	0.000031	-2.778322	
12035	Dysplasia	PTGER3	0.000005	-2.766417	
12036	Dysplasia	TNXB	0	-2.759854	
12037	Dysplasia	FAM101B	0.000016	-2.705595	
12038	Dysplasia	RELL1	0.000001	-2.704754	
12039	Dysplasia	ADCY2	0	-2.684973	
12040	Dysplasia	RASGRP3	0.000007	-2.665974	
12041	Dysplasia	JAM3	0.000012	-2.658057	
12042	Dysplasia	FAM38B	0	-2.654328	
12043	Dysplasia	SMPD1	0.000052	-2.647116	
12044	Dysplasia	ARMCX1	0.000019	-2.616212	
12045	Dysplasia	ADAP2	0.000031	-2.598393	
12046	Dysplasia	PLOD2	0	-2.597071	
12047	Dysplasia	PEG3	0.000003	-2.596887	
12048	Dysplasia	COL28A1	0.000001	-2.595261	

12049	Dysplasia	PRDX6	0.000015	-2.590845	
12050	Dysplasia	ANTXR1	0.000039	-2.588072	
12051	Dysplasia	GIMAP1	0.000037	-2.576192	
12052	Dysplasia	ABCA6	0.000001	-2.55698	
12053	Dysplasia	MYO5A	0.000048	-2.556934	
12054	Dysplasia	FOLR2	0.000043	-2.529423	
12055	Dysplasia	FOXF2	0.000023	-2.529215	
12056	Dysplasia	PTN	0	-2.525943	
12057	Dysplasia	PCDH18	0.000016	-2.525923	
12058	Dysplasia	SLC30A4	0.000003	-2.513062	
12059	Dysplasia	CD84	0.000019	-2.50676	
12060	Dysplasia	KRT20	0.000067	-2.501505	
12061	Dysplasia	RELN	0.000002	-2.493594	
12062	Dysplasia	CCDC80	0.000001	-2.493379	
12063	Dysplasia	LAPTM5	0.000074	-2.486932	
12064	Dysplasia	TLCD2	0.000008	-2.481418	
12065	Dysplasia	SALL1	0.000009	-2.477997	
12066	Dysplasia	GLTP	0.000002	-2.471048	
12067	Dysplasia	FZD8	0.000042	-2.467017	
12068	Dysplasia	TSPAN3	0.000007	-2.46009	
12069	Dysplasia	CYS1	0.000004	-2.42959	
12070	Dysplasia	TTC22	0.000079	-2.427972	
12071	Dysplasia	SHE	0.000029	-2.413364	
12072	Dysplasia	ZCCHC24	0.000026	-2.410483	
12073	Dysplasia	OLFML2A	0.000002	-2.402962	
12074	Dysplasia	SALL2	0.000034	-2.388978	
12075	Dysplasia	RBMS3	0.000048	-2.379696	
12076	Dysplasia	ARHGEF6	0.000017	-2.377721	
12077	Dysplasia	CACNA2D1	0.000005	-2.366345	
12078	Dysplasia	GAS7	0	-2.331612	
12079	Dysplasia	FXYD1	0.000004	-2.320937	
12080	Dysplasia	HSPC159	0.000034	-2.315064	
12081	Dysplasia	SLC2A13	0.000036	-2.311299	
12082	Dysplasia	TMEM133	0.000005	-2.302401	
12083	Dysplasia	CLEC3B	0.000002	-2.301045	
12084	Dysplasia	PPP1R3C	0.000007	-2.298722	
12085	Dysplasia	HDAC9	0.000002	-2.293507	
12086	Dysplasia	MIER3	0.000002	-2.292537	
12087	Dysplasia	ITPKA	0.000008	-2.28995	
12088	Dysplasia	SPPL2A	0.000001	-2.288713	
12089	Dysplasia	IGSF9	0.000003	-2.288617	
12090	Dysplasia	NAP1L3	0.000007	-2.269711	
12091	Dysplasia	LOC100509683	0.000054	-2.268836	
12092	Dysplasia	ITM2C	0.000036	-2.26781	
12093	Dysplasia	PDE2A	0	-2.25433	
12094	Dysplasia	THBS1	0.000001	-2.250588	
12095	Dysplasia	NAAA	0.000004	-2.241022	
12096	Dysplasia	SYNC	0	-2.233029	
12097	Dysplasia	ZNF542	0.000001	-2.229413	
12098	Dysplasia	ANKRD6	0.000007	-2.224363	

12099	Dysplasia	ST3GAL6	0.000007	-2.217513	
12100	Dysplasia	C15orf48	0.000019	-2.215116	
12101	Dysplasia	OLFML1	0.000001	-2.200346	
12102	Dysplasia	TSPAN4	0.000036	-2.182007	
12103	Dysplasia	SLCO2A1	0.000048	-2.17587	
12104	Dysplasia	XIAP	0.00003	-2.166723	
12105	Dysplasia	APOE	0.000002	-2.157823	
12106	Dysplasia	OSTM1	0.000001	-2.15371	
12107	Dysplasia	C2orf7	0.000003	-2.142112	
12108	Dysplasia	UBE2E2	0	-2.140143	
12109	Dysplasia	BCAP29	0.000079	-2.114582	
12110	Dysplasia	PIP5K1B	0.000059	-2.11295	
12111	Dysplasia	PER3	0.000003	-2.111868	
12112	Dysplasia	CFH	0.000001	-2.107982	
12113	Dysplasia	PDE1A	0.000005	-2.095001	
12114	Dysplasia	8-Sep	0	-2.09136	
12115	Dysplasia	FAM126A	0.000001	-2.078326	
12116	Dysplasia	GLI3	0	-2.077675	
12117	Dysplasia	BCL2L11	0.000002	-2.076004	
12118	Dysplasia	MYLK	0.000001	-2.068345	
12119	Dysplasia	FUCA1	0.000083	-2.063597	
12120	Dysplasia	PDLIM2	0	-2.062956	
12121	Dysplasia	CNNM2	0	-2.062836	
12122	Dysplasia	CES2	0.000004	-2.059435	
12123	Dysplasia	PEX26	0.000003	-2.0586	
12124	Dysplasia	MOBKL2B	0.000001	-2.057892	
12125	Dysplasia	GPD2	0.000027	-2.05634	
12126	Dysplasia	CNNM4	0.000002	-2.047069	
12127	Dysplasia	UGP2	0.000015	-2.045593	
12128	Dysplasia	ARNTL	0.000073	-2.04229	
12129	Dysplasia	MYO15B	0.000082	-2.0422	
12130	Dysplasia	TNS1	0.000002	-2.039982	
12131	Dysplasia	FAS	0.000076	-2.038597	
12132	Dysplasia	FXYD6	0	-2.037228	
12133	Dysplasia	ANKRD13A	0.000007	-2.030677	
12134	Dysplasia	CYGB	0	-2.028863	
12135	Dysplasia	ZNF304	0	-2.023245	
12136	Dysplasia	CPEB3	0.000056	-2.001948	
12137	Dysplasia	FERMT1	0.000003	2.006067	
12138	Dysplasia	STXBP6	0	2.00751	
12139	Dysplasia	ME3	0.000002	2.043797	
12140	Dysplasia	APIP	0.000003	2.055785	
12141	Dysplasia	S100P	0.000008	2.06215	
12142	Dysplasia	NEBL	0.000002	2.150993	
12143	Dysplasia	C19orf45	0.000004	2.234818	
12144	Dysplasia	BACE2	0	2.270814	
12145	Dysplasia	CCDC123	0.00005	2.300184	
12146	Dysplasia	SAMD5	0.000007	2.332025	
12147	Dysplasia	QTRT1	0.000082	2.345266	
12148	Dysplasia	RNF183	0.000008	2.570806	

12149	Dysplasia	CRB2	0	2.774161	
12150	Dysplasia	AXIN2	0.000003	2.846909	
12151	Dysplasia	HOXA10	0.000013	2.919364	
12152	Dysplasia	IGFBP2	0.000006	3.15496	
12153	Dysplasia	ASB4	0.000032	3.317859	
12154	Dysplasia	ETV4	0	3.658835	
12155	Electromagnetic Energy	REG1B	0.000008	-9.377253	
12156	Electromagnetic Energy	CLCA4	0	-7.004552	
12157	Electromagnetic Energy	MS4A12	0.000013	-6.113246	
12158	Electromagnetic Energy	CEACAM7	0.000004	-4.815933	
12159	Electromagnetic Energy	SI	0.000013	-4.602946	
12160	Electromagnetic Energy	SLC26A3	0.000001	-4.120587	
12161	Electromagnetic Energy	ASCL2	0.000004	-4.090253	
12162	Electromagnetic Energy	KLK10	0.000074	-3.447447	
12163	Electromagnetic Energy	C10orf99	0	-3.417173	
12164	Electromagnetic Energy	CA4	0.000027	-3.322223	
12165	Electromagnetic Energy	PI3	0.000053	-3.20333	
12166	Electromagnetic Energy	DMBT1	0.000005	-3.158941	
12167	Electromagnetic Energy	BTNL8	0.00003	-3.143397	
12168	Electromagnetic Energy	GAD1	0	-3.131845	
12169	Electromagnetic Energy	GRM8	0.000021	-3.036762	
12170	Electromagnetic Energy	SEMG1	0.000096	-3.02247	
12171	Electromagnetic Energy	GPA33	0.000133	-2.970294	
12172	Electromagnetic Energy	ETV4	0	-2.956175	
12173	Electromagnetic Energy	HOXA10	0.000001	-2.927409	
12174	Electromagnetic Energy	PPP1R1B	0.000003	-2.925221	
12175	Electromagnetic Energy	PLAC8	0	-2.869226	
12176	Electromagnetic Energy	GCNT3	0.000031	-2.818528	
12177	Electromagnetic Energy	HSD11B2	0.000006	-2.758098	
12178	Electromagnetic Energy	ACE2	0	-2.703972	
12179	Electromagnetic Energy	AFAP1-AS	0	-2.656585	
12180	Electromagnetic Energy	GAL3ST1	0	-2.642896	
12181	Electromagnetic Energy	GJB5	0	-2.619622	
12182	Electromagnetic Energy	SLC26A2	0.000004	-2.572697	
12183	Electromagnetic Energy	ACSL6	0	-2.558908	
12184	Electromagnetic Energy	CBLC	0.000087	-2.553178	
12185	Electromagnetic Energy	LRRC19	0.000013	-2.544712	
12186	Electromagnetic Energy	OSTBETA	0.000016	-2.541604	
12187	Electromagnetic Energy	PRR15	0	-2.516785	
12188	Electromagnetic Energy	HR	0.000005	-2.516208	
12189	Electromagnetic Energy	PLA2G10	0.000004	-2.461758	
12190	Electromagnetic Energy	SLC13A2	0.000004	-2.460392	
12191	Electromagnetic Energy	UGT8	0.000131	-2.430706	
12192	Electromagnetic Energy	HHLA2	0.000001	-2.426586	
12193	Electromagnetic Energy	NOX1	0.000123	-2.424593	
12194	Electromagnetic Energy	EPN3	0.000101	-2.407784	
12195	Electromagnetic Energy	PRSS3	0.000011	-2.399846	
12196	Electromagnetic Energy	TRPM6	0.000047	-2.38064	
12197	Electromagnetic Energy	ROBO2	0.000131	-2.373002	
12198	Electromagnetic Energy	CDX1	0.000121	-2.362139	

12199	Electromagnetic Energy	XK	0.000009	-2.360366	
12200	Electromagnetic Energy	PLEKHA6	0.000038	-2.355337	
12201	Electromagnetic Energy	HPDL	0.000137	-2.329338	
12202	Electromagnetic Energy	IHH	0.000002	-2.327418	
12203	Electromagnetic Energy	GPX2	0.00004	-2.31021	
12204	Electromagnetic Energy	CLDN2	0.000009	-2.291243	
12205	Electromagnetic Energy	RAPGEFL1	0.000308	-2.288837	
12206	Electromagnetic Energy	HOXA13	0.000002	-2.285029	
12207	Electromagnetic Energy	ZG16B	0.000092	-2.280213	
12208	Electromagnetic Energy	CKMT1A	0.000115	-2.276833	
12209	Electromagnetic Energy	PKP2	0.000008	-2.26335	
12210	Electromagnetic Energy	ITPKA	0.000006	-2.261717	
12211	Electromagnetic Energy	PRSS1	0	-2.261085	
12212	Electromagnetic Energy	TMEM54	0.000032	-2.249464	
12213	Electromagnetic Energy	CDH17	0.00014	-2.241173	
12214	Electromagnetic Energy	PLEK2	0.000022	-2.237062	
12215	Electromagnetic Energy	CDC45	0.000219	-2.210318	
12216	Electromagnetic Energy	GIPC2	0.0001	-2.203936	
12217	Electromagnetic Energy	NOS2	0.000008	-2.193151	
12218	Electromagnetic Energy	SCIN	0	-2.191931	
12219	Electromagnetic Energy	SELENBP1	0.000183	-2.187424	
12220	Electromagnetic Energy	LOC100505633	0.000266	-2.184313	
12221	Electromagnetic Energy	HOXA5	0	-2.174766	
12222	Electromagnetic Energy	PRSS12	0.000066	-2.167532	
12223	Electromagnetic Energy	SFN	0.000168	-2.167506	
12224	Electromagnetic Energy	GGT6	0.00013	-2.164095	
12225	Electromagnetic Energy	ARHGAP8	0.000121	-2.145479	
12226	Electromagnetic Energy	DSC3	0.000105	-2.13763	
12227	Electromagnetic Energy	LOC400573	0.000006	-2.13572	
12228	Electromagnetic Energy	LRRC31	0.000138	-2.12492	
12229	Electromagnetic Energy	LOC729680	0.000005	-2.124049	
12230	Electromagnetic Energy	PITX1	0.000128	-2.113922	
12231	Electromagnetic Energy	C16orf53	0.000145	-2.110319	
12232	Electromagnetic Energy	PLS1	0.000089	-2.108095	
12233	Electromagnetic Energy	TPX2	0.000007	-2.099725	
12234	Electromagnetic Energy	STYK1	0.000053	-2.099148	
12235	Electromagnetic Energy	ADAP1	0	-2.09866	
12236	Electromagnetic Energy	BCL2L15	0.000013	-2.096901	
12237	Electromagnetic Energy	ESRP1	0.000046	-2.096632	
12238	Electromagnetic Energy	ANLN	0.000004	-2.091956	
12239	Electromagnetic Energy	CEACAM1	0.000011	-2.086042	
12240	Electromagnetic Energy	YBX2	0.000065	-2.081157	
12241	Electromagnetic Energy	HKDC1	0.000075	-2.080243	
12242	Electromagnetic Energy	CDCA3	0.000001	-2.079912	
12243	Electromagnetic Energy	BCL2L14	0.000213	-2.078232	
12244	Electromagnetic Energy	NCAPH	0.000073	-2.072185	
12245	Electromagnetic Energy	ARHGAP32	0.000001	-2.070218	
12246	Electromagnetic Energy	C15orf48	0.000112	-2.06679	
12247	Electromagnetic Energy	FXYD3	0	-2.064529	
12248	Electromagnetic Energy	PLK1	0.000002	-2.064204	

12249	Electromagnetic Energy	TTC22	0.000212	-2.060377	
12250	Electromagnetic Energy	GRHL2	0.00002	-2.05978	
12251	Electromagnetic Energy	KLF5	0.000151	-2.049687	
12252	Electromagnetic Energy	TRIM2	0.000011	-2.045956	
12253	Electromagnetic Energy	SATB2	0.000201	-2.042722	
12254	Electromagnetic Energy	LAD1	0.000023	-2.040375	
12255	Electromagnetic Energy	C1orf106	0.000021	-2.038333	
12256	Electromagnetic Energy	MYO1A	0.000026	-2.032036	
12257	Electromagnetic Energy	KBTBD11	0.00003	-2.031752	
12258	Electromagnetic Energy	PRSS8	0.000014	-2.015174	
12259	Electromagnetic Energy	CDCA7	0.000114	-2.01385	
12260	Electromagnetic Energy	KIAA1244	0.000241	-2.011288	
12261	Electromagnetic Energy	FERMT1	0.000065	-2.002065	
12262	Electromagnetic Energy	NRARP	0.000219	-2.001723	
12263	Encounter due to therapy	LOC158257	0	2.23664	
12264	Encounter due to therapy	RPS11	0	2.266291	
12265	Esophagus normal	MVP	0.000004	3.215099	
12266	Esophagus normal	TOR1AIP2	0	3.858727	
12267	Esophagus normal	ANKRD11	0	3.92099	
12268	Esophagus normal	KRT37	0.000086	4.070831	
12269	Esophagus normal	ZNF133	0	15.289034	
12270	Fecal analysis procedure	TNNT1	0.018468	-3.240378	
12271	Fecal analysis procedure	KRT7	0.027034	-3.045607	
12272	Fecal analysis procedure	GALNT14	0.041388	-2.245997	
12273	Fecal analysis procedure	KLHL14	0.038715	-2.111714	
12274	Fecal analysis procedure	MAP2	0.02749	-2.035409	
12275	Fecal analysis procedure	TFAP2A	0.022418	-2.009505	
12276	Fecal analysis procedure	PRAC	0.010821	2.388571	
12277	Finding	ACBD3	0.007851	-3.97229	
12278	Finding	KRTAP5-5	0.007148	-3.883056	
12279	Finding	HLA-C	0.006669	-3.673555	
12280	Finding	ROBO2	0.00448	-3.662034	
12281	Finding	HLA-DQB1	0.006388	-2.89817	
12282	Finding	NCOA4	0.005756	-2.70302	
12283	Finding	PALLD	0.005369	-2.534954	
12284	Finding	CHUK	0.00462	-2.466915	
12285	Follicular thyroid carcinoma	TPST2	0.000258	2.332694	
12286	Genotype determination	AASS	0.010138	-23.946618	
12287	Genotype determination	CSTA	0.006426	-8.88579	
12288	Genotype determination	EPHX2	0.007544	-7.162683	
12289	Genotype determination	SERPINA5	0.005197	-7.134192	
12290	Genotype determination	MXD3	0.005664	-7.132956	
12291	Genotype determination	ANK1	0.014933	-7.057954	
12292	Genotype determination	SPARC	0.005664	-5.955613	
12293	Genotype determination	JMJD7	0.013483	-5.71994	
12294	Genotype determination	SLC7A11	0.004009	-5.464161	
12295	Genotype determination	C9orf3	0.005581	-4.750239	
12296	Genotype determination	PCSK9	0.013483	-4.566219	
12297	Genotype determination	INSIG1	0.003757	-4.271521	
12298	Genotype determination	SLC16A6	0.009601	-4.260433	

12299	Genotype determination	DNM3	0.003938	-4.000693	
12300	Genotype determination	SLC1A3	0.003757	-3.704426	
12301	Genotype determination	STARD4	0.003757	-3.664204	
12302	Genotype determination	NES	0.020473	-3.647101	
12303	Genotype determination	VSNL1	0.004402	-3.632594	
12304	Genotype determination	NR1D1	0.019914	-3.591285	
12305	Genotype determination	ZNF238	0.014933	-3.507031	
12306	Genotype determination	CCDC88B	0.009162	-3.503993	
12307	Genotype determination	CCNG2	0.014919	-3.333991	
12308	Genotype determination	UCA1	0.003757	-3.327641	
12309	Genotype determination	LIPG	0.0077	-3.293223	
12310	Genotype determination	NCRNA00182	0.016461	-3.214847	
12311	Genotype determination	LOC285735	0.007699	-3.014147	
12312	Genotype determination	NEURL1B	0.014096	-3.006844	
12313	Genotype determination	PHF20	0.020375	-2.964934	
12314	Genotype determination	HSPB8	0.019557	-2.864434	
12315	Genotype determination	OGFRL1	0.01184	-2.844648	
12316	Genotype determination	CHAC1	0.014002	-2.843169	
12317	Genotype determination	ARRDC4	0.007254	-2.811812	
12318	Genotype determination	C14orf139	0.013483	-2.792873	
12319	Genotype determination	DDIT4	0.005581	-2.721685	
12320	Genotype determination	SLC30A1	0.018726	-2.688404	
12321	Genotype determination	PHLDB2	0.003757	-2.672148	
12322	Genotype determination	NCRNA00173	0.007254	-2.643593	
12323	Genotype determination	NEAT1	0.003938	-2.58381	
12324	Genotype determination	APOBEC3F	0.017305	-2.565073	
12325	Genotype determination	COL1A1	0.010989	-2.558414	
12326	Genotype determination	LOC399959	0.021416	-2.550447	
12327	Genotype determination	GNE	0.017479	-2.538542	
12328	Genotype determination	EPPK1	0.009357	-2.518823	
12329	Genotype determination	PBX1	0.015693	-2.496228	
12330	Genotype determination	SYTL2	0.003938	-2.478127	
12331	Genotype determination	CTH	0.020124	-2.455897	
12332	Genotype determination	TOP1	0.013483	-2.438088	
12333	Genotype determination	ARHGAP18	0.010461	-2.43218	
12334	Genotype determination	SCD	0.005664	-2.41538	
12335	Genotype determination	RUNDC3B	0.016477	-2.412034	
12336	Genotype determination	ASNS	0.003757	-2.38874	
12337	Genotype determination	FHL1	0.005664	-2.382539	
12338	Genotype determination	RFX5	0.021063	-2.378002	
12339	Genotype determination	ATP6AP1L	0.020596	-2.375119	
12340	Genotype determination	C8orf83	0.019228	-2.373885	
12341	Genotype determination	FGFBP1	0.007267	-2.356262	
12342	Genotype determination	WBSCR27	0.007118	-2.347703	
12343	Genotype determination	RASA4	0.014933	-2.34242	
12344	Genotype determination	CCDC85B	0.009064	-2.341203	
12345	Genotype determination	TRIM2	0.017298	-2.335125	
12346	Genotype determination	SCNN1A	0.009064	-2.324225	
12347	Genotype determination	PACSIN1	0.012178	-2.318594	
12348	Genotype determination	PIR	0.005664	-2.279947	

12349	Genotype determination	SREBF1	0.005982	-2.272846	
12350	Genotype determination	LOC100507198	0.009162	-2.268518	
12351	Genotype determination	LPIN1	0.011148	-2.253629	
12352	Genotype determination	MIR17HG	0.022491	-2.249337	
12353	Genotype determination	GABARAPL1	0.017298	-2.222217	
12354	Genotype determination	HSD17B7	0.017305	-2.220293	
12355	Genotype determination	RNFT2	0.009495	-2.20381	
12356	Genotype determination	AQP3	0.005581	-2.20152	
12357	Genotype determination	SESTD1	0.011148	-2.200376	
12358	Genotype determination	FLJ31306	0.012178	-2.194664	
12359	Genotype determination	TNS3	0.009162	-2.191244	
12360	Genotype determination	RPS6KA5	0.0077	-2.183663	
12361	Genotype determination	C10orf58	0.007254	-2.170459	
12362	Genotype determination	DHCR7	0.009162	-2.139836	
12363	Genotype determination	NR1D2	0.009601	-2.138724	
12364	Genotype determination	PTPLA	0.016069	-2.130955	
12365	Genotype determination	CBLB	0.019557	-2.130216	
12366	Genotype determination	TM4SF18	0.009399	-2.125055	
12367	Genotype determination	MECOM	0.022445	-2.117703	
12368	Genotype determination	IDH1	0.0077	-2.05801	
12369	Genotype determination	CRYGS	0.020596	-2.051245	
12370	Genotype determination	RNASE4	0.011148	-2.04344	
12371	Genotype determination	RBKS	0.020734	-2.040256	
12372	Genotype determination	ARID5B	0.020917	-2.027216	
12373	Genotype determination	C1orf97	0.016069	-2.02511	
12374	Genotype determination	TAF9B	0.014933	-2.002427	
12375	Genotype determination	IL27RA	0.022019	2.001387	
12376	Genotype determination	LOC730755	0.007254	2.042732	
12377	Genotype determination	THBS1	0.020473	2.125055	
12378	Genotype determination	NUPL1	0.007699	2.183284	
12379	Genotype determination	S100A11	0.010462	2.19847	
12380	Genotype determination	HOXA3	0.003757	2.283901	
12381	Genotype determination	NT5E	0.009162	2.426287	
12382	Genotype determination	CPA4	0.01594	2.491907	
12383	Genotype determination	PVRL3	0.007254	2.558858	
12384	Genotype determination	ACSL5	0.005988	3.316129	
12385	Genotype determination	HIST1H2BD	0.014933	3.335724	
12386	Hematocrit procedure	TSPAN8	0	-548.008396	
12387	Hematocrit procedure	PLA2G2A	0.000006	-510.8109	
12388	Hematocrit procedure	AGR2	0.000006	-440.058646	
12389	Hematocrit procedure	LCK	0.000003	-297.044368	
12390	Hematocrit procedure	FAM3B	0.000009	-205.648843	
12391	Hematocrit procedure	MANSC1	0.000007	-164.830215	
12392	Hematocrit procedure	AKR1C2	0	-144.760834	
12393	Hematocrit procedure	CCL14-CCL15	0.000002	-143.80414	
12394	Hematocrit procedure	ASB4	0	-142.105173	
12395	Hematocrit procedure	C9orf152	0.000001	-124.698025	
12396	Hematocrit procedure	UGT1A6	0.000002	-123.718578	
12397	Hematocrit procedure	CASP1	0.000002	-120.264435	
12398	Hematocrit procedure	LOC100507192	0.000006	-109.086216	

12399	Hematocrit procedure	VIL1	0.000001	-107.071449	
12400	Hematocrit procedure	CARD16	0.000005	-105.058602	
12401	Hematocrit procedure	AKR1C1	0.000001	-101.557094	
12402	Hematocrit procedure	ABHD12B	0.000003	-96.415173	
12403	Hematocrit procedure	C9orf64	0.000008	-95.383544	
12404	Hematocrit procedure	KRT6B	0.000002	-80.131876	
12405	Hematocrit procedure	AZGP1	0.000005	-75.120812	
12406	Hematocrit procedure	PLAC8	0.000004	-67.825095	
12407	Hematocrit procedure	PDZK1	0.000001	-67.054252	
12408	Hematocrit procedure	EIF1AY	0.000001	-63.631934	
12409	Hematocrit procedure	OXGR1	0.000002	-63.151094	
12410	Hematocrit procedure	TFF3	0.000003	-60.13651	
12411	Hematocrit procedure	UGT1A8	0	-55.425596	
12412	Hematocrit procedure	LOC643201	0.000001	-53.597538	
12413	Hematocrit procedure	PSMB8	0	-52.118448	
12414	Hematocrit procedure	HIST1H3C	0.000172	-51.063572	
12415	Hematocrit procedure	ALDH1A1	0.000066	-49.933504	
12416	Hematocrit procedure	SCCPDH	0.000003	-49.108069	
12417	Hematocrit procedure	SORBS1	0.000004	-48.717851	
12418	Hematocrit procedure	HIST1H2BI	0.000014	-44.493057	
12419	Hematocrit procedure	RARRES1	0	-44.474544	
12420	Hematocrit procedure	PIP5K1B	0.000004	-43.841456	
12421	Hematocrit procedure	SLC35D3	0.000001	-42.916508	
12422	Hematocrit procedure	ZNF750	0.000002	-39.920646	
12423	Hematocrit procedure	GNG2	0.000006	-39.84096	
12424	Hematocrit procedure	GSTT1	0.000011	-39.362819	
12425	Hematocrit procedure	GIMAP2	0.000002	-38.94836	
12426	Hematocrit procedure	CALB1	0.000011	-38.922345	
12427	Hematocrit procedure	RASEF	0.000003	-38.209334	
12428	Hematocrit procedure	HIST1H2BB	0	-37.778601	
12429	Hematocrit procedure	SPRR3	0.000007	-37.149195	
12430	Hematocrit procedure	HCN1	0.000011	-36.841006	
12431	Hematocrit procedure	DDC	0.000002	-34.955331	
12432	Hematocrit procedure	SLC7A7	0.000004	-33.871488	
12433	Hematocrit procedure	GAS2	0.000004	-32.924259	
12434	Hematocrit procedure	ASCL2	0.000002	-32.801288	
12435	Hematocrit procedure	FLJ20184	0	-32.739482	
12436	Hematocrit procedure	UGT1A3	0.000215	-31.187261	
12437	Hematocrit procedure	PROX1	0.000002	-31.142485	
12438	Hematocrit procedure	LOC100124692	0.000008	-30.496008	
12439	Hematocrit procedure	C15orf48	0.000006	-30.344559	
12440	Hematocrit procedure	CCR6	0.000005	-29.162851	
12441	Hematocrit procedure	SGK2	0.000001	-29.128191	
12442	Hematocrit procedure	C4orf18	0.000016	-26.415101	
12443	Hematocrit procedure	SCML4	0.000008	-25.82047	
12444	Hematocrit procedure	CCR1	0.000006	-25.656619	
12445	Hematocrit procedure	LOC401522	0.000002	-23.921747	
12446	Hematocrit procedure	CYorf15A	0.000003	-23.376836	
12447	Hematocrit procedure	ZNF703	0.000007	-23.366143	
12448	Hematocrit procedure	CTSS	0.000006	-23.057346	

12449	Hematocrit procedure	EPSTI1	0.000006	-22.529177	
12450	Hematocrit procedure	PTGR1	0.000007	-22.056714	
12451	Hematocrit procedure	HSD17B2	0.000074	-21.748633	
12452	Hematocrit procedure	SORBS2	0.000003	-20.472901	
12453	Hematocrit procedure	C5orf17	0.000004	-19.220543	
12454	Hematocrit procedure	CXCR3	0.000008	-18.966222	
12455	Hematocrit procedure	FOXA2	0.000003	-18.266987	
12456	Hematocrit procedure	C6orf150	0.000175	-17.5689	
12457	Hematocrit procedure	CD302	0.000018	-17.464301	
12458	Hematocrit procedure	FCGRT	0.000007	-17.453322	
12459	Hematocrit procedure	BCL2L15	0.000006	-16.516903	
12460	Hematocrit procedure	CHST11	0.000007	-15.569454	
12461	Hematocrit procedure	ACOT1	0.000011	-15.376655	
12462	Hematocrit procedure	RNF128	0.000065	-14.813946	
12463	Hematocrit procedure	MGLL	0.000165	-14.614457	
12464	Hematocrit procedure	TNFRSF1B	0.000007	-14.592414	
12465	Hematocrit procedure	CACNA2D4	0.000007	-14.539368	
12466	Hematocrit procedure	IRF8	0.000003	-13.757054	
12467	Hematocrit procedure	A1CF	0.000008	-13.437266	
12468	Hematocrit procedure	SDC1	0.000003	-13.139692	
12469	Hematocrit procedure	IGF1R	0.000011	-12.967261	
12470	Hematocrit procedure	EPHB2	0.000004	-12.340973	
12471	Hematocrit procedure	NHSL1	0.000081	-12.23337	
12472	Hematocrit procedure	C12orf27	0.000002	-11.136132	
12473	Hematocrit procedure	SLC40A1	0.000033	-10.868366	
12474	Hematocrit procedure	RNF157	0.000085	-10.802542	
12475	Hematocrit procedure	PKP1	0.000012	-10.698267	
12476	Hematocrit procedure	ECM1	0.000002	-10.281889	
12477	Hematocrit procedure	ZNF506	0.000005	-10.048693	
12478	Hematocrit procedure	EDEM1	0.000009	-9.667083	
12479	Hematocrit procedure	CYorf15B	0.000005	-9.546186	
12480	Hematocrit procedure	DPEP1	0.000011	-9.477999	
12481	Hematocrit procedure	CDK6	0	-9.395292	
12482	Hematocrit procedure	OAS1	0.00027	-9.357664	
12483	Hematocrit procedure	C6orf64	0.000008	-9.350503	
12484	Hematocrit procedure	TUSC1	0.000071	-8.920776	
12485	Hematocrit procedure	ANP32E	0.000003	-8.890413	
12486	Hematocrit procedure	NSUN7	0.000002	-8.518426	
12487	Hematocrit procedure	TNFRSF19	0.000006	-8.17702	
12488	Hematocrit procedure	C10orf58	0.000003	-8.113021	
12489	Hematocrit procedure	SH3PXD2A	0.000006	-8.056364	
12490	Hematocrit procedure	EHF	0.000119	-8.010758	
12491	Hematocrit procedure	CHFR	0.000217	-8.003618	
12492	Hematocrit procedure	DNAJC3	0.000003	-7.962623	
12493	Hematocrit procedure	GPR39	0.000007	-7.846796	
12494	Hematocrit procedure	RIPK3	0.000099	-7.831119	
12495	Hematocrit procedure	MECOM	0.00001	-7.812382	
12496	Hematocrit procedure	RIN2	0	-7.78971	
12497	Hematocrit procedure	PERP	0.000011	-7.760576	
12498	Hematocrit procedure	MAD1L1	0.000002	-7.721161	

12499	Hematocrit procedure	CYP3A5	0.000271	-7.717247	
12500	Hematocrit procedure	PDK3	0.000003	-7.534799	
12501	Hematocrit procedure	TMEM62	0.000002	-7.375007	
12502	Hematocrit procedure	EPB41L4B	0.000001	-7.309877	
12503	Hematocrit procedure	ADAMTS17	0.000008	-7.30365	
12504	Hematocrit procedure	ZNF260	0.000009	-7.257563	
12505	Hematocrit procedure	PLIN2	0.000003	-7.13014	
12506	Hematocrit procedure	PRSS2	0.000029	-6.999257	
12507	Hematocrit procedure	UGT1A4	0.000277	-6.898427	
12508	Hematocrit procedure	FUT6	0.000011	-6.724803	
12509	Hematocrit procedure	HNF1A	0.000002	-6.722007	
12510	Hematocrit procedure	HIST1H3G	0.000001	-6.649406	
12511	Hematocrit procedure	MAST4	0.000008	-6.637752	
12512	Hematocrit procedure	TAF8	0.000008	-6.633628	
12513	Hematocrit procedure	HSPA12A	0.000001	-6.570195	
12514	Hematocrit procedure	FAM113B	0.000018	-6.458196	
12515	Hematocrit procedure	LOC400128	0.000008	-6.414813	
12516	Hematocrit procedure	GNAS	0.000106	-6.351093	
12517	Hematocrit procedure	SPATA13	0.000011	-6.214893	
12518	Hematocrit procedure	SRC	0.00013	-6.169824	
12519	Hematocrit procedure	MED20	0.000003	-6.097804	
12520	Hematocrit procedure	PRDM1	0.000091	-6.095754	
12521	Hematocrit procedure	EDAR	0.000008	-6.047987	
12522	Hematocrit procedure	TCF7	0.000005	-6.035586	
12523	Hematocrit procedure	LPCAT1	0.000007	-5.975302	
12524	Hematocrit procedure	LOC100499467	0.000019	-5.858976	
12525	Hematocrit procedure	APOL6	0.000009	-5.838919	
12526	Hematocrit procedure	MSH3	0.00001	-5.813158	
12527	Hematocrit procedure	EPHA4	0.000006	-5.611204	
12528	Hematocrit procedure	NF1	0.000011	-5.571354	
12529	Hematocrit procedure	CHN2	0.000002	-5.495506	
12530	Hematocrit procedure	ARHGAP26	0.000003	-5.448188	
12531	Hematocrit procedure	LAMA3	0.000005	-5.446837	
12532	Hematocrit procedure	PRSS1	0.000066	-5.37163	
12533	Hematocrit procedure	SIGLEC15	0.000008	-5.328116	
12534	Hematocrit procedure	SLC16A4	0.000241	-5.132544	
12535	Hematocrit procedure	MAP7	0.000007	-4.998464	
12536	Hematocrit procedure	MRPS10	0.000001	-4.993962	
12537	Hematocrit procedure	HPDL	0.000181	-4.976846	
12538	Hematocrit procedure	SMARCA2	0.000012	-4.928856	
12539	Hematocrit procedure	PSD4	0.000002	-4.836057	
12540	Hematocrit procedure	DNM1	0.000111	-4.792522	
12541	Hematocrit procedure	IFNGR1	0.000004	-4.775064	
12542	Hematocrit procedure	LOC100507328	0.000006	-4.76041	
12543	Hematocrit procedure	GALK2	0.000011	-4.648461	
12544	Hematocrit procedure	IL20RA	0.000092	-4.620432	
12545	Hematocrit procedure	ANKRD22	0.000008	-4.590764	
12546	Hematocrit procedure	LMO7	0	-4.488017	
12547	Hematocrit procedure	ARGLU1	0.000003	-4.387082	
12548	Hematocrit procedure	TBPL1	0.000001	-4.377912	

12549	Hematocrit procedure	SEC16B	0.000003	-4.300372	
12550	Hematocrit procedure	CTSH	0.000011	-4.285228	
12551	Hematocrit procedure	CD68	0.000005	-4.278797	
12552	Hematocrit procedure	MIA2	0.000019	-4.20487	
12553	Hematocrit procedure	PRSS3	0.000095	-4.109125	
12554	Hematocrit procedure	C5	0.000039	-4.072259	
12555	Hematocrit procedure	FRK	0.00013	-4.030782	
12556	Hematocrit procedure	FBLIM1	0.000102	-3.989457	
12557	Hematocrit procedure	TJP2	0.000003	-3.974191	
12558	Hematocrit procedure	RASSF5	0.000001	-3.967723	
12559	Hematocrit procedure	F5	0.000272	-3.810503	
12560	Hematocrit procedure	TRAK1	0.000004	-3.684815	
12561	Hematocrit procedure	IFT88	0.000004	-3.610645	
12562	Hematocrit procedure	PLEKHA6	0.000206	-3.528435	
12563	Hematocrit procedure	UBAC2	0.000002	-3.488265	
12564	Hematocrit procedure	USH1C	0.000014	-3.47718	
12565	Hematocrit procedure	RALGAPA2	0.000003	-3.471811	
12566	Hematocrit procedure	HOXC5	0.000005	-3.435105	
12567	Hematocrit procedure	S100A5	0.000012	-3.429268	
12568	Hematocrit procedure	TPP2	0	-3.361754	
12569	Hematocrit procedure	GFPT1	0.000008	-3.359642	
12570	Hematocrit procedure	GCOM1	0.000018	-3.35798	
12571	Hematocrit procedure	TNS4	0.000007	-3.341882	
12572	Hematocrit procedure	ST3GAL4	0.000263	-3.331892	
12573	Hematocrit procedure	FERMT1	0.000109	-3.325981	
12574	Hematocrit procedure	CARKD	0.000005	-3.322652	
12575	Hematocrit procedure	RABGAP1L	0	-3.273762	
12576	Hematocrit procedure	TMC7	0.000037	-3.231842	
12577	Hematocrit procedure	RNF6	0.000011	-3.218499	
12578	Hematocrit procedure	COL17A1	0.000084	-3.189146	
12579	Hematocrit procedure	TNRC18	0.000005	-3.135812	
12580	Hematocrit procedure	LAMB3	0.000027	-3.13326	
12581	Hematocrit procedure	BAIAP2L2	0.000084	-3.122287	
12582	Hematocrit procedure	KALRN	0.000069	-3.11255	
12583	Hematocrit procedure	LNX2	0.000003	-3.098544	
12584	Hematocrit procedure	SLC5A1	0.000046	-3.058335	
12585	Hematocrit procedure	RPP25	0.000177	-3.049872	
12586	Hematocrit procedure	PTK6	0.000071	-3.034597	
12587	Hematocrit procedure	MIPEP	0.000003	-3.028462	
12588	Hematocrit procedure	TSEN15	0.000008	-3.014576	
12589	Hematocrit procedure	SLC25A13	0.000108	-2.981576	
12590	Hematocrit procedure	TMEM133	0.000081	-2.945957	
12591	Hematocrit procedure	ITGA6	0.000017	-2.921719	
12592	Hematocrit procedure	FAM108C1	0.000281	-2.89171	
12593	Hematocrit procedure	STAMBPL1	0.000004	-2.891243	
12594	Hematocrit procedure	HBS1L	0.000005	-2.859622	
12595	Hematocrit procedure	PTPRB	0.000082	-2.847353	
12596	Hematocrit procedure	KRT8P41	0.001991	-2.833851	
12597	Hematocrit procedure	FUT4	0.000078	-2.815228	
12598	Hematocrit procedure	CHCHD7	0.000001	-2.802096	

12599	Hematocrit procedure	EPS8L3	0.000032	-2.795256	
12600	Hematocrit procedure	TUBA4A	0.000008	-2.789495	
12601	Hematocrit procedure	ELF3	0.000421	-2.787356	
12602	Hematocrit procedure	ARHGAP42	0.000055	-2.773032	
12603	Hematocrit procedure	SATB1	0.000097	-2.751735	
12604	Hematocrit procedure	RGS14	0.000096	-2.726364	
12605	Hematocrit procedure	JUN	0.000055	-2.725946	
12606	Hematocrit procedure	TDRKH	0.000007	-2.717036	
12607	Hematocrit procedure	ROD1	0.000008	-2.699594	
12608	Hematocrit procedure	DPAGT1	0	-2.696705	
12609	Hematocrit procedure	ADAP1	0.0001	-2.687005	
12610	Hematocrit procedure	METTL9	0.000027	-2.673071	
12611	Hematocrit procedure	MKKS	0.000002	-2.66267	
12612	Hematocrit procedure	AADACL1	0	-2.662401	
12613	Hematocrit procedure	SURF4	0.000006	-2.6467	
12614	Hematocrit procedure	MGST1	0.000911	-2.642064	
12615	Hematocrit procedure	EPHB3	0.000008	-2.630731	
12616	Hematocrit procedure	CASK	0.000008	-2.618893	
12617	Hematocrit procedure	MTMR6	0.000005	-2.609182	
12618	Hematocrit procedure	AGA	0.000215	-2.593541	
12619	Hematocrit procedure	CEBPD	0.001585	-2.58863	
12620	Hematocrit procedure	NCBP1	0.000007	-2.570732	
12621	Hematocrit procedure	MRPS31	0.000004	-2.566806	
12622	Hematocrit procedure	LAD1	0.000172	-2.563215	
12623	Hematocrit procedure	SBF1	0.00001	-2.529004	
12624	Hematocrit procedure	VDAC1	0	-2.524484	
12625	Hematocrit procedure	EFNB1	0.000003	-2.511787	
12626	Hematocrit procedure	GPR89A	0.000011	-2.508425	
12627	Hematocrit procedure	MLKL	0.000008	-2.480787	
12628	Hematocrit procedure	ARHGEF10L	0.000044	-2.468049	
12629	Hematocrit procedure	GPR160	0.000006	-2.457485	
12630	Hematocrit procedure	ECOP	0.000166	-2.441085	
12631	Hematocrit procedure	INPP1	0.000097	-2.422773	
12632	Hematocrit procedure	FAM62B	0.000001	-2.414755	
12633	Hematocrit procedure	PDIA4	0.00001	-2.411109	
12634	Hematocrit procedure	SIK1	0.000055	-2.407825	
12635	Hematocrit procedure	CTSD	0.000039	-2.401471	
12636	Hematocrit procedure	MCTP1	0.000112	-2.393982	
12637	Hematocrit procedure	ZNF223	0.000022	-2.384845	
12638	Hematocrit procedure	PRKCA	0.000153	-2.378513	
12639	Hematocrit procedure	GLA	0.000029	-2.353499	
12640	Hematocrit procedure	PLCH1	0.000044	-2.337335	
12641	Hematocrit procedure	TMSB4X	0.000008	-2.335537	
12642	Hematocrit procedure	SNRPA1	0.000007	-2.308718	
12643	Hematocrit procedure	SEC23B	0.000098	-2.308136	
12644	Hematocrit procedure	C1orf56	0.00082	-2.307062	
12645	Hematocrit procedure	ST7	0.000002	-2.304399	
12646	Hematocrit procedure	WNT11	0.000135	-2.301905	
12647	Hematocrit procedure	SPSB1	0.0013	-2.300466	
12648	Hematocrit procedure	ABHD12	0.000046	-2.296449	

12649	Hematocrit procedure	FAM73B	0.000007	-2.288975	
12650	Hematocrit procedure	C20orf74	0.000106	-2.282048	
12651	Hematocrit procedure	FAM60A	0.001316	-2.28002	
12652	Hematocrit procedure	PDIA6	0.000001	-2.277313	
12653	Hematocrit procedure	LOC100506922	0.000096	-2.256579	
12654	Hematocrit procedure	MNX1	0.000413	-2.247583	
12655	Hematocrit procedure	RICS	0.000012	-2.22689	
12656	Hematocrit procedure	GTF2IRD2B	0.000215	-2.225307	
12657	Hematocrit procedure	UBE2D2	0.000011	-2.218704	
12658	Hematocrit procedure	ZDHHC9	0.00026	-2.215214	
12659	Hematocrit procedure	HIST4H4	0.000273	-2.205272	
12660	Hematocrit procedure	ROMO1	0.000002	-2.201651	
12661	Hematocrit procedure	LMNA	0.000237	-2.191642	
12662	Hematocrit procedure	LFNG	0.000028	-2.189166	
12663	Hematocrit procedure	RIPK4	0.000018	-2.176778	
12664	Hematocrit procedure	MACF1	0.000004	-2.176726	
12665	Hematocrit procedure	KRT8	0.000684	-2.154823	
12666	Hematocrit procedure	LOC645249	0.000043	-2.153423	
12667	Hematocrit procedure	LMAN2	0.000009	-2.153212	
12668	Hematocrit procedure	CCDC82	0.000073	-2.151796	
12669	Hematocrit procedure	TOLLIP	0.000011	-2.144689	
12670	Hematocrit procedure	DLG3	0.000157	-2.144243	
12671	Hematocrit procedure	ETV6	0.000197	-2.139379	
12672	Hematocrit procedure	GSTK1	0.000047	-2.134457	
12673	Hematocrit procedure	LOC100132999	0.000077	-2.13246	
12674	Hematocrit procedure	WDR67	0.000066	-2.131068	
12675	Hematocrit procedure	RBCK1	0.00012	-2.130934	
12676	Hematocrit procedure	ACY1	0.000024	-2.124582	
12677	Hematocrit procedure	C3orf23	0	-2.119588	
12678	Hematocrit procedure	ARFGEF2	0.000032	-2.111078	
12679	Hematocrit procedure	PCGF5	0.000051	-2.107123	
12680	Hematocrit procedure	CCDC109A	0.000004	-2.105463	
12681	Hematocrit procedure	TMEM164	0.000033	-2.103535	
12682	Hematocrit procedure	VAPB	0.000791	-2.101942	
12683	Hematocrit procedure	CDC42SE2	0.000005	-2.101573	
12684	Hematocrit procedure	B4GALT5	0.00004	-2.087517	
12685	Hematocrit procedure	MTMR2	0.000008	-2.083627	
12686	Hematocrit procedure	CIB1	0.000575	-2.081377	
12687	Hematocrit procedure	MCCC1	0.000077	-2.080364	
12688	Hematocrit procedure	ADRBK1	0.000003	-2.075921	
12689	Hematocrit procedure	BGN	0.000202	-2.072609	
12690	Hematocrit procedure	CCDC64	0.000014	-2.069689	
12691	Hematocrit procedure	LOC339988	0.000008	-2.000944	
12692	Hematocrit procedure	GTPBP3	0.000709	2.000319	
12693	Hematocrit procedure	ZBTB8	0.000169	2.002427	
12694	Hematocrit procedure	MGC57346	0.000011	2.0041	
12695	Hematocrit procedure	NHLRC2	0.000173	2.005527	
12696	Hematocrit procedure	ADAM22	0.000216	2.00715	
12697	Hematocrit procedure	TIAL1	0.000008	2.008613	
12698	Hematocrit procedure	5-Mar	0.000021	2.009577	

12699	Hematocrit procedure	GCFC1	0.000028	2.012418	
12700	Hematocrit procedure	SMU1	0.00001	2.014751	
12701	Hematocrit procedure	TRMT1	0.000044	2.021316	
12702	Hematocrit procedure	RPL4	0.000192	2.02487	
12703	Hematocrit procedure	AUH	0.000004	2.02723	
12704	Hematocrit procedure	QRICH2	0.000234	2.027934	
12705	Hematocrit procedure	KIAA1333	0.000072	2.030055	
12706	Hematocrit procedure	METTL10	0.000008	2.03607	
12707	Hematocrit procedure	RAVER2	0.000087	2.039754	
12708	Hematocrit procedure	NACC1	0	2.041808	
12709	Hematocrit procedure	CDKL3	0.000247	2.04579	
12710	Hematocrit procedure	DENND5A	0.000169	2.04788	
12711	Hematocrit procedure	GEMIN5	0.000188	2.052325	
12712	Hematocrit procedure	MYBBP1A	0.000069	2.05959	
12713	Hematocrit procedure	PDLIM5	0.000004	2.061163	
12714	Hematocrit procedure	INSR	0.000057	2.074774	
12715	Hematocrit procedure	KPNA5	0.000021	2.080091	
12716	Hematocrit procedure	LAMB2	0.000265	2.080383	
12717	Hematocrit procedure	GTF2H2D	0.000424	2.082886	
12718	Hematocrit procedure	INTS2	0.000033	2.084261	
12719	Hematocrit procedure	IFT57	0.000858	2.09242	
12720	Hematocrit procedure	PLD6	0.000173	2.092744	
12721	Hematocrit procedure	DGCR8	0.000004	2.097377	
12722	Hematocrit procedure	ZMYM1	0.000171	2.098944	
12723	Hematocrit procedure	KTN1	0.000281	2.10316	
12724	Hematocrit procedure	MSTO2P	0.000064	2.105921	
12725	Hematocrit procedure	CP110	0.000081	2.107218	
12726	Hematocrit procedure	C18orf24	0.00007	2.107296	
12727	Hematocrit procedure	PQLC1	0.000005	2.11065	
12728	Hematocrit procedure	IMPACT	0.000011	2.111917	
12729	Hematocrit procedure	TOP3B	0.000022	2.113287	
12730	Hematocrit procedure	BMP8B	0.00001	2.118034	
12731	Hematocrit procedure	CCDC24	0.000018	2.120131	
12732	Hematocrit procedure	HDGF2	0.000008	2.121635	
12733	Hematocrit procedure	SPTB	0.000251	2.134205	
12734	Hematocrit procedure	CDCA2	0.000062	2.134742	
12735	Hematocrit procedure	ST6GALNAC6	0.000245	2.134926	
12736	Hematocrit procedure	C20orf96	0.000107	2.141774	
12737	Hematocrit procedure	BTN2A2	0.000132	2.143085	
12738	Hematocrit procedure	C14orf159	0.002043	2.143809	
12739	Hematocrit procedure	KIAA1432	0.000002	2.144912	
12740	Hematocrit procedure	AGPAT5	0.000227	2.148063	
12741	Hematocrit procedure	LOC646851	0.000099	2.150201	
12742	Hematocrit procedure	INVS	0.00029	2.150785	
12743	Hematocrit procedure	CTNNAL1	0.000186	2.152896	
12744	Hematocrit procedure	RTTN	0.00001	2.154799	
12745	Hematocrit procedure	USP33	0.000005	2.154806	
12746	Hematocrit procedure	CEPT1	0.00011	2.164874	
12747	Hematocrit procedure	HOXB13	0.00003	2.165502	
12748	Hematocrit procedure	YJEFN3	0.000022	2.167793	

12749	Hematocrit procedure	ACCN2	0.001269	2.170963	
12750	Hematocrit procedure	SETD4	0.001022	2.175046	
12751	Hematocrit procedure	PCNX	0.000005	2.176842	
12752	Hematocrit procedure	POMT2	0.000136	2.181866	
12753	Hematocrit procedure	HCG8	0.000105	2.182757	
12754	Hematocrit procedure	HMGB1	0.000257	2.183219	
12755	Hematocrit procedure	NSD1	0.00002	2.185498	
12756	Hematocrit procedure	MAP3K7	0.000008	2.186104	
12757	Hematocrit procedure	HSP90AA1	0.001783	2.189518	
12758	Hematocrit procedure	DUS3L	0.000158	2.196435	
12759	Hematocrit procedure	EIF2AK4	0.000004	2.199079	
12760	Hematocrit procedure	MON2	0.000038	2.203917	
12761	Hematocrit procedure	HEATR6	0.000171	2.205405	
12762	Hematocrit procedure	RPS28	0.000155	2.20962	
12763	Hematocrit procedure	DDR2	0.000228	2.217265	
12764	Hematocrit procedure	SLC25A30	0.0001	2.221969	
12765	Hematocrit procedure	MTX3	0.000005	2.2229	
12766	Hematocrit procedure	NPC2	0.001683	2.222995	
12767	Hematocrit procedure	SLC25A39	0.000001	2.228725	
12768	Hematocrit procedure	PNPLA6	0.000004	2.230106	
12769	Hematocrit procedure	BAG3	0.001683	2.2303	
12770	Hematocrit procedure	CITED2	0.000053	2.232065	
12771	Hematocrit procedure	CDK9	0.000844	2.233081	
12772	Hematocrit procedure	EXOC6B	0.000059	2.233084	
12773	Hematocrit procedure	RAB11FIP2	0.000073	2.234653	
12774	Hematocrit procedure	NTAN1	0.000203	2.23913	
12775	Hematocrit procedure	PPM1M	0.00004	2.24155	
12776	Hematocrit procedure	EFR3A	0.000005	2.248263	
12777	Hematocrit procedure	POLI	0.000007	2.267001	
12778	Hematocrit procedure	LYG1	0.000174	2.267478	
12779	Hematocrit procedure	DOCK1	0	2.267904	
12780	Hematocrit procedure	DCAKD	0.001901	2.272676	
12781	Hematocrit procedure	NOC3L	0.000093	2.275555	
12782	Hematocrit procedure	SHOX2	0.00001	2.276001	
12783	Hematocrit procedure	C17orf69	0.000223	2.279328	
12784	Hematocrit procedure	RANBP17	0.000015	2.279878	
12785	Hematocrit procedure	SRMS	0.000118	2.283625	
12786	Hematocrit procedure	C10orf137	0.000001	2.288498	
12787	Hematocrit procedure	PHTF2	0.00017	2.288694	
12788	Hematocrit procedure	RUSC2	0.000045	2.289799	
12789	Hematocrit procedure	ARHGEF37	0.000015	2.290725	
12790	Hematocrit procedure	CSPG5	0.000102	2.291194	
12791	Hematocrit procedure	SENP6	0.000165	2.296558	
12792	Hematocrit procedure	CDH24	0.000056	2.29812	
12793	Hematocrit procedure	MOSPD1	0.000053	2.301269	
12794	Hematocrit procedure	GLG1	0.000011	2.3013	
12795	Hematocrit procedure	CENPC1	0.000015	2.302085	
12796	Hematocrit procedure	FXR1	0.000005	2.303015	
12797	Hematocrit procedure	LRPAP1	0.000069	2.310307	
12798	Hematocrit procedure	TNFSF12	0.000061	2.313381	

12799	Hematocrit procedure	ZCCHC11	0.000028	2.321129	
12800	Hematocrit procedure	GOLGA9P	0.000092	2.322996	
12801	Hematocrit procedure	ZSCAN20	0.000184	2.334139	
12802	Hematocrit procedure	BTRC	0.000218	2.339372	
12803	Hematocrit procedure	TRAF3	0.000028	2.339781	
12804	Hematocrit procedure	CBX7	0.000421	2.340475	
12805	Hematocrit procedure	ERI1	0.000211	2.342181	
12806	Hematocrit procedure	INPP5E	0.000001	2.344917	
12807	Hematocrit procedure	KSR2	0.000041	2.345834	
12808	Hematocrit procedure	UNC119	0.000046	2.348208	
12809	Hematocrit procedure	BNIP3L	0.000002	2.359025	
12810	Hematocrit procedure	FUT10	0.000028	2.364717	
12811	Hematocrit procedure	CNOT7	0.000104	2.367895	
12812	Hematocrit procedure	CLUAP1	0.001451	2.37133	
12813	Hematocrit procedure	KCTD6	0.000059	2.376819	
12814	Hematocrit procedure	MAMLD1	0.000008	2.377772	
12815	Hematocrit procedure	C6orf170	0.000175	2.382184	
12816	Hematocrit procedure	C9orf61	0.000154	2.385151	
12817	Hematocrit procedure	PRKAR1A	0.000008	2.414002	
12818	Hematocrit procedure	NR2F2	0.001215	2.416529	
12819	Hematocrit procedure	CELSR2	0.000148	2.416698	
12820	Hematocrit procedure	C18orf54	0.00015	2.422807	
12821	Hematocrit procedure	LOC100507448	0.000008	2.423487	
12822	Hematocrit procedure	SEC31B	0.000003	2.428658	
12823	Hematocrit procedure	OSBP2	0.000125	2.441452	
12824	Hematocrit procedure	PIK3C3	0.000067	2.444206	
12825	Hematocrit procedure	NOTCH2NL	0.000099	2.448863	
12826	Hematocrit procedure	IFT74	0.000199	2.45617	
12827	Hematocrit procedure	DOCK7	0.00001	2.459611	
12828	Hematocrit procedure	F8	0.000054	2.460219	
12829	Hematocrit procedure	DCLK2	0.000008	2.466714	
12830	Hematocrit procedure	RNGTT	0.000007	2.474348	
12831	Hematocrit procedure	PLA2G6	0.000078	2.483683	
12832	Hematocrit procedure	TOP3A	0.000235	2.487689	
12833	Hematocrit procedure	DDHD1	0.000015	2.488128	
12834	Hematocrit procedure	LHPP	0.000262	2.491126	
12835	Hematocrit procedure	LYPLAL1	0.000019	2.496133	
12836	Hematocrit procedure	NTN5	0.000014	2.500072	
12837	Hematocrit procedure	GPAM	0.000217	2.502145	
12838	Hematocrit procedure	TPST1	0.000145	2.506486	
12839	Hematocrit procedure	RBM4	0.000055	2.508263	
12840	Hematocrit procedure	ANKRD36B	0.000012	2.511553	
12841	Hematocrit procedure	LOC728377	0.000002	2.515295	
12842	Hematocrit procedure	TGFB1I1	0.000292	2.521098	
12843	Hematocrit procedure	ELAC1	0.000083	2.535801	
12844	Hematocrit procedure	CHST3	0.000017	2.541441	
12845	Hematocrit procedure	SSX2IP	0.000237	2.543506	
12846	Hematocrit procedure	WDR7	0.00009	2.543718	
12847	Hematocrit procedure	RNFT2	0.000901	2.544818	
12848	Hematocrit procedure	RAD51C	0.000073	2.545745	

12849	Hematocrit procedure	THRAP3	0.000016	2.547111	
12850	Hematocrit procedure	SGSH	0.000136	2.547538	
12851	Hematocrit procedure	TRIM37	0.000077	2.557137	
12852	Hematocrit procedure	SEC61A2	0.000002	2.557307	
12853	Hematocrit procedure	ZDHHC17	0.000006	2.562145	
12854	Hematocrit procedure	TNRC6C	0.000008	2.56454	
12855	Hematocrit procedure	CROP	0.000254	2.584791	
12856	Hematocrit procedure	TIFA	0.000069	2.606932	
12857	Hematocrit procedure	BCORL1	0.000185	2.618549	
12858	Hematocrit procedure	C2orf76	0.000161	2.632252	
12859	Hematocrit procedure	OAT	0.001585	2.634221	
12860	Hematocrit procedure	C21orf7	0.000003	2.636255	
12861	Hematocrit procedure	ZNF57	0.000004	2.63682	
12862	Hematocrit procedure	NEK1	0.000095	2.644595	
12863	Hematocrit procedure	FBXL2	0.000111	2.645171	
12864	Hematocrit procedure	EIF3F	0.000039	2.645408	
12865	Hematocrit procedure	LOC652005	0.000018	2.648741	
12866	Hematocrit procedure	HS6ST1	0.000022	2.650736	
12867	Hematocrit procedure	PKN3	0.000087	2.66276	
12868	Hematocrit procedure	HYMAI	0.000108	2.667008	
12869	Hematocrit procedure	MEGF6	0.000094	2.674067	
12870	Hematocrit procedure	TARSL2	0.000001	2.682116	
12871	Hematocrit procedure	SOCS5	0.000008	2.690637	
12872	Hematocrit procedure	ASAM	0.000154	2.72168	
12873	Hematocrit procedure	PMAIP1	0.000012	2.735515	
12874	Hematocrit procedure	DZIP1L	0.000008	2.745394	
12875	Hematocrit procedure	PSMD3	0.000273	2.784817	
12876	Hematocrit procedure	NIPSNAP1	0.000006	2.789355	
12877	Hematocrit procedure	ADAM32	0.000228	2.80424	
12878	Hematocrit procedure	MFGE8	0.000003	2.808922	
12879	Hematocrit procedure	SFI1	0.000028	2.810339	
12880	Hematocrit procedure	MAP4	0.000005	2.832426	
12881	Hematocrit procedure	HEMK1	0.000041	2.837929	
12882	Hematocrit procedure	GCAT	0.00009	2.839744	
12883	Hematocrit procedure	CYP2U1	0.000128	2.843941	
12884	Hematocrit procedure	EPOR	0.000069	2.845683	
12885	Hematocrit procedure	TANC2	0.00003	2.848808	
12886	Hematocrit procedure	SMN1	0.000003	2.86054	
12887	Hematocrit procedure	JAZF1	0.000058	2.866248	
12888	Hematocrit procedure	3-Sep	0.000009	2.871437	
12889	Hematocrit procedure	COL18A1	0.000011	2.87342	
12890	Hematocrit procedure	ANKRD18A	0.000249	2.876288	
12891	Hematocrit procedure	ZNF25	0.000031	2.880115	
12892	Hematocrit procedure	GTF2H2	0.000295	2.882074	
12893	Hematocrit procedure	TNFAIP8L1	0.000026	2.906807	
12894	Hematocrit procedure	ACTR3B	0.000004	2.920274	
12895	Hematocrit procedure	STAT4	0.000066	2.930509	
12896	Hematocrit procedure	SLC38A2	0.000011	2.939973	
12897	Hematocrit procedure	LOC100132153	0.000003	2.945493	
12898	Hematocrit procedure	SFRS1	0.000059	2.952906	

12899	Hematocrit procedure	LOC54492	0.000069	2.953109	
12900	Hematocrit procedure	PAQR6	0.000747	2.958059	
12901	Hematocrit procedure	SYTL1	0.000103	2.96032	
12902	Hematocrit procedure	ATP2A1	0.000012	2.963659	
12903	Hematocrit procedure	CNTNAP1	0.000028	2.966271	
12904	Hematocrit procedure	SNX26	0.000022	2.967364	
12905	Hematocrit procedure	ZNF37A	0.000218	2.970766	
12906	Hematocrit procedure	RAB18	0.000004	2.978612	
12907	Hematocrit procedure	NPHP1	0.000147	2.986739	
12908	Hematocrit procedure	AGPAT4	0.000168	2.989244	
12909	Hematocrit procedure	SEMA4F	0.000162	2.992848	
12910	Hematocrit procedure	SCG5	0.00013	2.997883	
12911	Hematocrit procedure	CNNM2	0.000001	3.007572	
12912	Hematocrit procedure	ADORA1	0.000019	3.012767	
12913	Hematocrit procedure	CYB5B	0.000002	3.022785	
12914	Hematocrit procedure	HYLS1	0.000139	3.023997	
12915	Hematocrit procedure	BCYRN1	0.000048	3.029967	
12916	Hematocrit procedure	LOC728047	0.000131	3.034422	
12917	Hematocrit procedure	CAMK1	0.000151	3.043395	
12918	Hematocrit procedure	SLC30A4	0.00002	3.072022	
12919	Hematocrit procedure	C14orf4	0.000018	3.089096	
12920	Hematocrit procedure	SRSF6	0.000848	3.092448	
12921	Hematocrit procedure	RAPGEF2	0.000289	3.095119	
12922	Hematocrit procedure	KIAA1586	0.000186	3.096102	
12923	Hematocrit procedure	ELAVL1	0.000008	3.112688	
12924	Hematocrit procedure	SIM2	0.000077	3.115892	
12925	Hematocrit procedure	MAP3K3	0.000158	3.117407	
12926	Hematocrit procedure	USP45	0.000223	3.118887	
12927	Hematocrit procedure	CDRT4	0.000142	3.126199	
12928	Hematocrit procedure	SRGAP1	0.000261	3.134377	
12929	Hematocrit procedure	LPHN1	0.000201	3.149025	
12930	Hematocrit procedure	FRAS1	0.000002	3.150037	
12931	Hematocrit procedure	PARD3	0.000219	3.173436	
12932	Hematocrit procedure	CDH3	0.000159	3.193304	
12933	Hematocrit procedure	C2orf27	0.000148	3.202568	
12934	Hematocrit procedure	CR2	0.000124	3.206954	
12935	Hematocrit procedure	PBX1	0.000126	3.218884	
12936	Hematocrit procedure	CA11	0.000016	3.221219	
12937	Hematocrit procedure	GDI1	0.000006	3.232506	
12938	Hematocrit procedure	RAB36	0.00001	3.238865	
12939	Hematocrit procedure	TPBG	0.000069	3.240437	
12940	Hematocrit procedure	C3orf35	0.000084	3.24477	
12941	Hematocrit procedure	CST6	0.000119	3.246943	
12942	Hematocrit procedure	TAB2	0.000007	3.280706	
12943	Hematocrit procedure	KIAA1797	0.000023	3.287566	
12944	Hematocrit procedure	AP3S1	0.000001	3.304655	
12945	Hematocrit procedure	LTBP3	0.000008	3.322505	
12946	Hematocrit procedure	GDA	0.000005	3.335761	
12947	Hematocrit procedure	LOC375190	0.000135	3.342036	
12948	Hematocrit procedure	MDM4	0.00027	3.347505	

12949	Hematocrit procedure	NKIRAS1	0.000187	3.347774	
12950	Hematocrit procedure	CTTNBP2	0.000056	3.361674	
12951	Hematocrit procedure	C21orf63	0.000093	3.38271	
12952	Hematocrit procedure	HMSD	0.000144	3.407395	
12953	Hematocrit procedure	PKD1	0.000026	3.407801	
12954	Hematocrit procedure	GNB3	0.000037	3.409344	
12955	Hematocrit procedure	ANO8	0.000039	3.444012	
12956	Hematocrit procedure	CLTCL1	0.000153	3.469274	
12957	Hematocrit procedure	TBCEL	0.000057	3.487617	
12958	Hematocrit procedure	SHANK3	0.000095	3.507346	
12959	Hematocrit procedure	BTN2A3	0.000207	3.524927	
12960	Hematocrit procedure	KATNAL1	0	3.558586	
12961	Hematocrit procedure	ACTA2	0.000025	3.569658	
12962	Hematocrit procedure	VWDE	0.000006	3.608694	
12963	Hematocrit procedure	FLJ40113	0.000202	3.623957	
12964	Hematocrit procedure	GMPR	0.000117	3.634325	
12965	Hematocrit procedure	CUEDC2	0.000005	3.636746	
12966	Hematocrit procedure	EXOG	0.000269	3.658182	
12967	Hematocrit procedure	MYBL1	0.000015	3.678576	
12968	Hematocrit procedure	LOC201229	0.000041	3.693565	
12969	Hematocrit procedure	PPP3CA	0.000011	3.71873	
12970	Hematocrit procedure	LOC286440	0.000021	3.725239	
12971	Hematocrit procedure	LATS2	0.000002	3.740308	
12972	Hematocrit procedure	HECTD2	0.000301	3.764647	
12973	Hematocrit procedure	RAB6B	0.000009	3.773154	
12974	Hematocrit procedure	TSPAN4	0.000089	3.774971	
12975	Hematocrit procedure	ZNF280B	0.000144	3.80754	
12976	Hematocrit procedure	PPP2R3A	0.000143	3.81687	
12977	Hematocrit procedure	B3GNT4	0.000147	3.817794	
12978	Hematocrit procedure	CCDC136	0.000043	3.823805	
12979	Hematocrit procedure	DMKN	0.000003	3.83423	
12980	Hematocrit procedure	NMT2	0.000124	3.841508	
12981	Hematocrit procedure	RNF125	0.000073	3.89468	
12982	Hematocrit procedure	GLI2	0.000058	3.945516	
12983	Hematocrit procedure	MICB	0.000177	3.966082	
12984	Hematocrit procedure	CYR61	0.00002	4.046511	
12985	Hematocrit procedure	RPL22L1	0.002085	4.059774	
12986	Hematocrit procedure	IQCJ-SCHIP1	0.000032	4.075156	
12987	Hematocrit procedure	HRK	0.000005	4.082063	
12988	Hematocrit procedure	BEND7	0.000105	4.084679	
12989	Hematocrit procedure	NHEDC2	0.000076	4.100033	
12990	Hematocrit procedure	RASGRP1	0.000036	4.104226	
12991	Hematocrit procedure	TCF7L1	0.000005	4.135303	
12992	Hematocrit procedure	STOX2	0.000011	4.16124	
12993	Hematocrit procedure	PAQR7	0.000274	4.164603	
12994	Hematocrit procedure	N4BP2L2	0.000028	4.190006	
12995	Hematocrit procedure	MOSC2	0.000098	4.225067	
12996	Hematocrit procedure	ATP6AP1L	0.00001	4.29516	
12997	Hematocrit procedure	ADRBK2	0.000104	4.356837	
12998	Hematocrit procedure	AMZ2	0.000023	4.439814	

12999	Hematocrit procedure	MPP2	0.000017	4.450212	
13000	Hematocrit procedure	SNX10	0.000152	4.466907	
13001	Hematocrit procedure	FYCO1	0.000012	4.546863	
13002	Hematocrit procedure	MRC2	0	4.548474	
13003	Hematocrit procedure	EZR	0.000008	4.57178	
13004	Hematocrit procedure	ZNF204	0.000048	4.619756	
13005	Hematocrit procedure	EMID1	0.000209	4.622975	
13006	Hematocrit procedure	CTNNB1	0.000071	4.646194	
13007	Hematocrit procedure	GPR161	0.000053	4.663312	
13008	Hematocrit procedure	NUAK1	0	4.697471	
13009	Hematocrit procedure	DRAM1	0.000091	4.728488	
13010	Hematocrit procedure	HOMER3	0.00047	4.729957	
13011	Hematocrit procedure	DBP	0.000005	4.750197	
13012	Hematocrit procedure	TTC18	0.000014	4.779103	
13013	Hematocrit procedure	HNRNPH3	0.000111	4.787262	
13014	Hematocrit procedure	DRAM	0.000021	4.800351	
13015	Hematocrit procedure	KLHL5	0.000051	4.830654	
13016	Hematocrit procedure	CDGAP	0.000124	4.857344	
13017	Hematocrit procedure	SLC36A4	0.000253	4.881939	
13018	Hematocrit procedure	STARD9	0.000098	4.914114	
13019	Hematocrit procedure	FZD3	0.000009	5.022363	
13020	Hematocrit procedure	CCNG2	0.00001	5.12713	
13021	Hematocrit procedure	SCHIP1	0.000259	5.146005	
13022	Hematocrit procedure	KCNIP2	0.000285	5.252706	
13023	Hematocrit procedure	FLJ43276	0.000069	5.270924	
13024	Hematocrit procedure	SMURF2	0.000008	5.287704	
13025	Hematocrit procedure	CCDC46	0.00003	5.297978	
13026	Hematocrit procedure	BBS5	0.000036	5.299647	
13027	Hematocrit procedure	BEND6	0.000005	5.306019	
13028	Hematocrit procedure	SLC16A9	0.000163	5.333557	
13029	Hematocrit procedure	C6orf204	0.00008	5.372437	
13030	Hematocrit procedure	RNF141	0.000002	5.403097	
13031	Hematocrit procedure	PCDHA1	0.00001	5.429812	
13032	Hematocrit procedure	DENND2C	0.000007	5.481683	
13033	Hematocrit procedure	ASPHD1	0.00001	5.58353	
13034	Hematocrit procedure	POLN	0.000006	5.62979	
13035	Hematocrit procedure	PARD6G	0.000031	5.80562	
13036	Hematocrit procedure	LRRC8C	0.000016	5.817699	
13037	Hematocrit procedure	FAM7A1	0.000016	5.890325	
13038	Hematocrit procedure	MT1H	0.001681	5.914393	
13039	Hematocrit procedure	MLF1	0.00001	5.942251	
13040	Hematocrit procedure	NCS1	0.000008	5.978362	
13041	Hematocrit procedure	COL6A2	0.000005	6.07294	
13042	Hematocrit procedure	COL11A2	0.000016	6.081678	
13043	Hematocrit procedure	MID2	0.000003	6.122492	
13044	Hematocrit procedure	MAP1B	0.000008	6.150958	
13045	Hematocrit procedure	UTRN	0.000091	6.151177	
13046	Hematocrit procedure	PTPRU	0.000007	6.177564	
13047	Hematocrit procedure	SCN8A	0.000041	6.225373	
13048	Hematocrit procedure	DIP2C	0	6.344517	

13049	Hematocrit procedure	RASSF8	0.000153	6.447505	
13050	Hematocrit procedure	TMOD2	0.000008	6.46977	
13051	Hematocrit procedure	PSMG1	0.000091	6.486989	
13052	Hematocrit procedure	KIAA1920	0.000002	6.572179	
13053	Hematocrit procedure	AP1S3	0.000004	6.665484	
13054	Hematocrit procedure	PRKAR2B	0.000163	6.727933	
13055	Hematocrit procedure	CHRFAM7A	0.000021	6.761782	
13056	Hematocrit procedure	NEO1	0.000007	6.778429	
13057	Hematocrit procedure	DPYD	0.000008	6.845269	
13058	Hematocrit procedure	NAV3	0.000008	7.051791	
13059	Hematocrit procedure	CLDND1	0.000003	7.055015	
13060	Hematocrit procedure	EMP3	0.000002	7.129033	
13061	Hematocrit procedure	ANXA6	0.000111	7.199033	
13062	Hematocrit procedure	LOC51233	0	7.282324	
13063	Hematocrit procedure	RECK	0	7.333833	
13064	Hematocrit procedure	RHOQ	0.000001	7.383621	
13065	Hematocrit procedure	MYL9	0.000004	7.687039	
13066	Hematocrit procedure	GPR98	0.000043	7.791575	
13067	Hematocrit procedure	LOC643792	0.000011	7.85897	
13068	Hematocrit procedure	FLJ33996	0.000136	7.914756	
13069	Hematocrit procedure	PITPNC1	0.00001	7.945857	
13070	Hematocrit procedure	TTLL7	0.00022	7.996952	
13071	Hematocrit procedure	PKIG	0.000094	8.099702	
13072	Hematocrit procedure	LOC648795	0.00003	8.118847	
13073	Hematocrit procedure	LOC440302	0.000016	8.285117	
13074	Hematocrit procedure	PALLD	0.000005	8.486156	
13075	Hematocrit procedure	TTC7B	0.000076	8.657179	
13076	Hematocrit procedure	TLE2	0	8.702231	
13077	Hematocrit procedure	NME4	0.000007	9.047245	
13078	Hematocrit procedure	CFD	0.000004	9.061576	
13079	Hematocrit procedure	F2RL1	0.000003	9.207135	
13080	Hematocrit procedure	C9orf72	0.000004	10.188588	
13081	Hematocrit procedure	MARK1	0	10.217503	
13082	Hematocrit procedure	FAM7A3	0.000024	10.252432	
13083	Hematocrit procedure	KLK7	0	10.412573	
13084	Hematocrit procedure	CCNDBP1	0.000226	10.62795	
13085	Hematocrit procedure	GPX8	0.000005	10.645534	
13086	Hematocrit procedure	BDNF	0.000006	10.742728	
13087	Hematocrit procedure	NFIC	0.000009	10.751284	
13088	Hematocrit procedure	PTPN13	0.000013	11.195711	
13089	Hematocrit procedure	MFAP2	0.000003	11.296665	
13090	Hematocrit procedure	ZNF618	0.000012	11.312275	
13091	Hematocrit procedure	ADAP2	0	11.396105	
13092	Hematocrit procedure	RNF217	0.00002	11.55933	
13093	Hematocrit procedure	LAPTM4B	0	12.015325	
13094	Hematocrit procedure	MCOLN3	0.000004	12.019836	
13095	Hematocrit procedure	SYTL3	0.000004	12.586998	
13096	Hematocrit procedure	FBXO44	0.000015	12.898193	
13097	Hematocrit procedure	MT2A	0.000012	13.177214	
13098	Hematocrit procedure	SLC16A14	0.000003	13.373653	

13099	Hematocrit procedure	SMARCD3	0	15.594032	
13100	Hematocrit procedure	KLHL13	0.000003	15.91222	
13101	Hematocrit procedure	CYBRD1	0.000011	15.955871	
13102	Hematocrit procedure	KISS1R	0.000054	16.026044	
13103	Hematocrit procedure	MKX	0.000031	16.252809	
13104	Hematocrit procedure	CSAG2	0.000003	16.47408	
13105	Hematocrit procedure	EML1	0	16.504361	
13106	Hematocrit procedure	RBPMS2	0.000266	16.968109	
13107	Hematocrit procedure	SSFA2	0.000004	18.763377	
13108	Hematocrit procedure	PPP1R9A	0.000008	19.038244	
13109	Hematocrit procedure	IFITM3	0.000002	19.187446	
13110	Hematocrit procedure	RAB38	0	21.45702	
13111	Hematocrit procedure	NOV	0.000004	21.833935	
13112	Hematocrit procedure	FERMT2	0.000004	22.008593	
13113	Hematocrit procedure	ZNF385B	0.000001	23.716727	
13114	Hematocrit procedure	MAGEA12	0.000007	24.113279	
13115	Hematocrit procedure	MSI1	0.000005	24.721362	
13116	Hematocrit procedure	GPAT2	0.000007	25.125105	
13117	Hematocrit procedure	RRAGD	0.000011	25.191409	
13118	Hematocrit procedure	SCARA3	0.000003	25.93522	
13119	Hematocrit procedure	EPB41L2	0.000011	26.226904	
13120	Hematocrit procedure	SMARCA1	0.000004	27.270593	
13121	Hematocrit procedure	RAB12	0.000004	27.617611	
13122	Hematocrit procedure	UBE2E2	0.000004	29.738673	
13123	Hematocrit procedure	NR3C1	0.000003	31.321162	
13124	Hematocrit procedure	COL12A1	0.000005	31.924422	
13125	Hematocrit procedure	MEF2C	0	33.319674	
13126	Hematocrit procedure	ANXA10	0.000003	40.771601	
13127	Hematocrit procedure	TBC1D9	0.000002	41.505718	
13128	Hematocrit procedure	ANTXR1	0.000001	49.218279	
13129	Hematocrit procedure	FMNL2	0	54.602674	
13130	Hematocrit procedure	SELM	0.000003	60.753264	
13131	Hematocrit procedure	GLUL	0.000003	60.819892	
13132	Hematocrit procedure	PLA2G4A	0.000004	63.232318	
13133	Hematocrit procedure	PDP1	0	64.485389	
13134	Hematocrit procedure	PVRL3	0.000006	68.00041	
13135	Hematocrit procedure	WNT16	0.000003	72.070133	
13136	Hematocrit procedure	NPW	0.000004	75.322469	
13137	Hematocrit procedure	TMEM200A	0.000008	79.055071	
13138	Hematocrit procedure	PDE4B	0.000008	81.362804	
13139	Hematocrit procedure	CD55	0.000008	85.609602	
13140	Hematocrit procedure	ENAH	0.000001	87.225685	
13141	Hematocrit procedure	LOC399959	0.000008	91.493939	
13142	Hematocrit procedure	HBE1	0.000005	95.940684	
13143	Hematocrit procedure	SCG2	0.000007	97.219518	
13144	Hematocrit procedure	GPR110	0.00001	119.33262	
13145	Hematocrit procedure	MAGEA2	0.000003	195.042669	
13146	Hematocrit procedure	CALD1	0.000002	236.177902	
13147	Hematocrit procedure	GNAI1	0	249.518455	
13148	Hematocrit procedure	MAGEA3	0	286.243733	

13149	Hematocrit procedure	MAGEB2	0	317.484128	
13150	Hematocrit procedure	TUBA1A	0	362.306283	
13151	Hematocrit procedure	ALDH1A3	0.000006	365.306613	
13152	Hematocrit procedure	PCDH7	0.000001	387.281575	
13153	Hematocrit procedure	GTSF1	0.000008	399.301718	
13154	Hematocrit procedure	DKK1	0	561.358383	
13155	Hematocrit procedure	ARHGAP29	0.000004	574.399087	
13156	Hemorrhage	XIST	0	1.799884	
13157	Hereditary Malignant Neoplasm	NXF3	0.000001	-6.077497	
13158	Hereditary Malignant Neoplasm	TRIM7	0.000001	-4.303554	
13159	Hereditary Malignant Neoplasm	PLA2G3	0.001088	-3.170196	
13160	Hereditary Malignant Neoplasm	SEMG1	0.000982	-3.09763	
13161	Hereditary Malignant Neoplasm	AGR3	0	-2.876189	
13162	Hereditary Malignant Neoplasm	TFAP2A	0.000005	-2.665646	
13163	Hereditary Malignant Neoplasm	FOXA1	0.000001	-2.631543	
13164	Hereditary Malignant Neoplasm	GNLY	0.000026	-2.468766	
13165	Hereditary Malignant Neoplasm	HOXC6	0	-2.38922	
13166	Hereditary Malignant Neoplasm	C19orf51	0.000122	-2.378922	
13167	Hereditary Malignant Neoplasm	LILRA3	0.000194	-2.361116	
13168	Hereditary Malignant Neoplasm	TNFSF9	0.000129	-2.313163	
13169	Hereditary Malignant Neoplasm	PLAC8	0.000527	-2.301019	
13170	Hereditary Malignant Neoplasm	PBK	0	-2.2508	
13171	Hereditary Malignant Neoplasm	NUDT6	0.000072	-2.231892	
13172	Hereditary Malignant Neoplasm	CD164L2	0.000687	-2.217661	
13173	Hereditary Malignant Neoplasm	NOS2A	0.000687	-2.171185	
13174	Hereditary Malignant Neoplasm	PFKP	0.000063	-2.11219	
13175	Hereditary Malignant Neoplasm	LYPD5	0.00004	-2.106908	
13176	Hereditary Malignant Neoplasm	GZMA	0.000347	-2.097267	
13177	Hereditary Malignant Neoplasm	DEGS2	0.001158	-2.084829	
13178	Hereditary Malignant Neoplasm	C11orf9	0.000869	-2.081439	
13179	Hereditary Malignant Neoplasm	C19orf23	0.000238	-2.055148	
13180	Hereditary Malignant Neoplasm	GAD1	0.000302	-2.051646	
13181	Hereditary Malignant Neoplasm	GAR1	0.000026	-2.026844	
13182	Hereditary Malignant Neoplasm	PON3	0.000063	2.032295	
13183	Hereditary Malignant Neoplasm	POFUT1	0.000045	2.045593	
13184	Hereditary Malignant Neoplasm	CIB2	0.00111	2.052749	
13185	Hereditary Malignant Neoplasm	C20orf117	0.000266	2.068023	
13186	Hereditary Malignant Neoplasm	NOX1	0.001	2.082522	
13187	Hereditary Malignant Neoplasm	HOXA3	0.000247	2.119229	
13188	Hereditary Malignant Neoplasm	CEL	0.000912	2.141374	
13189	Hereditary Malignant Neoplasm	GRM8	0.000045	2.290591	
13190	Hereditary Malignant Neoplasm	PLA2G12B	0.000802	2.930102	
13191	Hereditary Malignant Neoplasm	KRT23	0.001	3.691753	
13192	Hereditary Non-Polyposis Colon Cancer Type 1	KRT23	0.01294	-3.193564	
13193	Hereditary Non-Polyposis Colon Cancer Type 1	SLC22A11	0.035716	-3.132747	
13194	Hereditary Non-Polyposis Colon Cancer Type 1	F7	0.009004	-3.058259	
13195	Hereditary Non-Polyposis Colon Cancer Type 1	PKLR	0.009334	-2.823042	
13196	Hereditary Non-Polyposis Colon Cancer Type 1	PPP2R2C	0.012359	-2.663028	
13197	Hereditary Non-Polyposis Colon Cancer Type 1	PIWIL1	0.03469	-2.641758	
13198	Hereditary Non-Polyposis Colon Cancer Type 1	NODAL	0.004931	-2.566286	

13199	Hereditary Non-Polyposis Colon Cancer Type 1	PAH	0.011581	-2.565458	
13200	Hereditary Non-Polyposis Colon Cancer Type 1	PLA2G12B	0.022296	-2.562045	
13201	Hereditary Non-Polyposis Colon Cancer Type 1	UMODL1	0.002468	-2.519158	
13202	Hereditary Non-Polyposis Colon Cancer Type 1	NOX1	0.000276	-2.476758	
13203	Hereditary Non-Polyposis Colon Cancer Type 1	GRM8	0.000276	-2.453255	
13204	Hereditary Non-Polyposis Colon Cancer Type 1	C6orf15	0.022668	-2.449986	
13205	Hereditary Non-Polyposis Colon Cancer Type 1	PCP4	0.010349	-2.336584	
13206	Hereditary Non-Polyposis Colon Cancer Type 1	GAS2	0.000014	-2.302761	
13207	Hereditary Non-Polyposis Colon Cancer Type 1	NKD1	0.003627	-2.224193	
13208	Hereditary Non-Polyposis Colon Cancer Type 1	KRT6B	0.028373	-2.206861	
13209	Hereditary Non-Polyposis Colon Cancer Type 1	CEL	0.019019	-2.072682	
13210	Hereditary Non-Polyposis Colon Cancer Type 1	PON3	0.000276	-2.042103	
13211	Hereditary Non-Polyposis Colon Cancer Type 1	RERGL	0.035657	-2.0279	
13212	Hereditary Non-Polyposis Colon Cancer Type 1	HOXA3	0.006324	-2.012612	
13213	Hereditary Non-Polyposis Colon Cancer Type 1	BNIP3	0.008021	2.029302	
13214	Hereditary Non-Polyposis Colon Cancer Type 1	KCNG3	0.038146	2.101529	
13215	Hereditary Non-Polyposis Colon Cancer Type 1	NCRNA00173	0.027758	2.148351	
13216	Hereditary Nonpolyposis Colorectal Neoplasms	KRT23	0.01294	-3.193564	
13217	Hereditary Nonpolyposis Colorectal Neoplasms	SLC22A11	0.035716	-3.132747	
13218	Hereditary Nonpolyposis Colorectal Neoplasms	F7	0.009004	-3.058259	
13219	Hereditary Nonpolyposis Colorectal Neoplasms	PKLR	0.009334	-2.823042	
13220	Hereditary Nonpolyposis Colorectal Neoplasms	PPP2R2C	0.012359	-2.663028	
13221	Hereditary Nonpolyposis Colorectal Neoplasms	PIWIL1	0.03469	-2.641758	
13222	Hereditary Nonpolyposis Colorectal Neoplasms	NODAL	0.004931	-2.566286	
13223	Hereditary Nonpolyposis Colorectal Neoplasms	PAH	0.011581	-2.565458	
13224	Hereditary Nonpolyposis Colorectal Neoplasms	PLA2G12B	0.022296	-2.562045	
13225	Hereditary Nonpolyposis Colorectal Neoplasms	UMODL1	0.002468	-2.519158	
13226	Hereditary Nonpolyposis Colorectal Neoplasms	NOX1	0.000276	-2.476758	
13227	Hereditary Nonpolyposis Colorectal Neoplasms	GRM8	0.000276	-2.453255	
13228	Hereditary Nonpolyposis Colorectal Neoplasms	C6orf15	0.022668	-2.449986	
13229	Hereditary Nonpolyposis Colorectal Neoplasms	PCP4	0.010349	-2.336584	
13230	Hereditary Nonpolyposis Colorectal Neoplasms	GAS2	0.000014	-2.302761	
13231	Hereditary Nonpolyposis Colorectal Neoplasms	NKD1	0.003627	-2.224193	
13232	Hereditary Nonpolyposis Colorectal Neoplasms	KRT6B	0.028373	-2.206861	
13233	Hereditary Nonpolyposis Colorectal Neoplasms	CEL	0.019019	-2.072682	
13234	Hereditary Nonpolyposis Colorectal Neoplasms	PON3	0.000276	-2.042103	
13235	Hereditary Nonpolyposis Colorectal Neoplasms	RERGL	0.035657	-2.0279	
13236	Hereditary Nonpolyposis Colorectal Neoplasms	HOXA3	0.006324	-2.012612	
13237	Hereditary Nonpolyposis Colorectal Neoplasms	BNIP3	0.008021	2.029302	
13238	Hereditary Nonpolyposis Colorectal Neoplasms	KCNG3	0.038146	2.101529	
13239	Hereditary Nonpolyposis Colorectal Neoplasms	NCRNA00173	0.027758	2.148351	
13240	Histopathologic Grade differentiation	LOC253264	0.008153	-37.865909	
13241	Histopathologic Grade differentiation	CHST11	0.025145	-23.192164	
13242	Histopathologic Grade differentiation	CHST15	0.000027	-18.940909	
13243	Histopathologic Grade differentiation	COL6A1	0.02114	-18.382448	
13244	Histopathologic Grade differentiation	MT1G	0.016225	-17.753374	
13245	Histopathologic Grade differentiation	FERMT2	0.007869	-17.250729	
13246	Histopathologic Grade differentiation	AKR1C4	0.005756	-16.076282	
13247	Histopathologic Grade differentiation	AKR1B1	0.004132	-14.721042	
13248	Histopathologic Grade differentiation	MAP1B	0.000104	-11.591654	

13249	Histopathologic Grade differentiation	PRF1	0.026553	-11.241086	
13250	Histopathologic Grade differentiation	BNIP3	0.02154	-9.573129	
13251	Histopathologic Grade differentiation	A1CF	0.030278	-9.053001	
13252	Histopathologic Grade differentiation	MUC1	0.032471	-8.474978	
13253	Histopathologic Grade differentiation	C3orf67	0.000123	-7.580945	
13254	Histopathologic Grade differentiation	HSPA12A	0.004076	-7.539433	
13255	Histopathologic Grade differentiation	MAOA	0.033587	-7.250366	
13256	Histopathologic Grade differentiation	LOC100288413	0.008586	-7.106915	
13257	Histopathologic Grade differentiation	ARHGEF10	0.009609	-6.871402	
13258	Histopathologic Grade differentiation	PTPRN2	0.037953	-6.863723	
13259	Histopathologic Grade differentiation	GRK5	0.031305	-6.729808	
13260	Histopathologic Grade differentiation	SLC39A5	0.029417	-6.704422	
13261	Histopathologic Grade differentiation	SESN3	0.043064	-6.566215	
13262	Histopathologic Grade differentiation	ESPN	0.005169	-6.53639	
13263	Histopathologic Grade differentiation	ZNF75A	0.011872	-6.492516	
13264	Histopathologic Grade differentiation	FGF9	0.013599	-6.47679	
13265	Histopathologic Grade differentiation	WASF3	0.004148	-6.447774	
13266	Histopathologic Grade differentiation	GGT8P	0.00551	-6.208695	
13267	Histopathologic Grade differentiation	PTPRS	0.013452	-5.796991	
13268	Histopathologic Grade differentiation	ZNF114	0.00264	-5.651843	
13269	Histopathologic Grade differentiation	PDE4A	0.000306	-5.516307	
13270	Histopathologic Grade differentiation	LOC91316	0.002145	-5.371458	
13271	Histopathologic Grade differentiation	NOS3	0.026338	-5.370638	
13272	Histopathologic Grade differentiation	PLIN2	0.016907	-5.28887	
13273	Histopathologic Grade differentiation	SYDE1	0.001308	-5.278165	
13274	Histopathologic Grade differentiation	ATP2A3	0.039947	-5.142808	
13275	Histopathologic Grade differentiation	TM4SF19	0.027907	-5.081657	
13276	Histopathologic Grade differentiation	ESF1	0.005882	-5.079711	
13277	Histopathologic Grade differentiation	ABLIM2	0.00801	-5.067568	
13278	Histopathologic Grade differentiation	AHRR	0.019963	-5.054049	
13279	Histopathologic Grade differentiation	GRIN2B	0.013798	-4.913585	
13280	Histopathologic Grade differentiation	SYT1	0.024843	-4.91192	
13281	Histopathologic Grade differentiation	HLA-DPB1	0.005882	-4.823096	
13282	Histopathologic Grade differentiation	EPHB2	0.019271	-4.790564	
13283	Histopathologic Grade differentiation	RASSF2	0.007846	-4.772242	
13284	Histopathologic Grade differentiation	LASS4	0.009769	-4.739372	
13285	Histopathologic Grade differentiation	SSTR1	0.036992	-4.738777	
13286	Histopathologic Grade differentiation	KCNAB2	0.008497	-4.725273	
13287	Histopathologic Grade differentiation	ARMC4	0.040299	-4.642121	
13288	Histopathologic Grade differentiation	SERPINI1	0.016342	-4.640182	
13289	Histopathologic Grade differentiation	CLDN15	0.033587	-4.63721	
13290	Histopathologic Grade differentiation	EMP3	0.007002	-4.56919	
13291	Histopathologic Grade differentiation	ZNF134	0.006982	-4.523203	
13292	Histopathologic Grade differentiation	TTYH2	0.00725	-4.485478	
13293	Histopathologic Grade differentiation	ARHGAP4	0.001865	-4.477802	
13294	Histopathologic Grade differentiation	LNP1	0.002634	-4.415655	
13295	Histopathologic Grade differentiation	CPPED1	0.007628	-4.414136	
13296	Histopathologic Grade differentiation	CLIP4	0.03694	-4.369098	
13297	Histopathologic Grade differentiation	COL6A2	0.023451	-4.34569	
13298	Histopathologic Grade differentiation	ZNF512	0.034731	-4.342236	

13299	Histopathologic Grade differentiation	PRPS2	0.008559	-4.324613	
13300	Histopathologic Grade differentiation	HDAC4	0.025457	-4.310722	
13301	Histopathologic Grade differentiation	ARAP3	0.005753	-4.304594	
13302	Histopathologic Grade differentiation	ITPRIPL1	0.027108	-4.214905	
13303	Histopathologic Grade differentiation	MSRB3	0.040078	-4.207933	
13304	Histopathologic Grade differentiation	C1orf61	0.024543	-4.1922	
13305	Histopathologic Grade differentiation	GCNT2	0.036962	-4.171809	
13306	Histopathologic Grade differentiation	ZDBF2	0.014768	-4.093295	
13307	Histopathologic Grade differentiation	OBSL1	0.041152	-4.032463	
13308	Histopathologic Grade differentiation	SYTL3	0.029295	-4.026876	
13309	Histopathologic Grade differentiation	TMEM45A	0.023014	-3.954123	
13310	Histopathologic Grade differentiation	CKB	0.032381	-3.887971	
13311	Histopathologic Grade differentiation	MCC	0.028644	-3.844278	
13312	Histopathologic Grade differentiation	MYT1	0.036238	-3.841417	
13313	Histopathologic Grade differentiation	MACC1	0.016257	-3.830158	
13314	Histopathologic Grade differentiation	NAV2	0.033281	-3.805845	
13315	Histopathologic Grade differentiation	B4GALNT1	0.028283	-3.795721	
13316	Histopathologic Grade differentiation	SNAI1	0.004793	-3.78132	
13317	Histopathologic Grade differentiation	RTDR1	0.000854	-3.774219	
13318	Histopathologic Grade differentiation	SMO	0.010648	-3.692875	
13319	Histopathologic Grade differentiation	TRMT12	0.011418	-3.619269	
13320	Histopathologic Grade differentiation	C20orf194	0.008142	-3.578665	
13321	Histopathologic Grade differentiation	FRMD4A	0.013394	-3.554957	
13322	Histopathologic Grade differentiation	LOC100128701	0.009055	-3.547076	
13323	Histopathologic Grade differentiation	B4GALNT4	0.001373	-3.529887	
13324	Histopathologic Grade differentiation	LOC286467	0.020403	-3.529359	
13325	Histopathologic Grade differentiation	FHL1	0.040489	-3.510914	
13326	Histopathologic Grade differentiation	OLFM1	0.043427	-3.405421	
13327	Histopathologic Grade differentiation	MGC3207	0.01372	-3.38984	
13328	Histopathologic Grade differentiation	HMGA2	0.028404	-3.340042	
13329	Histopathologic Grade differentiation	RAC2	0.003858	-3.309574	
13330	Histopathologic Grade differentiation	PEAR1	0.031116	-3.287253	
13331	Histopathologic Grade differentiation	DPYSL4	0.006208	-3.273479	
13332	Histopathologic Grade differentiation	AADAT	0.001012	-3.257492	
13333	Histopathologic Grade differentiation	KLF2	0.027738	-3.249612	
13334	Histopathologic Grade differentiation	DST	0.028653	-3.229441	
13335	Histopathologic Grade differentiation	CKMT1B	0.033219	-3.229426	
13336	Histopathologic Grade differentiation	RGPD1	0.028817	-3.204293	
13337	Histopathologic Grade differentiation	IFRD1	0.023079	-3.202562	
13338	Histopathologic Grade differentiation	ABCB6	0.043596	-3.199229	
13339	Histopathologic Grade differentiation	CCDC153	0.014322	-3.19386	
13340	Histopathologic Grade differentiation	GGT3P	0.020489	-3.158533	
13341	Histopathologic Grade differentiation	UNC13A	0.042705	-3.155158	
13342	Histopathologic Grade differentiation	ATN1	0.009915	-3.135751	
13343	Histopathologic Grade differentiation	PCLO	0.004132	-3.128338	
13344	Histopathologic Grade differentiation	HSD11B2	0.009725	-3.123884	
13345	Histopathologic Grade differentiation	CAPN6	0.032802	-3.113044	
13346	Histopathologic Grade differentiation	FLJ11151	0.003437	-3.0502	
13347	Histopathologic Grade differentiation	ANUBL1	0.020611	-3.035174	
13348	Histopathologic Grade differentiation	NLRP1	0.028383	-3.017563	

13349	Histopathologic Grade differentiation	NIN	0.027786	-3.015769	
13350	Histopathologic Grade differentiation	FGF3	0.012817	-2.990565	
13351	Histopathologic Grade differentiation	LOC643014	0.007368	-2.980847	
13352	Histopathologic Grade differentiation	IRX4	0.008801	-2.974759	
13353	Histopathologic Grade differentiation	SEMA6A	0.015628	-2.956109	
13354	Histopathologic Grade differentiation	DCP1B	0.007234	-2.952423	
13355	Histopathologic Grade differentiation	CUZD1	0.025845	-2.947615	
13356	Histopathologic Grade differentiation	LEPR	0.035524	-2.944764	
13357	Histopathologic Grade differentiation	PTMS	0.008879	-2.937181	
13358	Histopathologic Grade differentiation	DLG4	0.001121	-2.927324	
13359	Histopathologic Grade differentiation	DFNA5	0.006773	-2.915278	
13360	Histopathologic Grade differentiation	STX6	0.009915	-2.900236	
13361	Histopathologic Grade differentiation	MDK	0.021198	-2.894297	
13362	Histopathologic Grade differentiation	KCTD14	0.042718	-2.887407	
13363	Histopathologic Grade differentiation	PGM5P2	0.040185	-2.883112	
13364	Histopathologic Grade differentiation	LAYN	0.001336	-2.882634	
13365	Histopathologic Grade differentiation	PGM5	0.008447	-2.87976	
13366	Histopathologic Grade differentiation	ITGA7	0.00857	-2.857539	
13367	Histopathologic Grade differentiation	ZNF512B	0.006008	-2.853459	
13368	Histopathologic Grade differentiation	ZNF680	0.005839	-2.85288	
13369	Histopathologic Grade differentiation	CPEB4	0.001559	-2.841143	
13370	Histopathologic Grade differentiation	LOC126235	0.002112	-2.84049	
13371	Histopathologic Grade differentiation	DUSP9	0.004157	-2.833412	
13372	Histopathologic Grade differentiation	DNM3	0.032381	-2.799099	
13373	Histopathologic Grade differentiation	FARSB	0.0161	-2.792914	
13374	Histopathologic Grade differentiation	FAM131C	0.007119	-2.791825	
13375	Histopathologic Grade differentiation	L3MBTL3	0.038	-2.78939	
13376	Histopathologic Grade differentiation	FAM83F	0.022233	-2.788021	
13377	Histopathologic Grade differentiation	ANKRD53	0.009709	-2.782743	
13378	Histopathologic Grade differentiation	NEIL1	0.042313	-2.780295	
13379	Histopathologic Grade differentiation	CLGN	0.013521	-2.760418	
13380	Histopathologic Grade differentiation	TLR1	0.014957	-2.735952	
13381	Histopathologic Grade differentiation	BHLHE40	0.000853	-2.71711	
13382	Histopathologic Grade differentiation	IL6R	0.033058	-2.708157	
13383	Histopathologic Grade differentiation	USH1C	0.028884	-2.69668	
13384	Histopathologic Grade differentiation	ZNF323	0.035562	-2.667251	
13385	Histopathologic Grade differentiation	FAM167A	0.025073	-2.662123	
13386	Histopathologic Grade differentiation	VWA5A	0.001017	-2.654796	
13387	Histopathologic Grade differentiation	TET1	0.025162	-2.646146	
13388	Histopathologic Grade differentiation	TNXB	0.012499	-2.645106	
13389	Histopathologic Grade differentiation	TRPV6	0.008043	-2.634322	
13390	Histopathologic Grade differentiation	MMP14	0.04116	-2.632169	
13391	Histopathologic Grade differentiation	CBS	0.013784	-2.625631	
13392	Histopathologic Grade differentiation	GUCY1B2	0.013836	-2.625275	
13393	Histopathologic Grade differentiation	EVX1	0.012607	-2.617924	
13394	Histopathologic Grade differentiation	TPRG1	0.040209	-2.610256	
13395	Histopathologic Grade differentiation	KIF5A	0.033626	-2.600662	
13396	Histopathologic Grade differentiation	ABHD8	0.00063	-2.579723	
13397	Histopathologic Grade differentiation	ZBTB40	0.027738	-2.545984	
13398	Histopathologic Grade differentiation	JAKMIP3	0.028383	-2.541878	

13399	Histopathologic Grade differentiation	THEM5	0.005622	-2.539618	
13400	Histopathologic Grade differentiation	PREX1	0.025096	-2.533661	
13401	Histopathologic Grade differentiation	FLNC	0.041262	-2.531518	
13402	Histopathologic Grade differentiation	NXPH4	0.014629	-2.527925	
13403	Histopathologic Grade differentiation	PER3	0.013987	-2.520126	
13404	Histopathologic Grade differentiation	MDFI	0.007123	-2.513263	
13405	Histopathologic Grade differentiation	LOC441426	0.018551	-2.512	
13406	Histopathologic Grade differentiation	LTK	0.026317	-2.506235	
13407	Histopathologic Grade differentiation	ASCL5	0.012154	-2.496523	
13408	Histopathologic Grade differentiation	TBXAS1	0.01198	-2.492425	
13409	Histopathologic Grade differentiation	NBEA	0.012294	-2.491303	
13410	Histopathologic Grade differentiation	ENOX1	0.036851	-2.487519	
13411	Histopathologic Grade differentiation	PTPRE	0.008496	-2.486172	
13412	Histopathologic Grade differentiation	SIGLEC6	0.012802	-2.477504	
13413	Histopathologic Grade differentiation	TRAM2	0.043427	-2.461308	
13414	Histopathologic Grade differentiation	AP3B2	0.03778	-2.457357	
13415	Histopathologic Grade differentiation	PANX2	0.013448	-2.456609	
13416	Histopathologic Grade differentiation	TCEB2	0.031895	-2.443718	
13417	Histopathologic Grade differentiation	ARID3A	0.004672	-2.439908	
13418	Histopathologic Grade differentiation	AHI1	0.01448	-2.437804	
13419	Histopathologic Grade differentiation	C17orf51	0.011872	-2.422359	
13420	Histopathologic Grade differentiation	KLHDC8B	0.042535	-2.405243	
13421	Histopathologic Grade differentiation	ANKRD10	0.000183	-2.404043	
13422	Histopathologic Grade differentiation	ZNF273	0.008153	-2.398726	
13423	Histopathologic Grade differentiation	SPSB4	0.012607	-2.393359	
13424	Histopathologic Grade differentiation	SLC15A1	0.034731	-2.385036	
13425	Histopathologic Grade differentiation	ZMAT3	0.00771	-2.370959	
13426	Histopathologic Grade differentiation	FBXL14	0.000854	-2.369245	
13427	Histopathologic Grade differentiation	VPS53	0.006008	-2.36796	
13428	Histopathologic Grade differentiation	L1CAM	0.011974	-2.367031	
13429	Histopathologic Grade differentiation	TRIM9	0.028175	-2.363678	
13430	Histopathologic Grade differentiation	PHEX	0.036499	-2.363163	
13431	Histopathologic Grade differentiation	GGT2	0.017975	-2.359329	
13432	Histopathologic Grade differentiation	C17orf81	0.017824	-2.358031	
13433	Histopathologic Grade differentiation	NMNAT3	0.033391	-2.35604	
13434	Histopathologic Grade differentiation	MED10	0.03507	-2.350887	
13435	Histopathologic Grade differentiation	NBPF3	0.028404	-2.348766	
13436	Histopathologic Grade differentiation	FBXO17	0.012148	-2.347638	
13437	Histopathologic Grade differentiation	CLN5	0.040928	-2.343231	
13438	Histopathologic Grade differentiation	WHDC1L1	0.040457	-2.342218	
13439	Histopathologic Grade differentiation	IQCG	0.032869	-2.338604	
13440	Histopathologic Grade differentiation	NES	0.007869	-2.337123	
13441	Histopathologic Grade differentiation	TMED6	0.040209	-2.3353	
13442	Histopathologic Grade differentiation	KIF21B	0.00671	-2.328681	
13443	Histopathologic Grade differentiation	SCAMP5	0.014843	-2.32594	
13444	Histopathologic Grade differentiation	LOC283551	0.037922	-2.32451	
13445	Histopathologic Grade differentiation	CADPS2	0.015922	-2.304556	
13446	Histopathologic Grade differentiation	ADAMTS7	0.01112	-2.298841	
13447	Histopathologic Grade differentiation	FAM123A	0.006211	-2.297537	
13448	Histopathologic Grade differentiation	C2orf14	0.01198	-2.295711	

13449	Histopathologic Grade differentiation	FOXA3	0.030954	-2.295082	
13450	Histopathologic Grade differentiation	LCE1C	0.01305	-2.290654	
13451	Histopathologic Grade differentiation	ARL4A	0.007123	-2.284495	
13452	Histopathologic Grade differentiation	GBGT1	0.03811	-2.282766	
13453	Histopathologic Grade differentiation	CDADC1	0.008574	-2.282549	
13454	Histopathologic Grade differentiation	PIR	0.012294	-2.270394	
13455	Histopathologic Grade differentiation	ZEB1	0.028617	-2.265735	
13456	Histopathologic Grade differentiation	SEC31A	0.020211	-2.259838	
13457	Histopathologic Grade differentiation	FBXO27	0.014193	-2.259429	
13458	Histopathologic Grade differentiation	TMEFF1	0.001914	-2.25391	
13459	Histopathologic Grade differentiation	HCP5	0.038419	-2.250648	
13460	Histopathologic Grade differentiation	SOX18	0.009915	-2.249341	
13461	Histopathologic Grade differentiation	PNPLA3	0.002707	-2.245735	
13462	Histopathologic Grade differentiation	COL5A3	0.04019	-2.240643	
13463	Histopathologic Grade differentiation	LOC100131582	0.01305	-2.237252	
13464	Histopathologic Grade differentiation	CYB5B	0.013827	-2.233574	
13465	Histopathologic Grade differentiation	TERF2	0.001861	-2.224786	
13466	Histopathologic Grade differentiation	C22orf34	0.038	-2.216259	
13467	Histopathologic Grade differentiation	NELL2	0.039047	-2.215298	
13468	Histopathologic Grade differentiation	LOC284542	0.005942	-2.213846	
13469	Histopathologic Grade differentiation	LOC100133005	0.035395	-2.213144	
13470	Histopathologic Grade differentiation	ZNF350	0.004319	-2.200095	
13471	Histopathologic Grade differentiation	DAB2	0.006747	-2.193844	
13472	Histopathologic Grade differentiation	NUDT12	0.041563	-2.188496	
13473	Histopathologic Grade differentiation	SEC14L4	0.032718	-2.186528	
13474	Histopathologic Grade differentiation	LOC440338	0.001655	-2.185677	
13475	Histopathologic Grade differentiation	SMA4	0.011127	-2.185441	
13476	Histopathologic Grade differentiation	C10orf32	0.030082	-2.182572	
13477	Histopathologic Grade differentiation	IRX3	0.010331	-2.182127	
13478	Histopathologic Grade differentiation	ECHDC2	0.016115	-2.17972	
13479	Histopathologic Grade differentiation	CARKD	0.04147	-2.177246	
13480	Histopathologic Grade differentiation	C20orf94	0.019269	-2.174943	
13481	Histopathologic Grade differentiation	RTN1	0.032046	-2.17305	
13482	Histopathologic Grade differentiation	IFI27L2	0.031305	-2.169142	
13483	Histopathologic Grade differentiation	RASA4	0.001394	-2.168369	
13484	Histopathologic Grade differentiation	CES3	0.006602	-2.167399	
13485	Histopathologic Grade differentiation	TXNL4B	0.010129	-2.165419	
13486	Histopathologic Grade differentiation	HAPLN2	0.005842	-2.164992	
13487	Histopathologic Grade differentiation	ESPNP	0.004186	-2.162174	
13488	Histopathologic Grade differentiation	C1orf175	0.014483	-2.149121	
13489	Histopathologic Grade differentiation	DLEU1	0.004474	-2.145568	
13490	Histopathologic Grade differentiation	CCDC108	0.008701	-2.14138	
13491	Histopathologic Grade differentiation	TSPYL2	0.008237	-2.135386	
13492	Histopathologic Grade differentiation	SEPX1	0.003913	-2.132559	
13493	Histopathologic Grade differentiation	CLCN5	0.042308	-2.124566	
13494	Histopathologic Grade differentiation	GPNMB	0.028149	-2.123332	
13495	Histopathologic Grade differentiation	ADSL	0.04163	-2.117257	
13496	Histopathologic Grade differentiation	GEFT	0.0438	-2.11232	
13497	Histopathologic Grade differentiation	ZBTB46	0.040057	-2.104567	
13498	Histopathologic Grade differentiation	SSBP3	0.029421	-2.102732	

13499	Histopathologic Grade differentiation	ITGB4	0.013998	-2.095643	
13500	Histopathologic Grade differentiation	GPHA2	0.012999	-2.091295	
13501	Histopathologic Grade differentiation	P2RX7	0.02703	-2.079745	
13502	Histopathologic Grade differentiation	SLC22A1	0.003181	-2.076038	
13503	Histopathologic Grade differentiation	TMEM63C	0.040878	-2.070845	
13504	Histopathologic Grade differentiation	SEC22B	0.032277	-2.067543	
13505	Histopathologic Grade differentiation	DIS3	0.039037	-2.063113	
13506	Histopathologic Grade differentiation	RSRC1	0.032802	-2.062702	
13507	Histopathologic Grade differentiation	IKZF1	0.033846	-2.061818	
13508	Histopathologic Grade differentiation	CSNK2A1	0.025004	-2.057276	
13509	Histopathologic Grade differentiation	BBS10	0.024636	-2.05625	
13510	Histopathologic Grade differentiation	ALDH1A2	0.009433	-2.055429	
13511	Histopathologic Grade differentiation	PDZD7	0.003188	-2.053775	
13512	Histopathologic Grade differentiation	SRD5A1	0.022525	-2.053575	
13513	Histopathologic Grade differentiation	B3GNTL1	0.016148	-2.050805	
13514	Histopathologic Grade differentiation	ZNF569	0.027242	-2.046698	
13515	Histopathologic Grade differentiation	VGLL4	0.004693	-2.042269	
13516	Histopathologic Grade differentiation	PLCG2	0.03811	-2.041336	
13517	Histopathologic Grade differentiation	ANKRD5	0.014515	-2.040144	
13518	Histopathologic Grade differentiation	SDC3	0.004411	-2.0394	
13519	Histopathologic Grade differentiation	CISD1	0.032589	-2.036917	
13520	Histopathologic Grade differentiation	NUFIP1	0.003607	-2.031173	
13521	Histopathologic Grade differentiation	FGFR3	0.003981	-2.025636	
13522	Histopathologic Grade differentiation	RPS10	0.010176	-2.024502	
13523	Histopathologic Grade differentiation	LYNX1	0.010176	-2.02257	
13524	Histopathologic Grade differentiation	ZNF124	0.022161	-2.020978	
13525	Histopathologic Grade differentiation	ARHGAP20	0.038422	-2.020175	
13526	Histopathologic Grade differentiation	NOTCH1	0.011655	-2.019435	
13527	Histopathologic Grade differentiation	RNASE1	0.028336	-2.018029	
13528	Histopathologic Grade differentiation	MTRR	0.025096	-2.013575	
13529	Histopathologic Grade differentiation	C5orf35	0.002683	-2.013524	
13530	Histopathologic Grade differentiation	PIBF1	0.023169	-2.011467	
13531	Histopathologic Grade differentiation	MMP17	0.043276	-2.005543	
13532	Histopathologic Grade differentiation	ARID5B	0.033036	2.000468	
13533	Histopathologic Grade differentiation	TMEM91	0.008209	2.000854	
13534	Histopathologic Grade differentiation	EPHB4	0.005622	2.003675	
13535	Histopathologic Grade differentiation	KIAA1244	0.032417	2.003714	
13536	Histopathologic Grade differentiation	MYO1D	0.01395	2.004781	
13537	Histopathologic Grade differentiation	EXPH5	0.003329	2.005562	
13538	Histopathologic Grade differentiation	C15orf42	0.01198	2.018957	
13539	Histopathologic Grade differentiation	GIT2	0.012016	2.020047	
13540	Histopathologic Grade differentiation	RALGPS2	0.022825	2.024382	
13541	Histopathologic Grade differentiation	LOC728725	0.008196	2.029532	
13542	Histopathologic Grade differentiation	OAZ3	0.012099	2.039396	
13543	Histopathologic Grade differentiation	SCARB2	0.013417	2.042511	
13544	Histopathologic Grade differentiation	FLJ45949	0.001347	2.045498	
13545	Histopathologic Grade differentiation	HK1	0.033538	2.045737	
13546	Histopathologic Grade differentiation	GRLF1	0.000141	2.04598	
13547	Histopathologic Grade differentiation	SPINT1	0.005747	2.049275	
13548	Histopathologic Grade differentiation	C7orf44	0.008413	2.058893	

13549	Histopathologic Grade differentiation	RNF19B	0.009427	2.063531	
13550	Histopathologic Grade differentiation	RTKN2	0.009665	2.064106	
13551	Histopathologic Grade differentiation	DYNC2H1	0.02958	2.067276	
13552	Histopathologic Grade differentiation	FOSL2	0.00773	2.076551	
13553	Histopathologic Grade differentiation	HSD17B7	0.035036	2.078039	
13554	Histopathologic Grade differentiation	ELL3	0.00404	2.078055	
13555	Histopathologic Grade differentiation	ARHGEF17	0.035765	2.079253	
13556	Histopathologic Grade differentiation	MAK	0.029029	2.082775	
13557	Histopathologic Grade differentiation	PKP4	0.011288	2.084385	
13558	Histopathologic Grade differentiation	MAP2K1	0.001186	2.085403	
13559	Histopathologic Grade differentiation	TNFRSF12A	0.003462	2.087319	
13560	Histopathologic Grade differentiation	C10orf118	0.003238	2.088661	
13561	Histopathologic Grade differentiation	LCA5	0.028149	2.089859	
13562	Histopathologic Grade differentiation	COL24A1	0.031116	2.094635	
13563	Histopathologic Grade differentiation	ATP11B	0.003332	2.09744	
13564	Histopathologic Grade differentiation	WDR52	0.031817	2.098932	
13565	Histopathologic Grade differentiation	FAM83H	0.004088	2.100673	
13566	Histopathologic Grade differentiation	PKN2	0.025518	2.102949	
13567	Histopathologic Grade differentiation	VEGFA	0.002406	2.107409	
13568	Histopathologic Grade differentiation	LAMA3	0.041546	2.111382	
13569	Histopathologic Grade differentiation	HIATL1	0.001758	2.113762	
13570	Histopathologic Grade differentiation	GDE1	0.005622	2.118712	
13571	Histopathologic Grade differentiation	ZFYVE19	0.012029	2.127718	
13572	Histopathologic Grade differentiation	CHD2	0.009488	2.128848	
13573	Histopathologic Grade differentiation	DCPS	0.000868	2.133712	
13574	Histopathologic Grade differentiation	VPS13C	0.036308	2.140244	
13575	Histopathologic Grade differentiation	RNF180	0.0031	2.147948	
13576	Histopathologic Grade differentiation	PDK4	0.030654	2.151014	
13577	Histopathologic Grade differentiation	TTC17	0.010562	2.163514	
13578	Histopathologic Grade differentiation	STK17B	0.014467	2.167417	
13579	Histopathologic Grade differentiation	P4HA2	0.003607	2.170528	
13580	Histopathologic Grade differentiation	TMEM191A	0.00745	2.175649	
13581	Histopathologic Grade differentiation	TMEM170B	0.041546	2.19354	
13582	Histopathologic Grade differentiation	LCOR	0.002878	2.202271	
13583	Histopathologic Grade differentiation	DENND3	0.043798	2.203066	
13584	Histopathologic Grade differentiation	MUC4	0.001871	2.205676	
13585	Histopathologic Grade differentiation	LOC100129069	0.000267	2.206937	
13586	Histopathologic Grade differentiation	IFI27L1	0.007368	2.207626	
13587	Histopathologic Grade differentiation	B4GALT1	0.003332	2.22832	
13588	Histopathologic Grade differentiation	hCG_1783494	0.008153	2.232546	
13589	Histopathologic Grade differentiation	NR2F2	0.008574	2.254344	
13590	Histopathologic Grade differentiation	LOC388564	0.00801	2.274356	
13591	Histopathologic Grade differentiation	SHROOM3	0.011626	2.276485	
13592	Histopathologic Grade differentiation	HSH2D	0.029716	2.280942	
13593	Histopathologic Grade differentiation	USP53	0.011462	2.294196	
13594	Histopathologic Grade differentiation	USP34	0.01778	2.314252	
13595	Histopathologic Grade differentiation	ROCK1	0.022315	2.32363	
13596	Histopathologic Grade differentiation	ZNF321	0.007156	2.32491	
13597	Histopathologic Grade differentiation	FAM160A1	0.01112	2.35069	
13598	Histopathologic Grade differentiation	TSPAN13	0.011713	2.354123	

13599	Histopathologic Grade differentiation	C16orf75	0.009915	2.362487	
13600	Histopathologic Grade differentiation	APPL2	0.000066	2.376688	
13601	Histopathologic Grade differentiation	PDLIM1	0.008471	2.378194	
13602	Histopathologic Grade differentiation	C17orf67	0.036463	2.385931	
13603	Histopathologic Grade differentiation	LMO4	0.016045	2.412532	
13604	Histopathologic Grade differentiation	PIAS3	0.010123	2.426097	
13605	Histopathologic Grade differentiation	C6orf57	0.040796	2.439044	
13606	Histopathologic Grade differentiation	FLT3LG	0.000706	2.44382	
13607	Histopathologic Grade differentiation	PLAUR	0.014587	2.461881	
13608	Histopathologic Grade differentiation	AIM1	0.008659	2.473595	
13609	Histopathologic Grade differentiation	PNCK	0.000491	2.489765	
13610	Histopathologic Grade differentiation	DHRS9	0.001336	2.510497	
13611	Histopathologic Grade differentiation	ZBED5	0.035318	2.524241	
13612	Histopathologic Grade differentiation	CEP110	0.023699	2.537519	
13613	Histopathologic Grade differentiation	LIMS3	0.002489	2.558477	
13614	Histopathologic Grade differentiation	TTC6	0.015284	2.563837	
13615	Histopathologic Grade differentiation	LOC645381	0.007006	2.570939	
13616	Histopathologic Grade differentiation	SLCO5A1	0.00128	2.593504	
13617	Histopathologic Grade differentiation	ABL1	0.013206	2.61921	
13618	Histopathologic Grade differentiation	IRF6	0.002746	2.625752	
13619	Histopathologic Grade differentiation	KIAA0485	0.01411	2.635504	
13620	Histopathologic Grade differentiation	RARG	0.010199	2.635643	
13621	Histopathologic Grade differentiation	ADRB1	0.016634	2.638542	
13622	Histopathologic Grade differentiation	LOC100506748	0.006195	2.665503	
13623	Histopathologic Grade differentiation	FARP1	0.039876	2.679307	
13624	Histopathologic Grade differentiation	WLS	0.006195	2.703211	
13625	Histopathologic Grade differentiation	GRHL2	0.010176	2.720551	
13626	Histopathologic Grade differentiation	SH3RF2	0.008924	2.726288	
13627	Histopathologic Grade differentiation	MTHFD2L	0.001744	2.737343	
13628	Histopathologic Grade differentiation	LYSMD2	0.017063	2.757301	
13629	Histopathologic Grade differentiation	PRKD3	0.006949	2.761498	
13630	Histopathologic Grade differentiation	ITGB8	0.032802	2.767394	
13631	Histopathologic Grade differentiation	TIGD2	0.012807	2.787045	
13632	Histopathologic Grade differentiation	IL15RA	0.023241	2.807013	
13633	Histopathologic Grade differentiation	ATR	0.009124	2.834751	
13634	Histopathologic Grade differentiation	DUSP8	0.01992	2.881085	
13635	Histopathologic Grade differentiation	MPZL2	0.01198	2.910977	
13636	Histopathologic Grade differentiation	GPR160	0.002746	2.933982	
13637	Histopathologic Grade differentiation	SATB1	0.015439	2.951465	
13638	Histopathologic Grade differentiation	XDH	0.019852	2.9635	
13639	Histopathologic Grade differentiation	GAP43	0.043483	2.974488	
13640	Histopathologic Grade differentiation	SH3YL1	0.004381	2.99788	
13641	Histopathologic Grade differentiation	COBLL1	0.008605	3.005198	
13642	Histopathologic Grade differentiation	SGMS2	0.023583	3.031037	
13643	Histopathologic Grade differentiation	FBXO32	0.011127	3.031677	
13644	Histopathologic Grade differentiation	HIST2H2AA3	0.006195	3.086078	
13645	Histopathologic Grade differentiation	RASGRF2	0.015347	3.105836	
13646	Histopathologic Grade differentiation	C1orf21	0.020891	3.23382	
13647	Histopathologic Grade differentiation	TLE4	0.009549	3.286044	
13648	Histopathologic Grade differentiation	HOXA3	0.03074	3.319384	

13649	Histopathologic Grade differentiation	EHD2	0.040716	3.350242	
13650	Histopathologic Grade differentiation	TJP2	0.005622	3.414827	
13651	Histopathologic Grade differentiation	ZNF230	0.025457	3.436347	
13652	Histopathologic Grade differentiation	MGC45800	0.001509	3.584612	
13653	Histopathologic Grade differentiation	FAS	0.013987	3.656314	
13654	Histopathologic Grade differentiation	GNE	0.0243	3.767016	
13655	Histopathologic Grade differentiation	CHRM2	0.00745	3.773674	
13656	Histopathologic Grade differentiation	FOXP1	0.036097	3.825506	
13657	Histopathologic Grade differentiation	ESAM	0.034993	3.828741	
13658	Histopathologic Grade differentiation	TLR3	0.001097	3.836039	
13659	Histopathologic Grade differentiation	PLEKHG1	0.001094	3.850135	
13660	Histopathologic Grade differentiation	SAMD4A	0.011654	3.85908	
13661	Histopathologic Grade differentiation	KIAA1217	0.001509	3.912757	
13662	Histopathologic Grade differentiation	FAM13A	0.015224	4.162173	
13663	Histopathologic Grade differentiation	CABP1	0.033636	4.197124	
13664	Histopathologic Grade differentiation	RASEF	0.001097	4.208167	
13665	Histopathologic Grade differentiation	SAMD12	0.009379	4.472821	
13666	Histopathologic Grade differentiation	SYDE2	0.005995	4.54929	
13667	Histopathologic Grade differentiation	FAM107B	0.002878	4.641924	
13668	Histopathologic Grade differentiation	TNFRSF11A	0.036371	4.785876	
13669	Histopathologic Grade differentiation	C1orf133	0.00745	4.963146	
13670	Histopathologic Grade differentiation	FAM129A	0.003384	4.982378	
13671	Histopathologic Grade differentiation	KIAA0020	0.000758	4.991233	
13672	Histopathologic Grade differentiation	FGD4	0.039475	5.021743	
13673	Histopathologic Grade differentiation	SGPP2	0.008002	5.209606	
13674	Histopathologic Grade differentiation	TESC	0.033046	5.308697	
13675	Histopathologic Grade differentiation	NTN4	0.001303	5.395852	
13676	Histopathologic Grade differentiation	ETV7	0.009436	5.484766	
13677	Histopathologic Grade differentiation	SERTAD4	0.005622	6.02577	
13678	Histopathologic Grade differentiation	FGFR2	0.014514	6.16505	
13679	Histopathologic Grade differentiation	ZNF776	0.02958	6.171461	
13680	Histopathologic Grade differentiation	MGC10981	0.025518	6.343178	
13681	Histopathologic Grade differentiation	STAP2	0.000223	7.021882	
13682	Histopathologic Grade differentiation	CD55	0.027523	7.103775	
13683	Histopathologic Grade differentiation	NPNT	0.003534	7.179623	
13684	Histopathologic Grade differentiation	TRPS1	0.012266	11.776154	
13685	Histopathologic Grade differentiation	ODZ3	0.032373	11.8673	
13686	Histopathologic Grade differentiation	AFAP1-AS	0.013448	15.305128	
13687	Histopathologic Grade differentiation	PAPSS2	0.00404	16.135591	
13688	HMGA1b Protein	HMGA1	0.000094	-9.960206	
13689	HMGA1b Protein	EIF4EBP2	0.000549	-2.475915	
13690	HMGA1b Protein	LZTS1	0.000003	-2.240321	
13691	HMGA1b Protein	MAL2	0.000794	-2.098098	
13692	HMGA1b Protein	CDK4	0.001899	-2.075749	
13693	HMGA1b Protein	GLS2	0.0029	-2.02811	
13694	HMGA1b Protein	C19orf42	0.000009	-2.000794	
13695	HMGA1c Protein	HMGA1	0.000094	-9.960206	
13696	HMGA1c Protein	EIF4EBP2	0.000549	-2.475915	
13697	HMGA1c Protein	LZTS1	0.000003	-2.240321	
13698	HMGA1c Protein	MAL2	0.000794	-2.098098	

13699	HMGA1c Protein	CDK4	0.001899	-2.075749	
13700	HMGA1c Protein	GLS2	0.0029	-2.02811	
13701	HMGA1c Protein	C19orf42	0.000009	-2.000794	
13702	Human cells	C9orf125	0.000072	-1298.37944	
13703	Human cells	CNKSR3	0.000316	-124.830849	
13704	Human cells	FAM120B	0.000075	-100.720277	
13705	Human cells	MID1	0.000445	-92.787503	
13706	Human cells	ABAT	0.000333	-63.352896	
13707	Human cells	CADPS2	0.000355	-58.269955	
13708	Human cells	HIPK2	0.000291	-53.636186	
13709	Human cells	MLXIPL	0.000135	-49.95375	
13710	Human cells	ATP10D	0.000282	-27.855315	
13711	Human cells	EPHX2	0.000521	-17.745048	
13712	Human cells	BHLHE40	0.000286	-16.543643	
13713	Human cells	IL27RA	0.000402	-13.902396	
13714	Human cells	LOC100132273	0.000004	-13.370863	
13715	Human cells	CCDC146	0.000022	-11.056943	
13716	Human cells	FAM83F	0.000419	-10.407689	
13717	Human cells	SAT2	0.000099	-10.402509	
13718	Human cells	DTNB	0.000445	-10.209464	
13719	Human cells	CYP1B1	0	-9.781122	
13720	Human cells	FUT8	0.000282	-9.051947	
13721	Human cells	BIK	0.000004	-8.498466	
13722	Human cells	HACE1	0.000417	-8.496176	
13723	Human cells	ABCA5	0.00005	-8.236745	
13724	Human cells	KIAA1211	0.000154	-8.170336	
13725	Human cells	LYN	0.000005	-8.015746	
13726	Human cells	DNHD1	0.00009	-7.832598	
13727	Human cells	IQCG	0.000154	-7.705877	
13728	Human cells	LOC100130357	0.000038	-7.663775	
13729	Human cells	PLS3	0.00009	-7.214046	
13730	Human cells	CPSF2	0.000061	-6.975072	
13731	Human cells	TCEAL2	0.000006	-6.859401	
13732	Human cells	UGT1A1	0	-6.315951	
13733	Human cells	ZFP14	0.000424	-6.185132	
13734	Human cells	ITPR1	0.00003	-6.173306	
13735	Human cells	RABL4	0.000209	-5.953176	
13736	Human cells	FMNL2	0.000204	-5.762469	
13737	Human cells	JAG1	0.000087	-5.705999	
13738	Human cells	NT5M	0.000521	-5.666996	
13739	Human cells	FAM69A	0.000268	-5.60504	
13740	Human cells	CDADC1	0.000193	-5.542345	
13741	Human cells	ANKRD42	0.000082	-5.496973	
13742	Human cells	HDDC2	0.000414	-5.463333	
13743	Human cells	CYP4V2	0.000035	-5.342829	
13744	Human cells	IGLL3	0.000497	-5.135458	
13745	Human cells	RTDR1	0.000423	-5.050415	
13746	Human cells	HSPA4L	0	-5.000249	
13747	Human cells	ACOX3	0.000135	-5.000085	
13748	Human cells	STX6	0.000154	-4.962607	

13749	Human cells	MSI2	0.000015	-4.960336	
13750	Human cells	GBE1	0.000373	-4.92179	
13751	Human cells	NIN	0.000024	-4.790656	
13752	Human cells	BBS9	0.000093	-4.738447	
13753	Human cells	PRODH	0.000213	-4.705215	
13754	Human cells	ZBTB47	0.000521	-4.669141	
13755	Human cells	SLC2A4	0.000278	-4.512144	
13756	Human cells	OR4N4	0.000204	-4.395892	
13757	Human cells	CGGBP1	0.000061	-4.315759	
13758	Human cells	LOC728448	0.000234	-4.251742	
13759	Human cells	TBXAS1	0.000237	-4.081353	
13760	Human cells	MAPRE2	0.000193	-4.080819	
13761	Human cells	SAT1	0.000035	-4.017131	
13762	Human cells	SH3BGR	0.000209	-3.931459	
13763	Human cells	USP13	0.000208	-3.858849	
13764	Human cells	MTERFD2	0.000193	-3.831917	
13765	Human cells	LOC100134119	0.000393	-3.788561	
13766	Human cells	PRPS2	0.000025	-3.77657	
13767	Human cells	LOC442270	0.000058	-3.748188	
13768	Human cells	ACOT9	0.000039	-3.725177	
13769	Human cells	RSL1D1	0.000011	-3.713423	
13770	Human cells	SMA4	0.000392	-3.705228	
13771	Human cells	LOC157562	0.000135	-3.527667	
13772	Human cells	MYC	0.0005	-3.515492	
13773	Human cells	C1orf226	0.000353	-3.482497	
13774	Human cells	EXDL2	0.000144	-3.428238	
13775	Human cells	LOH12CR1	0.0001	-3.425623	
13776	Human cells	COBL	0.000144	-3.413096	
13777	Human cells	AHI1	0.000064	-3.345557	
13778	Human cells	LOC147645	0.000007	-3.257132	
13779	Human cells	ZCCHC7	0.000295	-3.231801	
13780	Human cells	ACSL3	0	-3.20428	
13781	Human cells	ZNF557	0.000119	-3.195714	
13782	Human cells	RBKS	0.000067	-3.187384	
13783	Human cells	FBXW2	0.000316	-3.154812	
13784	Human cells	KLHL15	0.000521	-3.15467	
13785	Human cells	LOC440396	0.000355	-3.064023	
13786	Human cells	SH2B2	0.000475	-3.059526	
13787	Human cells	PCSK5	0.000459	-3.056948	
13788	Human cells	NFYB	0	-3.033535	
13789	Human cells	SPATA7	0.000389	-3.028222	
13790	Human cells	UFSP2	0.000509	-3.020012	
13791	Human cells	RNU12	0.000261	-3.00526	
13792	Human cells	PIGH	0.000162	-2.993498	
13793	Human cells	ITM2C	0.000255	-2.993242	
13794	Human cells	ETFDH	0.000246	-2.961829	
13795	Human cells	APOO	0.000091	-2.909463	
13796	Human cells	C17orf45	0.000282	-2.883876	
13797	Human cells	POLR3F	0	-2.861953	
13798	Human cells	MRPL18	0	-2.842184	

13799	Human cells	MID1IP1	0.00003	-2.824787	
13800	Human cells	RMND1	0.000507	-2.81366	
13801	Human cells	ZNF18	0.000381	-2.794412	
13802	Human cells	RPGR	0.000351	-2.783919	
13803	Human cells	RNF130	0.000087	-2.773257	
13804	Human cells	C14orf100	0.000237	-2.7582	
13805	Human cells	CDC42EP1	0.000162	-2.728591	
13806	Human cells	MRPL34	0.000218	-2.717989	
13807	Human cells	SMCR7L	0.0001	-2.68474	
13808	Human cells	MAGOH	0	-2.677711	
13809	Human cells	DIRC1	0.000377	-2.673842	
13810	Human cells	FARP2	0.000187	-2.66268	
13811	Human cells	PAIP2B	0.000487	-2.655846	
13812	Human cells	TMTC4	0.000482	-2.654245	
13813	Human cells	HIST1H4B	0.000509	-2.626881	
13814	Human cells	PRDX4	0.000167	-2.617971	
13815	Human cells	CD97	0.000521	-2.587175	
13816	Human cells	ZNF409	0.000143	-2.582499	
13817	Human cells	TDP1	0.000402	-2.556651	
13818	Human cells	LIN52	0.000061	-2.555914	
13819	Human cells	LOC344967	0.000492	-2.544458	
13820	Human cells	AP4S1	0	-2.535025	
13821	Human cells	HSD17B11	0.0001	-2.534032	
13822	Human cells	ACOT7	0.000402	-2.498078	
13823	Human cells	C12orf57	0.000424	-2.492894	
13824	Human cells	C6orf35	0.000428	-2.480078	
13825	Human cells	SMARCE1	0	-2.470837	
13826	Human cells	BBS10	0	-2.458878	
13827	Human cells	ASTN2	0.000389	-2.440821	
13828	Human cells	HIST1H3G	0	-2.43851	
13829	Human cells	EIF3L	0.000268	-2.438209	
13830	Human cells	MEGF9	0.000161	-2.437944	
13831	Human cells	LOC643220	0.000286	-2.425949	
13832	Human cells	PDSS2	0.00044	-2.412645	
13833	Human cells	HIGD1A	0.000082	-2.411246	
13834	Human cells	NGDN	0.000014	-2.40989	
13835	Human cells	VDAC1	0.00036	-2.403048	
13836	Human cells	STXBP1	0.000521	-2.400944	
13837	Human cells	MR1	0.000082	-2.400894	
13838	Human cells	TLE3	0.000159	-2.393145	
13839	Human cells	EIF4B	0.00033	-2.387684	
13840	Human cells	CNGB1	0	-2.383365	
13841	Human cells	TNRC6B	0.000456	-2.383205	
13842	Human cells	COPZ1	0.000413	-2.373177	
13843	Human cells	FH	0	-2.353813	
13844	Human cells	C14orf126	0.000242	-2.350964	
13845	Human cells	DENND1A	0.000355	-2.342864	
13846	Human cells	ATG4A	0.000394	-2.333514	
13847	Human cells	MAFB	0.00033	-2.314435	
13848	Human cells	ZNF318	0.000124	-2.312317	

13849	Human cells	ACO2	0.000234	-2.277367	
13850	Human cells	LOC100190939	0.000113	-2.258036	
13851	Human cells	CDS2	0.000258	-2.256708	
13852	Human cells	LOC120364	0.000424	-2.256464	
13853	Human cells	IMPDH2	0.000492	-2.256004	
13854	Human cells	GRPEL1	0.000521	-2.240109	
13855	Human cells	CMC1	0.000309	-2.22477	
13856	Human cells	ARID1B	0.000099	-2.221163	
13857	Human cells	ZFYVE1	0.000278	-2.219254	
13858	Human cells	ACAD10	0.000112	-2.180166	
13859	Human cells	C9orf91	0.000268	-2.17898	
13860	Human cells	E2F2	0.000521	-2.175628	
13861	Human cells	TTLL3	0.000297	-2.156659	
13862	Human cells	PDXP	0.000006	-2.13219	
13863	Human cells	FAM125A	0.000355	-2.102634	
13864	Human cells	UQCRC1	0.000011	-2.101209	
13865	Human cells	C1QBP	0.000283	-2.075906	
13866	Human cells	TIMM44	0.000024	-2.066057	
13867	Human cells	CDK7	0	-2.053379	
13868	Human cells	JMJD6	0.000521	-2.044773	
13869	Human cells	RNMTL1	0.000277	-2.043078	
13870	Human cells	LOC148709	0.000354	-2.03822	
13871	Human cells	CNTROB	0.000316	-2.030106	
13872	Human cells	SF3B5	0.000414	-2.006777	
13873	Human cells	HIST1H1T	0	2.001387	
13874	Human cells	LASP1	0.000001	2.002258	
13875	Human cells	MOXD1	0	2.002775	
13876	Human cells	EHBP1L1	0.000208	2.003314	
13877	Human cells	PKLR	0	2.004163	
13878	Human cells	GRINA	0.000204	2.011493	
13879	Human cells	TIAM1	0	2.012516	
13880	Human cells	FCAR	0	2.020903	
13881	Human cells	FXYD1	0	2.02511	
13882	Human cells	IGLV1-40	0	2.026514	
13883	Human cells	CPE	0	2.026514	
13884	Human cells	RBM26	0	2.027919	
13885	Human cells	CD8A	0	2.029325	
13886	Human cells	FCER1G	0	2.03214	
13887	Human cells	RASL11B	0	2.033549	
13888	Human cells	GLP1R	0	2.039195	
13889	Human cells	LOC730286	0.000146	2.042263	
13890	Human cells	MICALL1	0.000242	2.04526	
13891	Human cells	LOC728769	0.000371	2.047471	
13892	Human cells	IL1B	0	2.049114	
13893	Human cells	CD53	0	2.066229	
13894	Human cells	PLA2G2D	0	2.066229	
13895	Human cells	IGHG1	0	2.07053	
13896	Human cells	ITGB1BP3	0	2.073402	
13897	Human cells	CD5L	0	2.086377	
13898	Human cells	MFSD10	0	2.087086	

13899	Human cells	SIGLEC7	0	2.089272	
13900	Human cells	AIM2	0	2.089272	
13901	Human cells	NDN	0	2.095072	
13902	Human cells	FBN1	0	2.096525	
13903	Human cells	STC1	0	2.100889	
13904	Human cells	NOS1	0	2.106722	
13905	Human cells	CXorf40B	0.000486	2.111282	
13906	Human cells	PAK3	0	2.112571	
13907	Human cells	GJB4	0	2.122846	
13908	Human cells	RNH1	0.000316	2.124698	
13909	Human cells	NAPRT1	0.000167	2.127462	
13910	Human cells	TAZ	0.000143	2.133776	
13911	Human cells	ADPRH	0	2.140577	
13912	Human cells	ZNF460	0	2.150989	
13913	Human cells	GPR161	0	2.16295	
13914	Human cells	LOC731275	0.000002	2.163543	
13915	Human cells	CYP2B7P1	0	2.16595	
13916	Human cells	LMOD1	0	2.174977	
13917	Human cells	LOC401357	0	2.17545	
13918	Human cells	DENND1C	0	2.181015	
13919	Human cells	C8A	0	2.188587	
13920	Human cells	RGSL1	0	2.191624	
13921	Human cells	MMP13	0	2.191624	
13922	Human cells	CARD14	0	2.194664	
13923	Human cells	CTSW	0	2.208398	
13924	Human cells	ECRP	0	2.216065	
13925	Human cells	RASSF4	0	2.229932	
13926	Human cells	CYP4A11	0	2.236124	
13927	Human cells	LRP4	0	2.237674	
13928	Human cells	ETS1	0	2.240778	
13929	Human cells	PDGFA	0	2.254801	
13930	Human cells	FAM3A	0.000237	2.271018	
13931	Human cells	NRG1	0	2.272059	
13932	Human cells	CCDC19	0.000041	2.280748	
13933	Human cells	ITGA10	0	2.283109	
13934	Human cells	JAK3	0	2.286277	
13935	Human cells	OR2S2	0	2.287862	
13936	Human cells	CMKLR1	0	2.306971	
13937	Human cells	RASSF7	0.000148	2.313444	
13938	Human cells	CACNA1A	0	2.316586	
13939	Human cells	SCGN	0	2.327853	
13940	Human cells	DAO	0	2.335935	
13941	Human cells	PRLR	0	2.340797	
13942	Human cells	ERGIC3	0	2.34242	
13943	Human cells	KIAA1881	0.000237	2.349848	
13944	Human cells	PAX6	0	2.355446	
13945	Human cells	ORAI2	0.000355	2.368232	
13946	Human cells	TCF4	0	2.370186	
13947	Human cells	CX3CL1	0	2.388326	
13948	Human cells	SORBS1	0	2.39164	

13949	Human cells	TEAD3	0.000061	2.404121	
13950	Human cells	HCLS1	0	2.431759	
13951	Human cells	TMEM184A	0.000061	2.443268	
13952	Human cells	SP140	0.000246	2.444219	
13953	Human cells	ACRV1	0	2.450371	
13954	Human cells	SULF1	0	2.45207	
13955	Human cells	MLLT6	0.000005	2.453535	
13956	Human cells	SPIN2A	0	2.45377	
13957	Human cells	GON4L	0	2.45377	
13958	Human cells	WDR1	0	2.463996	
13959	Human cells	SLC25A4	0	2.500125	
13960	Human cells	IRAK1	0.000428	2.500215	
13961	Human cells	ACTG2	0	2.512285	
13962	Human cells	PSG9	0	2.517514	
13963	Human cells	GPRC5C	0.000292	2.527811	
13964	Human cells	LOC100507666	0	2.538542	
13965	Human cells	SFXN5	0.000297	2.594348	
13966	Human cells	tcag7.907	0	2.602368	
13967	Human cells	HEATR7A	0.000492	2.614327	
13968	Human cells	GPR124	0	2.63536	
13969	Human cells	GLIPR1	0.000011	2.636184	
13970	Human cells	MEF2C	0	2.648178	
13971	Human cells	SLC10A1	0	2.685145	
13972	Human cells	PRB4	0	2.730187	
13973	Human cells	AKT1S1	0.000204	2.736527	
13974	Human cells	ERG	0	2.739666	
13975	Human cells	MDM2	0	2.745369	
13976	Human cells	C21orf71	0.000014	2.746564	
13977	Human cells	CTSD	0.000072	2.758571	
13978	Human cells	MOBKL2C	0.000413	2.821208	
13979	Human cells	MLL	0	2.840215	
13980	Human cells	SLC9A3R2	0.000054	2.842996	
13981	Human cells	RBM5	0	2.871889	
13982	Human cells	IQCE	0.000359	2.901126	
13983	Human cells	LPP	0	2.911979	
13984	Human cells	C4orf34	0	2.954676	
13985	Human cells	PDGFRL	0	2.96288	
13986	Human cells	DCN	0	3.025136	
13987	Human cells	TNFRSF21	0.000066	3.029186	
13988	Human cells	PHLDA3	0.000037	3.070606	
13989	Human cells	TINAGL1	0.000151	3.103956	
13990	Human cells	NACAP1	0	3.123146	
13991	Human cells	CXCR3	0	3.133989	
13992	Human cells	ITGA3	0.000291	3.174543	
13993	Human cells	HBG1	0	3.177738	
13994	Human cells	CTAG1A	0	3.18877	
13995	Human cells	PDGFRA	0	3.190981	
13996	Human cells	MYL4	0	3.21095	
13997	Human cells	C22orf29	0.000124	3.214952	
13998	Human cells	PGK2	0	3.224331	

13999	Human cells	UNC93B1	0.000002	3.228436	
14000	Human cells	CSNK1G1	0	3.228804	
14001	Human cells	KLHL35	0.000033	3.235531	
14002	Human cells	SPPL2B	0	3.244509	
14003	Human cells	SRCAP	0.000216	3.28083	
14004	Human cells	CDH11	0	3.282966	
14005	Human cells	RXRA	0.000037	3.293291	
14006	Human cells	CTNNB1	0	3.310387	
14007	Human cells	PRKD2	0.000124	3.334099	
14008	Human cells	FLRT2	0	3.335724	
14009	Human cells	TRIM56	0.000414	3.341161	
14010	Human cells	PLEKHA4	0.000011	3.432111	
14011	Human cells	KLHDC10	0	3.434262	
14012	Human cells	C14orf115	0	3.437692	
14013	Human cells	PTPRH	0.000291	3.466	
14014	Human cells	TMEM106A	0.000004	3.534425	
14015	Human cells	SSH3	0	3.644911	
14016	Human cells	PCSK6	0.000497	3.670522	
14017	Human cells	RGNEF	0.000156	3.713951	
14018	Human cells	ATP10B	0	3.810552	
14019	Human cells	CA9	0.000291	3.905632	
14020	Human cells	TNNI2	0.000011	3.944015	
14021	Human cells	BASP1	0	3.994459	
14022	Human cells	ZCCHC24	0	4.008326	
14023	Human cells	NLRC5	0.000204	4.032659	
14024	Human cells	LPHN1	0	4.041806	
14025	Human cells	SERPINE1	0.000061	4.1164	
14026	Human cells	DMBX1	0.000226	4.161581	
14027	Human cells	DNM1	0.000359	4.312468	
14028	Human cells	KIAA1659	0	4.371111	
14029	Human cells	PVRL1	0.000522	4.458198	
14030	Human cells	ATP6V1B1	0.000074	4.572513	
14031	Human cells	PTGS2	0.000139	4.634416	
14032	Human cells	SERPINF1	0	4.730524	
14033	Human cells	TYMP	0.000003	4.981838	
14034	Human cells	ABLIM3	0.000198	5.087636	
14035	Human cells	PPFIBP1	0	5.115942	
14036	Human cells	RHBDF1	0.000237	5.261642	
14037	Human cells	FAP	0	5.300028	
14038	Human cells	ITM2A	0	5.381469	
14039	Human cells	TRPS1	0.000394	5.397774	
14040	Human cells	HNF1B	0.000037	5.861257	
14041	Human cells	GATA2	0.000049	5.893886	
14042	Human cells	MUPCDH	0.000494	5.977978	
14043	Human cells	WNT10A	0.000038	6.06077	
14044	Human cells	LRG1	0.000309	6.245968	
14045	Human cells	HLA-DRB1	0	6.263634	
14046	Human cells	AGRN	0.000001	6.749664	
14047	Human cells	ETV7	0.000059	7.214569	
14048	Human cells	LGALS9	0.000378	7.278245	

14049	Human cells	C6orf145	0.000087	7.289675	
14050	Human cells	MMRN2	0.000099	7.512883	
14051	Human cells	TBL1X	0	7.594737	
14052	Human cells	LGALS9C	0.000155	8.105818	
14053	Human cells	DHRS9	0.000282	8.203724	
14054	Human cells	UPK3A	0.000419	8.411729	
14055	Human cells	TRIM29	0	9.058532	
14056	Human cells	RAB7B	0.000304	9.350441	
14057	Human cells	OLFML2B	0	10.253183	
14058	Human cells	FAM3D	0.00003	10.343878	
14059	Human cells	IFI27	0.000005	11.248729	
14060	Human cells	IFI6	0	20.704685	
14061	Incised wound	COL1A1	0	-5.27529	
14062	Incised wound	MMP7	0	-4.132738	
14063	Incised wound	SPP1	0	-4.130239	
14064	Incised wound	IL8	0	-3.187774	
14065	Incised wound	KRT23	0	-3.15084	
14066	Incised wound	COL1A2	0	-3.116379	
14067	Incised wound	TGFBI	0	-2.936139	
14068	Incised wound	INHBA	0	-2.666466	
14069	Incised wound	HKDC1	0	-2.660582	
14070	Incised wound	NFE2L3	0	-2.463859	
14071	Incised wound	CCL20	0	-2.310437	
14072	Incised wound	SFRP4	0	-2.294594	
14073	Incised wound	MMP12	0	-2.283803	
14074	Incised wound	COL10A1	0	-2.176511	
14075	Incised wound	MYC	0	-2.131113	
14076	Incised wound	JUB	0	-2.121437	
14077	Incised wound	GZMB	0	-2.088884	
14078	Incised wound	CDC123	0	2.016474	
14079	Incised wound	C6orf118	0	2.143404	
14080	Incised wound	CD1A	0	2.195462	
14081	Incised wound	NUDCD1	0	2.265265	
14082	Incised wound	ENPEP	0	2.363024	
14083	Independently able	MSMB	0.002956	-4.62731	
14084	Independently able	KIAA1244	0.000016	-3.114559	
14085	Independently able	IPO9	0.000579	-3.043129	
14086	Independently able	PLA1A	0.003508	-3.038372	
14087	Independently able	PRH2	0.008593	-3.022254	
14088	Independently able	UBE3A	0.000686	-2.271173	
14089	Independently able	SIM2	0.000128	-2.247735	
14090	Independently able	PLA2G7	0.006039	-2.243142	
14091	Independently able	C8orf4	0.001152	-2.225611	
14092	Independently able	CAB39L	0.003537	-2.162929	
14093	Independently able	IGFBPL1	0.000006	2.577189	
14094	Independently able	CCNE2	0.000512	2.788011	
14095	Infiltrating duct carcinoma	ENOSF1	0.04975	-2.163659	
14096	Infiltrating duct carcinoma	LOC728448	0.004106	2.432727	
14097	Infiltrating duct carcinoma	TCERG1	0.047633	2.472226	
14098	Infiltration	ENOSF1	0.04975	-2.163659	

14099	Infiltration	LOC728448	0.004106	2.432727	
14100	Infiltration	TCERG1	0.047633	2.472226	
14101	Inflammatory Bowel Diseases	CDHR1	0.000406	-4.901889	
14102	Inflammatory Bowel Diseases	AXIN2	0.000046	-4.587413	
14103	Inflammatory Bowel Diseases	ZNRF3	0.000007	-3.318925	
14104	Inflammatory Bowel Diseases	ZNF321	0.000074	-3.298804	
14105	Inflammatory Bowel Diseases	ZNF777	0.000089	-3.27148	
14106	Inflammatory Bowel Diseases	RNF43	0.000046	-3.214493	
14107	Inflammatory Bowel Diseases	B4GALT6	0.000355	-3.16038	
14108	Inflammatory Bowel Diseases	LOC100505730	0.000125	-3.101631	
14109	Inflammatory Bowel Diseases	FOXA2	0.000059	-2.956134	
14110	Inflammatory Bowel Diseases	DDX31	0.000436	-2.917829	
14111	Inflammatory Bowel Diseases	NPIPL3	0.000109	-2.876296	
14112	Inflammatory Bowel Diseases	RHEB	0.00009	-2.82394	
14113	Inflammatory Bowel Diseases	C19orf2	0.00002	-2.78316	
14114	Inflammatory Bowel Diseases	EFNA3	0.000334	-2.774743	
14115	Inflammatory Bowel Diseases	AMACR	0.000009	-2.733981	
14116	Inflammatory Bowel Diseases	CCDC113	0.000034	-2.696156	
14117	Inflammatory Bowel Diseases	ARHGAP8	0.000084	-2.637195	
14118	Inflammatory Bowel Diseases	CYP2S1	0.000037	-2.636245	
14119	Inflammatory Bowel Diseases	TOP1MT	0.000007	-2.597406	
14120	Inflammatory Bowel Diseases	CFTR	0.000155	-2.533039	
14121	Inflammatory Bowel Diseases	TMEM201	0.000349	-2.453447	
14122	Inflammatory Bowel Diseases	ZNF703	0.000227	-2.404688	
14123	Inflammatory Bowel Diseases	NMNAT3	0.000004	-2.403053	
14124	Inflammatory Bowel Diseases	ABHD11	0.000124	-2.312944	
14125	Inflammatory Bowel Diseases	PPM1H	0.000417	-2.307378	
14126	Inflammatory Bowel Diseases	L2HGDH	0.000098	-2.267435	
14127	Inflammatory Bowel Diseases	MACROD1	0.000251	-2.25448	
14128	Inflammatory Bowel Diseases	IYD	0.000401	-2.237171	
14129	Inflammatory Bowel Diseases	EXPH5	0.00009	-2.225301	
14130	Inflammatory Bowel Diseases	AADAT	0.000126	-2.18463	
14131	Inflammatory Bowel Diseases	POF1B	0.000296	-2.176879	
14132	Inflammatory Bowel Diseases	C8orf33	0.00002	-2.154067	
14133	Inflammatory Bowel Diseases	NETO2	0.000371	-2.153651	
14134	Inflammatory Bowel Diseases	PHF14	0.000107	-2.152611	
14135	Inflammatory Bowel Diseases	GGH	0.000049	-2.142053	
14136	Inflammatory Bowel Diseases	ALDH1B1	0.000296	-2.07956	
14137	Inflammatory Bowel Diseases	MEST	0.000006	-2.067237	
14138	Inflammatory Bowel Diseases	WNK4	0.000292	-2.063297	
14139	Inflammatory Bowel Diseases	CIRH1A	0.00011	-2.061629	
14140	Inflammatory Bowel Diseases	PRMT3	0.000227	-2.055489	
14141	Inflammatory Bowel Diseases	RAB40B	0.00004	-2.046246	
14142	Inflammatory Bowel Diseases	PUS7	0.000124	-2.045736	
14143	Inflammatory Bowel Diseases	ZKSCAN1	0.000046	-2.042176	
14144	Inflammatory Bowel Diseases	TBRG4	0.000124	-2.040378	
14145	Inflammatory Bowel Diseases	CXADR	0.000271	-2.036179	
14146	Inflammatory Bowel Diseases	ZNF704	0.000172	-2.025003	
14147	Inflammatory Bowel Diseases	SERF1A	0.000025	-2.011575	
14148	Inflammatory Bowel Diseases	APOL6	0.000126	2.03811	

14149	Inflammatory Bowel Diseases	HLA-DRA	0.000068	2.047747	
14150	Inflammatory Bowel Diseases	CRELD2	0.000328	2.054979	
14151	Inflammatory Bowel Diseases	XCR1	0.000226	2.073231	
14152	Inflammatory Bowel Diseases	PARP9	0.000398	2.084306	
14153	Inflammatory Bowel Diseases	NLRC5	0.00029	2.092173	
14154	Inflammatory Bowel Diseases	HLA-DRB1	0.000069	2.093284	
14155	Inflammatory Bowel Diseases	STAT1	0.000143	2.144517	
14156	Inflammatory Bowel Diseases	CYSLTR1	0.000331	2.152448	
14157	Inflammatory Bowel Diseases	IGLV2-23	0.000046	2.167195	
14158	Inflammatory Bowel Diseases	GBP2	0.00039	2.184644	
14159	Inflammatory Bowel Diseases	RASA4	0.000187	2.240732	
14160	Inflammatory Bowel Diseases	LOC728392	0.000239	2.27988	
14161	Inflammatory Bowel Diseases	UBE2L6	0.00012	2.29084	
14162	Inflammatory Bowel Diseases	IGKV4-1	0.000009	2.298194	
14163	Inflammatory Bowel Diseases	HLA-DPA1	0.00009	2.303741	
14164	Inflammatory Bowel Diseases	LST1	0.000179	2.307241	
14165	Inflammatory Bowel Diseases	CTSC	0.000008	2.318629	
14166	Inflammatory Bowel Diseases	CARD16	0.000164	2.397914	
14167	Inflammatory Bowel Diseases	CCR2	0.000251	2.474624	
14168	Inflammatory Bowel Diseases	BTN3A3	0.000042	2.50311	
14169	Inflammatory Bowel Diseases	IGLL5	0.000342	2.579993	
14170	Inflammatory Bowel Diseases	CD40	0.000376	2.586738	
14171	Inflammatory Bowel Diseases	PSMB9	0.000013	2.620499	
14172	Inflammatory Bowel Diseases	CIITA	0.000374	2.620771	
14173	Inflammatory Bowel Diseases	SFMBT2	0.00009	2.625821	
14174	Inflammatory Bowel Diseases	HLA-DMA	0.000045	2.735534	
14175	Inflammatory Bowel Diseases	KCND3	0.000126	3.374661	
14176	Inflammatory Bowel Diseases	SAMD9L	0.000353	3.700721	
14177	Inflammatory disorder	CDHR1	0.000406	-4.901889	
14178	Inflammatory disorder	AXIN2	0.000046	-4.587413	
14179	Inflammatory disorder	ZNRF3	0.000007	-3.318925	
14180	Inflammatory disorder	ZNF321	0.000074	-3.298804	
14181	Inflammatory disorder	ZNF777	0.000089	-3.27148	
14182	Inflammatory disorder	RNF43	0.000046	-3.214493	
14183	Inflammatory disorder	B4GALT6	0.000355	-3.16038	
14184	Inflammatory disorder	LOC100505730	0.000125	-3.101631	
14185	Inflammatory disorder	FOXA2	0.000059	-2.956134	
14186	Inflammatory disorder	DDX31	0.000436	-2.917829	
14187	Inflammatory disorder	NPIPL3	0.000109	-2.876296	
14188	Inflammatory disorder	RHEB	0.00009	-2.82394	
14189	Inflammatory disorder	C19orf2	0.00002	-2.78316	
14190	Inflammatory disorder	EFNA3	0.000334	-2.774743	
14191	Inflammatory disorder	AMACR	0.000009	-2.733981	
14192	Inflammatory disorder	CCDC113	0.000034	-2.696156	
14193	Inflammatory disorder	ARHGAP8	0.000084	-2.637195	
14194	Inflammatory disorder	CYP2S1	0.000037	-2.636245	
14195	Inflammatory disorder	TOP1MT	0.000007	-2.597406	
14196	Inflammatory disorder	CFTR	0.000155	-2.533039	
14197	Inflammatory disorder	TMEM201	0.000349	-2.453447	
14198	Inflammatory disorder	ZNF703	0.000227	-2.404688	

14199	Inflammatory disorder	NMNAT3	0.000004	-2.403053	
14200	Inflammatory disorder	ABHD11	0.000124	-2.312944	
14201	Inflammatory disorder	PPM1H	0.000417	-2.307378	
14202	Inflammatory disorder	L2HGDH	0.000098	-2.267435	
14203	Inflammatory disorder	MACROD1	0.000251	-2.25448	
14204	Inflammatory disorder	IYD	0.000401	-2.237171	
14205	Inflammatory disorder	EXPH5	0.00009	-2.225301	
14206	Inflammatory disorder	AADAT	0.000126	-2.18463	
14207	Inflammatory disorder	POF1B	0.000296	-2.176879	
14208	Inflammatory disorder	C8orf33	0.00002	-2.154067	
14209	Inflammatory disorder	NETO2	0.000371	-2.153651	
14210	Inflammatory disorder	PHF14	0.000107	-2.152611	
14211	Inflammatory disorder	GGH	0.000049	-2.142053	
14212	Inflammatory disorder	ALDH1B1	0.000296	-2.07956	
14213	Inflammatory disorder	MEST	0.000006	-2.067237	
14214	Inflammatory disorder	WNK4	0.000292	-2.063297	
14215	Inflammatory disorder	CIRH1A	0.00011	-2.061629	
14216	Inflammatory disorder	PRMT3	0.000227	-2.055489	
14217	Inflammatory disorder	RAB40B	0.00004	-2.046246	
14218	Inflammatory disorder	PUS7	0.000124	-2.045736	
14219	Inflammatory disorder	ZKSCAN1	0.000046	-2.042176	
14220	Inflammatory disorder	TBRG4	0.000124	-2.040378	
14221	Inflammatory disorder	CXADR	0.000271	-2.036179	
14222	Inflammatory disorder	ZNF704	0.000172	-2.025003	
14223	Inflammatory disorder	SERF1A	0.000025	-2.011575	
14224	Inflammatory disorder	APOL6	0.000126	2.03811	
14225	Inflammatory disorder	HLA-DRA	0.000068	2.047747	
14226	Inflammatory disorder	CRELD2	0.000328	2.054979	
14227	Inflammatory disorder	XCR1	0.000226	2.073231	
14228	Inflammatory disorder	PARP9	0.000398	2.084306	
14229	Inflammatory disorder	NLRC5	0.00029	2.092173	
14230	Inflammatory disorder	HLA-DRB1	0.000069	2.093284	
14231	Inflammatory disorder	STAT1	0.000143	2.144517	
14232	Inflammatory disorder	CYSLTR1	0.000331	2.152448	
14233	Inflammatory disorder	IGLV2-23	0.000046	2.167195	
14234	Inflammatory disorder	GBP2	0.00039	2.184644	
14235	Inflammatory disorder	RASA4	0.000187	2.240732	
14236	Inflammatory disorder	LOC728392	0.000239	2.27988	
14237	Inflammatory disorder	UBE2L6	0.00012	2.29084	
14238	Inflammatory disorder	IGKV4-1	0.000009	2.298194	
14239	Inflammatory disorder	HLA-DPA1	0.00009	2.303741	
14240	Inflammatory disorder	LST1	0.000179	2.307241	
14241	Inflammatory disorder	CTSC	0.000008	2.318629	
14242	Inflammatory disorder	CARD16	0.000164	2.397914	
14243	Inflammatory disorder	CCR2	0.000251	2.474624	
14244	Inflammatory disorder	BTN3A3	0.000042	2.50311	
14245	Inflammatory disorder	IGLL5	0.000342	2.579993	
14246	Inflammatory disorder	CD40	0.000376	2.586738	
14247	Inflammatory disorder	PSMB9	0.000013	2.620499	
14248	Inflammatory disorder	CIITA	0.000374	2.620771	

14249	Inflammatory disorder	SFMBT2	0.00009	2.625821	
14250	Inflammatory disorder	HLA-DMA	0.000045	2.735534	
14251	Inflammatory disorder	KCND3	0.000126	3.374661	
14252	Inflammatory disorder	SAMD9L	0.000353	3.700721	
14253	Instability	SLC26A3	0.000001	-39.432581	
14254	Instability	GUCA2A	0	-16.294008	
14255	Instability	KRT23	0.031055	-13.498961	
14256	Instability	CHRM3	0.038848	-5.36188	
14257	Instability	AMACR	0.013025	-3.921634	
14258	Instability	DST	0	-3.84509	
14259	Instability	SEMA5A	0.031272	-3.759141	
14260	Instability	MUC20	0.015636	-3.733484	
14261	Instability	TOB1	0.034358	-3.662296	
14262	Instability	AMT	0.038194	-3.50289	
14263	Instability	MGLL	0	-3.330854	
14264	Instability	SHROOM4	0.012577	-3.132474	
14265	Instability	PLAGL2	0.049045	-3.10742	
14266	Instability	SPIRE2	0.04666	-2.907811	
14267	Instability	NUDT7	0.038848	-2.789623	
14268	Instability	AXIN2	0.034678	-2.724455	
14269	Instability	DHRS12	0.044875	-2.469173	
14270	Instability	KCNE3	0.040552	-2.459474	
14271	Instability	LOC100292959	0.037228	-2.424323	
14272	Instability	MBTD1	0.007661	-2.387456	
14273	Instability	C10orf47	0.04666	-2.365174	
14274	Instability	SYNJ2	0.004314	-2.171962	
14275	Instability	LPAR1	0	-2.101231	
14276	Instability	RAP2A	0.004876	-2.069568	
14277	Instability	CAMKK2	0.004876	-2.058886	
14278	Instability	TXNDC9	0.034502	-2.032892	
14279	Instability	CBX3	0.000002	2.001406	
14280	Instability	LONP1	0	2.008715	
14281	Instability	MTA2	0.000001	2.010076	
14282	Instability	KIF15	0	2.015372	
14283	Instability	GOLT1B	0	2.015554	
14284	Instability	RIPK2	0	2.016821	
14285	Instability	MTERFD1	0.000001	2.018794	
14286	Instability	KIF11	0.000001	2.025286	
14287	Instability	NUP107	0	2.028406	
14288	Instability	CCDC59	0	2.030633	
14289	Instability	MFAP2	0	2.032549	
14290	Instability	SET	0	2.042096	
14291	Instability	TIMELESS	0	2.051896	
14292	Instability	OIP5	0.000001	2.065998	
14293	Instability	CALD1	0	2.066687	
14294	Instability	RUVBL1	0	2.069813	
14295	Instability	NUP205	0.000001	2.074642	
14296	Instability	STT3A	0	2.077468	
14297	Instability	ENO1	0.000002	2.078688	
14298	Instability	RUVBL2	0	2.080554	

14299	Instability	IGHG1	0	2.086508	
14300	Instability	SMCHD1	0.016022	2.088014	
14301	Instability	NUCB2	0.038592	2.090244	
14302	Instability	TPM2	0	2.094737	
14303	Instability	RFC5	0.000001	2.096438	
14304	Instability	EPPK1	0.000001	2.101393	
14305	Instability	SLC25A32	0.000001	2.113102	
14306	Instability	GINS2	0.000001	2.11675	
14307	Instability	NUP85	0.000001	2.11777	
14308	Instability	RBM28	0	2.120363	
14309	Instability	NCAPG2	0	2.125408	
14310	Instability	CAD	0.000001	2.127343	
14311	Instability	GTF2IRD1	0	2.133547	
14312	Instability	FANCL	0	2.139324	
14313	Instability	GZMB	0	2.14732	
14314	Instability	KCNRG	0.031716	2.151451	
14315	Instability	KIF2C	0.000001	2.154208	
14316	Instability	OAS3	0.000001	2.155018	
14317	Instability	MMP11	0	2.155168	
14318	Instability	FANCG	0	2.166676	
14319	Instability	TMEM158	0	2.168515	
14320	Instability	CCL4	0	2.174968	
14321	Instability	RAB15	0	2.188293	
14322	Instability	CTSK	0	2.189639	
14323	Instability	JAG2	0.000001	2.191192	
14324	Instability	RPL22L1	0.04666	2.194733	
14325	Instability	WDR43	0.000001	2.194866	
14326	Instability	SPOCK1	0	2.207104	
14327	Instability	WNT5A	0	2.230763	
14328	Instability	TAGLN	0	2.230853	
14329	Instability	POLR1D	0.000001	2.252838	
14330	Instability	COL15A1	0	2.260351	
14331	Instability	NUP37	0.000001	2.262069	
14332	Instability	FUT8	0	2.267452	
14333	Instability	PITX1	0.000001	2.26755	
14334	Instability	SFRP4	0	2.276769	
14335	Instability	KIF20A	0	2.295043	
14336	Instability	MICB	0	2.307009	
14337	Instability	SULF1	0	2.319154	
14338	Instability	HS2ST1	0.000002	2.328743	
14339	Instability	MYL9	0.000001	2.334374	
14340	Instability	CBFB	0	2.334836	
14341	Instability	CDC42EP1	0.000001	2.339437	
14342	Instability	SORD	0.000001	2.341712	
14343	Instability	MRPS17	0.000001	2.391333	
14344	Instability	NOP56	0.000001	2.40229	
14345	Instability	ZWILCH	0	2.403617	
14346	Instability	ARNTL2	0	2.425129	
14347	Instability	PSAT1	0.000001	2.45864	
14348	Instability	NFE2L3	0	2.463088	

14349	Instability	CSE1L	0.000001	2.467005	
14350	Instability	C12orf11	0	2.477935	
14351	Instability	COL6A1	0	2.48556	
14352	Instability	VARS	0	2.490948	
14353	Instability	ICAM1	0	2.498583	
14354	Instability	MCM7	0	2.562626	
14355	Instability	UBE2L6	0.000001	2.570384	
14356	Instability	PSMG1	0.000001	2.572526	
14357	Instability	CDC6	0	2.585624	
14358	Instability	AHCY	0.000002	2.587594	
14359	Instability	CCNA2	0.000001	2.6516	
14360	Instability	BOP1	0	2.693284	
14361	Instability	GBP1	0	2.698104	
14362	Instability	CDC25B	0.000001	2.70041	
14363	Instability	C7orf68	0.000001	2.713761	
14364	Instability	RFC3	0	2.720527	
14365	Instability	EIF5A	0	2.754041	
14366	Instability	FEN1	0.000001	2.754245	
14367	Instability	MMP3	0	2.756449	
14368	Instability	COL11A1	0	2.790647	
14369	Instability	TNC	0	2.807085	
14370	Instability	MMP1	0.000001	2.832884	
14371	Instability	TEAD4	0.000001	2.833619	
14372	Instability	LGR5	0.000001	2.855969	
14373	Instability	LMNB2	0	2.858719	
14374	Instability	NT5DC2	0	2.876001	
14375	Instability	HNRNPL	0	2.905758	
14376	Instability	SLC7A5	0.000001	2.932664	
14377	Instability	SHMT2	0	2.982462	
14378	Instability	GAS1	0	3.007882	
14379	Instability	KIF4A	0.000001	3.052197	
14380	Instability	GINS1	0	3.07162	
14381	Instability	F12	0	3.082553	
14382	Instability	STAT1	0	3.107809	
14383	Instability	TRIP13	0	3.129853	
14384	Instability	ASPHD2	0.021281	3.136229	
14385	Instability	NUTF2	0	3.241433	
14386	Instability	CEP55	0	3.248565	
14387	Instability	SOD2	0	3.254564	
14388	Instability	FOXM1	0	3.25519	
14389	Instability	MFAP5	0	3.321188	
14390	Instability	MCM4	0	3.415645	
14391	Instability	TACSTD2	0	3.446898	
14392	Instability	MIF	0.000001	3.504093	
14393	Instability	GREM1	0	3.545274	
14394	Instability	PBK	0.000001	3.714962	
14395	Instability	ECT2	0	3.796085	
14396	Instability	TMEM97	0	3.826889	
14397	Instability	PMAIP1	0	4.0525	
14398	Instability	CXCL9	0	4.116745	

14399	Instability	IFITM1	0	4.28338	
14400	Instability	CXCL10	0	4.8737	
14401	Instability	ACTG2	0	4.912122	
14402	Instability	CXCL1	0	5.226033	
14403	Instability	SRSF6	0.000001	6.156758	
14404	Instability	MMP12	0	6.617377	
14405	Instability	CXCL3	0	7.233392	
14406	Instability	SPP1	0	7.465531	
14407	Intestinal Diseases	CDHR1	0.000406	-4.901889	
14408	Intestinal Diseases	AXIN2	0.000046	-4.587413	
14409	Intestinal Diseases	ZNRF3	0.000007	-3.318925	
14410	Intestinal Diseases	ZNF321	0.000074	-3.298804	
14411	Intestinal Diseases	ZNF777	0.000089	-3.27148	
14412	Intestinal Diseases	RNF43	0.000046	-3.214493	
14413	Intestinal Diseases	B4GALT6	0.000355	-3.16038	
14414	Intestinal Diseases	LOC100505730	0.000125	-3.101631	
14415	Intestinal Diseases	FOXA2	0.000059	-2.956134	
14416	Intestinal Diseases	DDX31	0.000436	-2.917829	
14417	Intestinal Diseases	NPIPL3	0.000109	-2.876296	
14418	Intestinal Diseases	RHEB	0.00009	-2.82394	
14419	Intestinal Diseases	C19orf2	0.00002	-2.78316	
14420	Intestinal Diseases	EFNA3	0.000334	-2.774743	
14421	Intestinal Diseases	AMACR	0.000009	-2.733981	
14422	Intestinal Diseases	CCDC113	0.000034	-2.696156	
14423	Intestinal Diseases	ARHGAP8	0.000084	-2.637195	
14424	Intestinal Diseases	CYP2S1	0.000037	-2.636245	
14425	Intestinal Diseases	TOP1MT	0.000007	-2.597406	
14426	Intestinal Diseases	CFTR	0.000155	-2.533039	
14427	Intestinal Diseases	TMEM201	0.000349	-2.453447	
14428	Intestinal Diseases	ZNF703	0.000227	-2.404688	
14429	Intestinal Diseases	NMNAT3	0.000004	-2.403053	
14430	Intestinal Diseases	ABHD11	0.000124	-2.312944	
14431	Intestinal Diseases	PPM1H	0.000417	-2.307378	
14432	Intestinal Diseases	L2HGDH	0.000098	-2.267435	
14433	Intestinal Diseases	MACROD1	0.000251	-2.25448	
14434	Intestinal Diseases	IYD	0.000401	-2.237171	
14435	Intestinal Diseases	EXPH5	0.00009	-2.225301	
14436	Intestinal Diseases	AADAT	0.000126	-2.18463	
14437	Intestinal Diseases	POF1B	0.000296	-2.176879	
14438	Intestinal Diseases	C8orf33	0.00002	-2.154067	
14439	Intestinal Diseases	NETO2	0.000371	-2.153651	
14440	Intestinal Diseases	PHF14	0.000107	-2.152611	
14441	Intestinal Diseases	GGH	0.000049	-2.142053	
14442	Intestinal Diseases	ALDH1B1	0.000296	-2.07956	
14443	Intestinal Diseases	MEST	0.000006	-2.067237	
14444	Intestinal Diseases	WNK4	0.000292	-2.063297	
14445	Intestinal Diseases	CIRH1A	0.00011	-2.061629	
14446	Intestinal Diseases	PRMT3	0.000227	-2.055489	
14447	Intestinal Diseases	RAB40B	0.00004	-2.046246	
14448	Intestinal Diseases	PUS7	0.000124	-2.045736	

14449	Intestinal Diseases	ZKSCAN1	0.000046	-2.042176	
14450	Intestinal Diseases	TBRG4	0.000124	-2.040378	
14451	Intestinal Diseases	CXADR	0.000271	-2.036179	
14452	Intestinal Diseases	ZNF704	0.000172	-2.025003	
14453	Intestinal Diseases	SERF1A	0.000025	-2.011575	
14454	Intestinal Diseases	APOL6	0.000126	2.03811	
14455	Intestinal Diseases	HLA-DRA	0.000068	2.047747	
14456	Intestinal Diseases	CRELD2	0.000328	2.054979	
14457	Intestinal Diseases	XCR1	0.000226	2.073231	
14458	Intestinal Diseases	PARP9	0.000398	2.084306	
14459	Intestinal Diseases	NLRC5	0.00029	2.092173	
14460	Intestinal Diseases	HLA-DRB1	0.000069	2.093284	
14461	Intestinal Diseases	STAT1	0.000143	2.144517	
14462	Intestinal Diseases	CYSLTR1	0.000331	2.152448	
14463	Intestinal Diseases	IGLV2-23	0.000046	2.167195	
14464	Intestinal Diseases	GBP2	0.00039	2.184644	
14465	Intestinal Diseases	RASA4	0.000187	2.240732	
14466	Intestinal Diseases	LOC728392	0.000239	2.27988	
14467	Intestinal Diseases	UBE2L6	0.00012	2.29084	
14468	Intestinal Diseases	IGKV4-1	0.000009	2.298194	
14469	Intestinal Diseases	HLA-DPA1	0.00009	2.303741	
14470	Intestinal Diseases	LST1	0.000179	2.307241	
14471	Intestinal Diseases	CTSC	0.000008	2.318629	
14472	Intestinal Diseases	CARD16	0.000164	2.397914	
14473	Intestinal Diseases	CCR2	0.000251	2.474624	
14474	Intestinal Diseases	BTN3A3	0.000042	2.50311	
14475	Intestinal Diseases	IGLL5	0.000342	2.579993	
14476	Intestinal Diseases	CD40	0.000376	2.586738	
14477	Intestinal Diseases	PSMB9	0.000013	2.620499	
14478	Intestinal Diseases	CIITA	0.000374	2.620771	
14479	Intestinal Diseases	SFMBT2	0.00009	2.625821	
14480	Intestinal Diseases	HLA-DMA	0.000045	2.735534	
14481	Intestinal Diseases	KCND3	0.000126	3.374661	
14482	Intestinal Diseases	SAMD9L	0.000353	3.700721	
14483	Irritable Bowel Syndrome	REG1A	0.001231	-83.086651	
14484	Irritable Bowel Syndrome	ADAMTS4	0.003187	-12.858781	
14485	Irritable Bowel Syndrome	NR4A2	0.00141	-12.319624	
14486	Irritable Bowel Syndrome	C4BPB	0.002093	-11.220864	
14487	Irritable Bowel Syndrome	EGR2	0.00597	-11.198472	
14488	Irritable Bowel Syndrome	SPINK4	0.000062	-10.887655	
14489	Irritable Bowel Syndrome	CXCL1	0.002032	-7.262449	
14490	Irritable Bowel Syndrome	CYR61	0.002526	-5.503438	
14491	Irritable Bowel Syndrome	CXCL2	0.001029	-5.36852	
14492	Irritable Bowel Syndrome	FOS	0.001024	-5.232422	
14493	Irritable Bowel Syndrome	NOS2	0.002362	-5.151254	
14494	Irritable Bowel Syndrome	SOCS3	0.000879	-4.842444	
14495	Irritable Bowel Syndrome	REG4	0.00372	-4.605485	
14496	Irritable Bowel Syndrome	EGR1	0.003143	-4.268072	
14497	Irritable Bowel Syndrome	RGS1	0.001997	-3.297169	
14498	Irritable Bowel Syndrome	ZC3H12A	0.000421	-3.093546	

14499	Irritable Bowel Syndrome	CFB	0.004256	-3.045857	
14500	Irritable Bowel Syndrome	AREG	0.000857	-2.825324	
14501	Irritable Bowel Syndrome	IGFBP2	0.000553	-2.752176	
14502	Irritable Bowel Syndrome	GBP4	0.006339	-2.527755	
14503	Irritable Bowel Syndrome	IER3	0.001264	-2.52473	
14504	Irritable Bowel Syndrome	CCL4	0.004244	-2.509801	
14505	Irritable Bowel Syndrome	JUNB	0.000106	-2.496438	
14506	Irritable Bowel Syndrome	NCOA7	0.001264	-2.490791	
14507	Irritable Bowel Syndrome	DUSP1	0.00129	-2.481697	
14508	Irritable Bowel Syndrome	ERO1L	0.006313	-2.425163	
14509	Irritable Bowel Syndrome	PFKFB3	0.006233	-2.377068	
14510	Irritable Bowel Syndrome	KLF2	0.000505	-2.345314	
14511	Irritable Bowel Syndrome	RTEL1	0.001264	-2.302734	
14512	Irritable Bowel Syndrome	TYMP	0.003595	-2.300272	
14513	Irritable Bowel Syndrome	C2	0.003497	-2.277307	
14514	Irritable Bowel Syndrome	GBP1	0.003807	-2.272556	
14515	Irritable Bowel Syndrome	PHLDA1	0.007508	-2.213751	
14516	Irritable Bowel Syndrome	ERRFI1	0.004336	-2.192916	
14517	Irritable Bowel Syndrome	NAMPT	0.002526	-2.192039	
14518	Irritable Bowel Syndrome	ISG20	0.003187	-2.140839	
14519	Irritable Bowel Syndrome	NFKBIZ	0.000486	-2.117779	
14520	Irritable Bowel Syndrome	TAP1	0.000491	-2.110082	
14521	Irritable Bowel Syndrome	MAFF	0.000264	-2.102772	
14522	Irritable Bowel Syndrome	PSMB9	0.004336	-2.078523	
14523	Irritable Bowel Syndrome	HK2	0.002417	-2.074627	
14524	Irritable Bowel Syndrome	LINC00152	0.004936	-2.01997	
14525	Irritable Bowel Syndrome	CASP5	0.002526	-2.008472	
14526	Irritable Bowel Syndrome	APOL2	0.005286	-2.002322	
14527	Irritable Bowel Syndrome	AOC3	0.000264	2.002483	
14528	Irritable Bowel Syndrome	TMEM98	0.002032	2.031242	
14529	Irritable Bowel Syndrome	PTN	0.001921	2.034433	
14530	Irritable Bowel Syndrome	IL1R2	0.002032	2.192872	
14531	Irritable Bowel Syndrome	ISX	0.004015	2.223209	
14532	Irritable Bowel Syndrome	LOC100506542	0.000421	2.237208	
14533	Irritable Bowel Syndrome	CXCL12	0.001997	2.249309	
14534	Irritable Bowel Syndrome	LRRN2	0.006445	2.250284	
14535	Irritable Bowel Syndrome	SPON1	0.001264	2.372523	
14536	Irritable Bowel Syndrome	B3GNT7	0.000625	2.449062	
14537	Irritable Bowel Syndrome	MFSD4	0.007025	2.520304	
14538	Irritable Bowel Syndrome	CAPN13	0.002207	2.550225	
14539	Irritable Bowel Syndrome	LGALS2	0.001997	2.643832	
14540	Irritable Bowel Syndrome	CRYBA2	0.001997	2.679138	
14541	Irritable Bowel Syndrome	SOSTDC1	0.001276	2.904439	
14542	Irritable Bowel Syndrome	EFEMP1	0.000625	2.920202	
14543	Irritable Bowel Syndrome	TPH1	0.000625	3.013002	
14544	Irritable Bowel Syndrome	FN1	0.000816	3.022	
14545	Irritable Bowel Syndrome	SLC37A2	0.000324	3.111415	
14546	Irritable Bowel Syndrome	PYY	0.002014	3.138991	
14547	Irritable Bowel Syndrome	GCG	0.006348	3.18315	
14548	Irritable Bowel Syndrome	FRAS1	0.002818	3.39953	

14549	Irritable Bowel Syndrome	NTRK2	0.005068	3.656479	
14550	Irritable Bowel Syndrome	INSL5	0.003187	4.574727	
14551	Irritable Bowel Syndrome	LOC100506659	0.003692	4.8718	
14552	Irritable Bowel Syndrome	SST	0.007025	5.490615	
14553	Irritable Bowel Syndrome	HOXD13	0.003847	5.901125	
14554	Irritable Bowel Syndrome	TTR	0.005754	6.120098	
14555	Irritable Bowel Syndrome	WASF3	0.005456	7.015248	
14556	Irritable Bowel Syndrome	TNNC1	0.002186	7.839611	
14557	Irritable Bowel Syndrome	CLDN8	0.006339	8.740012	
14558	Irritable Bowel Syndrome	FMN2	0.003283	8.772553	
14559	Irritable Bowel Syndrome	LOC389023	0.001997	8.886277	
14560	Irritable Bowel Syndrome	DSCAML1	0.00141	9.19284	
14561	Irritable Bowel Syndrome	HOXD11	0.001231	10.815761	
14562	Irritable Bowel Syndrome	RASSF10	0.000857	11.359478	
14563	Irritable Bowel Syndrome	HOXD10	0.001868	16.483265	
14564	isolation aspects	CFH	0.001969	-11.140652	
14565	isolation aspects	RBM15	0.004791	-3.808127	
14566	isolation aspects	UQCC	0.005408	-3.337098	
14567	isolation aspects	PDCD6	0.000732	-3.30841	
14568	isolation aspects	HELLS	0.004596	-3.245423	
14569	isolation aspects	ZNF160	0.004021	-3.209786	
14570	isolation aspects	POLR2J2	0.004215	-3.038889	
14571	isolation aspects	ZNF207	0.004792	-2.972298	
14572	isolation aspects	WHSC1	0.001268	-2.880382	
14573	isolation aspects	LOC100129637	0.002981	-2.861468	
14574	isolation aspects	PLXND1	0.003286	-2.855506	
14575	isolation aspects	AFG3L1P	0.002109	-2.846511	
14576	isolation aspects	LOC595101	0.000327	-2.818768	
14577	isolation aspects	BMS1P1	0.000316	-2.804696	
14578	isolation aspects	PRKDC	0.00241	-2.803191	
14579	isolation aspects	ANGPT1	0.00071	-2.796247	
14580	isolation aspects	FAM173B	0.003152	-2.753963	
14581	isolation aspects	CDCA7	0.004748	-2.753797	
14582	isolation aspects	RGNEF	0.005098	-2.707656	
14583	isolation aspects	SRSF4	0.002427	-2.698683	
14584	isolation aspects	MMRN2	0.001969	-2.660392	
14585	isolation aspects	KIAA1377	0.001162	-2.631271	
14586	isolation aspects	ATP2C2	0.005467	-2.576461	
14587	isolation aspects	CDKN2C	0.001025	-2.504487	
14588	isolation aspects	LOC399753	0.006485	-2.480526	
14589	isolation aspects	CRIPAK	0.005174	-2.417205	
14590	isolation aspects	PTK2	0.001884	-2.380796	
14591	isolation aspects	IFT80	0.003512	-2.361122	
14592	isolation aspects	C2orf67	0.003336	-2.341392	
14593	isolation aspects	ZADH2	0.006011	-2.325923	
14594	isolation aspects	CREBZF	0.001794	-2.300725	
14595	isolation aspects	MYO19	0.006064	-2.275494	
14596	isolation aspects	CHD3	0.004639	-2.229819	
14597	isolation aspects	NOC2L	0.003181	-2.226973	
14598	isolation aspects	BRCA1	0.005839	-2.224053	

14599	isolation aspects	NF1	0.004108	-2.212946	
14600	isolation aspects	CHD9	0.004107	-2.20314	
14601	isolation aspects	XPO1	0.000517	-2.193761	
14602	isolation aspects	TTC23	0.005733	-2.18306	
14603	isolation aspects	EML4	0.002343	-2.177747	
14604	isolation aspects	SGK269	0.005301	-2.172195	
14605	isolation aspects	PHKA2	0.002728	-2.171707	
14606	isolation aspects	RBBP4	0.005467	-2.163389	
14607	isolation aspects	LOC100507153	0.002967	-2.133094	
14608	isolation aspects	ZBTB44	0.000125	-2.120909	
14609	isolation aspects	WDR90	0.003152	-2.115398	
14610	isolation aspects	PATZ1	0.002785	-2.11183	
14611	isolation aspects	ZNF248	0.004792	-2.098686	
14612	isolation aspects	SFRS4	0.003692	-2.091499	
14613	isolation aspects	SGK494	0.001756	-2.052257	
14614	isolation aspects	USP13	0.001498	-2.050189	
14615	isolation aspects	ALDH7A1	0.004792	-2.049889	
14616	isolation aspects	MPHOSPH9	0.004116	-2.020827	
14617	isolation aspects	KIAA0467	0.002485	-2.017488	
14618	isolation aspects	OSBPL3	0.000732	2.00378	
14619	isolation aspects	SERTAD1	0.003463	2.024163	
14620	isolation aspects	JMJD6	0.001662	2.039276	
14621	isolation aspects	RAP2B	0.000671	2.040388	
14622	isolation aspects	NDFIP2	0.002765	2.051303	
14623	isolation aspects	TRIM15	0.006381	2.052602	
14624	isolation aspects	IFNAR2	0.002588	2.053709	
14625	isolation aspects	HN1	0.006181	2.061183	
14626	isolation aspects	SLC25A25	0.00301	2.074173	
14627	isolation aspects	KLF10	0.001147	2.087965	
14628	isolation aspects	SBDS	0.001552	2.112631	
14629	isolation aspects	ATP6V0E1	0.005141	2.127361	
14630	isolation aspects	RNF7	0.004791	2.144269	
14631	isolation aspects	LHFPL2	0.006011	2.147585	
14632	isolation aspects	HIST1H2BF	0.000517	2.162432	
14633	isolation aspects	RHPN2	0.003692	2.162838	
14634	isolation aspects	MAP1LC3B	0.004108	2.164625	
14635	isolation aspects	ALAS1	0.004861	2.183211	
14636	isolation aspects	HIST1H2BE	0.003152	2.190406	
14637	isolation aspects	SELK	0.000728	2.191273	
14638	isolation aspects	CDV3	0.005041	2.199458	
14639	isolation aspects	PERP	0.00022	2.202057	
14640	isolation aspects	HLA-F	0.004108	2.207022	
14641	isolation aspects	AMOTL2	0.001552	2.213932	
14642	isolation aspects	PPP2CB	0.006104	2.239827	
14643	isolation aspects	ARL8B	0.005085	2.245124	
14644	isolation aspects	CFLAR	0.003477	2.250863	
14645	isolation aspects	DNAJB1	0.00241	2.280388	
14646	isolation aspects	PDE12	0.001268	2.282691	
14647	isolation aspects	S100A11	0.0036	2.290681	
14648	isolation aspects	IER5	0.004748	2.297363	

14649	isolation aspects	ARL5B	0.000244	2.30292	
14650	isolation aspects	FOXN2	0.001756	2.30656	
14651	isolation aspects	ERRFI1	0.004065	2.30757	
14652	isolation aspects	NFKBIE	0.005973	2.353556	
14653	isolation aspects	MTHFSD	0.005298	2.359503	
14654	isolation aspects	STK17A	0.000125	2.396145	
14655	isolation aspects	HIGD1A	0.001756	2.400591	
14656	isolation aspects	PLCXD2	0.004147	2.435619	
14657	isolation aspects	HIST1H2BI	0.002829	2.438257	
14658	isolation aspects	GLS	0.001794	2.44452	
14659	isolation aspects	TPMT	0.004143	2.452997	
14660	isolation aspects	PIM1	0.001147	2.458919	
14661	isolation aspects	LGALS8	0.001679	2.471341	
14662	isolation aspects	CCDC51	0.006104	2.480221	
14663	isolation aspects	HIST1H2BK	0.001662	2.514361	
14664	isolation aspects	SERTAD2	0.0044	2.534205	
14665	isolation aspects	AREG	0.000389	2.539078	
14666	isolation aspects	OCLN	0.001052	2.551247	
14667	isolation aspects	CPEB2	0.005988	2.565899	
14668	isolation aspects	MDFI	0.004314	2.570407	
14669	isolation aspects	HCCS	0.004748	2.584927	
14670	isolation aspects	C6orf132	0.001087	2.612396	
14671	isolation aspects	RAB22A	0.001756	2.613734	
14672	isolation aspects	ANXA3	0.00508	2.635969	
14673	isolation aspects	MXD1	0.000125	2.649865	
14674	isolation aspects	RALA	0.002459	2.658884	
14675	isolation aspects	TPM4	0.003336	2.660044	
14676	isolation aspects	TNFSF9	0.000316	2.669576	
14677	isolation aspects	RND3	0.000125	2.709598	
14678	isolation aspects	TIPARP	0.004639	2.712231	
14679	isolation aspects	DUSP10	0.000754	2.723635	
14680	isolation aspects	SFN	0.004323	2.72591	
14681	isolation aspects	EPB41L5	0.00032	2.728434	
14682	isolation aspects	KLF4	0.001474	2.76071	
14683	isolation aspects	OBFC2A	0.003595	2.787294	
14684	isolation aspects	TAP1	0.003545	2.824943	
14685	isolation aspects	FGD6	0.004373	2.855279	
14686	isolation aspects	NAMPT	0.005085	2.873318	
14687	isolation aspects	ZNF165	0.002504	2.875868	
14688	isolation aspects	CLDN4	0.00071	2.911135	
14689	isolation aspects	SEMA7A	0.001969	2.932253	
14690	isolation aspects	GJB3	0.004107	2.938013	
14691	isolation aspects	NFKBIB	0.003234	2.941764	
14692	isolation aspects	TAGAP	0.001969	2.980919	
14693	isolation aspects	CD47	0.005041	2.995743	
14694	isolation aspects	EZR	0.00178	3.023584	
14695	isolation aspects	CLIC5	0.004147	3.081047	
14696	isolation aspects	DPH3	0.001748	3.091732	
14697	isolation aspects	LAMB3	0.005141	3.103992	
14698	isolation aspects	LMO7	0.005034	3.110319	

14699	isolation aspects	LOC100506979	0.004639	3.130702	
14700	isolation aspects	HIST1H2BH	0.000517	3.147934	
14701	isolation aspects	TICAM1	0.000191	3.159202	
14702	isolation aspects	GAN	0.002595	3.170409	
14703	isolation aspects	IL23A	0.001147	3.182919	
14704	isolation aspects	LOC440896	0.001764	3.190618	
14705	isolation aspects	CLDN23	0.005467	3.243061	
14706	isolation aspects	H2BFS	0.00071	3.246703	
14707	isolation aspects	CHMP2B	0.000732	3.259293	
14708	isolation aspects	MAFF	0.00071	3.270931	
14709	isolation aspects	HIST1H2BD	0.001211	3.297822	
14710	isolation aspects	GADD45A	0.005141	3.349209	
14711	isolation aspects	IFRD1	0.000229	3.364611	
14712	isolation aspects	DDA1	0.006252	3.442754	
14713	isolation aspects	USP53	0.003981	3.463071	
14714	isolation aspects	RHOF	0.00178	3.465639	
14715	isolation aspects	RAB9A	0.003181	3.471487	
14716	isolation aspects	PPP1R15A	0.006064	3.541813	
14717	isolation aspects	LOC439990	0.004107	3.5739	
14718	isolation aspects	ID2	0.003434	3.739528	
14719	isolation aspects	HIST1H2BG	0.004577	3.788688	
14720	isolation aspects	PPP4R1L	0.003181	3.813426	
14721	isolation aspects	GPRC5A	0.001717	3.866214	
14722	isolation aspects	EDN1	0.001662	3.996734	
14723	isolation aspects	KLF6	0.000125	4.047716	
14724	isolation aspects	ZFAND2A	0.001662	4.179808	
14725	isolation aspects	TNFAIP3	0.001662	4.181002	
14726	isolation aspects	FOSL1	0.002785	4.261609	
14727	isolation aspects	HIST2H2AA3	0.002775	4.307458	
14728	isolation aspects	CXCL16	0.001552	4.316383	
14729	isolation aspects	NCEH1	0.004107	4.415097	
14730	isolation aspects	PMAIP1	0.002949	4.535741	
14731	isolation aspects	TUBB2A	0.001648	4.71727	
14732	isolation aspects	TM4SF1	0.004059	4.932563	
14733	isolation aspects	NFKBIA	0.004108	5.051207	
14734	isolation aspects	KIAA1609	0.003254	5.184877	
14735	isolation aspects	DUSP5	0.001662	5.413098	
14736	isolation aspects	EMP1	0.001764	5.681152	
14737	isolation aspects	PLAUR	0.000229	5.92149	
14738	isolation aspects	IL32	0.002728	6.867646	
14739	isolation aspects	C10orf116	0.003181	7.514991	
14740	isolation aspects	BIRC3	0.00642	7.908702	
14741	isolation aspects	LAMC2	0.003986	9.26201	
14742	isolation aspects	CDA	0.005862	9.815259	
14743	Laboratory culture	MAP7	0.000652	-7.749575	
14744	Laboratory culture	MAPK13	0.000093	-5.139744	
14745	Laboratory culture	ROD1	0.000155	-4.802888	
14746	Laboratory culture	RAB3IP	0.000218	-4.603243	
14747	Laboratory culture	WWP1	0.000529	-3.9455	
14748	Laboratory culture	RNASET2	0.000088	-3.751019	

14749	Laboratory culture	PPFIBP2	0.000541	-3.362162	
14750	Laboratory culture	BAZ2B	0.000375	-3.040968	
14751	Laboratory culture	CYP2R1	0.000348	-2.97435	
14752	Laboratory culture	PHIP	0.00018	-2.916824	
14753	Laboratory culture	OCIAD2	0.000516	-2.862596	
14754	Laboratory culture	SYK	0.000144	-2.815386	
14755	Laboratory culture	KIF21A	0.000493	-2.764031	
14756	Laboratory culture	LOC387921	0.000603	-2.703054	
14757	Laboratory culture	SLC25A5	0.000533	-2.551182	
14758	Laboratory culture	MRPS35	0.000166	-2.52774	
14759	Laboratory culture	PPIG	0.000425	-2.505525	
14760	Laboratory culture	GUSB	0.00007	-2.451689	
14761	Laboratory culture	ANXA4	0.000425	-2.440149	
14762	Laboratory culture	ZNF165	0.000062	-2.402117	
14763	Laboratory culture	TMEM41B	0.000615	-2.399405	
14764	Laboratory culture	ZNF721	0.000664	-2.377136	
14765	Laboratory culture	ZNF800	0.000597	-2.347068	
14766	Laboratory culture	C4ORF16	0.000093	-2.332935	
14767	Laboratory culture	FLJ34969	0.000597	-2.282324	
14768	Laboratory culture	CCDC76	0.000599	-2.225771	
14769	Laboratory culture	TARDBP	0.000574	-2.221869	
14770	Laboratory culture	RNF141	0.000516	-2.206677	
14771	Laboratory culture	C11ORF71	0.000545	-2.102567	
14772	Laboratory culture	RPS24	0.000286	-2.072647	
14773	Laboratory culture	DGKD	0.000219	-2.026221	
14774	Laboratory culture	RPL5	0.000282	-2.004885	
14775	Laboratory culture	RGS10	0.000375	2.006736	
14776	Laboratory culture	CCDC127	0.000514	2.023144	
14777	Laboratory culture	RBMS2	0.000284	2.071696	
14778	Laboratory culture	TNFAIP1	0.000398	2.089194	
14779	Laboratory culture	GAPDH	0.000425	2.158625	
14780	Laboratory culture	ADAMTSL5	0.000486	2.27493	
14781	Laboratory culture	NPR2	0.000399	2.282587	
14782	Laboratory culture	SDF4	0.000665	2.289924	
14783	Laboratory culture	MST150	0.000125	2.311495	
14784	Laboratory culture	SEC22C	0.000369	2.345013	
14785	Laboratory culture	NTAN1	0.00061	2.400488	
14786	Laboratory culture	RIC8A	0.000436	2.464435	
14787	Laboratory culture	ZBTB4	0.000201	2.481596	
14788	Laboratory culture	SLC35E1	0.000436	2.585884	
14789	Laboratory culture	PNMA1	0.000603	2.646874	
14790	Laboratory culture	ABI2	0.000191	2.657783	
14791	Laboratory culture	PVRL2	0.000187	2.739545	
14792	Laboratory culture	M-RIP	0.000292	2.875153	
14793	Laboratory culture	MPV17	0.000334	2.87788	
14794	Laboratory culture	SPARC	0.000665	2.977244	
14795	Laboratory culture	EXT1	0.000425	3.142807	
14796	Laboratory culture	DYRK3	0.000062	3.199536	
14797	Laboratory culture	TCEAL3	0.000602	3.283354	
14798	Laboratory culture	VKORC1	0.000031	3.288607	

14799	Laboratory culture	TPM1	0.000436	3.396558	
14800	Laboratory culture	KIAA1754	0.000235	3.642638	
14801	Laboratory culture	HSPB2	0.000603	3.783672	
14802	Laboratory culture	FKSG30	0.000436	3.868274	
14803	Laboratory culture	MYADM	0.000189	3.982227	
14804	Laboratory culture	DFNA5	0.000086	4.008277	
14805	Laboratory culture	PELO	0.000599	4.297751	
14806	Laboratory culture	RAB6IP1	0.000219	4.356722	
14807	Laboratory culture	NINJ1	0.00058	4.385068	
14808	Laboratory culture	GABARAPL1	0.000031	4.474687	
14809	Laboratory culture	ACTN1	0.000001	4.790828	
14810	Laboratory culture	MSN	0.000155	4.827876	
14811	Laboratory culture	VIM	0.000441	4.942041	
14812	Laboratory culture	ACTA2	0.000155	5.024653	
14813	Laboratory culture	CYR61	0.000093	5.299898	
14814	Laboratory culture	DEGS1	0.000035	5.682363	
14815	Laboratory culture	LGALS1	0.000071	5.933415	
14816	Laboratory culture	NGFRAP1	0.000501	6.226585	
14817	Laboratory culture	COL1A2	0.000375	10.191927	
14818	Laboratory culture	FAM20C	0.000468	10.313947	
14819	Laboratory culture	DKK3	0.000597	10.416492	
14820	Laboratory culture	VASN	0.000441	12.264142	
14821	Laser	APOB	0.000013	-22.114208	
14822	Laser	APOA2	0.000028	-18.076081	
14823	Laser	RBP4	0.000019	-16.715013	
14824	Laser	APOA1	0.000037	-16.502043	
14825	Laser	APOH	0.000028	-14.087162	
14826	Laser	KISS1R	0	-12.825872	
14827	Laser	FOLR1	0	-12.631292	
14828	Laser	DHRS2	0	-11.89824	
14829	Laser	HAVCR1	0.000009	-10.208146	
14830	Laser	LRP2	0.000006	-8.705283	
14831	Laser	ANXA9	0	-7.556247	
14832	Laser	DMKN	0.000008	-7.528223	
14833	Laser	TNNT1	0	-7.409935	
14834	Laser	REEP6	0.000016	-7.257684	
14835	Laser	SPAG4	0.000017	-7.002475	
14836	Laser	APOM	0.000007	-6.978881	
14837	Laser	SOAT2	0.000004	-6.287131	
14838	Laser	FBXO2	0.000006	-5.985957	
14839	Laser	TET1	0.000032	-5.869496	
14840	Laser	LOC728449	0.000015	-5.817347	
14841	Laser	LOC285733	0.000011	-5.770381	
14842	Laser	C12orf59	0.000006	-5.437558	
14843	Laser	DIO1	0.000011	-5.403243	
14844	Laser	DNASE1	0.000003	-5.360365	
14845	Laser	TMEM92	0.000001	-5.294438	
14846	Laser	UCA1	0.000024	-4.988	
14847	Laser	CDK19	0.00001	-4.894224	
14848	Laser	SLC46A1	0.000008	-4.35098	

14849	Laser	GATA3	0.00001	-4.335344	
14850	Laser	TM7SF2	0.00002	-4.293242	
14851	Laser	ZNF488	0.000002	-4.204328	
14852	Laser	TNNC1	0.000008	-4.031651	
14853	Laser	TFF2	0.000001	-3.972717	
14854	Laser	NCRNA00286A	0.000001	-3.939098	
14855	Laser	PRODH	0.000036	-3.888523	
14856	Laser	GET4	0.000035	-3.837749	
14857	Laser	PECR	0	-3.800593	
14858	Laser	F7	0	-3.759021	
14859	Laser	DLX4	0.000011	-3.716275	
14860	Laser	HSD17B12	0.000017	-3.708705	
14861	Laser	SLC6A8	0.000004	-3.701869	
14862	Laser	FAM169A	0.000003	-3.612246	
14863	Laser	SYTL1	0.00001	-3.596879	
14864	Laser	PALM3	0	-3.591714	
14865	Laser	SLC6A10P	0.000003	-3.552675	
14866	Laser	LRP5	0.000007	-3.479929	
14867	Laser	IGF2BP1	0.000031	-3.445045	
14868	Laser	AIG1	0	-3.403283	
14869	Laser	CHKA	0.000002	-3.397474	
14870	Laser	MSX2	0.00001	-3.388462	
14871	Laser	SLCO2B1	0.000014	-3.309305	
14872	Laser	RAB11FIP4	0.000028	-3.271163	
14873	Laser	TMEM27	0.000001	-3.230254	
14874	Laser	ASGR1	0.000028	-3.219771	
14875	Laser	TGFBR3	0	-3.157052	
14876	Laser	LOC285708	0.000001	-3.152352	
14877	Laser	FAM117A	0.000011	-3.132597	
14878	Laser	LOC285943	0.000007	-3.117586	
14879	Laser	DBP	0.000001	-3.101255	
14880	Laser	NAGS	0.000003	-3.099091	
14881	Laser	LIMS3	0.000009	-3.070937	
14882	Laser	SLC30A1	0.000014	-3.007005	
14883	Laser	DNAH14	0.00003	-2.95949	
14884	Laser	SERPINA5	0.000002	-2.956248	
14885	Laser	CHCHD5	0	-2.942043	
14886	Laser	TEX19	0.000009	-2.939232	
14887	Laser	P2RX4	0.000032	-2.932601	
14888	Laser	PEX13	0.000013	-2.92411	
14889	Laser	GGT1	0	-2.889186	
14890	Laser	PAK6	0.000003	-2.843385	
14891	Laser	LOC100505876	0	-2.820806	
14892	Laser	SOX30	0.000036	-2.813489	
14893	Laser	SUV420H1	0.000003	-2.811511	
14894	Laser	SH3TC1	0.000003	-2.807969	
14895	Laser	LPCAT2	0.00001	-2.801799	
14896	Laser	LOC100505712	0.000026	-2.760311	
14897	Laser	RHOF	0.000009	-2.758727	
14898	Laser	C21orf129	0.000007	-2.755186	

14899	Laser	TRMT2B	0	-2.723715	
14900	Laser	LOC439990	0.000002	-2.714572	
14901	Laser	FAM69B	0.000006	-2.706077	
14902	Laser	DHCR7	0.000019	-2.646961	
14903	Laser	C20orf11	0.000004	-2.637912	
14904	Laser	LHX2	0.000001	-2.632749	
14905	Laser	TMEM160	0.000024	-2.615883	
14906	Laser	C10orf140	0.000005	-2.57673	
14907	Laser	PPFIA1	0.000007	-2.566938	
14908	Laser	STX3	0.000021	-2.551077	
14909	Laser	CEP57	0.000032	-2.54686	
14910	Laser	C22orf45	0.000023	-2.535703	
14911	Laser	ZNF638	0.000039	-2.532399	
14912	Laser	SERPINA4	0.00003	-2.532089	
14913	Laser	SLC37A4	0.000023	-2.525965	
14914	Laser	TMEM38B	0.000022	-2.524008	
14915	Laser	CREG2	0.000021	-2.493	
14916	Laser	EGFR	0	-2.492946	
14917	Laser	CA11	0.000014	-2.46209	
14918	Laser	TFDP2	0.000001	-2.461942	
14919	Laser	LIAS	0.000003	-2.461932	
14920	Laser	TMEM86B	0.00003	-2.428842	
14921	Laser	CSTF1	0	-2.400523	
14922	Laser	CNNM3	0	-2.38134	
14923	Laser	RHBG	0.000005	-2.367879	
14924	Laser	SCAND1	0.000014	-2.364232	
14925	Laser	TFAM	0.000022	-2.359157	
14926	Laser	MEF2A	0.000007	-2.353104	
14927	Laser	MBIP	0.000033	-2.351988	
14928	Laser	TMEM18	0.000005	-2.334022	
14929	Laser	FAM184B	0.000006	-2.318637	
14930	Laser	CDKN2AIPNL	0	-2.31011	
14931	Laser	ANKHD1	0.000012	-2.308774	
14932	Laser	DHDPSL	0.000001	-2.302446	
14933	Laser	MRPL21	0.000015	-2.266093	
14934	Laser	ZNF236	0.000002	-2.255423	
14935	Laser	LOC284804	0.000015	-2.246998	
14936	Laser	PHF15	0.000022	-2.243242	
14937	Laser	RAB6B	0	-2.217003	
14938	Laser	RYR1	0.00003	-2.213525	
14939	Laser	RDM1	0.000003	-2.209673	
14940	Laser	HOXC13	0.000001	-2.203482	
14941	Laser	ACVR2B	0.000013	-2.200262	
14942	Laser	LOC100128988	0	-2.190348	
14943	Laser	GLS2	0.000003	-2.182317	
14944	Laser	NEURL2	0.000001	-2.170779	
14945	Laser	GNAL	0.00001	-2.164026	
14946	Laser	ARHGEF26	0.000015	-2.154755	
14947	Laser	LOC285074	0.000028	-2.150647	
14948	Laser	KLHL24	0.000005	-2.138031	

14949	Laser	SLC2A4RG	0.00001	-2.127678	
14950	Laser	STRN3	0.000034	-2.114436	
14951	Laser	SOHLH1	0.000024	-2.108161	
14952	Laser	RXRA	0.000006	-2.098693	
14953	Laser	LOC401233	0.000011	-2.08761	
14954	Laser	SUSD4	0.000034	-2.086744	
14955	Laser	TMPRSS6	0.000001	-2.066939	
14956	Laser	ANKRD16	0.000001	-2.064732	
14957	Laser	DENND5B	0.00001	-2.0426	
14958	Laser	OSBPL10	0	-2.034479	
14959	Laser	SRSF5	0.00003	-2.033889	
14960	Laser	FOXRED2	0.000014	-2.027633	
14961	Laser	ZNF614	0	-2.024856	
14962	Laser	LOC148709	0	-2.004196	
14963	Laser	MYO10	0	2.018429	
14964	Laser	CSNK1G1	0.000011	2.025083	
14965	Laser	S100PBP	0.00001	2.034711	
14966	Laser	SMAD2	0.000009	2.037005	
14967	Laser	ZNF438	0	2.049256	
14968	Laser	ADAMTS10	0.000003	2.065817	
14969	Laser	C14orf28	0.000004	2.06638	
14970	Laser	AMMECR1	0.000003	2.083943	
14971	Laser	GIPC1	0.000001	2.091423	
14972	Laser	UBQLN1	0.000027	2.102148	
14973	Laser	NRBP2	0.000035	2.108685	
14974	Laser	TCF25	0	2.109447	
14975	Laser	C15orf29	0.000029	2.116129	
14976	Laser	BTBD7	0.000002	2.126973	
14977	Laser	RWDD4	0.000003	2.134226	
14978	Laser	CD47	0.000001	2.140598	
14979	Laser	CUX1	0.000001	2.151	
14980	Laser	LPP	0	2.152973	
14981	Laser	GSPT1	0.000016	2.160143	
14982	Laser	SSBP3	0.000006	2.172406	
14983	Laser	2-Sep	0	2.191851	
14984	Laser	UHRF2	0.000007	2.214981	
14985	Laser	RPL37A	0.000029	2.225608	
14986	Laser	PTMA	0.000006	2.237295	
14987	Laser	FAM120A	0	2.238424	
14988	Laser	ZMPSTE24	0	2.277996	
14989	Laser	CDK5RAP2	0.000001	2.302563	
14990	Laser	TMEM181	0	2.31098	
14991	Laser	CCDC149	0.000025	2.335143	
14992	Laser	SAMD9	0.000038	2.341064	
14993	Laser	TIMM17A	0	2.342172	
14994	Laser	MBD1	0	2.350612	
14995	Laser	RFK	0.000001	2.368699	
14996	Laser	FAM41C	0	2.380954	
14997	Laser	SPCS3	0	2.421203	
14998	Laser	MBD2	0.000011	2.43081	

14999	Laser	TRIM38	0.000023	2.432439	
15000	Laser	FIG4	0.00001	2.443047	
15001	Laser	GNAQ	0	2.501042	
15002	Laser	CTTNBP2NL	0	2.578636	
15003	Laser	MYCBP2	0.00001	2.602477	
15004	Laser	IQGAP1	0.000001	2.669913	
15005	Laser	GPATCH4	0.000035	2.727949	
15006	Laser	PIK3C3	0	2.751652	
15007	Laser	ODF3B	0.000002	2.781268	
15008	Laser	LRRFIP1	0	2.782054	
15009	Laser	ANAPC4	0.000007	3.098416	
15010	Laser	AK3	0.000002	3.181674	
15011	Laser	VPS13B	0.000002	3.262962	
15012	Laser	ZDHHC21	0.000017	3.335738	
15013	Laser	BTN3A3	0.00002	3.360597	
15014	Laser	IKBKB	0.000001	3.370782	
15015	Laser	PTAR1	0.000001	3.396673	
15016	Laser	RABGAP1L	0.000011	3.401429	
15017	Laser	CRYBG3	0.000006	3.434472	
15018	Laser	ZBTB20	0	3.657749	
15019	Laser	RUNX1	0.000012	3.671123	
15020	Laser	TAP1	0.00003	3.697397	
15021	Laser	GALNT1	0.000013	3.774027	
15022	Laser	RAC2	0.000003	3.96965	
15023	Laser	ZDHHC2	0.000039	4.599631	
15024	Laser	HRH1	0.000001	4.733727	
15025	Laser	LYST	0.000005	4.875052	
15026	Laser	AHI1	0	4.9331	
15027	Laser	NAV2	0.000001	5.226256	
15028	Laser	TUSC1	0	5.406527	
15029	Laser	ZNF420	0.000038	5.43613	
15030	Laser	PSD3	0	5.751878	
15031	Laser	OXCT1	0.000013	5.786947	
15032	Laser	CADPS2	0.00003	5.823734	
15033	Laser	SPATA18	0.000015	6.297241	
15034	Laser	C3orf70	0.000001	6.970345	
15035	Laser	RCBTB2	0	7.4307	
15036	Laser	NIPAL2	0.000028	7.781628	
15037	Laser	ETS1	0.000003	8.555891	
15038	Laser	SEMA5A	0	9.239055	
15039	Laser	CELF2	0	9.856363	
15040	Laser	PHF11	0.000001	10.230079	
15041	Laser	STAMBPL1	0.000001	11.488363	
15042	Laser	NFIB	0.000001	11.57896	
15043	Laser	FAS	0.000001	15.020867	
15044	Laser	LXN	0.00003	15.84873	
15045	Laser	PRKACB	0	16.586677	
15046	Laser	ALCAM	0.000005	17.210558	
15047	Laser	RNF135	0.000003	18.253457	
15048	Laser	HLA-DPA1	0.000028	21.293448	

15049	Laser	COL3A1	0.000011	137.452893	
15050	Liver neoplasms	CLCA1	0	-37.400519	
15051	Liver neoplasms	SLC26A3	0	-23.603974	
15052	Liver neoplasms	IGHA1	0	-11.899987	
15053	Liver neoplasms	C9orf23	0	-10.808603	
15054	Liver neoplasms	GCG	0	-10.543371	
15055	Liver neoplasms	GTF2B	0	-9.59045	
15056	Liver neoplasms	ENAM	0	-8.013751	
15057	Liver neoplasms	CEACAM7	0	-7.413905	
15058	Liver neoplasms	PIGR	0	-6.867776	
15059	Liver neoplasms	CXCL14	0	-6.657576	
15060	Liver neoplasms	IGHG1	0	-6.475037	
15061	Liver neoplasms	NR4A2	0	-6.116631	
15062	Liver neoplasms	TSPAN1	0	-6.014172	
15063	Liver neoplasms	ADAMDEC1	0	-5.9163	
15064	Liver neoplasms	C10orf99	0	-5.869133	
15065	Liver neoplasms	PYCR2	0.000009	-5.73518	
15066	Liver neoplasms	HLA-C	0	-5.56222	
15067	Liver neoplasms	IGLL1	0	-4.856665	
15068	Liver neoplasms	CCL11	0	-4.849366	
15069	Liver neoplasms	PLA2G2A	0	-4.651739	
15070	Liver neoplasms	MUC2	0	-4.417867	
15071	Liver neoplasms	LRRC19	0.000002	-4.283599	
15072	Liver neoplasms	PADI2	0	-4.153545	
15073	Liver neoplasms	DHRS9	0	-4.146086	
15074	Liver neoplasms	REP15	0	-4.133384	
15075	Liver neoplasms	IGL@	0	-4.09573	
15076	Liver neoplasms	SPON1	0	-3.80835	
15077	Liver neoplasms	KLF4	0	-3.65689	
15078	Liver neoplasms	FN3K	0	-3.598249	
15079	Liver neoplasms	HSD11B2	0	-3.543672	
15080	Liver neoplasms	MALT1	0	-3.532754	
15081	Liver neoplasms	TFF3	0.000007	-3.525928	
15082	Liver neoplasms	FXYD3	0.000009	-3.353044	
15083	Liver neoplasms	DKFZP564O08	0	-3.231508	
15084	Liver neoplasms	C12orf32	0	-3.185808	
15085	Liver neoplasms	MEP1A	0.000011	-3.079247	
15086	Liver neoplasms	PIM2	0	-2.977715	
15087	Liver neoplasms	FAM44B	0	-2.849228	
15088	Liver neoplasms	RABL5	0	-2.830244	
15089	Liver neoplasms	PDGFRA	0.000009	-2.820959	
15090	Liver neoplasms	COLQ	0.000012	-2.787527	
15091	Liver neoplasms	CPA3	0	-2.742683	
15092	Liver neoplasms	FRZB	0	-2.731391	
15093	Liver neoplasms	MYLK	0	-2.727469	
15094	Liver neoplasms	PTGER4	0	-2.681329	
15095	Liver neoplasms	TNFRSF17	0	-2.654046	
15096	Liver neoplasms	FLJ21511	0	-2.626738	
15097	Liver neoplasms	RIPK1	0.000008	-2.600399	
15098	Liver neoplasms	MUC4	0	-2.546818	

15099	Liver neoplasms	SELENBP1	0.000002	-2.546401	
15100	Liver neoplasms	IGLV6-57	0	-2.543077	
15101	Liver neoplasms	CTSK	0	-2.504462	
15102	Liver neoplasms	GSN	0	-2.48907	
15103	Liver neoplasms	CD79A	0	-2.488098	
15104	Liver neoplasms	FGF7	0	-2.340139	
15105	Liver neoplasms	BDKRB2	0	-2.338755	
15106	Liver neoplasms	PARP10	0	-2.316422	
15107	Liver neoplasms	ETHE1	0	-2.311634	
15108	Liver neoplasms	DSCR1L1	0.000004	-2.302937	
15109	Liver neoplasms	SCNN1A	0.000011	-2.280739	
15110	Liver neoplasms	GOLPH2	0.000001	-2.261735	
15111	Liver neoplasms	IL1R2	0	-2.230464	
15112	Liver neoplasms	ST6GALNAC6	0	-2.21554	
15113	Liver neoplasms	LOH11CR2A	0	-2.173129	
15114	Liver neoplasms	MYL9	0.000002	-2.120174	
15115	Liver neoplasms	KCNMB1	0	-2.109556	
15116	Liver neoplasms	POU2AF1	0	-2.093548	
15117	Liver neoplasms	CNN1	0.000009	-2.089465	
15118	Liver neoplasms	TPSAB1	0	-2.074847	
15119	Liver neoplasms	SIAE	0	-2.056617	
15120	Liver neoplasms	FRYL	0.000001	-2.048906	
15121	Liver neoplasms	FKBP1A	0	-2.028103	
15122	Liver neoplasms	TIMP1	0.000006	2.422773	
15123	Liver neoplasms	ADFP	0.000005	3.308015	
15124	Liver neoplasms	TGM4	0.000011	3.756878	
15125	Liver neoplasms	CFB	0.000004	4.800839	
15126	Liver neoplasms	AGT	0.000002	6.2148	
15127	Liver neoplasms	PAH	0.000007	6.401863	
15128	Liver neoplasms	APOA2	0.000003	7.224515	
15129	Liver neoplasms	CFHR1	0.000011	7.42292	
15130	Liver neoplasms	C4BPA	0.000003	7.579072	
15131	Liver neoplasms	FMO3	0.000001	7.948161	
15132	Liver neoplasms	ALDOB	0	8.445676	
15133	Liver neoplasms	GC	0	8.682619	
15134	Liver neoplasms	TOMM40	0	9.333405	
15135	Liver neoplasms	AQP3	0	10.798013	
15136	Liver neoplasms	TTR	0.000001	11.607525	
15137	Liver neoplasms	PLG	0	11.915053	
15138	Liver neoplasms	SAA2	0	12.00583	
15139	Liver neoplasms	TF	0	14.54572	
15140	Malignant neoplasm of ovary	CGI-38	0.003991	-2.78059	
15141	Malignant neoplasm of ovary	SETBP1	0.006355	-2.081398	
15142	Malignant neoplasm of ovary	SDK1	0.003991	2.026087	
15143	Malignant neoplasm of ovary	MS4A4A	0.010815	2.180689	
15144	Malignant neoplasm of ovary	ARL4A	0.027133	2.30044	
15145	Malignant neoplasm of ovary	ST3GAL2	0.000845	2.361099	
15146	Malignant neoplasm of ovary	TAP2	0.020351	2.516374	
15147	Malignant neoplasm of ovary	LOC440607	0.044929	2.670231	
15148	Malignant neoplasm of ovary	SAMD9	0.035369	2.792032	

15149	Malignant neoplasm of ovary	IFI44	0.020351	3.348431	
15150	Malignant neoplasm of ovary	CLEC4D	0.027133	3.620512	
15151	Malignant neoplasm of ovary	MGC10233	0.031326	3.995307	
15152	Malignant neoplasm of prostate	SMCY	0.040675	6.353116	
15153	Malignant neoplasm of prostate	RPS4Y2	0.039175	13.187671	
15154	Malignant neoplasm of prostate	RPS4Y1	0.039175	56.699911	
15155	Malignant neoplasm of thyroid	TPST2	0.000258	2.332694	
15156	Malignant Neoplasms	EEF1D	0	-699.889116	
15157	Malignant Neoplasms	EIF4A1	0	-684.841838	
15158	Malignant Neoplasms	SUB1	0	-581.649816	
15159	Malignant Neoplasms	YWHAQ	0	-570.316395	
15160	Malignant Neoplasms	ZC3H11A	0	-525.045515	
15161	Malignant Neoplasms	EIF3E	0	-503.11703	
15162	Malignant Neoplasms	SRSF3	0	-473.877715	
15163	Malignant Neoplasms	PFDN5	0	-466.742321	
15164	Malignant Neoplasms	HSP90AB1	0	-439.562879	
15165	Malignant Neoplasms	SMNDC1	0	-355.774331	
15166	Malignant Neoplasms	PSMB1	0	-330.393137	
15167	Malignant Neoplasms	MPRIP	0	-322.778333	
15168	Malignant Neoplasms	SLC2A3	0.000047	-313.137976	
15169	Malignant Neoplasms	TRMT112	0	-270.472615	
15170	Malignant Neoplasms	APCDD1	0.000233	-263.329122	
15171	Malignant Neoplasms	DNAJB6	0	-256.868942	
15172	Malignant Neoplasms	C19orf43	0	-244.243227	
15173	Malignant Neoplasms	MYEOV2	0	-240.63904	
15174	Malignant Neoplasms	PSMB6	0	-235.554357	
15175	Malignant Neoplasms	LOC100506727	0	-229.540048	
15176	Malignant Neoplasms	DYNC1LI2	0	-229.119824	
15177	Malignant Neoplasms	ZNF410	0	-211.529045	
15178	Malignant Neoplasms	MZT2B	0	-209.966747	
15179	Malignant Neoplasms	YTHDC1	0	-202.676108	
15180	Malignant Neoplasms	JAK1	0	-202.082868	
15181	Malignant Neoplasms	ATP6V1F	0	-200.97053	
15182	Malignant Neoplasms	EIF1	0	-199.160288	
15183	Malignant Neoplasms	MZT2A	0	-199.159894	
15184	Malignant Neoplasms	CSNK1A1	0	-194.191192	
15185	Malignant Neoplasms	UBXN6	0	-192.129411	
15186	Malignant Neoplasms	ISCU	0	-190.072363	
15187	Malignant Neoplasms	BUD31	0	-188.93846	
15188	Malignant Neoplasms	PTMA	0	-186.499335	
15189	Malignant Neoplasms	C3orf10	0	-183.60437	
15190	Malignant Neoplasms	POLR2L	0	-181.373259	
15191	Malignant Neoplasms	DDX3X	0	-179.635009	
15192	Malignant Neoplasms	TBCB	0	-172.307453	
15193	Malignant Neoplasms	CFLAR	0	-170.967163	
15194	Malignant Neoplasms	ACLY	0	-170.716949	
15195	Malignant Neoplasms	MCL1	0	-161.393914	
15196	Malignant Neoplasms	SRRM1	0	-159.82399	
15197	Malignant Neoplasms	PRICKLE4	0	-158.754106	
15198	Malignant Neoplasms	REEP5	0	-157.942777	

15199	Malignant Neoplasms	DYNLRB1	0	-157.800676	
15200	Malignant Neoplasms	HNRNPC	0	-156.272469	
15201	Malignant Neoplasms	CNOT2	0	-156.005853	
15202	Malignant Neoplasms	NPIP	0	-155.421505	
15203	Malignant Neoplasms	TGFBR2	0	-153.220942	
15204	Malignant Neoplasms	BTG3	0	-149.838851	
15205	Malignant Neoplasms	ELOVL5	0.000647	-148.528674	
15206	Malignant Neoplasms	RBPJ	0	-146.705048	
15207	Malignant Neoplasms	MRPS10	0	-145.332824	
15208	Malignant Neoplasms	EIF2S2	0	-143.618265	
15209	Malignant Neoplasms	CIAPIN1	0	-143.238303	
15210	Malignant Neoplasms	SPCS3	0	-141.698023	
15211	Malignant Neoplasms	MLL2	0	-141.444741	
15212	Malignant Neoplasms	HNRNPH1	0	-139.54796	
15213	Malignant Neoplasms	RSL24D1	0	-136.723139	
15214	Malignant Neoplasms	SEC62	0	-136.128219	
15215	Malignant Neoplasms	NGFRAP1	0.000085	-135.816614	
15216	Malignant Neoplasms	ZFAND5	0	-135.27416	
15217	Malignant Neoplasms	C1orf52	0	-135.128515	
15218	Malignant Neoplasms	TMEM47	0.000084	-134.314673	
15219	Malignant Neoplasms	KRT5	0.000647	-131.072004	
15220	Malignant Neoplasms	HK1	0	-130.921963	
15221	Malignant Neoplasms	LOC220906	0	-129.519923	
15222	Malignant Neoplasms	WAC	0	-129.473693	
15223	Malignant Neoplasms	RBP1	0.000196	-125.448288	
15224	Malignant Neoplasms	CNKSR3	0.000316	-124.830849	
15225	Malignant Neoplasms	COMMD6	0	-124.577367	
15226	Malignant Neoplasms	MYST3	0	-122.384709	
15227	Malignant Neoplasms	H3F3B	0	-120.314174	
15228	Malignant Neoplasms	MACF1	0	-119.418087	
15229	Malignant Neoplasms	C14orf4	0	-119.038609	
15230	Malignant Neoplasms	ATP6V1G2	0	-118.084678	
15231	Malignant Neoplasms	PSMB4	0	-117.57619	
15232	Malignant Neoplasms	MSL1	0	-117.483846	
15233	Malignant Neoplasms	WTAP	0	-116.752754	
15234	Malignant Neoplasms	SRRM2	0	-116.658515	
15235	Malignant Neoplasms	RNF13	0	-115.277865	
15236	Malignant Neoplasms	RNASEH1	0	-114.769009	
15237	Malignant Neoplasms	SLC2A14	0.000351	-114.078426	
15238	Malignant Neoplasms	PSME4	0	-113.821707	
15239	Malignant Neoplasms	SETD5	0	-113.745575	
15240	Malignant Neoplasms	SCAND1	0	-113.527401	
15241	Malignant Neoplasms	SETD2	0	-113.230505	
15242	Malignant Neoplasms	NEDD8	0	-112.20158	
15243	Malignant Neoplasms	TMEM189-UBI	0	-111.605109	
15244	Malignant Neoplasms	C4orf3	0	-110.813019	
15245	Malignant Neoplasms	UBE3A	0	-108.416905	
15246	Malignant Neoplasms	ADD1	0	-107.933573	
15247	Malignant Neoplasms	BRD3	0	-107.645846	
15248	Malignant Neoplasms	POM121	0	-105.420051	

15249	Malignant Neoplasms	PICALM	0	-102.678326	
15250	Malignant Neoplasms	MRPL20	0	-101.125345	
15251	Malignant Neoplasms	DUSP6	0	-101.122739	
15252	Malignant Neoplasms	RBM5	0	-100.742038	
15253	Malignant Neoplasms	FAM120B	0.000075	-100.720277	
15254	Malignant Neoplasms	DCTD	0	-100.18793	
15255	Malignant Neoplasms	PSMD4	0	-98.660851	
15256	Malignant Neoplasms	SLC35E1	0	-97.57434	
15257	Malignant Neoplasms	C11orf58	0	-97.433569	
15258	Malignant Neoplasms	MEAF6	0	-97.309396	
15259	Malignant Neoplasms	SYNCRIP	0	-96.992912	
15260	Malignant Neoplasms	UBP1	0	-94.265215	
15261	Malignant Neoplasms	AFF4	0	-93.762331	
15262	Malignant Neoplasms	ARHGAP1	0	-93.423106	
15263	Malignant Neoplasms	RAD23A	0	-93.06712	
15264	Malignant Neoplasms	MID1	0.000445	-92.787503	
15265	Malignant Neoplasms	NFATC2IP	0	-91.7713	
15266	Malignant Neoplasms	PTPN11	0	-90.719416	
15267	Malignant Neoplasms	MGAT1	0	-88.827949	
15268	Malignant Neoplasms	MSX1	0.000572	-88.746168	
15269	Malignant Neoplasms	BRD4	0	-88.051181	
15270	Malignant Neoplasms	HERC1	0	-86.752432	
15271	Malignant Neoplasms	CHMP4A	0	-86.578022	
15272	Malignant Neoplasms	PHC3	0	-85.398545	
15273	Malignant Neoplasms	DNAJC2	0	-85.096815	
15274	Malignant Neoplasms	PARL	0	-84.900526	
15275	Malignant Neoplasms	RDBP	0	-83.966271	
15276	Malignant Neoplasms	SERINC3	0	-82.012606	
15277	Malignant Neoplasms	SIK3	0	-81.755965	
15278	Malignant Neoplasms	RABGAP1	0	-81.645394	
15279	Malignant Neoplasms	ZNRD1	0	-81.423108	
15280	Malignant Neoplasms	VIM	0.000244	-81.143008	
15281	Malignant Neoplasms	DIMT1L	0	-81.117746	
15282	Malignant Neoplasms	CTSB	0	-80.751663	
15283	Malignant Neoplasms	EPDR1	0.000647	-79.341374	
15284	Malignant Neoplasms	CCNL1	0	-77.954632	
15285	Malignant Neoplasms	PRF1	0.000647	-77.850672	
15286	Malignant Neoplasms	KPNA4	0	-77.504852	
15287	Malignant Neoplasms	HSP90AA1	0	-77.038638	
15288	Malignant Neoplasms	RABGGTB	0	-75.150883	
15289	Malignant Neoplasms	ANKRD10	0	-75.079577	
15290	Malignant Neoplasms	9-Sep	0	-74.975623	
15291	Malignant Neoplasms	GYG1	0	-74.679465	
15292	Malignant Neoplasms	CORO1C	0	-74.629684	
15293	Malignant Neoplasms	7-Sep	0	-74.404495	
15294	Malignant Neoplasms	STUB1	0	-74.062889	
15295	Malignant Neoplasms	ATRX	0	-73.382766	
15296	Malignant Neoplasms	SOAT1	0	-72.919006	
15297	Malignant Neoplasms	WBSCR22	0	-71.417114	
15298	Malignant Neoplasms	PEA15	0	-71.00403	

15299	Malignant Neoplasms	PEBP1	0	-69.881828	
15300	Malignant Neoplasms	UTP11L	0	-68.102018	
15301	Malignant Neoplasms	KCTD20	0	-68.072448	
15302	Malignant Neoplasms	WASF3	0.000458	-67.506757	
15303	Malignant Neoplasms	PHF2	0	-67.473383	
15304	Malignant Neoplasms	LUC7L2	0	-67.038861	
15305	Malignant Neoplasms	ABL1	0	-66.809892	
15306	Malignant Neoplasms	GATAD2A	0	-66.178048	
15307	Malignant Neoplasms	LYRM1	0	-65.524952	
15308	Malignant Neoplasms	MESDC1	0	-65.136486	
15309	Malignant Neoplasms	INO80C	0	-64.323668	
15310	Malignant Neoplasms	CST1	0.000284	-63.462269	
15311	Malignant Neoplasms	ABAT	0.000333	-63.352896	
15312	Malignant Neoplasms	PTOV1	0	-62.830318	
15313	Malignant Neoplasms	GNL3	0	-62.696975	
15314	Malignant Neoplasms	TDG	0	-62.606778	
15315	Malignant Neoplasms	DDX10	0	-62.26356	
15316	Malignant Neoplasms	IGFBP3	0.000244	-62.153712	
15317	Malignant Neoplasms	RBFOX2	0	-61.832666	
15318	Malignant Neoplasms	C2orf69	0	-61.637564	
15319	Malignant Neoplasms	C19orf70	0	-61.27213	
15320	Malignant Neoplasms	HIPK3	0	-60.905003	
15321	Malignant Neoplasms	TUBGCP2	0	-60.316121	
15322	Malignant Neoplasms	MUC4	0.001993	-60.311531	
15323	Malignant Neoplasms	SMC5	0	-60.104897	
15324	Malignant Neoplasms	MUC2	0.001405	-60.058351	
15325	Malignant Neoplasms	DPM1	0	-59.475498	
15326	Malignant Neoplasms	WASH3P	0	-59.124112	
15327	Malignant Neoplasms	TCEAL3	0	-58.771225	
15328	Malignant Neoplasms	NME4	0	-58.535729	
15329	Malignant Neoplasms	CADPS2	0.000355	-58.269955	
15330	Malignant Neoplasms	DSE	0	-57.530285	
15331	Malignant Neoplasms	GAL	0.000686	-57.026708	
15332	Malignant Neoplasms	GAPVD1	0	-56.718241	
15333	Malignant Neoplasms	PEF1	0	-56.575719	
15334	Malignant Neoplasms	PITPNB	0	-56.567139	
15335	Malignant Neoplasms	DTNBP1	0	-56.142809	
15336	Malignant Neoplasms	OGT	0	-55.316082	
15337	Malignant Neoplasms	ARPP19	0	-55.135499	
15338	Malignant Neoplasms	NOP2	0	-54.37258	
15339	Malignant Neoplasms	FAM38A	0	-54.307464	
15340	Malignant Neoplasms	MCM3AP	0	-54.062299	
15341	Malignant Neoplasms	HIPK2	0.000291	-53.636186	
15342	Malignant Neoplasms	MAP3K4	0	-53.438254	
15343	Malignant Neoplasms	SYNJ2	0	-53.419968	
15344	Malignant Neoplasms	RALGDS	0	-53.395158	
15345	Malignant Neoplasms	EIF1B	0	-53.285117	
15346	Malignant Neoplasms	ETF1	0	-52.741184	
15347	Malignant Neoplasms	AAK1	0	-52.387023	
15348	Malignant Neoplasms	WSB1	0	-52.153317	

15349	Malignant Neoplasms	ITPA	0	-52.109892	
15350	Malignant Neoplasms	CST7	0.00073	-51.995482	
15351	Malignant Neoplasms	BAT2L2	0	-51.783462	
15352	Malignant Neoplasms	AGFG1	0	-51.450292	
15353	Malignant Neoplasms	CD44	0	-51.404211	
15354	Malignant Neoplasms	YTHDF1	0	-51.053256	
15355	Malignant Neoplasms	SF1	0	-50.622916	
15356	Malignant Neoplasms	MLXIPL	0.000135	-49.95375	
15357	Malignant Neoplasms	FLOT1	0	-49.01746	
15358	Malignant Neoplasms	JMY	0	-48.76432	
15359	Malignant Neoplasms	GPATCH4	0	-48.577115	
15360	Malignant Neoplasms	RC3H1	0	-48.332081	
15361	Malignant Neoplasms	ESYT2	0	-48.124221	
15362	Malignant Neoplasms	USP48	0	-47.980034	
15363	Malignant Neoplasms	CARS	0	-47.938461	
15364	Malignant Neoplasms	GUK1	0	-47.833795	
15365	Malignant Neoplasms	ARPC5L	0	-46.684321	
15366	Malignant Neoplasms	WDR5	0	-46.139731	
15367	Malignant Neoplasms	CCDC130	0	-46.01197	
15368	Malignant Neoplasms	ABHD12B	0.000114	-45.527992	
15369	Malignant Neoplasms	SLC16A6	0.000112	-45.020838	
15370	Malignant Neoplasms	GLI3	0.000538	-44.985278	
15371	Malignant Neoplasms	RBM39	0	-44.756391	
15372	Malignant Neoplasms	TNPO1	0	-44.533533	
15373	Malignant Neoplasms	DDX18	0	-43.936319	
15374	Malignant Neoplasms	C17orf79	0	-43.463288	
15375	Malignant Neoplasms	LRRC58	0	-43.039506	
15376	Malignant Neoplasms	FERMT2	0.000392	-42.651875	
15377	Malignant Neoplasms	TUBB3	0	-42.432917	
15378	Malignant Neoplasms	SOD3	0	-42.369169	
15379	Malignant Neoplasms	DAZAP1	0	-42.33826	
15380	Malignant Neoplasms	RRAGD	0.000244	-41.635175	
15381	Malignant Neoplasms	TMED9	0	-41.153279	
15382	Malignant Neoplasms	SEC22C	0	-41.10736	
15383	Malignant Neoplasms	GSPT1	0	-41.093295	
15384	Malignant Neoplasms	NFX1	0	-40.983529	
15385	Malignant Neoplasms	PKIG	0	-40.875872	
15386	Malignant Neoplasms	AIDA	0	-40.547627	
15387	Malignant Neoplasms	RAI1	0	-40.436391	
15388	Malignant Neoplasms	SSH1	0	-40.001006	
15389	Malignant Neoplasms	PMP22	0.000573	-39.852473	
15390	Malignant Neoplasms	MPV17	0	-39.576468	
15391	Malignant Neoplasms	CHST11	0.000647	-39.427776	
15392	Malignant Neoplasms	SF3B4	0	-39.27583	
15393	Malignant Neoplasms	PCDHGA1	0	-39.092216	
15394	Malignant Neoplasms	SLC25A14	0	-39.035575	
15395	Malignant Neoplasms	SRSF5	0	-38.767781	
15396	Malignant Neoplasms	GTPBP4	0	-38.623321	
15397	Malignant Neoplasms	ACTN1	0	-38.003615	
15398	Malignant Neoplasms	ZNF259	0	-37.790142	

15399	Malignant Neoplasms	TNFAIP8L1	0	-36.129553	
15400	Malignant Neoplasms	USP14	0	-35.881885	
15401	Malignant Neoplasms	LOC283508	0	-35.404046	
15402	Malignant Neoplasms	NENF	0	-35.315207	
15403	Malignant Neoplasms	CDK11A	0	-35.199934	
15404	Malignant Neoplasms	EXOC3	0	-34.887351	
15405	Malignant Neoplasms	PURB	0	-34.512453	
15406	Malignant Neoplasms	PFDN2	0	-33.726677	
15407	Malignant Neoplasms	TNRC18	0	-33.087083	
15408	Malignant Neoplasms	LEF1	0.000244	-33.080288	
15409	Malignant Neoplasms	UBQLN2	0	-33.054777	
15410	Malignant Neoplasms	EIF5B	0	-32.997592	
15411	Malignant Neoplasms	DNM1L	0	-32.985717	
15412	Malignant Neoplasms	MRFAP1	0	-32.897746	
15413	Malignant Neoplasms	IDS	0	-32.894405	
15414	Malignant Neoplasms	OAZ2	0	-32.607813	
15415	Malignant Neoplasms	TAF1D	0	-32.601524	
15416	Malignant Neoplasms	LOC643837	0	-32.236147	
15417	Malignant Neoplasms	BRD8	0	-32.196672	
15418	Malignant Neoplasms	WNT5A	0.000088	-32.108019	
15419	Malignant Neoplasms	TCF25	0	-32.093011	
15420	Malignant Neoplasms	CAPZB	0	-31.885451	
15421	Malignant Neoplasms	CUEDC2	0	-31.847655	
15422	Malignant Neoplasms	RBMS1	0	-31.73637	
15423	Malignant Neoplasms	LOC221710	0	-31.649041	
15424	Malignant Neoplasms	TFE3	0	-31.46333	
15425	Malignant Neoplasms	TRBC1	0.000239	-31.338611	
15426	Malignant Neoplasms	SNRPB2	0	-31.300164	
15427	Malignant Neoplasms	C20orf43	0	-31.282945	
15428	Malignant Neoplasms	RFNG	0	-31.043018	
15429	Malignant Neoplasms	SEC16A	0	-30.972818	
15430	Malignant Neoplasms	GJC1	0.000244	-30.630617	
15431	Malignant Neoplasms	MAP7D2	0.000019	-30.599756	
15432	Malignant Neoplasms	NGRN	0	-30.570196	
15433	Malignant Neoplasms	MTCH1	0	-30.45331	
15434	Malignant Neoplasms	SMAD5	0	-30.406751	
15435	Malignant Neoplasms	LOC100130468	0.000244	-29.523954	
15436	Malignant Neoplasms	CDC42SE1	0	-29.52391	
15437	Malignant Neoplasms	C12orf44	0	-29.507961	
15438	Malignant Neoplasms	CNPY2	0	-29.461476	
15439	Malignant Neoplasms	SREK1IP1	0	-29.447805	
15440	Malignant Neoplasms	H1FX	0	-29.380688	
15441	Malignant Neoplasms	HDGFRP3	0.000085	-29.291365	
15442	Malignant Neoplasms	SNHG7	0	-28.954788	
15443	Malignant Neoplasms	ZNF451	0	-28.662582	
15444	Malignant Neoplasms	VKORC1	0	-28.591366	
15445	Malignant Neoplasms	CAPRIN2	0	-28.282264	
15446	Malignant Neoplasms	DCTN6	0	-28.167369	
15447	Malignant Neoplasms	CLASRP	0	-28.056142	
15448	Malignant Neoplasms	YPEL5	0	-27.965317	

15449	Malignant Neoplasms	UBE2F	0	-27.781424	
15450	Malignant Neoplasms	SRSF11	0	-27.780094	
15451	Malignant Neoplasms	FOXP2	0.002498	-27.659136	
15452	Malignant Neoplasms	B3GNT6	0.000062	-27.448227	
15453	Malignant Neoplasms	ZAP70	0.000462	-26.912814	
15454	Malignant Neoplasms	MAP1B	0.000047	-26.873965	
15455	Malignant Neoplasms	ATP8B2	0	-26.841983	
15456	Malignant Neoplasms	CSRP2	0.000095	-26.742258	
15457	Malignant Neoplasms	SOCS2	0	-26.017583	
15458	Malignant Neoplasms	SAR1A	0	-25.877226	
15459	Malignant Neoplasms	ASB4	0.000622	-25.719803	
15460	Malignant Neoplasms	DDX21	0	-25.718588	
15461	Malignant Neoplasms	DCTN3	0	-25.631929	
15462	Malignant Neoplasms	PER2	0	-25.560169	
15463	Malignant Neoplasms	SCGB2A1	0.00296	-25.494518	
15464	Malignant Neoplasms	CCNL2	0	-25.488195	
15465	Malignant Neoplasms	LRRN4	0.000377	-25.295268	
15466	Malignant Neoplasms	MYADM	0	-25.033701	
15467	Malignant Neoplasms	HRAS	0	-24.707673	
15468	Malignant Neoplasms	MMP28	0.00029	-24.383882	
15469	Malignant Neoplasms	KIAA0020	0	-24.258286	
15470	Malignant Neoplasms	NAA15	0	-24.236099	
15471	Malignant Neoplasms	UBAP2	0	-24.099723	
15472	Malignant Neoplasms	PKD1	0	-23.726431	
15473	Malignant Neoplasms	RAB28	0	-23.572336	
15474	Malignant Neoplasms	NMD3	0	-23.570179	
15475	Malignant Neoplasms	WDYHV1	0	-23.564175	
15476	Malignant Neoplasms	ADPGK	0	-23.419754	
15477	Malignant Neoplasms	DDX27	0	-23.366875	
15478	Malignant Neoplasms	HNRNPUL1	0	-23.192493	
15479	Malignant Neoplasms	C14orf138	0	-23.168943	
15480	Malignant Neoplasms	CIRBP	0	-23.102411	
15481	Malignant Neoplasms	AHSA1	0	-23.050441	
15482	Malignant Neoplasms	ATF5	0	-22.998995	
15483	Malignant Neoplasms	TNFRSF1B	0.000304	-22.658124	
15484	Malignant Neoplasms	LASS5	0	-22.475119	
15485	Malignant Neoplasms	MAPKAPK2	0	-22.431173	
15486	Malignant Neoplasms	LIMS3	0.000233	-22.420179	
15487	Malignant Neoplasms	SEPW1	0	-22.366304	
15488	Malignant Neoplasms	ABCE1	0	-22.149229	
15489	Malignant Neoplasms	TSSC4	0	-22.081952	
15490	Malignant Neoplasms	CTSL1	0	-22.06845	
15491	Malignant Neoplasms	ATP13A3	0	-21.795814	
15492	Malignant Neoplasms	KCNMA1	0.000529	-21.649194	
15493	Malignant Neoplasms	UBR4	0	-21.558458	
15494	Malignant Neoplasms	NCOR2	0	-21.492022	
15495	Malignant Neoplasms	NXF1	0	-21.392261	
15496	Malignant Neoplasms	PELO	0	-21.288685	
15497	Malignant Neoplasms	LRMP	0.0036	-20.996778	
15498	Malignant Neoplasms	CWH43	0.00122	-20.467763	

15499	Malignant Neoplasms	UBE2G2	0	-20.125611	
15500	Malignant Neoplasms	PASD1	0.000244	-20.078643	
15501	Malignant Neoplasms	SERTAD4	0.000207	-20.029386	
15502	Malignant Neoplasms	ID2	0	-19.990605	
15503	Malignant Neoplasms	PDE4A	0	-19.934587	
15504	Malignant Neoplasms	TRPV2	0.000056	-19.866161	
15505	Malignant Neoplasms	GJA3	0.000462	-19.010394	
15506	Malignant Neoplasms	SPDEF	0.003466	-18.98549	
15507	Malignant Neoplasms	MCFD2	0	-18.663903	
15508	Malignant Neoplasms	GPR124	0	-18.651304	
15509	Malignant Neoplasms	TRIM33	0	-18.618862	
15510	Malignant Neoplasms	QKI	0.000056	-18.551642	
15511	Malignant Neoplasms	L1CAM	0.000112	-18.539945	
15512	Malignant Neoplasms	MARK3	0	-18.521941	
15513	Malignant Neoplasms	ZCRB1	0	-18.277216	
15514	Malignant Neoplasms	UGCG	0	-18.250936	
15515	Malignant Neoplasms	LOC285074	0	-18.23513	
15516	Malignant Neoplasms	GDI1	0	-18.221594	
15517	Malignant Neoplasms	LHFP	0.000351	-18.214573	
15518	Malignant Neoplasms	PIP5K1A	0	-18.130832	
15519	Malignant Neoplasms	RUNX3	0.000095	-18.120496	
15520	Malignant Neoplasms	HEG1	0.000392	-17.931892	
15521	Malignant Neoplasms	DCAF16	0	-17.846178	
15522	Malignant Neoplasms	RNF8	0	-17.766129	
15523	Malignant Neoplasms	EPHX2	0.000521	-17.745048	
15524	Malignant Neoplasms	C4orf46	0	-17.37894	
15525	Malignant Neoplasms	UBE2H	0	-17.318821	
15526	Malignant Neoplasms	PLAGL1	0.000244	-17.310978	
15527	Malignant Neoplasms	NUB1	0	-17.289582	
15528	Malignant Neoplasms	STC2	0.000084	-17.253821	
15529	Malignant Neoplasms	RGPD4	0	-17.187576	
15530	Malignant Neoplasms	CDC37L1	0	-17.162162	
15531	Malignant Neoplasms	FN1	0.000122	-16.972906	
15532	Malignant Neoplasms	BHLHE40	0.000286	-16.543643	
15533	Malignant Neoplasms	BPGM	0	-16.456924	
15534	Malignant Neoplasms	LOC100509635	0.000651	-16.450784	
15535	Malignant Neoplasms	UBE2R2	0	-16.333046	
15536	Malignant Neoplasms	CDC34	0	-16.242861	
15537	Malignant Neoplasms	CHST5	0.003988	-16.204897	
15538	Malignant Neoplasms	RAB24	0	-16.104401	
15539	Malignant Neoplasms	PPFIBP1	0.000448	-15.954224	
15540	Malignant Neoplasms	ANO5	0.000982	-15.875208	
15541	Malignant Neoplasms	FAM92A1	0.000667	-15.491007	
15542	Malignant Neoplasms	MAFK	0	-15.376366	
15543	Malignant Neoplasms	RAB31	0.000244	-15.274008	
15544	Malignant Neoplasms	ERGIC1	0	-15.187199	
15545	Malignant Neoplasms	PNPLA8	0	-15.171413	
15546	Malignant Neoplasms	SORBS1	0.000382	-15.059719	
15547	Malignant Neoplasms	TIMM23	0	-14.732531	
15548	Malignant Neoplasms	SAMD8	0	-14.713755	

15549	Malignant Neoplasms	KHSRP	0	-14.479842	
15550	Malignant Neoplasms	FAM89B	0	-14.341477	
15551	Malignant Neoplasms	LOC389834	0.000794	-14.277037	
15552	Malignant Neoplasms	OLFM1	0.000649	-14.088867	
15553	Malignant Neoplasms	FAM126A	0.000628	-14.084965	
15554	Malignant Neoplasms	CADM1	0.000096	-13.912684	
15555	Malignant Neoplasms	IL27RA	0.000402	-13.902396	
15556	Malignant Neoplasms	FGFR1	0	-13.825174	
15557	Malignant Neoplasms	FOXL2	0.000531	-13.69078	
15558	Malignant Neoplasms	FAM131A	0	-13.474246	
15559	Malignant Neoplasms	LOC100132273	0.000004	-13.370863	
15560	Malignant Neoplasms	RPF2	0	-13.364589	
15561	Malignant Neoplasms	PKD1P1	0	-13.270869	
15562	Malignant Neoplasms	SAFB2	0	-13.192629	
15563	Malignant Neoplasms	NID1	0.000088	-13.144856	
15564	Malignant Neoplasms	CD109	0.000095	-13.07047	
15565	Malignant Neoplasms	FADS1	0.000114	-13.017573	
15566	Malignant Neoplasms	NELF	0	-12.971041	
15567	Malignant Neoplasms	ZNF639	0	-12.820294	
15568	Malignant Neoplasms	DKK3	0.000071	-12.616375	
15569	Malignant Neoplasms	IQCJ-SCHIP1	0.000647	-12.560094	
15570	Malignant Neoplasms	MSRB2	0	-12.344785	
15571	Malignant Neoplasms	HOXC10	0.000833	-12.296945	
15572	Malignant Neoplasms	STMN3	0.000102	-12.028023	
15573	Malignant Neoplasms	MAN1C1	0	-12.015512	
15574	Malignant Neoplasms	CD1D	0.000358	-11.888861	
15575	Malignant Neoplasms	CACHD1	0.000581	-11.709604	
15576	Malignant Neoplasms	RSRC2	0	-11.672737	
15577	Malignant Neoplasms	MYO5A	0.000647	-11.66966	
15578	Malignant Neoplasms	CKMT2	0.000169	-11.600607	
15579	Malignant Neoplasms	KLF9	0.003783	-11.537512	
15580	Malignant Neoplasms	CCDC12	0	-11.480802	
15581	Malignant Neoplasms	PPP1R14C	0.000471	-11.465546	
15582	Malignant Neoplasms	PNMA2	0.000104	-11.233506	
15583	Malignant Neoplasms	BRD2	0	-11.178387	
15584	Malignant Neoplasms	C9orf30	0	-11.165766	
15585	Malignant Neoplasms	CCDC146	0.000022	-11.056943	
15586	Malignant Neoplasms	PHC2	0	-10.775748	
15587	Malignant Neoplasms	SPINK4	0	-10.604966	
15588	Malignant Neoplasms	ISG20L2	0	-10.556466	
15589	Malignant Neoplasms	SCIN	0.000675	-10.535578	
15590	Malignant Neoplasms	ZG16	0.000001	-10.523496	
15591	Malignant Neoplasms	MAT2A	0	-10.435096	
15592	Malignant Neoplasms	TIMP3	0.000675	-10.419209	
15593	Malignant Neoplasms	FAM83F	0.000419	-10.407689	
15594	Malignant Neoplasms	SAT2	0.000099	-10.402509	
15595	Malignant Neoplasms	ACOT11	0.004247	-10.396793	
15596	Malignant Neoplasms	GXYLT2	0.000255	-10.359103	
15597	Malignant Neoplasms	NTRK2	0.000382	-10.306842	
15598	Malignant Neoplasms	NEAT1	0	-10.285	

15599	Malignant Neoplasms	DTNB	0.000445	-10.209464	
15600	Malignant Neoplasms	DDX47	0	-10.194378	
15601	Malignant Neoplasms	PPP1R9A	0.000004	-10.144455	
15602	Malignant Neoplasms	CA4	0.000001	-10.09118	
15603	Malignant Neoplasms	GGA1	0	-10.072485	
15604	Malignant Neoplasms	DQX1	0.004044	-10.025782	
15605	Malignant Neoplasms	ZEB1	0.00085	-9.880126	
15606	Malignant Neoplasms	KDM6B	0	-9.832849	
15607	Malignant Neoplasms	FLJ45445	0	-9.799125	
15608	Malignant Neoplasms	CREB3L1	0.003101	-9.594399	
15609	Malignant Neoplasms	RAB27B	0.000026	-9.499798	
15610	Malignant Neoplasms	FHOD3	0.00026	-9.498485	
15611	Malignant Neoplasms	USP42	0	-9.486286	
15612	Malignant Neoplasms	CACNB2	0.000114	-9.483538	
15613	Malignant Neoplasms	TMSB15B	0.00058	-9.46368	
15614	Malignant Neoplasms	WNT6	0.000244	-9.436647	
15615	Malignant Neoplasms	ASAP3	0.002559	-9.13715	
15616	Malignant Neoplasms	ZBTB7C	0.00056	-9.045275	
15617	Malignant Neoplasms	RBM20	0.000241	-8.976458	
15618	Malignant Neoplasms	FOXA1	0.003339	-8.939768	
15619	Malignant Neoplasms	ALDH1A1	0.003273	-8.905504	
15620	Malignant Neoplasms	P2RX5	0.000466	-8.892187	
15621	Malignant Neoplasms	4-Sep	0.000152	-8.870024	
15622	Malignant Neoplasms	FABP1	0	-8.719388	
15623	Malignant Neoplasms	SYT17	0.000365	-8.713691	
15624	Malignant Neoplasms	NOD2	0.000327	-8.711971	
15625	Malignant Neoplasms	KCNK10	0.000558	-8.692129	
15626	Malignant Neoplasms	LOC100129069	0.001754	-8.641304	
15627	Malignant Neoplasms	HMGCS2	0.000405	-8.54771	
15628	Malignant Neoplasms	BIK	0.000004	-8.498466	
15629	Malignant Neoplasms	HACE1	0.000417	-8.496176	
15630	Malignant Neoplasms	FKBP1B	0.000286	-8.458062	
15631	Malignant Neoplasms	C6orf168	0.000278	-8.391398	
15632	Malignant Neoplasms	SHROOM2	0.000326	-8.375547	
15633	Malignant Neoplasms	HGD	0.001124	-8.34414	
15634	Malignant Neoplasms	NCRNA00173	0.000861	-8.343959	
15635	Malignant Neoplasms	FLJ32063	0.00281	-8.296896	
15636	Malignant Neoplasms	KIAA1211	0.000154	-8.170336	
15637	Malignant Neoplasms	WWTR1	0.000448	-8.142748	
15638	Malignant Neoplasms	ELF2	0	-8.053555	
15639	Malignant Neoplasms	CLCA4	0.000002	-8.038073	
15640	Malignant Neoplasms	LYN	0.000005	-8.015746	
15641	Malignant Neoplasms	OSBPL6	0.000392	-7.987632	
15642	Malignant Neoplasms	CEACAM7	0.000001	-7.835955	
15643	Malignant Neoplasms	NR3C2	0.000114	-7.832787	
15644	Malignant Neoplasms	DNHD1	0.00009	-7.832598	
15645	Malignant Neoplasms	ITM2A	0.004857	-7.7829	
15646	Malignant Neoplasms	TPBG	0.005167	-7.771458	
15647	Malignant Neoplasms	FABP3	0.000339	-7.759696	
15648	Malignant Neoplasms	NFE2	0.000244	-7.710593	

15649	Malignant Neoplasms	IQCG	0.000154	-7.705877	
15650	Malignant Neoplasms	LOC100130357	0.000038	-7.663775	
15651	Malignant Neoplasms	BLVRA	0.000647	-7.631662	
15652	Malignant Neoplasms	KLF4	0.003466	-7.611532	
15653	Malignant Neoplasms	RYBP	0	-7.548279	
15654	Malignant Neoplasms	GLIS3	0.000782	-7.497044	
15655	Malignant Neoplasms	ERN2	0.000972	-7.486607	
15656	Malignant Neoplasms	TSPAN2	0.000263	-7.480316	
15657	Malignant Neoplasms	MOGAT2	0.000358	-7.460037	
15658	Malignant Neoplasms	PHF13	0	-7.426994	
15659	Malignant Neoplasms	ZNF420	0.000647	-7.423682	
15660	Malignant Neoplasms	CCDC104	0	-7.356899	
15661	Malignant Neoplasms	B3GALT5	0.000358	-7.341713	
15662	Malignant Neoplasms	OR51I1	0.004706	-7.291565	
15663	Malignant Neoplasms	MT1F	0.000538	-7.277401	
15664	Malignant Neoplasms	PLS3	0.00009	-7.214046	
15665	Malignant Neoplasms	DOCK8	0.004993	-7.212796	
15666	Malignant Neoplasms	LAMA1	0.002619	-7.153433	
15667	Malignant Neoplasms	SLC16A2	0.000647	-7.125774	
15668	Malignant Neoplasms	C14orf50	0.000358	-7.032244	
15669	Malignant Neoplasms	CPSF2	0.000061	-6.975072	
15670	Malignant Neoplasms	GRAMD3	0.000488	-6.964465	
15671	Malignant Neoplasms	FLJ21511	0	-6.869291	
15672	Malignant Neoplasms	TCEAL2	0.000006	-6.859401	
15673	Malignant Neoplasms	HP1BP3	0	-6.799139	
15674	Malignant Neoplasms	CCNJL	0.000163	-6.772087	
15675	Malignant Neoplasms	C11orf93	0.001565	-6.711384	
15676	Malignant Neoplasms	SPATA18	0.003433	-6.664482	
15677	Malignant Neoplasms	TAF15	0	-6.66311	
15678	Malignant Neoplasms	FGF20	0.000252	-6.654826	
15679	Malignant Neoplasms	LHX6	0.000273	-6.644605	
15680	Malignant Neoplasms	FARSB	0.00277	-6.56314	
15681	Malignant Neoplasms	GBA3	0	-6.560875	
15682	Malignant Neoplasms	DPF3	0.000202	-6.507991	
15683	Malignant Neoplasms	BCL2L15	0.000428	-6.502587	
15684	Malignant Neoplasms	C7	0	-6.423415	
15685	Malignant Neoplasms	SIX1	0.000433	-6.42248	
15686	Malignant Neoplasms	MDFIC	0.000923	-6.391231	
15687	Malignant Neoplasms	PLCL2	0.003101	-6.299826	
15688	Malignant Neoplasms	RAB39B	0.000179	-6.222458	
15689	Malignant Neoplasms	ZFP14	0.000424	-6.185132	
15690	Malignant Neoplasms	MT1G	0.000373	-6.174671	
15691	Malignant Neoplasms	ITPR1	0.00003	-6.173306	
15692	Malignant Neoplasms	NR3C1	0.000517	-6.067312	
15693	Malignant Neoplasms	CBFA2T3	0.000405	-6.06337	
15694	Malignant Neoplasms	CXCL12	0	-6.025157	
15695	Malignant Neoplasms	HPGDS	0.00138	-5.994216	
15696	Malignant Neoplasms	GPR155	0.000239	-5.97031	
15697	Malignant Neoplasms	RABL4	0.000209	-5.953176	
15698	Malignant Neoplasms	FAM43A	0.000491	-5.93246	

15699	Malignant Neoplasms	PSMG4	0.00332	-5.931413	
15700	Malignant Neoplasms	OSBPL3	0.000112	-5.89578	
15701	Malignant Neoplasms	EPHA4	0.000134	-5.882295	
15702	Malignant Neoplasms	SLC9A2	0.004579	-5.796233	
15703	Malignant Neoplasms	TPH1	0	-5.793849	
15704	Malignant Neoplasms	FMNL2	0.000204	-5.762469	
15705	Malignant Neoplasms	JAG1	0.000087	-5.705999	
15706	Malignant Neoplasms	FRMD3	0.004638	-5.691552	
15707	Malignant Neoplasms	ABCC5	0.000451	-5.681788	
15708	Malignant Neoplasms	NT5M	0.000521	-5.666996	
15709	Malignant Neoplasms	SLC16A7	0.003386	-5.613971	
15710	Malignant Neoplasms	FAM69A	0.000268	-5.60504	
15711	Malignant Neoplasms	ACTG2	0	-5.576115	
15712	Malignant Neoplasms	AQP8	0.000157	-5.570023	
15713	Malignant Neoplasms	ITPRIPL1	0.00058	-5.532122	
15714	Malignant Neoplasms	CHP2	0.004857	-5.506835	
15715	Malignant Neoplasms	ANKRD42	0.000082	-5.496973	
15716	Malignant Neoplasms	HDDC2	0.000414	-5.463333	
15717	Malignant Neoplasms	PRKAA2	0.000462	-5.444732	
15718	Malignant Neoplasms	KLF7	0.000649	-5.373335	
15719	Malignant Neoplasms	MS4A12	0.000032	-5.360331	
15720	Malignant Neoplasms	CYP4V2	0.000035	-5.342829	
15721	Malignant Neoplasms	ODZ2	0.000531	-5.32371	
15722	Malignant Neoplasms	LOC643072	0.003988	-5.284507	
15723	Malignant Neoplasms	C20orf194	0.000391	-5.282965	
15724	Malignant Neoplasms	XLKD1	0	-5.253411	
15725	Malignant Neoplasms	RNF125	0.003737	-5.236229	
15726	Malignant Neoplasms	SIDT1	0.000358	-5.216442	
15727	Malignant Neoplasms	ITGA7	0.000392	-5.212705	
15728	Malignant Neoplasms	MATN2	0.000471	-5.191578	
15729	Malignant Neoplasms	TIAM1	0.000244	-5.166496	
15730	Malignant Neoplasms	MCTP2	0.000114	-5.145834	
15731	Malignant Neoplasms	CSDE1	0	-5.143108	
15732	Malignant Neoplasms	IGLL3	0.000497	-5.135458	
15733	Malignant Neoplasms	ABCA8	0	-5.130991	
15734	Malignant Neoplasms	RTDR1	0.000423	-5.050415	
15735	Malignant Neoplasms	VSTM2A	0.002883	-5.04434	
15736	Malignant Neoplasms	NR5A2	0.00211	-5.041885	
15737	Malignant Neoplasms	ACOX3	0.000135	-5.000085	
15738	Malignant Neoplasms	TNFRSF11A	0.000529	-4.980546	
15739	Malignant Neoplasms	EFHC2	0.000215	-4.968653	
15740	Malignant Neoplasms	STX6	0.000154	-4.962607	
15741	Malignant Neoplasms	MSI2	0.000015	-4.960336	
15742	Malignant Neoplasms	VILL	0.000651	-4.956432	
15743	Malignant Neoplasms	GOLM1	0.000215	-4.926988	
15744	Malignant Neoplasms	GBE1	0.000373	-4.92179	
15745	Malignant Neoplasms	DENND1B	0.002619	-4.908923	
15746	Malignant Neoplasms	CFL2	0.00073	-4.877337	
15747	Malignant Neoplasms	C2orf88	0.001516	-4.861751	
15748	Malignant Neoplasms	KIAA1731	0.004141	-4.835946	

15749	Malignant Neoplasms	DEGS2	0.004217	-4.794887	
15750	Malignant Neoplasms	KSR2	0.004857	-4.793552	
15751	Malignant Neoplasms	NIN	0.000024	-4.790656	
15752	Malignant Neoplasms	TRPM6	0.000711	-4.765605	
15753	Malignant Neoplasms	PTPRN2	0.000425	-4.763522	
15754	Malignant Neoplasms	MAPK10	0.010117	-4.758095	
15755	Malignant Neoplasms	TEX9	0.003737	-4.753316	
15756	Malignant Neoplasms	BBS9	0.000093	-4.738447	
15757	Malignant Neoplasms	TEP1	0.00237	-4.711473	
15758	Malignant Neoplasms	SLC4A4	0	-4.708871	
15759	Malignant Neoplasms	PRODH	0.000213	-4.705215	
15760	Malignant Neoplasms	KLK1	0.00332	-4.696096	
15761	Malignant Neoplasms	BEX4	0.000647	-4.68933	
15762	Malignant Neoplasms	LCK	0.000158	-4.674262	
15763	Malignant Neoplasms	LOC100506589	0.001854	-4.673174	
15764	Malignant Neoplasms	ZBTB47	0.000521	-4.669141	
15765	Malignant Neoplasms	CCL28	0.000605	-4.654903	
15766	Malignant Neoplasms	C16orf53	0.003149	-4.649649	
15767	Malignant Neoplasms	OXGR1	0.00054	-4.647567	
15768	Malignant Neoplasms	CDK15	0.000637	-4.621657	
15769	Malignant Neoplasms	LOC650794	0.000112	-4.604266	
15770	Malignant Neoplasms	MYL9	0.000267	-4.572219	
15771	Malignant Neoplasms	GNE	0.004141	-4.563829	
15772	Malignant Neoplasms	CRTAP	0.000102	-4.533045	
15773	Malignant Neoplasms	RBP7	0.000326	-4.530603	
15774	Malignant Neoplasms	SLC2A4	0.000278	-4.512144	
15775	Malignant Neoplasms	PDLIM3	0.000847	-4.486607	
15776	Malignant Neoplasms	MCOLN2	0.000103	-4.485904	
15777	Malignant Neoplasms	TOX	0.001493	-4.475666	
15778	Malignant Neoplasms	NRP2	0.000636	-4.46815	
15779	Malignant Neoplasms	PARVA	0.002922	-4.438447	
15780	Malignant Neoplasms	TFCP2L1	0.000047	-4.434301	
15781	Malignant Neoplasms	AFAP1L1	0.000168	-4.429256	
15782	Malignant Neoplasms	SSTR1	0.003988	-4.42735	
15783	Malignant Neoplasms	SIAE	0.001854	-4.411799	
15784	Malignant Neoplasms	OR4N4	0.000204	-4.395892	
15785	Malignant Neoplasms	RAB27A	0.00002	-4.39503	
15786	Malignant Neoplasms	ARMCX4	0.003737	-4.391212	
15787	Malignant Neoplasms	LOC727916	0.005164	-4.380807	
15788	Malignant Neoplasms	FAS	0.002883	-4.344731	
15789	Malignant Neoplasms	NRG2	0.000392	-4.324364	
15790	Malignant Neoplasms	CGGBP1	0.000061	-4.315759	
15791	Malignant Neoplasms	LXN	0.001245	-4.295182	
15792	Malignant Neoplasms	SLC26A2	0	-4.277242	
15793	Malignant Neoplasms	LOC728448	0.000234	-4.251742	
15794	Malignant Neoplasms	LOC100128288	0.004857	-4.240265	
15795	Malignant Neoplasms	FYB	0.003433	-4.225236	
15796	Malignant Neoplasms	DDX26B	0.004644	-4.223636	
15797	Malignant Neoplasms	JAZF1	0.000057	-4.218977	
15798	Malignant Neoplasms	ANG	0.000488	-4.192727	

15799	Malignant Neoplasms	LOC100216546	0.000776	-4.176673	
15800	Malignant Neoplasms	EBF1	0.004544	-4.164009	
15801	Malignant Neoplasms	TEX11	0.001604	-4.140414	
15802	Malignant Neoplasms	TBXAS1	0.000237	-4.081353	
15803	Malignant Neoplasms	MAPRE2	0.000193	-4.080819	
15804	Malignant Neoplasms	STYK1	0.000446	-4.069078	
15805	Malignant Neoplasms	TCEA3	0.003014	-4.058089	
15806	Malignant Neoplasms	CXCL14	0.000133	-4.05581	
15807	Malignant Neoplasms	LOC728903	0.001325	-4.04142	
15808	Malignant Neoplasms	CLEC3B	0	-4.038001	
15809	Malignant Neoplasms	PAFAH2	0.001196	-4.022292	
15810	Malignant Neoplasms	SAT1	0.000035	-4.017131	
15811	Malignant Neoplasms	NMNAT3	0.000756	-4.014329	
15812	Malignant Neoplasms	KCTD1	0.00296	-4.01414	
15813	Malignant Neoplasms	GPR27	0.004668	-4.013727	
15814	Malignant Neoplasms	QSOX1	0.003047	-3.986606	
15815	Malignant Neoplasms	EFNB3	0.00058	-3.969069	
15816	Malignant Neoplasms	UBQLN4	0.004931	-3.93261	
15817	Malignant Neoplasms	SH3BGR	0.000209	-3.931459	
15818	Malignant Neoplasms	RUNX2	0.000095	-3.909275	
15819	Malignant Neoplasms	EHF	0.001404	-3.901229	
15820	Malignant Neoplasms	CARD16	0.003031	-3.878799	
15821	Malignant Neoplasms	CAPN13	0.002883	-3.8648	
15822	Malignant Neoplasms	USP13	0.000208	-3.858849	
15823	Malignant Neoplasms	TTLL5	0.004629	-3.848962	
15824	Malignant Neoplasms	XPO7	0.000273	-3.848652	
15825	Malignant Neoplasms	DST	0	-3.84509	
15826	Malignant Neoplasms	C4orf34	0.000493	-3.841658	
15827	Malignant Neoplasms	AKAP2	0.000749	-3.840142	
15828	Malignant Neoplasms	MTERFD2	0.000193	-3.831917	
15829	Malignant Neoplasms	BCAR3	0.001754	-3.82109	
15830	Malignant Neoplasms	C1orf21	0.000694	-3.812153	
15831	Malignant Neoplasms	SYTL2	0.001871	-3.807608	
15832	Malignant Neoplasms	LOC389160	0	-3.801994	
15833	Malignant Neoplasms	LOC100134119	0.000393	-3.788561	
15834	Malignant Neoplasms	HLA-DQB1	0.00013	-3.786985	
15835	Malignant Neoplasms	ACVRL1	0.002015	-3.786947	
15836	Malignant Neoplasms	PHF7	0.004347	-3.782336	
15837	Malignant Neoplasms	ANGPTL1	0	-3.78057	
15838	Malignant Neoplasms	PRPS2	0.000025	-3.77657	
15839	Malignant Neoplasms	CCDC68	0.002058	-3.775933	
15840	Malignant Neoplasms	LOC442270	0.000058	-3.748188	
15841	Malignant Neoplasms	ADH6	0.002922	-3.740311	
15842	Malignant Neoplasms	HNRNPD	0.004611	-3.731405	
15843	Malignant Neoplasms	ASPA	0	-3.729354	
15844	Malignant Neoplasms	ACOT9	0.000039	-3.725177	
15845	Malignant Neoplasms	RAB26	0.00113	-3.719949	
15846	Malignant Neoplasms	SMA4	0.000392	-3.705228	
15847	Malignant Neoplasms	SPECC1	0.004848	-3.698745	
15848	Malignant Neoplasms	SFRS18	0.001805	-3.6981	

15849	Malignant Neoplasms	CCDC88A	0.00058	-3.694598	
15850	Malignant Neoplasms	CDC42SE2	0.000392	-3.667925	
15851	Malignant Neoplasms	CLMN	0.000358	-3.643807	
15852	Malignant Neoplasms	FAM149A	0.004517	-3.641557	
15853	Malignant Neoplasms	MAOA	0.00219	-3.624132	
15854	Malignant Neoplasms	C14orf106	0.004644	-3.614466	
15855	Malignant Neoplasms	FBLN1	0.000002	-3.606652	
15856	Malignant Neoplasms	TNFRSF19	0.000449	-3.604152	
15857	Malignant Neoplasms	VPS26A	0.002559	-3.599722	
15858	Malignant Neoplasms	TRAPPC10	0.00293	-3.583657	
15859	Malignant Neoplasms	ADAMTS8	0.000466	-3.554192	
15860	Malignant Neoplasms	TPSG1	0	-3.549494	
15861	Malignant Neoplasms	DMXL2	0.002619	-3.539085	
15862	Malignant Neoplasms	LOC157562	0.000135	-3.527667	
15863	Malignant Neoplasms	ARMC8	0.00345	-3.52509	
15864	Malignant Neoplasms	C17orf51	0.000392	-3.520076	
15865	Malignant Neoplasms	MYC	0.0005	-3.515492	
15866	Malignant Neoplasms	FLJ27352	0.001463	-3.511211	
15867	Malignant Neoplasms	REG4	0.000059	-3.503367	
15868	Malignant Neoplasms	C1orf226	0.000353	-3.482497	
15869	Malignant Neoplasms	PTPN21	0.001057	-3.479038	
15870	Malignant Neoplasms	SAMD13	0.000333	-3.468445	
15871	Malignant Neoplasms	FRMD4B	0.0037	-3.467352	
15872	Malignant Neoplasms	RGL1	0.000801	-3.444498	
15873	Malignant Neoplasms	MIA	0.000886	-3.441869	
15874	Malignant Neoplasms	EXDL2	0.000144	-3.428238	
15875	Malignant Neoplasms	LOH12CR1	0.0001	-3.425623	
15876	Malignant Neoplasms	NCRNA00081	0.000927	-3.424376	
15877	Malignant Neoplasms	COBL	0.000144	-3.413096	
15878	Malignant Neoplasms	AKAP10	0.003047	-3.41148	
15879	Malignant Neoplasms	LIMA1	0.003047	-3.393403	
15880	Malignant Neoplasms	EHHADH	0.004243	-3.390529	
15881	Malignant Neoplasms	UGT1A8	0	-3.380884	
15882	Malignant Neoplasms	FLJ23867	0.000215	-3.375153	
15883	Malignant Neoplasms	PGAP1	0.001395	-3.368329	
15884	Malignant Neoplasms	RAP1GAP	0.004544	-3.366309	
15885	Malignant Neoplasms	RNFT1	0.000382	-3.361638	
15886	Malignant Neoplasms	GPR143	0.000254	-3.356835	
15887	Malignant Neoplasms	KIAA1324	0.00219	-3.346923	
15888	Malignant Neoplasms	MGC21881	0.004483	-3.345753	
15889	Malignant Neoplasms	AHI1	0.000064	-3.345557	
15890	Malignant Neoplasms	CDH13	0.000647	-3.335462	
15891	Malignant Neoplasms	MGLL	0	-3.330854	
15892	Malignant Neoplasms	SBF2	0.000987	-3.325026	
15893	Malignant Neoplasms	ENOSF1	0.000324	-3.307115	
15894	Malignant Neoplasms	ATP8B1	0.001005	-3.299743	
15895	Malignant Neoplasms	NAB1	0.001404	-3.298022	
15896	Malignant Neoplasms	TTRAP	0	-3.297063	
15897	Malignant Neoplasms	CAPN5	0.00049	-3.289094	
15898	Malignant Neoplasms	SLC44A1	0.000488	-3.283266	

15899	Malignant Neoplasms	PRORSD1P	0.000953	-3.274898	
15900	Malignant Neoplasms	SLC22A23	0.000488	-3.272734	
15901	Malignant Neoplasms	LOC147645	0.000007	-3.257132	
15902	Malignant Neoplasms	ZNF649	0.000432	-3.256486	
15903	Malignant Neoplasms	LOC100289632	0.002906	-3.25408	
15904	Malignant Neoplasms	CHD9	0.000795	-3.253819	
15905	Malignant Neoplasms	INTS10	0.000126	-3.245618	
15906	Malignant Neoplasms	SLC35A1	0.000136	-3.233704	
15907	Malignant Neoplasms	ZCCHC7	0.000295	-3.231801	
15908	Malignant Neoplasms	MPP6	0.000836	-3.214355	
15909	Malignant Neoplasms	SIPA1L2	0.004857	-3.210274	
15910	Malignant Neoplasms	KIAA1370	0.002922	-3.207262	
15911	Malignant Neoplasms	GNAQ	0.001906	-3.202496	
15912	Malignant Neoplasms	LMLN	0.000596	-3.201008	
15913	Malignant Neoplasms	ZNF557	0.000119	-3.195714	
15914	Malignant Neoplasms	SCARA3	0.000667	-3.187921	
15915	Malignant Neoplasms	RBKS	0.000067	-3.187384	
15916	Malignant Neoplasms	PLEKHH1	0.001405	-3.180597	
15917	Malignant Neoplasms	KIF13A	0.000802	-3.177314	
15918	Malignant Neoplasms	ALDH6A1	0.002957	-3.169574	
15919	Malignant Neoplasms	FBXW2	0.000316	-3.154812	
15920	Malignant Neoplasms	KLHL15	0.000521	-3.15467	
15921	Malignant Neoplasms	PPP1R12B	0.003424	-3.145353	
15922	Malignant Neoplasms	LEAP2	0.000488	-3.125762	
15923	Malignant Neoplasms	ADH1B	0	-3.123674	
15924	Malignant Neoplasms	MEIS3P1	0.002861	-3.102961	
15925	Malignant Neoplasms	TET2	0.00293	-3.096987	
15926	Malignant Neoplasms	SPARCL1	0.000004	-3.096174	
15927	Malignant Neoplasms	CMAS	0.000711	-3.085735	
15928	Malignant Neoplasms	TFF3	0.004638	-3.069182	
15929	Malignant Neoplasms	SELENBP1	0.000071	-3.066158	
15930	Malignant Neoplasms	C2orf72	0.002451	-3.066078	
15931	Malignant Neoplasms	LOC440396	0.000355	-3.064023	
15932	Malignant Neoplasms	C4orf19	0.001296	-3.063415	
15933	Malignant Neoplasms	SH2B2	0.000475	-3.059526	
15934	Malignant Neoplasms	PCSK5	0.000459	-3.056948	
15935	Malignant Neoplasms	LOC644538	0.000716	-3.055062	
15936	Malignant Neoplasms	SLC48A1	0.00029	-3.040614	
15937	Malignant Neoplasms	RECK	0.00073	-3.030721	
15938	Malignant Neoplasms	SPATA7	0.000389	-3.028222	
15939	Malignant Neoplasms	DARC	0.000001	-3.028116	
15940	Malignant Neoplasms	VIP	0.000001	-3.019982	
15941	Malignant Neoplasms	SR140	0.003805	-3.019369	
15942	Malignant Neoplasms	SCAPER	0.004247	-3.017734	
15943	Malignant Neoplasms	RNU12	0.000261	-3.00526	
15944	Malignant Neoplasms	GSTM2	0	-3.003643	
15945	Malignant Neoplasms	AKR1B10	0.000698	-3.002267	
15946	Malignant Neoplasms	PIGH	0.000162	-2.993498	
15947	Malignant Neoplasms	LOC400573	0.001278	-2.984874	
15948	Malignant Neoplasms	ACACB	0.004517	-2.983934	

15949	Malignant Neoplasms	ARL14	0	-2.982007	
15950	Malignant Neoplasms	DTWD1	0.004638	-2.969544	
15951	Malignant Neoplasms	C3orf38	0.00036	-2.968622	
15952	Malignant Neoplasms	NRIP3	0.000409	-2.958991	
15953	Malignant Neoplasms	SPTLC3	0	-2.945247	
15954	Malignant Neoplasms	MON2	0.000711	-2.938999	
15955	Malignant Neoplasms	FAM48A	0.002854	-2.936586	
15956	Malignant Neoplasms	DOCK11	0.000595	-2.924051	
15957	Malignant Neoplasms	APOO	0.000091	-2.909463	
15958	Malignant Neoplasms	VMD2L1	0.000134	-2.885943	
15959	Malignant Neoplasms	C17orf45	0.000282	-2.883876	
15960	Malignant Neoplasms	CEP70	0.001068	-2.88124	
15961	Malignant Neoplasms	CWF19L2	0.003589	-2.85482	
15962	Malignant Neoplasms	FGD4	0.000358	-2.846781	
15963	Malignant Neoplasms	MID1IP1	0.00003	-2.824787	
15964	Malignant Neoplasms	RBM25	0.000802	-2.822374	
15965	Malignant Neoplasms	HSBP1L1	0.000488	-2.82191	
15966	Malignant Neoplasms	ZNF503	0.000874	-2.82166	
15967	Malignant Neoplasms	ABCC3	0.002833	-2.821428	
15968	Malignant Neoplasms	PPP2R3A	0.003288	-2.821136	
15969	Malignant Neoplasms	GPA33	0.000665	-2.816748	
15970	Malignant Neoplasms	RNASE4	0.000488	-2.815054	
15971	Malignant Neoplasms	RMND1	0.000507	-2.81366	
15972	Malignant Neoplasms	PLCD1	0.004517	-2.809502	
15973	Malignant Neoplasms	SULT1A3	0.003634	-2.807614	
15974	Malignant Neoplasms	VWF	0.000708	-2.797215	
15975	Malignant Neoplasms	SAHH3	0.000006	-2.796622	
15976	Malignant Neoplasms	ZNF18	0.000381	-2.794412	
15977	Malignant Neoplasms	LYST	0.000207	-2.788893	
15978	Malignant Neoplasms	LOC100128893	0.004141	-2.787412	
15979	Malignant Neoplasms	RPGR	0.000351	-2.783919	
15980	Malignant Neoplasms	CPEB3	0.005131	-2.761783	
15981	Malignant Neoplasms	C14orf100	0.000237	-2.7582	
15982	Malignant Neoplasms	EXPH5	0.000446	-2.756191	
15983	Malignant Neoplasms	LOC100506168	0.003101	-2.737855	
15984	Malignant Neoplasms	TNFRSF10D	0.00281	-2.734711	
15985	Malignant Neoplasms	GK	0.000255	-2.719562	
15986	Malignant Neoplasms	MRPL34	0.000218	-2.717989	
15987	Malignant Neoplasms	HSD11B2	0.000411	-2.707869	
15988	Malignant Neoplasms	SGSM3	0.002619	-2.702811	
15989	Malignant Neoplasms	LOC100506493	0.005131	-2.692526	
15990	Malignant Neoplasms	NHSL1	0.002223	-2.69232	
15991	Malignant Neoplasms	ATP8A1	0	-2.689517	
15992	Malignant Neoplasms	SMCR7L	0.0001	-2.68474	
15993	Malignant Neoplasms	PCLO	0.003424	-2.682969	
15994	Malignant Neoplasms	ZNF253	0.000647	-2.681512	
15995	Malignant Neoplasms	LOC342918	0.031445	-2.676083	
15996	Malignant Neoplasms	NEDD4L	0.00332	-2.675522	
15997	Malignant Neoplasms	TRUB1	0.016286	-2.67491	
15998	Malignant Neoplasms	DIRC1	0.000377	-2.673842	

15999	Malignant Neoplasms	KRT74	0.000317	-2.670621	
16000	Malignant Neoplasms	TPT1	0.004857	-2.663384	
16001	Malignant Neoplasms	FARP2	0.000187	-2.66268	
16002	Malignant Neoplasms	PAIP2B	0.000487	-2.655846	
16003	Malignant Neoplasms	TMTC4	0.000482	-2.654245	
16004	Malignant Neoplasms	GPATCH2	0.003649	-2.652061	
16005	Malignant Neoplasms	SPON1	0.000432	-2.646048	
16006	Malignant Neoplasms	HIST1H4B	0.000509	-2.626881	
16007	Malignant Neoplasms	PRDX4	0.000167	-2.617971	
16008	Malignant Neoplasms	MED21	0.000088	-2.616987	
16009	Malignant Neoplasms	CCL13	0.000261	-2.615183	
16010	Malignant Neoplasms	C10orf54	0.003149	-2.612426	
16011	Malignant Neoplasms	CPA3	0	-2.60624	
16012	Malignant Neoplasms	NAPEPLD	0.001781	-2.604727	
16013	Malignant Neoplasms	SMCHD1	0.003343	-2.585213	
16014	Malignant Neoplasms	CCDC84	0.000425	-2.584065	
16015	Malignant Neoplasms	ZNF409	0.000143	-2.582499	
16016	Malignant Neoplasms	SRSF1	0.000679	-2.574245	
16017	Malignant Neoplasms	6-Sep	0.000085	-2.570746	
16018	Malignant Neoplasms	PTTG1IP	0.000102	-2.565068	
16019	Malignant Neoplasms	TDP1	0.000402	-2.556651	
16020	Malignant Neoplasms	LIN52	0.000061	-2.555914	
16021	Malignant Neoplasms	EPB41L4B	0.000062	-2.549991	
16022	Malignant Neoplasms	MGC13057	0	-2.547698	
16023	Malignant Neoplasms	LOC344967	0.000492	-2.544458	
16024	Malignant Neoplasms	C21orf70	0.000584	-2.536377	
16025	Malignant Neoplasms	HSD17B11	0.0001	-2.534032	
16026	Malignant Neoplasms	PIGN	0.001057	-2.524512	
16027	Malignant Neoplasms	PALLD	0.000893	-2.519625	
16028	Malignant Neoplasms	PROSC	0.000647	-2.51818	
16029	Malignant Neoplasms	CCDC112	0.001371	-2.515317	
16030	Malignant Neoplasms	ZSWIM6	0.003636	-2.505999	
16031	Malignant Neoplasms	LOC727995	0	-2.505991	
16032	Malignant Neoplasms	CASD1	0.004245	-2.501637	
16033	Malignant Neoplasms	ACOT7	0.000402	-2.498078	
16034	Malignant Neoplasms	SDCBP2	0.000331	-2.495947	
16035	Malignant Neoplasms	FUBP1	0.000607	-2.495646	
16036	Malignant Neoplasms	RNASEL	0.004644	-2.494672	
16037	Malignant Neoplasms	C12orf57	0.000424	-2.492894	
16038	Malignant Neoplasms	MYOF	0.003778	-2.488286	
16039	Malignant Neoplasms	PLD1	0.0037	-2.482875	
16040	Malignant Neoplasms	AMY2B	0.004544	-2.481498	
16041	Malignant Neoplasms	C6orf35	0.000428	-2.480078	
16042	Malignant Neoplasms	PDE9A	0	-2.477127	
16043	Malignant Neoplasms	TMEM30B	0.000555	-2.470234	
16044	Malignant Neoplasms	SLCO2A1	0.000002	-2.469144	
16045	Malignant Neoplasms	CAMK2D	0.000541	-2.468768	
16046	Malignant Neoplasms	LYRM5	0.000254	-2.45771	
16047	Malignant Neoplasms	COL6A3	0.000433	-2.45215	
16048	Malignant Neoplasms	AP4B1	0.000582	-2.447063	

16049	Malignant Neoplasms	ASTN2	0.000389	-2.440821	
16050	Malignant Neoplasms	EIF3L	0.000268	-2.438209	
16051	Malignant Neoplasms	MEGF9	0.000161	-2.437944	
16052	Malignant Neoplasms	FOXF1	0	-2.435512	
16053	Malignant Neoplasms	CD74	0.037564	-2.433393	
16054	Malignant Neoplasms	LOC643220	0.000286	-2.425949	
16055	Malignant Neoplasms	UGP2	0	-2.425445	
16056	Malignant Neoplasms	MYO1C	0.003288	-2.416458	
16057	Malignant Neoplasms	PDSS2	0.00044	-2.412645	
16058	Malignant Neoplasms	RBBP4	0.004235	-2.412335	
16059	Malignant Neoplasms	NGDN	0.000014	-2.40989	
16060	Malignant Neoplasms	MRPS22	0.000675	-2.404713	
16061	Malignant Neoplasms	VDAC1	0.00036	-2.403048	
16062	Malignant Neoplasms	GLTP	0.00293	-2.401717	
16063	Malignant Neoplasms	STXBP1	0.000521	-2.400944	
16064	Malignant Neoplasms	MR1	0.000082	-2.400894	
16065	Malignant Neoplasms	CYB5D1	0.000471	-2.396127	
16066	Malignant Neoplasms	CRYZL1	0.004152	-2.394317	
16067	Malignant Neoplasms	TLE3	0.000159	-2.393145	
16068	Malignant Neoplasms	SYTL4	0.001604	-2.391109	
16069	Malignant Neoplasms	EIF4B	0.00033	-2.387684	
16070	Malignant Neoplasms	TNRC6B	0.000456	-2.383205	
16071	Malignant Neoplasms	ASAHL	0	-2.379442	
16072	Malignant Neoplasms	KIAA1109	0.003038	-2.378572	
16073	Malignant Neoplasms	LOC253264	0.000346	-2.373921	
16074	Malignant Neoplasms	COPZ1	0.000413	-2.373177	
16075	Malignant Neoplasms	PGM5	0.000314	-2.364494	
16076	Malignant Neoplasms	C14orf126	0.000242	-2.350964	
16077	Malignant Neoplasms	BAP1	0.000656	-2.348392	
16078	Malignant Neoplasms	WDR7	0.005167	-2.346086	
16079	Malignant Neoplasms	DENND1A	0.000355	-2.342864	
16080	Malignant Neoplasms	DENND5A	0.000471	-2.338272	
16081	Malignant Neoplasms	ATG4A	0.000394	-2.333514	
16082	Malignant Neoplasms	SCARA5	0.000025	-2.332101	
16083	Malignant Neoplasms	PAPSS2	0.004629	-2.332038	
16084	Malignant Neoplasms	FAM162A	0.00025	-2.33122	
16085	Malignant Neoplasms	CCDC14	0.00051	-2.328058	
16086	Malignant Neoplasms	MAFB	0.00033	-2.314435	
16087	Malignant Neoplasms	RABGAP1L	0.003737	-2.314043	
16088	Malignant Neoplasms	ZNF318	0.000124	-2.312317	
16089	Malignant Neoplasms	PRKAR2A	0.000647	-2.305946	
16090	Malignant Neoplasms	KLRG2	0.000491	-2.281182	
16091	Malignant Neoplasms	ACO2	0.000234	-2.277367	
16092	Malignant Neoplasms	PPP2R1B	0.000649	-2.273801	
16093	Malignant Neoplasms	C15orf48	0.000105	-2.264361	
16094	Malignant Neoplasms	EFCAB4B	0.004437	-2.264241	
16095	Malignant Neoplasms	TSPAN13	0.002922	-2.261416	
16096	Malignant Neoplasms	AGXT2L2	0.00293	-2.258722	
16097	Malignant Neoplasms	LOC100190939	0.000113	-2.258036	
16098	Malignant Neoplasms	CDS2	0.000258	-2.256708	

16099	Malignant Neoplasms	LOC120364	0.000424	-2.256464	
16100	Malignant Neoplasms	IMPDH2	0.000492	-2.256004	
16101	Malignant Neoplasms	MAB21L2	0.000009	-2.254028	
16102	Malignant Neoplasms	RABEP1	0.000647	-2.25188	
16103	Malignant Neoplasms	NKTR	0.002223	-2.246367	
16104	Malignant Neoplasms	MS4A8B	0.000135	-2.245197	
16105	Malignant Neoplasms	EYA3	0.046824	-2.232375	
16106	Malignant Neoplasms	FGL2	0	-2.227979	
16107	Malignant Neoplasms	CMC1	0.000309	-2.22477	
16108	Malignant Neoplasms	ARID1B	0.000099	-2.221163	
16109	Malignant Neoplasms	IKBKE	0.000573	-2.219915	
16110	Malignant Neoplasms	ZFYVE1	0.000278	-2.219254	
16111	Malignant Neoplasms	RGMA	0.000001	-2.216106	
16112	Malignant Neoplasms	FAM161B	0.00488	-2.207343	
16113	Malignant Neoplasms	ANK3	0	-2.197682	
16114	Malignant Neoplasms	TMIGD	0.000335	-2.196593	
16115	Malignant Neoplasms	C8orf83	0.001854	-2.182153	
16116	Malignant Neoplasms	C9orf91	0.000268	-2.17898	
16117	Malignant Neoplasms	E2F2	0.000521	-2.175628	
16118	Malignant Neoplasms	TCF21	0	-2.173875	
16119	Malignant Neoplasms	DNAJC4	0.002861	-2.171379	
16120	Malignant Neoplasms	ANKRD36	0.005131	-2.167662	
16121	Malignant Neoplasms	PPAP2A	0	-2.166829	
16122	Malignant Neoplasms	PRDX6	0	-2.159598	
16123	Malignant Neoplasms	CTSG	0.000001	-2.15913	
16124	Malignant Neoplasms	TTLL3	0.000297	-2.156659	
16125	Malignant Neoplasms	TSPAN7	0.000705	-2.148268	
16126	Malignant Neoplasms	TMEM54	0.000211	-2.141421	
16127	Malignant Neoplasms	NSMAF	0.000255	-2.133912	
16128	Malignant Neoplasms	PDXP	0.000006	-2.13219	
16129	Malignant Neoplasms	Q8NBX4	0.000102	-2.127648	
16130	Malignant Neoplasms	SDCCAG1	0.00091	-2.126706	
16131	Malignant Neoplasms	ZNF252	0.002619	-2.12491	
16132	Malignant Neoplasms	FAM200A	0.003763	-2.122202	
16133	Malignant Neoplasms	NSG1	0	-2.121418	
16134	Malignant Neoplasms	RHAG	0.046824	-2.120604	
16135	Malignant Neoplasms	NAGK	0.00063	-2.11225	
16136	Malignant Neoplasms	FUCA1	0	-2.108848	
16137	Malignant Neoplasms	PLAT	0.000028	-2.107739	
16138	Malignant Neoplasms	UNC5B	0.003512	-2.103171	
16139	Malignant Neoplasms	FAM125A	0.000355	-2.102634	
16140	Malignant Neoplasms	UQCRC1	0.000011	-2.101209	
16141	Malignant Neoplasms	ACAA1	0.000802	-2.099543	
16142	Malignant Neoplasms	EPCAM	0.005086	-2.095787	
16143	Malignant Neoplasms	NEUROG3	0.000017	-2.095584	
16144	Malignant Neoplasms	LOC284454	0.004125	-2.081457	
16145	Malignant Neoplasms	ECHDC2	0.004558	-2.078043	
16146	Malignant Neoplasms	C1QBP	0.000283	-2.075906	
16147	Malignant Neoplasms	C14orf49	0.000647	-2.074229	
16148	Malignant Neoplasms	ADSV	0.000576	-2.070961	

16149	Malignant Neoplasms	CD79A	0.000427	-2.069078	
16150	Malignant Neoplasms	TIMM44	0.000024	-2.066057	
16151	Malignant Neoplasms	LOC653198	0	-2.059828	
16152	Malignant Neoplasms	SYNJ2BP	0.000369	-2.059174	
16153	Malignant Neoplasms	TMSB10	0.000217	-2.0569	
16154	Malignant Neoplasms	VPS13D	0.003805	-2.055066	
16155	Malignant Neoplasms	ARF3	0.000391	-2.054823	
16156	Malignant Neoplasms	C17orf85	0.000326	-2.043168	
16157	Malignant Neoplasms	RNMTL1	0.000277	-2.043078	
16158	Malignant Neoplasms	PPP1R13B	0.004557	-2.039713	
16159	Malignant Neoplasms	LOC148709	0.000354	-2.03822	
16160	Malignant Neoplasms	GRIN1	0.001189	-2.033231	
16161	Malignant Neoplasms	CNTROB	0.000316	-2.030106	
16162	Malignant Neoplasms	MTHFD2L	0.004857	-2.02129	
16163	Malignant Neoplasms	SF3B5	0.000414	-2.006777	
16164	Malignant Neoplasms	MALL	0.00058	-2.001755	
16165	Malignant Neoplasms	CYP2R1	0.001404	-2.000093	
16166	Malignant Neoplasms	CBX3	0.000002	2.001406	
16167	Malignant Neoplasms	ZMYND19	0	2.00155	
16168	Malignant Neoplasms	LASP1	0.000001	2.002258	
16169	Malignant Neoplasms	EHBP1L1	0.000208	2.003314	
16170	Malignant Neoplasms	CTNND1	0.000737	2.007598	
16171	Malignant Neoplasms	LONP1	0	2.008715	
16172	Malignant Neoplasms	RGS12	0	2.009921	
16173	Malignant Neoplasms	MTA2	0.000001	2.010076	
16174	Malignant Neoplasms	GRINA	0.000204	2.011493	
16175	Malignant Neoplasms	AAA1	0	2.01351	
16176	Malignant Neoplasms	KIF15	0	2.015372	
16177	Malignant Neoplasms	GOLT1B	0	2.015554	
16178	Malignant Neoplasms	RIPK2	0	2.016821	
16179	Malignant Neoplasms	MTERFD1	0.000001	2.018794	
16180	Malignant Neoplasms	KIF11	0.000001	2.025286	
16181	Malignant Neoplasms	NUP107	0	2.028406	
16182	Malignant Neoplasms	CCDC59	0	2.030633	
16183	Malignant Neoplasms	PSD2	0	2.036907	
16184	Malignant Neoplasms	GPR56	0	2.037559	
16185	Malignant Neoplasms	LOC730286	0.000146	2.042263	
16186	Malignant Neoplasms	MICALL1	0.000242	2.04526	
16187	Malignant Neoplasms	LOC728769	0.000371	2.047471	
16188	Malignant Neoplasms	TIMELESS	0	2.051896	
16189	Malignant Neoplasms	PHF11	0.001695	2.062975	
16190	Malignant Neoplasms	TMEM91	0	2.063926	
16191	Malignant Neoplasms	OIP5	0.000001	2.065998	
16192	Malignant Neoplasms	KDM2B	0.004141	2.06911	
16193	Malignant Neoplasms	NUP205	0.000001	2.074642	
16194	Malignant Neoplasms	ADCK4	0	2.081367	
16195	Malignant Neoplasms	LOC647000	0	2.081938	
16196	Malignant Neoplasms	QPCTL	0	2.085368	
16197	Malignant Neoplasms	BPI	0	2.085919	
16198	Malignant Neoplasms	MFSD10	0	2.087086	

16199	Malignant Neoplasms	NDE1	0	2.09224	
16200	Malignant Neoplasms	RFC5	0.000001	2.096438	
16201	Malignant Neoplasms	EPPK1	0.000001	2.101393	
16202	Malignant Neoplasms	IGFBP7	0	2.103439	
16203	Malignant Neoplasms	NOC2L	0.026594	2.104099	
16204	Malignant Neoplasms	L3MBTL2	0	2.106203	
16205	Malignant Neoplasms	C20orf132	0	2.106547	
16206	Malignant Neoplasms	PSMA1	0.004439	2.107412	
16207	Malignant Neoplasms	SLC35A3	0.000202	2.107679	
16208	Malignant Neoplasms	CXorf40B	0.000486	2.111282	
16209	Malignant Neoplasms	MORC4	0.004279	2.112565	
16210	Malignant Neoplasms	SLC25A32	0.000001	2.113102	
16211	Malignant Neoplasms	GINS2	0.000001	2.11675	
16212	Malignant Neoplasms	NUP85	0.000001	2.11777	
16213	Malignant Neoplasms	C1orf107	0.002301	2.118563	
16214	Malignant Neoplasms	RBM28	0	2.120363	
16215	Malignant Neoplasms	COL18A1	0	2.120507	
16216	Malignant Neoplasms	SPOPL	0.00026	2.120846	
16217	Malignant Neoplasms	NCAPG2	0	2.125408	
16218	Malignant Neoplasms	FCHSD1	0	2.126384	
16219	Malignant Neoplasms	CAD	0.000001	2.127343	
16220	Malignant Neoplasms	NAPRT1	0.000167	2.127462	
16221	Malignant Neoplasms	GTF2IRD1	0	2.133547	
16222	Malignant Neoplasms	TAZ	0.000143	2.133776	
16223	Malignant Neoplasms	FANCL	0	2.139324	
16224	Malignant Neoplasms	GZMB	0	2.14732	
16225	Malignant Neoplasms	KIF2C	0.000001	2.154208	
16226	Malignant Neoplasms	OAS3	0.000001	2.155018	
16227	Malignant Neoplasms	TIE1	0	2.16211	
16228	Malignant Neoplasms	PORCN	0	2.162538	
16229	Malignant Neoplasms	LOC731275	0.000002	2.163543	
16230	Malignant Neoplasms	ANO3	0	2.166345	
16231	Malignant Neoplasms	LOC90835	0.001125	2.166442	
16232	Malignant Neoplasms	FANCG	0	2.166676	
16233	Malignant Neoplasms	CCL4	0	2.174968	
16234	Malignant Neoplasms	LOC401357	0	2.17545	
16235	Malignant Neoplasms	SFRS3	0.023214	2.178891	
16236	Malignant Neoplasms	PPIA	0	2.185152	
16237	Malignant Neoplasms	PCBP3	0	2.186495	
16238	Malignant Neoplasms	WDR33	0	2.186975	
16239	Malignant Neoplasms	SLC38A3	0	2.187399	
16240	Malignant Neoplasms	RAB15	0	2.188293	
16241	Malignant Neoplasms	MCM3	0	2.189712	
16242	Malignant Neoplasms	JAG2	0.000001	2.191192	
16243	Malignant Neoplasms	WDR43	0.000001	2.194866	
16244	Malignant Neoplasms	C10orf71	0	2.194946	
16245	Malignant Neoplasms	CSNK2B	0.000285	2.197823	
16246	Malignant Neoplasms	FAM29A	0	2.20299	
16247	Malignant Neoplasms	OLR1	0.000557	2.207088	
16248	Malignant Neoplasms	GTPBP5	0	2.213754	

16249	Malignant Neoplasms	ARIH1	0.000605	2.21698	
16250	Malignant Neoplasms	HOXB6	0.022575	2.220702	
16251	Malignant Neoplasms	CLCN7	0	2.234566	
16252	Malignant Neoplasms	ITGB1	0.000252	2.23583	
16253	Malignant Neoplasms	EXOSC8	0.023557	2.246594	
16254	Malignant Neoplasms	KIF19	0	2.251696	
16255	Malignant Neoplasms	POLR1D	0.000001	2.252838	
16256	Malignant Neoplasms	PTCD1	0	2.255095	
16257	Malignant Neoplasms	KIAA1430	0.004953	2.262002	
16258	Malignant Neoplasms	NUP37	0.000001	2.262069	
16259	Malignant Neoplasms	AP1G1	0.000433	2.263637	
16260	Malignant Neoplasms	FAM3A	0.000237	2.271018	
16261	Malignant Neoplasms	COMT	0.0087	2.27312	
16262	Malignant Neoplasms	CCDC19	0.000041	2.280748	
16263	Malignant Neoplasms	CHD7	0.000445	2.281194	
16264	Malignant Neoplasms	CBX4	0	2.293435	
16265	Malignant Neoplasms	TMEM109	0	2.298206	
16266	Malignant Neoplasms	LOC731966	0	2.301043	
16267	Malignant Neoplasms	TPTE	0	2.301437	
16268	Malignant Neoplasms	SLC26A4	0	2.301524	
16269	Malignant Neoplasms	MYO1H	0	2.304578	
16270	Malignant Neoplasms	MICB	0	2.307009	
16271	Malignant Neoplasms	TNFRSF10A	0	2.308232	
16272	Malignant Neoplasms	RASSF7	0.000148	2.313444	
16273	Malignant Neoplasms	F2R	0	2.3171	
16274	Malignant Neoplasms	BHLHE22	0	2.322857	
16275	Malignant Neoplasms	DARS2	0.003721	2.328628	
16276	Malignant Neoplasms	HS2ST1	0.000002	2.328743	
16277	Malignant Neoplasms	CECR2	0	2.332804	
16278	Malignant Neoplasms	SOX9	0	2.336502	
16279	Malignant Neoplasms	CKS2	0	2.34218	
16280	Malignant Neoplasms	KIAA1881	0.000237	2.349848	
16281	Malignant Neoplasms	POLN	0	2.353199	
16282	Malignant Neoplasms	USH2A	0	2.355116	
16283	Malignant Neoplasms	NRK	0	2.357796	
16284	Malignant Neoplasms	ORAI2	0.000355	2.368232	
16285	Malignant Neoplasms	DOK1	0	2.368591	
16286	Malignant Neoplasms	COL24A1	0	2.375741	
16287	Malignant Neoplasms	HEATR4	0	2.383003	
16288	Malignant Neoplasms	ERO1L	0	2.38391	
16289	Malignant Neoplasms	MRPS17	0.000001	2.391333	
16290	Malignant Neoplasms	KIF9	0	2.398162	
16291	Malignant Neoplasms	ZWILCH	0	2.403617	
16292	Malignant Neoplasms	CDH26	0	2.404148	
16293	Malignant Neoplasms	JAKMIP2	0	2.406255	
16294	Malignant Neoplasms	AOC2	0	2.41181	
16295	Malignant Neoplasms	ITGAM	0	2.411873	
16296	Malignant Neoplasms	CSPP1	0.004279	2.417897	
16297	Malignant Neoplasms	ILF3	0.00296	2.417933	
16298	Malignant Neoplasms	RFX4	0	2.420886	

16299	Malignant Neoplasms	ADFP	0.00024	2.429958	
16300	Malignant Neoplasms	ANGPTL4	0.000173	2.440066	
16301	Malignant Neoplasms	TMEM184A	0.000061	2.443268	
16302	Malignant Neoplasms	SP140	0.000246	2.444219	
16303	Malignant Neoplasms	MLLT6	0.000005	2.453535	
16304	Malignant Neoplasms	ADAMTSL2	0.000012	2.453786	
16305	Malignant Neoplasms	PSAT1	0.000001	2.45864	
16306	Malignant Neoplasms	NFE2L3	0	2.463088	
16307	Malignant Neoplasms	CSE1L	0.000001	2.467005	
16308	Malignant Neoplasms	MAK	0	2.472108	
16309	Malignant Neoplasms	EYA1	0	2.472297	
16310	Malignant Neoplasms	ANKFN1	0	2.472601	
16311	Malignant Neoplasms	C12orf11	0	2.477935	
16312	Malignant Neoplasms	KRT39	0	2.480666	
16313	Malignant Neoplasms	FAM127A	0.000531	2.480875	
16314	Malignant Neoplasms	CARD11	0	2.480952	
16315	Malignant Neoplasms	PRDM8	0	2.481532	
16316	Malignant Neoplasms	PKD1L3	0	2.481772	
16317	Malignant Neoplasms	ZNF587	0.000704	2.482609	
16318	Malignant Neoplasms	CTPS	0	2.484479	
16319	Malignant Neoplasms	CA6	0	2.484544	
16320	Malignant Neoplasms	RPL35A	0.023557	2.488761	
16321	Malignant Neoplasms	ICAM1	0	2.498583	
16322	Malignant Neoplasms	IRAK1	0.000428	2.500215	
16323	Malignant Neoplasms	FBXO46	0.000685	2.505657	
16324	Malignant Neoplasms	GTF3A	0	2.514476	
16325	Malignant Neoplasms	IQCH	0	2.516142	
16326	Malignant Neoplasms	S100A11	0	2.517473	
16327	Malignant Neoplasms	S100A6	0.000473	2.524376	
16328	Malignant Neoplasms	YIPF3	0.000716	2.525645	
16329	Malignant Neoplasms	CYP1B1	0.000314	2.525663	
16330	Malignant Neoplasms	CCDC92	0.00358	2.52573	
16331	Malignant Neoplasms	GPRC5C	0.000292	2.527811	
16332	Malignant Neoplasms	PLD6	0.000062	2.530931	
16333	Malignant Neoplasms	FLJ22167	0	2.534436	
16334	Malignant Neoplasms	SLC7A6	0.005899	2.538138	
16335	Malignant Neoplasms	NPHP1	0	2.545304	
16336	Malignant Neoplasms	SLC7A1	0	2.546978	
16337	Malignant Neoplasms	CEP250	0	2.550342	
16338	Malignant Neoplasms	RAPGEF4	0	2.551132	
16339	Malignant Neoplasms	BCL6B	0	2.554916	
16340	Malignant Neoplasms	DCC	0	2.558436	
16341	Malignant Neoplasms	NALCN	0	2.560201	
16342	Malignant Neoplasms	MCM7	0	2.562626	
16343	Malignant Neoplasms	PSMB8	0.004857	2.5672	
16344	Malignant Neoplasms	UBE2L6	0.000001	2.570384	
16345	Malignant Neoplasms	TTC25	0	2.571125	
16346	Malignant Neoplasms	PSMG1	0.000001	2.572526	
16347	Malignant Neoplasms	TMEM14A	0.00296	2.575291	
16348	Malignant Neoplasms	EPS8L2	0.000233	2.589922	

16349	Malignant Neoplasms	ABCA13	0	2.590263	
16350	Malignant Neoplasms	SFXN5	0.000297	2.594348	
16351	Malignant Neoplasms	CCBL1	0	2.59711	
16352	Malignant Neoplasms	CASS4	0	2.601324	
16353	Malignant Neoplasms	tcag7.907	0	2.602368	
16354	Malignant Neoplasms	PSAP	0.002393	2.610683	
16355	Malignant Neoplasms	CTNNBL1	0	2.612824	
16356	Malignant Neoplasms	NPVF	0	2.613849	
16357	Malignant Neoplasms	HEATR7A	0.000492	2.614327	
16358	Malignant Neoplasms	UBE2V1	0.004279	2.625998	
16359	Malignant Neoplasms	LITAF	0.000104	2.628143	
16360	Malignant Neoplasms	LIN7A	0	2.628195	
16361	Malignant Neoplasms	FAM176A	0	2.634253	
16362	Malignant Neoplasms	GLIPR1	0.000011	2.636184	
16363	Malignant Neoplasms	DHCR7	0	2.637916	
16364	Malignant Neoplasms	MMP13	0	2.64178	
16365	Malignant Neoplasms	FBXL13	0	2.642704	
16366	Malignant Neoplasms	LRRC57	0.000491	2.6482	
16367	Malignant Neoplasms	WDR31	0	2.648876	
16368	Malignant Neoplasms	TMEM161A	0	2.649004	
16369	Malignant Neoplasms	CCNA2	0.000001	2.6516	
16370	Malignant Neoplasms	PPM1F	0.001854	2.653937	
16371	Malignant Neoplasms	HN1L	0	2.666329	
16372	Malignant Neoplasms	ALDH4A1	0.000232	2.672146	
16373	Malignant Neoplasms	NEB	0	2.673266	
16374	Malignant Neoplasms	ZNF474	0	2.681025	
16375	Malignant Neoplasms	MSLN	0	2.690638	
16376	Malignant Neoplasms	BOP1	0	2.693284	
16377	Malignant Neoplasms	AP1S2	0.003236	2.693499	
16378	Malignant Neoplasms	CGNL1	0.000004	2.694607	
16379	Malignant Neoplasms	GBP1	0	2.698104	
16380	Malignant Neoplasms	CDC25B	0.000001	2.70041	
16381	Malignant Neoplasms	EIF2AK2	0.004176	2.716897	
16382	Malignant Neoplasms	RDH10	0.000327	2.736183	
16383	Malignant Neoplasms	AKT1S1	0.000204	2.736527	
16384	Malignant Neoplasms	XKRX	0	2.73967	
16385	Malignant Neoplasms	C21orf71	0.000014	2.746564	
16386	Malignant Neoplasms	NPC1L1	0	2.754101	
16387	Malignant Neoplasms	FEN1	0.000001	2.754245	
16388	Malignant Neoplasms	LOC647979	0.004217	2.755327	
16389	Malignant Neoplasms	CTSD	0.000072	2.758571	
16390	Malignant Neoplasms	PTPRU	0	2.768652	
16391	Malignant Neoplasms	ABCC2	0	2.78484	
16392	Malignant Neoplasms	MET	0	2.796537	
16393	Malignant Neoplasms	SEC24D	0.000433	2.806777	
16394	Malignant Neoplasms	NCOA3	0.003805	2.809919	
16395	Malignant Neoplasms	IFIH1	0.000647	2.81145	
16396	Malignant Neoplasms	MOBKL2C	0.000413	2.821208	
16397	Malignant Neoplasms	CAMSAP1L1	0.002817	2.822373	
16398	Malignant Neoplasms	CLNK	0	2.822808	

16399	Malignant Neoplasms	ADAMTS3	0	2.824047	
16400	Malignant Neoplasms	SLC9A3R2	0.000054	2.842996	
16401	Malignant Neoplasms	LGR5	0.000001	2.855969	
16402	Malignant Neoplasms	TAPBP	0.000001	2.856384	
16403	Malignant Neoplasms	LMNB2	0	2.858719	
16404	Malignant Neoplasms	SLC12A7	0.000647	2.86646	
16405	Malignant Neoplasms	NT5DC2	0	2.876001	
16406	Malignant Neoplasms	MCM8	0.004563	2.888232	
16407	Malignant Neoplasms	ENTPD4	0	2.89158	
16408	Malignant Neoplasms	IQCE	0.000359	2.901126	
16409	Malignant Neoplasms	EFNA3	0	2.90539	
16410	Malignant Neoplasms	HNRNPL	0	2.905758	
16411	Malignant Neoplasms	DYX1C1	0	2.918594	
16412	Malignant Neoplasms	TFEB	0.000114	2.925089	
16413	Malignant Neoplasms	NNMT	0.000273	2.93126	
16414	Malignant Neoplasms	C11orf45	0	2.931764	
16415	Malignant Neoplasms	CFI	0.000174	2.93439	
16416	Malignant Neoplasms	IFIT3	0.000647	2.948601	
16417	Malignant Neoplasms	MED25	0.000405	2.953415	
16418	Malignant Neoplasms	C4orf39	0	2.956402	
16419	Malignant Neoplasms	GPX1	0.000488	2.957755	
16420	Malignant Neoplasms	SLC16A5	0.000605	2.961518	
16421	Malignant Neoplasms	INHBE	0.000642	2.971835	
16422	Malignant Neoplasms	SLPI	0	2.971966	
16423	Malignant Neoplasms	CDCA7	0	2.977459	
16424	Malignant Neoplasms	C10orf10	0.000005	2.992878	
16425	Malignant Neoplasms	MAEL	0	2.996037	
16426	Malignant Neoplasms	ARHGAP23	0.000516	2.996162	
16427	Malignant Neoplasms	EME2	0.00077	2.998752	
16428	Malignant Neoplasms	PPP4R4	0	2.999379	
16429	Malignant Neoplasms	SUV420H1	0.000554	3.007917	
16430	Malignant Neoplasms	LOC100505522	0.000847	3.015464	
16431	Malignant Neoplasms	MACC1	0.003549	3.024789	
16432	Malignant Neoplasms	TNFRSF21	0.000066	3.029186	
16433	Malignant Neoplasms	KIF4A	0.000001	3.052197	
16434	Malignant Neoplasms	LPIN2	0.000686	3.067679	
16435	Malignant Neoplasms	PHLDA3	0.000037	3.070606	
16436	Malignant Neoplasms	GINS1	0	3.07162	
16437	Malignant Neoplasms	MTMR10	0.000202	3.076297	
16438	Malignant Neoplasms	F12	0	3.082553	
16439	Malignant Neoplasms	EDAR	0	3.098888	
16440	Malignant Neoplasms	TINAGL1	0.000151	3.103956	
16441	Malignant Neoplasms	STAT1	0	3.107809	
16442	Malignant Neoplasms	LCA5	0	3.115799	
16443	Malignant Neoplasms	TRIP13	0	3.129853	
16444	Malignant Neoplasms	RNF219	0.000471	3.147289	
16445	Malignant Neoplasms	STK31	0	3.147914	
16446	Malignant Neoplasms	ITGA3	0.000291	3.174543	
16447	Malignant Neoplasms	LOC650331	0	3.206762	
16448	Malignant Neoplasms	ASPH	0.000671	3.221404	

16449	Malignant Neoplasms	UNC93B1	0.000002	3.228436	
16450	Malignant Neoplasms	F5	0.00014	3.231423	
16451	Malignant Neoplasms	KLHL35	0.000033	3.235531	
16452	Malignant Neoplasms	NUTF2	0	3.241433	
16453	Malignant Neoplasms	CEP55	0	3.248565	
16454	Malignant Neoplasms	MMP14	0	3.249611	
16455	Malignant Neoplasms	SOD2	0	3.254564	
16456	Malignant Neoplasms	FOXM1	0	3.25519	
16457	Malignant Neoplasms	LOC731404	0	3.266765	
16458	Malignant Neoplasms	BUB1	0.003759	3.279526	
16459	Malignant Neoplasms	SRCAP	0.000216	3.28083	
16460	Malignant Neoplasms	RXRA	0.000037	3.293291	
16461	Malignant Neoplasms	FANCI	0.0087	3.309157	
16462	Malignant Neoplasms	PRKD2	0.000124	3.334099	
16463	Malignant Neoplasms	TRIM56	0.000414	3.341161	
16464	Malignant Neoplasms	PIWIL1	0	3.34161	
16465	Malignant Neoplasms	CERKL	0	3.342308	
16466	Malignant Neoplasms	PPARA	0.000707	3.343854	
16467	Malignant Neoplasms	DPEP1	0.000827	3.34871	
16468	Malignant Neoplasms	CDH2	0	3.362404	
16469	Malignant Neoplasms	RAB3D	0.000388	3.404948	
16470	Malignant Neoplasms	MCM4	0	3.415645	
16471	Malignant Neoplasms	PLEKHA4	0.000011	3.432111	
16472	Malignant Neoplasms	C14orf115	0	3.437692	
16473	Malignant Neoplasms	IDUA	0.000729	3.446031	
16474	Malignant Neoplasms	TACSTD2	0	3.446898	
16475	Malignant Neoplasms	FZD5	0.000169	3.460795	
16476	Malignant Neoplasms	GFPT1	0.00021	3.46894	
16477	Malignant Neoplasms	PHLDA1	0	3.493862	
16478	Malignant Neoplasms	MIF	0.000001	3.504093	
16479	Malignant Neoplasms	TMEM106A	0.000004	3.534425	
16480	Malignant Neoplasms	TRPM4	0.000886	3.538012	
16481	Malignant Neoplasms	DOK4	0.00054	3.553059	
16482	Malignant Neoplasms	F11R	0.000851	3.555513	
16483	Malignant Neoplasms	GPSM2	0.000018	3.562648	
16484	Malignant Neoplasms	IFIT5	0.002833	3.586507	
16485	Malignant Neoplasms	DYRK2	0.000433	3.632288	
16486	Malignant Neoplasms	SSH3	0	3.644911	
16487	Malignant Neoplasms	BGN	0	3.64899	
16488	Malignant Neoplasms	PCSK6	0.000497	3.670522	
16489	Malignant Neoplasms	VNN1	0.000533	3.670827	
16490	Malignant Neoplasms	NEBL	0	3.699267	
16491	Malignant Neoplasms	WDSOF1	0.001352	3.709982	
16492	Malignant Neoplasms	PBK	0.000001	3.714962	
16493	Malignant Neoplasms	COL4A1	0	3.732771	
16494	Malignant Neoplasms	KLK6	0	3.733988	
16495	Malignant Neoplasms	RNASET2	0.000257	3.741787	
16496	Malignant Neoplasms	HIG2	0	3.74435	
16497	Malignant Neoplasms	ANLN	0	3.792495	
16498	Malignant Neoplasms	APOE	0	3.797515	

16499	Malignant Neoplasms	TMPRSS3	0	3.798986	
16500	Malignant Neoplasms	TMEM97	0	3.826889	
16501	Malignant Neoplasms	KCNJ15	0	3.837106	
16502	Malignant Neoplasms	B3GALTL	0.049356	3.840546	
16503	Malignant Neoplasms	ALDH3A1	0.000597	3.841355	
16504	Malignant Neoplasms	AQP9	0.000053	3.849158	
16505	Malignant Neoplasms	RELL2	0.003759	3.855649	
16506	Malignant Neoplasms	CCDC86	0.002201	3.867938	
16507	Malignant Neoplasms	E2F5	0.005272	3.890783	
16508	Malignant Neoplasms	HSD11B1	0.000121	3.932743	
16509	Malignant Neoplasms	TNNI2	0.000011	3.944015	
16510	Malignant Neoplasms	PUS7	0	3.945489	
16511	Malignant Neoplasms	PVRL2	0.000263	3.967878	
16512	Malignant Neoplasms	CCRL2	0.000466	3.970309	
16513	Malignant Neoplasms	XPOT	0.014459	3.992622	
16514	Malignant Neoplasms	PWWP2B	0.000355	4.024844	
16515	Malignant Neoplasms	NLRC5	0.000204	4.032659	
16516	Malignant Neoplasms	PMAIP1	0	4.0525	
16517	Malignant Neoplasms	CDC2	0	4.067238	
16518	Malignant Neoplasms	CLDN2	0	4.07628	
16519	Malignant Neoplasms	SLC45A4	0.000471	4.093217	
16520	Malignant Neoplasms	ADAP1	0.000647	4.096481	
16521	Malignant Neoplasms	SERPINE1	0.000061	4.1164	
16522	Malignant Neoplasms	CXCL9	0	4.116745	
16523	Malignant Neoplasms	FTL	0.003014	4.124829	
16524	Malignant Neoplasms	DMBX1	0.000226	4.161581	
16525	Malignant Neoplasms	CKB	0.000302	4.191771	
16526	Malignant Neoplasms	IFITM1	0	4.28338	
16527	Malignant Neoplasms	KLF5	0.00032	4.284359	
16528	Malignant Neoplasms	ASGR2	0.000553	4.302714	
16529	Malignant Neoplasms	DNM1	0.000359	4.312468	
16530	Malignant Neoplasms	SLCO4A1	0	4.326759	
16531	Malignant Neoplasms	SERPINA5	0.000352	4.345646	
16532	Malignant Neoplasms	PVRL1	0.000522	4.458198	
16533	Malignant Neoplasms	SRPX2	0	4.507537	
16534	Malignant Neoplasms	NOXO1	0.000183	4.549523	
16535	Malignant Neoplasms	ATP6V1B1	0.000074	4.572513	
16536	Malignant Neoplasms	SLC6A6	0	4.621348	
16537	Malignant Neoplasms	PTGS2	0.000139	4.634416	
16538	Malignant Neoplasms	IRF9	0.000104	4.634974	
16539	Malignant Neoplasms	SERPINA1	0.000392	4.686709	
16540	Malignant Neoplasms	SULT2A1	0.000605	4.720173	
16541	Malignant Neoplasms	FMO3	0.000071	4.720673	
16542	Malignant Neoplasms	CYTH2	0.000085	4.752988	
16543	Malignant Neoplasms	CLCC1	0.000647	4.781185	
16544	Malignant Neoplasms	IL15	0.000239	4.831838	
16545	Malignant Neoplasms	SERPINF2	0.00067	4.841546	
16546	Malignant Neoplasms	CXCL10	0	4.8737	
16547	Malignant Neoplasms	MBP	0.000647	4.960741	
16548	Malignant Neoplasms	RICTOR	0.000704	4.981218	

16549	Malignant Neoplasms	TYMP	0.000003	4.981838	
16550	Malignant Neoplasms	AHR	0.000426	5.006937	
16551	Malignant Neoplasms	SLCO1B3	0	5.007902	
16552	Malignant Neoplasms	DACH1	0.001964	5.017765	
16553	Malignant Neoplasms	ATP10B	0.000833	5.058226	
16554	Malignant Neoplasms	HKDC1	0	5.076388	
16555	Malignant Neoplasms	ABLIM3	0.000198	5.087636	
16556	Malignant Neoplasms	IL22RA1	0.000603	5.144601	
16557	Malignant Neoplasms	CXCL1	0	5.226033	
16558	Malignant Neoplasms	FAM113B	0.000471	5.288628	
16559	Malignant Neoplasms	COL1A2	0	5.292898	
16560	Malignant Neoplasms	CP	0.00038	5.509002	
16561	Malignant Neoplasms	CPB2	0.000432	5.613978	
16562	Malignant Neoplasms	C3	0.000006	5.668511	
16563	Malignant Neoplasms	PTK2	0.000112	5.687439	
16564	Malignant Neoplasms	HMMR	0.000025	5.804377	
16565	Malignant Neoplasms	GATA2	0.000049	5.893886	
16566	Malignant Neoplasms	CTSE	0.000727	5.963868	
16567	Malignant Neoplasms	MUPCDH	0.000494	5.977978	
16568	Malignant Neoplasms	ZNF702P	0.000417	6.02341	
16569	Malignant Neoplasms	TINAG	0.000471	6.02389	
16570	Malignant Neoplasms	WNT10A	0.000038	6.06077	
16571	Malignant Neoplasms	SLC13A5	0.000331	6.076943	
16572	Malignant Neoplasms	C8A	0.000531	6.120991	
16573	Malignant Neoplasms	PLEKHG1	0.00029	6.186895	
16574	Malignant Neoplasms	PAK1	0.000697	6.20532	
16575	Malignant Neoplasms	ELL3	0.000471	6.218115	
16576	Malignant Neoplasms	LRG1	0.000309	6.245968	
16577	Malignant Neoplasms	PLK2	0.000051	6.303497	
16578	Malignant Neoplasms	BTG1	0.000133	6.312695	
16579	Malignant Neoplasms	MMP10	0	6.397166	
16580	Malignant Neoplasms	DSC3	0	6.40146	
16581	Malignant Neoplasms	ZHX1	0.000538	6.473214	
16582	Malignant Neoplasms	PIK3R5	0	6.528764	
16583	Malignant Neoplasms	RBP4	0.000557	6.658636	
16584	Malignant Neoplasms	PYGB	0.000168	6.672657	
16585	Malignant Neoplasms	ASGR1	0.000048	6.679332	
16586	Malignant Neoplasms	SLC28A3	0.000255	6.682218	
16587	Malignant Neoplasms	RHBDL2	0.000597	6.686852	
16588	Malignant Neoplasms	C5	0.00011	6.687382	
16589	Malignant Neoplasms	AGRN	0.000001	6.749664	
16590	Malignant Neoplasms	PLEKHA7	0.000244	6.787971	
16591	Malignant Neoplasms	FCN3	0.000009	6.844049	
16592	Malignant Neoplasms	APOC2	0.000051	6.904064	
16593	Malignant Neoplasms	LOC651255	0	6.905853	
16594	Malignant Neoplasms	AGXT	0.000773	6.935567	
16595	Malignant Neoplasms	HPD	0.000531	7.014538	
16596	Malignant Neoplasms	SEMA4B	0.000534	7.032327	
16597	Malignant Neoplasms	C6	0.00046	7.054535	
16598	Malignant Neoplasms	SAA4	0.000529	7.065	

16599	Malignant Neoplasms	LRRC31	0.00014	7.071815	
16600	Malignant Neoplasms	ETV7	0.000059	7.214569	
16601	Malignant Neoplasms	CXCL3	0	7.233392	
16602	Malignant Neoplasms	LGALS9	0.000378	7.278245	
16603	Malignant Neoplasms	C6orf145	0.000087	7.289675	
16604	Malignant Neoplasms	PLG	0.000498	7.361402	
16605	Malignant Neoplasms	PON3	0.000647	7.506333	
16606	Malignant Neoplasms	MMRN2	0.000099	7.512883	
16607	Malignant Neoplasms	ITIH1	0.000337	7.554118	
16608	Malignant Neoplasms	DDC	0.000448	7.746083	
16609	Malignant Neoplasms	ZAK	0.000658	7.747274	
16610	Malignant Neoplasms	GAB1	0.000275	7.851537	
16611	Malignant Neoplasms	PLXNA2	0.000462	7.884722	
16612	Malignant Neoplasms	C4BPA	0.000535	7.948833	
16613	Malignant Neoplasms	APOC1	0.000001	8.000367	
16614	Malignant Neoplasms	LGALS9C	0.000155	8.105818	
16615	Malignant Neoplasms	TTR	0.000676	8.109081	
16616	Malignant Neoplasms	DHRS9	0.000282	8.203724	
16617	Malignant Neoplasms	CYP2C8	0.000673	8.207707	
16618	Malignant Neoplasms	TESC	0	8.282366	
16619	Malignant Neoplasms	UPK3A	0.000419	8.411729	
16620	Malignant Neoplasms	DTX4	0.000202	8.54253	
16621	Malignant Neoplasms	SLC16A4	0.000293	8.722472	
16622	Malignant Neoplasms	CLDN1	0	8.769876	
16623	Malignant Neoplasms	VEGFA	0.00073	8.807001	
16624	Malignant Neoplasms	DSG3	0	8.93798	
16625	Malignant Neoplasms	CXCL2	0	8.951083	
16626	Malignant Neoplasms	LBP	0.000127	8.984359	
16627	Malignant Neoplasms	HAMP	0.000059	9.033264	
16628	Malignant Neoplasms	IL1A	0	9.050041	
16629	Malignant Neoplasms	TRIM29	0	9.058532	
16630	Malignant Neoplasms	TCN1	0	9.094781	
16631	Malignant Neoplasms	KRT23	0	9.22809	
16632	Malignant Neoplasms	KRTCAP3	0.000382	9.275024	
16633	Malignant Neoplasms	RAB7B	0.000304	9.350441	
16634	Malignant Neoplasms	KIAA0182	0.000139	9.586452	
16635	Malignant Neoplasms	APCS	0.000082	9.596353	
16636	Malignant Neoplasms	ETV4	0	9.616632	
16637	Malignant Neoplasms	DDX60	0.00012	9.946946	
16638	Malignant Neoplasms	TMPRSS4	0.000313	10.114752	
16639	Malignant Neoplasms	FAM3D	0.00003	10.343878	
16640	Malignant Neoplasms	KNG1	0.000331	10.388199	
16641	Malignant Neoplasms	GJB6	0.000095	10.461198	
16642	Malignant Neoplasms	ARSJ	0.000233	10.87893	
16643	Malignant Neoplasms	HRG	0.000312	11.044669	
16644	Malignant Neoplasms	SGK1	0.000413	11.064812	
16645	Malignant Neoplasms	TMC4	0.000486	11.248471	
16646	Malignant Neoplasms	IFI27	0.000005	11.248729	
16647	Malignant Neoplasms	CEACAM1	0.000605	11.382833	
16648	Malignant Neoplasms	SERPINC1	0.000285	11.791284	

16649	Malignant Neoplasms	SERPINA3	0.000001	11.942192	
16650	Malignant Neoplasms	CMBL	0.000647	11.99801	
16651	Malignant Neoplasms	FAM13A	0.000647	12.331898	
16652	Malignant Neoplasms	APOB	0.000088	13.0809	
16653	Malignant Neoplasms	AHSG	0.000112	13.371622	
16654	Malignant Neoplasms	TUSC1	0.000186	13.373626	
16655	Malignant Neoplasms	APOC3	0.000181	13.701454	
16656	Malignant Neoplasms	ZNF264	0.000244	13.760516	
16657	Malignant Neoplasms	MMP1	0	13.93607	
16658	Malignant Neoplasms	LGR4	0.000266	13.954556	
16659	Malignant Neoplasms	CRIP2	0.000514	14.058359	
16660	Malignant Neoplasms	HPX	0.000192	14.287143	
16661	Malignant Neoplasms	MVP	0.000139	14.559649	
16662	Malignant Neoplasms	FGG	0.000014	14.651355	
16663	Malignant Neoplasms	FGFR3	0.000591	14.82059	
16664	Malignant Neoplasms	PROS1	0.000392	15.1562	
16665	Malignant Neoplasms	F2	0.000047	15.443368	
16666	Malignant Neoplasms	VTN	0.000044	15.844447	
16667	Malignant Neoplasms	LOC100506781	0.000686	15.886755	
16668	Malignant Neoplasms	HNMT	0.000462	15.912229	
16669	Malignant Neoplasms	ORM2	0.00009	16.094898	
16670	Malignant Neoplasms	CYP2E1	0.000072	16.227626	
16671	Malignant Neoplasms	CYP3A5	0.000647	16.404272	
16672	Malignant Neoplasms	SAMD9	0.000225	17.780734	
16673	Malignant Neoplasms	FGL1	0.000039	18.32827	
16674	Malignant Neoplasms	TF	0.000068	18.608249	
16675	Malignant Neoplasms	PLEKHA6	0.000104	18.991039	
16676	Malignant Neoplasms	CTSH	0.00045	19.875231	
16677	Malignant Neoplasms	ZNF655	0.000239	20.238714	
16678	Malignant Neoplasms	IFI6	0	20.704685	
16679	Malignant Neoplasms	GC	0.000013	20.947027	
16680	Malignant Neoplasms	AMBP	0.000021	21.099729	
16681	Malignant Neoplasms	APOA2	0.000023	21.365529	
16682	Malignant Neoplasms	HP	0.000009	21.852969	
16683	Malignant Neoplasms	RASSF6	0.000063	22.251862	
16684	Malignant Neoplasms	NUDC	0	22.897111	
16685	Malignant Neoplasms	ALB	0.000007	23.019959	
16686	Malignant Neoplasms	APOA1	0.000018	23.169381	
16687	Malignant Neoplasms	ANXA13	0.000244	23.216715	
16688	Malignant Neoplasms	FGB	0.000008	23.984148	
16689	Malignant Neoplasms	FGA	0.000008	25.158992	
16690	Malignant Neoplasms	AIM1	0.000428	25.235589	
16691	Malignant Neoplasms	CRP	0.000002	25.311911	
16692	Malignant Neoplasms	ORM1	0.000008	25.514711	
16693	Malignant Neoplasms	POF1B	0.000531	25.953685	
16694	Malignant Neoplasms	GPR37	0.000647	26.551556	
16695	Malignant Neoplasms	RNF135	0.000244	26.826077	
16696	Malignant Neoplasms	SLC44A4	0.000317	32.806091	
16697	Malignant Neoplasms	DPP4	0.000085	35.946916	
16698	Malignant Neoplasms	AZGP1	0.000547	37.046467	

16699	Malignant Neoplasms	LOC100128511	0	37.054894	
16700	Malignant Neoplasms	XIST	0.000382	42.125514	
16701	Malignant Neoplasms	DAPK1	0.000189	47.300908	
16702	Malignant Neoplasms	TMED3	0.000647	48.224737	
16703	Malignant Neoplasms	AQP3	0.00055	49.138813	
16704	Malignant Neoplasms	RASEF	0.000163	49.84891	
16705	Malignant Neoplasms	C2orf55	0	54.032204	
16706	Malignant Neoplasms	MUC20	0.000067	55.376958	
16707	Malignant Neoplasms	CLRN3	0.00058	58.316622	
16708	Malignant Neoplasms	SEMA3C	0.000382	70.986243	
16709	Malignant Neoplasms	GPX2	0.000104	81.770143	
16710	Malignant Neoplasms	CCL14-CCL15	0.000879	87.224563	
16711	Malignant Neoplasms	RAB25	0.000102	101.425117	
16712	Malignant Neoplasms	TSPAN8	0.000392	151.056058	
16713	Malignant Neoplasms	AKR1C3	0.000095	258.985756	
16714	Malignant Neoplasms	LYZ	0.000067	378.645226	
16715	Malignant tumor of colon	MAPK10	0.010117	-4.758095	
16716	Malignant tumor of colon	DLGAP4	0.009754	-2.082438	
16717	Malignant tumor of colon	NOC2L	0.026594	2.104099	
16718	Malignant tumor of colon	MORC4	0.004279	2.112565	
16719	Malignant tumor of colon	LOC90835	0.001125	2.166442	
16720	Malignant tumor of colon	SFRS3	0.023214	2.178891	
16721	Malignant tumor of colon	EXOSC8	0.023557	2.246594	
16722	Malignant tumor of colon	COMT	0.0087	2.27312	
16723	Malignant tumor of colon	DARS2	0.003721	2.328628	
16724	Malignant tumor of colon	ENC1	0.005205	2.353768	
16725	Malignant tumor of colon	CSPP1	0.004279	2.417897	
16726	Malignant tumor of colon	RPL35A	0.023557	2.488761	
16727	Malignant tumor of colon	SLC7A6	0.005899	2.538138	
16728	Malignant tumor of colon	UBE2V1	0.004279	2.625998	
16729	Malignant tumor of colon	MCM8	0.004563	2.888232	
16730	Malignant tumor of colon	C13orf3	0.010117	3.221261	
16731	Malignant tumor of colon	BUB1	0.003759	3.279526	
16732	Malignant tumor of colon	FANCI	0.0087	3.309157	
16733	Malignant tumor of colon	CDC6	0.011855	3.551337	
16734	Malignant tumor of colon	WDSOF1	0.001352	3.709982	
16735	Malignant tumor of colon	RELL2	0.003759	3.855649	
16736	Malignant tumor of colon	CCDC86	0.002201	3.867938	
16737	Malignant tumor of colon	E2F5	0.005272	3.890783	
16738	Malignant tumor of colon	XPOT	0.014459	3.992622	
16739	Malignant tumor of colon	KIF20A	0.000391	5.468525	
16740	Malignant tumor of colon	HMMR	0.000025	5.804377	
16741	Maximum	PTPRR	0	-6.324252	
16742	Maximum	LGR5	0	-5.820083	
16743	Maximum	MEF2C	0	-3.441646	
16744	Maximum	LOC100507039	0	-3.435516	
16745	Maximum	SAMD5	0	-3.412468	
16746	Maximum	PDE4D	0	-3.398408	
16747	Maximum	FAM198B	0	-3.312024	
16748	Maximum	OAS1	0	-3.107858	

16749	Maximum	GPR125	0	-2.995118	
16750	Maximum	NAV3	0	-2.93174	
16751	Maximum	ANKRD29	0	-2.772202	
16752	Maximum	PLK2	0	-2.70096	
16753	Maximum	RORA	0	-2.564867	
16754	Maximum	SLC35D1	0	-2.486279	
16755	Maximum	MTHFD1L	0	-2.387688	
16756	Maximum	PDCD2	0	-2.293398	
16757	Maximum	ABHD6	0	-2.268189	
16758	Maximum	LOC100288413	0	-2.219809	
16759	Maximum	BMPR1B	0	-2.203381	
16760	Maximum	REPS1	0	-2.177362	
16761	Maximum	SATB2	0	-2.166841	
16762	Maximum	PTGR1	0	-2.15227	
16763	Maximum	PDK1	0	-2.121029	
16764	Maximum	KITLG	0	-2.115484	
16765	Maximum	PHLDA1	0	-2.110744	
16766	Maximum	LIMS1	0	-2.08165	
16767	Maximum	ELF1	0	-2.072611	
16768	Maximum	SPATA5	0	-2.069731	
16769	Maximum	LOC653602	0	-2.020459	
16770	Maximum	COMMD3	0	-2.020129	
16771	Maximum	E2F5	0	-2.002048	
16772	Maximum	STX7	0	2.022025	
16773	Maximum	MTMR11	0	2.039651	
16774	Maximum	EPHB3	0	2.044202	
16775	Maximum	TNS1	0	2.124447	
16776	Maximum	MUC20	0	2.146854	
16777	Maximum	MUC5AC	0	2.187337	
16778	Maximum	TMEM61	0	2.206764	
16779	Maximum	ENPP5	0	2.207396	
16780	Maximum	TPPP	0	2.273993	
16781	Maximum	FBXO8	0	2.462356	
16782	Maximum	ZMIZ1	0	2.502177	
16783	Maximum	TRPC1	0	2.522467	
16784	Maximum	FAM43A	0	2.526297	
16785	Maximum	ELF3	0	2.596663	
16786	Maximum	KLK10	0	2.882344	
16787	Maximum	KRT80	0	3.053349	
16788	Maximum	TM4SF1	0	3.334602	
16789	Maximum	MYLK	0	3.437058	
16790	Maximum	LOC146336	0	3.445174	
16791	Maximum	SERPINA1	0	3.687605	
16792	Maximum	MAP2K5	0	3.983236	
16793	Maximum	CYBRD1	0	4.140123	
16794	Maximum	CRYAB	0	5.835111	
16795	Maximum	AHNAK2	0	6.115869	
16796	Maximum	AQP3	0	13.694997	
16797	Maximum	HSPA1A	0	13.695802	
16798	Maximum	TCF7	0	14.43689	

16799	Melena	TM4SF4	0.009552	-5.804545	
16800	Melena	LCN15	0.001238	-5.790822	
16801	Melena	MTTP	0.002466	-4.174457	
16802	Melena	NOS1	0	-3.789628	
16803	Melena	SOX1	0	-3.509699	
16804	Melena	KHDC1L	0.001333	-3.422489	
16805	Melena	HGFAC	0.016996	-3.333027	
16806	Melena	WT1	0.000193	-3.279199	
16807	Melena	SLC34A2	0.01743	-3.262472	
16808	Melena	PROC	0.009352	-3.257868	
16809	Melena	APOC1	0.005004	-3.114316	
16810	Melena	C4orf49	0.000868	-3.090347	
16811	Melena	FLJ36840	0	-3.056013	
16812	Melena	KCNK15	0.000002	-3.050944	
16813	Melena	LONRF2	0.003702	-2.916848	
16814	Melena	PKLR	0.001163	-2.906511	
16815	Melena	CLU	0.002405	-2.894311	
16816	Melena	TKTL1	0.010266	-2.872553	
16817	Melena	MMP23A	0	-2.85111	
16818	Melena	ALDH3B2	0.000011	-2.817105	
16819	Melena	LOC100507193	0.000348	-2.800697	
16820	Melena	GALNT14	0.004525	-2.792721	
16821	Melena	BCHE	0.010633	-2.792177	
16822	Melena	CDH2	0.000405	-2.786761	
16823	Melena	RNF144B	0	-2.776336	
16824	Melena	CELF4	0.000615	-2.759974	
16825	Melena	FCGR2A	0.000005	-2.743996	
16826	Melena	HEATR7B1	0.00359	-2.732051	
16827	Melena	TMOD1	0.002559	-2.693288	
16828	Melena	SPP1	0.000482	-2.677758	
16829	Melena	CRABP2	0.006914	-2.675786	
16830	Melena	PLG	0.016473	-2.674303	
16831	Melena	HYDIN	0.000023	-2.655811	
16832	Melena	CDH4	0.003702	-2.643302	
16833	Melena	NBLA00301	0.002423	-2.619192	
16834	Melena	LOC100287547	0.015261	-2.616197	
16835	Melena	C3orf16	0.000447	-2.610502	
16836	Melena	HCG4	0.000047	-2.54578	
16837	Melena	TRIM55	0.000115	-2.537364	
16838	Melena	KCNN3	0.004525	-2.524265	
16839	Melena	L1CAM	0.000243	-2.519861	
16840	Melena	KCNC3	0	-2.491593	
16841	Melena	GH1	0.000039	-2.487859	
16842	Melena	DCDC2	0.001535	-2.487473	
16843	Melena	RUNX2	0.008775	-2.468869	
16844	Melena	MYOZ2	0.000967	-2.464938	
16845	Melena	FAM20A	0.012432	-2.448715	
16846	Melena	KCNMB1	0.000348	-2.447436	
16847	Melena	GBA	0.000027	-2.446266	
16848	Melena	C1orf61	0.000008	-2.41909	

16849	Melena	AGXT2	0.008935	-2.414805	
16850	Melena	ABCB4	0.001755	-2.399986	
16851	Melena	CYP2B7P1	0.00796	-2.390564	
16852	Melena	RPS6KA6	0.008891	-2.389067	
16853	Melena	CCND3	0.000069	-2.387478	
16854	Melena	SLC1A3	0.001982	-2.387428	
16855	Melena	C13orf30	0.003286	-2.384857	
16856	Melena	CXorf18	0.000084	-2.383912	
16857	Melena	C8orf46	0.013239	-2.379698	
16858	Melena	LOC338667	0.000001	-2.378794	
16859	Melena	SLC14A1	0.011801	-2.377959	
16860	Melena	PFKFB1	0.014286	-2.371519	
16861	Melena	MAF	0.010084	-2.369808	
16862	Melena	LOC100507531	0.002457	-2.367032	
16863	Melena	EMID2	0.000001	-2.36071	
16864	Melena	CLIP3	0.000808	-2.358243	
16865	Melena	PIK3R4	0.010003	-2.356814	
16866	Melena	TCEAL2	0.00014	-2.351044	
16867	Melena	FGF20	0.003286	-2.342792	
16868	Melena	CILP	0.011459	-2.341901	
16869	Melena	PDGFRL	0.007956	-2.337221	
16870	Melena	PIPOX	0.004068	-2.333892	
16871	Melena	LOC100510224	0.000045	-2.333025	
16872	Melena	CCDC8	0.000734	-2.329442	
16873	Melena	HABP2	0.013195	-2.326162	
16874	Melena	MAGEA12	0.011803	-2.325203	
16875	Melena	RIMS1	0.000929	-2.319664	
16876	Melena	IQCA1	0.0003	-2.308267	
16877	Melena	CCDC74B	0.000021	-2.300878	
16878	Melena	SIPA1L3	0.001219	-2.294633	
16879	Melena	DNAI2	0.000734	-2.293562	
16880	Melena	COL2A1	0.008515	-2.287112	
16881	Melena	VGLL1	0.004404	-2.283688	
16882	Melena	SLC22A2	0.000157	-2.278474	
16883	Melena	KAL1	0.000902	-2.260635	
16884	Melena	WDR86	0.013364	-2.260613	
16885	Melena	PQLC3	0.000243	-2.259458	
16886	Melena	RBP4	0.010449	-2.257376	
16887	Melena	PRSS54	0.000348	-2.249045	
16888	Melena	NCRNA00161	0.00372	-2.246095	
16889	Melena	BRSK2	0.000005	-2.245425	
16890	Melena	DPEP2	0.010022	-2.237906	
16891	Melena	ANO4	0.001501	-2.234775	
16892	Melena	C10orf112	0.002405	-2.230455	
16893	Melena	RIMS2	0.01025	-2.230412	
16894	Melena	TMEM132B	0.009214	-2.211613	
16895	Melena	FBXO17	0.000413	-2.201327	
16896	Melena	C3	0.003013	-2.200054	
16897	Melena	KCNE1	0.009581	-2.197229	
16898	Melena	SNAP25	0.003881	-2.18833	

16899	Melena	NCRNA00260	0.004093	-2.186592	
16900	Melena	GPM6B	0.005321	-2.184446	
16901	Melena	C4A	0.000394	-2.181772	
16902	Melena	OR2B3	0.014472	-2.170836	
16903	Melena	LOC644192	0.011201	-2.167257	
16904	Melena	APOE	0.004404	-2.165091	
16905	Melena	GTPBP1	0.000202	-2.16476	
16906	Melena	PSG2	0.002592	-2.161281	
16907	Melena	MAP2	0.013837	-2.159646	
16908	Melena	NRK	0.002737	-2.157668	
16909	Melena	OR10D1P	0.000005	-2.138024	
16910	Melena	TTC23	0.00015	-2.134911	
16911	Melena	PATE1	0.000134	-2.134676	
16912	Melena	KIAA1755	0.000047	-2.131913	
16913	Melena	GTSF1L	0.000325	-2.131019	
16914	Melena	TNNI2	0.013239	-2.123705	
16915	Melena	MTUS2	0.012319	-2.117955	
16916	Melena	PRSS37	0.001238	-2.117332	
16917	Melena	FAM124A	0.002405	-2.11708	
16918	Melena	PDLIM3	0.001392	-2.113238	
16919	Melena	GRIK2	0.014609	-2.111409	
16920	Melena	PGAM2	0.001509	-2.106355	
16921	Melena	CFC1	0.001962	-2.105086	
16922	Melena	LMOD1	0.016263	-2.092941	
16923	Melena	DGCR10	0.009464	-2.08544	
16924	Melena	BGN	0.001219	-2.081584	
16925	Melena	LOC646168	0.016516	-2.079598	
16926	Melena	LOC100131763	0.001535	-2.076241	
16927	Melena	FAM38B	0.000055	-2.069758	
16928	Melena	NLGN4Y	0.008541	-2.069476	
16929	Melena	CBLN1	0.006914	-2.066759	
16930	Melena	MGC4294	0.004068	-2.061471	
16931	Melena	CDK15	0.00835	-2.05795	
16932	Melena	PDE7B	0.003052	-2.054852	
16933	Melena	DPT	0.003976	-2.053275	
16934	Melena	TSPY1	0.004097	-2.051727	
16935	Melena	TMEFF2	0.003134	-2.050539	
16936	Melena	NTNG2	0.003286	-2.043375	
16937	Melena	GLRA2	0.004525	-2.042295	
16938	Melena	ZNF707	0.005738	-2.040481	
16939	Melena	KANK1	0.014937	-2.032252	
16940	Melena	PDZK1	0.012877	-2.027784	
16941	Melena	SLC47A1	0.013195	-2.027398	
16942	Melena	CBLC	0.011334	-2.023179	
16943	Melena	CES4A	0.002466	-2.023083	
16944	Melena	MTMR7	0.006014	-2.021831	
16945	Melena	CPEB1	0.000397	-2.020517	
16946	Melena	KIAA0226	0.011334	-2.015145	
16947	Melena	PNMAL1	0.001392	-2.012006	
16948	Melena	PLCXD3	0.00922	-2.009573	

16949	Melena	PEG3	0.007495	-2.006987	
16950	Melena	RGAG4	0.00835	-2.006358	
16951	Melena	LOC100507851	0.004068	-2.00194	
16952	Melena	BMP8A	0.000952	-2.001615	
16953	Metastatic Carcinoma	MMP3	0	-42.185084	
16954	Metastatic Carcinoma	ZG16	0	-26.512472	
16955	Metastatic Carcinoma	MS4A12	0	-20.652872	
16956	Metastatic Carcinoma	CEACAM7	0	-15.920289	
16957	Metastatic Carcinoma	CLCA4	0	-13.164673	
16958	Metastatic Carcinoma	PLIN1	0.001476	-11.891684	
16959	Metastatic Carcinoma	UGT2B17	0	-9.955452	
16960	Metastatic Carcinoma	FAM55D	0	-8.813167	
16961	Metastatic Carcinoma	LBP	0.018054	-8.475955	
16962	Metastatic Carcinoma	MMP1	0	-8.123524	
16963	Metastatic Carcinoma	ASCL2	0	-7.340432	
16964	Metastatic Carcinoma	LOC646627	0	-6.99836	
16965	Metastatic Carcinoma	CST1	0	-6.958478	
16966	Metastatic Carcinoma	CA4	0	-6.524388	
16967	Metastatic Carcinoma	CCL20	0	-6.503237	
16968	Metastatic Carcinoma	PLIN4	0.005948	-5.667384	
16969	Metastatic Carcinoma	ADIPOQ	0.000853	-5.642681	
16970	Metastatic Carcinoma	AQP8	0.00004	-5.63299	
16971	Metastatic Carcinoma	FABP4	0.000342	-5.49393	
16972	Metastatic Carcinoma	PCK1	0	-5.335896	
16973	Metastatic Carcinoma	NOX1	0.000064	-5.273299	
16974	Metastatic Carcinoma	IL8	0.000015	-5.161208	
16975	Metastatic Carcinoma	GUCA2B	0.000001	-5.066255	
16976	Metastatic Carcinoma	MXD1	0	-4.929587	
16977	Metastatic Carcinoma	CXCL3	0	-4.925059	
16978	Metastatic Carcinoma	IGHG1	0	-4.899017	
16979	Metastatic Carcinoma	GUCA2A	0	-4.357917	
16980	Metastatic Carcinoma	GPR109B	0.000139	-4.295004	
16981	Metastatic Carcinoma	MAGEA3	0.005832	-4.287409	
16982	Metastatic Carcinoma	ADH1C	0.022362	-4.285991	
16983	Metastatic Carcinoma	CA1	0.000034	-4.241507	
16984	Metastatic Carcinoma	AGT	0.000004	-4.12723	
16985	Metastatic Carcinoma	MUC12	0.021097	-4.08488	
16986	Metastatic Carcinoma	S100B	0.000019	-4.044414	
16987	Metastatic Carcinoma	SAA1	0.016341	-4.036917	
16988	Metastatic Carcinoma	C1orf125	0	-3.972715	
16989	Metastatic Carcinoma	ACSL6	0	-3.905292	
16990	Metastatic Carcinoma	C10orf99	0.001678	-3.813436	
16991	Metastatic Carcinoma	FLJ32063	0.000032	-3.807157	
16992	Metastatic Carcinoma	CDHR5	0	-3.724454	
16993	Metastatic Carcinoma	SLC26A3	0	-3.71925	
16994	Metastatic Carcinoma	MAGEA6	0.015537	-3.655734	
16995	Metastatic Carcinoma	MYH11	0.013957	-3.631599	
16996	Metastatic Carcinoma	DSG3	0.000027	-3.610608	
16997	Metastatic Carcinoma	CCL22	0	-3.597257	
16998	Metastatic Carcinoma	CXCL1	0.000015	-3.587934	

16999	Metastatic Carcinoma	MMP10	0	-3.529419	
17000	Metastatic Carcinoma	CFTR	0.000012	-3.492785	
17001	Metastatic Carcinoma	DHRS9	0.000036	-3.370458	
17002	Metastatic Carcinoma	CDX2	0.003894	-3.369222	
17003	Metastatic Carcinoma	UGT2B15	0.000036	-3.357359	
17004	Metastatic Carcinoma	EREG	0.002352	-3.339471	
17005	Metastatic Carcinoma	TNFSF11	0.000001	-3.337225	
17006	Metastatic Carcinoma	HSD11B2	0.00001	-3.326244	
17007	Metastatic Carcinoma	TDGF1	0.004942	-3.276759	
17008	Metastatic Carcinoma	PROK2	0.000099	-3.269483	
17009	Metastatic Carcinoma	SYNM	0.000201	-3.246326	
17010	Metastatic Carcinoma	FSTL5	0.020964	-3.212091	
17011	Metastatic Carcinoma	MEP1A	0.004731	-3.150256	
17012	Metastatic Carcinoma	ATP6V1C2	0.001572	-3.145924	
17013	Metastatic Carcinoma	PI3	0.006165	-3.116435	
17014	Metastatic Carcinoma	AREG	0.017169	-3.093726	
17015	Metastatic Carcinoma	GNG4	0.000001	-3.078896	
17016	Metastatic Carcinoma	IL1B	0	-3.074022	
17017	Metastatic Carcinoma	LGR5	0.007914	-3.072151	
17018	Metastatic Carcinoma	TRPM6	0	-3.067398	
17019	Metastatic Carcinoma	SLC13A3	0	-3.056251	
17020	Metastatic Carcinoma	TRIM31	0.001286	-2.947836	
17021	Metastatic Carcinoma	SELENBP1	0	-2.922294	
17022	Metastatic Carcinoma	PROX1	0.000393	-2.855246	
17023	Metastatic Carcinoma	IL11	0.000001	-2.854065	
17024	Metastatic Carcinoma	SPAG1	0.000027	-2.836507	
17025	Metastatic Carcinoma	SLC6A20	0.000145	-2.833047	
17026	Metastatic Carcinoma	SATB2	0.001401	-2.772726	
17027	Metastatic Carcinoma	LOC100288092	0.003541	-2.754075	
17028	Metastatic Carcinoma	DNASE1	0.018463	-2.708691	
17029	Metastatic Carcinoma	GPA33	0.008802	-2.693916	
17030	Metastatic Carcinoma	FAM55A	0	-2.69071	
17031	Metastatic Carcinoma	PKP1	0.018463	-2.684428	
17032	Metastatic Carcinoma	LOC100506860	0.009723	-2.674169	
17033	Metastatic Carcinoma	GPSM2	0.004614	-2.652971	
17034	Metastatic Carcinoma	CLEC4G	0.007414	-2.635787	
17035	Metastatic Carcinoma	CDHR1	0.000157	-2.634172	
17036	Metastatic Carcinoma	NAT8	0.00085	-2.634131	
17037	Metastatic Carcinoma	RNF43	0.000133	-2.617698	
17038	Metastatic Carcinoma	DNASE1L3	0.010674	-2.614105	
17039	Metastatic Carcinoma	ACE2	0.022362	-2.612884	
17040	Metastatic Carcinoma	CLC	0.000034	-2.607582	
17041	Metastatic Carcinoma	RHOH	0.000013	-2.604483	
17042	Metastatic Carcinoma	DDX17	0.000853	-2.584311	
17043	Metastatic Carcinoma	IGHA1	0.007222	-2.543227	
17044	Metastatic Carcinoma	CAPN10	0.006699	-2.536859	
17045	Metastatic Carcinoma	G0S2	0	-2.51811	
17046	Metastatic Carcinoma	PCNXL3	0.022937	-2.484637	
17047	Metastatic Carcinoma	NOTUM	0.009749	-2.478713	
17048	Metastatic Carcinoma	ARL14	0.005073	-2.478015	

17049	Metastatic Carcinoma	DDX3X	0.015482	-2.4725	
17050	Metastatic Carcinoma	NEGR1	0.016485	-2.439633	
17051	Metastatic Carcinoma	IL1RAPL1	0.002672	-2.439588	
17052	Metastatic Carcinoma	PHLDA1	0.00127	-2.438842	
17053	Metastatic Carcinoma	MYB	0.002422	-2.434565	
17054	Metastatic Carcinoma	IL1RN	0.000621	-2.426311	
17055	Metastatic Carcinoma	C10orf116	0.011016	-2.412165	
17056	Metastatic Carcinoma	ODAM	0.012053	-2.395621	
17057	Metastatic Carcinoma	CD177	0.00138	-2.374753	
17058	Metastatic Carcinoma	SLC26A2	0.006401	-2.370958	
17059	Metastatic Carcinoma	PRR15	0.008802	-2.358376	
17060	Metastatic Carcinoma	CST4	0.001635	-2.338289	
17061	Metastatic Carcinoma	PARM1	0.001697	-2.335607	
17062	Metastatic Carcinoma	NR4A1	0.002308	-2.309243	
17063	Metastatic Carcinoma	HBEGF	0.000648	-2.307207	
17064	Metastatic Carcinoma	SKP2	0.016341	-2.282677	
17065	Metastatic Carcinoma	LOC285628	0.021601	-2.274329	
17066	Metastatic Carcinoma	BEST2	0.01358	-2.264696	
17067	Metastatic Carcinoma	FGGY	0.000042	-2.261405	
17068	Metastatic Carcinoma	PKP2	0.007657	-2.259458	
17069	Metastatic Carcinoma	CFP	0.003705	-2.257573	
17070	Metastatic Carcinoma	HR	0.005879	-2.248453	
17071	Metastatic Carcinoma	HSPH1	0.010344	-2.215763	
17072	Metastatic Carcinoma	PI15	0.000818	-2.215594	
17073	Metastatic Carcinoma	RBM33	0.006957	-2.211871	
17074	Metastatic Carcinoma	IL21R	0.021392	-2.207299	
17075	Metastatic Carcinoma	BCL2L15	0.002197	-2.193376	
17076	Metastatic Carcinoma	CLIC5	0.000024	-2.182391	
17077	Metastatic Carcinoma	GRIK4	0.00094	-2.170083	
17078	Metastatic Carcinoma	LOC25845	0.020904	-2.163282	
17079	Metastatic Carcinoma	LRRC2	0.022352	-2.156873	
17080	Metastatic Carcinoma	MIER3	0.000161	-2.148694	
17081	Metastatic Carcinoma	KLF5	0.014547	-2.147231	
17082	Metastatic Carcinoma	ZFP36L2	0.00085	-2.142224	
17083	Metastatic Carcinoma	ABCB11	0.00368	-2.137852	
17084	Metastatic Carcinoma	IL24	0.008149	-2.137242	
17085	Metastatic Carcinoma	ACVRL1	0.001808	-2.130668	
17086	Metastatic Carcinoma	IL33	0.001808	-2.125832	
17087	Metastatic Carcinoma	CST2	0.019868	-2.12506	
17088	Metastatic Carcinoma	FUT4	0.020291	-2.122425	
17089	Metastatic Carcinoma	CYP4F3	0.011991	-2.122185	
17090	Metastatic Carcinoma	NEK3	0.003129	-2.116361	
17091	Metastatic Carcinoma	SCML1	0.000431	-2.110679	
17092	Metastatic Carcinoma	MYOCD	0.022717	-2.105389	
17093	Metastatic Carcinoma	HEPACAM	0.009626	-2.103795	
17094	Metastatic Carcinoma	LCK	0.003276	-2.09352	
17095	Metastatic Carcinoma	GPAM	0.001236	-2.092237	
17096	Metastatic Carcinoma	ENPP3	0.012155	-2.088685	
17097	Metastatic Carcinoma	ACSL5	0.008439	-2.087155	
17098	Metastatic Carcinoma	GTF2F2	0.000139	-2.082201	

17099	Metastatic Carcinoma	MCOLN2	0.00085	-2.079015	
17100	Metastatic Carcinoma	UCHL3	0.000086	-2.073443	
17101	Metastatic Carcinoma	LOC284801	0.018054	-2.069469	
17102	Metastatic Carcinoma	FOXP2	0.012017	-2.067579	
17103	Metastatic Carcinoma	PDE4B	0.000007	-2.04567	
17104	Metastatic Carcinoma	DGAT2	0.011239	-2.042118	
17105	Metastatic Carcinoma	STAMBPL1	0.004161	-2.038423	
17106	Metastatic Carcinoma	BMP2	0.001116	-2.036493	
17107	Metastatic Carcinoma	SLMO2	0.000055	-2.012657	
17108	Metastatic Carcinoma	FAR2	0.00951	-2.003805	
17109	Methionine	ITLN1	0	-292.109149	
17110	Methionine	C6orf105	0.000006	-104.893373	
17111	Methionine	CA2	0.000001	-88.178809	
17112	Methionine	MS4A12	0.000002	-16.670782	
17113	Methionine	IGJ	0.000004	-11.651369	
17114	Methionine	FRMD3	0.000006	-6.633007	
17115	Methionine	GSR	0.000011	-5.729375	
17116	Methionine	CA4	0.000001	-5.632541	
17117	Methionine	NTN2L	0.000006	-5.322837	
17118	Methionine	HLA-C	0.000011	-5.090134	
17119	Methionine	GUCA2A	0.000002	-4.858079	
17120	Methionine	SPINK4	0.000001	-3.238074	
17121	Methionine	PIGR	0	-2.867408	
17122	Methionine	SPARCL1	0.000018	-2.801931	
17123	Methionine	FCGBP	0	-2.719633	
17124	Methionine	IGHA1	0.000001	-2.6695	
17125	Methionine	ELOVL6	0	-2.453167	
17126	Methionine	KIAA0828	0	-2.389916	
17127	Methionine	HMGCR	0.000015	-2.289966	
17128	Methionine	FAM46C	0.000005	-2.22057	
17129	Methionine	CCDC93	0	2.078317	
17130	Methionine	KLF7	0.000006	2.092401	
17131	Methionine	TMEM49	0	2.095377	
17132	Methionine	USP34	0.000005	2.109417	
17133	Methionine	SERINC5	0.000004	2.188872	
17134	Methionine	EML4	0	2.191544	
17135	Methionine	ARHGEF2	0.000016	2.197076	
17136	Methionine	MPHOSPH8	0.000004	2.214199	
17137	Methionine	NNMT	0.000001	2.298865	
17138	Methionine	DGKH	0.000001	2.302271	
17139	Methionine	LOC727820	0	2.307956	
17140	Methionine	MGC16384	0.000006	2.346782	
17141	Methionine	COL1A1	0	2.395517	
17142	Methionine	CSNK1A1	0.000004	2.40641	
17143	Methionine	NRBP2	0	2.409324	
17144	Methionine	CLK1	0.000007	2.490114	
17145	Methionine	SFRS18	0	2.509521	
17146	Methionine	EIF3B	0.000014	2.53144	
17147	Methionine	NSFL1C	0.000011	2.537637	
17148	Methionine	C4orf30	0.000017	2.621492	

17149	Methionine	FOXJ3	0.000019	2.674279	
17150	Methionine	RP11-298P3.3	0.000001	2.733297	
17151	Methionine	AKAP8L	0.000016	2.772168	
17152	Methionine	FNBP4	0	2.773925	
17153	Methionine	ZNF83	0.000021	2.8262	
17154	Methionine	VPS13C	0.000001	2.860054	
17155	Methionine	LOC26010	0.000013	2.919209	
17156	Methionine	YAP1	0.000001	3.066516	
17157	Methionine	ZNF207	0.000004	3.084116	
17158	Methionine	C7orf54	0.000012	3.097033	
17159	Methionine	ZNF364	0.000009	3.107203	
17160	Methionine	RBM39	0.000001	3.303166	
17161	Methionine	MGC23985	0.000004	3.382113	
17162	Methionine	U2AF1	0.000002	3.513583	
17163	Methionine	CHD2	0.00001	3.596148	
17164	Methionine	THBS2	0	3.628814	
17165	Methionine	SYK	0.000006	3.751209	
17166	Methionine	GTF3A	0.000012	3.956153	
17167	Methionine	TBRG1	0	4.005367	
17168	Methionine	SNAPC3	0.000002	4.188509	
17169	Methionine	CCNL1	0.000021	4.215768	
17170	Methionine	C11orf58	0.000007	4.378468	
17171	Methionine	SMURF2	0.000012	4.524036	
17172	Methionine	GLUD1	0.000014	4.578576	
17173	Methionine	ZNF785	0.000008	4.674523	
17174	Methionine	IFNGR1	0	4.786103	
17175	Methionine	PCF11	0.000007	4.990015	
17176	Methionine	PICALM	0.000013	5.138198	
17177	Methionine	KIAA0907	0.000007	5.62551	
17178	Methionine	SPP1	0	5.857485	
17179	Methionine	ZNF638	0	5.912399	
17180	Methionine	LOC440354	0.000014	6.198988	
17181	Methionine	ZMYND8	0.000009	6.92901	
17182	Methionine	MBNL2	0	7.194229	
17183	Methionine	C10orf10	0.000003	7.305578	
17184	Methionine	MEGF6	0.000022	7.507018	
17185	Methionine	KIAA0368	0	9.302099	
17186	Methionine	XPO1	0.000002	10.357944	
17187	Methionine	CFLAR	0.000011	11.328732	
17188	Methionine	GART	0.000001	15.363893	
17189	Methionine	ZBTB20	0.000004	17.367361	
17190	Methionine	ZFAND6	0.000004	17.449033	
17191	Methionine	PIAS1	0.000009	18.056731	
17192	Methionine	ASXL1	0	20.752404	
17193	Methionine	UBC	0.000009	23.283036	
17194	Methionine	SLC22A3	0	141.230932	
17195	Methionine	PHKB	0	201.69213	
17196	Microsatellite Instability	SLC26A3	0.000001	-39.432581	
17197	Microsatellite Instability	GUCA2A	0	-16.294008	
17198	Microsatellite Instability	KRT23	0.031055	-13.498961	

17199	Microsatellite Instability	MYH11	0.000001	-12.464317	
17200	Microsatellite Instability	CHRM3	0.038848	-5.36188	
17201	Microsatellite Instability	AMACR	0.013025	-3.921634	
17202	Microsatellite Instability	DST	0	-3.84509	
17203	Microsatellite Instability	SEMA5A	0.031272	-3.759141	
17204	Microsatellite Instability	MUC20	0.015636	-3.733484	
17205	Microsatellite Instability	TOB1	0.034358	-3.662296	
17206	Microsatellite Instability	AMT	0.038194	-3.50289	
17207	Microsatellite Instability	MGLL	0	-3.330854	
17208	Microsatellite Instability	SHROOM4	0.012577	-3.132474	
17209	Microsatellite Instability	PLAGL2	0.049045	-3.10742	
17210	Microsatellite Instability	SPIRE2	0.04666	-2.907811	
17211	Microsatellite Instability	NUDT7	0.038848	-2.789623	
17212	Microsatellite Instability	AXIN2	0.034678	-2.724455	
17213	Microsatellite Instability	DHRS12	0.044875	-2.469173	
17214	Microsatellite Instability	KCNE3	0.040552	-2.459474	
17215	Microsatellite Instability	LOC100292959	0.037228	-2.424323	
17216	Microsatellite Instability	MBTD1	0.007661	-2.387456	
17217	Microsatellite Instability	C10orf47	0.04666	-2.365174	
17218	Microsatellite Instability	SYNJ2	0.004314	-2.171962	
17219	Microsatellite Instability	LPAR1	0	-2.101231	
17220	Microsatellite Instability	RAP2A	0.004876	-2.069568	
17221	Microsatellite Instability	CAMKK2	0.004876	-2.058886	
17222	Microsatellite Instability	TXNDC9	0.034502	-2.032892	
17223	Microsatellite Instability	CBX3	0.000002	2.001406	
17224	Microsatellite Instability	LONP1	0	2.008715	
17225	Microsatellite Instability	MTA2	0.000001	2.010076	
17226	Microsatellite Instability	KIF15	0	2.015372	
17227	Microsatellite Instability	GOLT1B	0	2.015554	
17228	Microsatellite Instability	RIPK2	0	2.016821	
17229	Microsatellite Instability	MTERFD1	0.000001	2.018794	
17230	Microsatellite Instability	KIF11	0.000001	2.025286	
17231	Microsatellite Instability	NUP107	0	2.028406	
17232	Microsatellite Instability	CCDC59	0	2.030633	
17233	Microsatellite Instability	SET	0	2.042096	
17234	Microsatellite Instability	TIMELESS	0	2.051896	
17235	Microsatellite Instability	OIP5	0.000001	2.065998	
17236	Microsatellite Instability	RUVBL1	0	2.069813	
17237	Microsatellite Instability	NUP205	0.000001	2.074642	
17238	Microsatellite Instability	STT3A	0	2.077468	
17239	Microsatellite Instability	ENO1	0.000002	2.078688	
17240	Microsatellite Instability	RUVBL2	0	2.080554	
17241	Microsatellite Instability	SMCHD1	0.016022	2.088014	
17242	Microsatellite Instability	NUCB2	0.038592	2.090244	
17243	Microsatellite Instability	RFC5	0.000001	2.096438	
17244	Microsatellite Instability	EPPK1	0.000001	2.101393	
17245	Microsatellite Instability	SLC25A32	0.000001	2.113102	
17246	Microsatellite Instability	GINS2	0.000001	2.11675	
17247	Microsatellite Instability	NUP85	0.000001	2.11777	
17248	Microsatellite Instability	RBM28	0	2.120363	

17249	Microsatellite Instability	NCAPG2	0	2.125408	
17250	Microsatellite Instability	CAD	0.000001	2.127343	
17251	Microsatellite Instability	GTF2IRD1	0	2.133547	
17252	Microsatellite Instability	FANCL	0	2.139324	
17253	Microsatellite Instability	GZMB	0	2.14732	
17254	Microsatellite Instability	KCNRG	0.031716	2.151451	
17255	Microsatellite Instability	KIF2C	0.000001	2.154208	
17256	Microsatellite Instability	OAS3	0.000001	2.155018	
17257	Microsatellite Instability	FANCG	0	2.166676	
17258	Microsatellite Instability	CCL4	0	2.174968	
17259	Microsatellite Instability	RAB15	0	2.188293	
17260	Microsatellite Instability	JAG2	0.000001	2.191192	
17261	Microsatellite Instability	RPL22L1	0.04666	2.194733	
17262	Microsatellite Instability	WDR43	0.000001	2.194866	
17263	Microsatellite Instability	POLR1D	0.000001	2.252838	
17264	Microsatellite Instability	NUP37	0.000001	2.262069	
17265	Microsatellite Instability	FUT8	0	2.267452	
17266	Microsatellite Instability	PITX1	0.000001	2.26755	
17267	Microsatellite Instability	KIF20A	0	2.295043	
17268	Microsatellite Instability	MICB	0	2.307009	
17269	Microsatellite Instability	HS2ST1	0.000002	2.328743	
17270	Microsatellite Instability	CBFB	0	2.334836	
17271	Microsatellite Instability	CDC42EP1	0.000001	2.339437	
17272	Microsatellite Instability	SORD	0.000001	2.341712	
17273	Microsatellite Instability	MRPS17	0.000001	2.391333	
17274	Microsatellite Instability	NOP56	0.000001	2.40229	
17275	Microsatellite Instability	ZWILCH	0	2.403617	
17276	Microsatellite Instability	ARNTL2	0	2.425129	
17277	Microsatellite Instability	PSAT1	0.000001	2.45864	
17278	Microsatellite Instability	NFE2L3	0	2.463088	
17279	Microsatellite Instability	CSE1L	0.000001	2.467005	
17280	Microsatellite Instability	C12orf11	0	2.477935	
17281	Microsatellite Instability	VARS	0	2.490948	
17282	Microsatellite Instability	ICAM1	0	2.498583	
17283	Microsatellite Instability	MCM7	0	2.562626	
17284	Microsatellite Instability	UBE2L6	0.000001	2.570384	
17285	Microsatellite Instability	PSMG1	0.000001	2.572526	
17286	Microsatellite Instability	CDC6	0	2.585624	
17287	Microsatellite Instability	AHCY	0.000002	2.587594	
17288	Microsatellite Instability	CCNA2	0.000001	2.6516	
17289	Microsatellite Instability	BOP1	0	2.693284	
17290	Microsatellite Instability	GBP1	0	2.698104	
17291	Microsatellite Instability	CDC25B	0.000001	2.70041	
17292	Microsatellite Instability	C7orf68	0.000001	2.713761	
17293	Microsatellite Instability	RFC3	0	2.720527	
17294	Microsatellite Instability	EIF5A	0	2.754041	
17295	Microsatellite Instability	FEN1	0.000001	2.754245	
17296	Microsatellite Instability	TEAD4	0.000001	2.833619	
17297	Microsatellite Instability	LGR5	0.000001	2.855969	
17298	Microsatellite Instability	LMNB2	0	2.858719	

17299	Microsatellite Instability	NT5DC2	0	2.876001	
17300	Microsatellite Instability	HNRNPL	0	2.905758	
17301	Microsatellite Instability	SLC7A5	0.000001	2.932664	
17302	Microsatellite Instability	SHMT2	0	2.982462	
17303	Microsatellite Instability	KIF4A	0.000001	3.052197	
17304	Microsatellite Instability	GINS1	0	3.07162	
17305	Microsatellite Instability	F12	0	3.082553	
17306	Microsatellite Instability	STAT1	0	3.107809	
17307	Microsatellite Instability	TRIP13	0	3.129853	
17308	Microsatellite Instability	ASPHD2	0.021281	3.136229	
17309	Microsatellite Instability	NUTF2	0	3.241433	
17310	Microsatellite Instability	CEP55	0	3.248565	
17311	Microsatellite Instability	SOD2	0	3.254564	
17312	Microsatellite Instability	FOXM1	0	3.25519	
17313	Microsatellite Instability	MCM4	0	3.415645	
17314	Microsatellite Instability	TACSTD2	0	3.446898	
17315	Microsatellite Instability	MIF	0.000001	3.504093	
17316	Microsatellite Instability	PBK	0.000001	3.714962	
17317	Microsatellite Instability	ECT2	0	3.796085	
17318	Microsatellite Instability	TMEM97	0	3.826889	
17319	Microsatellite Instability	PMAIP1	0	4.0525	
17320	Microsatellite Instability	CXCL9	0	4.116745	
17321	Microsatellite Instability	IFITM1	0	4.28338	
17322	Microsatellite Instability	CXCL10	0	4.8737	
17323	Microsatellite Instability	CXCL1	0	5.226033	
17324	Microsatellite Instability	SRSF6	0.000001	6.156758	
17325	Microsatellite Instability	MMP12	0	6.617377	
17326	Microsatellite Instability	CXCL3	0	7.233392	
17327	Microsatellite Instability	SPP1	0	7.465531	
17328	Microsatellite Repeat	SLC26A3	0.000001	-39.432581	
17329	Microsatellite Repeat	GUCA2A	0	-16.294008	
17330	Microsatellite Repeat	MYH11	0.000001	-12.464317	
17331	Microsatellite Repeat	DST	0	-3.84509	
17332	Microsatellite Repeat	MGLL	0	-3.330854	
17333	Microsatellite Repeat	LPAR1	0	-2.101231	
17334	Microsatellite Repeat	CBX3	0.000002	2.001406	
17335	Microsatellite Repeat	LONP1	0	2.008715	
17336	Microsatellite Repeat	MTA2	0.000001	2.010076	
17337	Microsatellite Repeat	KIF15	0	2.015372	
17338	Microsatellite Repeat	GOLT1B	0	2.015554	
17339	Microsatellite Repeat	RIPK2	0	2.016821	
17340	Microsatellite Repeat	MTERFD1	0.000001	2.018794	
17341	Microsatellite Repeat	KIF11	0.000001	2.025286	
17342	Microsatellite Repeat	NUP107	0	2.028406	
17343	Microsatellite Repeat	CCDC59	0	2.030633	
17344	Microsatellite Repeat	SET	0	2.042096	
17345	Microsatellite Repeat	TIMELESS	0	2.051896	
17346	Microsatellite Repeat	OIP5	0.000001	2.065998	
17347	Microsatellite Repeat	RUVBL1	0	2.069813	
17348	Microsatellite Repeat	NUP205	0.000001	2.074642	

17349	Microsatellite Repeat	STT3A	0	2.077468	
17350	Microsatellite Repeat	ENO1	0.000002	2.078688	
17351	Microsatellite Repeat	RUVBL2	0	2.080554	
17352	Microsatellite Repeat	RFC5	0.000001	2.096438	
17353	Microsatellite Repeat	EPPK1	0.000001	2.101393	
17354	Microsatellite Repeat	SLC25A32	0.000001	2.113102	
17355	Microsatellite Repeat	GINS2	0.000001	2.11675	
17356	Microsatellite Repeat	NUP85	0.000001	2.11777	
17357	Microsatellite Repeat	RBM28	0	2.120363	
17358	Microsatellite Repeat	NCAPG2	0	2.125408	
17359	Microsatellite Repeat	CAD	0.000001	2.127343	
17360	Microsatellite Repeat	GTF2IRD1	0	2.133547	
17361	Microsatellite Repeat	FANCL	0	2.139324	
17362	Microsatellite Repeat	GZMB	0	2.14732	
17363	Microsatellite Repeat	KIF2C	0.000001	2.154208	
17364	Microsatellite Repeat	OAS3	0.000001	2.155018	
17365	Microsatellite Repeat	FANCG	0	2.166676	
17366	Microsatellite Repeat	CCL4	0	2.174968	
17367	Microsatellite Repeat	RAB15	0	2.188293	
17368	Microsatellite Repeat	JAG2	0.000001	2.191192	
17369	Microsatellite Repeat	WDR43	0.000001	2.194866	
17370	Microsatellite Repeat	POLR1D	0.000001	2.252838	
17371	Microsatellite Repeat	NUP37	0.000001	2.262069	
17372	Microsatellite Repeat	FUT8	0	2.267452	
17373	Microsatellite Repeat	PITX1	0.000001	2.26755	
17374	Microsatellite Repeat	KIF20A	0	2.295043	
17375	Microsatellite Repeat	MICB	0	2.307009	
17376	Microsatellite Repeat	HS2ST1	0.000002	2.328743	
17377	Microsatellite Repeat	CBFB	0	2.334836	
17378	Microsatellite Repeat	CDC42EP1	0.000001	2.339437	
17379	Microsatellite Repeat	SORD	0.000001	2.341712	
17380	Microsatellite Repeat	MRPS17	0.000001	2.391333	
17381	Microsatellite Repeat	NOP56	0.000001	2.40229	
17382	Microsatellite Repeat	ZWILCH	0	2.403617	
17383	Microsatellite Repeat	ARNTL2	0	2.425129	
17384	Microsatellite Repeat	PSAT1	0.000001	2.45864	
17385	Microsatellite Repeat	NFE2L3	0	2.463088	
17386	Microsatellite Repeat	CSE1L	0.000001	2.467005	
17387	Microsatellite Repeat	C12orf11	0	2.477935	
17388	Microsatellite Repeat	VARS	0	2.490948	
17389	Microsatellite Repeat	ICAM1	0	2.498583	
17390	Microsatellite Repeat	MCM7	0	2.562626	
17391	Microsatellite Repeat	UBE2L6	0.000001	2.570384	
17392	Microsatellite Repeat	PSMG1	0.000001	2.572526	
17393	Microsatellite Repeat	CDC6	0	2.585624	
17394	Microsatellite Repeat	AHCY	0.000002	2.587594	
17395	Microsatellite Repeat	CCNA2	0.000001	2.6516	
17396	Microsatellite Repeat	BOP1	0	2.693284	
17397	Microsatellite Repeat	GBP1	0	2.698104	
17398	Microsatellite Repeat	CDC25B	0.000001	2.70041	

17399	Microsatellite Repeat	C7orf68	0.000001	2.713761	
17400	Microsatellite Repeat	RFC3	0	2.720527	
17401	Microsatellite Repeat	EIF5A	0	2.754041	
17402	Microsatellite Repeat	FEN1	0.000001	2.754245	
17403	Microsatellite Repeat	TEAD4	0.000001	2.833619	
17404	Microsatellite Repeat	LGR5	0.000001	2.855969	
17405	Microsatellite Repeat	LMNB2	0	2.858719	
17406	Microsatellite Repeat	NT5DC2	0	2.876001	
17407	Microsatellite Repeat	HNRNPL	0	2.905758	
17408	Microsatellite Repeat	SLC7A5	0.000001	2.932664	
17409	Microsatellite Repeat	SHMT2	0	2.982462	
17410	Microsatellite Repeat	KIF4A	0.000001	3.052197	
17411	Microsatellite Repeat	GINS1	0	3.07162	
17412	Microsatellite Repeat	F12	0	3.082553	
17413	Microsatellite Repeat	STAT1	0	3.107809	
17414	Microsatellite Repeat	TRIP13	0	3.129853	
17415	Microsatellite Repeat	NUTF2	0	3.241433	
17416	Microsatellite Repeat	CEP55	0	3.248565	
17417	Microsatellite Repeat	SOD2	0	3.254564	
17418	Microsatellite Repeat	FOXM1	0	3.25519	
17419	Microsatellite Repeat	MCM4	0	3.415645	
17420	Microsatellite Repeat	TACSTD2	0	3.446898	
17421	Microsatellite Repeat	MIF	0.000001	3.504093	
17422	Microsatellite Repeat	PBK	0.000001	3.714962	
17423	Microsatellite Repeat	ECT2	0	3.796085	
17424	Microsatellite Repeat	TMEM97	0	3.826889	
17425	Microsatellite Repeat	PMAIP1	0	4.0525	
17426	Microsatellite Repeat	CXCL9	0	4.116745	
17427	Microsatellite Repeat	IFITM1	0	4.28338	
17428	Microsatellite Repeat	CXCL10	0	4.8737	
17429	Microsatellite Repeat	CXCL1	0	5.226033	
17430	Microsatellite Repeat	SRSF6	0.000001	6.156758	
17431	Microsatellite Repeat	MMP12	0	6.617377	
17432	Microsatellite Repeat	CXCL3	0	7.233392	
17433	Microsatellite Repeat	SPP1	0	7.465531	
17434	Mild asthma	XIST	0	1.981451	
17435	monoclonal antibody CAL	COL3A1	0.000526	-3.226991	
17436	monoclonal antibody CAL	AFF4	0	-2.612971	
17437	monoclonal antibody CAL	C7orf44	0.000036	-2.449435	
17438	monoclonal antibody CAL	JMJD1C	0.000019	-2.224513	
17439	monoclonal antibody CAL	TMEM43	0.000012	-2.17963	
17440	monoclonal antibody CAL	PLD1	0.000374	-2.179092	
17441	monoclonal antibody CAL	TRAPPC10	0.000044	-2.17602	
17442	monoclonal antibody CAL	GSPT1	0.000206	-2.157966	
17443	monoclonal antibody CAL	VPS13C	0.000036	-2.152	
17444	monoclonal antibody CAL	MGEA5	0.000008	-2.134887	
17445	monoclonal antibody CAL	PELI1	0.000503	-2.078611	
17446	monoclonal antibody CAL	RNF213	0.001452	-2.057288	
17447	monoclonal antibody CAL	DNAJC7	0.000133	-2.055506	
17448	monoclonal antibody CAL	ZNF638	0.000158	-2.053607	

17449	monoclonal antibody CAL	RUFY2	0.000101	-2.049441	
17450	monoclonal antibody CAL	LPP	0.000015	-2.030402	
17451	monoclonal antibody CAL	PIK3C2A	0.000966	-2.020127	
17452	monoclonal antibody CAL	GTF2I	0.000121	2.003362	
17453	monoclonal antibody CAL	SH3D19	0.000015	2.019181	
17454	monoclonal antibody CAL	ERBB2	0.000861	2.020716	
17455	monoclonal antibody CAL	HSF1	0.000038	2.023021	
17456	monoclonal antibody CAL	MET	0.000133	2.025802	
17457	monoclonal antibody CAL	BCL2L1	0.000301	2.026625	
17458	monoclonal antibody CAL	SUPT16H	0.000865	2.05419	
17459	monoclonal antibody CAL	NFS1	0.000133	2.054213	
17460	monoclonal antibody CAL	LASS6	0.000247	2.093112	
17461	monoclonal antibody CAL	PRPF6	0.000002	2.160814	
17462	monoclonal antibody CAL	ST6GAL1	0.001722	2.345209	
17463	monoclonal antibody CAL	REPIN1	0.000101	2.415475	
17464	monoclonal antibody CAL	COPA	0.000015	2.579514	
17465	Multiple tumors	COPG	0	-1.774521	
17466	Multiple tumors	LOC158257	0	1.8571	
17467	Mutant	ARHGAP29	0.000004	-574.399087	
17468	Mutant	DKK1	0	-561.358383	
17469	Mutant	GTSF1	0.000008	-399.301718	
17470	Mutant	PCDH7	0.000001	-387.281575	
17471	Mutant	ALDH1A3	0.000006	-365.306613	
17472	Mutant	TUBA1A	0	-362.306283	
17473	Mutant	MAGEB2	0	-317.484128	
17474	Mutant	MAGEA3	0	-286.243733	
17475	Mutant	GNAI1	0	-249.518455	
17476	Mutant	CALD1	0.000002	-236.177902	
17477	Mutant	MAGEA2	0.000003	-195.042669	
17478	Mutant	RDX	0.000002	-174.326299	
17479	Mutant	HLTF	0.000003	-143.882085	
17480	Mutant	WBP5	0.000004	-121.981655	
17481	Mutant	GPR110	0.00001	-119.33262	
17482	Mutant	SCG2	0.000007	-97.219518	
17483	Mutant	HBE1	0.000005	-95.940684	
17484	Mutant	LOC399959	0.000008	-91.493939	
17485	Mutant	ENAH	0.000001	-87.225685	
17486	Mutant	CD55	0.000008	-85.609602	
17487	Mutant	PDE4B	0.000008	-81.362804	
17488	Mutant	TMEM200A	0.000008	-79.055071	
17489	Mutant	NPW	0.000004	-75.322469	
17490	Mutant	WNT16	0.000003	-72.070133	
17491	Mutant	PVRL3	0.000006	-68.00041	
17492	Mutant	PDP1	0	-64.485389	
17493	Mutant	PLA2G4A	0.000004	-63.232318	
17494	Mutant	GLUL	0.000003	-60.819892	
17495	Mutant	SELM	0.000003	-60.753264	
17496	Mutant	FMNL2	0	-54.602674	
17497	Mutant	ANTXR1	0.000001	-49.218279	
17498	Mutant	DSE	0.000008	-44.952396	

17499	Mutant	PHLDB2	0.000003	-44.745438	
17500	Mutant	TBC1D9	0.000002	-41.505718	
17501	Mutant	ANXA10	0.000003	-40.771601	
17502	Mutant	FAM171B	0.000005	-38.639504	
17503	Mutant	FAM92A1	0.000002	-34.853387	
17504	Mutant	MEF2C	0	-33.319674	
17505	Mutant	COL12A1	0.000005	-31.924422	
17506	Mutant	NR3C1	0.000003	-31.321162	
17507	Mutant	UBE2E2	0.000004	-29.738673	
17508	Mutant	RAB12	0.000004	-27.617611	
17509	Mutant	SMARCA1	0.000004	-27.270593	
17510	Mutant	INPP5D	0.007407	-27.074795	
17511	Mutant	EPB41L2	0.000011	-26.226904	
17512	Mutant	RRAGD	0.000011	-25.191409	
17513	Mutant	GPAT2	0.000007	-25.125105	
17514	Mutant	MAGEA12	0.000007	-24.113279	
17515	Mutant	ZNF385B	0.000001	-23.716727	
17516	Mutant	RUNDC3B	0.000012	-22.999973	
17517	Mutant	FERMT2	0.000004	-22.008593	
17518	Mutant	NOV	0.000004	-21.833935	
17519	Mutant	SFTPB	0.002748	-19.68976	
17520	Mutant	IFITM3	0.000002	-19.187446	
17521	Mutant	PPP1R9A	0.000008	-19.038244	
17522	Mutant	SSFA2	0.000004	-18.763377	
17523	Mutant	SPIRE1	0.000001	-18.354058	
17524	Mutant	SMAD4	0.000004	-18.303144	
17525	Mutant	RBM24	0.000003	-17.565277	
17526	Mutant	NCRNA00173	0.000006	-16.974223	
17527	Mutant	CSAG2	0.000003	-16.47408	
17528	Mutant	KLHL13	0.000003	-15.91222	
17529	Mutant	B3GALNT1	0.000003	-15.014644	
17530	Mutant	GABBR1	0.040359	-14.781427	
17531	Mutant	OGFRL1	0.00001	-14.609415	
17532	Mutant	SERPINB9	0.000001	-14.452624	
17533	Mutant	MT1X	0.000004	-13.99134	
17534	Mutant	CFL2	0.000008	-13.734276	
17535	Mutant	MT2A	0.000012	-13.177214	
17536	Mutant	SYTL3	0.000004	-12.586998	
17537	Mutant	SIX4	0.000011	-12.215531	
17538	Mutant	C8orf47	0.000005	-12.045282	
17539	Mutant	LAPTM4B	0	-12.015325	
17540	Mutant	MFAP2	0.000003	-11.296665	
17541	Mutant	NFIC	0.000009	-10.751284	
17542	Mutant	BDNF	0.000006	-10.742728	
17543	Mutant	C9orf72	0.000004	-10.188588	
17544	Mutant	F2RL1	0.000003	-9.207135	
17545	Mutant	CFD	0.000004	-9.061576	
17546	Mutant	NME4	0.000007	-9.047245	
17547	Mutant	TIMP2	0.000006	-8.705022	
17548	Mutant	PALLD	0.000005	-8.486156	

17549	Mutant	UGT8	0	-7.983426	
17550	Mutant	PITPNC1	0.00001	-7.945857	
17551	Mutant	LOC643792	0.000011	-7.85897	
17552	Mutant	MYL9	0.000004	-7.687039	
17553	Mutant	RHOQ	0.000001	-7.383621	
17554	Mutant	EMP3	0.000002	-7.129033	
17555	Mutant	RHBDL2	0.040359	-7.122631	
17556	Mutant	CLDND1	0.000003	-7.055015	
17557	Mutant	NAV3	0.000008	-7.051791	
17558	Mutant	DPYD	0.000008	-6.845269	
17559	Mutant	NEO1	0.000007	-6.778429	
17560	Mutant	AP1S3	0.000004	-6.665484	
17561	Mutant	KLC3	0.000003	-6.402367	
17562	Mutant	MAP1B	0.000008	-6.150958	
17563	Mutant	COL6A2	0.000005	-6.07294	
17564	Mutant	NCS1	0.000008	-5.978362	
17565	Mutant	MLF1	0.00001	-5.942251	
17566	Mutant	FAM164A	0.000005	-5.904321	
17567	Mutant	TMEM47	0	-5.826227	
17568	Mutant	PCP4	0	-5.673615	
17569	Mutant	ASPHD1	0.00001	-5.58353	
17570	Mutant	PCDHA1	0.00001	-5.429812	
17571	Mutant	RNF141	0.000002	-5.403097	
17572	Mutant	BEND6	0.000005	-5.306019	
17573	Mutant	SMURF2	0.000008	-5.287704	
17574	Mutant	CCNG2	0.00001	-5.12713	
17575	Mutant	SOHLH2	0	-5.113841	
17576	Mutant	FZD3	0.000009	-5.022363	
17577	Mutant	ABCB1	0.000001	-4.984489	
17578	Mutant	CRTAM	0.000002	-4.836897	
17579	Mutant	RHOF	0.000006	-4.754852	
17580	Mutant	ARAP2	0	-4.612948	
17581	Mutant	EZR	0.000008	-4.57178	
17582	Mutant	FYCO1	0.000012	-4.546863	
17583	Mutant	CADM1	0.000001	-4.379939	
17584	Mutant	ATP6AP1L	0.00001	-4.29516	
17585	Mutant	RSAD2	0.042664	-4.140086	
17586	Mutant	GPC1	0.000011	-4.112104	
17587	Mutant	MID1	0	-4.045758	
17588	Mutant	TWSG1	0.000008	-3.912104	
17589	Mutant	DMKN	0.000003	-3.83423	
17590	Mutant	LATS2	0.000002	-3.740308	
17591	Mutant	PPP3CA	0.000011	-3.71873	
17592	Mutant	MXRA7	0.000007	-3.70477	
17593	Mutant	CUEDC2	0.000005	-3.636746	
17594	Mutant	ACVR2B	0.000005	-3.390992	
17595	Mutant	GDA	0.000005	-3.335761	
17596	Mutant	LTBP3	0.000008	-3.322505	
17597	Mutant	AP3S1	0.000001	-3.304655	
17598	Mutant	SFTPC	0.003295	-3.29668	

17599	Mutant	TAB2	0.000007	-3.280706	
17600	Mutant	GDI1	0.000006	-3.232506	
17601	Mutant	CYB5B	0.000002	-3.022785	
17602	Mutant	RAB18	0.000004	-2.978612	
17603	Mutant	SLC38A2	0.000011	-2.939973	
17604	Mutant	FAM160B1	0.000002	-2.933826	
17605	Mutant	ALCAM	0	-2.928731	
17606	Mutant	ACTR3B	0.000004	-2.920274	
17607	Mutant	COL18A1	0.000011	-2.87342	
17608	Mutant	SMN1	0.000003	-2.86054	
17609	Mutant	MAP4	0.000005	-2.832426	
17610	Mutant	NIPSNAP1	0.000006	-2.789355	
17611	Mutant	SCG5	0	-2.784814	
17612	Mutant	CIRBP	0.000011	-2.747841	
17613	Mutant	DHX30	0.000003	-2.694996	
17614	Mutant	SOCS5	0.000008	-2.690637	
17615	Mutant	ETS1	0	-2.665223	
17616	Mutant	SRPX	0.000001	-2.641714	
17617	Mutant	TMEM33	0	-2.634908	
17618	Mutant	ZDHHC17	0.000006	-2.562145	
17619	Mutant	CTGF	0	-2.556206	
17620	Mutant	IL1RAP	0	-2.550988	
17621	Mutant	RNGTT	0.000007	-2.474348	
17622	Mutant	HECTD2	0	-2.422578	
17623	Mutant	PRKAR1A	0.000008	-2.414002	
17624	Mutant	NEIL3	0	-2.366079	
17625	Mutant	MAGEA1	0	-2.340793	
17626	Mutant	FXR1	0.000005	-2.303015	
17627	Mutant	GLG1	0.000011	-2.3013	
17628	Mutant	FAT1	0	-2.282566	
17629	Mutant	ACSM3	0	-2.272923	
17630	Mutant	APBB2	0	-2.26671	
17631	Mutant	FOXQ1	0.000001	-2.258836	
17632	Mutant	EFR3A	0.000005	-2.248263	
17633	Mutant	BTBD3	0	-2.240024	
17634	Mutant	IRX3	0.000001	-2.218727	
17635	Mutant	SERPINA1	0.000023	-2.200309	
17636	Mutant	C21orf56	0.000002	-2.194339	
17637	Mutant	SNX25	0	-2.194028	
17638	Mutant	MAP3K7	0.000008	-2.186104	
17639	Mutant	RHOBTB3	0	-2.164787	
17640	Mutant	USP33	0.000005	-2.154806	
17641	Mutant	CYB5R2	0	-2.126386	
17642	Mutant	KIAA1712	0.000001	-2.121687	
17643	Mutant	FRYL	0	-2.120305	
17644	Mutant	PGM2	0	-2.120036	
17645	Mutant	CHURC1	0	-2.07728	
17646	Mutant	ARHGAP18	0	-2.071233	
17647	Mutant	TMEM30B	0	-2.061164	
17648	Mutant	PDLIM5	0.000004	-2.061163	

17649	Mutant	ARMCX4	0	-2.03856	
17650	Mutant	KLK6	0	-2.025113	
17651	Mutant	SMU1	0.00001	-2.014751	
17652	Mutant	TIAL1	0.000008	-2.008613	
17653	Mutant	GNPDA2	0	-2.007448	
17654	Mutant	MGC57346	0.000011	-2.0041	
17655	Mutant	PKNOX1	0	2.039579	
17656	Mutant	LAMA1	0	2.085149	
17657	Mutant	DCAF16	0	2.090845	
17658	Mutant	CDC42SE2	0.000005	2.101573	
17659	Mutant	PAH	0.000001	2.101812	
17660	Mutant	CCDC109A	0.000004	2.105463	
17661	Mutant	KLF11	0	2.14228	
17662	Mutant	DHX15	0	2.14451	
17663	Mutant	TOLLIP	0.000011	2.144689	
17664	Mutant	LMAN2	0.000009	2.153212	
17665	Mutant	MACF1	0.000004	2.176726	
17666	Mutant	UST	0	2.208555	
17667	Mutant	HNRNPH1	0.000008	2.244705	
17668	Mutant	PDIA6	0.000001	2.277313	
17669	Mutant	FAM73B	0.000007	2.288975	
17670	Mutant	ST7	0.000002	2.304399	
17671	Mutant	RFC1	0	2.325179	
17672	Mutant	TMSB4X	0.000008	2.335537	
17673	Mutant	PDIA4	0.00001	2.411109	
17674	Mutant	GPR160	0.000006	2.457485	
17675	Mutant	GPR89A	0.000011	2.508425	
17676	Mutant	VDAC1	0	2.524484	
17677	Mutant	SBF1	0.00001	2.529004	
17678	Mutant	MRPS31	0.000004	2.566806	
17679	Mutant	NCBP1	0.000007	2.570732	
17680	Mutant	MTMR6	0.000005	2.609182	
17681	Mutant	CASK	0.000008	2.618893	
17682	Mutant	EPHB3	0.000008	2.630731	
17683	Mutant	SURF4	0.000006	2.6467	
17684	Mutant	KIAA0895	0	2.655167	
17685	Mutant	MKKS	0.000002	2.66267	
17686	Mutant	KRTAP4-1	0.000001	2.677109	
17687	Mutant	CD163L1	0	2.686773	
17688	Mutant	ROD1	0.000008	2.699594	
17689	Mutant	TBC1D19	0	2.703141	
17690	Mutant	TDRKH	0.000007	2.717036	
17691	Mutant	ANAPC4	0	2.719132	
17692	Mutant	ZCCHC4	0	2.743536	
17693	Mutant	PI4K2B	0	2.780289	
17694	Mutant	CHCHD7	0.000001	2.802096	
17695	Mutant	PDS5A	0	2.81167	
17696	Mutant	GPR125	0	2.853912	
17697	Mutant	HBS1L	0.000005	2.859622	
17698	Mutant	UGDH	0	2.871911	

17699	Mutant	TAF13	0	2.8756	
17700	Mutant	HSDL2	0.000005	2.883179	
17701	Mutant	STAMBPL1	0.000004	2.891243	
17702	Mutant	PDGFC	0	2.969628	
17703	Mutant	ZNF702P	0	2.999537	
17704	Mutant	ECH1	0.000006	3.002113	
17705	Mutant	TSEN15	0.000008	3.014576	
17706	Mutant	C4orf34	0	3.021017	
17707	Mutant	MIPEP	0.000003	3.028462	
17708	Mutant	RBPJ	0	3.033798	
17709	Mutant	LIAS	0	3.034223	
17710	Mutant	LNX2	0.000003	3.098544	
17711	Mutant	SEL1L3	0	3.136382	
17712	Mutant	LCORL	0	3.189998	
17713	Mutant	UBE2K	0	3.208984	
17714	Mutant	RNF6	0.000011	3.218499	
17715	Mutant	SEPSECS	0	3.25347	
17716	Mutant	CARKD	0.000005	3.322652	
17717	Mutant	TNS4	0.000007	3.341882	
17718	Mutant	GFPT1	0.000008	3.359642	
17719	Mutant	TPP2	0	3.361754	
17720	Mutant	MCF2L	0.000008	3.374845	
17721	Mutant	S100A5	0.000012	3.429268	
17722	Mutant	HOXC5	0.000005	3.435105	
17723	Mutant	STIM2	0	3.440687	
17724	Mutant	RALGAPA2	0.000003	3.471811	
17725	Mutant	UBAC2	0.000002	3.488265	
17726	Mutant	C4orf52	0	3.544815	
17727	Mutant	IFT88	0.000004	3.610645	
17728	Mutant	TXNDC9	0.000004	3.677724	
17729	Mutant	RASSF5	0.000001	3.967723	
17730	Mutant	TJP2	0.000003	3.974191	
17731	Mutant	C20orf24	0	4.02621	
17732	Mutant	CD68	0.000005	4.278797	
17733	Mutant	CTSH	0.000011	4.285228	
17734	Mutant	SEC16B	0.000003	4.300372	
17735	Mutant	TBPL1	0.000001	4.377912	
17736	Mutant	ARGLU1	0.000003	4.387082	
17737	Mutant	SLC12A7	0.000006	4.427164	
17738	Mutant	N4BP2	0	4.532577	
17739	Mutant	SERPINB1	0.000006	4.559866	
17740	Mutant	H19	0	4.564358	
17741	Mutant	ANKRD22	0.000008	4.590764	
17742	Mutant	GALK2	0.000011	4.648461	
17743	Mutant	LOC100507328	0.000006	4.76041	
17744	Mutant	IFNGR1	0.000004	4.775064	
17745	Mutant	SMARCA2	0.000012	4.928856	
17746	Mutant	MRPS10	0.000001	4.993962	
17747	Mutant	MAP7	0.000007	4.998464	
17748	Mutant	SIGLEC15	0.000008	5.328116	

17749	Mutant	ARHGAP26	0.000003	5.448188	
17750	Mutant	NF1	0.000011	5.571354	
17751	Mutant	EPHA4	0.000006	5.611204	
17752	Mutant	MSH3	0.00001	5.813158	
17753	Mutant	APOL6	0.000009	5.838919	
17754	Mutant	LPCAT1	0.000007	5.975302	
17755	Mutant	TCF7	0.000005	6.035586	
17756	Mutant	EDAR	0.000008	6.047987	
17757	Mutant	MED20	0.000003	6.097804	
17758	Mutant	SPATA13	0.000011	6.214893	
17759	Mutant	LOC400128	0.000008	6.414813	
17760	Mutant	TST	0.000009	6.449722	
17761	Mutant	HSPA12A	0.000001	6.570195	
17762	Mutant	TAF8	0.000008	6.633628	
17763	Mutant	MAST4	0.000008	6.637752	
17764	Mutant	FUT6	0.000011	6.724803	
17765	Mutant	GMDS	0	7.063824	
17766	Mutant	PLIN2	0.000003	7.13014	
17767	Mutant	ZNF260	0.000009	7.257563	
17768	Mutant	ADAMTS17	0.000008	7.30365	
17769	Mutant	EPB41L4B	0.000001	7.309877	
17770	Mutant	TMEM62	0.000002	7.375007	
17771	Mutant	PDK3	0.000003	7.534799	
17772	Mutant	MAD1L1	0.000002	7.721161	
17773	Mutant	PERP	0.000011	7.760576	
17774	Mutant	MECOM	0.00001	7.812382	
17775	Mutant	AMACR	0.000008	7.819271	
17776	Mutant	GPR39	0.000007	7.846796	
17777	Mutant	DNAJC3	0.000003	7.962623	
17778	Mutant	SH3PXD2A	0.000006	8.056364	
17779	Mutant	C10orf58	0.000003	8.113021	
17780	Mutant	TNFRSF19	0.000006	8.17702	
17781	Mutant	ANP32E	0.000003	8.890413	
17782	Mutant	C6orf64	0.000008	9.350503	
17783	Mutant	DPEP1	0.000011	9.477999	
17784	Mutant	CYorf15B	0.000005	9.546186	
17785	Mutant	EDEM1	0.000009	9.667083	
17786	Mutant	NCRNA00094	0.000004	9.731985	
17787	Mutant	ANKS4B	0.000008	9.996164	
17788	Mutant	ECM1	0.000002	10.281889	
17789	Mutant	PKP1	0.000012	10.698267	
17790	Mutant	EPHB2	0.000004	12.340973	
17791	Mutant	LOC339290	0.000008	12.446039	
17792	Mutant	IGF1R	0.000011	12.967261	
17793	Mutant	SDC1	0.000003	13.139692	
17794	Mutant	A1CF	0.000008	13.437266	
17795	Mutant	IRF8	0.000003	13.757054	
17796	Mutant	ABHD2	0.00001	14.102907	
17797	Mutant	CACNA2D4	0.000007	14.539368	
17798	Mutant	TNFRSF1B	0.000007	14.592414	

17799	Mutant	ACOT1	0.000011	15.376655	
17800	Mutant	CHST11	0.000007	15.569454	
17801	Mutant	BCL2L15	0.000006	16.516903	
17802	Mutant	FCGRT	0.000007	17.453322	
17803	Mutant	TNFSF10	0.000002	18.515111	
17804	Mutant	TMPRSS2	0.000008	18.650402	
17805	Mutant	CXCR3	0.000008	18.966222	
17806	Mutant	C5orf17	0.000004	19.220543	
17807	Mutant	SORBS2	0.000003	20.472901	
17808	Mutant	LGR5	0.000003	21.490774	
17809	Mutant	PTGR1	0.000007	22.056714	
17810	Mutant	EPSTI1	0.000006	22.529177	
17811	Mutant	CTSS	0.000006	23.057346	
17812	Mutant	ZNF703	0.000007	23.366143	
17813	Mutant	CYorf15A	0.000003	23.376836	
17814	Mutant	LOC401522	0.000002	23.921747	
17815	Mutant	CD24	0.000001	25.242614	
17816	Mutant	CCR1	0.000006	25.656619	
17817	Mutant	SCML4	0.000008	25.82047	
17818	Mutant	GCNT3	0.000006	27.110823	
17819	Mutant	SPAG16	0.000005	27.406277	
17820	Mutant	CCR6	0.000005	29.162851	
17821	Mutant	C15orf48	0.000006	30.344559	
17822	Mutant	PROX1	0.000002	31.142485	
17823	Mutant	ASCL2	0.000002	32.801288	
17824	Mutant	GAS2	0.000004	32.924259	
17825	Mutant	SLC7A7	0.000004	33.871488	
17826	Mutant	DDC	0.000002	34.955331	
17827	Mutant	HCN1	0.000011	36.841006	
17828	Mutant	SPRR3	0.000007	37.149195	
17829	Mutant	RASEF	0.000003	38.209334	
17830	Mutant	GIPC2	0.000001	38.52687	
17831	Mutant	CALB1	0.000011	38.922345	
17832	Mutant	GIMAP2	0.000002	38.94836	
17833	Mutant	GSTT1	0.000011	39.362819	
17834	Mutant	GNG2	0.000006	39.84096	
17835	Mutant	ZNF750	0.000002	39.920646	
17836	Mutant	LOC283352	0	40.894875	
17837	Mutant	SLC35D3	0.000001	42.916508	
17838	Mutant	PIP5K1B	0.000004	43.841456	
17839	Mutant	RARRES1	0	44.474544	
17840	Mutant	HGD	0.000005	48.06248	
17841	Mutant	SORBS1	0.000004	48.717851	
17842	Mutant	SCCPDH	0.000003	49.108069	
17843	Mutant	PSMB8	0	52.118448	
17844	Mutant	LOC643201	0.000001	53.597538	
17845	Mutant	UGT1A8	0	55.425596	
17846	Mutant	TFF3	0.000003	60.13651	
17847	Mutant	POF1B	0	61.925637	
17848	Mutant	OXGR1	0.000002	63.151094	

17849	Mutant	EIF1AY	0.000001	63.631934	
17850	Mutant	HKDC1	0.000001	65.399986	
17851	Mutant	PDZK1	0.000001	67.054252	
17852	Mutant	PLAC8	0.000004	67.825095	
17853	Mutant	KCNE3	0.000003	70.051982	
17854	Mutant	AZGP1	0.000005	75.120812	
17855	Mutant	KRT6B	0.000002	80.131876	
17856	Mutant	C9orf64	0.000008	95.383544	
17857	Mutant	ABHD12B	0.000003	96.415173	
17858	Mutant	AKR1C1	0.000001	101.557094	
17859	Mutant	CARD16	0.000005	105.058602	
17860	Mutant	VIL1	0.000001	107.071449	
17861	Mutant	LOC100507192	0.000006	109.086216	
17862	Mutant	CASP1	0.000002	120.264435	
17863	Mutant	UGT1A6	0.000002	123.718578	
17864	Mutant	C9orf152	0.000001	124.698025	
17865	Mutant	ASB4	0	142.105173	
17866	Mutant	CCL14-CCL15	0.000002	143.80414	
17867	Mutant	AKR1C2	0	144.760834	
17868	Mutant	MANSC1	0.000007	164.830215	
17869	Mutant	CLRN3	0.00001	187.914971	
17870	Mutant	TOX3	0	188.756066	
17871	Mutant	FAM3B	0.000009	205.648843	
17872	Mutant	LGALS4	0.000001	249.000167	
17873	Mutant	UGT1A1	0	289.754891	
17874	Mutant	LCK	0.000003	297.044368	
17875	Mutant	AGR2	0.000006	440.058646	
17876	Mutant	PLA2G2A	0.000006	510.8109	
17877	Mutant	TSPAN8	0	548.008396	
17878	Mutant	LYZ	0	553.965593	
17879	Mutant	GPX2	0.000008	649.937377	
17880	Myocardial Ischemia	AGGF1	0.000005	2.12439	
17881	Nausea	MAGEA3	0.001898	-7.658879	
17882	Nausea	MAGEA6	0.0066	-5.763705	
17883	Nausea	PGBD5	0.001898	-2.185691	
17884	Nausea	UCA1	0.034469	-2.108421	
17885	Neck Carcinoma	TOR1AIP2	0.003674	2.578503	
17886	Neoplasm	LOC646014	0.000324	-520.819672	
17887	Neoplasm	SLC2A3	0.012839	-38.132322	
17888	Neoplasm	CHST11	0.005083	-30.759622	
17889	Neoplasm	OTOP2	0	-27.178143	
17890	Neoplasm	GCG	0	-26.920825	
17891	Neoplasm	POPDC3	0.003914	-22.914702	
17892	Neoplasm	TMIGD1	0	-19.655689	
17893	Neoplasm	CHGB	0	-14.584818	
17894	Neoplasm	TGFA	0.001734	-14.284298	
17895	Neoplasm	MKX	0.011387	-13.13189	
17896	Neoplasm	EMID1	0.036913	-12.047588	
17897	Neoplasm	DAB2	0.004511	-11.772476	
17898	Neoplasm	PRF1	0.012737	-11.739147	

17899	Neoplasm	DPYSL3	0.002716	-10.911233	
17900	Neoplasm	PLN	0.000977	-10.884812	
17901	Neoplasm	USP24	0.006177	-10.253317	
17902	Neoplasm	PCP4	0.012116	-9.696901	
17903	Neoplasm	VWCE	0.037347	-9.218495	
17904	Neoplasm	CLU	0.000387	-8.79937	
17905	Neoplasm	TRPM6	0	-8.618878	
17906	Neoplasm	NAV2	0.014313	-8.538061	
17907	Neoplasm	CRYBA2	0	-8.522861	
17908	Neoplasm	TTR	0	-8.441377	
17909	Neoplasm	CXCL12	0	-8.439793	
17910	Neoplasm	ITPR2	0.000357	-8.273259	
17911	Neoplasm	CPE	0.010432	-8.067884	
17912	Neoplasm	HLA-C	0.000032	-7.979817	
17913	Neoplasm	GLDN	0	-7.900203	
17914	Neoplasm	INSL5	0.000002	-7.817222	
17915	Neoplasm	RNF217	0.00966	-7.638889	
17916	Neoplasm	EML1	0.013577	-7.611199	
17917	Neoplasm	JCLN	0	-7.580735	
17918	Neoplasm	TACR2	0	-7.516182	
17919	Neoplasm	SLC16A14	0.011625	-7.472792	
17920	Neoplasm	MARK1	0.005692	-7.342574	
17921	Neoplasm	TAF9B	0.010392	-7.240622	
17922	Neoplasm	CLCA4	0	-7.137504	
17923	Neoplasm	OSBPL6	0.003447	-6.973452	
17924	Neoplasm	OR51E2	0	-6.96699	
17925	Neoplasm	MMRN1	0.000018	-6.936042	
17926	Neoplasm	EPHA7	0	-6.874477	
17927	Neoplasm	HAND1	0	-6.79748	
17928	Neoplasm	CPM	0	-6.779395	
17929	Neoplasm	CA4	0	-6.659526	
17930	Neoplasm	PIGZ	0.00064	-6.641919	
17931	Neoplasm	FHOD3	0.007174	-6.587318	
17932	Neoplasm	FLJ33996	0.008879	-6.481953	
17933	Neoplasm	LOC440302	0.002833	-6.480966	
17934	Neoplasm	LIN9	0.000021	-6.468183	
17935	Neoplasm	AIM1L	0.001601	-6.426495	
17936	Neoplasm	SCN7A	0	-6.303923	
17937	Neoplasm	ZFYVE28	0.028592	-6.296123	
17938	Neoplasm	AMPD1	0	-6.229256	
17939	Neoplasm	SLC17A4	0	-6.211751	
17940	Neoplasm	CLDN23	0	-6.125699	
17941	Neoplasm	MYO5A	0.006913	-6.015864	
17942	Neoplasm	TM4SF1	0.000456	-6.003213	
17943	Neoplasm	GNAO1	0	-5.938094	
17944	Neoplasm	CCR7	0	-5.937196	
17945	Neoplasm	GOLGA9P	0.002632	-5.921016	
17946	Neoplasm	ESPN	0.007977	-5.850137	
17947	Neoplasm	IGJ	0.000115	-5.661065	
17948	Neoplasm	CLEC3B	0	-5.646384	

17949	Neoplasm	PYGM	0	-5.639726	
17950	Neoplasm	MYL9	0.000983	-5.633666	
17951	Neoplasm	MUC4	0.000036	-5.548673	
17952	Neoplasm	SEMA3D	0	-5.539271	
17953	Neoplasm	POPDC2	0	-5.538038	
17954	Neoplasm	CA12	0	-5.531367	
17955	Neoplasm	NNAT	0	-5.523662	
17956	Neoplasm	YPEL1	0.000257	-5.42767	
17957	Neoplasm	HRNBP3	0	-5.412331	
17958	Neoplasm	RBPMS2	0	-5.407643	
17959	Neoplasm	RIMS3	0.031932	-5.391005	
17960	Neoplasm	CD177	0	-5.309023	
17961	Neoplasm	EDN3	0.000002	-5.280036	
17962	Neoplasm	KIF5C	0	-5.213308	
17963	Neoplasm	COMMD1	0.005347	-5.181986	
17964	Neoplasm	LOC389634	0.032934	-5.130612	
17965	Neoplasm	MUCDHL	0.000006	-5.11399	
17966	Neoplasm	LYVE1	0	-5.099281	
17967	Neoplasm	C4orf34	0	-5.07859	
17968	Neoplasm	FOSL1	0.001501	-5.057825	
17969	Neoplasm	CLEC2D	0.001515	-5.007768	
17970	Neoplasm	SDCBP2	0.000001	-5.00517	
17971	Neoplasm	ADH1A	0	-4.942129	
17972	Neoplasm	PDZRN4	0	-4.918182	
17973	Neoplasm	ANKRD20A2	0	-4.869268	
17974	Neoplasm	ADRA2C	0.00673	-4.838363	
17975	Neoplasm	PLG	0.000037	-4.831109	
17976	Neoplasm	RTN1	0.000009	-4.816378	
17977	Neoplasm	LOC149134	0.000056	-4.789165	
17978	Neoplasm	ANKRD18A	0.001132	-4.780088	
17979	Neoplasm	RASL10A	0.023576	-4.776279	
17980	Neoplasm	GNAL	0.011017	-4.772214	
17981	Neoplasm	RNF112	0	-4.763975	
17982	Neoplasm	FMN2	0	-4.762531	
17983	Neoplasm	3-Sep	0.013062	-4.760085	
17984	Neoplasm	FOLR1	0.00455	-4.7329	
17985	Neoplasm	F13A1	0	-4.717275	
17986	Neoplasm	CNR1	0	-4.666203	
17987	Neoplasm	NLGN3	0.021845	-4.643915	
17988	Neoplasm	HSD11B2	0	-4.642659	
17989	Neoplasm	CWH43	0	-4.641098	
17990	Neoplasm	CILP	0	-4.580109	
17991	Neoplasm	REP15	0	-4.575296	
17992	Neoplasm	KIAA1920	0.006147	-4.564109	
17993	Neoplasm	ABCC13	0.00001	-4.56359	
17994	Neoplasm	RECK	0.00532	-4.54864	
17995	Neoplasm	CHN1	0.007548	-4.534394	
17996	Neoplasm	ANXA1	0.001093	-4.504772	
17997	Neoplasm	MGC4172	0	-4.475538	
17998	Neoplasm	APBB2	0.0055	-4.461363	

17999	Neoplasm	CDH19	0	-4.455978	
18000	Neoplasm	PI16	0	-4.454597	
18001	Neoplasm	SPARCL1	0	-4.431656	
18002	Neoplasm	DIXDC1	0.000031	-4.430355	
18003	Neoplasm	TSPAN1	0.000001	-4.421269	
18004	Neoplasm	ABLIM2	0.030738	-4.41836	
18005	Neoplasm	TXNRD1	0.000971	-4.409184	
18006	Neoplasm	MAOB	0	-4.38741	
18007	Neoplasm	TTLL7	0.00057	-4.381729	
18008	Neoplasm	NEO1	0.003073	-4.378099	
18009	Neoplasm	NTN1	0	-4.369826	
18010	Neoplasm	DUSP5	0.002893	-4.355168	
18011	Neoplasm	FLJ32063	0.00001	-4.349534	
18012	Neoplasm	MT1P2	0.002776	-4.347342	
18013	Neoplasm	TNS1	0	-4.345642	
18014	Neoplasm	NCEH1	0.003543	-4.34024	
18015	Neoplasm	FRMD4A	0.004723	-4.336177	
18016	Neoplasm	AP1S3	0.002644	-4.331429	
18017	Neoplasm	C1orf67	0.009553	-4.33118	
18018	Neoplasm	GRASP	0.03682	-4.326048	
18019	Neoplasm	PLCD3	0.003048	-4.324389	
18020	Neoplasm	SPINK5	0.000007	-4.320209	
18021	Neoplasm	MT1JP	0	-4.303425	
18022	Neoplasm	TNXA	0.000599	-4.287645	
18023	Neoplasm	FLJ10357	0.01336	-4.287287	
18024	Neoplasm	TUBB2A	0.00203	-4.280061	
18025	Neoplasm	GCLM	0.003155	-4.246441	
18026	Neoplasm	DMD	0	-4.231737	
18027	Neoplasm	TMEM136	0.011149	-4.229345	
18028	Neoplasm	CCDC29	0.010139	-4.223237	
18029	Neoplasm	HIGD1A	0	-4.221989	
18030	Neoplasm	CES2	0.000001	-4.217799	
18031	Neoplasm	DUXAP10	0.010683	-4.194812	
18032	Neoplasm	FRZB	0	-4.185366	
18033	Neoplasm	GGT7	0.000807	-4.185339	
18034	Neoplasm	GFI1	0.000876	-4.145906	
18035	Neoplasm	H2BFS	0.000199	-4.137364	
18036	Neoplasm	DZIP1L	0.00824	-4.128642	
18037	Neoplasm	SCG2	0	-4.0968	
18038	Neoplasm	ANKRD20B	0.007322	-4.092036	
18039	Neoplasm	ADCY5	0	-4.08228	
18040	Neoplasm	TBRG4	0.039971	-4.077619	
18041	Neoplasm	C19orf33	0.000971	-4.077605	
18042	Neoplasm	JMJD2C	0.000278	-4.077496	
18043	Neoplasm	FGFR1	0.003111	-4.076248	
18044	Neoplasm	DSCR1L1	0	-4.065147	
18045	Neoplasm	TMEM16E	0	-4.062872	
18046	Neoplasm	FXYD3	0	-4.060949	
18047	Neoplasm	C11orf33	0.000012	-4.060275	
18048	Neoplasm	LOC727770	0.00775	-4.056873	

18049	Neoplasm	hCG_1776259	0.000229	-4.051954	
18050	Neoplasm	PTK7	0.003109	-4.015131	
18051	Neoplasm	JARID1D	0	-3.998035	
18052	Neoplasm	CAP2	0	-3.956912	
18053	Neoplasm	FAM151A	0	-3.955291	
18054	Neoplasm	TCP11L2	0.003151	-3.946101	
18055	Neoplasm	INSM1	0	-3.935096	
18056	Neoplasm	PPP1R14A	0	-3.931288	
18057	Neoplasm	CCDC46	0.001963	-3.926507	
18058	Neoplasm	LARP6	0.0088	-3.920801	
18059	Neoplasm	NEU4	0.000001	-3.914172	
18060	Neoplasm	LEFTY1	0.023473	-3.90932	
18061	Neoplasm	SLC22A23	0	-3.901711	
18062	Neoplasm	PLA2G10	0.000013	-3.896015	
18063	Neoplasm	GLUD1	0.000349	-3.895446	
18064	Neoplasm	LOC284120	0.019682	-3.888264	
18065	Neoplasm	CFL2	0	-3.885567	
18066	Neoplasm	IQGAP2	0	-3.87736	
18067	Neoplasm	ZNF204	0.007006	-3.871358	
18068	Neoplasm	LOC644662	0.019548	-3.850669	
18069	Neoplasm	RASSF8	0.015238	-3.833393	
18070	Neoplasm	SLC4A8	0.002264	-3.806897	
18071	Neoplasm	MPP2	0.003307	-3.806164	
18072	Neoplasm	ALPI	0	-3.776042	
18073	Neoplasm	DPP6	0	-3.769505	
18074	Neoplasm	GADD45A	0.002622	-3.749182	
18075	Neoplasm	NMES1	0	-3.734037	
18076	Neoplasm	MGC24975	0.021845	-3.725756	
18077	Neoplasm	HOXB4	0.021845	-3.702856	
18078	Neoplasm	LOC286272	0.042711	-3.698942	
18079	Neoplasm	SLC4A3	0.004901	-3.697639	
18080	Neoplasm	TEP1	0.000306	-3.688525	
18081	Neoplasm	SNIP	0.018508	-3.684335	
18082	Neoplasm	NFKBIB	0.001934	-3.659229	
18083	Neoplasm	MEP1A	0	-3.653428	
18084	Neoplasm	P2RY14	0.000446	-3.628568	
18085	Neoplasm	BEX1	0	-3.617089	
18086	Neoplasm	SCG3	0	-3.610162	
18087	Neoplasm	TEX11	0	-3.602828	
18088	Neoplasm	GHR	0	-3.600481	
18089	Neoplasm	TNFRSF19	0.00673	-3.596454	
18090	Neoplasm	DSC2	0.000004	-3.593929	
18091	Neoplasm	SOSTDC1	0.000002	-3.574382	
18092	Neoplasm	ZBTB10	0.015219	-3.570175	
18093	Neoplasm	AKAP12	0.000109	-3.559876	
18094	Neoplasm	C2orf40	0	-3.530774	
18095	Neoplasm	SVIL	0	-3.527754	
18096	Neoplasm	HSD17B2	0.00001	-3.522316	
18097	Neoplasm	HIST1H2BH	0.000401	-3.516686	
18098	Neoplasm	ROR1	0	-3.509181	

18099	Neoplasm	TACC1	0.001012	-3.504349	
18100	Neoplasm	RHBDL2	0	-3.502465	
18101	Neoplasm	C17orf91	0	-3.497267	
18102	Neoplasm	KIAA1467	0.041857	-3.495127	
18103	Neoplasm	USP2	0	-3.494292	
18104	Neoplasm	NSMAF	0	-3.490679	
18105	Neoplasm	GJB3	0.001571	-3.488437	
18106	Neoplasm	RFX6	0	-3.483487	
18107	Neoplasm	TNFRSF17	0	-3.482547	
18108	Neoplasm	ZNF559	0.04222	-3.479822	
18109	Neoplasm	ZNF396	0.021845	-3.477483	
18110	Neoplasm	TECTA	0.009208	-3.467115	
18111	Neoplasm	MTSS1	0.000145	-3.463537	
18112	Neoplasm	TBRG1	0.0001	-3.460295	
18113	Neoplasm	PRKG2	0	-3.453657	
18114	Neoplasm	EPHA10	0.000008	-3.435053	
18115	Neoplasm	GPA33	0.000001	-3.429682	
18116	Neoplasm	CDKL2	0	-3.426307	
18117	Neoplasm	CD47	0	-3.425163	
18118	Neoplasm	KRT19	0.000002	-3.421713	
18119	Neoplasm	B3GALT5	0	-3.421405	
18120	Neoplasm	IER3	0.001481	-3.421316	
18121	Neoplasm	NBEA	0	-3.41054	
18122	Neoplasm	MMP28	0	-3.407586	
18123	Neoplasm	CCDC68	0	-3.401939	
18124	Neoplasm	NR5A2	0.000094	-3.394343	
18125	Neoplasm	EYA2	0	-3.391627	
18126	Neoplasm	ZBTB16	0	-3.381649	
18127	Neoplasm	LILRB5	0	-3.379378	
18128	Neoplasm	ACBD7	0.002306	-3.371246	
18129	Neoplasm	GPR143	0.001153	-3.368676	
18130	Neoplasm	ARL4D	0.007212	-3.366434	
18131	Neoplasm	METTL2A	0.006997	-3.361801	
18132	Neoplasm	LOC440896	0.001452	-3.357173	
18133	Neoplasm	RIMBP3	0.042322	-3.3527	
18134	Neoplasm	CBX7	0	-3.346332	
18135	Neoplasm	DCN	0.000641	-3.343137	
18136	Neoplasm	GPT	0	-3.336013	
18137	Neoplasm	SLC7A2	0.000037	-3.332803	
18138	Neoplasm	SERPINB9	0.004372	-3.332031	
18139	Neoplasm	FXYD1	0	-3.331368	
18140	Neoplasm	TIPARP	0.001076	-3.323843	
18141	Neoplasm	SCN8A	0.005008	-3.313073	
18142	Neoplasm	INA	0	-3.294364	
18143	Neoplasm	ABCC6	0.008661	-3.29217	
18144	Neoplasm	DKFZP564O08	0.000001	-3.288601	
18145	Neoplasm	IL11RA	0	-3.283501	
18146	Neoplasm	GDA	0.000949	-3.2778	
18147	Neoplasm	ADH1C	0	-3.263362	
18148	Neoplasm	CACNB2	0	-3.256055	

18149	Neoplasm	CTSG	0	-3.250081	
18150	Neoplasm	SMAD3	0.003967	-3.241167	
18151	Neoplasm	IFRD1	0.000456	-3.231977	
18152	Neoplasm	KLRB1	0	-3.231369	
18153	Neoplasm	GPRASP2	0.011255	-3.229416	
18154	Neoplasm	ATP8B1	0	-3.228551	
18155	Neoplasm	DSCAML1	0	-3.228012	
18156	Neoplasm	RFESD	0.007548	-3.218537	
18157	Neoplasm	CNNM4	0	-3.209167	
18158	Neoplasm	HLA-DQB1	0.013951	-3.207945	
18159	Neoplasm	TXNIP	0	-3.20474	
18160	Neoplasm	FOXF1	0.000296	-3.198543	
18161	Neoplasm	CCDC18	0	-3.194796	
18162	Neoplasm	PFKFB1	0.035536	-3.186372	
18163	Neoplasm	PTGER4	0	-3.176752	
18164	Neoplasm	BRP44L	0	-3.173014	
18165	Neoplasm	GPRC5A	0.003894	-3.17029	
18166	Neoplasm	ACRBP	0.01081	-3.16471	
18167	Neoplasm	FKBP4	0.002764	-3.15985	
18168	Neoplasm	MFI2	0.004051	-3.156531	
18169	Neoplasm	PDE4D	0	-3.150488	
18170	Neoplasm	TMEM220	0	-3.143404	
18171	Neoplasm	SIDT1	0	-3.139651	
18172	Neoplasm	SCNN1A	0.000005	-3.135971	
18173	Neoplasm	IER5	0.006768	-3.134763	
18174	Neoplasm	FBLN1	0	-3.123876	
18175	Neoplasm	NBLA00301	0	-3.122942	
18176	Neoplasm	TP53I5	0.000002	-3.120461	
18177	Neoplasm	SNX10	0.013926	-3.114328	
18178	Neoplasm	FAM162A	0	-3.112417	
18179	Neoplasm	FLJ20152	0.000005	-3.093667	
18180	Neoplasm	TST	0.000001	-3.086522	
18181	Neoplasm	RAB27A	0	-3.084845	
18182	Neoplasm	LOC374443	0.009553	-3.083184	
18183	Neoplasm	C5orf4	0	-3.081489	
18184	Neoplasm	ZNF503	0.008386	-3.080527	
18185	Neoplasm	FAM167A	0.006978	-3.079468	
18186	Neoplasm	RHOF	0.002825	-3.060426	
18187	Neoplasm	MAFF	0.001055	-3.059935	
18188	Neoplasm	C6orf29	0.000003	-3.059207	
18189	Neoplasm	DNAH14	0.006182	-3.054123	
18190	Neoplasm	NQO1	0.001934	-3.052136	
18191	Neoplasm	MLXIPL	0.001005	-3.044892	
18192	Neoplasm	LOC100132288	0.003307	-3.042215	
18193	Neoplasm	PIGR	0.000268	-3.039826	
18194	Neoplasm	LOC100133572	0.00044	-3.038936	
18195	Neoplasm	PDLIM3	0.00997	-3.035388	
18196	Neoplasm	POLN	0.006577	-3.027885	
18197	Neoplasm	DPT	0	-3.027548	
18198	Neoplasm	HIG1	0.000002	-3.027138	

18199	Neoplasm	NEUROD1	0	-3.026664	
18200	Neoplasm	MT1E	0	-3.02559	
18201	Neoplasm	CALHM2	0.009282	-3.023566	
18202	Neoplasm	BCLP	0.000001	-3.020425	
18203	Neoplasm	ZNF575	0	-3.018086	
18204	Neoplasm	ACACB	0.000014	-3.000493	
18205	Neoplasm	CPA3	0	-2.996718	
18206	Neoplasm	C2orf88	0	-2.994556	
18207	Neoplasm	LOC100128288	0.010175	-2.991754	
18208	Neoplasm	VSTM2A	0	-2.990994	
18209	Neoplasm	DCLK1	0	-2.977075	
18210	Neoplasm	MR1	0.000015	-2.97589	
18211	Neoplasm	DEFB1	0.000061	-2.97051	
18212	Neoplasm	HRK	0.007558	-2.96953	
18213	Neoplasm	MYO1A	0.000007	-2.962363	
18214	Neoplasm	FAM3D	0.000002	-2.95883	
18215	Neoplasm	TM4SF2	0.000002	-2.95869	
18216	Neoplasm	TUBB2B	0.000163	-2.956319	
18217	Neoplasm	CBLN2	0	-2.947698	
18218	Neoplasm	PHLPPL	0	-2.947295	
18219	Neoplasm	C6orf168	0.007977	-2.944648	
18220	Neoplasm	GAGE12F	0.002593	-2.935788	
18221	Neoplasm	MT1L	0.012569	-2.931789	
18222	Neoplasm	SMPD3	0.000001	-2.930047	
18223	Neoplasm	IFFO2	0.003963	-2.927913	
18224	Neoplasm	SERTAD2	0.001331	-2.923702	
18225	Neoplasm	ZNF165	0.002355	-2.914214	
18226	Neoplasm	GPX3	0.000109	-2.906212	
18227	Neoplasm	DHRS11	0.000418	-2.904213	
18228	Neoplasm	KRT14	0.000004	-2.898214	
18229	Neoplasm	TTC18	0.009553	-2.893427	
18230	Neoplasm	JDP2	0.013717	-2.891871	
18231	Neoplasm	PRIC285	0	-2.887749	
18232	Neoplasm	PPAP2A	0	-2.884461	
18233	Neoplasm	VILL	0.000003	-2.882666	
18234	Neoplasm	STX6	0.019548	-2.881852	
18235	Neoplasm	RND3	0.000038	-2.872728	
18236	Neoplasm	EPB49	0.041352	-2.864503	
18237	Neoplasm	CDKN2B	0.000179	-2.863855	
18238	Neoplasm	CALCOCO2	0.007534	-2.863197	
18239	Neoplasm	MUSTN1	0	-2.844608	
18240	Neoplasm	ST6GALNAC6	0	-2.840149	
18241	Neoplasm	CYP4F12	0.000003	-2.837124	
18242	Neoplasm	C9orf61	0	-2.833333	
18243	Neoplasm	SHROOM2	0.003869	-2.831886	
18244	Neoplasm	PCSK5	0	-2.830533	
18245	Neoplasm	MAML2	0	-2.829664	
18246	Neoplasm	CLDN7	0.000004	-2.827828	
18247	Neoplasm	F2	0.023757	-2.822927	
18248	Neoplasm	TMEM45B	0.000002	-2.820958	

18249	Neoplasm	ERRFI1	0.000738	-2.815094	
18250	Neoplasm	HIST1H2BD	0.002609	-2.811862	
18251	Neoplasm	CASD1	0	-2.80623	
18252	Neoplasm	NAIP	0.003138	-2.800591	
18253	Neoplasm	C14orf139	0	-2.797652	
18254	Neoplasm	CHST5	0.000004	-2.795886	
18255	Neoplasm	HIST1H2BI	0.000973	-2.794488	
18256	Neoplasm	PPARGC1A	0.000012	-2.794212	
18257	Neoplasm	TBCEL	0.004372	-2.792979	
18258	Neoplasm	CHORDC1	0.006682	-2.792148	
18259	Neoplasm	LOC652005	0.014757	-2.78945	
18260	Neoplasm	HIST1H2AD	0.001452	-2.787514	
18261	Neoplasm	RGS19IP1	0.00001	-2.784519	
18262	Neoplasm	AFF3	0	-2.783452	
18263	Neoplasm	FLJ11017	0.000002	-2.782371	
18264	Neoplasm	BFSP1	0.001604	-2.782365	
18265	Neoplasm	C11orf63	0.000128	-2.77536	
18266	Neoplasm	CLDN4	0.001197	-2.769576	
18267	Neoplasm	LRRC1	0.000013	-2.764394	
18268	Neoplasm	DISP2	0	-2.755855	
18269	Neoplasm	ABCC8	0	-2.755043	
18270	Neoplasm	PGBD1	0.0097	-2.75087	
18271	Neoplasm	CD59	0.002521	-2.749877	
18272	Neoplasm	TUB	0.000002	-2.748186	
18273	Neoplasm	GABPB1	0.002342	-2.746774	
18274	Neoplasm	KIAA0574	0	-2.743239	
18275	Neoplasm	TMEM98	0.007833	-2.741353	
18276	Neoplasm	PDE5A	0.000791	-2.738766	
18277	Neoplasm	MT4	0.009717	-2.735925	
18278	Neoplasm	EHD1	0.002556	-2.735799	
18279	Neoplasm	CLYBL	0.00019	-2.733223	
18280	Neoplasm	KIAA1586	0.009932	-2.730824	
18281	Neoplasm	CCL14	0.00017	-2.725208	
18282	Neoplasm	MGC4171	0.00001	-2.724735	
18283	Neoplasm	CEP70	0.014313	-2.723069	
18284	Neoplasm	KIT	0	-2.722354	
18285	Neoplasm	TPK1	0.012249	-2.715114	
18286	Neoplasm	MTL5	0.003175	-2.707026	
18287	Neoplasm	GOLPH2	0.000005	-2.702898	
18288	Neoplasm	PBRM1	0.004716	-2.702513	
18289	Neoplasm	HIST1H2BK	0.000971	-2.69846	
18290	Neoplasm	PPL	0.004048	-2.698124	
18291	Neoplasm	MYL6	0	-2.69657	
18292	Neoplasm	RAD51C	0.000145	-2.695589	
18293	Neoplasm	UTRN	0.000897	-2.694507	
18294	Neoplasm	PXDN	0.009941	-2.692994	
18295	Neoplasm	C16orf89	0	-2.685176	
18296	Neoplasm	HIST1H2BE	0.000348	-2.681598	
18297	Neoplasm	SATB2	0.000007	-2.679973	
18298	Neoplasm	LOC100505584	0.007414	-2.672755	

18299	Neoplasm	CTNND2	0	-2.672752	
18300	Neoplasm	ATL1	0.00856	-2.672005	
18301	Neoplasm	CGN	0.000009	-2.669418	
18302	Neoplasm	LOC728047	0.00775	-2.666216	
18303	Neoplasm	TMOD3	0.000971	-2.662719	
18304	Neoplasm	ACADS	0	-2.654603	
18305	Neoplasm	PDGFRA	0	-2.653227	
18306	Neoplasm	TTRAP	0	-2.65296	
18307	Neoplasm	HIST1H4D	0.011245	-2.651001	
18308	Neoplasm	PROM2	0.001214	-2.647331	
18309	Neoplasm	KLB	0	-2.644092	
18310	Neoplasm	TACC2	0	-2.643764	
18311	Neoplasm	LOC283454	0	-2.641204	
18312	Neoplasm	DPH3	0.003586	-2.639339	
18313	Neoplasm	LOC199800	0.037332	-2.637131	
18314	Neoplasm	SMG7	0.00313	-2.625613	
18315	Neoplasm	HSD17B6	0.000957	-2.620009	
18316	Neoplasm	AOC3	0	-2.617512	
18317	Neoplasm	KIAA0513	0	-2.615759	
18318	Neoplasm	CYP20A1	0.003548	-2.613685	
18319	Neoplasm	PXMP2	0	-2.613262	
18320	Neoplasm	IL17RB	0.004078	-2.609437	
18321	Neoplasm	PIP5K1B	0.000006	-2.603886	
18322	Neoplasm	ANXA2P2	0.003101	-2.59763	
18323	Neoplasm	FLNC	0.000273	-2.597163	
18324	Neoplasm	SSBP2	0	-2.589961	
18325	Neoplasm	STAP2	0	-2.586791	
18326	Neoplasm	ITM2A	0	-2.583142	
18327	Neoplasm	FMO4	0.000009	-2.582345	
18328	Neoplasm	PTD012	0	-2.571727	
18329	Neoplasm	ORMDL2	0.003586	-2.567817	
18330	Neoplasm	NUB1	0.002696	-2.567548	
18331	Neoplasm	MTHFSD	0.003134	-2.56662	
18332	Neoplasm	CKMT1	0.000013	-2.56467	
18333	Neoplasm	SQSTM1	0.003381	-2.564317	
18334	Neoplasm	FLJ30901	0	-2.56354	
18335	Neoplasm	RALA	0.002521	-2.560907	
18336	Neoplasm	EIF3F	0.000568	-2.560412	
18337	Neoplasm	PSMD12	0.000878	-2.55911	
18338	Neoplasm	LOC728897	0.002375	-2.557461	
18339	Neoplasm	FAM69A	0.043208	-2.55646	
18340	Neoplasm	GSTM3	0.000427	-2.553493	
18341	Neoplasm	ZNF33A	0.02751	-2.540084	
18342	Neoplasm	NANS	0.000001	-2.539289	
18343	Neoplasm	CNGA3	0	-2.530801	
18344	Neoplasm	SYNJ2BP	0.003111	-2.524137	
18345	Neoplasm	PSMC4	0.001866	-2.52329	
18346	Neoplasm	TDE2L	0.000006	-2.522827	
18347	Neoplasm	TEF	0	-2.522685	
18348	Neoplasm	LOC201895	0.000001	-2.520197	

18349	Neoplasm	TBXAS1	0.019548	-2.51981	
18350	Neoplasm	SPATA6	0.011536	-2.51343	
18351	Neoplasm	MYH10	0.012046	-2.501664	
18352	Neoplasm	GNAI1	0.000428	-2.500595	
18353	Neoplasm	CHMP2B	0.003529	-2.500537	
18354	Neoplasm	HIST1H2BF	0.000036	-2.495865	
18355	Neoplasm	GPLD1	0.015141	-2.492915	
18356	Neoplasm	STX8	0.003035	-2.492302	
18357	Neoplasm	LRRC27	0.006344	-2.492235	
18358	Neoplasm	FLJ20273	0.000008	-2.483385	
18359	Neoplasm	SFN	0.003433	-2.482749	
18360	Neoplasm	ESF1	0.014293	-2.481679	
18361	Neoplasm	ARHGAP44	0	-2.479384	
18362	Neoplasm	YRDC	0.000454	-2.478321	
18363	Neoplasm	PARD6B	0.002438	-2.474391	
18364	Neoplasm	KIAA0802	0.007178	-2.470119	
18365	Neoplasm	PLIN	0	-2.469331	
18366	Neoplasm	LMNA	0	-2.46552	
18367	Neoplasm	CDV3	0.001501	-2.464801	
18368	Neoplasm	GNA11	0	-2.459933	
18369	Neoplasm	XRRA1	0.000059	-2.459166	
18370	Neoplasm	ILDR2	0.010829	-2.455843	
18371	Neoplasm	SNX5	0.019682	-2.452863	
18372	Neoplasm	FBLN5	0.000608	-2.450308	
18373	Neoplasm	TMEM16G	0	-2.449522	
18374	Neoplasm	LOC728448	0.026406	-2.448021	
18375	Neoplasm	ANK3	0	-2.447477	
18376	Neoplasm	ABTB2	0.002911	-2.445905	
18377	Neoplasm	GPR132	0.009949	-2.438478	
18378	Neoplasm	HIPK2	0.005659	-2.437563	
18379	Neoplasm	FLJ34515	0	-2.434938	
18380	Neoplasm	C16orf46	0.009657	-2.433476	
18381	Neoplasm	SLC36A1	0	-2.431548	
18382	Neoplasm	C11orf65	0.004241	-2.427485	
18383	Neoplasm	STIP1	0.0035	-2.426644	
18384	Neoplasm	FAM95B1	0.010036	-2.426516	
18385	Neoplasm	SUGT1	0.01256	-2.426019	
18386	Neoplasm	PSMA7	0.001063	-2.423479	
18387	Neoplasm	C3orf34	0.017983	-2.423243	
18388	Neoplasm	ESCO1	0	-2.420007	
18389	Neoplasm	C14orf145	0.000527	-2.416938	
18390	Neoplasm	AKAP9	0	-2.41686	
18391	Neoplasm	KRT8	0.002996	-2.415704	
18392	Neoplasm	MAMLD1	0.002865	-2.415506	
18393	Neoplasm	CAMK2D	0	-2.411323	
18394	Neoplasm	WWC2	0.001321	-2.40907	
18395	Neoplasm	WSCD1	0	-2.408483	
18396	Neoplasm	IGHA1	0.00002	-2.402752	
18397	Neoplasm	LTBP4	0	-2.401606	
18398	Neoplasm	PHLDA2	0.000351	-2.397214	

18399	Neoplasm	RSPO2	0	-2.395995	
18400	Neoplasm	TUBA1A	0.005009	-2.390564	
18401	Neoplasm	CDKL1	0	-2.390308	
18402	Neoplasm	ZNF23	0.01363	-2.389468	
18403	Neoplasm	C17orf81	0.006933	-2.388388	
18404	Neoplasm	RIOK3	0.000007	-2.387403	
18405	Neoplasm	GLIPR2	0	-2.384521	
18406	Neoplasm	SERPINA3	0.000286	-2.383856	
18407	Neoplasm	TICAM1	0.002593	-2.373531	
18408	Neoplasm	VPS4B	0	-2.372544	
18409	Neoplasm	PCSK6	0.000006	-2.366172	
18410	Neoplasm	BARX2	0	-2.355854	
18411	Neoplasm	POLR2E	0.008616	-2.351994	
18412	Neoplasm	STOX2	0.011178	-2.350231	
18413	Neoplasm	TNFAIP1	0.003833	-2.33892	
18414	Neoplasm	PSMD8	0.001734	-2.338625	
18415	Neoplasm	SPAG9	0.003672	-2.338502	
18416	Neoplasm	FOXN3	0	-2.338288	
18417	Neoplasm	ARMCX3	0.007254	-2.33515	
18418	Neoplasm	PPP1R12C	0	-2.334646	
18419	Neoplasm	TRPM4	0.000006	-2.333557	
18420	Neoplasm	CCBL1	0.008162	-2.329147	
18421	Neoplasm	KIAA1274	0.004043	-2.327109	
18422	Neoplasm	LOC100509231	0.008704	-2.326008	
18423	Neoplasm	ARRDC4	0	-2.325857	
18424	Neoplasm	SCRN2	0.003139	-2.325757	
18425	Neoplasm	ADARB1	0.003201	-2.32474	
18426	Neoplasm	CAPN5	0	-2.323863	
18427	Neoplasm	C9ORF19	0.000083	-2.320293	
18428	Neoplasm	BTN2A2	0.004519	-2.318143	
18429	Neoplasm	RASSF6	0	-2.317665	
18430	Neoplasm	TNRC6C	0.000079	-2.315086	
18431	Neoplasm	AMOTL2	0.000971	-2.313122	
18432	Neoplasm	RILP	0	-2.310785	
18433	Neoplasm	RBM24	0	-2.307876	
18434	Neoplasm	EHD4	0.003433	-2.301779	
18435	Neoplasm	SSR1	0.006177	-2.300646	
18436	Neoplasm	MCPH1	0.015285	-2.300264	
18437	Neoplasm	FASTKD1	0.020529	-2.298061	
18438	Neoplasm	SH3BGRL2	0.000004	-2.297834	
18439	Neoplasm	SFRP4	0	-2.294594	
18440	Neoplasm	SCUBE2	0	-2.293887	
18441	Neoplasm	IL6ST	0.00012	-2.293095	
18442	Neoplasm	ATP2A1	0.00455	-2.290934	
18443	Neoplasm	TMEM35	0	-2.29044	
18444	Neoplasm	CCDC45	0.001144	-2.289958	
18445	Neoplasm	PLCD1	0	-2.282423	
18446	Neoplasm	FBXW2	0.015016	-2.280679	
18447	Neoplasm	HIST1H4C	0.001424	-2.279835	
18448	Neoplasm	FLJ14981	0.000001	-2.278628	

18449	Neoplasm	M6PRBP1	0.00455	-2.274876	
18450	Neoplasm	ADORA2A	0.015404	-2.274787	
18451	Neoplasm	LOC150759	0.026447	-2.274319	
18452	Neoplasm	TNFSF9	0.001682	-2.272151	
18453	Neoplasm	MEF2C	0	-2.269451	
18454	Neoplasm	AES	0	-2.268848	
18455	Neoplasm	TCF21	0	-2.263516	
18456	Neoplasm	TBC1D8	0.001763	-2.260635	
18457	Neoplasm	GPD2	0.003773	-2.259928	
18458	Neoplasm	FRYL	0	-2.255563	
18459	Neoplasm	EIF4E3	0	-2.254867	
18460	Neoplasm	HSD17B1	0.006954	-2.249908	
18461	Neoplasm	IFNAR2	0.00381	-2.247319	
18462	Neoplasm	RHOB	0.000018	-2.246938	
18463	Neoplasm	RCBTB2	0.033618	-2.244056	
18464	Neoplasm	RHOBTB3	0.011324	-2.24344	
18465	Neoplasm	C21orf2	0.000152	-2.243289	
18466	Neoplasm	MYO10	0.007777	-2.238264	
18467	Neoplasm	DMXL1	0	-2.23329	
18468	Neoplasm	NEK1	0.005663	-2.232097	
18469	Neoplasm	CAS1	0	-2.229294	
18470	Neoplasm	C21orf49	0.0238	-2.228355	
18471	Neoplasm	SNRPA1	0.003398	-2.224707	
18472	Neoplasm	ENTPD3	0	-2.224412	
18473	Neoplasm	SLC1A7	0.000001	-2.224365	
18474	Neoplasm	ST7L	0	-2.223282	
18475	Neoplasm	ULK4	0.009941	-2.221932	
18476	Neoplasm	JUP	0.002556	-2.220965	
18477	Neoplasm	SLC25A34	0.000033	-2.218708	
18478	Neoplasm	FAIM	0.03611	-2.217805	
18479	Neoplasm	ALDH7A1	0.014981	-2.215403	
18480	Neoplasm	PLAC2	0.021845	-2.210621	
18481	Neoplasm	EDG2	0.000005	-2.207748	
18482	Neoplasm	RAP2B	0.000143	-2.205439	
18483	Neoplasm	CRNKL1	0.003882	-2.205356	
18484	Neoplasm	BHLHB3	0	-2.202249	
18485	Neoplasm	LOC285016	0	-2.201143	
18486	Neoplasm	INTS2	0.000039	-2.200614	
18487	Neoplasm	EYA4	0.008495	-2.196879	
18488	Neoplasm	MYLK	0.009119	-2.196158	
18489	Neoplasm	SH2B3	0.00632	-2.193403	
18490	Neoplasm	GALIG	0	-2.192145	
18491	Neoplasm	SBDS	0.002176	-2.191661	
18492	Neoplasm	PSPH	0.031932	-2.189757	
18493	Neoplasm	IL17RD	0.002557	-2.189348	
18494	Neoplasm	FAM36A	0.003976	-2.188178	
18495	Neoplasm	MAL	0	-2.184336	
18496	Neoplasm	GNAQ	0.000004	-2.183832	
18497	Neoplasm	ABHD8	0.019682	-2.183225	
18498	Neoplasm	TCP11L1	0.00036	-2.182346	

18499	Neoplasm	MACF1	0.012878	-2.181325	
18500	Neoplasm	MSI1	0.036913	-2.178791	
18501	Neoplasm	ERCC6	0.002672	-2.177854	
18502	Neoplasm	ALDH5A1	0.006378	-2.177339	
18503	Neoplasm	EPB41L5	0.002728	-2.1762	
18504	Neoplasm	MRLC2	0	-2.176183	
18505	Neoplasm	GSTM2	0.000115	-2.175818	
18506	Neoplasm	MXD1	0.002178	-2.174424	
18507	Neoplasm	CARS2	0.000888	-2.173453	
18508	Neoplasm	ARHGAP20	0	-2.170826	
18509	Neoplasm	PTPN21	0	-2.169707	
18510	Neoplasm	HUS1	0.009117	-2.169651	
18511	Neoplasm	LARP4	0.009225	-2.169576	
18512	Neoplasm	ORF1-FL49	0.000001	-2.166765	
18513	Neoplasm	CNNM2	0	-2.1622	
18514	Neoplasm	GPD1L	0.000012	-2.159267	
18515	Neoplasm	PTK6	0.000009	-2.155801	
18516	Neoplasm	FNIP2	0	-2.155429	
18517	Neoplasm	CPEB3	0	-2.153786	
18518	Neoplasm	KIAA1598	0.010833	-2.153431	
18519	Neoplasm	MOSPD1	0.003914	-2.151393	
18520	Neoplasm	HSPA4	0.000878	-2.150924	
18521	Neoplasm	NME7	0.000037	-2.150461	
18522	Neoplasm	MAL2	0.003554	-2.149676	
18523	Neoplasm	BLMH	0.002902	-2.146388	
18524	Neoplasm	CAMK2N1	0.000957	-2.143111	
18525	Neoplasm	PRRT1	0.027768	-2.143055	
18526	Neoplasm	GPR30	0.000043	-2.142041	
18527	Neoplasm	HOXC6	0.000644	-2.138978	
18528	Neoplasm	C21orf57	0.020347	-2.135568	
18529	Neoplasm	HMGB3	0.014104	-2.131491	
18530	Neoplasm	PSMB7	0.000971	-2.130678	
18531	Neoplasm	ZNF219	0	-2.12931	
18532	Neoplasm	PHF6	0	-2.128406	
18533	Neoplasm	FNDC3A	0.005161	-2.128188	
18534	Neoplasm	ATP6V1C1	0.002176	-2.128012	
18535	Neoplasm	MAGED2	0.000967	-2.127688	
18536	Neoplasm	RIOK1	0.001923	-2.127121	
18537	Neoplasm	IQSEC1	0.002899	-2.12707	
18538	Neoplasm	TTC7B	0.006621	-2.125402	
18539	Neoplasm	CALM1	0	-2.124523	
18540	Neoplasm	APOBEC3C	0.001266	-2.124361	
18541	Neoplasm	F2RL1	0.001934	-2.122474	
18542	Neoplasm	PINK1	0	-2.117277	
18543	Neoplasm	FANCB	0.01232	-2.116075	
18544	Neoplasm	OSBPL1A	0.000001	-2.114528	
18545	Neoplasm	BLVRA	0.001896	-2.112777	
18546	Neoplasm	ZCCHC10	0.001102	-2.112274	
18547	Neoplasm	GLRX3	0.001601	-2.111907	
18548	Neoplasm	WDR79	0.036355	-2.111652	

18549	Neoplasm	LOC653391	0.011795	-2.111207	
18550	Neoplasm	LOC285535	0	-2.111181	
18551	Neoplasm	KRT18	0.000444	-2.109715	
18552	Neoplasm	CHP	0.000003	-2.107398	
18553	Neoplasm	MGC14376	0.000209	-2.105584	
18554	Neoplasm	LOC81691	0.004976	-2.103122	
18555	Neoplasm	GBP2	0.000284	-2.102945	
18556	Neoplasm	KIAA1737	0	-2.102382	
18557	Neoplasm	SPPL2A	0	-2.102164	
18558	Neoplasm	C9orf45	0.0131	-2.102002	
18559	Neoplasm	ShrmL	0.00001	-2.101674	
18560	Neoplasm	ARL5B	0.001361	-2.101628	
18561	Neoplasm	TCEB1	0.004051	-2.099726	
18562	Neoplasm	JUN	0	-2.099439	
18563	Neoplasm	MARVELD3	0	-2.098484	
18564	Neoplasm	FAS	0	-2.09611	
18565	Neoplasm	FLJ12610	0.000009	-2.095074	
18566	Neoplasm	ZNF362	0.009242	-2.094542	
18567	Neoplasm	USP36	0.005902	-2.093428	
18568	Neoplasm	CRY2	0	-2.091582	
18569	Neoplasm	NXT2	0.009712	-2.091424	
18570	Neoplasm	POLA1	0.000257	-2.091156	
18571	Neoplasm	ACOT7	0.012699	-2.089218	
18572	Neoplasm	PTS	0.001084	-2.087676	
18573	Neoplasm	SERTAD1	0.002438	-2.087445	
18574	Neoplasm	CDC42SE2	0	-2.085814	
18575	Neoplasm	TOM1L2	0	-2.085025	
18576	Neoplasm	DNAL1	0.004103	-2.084179	
18577	Neoplasm	ILF3	0.001714	-2.083165	
18578	Neoplasm	VAMP8	0.000007	-2.083103	
18579	Neoplasm	TRPV1	0.015171	-2.082321	
18580	Neoplasm	LOC100129113	0.040603	-2.082276	
18581	Neoplasm	LOC642826	0.006082	-2.078366	
18582	Neoplasm	PSMB3	0.000456	-2.077504	
18583	Neoplasm	C10orf47	0.001521	-2.076716	
18584	Neoplasm	PFAS	0.006474	-2.075764	
18585	Neoplasm	BMP2K	0	-2.075154	
18586	Neoplasm	ATP6V1E1	0.002694	-2.074947	
18587	Neoplasm	MYO1C	0	-2.074156	
18588	Neoplasm	MRPS10	0.002949	-2.072435	
18589	Neoplasm	CENPC1	0.002892	-2.071658	
18590	Neoplasm	CCL14-CCL15	0	-2.070167	
18591	Neoplasm	SLC22A18AS	0	-2.068933	
18592	Neoplasm	PLXNA2	0.000002	-2.068311	
18593	Neoplasm	MAP1LC3B	0.003955	-2.067947	
18594	Neoplasm	MALT1	0.005468	-2.06701	
18595	Neoplasm	SLC25A25	0.003091	-2.066605	
18596	Neoplasm	ATM	0.006159	-2.063517	
18597	Neoplasm	TTC5	0.008495	-2.063407	
18598	Neoplasm	CARM1	0.010012	-2.060209	

18599	Neoplasm	TDRD10	0	-2.058953	
18600	Neoplasm	RIOK2	0.011041	-2.05801	
18601	Neoplasm	TMEM67	0.001515	-2.056877	
18602	Neoplasm	SQRDL	0.000007	-2.056851	
18603	Neoplasm	INPP5A	0	-2.056516	
18604	Neoplasm	ARRB1	0.012011	-2.055017	
18605	Neoplasm	NKX2-3	0	-2.052668	
18606	Neoplasm	PRDM11	0.003139	-2.052501	
18607	Neoplasm	GDPD2	0.000372	-2.052014	
18608	Neoplasm	HIST1H4B	0.026406	-2.047212	
18609	Neoplasm	LARGE	0	-2.046539	
18610	Neoplasm	HN1	0.003798	-2.046453	
18611	Neoplasm	LOC729852	0.014188	-2.04438	
18612	Neoplasm	FLJ41603	0	-2.04177	
18613	Neoplasm	CD58	0.002825	-2.041293	
18614	Neoplasm	EPB41L3	0	-2.03821	
18615	Neoplasm	S100A10	0.000004	-2.036907	
18616	Neoplasm	DUS3L	0.000036	-2.036739	
18617	Neoplasm	SYTL4	0.000002	-2.034218	
18618	Neoplasm	CLTB	0.000008	-2.034168	
18619	Neoplasm	PCCA	0.002848	-2.03407	
18620	Neoplasm	PAG1	0	-2.033606	
18621	Neoplasm	SUPT4H1	0.002137	-2.032625	
18622	Neoplasm	CADM3	0	-2.031083	
18623	Neoplasm	GRHPR	0.009696	-2.030113	
18624	Neoplasm	MAGI2	0	-2.029715	
18625	Neoplasm	SNRPB2	0.021713	-2.029005	
18626	Neoplasm	YJEFN3	0.00683	-2.028748	
18627	Neoplasm	AGPAT5	0.006797	-2.028322	
18628	Neoplasm	CPT2	0	-2.027885	
18629	Neoplasm	ZNF101	0.008117	-2.026637	
18630	Neoplasm	ACVR2B	0.011553	-2.023435	
18631	Neoplasm	TSPAN3	0	-2.021741	
18632	Neoplasm	STK17A	0.001462	-2.021667	
18633	Neoplasm	MXI1	0	-2.021021	
18634	Neoplasm	NRAS	0.003776	-2.020621	
18635	Neoplasm	BCLAF1	0.00092	-2.019804	
18636	Neoplasm	SVOP	0	-2.017975	
18637	Neoplasm	HEATR6	0.001574	-2.017191	
18638	Neoplasm	ZFAND5	0.000486	-2.016045	
18639	Neoplasm	EFCAB7	0.003209	-2.014921	
18640	Neoplasm	PMFBP1	0.004078	-2.014917	
18641	Neoplasm	MRPS18C	0.002825	-2.014221	
18642	Neoplasm	POLD4	0.000002	-2.014145	
18643	Neoplasm	ACOT9	0.009912	-2.01377	
18644	Neoplasm	FRG1B	0.012116	-2.013501	
18645	Neoplasm	LTB4R	0.040194	-2.012137	
18646	Neoplasm	STRADA	0.000227	-2.011836	
18647	Neoplasm	DNAJA1	0.002609	-2.009888	
18648	Neoplasm	REEP5	0.0025	-2.009849	

18649	Neoplasm	MSI2	0.013436	-2.008565	
18650	Neoplasm	HERPUD1	0	-2.008187	
18651	Neoplasm	PCSK1N	0.000002	-2.008034	
18652	Neoplasm	KDELR3	0.005663	-2.007188	
18653	Neoplasm	ARMCX5	0.004295	-2.005073	
18654	Neoplasm	ANKRD57	0.002996	-2.004823	
18655	Neoplasm	PSMF1	0.006577	-2.0029	
18656	Neoplasm	HMP19	0	-2.002601	
18657	Neoplasm	CTNND1	0.000001	-2.001972	
18658	Neoplasm	COPG	0	-1.774521	
18659	Neoplasm	LOC158257	0	1.8571	
18660	Neoplasm	ECSCR	0.002438	2.000173	
18661	Neoplasm	PRR16	0	2.000888	
18662	Neoplasm	CDT1	0.000228	2.001072	
18663	Neoplasm	TMEM166	0.000065	2.00184	
18664	Neoplasm	DACH	0.000215	2.00298	
18665	Neoplasm	IBSP	0	2.003274	
18666	Neoplasm	RUNX1	0.000036	2.004859	
18667	Neoplasm	CTSD	0.007351	2.0054	
18668	Neoplasm	COL5A1	0	2.00545	
18669	Neoplasm	LOC729088	0.002178	2.00553	
18670	Neoplasm	TG	0	2.006777	
18671	Neoplasm	HIBADH	0.000716	2.006852	
18672	Neoplasm	ZNF703	0	2.007057	
18673	Neoplasm	WDR51B	0	2.007203	
18674	Neoplasm	LONP1	0	2.008715	
18675	Neoplasm	CCT2	0.000355	2.00934	
18676	Neoplasm	MTA2	0.000001	2.010076	
18677	Neoplasm	MLKL	0.010752	2.013094	
18678	Neoplasm	LGALS8	0	2.013911	
18679	Neoplasm	PRPF6	0	2.014784	
18680	Neoplasm	C5orf46	0	2.015334	
18681	Neoplasm	MED30	0.00024	2.015358	
18682	Neoplasm	KIF15	0	2.015372	
18683	Neoplasm	GOLT1B	0	2.015554	
18684	Neoplasm	CDC123	0	2.016474	
18685	Neoplasm	SH3BGRL3	0.017778	2.016755	
18686	Neoplasm	PYCR1	0.000055	2.016871	
18687	Neoplasm	NCOA7	0.000121	2.017206	
18688	Neoplasm	LOC642869	0.000007	2.017381	
18689	Neoplasm	HDAC2	0.000892	2.017489	
18690	Neoplasm	MTERFD1	0.000001	2.018794	
18691	Neoplasm	KIAA1912	0	2.020831	
18692	Neoplasm	GRAMD1A	0	2.022655	
18693	Neoplasm	KIF11	0.000001	2.025286	
18694	Neoplasm	IRF6	0.005045	2.025898	
18695	Neoplasm	BCL10	0.039875	2.027342	
18696	Neoplasm	NUP107	0	2.028406	
18697	Neoplasm	CCDC59	0	2.030633	
18698	Neoplasm	PDZK1P1	0.002728	2.031559	

18699	Neoplasm	ANTXR1	0	2.033572	
18700	Neoplasm	LOXL2	0	2.033579	
18701	Neoplasm	FOXO1A	0	2.034916	
18702	Neoplasm	STAT6	0.00843	2.036991	
18703	Neoplasm	SSX1	0	2.037624	
18704	Neoplasm	G0S2	0.000527	2.038066	
18705	Neoplasm	KLHL35	0.006167	2.038212	
18706	Neoplasm	NME1-NME2	0.000103	2.03843	
18707	Neoplasm	SHE	0.001063	2.039587	
18708	Neoplasm	DKK4	0	2.040699	
18709	Neoplasm	SMOX	0.000001	2.041865	
18710	Neoplasm	SET	0	2.042096	
18711	Neoplasm	SDC2	0.003406	2.042346	
18712	Neoplasm	DLG1	0.000784	2.048059	
18713	Neoplasm	DCUN1D4	0.002398	2.048193	
18714	Neoplasm	SPOCK1	0	2.049475	
18715	Neoplasm	TIMELESS	0	2.051896	
18716	Neoplasm	NOV	0.014665	2.054291	
18717	Neoplasm	SQLE	0.008905	2.058426	
18718	Neoplasm	CDH24	0	2.058912	
18719	Neoplasm	DPH2	0.000054	2.059234	
18720	Neoplasm	C7orf30	0.001055	2.059312	
18721	Neoplasm	CCDC52	0.019682	2.059836	
18722	Neoplasm	RCAN3	0.033618	2.061511	
18723	Neoplasm	C7orf36	0	2.064753	
18724	Neoplasm	OIP5	0.000001	2.065998	
18725	Neoplasm	GP9	0.000021	2.066583	
18726	Neoplasm	PITPNM3	0.001321	2.068913	
18727	Neoplasm	RHBDF2	0	2.069633	
18728	Neoplasm	ZDHHC6	0	2.070848	
18729	Neoplasm	SNAP25	0.000994	2.072246	
18730	Neoplasm	MGAM	0.015147	2.072616	
18731	Neoplasm	OSBPL3	0	2.072849	
18732	Neoplasm	NUP205	0.000001	2.074642	
18733	Neoplasm	SEMA4B	0.011658	2.074967	
18734	Neoplasm	BMS1	0.000648	2.07506	
18735	Neoplasm	FA2H	0.002111	2.075208	
18736	Neoplasm	GAPDH	0.000008	2.076062	
18737	Neoplasm	STT3A	0	2.077468	
18738	Neoplasm	ENO1	0.000002	2.078688	
18739	Neoplasm	EIF2C2	0.000983	2.081042	
18740	Neoplasm	PRKCI	0.024617	2.082491	
18741	Neoplasm	PSMB9	0.015099	2.083567	
18742	Neoplasm	C11orf71	0.003975	2.084208	
18743	Neoplasm	PPRC1	0.000565	2.084755	
18744	Neoplasm	MB	0.000079	2.084983	
18745	Neoplasm	TEX10	0.000091	2.089342	
18746	Neoplasm	DTX4	0.01187	2.089791	
18747	Neoplasm	C14orf118	0.002224	2.090132	
18748	Neoplasm	FOSL2	0.005768	2.090826	

18749	Neoplasm	KIAA0907	0.000141	2.092142	
18750	Neoplasm	THY1	0	2.09267	
18751	Neoplasm	RFC5	0.000001	2.096438	
18752	Neoplasm	ANKRD28	0.003161	2.097112	
18753	Neoplasm	KAT2A	0	2.099237	
18754	Neoplasm	GPR161	0.003151	2.099688	
18755	Neoplasm	TUBA4A	0.000277	2.100791	
18756	Neoplasm	CLEC5A	0	2.101128	
18757	Neoplasm	EFNA5	0.00968	2.101146	
18758	Neoplasm	EPPK1	0.000001	2.101393	
18759	Neoplasm	C1orf116	0	2.101424	
18760	Neoplasm	FLJ10826	0.000009	2.102346	
18761	Neoplasm	GSTO1	0.000868	2.103585	
18762	Neoplasm	GSPT1	0.002224	2.104227	
18763	Neoplasm	TP53I11	0.006536	2.104362	
18764	Neoplasm	XPO1	0.001601	2.107384	
18765	Neoplasm	SFTA2	0	2.108096	
18766	Neoplasm	SNRPE	0.000957	2.108572	
18767	Neoplasm	DCP1A	0.005453	2.108943	
18768	Neoplasm	SLC25A32	0.000001	2.113102	
18769	Neoplasm	KIAA1522	0.002098	2.114419	
18770	Neoplasm	GINS2	0.000001	2.11675	
18771	Neoplasm	NUP85	0.000001	2.11777	
18772	Neoplasm	INTS12	0.007343	2.117921	
18773	Neoplasm	RBM28	0	2.120363	
18774	Neoplasm	LOC727869	0.015721	2.121104	
18775	Neoplasm	SFRS15	0.002521	2.121254	
18776	Neoplasm	MTHFD1	0.000106	2.122304	
18777	Neoplasm	COL7A1	0	2.12324	
18778	Neoplasm	NCAPG2	0	2.125408	
18779	Neoplasm	CPEB2	0.010242	2.12979	
18780	Neoplasm	TNIP1	0.007915	2.130822	
18781	Neoplasm	FANCL	0	2.139324	
18782	Neoplasm	FLNB	0.000002	2.142236	
18783	Neoplasm	RDH13	0.001311	2.142516	
18784	Neoplasm	PRKACB	0.002696	2.143077	
18785	Neoplasm	C6orf118	0	2.143404	
18786	Neoplasm	RPL28	0.000284	2.146094	
18787	Neoplasm	CBX2	0.000054	2.149236	
18788	Neoplasm	PAQR4	0	2.151409	
18789	Neoplasm	OAS3	0.000001	2.155018	
18790	Neoplasm	FANCG	0	2.166676	
18791	Neoplasm	C1orf75	0	2.166889	
18792	Neoplasm	CKAP5	0.000231	2.168001	
18793	Neoplasm	IL24	0	2.168089	
18794	Neoplasm	SHISA5	0.012794	2.168146	
18795	Neoplasm	PTK2	0.000052	2.169503	
18796	Neoplasm	SLC25A24	0.010859	2.170225	
18797	Neoplasm	CCNU	0.000048	2.172188	
18798	Neoplasm	CCL4	0	2.174968	

18799	Neoplasm	NBEAL2	0.000678	2.177044	
18800	Neoplasm	LOC644242	0.001622	2.179539	
18801	Neoplasm	PLS3	0	2.181015	
18802	Neoplasm	SERPINH1	0	2.184798	
18803	Neoplasm	VPS8	0.000456	2.185653	
18804	Neoplasm	ZNF259	0.000263	2.187872	
18805	Neoplasm	RAB15	0	2.188293	
18806	Neoplasm	JAG2	0.000001	2.191192	
18807	Neoplasm	LPGAT1	0.000214	2.192642	
18808	Neoplasm	WDR43	0.000001	2.194866	
18809	Neoplasm	PLSCR4	0.000705	2.195004	
18810	Neoplasm	CD1A	0	2.195462	
18811	Neoplasm	CCND2	0.000586	2.19821	
18812	Neoplasm	CTSC	0.043208	2.198758	
18813	Neoplasm	ACSS1	0.034762	2.200019	
18814	Neoplasm	HM13	0	2.200948	
18815	Neoplasm	NOL6	0	2.200948	
18816	Neoplasm	SLC7A1	0	2.204228	
18817	Neoplasm	PTPRK	0.002737	2.208975	
18818	Neoplasm	A4GALT	0.002329	2.214058	
18819	Neoplasm	ALG5	0	2.216436	
18820	Neoplasm	C13orf18	0	2.218657	
18821	Neoplasm	REV3L	0.003783	2.22224	
18822	Neoplasm	SETD4	0.003586	2.225888	
18823	Neoplasm	TMBIM1	0.026406	2.230122	
18824	Neoplasm	FLJ31306	0.003702	2.233375	
18825	Neoplasm	CHD9	0.003944	2.242315	
18826	Neoplasm	LOC728264	0.00086	2.24295	
18827	Neoplasm	LTA	0.000018	2.24405	
18828	Neoplasm	WDR33	0.002694	2.247316	
18829	Neoplasm	POLR1D	0.000001	2.252838	
18830	Neoplasm	CTSK	0	2.261573	
18831	Neoplasm	NUP37	0.000001	2.262069	
18832	Neoplasm	NUDCD1	0	2.265265	
18833	Neoplasm	LOC100507376	0.001901	2.266706	
18834	Neoplasm	ID4	0.0035	2.267087	
18835	Neoplasm	FUT8	0	2.267452	
18836	Neoplasm	PITX1	0.000001	2.26755	
18837	Neoplasm	LOC153546	0.000905	2.273416	
18838	Neoplasm	SH3PXD2A	0.001214	2.275325	
18839	Neoplasm	LCOR	0.035536	2.276058	
18840	Neoplasm	RBBP5	0.000265	2.277786	
18841	Neoplasm	CAPN10	0.002776	2.281419	
18842	Neoplasm	BCKDHB	0.033618	2.281949	
18843	Neoplasm	DIAPH1	0.000277	2.289976	
18844	Neoplasm	TM4SF18	0.000088	2.291425	
18845	Neoplasm	NF1	0.000456	2.294154	
18846	Neoplasm	EPHA1	0.007652	2.294364	
18847	Neoplasm	KIF20A	0	2.295043	
18848	Neoplasm	TNFRSF10A	0	2.304169	

18849	Neoplasm	DDIT4	0.000008	2.30636	
18850	Neoplasm	GGCT	0	2.307429	
18851	Neoplasm	MPZL1	0.000971	2.309346	
18852	Neoplasm	UBD	0	2.311269	
18853	Neoplasm	ARL8A	0	2.312248	
18854	Neoplasm	NPAS2	0.000307	2.31264	
18855	Neoplasm	ADAM12	0	2.314582	
18856	Neoplasm	SLC41A2	0.009376	2.321086	
18857	Neoplasm	IGHG1	0	2.321187	
18858	Neoplasm	C20orf42	0	2.321584	
18859	Neoplasm	CMTM8	0	2.325083	
18860	Neoplasm	GTF2IRD2	0.026368	2.328707	
18861	Neoplasm	HS2ST1	0.000002	2.328743	
18862	Neoplasm	LOC100133056	0	2.328852	
18863	Neoplasm	SNRPF	0.000586	2.337388	
18864	Neoplasm	CDC42EP1	0.000001	2.339437	
18865	Neoplasm	LOC595101	0.003321	2.349853	
18866	Neoplasm	RACGAP1	0.000285	2.352492	
18867	Neoplasm	ENPEP	0	2.363024	
18868	Neoplasm	CDH11	0	2.363087	
18869	Neoplasm	WNT5A	0	2.363404	
18870	Neoplasm	COL5A2	0	2.3652	
18871	Neoplasm	LOC285636	0.00022	2.367596	
18872	Neoplasm	MMP14	0	2.369605	
18873	Neoplasm	MADH3	0.000016	2.371723	
18874	Neoplasm	CCND1	0.000167	2.37227	
18875	Neoplasm	TH1L	0	2.374284	
18876	Neoplasm	ISL1	0.00781	2.387441	
18877	Neoplasm	SHC3	0.002224	2.389555	
18878	Neoplasm	MRPS17	0.000001	2.391333	
18879	Neoplasm	KIAA1257	0	2.392024	
18880	Neoplasm	ITGA6	0	2.395665	
18881	Neoplasm	LOC541469	0.002288	2.395799	
18882	Neoplasm	PTTG1	0.000355	2.399344	
18883	Neoplasm	GRIN2D	0	2.400151	
18884	Neoplasm	CASC4	0.000401	2.401116	
18885	Neoplasm	LOC100131564	0.000401	2.401604	
18886	Neoplasm	NOP56	0.000001	2.40229	
18887	Neoplasm	ZWILCH	0	2.403617	
18888	Neoplasm	ACER2	0.002288	2.405364	
18889	Neoplasm	hCG_1981531	0	2.40923	
18890	Neoplasm	ATP2C1	0.013657	2.409518	
18891	Neoplasm	MYB	0.001734	2.413454	
18892	Neoplasm	LOC729680	0.00039	2.414852	
18893	Neoplasm	E2F3	0.000284	2.418337	
18894	Neoplasm	GCA	0.001553	2.431306	
18895	Neoplasm	BCL2L12	0	2.434522	
18896	Neoplasm	PIK3C2A	0.002593	2.43464	
18897	Neoplasm	TWIST1	0	2.436027	
18898	Neoplasm	INSIG2	0.011477	2.436373	

18899	Neoplasm	DGAT2	0	2.437801	
18900	Neoplasm	AXL	0.003433	2.444911	
18901	Neoplasm	ZFP36	0.008906	2.452941	
18902	Neoplasm	FASN	0	2.454801	
18903	Neoplasm	LOC388564	0.003446	2.45847	
18904	Neoplasm	SERINC2	0.002	2.459978	
18905	Neoplasm	NOL8	0.000116	2.460398	
18906	Neoplasm	PDXK	0.000174	2.460748	
18907	Neoplasm	MARCKSL1	0.000038	2.462448	
18908	Neoplasm	FANCA	0.004036	2.463241	
18909	Neoplasm	DNAJB4	0.013657	2.464493	
18910	Neoplasm	C12orf11	0	2.477935	
18911	Neoplasm	ZNF529	0.003381	2.486826	
18912	Neoplasm	VARS	0	2.490948	
18913	Neoplasm	TMEM158	0	2.496317	
18914	Neoplasm	ICAM1	0	2.498583	
18915	Neoplasm	C12orf48	0.000192	2.508405	
18916	Neoplasm	GYPC	0.000971	2.521104	
18917	Neoplasm	PKM2	0.000527	2.525776	
18918	Neoplasm	GNL3	0.000862	2.527467	
18919	Neoplasm	SERPINE2	0.00017	2.527904	
18920	Neoplasm	ATP5C1	0.002593	2.528303	
18921	Neoplasm	TAF1D	0.00002	2.528423	
18922	Neoplasm	RALGAPA2	0.003167	2.530418	
18923	Neoplasm	IARS	0.000066	2.534859	
18924	Neoplasm	ALPK3	0.011832	2.536828	
18925	Neoplasm	ITGA4	0.00231	2.538836	
18926	Neoplasm	GNE	0.031845	2.540196	
18927	Neoplasm	TRAIP	0	2.54541	
18928	Neoplasm	RPS27	0.001601	2.550802	
18929	Neoplasm	COL2A1	0	2.555688	
18930	Neoplasm	GPR56	0	2.556583	
18931	Neoplasm	FNDC3B	0.000829	2.559003	
18932	Neoplasm	TRUB1	0.000049	2.560965	
18933	Neoplasm	GPR34	0.000285	2.56189	
18934	Neoplasm	MCM7	0	2.562626	
18935	Neoplasm	OLA1	0.000115	2.567925	
18936	Neoplasm	UBE2L6	0.000001	2.570384	
18937	Neoplasm	SAMD13	0.003394	2.572244	
18938	Neoplasm	PSMG1	0.000001	2.572526	
18939	Neoplasm	CYP2U1	0.001452	2.573834	
18940	Neoplasm	CEP68	0.001734	2.584989	
18941	Neoplasm	CDC6	0	2.585624	
18942	Neoplasm	KLK7	0	2.5896	
18943	Neoplasm	SLC5A1	0.005513	2.592929	
18944	Neoplasm	CENPN	0	2.609111	
18945	Neoplasm	MYL6B	0	2.611333	
18946	Neoplasm	C2orf70	0	2.620584	
18947	Neoplasm	HIST3H2A	0.00005	2.620932	
18948	Neoplasm	MGC4308	0.000012	2.624252	

18949	Neoplasm	SMAD1	0.000456	2.624873	
18950	Neoplasm	SH3YL1	0.014111	2.626305	
18951	Neoplasm	LOC642103	0.001103	2.632824	
18952	Neoplasm	CHCHD6	0	2.643255	
18953	Neoplasm	GDF15	0.000083	2.644731	
18954	Neoplasm	SYCP3	0.003101	2.648587	
18955	Neoplasm	CCNA2	0.000001	2.6516	
18956	Neoplasm	C3orf31	0.000179	2.665299	
18957	Neoplasm	SKA3	0	2.687415	
18958	Neoplasm	BOP1	0	2.693284	
18959	Neoplasm	E2F5	0	2.697971	
18960	Neoplasm	GBP1	0	2.698104	
18961	Neoplasm	ZNF593	0.000689	2.699951	
18962	Neoplasm	METTL13	0.000434	2.712642	
18963	Neoplasm	C8orf83	0.003511	2.713181	
18964	Neoplasm	RALGAPA1	0.001977	2.714432	
18965	Neoplasm	KLK11	0.006314	2.714457	
18966	Neoplasm	CSMD1	0.002775	2.722372	
18967	Neoplasm	LRRC6	0	2.722628	
18968	Neoplasm	SPG20	0.001197	2.726715	
18969	Neoplasm	RAD54B	0.000645	2.728082	
18970	Neoplasm	CDK13	0.001661	2.733786	
18971	Neoplasm	PAFAH1B3	0	2.744808	
18972	Neoplasm	REPS1	0.0035	2.749114	
18973	Neoplasm	SULT1C2	0.000809	2.7538	
18974	Neoplasm	EIF5A	0	2.754041	
18975	Neoplasm	PLCB4	0	2.756105	
18976	Neoplasm	ADAP1	0.007915	2.777658	
18977	Neoplasm	PCDHA4	0.00532	2.815103	
18978	Neoplasm	NPC1	0.005291	2.816706	
18979	Neoplasm	QPCT	0.0001	2.81904	
18980	Neoplasm	MFAP2	0	2.834898	
18981	Neoplasm	PROX1	0	2.850013	
18982	Neoplasm	GNB1L	0	2.851117	
18983	Neoplasm	LGR5	0.000001	2.855969	
18984	Neoplasm	LMNB2	0	2.858719	
18985	Neoplasm	RAD23B	0.00064	2.859517	
18986	Neoplasm	SLC7A6	0	2.87069	
18987	Neoplasm	WISP1	0	2.875563	
18988	Neoplasm	NT5DC2	0	2.876001	
18989	Neoplasm	RSPO3	0.001791	2.880999	
18990	Neoplasm	LOC728153	0.003101	2.893528	
18991	Neoplasm	HNRNPL	0	2.905758	
18992	Neoplasm	LOC100287896	0.000107	2.912588	
18993	Neoplasm	ESPL1	0	2.913415	
18994	Neoplasm	CDK1	0.000371	2.916923	
18995	Neoplasm	TPD52L1	0.000259	2.917089	
18996	Neoplasm	CXCL6	0	2.921339	
18997	Neoplasm	NUDT1	0	2.924762	
18998	Neoplasm	FAM89A	0	2.927309	

18999	Neoplasm	CCT6A	0.000387	2.927714	
19000	Neoplasm	DTL	0.000149	2.931445	
19001	Neoplasm	SULF1	0	2.936796	
19002	Neoplasm	LACTB2	0.008833	2.941019	
19003	Neoplasm	CDKN2A	0.010583	2.944264	
19004	Neoplasm	ETS2	0	2.951832	
19005	Neoplasm	CYP4X1	0.000005	2.957464	
19006	Neoplasm	SNORA71A	0	2.960057	
19007	Neoplasm	RPGRIP1L	0	2.967761	
19008	Neoplasm	HEG1	0.003101	2.973493	
19009	Neoplasm	IL22RA1	0.007915	3.005191	
19010	Neoplasm	KCTD1	0.004402	3.007065	
19011	Neoplasm	TMEM159	0.013783	3.016472	
19012	Neoplasm	LOC100133920	0	3.017109	
19013	Neoplasm	NRARP	0.002776	3.021461	
19014	Neoplasm	NOP16	0.000752	3.023389	
19015	Neoplasm	RNF183	0	3.027376	
19016	Neoplasm	CTSE	0.005485	3.036036	
19017	Neoplasm	KIF4A	0.000001	3.052197	
19018	Neoplasm	NPIPL3	0.002977	3.055344	
19019	Neoplasm	ROM1	0.027768	3.058661	
19020	Neoplasm	SLC19A1	0	3.061448	
19021	Neoplasm	CTPS	0.000415	3.06325	
19022	Neoplasm	PLD1	0.011998	3.068428	
19023	Neoplasm	MGC48628	0.0067	3.068882	
19024	Neoplasm	GINS1	0	3.07162	
19025	Neoplasm	UTP14A	0.000259	3.072644	
19026	Neoplasm	FAM171B	0.003554	3.087231	
19027	Neoplasm	PTGFR	0.000456	3.090097	
19028	Neoplasm	TFF3	0	3.103154	
19029	Neoplasm	STAT1	0	3.107809	
19030	Neoplasm	IL15RA	0.000751	3.127308	
19031	Neoplasm	MYEOV	0.000002	3.139384	
19032	Neoplasm	PLXDC1	0.0035	3.144569	
19033	Neoplasm	ANKLE2	0.000987	3.151218	
19034	Neoplasm	C7orf46	0.002119	3.161961	
19035	Neoplasm	LOC100132891	0.000088	3.166236	
19036	Neoplasm	C20orf20	0	3.177504	
19037	Neoplasm	DKC1	0.000152	3.185826	
19038	Neoplasm	GREM1	0	3.195002	
19039	Neoplasm	KIF26B	0	3.195147	
19040	Neoplasm	C11orf82	0	3.210526	
19041	Neoplasm	EEF1E1	0.000375	3.219963	
19042	Neoplasm	FAM150A	0	3.226216	
19043	Neoplasm	XK	0.012506	3.234752	
19044	Neoplasm	NUTF2	0	3.241433	
19045	Neoplasm	CEP55	0	3.248565	
19046	Neoplasm	LOC100507273	0.001601	3.2515	
19047	Neoplasm	SOD2	0	3.254564	
19048	Neoplasm	SRPK2	0.001532	3.255359	

19049	Neoplasm	GOLT1A	0.026406	3.283054	
19050	Neoplasm	GPSM2	0	3.29156	
19051	Neoplasm	NEBL	0	3.311461	
19052	Neoplasm	MKI67	0.000284	3.320027	
19053	Neoplasm	DSG3	0	3.327281	
19054	Neoplasm	KCNH8	0	3.352898	
19055	Neoplasm	CENPP	0	3.361062	
19056	Neoplasm	CD3EAP	0	3.363024	
19057	Neoplasm	ZNF521	0.003047	3.364834	
19058	Neoplasm	SLC5A6	0	3.37519	
19059	Neoplasm	CTAGE5	0.003101	3.383509	
19060	Neoplasm	USP18	0.009776	3.38358	
19061	Neoplasm	CKS2	0.000011	3.385444	
19062	Neoplasm	VEGFA	0.009062	3.388828	
19063	Neoplasm	ZCCHC7	0.002825	3.39953	
19064	Neoplasm	MCM4	0	3.415645	
19065	Neoplasm	IGFL2	0	3.42052	
19066	Neoplasm	RAB36	0	3.445516	
19067	Neoplasm	TACSTD2	0	3.446898	
19068	Neoplasm	KRT17P3	0.0208	3.447893	
19069	Neoplasm	UBE2S	0	3.484518	
19070	Neoplasm	MIF	0.000001	3.504093	
19071	Neoplasm	KPNA2	0.000286	3.504182	
19072	Neoplasm	SGCD	0	3.5154	
19073	Neoplasm	CTSO	0.000276	3.531421	
19074	Neoplasm	RNASEH2A	0	3.538918	
19075	Neoplasm	NCRNA00152	0	3.57495	
19076	Neoplasm	JAM3	0.003955	3.583301	
19077	Neoplasm	CST1	0.000009	3.584124	
19078	Neoplasm	CTHRC1	0.000001	3.591327	
19079	Neoplasm	DIAPH3	0	3.601613	
19080	Neoplasm	FAM105A	0.004151	3.608109	
19081	Neoplasm	LOC541471	0	3.618415	
19082	Neoplasm	C1orf135	0	3.625709	
19083	Neoplasm	TMEPAI	0	3.644258	
19084	Neoplasm	IMPDH1	0	3.647148	
19085	Neoplasm	GALNT4	0.003537	3.660364	
19086	Neoplasm	OLFML1	0.001452	3.668098	
19087	Neoplasm	UNQ9368	0.04047	3.68205	
19088	Neoplasm	CCDC28B	0.017642	3.704768	
19089	Neoplasm	TSHZ3	0.004036	3.708105	
19090	Neoplasm	PBK	0.000001	3.714962	
19091	Neoplasm	DACH1	0	3.730219	
19092	Neoplasm	LOC729983	0	3.744879	
19093	Neoplasm	CLDN2	0.000001	3.758097	
19094	Neoplasm	NOX4	0.0035	3.76034	
19095	Neoplasm	OSMR	0.002305	3.789427	
19096	Neoplasm	TMEM97	0	3.826889	
19097	Neoplasm	PLK1	0	3.856794	
19098	Neoplasm	CEP72	0	3.868512	

19099	Neoplasm	EFNB1	0.043208	3.894275	
19100	Neoplasm	CDCA5	0	3.905083	
19101	Neoplasm	C20orf194	0.003783	3.918744	
19102	Neoplasm	RECQL4	0	3.936322	
19103	Neoplasm	RRM2	0.000829	3.969704	
19104	Neoplasm	PRR7	0	3.97367	
19105	Neoplasm	BUB1	0.000689	3.980634	
19106	Neoplasm	CRISPLD1	0.002722	4.001952	
19107	Neoplasm	C13orf3	0	4.004113	
19108	Neoplasm	S100A14	0.010357	4.07712	
19109	Neoplasm	PMEPA1	0	4.105964	
19110	Neoplasm	CXCL9	0	4.116745	
19111	Neoplasm	RNASE4	0.002448	4.132003	
19112	Neoplasm	C5orf22	0.000215	4.134619	
19113	Neoplasm	PALMD	0.000401	4.163473	
19114	Neoplasm	AGT	0	4.16549	
19115	Neoplasm	HOMER1	0.000221	4.184058	
19116	Neoplasm	FLRT3	0.001996	4.187701	
19117	Neoplasm	GPT2	0	4.188257	
19118	Neoplasm	ANLN	0	4.191852	
19119	Neoplasm	ADAMTS12	0	4.218924	
19120	Neoplasm	SHANK3	0.000088	4.241882	
19121	Neoplasm	KLK12	0	4.257902	
19122	Neoplasm	MDS1	0.019251	4.274456	
19123	Neoplasm	UBE2C	0	4.291768	
19124	Neoplasm	ATP11A	0	4.320655	
19125	Neoplasm	CENPA	0.000102	4.326599	
19126	Neoplasm	CTGF	0.007694	4.35217	
19127	Neoplasm	MAD2L1	0.000242	4.362649	
19128	Neoplasm	MET	0.000367	4.404876	
19129	Neoplasm	HRASLS5	0.015219	4.453144	
19130	Neoplasm	PKDCC	0.003235	4.47494	
19131	Neoplasm	PLSCR1	0.013005	4.549961	
19132	Neoplasm	C5orf13	0.00134	4.615696	
19133	Neoplasm	CRYZ	0.000002	4.621228	
19134	Neoplasm	ST3GAL4	0.012273	4.627901	
19135	Neoplasm	EMCN	0.000005	4.630426	
19136	Neoplasm	LHFP	0.001286	4.637089	
19137	Neoplasm	FJX1	0	4.63841	
19138	Neoplasm	PPARG	0.00703	4.640094	
19139	Neoplasm	SLC29A1	0	4.643131	
19140	Neoplasm	GPR124	0.002825	4.654939	
19141	Neoplasm	MMP10	0	4.667151	
19142	Neoplasm	MDFI	0	4.672014	
19143	Neoplasm	BGN	0	4.679161	
19144	Neoplasm	BCL2L14	0.000398	4.680959	
19145	Neoplasm	LOC100505633	0.002448	4.693542	
19146	Neoplasm	OXR1	0.002833	4.754361	
19147	Neoplasm	DLC1	0.000604	4.787099	
19148	Neoplasm	CXCL10	0	4.8737	

19149	Neoplasm	RAET1L	0.004617	4.928519	
19150	Neoplasm	MRPL39	0.000257	5.00501	
19151	Neoplasm	C1QC	0.001084	5.072804	
19152	Neoplasm	RSPH1	0.012291	5.126456	
19153	Neoplasm	C4orf48	0	5.141066	
19154	Neoplasm	MYBL2	0	5.144589	
19155	Neoplasm	MEX3A	0	5.153724	
19156	Neoplasm	PTGS2	0.001724	5.186053	
19157	Neoplasm	STXBP6	0.002233	5.208208	
19158	Neoplasm	RHOJ	0.003381	5.347863	
19159	Neoplasm	CXCL5	0	5.376774	
19160	Neoplasm	S100P	0.005175	5.387289	
19161	Neoplasm	MS4A6A	0.003928	5.396421	
19162	Neoplasm	IGHG3	0	5.509341	
19163	Neoplasm	SGPP2	0.010821	5.512257	
19164	Neoplasm	TMEM132A	0	5.551164	
19165	Neoplasm	LCN2	0	5.767922	
19166	Neoplasm	RGS16	0	5.773838	
19167	Neoplasm	HTRA1	0.000987	5.780102	
19168	Neoplasm	TCF4	0.00134	5.904301	
19169	Neoplasm	SRSF6	0.000001	6.156758	
19170	Neoplasm	APOL1	0.0141	6.300155	
19171	Neoplasm	ABHD7	0	6.354725	
19172	Neoplasm	MYOCD	0.002407	6.59146	
19173	Neoplasm	LGR4	0.002809	6.622405	
19174	Neoplasm	TGM2	0	6.930905	
19175	Neoplasm	FAR2	0.000226	7.073809	
19176	Neoplasm	IFITM4P	0	7.380925	
19177	Neoplasm	MECOM	0.003866	7.563299	
19178	Neoplasm	GUCY1B3	0.001934	7.625543	
19179	Neoplasm	LOC643100	0.030738	7.826251	
19180	Neoplasm	SALL4	0	8.221887	
19181	Neoplasm	TUBB3	0	8.247184	
19182	Neoplasm	TNFRSF11A	0.004078	8.379459	
19183	Neoplasm	TNS4	0	8.857143	
19184	Neoplasm	AZGP1	0	9.230461	
19185	Neoplasm	APLN	0	9.587494	
19186	Neoplasm	SLFN5	0.014912	9.754985	
19187	Neoplasm	COL12A1	0	9.976386	
19188	Neoplasm	COL8A1	0	10.33022	
19189	Neoplasm	LAMA4	0.000582	10.435952	
19190	Neoplasm	CEACAM3	0.019919	10.675148	
19191	Neoplasm	PITX2	0	11.538284	
19192	Neoplasm	DPP4	0.000314	13.371282	
19193	Neoplasm	LOC100124692	0.000007	17.396142	
19194	Neoplasm	IGFBP7	0.004056	18.961215	
19195	Neoplasm	PTPRR	0.000377	27.455622	
19196	Neoplasm	FAM198B	0.000001	29.031479	
19197	Neoplasm	NAT12	0.000307	32.336224	
19198	Neoplasm	RARRES3	0.002013	42.38824	

19199	Neoplasm	NUDT5	0.000037	376.844444	
19200	Neoplasm Metastasis	SLC26A3	0.000017	-7.67746	
19201	Neoplasm Metastasis	MUC2	0	-7.661883	
19202	Neoplasm Metastasis	DMN	0	-7.500718	
19203	Neoplasm Metastasis	FRMD3	0.000006	-6.633007	
19204	Neoplasm Metastasis	CHGA	0.000004	-6.084005	
19205	Neoplasm Metastasis	DES	0	-6.019378	
19206	Neoplasm Metastasis	LMO3	0	-5.73808	
19207	Neoplasm Metastasis	GSR	0.000011	-5.729375	
19208	Neoplasm Metastasis	CNN1	0	-5.726561	
19209	Neoplasm Metastasis	NTN2L	0.000006	-5.322837	
19210	Neoplasm Metastasis	PDE5A	0	-5.13904	
19211	Neoplasm Metastasis	HLA-C	0.000011	-5.090134	
19212	Neoplasm Metastasis	CWH43	0	-4.72983	
19213	Neoplasm Metastasis	PRPH	0	-4.504848	
19214	Neoplasm Metastasis	HAND1	0	-4.491088	
19215	Neoplasm Metastasis	ST6GALNAC1	0	-4.4897	
19216	Neoplasm Metastasis	CXCL14	0.000133	-4.05581	
19217	Neoplasm Metastasis	THBS4	0	-3.966928	
19218	Neoplasm Metastasis	FAM55A	0	-3.924033	
19219	Neoplasm Metastasis	CASQ2	0	-3.861067	
19220	Neoplasm Metastasis	MFAP5	0	-3.840655	
19221	Neoplasm Metastasis	APEG1	0	-3.817307	
19222	Neoplasm Metastasis	BEST2	0	-3.779822	
19223	Neoplasm Metastasis	BAPX1	0	-3.764069	
19224	Neoplasm Metastasis	LOC340843	0	-3.741315	
19225	Neoplasm Metastasis	PLA2G10	0	-3.722207	
19226	Neoplasm Metastasis	ATP1A2	0	-3.719575	
19227	Neoplasm Metastasis	P2RY14	0	-3.687764	
19228	Neoplasm Metastasis	FBLN1	0.000002	-3.606652	
19229	Neoplasm Metastasis	REG4	0.000059	-3.503367	
19230	Neoplasm Metastasis	WFDC1	0	-3.487965	
19231	Neoplasm Metastasis	C14ORF132	0	-3.474085	
19232	Neoplasm Metastasis	JPH2	0	-3.444662	
19233	Neoplasm Metastasis	KCNMA1	0	-3.420412	
19234	Neoplasm Metastasis	RASL12	0	-3.339297	
19235	Neoplasm Metastasis	SLC4A4	0	-3.289428	
19236	Neoplasm Metastasis	NKX2-3	0	-3.194695	
19237	Neoplasm Metastasis	CLDN8	0	-3.15702	
19238	Neoplasm Metastasis	PRKAR2B	0	-3.118384	
19239	Neoplasm Metastasis	CALD1	0	-3.105043	
19240	Neoplasm Metastasis	SELENBP1	0.000071	-3.066158	
19241	Neoplasm Metastasis	DARC	0.000001	-3.028116	
19242	Neoplasm Metastasis	PSG4	0.004305	-3.006374	
19243	Neoplasm Metastasis	AKR1B10	0.000698	-3.002267	
19244	Neoplasm Metastasis	LRRN2	0	-2.998523	
19245	Neoplasm Metastasis	HOXD11	0	-2.977418	
19246	Neoplasm Metastasis	MKKS	0.003646	-2.939498	
19247	Neoplasm Metastasis	VMD2L1	0.000134	-2.885943	
19248	Neoplasm Metastasis	SSPN	0	-2.865316	

19249	Neoplasm Metastasis	GCG	0	-2.841612	
19250	Neoplasm Metastasis	OGDH	0.000409	-2.824938	
19251	Neoplasm Metastasis	TOX	0	-2.822165	
19252	Neoplasm Metastasis	GPA33	0.000665	-2.816748	
19253	Neoplasm Metastasis	VWF	0.000708	-2.797215	
19254	Neoplasm Metastasis	SAHH3	0.000006	-2.796622	
19255	Neoplasm Metastasis	CA12	0.000281	-2.753664	
19256	Neoplasm Metastasis	ZFYVE1	0	-2.721154	
19257	Neoplasm Metastasis	HSD11B2	0.000411	-2.707869	
19258	Neoplasm Metastasis	OLFML2A	0	-2.701749	
19259	Neoplasm Metastasis	IGHA1	0.000001	-2.6695	
19260	Neoplasm Metastasis	TWSG1	0	-2.659835	
19261	Neoplasm Metastasis	SPON1	0.000432	-2.646048	
19262	Neoplasm Metastasis	CAPN9	0.000016	-2.643441	
19263	Neoplasm Metastasis	LMOD1	0	-2.638127	
19264	Neoplasm Metastasis	CCL13	0.000261	-2.615183	
19265	Neoplasm Metastasis	CPA3	0	-2.60624	
19266	Neoplasm Metastasis	VCL	0	-2.583848	
19267	Neoplasm Metastasis	PTGER2	0	-2.583267	
19268	Neoplasm Metastasis	STON1	0	-2.562703	
19269	Neoplasm Metastasis	FHL1	0	-2.554559	
19270	Neoplasm Metastasis	DACT3	0	-2.549765	
19271	Neoplasm Metastasis	FLJ21511	0	-2.545136	
19272	Neoplasm Metastasis	GREM1	0	-2.542547	
19273	Neoplasm Metastasis	HSPA2	0	-2.541351	
19274	Neoplasm Metastasis	AGR3	0.000704	-2.508426	
19275	Neoplasm Metastasis	FOXF2	0	-2.48074	
19276	Neoplasm Metastasis	NR3C2	0	-2.469881	
19277	Neoplasm Metastasis	SLCO2A1	0.000002	-2.469144	
19278	Neoplasm Metastasis	CHL1	0	-2.465884	
19279	Neoplasm Metastasis	ELOVL6	0	-2.453167	
19280	Neoplasm Metastasis	TMIGD1	0	-2.446872	
19281	Neoplasm Metastasis	SERTAD4	0	-2.433668	
19282	Neoplasm Metastasis	MATN2	0	-2.427093	
19283	Neoplasm Metastasis	WNT5A	0	-2.425019	
19284	Neoplasm Metastasis	AGR2	0.000531	-2.405779	
19285	Neoplasm Metastasis	KIAA0828	0	-2.389916	
19286	Neoplasm Metastasis	ABP1	0.000179	-2.385219	
19287	Neoplasm Metastasis	PGM5	0.000314	-2.364494	
19288	Neoplasm Metastasis	DIO2	0	-2.356839	
19289	Neoplasm Metastasis	ARHGAP1	0	-2.348833	
19290	Neoplasm Metastasis	SCARA5	0.000025	-2.332101	
19291	Neoplasm Metastasis	MYO1A	0.000642	-2.309044	
19292	Neoplasm Metastasis	INSL5	0	-2.298135	
19293	Neoplasm Metastasis	NNAT	0	-2.292745	
19294	Neoplasm Metastasis	HMGCR	0.000015	-2.289966	
19295	Neoplasm Metastasis	SCNN1B	0.000177	-2.287546	
19296	Neoplasm Metastasis	KLF4	0	-2.276513	
19297	Neoplasm Metastasis	C15orf48	0.000105	-2.264361	
19298	Neoplasm Metastasis	FRZB	0.000071	-2.263728	

19299	Neoplasm Metastasis	BTNL3	0.000071	-2.24713	
19300	Neoplasm Metastasis	MS4A8B	0.000135	-2.245197	
19301	Neoplasm Metastasis	DDR2	0	-2.222107	
19302	Neoplasm Metastasis	RGMA	0.000001	-2.216106	
19303	Neoplasm Metastasis	BVES	0	-2.211583	
19304	Neoplasm Metastasis	C5ORF5	0	-2.20954	
19305	Neoplasm Metastasis	AOC3	0	-2.202195	
19306	Neoplasm Metastasis	TMIGD	0.000335	-2.196593	
19307	Neoplasm Metastasis	NLGN4X	0	-2.184679	
19308	Neoplasm Metastasis	ZMIZ1	0	-2.174006	
19309	Neoplasm Metastasis	HERC1	0.00032	-2.164396	
19310	Neoplasm Metastasis	CTSG	0.000001	-2.15913	
19311	Neoplasm Metastasis	SYNC1	0	-2.154301	
19312	Neoplasm Metastasis	DRD1IP	0	-2.152948	
19313	Neoplasm Metastasis	OXCT1	0	-2.149785	
19314	Neoplasm Metastasis	BMP2	0	-2.148424	
19315	Neoplasm Metastasis	TSPAN7	0.000705	-2.148268	
19316	Neoplasm Metastasis	LRCH2	0	-2.1482	
19317	Neoplasm Metastasis	SOX15	0	-2.145645	
19318	Neoplasm Metastasis	LAMA1	0	-2.144402	
19319	Neoplasm Metastasis	TMEM54	0.000211	-2.141421	
19320	Neoplasm Metastasis	TAGLN	0.003011	-2.13702	
19321	Neoplasm Metastasis	PDE4D	0	-2.135113	
19322	Neoplasm Metastasis	Q8NBX4	0.000102	-2.127648	
19323	Neoplasm Metastasis	NSG1	0	-2.121418	
19324	Neoplasm Metastasis	DIXDC1	0	-2.11124	
19325	Neoplasm Metastasis	PLAT	0.000028	-2.107739	
19326	Neoplasm Metastasis	PBK	0	-2.096207	
19327	Neoplasm Metastasis	SYNC	0	-2.093807	
19328	Neoplasm Metastasis	HSPB3	0	-2.092056	
19329	Neoplasm Metastasis	CSRP1	0.000153	-2.081023	
19330	Neoplasm Metastasis	RPRM	0	-2.078294	
19331	Neoplasm Metastasis	ADSV	0.000576	-2.070961	
19332	Neoplasm Metastasis	HAPLN1	0	-2.070549	
19333	Neoplasm Metastasis	CD79A	0.000427	-2.069078	
19334	Neoplasm Metastasis	SCUBE2	0	-2.068644	
19335	Neoplasm Metastasis	TPM1	0	-2.067855	
19336	Neoplasm Metastasis	PTCHD1	0	-2.064622	
19337	Neoplasm Metastasis	LOH11CR2A	0.003159	-2.056708	
19338	Neoplasm Metastasis	PDZRN4	0	-2.044879	
19339	Neoplasm Metastasis	NUDT6	0	-2.043593	
19340	Neoplasm Metastasis	ITIH5	0	-2.029161	
19341	Neoplasm Metastasis	EXOC5	0	-2.027885	
19342	Neoplasm Metastasis	ZMYND11	0	-2.02749	
19343	Neoplasm Metastasis	SNAP23	0	-2.011665	
19344	Neoplasm Metastasis	PRRT2	0	-2.008478	
19345	Neoplasm Metastasis	MALL	0.00058	-2.001755	
19346	Neoplasm Metastasis	H19	0.002505	2.028469	
19347	Neoplasm Metastasis	C2	0.000472	2.039869	
19348	Neoplasm Metastasis	CKLF	0	2.057666	

19349	Neoplasm Metastasis	CCDC93	0	2.078317	
19350	Neoplasm Metastasis	KLF7	0.000006	2.092401	
19351	Neoplasm Metastasis	TMEM49	0	2.095377	
19352	Neoplasm Metastasis	USP34	0.000005	2.109417	
19353	Neoplasm Metastasis	SERINC5	0.000004	2.188872	
19354	Neoplasm Metastasis	EML4	0	2.191544	
19355	Neoplasm Metastasis	MSLN	0.002114	2.196121	
19356	Neoplasm Metastasis	ARHGEF2	0.000016	2.197076	
19357	Neoplasm Metastasis	OLR1	0.000557	2.207088	
19358	Neoplasm Metastasis	MPHOSPH8	0.000004	2.214199	
19359	Neoplasm Metastasis	COX6C	0	2.244844	
19360	Neoplasm Metastasis	LOC440589	0	2.249777	
19361	Neoplasm Metastasis	DGKH	0.000001	2.302271	
19362	Neoplasm Metastasis	LOC727820	0	2.307956	
19363	Neoplasm Metastasis	MGC16384	0.000006	2.346782	
19364	Neoplasm Metastasis	WDR12	0	2.379028	
19365	Neoplasm Metastasis	COL1A1	0	2.395517	
19366	Neoplasm Metastasis	CSNK1A1	0.000004	2.40641	
19367	Neoplasm Metastasis	NRBP2	0	2.409324	
19368	Neoplasm Metastasis	CBS	0.000703	2.411396	
19369	Neoplasm Metastasis	ADFP	0.00024	2.429958	
19370	Neoplasm Metastasis	ANGPTL4	0.000173	2.440066	
19371	Neoplasm Metastasis	ADAMTSL2	0.000012	2.453786	
19372	Neoplasm Metastasis	CYBA	0	2.455506	
19373	Neoplasm Metastasis	RPL29	0	2.488888	
19374	Neoplasm Metastasis	CLK1	0.000007	2.490114	
19375	Neoplasm Metastasis	SFRS18	0	2.509521	
19376	Neoplasm Metastasis	CYP1B1	0.000314	2.525663	
19377	Neoplasm Metastasis	EIF3B	0.000014	2.53144	
19378	Neoplasm Metastasis	NSFL1C	0.000011	2.537637	
19379	Neoplasm Metastasis	CTSB	0	2.543662	
19380	Neoplasm Metastasis	C4orf30	0.000017	2.621492	
19381	Neoplasm Metastasis	ALDH4A1	0.000232	2.672146	
19382	Neoplasm Metastasis	FOXJ3	0.000019	2.674279	
19383	Neoplasm Metastasis	CGNL1	0.000004	2.694607	
19384	Neoplasm Metastasis	RP11-298P3.3	0.000001	2.733297	
19385	Neoplasm Metastasis	CFHL1	0.002892	2.741774	
19386	Neoplasm Metastasis	AKAP8L	0.000016	2.772168	
19387	Neoplasm Metastasis	FNBP4	0	2.773925	
19388	Neoplasm Metastasis	ZNF83	0.000021	2.8262	
19389	Neoplasm Metastasis	AZGP1	0.002972	2.844943	
19390	Neoplasm Metastasis	VPS13C	0.000001	2.860054	
19391	Neoplasm Metastasis	TRIF	0.000391	2.916183	
19392	Neoplasm Metastasis	LOC26010	0.000013	2.919209	
19393	Neoplasm Metastasis	CFI	0.000174	2.93439	
19394	Neoplasm Metastasis	INHBE	0.000642	2.971835	
19395	Neoplasm Metastasis	TM4SF4	0.000002	2.983023	
19396	Neoplasm Metastasis	RPL38	0	3.002224	
19397	Neoplasm Metastasis	YAP1	0.000001	3.066516	
19398	Neoplasm Metastasis	ZNF207	0.000004	3.084116	

19399	Neoplasm Metastasis	C7orf54	0.000012	3.097033	
19400	Neoplasm Metastasis	ZNF364	0.000009	3.107203	
19401	Neoplasm Metastasis	LOC441775	0	3.107298	
19402	Neoplasm Metastasis	F5	0.00014	3.231423	
19403	Neoplasm Metastasis	BF	0.000076	3.280719	
19404	Neoplasm Metastasis	RBM39	0.000001	3.303166	
19405	Neoplasm Metastasis	CDH2	0	3.362404	
19406	Neoplasm Metastasis	MGC23985	0.000004	3.382113	
19407	Neoplasm Metastasis	HABP2	0.000351	3.485311	
19408	Neoplasm Metastasis	U2AF1	0.000002	3.513583	
19409	Neoplasm Metastasis	CHD2	0.00001	3.596148	
19410	Neoplasm Metastasis	THBS2	0	3.628814	
19411	Neoplasm Metastasis	VNN1	0.000533	3.670827	
19412	Neoplasm Metastasis	GRINA	0	3.747434	
19413	Neoplasm Metastasis	SYK	0.000006	3.751209	
19414	Neoplasm Metastasis	APOE	0	3.797515	
19415	Neoplasm Metastasis	AQP9	0.000053	3.849158	
19416	Neoplasm Metastasis	HSD11B1	0.000121	3.932743	
19417	Neoplasm Metastasis	GTF3A	0.000012	3.956153	
19418	Neoplasm Metastasis	TBRG1	0	4.005367	
19419	Neoplasm Metastasis	SERF2	0	4.007006	
19420	Neoplasm Metastasis	TXN	0	4.097941	
19421	Neoplasm Metastasis	SNAPC3	0.000002	4.188509	
19422	Neoplasm Metastasis	CCNL1	0.000021	4.215768	
19423	Neoplasm Metastasis	ASGR2	0.000553	4.302714	
19424	Neoplasm Metastasis	SERPINA5	0.000352	4.345646	
19425	Neoplasm Metastasis	C11orf58	0.000007	4.378468	
19426	Neoplasm Metastasis	ALDOB	0.001493	4.480913	
19427	Neoplasm Metastasis	SMURF2	0.000012	4.524036	
19428	Neoplasm Metastasis	GLUD1	0.000014	4.578576	
19429	Neoplasm Metastasis	ZNF785	0.000008	4.674523	
19430	Neoplasm Metastasis	SULT2A1	0.000605	4.720173	
19431	Neoplasm Metastasis	FMO3	0.000071	4.720673	
19432	Neoplasm Metastasis	RPS15A	0	4.778026	
19433	Neoplasm Metastasis	IFNGR1	0	4.786103	
19434	Neoplasm Metastasis	SERPINF2	0.00067	4.841546	
19435	Neoplasm Metastasis	PCF11	0.000007	4.990015	
19436	Neoplasm Metastasis	PICALM	0.000013	5.138198	
19437	Neoplasm Metastasis	RPL35A	0	5.301532	
19438	Neoplasm Metastasis	CP	0.00038	5.509002	
19439	Neoplasm Metastasis	CPB2	0.000432	5.613978	
19440	Neoplasm Metastasis	KIAA0907	0.000007	5.62551	
19441	Neoplasm Metastasis	C3	0.000006	5.668511	
19442	Neoplasm Metastasis	RPS17	0	5.675517	
19443	Neoplasm Metastasis	ZNF638	0	5.912399	
19444	Neoplasm Metastasis	SLC13A5	0.000331	6.076943	
19445	Neoplasm Metastasis	C8A	0.000531	6.120991	
19446	Neoplasm Metastasis	LOC440354	0.000014	6.198988	
19447	Neoplasm Metastasis	RBP4	0.000557	6.658636	
19448	Neoplasm Metastasis	ASGR1	0.000048	6.679332	

19449	Neoplasm Metastasis	C5	0.00011	6.687382	
19450	Neoplasm Metastasis	APOH	0.000429	6.75181	
19451	Neoplasm Metastasis	FCN3	0.000009	6.844049	
19452	Neoplasm Metastasis	APOC2	0.000051	6.904064	
19453	Neoplasm Metastasis	ZMYND8	0.000009	6.92901	
19454	Neoplasm Metastasis	AGXT	0.000773	6.935567	
19455	Neoplasm Metastasis	HPD	0.000531	7.014538	
19456	Neoplasm Metastasis	ITIH2	0.000149	7.015858	
19457	Neoplasm Metastasis	C6	0.00046	7.054535	
19458	Neoplasm Metastasis	SAA1	0.000035	7.064054	
19459	Neoplasm Metastasis	SAA4	0.000529	7.065	
19460	Neoplasm Metastasis	MBNL2	0	7.194229	
19461	Neoplasm Metastasis	PLG	0.000498	7.361402	
19462	Neoplasm Metastasis	MEGF6	0.000022	7.507018	
19463	Neoplasm Metastasis	ITIH1	0.000337	7.554118	
19464	Neoplasm Metastasis	C4BPA	0.000535	7.948833	
19465	Neoplasm Metastasis	APOC1	0.000001	8.000367	
19466	Neoplasm Metastasis	TTR	0.000676	8.109081	
19467	Neoplasm Metastasis	CYP2C8	0.000673	8.207707	
19468	Neoplasm Metastasis	LBP	0.000127	8.984359	
19469	Neoplasm Metastasis	HAMP	0.000059	9.033264	
19470	Neoplasm Metastasis	KIAA0368	0	9.302099	
19471	Neoplasm Metastasis	APCS	0.000082	9.596353	
19472	Neoplasm Metastasis	XPO1	0.000002	10.357944	
19473	Neoplasm Metastasis	KNG1	0.000331	10.388199	
19474	Neoplasm Metastasis	HRG	0.000312	11.044669	
19475	Neoplasm Metastasis	CFLAR	0.000011	11.328732	
19476	Neoplasm Metastasis	SERPINC1	0.000285	11.791284	
19477	Neoplasm Metastasis	SERPINA3	0.000001	11.942192	
19478	Neoplasm Metastasis	APOB	0.000088	13.0809	
19479	Neoplasm Metastasis	AHSG	0.000112	13.371622	
19480	Neoplasm Metastasis	APOC3	0.000181	13.701454	
19481	Neoplasm Metastasis	HPX	0.000192	14.287143	
19482	Neoplasm Metastasis	FGG	0.000014	14.651355	
19483	Neoplasm Metastasis	GART	0.000001	15.363893	
19484	Neoplasm Metastasis	F2	0.000047	15.443368	
19485	Neoplasm Metastasis	VTN	0.000044	15.844447	
19486	Neoplasm Metastasis	ORM2	0.00009	16.094898	
19487	Neoplasm Metastasis	CYP2E1	0.000072	16.227626	
19488	Neoplasm Metastasis	ZBTB20	0.000004	17.367361	
19489	Neoplasm Metastasis	ZFAND6	0.000004	17.449033	
19490	Neoplasm Metastasis	PIAS1	0.000009	18.056731	
19491	Neoplasm Metastasis	FGL1	0.000039	18.32827	
19492	Neoplasm Metastasis	TF	0.000068	18.608249	
19493	Neoplasm Metastasis	ASXL1	0	20.752404	
19494	Neoplasm Metastasis	GC	0.000013	20.947027	
19495	Neoplasm Metastasis	AMBP	0.000021	21.099729	
19496	Neoplasm Metastasis	APOA2	0.000023	21.365529	
19497	Neoplasm Metastasis	ALB	0.000007	23.019959	
19498	Neoplasm Metastasis	APOA1	0.000018	23.169381	

19499	Neoplasm Metastasis	UBC	0.000009	23.283036	
19500	Neoplasm Metastasis	FGB	0.000008	23.984148	
19501	Neoplasm Metastasis	FGA	0.000008	25.158992	
19502	Neoplasm Metastasis	ORM1	0.000008	25.514711	
19503	Neoplasm Metastasis	SLC22A3	0	141.230932	
19504	Neoplasm Metastasis	PHKB	0	201.69213	
19505	Normal colon	KLK10	0	-32.769	
19506	Normal colon	PRMT3	0	-18.97913	
19507	Normal colon	SLC6A6	0	-13.84193	
19508	Normal colon	EIF3B	0	-12.407213	
19509	Normal colon	SLC39A10	0	-9.756089	
19510	Normal colon	SPP1	0	-9.715651	
19511	Normal colon	INHBA	0	-9.354067	
19512	Normal colon	SULT2B1	0	-9.136537	
19513	Normal colon	C9orf140	0	-8.873791	
19514	Normal colon	THBS2	0	-8.219835	
19515	Normal colon	GINS1	0	-7.92122	
19516	Normal colon	CASC5	0	-7.794743	
19517	Normal colon	FNBP4	0	-7.396388	
19518	Normal colon	MMP7	0	-7.340662	
19519	Normal colon	EFNA3	0	-7.271941	
19520	Normal colon	ASPM	0	-6.78092	
19521	Normal colon	YAP1	0	-6.763694	
19522	Normal colon	KIAA1333	0	-6.690645	
19523	Normal colon	CYB5B	0	-6.558687	
19524	Normal colon	HMMR	0.000025	-5.804377	
19525	Normal colon	CTHRC1	0	-5.556118	
19526	Normal colon	TDGF1	0	-5.512866	
19527	Normal colon	BMP7	0	-5.509887	
19528	Normal colon	WNT2	0.029803	-5.494799	
19529	Normal colon	LGR5	0	-5.401572	
19530	Normal colon	ATAD2	0	-5.257683	
19531	Normal colon	PSAT1	0	-5.234669	
19532	Normal colon	SP5	0	-5.135378	
19533	Normal colon	CXCL3	0	-5.112398	
19534	Normal colon	WDR72	0	-4.874852	
19535	Normal colon	SULF1	0	-4.791261	
19536	Normal colon	TPR	0.040144	-4.597873	
19537	Normal colon	BGN	0	-4.573511	
19538	Normal colon	IL8	0	-4.462238	
19539	Normal colon	PEO1	0	-4.420263	
19540	Normal colon	GDF15	0	-4.402386	
19541	Normal colon	MSX2	0	-4.390581	
19542	Normal colon	CELSR3	0	-4.276832	
19543	Normal colon	RGS5	0.012324	-4.256989	
19544	Normal colon	MGC16384	0	-4.17907	
19545	Normal colon	CKS2	0	-4.154271	
19546	Normal colon	FABP6	0	-4.123797	
19547	Normal colon	XPOT	0.014459	-3.992622	
19548	Normal colon	AXIN2	0	-3.962	

19549	Normal colon	PDCD2L	0	-3.921773	
19550	Normal colon	E2F5	0.005272	-3.890783	
19551	Normal colon	CCDC86	0.002201	-3.867938	
19552	Normal colon	RELL2	0.003759	-3.855649	
19553	Normal colon	RANBP5	0	-3.845853	
19554	Normal colon	B3GALTL	0.049356	-3.840546	
19555	Normal colon	DDIT4	0	-3.770709	
19556	Normal colon	CXCL1	0	-3.733754	
19557	Normal colon	WDSOF1	0.001352	-3.709982	
19558	Normal colon	COL5A2	0	-3.67529	
19559	Normal colon	COL8A1	0	-3.671237	
19560	Normal colon	IFITM2	0.026998	-3.630915	
19561	Normal colon	NEBL	0	-3.58656	
19562	Normal colon	CEP55	0	-3.572883	
19563	Normal colon	RAET1L	0	-3.565401	
19564	Normal colon	CGREF1	0	-3.556432	
19565	Normal colon	CDC6	0.011855	-3.551337	
19566	Normal colon	ZNF703	0.014334	-3.548477	
19567	Normal colon	THY1	0	-3.542619	
19568	Normal colon	VSNL1	0	-3.514441	
19569	Normal colon	CST1	0	-3.501851	
19570	Normal colon	CCL4L1	0.029803	-3.423804	
19571	Normal colon	PTP4A3	0.000168	-3.409801	
19572	Normal colon	PLEKHG4	0	-3.370452	
19573	Normal colon	FANCI	0.0087	-3.309157	
19574	Normal colon	TPD52L1	0	-3.298483	
19575	Normal colon	BUB1	0.003759	-3.279526	
19576	Normal colon	CTSK	0.016201	-3.25472	
19577	Normal colon	C13orf3	0.010117	-3.221261	
19578	Normal colon	COL3A1	0	-3.185474	
19579	Normal colon	SERPINE2	0	-3.167968	
19580	Normal colon	HIST3H2A	0	-3.152904	
19581	Normal colon	GUCY1A3	0.036003	-3.146832	
19582	Normal colon	LY6E	0	-3.111251	
19583	Normal colon	C13orf23	0	-3.103547	
19584	Normal colon	TRIP13	0	-3.030456	
19585	Normal colon	MET	0	-3.021002	
19586	Normal colon	POLR1D	0	-3.015749	
19587	Normal colon	HRASLS3	0	-2.971828	
19588	Normal colon	POLR1C	0	-2.968407	
19589	Normal colon	GALNT6	0	-2.96054	
19590	Normal colon	COMP	0	-2.956944	
19591	Normal colon	TGS1	0.016333	-2.947857	
19592	Normal colon	URG4	0	-2.94292	
19593	Normal colon	GCLM	0.03042	-2.916423	
19594	Normal colon	MYH10	0	-2.889597	
19595	Normal colon	MCM8	0.004563	-2.888232	
19596	Normal colon	PRR7	0	-2.887817	
19597	Normal colon	SORD	0	-2.881162	
19598	Normal colon	C2	0	-2.869311	

19599	Normal colon	NUPL1	0	-2.849538	
19600	Normal colon	ASPN	0	-2.844331	
19601	Normal colon	MTERFD1	0	-2.84095	
19602	Normal colon	OLR1	0	-2.810905	
19603	Normal colon	LUM	0.005607	-2.802796	
19604	Normal colon	DPEP1	0	-2.791883	
19605	Normal colon	PDZK1IP1	0	-2.788647	
19606	Normal colon	FAP	0	-2.767748	
19607	Normal colon	NOL5A	0	-2.751127	
19608	Normal colon	CLIC3	0.000063	-2.704234	
19609	Normal colon	TNS4	0	-2.669075	
19610	Normal colon	SRPX2	0	-2.667525	
19611	Normal colon	PTK7	0	-2.657063	
19612	Normal colon	RP5-1022P6.2	0.036178	-2.655837	
19613	Normal colon	RBM4	0	-2.651058	
19614	Normal colon	UBE2V1	0.004279	-2.625998	
19615	Normal colon	FJX1	0	-2.622869	
19616	Normal colon	VEGFA	0	-2.615163	
19617	Normal colon	EIF2C2	0	-2.602762	
19618	Normal colon	LRP8	0	-2.599647	
19619	Normal colon	CAD	0	-2.598895	
19620	Normal colon	KIF5B	0.04388	-2.59278	
19621	Normal colon	MTHFD1L	0	-2.58207	
19622	Normal colon	IFI6	0	-2.565786	
19623	Normal colon	NOX4	0	-2.545509	
19624	Normal colon	SLC7A6	0.005899	-2.538138	
19625	Normal colon	GPR56	0	-2.513165	
19626	Normal colon	AMIGO2	0.000027	-2.510275	
19627	Normal colon	CDCA5	0	-2.498019	
19628	Normal colon	RPL35A	0.023557	-2.488761	
19629	Normal colon	TMEM132A	0	-2.455723	
19630	Normal colon	BLCAP	0.018234	-2.455585	
19631	Normal colon	ALG11	0.030134	-2.45445	
19632	Normal colon	RAD54B	0	-2.430731	
19633	Normal colon	ATP11A	0	-2.429617	
19634	Normal colon	CSPP1	0.004279	-2.417897	
19635	Normal colon	TNFRSF11B	0.000263	-2.412846	
19636	Normal colon	RSF1	0.025728	-2.405661	
19637	Normal colon	CCND1	0	-2.37772	
19638	Normal colon	SLC29A1	0	-2.371082	
19639	Normal colon	PAFAH1B3	0	-2.368768	
19640	Normal colon	TMEM63A	0	-2.36793	
19641	Normal colon	PLS3	0	-2.366803	
19642	Normal colon	RGS16	0	-2.362545	
19643	Normal colon	PCSK9	0	-2.346258	
19644	Normal colon	C13orf27	0.000057	-2.341832	
19645	Normal colon	WDR5	0	-2.33504	
19646	Normal colon	ProSAPiP1	0	-2.329098	
19647	Normal colon	DARS2	0.003721	-2.328628	
19648	Normal colon	GPSM2	0	-2.323712	

19649	Normal colon	PCID2	0	-2.322111	
19650	Normal colon	HTRA1	0	-2.313181	
19651	Normal colon	GPR160	0.025625	-2.310118	
19652	Normal colon	CCDC16	0.019808	-2.305799	
19653	Normal colon	CBFB	0	-2.28716	
19654	Normal colon	PLAU	0	-2.274784	
19655	Normal colon	COMT	0.0087	-2.27312	
19656	Normal colon	ETS2	0.021123	-2.272741	
19657	Normal colon	CDC16	0.036178	-2.268282	
19658	Normal colon	KIAA0406	0	-2.26134	
19659	Normal colon	S100A11	0	-2.260263	
19660	Normal colon	TMEM206	0.000151	-2.251382	
19661	Normal colon	EXOSC8	0.023557	-2.246594	
19662	Normal colon	CDC25B	0	-2.23736	
19663	Normal colon	CELSR1	0	-2.236188	
19664	Normal colon	RAI14	0.000238	-2.235023	
19665	Normal colon	LIMK1	0	-2.232682	
19666	Normal colon	OLFML2B	0	-2.23075	
19667	Normal colon	IL1RN	0	-2.22198	
19668	Normal colon	EXOSC4	0	-2.219448	
19669	Normal colon	SNX10	0	-2.214407	
19670	Normal colon	MAFG	0.020108	-2.213302	
19671	Normal colon	KCNN4	0	-2.189989	
19672	Normal colon	POFUT1	0	-2.187818	
19673	Normal colon	SFRS3	0.023214	-2.178891	
19674	Normal colon	PRRX1	0	-2.173188	
19675	Normal colon	PLXNA1	0	-2.172769	
19676	Normal colon	LOC90835	0.001125	-2.166442	
19677	Normal colon	ACTR3B	0	-2.164008	
19678	Normal colon	TTC17	0.029697	-2.1624	
19679	Normal colon	C6orf26	0	-2.161049	
19680	Normal colon	EVPL	0	-2.137785	
19681	Normal colon	RIPK2	0	-2.131233	
19682	Normal colon	TPX2	0	-2.124068	
19683	Normal colon	IRF2BP2	0.029751	-2.121326	
19684	Normal colon	CBX8	0	-2.120196	
19685	Normal colon	MORC4	0.004279	-2.112565	
19686	Normal colon	RUNX1	0	-2.112467	
19687	Normal colon	CALU	0	-2.106678	
19688	Normal colon	NOC2L	0.026594	-2.104099	
19689	Normal colon	IGFBP7	0	-2.10232	
19690	Normal colon	DUSP14	0	-2.098726	
19691	Normal colon	NOL8	0	-2.07615	
19692	Normal colon	DCUN1D5	0	-2.073674	
19693	Normal colon	IRAK2	0	-2.070172	
19694	Normal colon	FAM60A	0	-2.066113	
19695	Normal colon	TCFL5	0	-2.061709	
19696	Normal colon	PFDN4	0	-2.052213	
19697	Normal colon	IMPDH1	0	-2.052053	
19698	Normal colon	NOP5/NOP58	0	-2.050744	

19699	Normal colon	SNHG1	0	-2.049278	
19700	Normal colon	NUP107	0	-2.047995	
19701	Normal colon	EEF1E1	0	-2.044514	
19702	Normal colon	FOXM1	0	-2.043947	
19703	Normal colon	POLR2B	0.030828	-2.04259	
19704	Normal colon	STK3	0	-2.036381	
19705	Normal colon	BCL2L1	0	-2.036074	
19706	Normal colon	MIF	0	-2.035911	
19707	Normal colon	NUFIP1	0	-2.035198	
19708	Normal colon	GNL3	0	-2.032758	
19709	Normal colon	RBCK1	0	-2.028985	
19710	Normal colon	C19orf48	0	-2.027782	
19711	Normal colon	TM16F	0	-2.023765	
19712	Normal colon	HM13	0	-2.021461	
19713	Normal colon	STC1	0	-2.018631	
19714	Normal colon	CNIH4	0	-2.006838	
19715	Normal colon	NPM1	0	-2.004808	
19716	Normal colon	LDHA	0.020558	-1.827477	
19717	Normal colon	LASS4	0.035981	2.000508	
19718	Normal colon	LOC100289019	0.000053	2.00295	
19719	Normal colon	SGK2	0.000043	2.008301	
19720	Normal colon	LRRFIP2	0.000067	2.011938	
19721	Normal colon	MTUS1	0.000071	2.012135	
19722	Normal colon	CLDN18	0.024964	2.01326	
19723	Normal colon	ILDR1	0	2.015816	
19724	Normal colon	ECH1	0	2.02837	
19725	Normal colon	IMPA2	0	2.033531	
19726	Normal colon	C22orf16	0	2.041015	
19727	Normal colon	HIBCH	0.000127	2.041443	
19728	Normal colon	S100A14	0.000183	2.042127	
19729	Normal colon	ABHD3	0.000238	2.042615	
19730	Normal colon	CMAS	0.000052	2.042658	
19731	Normal colon	BRP44L	0.000027	2.046297	
19732	Normal colon	PPARGC1A	0	2.050503	
19733	Normal colon	ECHDC2	0.031098	2.052193	
19734	Normal colon	BCAR3	0.00004	2.053906	
19735	Normal colon	FXYD1	0.000047	2.055213	
19736	Normal colon	MAN2C1	0.035766	2.05561	
19737	Normal colon	UGP2	0.00004	2.056388	
19738	Normal colon	SHPK	0.000083	2.058742	
19739	Normal colon	GIPC1	0.040276	2.065751	
19740	Normal colon	CD34	0.014703	2.066563	
19741	Normal colon	ACAT1	0.000077	2.066848	
19742	Normal colon	ATG4A	0.000012	2.067547	
19743	Normal colon	CALM1	0.00025	2.069654	
19744	Normal colon	KRT8	0	2.070414	
19745	Normal colon	P2RX1	0.039725	2.07815	
19746	Normal colon	CPT1B	0.039004	2.086474	
19747	Normal colon	CI152	0	2.091124	
19748	Normal colon	CASD1	0.00029	2.091419	

19749	Normal colon	PDSS2	0.000052	2.099445	
19750	Normal colon	PCDHA6	0.000109	2.10056	
19751	Normal colon	GLCCI1	0.000282	2.101006	
19752	Normal colon	ATP5S	0.000026	2.103186	
19753	Normal colon	MRPL35	0.000297	2.11346	
19754	Normal colon	GLOD5	0.000271	2.113893	
19755	Normal colon	PLD1	0.000135	2.115702	
19756	Normal colon	MAP7D3	0.028966	2.11873	
19757	Normal colon	C14orf159	0.000015	2.122629	
19758	Normal colon	TNFSF10	0	2.123927	
19759	Normal colon	PIGR	0	2.126518	
19760	Normal colon	STAP2	0.000189	2.132629	
19761	Normal colon	UQCRC1	0.000063	2.132919	
19762	Normal colon	SLC25A20	0.01864	2.133911	
19763	Normal colon	CD27	0	2.134498	
19764	Normal colon	FAM107B	0.042246	2.138452	
19765	Normal colon	TFG	0.014334	2.139638	
19766	Normal colon	SPIB	0	2.141577	
19767	Normal colon	CC50B	0	2.14326	
19768	Normal colon	TMEM30B	0.031638	2.151088	
19769	Normal colon	C9orf125	0.000112	2.151976	
19770	Normal colon	MYO1D	0.000211	2.153935	
19771	Normal colon	CDS1	0.000028	2.155889	
19772	Normal colon	MARVELD3	0	2.156015	
19773	Normal colon	P2RX4	0	2.1678	
19774	Normal colon	GBA2	0.000285	2.169336	
19775	Normal colon	ANTXR2	0.033986	2.172566	
19776	Normal colon	FKBP5	0.000164	2.181115	
19777	Normal colon	LIMA1	0.000009	2.19754	
19778	Normal colon	GALM	0.000162	2.201462	
19779	Normal colon	FZD5	0.000266	2.202912	
19780	Normal colon	FLJ36848	0.000256	2.20681	
19781	Normal colon	ANO10	0.000053	2.207056	
19782	Normal colon	SLC9A3R1	0	2.208417	
19783	Normal colon	MGC45438	0	2.209773	
19784	Normal colon	MADCAM1	0.020182	2.213199	
19785	Normal colon	ATP1B2	0.009431	2.215758	
19786	Normal colon	C6orf136	0.000023	2.216746	
19787	Normal colon	ULA4	0	2.22407	
19788	Normal colon	FAM149A	0.000141	2.224178	
19789	Normal colon	CASZ1	0.022152	2.233026	
19790	Normal colon	PEX26	0.000241	2.233614	
19791	Normal colon	ITGAL	0.03857	2.238915	
19792	Normal colon	LOC553137	0.029803	2.241503	
19793	Normal colon	FLNB	0.000297	2.244974	
19794	Normal colon	L3MBTL4	0.040681	2.245339	
19795	Normal colon	FAM107A	0.000228	2.246606	
19796	Normal colon	SHD	0.000098	2.248522	
19797	Normal colon	PVRL3	0.034791	2.248662	
19798	Normal colon	SEMA6D	0	2.250367	

19799	Normal colon	PRDX6	0.000048	2.2515	
19800	Normal colon	LOC92482	0.018452	2.256469	
19801	Normal colon	KRT19P2	0.000135	2.260004	
19802	Normal colon	VPS13D	0.039004	2.263204	
19803	Normal colon	D2HGDH	0.03042	2.266187	
19804	Normal colon	PCSK7	0.018234	2.267915	
19805	Normal colon	SLC35D1	0.000296	2.279274	
19806	Normal colon	GLIPR2	0.000116	2.281672	
19807	Normal colon	KBTBD11	0.000046	2.294318	
19808	Normal colon	IDH3A	0.000023	2.296197	
19809	Normal colon	SLC44A1	0.000107	2.300155	
19810	Normal colon	LETM1	0.000028	2.307655	
19811	Normal colon	ALDH6A1	0.000229	2.310912	
19812	Normal colon	APPL2	0.000084	2.313575	
19813	Normal colon	SVEP1	0.036003	2.327369	
19814	Normal colon	TPK1	0.028966	2.337122	
19815	Normal colon	SHROOM3	0.000168	2.337508	
19816	Normal colon	CLMN	0.000297	2.341175	
19817	Normal colon	GLTP	0.000012	2.341238	
19818	Normal colon	PLCD3	0.000143	2.342746	
19819	Normal colon	RUNDC3B	0.000168	2.344697	
19820	Normal colon	USP22	0.000098	2.348289	
19821	Normal colon	MTM1	0.000299	2.356838	
19822	Normal colon	FAM82A	0.014703	2.359803	
19823	Normal colon	LOC100505483	0.000238	2.372839	
19824	Normal colon	LOC100287411	0.00014	2.377259	
19825	Normal colon	LOC100289632	0.000206	2.377517	
19826	Normal colon	FAM47E	0.000256	2.385583	
19827	Normal colon	EIF4E3	0.000052	2.389208	
19828	Normal colon	CNNM4	0.000074	2.390598	
19829	Normal colon	FOXD2	0	2.393982	
19830	Normal colon	SPPL2A	0.000009	2.398902	
19831	Normal colon	PGM1	0.000017	2.39987	
19832	Normal colon	GNA11	0.000116	2.400654	
19833	Normal colon	BAIAP2L2	0.040379	2.406717	
19834	Normal colon	C17orf76	0.000252	2.408479	
19835	Normal colon	SLC39A13	0.012324	2.413232	
19836	Normal colon	TDP2	0.000009	2.417162	
19837	Normal colon	SVIL	0.039004	2.417473	
19838	Normal colon	PAFAH2	0	2.419754	
19839	Normal colon	FA2H	0.000221	2.420133	
19840	Normal colon	SEPP1	0	2.420406	
19841	Normal colon	SAMD13	0.000173	2.421725	
19842	Normal colon	GOLM1	0.000252	2.421836	
19843	Normal colon	PTP4A1	0.000012	2.42263	
19844	Normal colon	ADCY6	0.020642	2.425586	
19845	Normal colon	FLJ32063	0.000136	2.428407	
19846	Normal colon	ACOX1	0.039431	2.429175	
19847	Normal colon	ELOVL6	0.000034	2.429614	
19848	Normal colon	KIAA0415	0.000053	2.431677	

19849	Normal colon	ASAP3	0.000135	2.432569	
19850	Normal colon	PPP1R14D	0	2.453992	
19851	Normal colon	A1CF	0.000028	2.459517	
19852	Normal colon	HADH	0.000053	2.460606	
19853	Normal colon	RHOF	0.000258	2.461934	
19854	Normal colon	LPAR1	0.000066	2.468301	
19855	Normal colon	PER1	0.029825	2.474265	
19856	Normal colon	MYOT	0.00017	2.475259	
19857	Normal colon	RETSAT	0.000202	2.47862	
19858	Normal colon	SLC41A2	0.029803	2.482281	
19859	Normal colon	HNRPH1	0.039431	2.491935	
19860	Normal colon	IGHG1	0	2.494665	
19861	Normal colon	CLDN7	0	2.499912	
19862	Normal colon	SGSM3	0.018234	2.503246	
19863	Normal colon	SULT1A3	0.000217	2.511955	
19864	Normal colon	SLC39A5	0	2.514976	
19865	Normal colon	RAP1GAP	0	2.515	
19866	Normal colon	PRKACB	0	2.522326	
19867	Normal colon	C2orf72	0.000037	2.559506	
19868	Normal colon	MIER3	0.026637	2.565192	
19869	Normal colon	HLA-C	0	2.567838	
19870	Normal colon	PLXNA2	0.035292	2.568454	
19871	Normal colon	PECI	0.000276	2.573106	
19872	Normal colon	UGDH	0.000029	2.5748	
19873	Normal colon	CCL15	0.018452	2.585124	
19874	Normal colon	FOXA1	0.000292	2.593877	
19875	Normal colon	C4orf19	0.000122	2.613077	
19876	Normal colon	GGA2	0.035981	2.621938	
19877	Normal colon	KRT24	0.000012	2.625213	
19878	Normal colon	USP2	0.000085	2.636411	
19879	Normal colon	NR5A2	0.0003	2.636983	
19880	Normal colon	FMO5	0.000299	2.660514	
19881	Normal colon	SRPX	0.00029	2.663967	
19882	Normal colon	SRI	0.018452	2.676535	
19883	Normal colon	RBM47	0.00004	2.686042	
19884	Normal colon	HRBL	0.041333	2.728359	
19885	Normal colon	TMEM56	0.000041	2.746816	
19886	Normal colon	ETFDH	0.000023	2.757237	
19887	Normal colon	TJP3	0	2.763834	
19888	Normal colon	PPP2R3A	0.000074	2.772831	
19889	Normal colon	PPID	0.000083	2.778977	
19890	Normal colon	DSC2	0.000053	2.779295	
19891	Normal colon	SGK1	0.000007	2.786882	
19892	Normal colon	MMP28	0.000034	2.79525	
19893	Normal colon	SLC1A7	0.000015	2.805395	
19894	Normal colon	LOC400573	0.000123	2.834344	
19895	Normal colon	CLDN23	0.0002	2.834475	
19896	Normal colon	BEST2	0.04355	2.835035	
19897	Normal colon	KRT20	0.000255	2.836996	
19898	Normal colon	CMBL	0	2.855768	

19899	Normal colon	PRKAR2B	0.000238	2.861024	
19900	Normal colon	MS4A8B	0	2.868661	
19901	Normal colon	LARP4	0.036623	2.878931	
19902	Normal colon	CFH	0.038575	2.879264	
19903	Normal colon	TMEM171	0.000043	2.885524	
19904	Normal colon	IL10RA	0	2.903981	
19905	Normal colon	ETHE1	0.000003	2.915623	
19906	Normal colon	DPF3	0.024511	2.924453	
19907	Normal colon	MFSD4	0.023772	2.970282	
19908	Normal colon	PTPRH	0	2.97681	
19909	Normal colon	DENND2A	0.000061	2.97707	
19910	Normal colon	FAM46C	0.000015	2.977186	
19911	Normal colon	TTC23	0.000018	2.979335	
19912	Normal colon	SULT1A2	0.000079	2.995262	
19913	Normal colon	EPHX2	0	3.014573	
19914	Normal colon	AGPAT7	0.038656	3.02304	
19915	Normal colon	RIPK3	0.008025	3.029823	
19916	Normal colon	KIF16B	0.000012	3.030102	
19917	Normal colon	ADH6	0.04073	3.031853	
19918	Normal colon	ARSF	0.035494	3.03999	
19919	Normal colon	KLF4	0.000031	3.042323	
19920	Normal colon	ZZEF1	0.000261	3.16765	
19921	Normal colon	TNKS2	0.014715	3.169964	
19922	Normal colon	SYNPO	0.018452	3.178325	
19923	Normal colon	MTR	0.011207	3.179868	
19924	Normal colon	PROM2	0	3.182083	
19925	Normal colon	STMN2	0.00025	3.185564	
19926	Normal colon	FLJ14213	0.02244	3.216296	
19927	Normal colon	FABP1	0.000007	3.261659	
19928	Normal colon	ZBTB7C	0.000141	3.266716	
19929	Normal colon	DGKA	0.017193	3.286078	
19930	Normal colon	C2orf88	0.000052	3.296037	
19931	Normal colon	MGLL	0.026227	3.304656	
19932	Normal colon	ACADS	0	3.306832	
19933	Normal colon	ACADVL	0.016333	3.309393	
19934	Normal colon	AHCYL2	0.000007	3.330288	
19935	Normal colon	NR3C2	0.000074	3.350237	
19936	Normal colon	CKMT1A	0.000012	3.35529	
19937	Normal colon	EYA2	0.000292	3.360873	
19938	Normal colon	ZNF575	0.000035	3.378636	
19939	Normal colon	ITPKA	0	3.397037	
19940	Normal colon	CAMK2D	0.017024	3.426336	
19941	Normal colon	GIYD2	0.029711	3.430614	
19942	Normal colon	RDH5	0.000083	3.450638	
19943	Normal colon	VILL	0.000078	3.451077	
19944	Normal colon	FAM3D	0	3.45531	
19945	Normal colon	ASAHL	0.032961	3.503346	
19946	Normal colon	NAT2	0.038865	3.513072	
19947	Normal colon	OTC	0.039683	3.517702	
19948	Normal colon	SULT1B1	0.000027	3.521881	

19949	Normal colon	HIGD1A	0.000009	3.524229	
19950	Normal colon	CAPN9	0	3.526891	
19951	Normal colon	CES3	0.000071	3.541904	
19952	Normal colon	GGT6	0	3.547953	
19953	Normal colon	ITM2C	0	3.551243	
19954	Normal colon	APBA3	0.018234	3.602421	
19955	Normal colon	PCK1	0.000292	3.635801	
19956	Normal colon	CFD	0	3.644605	
19957	Normal colon	TLN2	0.02718	3.659044	
19958	Normal colon	ARL14	0.000007	3.700351	
19959	Normal colon	ABCA8	0.000244	3.701992	
19960	Normal colon	SLCO2A1	0.033834	3.702843	
19961	Normal colon	ACAA2	0.010089	3.766697	
19962	Normal colon	PLA2G10	0	3.810911	
19963	Normal colon	SCIN	0.000238	3.855473	
19964	Normal colon	PLCL2	0.036003	3.886128	
19965	Normal colon	IGHA1	0	3.894704	
19966	Normal colon	CXorf30	0.029697	3.899349	
19967	Normal colon	DNALI1	0.01888	3.904849	
19968	Normal colon	RICH2	0	3.92115	
19969	Normal colon	MOGAT2	0.000064	3.948991	
19970	Normal colon	PDK4	0.009431	3.957255	
19971	Normal colon	LRRC19	0.000069	4.006808	
19972	Normal colon	ARHGAP44	0.000252	4.0241	
19973	Normal colon	AGPAT9	0.000063	4.032915	
19974	Normal colon	ST6GALNAC1	0	4.044724	
19975	Normal colon	CCL14	0.029803	4.046945	
19976	Normal colon	ABCC3	0.017251	4.098417	
19977	Normal colon	UGT2B15	0.000038	4.136587	
19978	Normal colon	CCL23	0.014703	4.24844	
19979	Normal colon	HMCN2	0.035016	4.259842	
19980	Normal colon	SLIT3	0.028203	4.271669	
19981	Normal colon	DHR11	0	4.335302	
19982	Normal colon	CWH43	0.000237	4.42692	
19983	Normal colon	RCAN2	0.01354	4.456363	
19984	Normal colon	FBLN1	0.041341	4.492027	
19985	Normal colon	DHRS11	0.000021	4.552529	
19986	Normal colon	SAHH3	0	4.632689	
19987	Normal colon	SMPDL3A	0.028512	4.656029	
19988	Normal colon	GCNT2	0.000098	4.728089	
19989	Normal colon	DNASE1L3	0	4.733923	
19990	Normal colon	LYVE1	0.04014	5.072159	
19991	Normal colon	CDKN2B	0	5.110098	
19992	Normal colon	PLCD1	0.024043	5.270598	
19993	Normal colon	C14orf176	0.028966	5.297335	
19994	Normal colon	IL1R2	0.000052	5.493194	
19995	Normal colon	TMEM37	0.02234	5.658031	
19996	Normal colon	SCNN1A	0.018452	5.663262	
19997	Normal colon	ST6GALNAC6	0.039683	5.667975	
19998	Normal colon	GREM2	0.026621	5.752544	

19999	Normal colon	MUC5B	0.016333	5.913723	
20000	Normal colon	SEMA6A	0	6.067993	
20001	Normal colon	PKIB	0.000009	6.113732	
20002	Normal colon	DHRS9	0.000007	6.14952	
20003	Normal colon	WDR78	0.033986	6.221949	
20004	Normal colon	TEX11	0.026101	6.407819	
20005	Normal colon	ITLN1	0	6.492572	
20006	Normal colon	ABCG2	0.000246	6.568358	
20007	Normal colon	VIPR1	0.020108	6.60046	
20008	Normal colon	SELENBP1	0	6.712648	
20009	Normal colon	LOC388335	0.010896	6.811472	
20010	Normal colon	MARCKSL1	0.024437	7.3093	
20011	Normal colon	AKR1B10	0.00018	7.408942	
20012	Normal colon	PYY	0	7.447677	
20013	Normal colon	CLCA1	0	7.773476	
20014	Normal colon	JAK1	0.023672	7.942721	
20015	Normal colon	SCNN1B	0.006411	8.610319	
20016	Normal colon	MGC13057	0.017251	9.326053	
20017	Normal colon	C6orf105	0	10.406819	
20018	Normal colon	ADAMDEC1	0	11.367719	
20019	Normal colon	MAPK8	0.029803	12.237187	
20020	Normal colon	CLCA4	0	13.635842	
20021	Normal colon	MT1H	0.040019	14.19686	
20022	Normal colon	MT1M	0.036178	15.982265	
20023	Normal colon	AQP8	0	22.472268	
20024	Normal colon	SLC17A4	0.018452	25.321074	
20025	Normal colon	MUC2	0.036586	26.629129	
20026	Normal colon	CHGA	0.038865	27.658161	
20027	Normal colon	GUCA2B	0.005607	43.926917	
20028	Normal rectum	DLGAP4	0.009754	2.082438	
20029	Normal tissue morphology	AQP8	0	-44.069335	
20030	Normal tissue morphology	CA1	0	-36.110772	
20031	Normal tissue morphology	CLCA4	0	-31.629654	
20032	Normal tissue morphology	GUCA2A	0	-26.964687	
20033	Normal tissue morphology	GUCA2B	0	-24.924778	
20034	Normal tissue morphology	CA4	0	-18.548553	
20035	Normal tissue morphology	MS4A12	0	-17.955812	
20036	Normal tissue morphology	ADH1A	0	-11.751594	
20037	Normal tissue morphology	TMIGD	0	-10.791315	
20038	Normal tissue morphology	LOC63928	0	-8.915585	
20039	Normal tissue morphology	MT1M	0	-8.12885	
20040	Normal tissue morphology	CFD	0	-7.915842	
20041	Normal tissue morphology	MAMDC2	0	-7.045446	
20042	Normal tissue morphology	SFRP1	0	-6.684432	
20043	Normal tissue morphology	OSTbeta	0	-6.304743	
20044	Normal tissue morphology	SCARA5	0	-6.298371	
20045	Normal tissue morphology	HSD11B2	0	-6.08248	
20046	Normal tissue morphology	CXCL12	0	-5.943106	
20047	Normal tissue morphology	HMMR	0.000025	-5.804377	
20048	Normal tissue morphology	VMD2L1	0	-5.755075	

20049	Normal tissue morphology	PI16	0	-5.715154	
20050	Normal tissue morphology	KIF20A	0.000391	-5.468525	
20051	Normal tissue morphology	VMD2L2	0	-5.32281	
20052	Normal tissue morphology	PCOLCE2	0	-5.114989	
20053	Normal tissue morphology	UGT1A10	0	-4.847321	
20054	Normal tissue morphology	SLC16A9	0	-4.611893	
20055	Normal tissue morphology	VIP	0	-4.583618	
20056	Normal tissue morphology	LOC340843	0	-4.503637	
20057	Normal tissue morphology	PLAC9	0	-4.463959	
20058	Normal tissue morphology	PDK4	0	-4.232559	
20059	Normal tissue morphology	PRPH	0	-4.228059	
20060	Normal tissue morphology	KIAA0828	0	-4.161943	
20061	Normal tissue morphology	LOC441282	0	-4.058839	
20062	Normal tissue morphology	XPOT	0.014459	-3.992622	
20063	Normal tissue morphology	MATN2	0	-3.91557	
20064	Normal tissue morphology	E2F5	0.005272	-3.890783	
20065	Normal tissue morphology	CCDC86	0.002201	-3.867938	
20066	Normal tissue morphology	RELL2	0.003759	-3.855649	
20067	Normal tissue morphology	B3GALTL	0.049356	-3.840546	
20068	Normal tissue morphology	GPX3	0	-3.748229	
20069	Normal tissue morphology	WDSOF1	0.001352	-3.709982	
20070	Normal tissue morphology	SNTB1	0.000297	-3.60564	
20071	Normal tissue morphology	CDC6	0.011855	-3.551337	
20072	Normal tissue morphology	PPM1H	0.000249	-3.479492	
20073	Normal tissue morphology	OTOP2	0	-3.429898	
20074	Normal tissue morphology	PTP4A3	0.000168	-3.409801	
20075	Normal tissue morphology	MAL	0	-3.354032	
20076	Normal tissue morphology	FANCI	0.0087	-3.309157	
20077	Normal tissue morphology	BUB1	0.003759	-3.279526	
20078	Normal tissue morphology	C13orf3	0.010117	-3.221261	
20079	Normal tissue morphology	GPM6B	0	-3.220923	
20080	Normal tissue morphology	SLC1A5	0	-3.204578	
20081	Normal tissue morphology	CNTFR	0	-3.157038	
20082	Normal tissue morphology	XKR4	0	-3.097864	
20083	Normal tissue morphology	KCNIP4	0	-3.090417	
20084	Normal tissue morphology	HMFN0839	0	-3.055462	
20085	Normal tissue morphology	C8ORF30A	0	-3.037225	
20086	Normal tissue morphology	OGN	0	-3.035103	
20087	Normal tissue morphology	GREM2	0	-3.005481	
20088	Normal tissue morphology	ABI3BP	0	-3.004368	
20089	Normal tissue morphology	SLC25A34	0	-2.894841	
20090	Normal tissue morphology	MCM8	0.004563	-2.888232	
20091	Normal tissue morphology	COL1A1	0.000263	-2.887807	
20092	Normal tissue morphology	EDG2	0	-2.8128	
20093	Normal tissue morphology	LOC285016	0	-2.803106	
20094	Normal tissue morphology	HTR4	0	-2.796495	
20095	Normal tissue morphology	LOC731049	0	-2.775363	
20096	Normal tissue morphology	EPB41L3	0	-2.762123	
20097	Normal tissue morphology	SLC5A6	0	-2.75067	
20098	Normal tissue morphology	PLCD1	0	-2.749102	

20099	Normal tissue morphology	TNFRSF10B	0	-2.747858	
20100	Normal tissue morphology	HIG2	0	-2.736757	
20101	Normal tissue morphology	CLIC3	0.000063	-2.704234	
20102	Normal tissue morphology	CCND1	0	-2.692153	
20103	Normal tissue morphology	PHLPPL	0	-2.688005	
20104	Normal tissue morphology	ASAHL	0	-2.687594	
20105	Normal tissue morphology	ZNHIT4	0	-2.684367	
20106	Normal tissue morphology	FASN	0	-2.627448	
20107	Normal tissue morphology	IMPDH1	0	-2.626598	
20108	Normal tissue morphology	UBE2V1	0.004279	-2.625998	
20109	Normal tissue morphology	MGC4308	0.000012	-2.624252	
20110	Normal tissue morphology	TMEM97	0	-2.606366	
20111	Normal tissue morphology	SLC7A1	0	-2.593797	
20112	Normal tissue morphology	FUCA1	0	-2.564942	
20113	Normal tissue morphology	LOC643940	0	-2.564427	
20114	Normal tissue morphology	PDE9A	0	-2.559518	
20115	Normal tissue morphology	PAQR5	0	-2.542337	
20116	Normal tissue morphology	SLC7A6	0.005899	-2.538138	
20117	Normal tissue morphology	TMEM100	0	-2.527418	
20118	Normal tissue morphology	LOC388796	0.000056	-2.525723	
20119	Normal tissue morphology	AMIGO2	0.000027	-2.510275	
20120	Normal tissue morphology	RUNX1	0	-2.507444	
20121	Normal tissue morphology	RPL35A	0.023557	-2.488761	
20122	Normal tissue morphology	DACH1	0	-2.479156	
20123	Normal tissue morphology	KPNA2	0	-2.47461	
20124	Normal tissue morphology	CBX2	0.000072	-2.45772	
20125	Normal tissue morphology	FOXN2	0	-2.456034	
20126	Normal tissue morphology	TOP1MT	0.000095	-2.441191	
20127	Normal tissue morphology	CSPP1	0.004279	-2.417897	
20128	Normal tissue morphology	TNFRSF11B	0.000263	-2.412846	
20129	Normal tissue morphology	IRAK2	0	-2.400199	
20130	Normal tissue morphology	TCF21	0	-2.391464	
20131	Normal tissue morphology	JAG2	0.000098	-2.357991	
20132	Normal tissue morphology	DKC1	0	-2.357733	
20133	Normal tissue morphology	FKBP4	0	-2.352568	
20134	Normal tissue morphology	CBFB	0	-2.344402	
20135	Normal tissue morphology	C13orf27	0.000057	-2.341832	
20136	Normal tissue morphology	PMP2	0	-2.341572	
20137	Normal tissue morphology	DARS2	0.003721	-2.328628	
20138	Normal tissue morphology	POLR1C	0	-2.325351	
20139	Normal tissue morphology	DTD1	0	-2.323472	
20140	Normal tissue morphology	MYC	0.000043	-2.32116	
20141	Normal tissue morphology	BOP1	0	-2.317033	
20142	Normal tissue morphology	LGI1	0	-2.314818	
20143	Normal tissue morphology	NKX2-3	0	-2.302686	
20144	Normal tissue morphology	AKAP7	0	-2.2988	
20145	Normal tissue morphology	ALDH4A1	0	-2.292465	
20146	Normal tissue morphology	KIF5C	0	-2.283957	
20147	Normal tissue morphology	NOL6	0	-2.279329	
20148	Normal tissue morphology	C7ORF47	0	-2.277622	

20149	Normal tissue morphology	COMT	0.0087	-2.27312	
20150	Normal tissue morphology	TMEFF2	0	-2.267269	
20151	Normal tissue morphology	RPIB9	0	-2.251726	
20152	Normal tissue morphology	TMEM206	0.000151	-2.251382	
20153	Normal tissue morphology	RBM15	0	-2.250458	
20154	Normal tissue morphology	CYB5R2	0	-2.247061	
20155	Normal tissue morphology	EXOSC8	0.023557	-2.246594	
20156	Normal tissue morphology	RAI14	0.000238	-2.235023	
20157	Normal tissue morphology	MCM2	0	-2.216769	
20158	Normal tissue morphology	U2AF2	0	-2.209129	
20159	Normal tissue morphology	TNFRSF13B	0	-2.195702	
20160	Normal tissue morphology	CCDC113	0	-2.192512	
20161	Normal tissue morphology	SFRS3	0.023214	-2.178891	
20162	Normal tissue morphology	ZMIZ2	0	-2.174462	
20163	Normal tissue morphology	TDP1	0	-2.17419	
20164	Normal tissue morphology	AFF3	0	-2.168501	
20165	Normal tissue morphology	LOC90835	0.001125	-2.166442	
20166	Normal tissue morphology	STOX2	0	-2.16469	
20167	Normal tissue morphology	TBC1D16	0	-2.152824	
20168	Normal tissue morphology	NAT10	0	-2.14404	
20169	Normal tissue morphology	C20ORF198	0	-2.140572	
20170	Normal tissue morphology	SLC12A2	0.000235	-2.139849	
20171	Normal tissue morphology	TPD52L2	0.000076	-2.120266	
20172	Normal tissue morphology	HPCAL1	0	-2.119658	
20173	Normal tissue morphology	TP53INP2	0	-2.11798	
20174	Normal tissue morphology	PPARGC1B	0	-2.109637	
20175	Normal tissue morphology	TRPC4AP	0	-2.107255	
20176	Normal tissue morphology	NOC2L	0.026594	-2.104099	
20177	Normal tissue morphology	TUT1	0	-2.102384	
20178	Normal tissue morphology	FLJ10826	0.000009	-2.102346	
20179	Normal tissue morphology	SF3B3	0	-2.100833	
20180	Normal tissue morphology	RAVER2	0	-2.08924	
20181	Normal tissue morphology	TUBA4A	0	-2.088663	
20182	Normal tissue morphology	WISP2	0	-2.08814	
20183	Normal tissue morphology	MET	0	-2.088048	
20184	Normal tissue morphology	RPUSD4	0	-2.084899	
20185	Normal tissue morphology	CITED2	0	-2.074953	
20186	Normal tissue morphology	CCNB1IP1	0	-2.074775	
20187	Normal tissue morphology	SNX5	0	-2.066829	
20188	Normal tissue morphology	CCNF	0	-2.066788	
20189	Normal tissue morphology	HEATR2	0	-2.058055	
20190	Normal tissue morphology	NFXL1	0	-2.053474	
20191	Normal tissue morphology	GTPBP4	0	-2.049344	
20192	Normal tissue morphology	ABCB6	0	-2.043855	
20193	Normal tissue morphology	PLP1	0	-2.034127	
20194	Normal tissue morphology	MIF	0	-2.034082	
20195	Normal tissue morphology	CCBP2	0	-2.027799	
20196	Normal tissue morphology	SLC25A22	0	-2.020754	
20197	Normal tissue morphology	TCERG1	0.045229	-1.530054	
20198	Normal tissue morphology	CTNND1	0.000001	2.001972	

20199	Normal tissue morphology	LOC100289019	0.000053	2.00295	
20200	Normal tissue morphology	BDH1	0.00027	2.003198	
20201	Normal tissue morphology	BAD	0	2.004266	
20202	Normal tissue morphology	PCSK1N	0.000002	2.008034	
20203	Normal tissue morphology	SGK2	0.000043	2.008301	
20204	Normal tissue morphology	LRRFIP2	0.000067	2.011938	
20205	Normal tissue morphology	MTUS1	0.000071	2.012135	
20206	Normal tissue morphology	C10ORF26	0	2.013443	
20207	Normal tissue morphology	POLD4	0.000002	2.014145	
20208	Normal tissue morphology	FJX1	0	2.028496	
20209	Normal tissue morphology	CAPN5	0.000138	2.031624	
20210	Normal tissue morphology	CDC2L1	0	2.032829	
20211	Normal tissue morphology	CLTB	0.000008	2.034168	
20212	Normal tissue morphology	SYTL4	0.000002	2.034218	
20213	Normal tissue morphology	S100A10	0.000004	2.036907	
20214	Normal tissue morphology	HIBCH	0.000127	2.041443	
20215	Normal tissue morphology	S100A14	0.000183	2.042127	
20216	Normal tissue morphology	CMAS	0.000052	2.042658	
20217	Normal tissue morphology	C14ORF147	0	2.045618	
20218	Normal tissue morphology	FXYD1	0.000047	2.055213	
20219	Normal tissue morphology	SHPK	0.000083	2.058742	
20220	Normal tissue morphology	TFCP2L1	0.000006	2.064292	
20221	Normal tissue morphology	ACAT1	0.000077	2.066848	
20222	Normal tissue morphology	ATG4A	0.000012	2.067547	
20223	Normal tissue morphology	TP53I3	0.000116	2.067676	
20224	Normal tissue morphology	PLXNA2	0.000002	2.068311	
20225	Normal tissue morphology	CALM1	0.00025	2.069654	
20226	Normal tissue morphology	NAALADL1	0	2.069962	
20227	Normal tissue morphology	MGLL	0.000004	2.073045	
20228	Normal tissue morphology	VAMP8	0.000007	2.083103	
20229	Normal tissue morphology	MEF2D	0	2.087322	
20230	Normal tissue morphology	CASD1	0.00029	2.091419	
20231	Normal tissue morphology	FLJ12610	0.000009	2.095074	
20232	Normal tissue morphology	PDSS2	0.000052	2.099445	
20233	Normal tissue morphology	PCDHA6	0.000109	2.10056	
20234	Normal tissue morphology	GLCCI1	0.000282	2.101006	
20235	Normal tissue morphology	ShrmL	0.00001	2.101674	
20236	Normal tissue morphology	ATP5S	0.000026	2.103186	
20237	Normal tissue morphology	CHP	0.000003	2.107398	
20238	Normal tissue morphology	MRPL35	0.000297	2.11346	
20239	Normal tissue morphology	GLOD5	0.000271	2.113893	
20240	Normal tissue morphology	C1ORF108	0	2.115181	
20241	Normal tissue morphology	PLD1	0.000135	2.115702	
20242	Normal tissue morphology	PPP1CB	0	2.116879	
20243	Normal tissue morphology	C14orf159	0.000015	2.122629	
20244	Normal tissue morphology	PAQR8	0.000098	2.124901	
20245	Normal tissue morphology	UQCRC1	0.000063	2.132919	
20246	Normal tissue morphology	STXBP3	0	2.133796	
20247	Normal tissue morphology	RIPK2	0	2.137108	
20248	Normal tissue morphology	C9orf125	0.000112	2.151976	

20249	Normal tissue morphology	MYO1D	0.000211	2.153935	
20250	Normal tissue morphology	MXD1	0.000104	2.154847	
20251	Normal tissue morphology	PTK6	0.000009	2.155801	
20252	Normal tissue morphology	CDS1	0.000028	2.155889	
20253	Normal tissue morphology	ORF1-FL49	0.000001	2.166765	
20254	Normal tissue morphology	GBA2	0.000285	2.169336	
20255	Normal tissue morphology	FKBP5	0.000164	2.181115	
20256	Normal tissue morphology	OSBPL7	0.000066	2.183997	
20257	Normal tissue morphology	GALIG	0	2.192145	
20258	Normal tissue morphology	SLC35A3	0.000006	2.193479	
20259	Normal tissue morphology	LIMA1	0.000009	2.19754	
20260	Normal tissue morphology	GALM	0.000162	2.201462	
20261	Normal tissue morphology	FZD5	0.000266	2.202912	
20262	Normal tissue morphology	FLJ36848	0.000256	2.20681	
20263	Normal tissue morphology	ANO10	0.000053	2.207056	
20264	Normal tissue morphology	WISP1	0	2.212239	
20265	Normal tissue morphology	C6orf136	0.000023	2.216746	
20266	Normal tissue morphology	FAM149A	0.000141	2.224178	
20267	Normal tissue morphology	CAS1	0	2.229294	
20268	Normal tissue morphology	LGMN	0	2.231319	
20269	Normal tissue morphology	PEX26	0.000241	2.233614	
20270	Normal tissue morphology	FLNB	0.000297	2.244974	
20271	Normal tissue morphology	SHD	0.000098	2.248522	
20272	Normal tissue morphology	RHBDF2	0	2.25911	
20273	Normal tissue morphology	KRT19P2	0.000135	2.260004	
20274	Normal tissue morphology	FLJ14981	0.000001	2.278628	
20275	Normal tissue morphology	SLC35D1	0.000296	2.279274	
20276	Normal tissue morphology	GLIPR2	0.000116	2.281672	
20277	Normal tissue morphology	KBTBD11	0.000046	2.294318	
20278	Normal tissue morphology	IDH3A	0.000023	2.296197	
20279	Normal tissue morphology	SH3BGRL2	0.000004	2.297834	
20280	Normal tissue morphology	SLC44A1	0.000107	2.300155	
20281	Normal tissue morphology	LETM1	0.000028	2.307655	
20282	Normal tissue morphology	ALDH6A1	0.000229	2.310912	
20283	Normal tissue morphology	SYTL2	0.000012	2.312394	
20284	Normal tissue morphology	APPL2	0.000084	2.313575	
20285	Normal tissue morphology	TRPM4	0.000006	2.333557	
20286	Normal tissue morphology	SHROOM3	0.000168	2.337508	
20287	Normal tissue morphology	RUNDC3B	0.000168	2.344697	
20288	Normal tissue morphology	USP22	0.000098	2.348289	
20289	Normal tissue morphology	PCSK6	0.000006	2.366172	
20290	Normal tissue morphology	MTHFD1L	0	2.368066	
20291	Normal tissue morphology	CYCS	0	2.369188	
20292	Normal tissue morphology	LOC100505483	0.000238	2.372839	
20293	Normal tissue morphology	NDUFS4	0	2.374655	
20294	Normal tissue morphology	LOC100287411	0.00014	2.377259	
20295	Normal tissue morphology	LOC100289632	0.000206	2.377517	
20296	Normal tissue morphology	FAM47E	0.000256	2.385583	
20297	Normal tissue morphology	EIF4E3	0.000052	2.389208	
20298	Normal tissue morphology	CNNM4	0.000074	2.390598	

20299	Normal tissue morphology	MGST3	0	2.39558	
20300	Normal tissue morphology	SPPL2A	0.000009	2.398902	
20301	Normal tissue morphology	PGM1	0.000017	2.39987	
20302	Normal tissue morphology	MDH1	0	2.400904	
20303	Normal tissue morphology	FBXO3	0	2.403039	
20304	Normal tissue morphology	C17orf76	0.000252	2.408479	
20305	Normal tissue morphology	TDP2	0.000009	2.417162	
20306	Normal tissue morphology	FA2H	0.000221	2.420133	
20307	Normal tissue morphology	SAMD13	0.000173	2.421725	
20308	Normal tissue morphology	GOLM1	0.000252	2.421836	
20309	Normal tissue morphology	PTP4A1	0.000012	2.42263	
20310	Normal tissue morphology	ELOVL6	0.000034	2.429614	
20311	Normal tissue morphology	KIAA0415	0.000053	2.431677	
20312	Normal tissue morphology	ASAP3	0.000135	2.432569	
20313	Normal tissue morphology	CRK	0	2.453192	
20314	Normal tissue morphology	A1CF	0.000028	2.459517	
20315	Normal tissue morphology	HADH	0.000053	2.460606	
20316	Normal tissue morphology	RHOF	0.000258	2.461934	
20317	Normal tissue morphology	LPAR1	0.000066	2.468301	
20318	Normal tissue morphology	CRYZL1	0	2.474973	
20319	Normal tissue morphology	MYO1C	0	2.479873	
20320	Normal tissue morphology	FLJ20273	0.000008	2.483385	
20321	Normal tissue morphology	SULT1A3	0.000217	2.511955	
20322	Normal tissue morphology	LOC201895	0.000001	2.520197	
20323	Normal tissue morphology	TDE2L	0.000006	2.522827	
20324	Normal tissue morphology	NANS	0.000001	2.539289	
20325	Normal tissue morphology	TCEA3	0.000297	2.544402	
20326	Normal tissue morphology	FXYD5	0	2.548632	
20327	Normal tissue morphology	C2orf72	0.000037	2.559506	
20328	Normal tissue morphology	CKMT1	0.000013	2.56467	
20329	Normal tissue morphology	PECI	0.000276	2.573106	
20330	Normal tissue morphology	BMP2	0.000162	2.579571	
20331	Normal tissue morphology	FMO4	0.000009	2.582345	
20332	Normal tissue morphology	FOXA1	0.000292	2.593877	
20333	Normal tissue morphology	PIP5K1B	0.000006	2.603886	
20334	Normal tissue morphology	C4orf19	0.000122	2.613077	
20335	Normal tissue morphology	PXMP2	0	2.613262	
20336	Normal tissue morphology	SMOX	0	2.620293	
20337	Normal tissue morphology	CPM	0.000051	2.621781	
20338	Normal tissue morphology	MALL	0.00003	2.631392	
20339	Normal tissue morphology	USP2	0.000085	2.636411	
20340	Normal tissue morphology	FMO5	0.000299	2.660514	
20341	Normal tissue morphology	CGN	0.000009	2.669418	
20342	Normal tissue morphology	SATB2	0.000007	2.679973	
20343	Normal tissue morphology	LMBRD1	0	2.685609	
20344	Normal tissue morphology	RBM47	0.00004	2.686042	
20345	Normal tissue morphology	GOLPH2	0.000005	2.702898	
20346	Normal tissue morphology	C4orf34	0.000284	2.70772	
20347	Normal tissue morphology	MGC4171	0.00001	2.724735	
20348	Normal tissue morphology	CCL15	0.000004	2.725844	

20349	Normal tissue morphology	TMEM56	0.000041	2.746816	
20350	Normal tissue morphology	TUB	0.000002	2.748186	
20351	Normal tissue morphology	STX8	0	2.749908	
20352	Normal tissue morphology	LRRC1	0.000013	2.764394	
20353	Normal tissue morphology	PPP2R3A	0.000074	2.772831	
20354	Normal tissue morphology	PPID	0.000083	2.778977	
20355	Normal tissue morphology	FLJ11017	0.000002	2.782371	
20356	Normal tissue morphology	RGS19IP1	0.00001	2.784519	
20357	Normal tissue morphology	SGK1	0.000007	2.786882	
20358	Normal tissue morphology	PPARGC1A	0.000012	2.794212	
20359	Normal tissue morphology	MMP28	0.000034	2.79525	
20360	Normal tissue morphology	CHST5	0.000004	2.795886	
20361	Normal tissue morphology	SEMA4G	0.000002	2.809155	
20362	Normal tissue morphology	C10ORF32	0	2.820709	
20363	Normal tissue morphology	TMEM45B	0.000002	2.820958	
20364	Normal tissue morphology	CLDN7	0.000004	2.827828	
20365	Normal tissue morphology	LOC400573	0.000123	2.834344	
20366	Normal tissue morphology	CYP4F12	0.000003	2.837124	
20367	Normal tissue morphology	PRKAR2B	0.000238	2.861024	
20368	Normal tissue morphology	TMEM171	0.000043	2.885524	
20369	Normal tissue morphology	KRT14	0.000004	2.898214	
20370	Normal tissue morphology	LRP8	0	2.905623	
20371	Normal tissue morphology	SULT1A1	0.000206	2.925544	
20372	Normal tissue morphology	SMPD3	0.000001	2.930047	
20373	Normal tissue morphology	TM4SF2	0.000002	2.95869	
20374	Normal tissue morphology	FAM3D	0.000002	2.95883	
20375	Normal tissue morphology	DENND2A	0.000061	2.97707	
20376	Normal tissue morphology	FAM46C	0.000015	2.977186	
20377	Normal tissue morphology	TTC23	0.000018	2.979335	
20378	Normal tissue morphology	ATP5F1	0	2.984265	
20379	Normal tissue morphology	SULT1A2	0.000079	2.995262	
20380	Normal tissue morphology	BCLP	0.000001	3.020425	
20381	Normal tissue morphology	PDCD4	0	3.025533	
20382	Normal tissue morphology	HIG1	0.000002	3.027138	
20383	Normal tissue morphology	CCDC68	0.000028	3.028445	
20384	Normal tissue morphology	KIF16B	0.000012	3.030102	
20385	Normal tissue morphology	GPR177	0	3.038642	
20386	Normal tissue morphology	METTL7A	0.000069	3.051391	
20387	Normal tissue morphology	C6orf29	0.000003	3.059207	
20388	Normal tissue morphology	CDC25B	0	3.070827	
20389	Normal tissue morphology	PTTG1IP	0	3.078129	
20390	Normal tissue morphology	CEACAM1	0.000043	3.080438	
20391	Normal tissue morphology	FLJ20152	0.000005	3.093667	
20392	Normal tissue morphology	TP53I5	0.000002	3.120461	
20393	Normal tissue morphology	SCNN1A	0.000005	3.135971	
20394	Normal tissue morphology	SIDT1	0	3.139651	
20395	Normal tissue morphology	LYRM2	0	3.163281	
20396	Normal tissue morphology	CA12	0.00004	3.220574	
20397	Normal tissue morphology	ZBTB7C	0.000141	3.266716	
20398	Normal tissue morphology	PDPN	0	3.271364	

20399	Normal tissue morphology	DKFZP564O08	0.000001	3.288601	
20400	Normal tissue morphology	C2orf88	0.000052	3.296037	
20401	Normal tissue morphology	GCNT3	0.000089	3.318624	
20402	Normal tissue morphology	AHCYL2	0.000007	3.330288	
20403	Normal tissue morphology	CKMT1A	0.000012	3.35529	
20404	Normal tissue morphology	EYA2	0.000292	3.360873	
20405	Normal tissue morphology	STYK1	0	3.378539	
20406	Normal tissue morphology	ZNF575	0.000035	3.378636	
20407	Normal tissue morphology	SALL4	0	3.38018	
20408	Normal tissue morphology	KIAA1737	0	3.412045	
20409	Normal tissue morphology	KRT19	0.000002	3.421713	
20410	Normal tissue morphology	ATP5J	0	3.430434	
20411	Normal tissue morphology	EPHA10	0.000008	3.435053	
20412	Normal tissue morphology	ITM2C	0.000001	3.435864	
20413	Normal tissue morphology	SLC9A2	0.000098	3.438543	
20414	Normal tissue morphology	BSG	0	3.443386	
20415	Normal tissue morphology	ALDH1A1	0	3.48098	
20416	Normal tissue morphology	SMPDL3A	0.000003	3.486516	
20417	Normal tissue morphology	RHBDL2	0	3.502465	
20418	Normal tissue morphology	TIMP1	0	3.518513	
20419	Normal tissue morphology	SULT1B1	0.000027	3.521881	
20420	Normal tissue morphology	FEV	0.000012	3.526807	
20421	Normal tissue morphology	CES3	0.000071	3.541904	
20422	Normal tissue morphology	EIF5	0	3.542339	
20423	Normal tissue morphology	MAOA	0.00002	3.573062	
20424	Normal tissue morphology	SOSTDC1	0.000002	3.574382	
20425	Normal tissue morphology	UGT1A8	0.000013	3.581855	
20426	Normal tissue morphology	FLJ13391	0	3.591249	
20427	Normal tissue morphology	ID2	0	3.593484	
20428	Normal tissue morphology	C10orf99	0.000028	3.620081	
20429	Normal tissue morphology	PCK1	0.000292	3.635801	
20430	Normal tissue morphology	TUBAL3	0.00023	3.650291	
20431	Normal tissue morphology	ARL14	0.000007	3.700351	
20432	Normal tissue morphology	ABCA8	0.000244	3.701992	
20433	Normal tissue morphology	NMES1	0	3.734037	
20434	Normal tissue morphology	TGFBI	0	3.780405	
20435	Normal tissue morphology	PADI2	0.00016	3.803735	
20436	Normal tissue morphology	FAM55D	0.000219	3.823774	
20437	Normal tissue morphology	KLF6	0	3.841979	
20438	Normal tissue morphology	PTPRH	0.000006	3.847104	
20439	Normal tissue morphology	SELENBP1	0.000008	3.851216	
20440	Normal tissue morphology	PLA2G10	0.000013	3.896015	
20441	Normal tissue morphology	NEU4	0.000001	3.914172	
20442	Normal tissue morphology	UGT2B17	0.000072	3.933187	
20443	Normal tissue morphology	MOGAT2	0.000064	3.948991	
20444	Normal tissue morphology	SNRPN	0	4.007894	
20445	Normal tissue morphology	ARHGAP44	0.000252	4.0241	
20446	Normal tissue morphology	AGPAT9	0.000063	4.032915	
20447	Normal tissue morphology	CKB	0.000008	4.034661	
20448	Normal tissue morphology	ESM1	0	4.042148	

20449	Normal tissue morphology	NFE2L3	0	4.056847	
20450	Normal tissue morphology	C11orf33	0.000012	4.060275	
20451	Normal tissue morphology	TRIB3	0	4.096233	
20452	Normal tissue morphology	UGT2B15	0.000038	4.136587	
20453	Normal tissue morphology	SLC7A5	0	4.182042	
20454	Normal tissue morphology	SPINK5	0.000007	4.320209	
20455	Normal tissue morphology	LGALS2	0.000009	4.324702	
20456	Normal tissue morphology	GSN	0	4.329842	
20457	Normal tissue morphology	CWH43	0.000237	4.42692	
20458	Normal tissue morphology	LGALS4	0.000001	4.552136	
20459	Normal tissue morphology	DHRS11	0.000021	4.552529	
20460	Normal tissue morphology	ABCC13	0.00001	4.56359	
20461	Normal tissue morphology	BGN	0	4.605698	
20462	Normal tissue morphology	COL1A2	0	4.622895	
20463	Normal tissue morphology	MAPK10	0.010117	4.758095	
20464	Normal tissue morphology	LOC144501	0	4.899047	
20465	Normal tissue morphology	VSIG2	0	4.980351	
20466	Normal tissue morphology	PYY2	0.000003	5.050842	
20467	Normal tissue morphology	MUCDHL	0.000006	5.11399	
20468	Normal tissue morphology	PHLDA1	0	5.471027	
20469	Normal tissue morphology	CHP2	0.000168	5.791364	
20470	Normal tissue morphology	ABCG2	0.000246	6.568358	
20471	Normal tissue morphology	CEACAM7	0.000003	6.587967	
20472	Normal tissue morphology	MT1X	0	6.852298	
20473	Normal tissue morphology	COL11A1	0	7.083734	
20474	Normal tissue morphology	ACTB	0	7.182208	
20475	Normal tissue morphology	INSL5	0.000002	7.817222	
20476	Normal tissue morphology	SLC26A3	0.000007	10.831482	
20477	Normal tissue morphology	IL8	0	12.714234	
20478	Normal tissue morphology	KIAA1199	0	13.538706	
20479	Normal tissue morphology	FOXQ1	0	15.928664	
20480	Normal tissue morphology	MMP7	0	18.824692	
20481	Osteoporosis	IRX5	0.015188	-1.707782	
20482	Osteoporosis	CD86	0.023613	-1.650987	
20483	Osteoporosis	CGB1	0.031122	-1.650652	
20484	Osteoporosis	CCDC66	0.010377	-1.631498	
20485	Osteoporosis	BCR	0.001675	-1.628805	
20486	Osteoporosis	LRRC2	0.022407	-1.617841	
20487	Osteoporosis	VPS18	0.022524	-1.617524	
20488	Osteoporosis	SAPS1	0.017665	-1.612422	
20489	Osteoporosis	EFTUD1	0.031923	-1.608905	
20490	Osteoporosis	RBM22	0.030823	-1.602585	
20491	Osteoporosis	CABP7	0.022796	-1.596781	
20492	Osteoporosis	RAP1GAP	0.005335	-1.590147	
20493	Osteoporosis	CRISPLD2	0.013591	-1.583184	
20494	Osteoporosis	WDR33	0.017679	-1.575636	
20495	Osteoporosis	C1orf173	0.000307	-1.545916	
20496	Osteoporosis	SLC45A4	0.009605	-1.536165	
20497	Osteoporosis	TEF	0.004786	-1.525566	
20498	Osteoporosis	LOC286272	0.023598	-1.52479	

20499	Osteoporosis	RPA4	0.017736	-1.524142	
20500	Osteoporosis	PIK3R5	0.028155	-1.510486	
20501	Osteoporosis	TTC23	0.000405	-1.510329	
20502	Osteoporosis	FLJ10404	0.026901	-1.504107	
20503	Osteoporosis family history	IRX5	0.015188	-1.707782	
20504	Osteoporosis family history	CD86	0.023613	-1.650987	
20505	Osteoporosis family history	CGB1	0.031122	-1.650652	
20506	Osteoporosis family history	CCDC66	0.010377	-1.631498	
20507	Osteoporosis family history	BCR	0.001675	-1.628805	
20508	Osteoporosis family history	LRRC2	0.022407	-1.617841	
20509	Osteoporosis family history	VPS18	0.022524	-1.617524	
20510	Osteoporosis family history	SAPS1	0.017665	-1.612422	
20511	Osteoporosis family history	EFTUD1	0.031923	-1.608905	
20512	Osteoporosis family history	RBM22	0.030823	-1.602585	
20513	Osteoporosis family history	CABP7	0.022796	-1.596781	
20514	Osteoporosis family history	RAP1GAP	0.005335	-1.590147	
20515	Osteoporosis family history	CRISPLD2	0.013591	-1.583184	
20516	Osteoporosis family history	WDR33	0.017679	-1.575636	
20517	Osteoporosis family history	C1orf173	0.000307	-1.545916	
20518	Osteoporosis family history	SLC45A4	0.009605	-1.536165	
20519	Osteoporosis family history	TEF	0.004786	-1.525566	
20520	Osteoporosis family history	LOC286272	0.023598	-1.52479	
20521	Osteoporosis family history	RPA4	0.017736	-1.524142	
20522	Osteoporosis family history	PIK3R5	0.028155	-1.510486	
20523	Osteoporosis family history	TTC23	0.000405	-1.510329	
20524	Osteoporosis family history	FLJ10404	0.026901	-1.504107	
20525	Ovarian Carcinoma	CGI-38	0.003991	-2.78059	
20526	Ovarian Carcinoma	SETBP1	0.006355	-2.081398	
20527	Ovarian Carcinoma	SDK1	0.003991	2.026087	
20528	Ovarian Carcinoma	MS4A4A	0.010815	2.180689	
20529	Ovarian Carcinoma	ARL4A	0.027133	2.30044	
20530	Ovarian Carcinoma	ST3GAL2	0.000845	2.361099	
20531	Ovarian Carcinoma	TAP2	0.020351	2.516374	
20532	Ovarian Carcinoma	LOC440607	0.044929	2.670231	
20533	Ovarian Carcinoma	SAMD9	0.035369	2.792032	
20534	Ovarian Carcinoma	IFI44	0.020351	3.348431	
20535	Ovarian Carcinoma	CLEC4D	0.027133	3.620512	
20536	Ovarian Carcinoma	MGC10233	0.031326	3.995307	
20537	P1	SAA1	0.049023	-3.73593	
20538	P1	C5orf4	0.008187	2.134085	
20539	PAIN	XIST	0	2.098876	
20540	Papillary adenocarcinoma	FABP1	0	-1783.98152	
20541	Papillary adenocarcinoma	TSPAN8	0	-734.676541	
20542	Papillary adenocarcinoma	SLC26A3	0	-395.203791	
20543	Papillary adenocarcinoma	LGALS4	0	-224.746236	
20544	Papillary adenocarcinoma	CEACAM5	0	-219.84559	
20545	Papillary adenocarcinoma	C10orf99	0	-178.128302	
20546	Papillary adenocarcinoma	MUC12	0	-167.119962	
20547	Papillary adenocarcinoma	CEACAM7	0	-154.915537	
20548	Papillary adenocarcinoma	GPX2	0	-154.567716	

20549	Papillary adenocarcinoma	KRT20	0	-153.448325	
20550	Papillary adenocarcinoma	CDH17	0	-149.50701	
20551	Papillary adenocarcinoma	TFF3	0	-149.453515	
20552	Papillary adenocarcinoma	OLFM4	0	-140.332281	
20553	Papillary adenocarcinoma	S100P	0	-136.299851	
20554	Papillary adenocarcinoma	IGL@	0	-133.595203	
20555	Papillary adenocarcinoma	KIAA1324	0	-123.761955	
20556	Papillary adenocarcinoma	RPS4Y1	0	-123.588928	
20557	Papillary adenocarcinoma	CLCA1	0	-123.190859	
20558	Papillary adenocarcinoma	CLRN3	0	-117.289944	
20559	Papillary adenocarcinoma	REG4	0	-96.670892	
20560	Papillary adenocarcinoma	REG1A	0	-92.52442	
20561	Papillary adenocarcinoma	SPINK4	0	-90.100776	
20562	Papillary adenocarcinoma	DDC	0	-89.997549	
20563	Papillary adenocarcinoma	MUC2	0	-84.600435	
20564	Papillary adenocarcinoma	REG3A	0	-78.863999	
20565	Papillary adenocarcinoma	IGLV2-23	0	-77.613524	
20566	Papillary adenocarcinoma	IGHM	0	-77.183227	
20567	Papillary adenocarcinoma	IGK@	0	-76.269111	
20568	Papillary adenocarcinoma	MEP1A	0	-72.369017	
20569	Papillary adenocarcinoma	TFF1	0	-72.136157	
20570	Papillary adenocarcinoma	NOX1	0	-69.990168	
20571	Papillary adenocarcinoma	CLCA4	0	-69.233961	
20572	Papillary adenocarcinoma	CEACAM6	0	-68.670651	
20573	Papillary adenocarcinoma	ZG16	0	-68.377934	
20574	Papillary adenocarcinoma	DEFA5	0	-66.01064	
20575	Papillary adenocarcinoma	MUC13	0	-62.426339	
20576	Papillary adenocarcinoma	HMGCS2	0	-61.065686	
20577	Papillary adenocarcinoma	GPA33	0	-59.972802	
20578	Papillary adenocarcinoma	IGJ	0	-56.228497	
20579	Papillary adenocarcinoma	ASCL2	0	-55.773054	
20580	Papillary adenocarcinoma	SPINK1	0	-53.698726	
20581	Papillary adenocarcinoma	CYP2B6	0	-52.886559	
20582	Papillary adenocarcinoma	IGHA1	0	-52.816301	
20583	Papillary adenocarcinoma	UGT2B17	0	-51.817487	
20584	Papillary adenocarcinoma	ARL14	0	-50.58906	
20585	Papillary adenocarcinoma	NR1I2	0	-50.560411	
20586	Papillary adenocarcinoma	CCL14-CCL15	0	-50.166247	
20587	Papillary adenocarcinoma	TMPRSS2	0	-49.054104	
20588	Papillary adenocarcinoma	ITLN1	0	-48.78804	
20589	Papillary adenocarcinoma	RNF128	0	-48.37345	
20590	Papillary adenocarcinoma	NCRNA00261	0	-46.045832	
20591	Papillary adenocarcinoma	GUCY2C	0	-44.202045	
20592	Papillary adenocarcinoma	MMP3	0	-43.589004	
20593	Papillary adenocarcinoma	EPS8L3	0	-41.826155	
20594	Papillary adenocarcinoma	PLA2G2A	0	-41.478404	
20595	Papillary adenocarcinoma	FAM55D	0	-40.190006	
20596	Papillary adenocarcinoma	DDX3Y	0	-37.739695	
20597	Papillary adenocarcinoma	PIGR	0	-36.448948	
20598	Papillary adenocarcinoma	MS4A12	0	-35.634078	

20599	Papillary adenocarcinoma	IGKV4-1	0	-35.548321	
20600	Papillary adenocarcinoma	AGR2	0	-34.859634	
20601	Papillary adenocarcinoma	PRAC	0	-34.632391	
20602	Papillary adenocarcinoma	ZG16B	0	-32.921317	
20603	Papillary adenocarcinoma	TDGF1	0	-31.737572	
20604	Papillary adenocarcinoma	TNFRSF17	0	-31.444954	
20605	Papillary adenocarcinoma	VIL1	0	-29.601064	
20606	Papillary adenocarcinoma	CYorf15B	0	-29.362298	
20607	Papillary adenocarcinoma	USH1C	0	-29.230689	
20608	Papillary adenocarcinoma	CDX1	0	-29.218085	
20609	Papillary adenocarcinoma	CDX2	0	-29.1671	
20610	Papillary adenocarcinoma	HSD17B2	0	-29.102854	
20611	Papillary adenocarcinoma	CDHR5	0	-27.950353	
20612	Papillary adenocarcinoma	IGLC7	0	-27.852389	
20613	Papillary adenocarcinoma	TRIM15	0	-27.716643	
20614	Papillary adenocarcinoma	ADH1C	0	-27.259597	
20615	Papillary adenocarcinoma	TMEM45B	0	-26.912887	
20616	Papillary adenocarcinoma	PRSS3	0	-26.772156	
20617	Papillary adenocarcinoma	AZGP1	0	-26.027685	
20618	Papillary adenocarcinoma	DUOX2	0	-25.195539	
20619	Papillary adenocarcinoma	EIF1AY	0	-24.807507	
20620	Papillary adenocarcinoma	CCL20	0	-24.178354	
20621	Papillary adenocarcinoma	HEPACAM2	0	-24.016373	
20622	Papillary adenocarcinoma	COL17A1	0	-23.746549	
20623	Papillary adenocarcinoma	C17orf73	0	-23.489011	
20624	Papillary adenocarcinoma	PLAC8	0	-23.193952	
20625	Papillary adenocarcinoma	CEACAM1	0	-23.100644	
20626	Papillary adenocarcinoma	HOXB13	0	-22.00054	
20627	Papillary adenocarcinoma	POF1B	0	-21.924313	
20628	Papillary adenocarcinoma	RETNLB	0	-21.523907	
20629	Papillary adenocarcinoma	ATP10B	0	-21.440224	
20630	Papillary adenocarcinoma	HOXA10	0	-20.502457	
20631	Papillary adenocarcinoma	IGLV1-36	0	-20.41857	
20632	Papillary adenocarcinoma	ATP2A3	0	-20.352857	
20633	Papillary adenocarcinoma	IGKC	0	-20.220252	
20634	Papillary adenocarcinoma	DPEP1	0	-20.1644	
20635	Papillary adenocarcinoma	CELP	0	-19.979544	
20636	Papillary adenocarcinoma	CDA	0	-19.848012	
20637	Papillary adenocarcinoma	PARM1	0	-19.524159	
20638	Papillary adenocarcinoma	TRIM31	0	-19.427096	
20639	Papillary adenocarcinoma	C4BPB	0	-18.999893	
20640	Papillary adenocarcinoma	ISX	0	-18.740554	
20641	Papillary adenocarcinoma	IGKV3-20	0	-18.577828	
20642	Papillary adenocarcinoma	UGT1A1	0	-18.403763	
20643	Papillary adenocarcinoma	AREG	0	-18.233147	
20644	Papillary adenocarcinoma	CENPV	0	-17.999801	
20645	Papillary adenocarcinoma	LEFTY1	0	-17.105627	
20646	Papillary adenocarcinoma	HOXA13	0	-16.89773	
20647	Papillary adenocarcinoma	LGR5	0	-16.892466	
20648	Papillary adenocarcinoma	AKR1B10	0	-16.655681	

20649	Papillary adenocarcinoma	TOX3	0	-16.403757	
20650	Papillary adenocarcinoma	FUT6	0	-16.370011	
20651	Papillary adenocarcinoma	LRRC19	0	-16.268962	
20652	Papillary adenocarcinoma	SI	0	-16.15328	
20653	Papillary adenocarcinoma	CYorf15A	0	-16.099526	
20654	Papillary adenocarcinoma	AQP8	0	-15.99193	
20655	Papillary adenocarcinoma	TRAC	0	-15.885642	
20656	Papillary adenocarcinoma	DSG3	0	-15.808497	
20657	Papillary adenocarcinoma	RNF186	0	-15.808286	
20658	Papillary adenocarcinoma	LOC646627	0	-15.693473	
20659	Papillary adenocarcinoma	NR1H4	0	-15.617785	
20660	Papillary adenocarcinoma	MAOA	0	-15.543043	
20661	Papillary adenocarcinoma	HNF4A	0	-15.294299	
20662	Papillary adenocarcinoma	LOC100288092	0	-15.21226	
20663	Papillary adenocarcinoma	KDM5D	0	-15.210829	
20664	Papillary adenocarcinoma	IGLL3P	0	-15.202068	
20665	Papillary adenocarcinoma	PCK1	0	-14.874927	
20666	Papillary adenocarcinoma	UGT2A3	0	-14.604468	
20667	Papillary adenocarcinoma	TMC5	0	-14.436054	
20668	Papillary adenocarcinoma	SOX8	0	-14.423931	
20669	Papillary adenocarcinoma	RASSF6	0	-13.855458	
20670	Papillary adenocarcinoma	ACSL5	0	-13.618466	
20671	Papillary adenocarcinoma	DMBT1	0	-13.498516	
20672	Papillary adenocarcinoma	CA4	0	-13.274225	
20673	Papillary adenocarcinoma	VSNL1	0	-13.219894	
20674	Papillary adenocarcinoma	CST1	0	-13.113854	
20675	Papillary adenocarcinoma	SSTR1	0	-13.016504	
20676	Papillary adenocarcinoma	PNLIPRP2	0	-12.906909	
20677	Papillary adenocarcinoma	IGLL5	0	-12.823177	
20678	Papillary adenocarcinoma	TMED6	0	-12.803579	
20679	Papillary adenocarcinoma	SLC39A5	0	-12.669456	
20680	Papillary adenocarcinoma	FAM55A	0	-12.459692	
20681	Papillary adenocarcinoma	IGLV1-40	0	-12.275435	
20682	Papillary adenocarcinoma	PLCB4	0	-12.157713	
20683	Papillary adenocarcinoma	UGT2B15	0	-11.979497	
20684	Papillary adenocarcinoma	WNK4	0	-11.534228	
20685	Papillary adenocarcinoma	LOC100507192	0	-11.479024	
20686	Papillary adenocarcinoma	LOC96610	0	-11.245215	
20687	Papillary adenocarcinoma	SERPINB5	0	-11.212683	
20688	Papillary adenocarcinoma	CYP2C18	0	-11.177066	
20689	Papillary adenocarcinoma	GPR160	0	-11.017795	
20690	Papillary adenocarcinoma	FGFR4	0	-11.010292	
20691	Papillary adenocarcinoma	DGAT2	0	-10.896638	
20692	Papillary adenocarcinoma	AHNAK	0	-10.775066	
20693	Papillary adenocarcinoma	IGLJ3	0	-10.591129	
20694	Papillary adenocarcinoma	FAM40B	0	-10.51281	
20695	Papillary adenocarcinoma	C19orf77	0	-10.374184	
20696	Papillary adenocarcinoma	HLA-DQA1	0	-10.373276	
20697	Papillary adenocarcinoma	MMP1	0	-10.323301	
20698	Papillary adenocarcinoma	LY6G6D	0	-10.286764	

20699	Papillary adenocarcinoma	PIP5K1B	0	-10.25627	
20700	Papillary adenocarcinoma	EREG	0	-10.228443	
20701	Papillary adenocarcinoma	SLCO1B3	0	-10.042318	
20702	Papillary adenocarcinoma	RCBTB1	0	-10.034885	
20703	Papillary adenocarcinoma	C20orf118	0	-10.001177	
20704	Papillary adenocarcinoma	CA1	0	-9.999398	
20705	Papillary adenocarcinoma	SLC27A2	0	-9.856283	
20706	Papillary adenocarcinoma	MUC17	0	-9.634307	
20707	Papillary adenocarcinoma	SCARA5	0	-9.554463	
20708	Papillary adenocarcinoma	SATB2	0	-9.548549	
20709	Papillary adenocarcinoma	AKR1C3	0	-9.427411	
20710	Papillary adenocarcinoma	ETS2	0	-9.415206	
20711	Papillary adenocarcinoma	ADAMDEC1	0	-9.363608	
20712	Papillary adenocarcinoma	P2RX5	0	-9.307098	
20713	Papillary adenocarcinoma	BTNL8	0	-9.241355	
20714	Papillary adenocarcinoma	SLC25A15	0	-9.229452	
20715	Papillary adenocarcinoma	GUCA2A	0	-9.215059	
20716	Papillary adenocarcinoma	SLC4A4	0	-9.20736	
20717	Papillary adenocarcinoma	C1orf125	0	-9.090797	
20718	Papillary adenocarcinoma	C2orf89	0	-9.032453	
20719	Papillary adenocarcinoma	KDM4C	0	-9.018504	
20720	Papillary adenocarcinoma	C2CD4A	0	-9.005936	
20721	Papillary adenocarcinoma	CHP2	0	-8.957452	
20722	Papillary adenocarcinoma	C21orf88	0	-8.922461	
20723	Papillary adenocarcinoma	FOXD1	0	-8.920909	
20724	Papillary adenocarcinoma	MYB	0	-8.801258	
20725	Papillary adenocarcinoma	SLC6A14	0	-8.79646	
20726	Papillary adenocarcinoma	ACE2	0	-8.746041	
20727	Papillary adenocarcinoma	PROX1	0	-8.718629	
20728	Papillary adenocarcinoma	LGALS2	0	-8.707535	
20729	Papillary adenocarcinoma	TPSAB1	0	-8.50733	
20730	Papillary adenocarcinoma	IGHG1	0	-8.424752	
20731	Papillary adenocarcinoma	SLC3A1	0	-8.361984	
20732	Papillary adenocarcinoma	GREM2	0	-8.354549	
20733	Papillary adenocarcinoma	PTPRN2	0	-8.352914	
20734	Papillary adenocarcinoma	GNG4	0	-8.351523	
20735	Papillary adenocarcinoma	WNK2	0	-8.32134	
20736	Papillary adenocarcinoma	NEBL	0	-8.254719	
20737	Papillary adenocarcinoma	FIBCD1	0	-8.249466	
20738	Papillary adenocarcinoma	C6orf105	0	-8.167827	
20739	Papillary adenocarcinoma	CTSE	0	-8.165862	
20740	Papillary adenocarcinoma	NQO1	0	-8.049924	
20741	Papillary adenocarcinoma	SCD	0	-8.03653	
20742	Papillary adenocarcinoma	ARSE	0	-8.021958	
20743	Papillary adenocarcinoma	SLC1A7	0	-7.746176	
20744	Papillary adenocarcinoma	IHH	0	-7.744896	
20745	Papillary adenocarcinoma	FAR2	0	-7.688133	
20746	Papillary adenocarcinoma	TAS2R16	0	-7.370474	
20747	Papillary adenocarcinoma	GABBR1	0	-7.329296	
20748	Papillary adenocarcinoma	NCRNA00238	0	-7.298512	

20749	Papillary adenocarcinoma	POU2AF1	0	-7.135486	
20750	Papillary adenocarcinoma	SLC5A9	0	-7.120683	
20751	Papillary adenocarcinoma	P2RY13	0	-7.08136	
20752	Papillary adenocarcinoma	SLA2	0	-7.064962	
20753	Papillary adenocarcinoma	C16orf81	0	-7.049039	
20754	Papillary adenocarcinoma	C13orf18	0	-6.929746	
20755	Papillary adenocarcinoma	ZNF460	0	-6.864245	
20756	Papillary adenocarcinoma	FUT4	0	-6.810964	
20757	Papillary adenocarcinoma	GMDS	0	-6.762556	
20758	Papillary adenocarcinoma	MARVELD3	0	-6.72383	
20759	Papillary adenocarcinoma	LOC285943	0	-6.706774	
20760	Papillary adenocarcinoma	IGHD	0	-6.691216	
20761	Papillary adenocarcinoma	URB1	0	-6.648354	
20762	Papillary adenocarcinoma	AXIN2	0	-6.485979	
20763	Papillary adenocarcinoma	TRPM6	0	-6.44014	
20764	Papillary adenocarcinoma	CAPN9	0	-6.394351	
20765	Papillary adenocarcinoma	SMOX	0	-6.363351	
20766	Papillary adenocarcinoma	CCL14	0	-6.359321	
20767	Papillary adenocarcinoma	ITGA6	0	-6.324961	
20768	Papillary adenocarcinoma	EFCAB4B	0	-6.270735	
20769	Papillary adenocarcinoma	VSIG2	0	-6.270541	
20770	Papillary adenocarcinoma	NPNT	0	-6.183007	
20771	Papillary adenocarcinoma	HOXD13	0	-6.161745	
20772	Papillary adenocarcinoma	RAB3B	0	-6.159617	
20773	Papillary adenocarcinoma	SLC44A3	0	-6.147693	
20774	Papillary adenocarcinoma	ABCB1	0	-6.08622	
20775	Papillary adenocarcinoma	PCCA	0	-5.998867	
20776	Papillary adenocarcinoma	HES6	0	-5.854675	
20777	Papillary adenocarcinoma	PPP1R1B	0	-5.851692	
20778	Papillary adenocarcinoma	SKP2	0	-5.764666	
20779	Papillary adenocarcinoma	SMYD1	0	-5.746991	
20780	Papillary adenocarcinoma	GAD1	0	-5.746076	
20781	Papillary adenocarcinoma	PIWIL1	0	-5.529757	
20782	Papillary adenocarcinoma	IL2RG	0	-5.508244	
20783	Papillary adenocarcinoma	TMEM54	0	-5.487605	
20784	Papillary adenocarcinoma	RNASE4	0	-5.421645	
20785	Papillary adenocarcinoma	ENPP3	0	-5.400357	
20786	Papillary adenocarcinoma	ATP2A1	0	-5.396463	
20787	Papillary adenocarcinoma	SLAMF1	0	-5.291965	
20788	Papillary adenocarcinoma	OFCC1	0	-5.211818	
20789	Papillary adenocarcinoma	TTLL6	0	-5.199469	
20790	Papillary adenocarcinoma	DNAJC12	0	-5.177963	
20791	Papillary adenocarcinoma	ANG	0	-5.147582	
20792	Papillary adenocarcinoma	CTLA4	0	-5.145889	
20793	Papillary adenocarcinoma	COL4A4	0	-5.087308	
20794	Papillary adenocarcinoma	KCNJ13	0	-5.084975	
20795	Papillary adenocarcinoma	SMAGP	0	-5.061199	
20796	Papillary adenocarcinoma	SAMD5	0	-5.017224	
20797	Papillary adenocarcinoma	DMRT2	0	-5.008078	
20798	Papillary adenocarcinoma	AMBRA1	0	-5.001326	

20799	Papillary adenocarcinoma	SLC26A2	0	-4.956791	
20800	Papillary adenocarcinoma	MSI2	0	-4.956291	
20801	Papillary adenocarcinoma	FCGBP	0	-4.925889	
20802	Papillary adenocarcinoma	RND1	0	-4.914964	
20803	Papillary adenocarcinoma	TFPI	0	-4.885753	
20804	Papillary adenocarcinoma	HAVCR2	0	-4.873647	
20805	Papillary adenocarcinoma	LOC283516	0	-4.85353	
20806	Papillary adenocarcinoma	CHRDL1	0	-4.850918	
20807	Papillary adenocarcinoma	GPSM2	0	-4.791922	
20808	Papillary adenocarcinoma	GAL	0	-4.764126	
20809	Papillary adenocarcinoma	FAM83E	0	-4.704437	
20810	Papillary adenocarcinoma	TTBK2	0	-4.683085	
20811	Papillary adenocarcinoma	ANK2	0	-4.636445	
20812	Papillary adenocarcinoma	PLA1A	0	-4.624572	
20813	Papillary adenocarcinoma	KAZALD1	0	-4.614068	
20814	Papillary adenocarcinoma	HS6ST2	0	-4.579907	
20815	Papillary adenocarcinoma	CDC42EP5	0	-4.511951	
20816	Papillary adenocarcinoma	CKB	0	-4.506526	
20817	Papillary adenocarcinoma	KBTBD11	0	-4.504592	
20818	Papillary adenocarcinoma	PTPRH	0	-4.45561	
20819	Papillary adenocarcinoma	MFSD4	0	-4.441447	
20820	Papillary adenocarcinoma	ME1	0	-4.421035	
20821	Papillary adenocarcinoma	LOC100240728	0	-4.405836	
20822	Papillary adenocarcinoma	DUSP21	0	-4.403635	
20823	Papillary adenocarcinoma	TRBC1	0	-4.392807	
20824	Papillary adenocarcinoma	RARA	0	-4.360997	
20825	Papillary adenocarcinoma	ZAK	0	-4.31007	
20826	Papillary adenocarcinoma	COL15A1	0	-4.247255	
20827	Papillary adenocarcinoma	UPB1	0	-4.197305	
20828	Papillary adenocarcinoma	CCND2	0	-4.160703	
20829	Papillary adenocarcinoma	MYOCD	0	-4.145254	
20830	Papillary adenocarcinoma	CD1B	0	-3.980773	
20831	Papillary adenocarcinoma	AGPAT3	0	-3.783545	
20832	Papillary adenocarcinoma	PDE5A	0	-3.764329	
20833	Papillary adenocarcinoma	PBLD	0	-3.754507	
20834	Papillary adenocarcinoma	CLDN15	0	-3.666112	
20835	Papillary adenocarcinoma	PIGZ	0	-3.657187	
20836	Papillary adenocarcinoma	RBMY1A1	0	-3.621811	
20837	Papillary adenocarcinoma	P2RY14	0	-3.609094	
20838	Papillary adenocarcinoma	TTC6	0	-3.584985	
20839	Papillary adenocarcinoma	NRN1	0	-3.52086	
20840	Papillary adenocarcinoma	PKDCC	0	-3.503283	
20841	Papillary adenocarcinoma	GRIN1	0	-3.471223	
20842	Papillary adenocarcinoma	ELMOD2	0	-3.435836	
20843	Papillary adenocarcinoma	PIWIL4	0	-3.434728	
20844	Papillary adenocarcinoma	CHPT1	0	-3.408503	
20845	Papillary adenocarcinoma	GRK5	0	-3.387093	
20846	Papillary adenocarcinoma	SCLY	0	-3.374821	
20847	Papillary adenocarcinoma	LOC644090	0	-3.26994	
20848	Papillary adenocarcinoma	ABAT	0	-3.089712	

20849	Papillary adenocarcinoma	EGFR	0	-2.981094	
20850	Papillary adenocarcinoma	C19orf45	0	-2.974887	
20851	Papillary adenocarcinoma	RNF6	0	-2.929863	
20852	Papillary adenocarcinoma	PADI2	0	-2.927352	
20853	Papillary adenocarcinoma	REEP1	0	-2.916067	
20854	Papillary adenocarcinoma	RBM33	0	-2.901821	
20855	Papillary adenocarcinoma	LOC25845	0	-2.887602	
20856	Papillary adenocarcinoma	PXMP4	0	-2.848437	
20857	Papillary adenocarcinoma	LNX2	0	-2.820126	
20858	Papillary adenocarcinoma	LOC283508	0	-2.792945	
20859	Papillary adenocarcinoma	LONRF3	0	-2.784971	
20860	Papillary adenocarcinoma	C19orf6	0	-2.736061	
20861	Papillary adenocarcinoma	STEAP1	0	-2.644491	
20862	Papillary adenocarcinoma	STMN2	0	-2.588828	
20863	Papillary adenocarcinoma	ITGA1	0	-2.583244	
20864	Papillary adenocarcinoma	CIT	0	-2.561015	
20865	Papillary adenocarcinoma	SYTL2	0	-2.554634	
20866	Papillary adenocarcinoma	ZMYM5	0	-2.500843	
20867	Papillary adenocarcinoma	SPTBN1	0	-2.472947	
20868	Papillary adenocarcinoma	PSD3	0	-2.370799	
20869	Papillary adenocarcinoma	CD300A	0	-2.357685	
20870	Papillary adenocarcinoma	NOXO1	0	-2.356434	
20871	Papillary adenocarcinoma	WSCD1	0	-2.333715	
20872	Papillary adenocarcinoma	FGL2	0	-2.255058	
20873	Papillary adenocarcinoma	HLA-G	0	-2.248603	
20874	Papillary adenocarcinoma	KBTBD2	0	-2.136272	
20875	Papillary adenocarcinoma	LCP2	0	-2.102294	
20876	Papillary adenocarcinoma	PTPRC	0	-2.080858	
20877	Papillary adenocarcinoma	MGC45800	0	-2.075695	
20878	Papillary adenocarcinoma	PIGG	0	-2.064807	
20879	Papillary adenocarcinoma	SCIN	0	-2.052243	
20880	Papillary adenocarcinoma	CTTNBP2NL	0	-2.046079	
20881	Papillary adenocarcinoma	BZW1	0	-2.03949	
20882	Papillary adenocarcinoma	ENPP2	0	-2.004327	
20883	Papillary serous cystadenocarcinoma	FABP1	0	-1813.6329	
20884	Papillary serous cystadenocarcinoma	SLC26A3	0	-218.117964	
20885	Papillary serous cystadenocarcinoma	CEACAM5	0	-217.642189	
20886	Papillary serous cystadenocarcinoma	LGALS4	0	-190.908413	
20887	Papillary serous cystadenocarcinoma	MUC12	0	-156.084886	
20888	Papillary serous cystadenocarcinoma	OLFM4	0	-142.770959	
20889	Papillary serous cystadenocarcinoma	GPX2	0	-141.384873	
20890	Papillary serous cystadenocarcinoma	CDH17	0	-131.255404	
20891	Papillary serous cystadenocarcinoma	CLCA1	0	-119.870007	
20892	Papillary serous cystadenocarcinoma	CEACAM7	0	-111.453658	
20893	Papillary serous cystadenocarcinoma	S100P	0	-104.454295	
20894	Papillary serous cystadenocarcinoma	CEACAM6	0	-97.93911	
20895	Papillary serous cystadenocarcinoma	CLRN3	0	-93.927435	
20896	Papillary serous cystadenocarcinoma	REG4	0	-90.9717	
20897	Papillary serous cystadenocarcinoma	KRT20	0	-88.831716	
20898	Papillary serous cystadenocarcinoma	REG1B	0	-85.184926	

20899	Papillary serous cystadenocarcinoma	TFF3	0	-80.705014	
20900	Papillary serous cystadenocarcinoma	MUC13	0	-71.787252	
20901	Papillary serous cystadenocarcinoma	ZG16	0	-71.087054	
20902	Papillary serous cystadenocarcinoma	C10orf99	0	-69.125517	
20903	Papillary serous cystadenocarcinoma	MUC2	0	-68.86284	
20904	Papillary serous cystadenocarcinoma	CCL14-CCL15	0	-68.72669	
20905	Papillary serous cystadenocarcinoma	NOX1	0	-66.173947	
20906	Papillary serous cystadenocarcinoma	REG1A	0	-61.971075	
20907	Papillary serous cystadenocarcinoma	SPINK1	0	-60.895576	
20908	Papillary serous cystadenocarcinoma	SPINK4	0	-51.233923	
20909	Papillary serous cystadenocarcinoma	REG3A	0	-49.425476	
20910	Papillary serous cystadenocarcinoma	MEP1A	0	-47.882347	
20911	Papillary serous cystadenocarcinoma	MMP3	0	-45.377726	
20912	Papillary serous cystadenocarcinoma	UGT2B17	0	-44.019805	
20913	Papillary serous cystadenocarcinoma	MS4A12	0	-41.249497	
20914	Papillary serous cystadenocarcinoma	TFF1	0	-36.003164	
20915	Papillary serous cystadenocarcinoma	ITLN1	0	-35.803322	
20916	Papillary serous cystadenocarcinoma	RNF128	0	-33.813266	
20917	Papillary serous cystadenocarcinoma	DEFA5	0	-31.955893	
20918	Papillary serous cystadenocarcinoma	ADH1C	0	-30.7402	
20919	Papillary serous cystadenocarcinoma	CDHR5	0	-30.683488	
20920	Papillary serous cystadenocarcinoma	VIL1	0	-29.57058	
20921	Papillary serous cystadenocarcinoma	GPA33	0	-29.191359	
20922	Papillary serous cystadenocarcinoma	CDX1	0	-28.640954	
20923	Papillary serous cystadenocarcinoma	EPS8L3	0	-28.145755	
20924	Papillary serous cystadenocarcinoma	CCL20	0	-27.766552	
20925	Papillary serous cystadenocarcinoma	CDX2	0	-27.762252	
20926	Papillary serous cystadenocarcinoma	ARL14	0	-27.600398	
20927	Papillary serous cystadenocarcinoma	TRIM31	0	-25.821363	
20928	Papillary serous cystadenocarcinoma	NR1I2	0	-25.737503	
20929	Papillary serous cystadenocarcinoma	TSPAN8	0	-25.655004	
20930	Papillary serous cystadenocarcinoma	DUOX2	0	-25.015621	
20931	Papillary serous cystadenocarcinoma	TDGF1	0	-24.871086	
20932	Papillary serous cystadenocarcinoma	CYP2B6	0	-24.538568	
20933	Papillary serous cystadenocarcinoma	MUC3B	0	-24.248161	
20934	Papillary serous cystadenocarcinoma	CLCA4	0	-24.246042	
20935	Papillary serous cystadenocarcinoma	LOC646627	0	-24.110677	
20936	Papillary serous cystadenocarcinoma	GUCY2C	0	-23.741122	
20937	Papillary serous cystadenocarcinoma	PLAC8	0	-23.702882	
20938	Papillary serous cystadenocarcinoma	POF1B	0	-23.469619	
20939	Papillary serous cystadenocarcinoma	DPEP1	0	-23.153817	
20940	Papillary serous cystadenocarcinoma	ATP10B	0	-22.482454	
20941	Papillary serous cystadenocarcinoma	RETNLB	0	-21.523836	
20942	Papillary serous cystadenocarcinoma	NCRNA00261	0	-21.06436	
20943	Papillary serous cystadenocarcinoma	CEACAM1	0	-21.038582	
20944	Papillary serous cystadenocarcinoma	AREG	0	-20.56819	
20945	Papillary serous cystadenocarcinoma	HSD17B2	0	-20.2349	
20946	Papillary serous cystadenocarcinoma	COL17A1	0	-19.819643	
20947	Papillary serous cystadenocarcinoma	HNF4A	0	-19.625474	
20948	Papillary serous cystadenocarcinoma	PIGR	0	-19.236222	

20949	Papillary serous cystadenocarcinoma	ASCL2	0	-19.102645	
20950	Papillary serous cystadenocarcinoma	FAM55D	0	-17.83085	
20951	Papillary serous cystadenocarcinoma	USH1C	0	-17.272388	
20952	Papillary serous cystadenocarcinoma	FUT6	0	-17.213828	
20953	Papillary serous cystadenocarcinoma	FERMT1	0	-16.900577	
20954	Papillary serous cystadenocarcinoma	TOX3	0	-15.604274	
20955	Papillary serous cystadenocarcinoma	TRIM15	0	-15.581619	
20956	Papillary serous cystadenocarcinoma	PRSS3	0	-15.530121	
20957	Papillary serous cystadenocarcinoma	LRRC19	0	-15.392121	
20958	Papillary serous cystadenocarcinoma	RNF186	0	-15.251615	
20959	Papillary serous cystadenocarcinoma	DDC	0	-15.110289	
20960	Papillary serous cystadenocarcinoma	PLA2G2A	0	-14.974467	
20961	Papillary serous cystadenocarcinoma	UGT1A1	0	-14.788082	
20962	Papillary serous cystadenocarcinoma	CELP	0	-14.603192	
20963	Papillary serous cystadenocarcinoma	DSG3	0	-14.453225	
20964	Papillary serous cystadenocarcinoma	LEFTY1	0	-14.376584	
20965	Papillary serous cystadenocarcinoma	AKR1C3	0	-14.248165	
20966	Papillary serous cystadenocarcinoma	C17orf73	0	-13.801599	
20967	Papillary serous cystadenocarcinoma	IGHA1	0	-13.771468	
20968	Papillary serous cystadenocarcinoma	DMBT1	0	-13.565916	
20969	Papillary serous cystadenocarcinoma	PPARG	0	-13.393906	
20970	Papillary serous cystadenocarcinoma	RAPGEFL1	0	-13.189812	
20971	Papillary serous cystadenocarcinoma	HMGCS2	0	-13.030134	
20972	Papillary serous cystadenocarcinoma	TFF2	0	-12.932018	
20973	Papillary serous cystadenocarcinoma	C4BPB	0	-12.376787	
20974	Papillary serous cystadenocarcinoma	UGT2A3	0	-12.178351	
20975	Papillary serous cystadenocarcinoma	ACSL5	0	-12.155901	
20976	Papillary serous cystadenocarcinoma	HEPACAM2	0	-12.142406	
20977	Papillary serous cystadenocarcinoma	SLC39A5	0	-11.938082	
20978	Papillary serous cystadenocarcinoma	AQP8	0	-11.93172	
20979	Papillary serous cystadenocarcinoma	ISX	0	-11.491018	
20980	Papillary serous cystadenocarcinoma	IGJ	0	-11.445576	
20981	Papillary serous cystadenocarcinoma	C2orf89	0	-11.278608	
20982	Papillary serous cystadenocarcinoma	LGR5	0	-11.107126	
20983	Papillary serous cystadenocarcinoma	IYD	0	-11.014753	
20984	Papillary serous cystadenocarcinoma	LY6G6D	0	-10.887675	
20985	Papillary serous cystadenocarcinoma	NAT2	0	-10.441214	
20986	Papillary serous cystadenocarcinoma	CDA	0	-10.359094	
20987	Papillary serous cystadenocarcinoma	ETS2	0	-10.335517	
20988	Papillary serous cystadenocarcinoma	WNK4	0	-10.19815	
20989	Papillary serous cystadenocarcinoma	AGR2	0	-10.189468	
20990	Papillary serous cystadenocarcinoma	ACE2	0	-10.048438	
20991	Papillary serous cystadenocarcinoma	C1orf125	0	-10.009882	
20992	Papillary serous cystadenocarcinoma	MUC17	0	-9.947927	
20993	Papillary serous cystadenocarcinoma	SERPINB5	0	-9.841761	
20994	Papillary serous cystadenocarcinoma	PLS1	0	-9.821578	
20995	Papillary serous cystadenocarcinoma	EREG	0	-9.780134	
20996	Papillary serous cystadenocarcinoma	CFTR	0	-9.694404	
20997	Papillary serous cystadenocarcinoma	PRAP1	0	-9.583558	
20998	Papillary serous cystadenocarcinoma	ARSE	0	-9.513805	

20999	Papillary serous cystadenocarcinoma	CST1	0	-9.288534	
21000	Papillary serous cystadenocarcinoma	C2CD4A	0	-9.272186	
21001	Papillary serous cystadenocarcinoma	IL8	0	-9.060437	
21002	Papillary serous cystadenocarcinoma	PCK1	0	-9.055649	
21003	Papillary serous cystadenocarcinoma	GIPC2	0	-9.041035	
21004	Papillary serous cystadenocarcinoma	CHP2	0	-9.036996	
21005	Papillary serous cystadenocarcinoma	LOC100288092	0	-8.902245	
21006	Papillary serous cystadenocarcinoma	SDR16C5	0	-8.870465	
21007	Papillary serous cystadenocarcinoma	PIP5K1B	0	-8.694881	
21008	Papillary serous cystadenocarcinoma	FGFR4	0	-8.671163	
21009	Papillary serous cystadenocarcinoma	BCL2L15	0	-8.669141	
21010	Papillary serous cystadenocarcinoma	IHH	0	-8.565944	
21011	Papillary serous cystadenocarcinoma	LOC283859	0	-8.559038	
21012	Papillary serous cystadenocarcinoma	BCL2L14	0	-8.53957	
21013	Papillary serous cystadenocarcinoma	C13orf18	0	-8.364655	
21014	Papillary serous cystadenocarcinoma	CA4	0	-8.363636	
21015	Papillary serous cystadenocarcinoma	CA2	0	-8.260271	
21016	Papillary serous cystadenocarcinoma	GUCA2A	0	-8.255475	
21017	Papillary serous cystadenocarcinoma	MMP1	0	-8.242392	
21018	Papillary serous cystadenocarcinoma	BTNL8	0	-8.156451	
21019	Papillary serous cystadenocarcinoma	CDCA7	0	-8.093391	
21020	Papillary serous cystadenocarcinoma	HNF4G	0	-8.076875	
21021	Papillary serous cystadenocarcinoma	FLJ32063	0	-7.980502	
21022	Papillary serous cystadenocarcinoma	UGT8	0	-7.913594	
21023	Papillary serous cystadenocarcinoma	BAIAP2L2	0	-7.907898	
21024	Papillary serous cystadenocarcinoma	PLCB4	0	-7.780927	
21025	Papillary serous cystadenocarcinoma	PARM1	0	-7.767468	
21026	Papillary serous cystadenocarcinoma	SLCO1B3	0	-7.756764	
21027	Papillary serous cystadenocarcinoma	CA1	0	-7.695016	
21028	Papillary serous cystadenocarcinoma	ADAMDEC1	0	-7.69323	
21029	Papillary serous cystadenocarcinoma	ITGA2	0	-7.676935	
21030	Papillary serous cystadenocarcinoma	FUT4	0	-7.584596	
21031	Papillary serous cystadenocarcinoma	MYO7B	0	-7.581547	
21032	Papillary serous cystadenocarcinoma	FAM3D	0	-7.574605	
21033	Papillary serous cystadenocarcinoma	CTSE	0	-7.544051	
21034	Papillary serous cystadenocarcinoma	C19orf77	0	-7.541781	
21035	Papillary serous cystadenocarcinoma	CALML4	0	-7.481745	
21036	Papillary serous cystadenocarcinoma	TMEM37	0	-7.330929	
21037	Papillary serous cystadenocarcinoma	FAR2	0	-7.298522	
21038	Papillary serous cystadenocarcinoma	ABCC2	0	-7.245633	
21039	Papillary serous cystadenocarcinoma	SATB2	0	-7.241952	
21040	Papillary serous cystadenocarcinoma	HSD11B2	0	-7.186406	
21041	Papillary serous cystadenocarcinoma	FOXA3	0	-7.145027	
21042	Papillary serous cystadenocarcinoma	TMED6	0	-7.125246	
21043	Papillary serous cystadenocarcinoma	FCGBP	0	-7.091029	
21044	Papillary serous cystadenocarcinoma	SLC25A15	0	-7.082767	
21045	Papillary serous cystadenocarcinoma	FXYD3	0	-7.045833	
21046	Papillary serous cystadenocarcinoma	PNLIPRP2	0	-7.032782	
21047	Papillary serous cystadenocarcinoma	GPR120	0	-7.023061	
21048	Papillary serous cystadenocarcinoma	NQO1	0	-6.895859	

21049	Papillary serous cystadenocarcinoma	TFPI	0	-6.7882	
21050	Papillary serous cystadenocarcinoma	DHRS9	0	-6.719865	
21051	Papillary serous cystadenocarcinoma	SLC12A2	0	-6.643269	
21052	Papillary serous cystadenocarcinoma	CHRDL2	0	-6.553529	
21053	Papillary serous cystadenocarcinoma	GPR109B	0	-6.448091	
21054	Papillary serous cystadenocarcinoma	FAM84A	0	-6.387531	
21055	Papillary serous cystadenocarcinoma	GMDS	0	-6.38682	
21056	Papillary serous cystadenocarcinoma	HES6	0	-6.380977	
21057	Papillary serous cystadenocarcinoma	CCL18	0	-6.364031	
21058	Papillary serous cystadenocarcinoma	XK	0	-6.362826	
21059	Papillary serous cystadenocarcinoma	FUT3	0	-6.347937	
21060	Papillary serous cystadenocarcinoma	PROX1	0	-6.345763	
21061	Papillary serous cystadenocarcinoma	ABCB1	0	-6.303167	
21062	Papillary serous cystadenocarcinoma	MYO1A	0	-6.250814	
21063	Papillary serous cystadenocarcinoma	PLEK2	0	-6.218339	
21064	Papillary serous cystadenocarcinoma	ITPKA	0	-6.19036	
21065	Papillary serous cystadenocarcinoma	ATP2A3	0	-6.188264	
21066	Papillary serous cystadenocarcinoma	XPNPEP2	0	-6.152923	
21067	Papillary serous cystadenocarcinoma	RNASE4	0	-6.130707	
21068	Papillary serous cystadenocarcinoma	UGT2B15	0	-6.064037	
21069	Papillary serous cystadenocarcinoma	SLC1A7	0	-5.961814	
21070	Papillary serous cystadenocarcinoma	MYB	0	-5.956076	
21071	Papillary serous cystadenocarcinoma	CYP3A5	0	-5.895882	
21072	Papillary serous cystadenocarcinoma	GPSM2	0	-5.889795	
21073	Papillary serous cystadenocarcinoma	TM4SF20	0	-5.863566	
21074	Papillary serous cystadenocarcinoma	SLCO4A1	0	-5.822418	
21075	Papillary serous cystadenocarcinoma	CXCL2	0	-5.806976	
21076	Papillary serous cystadenocarcinoma	L1TD1	0	-5.790944	
21077	Papillary serous cystadenocarcinoma	IL32	0	-5.754097	
21078	Papillary serous cystadenocarcinoma	PTPRH	0	-5.753249	
21079	Papillary serous cystadenocarcinoma	LRP4	0	-5.723495	
21080	Papillary serous cystadenocarcinoma	TINAG	0	-5.668535	
21081	Papillary serous cystadenocarcinoma	PTPRO	0	-5.65315	
21082	Papillary serous cystadenocarcinoma	HEPH	0	-5.62032	
21083	Papillary serous cystadenocarcinoma	LOC100507192	0	-5.613288	
21084	Papillary serous cystadenocarcinoma	ANG	0	-5.612826	
21085	Papillary serous cystadenocarcinoma	SLC6A14	0	-5.601961	
21086	Papillary serous cystadenocarcinoma	C6orf105	0	-5.600973	
21087	Papillary serous cystadenocarcinoma	LOC100505633	0	-5.590267	
21088	Papillary serous cystadenocarcinoma	SLC5A9	0	-5.542619	
21089	Papillary serous cystadenocarcinoma	PCCA	0	-5.535052	
21090	Papillary serous cystadenocarcinoma	FAM55A	0	-5.484236	
21091	Papillary serous cystadenocarcinoma	TRPA1	0	-5.467421	
21092	Papillary serous cystadenocarcinoma	IL1R2	0	-5.456309	
21093	Papillary serous cystadenocarcinoma	NOTUM	0	-5.443763	
21094	Papillary serous cystadenocarcinoma	LOC400573	0	-5.41785	
21095	Papillary serous cystadenocarcinoma	CLDN2	0	-5.403485	
21096	Papillary serous cystadenocarcinoma	MXD1	0	-5.371909	
21097	Papillary serous cystadenocarcinoma	FA2H	0	-5.364683	
21098	Papillary serous cystadenocarcinoma	ADCYAP1	0	-5.354255	

21099	Papillary serous cystadenocarcinoma	FIBCD1	0	-5.344777	
21100	Papillary serous cystadenocarcinoma	IL1A	0	-5.302392	
21101	Papillary serous cystadenocarcinoma	BCAN	0	-5.288358	
21102	Papillary serous cystadenocarcinoma	IL22RA1	0	-5.249938	
21103	Papillary serous cystadenocarcinoma	VAV3	0	-5.248442	
21104	Papillary serous cystadenocarcinoma	SDCBP2	0	-5.241698	
21105	Papillary serous cystadenocarcinoma	EDN3	0	-5.214098	
21106	Papillary serous cystadenocarcinoma	A1CF	0	-5.152207	
21107	Papillary serous cystadenocarcinoma	ITGA6	0	-5.109066	
21108	Papillary serous cystadenocarcinoma	CXCL6	0	-5.023301	
21109	Papillary serous cystadenocarcinoma	RNF43	0	-5.005845	
21110	Papillary serous cystadenocarcinoma	IGHG1	0	-4.97945	
21111	Papillary serous cystadenocarcinoma	DNASE1L3	0	-4.974289	
21112	Papillary serous cystadenocarcinoma	TMEM54	0	-4.970318	
21113	Papillary serous cystadenocarcinoma	SMAGP	0	-4.963428	
21114	Papillary serous cystadenocarcinoma	SKP2	0	-4.918784	
21115	Papillary serous cystadenocarcinoma	TRPM6	0	-4.906221	
21116	Papillary serous cystadenocarcinoma	GSDMB	0	-4.897099	
21117	Papillary serous cystadenocarcinoma	LOC100240728	0	-4.895336	
21118	Papillary serous cystadenocarcinoma	GAL	0	-4.891549	
21119	Papillary serous cystadenocarcinoma	F2RL1	0	-4.888513	
21120	Papillary serous cystadenocarcinoma	KIF21B	0	-4.880464	
21121	Papillary serous cystadenocarcinoma	GGH	0	-4.872245	
21122	Papillary serous cystadenocarcinoma	CKMT1A	0	-4.812564	
21123	Papillary serous cystadenocarcinoma	PPP1R14D	0	-4.785523	
21124	Papillary serous cystadenocarcinoma	PTGER4	0	-4.736591	
21125	Papillary serous cystadenocarcinoma	RARA	0	-4.704798	
21126	Papillary serous cystadenocarcinoma	GNG4	0	-4.681705	
21127	Papillary serous cystadenocarcinoma	AKR1C2	0	-4.673443	
21128	Papillary serous cystadenocarcinoma	LOC285943	0	-4.569348	
21129	Papillary serous cystadenocarcinoma	PLA2G10	0	-4.565407	
21130	Papillary serous cystadenocarcinoma	HOXB9	0	-4.549691	
21131	Papillary serous cystadenocarcinoma	CLC	0	-4.537395	
21132	Papillary serous cystadenocarcinoma	GREM1	0	-4.536038	
21133	Papillary serous cystadenocarcinoma	TST	0	-4.5328	
21134	Papillary serous cystadenocarcinoma	ACSL6	0	-4.494898	
21135	Papillary serous cystadenocarcinoma	GNLY	0	-4.4667	
21136	Papillary serous cystadenocarcinoma	CES2	0	-4.44194	
21137	Papillary serous cystadenocarcinoma	NEK3	0	-4.431147	
21138	Papillary serous cystadenocarcinoma	KBTBD11	0	-4.41862	
21139	Papillary serous cystadenocarcinoma	ANKRD22	0	-4.400059	
21140	Papillary serous cystadenocarcinoma	CYP2S1	0	-4.396895	
21141	Papillary serous cystadenocarcinoma	SLC44A3	0	-4.368096	
21142	Papillary serous cystadenocarcinoma	TESC	0	-4.334812	
21143	Papillary serous cystadenocarcinoma	F2RL2	0	-4.329708	
21144	Papillary serous cystadenocarcinoma	ALDH1A1	0	-4.328879	
21145	Papillary serous cystadenocarcinoma	F12	0	-4.32808	
21146	Papillary serous cystadenocarcinoma	FLJ22763	0	-4.219927	
21147	Papillary serous cystadenocarcinoma	GPR128	0	-4.206215	
21148	Papillary serous cystadenocarcinoma	JPH1	0	-4.165232	

21149	Papillary serous cystadenocarcinoma	LOC100288781	0	-4.107346	
21150	Papillary serous cystadenocarcinoma	CA12	0	-4.103773	
21151	Papillary serous cystadenocarcinoma	HAVCR2	0	-4.053819	
21152	Papillary serous cystadenocarcinoma	STAMBPL1	0	-4.043653	
21153	Papillary serous cystadenocarcinoma	CMBL	0	-4.037797	
21154	Papillary serous cystadenocarcinoma	TPSG1	0	-4.034993	
21155	Papillary serous cystadenocarcinoma	ATP5O	0	-4.016376	
21156	Papillary serous cystadenocarcinoma	AKR1C1	0	-4.014038	
21157	Papillary serous cystadenocarcinoma	GRTP1	0	-4.012883	
21158	Papillary serous cystadenocarcinoma	IL1B	0	-4.007051	
21159	Papillary serous cystadenocarcinoma	MTMR11	0	-3.99614	
21160	Papillary serous cystadenocarcinoma	CTLA4	0	-3.976031	
21161	Papillary serous cystadenocarcinoma	IL2RG	0	-3.919462	
21162	Papillary serous cystadenocarcinoma	ENPP3	0	-3.772192	
21163	Papillary serous cystadenocarcinoma	GLRX	0	-3.698391	
21164	Papillary serous cystadenocarcinoma	BIRC3	0	-3.685867	
21165	Papillary serous cystadenocarcinoma	LOC653602	0	-3.659397	
21166	Papillary serous cystadenocarcinoma	SLC26A2	0	-3.655878	
21167	Papillary serous cystadenocarcinoma	SLCO2B1	0	-3.635006	
21168	Papillary serous cystadenocarcinoma	PDZD3	0	-3.583991	
21169	Papillary serous cystadenocarcinoma	MYO1D	0	-3.54547	
21170	Papillary serous cystadenocarcinoma	AIFM3	0	-3.537869	
21171	Papillary serous cystadenocarcinoma	PIWIL1	0	-3.508169	
21172	Papillary serous cystadenocarcinoma	SLC13A3	0	-3.499762	
21173	Papillary serous cystadenocarcinoma	NRARP	0	-3.494361	
21174	Papillary serous cystadenocarcinoma	PBLD	0	-3.443629	
21175	Papillary serous cystadenocarcinoma	APOBEC1	0	-3.434815	
21176	Papillary serous cystadenocarcinoma	SEMA4G	0	-3.426409	
21177	Papillary serous cystadenocarcinoma	PIGZ	0	-3.376707	
21178	Papillary serous cystadenocarcinoma	NHSL1	0	-3.355764	
21179	Papillary serous cystadenocarcinoma	C19orf6	0	-3.328838	
21180	Papillary serous cystadenocarcinoma	ME1	0	-3.286947	
21181	Papillary serous cystadenocarcinoma	AHCYL2	0	-3.274456	
21182	Papillary serous cystadenocarcinoma	P2RY14	0	-3.179148	
21183	Papillary serous cystadenocarcinoma	FOXP2	0	-3.149431	
21184	Papillary serous cystadenocarcinoma	FGGY	0	-3.148795	
21185	Papillary serous cystadenocarcinoma	C4orf19	0	-3.147037	
21186	Papillary serous cystadenocarcinoma	FLJ40292	0	-3.123327	
21187	Papillary serous cystadenocarcinoma	IL3RA	0	-3.122227	
21188	Papillary serous cystadenocarcinoma	EPB41L2	0	-3.040553	
21189	Papillary serous cystadenocarcinoma	LOC25845	0	-3.036608	
21190	Papillary serous cystadenocarcinoma	IMPA2	0	-3.011284	
21191	Papillary serous cystadenocarcinoma	ETHE1	0	-2.910183	
21192	Papillary serous cystadenocarcinoma	ASL	0	-2.909534	
21193	Papillary serous cystadenocarcinoma	HR	0	-2.903201	
21194	Papillary serous cystadenocarcinoma	LONRF3	0	-2.893128	
21195	Papillary serous cystadenocarcinoma	AKR7A3	0	-2.838918	
21196	Papillary serous cystadenocarcinoma	GNE	0	-2.835651	
21197	Papillary serous cystadenocarcinoma	EGFR	0	-2.750207	
21198	Papillary serous cystadenocarcinoma	FMO5	0	-2.739365	

21199	Papillary serous cystadenocarcinoma	SOD2	0	-2.716685	
21200	Papillary serous cystadenocarcinoma	UNC5CL	0	-2.704416	
21201	Papillary serous cystadenocarcinoma	ANKRD57	0	-2.646314	
21202	Papillary serous cystadenocarcinoma	LIMA1	0	-2.637912	
21203	Papillary serous cystadenocarcinoma	FRYL	0	-2.610084	
21204	Papillary serous cystadenocarcinoma	KITLG	0	-2.576882	
21205	Papillary serous cystadenocarcinoma	ACOT7	0	-2.537887	
21206	Papillary serous cystadenocarcinoma	MAP2K6	0	-2.483627	
21207	Papillary serous cystadenocarcinoma	LASS6	0	-2.465355	
21208	Papillary serous cystadenocarcinoma	ZAK	0	-2.423467	
21209	Papillary serous cystadenocarcinoma	C13orf23	0	-2.419734	
21210	Papillary serous cystadenocarcinoma	PDSS1	0	-2.416681	
21211	Papillary serous cystadenocarcinoma	PSMG1	0	-2.378961	
21212	Papillary serous cystadenocarcinoma	RFC3	0	-2.309583	
21213	Papillary serous cystadenocarcinoma	MYO15B	0	-2.295333	
21214	Papillary serous cystadenocarcinoma	FAM102B	0	-2.292164	
21215	Papillary serous cystadenocarcinoma	LLGL2	0	-2.185846	
21216	Papillary serous cystadenocarcinoma	PRKAB1	0	-2.01549	
21217	polyps	GAS1	0	-14.683724	
21218	polyps	SPP1	0	-7.837025	
21219	polyps	ITGBL1	0	-7.765214	
21220	polyps	COL10A1	0	-7.354644	
21221	polyps	THBS2	0	-6.932078	
21222	polyps	ASPN	0	-6.83853	
21223	polyps	APOE	0	-6.196602	
21224	polyps	FN1	0	-5.770918	
21225	polyps	MYL9	0	-5.516397	
21226	polyps	MGP	0	-5.433495	
21227	polyps	TNS1	0	-4.91218	
21228	polyps	PTGIS	0	-4.689587	
21229	polyps	BGN	0	-4.533275	
21230	polyps	FXYD6	0	-4.50632	
21231	polyps	C3	0	-4.302186	
21232	polyps	TAGLN	0	-4.205107	
21233	polyps	EHD2	0	-4.170181	
21234	polyps	HOPX	0	-4.126677	
21235	polyps	NNMT	0	-3.947345	
21236	polyps	TPM2	0	-3.946697	
21237	polyps	RBMS1	0	-3.913365	
21238	polyps	CYP1B1	0	-3.895219	
21239	polyps	WWC2	0	-3.875849	
21240	polyps	ZCCHC24	0	-3.8594	
21241	polyps	FLNA	0	-3.857171	
21242	polyps	EPHA3	0	-3.740391	
21243	polyps	PDLIM3	0	-3.735855	
21244	polyps	AKAP12	0	-3.682323	
21245	polyps	AEBP1	0	-3.641108	
21246	polyps	MEIS1	0	-3.628587	
21247	polyps	RDX	0	-3.584285	
21248	polyps	EDNRA	0	-3.569713	

21249	polyps	CALD1	0	-3.55865	
21250	polyps	FBLN2	0	-3.532621	
21251	polyps	GPNMB	0	-3.49659	
21252	polyps	TRPC1	0	-3.402634	
21253	polyps	FERMT2	0	-3.377365	
21254	polyps	TIMP3	0	-3.311889	
21255	polyps	COX7A1	0	-3.31043	
21256	polyps	NTM	0	-3.265553	
21257	polyps	WWTR1	0	-3.201305	
21258	polyps	DCN	0	-3.198334	
21259	polyps	RAB31	0	-3.194913	
21260	polyps	VCAN	0	-3.172125	
21261	polyps	EGR2	0	-3.133039	
21262	polyps	PTRF	0	-3.122768	
21263	polyps	COL6A2	0	-3.10088	
21264	polyps	SPARC	0	-3.076194	
21265	polyps	DDR2	0	-3.058948	
21266	polyps	PDLIM7	0	-3.056306	
21267	polyps	COL18A1	0	-3.050161	
21268	polyps	SERPINF1	0	-2.93389	
21269	polyps	NRP1	0	-2.931343	
21270	polyps	WASF3	0	-2.925762	
21271	polyps	LOX	0	-2.872032	
21272	polyps	TUBB6	0	-2.795967	
21273	polyps	QKI	0	-2.78205	
21274	polyps	LTBP3	0	-2.767249	
21275	polyps	PRELP	0	-2.741759	
21276	polyps	HTRA1	0	-2.725609	
21277	polyps	MAFB	0	-2.717956	
21278	polyps	C1S	0	-2.701801	
21279	polyps	SDC2	0	-2.684277	
21280	polyps	DPYD	0	-2.67381	
21281	polyps	NR3C1	0	-2.642954	
21282	polyps	MYH10	0	-2.624618	
21283	polyps	GPR124	0	-2.607536	
21284	polyps	EFEMP1	0	-2.527422	
21285	polyps	RUNX1T1	0	-2.527036	
21286	polyps	ZEB2	0	-2.508715	
21287	polyps	NOTCH3	0	-2.50001	
21288	polyps	DZIP1	0	-2.453138	
21289	polyps	KIAA1462	0	-2.44757	
21290	polyps	MAP1B	0	-2.434164	
21291	polyps	AKAP2	0	-2.428331	
21292	polyps	RAI14	0	-2.419962	
21293	polyps	OLFML2B	0	-2.398179	
21294	polyps	ZEB1	0	-2.392731	
21295	polyps	SYNE1	0	-2.383231	
21296	polyps	LHFP	0	-2.378204	
21297	polyps	FGFR1	0	-2.375	
21298	polyps	NOX4	0	-2.361673	

21299	polyps	ACTA2	0	-2.357686	
21300	polyps	NPTXR	0	-2.340919	
21301	polyps	ZFPM2	0	-2.312991	
21302	polyps	PXDN	0	-2.282177	
21303	polyps	CBX6	0	-2.275591	
21304	polyps	JAM3	0	-2.261724	
21305	polyps	MEG3	0	-2.257411	
21306	polyps	COL4A2	0	-2.255471	
21307	polyps	RASSF4	0	-2.238971	
21308	polyps	NXN	0	-2.220724	
21309	polyps	GUCY1B3	0	-2.217627	
21310	polyps	FADS3	0	-2.190039	
21311	polyps	MSN	0	-2.189906	
21312	polyps	ECM2	0	-2.18759	
21313	polyps	MAF	0	-2.164818	
21314	polyps	NCF2	0	-2.163869	
21315	polyps	ITGAM	0	-2.163429	
21316	polyps	PRNP	0	-2.162198	
21317	polyps	CDK14	0	-2.16181	
21318	polyps	IQCJ-SCHIP1	0	-2.159421	
21319	polyps	PBX3	0	-2.148824	
21320	polyps	AKT3	0	-2.147263	
21321	polyps	PDLIM2	0	-2.144072	
21322	polyps	SIRPA	0	-2.14184	
21323	polyps	MPDZ	0	-2.127208	
21324	polyps	FSTL3	0	-2.123976	
21325	polyps	BCL6	0	-2.109224	
21326	polyps	TCF4	0	-2.095243	
21327	polyps	HSPG2	0	-2.093501	
21328	polyps	CEP170	0	-2.081822	
21329	polyps	ELTD1	0	-2.080908	
21330	polyps	GUCY1A3	0	-2.080711	
21331	polyps	GPC1	0	-2.047464	
21332	polyps	FLRT2	0	-2.034747	
21333	polyps	PKD2	0	-2.026586	
21334	polyps	CTSL1	0	-2.01876	
21335	polyps	CHST15	0	-2.0099	
21336	Primary Cell Culture	MAP7	0.000652	-7.749575	
21337	Primary Cell Culture	MAPK13	0.000093	-5.139744	
21338	Primary Cell Culture	ROD1	0.000155	-4.802888	
21339	Primary Cell Culture	RAB3IP	0.000218	-4.603243	
21340	Primary Cell Culture	WWP1	0.000529	-3.9455	
21341	Primary Cell Culture	RNASET2	0.000088	-3.751019	
21342	Primary Cell Culture	PPFIBP2	0.000541	-3.362162	
21343	Primary Cell Culture	BAZ2B	0.000375	-3.040968	
21344	Primary Cell Culture	CYP2R1	0.000348	-2.97435	
21345	Primary Cell Culture	PHIP	0.00018	-2.916824	
21346	Primary Cell Culture	OCIAD2	0.000516	-2.862596	
21347	Primary Cell Culture	SYK	0.000144	-2.815386	
21348	Primary Cell Culture	KIF21A	0.000493	-2.764031	

21349	Primary Cell Culture	LOC387921	0.000603	-2.703054	
21350	Primary Cell Culture	SLC25A5	0.000533	-2.551182	
21351	Primary Cell Culture	MRPS35	0.000166	-2.52774	
21352	Primary Cell Culture	PPIG	0.000425	-2.505525	
21353	Primary Cell Culture	GUSB	0.00007	-2.451689	
21354	Primary Cell Culture	ANXA4	0.000425	-2.440149	
21355	Primary Cell Culture	ZNF165	0.000062	-2.402117	
21356	Primary Cell Culture	TMEM41B	0.000615	-2.399405	
21357	Primary Cell Culture	ZNF721	0.000664	-2.377136	
21358	Primary Cell Culture	ZNF800	0.000597	-2.347068	
21359	Primary Cell Culture	C4ORF16	0.000093	-2.332935	
21360	Primary Cell Culture	FLJ34969	0.000597	-2.282324	
21361	Primary Cell Culture	CCDC76	0.000599	-2.225771	
21362	Primary Cell Culture	TARDBP	0.000574	-2.221869	
21363	Primary Cell Culture	RNF141	0.000516	-2.206677	
21364	Primary Cell Culture	C11ORF71	0.000545	-2.102567	
21365	Primary Cell Culture	RPS24	0.000286	-2.072647	
21366	Primary Cell Culture	DGKD	0.000219	-2.026221	
21367	Primary Cell Culture	RPL5	0.000282	-2.004885	
21368	Primary Cell Culture	RGS10	0.000375	2.006736	
21369	Primary Cell Culture	CCDC127	0.000514	2.023144	
21370	Primary Cell Culture	RBMS2	0.000284	2.071696	
21371	Primary Cell Culture	TNFAIP1	0.000398	2.089194	
21372	Primary Cell Culture	GAPDH	0.000425	2.158625	
21373	Primary Cell Culture	ADAMTSL5	0.000486	2.27493	
21374	Primary Cell Culture	NPR2	0.000399	2.282587	
21375	Primary Cell Culture	SDF4	0.000665	2.289924	
21376	Primary Cell Culture	MST150	0.000125	2.311495	
21377	Primary Cell Culture	SEC22C	0.000369	2.345013	
21378	Primary Cell Culture	NTAN1	0.00061	2.400488	
21379	Primary Cell Culture	RIC8A	0.000436	2.464435	
21380	Primary Cell Culture	ZBTB4	0.000201	2.481596	
21381	Primary Cell Culture	SLC35E1	0.000436	2.585884	
21382	Primary Cell Culture	PNMA1	0.000603	2.646874	
21383	Primary Cell Culture	ABI2	0.000191	2.657783	
21384	Primary Cell Culture	PVRL2	0.000187	2.739545	
21385	Primary Cell Culture	M-RIP	0.000292	2.875153	
21386	Primary Cell Culture	MPV17	0.000334	2.87788	
21387	Primary Cell Culture	SPARC	0.000665	2.977244	
21388	Primary Cell Culture	EXT1	0.000425	3.142807	
21389	Primary Cell Culture	DYRK3	0.000062	3.199536	
21390	Primary Cell Culture	TCEAL3	0.000602	3.283354	
21391	Primary Cell Culture	VKORC1	0.000031	3.288607	
21392	Primary Cell Culture	TPM1	0.000436	3.396558	
21393	Primary Cell Culture	KIAA1754	0.000235	3.642638	
21394	Primary Cell Culture	HSPB2	0.000603	3.783672	
21395	Primary Cell Culture	FKSG30	0.000436	3.868274	
21396	Primary Cell Culture	MYADM	0.000189	3.982227	
21397	Primary Cell Culture	DFNA5	0.000086	4.008277	
21398	Primary Cell Culture	PELO	0.000599	4.297751	

21399	Primary Cell Culture	RAB6IP1	0.000219	4.356722	
21400	Primary Cell Culture	NINJ1	0.00058	4.385068	
21401	Primary Cell Culture	GABARAPL1	0.000031	4.474687	
21402	Primary Cell Culture	ACTN1	0.000001	4.790828	
21403	Primary Cell Culture	MSN	0.000155	4.827876	
21404	Primary Cell Culture	VIM	0.000441	4.942041	
21405	Primary Cell Culture	ACTA2	0.000155	5.024653	
21406	Primary Cell Culture	CYR61	0.000093	5.299898	
21407	Primary Cell Culture	DEGS1	0.000035	5.682363	
21408	Primary Cell Culture	LGALS1	0.000071	5.933415	
21409	Primary Cell Culture	NGFRAP1	0.000501	6.226585	
21410	Primary Cell Culture	COL1A2	0.000375	10.191927	
21411	Primary Cell Culture	FAM20C	0.000468	10.313947	
21412	Primary Cell Culture	DKK3	0.000597	10.416492	
21413	Primary Cell Culture	VASN	0.000441	12.264142	
21414	Primary Neoplasm	LOC646014	0.000324	-520.819672	
21415	Primary Neoplasm	CA1	0	-179.066282	
21416	Primary Neoplasm	GUCA2A	0	-60.339935	
21417	Primary Neoplasm	SLC26A3	0	-49.393405	
21418	Primary Neoplasm	AAAS	0	-40.694998	
21419	Primary Neoplasm	EYA2	0	-31.798124	
21420	Primary Neoplasm	LRRN5	0	-26.292493	
21421	Primary Neoplasm	LOC388886	0	-22.29625	
21422	Primary Neoplasm	APG4B	0	-20.338664	
21423	Primary Neoplasm	HPGD	0.000319	-20.107995	
21424	Primary Neoplasm	TNFRSF10B	0	-20.038294	
21425	Primary Neoplasm	CEACAM7	0	-18.883814	
21426	Primary Neoplasm	FLJ21511	0	-18.632775	
21427	Primary Neoplasm	BTNL2	0	-17.91367	
21428	Primary Neoplasm	PKIB	0	-15.838691	
21429	Primary Neoplasm	HPRP8BP	0	-12.675516	
21430	Primary Neoplasm	C17orf27	0	-12.373756	
21431	Primary Neoplasm	MGC12966	0	-12.008485	
21432	Primary Neoplasm	KRT3	0	-11.681813	
21433	Primary Neoplasm	CA12	0	-11.253557	
21434	Primary Neoplasm	LOC56920	0	-10.21857	
21435	Primary Neoplasm	TNFRSF25	0	-9.667726	
21436	Primary Neoplasm	MRGX3	0	-9.507378	
21437	Primary Neoplasm	GGTLA1	0	-9.421108	
21438	Primary Neoplasm	UGT1A10	0	-8.892093	
21439	Primary Neoplasm	CFLAR	0	-8.826995	
21440	Primary Neoplasm	USP49	0	-8.785469	
21441	Primary Neoplasm	AQP3	0	-8.176899	
21442	Primary Neoplasm	VPS18	0	-8.11852	
21443	Primary Neoplasm	UPK3B	0	-8.112432	
21444	Primary Neoplasm	EZR	0.000093	-7.689489	
21445	Primary Neoplasm	ABCD1	0	-7.582764	
21446	Primary Neoplasm	PLCL3	0	-7.529323	
21447	Primary Neoplasm	CNKSR1	0	-7.218778	
21448	Primary Neoplasm	UAP1L1	0	-7.166103	

21449	Primary Neoplasm	OTOP2	0	-7.094136	
21450	Primary Neoplasm	DTX2	0	-6.994055	
21451	Primary Neoplasm	MMRN1	0.000018	-6.936042	
21452	Primary Neoplasm	ADH1C	0	-6.643184	
21453	Primary Neoplasm	PDCD1	0	-6.466146	
21454	Primary Neoplasm	NGFR	0	-6.422489	
21455	Primary Neoplasm	ARMC7	0	-6.268626	
21456	Primary Neoplasm	ADH4	0	-5.622253	
21457	Primary Neoplasm	GPR155	0	-5.270132	
21458	Primary Neoplasm	GPR126	0	-5.042823	
21459	Primary Neoplasm	FLJ43855	0	-4.95643	
21460	Primary Neoplasm	LOC149134	0.000056	-4.789165	
21461	Primary Neoplasm	TTR	0.000004	-4.645625	
21462	Primary Neoplasm	CFHR1	0	-4.520135	
21463	Primary Neoplasm	RIPK3	0	-4.484363	
21464	Primary Neoplasm	TF	0.000015	-4.271676	
21465	Primary Neoplasm	ACAT1	0	-4.24978	
21466	Primary Neoplasm	UGT2B4	0	-4.116567	
21467	Primary Neoplasm	JMJD2C	0.000278	-4.077496	
21468	Primary Neoplasm	HERPUD1	0	-4.065191	
21469	Primary Neoplasm	hCG_1776259	0.000229	-4.051954	
21470	Primary Neoplasm	PDK4	0.000289	-4.038259	
21471	Primary Neoplasm	UGT2B7	0	-3.964991	
21472	Primary Neoplasm	SLC4A4	0	-3.958086	
21473	Primary Neoplasm	ISYNA1	0	-3.926594	
21474	Primary Neoplasm	PCK1	0	-3.913386	
21475	Primary Neoplasm	CXCL12	0	-3.91019	
21476	Primary Neoplasm	GLUD1	0.000349	-3.895446	
21477	Primary Neoplasm	CFH	0	-3.78256	
21478	Primary Neoplasm	C20orf98	0	-3.688656	
21479	Primary Neoplasm	TEP1	0.000306	-3.688525	
21480	Primary Neoplasm	GHSR	0	-3.655671	
21481	Primary Neoplasm	PIGQ	0	-3.559043	
21482	Primary Neoplasm	GC	0.000002	-3.48362	
21483	Primary Neoplasm	TBRG1	0.0001	-3.460295	
21484	Primary Neoplasm	PAX1	0	-3.43594	
21485	Primary Neoplasm	MATN1	0	-3.416291	
21486	Primary Neoplasm	MCART1	0	-3.412675	
21487	Primary Neoplasm	APOA2	0.000002	-3.396929	
21488	Primary Neoplasm	C7	0	-3.336013	
21489	Primary Neoplasm	SLC7A2	0.000037	-3.332803	
21490	Primary Neoplasm	PPY	0	-3.292544	
21491	Primary Neoplasm	XLKD1	0	-3.279393	
21492	Primary Neoplasm	FAM14A	0	-3.217157	
21493	Primary Neoplasm	FPRL1	0.000011	-3.169335	
21494	Primary Neoplasm	CYP3A4	0.000003	-3.150967	
21495	Primary Neoplasm	EME2	0	-3.130618	
21496	Primary Neoplasm	HES5	0	-3.094644	
21497	Primary Neoplasm	HMGCS2	0.000001	-3.080903	
21498	Primary Neoplasm	EPS15	0.000149	-3.080798	

21499	Primary Neoplasm	MGC33486	0	-3.061939	
21500	Primary Neoplasm	PBLD	0.000273	-3.038754	
21501	Primary Neoplasm	CYP2C8	0.000023	-2.984006	
21502	Primary Neoplasm	OCSP	0	-2.979459	
21503	Primary Neoplasm	PAH	0.000012	-2.971188	
21504	Primary Neoplasm	DEFB1	0.000061	-2.97051	
21505	Primary Neoplasm	MOBP	0	-2.910332	
21506	Primary Neoplasm	UGT1A8	0	-2.880971	
21507	Primary Neoplasm	FMO3	0.000103	-2.866698	
21508	Primary Neoplasm	CDKN2B	0.000179	-2.863855	
21509	Primary Neoplasm	MT1G	0.000021	-2.861547	
21510	Primary Neoplasm	ZNF608	0	-2.844654	
21511	Primary Neoplasm	EPS8	0	-2.799162	
21512	Primary Neoplasm	KIAA0564	0	-2.784731	
21513	Primary Neoplasm	LOC440838	0.000153	-2.754453	
21514	Primary Neoplasm	CLYBL	0.00019	-2.733223	
21515	Primary Neoplasm	HIGD2A	0.000015	-2.723354	
21516	Primary Neoplasm	SCNN1B	0	-2.70524	
21517	Primary Neoplasm	AGXT	0	-2.700679	
21518	Primary Neoplasm	METTL7A	0	-2.682637	
21519	Primary Neoplasm	MAOB	0	-2.680449	
21520	Primary Neoplasm	SLC6A3	0	-2.645483	
21521	Primary Neoplasm	TMEM1	0	-2.531473	
21522	Primary Neoplasm	FLJ10945	0	-2.529667	
21523	Primary Neoplasm	ADH6	0	-2.494864	
21524	Primary Neoplasm	SERPINA6	0.000004	-2.474711	
21525	Primary Neoplasm	KLKB1	0.000008	-2.428999	
21526	Primary Neoplasm	ANXA11	0.000009	-2.425022	
21527	Primary Neoplasm	PXMP2	0	-2.420483	
21528	Primary Neoplasm	TGM4	0.000001	-2.393012	
21529	Primary Neoplasm	SERPINA3	0.000286	-2.383856	
21530	Primary Neoplasm	C6orf111	0	-2.38063	
21531	Primary Neoplasm	KRTHA5	0	-2.365889	
21532	Primary Neoplasm	NR3C2	0	-2.356602	
21533	Primary Neoplasm	ABCA6	0	-2.307646	
21534	Primary Neoplasm	ETFA	0	-2.241444	
21535	Primary Neoplasm	C1orf115	0	-2.236629	
21536	Primary Neoplasm	MGC11134	0	-2.218774	
21537	Primary Neoplasm	EIF2B1	0	-2.173763	
21538	Primary Neoplasm	ANPEP	0.000387	-2.16877	
21539	Primary Neoplasm	CASQ2	0.000094	-2.147198	
21540	Primary Neoplasm	CDH19	0.000056	-2.136546	
21541	Primary Neoplasm	ADD1	0.000146	-2.122039	
21542	Primary Neoplasm	GPX3	0	-2.082077	
21543	Primary Neoplasm	GDPD2	0.000372	-2.052014	
21544	Primary Neoplasm	CYP2W1	0	-2.046129	
21545	Primary Neoplasm	LOC653483	0.00001	-2.006975	
21546	Primary Neoplasm	TSPAN7	0	-2.004631	
21547	Primary Neoplasm	PRR16	0	2.000888	
21548	Primary Neoplasm	CDT1	0.000228	2.001072	

21549	Primary Neoplasm	COL5A1	0	2.00545	
21550	Primary Neoplasm	ANTXR1	0	2.033572	
21551	Primary Neoplasm	LOXL2	0	2.033579	
21552	Primary Neoplasm	SPOCK1	0	2.049475	
21553	Primary Neoplasm	DPH2	0.000054	2.059234	
21554	Primary Neoplasm	FUNDC1	0	2.065707	
21555	Primary Neoplasm	TEX10	0.000091	2.089342	
21556	Primary Neoplasm	PLAU	0	2.089529	
21557	Primary Neoplasm	THY1	0	2.09267	
21558	Primary Neoplasm	SND1	0	2.118394	
21559	Primary Neoplasm	COL3A1	0	2.121972	
21560	Primary Neoplasm	MTHFD1	0.000106	2.122304	
21561	Primary Neoplasm	COL7A1	0	2.12324	
21562	Primary Neoplasm	MDS025	0	2.134613	
21563	Primary Neoplasm	EGFL6	0.000026	2.144819	
21564	Primary Neoplasm	MXRA5	0	2.148615	
21565	Primary Neoplasm	IFITM1	0.000001	2.159871	
21566	Primary Neoplasm	CKAP5	0.000231	2.168001	
21567	Primary Neoplasm	HSA272196	0	2.188173	
21568	Primary Neoplasm	AURKA	0.000122	2.211307	
21569	Primary Neoplasm	MAD2L1	0	2.225257	
21570	Primary Neoplasm	TRIM21	0.000005	2.260939	
21571	Primary Neoplasm	CEACAM5	0.000104	2.273474	
21572	Primary Neoplasm	RBBP5	0.000265	2.277786	
21573	Primary Neoplasm	IGHMBP2	0	2.281069	
21574	Primary Neoplasm	ADAM12	0	2.314582	
21575	Primary Neoplasm	DIO2	0	2.327507	
21576	Primary Neoplasm	RACGAP1	0.000285	2.352492	
21577	Primary Neoplasm	CDH11	0	2.363087	
21578	Primary Neoplasm	WNT5A	0	2.363404	
21579	Primary Neoplasm	COL5A2	0	2.3652	
21580	Primary Neoplasm	LOC285636	0.00022	2.367596	
21581	Primary Neoplasm	MMP14	0	2.369605	
21582	Primary Neoplasm	GART	0.000004	2.378751	
21583	Primary Neoplasm	MGC3234	0	2.394413	
21584	Primary Neoplasm	ADCK2	0	2.402143	
21585	Primary Neoplasm	BARX1	0.000009	2.406034	
21586	Primary Neoplasm	LOC729680	0.00039	2.414852	
21587	Primary Neoplasm	IL8	0	2.433447	
21588	Primary Neoplasm	TWIST1	0	2.436027	
21589	Primary Neoplasm	CCL11	0.000018	2.447059	
21590	Primary Neoplasm	TMEM158	0	2.496317	
21591	Primary Neoplasm	COL1A1	0	2.505787	
21592	Primary Neoplasm	GRB10	0	2.553492	
21593	Primary Neoplasm	COL2A1	0	2.555688	
21594	Primary Neoplasm	TRUB1	0.000049	2.560965	
21595	Primary Neoplasm	C20orf4	0	2.618354	
21596	Primary Neoplasm	CALU	0	2.663052	
21597	Primary Neoplasm	C3orf31	0.000179	2.665299	
21598	Primary Neoplasm	PHF16	0	2.742254	

21599	Primary Neoplasm	LACTB2	0	2.797572	
21600	Primary Neoplasm	MFAP2	0	2.834898	
21601	Primary Neoplasm	FAP	0	2.874475	
21602	Primary Neoplasm	CTPS	0.000415	3.06325	
21603	Primary Neoplasm	CDCA8	0	3.191028	
21604	Primary Neoplasm	GREM1	0	3.195002	
21605	Primary Neoplasm	CCL20	0.000014	3.200013	
21606	Primary Neoplasm	IFIT2	0	3.267003	
21607	Primary Neoplasm	MMP11	0	3.315333	
21608	Primary Neoplasm	RFT1	0	3.327087	
21609	Primary Neoplasm	DDA3	0	3.390333	
21610	Primary Neoplasm	MMP3	0	3.411777	
21611	Primary Neoplasm	CXCL1	0	3.435957	
21612	Primary Neoplasm	PLOD3	0	3.655106	
21613	Primary Neoplasm	pp9099	0	3.70692	
21614	Primary Neoplasm	CXCL3	0	3.726125	
21615	Primary Neoplasm	COL10A1	0	3.736957	
21616	Primary Neoplasm	PSAT1	0.000019	3.737574	
21617	Primary Neoplasm	TM4SF9	0	3.737685	
21618	Primary Neoplasm	C20orf20	0	4.054074	
21619	Primary Neoplasm	C5orf22	0.000215	4.134619	
21620	Primary Neoplasm	BM039	0	4.454331	
21621	Primary Neoplasm	CDC25B	0	4.542251	
21622	Primary Neoplasm	INPP5D	0	4.562732	
21623	Primary Neoplasm	IFITM3	0	4.62915	
21624	Primary Neoplasm	PI3	0	4.702185	
21625	Primary Neoplasm	SHMT2	0	4.839783	
21626	Primary Neoplasm	MRPL39	0.000257	5.00501	
21627	Primary Neoplasm	VGF	0	5.557272	
21628	Primary Neoplasm	T1A-2	0	6.643422	
21629	Primary Neoplasm	LSM11	0	6.665394	
21630	Primary Neoplasm	DUSP4	0	7.233757	
21631	Primary Neoplasm	DUSP14	0	7.807937	
21632	Primary Neoplasm	ANXA9	0	7.815307	
21633	Primary Neoplasm	AZGP1	0	9.954115	
21634	Primary Neoplasm	C17orf39	0	10.213209	
21635	Primary Neoplasm	VNN1	0	10.364018	
21636	Primary Neoplasm	ESM1	0	14.29859	
21637	Primary Neoplasm	CTHRC1	0	19.152669	
21638	Primary Neoplasm	NAT12	0.000307	32.336224	
21639	Primary Neoplasm	SPP1	0	41.897472	
21640	Primary Neoplasm	DPEP1	0	45.452708	
21641	Primary Neoplasm	MMP7	0	128.551767	
21642	Primary Neoplasm	NUDT5	0.000037	376.844444	
21643	Prior Therapy	CLCA4	0	-9.827755	
21644	Prior Therapy	CEACAM7	0	-7.684138	
21645	Prior Therapy	MS4A12	0	-6.295387	
21646	Prior Therapy	ZG16	0	-6.238001	
21647	Prior Therapy	AQP8	0.000003	-5.147994	
21648	Prior Therapy	SLC26A3	0	-4.508956	

21649	Prior Therapy	CA4	0	-4.347833	
21650	Prior Therapy	GUCA2A	0	-3.472724	
21651	Prior Therapy	DUOX2	0	-3.28592	
21652	Prior Therapy	LOC646627	0	-3.122643	
21653	Prior Therapy	GUCA2B	0.000007	-3.075463	
21654	Prior Therapy	PI3	0	-2.996499	
21655	Prior Therapy	DHRS9	0	-2.738318	
21656	Prior Therapy	PLAC8	0	-2.731363	
21657	Prior Therapy	CA2	0.000001	-2.619544	
21658	Prior Therapy	CAPN9	0	-2.61569	
21659	Prior Therapy	HSD11B2	0	-2.405776	
21660	Prior Therapy	DUOXA2	0.000005	-2.396862	
21661	Prior Therapy	GPR120	0.000014	-2.389006	
21662	Prior Therapy	FAM3D	0	-2.335327	
21663	Prior Therapy	C10orf99	0	-2.323356	
21664	Prior Therapy	FAM55A	0	-2.290131	
21665	Prior Therapy	SELENBP1	0	-2.263722	
21666	Prior Therapy	KRT20	0	-2.25864	
21667	Prior Therapy	POF1B	0	-2.250425	
21668	Prior Therapy	C6orf105	0	-2.248023	
21669	Prior Therapy	LRRC19	0	-2.234078	
21670	Prior Therapy	C15orf48	0	-2.223382	
21671	Prior Therapy	TSPAN1	0.000004	-2.204885	
21672	Prior Therapy	BEST2	0	-2.17273	
21673	Prior Therapy	SLC26A2	0.000009	-2.153292	
21674	Prior Therapy	GPA33	0	-2.150576	
21675	Prior Therapy	FUT6	0	-2.144658	
21676	Prior Therapy	HHLA2	0	-2.107685	
21677	Prior Therapy	AXIN2	0	-2.10624	
21678	Prior Therapy	GCNT3	0.000012	-2.071163	
21679	Prior Therapy	CCL20	0.000003	-2.067934	
21680	Prior Therapy	ITM2C	0	-2.064866	
21681	Prior Therapy	TRPA1	0.000013	-2.050908	
21682	Prior Therapy	NOX1	0.000005	-2.042676	
21683	Prior Therapy	PKP2	0	-2.036926	
21684	Prior Therapy	NOS2	0	-2.033758	
21685	Prior Therapy	TMEM54	0	-2.029737	
21686	Prior Therapy	ACE2	0.000004	-2.026441	
21687	Prior Therapy	PADI2	0.000002	-2.021056	
21688	Prior Therapy	UGT8	0	-2.01583	
21689	Prior Therapy	FXYD3	0	-2.004031	
21690	Prior Therapy	EDN3	0	-2.002887	
21691	Prostate carcinoma	SMCY	0.040675	6.353116	
21692	Prostate carcinoma	RPS4Y2	0.039175	13.187671	
21693	Prostate carcinoma	RPS4Y1	0.039175	56.699911	
21694	Protein p53	PHLDB2	0	-79.357183	
21695	Protein p53	PROM1	0.000007	-68.495897	
21696	Protein p53	TP53	0.000023	-47.096593	
21697	Protein p53	GDF15	0.00041	-42.95538	
21698	Protein p53	NRIP1	0.000057	-38.869276	

21699	Protein p53	SCARA3	0.000003	-25.93522	
21700	Protein p53	MSI1	0.000005	-24.721362	
21701	Protein p53	FAM171B	0.000008	-22.644665	
21702	Protein p53	RAB38	0	-21.45702	
21703	Protein p53	S100A4	0	-20.156038	
21704	Protein p53	RBPMS2	0.000266	-16.968109	
21705	Protein p53	RGS2	0.000003	-16.540364	
21706	Protein p53	EML1	0	-16.504361	
21707	Protein p53	MKX	0.000031	-16.252809	
21708	Protein p53	KISS1R	0.000054	-16.026044	
21709	Protein p53	CYBRD1	0.000011	-15.955871	
21710	Protein p53	MARCKS	0.000001	-15.877995	
21711	Protein p53	SMARCD3	0	-15.594032	
21712	Protein p53	HLTF	0.000029	-13.771127	
21713	Protein p53	SLC16A14	0.000003	-13.373653	
21714	Protein p53	FBXO44	0.000015	-12.898193	
21715	Protein p53	MCOLN3	0.000004	-12.019836	
21716	Protein p53	DSE	0.000021	-11.580355	
21717	Protein p53	RNF217	0.00002	-11.55933	
21718	Protein p53	ADAP2	0	-11.396105	
21719	Protein p53	ZNF618	0.000012	-11.312275	
21720	Protein p53	PTPN13	0.000013	-11.195711	
21721	Protein p53	GPX8	0.000005	-10.645534	
21722	Protein p53	CCNDBP1	0.000226	-10.62795	
21723	Protein p53	KLK7	0	-10.412573	
21724	Protein p53	FAM7A3	0.000024	-10.252432	
21725	Protein p53	MARK1	0	-10.217503	
21726	Protein p53	TLE2	0	-8.702231	
21727	Protein p53	TTC7B	0.000076	-8.657179	
21728	Protein p53	LOC440302	0.000016	-8.285117	
21729	Protein p53	LOC648795	0.00003	-8.118847	
21730	Protein p53	PKIG	0.000094	-8.099702	
21731	Protein p53	TTLL7	0.00022	-7.996952	
21732	Protein p53	FLJ33996	0.000136	-7.914756	
21733	Protein p53	B3GALNT1	0.000024	-7.89949	
21734	Protein p53	GPR98	0.000043	-7.791575	
21735	Protein p53	RECK	0	-7.333833	
21736	Protein p53	LOC51233	0	-7.282324	
21737	Protein p53	ANXA6	0.000111	-7.199033	
21738	Protein p53	CHRFAM7A	0.000021	-6.761782	
21739	Protein p53	PRKAR2B	0.000163	-6.727933	
21740	Protein p53	FAM111A	0.00023	-6.605117	
21741	Protein p53	KIAA1920	0.000002	-6.572179	
21742	Protein p53	PSMG1	0.000091	-6.486989	
21743	Protein p53	TMOD2	0.000008	-6.46977	
21744	Protein p53	RASSF8	0.000153	-6.447505	
21745	Protein p53	DIP2C	0	-6.344517	
21746	Protein p53	SCN8A	0.000041	-6.225373	
21747	Protein p53	PTPRU	0.000007	-6.177564	
21748	Protein p53	UTRN	0.000091	-6.151177	

21749	Protein p53	MID2	0.000003	-6.122492	
21750	Protein p53	COL11A2	0.000016	-6.081678	
21751	Protein p53	CFL2	0	-6.04272	
21752	Protein p53	FAM7A1	0.000016	-5.890325	
21753	Protein p53	LRRC8C	0.000016	-5.817699	
21754	Protein p53	PARD6G	0.000031	-5.80562	
21755	Protein p53	POLN	0.000006	-5.62979	
21756	Protein p53	C8orf47	0.000064	-5.581816	
21757	Protein p53	ENC1	0.000105	-5.491418	
21758	Protein p53	DDB2	0.002509	-5.489356	
21759	Protein p53	DENND2C	0.000007	-5.481683	
21760	Protein p53	C6orf204	0.00008	-5.372437	
21761	Protein p53	SLC16A9	0.000163	-5.333557	
21762	Protein p53	BBS5	0.000036	-5.299647	
21763	Protein p53	CCDC46	0.00003	-5.297978	
21764	Protein p53	FLJ43276	0.000069	-5.270924	
21765	Protein p53	KCNIP2	0.000285	-5.252706	
21766	Protein p53	SCHIP1	0.000259	-5.146005	
21767	Protein p53	STARD9	0.000098	-4.914114	
21768	Protein p53	SERPINB9	0.000001	-4.911115	
21769	Protein p53	SLC36A4	0.000253	-4.881939	
21770	Protein p53	CDGAP	0.000124	-4.857344	
21771	Protein p53	KLHL5	0.000051	-4.830654	
21772	Protein p53	DRAM	0.000021	-4.800351	
21773	Protein p53	HNRNPH3	0.000111	-4.787262	
21774	Protein p53	TTC18	0.000014	-4.779103	
21775	Protein p53	DBP	0.000005	-4.750197	
21776	Protein p53	SIX4	0.000044	-4.74543	
21777	Protein p53	DRAM1	0.000091	-4.728488	
21778	Protein p53	NUAK1	0	-4.697471	
21779	Protein p53	SPIRE1	0.000098	-4.683553	
21780	Protein p53	GPR161	0.000053	-4.663312	
21781	Protein p53	CTNNB1	0.000071	-4.646194	
21782	Protein p53	EMID1	0.000209	-4.622975	
21783	Protein p53	ZNF204	0.000048	-4.619756	
21784	Protein p53	MRC2	0	-4.548474	
21785	Protein p53	SNX10	0.000152	-4.466907	
21786	Protein p53	MPP2	0.000017	-4.450212	
21787	Protein p53	AMZ2	0.000023	-4.439814	
21788	Protein p53	ADRBK2	0.000104	-4.356837	
21789	Protein p53	RBM24	0.000022	-4.322164	
21790	Protein p53	MOSC2	0.000098	-4.225067	
21791	Protein p53	N4BP2L2	0.000028	-4.190006	
21792	Protein p53	PAQR7	0.000274	-4.164603	
21793	Protein p53	STOX2	0.000011	-4.16124	
21794	Protein p53	TCF7L1	0.000005	-4.135303	
21795	Protein p53	RASGRP1	0.000036	-4.104226	
21796	Protein p53	NHEDC2	0.000076	-4.100033	
21797	Protein p53	BEND7	0.000105	-4.084679	
21798	Protein p53	HRK	0.000005	-4.082063	

21799	Protein p53	IQCJ-SCHIP1	0.000032	-4.075156	
21800	Protein p53	CYR61	0.00002	-4.046511	
21801	Protein p53	MXRA7	0.000187	-3.993349	
21802	Protein p53	MICB	0.000177	-3.966082	
21803	Protein p53	GLI2	0.000058	-3.945516	
21804	Protein p53	RNF125	0.000073	-3.89468	
21805	Protein p53	KLC3	0.00014	-3.88916	
21806	Protein p53	NMT2	0.000124	-3.841508	
21807	Protein p53	CCDC136	0.000043	-3.823805	
21808	Protein p53	B3GNT4	0.000147	-3.817794	
21809	Protein p53	PPP2R3A	0.000143	-3.81687	
21810	Protein p53	ZNF280B	0.000144	-3.80754	
21811	Protein p53	TSPAN4	0.000089	-3.774971	
21812	Protein p53	MT1E	0.000058	-3.774363	
21813	Protein p53	RAB6B	0.000009	-3.773154	
21814	Protein p53	HECTD2	0.000301	-3.764647	
21815	Protein p53	HMGN5	0.000806	-3.741627	
21816	Protein p53	LOC286440	0.000021	-3.725239	
21817	Protein p53	LOC201229	0.000041	-3.693565	
21818	Protein p53	WBP5	0.000287	-3.688804	
21819	Protein p53	MYBL1	0.000015	-3.678576	
21820	Protein p53	SLC1A3	0.000007	-3.673599	
21821	Protein p53	ARAF	0.000007	-3.671667	
21822	Protein p53	EXOG	0.000269	-3.658182	
21823	Protein p53	GMPR	0.000117	-3.634325	
21824	Protein p53	FLJ40113	0.000202	-3.623957	
21825	Protein p53	VWDE	0.000006	-3.608694	
21826	Protein p53	GCC2	0.004317	-3.580079	
21827	Protein p53	ACTA2	0.000025	-3.569658	
21828	Protein p53	KATNAL1	0	-3.558586	
21829	Protein p53	NCRNA00173	0.000111	-3.527212	
21830	Protein p53	BTN2A3	0.000207	-3.524927	
21831	Protein p53	SHANK3	0.000095	-3.507346	
21832	Protein p53	TBCEL	0.000057	-3.487617	
21833	Protein p53	CLTCL1	0.000153	-3.469274	
21834	Protein p53	CBX6	0.000218	-3.446218	
21835	Protein p53	ANO8	0.000039	-3.444012	
21836	Protein p53	GNB3	0.000037	-3.409344	
21837	Protein p53	CTTN	0.000929	-3.407844	
21838	Protein p53	PKD1	0.000026	-3.407801	
21839	Protein p53	HMSD	0.000144	-3.407395	
21840	Protein p53	TIMP2	0.000175	-3.386567	
21841	Protein p53	C21orf63	0.000093	-3.38271	
21842	Protein p53	CTTNBP2	0.000056	-3.361674	
21843	Protein p53	NKIRAS1	0.000187	-3.347774	
21844	Protein p53	MDM4	0.00027	-3.347505	
21845	Protein p53	LOC375190	0.000135	-3.342036	
21846	Protein p53	DPYSL3	0.000626	-3.325823	
21847	Protein p53	RHOF	0.000046	-3.323627	
21848	Protein p53	KIAA1797	0.000023	-3.287566	

21849	Protein p53	C3orf35	0.000084	-3.24477	
21850	Protein p53	TPBG	0.000069	-3.240437	
21851	Protein p53	RAB36	0.00001	-3.238865	
21852	Protein p53	CA11	0.000016	-3.221219	
21853	Protein p53	PBX1	0.000126	-3.218884	
21854	Protein p53	CR2	0.000124	-3.206954	
21855	Protein p53	C2orf27	0.000148	-3.202568	
21856	Protein p53	CDH3	0.000159	-3.193304	
21857	Protein p53	PARD3	0.000219	-3.173436	
21858	Protein p53	FRAS1	0.000002	-3.150037	
21859	Protein p53	SRGAP1	0.000261	-3.134377	
21860	Protein p53	CDRT4	0.000142	-3.126199	
21861	Protein p53	TCEAL4	0.000082	-3.120772	
21862	Protein p53	USP45	0.000223	-3.118887	
21863	Protein p53	MAP3K3	0.000158	-3.117407	
21864	Protein p53	SIM2	0.000077	-3.115892	
21865	Protein p53	ELAVL1	0.000008	-3.112688	
21866	Protein p53	KIAA1586	0.000186	-3.096102	
21867	Protein p53	RAPGEF2	0.000289	-3.095119	
21868	Protein p53	C14orf4	0.000018	-3.089096	
21869	Protein p53	SLC30A4	0.00002	-3.072022	
21870	Protein p53	GCA	0.000636	-3.054701	
21871	Protein p53	CAMK1	0.000151	-3.043395	
21872	Protein p53	LOC728047	0.000131	-3.034422	
21873	Protein p53	BCYRN1	0.000048	-3.029967	
21874	Protein p53	HYLS1	0.000139	-3.023997	
21875	Protein p53	ADORA1	0.000019	-3.012767	
21876	Protein p53	CNNM2	0.000001	-3.007572	
21877	Protein p53	HLA-E	0.000244	-3.000298	
21878	Protein p53	SCG5	0.00013	-2.997883	
21879	Protein p53	SEMA4F	0.000162	-2.992848	
21880	Protein p53	AGPAT4	0.000168	-2.989244	
21881	Protein p53	NPHP1	0.000147	-2.986739	
21882	Protein p53	ZNF37A	0.000218	-2.970766	
21883	Protein p53	SNX26	0.000022	-2.967364	
21884	Protein p53	CNTNAP1	0.000028	-2.966271	
21885	Protein p53	ATP2A1	0.000012	-2.963659	
21886	Protein p53	SYTL1	0.000103	-2.96032	
21887	Protein p53	LOC54492	0.000069	-2.953109	
21888	Protein p53	SFRS1	0.000059	-2.952906	
21889	Protein p53	LOC100132153	0.000003	-2.945493	
21890	Protein p53	STAT4	0.000066	-2.930509	
21891	Protein p53	TNFAIP8L1	0.000026	-2.906807	
21892	Protein p53	RUNDC3B	0.000047	-2.884113	
21893	Protein p53	GTF2H2	0.000295	-2.882074	
21894	Protein p53	ZNF25	0.000031	-2.880115	
21895	Protein p53	ANKRD18A	0.000249	-2.876288	
21896	Protein p53	3-Sep	0.000009	-2.871437	
21897	Protein p53	JAZF1	0.000058	-2.866248	
21898	Protein p53	TANC2	0.00003	-2.848808	

21899	Protein p53	EPOR	0.000069	-2.845683	
21900	Protein p53	CYP2U1	0.000128	-2.843941	
21901	Protein p53	GCAT	0.00009	-2.839744	
21902	Protein p53	HEMK1	0.000041	-2.837929	
21903	Protein p53	SFI1	0.000028	-2.810339	
21904	Protein p53	MFGE8	0.000003	-2.808922	
21905	Protein p53	ADAM32	0.000228	-2.80424	
21906	Protein p53	PEG10	0.003647	-2.800432	
21907	Protein p53	PSMD3	0.000273	-2.784817	
21908	Protein p53	DZIP1L	0.000008	-2.745394	
21909	Protein p53	PMAIP1	0.000012	-2.735515	
21910	Protein p53	HDAC2	0.000007	-2.728819	
21911	Protein p53	ASAM	0.000154	-2.72168	
21912	Protein p53	ITPRIPL2	0.000075	-2.686906	
21913	Protein p53	TARSL2	0.000001	-2.682116	
21914	Protein p53	RDX	0.000073	-2.67983	
21915	Protein p53	MEGF6	0.000094	-2.674067	
21916	Protein p53	HYMAI	0.000108	-2.667008	
21917	Protein p53	PKN3	0.000087	-2.66276	
21918	Protein p53	HS6ST1	0.000022	-2.650736	
21919	Protein p53	LOC652005	0.000018	-2.648741	
21920	Protein p53	EIF3F	0.000039	-2.645408	
21921	Protein p53	FBXL2	0.000111	-2.645171	
21922	Protein p53	NEK1	0.000095	-2.644595	
21923	Protein p53	ZNF57	0.000004	-2.63682	
21924	Protein p53	C21orf7	0.000003	-2.636255	
21925	Protein p53	C2orf76	0.000161	-2.632252	
21926	Protein p53	BCORL1	0.000185	-2.618549	
21927	Protein p53	TIFA	0.000069	-2.606932	
21928	Protein p53	CROP	0.000254	-2.584791	
21929	Protein p53	TNRC6C	0.000008	-2.56454	
21930	Protein p53	SEC61A2	0.000002	-2.557307	
21931	Protein p53	TRIM37	0.000077	-2.557137	
21932	Protein p53	CYFIP2	0.001529	-2.548694	
21933	Protein p53	SGSH	0.000136	-2.547538	
21934	Protein p53	THRAP3	0.000016	-2.547111	
21935	Protein p53	RAD51C	0.000073	-2.545745	
21936	Protein p53	GLT8D1	0.001959	-2.545454	
21937	Protein p53	WDR7	0.00009	-2.543718	
21938	Protein p53	SSX2IP	0.000237	-2.543506	
21939	Protein p53	CHST3	0.000017	-2.541441	
21940	Protein p53	ELAC1	0.000083	-2.535801	
21941	Protein p53	PSIP1	0.000032	-2.535774	
21942	Protein p53	TGFB1I1	0.000292	-2.521098	
21943	Protein p53	LOC728377	0.000002	-2.515295	
21944	Protein p53	ANKRD36B	0.000012	-2.511553	
21945	Protein p53	RBM4	0.000055	-2.508263	
21946	Protein p53	FAM164A	0.000078	-2.506848	
21947	Protein p53	TPST1	0.000145	-2.506486	
21948	Protein p53	GPAM	0.000217	-2.502145	

21949	Protein p53	NTN5	0.000014	-2.500072	
21950	Protein p53	FAT1	0.000041	-2.49786	
21951	Protein p53	LYPLAL1	0.000019	-2.496133	
21952	Protein p53	SULT1A1	0.000278	-2.494731	
21953	Protein p53	DDHD1	0.000015	-2.488128	
21954	Protein p53	TOP3A	0.000235	-2.487689	
21955	Protein p53	PLA2G6	0.000078	-2.483683	
21956	Protein p53	DCLK2	0.000008	-2.466714	
21957	Protein p53	F8	0.000054	-2.460219	
21958	Protein p53	DOCK7	0.00001	-2.459611	
21959	Protein p53	IFT74	0.000199	-2.45617	
21960	Protein p53	NOTCH2NL	0.000099	-2.448863	
21961	Protein p53	PIK3C3	0.000067	-2.444206	
21962	Protein p53	NOLC1	0.000003	-2.443868	
21963	Protein p53	OSBP2	0.000125	-2.441452	
21964	Protein p53	SEC31B	0.000003	-2.428658	
21965	Protein p53	TOMM34	0.003004	-2.426347	
21966	Protein p53	NFAT5	0.000137	-2.425518	
21967	Protein p53	LOC100507448	0.000008	-2.423487	
21968	Protein p53	C18orf54	0.00015	-2.422807	
21969	Protein p53	SYNCRIP	0	-2.42249	
21970	Protein p53	CELSR2	0.000148	-2.416698	
21971	Protein p53	C9orf61	0.000154	-2.385151	
21972	Protein p53	C6orf170	0.000175	-2.382184	
21973	Protein p53	EIF4EBP1	0.003167	-2.37925	
21974	Protein p53	MAMLD1	0.000008	-2.377772	
21975	Protein p53	KCTD6	0.000059	-2.376819	
21976	Protein p53	CNOT7	0.000104	-2.367895	
21977	Protein p53	DHX30	0.000125	-2.366956	
21978	Protein p53	FUT10	0.000028	-2.364717	
21979	Protein p53	BNIP3L	0.000002	-2.359025	
21980	Protein p53	UNC119	0.000046	-2.348208	
21981	Protein p53	KSR2	0.000041	-2.345834	
21982	Protein p53	INPP5E	0.000001	-2.344917	
21983	Protein p53	HSPB1	0.000012	-2.343433	
21984	Protein p53	ERI1	0.000211	-2.342181	
21985	Protein p53	TRAF3	0.000028	-2.339781	
21986	Protein p53	BTRC	0.000218	-2.339372	
21987	Protein p53	SRP72	0.000178	-2.338873	
21988	Protein p53	ZSCAN20	0.000184	-2.334139	
21989	Protein p53	GOLGA9P	0.000092	-2.322996	
21990	Protein p53	MED13L	0.000012	-2.322913	
21991	Protein p53	ZCCHC11	0.000028	-2.321129	
21992	Protein p53	FABP6	0.001061	-2.320459	
21993	Protein p53	TNFSF12	0.000061	-2.313381	
21994	Protein p53	LRPAP1	0.000069	-2.310307	
21995	Protein p53	TYMS	0.000001	-2.30578	
21996	Protein p53	MOSPD1	0.000053	-2.301269	
21997	Protein p53	CDH24	0.000056	-2.29812	
21998	Protein p53	SENP6	0.000165	-2.296558	

21999	Protein p53	UCP2	0.000188	-2.293495	
22000	Protein p53	CSPG5	0.000102	-2.291194	
22001	Protein p53	ARHGEF37	0.000015	-2.290725	
22002	Protein p53	RUSC2	0.000045	-2.289799	
22003	Protein p53	PHTF2	0.00017	-2.288694	
22004	Protein p53	C10orf137	0.000001	-2.288498	
22005	Protein p53	SRMS	0.000118	-2.283625	
22006	Protein p53	RANBP17	0.000015	-2.279878	
22007	Protein p53	C17orf69	0.000223	-2.279328	
22008	Protein p53	SHOX2	0.00001	-2.276001	
22009	Protein p53	NOC3L	0.000093	-2.275555	
22010	Protein p53	DOCK1	0	-2.267904	
22011	Protein p53	LYG1	0.000174	-2.267478	
22012	Protein p53	POLI	0.000007	-2.267001	
22013	Protein p53	NCRNA00094	0.000051	-2.255855	
22014	Protein p53	ACVR2B	0.000019	-2.243514	
22015	Protein p53	PPM1M	0.00004	-2.24155	
22016	Protein p53	NTAN1	0.000203	-2.23913	
22017	Protein p53	RAB11FIP2	0.000073	-2.234653	
22018	Protein p53	EXOC6B	0.000059	-2.233084	
22019	Protein p53	CITED2	0.000053	-2.232065	
22020	Protein p53	PNPLA6	0.000004	-2.230106	
22021	Protein p53	SLC25A39	0.000001	-2.228725	
22022	Protein p53	GNG10	0.000082	-2.228616	
22023	Protein p53	MTX3	0.000005	-2.2229	
22024	Protein p53	SLC25A30	0.0001	-2.221969	
22025	Protein p53	DDR2	0.000228	-2.217265	
22026	Protein p53	PBK	0.00004	-2.213962	
22027	Protein p53	RPS28	0.000155	-2.20962	
22028	Protein p53	HEATR6	0.000171	-2.205405	
22029	Protein p53	RNF5	0.004317	-2.204465	
22030	Protein p53	MON2	0.000038	-2.203917	
22031	Protein p53	DCLK1	0.001007	-2.202188	
22032	Protein p53	FECH	0.000165	-2.201653	
22033	Protein p53	EIF2AK4	0.000004	-2.199079	
22034	Protein p53	DUS3L	0.000158	-2.196435	
22035	Protein p53	KCTD12	0.00009	-2.188174	
22036	Protein p53	NSD1	0.00002	-2.185498	
22037	Protein p53	HMGB1	0.000257	-2.183219	
22038	Protein p53	HCG8	0.000105	-2.182757	
22039	Protein p53	POMT2	0.000136	-2.181866	
22040	Protein p53	PCNX	0.000005	-2.176842	
22041	Protein p53	YJEFN3	0.000022	-2.167793	
22042	Protein p53	HOXB13	0.00003	-2.165502	
22043	Protein p53	CEPT1	0.00011	-2.164874	
22044	Protein p53	RTTN	0.00001	-2.154799	
22045	Protein p53	CTNNAL1	0.000186	-2.152896	
22046	Protein p53	INVS	0.00029	-2.150785	
22047	Protein p53	LOC646851	0.000099	-2.150201	
22048	Protein p53	ZYG11B	0.000024	-2.149045	

22049	Protein p53	KIAA1432	0.000002	-2.144912	
22050	Protein p53	BTN2A2	0.000132	-2.143085	
22051	Protein p53	C20orf96	0.000107	-2.141774	
22052	Protein p53	FAM160B1	0.000093	-2.136974	
22053	Protein p53	ST6GALNAC6	0.000245	-2.134926	
22054	Protein p53	CDCA2	0.000062	-2.134742	
22055	Protein p53	SPTB	0.000251	-2.134205	
22056	Protein p53	DCTN5	0.002608	-2.127463	
22057	Protein p53	HDGF2	0.000008	-2.121635	
22058	Protein p53	BMP8B	0.00001	-2.118034	
22059	Protein p53	GPC1	0.000014	-2.115008	
22060	Protein p53	NISCH	0.000018	-2.113714	
22061	Protein p53	RGS10	0.000017	-2.113307	
22062	Protein p53	TOP3B	0.000022	-2.113287	
22063	Protein p53	IMPACT	0.000011	-2.111917	
22064	Protein p53	PQLC1	0.000005	-2.11065	
22065	Protein p53	C18orf24	0.00007	-2.107296	
22066	Protein p53	CP110	0.000081	-2.107218	
22067	Protein p53	MSTO2P	0.000064	-2.105921	
22068	Protein p53	KTN1	0.000281	-2.10316	
22069	Protein p53	ZMYM1	0.000171	-2.098944	
22070	Protein p53	DGCR8	0.000004	-2.097377	
22071	Protein p53	PLD6	0.000173	-2.092744	
22072	Protein p53	INTS2	0.000033	-2.084261	
22073	Protein p53	FABP5	0.00025	-2.081836	
22074	Protein p53	LAMB2	0.000265	-2.080383	
22075	Protein p53	KPNA5	0.000021	-2.080091	
22076	Protein p53	INSR	0.000057	-2.074774	
22077	Protein p53	IFI30	0.001901	-2.073481	
22078	Protein p53	LRP10	0.001222	-2.069649	
22079	Protein p53	GCN1L1	0.000426	-2.066851	
22080	Protein p53	MYBBP1A	0.000069	-2.05959	
22081	Protein p53	PPAP2B	0.002708	-2.057697	
22082	Protein p53	CUL9	0.000024	-2.055442	
22083	Protein p53	GEMIN5	0.000188	-2.052325	
22084	Protein p53	C3orf39	0.000289	-2.04922	
22085	Protein p53	DENND5A	0.000169	-2.04788	
22086	Protein p53	CDKL3	0.000247	-2.04579	
22087	Protein p53	ERCC1	0.000587	-2.044943	
22088	Protein p53	RAVER2	0.000087	-2.039754	
22089	Protein p53	METTL10	0.000008	-2.03607	
22090	Protein p53	KIAA1333	0.000072	-2.030055	
22091	Protein p53	QRICH2	0.000234	-2.027934	
22092	Protein p53	AUH	0.000004	-2.02723	
22093	Protein p53	NOL3	0.002096	-2.024963	
22094	Protein p53	RPL4	0.000192	-2.02487	
22095	Protein p53	TRMT1	0.000044	-2.021316	
22096	Protein p53	ABCF1	0.000092	-2.019239	
22097	Protein p53	AVL9	0.001231	-2.01783	
22098	Protein p53	MTHFD1	0.001476	-2.017781	

22099	Protein p53	GCFC1	0.000028	-2.012418	
22100	Protein p53	CD44	0.003366	-2.010455	
22101	Protein p53	5-Mar	0.000021	-2.009577	
22102	Protein p53	ADAM22	0.000216	-2.00715	
22103	Protein p53	NHLRC2	0.000173	-2.005527	
22104	Protein p53	ZBTB8	0.000169	-2.002427	
22105	Protein p53	LOC339988	0.000008	2.000944	
22106	Protein p53	MFAP2	0	2.032549	
22107	Protein p53	GPR56	0.000287	2.03382	
22108	Protein p53	YLPM1	0.000302	2.056725	
22109	Protein p53	LEPROT	0.000244	2.056876	
22110	Protein p53	PRKCDBP	0.000636	2.06511	
22111	Protein p53	CALD1	0	2.066687	
22112	Protein p53	S100A11	0.000071	2.068479	
22113	Protein p53	CCDC64	0.000014	2.069689	
22114	Protein p53	BGN	0.000202	2.072609	
22115	Protein p53	ADRBK1	0.000003	2.075921	
22116	Protein p53	TNKS2	0.001371	2.077541	
22117	Protein p53	MCCC1	0.000077	2.080364	
22118	Protein p53	MTMR2	0.000008	2.083627	
22119	Protein p53	IGHG1	0	2.086508	
22120	Protein p53	TPM2	0	2.094737	
22121	Protein p53	TMEM164	0.000033	2.103535	
22122	Protein p53	FTH1	0.000196	2.10661	
22123	Protein p53	PCGF5	0.000051	2.107123	
22124	Protein p53	ARFGEF2	0.000032	2.111078	
22125	Protein p53	C3orf23	0	2.119588	
22126	Protein p53	ACY1	0.000024	2.124582	
22127	Protein p53	LRRC8A	0.00026	2.128568	
22128	Protein p53	RBCK1	0.00012	2.130934	
22129	Protein p53	WDR67	0.000066	2.131068	
22130	Protein p53	LOC100132999	0.000077	2.13246	
22131	Protein p53	GSTK1	0.000047	2.134457	
22132	Protein p53	ECH1	0.000232	2.136261	
22133	Protein p53	SRI	0.0047	2.139194	
22134	Protein p53	ETV6	0.000197	2.139379	
22135	Protein p53	KIF13B	0.000002	2.140129	
22136	Protein p53	DLG3	0.000157	2.144243	
22137	Protein p53	CCDC82	0.000073	2.151796	
22138	Protein p53	MMP11	0	2.155168	
22139	Protein p53	HSDL2	0.000052	2.158646	
22140	Protein p53	TMEM158	0	2.168515	
22141	Protein p53	MCF2L	0.000021	2.184457	
22142	Protein p53	LFNG	0.000028	2.189166	
22143	Protein p53	CTSK	0	2.189639	
22144	Protein p53	ROMO1	0.000002	2.201651	
22145	Protein p53	HIST4H4	0.000273	2.205272	
22146	Protein p53	SPOCK1	0	2.207104	
22147	Protein p53	ZDHHC9	0.00026	2.215214	
22148	Protein p53	UBE2D2	0.000011	2.218704	

22149	Protein p53	RICS	0.000012	2.22689	
22150	Protein p53	WNT5A	0	2.230763	
22151	Protein p53	TAGLN	0	2.230853	
22152	Protein p53	LOC100506922	0.000096	2.256579	
22153	Protein p53	COL15A1	0	2.260351	
22154	Protein p53	C20orf24	0.000011	2.267668	
22155	Protein p53	NEK3	0.001873	2.270858	
22156	Protein p53	SFRP4	0	2.276769	
22157	Protein p53	C20orf74	0.000106	2.282048	
22158	Protein p53	ABHD12	0.000046	2.296449	
22159	Protein p53	WNT11	0.000135	2.301905	
22160	Protein p53	SEC23B	0.000098	2.308136	
22161	Protein p53	SNRPA1	0.000007	2.308718	
22162	Protein p53	ODZ3	0.002619	2.310428	
22163	Protein p53	SULF1	0	2.319154	
22164	Protein p53	MYL9	0.000001	2.334374	
22165	Protein p53	PLCH1	0.000044	2.337335	
22166	Protein p53	CHMP4B	0.000095	2.343461	
22167	Protein p53	GLA	0.000029	2.353499	
22168	Protein p53	DACT1	0.000557	2.364469	
22169	Protein p53	PRKCA	0.000153	2.378513	
22170	Protein p53	ZNF223	0.000022	2.384845	
22171	Protein p53	MCTP1	0.000112	2.393982	
22172	Protein p53	CTSD	0.000039	2.401471	
22173	Protein p53	SIK1	0.000055	2.407825	
22174	Protein p53	BAIAP2	0.000699	2.414017	
22175	Protein p53	FAM62B	0.000001	2.414755	
22176	Protein p53	NUP98	0.000004	2.419936	
22177	Protein p53	INPP1	0.000097	2.422773	
22178	Protein p53	WNK1	0.000584	2.426189	
22179	Protein p53	ECOP	0.000166	2.441085	
22180	Protein p53	ARHGEF10L	0.000044	2.468049	
22181	Protein p53	MLKL	0.000008	2.480787	
22182	Protein p53	COL6A1	0	2.48556	
22183	Protein p53	PSME4	0.003031	2.503896	
22184	Protein p53	EFNB1	0.000003	2.511787	
22185	Protein p53	LAD1	0.000172	2.563215	
22186	Protein p53	NARG2	0.000031	2.593275	
22187	Protein p53	AGA	0.000215	2.593541	
22188	Protein p53	POLD4	0.000018	2.59396	
22189	Protein p53	AADACL1	0	2.662401	
22190	Protein p53	METTL9	0.000027	2.673071	
22191	Protein p53	ADAP1	0.0001	2.687005	
22192	Protein p53	DPAGT1	0	2.696705	
22193	Protein p53	AQR	0.000636	2.721913	
22194	Protein p53	JUN	0.000055	2.725946	
22195	Protein p53	RGS14	0.000096	2.726364	
22196	Protein p53	SATB1	0.000097	2.751735	
22197	Protein p53	MMP3	0	2.756449	
22198	Protein p53	ARHGAP42	0.000055	2.773032	

22199	Protein p53	TUBA4A	0.000008	2.789495	
22200	Protein p53	COL11A1	0	2.790647	
22201	Protein p53	EPS8L3	0.000032	2.795256	
22202	Protein p53	ZNF185	0.00026	2.799903	
22203	Protein p53	TNC	0	2.807085	
22204	Protein p53	FUT4	0.000078	2.815228	
22205	Protein p53	TST	0.000291	2.81638	
22206	Protein p53	IGF2R	0.001778	2.825601	
22207	Protein p53	MMP1	0.000001	2.832884	
22208	Protein p53	PTPRB	0.000082	2.847353	
22209	Protein p53	FAM108C1	0.000281	2.89171	
22210	Protein p53	MYH11	0	2.897326	
22211	Protein p53	SEC14L1	0.003716	2.912447	
22212	Protein p53	SLC12A7	0	2.913571	
22213	Protein p53	ITGA6	0.000017	2.921719	
22214	Protein p53	TMEM133	0.000081	2.945957	
22215	Protein p53	SLC25A13	0.000108	2.981576	
22216	Protein p53	CHPT1	0.000001	2.983401	
22217	Protein p53	GAS1	0	3.007882	
22218	Protein p53	PTK6	0.000071	3.034597	
22219	Protein p53	RPP25	0.000177	3.049872	
22220	Protein p53	SLC5A1	0.000046	3.058335	
22221	Protein p53	MBD4	0.002309	3.082864	
22222	Protein p53	KALRN	0.000069	3.11255	
22223	Protein p53	BAIAP2L2	0.000084	3.122287	
22224	Protein p53	LAMB3	0.000027	3.13326	
22225	Protein p53	TNRC18	0.000005	3.135812	
22226	Protein p53	GMDS	0.000027	3.157851	
22227	Protein p53	TGFBR2	0.000016	3.161223	
22228	Protein p53	AMACR	0.000075	3.18741	
22229	Protein p53	COL17A1	0.000084	3.189146	
22230	Protein p53	TTC37	0.000244	3.197723	
22231	Protein p53	ITGB8	0.000932	3.228952	
22232	Protein p53	TMC7	0.000037	3.231842	
22233	Protein p53	RABGAP1L	0	3.273762	
22234	Protein p53	WWC2	0.003224	3.308669	
22235	Protein p53	MFAP5	0	3.321188	
22236	Protein p53	FERMT1	0.000109	3.325981	
22237	Protein p53	ST3GAL4	0.000263	3.331892	
22238	Protein p53	GCOM1	0.000018	3.35798	
22239	Protein p53	HSPA4	0.003679	3.426198	
22240	Protein p53	USH1C	0.000014	3.47718	
22241	Protein p53	PLEKHA6	0.000206	3.528435	
22242	Protein p53	GREM1	0	3.545274	
22243	Protein p53	TIPARP	0.002244	3.652352	
22244	Protein p53	TRAK1	0.000004	3.684815	
22245	Protein p53	ISG20	0.000055	3.692702	
22246	Protein p53	BMP4	0.000636	3.741762	
22247	Protein p53	F5	0.000272	3.810503	
22248	Protein p53	FBLIM1	0.000102	3.989457	

22249	Protein p53	FRK	0.00013	4.030782	
22250	Protein p53	C5	0.000039	4.072259	
22251	Protein p53	PRSS3	0.000095	4.109125	
22252	Protein p53	MIA2	0.000019	4.20487	
22253	Protein p53	LMO7	0	4.488017	
22254	Protein p53	TIMM17B	0.000009	4.493556	
22255	Protein p53	IL20RA	0.000092	4.620432	
22256	Protein p53	LOC339290	0.000114	4.671562	
22257	Protein p53	DNM1	0.000111	4.792522	
22258	Protein p53	PSD4	0.000002	4.836057	
22259	Protein p53	ZNF91	0.002866	4.906171	
22260	Protein p53	ACTG2	0	4.912122	
22261	Protein p53	HPDL	0.000181	4.976846	
22262	Protein p53	SLC16A4	0.000241	5.132544	
22263	Protein p53	PRSS1	0.000066	5.37163	
22264	Protein p53	LAMA3	0.000005	5.446837	
22265	Protein p53	CHN2	0.000002	5.495506	
22266	Protein p53	SLCO1B3	0.000699	5.667167	
22267	Protein p53	LOC100499467	0.000019	5.858976	
22268	Protein p53	PRDM1	0.000091	6.095754	
22269	Protein p53	NAV3	0.000312	6.131481	
22270	Protein p53	SRC	0.00013	6.169824	
22271	Protein p53	SLC2A3	0.001743	6.361693	
22272	Protein p53	FAM113B	0.000018	6.458196	
22273	Protein p53	HIST1H3G	0.000001	6.649406	
22274	Protein p53	HNF1A	0.000002	6.722007	
22275	Protein p53	UGT1A4	0.000277	6.898427	
22276	Protein p53	PRSS2	0.000029	6.999257	
22277	Protein p53	SLC2A14	0.002026	7.058454	
22278	Protein p53	ACSL5	0.00003	7.491147	
22279	Protein p53	CYP3A5	0.000271	7.717247	
22280	Protein p53	NEBL	0.003043	7.778324	
22281	Protein p53	RIN2	0	7.78971	
22282	Protein p53	RIPK3	0.000099	7.831119	
22283	Protein p53	CHFR	0.000217	8.003618	
22284	Protein p53	EHF	0.000119	8.010758	
22285	Protein p53	NSUN7	0.000002	8.518426	
22286	Protein p53	TUSC1	0.000071	8.920776	
22287	Protein p53	OAS1	0.00027	9.357664	
22288	Protein p53	CDK6	0	9.395292	
22289	Protein p53	ZNF506	0.000005	10.048693	
22290	Protein p53	RNF157	0.000085	10.802542	
22291	Protein p53	SLC40A1	0.000033	10.868366	
22292	Protein p53	C12orf27	0.000002	11.136132	
22293	Protein p53	ARL4C	0.000115	11.416531	
22294	Protein p53	NHSL1	0.000081	12.23337	
22295	Protein p53	ANKS4B	0.000009	13.769932	
22296	Protein p53	SPAG16	0.000027	14.054657	
22297	Protein p53	MGLL	0.000165	14.614457	
22298	Protein p53	RNF128	0.000065	14.813946	

22299	Protein p53	TNFSF10	0.000031	14.820611	
22300	Protein p53	CD302	0.000018	17.464301	
22301	Protein p53	C6orf150	0.000175	17.5689	
22302	Protein p53	HSD17B2	0.000074	21.748633	
22303	Protein p53	GCNT3	0.000067	24.623659	
22304	Protein p53	C4orf18	0.000016	26.415101	
22305	Protein p53	SGK2	0.000001	29.128191	
22306	Protein p53	LOC100124692	0.000008	30.496008	
22307	Protein p53	UGT1A3	0.000215	31.187261	
22308	Protein p53	FLJ20184	0	32.739482	
22309	Protein p53	ZBED2	0.000527	34.369777	
22310	Protein p53	HIST1H2BB	0	37.778601	
22311	Protein p53	HIST1H2BI	0.000014	44.493057	
22312	Protein p53	GIPC2	0.000041	47.919546	
22313	Protein p53	ALDH1A1	0.000066	49.933504	
22314	Protein p53	HIST1H3C	0.000172	51.063572	
22315	Protein p53	UGT1A1	0.000056	57.932938	
22316	Protein p53	TM4SF1	0.000002	62.391396	
22317	Protein p53	LGALS4	0.000069	100.099554	
22318	Protein p53	LYZ	0.000006	197.566048	
22319	Radiation	REG1B	0.000008	-9.377253	
22320	Radiation	CLCA4	0	-7.004552	
22321	Radiation	MS4A12	0.000013	-6.113246	
22322	Radiation	CEACAM7	0.000004	-4.815933	
22323	Radiation	SI	0.000013	-4.602946	
22324	Radiation	SLC26A3	0.000001	-4.120587	
22325	Radiation	ASCL2	0.000004	-4.090253	
22326	Radiation	KLK10	0.000074	-3.447447	
22327	Radiation	C10orf99	0	-3.417173	
22328	Radiation	CA4	0.000027	-3.322223	
22329	Radiation	PI3	0.000053	-3.20333	
22330	Radiation	DMBT1	0.000005	-3.158941	
22331	Radiation	BTNL8	0.00003	-3.143397	
22332	Radiation	GAD1	0	-3.131845	
22333	Radiation	GRM8	0.000021	-3.036762	
22334	Radiation	SEMG1	0.000096	-3.02247	
22335	Radiation	GPA33	0.000133	-2.970294	
22336	Radiation	ETV4	0	-2.956175	
22337	Radiation	HOXA10	0.000001	-2.927409	
22338	Radiation	PPP1R1B	0.000003	-2.925221	
22339	Radiation	PLAC8	0	-2.869226	
22340	Radiation	GCNT3	0.000031	-2.818528	
22341	Radiation	HSD11B2	0.000006	-2.758098	
22342	Radiation	ACE2	0	-2.703972	
22343	Radiation	AFAP1-AS	0	-2.656585	
22344	Radiation	GAL3ST1	0	-2.642896	
22345	Radiation	GJB5	0	-2.619622	
22346	Radiation	SLC26A2	0.000004	-2.572697	
22347	Radiation	ACSL6	0	-2.558908	
22348	Radiation	CBLC	0.000087	-2.553178	

22349	Radiation	LRRC19	0.000013	-2.544712	
22350	Radiation	OSTBETA	0.000016	-2.541604	
22351	Radiation	PRR15	0	-2.516785	
22352	Radiation	HR	0.000005	-2.516208	
22353	Radiation	PLA2G10	0.000004	-2.461758	
22354	Radiation	SLC13A2	0.000004	-2.460392	
22355	Radiation	UGT8	0.000131	-2.430706	
22356	Radiation	HHLA2	0.000001	-2.426586	
22357	Radiation	NOX1	0.000123	-2.424593	
22358	Radiation	EPN3	0.000101	-2.407784	
22359	Radiation	PRSS3	0.000011	-2.399846	
22360	Radiation	TRPM6	0.000047	-2.38064	
22361	Radiation	ROBO2	0.000131	-2.373002	
22362	Radiation	CDX1	0.000121	-2.362139	
22363	Radiation	XK	0.000009	-2.360366	
22364	Radiation	PLEKHA6	0.000038	-2.355337	
22365	Radiation	HPDL	0.000137	-2.329338	
22366	Radiation	IHH	0.000002	-2.327418	
22367	Radiation	GPX2	0.00004	-2.31021	
22368	Radiation	CLDN2	0.000009	-2.291243	
22369	Radiation	RAPGEFL1	0.000308	-2.288837	
22370	Radiation	HOXA13	0.000002	-2.285029	
22371	Radiation	ZG16B	0.000092	-2.280213	
22372	Radiation	CKMT1A	0.000115	-2.276833	
22373	Radiation	PKP2	0.000008	-2.26335	
22374	Radiation	ITPKA	0.000006	-2.261717	
22375	Radiation	PRSS1	0	-2.261085	
22376	Radiation	TMEM54	0.000032	-2.249464	
22377	Radiation	CDH17	0.00014	-2.241173	
22378	Radiation	PLEK2	0.000022	-2.237062	
22379	Radiation	CDC45	0.000219	-2.210318	
22380	Radiation	GIPC2	0.0001	-2.203936	
22381	Radiation	NOS2	0.000008	-2.193151	
22382	Radiation	SCIN	0	-2.191931	
22383	Radiation	SELENBP1	0.000183	-2.187424	
22384	Radiation	LOC100505633	0.000266	-2.184313	
22385	Radiation	HOXA5	0	-2.174766	
22386	Radiation	PRSS12	0.000066	-2.167532	
22387	Radiation	SFN	0.000168	-2.167506	
22388	Radiation	GGT6	0.00013	-2.164095	
22389	Radiation	ARHGAP8	0.000121	-2.145479	
22390	Radiation	DSC3	0.000105	-2.13763	
22391	Radiation	LOC400573	0.000006	-2.13572	
22392	Radiation	LRRC31	0.000138	-2.12492	
22393	Radiation	LOC729680	0.000005	-2.124049	
22394	Radiation	PITX1	0.000128	-2.113922	
22395	Radiation	C16orf53	0.000145	-2.110319	
22396	Radiation	PLS1	0.000089	-2.108095	
22397	Radiation	TPX2	0.000007	-2.099725	
22398	Radiation	STYK1	0.000053	-2.099148	

22399	Radiation	ADAP1	0	-2.09866	
22400	Radiation	BCL2L15	0.000013	-2.096901	
22401	Radiation	ESRP1	0.000046	-2.096632	
22402	Radiation	ANLN	0.000004	-2.091956	
22403	Radiation	CEACAM1	0.000011	-2.086042	
22404	Radiation	YBX2	0.000065	-2.081157	
22405	Radiation	HKDC1	0.000075	-2.080243	
22406	Radiation	CDCA3	0.000001	-2.079912	
22407	Radiation	BCL2L14	0.000213	-2.078232	
22408	Radiation	NCAPH	0.000073	-2.072185	
22409	Radiation	ARHGAP32	0.000001	-2.070218	
22410	Radiation	C15orf48	0.000112	-2.06679	
22411	Radiation	FXYD3	0	-2.064529	
22412	Radiation	PLK1	0.000002	-2.064204	
22413	Radiation	TTC22	0.000212	-2.060377	
22414	Radiation	GRHL2	0.00002	-2.05978	
22415	Radiation	KLF5	0.000151	-2.049687	
22416	Radiation	TRIM2	0.000011	-2.045956	
22417	Radiation	SATB2	0.000201	-2.042722	
22418	Radiation	LAD1	0.000023	-2.040375	
22419	Radiation	C1orf106	0.000021	-2.038333	
22420	Radiation	MYO1A	0.000026	-2.032036	
22421	Radiation	KBTBD11	0.00003	-2.031752	
22422	Radiation	PRSS8	0.000014	-2.015174	
22423	Radiation	CDCA7	0.000114	-2.01385	
22424	Radiation	KIAA1244	0.000241	-2.011288	
22425	Radiation	FERMT1	0.000065	-2.002065	
22426	Radiation	NRARP	0.000219	-2.001723	
22427	Recurrence	MFAP2	0	2.032549	
22428	Recurrence	CALD1	0	2.066687	
22429	Recurrence	IGHG1	0	2.086508	
22430	Recurrence	TPM2	0	2.094737	
22431	Recurrence	MMP11	0	2.155168	
22432	Recurrence	TMEM158	0	2.168515	
22433	Recurrence	CTSK	0	2.189639	
22434	Recurrence	SPOCK1	0	2.207104	
22435	Recurrence	WNT5A	0	2.230763	
22436	Recurrence	TAGLN	0	2.230853	
22437	Recurrence	COL15A1	0	2.260351	
22438	Recurrence	SFRP4	0	2.276769	
22439	Recurrence	SULF1	0	2.319154	
22440	Recurrence	MYL9	0.000001	2.334374	
22441	Recurrence	COL6A1	0	2.48556	
22442	Recurrence	MMP3	0	2.756449	
22443	Recurrence	COL11A1	0	2.790647	
22444	Recurrence	TNC	0	2.807085	
22445	Recurrence	MMP1	0.000001	2.832884	
22446	Recurrence	MYH11	0	2.897326	
22447	Recurrence	GAS1	0	3.007882	
22448	Recurrence	MFAP5	0	3.321188	

22449	Recurrence	GREM1	0	3.545274	
22450	Recurrence	ACTG2	0	4.912122	
22451	Red stools	KRT7	0.014466	-3.354952	
22452	Red stools	TNNT1	0.015899	-3.260496	
22453	Red stools	KLHL14	0.026774	-2.208134	
22454	Red stools	TFAP2A	0.013004	-2.126966	
22455	Red stools	PRAC	0.005446	2.523307	
22456	Redness	KRT7	0.014466	-3.354952	
22457	Redness	TNNT1	0.015899	-3.260496	
22458	Redness	KLHL14	0.026774	-2.208134	
22459	Redness	TFAP2A	0.013004	-2.126966	
22460	Redness	PRAC	0.005446	2.523307	
22461	Relapse	CLCA4	0	-6.997335	
22462	Relapse	PLIN1	0.003838	-6.51506	
22463	Relapse	ADIPOQ	0.000471	-5.700469	
22464	Relapse	FABP4	0.000098	-5.506863	
22465	Relapse	ITLN1	0.000035	-5.058006	
22466	Relapse	RETNLB	0.000023	-5.014788	
22467	Relapse	REG3A	0.001014	-4.68324	
22468	Relapse	CLCA1	0.000005	-4.662234	
22469	Relapse	AQP8	0.000232	-4.08574	
22470	Relapse	CEACAM7	0.000005	-3.86524	
22471	Relapse	CA4	0	-3.800329	
22472	Relapse	SPINK4	0.004038	-3.650813	
22473	Relapse	FCGBP	0.000157	-3.520092	
22474	Relapse	NKX2-3	0	-3.498712	
22475	Relapse	DUOX2	0	-3.434831	
22476	Relapse	SLC26A3	0.000039	-3.303359	
22477	Relapse	TNMD	0.004579	-3.194664	
22478	Relapse	SCARA5	0	-3.148793	
22479	Relapse	IGJ	0.000001	-3.109702	
22480	Relapse	LOC646627	0.000025	-3.033957	
22481	Relapse	PI3	0.000162	-2.915444	
22482	Relapse	GUCA2A	0.001833	-2.744463	
22483	Relapse	DUOXA2	0	-2.730663	
22484	Relapse	IGHM	0.000149	-2.710103	
22485	Relapse	GUCA2B	0.000875	-2.672136	
22486	Relapse	ST6GALNAC1	0.00122	-2.632715	
22487	Relapse	LPL	0.002952	-2.541677	
22488	Relapse	MAB21L2	0.000051	-2.535263	
22489	Relapse	DHRS9	0.000047	-2.512144	
22490	Relapse	TRPA1	0.000003	-2.497909	
22491	Relapse	PLAC8	0.000129	-2.465376	
22492	Relapse	L1TD1	0.000019	-2.455642	
22493	Relapse	S100B	0.004234	-2.381022	
22494	Relapse	VIP	0.002087	-2.360163	
22495	Relapse	GPR120	0.002042	-2.351596	
22496	Relapse	C6orf105	0.000007	-2.274063	
22497	Relapse	TIMP4	0.000026	-2.267333	
22498	Relapse	CAPN9	0.001351	-2.26588	

22499	Relapse	FAM3D	0.000022	-2.241042	
22500	Relapse	CLC	0.000007	-2.176222	
22501	Relapse	BTNL8	0.000939	-2.157317	
22502	Relapse	SELENBP1	0.000003	-2.15416	
22503	Relapse	TNFRSF17	0.000958	-2.153672	
22504	Relapse	NOS2	0	-2.136165	
22505	Relapse	CCL20	0.000022	-2.133764	
22506	Relapse	TSPAN11	0.000001	-2.129942	
22507	Relapse	FAM5C	0.004156	-2.090436	
22508	Relapse	LOC100507804	0.000414	-2.082244	
22509	Relapse	TPSAB1	0.00003	-2.077828	
22510	Relapse	TPSG1	0.003084	-2.076429	
22511	Relapse	FAM55A	0.004579	-2.023956	
22512	Relapse	PCDH18	0.000135	-2.009583	
22513	Relapse	GMDS	0	-2.004959	
22514	Relapse	ATP2A3	0.001074	-2.004061	
22515	Relapse	C10orf99	0.000499	-2.003806	
22516	Relapse	JARID1A	0	2.127838	
22517	Relapse	CILP	0.000001	2.143193	
22518	Relapse	C1S	0	2.287361	
22519	Relapse	SPP1	0.000445	2.478418	
22520	Relapse	MYEOV2	0	2.715434	
22521	Relapse	BCL9	0.000004	2.826559	
22522	Relapse	EYA1	0	2.863305	
22523	Relapse	SMARCE1	0	3.040492	
22524	Relapse	LOC284058	0	4.007837	
22525	Relapse	NNAT	0	4.850585	
22526	Relapse	CRABP2	0	5.164568	
22527	Relapse	MMP11	0	6.757327	
22528	Relapse	COL9A2	0	7.314118	
22529	Relapse	PRKX	0	7.847356	
22530	Relapse	COL2A1	0	15.420948	
22531	RNA	COL1A1	0	-5.27529	
22532	RNA	MMP7	0	-4.132738	
22533	RNA	SPP1	0	-4.130239	
22534	RNA	IL8	0	-3.187774	
22535	RNA	KRT23	0	-3.15084	
22536	RNA	COL1A2	0	-3.116379	
22537	RNA	TGFBI	0	-2.936139	
22538	RNA	INHBA	0	-2.666466	
22539	RNA	HKDC1	0	-2.660582	
22540	RNA	NFE2L3	0	-2.463859	
22541	RNA	CCL20	0	-2.310437	
22542	RNA	SFRP4	0	-2.294594	
22543	RNA	MMP12	0	-2.283803	
22544	RNA	COL10A1	0	-2.176511	
22545	RNA	MYC	0	-2.131113	
22546	RNA	JUB	0	-2.121437	
22547	RNA	GZMB	0	-2.088884	
22548	RNA	CDC123	0	2.016474	

22549	RNA	C6orf118	0	2.143404	
22550	RNA	CD1A	0	2.195462	
22551	RNA	NUDCD1	0	2.265265	
22552	RNA	ENPEP	0	2.363024	
22553	RNA, Small Interfering	HIST1H1B	0	-2.64695	
22554	RNA, Small Interfering	HIST2H2AC	0	-2.415358	
22555	RNA, Small Interfering	CASP8AP2	0	-2.371293	
22556	RNA, Small Interfering	FCGBP	0.000001	2.00844	
22557	RNA, Small Interfering	NUPR1	0.000001	2.016364	
22558	RNA, Small Interfering	PLAC1	0	2.020517	
22559	RNA, Small Interfering	FOSB	0.000001	2.032871	
22560	RNA, Small Interfering	MMP9	0.000002	2.041901	
22561	RNA, Small Interfering	ANTXR2	0.000001	2.053014	
22562	RNA, Small Interfering	CYP24A1	0	2.057296	
22563	RNA, Small Interfering	CA13	0.000001	2.081924	
22564	RNA, Small Interfering	LOC644774	0.028042	2.109325	
22565	RNA, Small Interfering	CITED2	0.024419	2.111216	
22566	RNA, Small Interfering	ULBP2	0	2.114992	
22567	RNA, Small Interfering	SLC16A6	0	2.118581	
22568	RNA, Small Interfering	B3GNT3	0	2.131492	
22569	RNA, Small Interfering	HIC1	0	2.133742	
22570	RNA, Small Interfering	IL23A	0.000001	2.153786	
22571	RNA, Small Interfering	LY6K	0.000001	2.204235	
22572	RNA, Small Interfering	IRF7	0.000001	2.207127	
22573	RNA, Small Interfering	KRT34	0	2.211508	
22574	RNA, Small Interfering	SNAI1	0	2.212177	
22575	RNA, Small Interfering	TBX2	0	2.270576	
22576	RNA, Small Interfering	SMPD3	0	2.28961	
22577	RNA, Small Interfering	AREG	0	2.300718	
22578	RNA, Small Interfering	EEF1A2	0	2.317045	
22579	RNA, Small Interfering	CFTR	0.000001	2.32035	
22580	RNA, Small Interfering	FAM83D	0.030884	2.323368	
22581	RNA, Small Interfering	SERPINB2	0.000001	2.330261	
22582	RNA, Small Interfering	LOC646626	0.000001	2.34748	
22583	RNA, Small Interfering	DIRAS1	0.000002	2.374302	
22584	RNA, Small Interfering	SRPK3	0.000001	2.390633	
22585	RNA, Small Interfering	TMEM173	0.000001	2.426391	
22586	RNA, Small Interfering	JMJD1A	0.028597	2.426486	
22587	RNA, Small Interfering	EPPK1	0	2.435479	
22588	RNA, Small Interfering	ADRB2	0	2.456163	
22589	RNA, Small Interfering	ZC3H7B	0.000001	2.506225	
22590	RNA, Small Interfering	IL11	0.000001	2.52461	
22591	RNA, Small Interfering	EREG	0.000002	2.526433	
22592	RNA, Small Interfering	ADM	0.033883	2.53193	
22593	RNA, Small Interfering	CKM	0.000001	2.533892	
22594	RNA, Small Interfering	C3orf32	0	2.595676	
22595	RNA, Small Interfering	SGK1	0	2.605669	
22596	RNA, Small Interfering	OVOL1	0.000001	2.626017	
22597	RNA, Small Interfering	HIST1H2BB	0	2.673884	
22598	RNA, Small Interfering	ANGPT2	0	2.677362	

22599	RNA, Small Interfering	PFKFB3	0.031328	2.682371	
22600	RNA, Small Interfering	CX3CL1	0.000001	2.688947	
22601	RNA, Small Interfering	BNIP3L	0.030536	2.760907	
22602	RNA, Small Interfering	COMP	0.000001	2.781728	
22603	RNA, Small Interfering	SPOCK2	0.000001	2.834058	
22604	RNA, Small Interfering	LYPD5	0	2.848822	
22605	RNA, Small Interfering	IL3RA	0.000002	2.944213	
22606	RNA, Small Interfering	WSB1	0.027134	2.964485	
22607	RNA, Small Interfering	HIST1H2BC	0	2.989315	
22608	RNA, Small Interfering	DEFA3	0	3.007719	
22609	RNA, Small Interfering	CH25H	0	3.023039	
22610	RNA, Small Interfering	TKTL1	0.000001	3.062219	
22611	RNA, Small Interfering	HIST1H2BK	0	3.07581	
22612	RNA, Small Interfering	ARC	0	3.076423	
22613	RNA, Small Interfering	PFKFB4	0.028784	3.087503	
22614	RNA, Small Interfering	SLPI	0	3.096307	
22615	RNA, Small Interfering	CSF1R	0	3.106477	
22616	RNA, Small Interfering	LGALS7B	0	3.128339	
22617	RNA, Small Interfering	MC5R	0.000001	3.129998	
22618	RNA, Small Interfering	HIST1H3D	0	3.158737	
22619	RNA, Small Interfering	HIST1H2BN	0	3.181743	
22620	RNA, Small Interfering	HIST1H2BH	0	3.199799	
22621	RNA, Small Interfering	HIST1H2BM	0	3.201212	
22622	RNA, Small Interfering	HIST2H2BE	0	3.22091	
22623	RNA, Small Interfering	IL32	0.000002	3.238301	
22624	RNA, Small Interfering	ATP10A	0	3.267799	
22625	RNA, Small Interfering	HIST1H2BO	0	3.298525	
22626	RNA, Small Interfering	BNIP3	0.018093	3.31517	
22627	RNA, Small Interfering	HIST1H2BE	0	3.324678	
22628	RNA, Small Interfering	GAST	0	3.327484	
22629	RNA, Small Interfering	HIST1H2BL	0	3.328004	
22630	RNA, Small Interfering	HIST1H2BF	0	3.352824	
22631	RNA, Small Interfering	SPON2	0.000002	3.355216	
22632	RNA, Small Interfering	LGALS7	0	3.359796	
22633	RNA, Small Interfering	FGFR3	0	3.375565	
22634	RNA, Small Interfering	HIST1H2BJ	0	3.427326	
22635	RNA, Small Interfering	HIST1H2BI	0	3.497828	
22636	RNA, Small Interfering	HIST1H2AC	0	3.504752	
22637	RNA, Small Interfering	HIST3H2BB	0	3.505771	
22638	RNA, Small Interfering	AQP3	0	3.547453	
22639	RNA, Small Interfering	HIST1H2BG	0	3.588124	
22640	RNA, Small Interfering	HIST1H2AG	0	3.588856	
22641	RNA, Small Interfering	HIST1H3H	0.000002	3.595731	
22642	RNA, Small Interfering	RRAD	0.000001	3.668734	
22643	RNA, Small Interfering	HIST1H2BD	0	3.712095	
22644	RNA, Small Interfering	CTSG	0.000001	3.754189	
22645	RNA, Small Interfering	HIST2H2AA4	0.000001	3.778594	
22646	RNA, Small Interfering	HIST1H2AD	0.000002	4.007391	
22647	RNA, Small Interfering	DACT3	0.000001	4.12493	
22648	RNA, Small Interfering	ASMT	0.000001	4.146187	

22649	RNA, Small Interfering	GZMB	0	4.160592	
22650	RNA, Small Interfering	DDIT4	0.024419	4.482117	
22651	RNA, Small Interfering	LILRB3	0.000001	4.497505	
22652	RNA, Small Interfering	C5orf27	0.000001	4.82028	
22653	RNA, Small Interfering	RASD1	0	5.446898	
22654	RNA, Small Interfering	HIST1H3F	0	6.362996	
22655	S100A12 protein, human	CES2	0.000751	-2.214224	
22656	S100A12 protein, human	OAS1	0.004728	2.021775	
22657	S100A12 protein, human	RAB6IP1	0	2.15584	
22658	S100A12 protein, human	SYK	0.000765	2.237635	
22659	S100A12 protein, human	TSPAN1	0.015027	2.403828	
22660	S100A12 protein, human	MGC11242	0.042262	2.464037	
22661	S100A12 protein, human	KLF13	0.008887	2.916756	
22662	S100A12 protein, human	CKB	0.00004	3.319031	
22663	S100A12 protein, human	PTP4A3	0.002022	3.737014	
22664	Schizophrenia	M-RIP	0.028743	-2.378667	
22665	Schizophrenia	OXTR	0.028743	2.132131	
22666	Scientific Study	ACBD3	0.007851	-3.97229	
22667	Scientific Study	KRTAP5-5	0.007148	-3.883056	
22668	Scientific Study	HLA-C	0.006669	-3.673555	
22669	Scientific Study	ROBO2	0.00448	-3.662034	
22670	Scientific Study	HLA-DQB1	0.006388	-2.89817	
22671	Scientific Study	NCOA4	0.005756	-2.70302	
22672	Scientific Study	PALLD	0.005369	-2.534954	
22673	Scientific Study	CHUK	0.00462	-2.466915	
22674	Secondary malignant neoplasm of liver	GUCA2A	0	-8.661259	
22675	Secondary malignant neoplasm of liver	SLC26A3	0.000017	-7.67746	
22676	Secondary malignant neoplasm of liver	DMN	0	-7.500718	
22677	Secondary malignant neoplasm of liver	IGLV1-40	0	-7.476615	
22678	Secondary malignant neoplasm of liver	MUC2	0	-7.367205	
22679	Secondary malignant neoplasm of liver	MFAP5	0	-7.082286	
22680	Secondary malignant neoplasm of liver	IGHA1	0	-6.57383	
22681	Secondary malignant neoplasm of liver	MMP1	0	-6.222971	
22682	Secondary malignant neoplasm of liver	CHGA	0.000004	-6.084005	
22683	Secondary malignant neoplasm of liver	DES	0	-6.019378	
22684	Secondary malignant neoplasm of liver	CHRDL1	0	-5.85837	
22685	Secondary malignant neoplasm of liver	LMO3	0	-5.73808	
22686	Secondary malignant neoplasm of liver	CNN1	0	-5.726561	
22687	Secondary malignant neoplasm of liver	IGLL1	0	-5.290361	
22688	Secondary malignant neoplasm of liver	PLN	0	-5.142187	
22689	Secondary malignant neoplasm of liver	PDE5A	0	-5.13904	
22690	Secondary malignant neoplasm of liver	IGKV1-5	0	-4.874983	
22691	Secondary malignant neoplasm of liver	FHL1	0	-4.750128	
22692	Secondary malignant neoplasm of liver	POU2AF1	0	-4.745817	
22693	Secondary malignant neoplasm of liver	IGK@	0	-4.692706	
22694	Secondary malignant neoplasm of liver	IGHM	0	-4.602592	
22695	Secondary malignant neoplasm of liver	IGHV3-48	0	-4.592864	
22696	Secondary malignant neoplasm of liver	PRPH	0	-4.504848	
22697	Secondary malignant neoplasm of liver	HAND1	0	-4.491088	
22698	Secondary malignant neoplasm of liver	ST6GALNAC1	0	-4.4897	

22699	Secondary malignant neoplasm of liver	IGKC	0	-4.358594	
22700	Secondary malignant neoplasm of liver	P2RY14	0	-4.289047	
22701	Secondary malignant neoplasm of liver	LOC100508797	0	-4.252415	
22702	Secondary malignant neoplasm of liver	IGHD	0	-4.210615	
22703	Secondary malignant neoplasm of liver	LOC100507804	0	-4.15266	
22704	Secondary malignant neoplasm of liver	IGKV3-20	0	-4.097869	
22705	Secondary malignant neoplasm of liver	IGKV1D-8	0	-4.09541	
22706	Secondary malignant neoplasm of liver	CXCL14	0.000133	-4.05581	
22707	Secondary malignant neoplasm of liver	IGLJ3	0	-4.034729	
22708	Secondary malignant neoplasm of liver	TPSAB1	0	-4.029503	
22709	Secondary malignant neoplasm of liver	THBS4	0	-3.966928	
22710	Secondary malignant neoplasm of liver	FAM55A	0	-3.924033	
22711	Secondary malignant neoplasm of liver	CASQ2	0	-3.861067	
22712	Secondary malignant neoplasm of liver	IGLV2-23	0	-3.857226	
22713	Secondary malignant neoplasm of liver	APEG1	0	-3.817307	
22714	Secondary malignant neoplasm of liver	ITLN1	0	-3.790869	
22715	Secondary malignant neoplasm of liver	BEST2	0	-3.779822	
22716	Secondary malignant neoplasm of liver	BAPX1	0	-3.764069	
22717	Secondary malignant neoplasm of liver	LOC340843	0	-3.741315	
22718	Secondary malignant neoplasm of liver	PLA2G10	0	-3.722207	
22719	Secondary malignant neoplasm of liver	ATP1A2	0	-3.719575	
22720	Secondary malignant neoplasm of liver	KCNMA1	0	-3.648415	
22721	Secondary malignant neoplasm of liver	IGLL3P	0	-3.632372	
22722	Secondary malignant neoplasm of liver	CHL1	0	-3.539771	
22723	Secondary malignant neoplasm of liver	COL6A1	0	-3.510292	
22724	Secondary malignant neoplasm of liver	REG4	0.000059	-3.503367	
22725	Secondary malignant neoplasm of liver	WFDC1	0	-3.487965	
22726	Secondary malignant neoplasm of liver	C14ORF132	0	-3.474085	
22727	Secondary malignant neoplasm of liver	JPH2	0	-3.444662	
22728	Secondary malignant neoplasm of liver	LOC91316	0	-3.389754	
22729	Secondary malignant neoplasm of liver	RASL12	0	-3.339297	
22730	Secondary malignant neoplasm of liver	IGLC7	0	-3.331625	
22731	Secondary malignant neoplasm of liver	TWSG1	0	-3.318707	
22732	Secondary malignant neoplasm of liver	GREM1	0	-3.268083	
22733	Secondary malignant neoplasm of liver	EPB41L3	0	-3.256519	
22734	Secondary malignant neoplasm of liver	IGLL5	0	-3.245964	
22735	Secondary malignant neoplasm of liver	NKX2-3	0	-3.194695	
22736	Secondary malignant neoplasm of liver	IGL@	0	-3.185547	
22737	Secondary malignant neoplasm of liver	CLDN8	0	-3.15702	
22738	Secondary malignant neoplasm of liver	PRKAR2B	0	-3.156296	
22739	Secondary malignant neoplasm of liver	TPSB2	0	-3.153278	
22740	Secondary malignant neoplasm of liver	IGLV1-44	0	-3.125024	
22741	Secondary malignant neoplasm of liver	SLC4A4	0	-3.114459	
22742	Secondary malignant neoplasm of liver	CALD1	0	-3.105043	
22743	Secondary malignant neoplasm of liver	SELENBP1	0.000071	-3.066158	
22744	Secondary malignant neoplasm of liver	DARC	0.000001	-3.028116	
22745	Secondary malignant neoplasm of liver	PSG4	0.004305	-3.006374	
22746	Secondary malignant neoplasm of liver	AKR1B10	0.000698	-3.002267	
22747	Secondary malignant neoplasm of liver	LRRN2	0	-2.998523	
22748	Secondary malignant neoplasm of liver	HOXD11	0	-2.977418	

22749	Secondary malignant neoplasm of liver	MKKS	0.003646	-2.939498	
22750	Secondary malignant neoplasm of liver	WNT5A	0	-2.911278	
22751	Secondary malignant neoplasm of liver	VMD2L1	0.000134	-2.885943	
22752	Secondary malignant neoplasm of liver	SSPN	0	-2.865316	
22753	Secondary malignant neoplasm of liver	GCG	0	-2.841612	
22754	Secondary malignant neoplasm of liver	OGDH	0.000409	-2.824938	
22755	Secondary malignant neoplasm of liver	GPA33	0.000665	-2.816748	
22756	Secondary malignant neoplasm of liver	SAHH3	0.000006	-2.796622	
22757	Secondary malignant neoplasm of liver	TOX	0	-2.793252	
22758	Secondary malignant neoplasm of liver	ITM2A	0	-2.755766	
22759	Secondary malignant neoplasm of liver	CA12	0.000281	-2.753664	
22760	Secondary malignant neoplasm of liver	MEOX2	0	-2.729391	
22761	Secondary malignant neoplasm of liver	ZFYVE1	0	-2.721154	
22762	Secondary malignant neoplasm of liver	HSD11B2	0.000411	-2.707869	
22763	Secondary malignant neoplasm of liver	OLFML2A	0	-2.701749	
22764	Secondary malignant neoplasm of liver	MMP2	0	-2.683132	
22765	Secondary malignant neoplasm of liver	SPON1	0.000432	-2.646048	
22766	Secondary malignant neoplasm of liver	CAPN9	0.000016	-2.643441	
22767	Secondary malignant neoplasm of liver	LMOD1	0	-2.638127	
22768	Secondary malignant neoplasm of liver	PDZD2	0	-2.626499	
22769	Secondary malignant neoplasm of liver	CCL13	0.000261	-2.615183	
22770	Secondary malignant neoplasm of liver	VCL	0	-2.583848	
22771	Secondary malignant neoplasm of liver	STON1	0	-2.562703	
22772	Secondary malignant neoplasm of liver	DACT3	0	-2.549765	
22773	Secondary malignant neoplasm of liver	FLJ21511	0	-2.545136	
22774	Secondary malignant neoplasm of liver	TNFRSF17	0	-2.53665	
22775	Secondary malignant neoplasm of liver	AGR3	0.000704	-2.508426	
22776	Secondary malignant neoplasm of liver	FOXF2	0	-2.48074	
22777	Secondary malignant neoplasm of liver	SLCO2A1	0.000002	-2.469144	
22778	Secondary malignant neoplasm of liver	CITED2	0	-2.446933	
22779	Secondary malignant neoplasm of liver	TMIGD1	0	-2.446872	
22780	Secondary malignant neoplasm of liver	MEIS1	0	-2.442161	
22781	Secondary malignant neoplasm of liver	SERTAD4	0	-2.433668	
22782	Secondary malignant neoplasm of liver	SYNC	0	-2.431513	
22783	Secondary malignant neoplasm of liver	CTSK	0	-2.415363	
22784	Secondary malignant neoplasm of liver	AGR2	0.000531	-2.405779	
22785	Secondary malignant neoplasm of liver	KLF4	0	-2.397191	
22786	Secondary malignant neoplasm of liver	ABP1	0.000179	-2.385219	
22787	Secondary malignant neoplasm of liver	PDPN	0	-2.374417	
22788	Secondary malignant neoplasm of liver	PGM5	0.000314	-2.364494	
22789	Secondary malignant neoplasm of liver	PDZRN3	0	-2.354119	
22790	Secondary malignant neoplasm of liver	ARHGAP1	0	-2.348833	
22791	Secondary malignant neoplasm of liver	ENPP2	0	-2.348584	
22792	Secondary malignant neoplasm of liver	TPM1	0	-2.348216	
22793	Secondary malignant neoplasm of liver	SCARA5	0.000025	-2.332101	
22794	Secondary malignant neoplasm of liver	NR3C2	0	-2.319695	
22795	Secondary malignant neoplasm of liver	MYO1A	0.000642	-2.309044	
22796	Secondary malignant neoplasm of liver	INSL5	0	-2.298135	
22797	Secondary malignant neoplasm of liver	NNAT	0	-2.292745	
22798	Secondary malignant neoplasm of liver	SCNN1B	0.000177	-2.287546	

22799	Secondary malignant neoplasm of liver	FAM46C	0.000021	-2.273251	
22800	Secondary malignant neoplasm of liver	C15orf48	0.000105	-2.264361	
22801	Secondary malignant neoplasm of liver	FRZB	0.000071	-2.263728	
22802	Secondary malignant neoplasm of liver	IGFBP5	0	-2.251161	
22803	Secondary malignant neoplasm of liver	BTNL3	0.000071	-2.24713	
22804	Secondary malignant neoplasm of liver	MS4A8B	0.000135	-2.245197	
22805	Secondary malignant neoplasm of liver	RGMA	0.000001	-2.216106	
22806	Secondary malignant neoplasm of liver	BVES	0	-2.211583	
22807	Secondary malignant neoplasm of liver	C5ORF5	0	-2.20954	
22808	Secondary malignant neoplasm of liver	AOC3	0	-2.202195	
22809	Secondary malignant neoplasm of liver	DIXDC1	0	-2.201468	
22810	Secondary malignant neoplasm of liver	OXCT1	0	-2.196845	
22811	Secondary malignant neoplasm of liver	TMIGD	0.000335	-2.196593	
22812	Secondary malignant neoplasm of liver	ITIH5	0	-2.195423	
22813	Secondary malignant neoplasm of liver	COL6A2	0	-2.183643	
22814	Secondary malignant neoplasm of liver	ZMIZ1	0	-2.174006	
22815	Secondary malignant neoplasm of liver	HERC1	0.00032	-2.164396	
22816	Secondary malignant neoplasm of liver	CTSG	0.000001	-2.15913	
22817	Secondary malignant neoplasm of liver	CHN1	0	-2.155797	
22818	Secondary malignant neoplasm of liver	ATP2A3	0	-2.155052	
22819	Secondary malignant neoplasm of liver	SYNC1	0	-2.154301	
22820	Secondary malignant neoplasm of liver	DRD1IP	0	-2.152948	
22821	Secondary malignant neoplasm of liver	TSPAN7	0.000705	-2.148268	
22822	Secondary malignant neoplasm of liver	LRCH2	0	-2.1482	
22823	Secondary malignant neoplasm of liver	SOX15	0	-2.145645	
22824	Secondary malignant neoplasm of liver	LAMA1	0	-2.144402	
22825	Secondary malignant neoplasm of liver	TMEM54	0.000211	-2.141421	
22826	Secondary malignant neoplasm of liver	TAGLN	0.003011	-2.13702	
22827	Secondary malignant neoplasm of liver	PDE4D	0	-2.133546	
22828	Secondary malignant neoplasm of liver	Q8NBX4	0.000102	-2.127648	
22829	Secondary malignant neoplasm of liver	NSG1	0	-2.121418	
22830	Secondary malignant neoplasm of liver	NLGN4X	0	-2.116154	
22831	Secondary malignant neoplasm of liver	PLAT	0.000028	-2.107739	
22832	Secondary malignant neoplasm of liver	HSPB3	0	-2.092056	
22833	Secondary malignant neoplasm of liver	CSRP1	0.000153	-2.081023	
22834	Secondary malignant neoplasm of liver	PBK	0	-2.078584	
22835	Secondary malignant neoplasm of liver	RPRM	0	-2.078294	
22836	Secondary malignant neoplasm of liver	ADSV	0.000576	-2.070961	
22837	Secondary malignant neoplasm of liver	SCUBE2	0	-2.068644	
22838	Secondary malignant neoplasm of liver	PTCHD1	0	-2.064622	
22839	Secondary malignant neoplasm of liver	LOH11CR2A	0.003159	-2.056708	
22840	Secondary malignant neoplasm of liver	PDZRN4	0	-2.044879	
22841	Secondary malignant neoplasm of liver	PLCL2	0	-2.040657	
22842	Secondary malignant neoplasm of liver	ZMYND11	0	-2.02749	
22843	Secondary malignant neoplasm of liver	FAS	0	-2.026563	
22844	Secondary malignant neoplasm of liver	MGC29506	0	-2.011257	
22845	Secondary malignant neoplasm of liver	PRRT2	0	-2.008478	
22846	Secondary malignant neoplasm of liver	MALL	0.00058	-2.001755	
22847	Secondary malignant neoplasm of liver	H19	0.002505	2.028469	
22848	Secondary malignant neoplasm of liver	C2	0.000472	2.039869	

22849	Secondary malignant neoplasm of liver	CKLF	0	2.057666	
22850	Secondary malignant neoplasm of liver	MSLN	0.002114	2.196121	
22851	Secondary malignant neoplasm of liver	OLR1	0.000557	2.207088	
22852	Secondary malignant neoplasm of liver	COX6C	0	2.244844	
22853	Secondary malignant neoplasm of liver	LOC440589	0	2.249777	
22854	Secondary malignant neoplasm of liver	BGN	0.000689	2.378415	
22855	Secondary malignant neoplasm of liver	WDR12	0	2.379028	
22856	Secondary malignant neoplasm of liver	CBS	0.000703	2.411396	
22857	Secondary malignant neoplasm of liver	ADFP	0.00024	2.429958	
22858	Secondary malignant neoplasm of liver	ANGPTL4	0.000173	2.440066	
22859	Secondary malignant neoplasm of liver	ADAMTSL2	0.000012	2.453786	
22860	Secondary malignant neoplasm of liver	CYBA	0	2.455506	
22861	Secondary malignant neoplasm of liver	RPL29	0	2.488888	
22862	Secondary malignant neoplasm of liver	CYP1B1	0.000314	2.525663	
22863	Secondary malignant neoplasm of liver	CTSB	0	2.543662	
22864	Secondary malignant neoplasm of liver	ALDH4A1	0.000232	2.672146	
22865	Secondary malignant neoplasm of liver	CGNL1	0.000004	2.694607	
22866	Secondary malignant neoplasm of liver	CFHL1	0.002892	2.741774	
22867	Secondary malignant neoplasm of liver	AZGP1	0.002972	2.844943	
22868	Secondary malignant neoplasm of liver	TRIF	0.000391	2.916183	
22869	Secondary malignant neoplasm of liver	NNMT	0.000273	2.93126	
22870	Secondary malignant neoplasm of liver	CFI	0.000174	2.93439	
22871	Secondary malignant neoplasm of liver	INHBE	0.000642	2.971835	
22872	Secondary malignant neoplasm of liver	TM4SF4	0.000002	2.983023	
22873	Secondary malignant neoplasm of liver	C10orf10	0.000005	2.992878	
22874	Secondary malignant neoplasm of liver	RPL38	0	3.002224	
22875	Secondary malignant neoplasm of liver	LOC441775	0	3.107298	
22876	Secondary malignant neoplasm of liver	F5	0.00014	3.231423	
22877	Secondary malignant neoplasm of liver	BF	0.000076	3.280719	
22878	Secondary malignant neoplasm of liver	CDH2	0	3.362404	
22879	Secondary malignant neoplasm of liver	HABP2	0.000351	3.485311	
22880	Secondary malignant neoplasm of liver	VNN1	0.000533	3.670827	
22881	Secondary malignant neoplasm of liver	GRINA	0	3.747434	
22882	Secondary malignant neoplasm of liver	APOE	0	3.797515	
22883	Secondary malignant neoplasm of liver	AQP9	0.000053	3.849158	
22884	Secondary malignant neoplasm of liver	HSD11B1	0.000121	3.932743	
22885	Secondary malignant neoplasm of liver	SERF2	0	4.007006	
22886	Secondary malignant neoplasm of liver	TXN	0	4.097941	
22887	Secondary malignant neoplasm of liver	ASGR2	0.000553	4.302714	
22888	Secondary malignant neoplasm of liver	SERPINA5	0.000352	4.345646	
22889	Secondary malignant neoplasm of liver	ALDOB	0.001493	4.480913	
22890	Secondary malignant neoplasm of liver	SULT2A1	0.000605	4.720173	
22891	Secondary malignant neoplasm of liver	FMO3	0.000071	4.720673	
22892	Secondary malignant neoplasm of liver	RPS15A	0	4.778026	
22893	Secondary malignant neoplasm of liver	SERPINF2	0.00067	4.841546	
22894	Secondary malignant neoplasm of liver	SPP1	0.000007	5.105742	
22895	Secondary malignant neoplasm of liver	RPL35A	0	5.301532	
22896	Secondary malignant neoplasm of liver	CP	0.00038	5.509002	
22897	Secondary malignant neoplasm of liver	CPB2	0.000432	5.613978	
22898	Secondary malignant neoplasm of liver	C3	0.000006	5.668511	

22899	Secondary malignant neoplasm of liver	RPS17	0	5.675517	
22900	Secondary malignant neoplasm of liver	SLC13A5	0.000331	6.076943	
22901	Secondary malignant neoplasm of liver	C8A	0.000531	6.120991	
22902	Secondary malignant neoplasm of liver	RBP4	0.000557	6.658636	
22903	Secondary malignant neoplasm of liver	ASGR1	0.000048	6.679332	
22904	Secondary malignant neoplasm of liver	C5	0.00011	6.687382	
22905	Secondary malignant neoplasm of liver	APOH	0.000429	6.75181	
22906	Secondary malignant neoplasm of liver	FCN3	0.000009	6.844049	
22907	Secondary malignant neoplasm of liver	APOC2	0.000051	6.904064	
22908	Secondary malignant neoplasm of liver	AGXT	0.000773	6.935567	
22909	Secondary malignant neoplasm of liver	HPD	0.000531	7.014538	
22910	Secondary malignant neoplasm of liver	ITIH2	0.000149	7.015858	
22911	Secondary malignant neoplasm of liver	C6	0.00046	7.054535	
22912	Secondary malignant neoplasm of liver	SAA1	0.000035	7.064054	
22913	Secondary malignant neoplasm of liver	SAA4	0.000529	7.065	
22914	Secondary malignant neoplasm of liver	PLG	0.000498	7.361402	
22915	Secondary malignant neoplasm of liver	ITIH1	0.000337	7.554118	
22916	Secondary malignant neoplasm of liver	C4BPA	0.000535	7.948833	
22917	Secondary malignant neoplasm of liver	APOC1	0.000001	8.000367	
22918	Secondary malignant neoplasm of liver	TTR	0.000676	8.109081	
22919	Secondary malignant neoplasm of liver	CYP2C8	0.000673	8.207707	
22920	Secondary malignant neoplasm of liver	LBP	0.000127	8.984359	
22921	Secondary malignant neoplasm of liver	HAMP	0.000059	9.033264	
22922	Secondary malignant neoplasm of liver	APCS	0.000082	9.596353	
22923	Secondary malignant neoplasm of liver	KNG1	0.000331	10.388199	
22924	Secondary malignant neoplasm of liver	HRG	0.000312	11.044669	
22925	Secondary malignant neoplasm of liver	SERPINC1	0.000285	11.791284	
22926	Secondary malignant neoplasm of liver	SERPINA3	0.000001	11.942192	
22927	Secondary malignant neoplasm of liver	APOB	0.000088	13.0809	
22928	Secondary malignant neoplasm of liver	AHSG	0.000112	13.371622	
22929	Secondary malignant neoplasm of liver	APOC3	0.000181	13.701454	
22930	Secondary malignant neoplasm of liver	HPX	0.000192	14.287143	
22931	Secondary malignant neoplasm of liver	FGG	0.000014	14.651355	
22932	Secondary malignant neoplasm of liver	F2	0.000047	15.443368	
22933	Secondary malignant neoplasm of liver	VTN	0.000044	15.844447	
22934	Secondary malignant neoplasm of liver	ORM2	0.00009	16.094898	
22935	Secondary malignant neoplasm of liver	CYP2E1	0.000072	16.227626	
22936	Secondary malignant neoplasm of liver	FGL1	0.000039	18.32827	
22937	Secondary malignant neoplasm of liver	TF	0.000068	18.608249	
22938	Secondary malignant neoplasm of liver	GC	0.000013	20.947027	
22939	Secondary malignant neoplasm of liver	AMBP	0.000021	21.099729	
22940	Secondary malignant neoplasm of liver	APOA2	0.000023	21.365529	
22941	Secondary malignant neoplasm of liver	ALB	0.000007	23.019959	
22942	Secondary malignant neoplasm of liver	APOA1	0.000018	23.169381	
22943	Secondary malignant neoplasm of liver	FGB	0.000008	23.984148	
22944	Secondary malignant neoplasm of liver	FGA	0.000008	25.158992	
22945	Secondary malignant neoplasm of liver	ORM1	0.000008	25.514711	
22946	Secondary malignant neoplasm of lung	ZG16	0	-29.964456	
22947	Secondary malignant neoplasm of lung	CLCA4	0	-20.537645	
22948	Secondary malignant neoplasm of lung	GPM6A	0	-13.853163	

22949	Secondary malignant neoplasm of lung	SPINK4	0	-13.465482	
22950	Secondary malignant neoplasm of lung	MAB21L2	0	-10.983295	
22951	Secondary malignant neoplasm of lung	UGT2B17	0	-10.643243	
22952	Secondary malignant neoplasm of lung	MS4A12	0	-7.205396	
22953	Secondary malignant neoplasm of lung	MT1M	0	-6.978645	
22954	Secondary malignant neoplasm of lung	MUC2	0	-6.619673	
22955	Secondary malignant neoplasm of lung	DES	0	-6.381146	
22956	Secondary malignant neoplasm of lung	CLCA1	0	-6.043342	
22957	Secondary malignant neoplasm of lung	SYNM	0	-5.719373	
22958	Secondary malignant neoplasm of lung	PCK1	0	-5.468067	
22959	Secondary malignant neoplasm of lung	ACTG2	0	-5.123446	
22960	Secondary malignant neoplasm of lung	UGT2B15	0	-5.05358	
22961	Secondary malignant neoplasm of lung	CWH43	0	-5.032682	
22962	Secondary malignant neoplasm of lung	CA2	0	-5.027271	
22963	Secondary malignant neoplasm of lung	CNN1	0	-4.511228	
22964	Secondary malignant neoplasm of lung	ADAMDEC1	0	-3.897804	
22965	Secondary malignant neoplasm of lung	ZNF771	0	-3.665034	
22966	Secondary malignant neoplasm of lung	NPY1R	0	-3.492776	
22967	Secondary malignant neoplasm of lung	FHL1	0	-3.384516	
22968	Secondary malignant neoplasm of lung	CDHR5	0	-3.382035	
22969	Secondary malignant neoplasm of lung	IL1R2	0	-3.364265	
22970	Secondary malignant neoplasm of lung	UGT1A1	0	-3.361537	
22971	Secondary malignant neoplasm of lung	DNASE1L3	0	-3.330532	
22972	Secondary malignant neoplasm of lung	MATN2	0	-3.310375	
22973	Secondary malignant neoplasm of lung	SLC4A4	0	-3.182669	
22974	Secondary malignant neoplasm of lung	EIF4G2	0	-2.98055	
22975	Secondary malignant neoplasm of lung	HSPA2	0	-2.947619	
22976	Secondary malignant neoplasm of lung	FMO5	0	-2.947409	
22977	Secondary malignant neoplasm of lung	MAOA	0	-2.750041	
22978	Secondary malignant neoplasm of lung	VIP	0	-2.731216	
22979	Secondary malignant neoplasm of lung	TPH1	0	-2.679425	
22980	Secondary malignant neoplasm of lung	PLA2G4A	0	-2.665031	
22981	Secondary malignant neoplasm of lung	ABCA8	0	-2.622711	
22982	Secondary malignant neoplasm of lung	PTPN11	0	-2.594371	
22983	Secondary malignant neoplasm of lung	PRKAR2B	0	-2.549393	
22984	Secondary malignant neoplasm of lung	UGT1A8	0	-2.549025	
22985	Secondary malignant neoplasm of lung	KCNMA1	0	-2.522512	
22986	Secondary malignant neoplasm of lung	NAT2	0	-2.452153	
22987	Secondary malignant neoplasm of lung	CTH	0	-2.447057	
22988	Secondary malignant neoplasm of lung	NR3C2	0	-2.436797	
22989	Secondary malignant neoplasm of lung	TOX	0	-2.436251	
22990	Secondary malignant neoplasm of lung	DYNC1I1	0	-2.408172	
22991	Secondary malignant neoplasm of lung	ARL14	0	-2.392322	
22992	Secondary malignant neoplasm of lung	P2RY14	0	-2.370545	
22993	Secondary malignant neoplasm of lung	PTGER2	0	-2.28567	
22994	Secondary malignant neoplasm of lung	SCAMP1	0	-2.266666	
22995	Secondary malignant neoplasm of lung	MMP28	0	-2.245387	
22996	Secondary malignant neoplasm of lung	EXOC5	0	-2.228186	
22997	Secondary malignant neoplasm of lung	STK17A	0	-2.216876	
22998	Secondary malignant neoplasm of lung	LOC100506517	0	-2.14702	

22999	Secondary malignant neoplasm of lung	ALDH6A1	0	-2.116316	
23000	Secondary malignant neoplasm of lung	AKAP7	0	-2.100857	
23001	Secondary malignant neoplasm of lung	TFPI2	0	-2.095608	
23002	Secondary malignant neoplasm of lung	SLC35D1	0	-2.072882	
23003	Secondary malignant neoplasm of lung	ASPA	0	-2.044247	
23004	Secondary malignant neoplasm of lung	CHRNE	0	-2.033283	
23005	Secondary malignant neoplasm of lung	NLGN4X	0	-2.032991	
23006	Staphylococcal Protein A	HMGA1	0.000094	-9.960206	
23007	Staphylococcal Protein A	EIF4EBP2	0.000549	-2.475915	
23008	Staphylococcal Protein A	LZTS1	0.000003	-2.240321	
23009	Staphylococcal Protein A	MAL2	0.000794	-2.098098	
23010	Staphylococcal Protein A	CDK4	0.001899	-2.075749	
23011	Staphylococcal Protein A	GLS2	0.0029	-2.02811	
23012	Staphylococcal Protein A	C19orf42	0.000009	-2.000794	
23013	Stomach Diseases	COL18A1	0.000012	2.054137	
23014	Stomach Diseases	SCAF1	0.000072	2.493133	
23015	Stomach Diseases	CTSH	0.000009	2.667604	
23016	Stomach Diseases	LOC728448	0.000219	3.813428	
23017	Stomach normal	EDG2	0.005428	2.07883	
23018	Stomach normal	SCAF1	0.001266	2.126431	
23019	Stomach normal	COL18A1	0.000318	2.264734	
23020	Stomach normal	ANKRD11	0.000022	2.67729	
23021	Stomach normal	ENOSF1	0.000073	3.006317	
23022	Syndrome	KRT23	0.01294	-3.193564	
23023	Syndrome	SLC22A11	0.035716	-3.132747	
23024	Syndrome	F7	0.009004	-3.058259	
23025	Syndrome	PKLR	0.009334	-2.823042	
23026	Syndrome	PPP2R2C	0.012359	-2.663028	
23027	Syndrome	PIWIL1	0.03469	-2.641758	
23028	Syndrome	NODAL	0.004931	-2.566286	
23029	Syndrome	PAH	0.011581	-2.565458	
23030	Syndrome	PLA2G12B	0.022296	-2.562045	
23031	Syndrome	UMODL1	0.002468	-2.519158	
23032	Syndrome	NOX1	0.000276	-2.476758	
23033	Syndrome	GRM8	0.000276	-2.453255	
23034	Syndrome	C6orf15	0.022668	-2.449986	
23035	Syndrome	PCP4	0.010349	-2.336584	
23036	Syndrome	GAS2	0.000014	-2.302761	
23037	Syndrome	NKD1	0.003627	-2.224193	
23038	Syndrome	KRT6B	0.028373	-2.206861	
23039	Syndrome	CEL	0.019019	-2.072682	
23040	Syndrome	PON3	0.000276	-2.042103	
23041	Syndrome	RERGL	0.035657	-2.0279	
23042	Syndrome	HOXA3	0.006324	-2.012612	
23043	Syndrome	XIST	0.000003	1.801367	
23044	Syndrome	BNIP3	0.008021	2.029302	
23045	Syndrome	KCNG3	0.038146	2.101529	
23046	Syndrome	NCRNA00173	0.027758	2.148351	
23047	TACSTD2 protein, human	TACSTD2	0	-9.454059	
23048	TACSTD2 protein, human	VMA21	0.004565	-2.260221	

23049	TACSTD2 protein, human	TNFRSF11B	0	2.511447	
23050	TACSTD2 protein, human	ALB	0	3.762161	
23051	TCF7L2 protein, human	CYB5A	0.000114	-497.719424	
23052	TCF7L2 protein, human	PXDN	0.00003	-34.689751	
23053	TCF7L2 protein, human	ZIC2	0.000437	-31.843284	
23054	TCF7L2 protein, human	LITAF	0.000053	-26.621554	
23055	TCF7L2 protein, human	TCEA3	0.000058	-23.553522	
23056	TCF7L2 protein, human	EPB41L3	0.000351	-22.172662	
23057	TCF7L2 protein, human	UNC5A	0.000607	-21.517442	
23058	TCF7L2 protein, human	ABCB1	0.000482	-18.547662	
23059	TCF7L2 protein, human	TLE2	0.000486	-15.588353	
23060	TCF7L2 protein, human	PARVG	0.00005	-15.210227	
23061	TCF7L2 protein, human	LOC100289026	0.000015	-13.35725	
23062	TCF7L2 protein, human	NPL	0.000551	-12.480296	
23063	TCF7L2 protein, human	GNAL	0.00003	-11.805769	
23064	TCF7L2 protein, human	TNFRSF18	0.000187	-9.901515	
23065	TCF7L2 protein, human	RHBDL1	0.000075	-8.661224	
23066	TCF7L2 protein, human	TIMP3	0.000064	-8.627737	
23067	TCF7L2 protein, human	RAC3	0.000196	-8.302583	
23068	TCF7L2 protein, human	CIB2	0.000564	-8.107981	
23069	TCF7L2 protein, human	KLC3	0.000382	-7.331104	
23070	TCF7L2 protein, human	C11orf93	0.000061	-7.250724	
23071	TCF7L2 protein, human	C11orf53	0.000115	-7.249453	
23072	TCF7L2 protein, human	TTL	0.000202	-7.123457	
23073	TCF7L2 protein, human	FUT1	0.000219	-6.783496	
23074	TCF7L2 protein, human	DLX2	0.000271	-6.697201	
23075	TCF7L2 protein, human	WNT4	0.000393	-6.517647	
23076	TCF7L2 protein, human	SLC6A10P	0.000156	-6.252374	
23077	TCF7L2 protein, human	GPR124	0.000137	-6.185279	
23078	TCF7L2 protein, human	IL1F7	0.000275	-5.996226	
23079	TCF7L2 protein, human	MRAS	0.000002	-5.970402	
23080	TCF7L2 protein, human	CDH3	0.000564	-5.904728	
23081	TCF7L2 protein, human	C11orf30	0.000021	-5.900891	
23082	TCF7L2 protein, human	ZNF704	0.000142	-5.88162	
23083	TCF7L2 protein, human	PRODH	0.000408	-5.875641	
23084	TCF7L2 protein, human	TCF4	0.000341	-5.712788	
23085	TCF7L2 protein, human	ABCC4	0.000219	-5.561098	
23086	TCF7L2 protein, human	APH1B	0.000114	-5.553459	
23087	TCF7L2 protein, human	LMF1	0.000513	-5.430435	
23088	TCF7L2 protein, human	C4orf19	0.000367	-5.339512	
23089	TCF7L2 protein, human	C1QTNF1	0.000228	-5.327957	
23090	TCF7L2 protein, human	GALNT12	0.000082	-5.173536	
23091	TCF7L2 protein, human	SORBS3	0.000259	-5.101449	
23092	TCF7L2 protein, human	MXRA7	0.000001	-4.994552	
23093	TCF7L2 protein, human	PDGFC	0.000001	-4.989535	
23094	TCF7L2 protein, human	CD70	0.000555	-4.937659	
23095	TCF7L2 protein, human	CDC42EP5	0.000176	-4.798045	
23096	TCF7L2 protein, human	FLJ37644	0.000413	-4.712144	
23097	TCF7L2 protein, human	SHH	0.000143	-4.677488	
23098	TCF7L2 protein, human	RAB26	0.000042	-4.644335	

23099	TCF7L2 protein, human	PHLDA3	0.00002	-4.586603	
23100	TCF7L2 protein, human	ZNF358	0.000279	-4.585557	
23101	TCF7L2 protein, human	FLJ36031	0.000142	-4.480278	
23102	TCF7L2 protein, human	FSCN1	0.000014	-4.370552	
23103	TCF7L2 protein, human	GXYLT2	0.000569	-4.264637	
23104	TCF7L2 protein, human	LOC283104	0.000279	-4.155987	
23105	TCF7L2 protein, human	FADS2	0.000551	-3.903226	
23106	TCF7L2 protein, human	MCCC1	0.000187	-3.848696	
23107	TCF7L2 protein, human	PROCR	0.000259	-3.835196	
23108	TCF7L2 protein, human	FAM119A	0.000005	-3.765769	
23109	TCF7L2 protein, human	LTBP3	0.000002	-3.619344	
23110	TCF7L2 protein, human	FLJ41757	0.00048	-3.605058	
23111	TCF7L2 protein, human	CNFN	0.000266	-3.585442	
23112	TCF7L2 protein, human	ARHGAP32	0.00003	-3.550023	
23113	TCF7L2 protein, human	LOC729680	0.000244	-3.501818	
23114	TCF7L2 protein, human	PCCA	0.000125	-3.463857	
23115	TCF7L2 protein, human	C19orf45	0.000612	-3.436083	
23116	TCF7L2 protein, human	NOX1	0.000279	-3.434399	
23117	TCF7L2 protein, human	NAGS	0.00012	-3.375294	
23118	TCF7L2 protein, human	ATP6V1E2	0.000492	-3.370556	
23119	TCF7L2 protein, human	AGMAT	0.000114	-3.339683	
23120	TCF7L2 protein, human	AIFM3	0.000228	-3.295077	
23121	TCF7L2 protein, human	HSPB1	0.00007	-3.269693	
23122	TCF7L2 protein, human	KLF11	0.000219	-3.248634	
23123	TCF7L2 protein, human	NEO1	0.000564	-3.231681	
23124	TCF7L2 protein, human	KAZ	0.000187	-3.209017	
23125	TCF7L2 protein, human	HYI	0.000441	-3.155569	
23126	TCF7L2 protein, human	THNSL1	0.000149	-3.124434	
23127	TCF7L2 protein, human	ATPBD4	0.000112	-3.109684	
23128	TCF7L2 protein, human	MDK	0.000446	-3.092344	
23129	TCF7L2 protein, human	ASPHD2	0.000341	-3.03538	
23130	TCF7L2 protein, human	ATP13A2	0.0002	-3.004528	
23131	TCF7L2 protein, human	CPS1	0.000002	-2.967478	
23132	TCF7L2 protein, human	LOC255512	0.000361	-2.94404	
23133	TCF7L2 protein, human	ARHGEF10	0.000279	-2.912243	
23134	TCF7L2 protein, human	KRTCAP3	0.000414	-2.902283	
23135	TCF7L2 protein, human	C9orf40	0.000602	-2.854545	
23136	TCF7L2 protein, human	MMS19	0.000018	-2.84468	
23137	TCF7L2 protein, human	CLN3	0.000412	-2.84375	
23138	TCF7L2 protein, human	SH2D2A	0.000499	-2.836379	
23139	TCF7L2 protein, human	DPH5	0.000437	-2.807949	
23140	TCF7L2 protein, human	DNAJC4	0.000125	-2.793849	
23141	TCF7L2 protein, human	RPL35A	0.000129	-2.785454	
23142	TCF7L2 protein, human	CNDP2	0.00012	-2.744491	
23143	TCF7L2 protein, human	PKIG	0.000445	-2.741006	
23144	TCF7L2 protein, human	KLK12	0.000259	-2.740593	
23145	TCF7L2 protein, human	ALDOC	0.000382	-2.711191	
23146	TCF7L2 protein, human	SH3YL1	0.000002	-2.683318	
23147	TCF7L2 protein, human	GAMT	0.000143	-2.666422	
23148	TCF7L2 protein, human	GAS5	0.000142	-2.653625	

23149	TCF7L2 protein, human	WDYHV1	0.000235	-2.653118	
23150	TCF7L2 protein, human	RPL13A	0.000331	-2.645398	
23151	TCF7L2 protein, human	HAUS4	0.000177	-2.620348	
23152	TCF7L2 protein, human	DYRK4	0.00007	-2.615305	
23153	TCF7L2 protein, human	SLC25A21	0.000271	-2.542119	
23154	TCF7L2 protein, human	C9orf86	0.000367	-2.540431	
23155	TCF7L2 protein, human	STRADB	0.000143	-2.532233	
23156	TCF7L2 protein, human	SMARCD3	0.000128	-2.530565	
23157	TCF7L2 protein, human	NOB1	0.000553	-2.523267	
23158	TCF7L2 protein, human	MARVELD1	0.000568	-2.514161	
23159	TCF7L2 protein, human	DPY30	0.000143	-2.4865	
23160	TCF7L2 protein, human	GDF11	0.000432	-2.480144	
23161	TCF7L2 protein, human	C4orf14	0.000437	-2.47461	
23162	TCF7L2 protein, human	TBC1D1	0.000308	-2.448241	
23163	TCF7L2 protein, human	SNORD104	0.000624	-2.440978	
23164	TCF7L2 protein, human	SLC25A6	0.00034	-2.434351	
23165	TCF7L2 protein, human	PYGL	0.000602	-2.427988	
23166	TCF7L2 protein, human	WDR4	0.000532	-2.42595	
23167	TCF7L2 protein, human	GART	0.000434	-2.418457	
23168	TCF7L2 protein, human	SLC38A10	0.000553	-2.412111	
23169	TCF7L2 protein, human	CXXC5	0.000079	-2.389303	
23170	TCF7L2 protein, human	EIF4B	0.000312	-2.381829	
23171	TCF7L2 protein, human	ZFAND2B	0.000489	-2.366606	
23172	TCF7L2 protein, human	AGPHD1	0.000091	-2.36445	
23173	TCF7L2 protein, human	KIAA0141	0.000179	-2.349644	
23174	TCF7L2 protein, human	RBCK1	0.000486	-2.340571	
23175	TCF7L2 protein, human	ANKHD1-EIF4	0.000437	-2.305972	
23176	TCF7L2 protein, human	ATP5G2	0.000259	-2.290497	
23177	TCF7L2 protein, human	HSBP1L1	0.000067	-2.281044	
23178	TCF7L2 protein, human	ARPC3	0.000612	-2.271593	
23179	TCF7L2 protein, human	TPD52L1	0.000315	-2.270668	
23180	TCF7L2 protein, human	FRAT2	0.000418	-2.247018	
23181	TCF7L2 protein, human	ARL2	0.000607	-2.246019	
23182	TCF7L2 protein, human	SCCPDH	0.000259	-2.231617	
23183	TCF7L2 protein, human	TIMM8B	0.000254	-2.230223	
23184	TCF7L2 protein, human	BIRC6	0.000564	-2.20328	
23185	TCF7L2 protein, human	GBAS	0.000367	-2.202874	
23186	TCF7L2 protein, human	C6orf108	0.000196	-2.158936	
23187	TCF7L2 protein, human	CHST3	0.000407	-2.132689	
23188	TCF7L2 protein, human	SLC39A4	0.000378	-2.128275	
23189	TCF7L2 protein, human	MIPEP	0.00007	-2.116788	
23190	TCF7L2 protein, human	PREPL	0.000058	-2.095728	
23191	TCF7L2 protein, human	TAF1D	0.00007	-2.095709	
23192	TCF7L2 protein, human	SLC17A9	0.000143	-2.079349	
23193	TCF7L2 protein, human	EXOSC1	0.000259	-2.075314	
23194	TCF7L2 protein, human	MAGED1	0.000376	-2.073153	
23195	TCF7L2 protein, human	C1orf57	0.000615	-2.042778	
23196	TCF7L2 protein, human	C2orf28	0.00052	-2.041845	
23197	TCF7L2 protein, human	ZFP62	0.000058	-2.032576	
23198	TCF7L2 protein, human	TMEM18	0.000257	-2.026822	

23199	TCF7L2 protein, human	HN1	0.000033	2.003492	
23200	TCF7L2 protein, human	MYST3	0.000605	2.012837	
23201	TCF7L2 protein, human	MBD4	0.000087	2.02786	
23202	TCF7L2 protein, human	C17orf85	0.000287	2.030274	
23203	TCF7L2 protein, human	SH3BGRL2	0.000259	2.031019	
23204	TCF7L2 protein, human	TMEM50A	0.000259	2.047113	
23205	TCF7L2 protein, human	GPR107	0.000266	2.051135	
23206	TCF7L2 protein, human	RB1	0.000078	2.05352	
23207	TCF7L2 protein, human	FAM122B	0.000129	2.05692	
23208	TCF7L2 protein, human	GGCX	0.000191	2.075676	
23209	TCF7L2 protein, human	MAPRE2	0.000392	2.088331	
23210	TCF7L2 protein, human	UPF3A	0.000487	2.116966	
23211	TCF7L2 protein, human	ARPC5L	0.000064	2.134362	
23212	TCF7L2 protein, human	PSMD5	0.000018	2.135035	
23213	TCF7L2 protein, human	YTHDC2	0.000096	2.176141	
23214	TCF7L2 protein, human	FRG1	0.000112	2.210131	
23215	TCF7L2 protein, human	VAPA	0.000437	2.236095	
23216	TCF7L2 protein, human	ROD1	0.000114	2.270856	
23217	TCF7L2 protein, human	FAM91A2	0.000614	2.287029	
23218	TCF7L2 protein, human	HIST1H1C	0.000058	2.309695	
23219	TCF7L2 protein, human	GAPVD1	0.000259	2.351562	
23220	TCF7L2 protein, human	GPS2	0.000015	2.357439	
23221	TCF7L2 protein, human	DOLPP1	0.000064	2.403751	
23222	TCF7L2 protein, human	SUGT1	0.000413	2.430777	
23223	TCF7L2 protein, human	VAPB	0.000259	2.433886	
23224	TCF7L2 protein, human	ZC3H13	0.00008	2.449023	
23225	TCF7L2 protein, human	DSG2	0.000062	2.460754	
23226	TCF7L2 protein, human	UBE3C	0.000058	2.522976	
23227	TCF7L2 protein, human	HOXA13	0.000143	2.550304	
23228	TCF7L2 protein, human	ZNF271	0.000626	2.562873	
23229	TCF7L2 protein, human	ZCCHC2	0.000382	2.590748	
23230	TCF7L2 protein, human	TM9SF3	0.000143	2.59314	
23231	TCF7L2 protein, human	SGCB	0.000015	2.689522	
23232	TCF7L2 protein, human	MRPL35	0.000564	2.712121	
23233	TCF7L2 protein, human	SLC16A1	0.000175	2.715163	
23234	TCF7L2 protein, human	NBN	0.000065	2.803623	
23235	TCF7L2 protein, human	HOXB7	0.000109	2.850091	
23236	TCF7L2 protein, human	PRRG4	0.000001	2.878893	
23237	TCF7L2 protein, human	SEPW1	0.000014	2.899693	
23238	TCF7L2 protein, human	ASH1L	0.000058	2.991166	
23239	TCF7L2 protein, human	ALDH3A1	0.00048	3.025803	
23240	TCF7L2 protein, human	MSN	0.000121	3.047942	
23241	TCF7L2 protein, human	PIGN	0.000049	3.062276	
23242	TCF7L2 protein, human	DNAJB6	0.00007	3.07301	
23243	TCF7L2 protein, human	ARGLU1	0.000279	3.146774	
23244	TCF7L2 protein, human	TTC39C	0.00052	3.154439	
23245	TCF7L2 protein, human	ING2	0.000177	3.185976	
23246	TCF7L2 protein, human	MECOM	0.000112	3.198311	
23247	TCF7L2 protein, human	DSC2	0.000018	3.231242	
23248	TCF7L2 protein, human	TXNDC9	0.000143	3.328385	

23249	TCF7L2 protein, human	PIK3C3	0.000003	3.343665	
23250	TCF7L2 protein, human	ATXN1	0.000166	3.387009	
23251	TCF7L2 protein, human	TNRC18	0.000082	3.412363	
23252	TCF7L2 protein, human	RHOU	0.000078	3.614124	
23253	TCF7L2 protein, human	ESYT2	0.000458	3.665878	
23254	TCF7L2 protein, human	INO80C	0.000135	3.731187	
23255	TCF7L2 protein, human	TUG1	0.000015	3.752775	
23256	TCF7L2 protein, human	RTTN	0.000002	3.816143	
23257	TCF7L2 protein, human	GEM	0.000143	3.9926	
23258	TCF7L2 protein, human	TPRXL	0.00018	4.054795	
23259	TCF7L2 protein, human	LOC100505875	0.000492	4.152001	
23260	TCF7L2 protein, human	FAM50B	0.000018	4.335119	
23261	TCF7L2 protein, human	PTGR1	0.000001	4.486203	
23262	TCF7L2 protein, human	UGT1A6	0.000406	4.496336	
23263	TCF7L2 protein, human	SYNJ2	0.000382	4.635881	
23264	TCF7L2 protein, human	LIMCH1	0.000303	4.710667	
23265	TCF7L2 protein, human	ZNF558	0.000114	5.356884	
23266	TCF7L2 protein, human	ZNF426	0.000112	5.90991	
23267	TCF7L2 protein, human	SH3TC2	0.000208	6.079137	
23268	TCF7L2 protein, human	SPIN3	0.000147	7.489164	
23269	TCF7L2 protein, human	ZNF204P	0.000614	8.492386	
23270	TCF7L2 protein, human	BAT3	0.000087	9.181054	
23271	TCF7L2 protein, human	PRUNE2	0.000015	9.361624	
23272	TCF7L2 protein, human	RASA1	0.00018	9.621369	
23273	TCF7L2 protein, human	MT1M	0.000016	11.080972	
23274	TCF7L2 protein, human	FAM129A	0.000243	14.358025	
23275	TCF7L2 protein, human	SERPINB5	0.00007	14.794114	
23276	TCF7L2 protein, human	AKAP12	0.000382	19.882979	
23277	TCF7L2 protein, human	REG1A	0.000149	36.576833	
23278	Test Result	WHAMML1	0.000001	-6.860537	
23279	Test Result	CPA3	0.000003	-5.044826	
23280	Test Result	ATP7B	0.011554	-4.888935	
23281	Test Result	SNX4	0.000002	-4.667777	
23282	Test Result	PLA2G4A	0.00577	-4.500787	
23283	Test Result	DNAJB9	0.000189	-4.290727	
23284	Test Result	AZGP1	0.001881	-3.990623	
23285	Test Result	STK3	0.001294	-3.983015	
23286	Test Result	ANXA2P1	0.000976	-3.91317	
23287	Test Result	SERPINB5	0.029945	-3.882397	
23288	Test Result	UBE2M	0.005112	-3.464159	
23289	Test Result	SLC5A1	0.001881	-3.343978	
23290	Test Result	C1orf77	0.019141	-3.249476	
23291	Test Result	ITGAL	0.002207	-3.226547	
23292	Test Result	PHF20	0.004249	-3.173091	
23293	Test Result	DHFR	0.000227	-3.161007	
23294	Test Result	UBE2G1	0.00108	-3.159966	
23295	Test Result	PION	0.001009	-3.092732	
23296	Test Result	SKIL	0.001289	-3.07535	
23297	Test Result	HLA-DQB1	0.002207	-3.056883	
23298	Test Result	PPFIA1	0.000339	-3.01347	

23299	Test Result	BIRC2	0.000189	-3.001755	
23300	Test Result	PTGER4	0.000705	-2.983232	
23301	Test Result	SLC35A2	0.000279	-2.890172	
23302	Test Result	GORASP1	0.000436	-2.758516	
23303	Test Result	NTS	0.027001	-2.714367	
23304	Test Result	TGFBI	0.00009	-2.677548	
23305	Test Result	NEK1	0.029945	-2.676128	
23306	Test Result	CNR1	0.009133	-2.600063	
23307	Test Result	AIF1	0.002207	-2.587546	
23308	Test Result	ZNF7	0.001881	-2.585058	
23309	Test Result	CEP57	0.004489	-2.551301	
23310	Test Result	PTPRO	0.029339	-2.544664	
23311	Test Result	MTUS2	0.009965	-2.539415	
23312	Test Result	PRRX1	0.009965	-2.516925	
23313	Test Result	ATP2C1	0.025707	-2.462486	
23314	Test Result	LYST	0.004489	-2.419571	
23315	Test Result	RER1	0.019141	-2.318795	
23316	Test Result	UCHL3	0.029945	-2.25664	
23317	Test Result	ICAM1	0.029339	-2.238621	
23318	Test Result	CAPN3	0.000339	-2.235597	
23319	Test Result	CDH3	0.019141	-2.234701	
23320	Test Result	SPI1	0.019141	-2.21907	
23321	Test Result	HDAC4	0.027182	-2.21749	
23322	Test Result	IFITM3	0.029433	-2.212471	
23323	Test Result	APC	0.025707	-2.180582	
23324	Test Result	PTK2B	0.000773	-2.167113	
23325	Test Result	ZBTB33	0.00212	-2.133658	
23326	Test Result	MANBA	0.029433	-2.13317	
23327	Test Result	DPY19L2P2	0.027045	-2.124387	
23328	Test Result	SBNO2	0.029945	-2.10854	
23329	Test Result	PACRG	0.000302	-2.099506	
23330	Test Result	CADM3	0.000527	-2.075309	
23331	Test Result	EFHA1	0.009963	-2.070418	
23332	Test Result	CYB5R1	0.021608	-2.043114	
23333	Test Result	PAX2	0.025707	-2.027201	
23334	Test Result	TM9SF2	0.004249	-2.026946	
23335	Test Result	PTPN18	0.004489	-2.003421	
23336	Test Result	CYP2A13	0.017501	2.262004	
23337	Test Result	ZNF197	0.027955	2.435839	
23338	Thyroid carcinoma	TPST2	0.000258	2.332694	
23339	Tongue Carcinoma	TOR1AIP2	0	3.057763	
23340	Tumor Necrosis Factor-Beta	SOD2	0	-8.112067	
23341	Tumor Necrosis Factor-Beta	S100A8	0	-7.784066	
23342	Tumor Necrosis Factor-Beta	RCHY1	0.000002	-6.905795	
23343	Tumor Necrosis Factor-Beta	FN1	0	-6.761156	
23344	Tumor Necrosis Factor-Beta	SLC2A3	0	-6.570534	
23345	Tumor Necrosis Factor-Beta	NR1I2	0.000001	-6.339547	
23346	Tumor Necrosis Factor-Beta	FCGR2A	0.000001	-6.291089	
23347	Tumor Necrosis Factor-Beta	RAB31	0	-5.912531	
23348	Tumor Necrosis Factor-Beta	C15orf24	0	-5.535157	

23349	Tumor Necrosis Factor-Beta	GLCE	0.000002	-5.524381	
23350	Tumor Necrosis Factor-Beta	NGRN	0	-5.471482	
23351	Tumor Necrosis Factor-Beta	CD55	0	-5.459106	
23352	Tumor Necrosis Factor-Beta	BHLHE40	0.000001	-5.315644	
23353	Tumor Necrosis Factor-Beta	BBS4	0	-5.239002	
23354	Tumor Necrosis Factor-Beta	ETFA	0	-5.17691	
23355	Tumor Necrosis Factor-Beta	PPARG	0	-5.111144	
23356	Tumor Necrosis Factor-Beta	KLF6	0.000002	-5.014973	
23357	Tumor Necrosis Factor-Beta	EZR	0.000001	-5.013787	
23358	Tumor Necrosis Factor-Beta	SERPINF1	0	-4.967764	
23359	Tumor Necrosis Factor-Beta	THBD	0.000001	-4.944449	
23360	Tumor Necrosis Factor-Beta	IER3	0	-4.785686	
23361	Tumor Necrosis Factor-Beta	MGA	0	-4.782386	
23362	Tumor Necrosis Factor-Beta	NPC2	0	-4.761235	
23363	Tumor Necrosis Factor-Beta	ACAA2	0.000001	-4.675696	
23364	Tumor Necrosis Factor-Beta	SNRPN	0	-4.645634	
23365	Tumor Necrosis Factor-Beta	ARID5B	0	-4.638497	
23366	Tumor Necrosis Factor-Beta	PPP3CA	0.000002	-4.59796	
23367	Tumor Necrosis Factor-Beta	SEL1L	0	-4.588009	
23368	Tumor Necrosis Factor-Beta	CTGF	0	-4.584219	
23369	Tumor Necrosis Factor-Beta	PCTP	0	-4.507978	
23370	Tumor Necrosis Factor-Beta	TMEM66	0	-4.483211	
23371	Tumor Necrosis Factor-Beta	CASP6	0	-4.423836	
23372	Tumor Necrosis Factor-Beta	FSTL1	0	-4.387206	
23373	Tumor Necrosis Factor-Beta	MTUS1	0	-4.365914	
23374	Tumor Necrosis Factor-Beta	THUMPD1	0	-4.359705	
23375	Tumor Necrosis Factor-Beta	SLC2A14	0	-4.338697	
23376	Tumor Necrosis Factor-Beta	TMEM87A	0	-4.323575	
23377	Tumor Necrosis Factor-Beta	SNAP23	0	-4.318045	
23378	Tumor Necrosis Factor-Beta	DNAJA2	0	-4.224361	
23379	Tumor Necrosis Factor-Beta	MECOM	0.000001	-4.209247	
23380	Tumor Necrosis Factor-Beta	KIAA0247	0	-4.184222	
23381	Tumor Necrosis Factor-Beta	SPARC	0.000001	-4.17627	
23382	Tumor Necrosis Factor-Beta	PEX2	0.000001	-4.172725	
23383	Tumor Necrosis Factor-Beta	KIAA0430	0	-4.136647	
23384	Tumor Necrosis Factor-Beta	USO1	0.000003	-4.129096	
23385	Tumor Necrosis Factor-Beta	IGFBP7	0.000003	-4.081525	
23386	Tumor Necrosis Factor-Beta	MAPKSP1	0.000003	-4.053762	
23387	Tumor Necrosis Factor-Beta	RRN3	0.000001	-4.031623	
23388	Tumor Necrosis Factor-Beta	BCL6	0.000002	-3.985867	
23389	Tumor Necrosis Factor-Beta	BACH1	0.000002	-3.980111	
23390	Tumor Necrosis Factor-Beta	THG1L	0	-3.971637	
23391	Tumor Necrosis Factor-Beta	COPS4	0	-3.969351	
23392	Tumor Necrosis Factor-Beta	VIM	0	-3.920609	
23393	Tumor Necrosis Factor-Beta	NEK1	0.000001	-3.919012	
23394	Tumor Necrosis Factor-Beta	GJA1	0	-3.894751	
23395	Tumor Necrosis Factor-Beta	SAMM50	0	-3.863954	
23396	Tumor Necrosis Factor-Beta	KDELR3	0	-3.804807	
23397	Tumor Necrosis Factor-Beta	ERBB2IP	0.000001	-3.783421	
23398	Tumor Necrosis Factor-Beta	FBN1	0	-3.781672	

23399	Tumor Necrosis Factor-Beta	EID1	0.000001	-3.773214	
23400	Tumor Necrosis Factor-Beta	HERPUD1	0	-3.772011	
23401	Tumor Necrosis Factor-Beta	TANK	0.000002	-3.75804	
23402	Tumor Necrosis Factor-Beta	COX4I1	0.000001	-3.731766	
23403	Tumor Necrosis Factor-Beta	DEXI	0.000001	-3.723568	
23404	Tumor Necrosis Factor-Beta	RPS15A	0	-3.693139	
23405	Tumor Necrosis Factor-Beta	PSMB6	0.000001	-3.682886	
23406	Tumor Necrosis Factor-Beta	PCM1	0	-3.680223	
23407	Tumor Necrosis Factor-Beta	HNMT	0	-3.654111	
23408	Tumor Necrosis Factor-Beta	COL3A1	0	-3.631809	
23409	Tumor Necrosis Factor-Beta	WDFY3	0	-3.613076	
23410	Tumor Necrosis Factor-Beta	YIPF5	0	-3.593571	
23411	Tumor Necrosis Factor-Beta	KIAA0355	0	-3.578624	
23412	Tumor Necrosis Factor-Beta	CCNI	0	-3.564784	
23413	Tumor Necrosis Factor-Beta	SEC31A	0.000003	-3.529623	
23414	Tumor Necrosis Factor-Beta	PXDN	0	-3.493294	
23415	Tumor Necrosis Factor-Beta	IGLC7	0	-3.468113	
23416	Tumor Necrosis Factor-Beta	DDC	0	-3.464472	
23417	Tumor Necrosis Factor-Beta	KLHL24	0.000002	-3.378042	
23418	Tumor Necrosis Factor-Beta	NOP16	0	-3.358879	
23419	Tumor Necrosis Factor-Beta	JAG1	0	-3.356608	
23420	Tumor Necrosis Factor-Beta	10-Sep	0.000001	-3.328721	
23421	Tumor Necrosis Factor-Beta	CLASP2	0	-3.308735	
23422	Tumor Necrosis Factor-Beta	CFTR	0	-3.299949	
23423	Tumor Necrosis Factor-Beta	TTC19	0.000001	-3.272009	
23424	Tumor Necrosis Factor-Beta	NHP2L1	0.000001	-3.259085	
23425	Tumor Necrosis Factor-Beta	ARIH1	0.000001	-3.243082	
23426	Tumor Necrosis Factor-Beta	RAD17	0	-3.223498	
23427	Tumor Necrosis Factor-Beta	FEM1B	0	-3.213209	
23428	Tumor Necrosis Factor-Beta	RPL17	0	-3.210999	
23429	Tumor Necrosis Factor-Beta	C5AR1	0.000002	-3.177691	
23430	Tumor Necrosis Factor-Beta	COPS3	0.000001	-3.168768	
23431	Tumor Necrosis Factor-Beta	ADAM10	0	-3.162575	
23432	Tumor Necrosis Factor-Beta	EMP2	0	-3.148701	
23433	Tumor Necrosis Factor-Beta	NFIB	0	-3.141033	
23434	Tumor Necrosis Factor-Beta	NNMT	0	-3.084436	
23435	Tumor Necrosis Factor-Beta	BFAR	0	-3.077174	
23436	Tumor Necrosis Factor-Beta	BRD1	0	-3.064709	
23437	Tumor Necrosis Factor-Beta	CHMP1B	0.000001	-3.056347	
23438	Tumor Necrosis Factor-Beta	SKP1	0	-3.030561	
23439	Tumor Necrosis Factor-Beta	LSM6	0.000002	-3.022696	
23440	Tumor Necrosis Factor-Beta	PNP	0.000002	-3.005123	
23441	Tumor Necrosis Factor-Beta	HEG1	0.000002	-3.004856	
23442	Tumor Necrosis Factor-Beta	DHRS7	0.000001	-2.991803	
23443	Tumor Necrosis Factor-Beta	CRTC3	0	-2.9627	
23444	Tumor Necrosis Factor-Beta	DCTN6	0	-2.959168	
23445	Tumor Necrosis Factor-Beta	ITFG1	0.000002	-2.952976	
23446	Tumor Necrosis Factor-Beta	PDHB	0	-2.920123	
23447	Tumor Necrosis Factor-Beta	TSPAN12	0.000001	-2.901965	
23448	Tumor Necrosis Factor-Beta	CD9	0.000002	-2.889606	

23449	Tumor Necrosis Factor-Beta	KIF13B	0.000001	-2.886321	
23450	Tumor Necrosis Factor-Beta	IFNGR1	0	-2.885487	
23451	Tumor Necrosis Factor-Beta	WDR61	0	-2.879669	
23452	Tumor Necrosis Factor-Beta	CAPZA2	0.000002	-2.851265	
23453	Tumor Necrosis Factor-Beta	USP7	0.000001	-2.836964	
23454	Tumor Necrosis Factor-Beta	CBY1	0.000001	-2.818675	
23455	Tumor Necrosis Factor-Beta	CFDP1	0	-2.815822	
23456	Tumor Necrosis Factor-Beta	TSPYL1	0.000001	-2.786069	
23457	Tumor Necrosis Factor-Beta	TMEM184C	0.000002	-2.783419	
23458	Tumor Necrosis Factor-Beta	AKAP13	0	-2.77845	
23459	Tumor Necrosis Factor-Beta	ACSL1	0.000001	-2.776639	
23460	Tumor Necrosis Factor-Beta	HSBP1	0	-2.755915	
23461	Tumor Necrosis Factor-Beta	PITPNB	0.000001	-2.744486	
23462	Tumor Necrosis Factor-Beta	TM2D3	0.000002	-2.737388	
23463	Tumor Necrosis Factor-Beta	GPC3	0	-2.735538	
23464	Tumor Necrosis Factor-Beta	C4orf27	0	-2.722139	
23465	Tumor Necrosis Factor-Beta	DYNLT1	0	-2.719459	
23466	Tumor Necrosis Factor-Beta	LOC100505503	0	-2.689223	
23467	Tumor Necrosis Factor-Beta	SCAMP1	0.000001	-2.653625	
23468	Tumor Necrosis Factor-Beta	NCOA2	0	-2.64238	
23469	Tumor Necrosis Factor-Beta	FKBP3	0	-2.642153	
23470	Tumor Necrosis Factor-Beta	ADH5	0	-2.63693	
23471	Tumor Necrosis Factor-Beta	CCDC28A	0.000001	-2.588423	
23472	Tumor Necrosis Factor-Beta	BNIP2	0	-2.570422	
23473	Tumor Necrosis Factor-Beta	NET1	0.000001	-2.524456	
23474	Tumor Necrosis Factor-Beta	KRAS	0	-2.524198	
23475	Tumor Necrosis Factor-Beta	EPS8	0.000003	-2.510814	
23476	Tumor Necrosis Factor-Beta	HMGCR	0	-2.504898	
23477	Tumor Necrosis Factor-Beta	PARN	0	-2.486416	
23478	Tumor Necrosis Factor-Beta	P4HA2	0.000001	-2.454161	
23479	Tumor Necrosis Factor-Beta	CNOT8	0.000002	-2.427209	
23480	Tumor Necrosis Factor-Beta	TAPBP	0	-2.414875	
23481	Tumor Necrosis Factor-Beta	PAFAH1B1	0.000003	-2.405888	
23482	Tumor Necrosis Factor-Beta	ATP10B	0.000002	-2.377451	
23483	Tumor Necrosis Factor-Beta	PREPL	0.000001	-2.37571	
23484	Tumor Necrosis Factor-Beta	BTBD1	0	-2.369424	
23485	Tumor Necrosis Factor-Beta	NEK9	0.000003	-2.346205	
23486	Tumor Necrosis Factor-Beta	NUDT9	0.000001	-2.341673	
23487	Tumor Necrosis Factor-Beta	DUT	0.000001	-2.339239	
23488	Tumor Necrosis Factor-Beta	IQGAP1	0	-2.287108	
23489	Tumor Necrosis Factor-Beta	NSA2	0.000001	-2.277827	
23490	Tumor Necrosis Factor-Beta	SPTBN1	0	-2.266274	
23491	Tumor Necrosis Factor-Beta	NGLY1	0.000003	-2.221813	
23492	Tumor Necrosis Factor-Beta	EXOC1	0.000002	-2.187854	
23493	Tumor Necrosis Factor-Beta	C4orf46	0	-2.158543	
23494	Tumor Necrosis Factor-Beta	RPS17	0.000001	-2.153819	
23495	Tumor Necrosis Factor-Beta	ST13	0	-2.133898	
23496	Tumor Necrosis Factor-Beta	RPL10A	0	-2.119268	
23497	Tumor Necrosis Factor-Beta	TSPAN8	0.000002	-2.115194	
23498	Tumor Necrosis Factor-Beta	SEC11A	0	-2.109864	

23499	Tumor Necrosis Factor-Beta	DCAF6	0.000003	-2.083197	
23500	Tumor Necrosis Factor-Beta	ASAH1	0	-2.060228	
23501	Tumor Necrosis Factor-Beta	RER1	0	2.071819	
23502	Tumor Necrosis Factor-Beta	H2AFX	0.000002	2.148138	
23503	Tumor Necrosis Factor-Beta	HLA-A	0	2.15835	
23504	Tumor Necrosis Factor-Beta	TP73	0	2.244648	
23505	Vomiting	IGF2	0.007315	-7.056791	
23506	Vomiting	HLA-DRB4	0.006519	-4.148371	
23507	Vomiting	CXCL11	0.000089	-3.227446	
23508	Vomiting	CXCL5	0.000891	-3.025244	
23509	Vomiting	CASQ2	0.002929	-2.96268	
23510	Vomiting	IL11	0.025439	-2.222248	
23511	Vomiting	JAM3	0.012073	-2.194845	
23512	Vomiting	LOC100287689	0.0074	-2.170705	
23513	Vomiting	EHD2	0.007315	-2.143731	
23514	Vomiting	UCA1	0.027932	-2.137298	
23515	Vomiting	CXCL1	0.007315	-2.044647	
23516	Vomiting	FAM19A1	0.043807	-2.002023	
23517	White color finding	ARSE	0.046405	2.34408	
